# Abstract Supplement HIV Glasgow 8–11 November 2026, Glasgow, UK/Virtual

**DOI:** 10.1002/jia2.70211

**Published:** 2026-11-06

## Contents

Commonly‐Used Abbreviations 1

Oral Presentations

    PrEP Today and Tomorrow: Challenges, Advances and Equitable Access 3

    Novel Strategies and Treatment 5

    The Hepatitis Alphabet: What's the Latest? 7

    Cardiometabolic Complications 9

    Antiretroviral Treatment and Resistance 14

    Under‐Served Populations 18

    Hot Topics 22

Poster Presentations

    ARV‐Based Prevention: Vertical Transmission and PEP 28

    ARV‐Based Prevention: PrEP 35

    Treatment Strategies: Novel Therapeutic Targets (Phase I and II) 61

    Treatment Strategies: RCTs—Oral and Injectable Therapy in First‐Line and Suppressed Switch Populations 72

    Treatment Strategies: Real‐World and Implementation Science Studies, Oral and Injectable Therapy 95

    Treatment Strategies: Treatment Experienced Adults (Second‐Line and Multi‐Drug Resistance Studies) 182

    Treatment Strategies: Models of Care for Ageing/Frail Populations Including Virological Failure and Switching 204

    Treatment Strategies: Rapid ART Initiation 213

    Treatment Strategies: Adherence 222

    Clinical Management Considerations: Women 232

    Clinical Management Considerations: Late Presenters 245

    Clinical Management Considerations: Transgender People 255

    Clinical Management Considerations: Other 261

    Cure/Post‐Treatment Control 269

    Opportunistic Infections and AIDS‐Defining Cancers 274

    Clinical Pharmacology 280

    Community‐Based Treatment and Prevention Initiatives, Including Primary Care Screening 289

    Public Health Strategies and Options 303

    Cost and Cost‐Effectiveness 340

    Models of Care: Evaluation of ARV Delivery and Coverage 348

    Co‐Morbidities and Complications of Disease and/or Treatment: Ageing and Frailty 364

    Co‐Morbidities and Complications of Disease and/or Treatment: CV/Metabolic Including Weight Gain 380

    Co‐Morbidities and Complications of Disease and/or Treatment: Malignancies (Non‐AIDS Defining) 433

    Co‐Morbidities and Complications of Disease and/or Treatment: Neurological 440

    Co‐Morbidities and Complications of Disease and/or Treatment: Mental Health Disorders 445

    Co‐Morbidities and Complications of Disease and/or Treatment: Other 455

    People Living With HIV and Mpox Virus 481

    People Living With HIV and Sexually Transmitted Diseases 487

    People Living With HIV and Tuberculosis 494

    People Living With HIV and Viral Hepatitis 497

    People Living With HIV and Other Infections 508

Author Index 517

## Commonly‐Used Abbreviations

HIV Glasgow 2026 Abbreviations List

Common abbreviations used throughout this supplement are noted here to avoid repetition of definitions

**Table:** 

2DR	two‐drug regimen
3DR	three‐drug regimen
3TC	lamivudine
ABC	abacavir
ACC	American College of Cardiology
AE	adverse event
AHA	American Heart Association
aHR	adjusted hazard ratio
AI	artificial intelligence
AIDS	acquired immunodeficiency syndrome
ANOVA	analysis of variance
anti‐HBc	hepatitis B core antibody
aOR	adjusted odds ratio
ART	antiretroviral therapy
ATV	atazanavir
ATV/r	atazanavir/ritonavir
AZT	zidovudine
B	bictegravir
BHIVA	British HIV Association
BIC	bictegravir
BID	twice‐daily
BL	baseline
BMI	body mass index
BP	blood pressure
C	cobicistat
CAB	cabotegravir
cART	combination antiretroviral therapy
CDC	Centers for Disease Control and Prevention
CI	confidence interval
CNS	central nervous system
COBI	cobicistat
CT	computed tomography
DAA	direct‐acting antivirals
DDI	drug‐drug interaction
DNA	deoxyribonucleic acid
DOR	doravirine
doxyPEP	doxycycline post‐exposure prophylaxis
DR	drug regimen
DRV	darunavir
DRV/b	boosted darunavir
DRV/c	cobicistat‐boosted darunavir
DRV/r	ritonavir‐boosted darunavir
DTG	dolutegravir
E	elvitegravir
EACS	European AIDS Clinical Society
ECDC	European Centre for Disease Prevention and Control
EEA	European Economic Area
EFV	efavirenz
ELISA	enzyme‐linked immunosorbent assay
EMA	European Medicines Agency
ETR	etravirine
EVG	elvitegravir
EVG/c	cobicistat‐boosted elvitegravir
EU	European Union
F	emtricitabine
FDA	United States Food and Drug Administration
FTC	emtricitabine
GFR	glomerular filtration rate
GRT	genotypic resistance test
GP	general practitioner
HAART	highly active antiretroviral therapy
HbA1c	haemoglobin A1c
HBcAb	hepatitis B core antibody
HBeAg	hepatitis B e‐antigen
HBsAb	hepatitis B surface antibody
HBsAg	hepatitis B surface antigen
HBV	hepatitis B virus
HCV	hepatitis C virus
HCVAb	hepatitis C antibody
HDL	high‐density lipoprotein
HIRI	HIV incidence risk index
HIV	human immunodeficiency virus
HLA	human leukocyte antigen
HPV	human papillomavirus
HR	hazard ratio
HRQoL	health‐related quality of life
HSV	herpes simplex virus
IC	inhibitory concentration
IFN	interferon
IgG	immunoglobulin G
IM	intramuscular
INI	integrase inhibitor
INSTI	integrase inhibitor
IQR	interquartile range
ITT	intention‐to‐treat
IV	intravenous
LA	long‐acting
LAR	long‐acting regimen
LDL	low‐density lipoprotein
LEN	lenacapavir
LGBT	lesbian, gay, bisexual, transgender
LPV	lopinavir
LPV/r	ritonavir‐boosted lopinavir
MedDRA	Medical Dictionary for Regulatory Activities
MRI	magnetic resonance imaging
MSM	men who have sex with men
MSW	men who have sex with women
NEV	nevirapine
NGO	non‐governmental organisation
NHS	National Health Service
NNRTI	non‐nucleoside reverse transcriptase inhibitor
NRTI	nucleoside reverse transcriptase inhibitor
NVP	nevirapine
OR	odds ratio
PCR	polymerase chain reaction test
PD1	programmed death‐1
PDL1	programmed death‐ligand‐1
PEP	post‐exposure prophylaxis
PI	protease inhibitor
PLWH	people living with HIV
PrEP	pre‐exposure prophylaxis
PRO	patient‐reported outcomes
PWH	people with HIV
PWID	people who inject drugs
QD	once‐daily
QoL	quality of life
RAL	raltegravir
RAM	resistance‐associated mutation
RBV	ribavirin
RCT	randomized controlled trial
RI	renal impairment
RNA	ribonucleic acid
RR	risk ratio
RTV	ritonavir
SC	subcutaneous
SD	standard deviation
SE	standard error
SQVr	ritonavir‐boosted saquinavir
STD	sexually transmitted disease
STI	sexually transmitted infection
TAF	tenofovir alafenamide
TB	tuberculosis
TDF	tenofovir disoproxil fumarate
U=U	undetectable = untransmissible
VF	virological failure
VL	viral load
VS	virological suppression
WHO	World Health Organization
WHODD	WHO Drug Dictionary
XTC	lamivudine or emtricitabine
ZDV	zidovudine

Disclaimer: Some abstracts have been edited in line with the Publisher's requirements to ensure consistency with the publication format. These changes relate to formatting and style only, and do not affect the scientific content.

## Oral Presentations

## PrEP Today and Tomorrow: Challenges, Advances and Equitable Access

### O21 Higher Persistence on Twice‐Yearly Lenacapavir Versus Daily F/TDF for PrEP in the UK and France in the PURPOSE 5 Study

Chloe Orkin ^1^, Jade Ghosn^2^, Karine Lacombe^3^, Victoria Tittle^4^, Killian Quinn^5^, Amanda Clarke^6^, Gabriel Schembri^7^, Patrick Philibert^8^, Emily Clarke^9^, Hughes Cordel^10^, Stephen Taylor^11^, Eric Cua^12^, Iain Reeves^13^, Geoffroy Liegeon^14^, Jenna Yager^15^, Christoph C. Carter^15^, Yuliang Chen^15^, Pamela Wong^15^, Moupali Das^15^, Jean‐Michel Molina^14^

^1^SHARE Collaborative, Blizard Institute, Faculty of Medicine and Dentistry, Queen Mary University of London, London, UK; ^2^Université Paris Cité, AP‐HP, Hôpital Bichat–Claude Bernard, Paris, France; ^3^Sorbonne Université, AP‐HP, Hôpital Saint‐Antoine, Paris, France; ^4^Chelsea and Westminster Hospital NHS Foundation Trust, London, UK; ^5^Caldecot Centre, King's College Hospital NHS Foundation Trust, London, UK; ^6^University Hospitals Sussex NHS Foundation Trust, Brighton, UK; ^7^Manchester Centre for Sexual Health, Manchester Royal Infirmary, Manchester, UK; ^8^Pôle Multidisciplinaire de Médecine Interne et Maladies Infectieuses, Hôpital Européen, Marseille, France; ^9^Liverpool University Hospitals NHS Foundation Trust, Liverpool, UK; ^10^Hôpital Avicenne, AP‐HP, Bobigny, France; ^11^University of Birmingham and Birmingham Heartlands Hospital, Birmingham, UK; ^12^University of Nice‐Sophia Antipolis, Nice, France; ^13^Homerton Healthcare NHS Foundation Trust, London, UK; ^14^Université Paris Cité, AP‐HP, Hôpital Saint‐Louis and Lariboisière, INSERM U944, Paris, France; ^15^Gilead Sciences, Inc., Foster City, CA, US

**Background**: Twice‐yearly lenacapavir demonstrated superior efficacy versus daily oral emtricitabine/tenofovir disoproxil fumarate (F/TDF) for pre‐exposure prophylaxis (PrEP). PURPOSE 5 aimed to assess PrEP persistence on lenacapavir versus daily F/TDF among individuals with increased PrEP need but not using PrEP.

**Methods**: PURPOSE 5 (NCT06513312) is a randomized, open‐label study among people not currently taking oral PrEP in the UK/France. Participants were randomized 2:1 to twice‐yearly lenacapavir injection or daily oral F/TDF for 52 weeks. Persistence (primary endpoint) was assessed as continued adherence over time through week 52. Lenacapavir persistence was defined as on‐time injections (±2 weeks) at week 26 and on‐time follow‐up at week 52. F/TDF persistence was defined as tenofovir‐diphosphate concentration in dried blood spots consistent with ≥4 doses/week at on‐time follow‐up visits at weeks 13, 26, 39 and 52. Persistence was compared using the Fisher exact test.

**Results**: Of 267 participants (lenacapavir, 180; F/TDF, 87), 72.3% were cisgender men, 7.1% were cisgender women and 20.6% were gender diverse. Median age was 34 years, 68.8% of participants were white, and 37.8% were PrEP‐naïve. 161 (89.4%) and 73 (83.9%) participants attended the week 52 visit in the lenacapavir and F/TDF groups, respectively. Significantly more participants persisted on lenacapavir versus F/TDF at week 52 (85.6% [154/180] vs. 46.3% [37/80]; *p* < 0.0001) (Table 1). No participants in the lenacapavir group (0 per 100 person‐years [95% CI: 0.000, 2.162]) and one in the F/TDF group (1.273 per 100 person‐years [95% CI: 0.032, 7.091]) acquired HIV. At diagnosis, tenofovir‐diphosphate concentrations in this participant were consistent with <2 F/TDF doses/week. The most common adverse events with lenacapavir were injection‐site reactions (93.9%), most frequently nodules (67.8%), pain (60.0%), indurations (20.6%) and erythema (17.8%). Serious adverse events, unrelated to study drug, occurred in 10 participants (lenacapavir 7 [3.9%]; F/TDF 3 [3.4%]). PrEP preference at day 1 and week 26 is shown (Figure 1).

**Conclusions**: At week 52, PrEP persistence was significantly higher with twice‐yearly lenacapavir than daily F/TDF in people in the UK and France not currently on oral PrEP. This supports the potential public health benefit of lenacapavir to improve persistence and thereby effectiveness, of PrEP.

**Funding**: Gilead Sciences, Inc.

**FIGURE 1:**
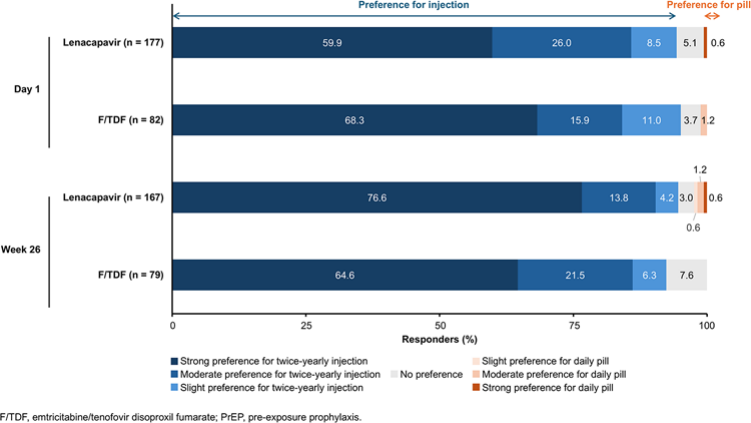
O21 | PrEP preference at day 1 and week 26.

**TABLE 1:**
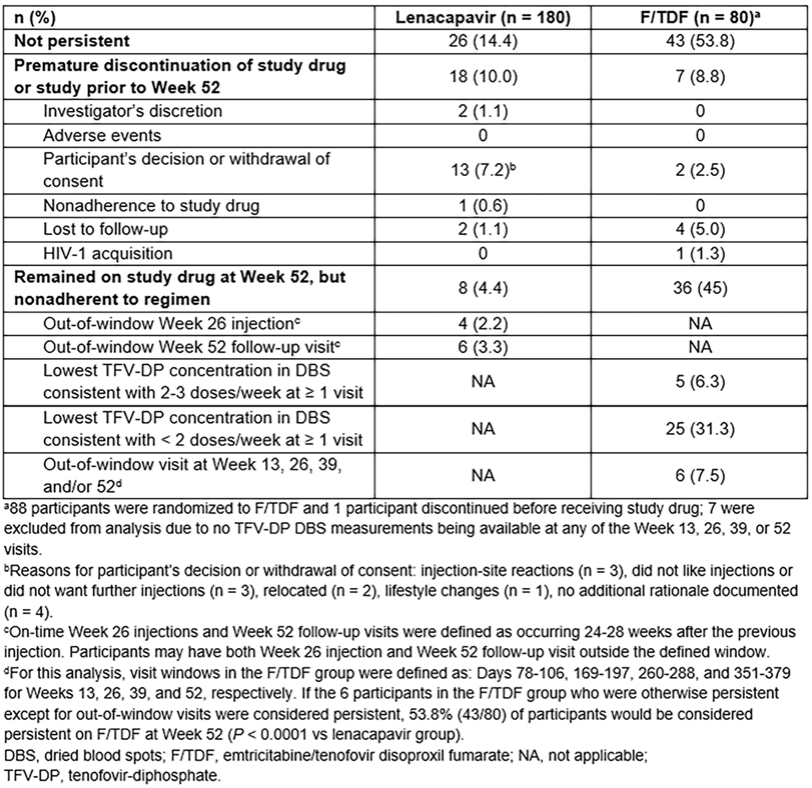
O21 | Non‐persistence through week 52.

### O22 Mind the Gap: Estimating the Impact of HIV PrEP on the Epidemic in Switzerland

Yuqian Wang^1^, Katharina Kusejko^1^, Bashkim Jaha^1^, Roger Kouyos^1^, Vanessa Christinet^2^, Florian Vock^3^, Dominique Braun^4^, Julia Notter^5^, Bernard Surial^6^, Matthias Cavassini^7^, Alexandra Calmy^8^, Luigia Elzi^9^, Patrick Schmid^10^, Irene A. Abela^1^, Andrea Farnham^11^, Jan Fehr^11^, Benjamin Hampel ^11^

^1^Division of Infectious Diseases and Hospital Epidemiology, University Hospital Zurich, Zurich, Switzerland; ^2^Medical Center du Flon, Lausanne, Switzerland; ^3^Swiss AIDS Federation, Zurich, Switzerland; ^4^Hirslanden Klinik Zurich, Zurich, Switzerland; ^5^Division of Infectious Diseases, University Hospital Basel, University of Basel, Basel, Switzerland; ^6^Inselspital Bern, University Hospital, University of Bern, Bern, Switzerland; ^7^Division of Infectious Diseases, University Hospital Lausanne, University of Lausanne, Lausanne, Switzerland; ^8^Division of Infectious Diseases, University Hospital Geneva, University of Geneva, Geneva, Switzerland; ^9^Division of Infectious Diseases, Regional Hospital Bellinzona, Bellinzona, Switzerland; ^10^Division of Infectious Diseases, Infection Prevention and Travel Medicine, HOCH Health Ostschweiz, Cantonal Hospital St. Gallen (University Teaching and Research Hospital), St. Gallen, Switzerland; ^11^Department of Public & Global Health, Epidemiology, University of Zurich Biostatistics and Prevention Institute, Zurich, Switzerland

**Background**: Aiming towards the ending of HIV transmission, we assess the impact of HIV pre‐exposure prophylaxis (PrEP) on the HIV epidemic in Switzerland since the initiation of the SwissPrEPared programme in 2019 and identify gaps in PrEP‐access.

**Methods**: We used three data sources for a descriptive analysis of the HIV epidemic and estimated the impact of PrEP with synthetic difference‐in‐differences (SDID) modelling. The three data sources were: (i) mandatory HIV surveillance data from the Swiss Federal Office of Public Health (FOPH) (2011–2024); (ii) the SwissPrEPared study, enrolling individuals taking PrEP (2019–2025); and (iii) the Swiss HIV Cohort Study (SHCS), enrolling people with HIV (2016–2025). For the SDID (FOPH data), Swiss men who have sex with men (MSM) were defined as the intervention group, as SwissPrEPared primarily enrols MSM populations. The control groups included Swiss heterosexual individuals and heterosexual individuals from HIV high‐prevalence countries.

**Results**: 9668 people started PrEP within the SwissPrEPared programme between 2019 and 2024 in Switzerland. During the same period, 2070 people were diagnosed with HIV in Switzerland according to the FOPH. While overall HIV diagnoses decreased by 24% between 2019 (421) and 2024 (318), HIV diagnoses among Swiss MSM decreased by 64% between 2019 (74) and 2024 (27). In contrast, diagnoses among MSM of foreign or unknown nationality increased. The SDID estimated an average yearly difference in HIV diagnoses during the post‐intervention period between the intervention and the synthetic control group of −42 HIV diagnoses/year (95% CI: −50.51, −33.64) (Figure 1). Thus, we estimate that during the study period (2019–2024), in total 252 HIV diagnoses were averted (95% CI: 201.84, 303.06). Compared to MSM diagnosed with HIV and enrolled in the SHCS, MSM on PrEP were older and had higher education. Main reasons why MSM diagnosed with HIV did not initiate PrEP were the absence of risk perception and a lack of awareness of PrEP. MSM who were diagnosed with HIV within SwissPrEPared were more likely to engage in sex work and chemsex.

**Conclusions**: The current PrEP implementation strategy in Switzerland shows high impact among Swiss MSM. Barriers in access must be addressed and non‐MSM individuals at increased risk need to be identified to achieve zero HIV transmission.

**FIGURE 1:**
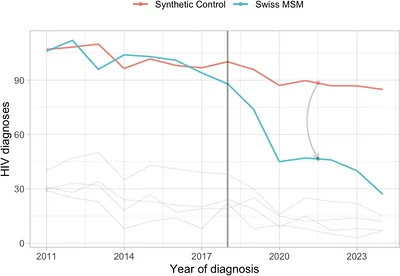
O22 | Estimated average intervention effect on the treatment group (ATT) compared with the synthetic control, 2011–2024 (grey lines represent the control group trajectories).

## Novel Strategies and Treatment

### O31 A 24‐Week Phase 2a Maintenance Study of TMB‐365/TMB‐380 q8w in People With Suppressed HIV‐1 Infection

Martin Markowitz ^1^, Jacob P. Lalezari^2^, Edwin DeJesus^3^, Michael Sension^4^, Gordon Crofoot^5^, Moti Ramgopal^6^, Mezgebe Berhe^7^, Kuei‐Ling Kuo^8^, Ying‐An Lai^8^

^1^Irving Medical Center, Columbia University, Irvine, CA, US; ^2^Quest Clinical Research, San Francisco, CA, US; ^3^Orlando Immunology Center, Orlando, FL, US; ^4^CAN Community Health, Fort Lauderdale, FL, US; ^5^Crofoot Research Center, Houston, TX, US; ^6^Midway Immunology and Research Center, Fort Pierce, FL, US; ^7^North Texas Infectious Diseases Consultants, Dallas, TX, US; ^8^TaiMed Biologics Inc., Taipei City, Taiwan

**Background**: TMB‐365, a second‐generation post‐attachment monoclonal antibody binds to the second domain of CD4 and displays improved PK, antiviral activity and breadth of coverage when compared to ibalizumab. TMB‐380 (VRC07523‐LS) is a broadly neutralizing antibodies that binds to the CD4 binding site of HIV Env and has potent antiviral activity and favourable safety and PK profile. Here, we present results of a 24‐week phase 2a maintenance study of the combination in people with suppressed HIV‐1 infection (NCT05275998).

**Methods**: Participants suppressed (VL <50 c/mL) with continuous oral combination antiretroviral therapy (cART) for at least 6 months, clinically stable and had no history of virologic failure (VF) were enrolled (target *N* = 20). Baseline screening for antibody susceptibility was not performed. Enrolled participants ceased oral cART and 4800 mg of TMB‐365 and TMB‐380 were infused every 8 weeks for 3 doses (baseline, week 8, week 16). Oral cART was restarted at the week 24 visit. All infusions were performed over 60 min. VF was defined as 2 consecutive HIV‐RNA values > 50 c/mL at least 2 weeks apart. Efficacy was evaluated as % participants with VL <50 c/mL at week 24.

**Results**: 21 individuals were enrolled; 1 was replaced due to GI bleeding before dosing. 18 of 20 completed the study. Two participants discontinued after infusion #1 due to rash‐related events (generalized rash on day 32 and atypical papular eruption on day 7). No serious adverse events, grade 3 or 4 adverse events (AEs) or acute infusion reactions were observed. Treatment‐emergent AEs (*N* = 48) were mild to moderate. 16 were attributed to infusions with drugs; no cases of protocol‐defined VF were observed. At week 24, 16/17 (94%) had plasma VL below 50 c/mL, one had VL of 59 c/mL, *N* = 2 early discontinuation, *N* = 1 no data. Mean week 24 TMB‐365 and TMB‐380 concentrations were 29 and 107 µg/mL, exceeding targets by 96‐ and 1.6‐fold, respectively.

**Conclusions**: Every 8‐week infusions of TMB‐365 and TMB‐380 without pre‐switch susceptibility testing at 4800 mg each are safe and effective as a complete maintenance therapy in people with suppressed HIV‐1 infection. No confirmed VF was observed. Results support the development of TMB‐365/TMB‐380 in phase 2b.

### O33 Immunogenicity and Associated Safety of Amtabafusp Alfa, a Bispecific T‐Cell Engager, in a Phase 1b Study in People With HIV

Kimberly Workowski^1^, Yanan Zheng^2^, Xiang Liu^2^, Monika Sobczyk^2^, Devi SenGupta^2^, Elena Vendrame^2^, Tina Chakrabarti^2^, Princy Kumar ^3^

^1^Department of Medicine, Emory University School of Medicine, Atlanta, GA, US; ^2^Gilead Sciences, Inc., Foster City, CA, US; ^3^Division of Infectious Disease, MedStar Georgetown University Hospital, Washington, DC, US

**Background**: Amtabafusp alfa (ABF) is a novel HIV bispecific T‐cell engager comprising an Env‐targeting modified human CD4 domain 1 variant (D1v) fused to a humanized anti‐CD3 antibody binding fragment that enables T‐cell redirection and activation against cells with HIV. Herein, safety and immunogenicity following ABF administration were evaluated in virologically suppressed (VS) people with HIV (PWH).

**Methods**: In a randomized, blinded, placebo‐controlled phase 1b study, VS PWH on antiretroviral therapy were randomized to receive intravenous ABF or placebo in single ascending doses (SAD; 0.3–150 mg) or multiple ascending doses (MAD; 30–75 mg once every 2 weeks [dose 1, 30 mg; doses 2–5, 30–75 mg]). Immunogenicity to ABF was assessed by measuring serum anti‑drug antibodies (ADAs) using a validated, semi‑quantitative electrochemiluminescence bridging assay that characterized specificity to both the CD4 D1v and anti‑human CD3ε domains. Adverse events (AEs) were monitored throughout the study.

**Results**: All randomized participants (*n* = 54) received ≥1 dose of study drug and were evaluated for safety outcomes; immunogenicity was evaluated in participants who received ABF (*n* = 39). Treatment‐emergent ADAs were observed in 14/26 (54%) SAD and 7/13 (54%) MAD participants (median ADA titre over time: 270–14,595 and 30–32,800, respectively); all treatment‐emergent ADAs were treatment‐induced and persisted throughout the study once detected (median onset time: 56 and 48 days, respectively). All participants with ADAs had anti‐CD3 ADAs. One participant in the 30 mg SAD cohort had ADAs against the CD4 D1v domain of ABF. No association was observed between the incidence of ADAs and AEs, including AEs of interest (Table 1). Most AEs were Grade ≤2. Grade ≥3 treatment‐related AEs occurred in 1/21 (5%) and 5/18 (28%) ADA‐positive and ADA‐negative participants, respectively; one participant with Grade 3 treatment‐related pyrexia in the setting of cytokine release syndrome on day 1 had ADAs on day 29. One ABF 150 mg SAD participant and one ABF 30 mg MAD participant had a significant drop in serum ABF concentrations after initial ADA detection.

**Conclusions**: ADAs occurred in approximately half of ABF participants, with no apparent dose−response trend and no association between ADAs and AEs. ABF was generally safe and well tolerated.

**TABLE 1:**
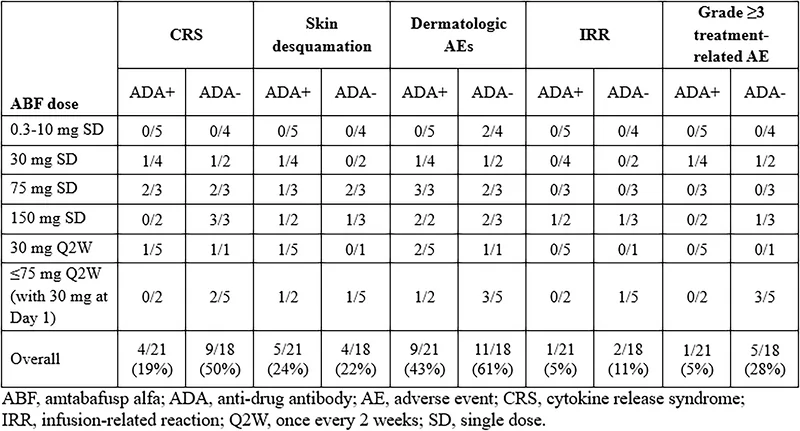
O33 | Summary of incidence of AEs of interest by ADA status.

## The Hepatitis Alphabet: What's the Latest?

### O34 Isolated Hepatitis B Core Antibody and Liver‐Related Outcomes in People Living With HIV Enrolled in the Swiss HIV Cohort Study

Adrien Calame ^1^, Estelle Aymes^2^, Lorin Begré^3^, Gilles Wandeler^3^, Oksana Fiammingo^1^, Matthias Cavassini^4^, Dominique Braun^5^, Alexandra Calmy^1^, Maël Barthoulot^2^, Olivier Ségéral^1^, on behalf of the Swiss HIV Cohort Study

^1^Division of Infectious Diseases, Department of Medicine, Geneva University Hospitals and Medical School, Geneva, Switzerland; ^2^Clinical Research Centre, Geneva University Hospital, Geneva, Switzerland; ^3^Department of Infectious Diseases, Inselspital, Bern University Hospital, University of Bern, Bern, Switzerland; ^4^Division of Infectious Diseases, Lausanne University Hospital, University of Lausanne, Lausanne, Switzerland; ^5^Department of Infectious Diseases and Hospital Epidemiology, University Hospital Zurich, University of Zurich, Zurich, Switzerland

**Background**: The clinical significance of isolated antibody to hepatitis B core antigen (anti‐HBc) in people living with HIV (PLWH) remains uncertain. We assessed its association with liver‐related morbidity and mortality in a large PLWH cohort.

**Methods**: This cohort study used data from the Swiss HIV Cohort Study. Eligible participants had ≥1 complete HBV serology, received highly active antiretroviral therapy (ART), and attended ≥2 visits ≥6 months apart between 1997 and 2018. The primary exposure was HBV serological profile at baseline classified into three categories: resolved HBV infection, or HBV‐unexposed/vaccinated (reference). The primary outcome was a composite of liver‐related mortality, hepatocellular carcinoma or advanced fibrosis/cirrhosis. Ten‐year cumulative incidence was estimated using competing‐risk regression (Fine and Gray), and independent associations using Cox proportional hazards models adjusted for established risk factors.

**Results**: Among 9595 participants, 1334 (13.9%) had isolated anti‐HBc, 2734 (28.5%) resolved HBV infection and 5527 (57.6%) were unexposed/vaccinated. Median follow‐up was 13.6 years (IQR 8.1–19.6). The 10‐year cumulative incidence of liver‐related morbidity and mortality was 15.7% (95% CI: 13.6–17.9) with isolated anti‐HBc, 10.4% (9.2–11.7) with resolved HBV infection and 7.2% (6.5–8.0) in unexposed/vaccinated participants (Figure 1). Compared with unexposed/vaccinated participants, isolated anti‐HBc (sub‐hazard ratio [SHR] 1.99, 95% CI: 1.73–2.29; *p* < 0.0001) and resolved HBV infection (SHR 1.46, 1.30–1.65; *p* < 0.0001) were associated with increased risk. After adjustment, neither isolated anti‐HBc (adjusted HR 1.12, 95% CI: 0.95–1.31; *p* = 0.18) nor resolved HBV infection (0.99, 0.88–1.11; *p* = 0.85) remained associated with the outcome. HCV co‐infection was the strongest independent risk factor (adjusted HR 3.15, 95% CI: 2.79–3.55; *p* < 0.0001) (Table 1).

**Conclusions**: Isolated anti‐HBc identifies PLWH at increased risk of liver‐related outcomes, but this excess risk is largely explained by concomitant HCV co‐infection rather than isolated anti‐HBc itself. These findings may refine liver disease surveillance in PLWH, particularly after switching to non‐HBV‐active ART.

**FIGURE 1:**
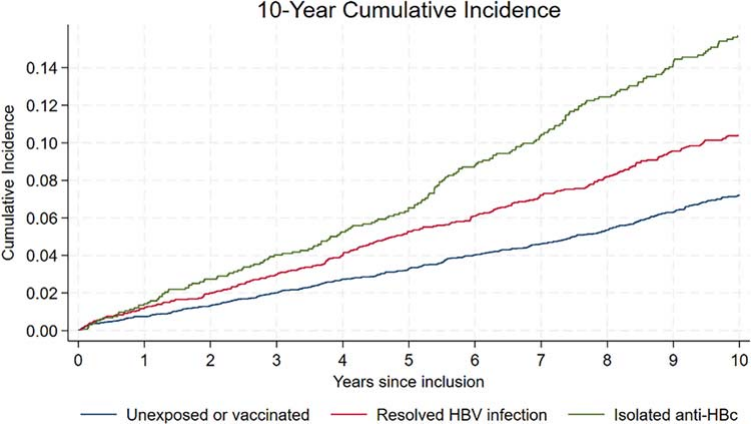
O34 | Cumulative incidence of liver‐related morbidity and mortality over 10 years, by HBV serological profile.

**TABLE 1:**
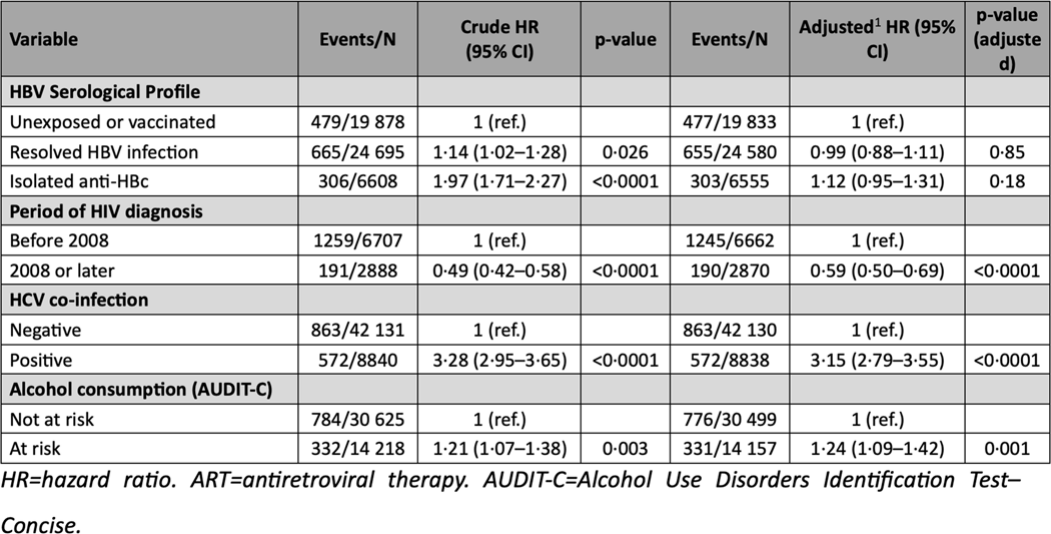
O34 | Crude and adjusted hazard ratios for liver‐related morbidity and mortality according to HBV serological profile and other covariates.

### O35 HBV Outcomes After Switching to Long‐Acting Cabotegravir Plus Rilpivirine in the Spanish RELATIVITY Cohort

María del Mar Arcos Rueda^1^, Miguel Torralba González de Suso^2^, Juan Tiraboschi^3^, Analuz Fernández^3^, Maria Jose Nuñez Orantos^4^, Jesús Troya García^5^, Luis Buzón^6^, María José Galindo Puerto^7^, Ruth Caballero Asensio^8^, María Remedios Alemán Valls^9^, María Belén Sánchez López^10^, María José Crusells Canales^11^, Francisco Fanjul^12^, Adrián Rodríguez^13^, María Carmen Fariñas Álvarez^14^, Laura Gisbert Pérez^15^, Maria del Carmen Navarro Sáez^16^, Ignacio De los Santos Gil^17^, Carmen Hidalgo‐Tenorio^18^, Luis Enrique Morano Amado^19^, Enrique Bernal Morell^20^, David Vinuesa García^21^, María Josefa De la Asunción Villaverde^22^, Alejandro David Bendala Estrada^23^, Luz Martín-Carbonero ^1^, on behalf of RELATIVITY Cohort Group

^1^HIV Unit, Hospital Universitario La Paz, Madrid, Spain; ^2^Hospital Universitario Guadalajara, Guadalajara, Spain; ^3^Hospital Universitario de Bellvitge, Barcelona, Spain; ^4^Hospital Clínico San Carlos, Madrid, Spain; ^5^Hospital Universitario Infanta Leonor, Madrid, Spain; ^6^Hospital de Burgos, Burgos, Spain; ^7^Hospital Clínico Universitario de Valencia, Valencia, Spain; ^8^Hospital Universitario Miguel Servet, Zaragoza, Spain; ^9^Hospital Universitario de Canarias, Canarias, Spain; ^10^Hospital Universitario 12 de Octubre, Madrid, Spain; ^11^Hospital Clínico Universitario Lozano Blesa, Zaragoza, Spain; ^12^Hospital Universitario Son Espases, Mallorca, Spain; ^13^Hospital Universitario Son Llatzer (Palma de Mallorca), Mallorca, Spain; ^14^Hospital Universitario Marqués de Valdecilla, Santander, Spain; ^15^Hospital Universitario Mutua de Terrassa, Barcelona, Spain; ^16^Parc Taulí Hospital Universitari, Sabadell, Spain; ^17^Hospital Universitario La Princesa, Madrid, Spain; ^18^Hospital Universitario Virgen de las Nieves, Granada, Spain; ^19^Hospital Universitario Álvaro Cunqueiro, Vigo, Spain; ^20^Hospital General Universitario Reina Sofía, Murcia, Spain; ^21^Hospital Clínico San Cecilio, Granada, Spain; ^22^Hospital de Denia Marina Salud, Alicante, Spain; ^23^Hospital Universitario de Torrejón, Madrid, Spain

**Background**: Long‐acting cabotegravir plus rilpivirine (LA‐CAB+RPV) is a safe and effective maintenance antiretroviral treatment in clinical trials and real‐world cohorts. However, due to the lack of activity against hepatitis B virus (HBV), concerns remain about the risk of HBV acquisition in people with HIV (PWH) without serological evidence of HBV immunity and about HBV reactivation in PWH with previous HBV infection, particularly those with an isolated anti‐HBc pattern. We assessed the rest of HBV acquisition and HBV reactivation after switching to LA‐CAB+RPV in the Spanish RELATIVITY cohort.

**Methods**: RELATIVITY is a Spanish real‐world cohort including PWH aged >18 years who switched to LA‐CAB+RPV before 31 December 2024. HBV reactivation was defined as new HBsAg positivity after previously negative HBsAg, or new detection of HBV DNA after switching to LA‐CAB+RPV.

**Results**: The study population included 3349 individuals. Median age was 45 years (IQR 37–54), and median follow‐up was 25.1 months (IQR 19.3–30.8). Most had recorded male sex at birth (84.1%) and Spanish nationality (69.6%). The most common route of HIV acquisition was sex between men (56.4%), followed by heterosexual transmission (16.9%). HBV serological status is summarized in Table 1. Among PWH with isolated anti‐HBc, HBV DNA was assessed 50 times in 43 out of 115 individuals (37.4%); no confirmed HBV reactivation was documented. Among 403 vaccinated PWH without serological evidence of vaccine response, one case of acute hepatitis B was identified (0.25%; 95% CI: 0.01–1.39) and HBV DNA was available in 50 individuals (12.4%), with no additional positive results recorded. Among 247 unvaccinated seronegative PWH, HBV DNA was available in 30 individuals (12.1%) and was negative in all cases.

**Conclusions**: No HBV reactivation was observed after switching to LA‐CAB+RPV among PWH with isolated anti‐HBc. However, acute hepatitis B can occur in susceptible PWH without protective anti‐HBs, supporting systematic HBV serological review, vaccination or revaccination when needed.

**TABLE 1:**
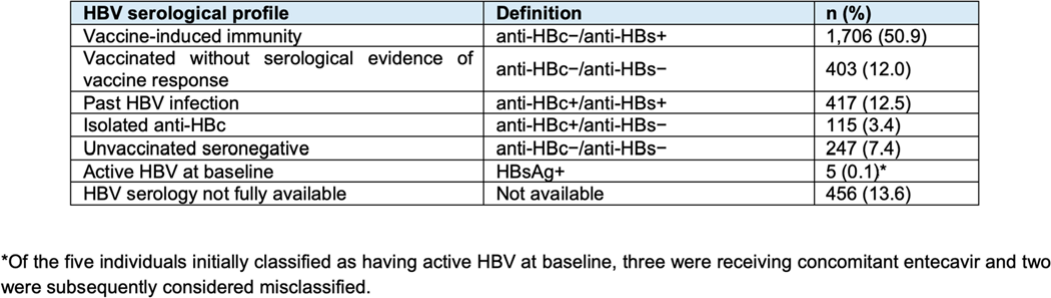
O35 | HBV serological profile at switch to LA‐CAB+RPV.

### O36 Hepatitis B After Switch to Long‐Acting Cabotegravir/Rilpivirine: Data From the SCohoLART Study

Angelo Roberto Raccagni ^1,2^, Tommaso Clemente^1^, Riccardo Lolatto^1^, Nicolò Capra^1^, Serena Rolla^1^, Hasson Hamid^1^, Emanuela Messina^1^, Vincenzo Spagnuolo^1,2^, Elena Bruzzesi^1^, Paola Striglia^1^, Silvia Nozza^1,2^, Antonella Castagna^1,2^

^1^Infectious Diseases Unit, IRCCS San Raffaele Scientific Institute, Milan, Italy; ^2^Vita‐Salute San Raffaele, Milan, Italy

**Background**: Long‐acting cabotegravir/rilpivirine (LA‐CAB/RPV) lacks activity against hepatitis B virus (HBV). We describe HBV serological profiles and HBV events after switch to LA‐CAB/RPV in people with HIV (PWH) with negative hepatitis B surface antigen (HbsAg) in the SCohoLART cohort.

**Methods**: All PWH initiating LA‐CAB/RPV at IRCCS San Raffaele Scientific Institute, Milan, Italy, and enrolled in the prospective SCohoLART study (NCT05663580) were included. HBV status at switch was classified by hepatitis B core antibody (anti‐HBc) and hepatitis B surface antibody (anti‐HBs) as resolved HBV (anti‐HBc+/anti‐HBs+), isolated anti‐HBc (anti‐HBc+/anti‐HBs–), no HBV immunity (anti‐HBc–/anti‐HBs) or vaccine‐induced immunity (anti‐HBc–/anti‐HBs+). Liver enzymes, HBV serology and/or HBV DNA were assessed according to routine practice.

**Results**: Overall, 627 PWH were included: median age was 48.5 years (IQR 40.0–56.4), 567 (90.4%) were male at birth, 468 (74.6%) were men who have sex with men and median time from HIV diagnosis was 13.3 years (8.6–20.0). At switch, 189/627 (30.1%) had previous HBV exposure (positive anti‐HBc: 165/627 [26.3%] with positive anti‐HBs, 24/627 [3.8%] isolated anti‐HBc) and 438/627 (69.9%) no HBV exposure (319/627 [50.9%] vaccine‐induced positive anti‐HBs and 119/627 [19.0%] negative anti‐HBs). Baseline characteristics are summarized in Table 1. Over 2.73 years (1.08–3.24) of median follow‐up, 4/627 (0.6%) HBV events occurred. One clinically overt acute hepatitis due to HBV reactivation occurred within 6 months in a participant with isolated anti‐HBc at switch (1/24; 4.2%), despite previous re‐vaccination without anti‐HBs seroconversion; it required hospitalization and LA‐CAB/RPV discontinuation. Three incident HBV infections were later documented during routine serological follow‐up among anti‐HBc‐negative participants: two with no HBV immunity (2/119; 1.7%) despite vaccination and one with vaccine‐induced immunity but low anti‐HBs (1/319; 0.3%). All three were asymptomatic, showed anti‐HBc and anti‐HBs seroconversion, had no liver toxicity and did not require LA‐CAB/RPV discontinuation.

**Conclusions**: HBV events after LA‐CAB/RPV were infrequent but clinically heterogeneous. Isolated anti‐HBc identified a small subgroup at risk of early clinically significant HBV reactivation, whereas incident infections in incompletely protected anti‐HBc‐negative individuals were late and silent. Quantitative anti‐HBs assessment, optimization of HBV vaccination and prospective HBV monitoring should accompany withdrawal of HBV‐active antiretrovirals.

**TABLE 1:**
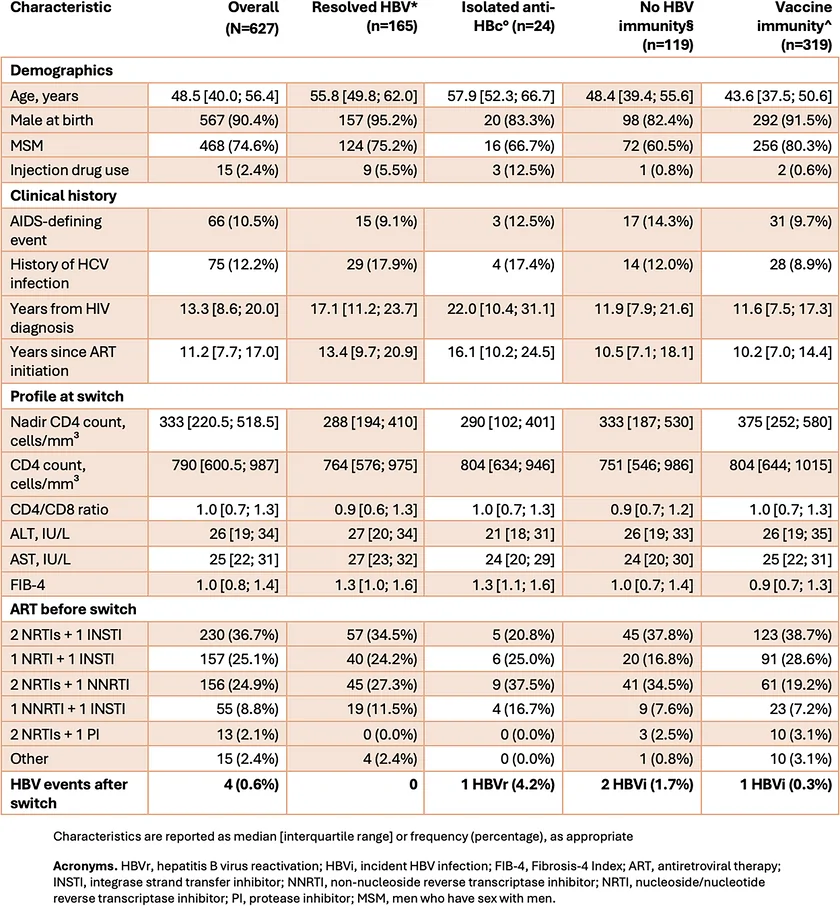
O36 | Baseline characteristics by HBV serologic profile at switch to long‐acting cabotegravir and rilpivirine.

## Cardiometabolic Complications

### O37.1 Performance of the SMART2 Cardiovascular Risk Model in People With HIV and Prior Atherosclerotic Cardiovascular Disease: A Nationwide Cohort Study in the Netherlands

Rosa Elias ^1,2^, Tessa Reitsma^3^, Ferdinand Wit^4,5^, Marc van der Valk^4,5^, Casper Rokx^2^, Annelies Verbon^1^, Jannick Dorresteijn^3^, Berend van Welzen^1^

^1^Department of Infectious Diseases, University Medical Centre Utrecht, Utrecht, Netherlands; ^2^Department of Internal Medicine, and Department of Medical Microbiology and Infectious Diseases, Erasmus University Medical Centre, Rotterdam, Netherlands; ^3^Department of Vascular Medicine and Endocrinology, University Medical Centre Utrecht, Utrecht, Netherlands; ^4^Stichting HIV Monitoring, Amsterdam, Netherlands; ^5^Department of Infectious Diseases, Amsterdam Infection and Immunity Institute, I&II, Amsterdam UMC, University of Amsterdam, Amsterdam, Netherlands

**Background**: People with HIV (PWH) and prior atherosclerotic cardiovascular disease (ASCVD) are at high risk of recurrent cardiovascular events. However, it remains unclear how existing risk prediction tools perform in this population, leaving their clinical utility uncertain. We aimed to evaluate the performance of the SMART2 risk prediction model in PWH with prior ASCVD and assess whether incorporating HIV‐specific characteristics improves risk estimation.

**Methods**: We conducted a retrospective cohort study using data from the Dutch national HIV cohort (ATHENA), including PWH with prior ASCVD in care between January 2010 and February 2024. Baseline was 1 January 2010 for pre‐existing ASCVD, or at least 1 month post‐event for ASCVD developing during follow‐up, ensuring clinical stability. SMART2 was used to estimate baseline 10‐year risk of recurrent cardiovascular events (non‐fatal myocardial infarction, non‐fatal ischemic stroke or cardiovascular death). Performance was assessed by calibration (expected‐to‐observed [E/O] ratio and calibration plots) and discrimination (time‐dependent C‐statistic), accounting for competing risk of non‐cardiovascular death. Subsequently, the baseline hazard was recalibrated using the E/O ratio. SMART2‐adjusted univariate Cox regression was performed to identify correlations between candidate HIV‐specific predictors and events. Using the recalibrated model, these predictors were individually added using Fine–Gray subdistribution hazard models to assess incremental predictive value.

**Results**: Among 1627 PWH with prior ASCVD (Table 1), 310 recurrent cardiovascular events and 240 non‐cardiovascular deaths occurred. Competing‐risk cumulative incidence at 10 years was 23.0% (95% CI: 20.5–25.5%). Before recalibration, SMART2 showed poor discrimination (C‐statistic 0.565, 95% CI: 0.524–0.606) and modest underestimation (E/O ratio 0.93 ± 0.01) (Figure 1). Recalibration improved calibration to a near‐perfect E/O ratio of 0.99. Although regression analysis of candidate HIV‐specific factors (including baseline CD4/CD8 ratio and exposure to metabolically unfavourable antiretrovirals) suggested an association with recurrent events, their addition to the recalibrated model did not improve C‐statistics.

**Conclusions**: SMART2 slightly underestimated absolute risk and showed poor discriminatory power for recurrent cardiovascular events in PWH. Recalibration corrected the risk estimates to reflect the observed incidence. Addition of HIV‐specific predictors did not improve the model's ability to distinguish higher‐risk and lower‐risk individuals, which underscores the need for alternative prediction approaches to better capture underlying cardiovascular risk.

**FIGURE 1:**
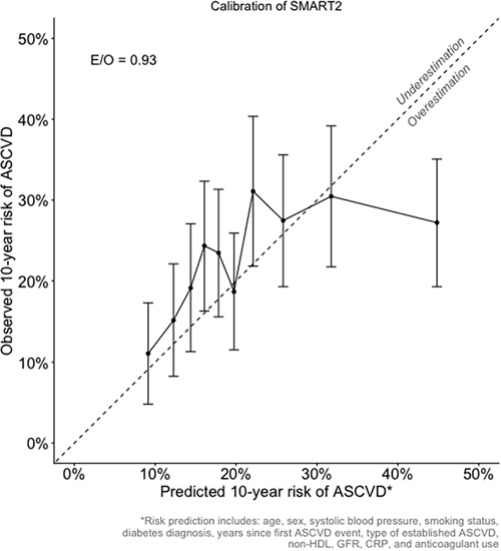
O37.1 | Calibration plot of the 10‐year recurrent ASCVD risk estimated with the SMART2 model with expected/observed (E/O) rate of 0.93.

**TABLE 1:**
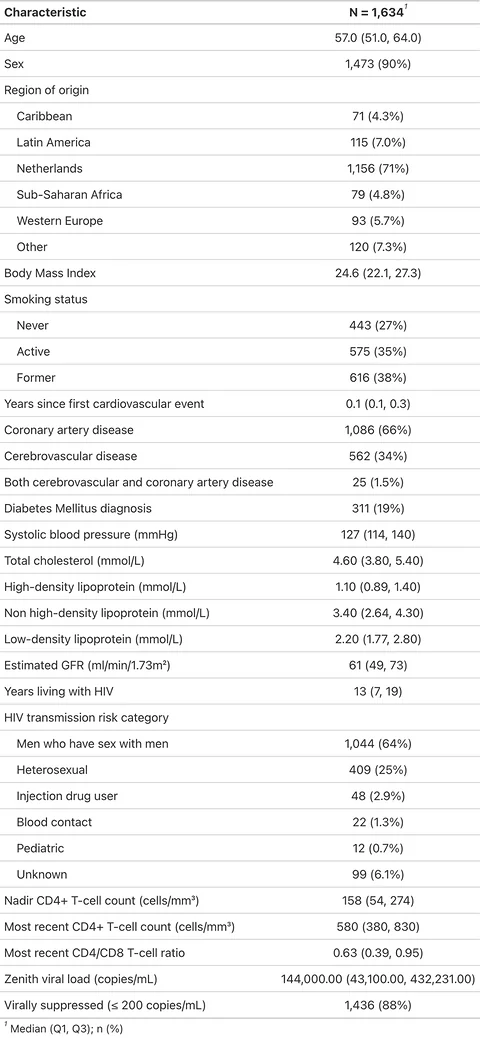
O37.1 | Baseline characteristics at study entry including demographics, medical history, cardiovascular parameters and HIV‐specific parameters.

### O37.2 Statin Use for Primary Prevention of Cardiovascular Disease Among Individuals With HIV in the Netherlands, Impact of Updated (Inter)National Guidelines

Colette Smit ^1,2^, Ferdinand Wit^1,2^, Dorine De Vries‐Sluijs^3^, Jan Den Hollander^4^, Kees Brinkman^5^, Berend van Welzen^6^, Anna Roukens^7^, Wouter Bierman^8^, Marc van der Valk^1,2^, on behalf of the Athena Observational Cohort

^1^Stichting HIV Monitoring, Amsterdam, Netherlands; ^2^Department of Infectious Diseases, Amsterdam University Medical Center, Amsterdam Institute for Infection and Immunity, Amsterdam, Netherlands; ^3^Department of Medical Microbiology & Infectious Diseases, Department of Internal Medicine, Erasmus University Medical Centre, Rotterdam, Netherlands; ^4^Department of Internal Medicine and Infectious Diseases, Maasstad Ziekenhuis, Rotterdam, Netherlands; ^5^Department of Internal Medicine and Infectious Diseases, Onze Lieve Vrouwe Gasthuis, Amsterdam, Netherlands; ^6^Department of Infectious Diseases, University Medical Centre Utrecht, Utrecht, Netherlands; ^7^Department of Infectious Diseases, Leiden University Medical Centre, Leiden, Netherlands; ^8^Department of Internal Medicine/Infectious Diseases, University Medical Centre Groningen, Groningen, Netherlands

**Background**: Cardiovascular disease (CVD) is a concerning comorbidity for individuals with HIV. The REPRIEVE study, published in 2023, led to updated EACS recommendations for statin use as primary CVD prevention in individuals with HIV. We assessed the prevalence of statin use according to the EACS recommendations and identified factors associated with statin non‐use.

**Methods**: We selected individuals in the ATHENA observational HIV cohort, 40–75 years, without prior CVD or T2DM or censored when diagnosed during follow up. Time‐updated information for 2022–2025 on age, lipid profile, blood pressure and smoking status was used. Missing time‐updated data was imputed with last observation carried forward, a maximum of 2 years was used, except for smoking. The 10‐year CVD risk was estimated using SCORE2 (aged 40–69 years) or SCORE2‐OP (aged ≥70 years) and was categorized as low/moderate, high and very high according to the EACS guideline recommendations. Statin use included a statin prescription or other lipid‐lowering drugs. Factors associated with statin non‐use (which included prior and refused statin use) were assessed by prevalence rate ratios (PRR) and 95% confidence intervals (CI), using a robust multivariable Poisson regression model.

**Results**: 15,243 individuals were included, 57% were born in the Netherlands and 82% were male. Statin use increased between 2023 and 2025 from 13% > 19% (PRR 1.46) for individuals with low/moderate risk, 22% > 32% (PRR 1.45) with high risk and from 29% > 46% (PRR 1.58) with very high risk (*p* < 0.0001, Figure 1). Among individuals with very high risk in 2025, 8% had discontinued prior statin use and 3% refused statin use. Non‐use was lower in 2025 compared to 2023 (aPRR 0.89; 95% CI: 0.88–0.90; *p* < 0.0001). When limiting to 2025, non‐use decreased with increasing age (aPRR 0.80; 0.79–0.81 per 10 year increase), was higher for women (aPRR 1.08; 1.06–1.11) and for individuals with low/moderate risk (aPRR 1.12; 1.04–1.02, *p* = 0.001) compared to very high risk, but did not differ between those with high and very high risk.

**Conclusions**: Statin use for primary CVD prevention increased since the publication of the REPRIEVE study. Since in all categories > 40% of the individuals did still not use a statin, there remains room for improvement.

**FIGURE 1:**
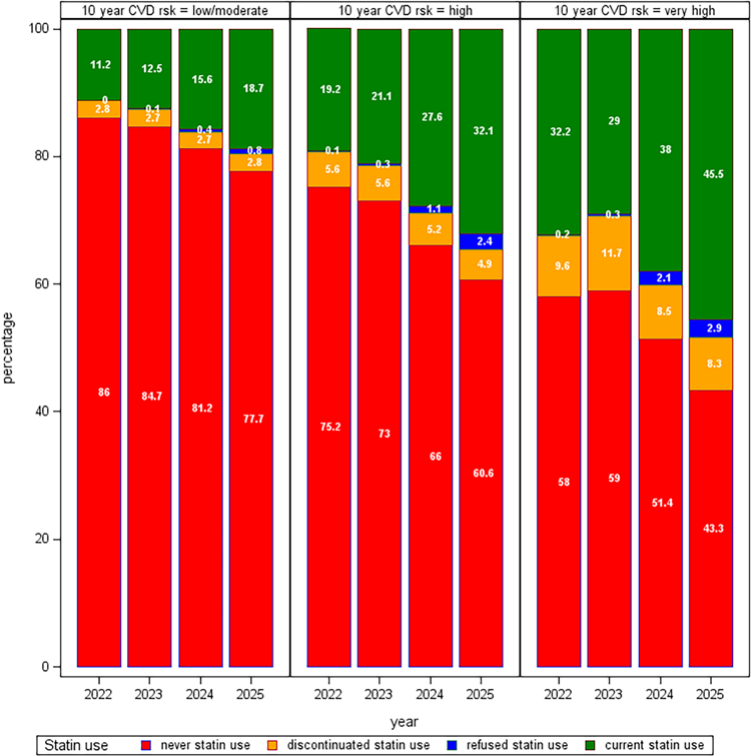
O37.2 | Current and ever use of statin among individuals with HIV aged between 40 and 75 year and in care in the Netherlands, stratified by 10‐year CVD risk using SCORE2 in individuals aged 40–69 year and SCORE2 OP in individuals ≥70 year: Low/moderate risk (aged <50 years <2.5%; 50–69 years <5%; ≥70 years < 7.5%), high (<50 years 2.5%–7.5%; 50−69 years 5%–10%; ≥70 years 7.5%–15%) and very high (<50 years ≥7.5%; 50–69 years ≥10%; ≥70 year ≥15%) 10‐year CVD risk. The numbers in the bars represent the percentages of individuals in each category.

### O37.3 T‐Cell Phenotypes and Coronary Artery Disease in People With HIV

Hans J. Hansen^1^, Moises A lberto Suarez‐Zdunek ^1^, Dina Leth Møller^1^, Ask Bock^1^, Andreas Dehlbæk Knudsen^1^, Andreas Fuchs^2^, Sisse R. Ostrowski^3,4^, Thomas L. Benfield^4,5^, Klaus F. Kofoed^2,4^, Susanne Dam Nielsen^1,4^

^1^Department of Infectious Diseases, Copenhagen University Hospital, Rigshospitalet, Copenhagen, Denmark; ^2^Department of Cardiology, Copenhagen University Hospital, Rigshospitalet, Copenhagen, Denmark; ^3^Department of Clinical Immunology, Copenhagen University Hospital, Rigshospitalet, Copenhagen, Denmark; ^4^Department of Clinical Medicine, Faculty of Health and Medical Sciences, University of Copenhagen, Copenhagen, Denmark; ^5^Department of Infectious Diseases, Copenhagen University Hospital, Amager and Hvidovre, Hvidovre, Denmark

**Background**: Living with HIV is associated with coronary artery disease (CAD), which can be accurately phenotyped using coronary computed tomography angiography (CCTA). T‐cells are important in atherogenesis, but the role of circulating T‐cells on CAD is poorly understood. We aimed to determine whether activated, apoptotic or senescent CD4+ and CD8+ T‐cells are associated with CAD and plaque phenotypes in people with HIV.

**Methods**: People with HIV in the Copenhagen Comorbidity in HIV Infection (COCOMO) study underwent a protocolized research CCTA and collection of blood samples for flow cytometry of peripheral blood mononuclear cells. We determined proportions of activated (HLA‐DR+CD38+), apoptotic (CD28−CD95+) and senescent (CD28−CD57+) CD4+ and CD8+ T‐cells. CCTAs were analysed for any CAD (maximum stenosis ≥1%) and obstructive CAD (maximum stenosis ≥50%). Furthermore, plaque volumes and their components were quantified. Multivariable regressions adjusted for age, sex, smoking status, dyslipidaemia, diabetes and current CD4+ T‐cell count determined associations of T‐cell proportions with CAD severity and plaque phenotypes.

**Results**: We included 547 people with HIV with CCTA and flow cytometry data, of whom 44% had any CAD (Table 1). Each doubling in proportions of activated CD4^+^ (aOR 0.58 [95% CI: 0.42–0.86]) and CD8^+^ T‐cells (aOR 0.66 [95% CI: 0.46–0.94]) was associated with lower odds of any CAD after adjustments. Likewise, after adjustments, each doubling of proportions of activated CD4+ and CD8+ T‐cells was associated with 52% lower total and 43% lower lipid‐rich plaque volumes, respectively (Figure 1). While each doubling of apoptotic CD4+ (OR 1.13 [95% CI: 1.00–1.27), apoptotic CD8+ (OR 1.40 [95% CI: 1.13–1.74]) and senescent CD8+ T‐cells proportions (OR 1.28 [95% CI: 1.06–1.55]) were associated with any CAD and total and lipid‐rich plaque volume, the associations did not remain significant in the adjusted models (Figure 1). No significant associations were found for obstructive CAD.

**Conclusions**: Among people with HIV, higher proportions of circulating activated, but not apoptotic or senescent T‐cells were independently associated with less CAD and plaque burden. This highlights a role for T‐cells on atherogenesis in people with HIV while also suggesting that circulating T‐cell phenotypes may differ from those previously observed within atherogenic plaques.

**FIGURE 1:**
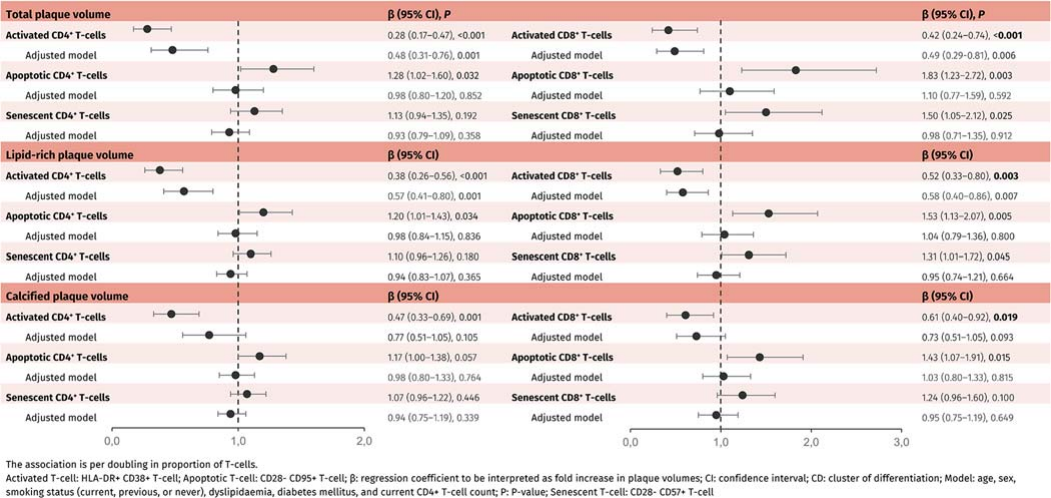
O37.3 | Associations between proportions of T‐cell subsets and plaque composition.

**TABLE 1:**
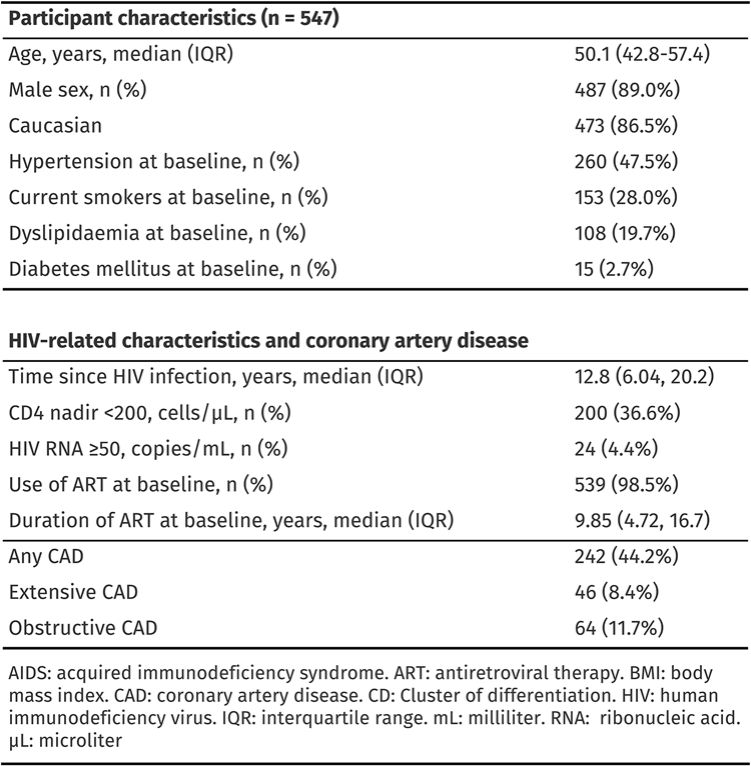
O37.3 | Baseline characteristics.

## Antiretroviral Treatment and Resistance

### O38.1 Treatment Uptake and Virological and Immunological Outcomes of Cabotegravir+Rilpivirine Long‐Acting Therapy Versus Dolutegravir or Bictegravir‐Based Regimens in People Living With HIV: RESPOND HIV Cohort Collaboration

Alisa Timiryasova ^1^, Lauren Greenberg^1^, Katharina Kusejko^2,3^, Lene Ryom Nielsen^1,4^, on behalf of the RESPOND Study Group

^1^Centre of Excellence for Health, Immunity and Infections, Rigshospitalet, University of Copenhagen, Copenhagen, Denmark; ^2^Department of Infectious Diseases and Hospital Epidemiology, University Hospital Zurich, University of Zurich, Zurich, Switzerland; ^3^Institute of Medical Virology, University of Zurich, Zurich, Switzerland; ^4^Department of Clinical Medicine, University of Copenhagen, Copenhagen, Denmark

**Background**: While randomized clinical trials and national observational studies showed high efficacy and effectiveness of cabotegravir‐rilpivirine long‐acting (CAB+RPV), there is limited real‐world data comparing CAB+RPV to contemporary antiretroviral treatment (ART). We compared uptake, virological and immunological outcomes in CAB+RPV versus dolutegravir‐based or bictegravir‐based (DTG/BIC) regimens across Europe−Australia.

**Methods**: ART‐experienced RESPOND participants switching to CAB+RPV or DTG/BIC after 1 January 2021, were followed until earliest of final follow‐up/drop out 31 August 2024. Virological and immunological outcomes were compared between groups at 6, 12, 18 months after treatment initiation. We assessed (i) composite treatment outcome, with success as HIV‐viral load (VL) <200 copies/mL (lower level of detection), available VL measurement and no ART change/AIDS/death; (ii) on‐treatment virological success: VL <200 including only individuals with available VL and no ART change; (iii) favourable immunological response: 10% CD4 increase or reaching CD4 ≥350 cells/µL. To adjust for potential confounding, we used inverse probability of treatment weighting with Poisson regression and propensity score matching with cluster‐robust standard errors.

**Results**: We studied 817 participants on CAB+RPV (median age 48 years, 82% male) and 5426 participants on DTG/BIC (median age 52, 72% male). Participants switching to CAB+RPV were younger, more frequently from Southern and less frequently from Eastern or Northern Europe (vs. Western Europe), with a higher proportion virally suppressed at treatment start (98% vs. 86%). Availability of VL measurements differed between groups: 10% CAB+RPV versus 20% DTG/BIC without VL measurement at each time point. During a median 1.3 years follow‐up (interquartile range 0.7–1.7) on CAB+RPV and 1.9 years (1.0–2.6) on DTG/BIC, overall discontinuation rates (Figure 1) and discontinuation due to treatment failure rates (1.7% and 1.5%) were similar. Crude rates of VL > 200 during follow‐up were small (CAB+RPV 2.7%, DTG/BIC 5.4%) with 68% (CAB+RPV) and 69% (DTG/BIC) spontaneously resuppressed. In adjusted analyses, virological and immunological outcomes were similar between groups, after excluding participants with no VL measurement (Table 1).

**Conclusions**: In this large cohort, viral suppression was high in both CAB+RPV and DTG/BIC groups. After accounting for differential VL measurement frequency, discontinuations and immunological and virological outcomes were similar between groups.

**FIGURE 1:**
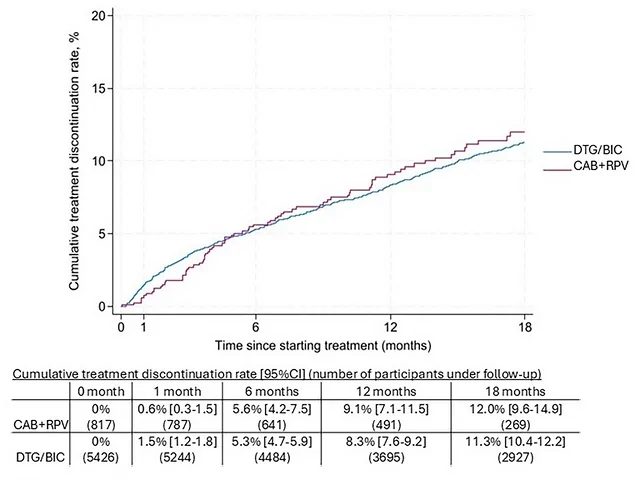
O38.1 | Kaplan–Meier estimates of cumulative treatment discontinuation for CAB+RPV versus DTG/BIC‐based regimens over 18 months.

**TABLE 1:**
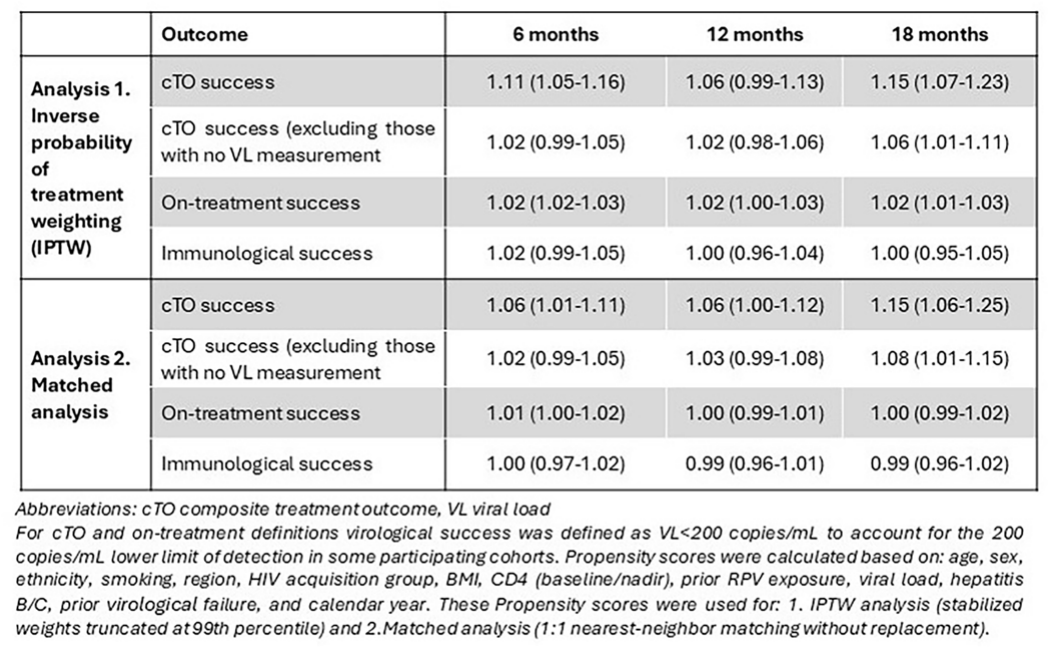
O38.1 | Virological and immunological outcomes on CAB+RPV versus DTG/BIC‐based regimens at 6, 12, 18 months, adjusted risk ratio (aRR) (95% confidence interval [CI]).

### O38.2 Long‐Term Effectiveness of Long‐Acting Cabotegravir/Rilpivirine in People With HIV Harbouring Non‐Rilpivirine NNRTI Resistance Mutations: 2‐Year Results From the RELATIVITY Cohort

David Rial‐Crestelo ^1,2^, Jara Llenas‐García^2,3^, Carmen Hidalgo‐Tenorio^4^, Jesús Troya García^5^, Noemí Cabello‐Clotet^6^, María José Galindo Puerto^7^, María Remedios Alemán^8^, Miguel Torralba González de Suso^9^, Luis Buzón^10^, Adrián Rodríguez^11^, Sonia Calzado Isbert^12^, María Aguilera García^13^, Luis Enrique Morano Amado^14^, David Vinuesa García^15^, Enrique Bernal Morell^16^, Rosa María Martínez Álvarez^17^, Analuz Fernández^18^, María del Carmen Montero Hernández^19^, Cristina Díez Romero^20^, Ruth Calderón Hernáiz^21^, Desirée Pérez Martínez^22^, Laura Gisbert Pérez^23^, Víctor Arenas García^24^, Cristina Escrich^25^, María Carmen Fariñas Álvarez^26^, Ana Lucas Dato^27^, Juan Emilio Losa García^28^, Hadrián Pernas Pardavila^29^, Juan Carlos Gainzarain^30^, Miriam Estebánez^31^, Patricia Barragán Gallo^32^, Marta Clavero Olmos^33^, Miguel Vicente Egido Murciano^34^, Óscar Luis Ferrero Benéitez^35^, Luz Martín‐Carbonero^2,3^, on behalf of the RELATIVITY Cohort Group

^1^Hospital Universitario 12 de Octubre, imas12 Health Research Institute, Madrid, Spain; ^2^CIBERINFEC, Instituto de Salud Carlos III, Madrid, Spain; ^3^Hospital Universitario La Paz, IdiPAZ, Madrid, Spain; ^4^Hospital Universitario Virgen de las Nieves, Instituto de Investigación Biosanitaria de Granada, IBS‐Granada, Granada, Spain; ^5^Hospital Universitario Infanta Leonor, Madrid, Spain; ^6^Hospital Clínico San Carlos, Madrid, Spain. Instituto de Investigación Sanitaria San Carlos, IdISSC, Madrid, Spain; ^7^Hospital Clínico Universitario de Valencia, Valencia, Spain; ^8^Hospital Universitario de Canarias, Tenerife, Spain; ^9^Hospital Universitario de Guadalajara, Guadalajara, Spain; ^10^Hospital de Burgos, Burgos, Spain; ^11^Hospital Universitario Son Llàtzer, Health Research Institute of the Balearic Islands, IdISBa, Palma de Mallorca, Spain; ^12^Parc Taulí Hospital Universitari, Sabadell, Spain. Institut d'Investigació i Innovació Parc Taulí, I3PT, Sabadell, Spain; ^13^Hospital Universitario de La Princesa, Madrid, Spain; ^14^Álvaro Cunqueiro University Hospital, Vigo, Spain; ^15^Hospital Clínico San Cecilio‐PTS, Granada, Spain; ^16^Hospital General Universitario Reina Sofía, Murcia, Spain; ^17^Hospital Universitario Miguel Servet, Zaragoza, Spain; ^18^Bellvitge University Hospital, Barcelona, Spain. Bellvitge Biomedical Research Institute, IDIBELL, Barcelona, Spain; ^19^Hospital Universitario de Torrejón, Madrid, Spain; ^20^Hospital General Universitario Gregorio Marañón, Madrid, Spain. Instituto de Investigación Sanitaria Gregorio Marañón, IiSGM, Madrid, Spain; ^21^Hospital Universitario de Fuenlabrada, Madrid, Spain; ^22^Hospital Universitario San Agustín, Avilés, Spain; ^23^Hospital Universitario Mutua Terrassa, Barcelona, Spain; ^24^Hospital Universitario de Cabueñes, Gijón, Spain; ^25^Hospital Verge de la Cinta, Tortosa, Spain; ^26^Hospital Universitario Marqués de Valdecilla, Santander, Spain; ^27^Hospital Comarcal de la Vega Baja, Alicante, Spain; ^28^Hospital Universitario Fundación Alcorcón, Madrid, Spain; ^29^Complexo Hospitalario Universitario de Pontevedra, Instituto de Investigación Sanitaria Galicia Sur, Pontevedra, Spain; ^30^Hospital Universitario de Álava, Vitoria‐Gasteiz, Spain; ^31^Hospital Central de la Defensa Gómez Ulla, Madrid, Spain; ^32^Hospital Residencia Sant Camil, Barcelona, Spain; ^33^Hospital Universitario Infanta Elena, Madrid, Spain; ^34^Hospital Universitario San Jorge, Huesca, Spain; ^35^Hospital Universitario de Basurto, Bilbao, Spain

**Background**: Long‐acting cabotegravir plus rilpivirine (CAB+RPV LA) is a highly effective maintenance therapy for people with HIV (PWH). Although European prescribing information contraindicates this combination in the presence of non‐nucleoside reverse transcriptase inhibitor (NNRTI) resistance‐associated mutations (RAMs), we previously demonstrated comparable effectiveness at 48 weeks in people with HIV (PWH) harbouring NNRTI RAMs that do not compromise RPV susceptibility (non‐RPV NNRTI RAMs) versus those without NNRTI resistance. Here, we report updated outcomes after 2 years of follow‐up.

**Methods**: This sub‐study within the nationwide Spanish RELATIVITY cohort evaluated CAB+RPV LA effectiveness in virologically suppressed adults with historical NNRTI RAMs. RAMs were interpreted using the IAS–USA criteria and the Stanford HIV Drug Resistance Database. The primary outcome was virologic failure (VF) in adults with non‐RPV NNRTI RAMs. Secondary outcomes: occurrence of viral blips and VF in two other subgroups: participants harbouring the K103N mutation and those with RPV NNRTI RAMs.

**Results**: Among 3349 participants included in the updated cohort, 1540 had an available historical genotype. 123/1540 participants (8%) had NNRTI RAMs: 95 (6.2%) had non‐RPV NNRTI RAMs and 28 (1.8%) had RPV NNRTI RAMs. K103N was the most frequent mutation (48 participants). After a median follow‐up of 26.7 months (IQR 21.1–31.1), VF was uncommon and did not differ between participants with and without non‐RPV NNRTI RAMs (1.1% vs. 1.2%, *p* = 1.000). Viral blips occurred at similar frequencies (10.5% vs. 10.7%, *p* = 1.000). In the K103N subgroup, VF occurred in 1/48 participants (2.1%), and viral blips in 4/48 (8.3%). In the RPV NNRTI RAMs subgroup, VF occurred in 1/28 participants (3.6%) and viral blips in 2/28 (7.1%).

**Conclusions**: After a median follow‐up of 26.7 months, CAB+RPV LA maintained high levels of virological suppression in PWH with NNRTI RAMs. Non‐RPV NNRTI RAMs were not associated with increased VF or viral blips. Outcomes in participants with RPV NNRTI RAMs should be interpreted cautiously because of the very small sample size and the presence of only one VF event.

### O38.3 Doravirine Susceptibility in People Living With HIV With Multi‐Class Antiretroviral Resistance: A Real‐World Cohort Analysis

Alejandro De Gea Grela^1^, José Ignacio Bernardino^1^, Luz Martín‐Carbonero^1^, Jara Llenas‐García^1^, Carmen Busca^1^, María del Mar ArcosRueda^1^, Rosa De Miguel‐Buckley^1^, Maria Luisa Montes^1^, Rafael Mican^1^, Juan Gonzalez‐Garcia^1^, Jose R. Arribas^1^, Rocio Montejano ^1,2^

^1^Hospital Universitario La Paz, IdiPAZ, Madrid, Spain; ^2^CIBERINFEC, Madrid, Spain

**Background**: Doravirine (DOR) is a next‐generation NNRTI with a resistance profile distinct from first‐generation agents: it retains activity against K103N/S but not against Y188L, L100I or V106A/M. We aimed to characterize DOR susceptibility across resistance subgroups in a large real‐world HIV cohort and evaluate its potential role in treatment simplification.

**Methods**: We analysed all plasma HIV genotypic resistance tests with documented mutations in the clinical database of Hospital Universitario La Paz in Madrid (3995 people living with HIV [PLWH] in active follow‐up). DOR susceptibility was scored using Stanford HIVdb v9.x (score <10 = susceptible; 10–29 = low/potential low‐level resistance; 30–59 = intermediate; ≥60 = high‐level resistance). The cohort was stratified by NNRTI resistance status and number of resistant drug classes.

**Results**: Of 3995 PLWH, 1701 (42.6%) had genotypic resistance data. Baseline characteristics by resistance subgroup are shown in Table 1. Among the 996 PLWH with reverse transcriptase resistance associated mutations (RT‐RAMs), DOR susceptibility was maintained in 728 (73.1%). In the 413 PLWH with NNRTI‐RAMs (24.3% of the genotyped cohort), dominant mutations were K103N/S (61.7%), Y181C (22.3%), G190A (20.3%), K101E (11.9%), L100I (5.8%) and P225H (5.6%). Despite class‐level NNRTI resistance, DOR susceptibility was retained in 212 (51.3%). Among the 275 PLWH with K103N/S, DOR susceptibility was preserved in 173 (62.9%). In PLWH with multi‐class resistance (≥2 classes; *n* = 390), DOR was susceptible in 213 (54.6%), and in the 132 HTE PLWH (≥3 classes), in 70 (53.0%). A total of 1417 PLWH (83.3%) were susceptible to both DOR and DTG, representing a large candidate population for two‐drug simplification strategies.

**Conclusions**: DOR susceptibility is preserved in nearly three‐quarters of PLWH with a reverse transcriptase genotype, and in over half of NNRTI‐resistant and HTE PLWH—driven by the high prevalence of K103N/S, which does not affect DOR. Systematic retrospective re‐scoring of historical genotypes under Stanford HIVdb reveals a substantially larger eligible population for DOR‐based two‐drug.

**TABLE 1:**
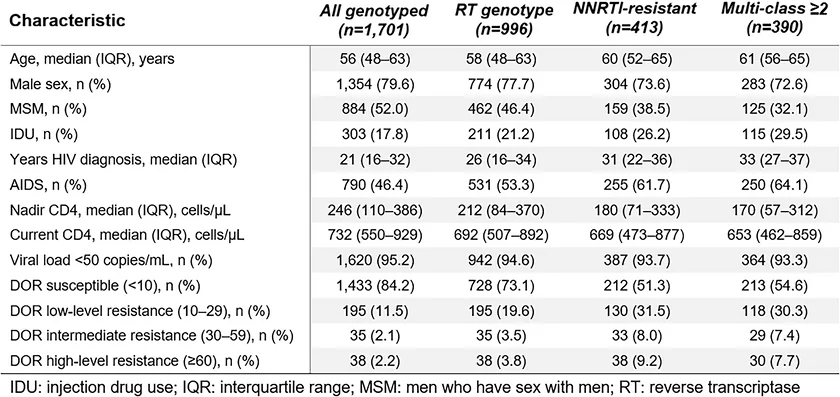
O38.3 | Baseline characteristics and DOR susceptibility by resistance subgroup.

## Under‐Served Populations

### O39.1 Viral Resistance and Antiretroviral Treatment Regimens in French Adults With Perinatally Acquired HIV‐1

Thomas Guilhou ^1^, Valentine Fihman^2^, Patricia Brazille^3^, Dominique Batisse^1^, Narjis Boukli^4^, Clémentine De la Porte des Vaux^1^, Claudine Duvivier^5^, Marina Karmochkine^1^, Odile Launay^6^, Alice‐Andrée Mariaggi^2^, Juliette Pavie^1^, Anne Rachline^1^, Laurence Slama^1^, Jean‐Paul Viard^1^, Pierre Frange^2^, Romain Palich^1^

^1^Paris Cité University, AP‐HP, Hôtel‐Dieu Hospital, Immunology and Infectiology Department, Paris, France; ^2^Paris Cité University, AP‐HP, Cochin Hospital, Department of Virology, Paris, France; ^3^Paris Cité University, AP‐HP, Hôtel‐Dieu Hospital, Espace Santé Jeunes, Paris, France; ^4^Paris Cité University, AP‐HP, Georges‐Pompidou European Hospital, Department of Virology, Paris, France; ^5^University of Paris Cité, AP‐HP, Necker Pasteur Infectiology Centre, Department of Infectious Diseases, Paris, France; ^6^Université Paris Cité Inserm, AP‐HP, Hôpital Cochin, CIC Cochin Pasteur, Paris, France

**Background**: Adults with perinatally acquired HIV‐1 (APAH) are a minority of PWH in high‐resource settings, but have experienced lifelong antiretroviral exposure, historical suboptimal regimens and high risk of virological failure. We aimed to assess the prevalence of multidrug resistance and describe current ART in this population.

**Methods**: This multicentre cohort study included APAH, defined as individuals diagnosed before age 10, followed in three French referral centres, with ≥1 follow‐up visit in 2025 and ≥1 available result of genotypic resistance test since birth. Cumulative genotypic resistance was reassessed using the ANRS‐MIE algorithm v35. Multidrug resistance (MDR) was defined as resistance to the most recent drugs in ≥2 antiretroviral classes (TDF/TAF, RPV or DOR, DRV QD and CAB/BIC/DTG QD).

**Results**: Among 233 APAH, with a median age of 29 years (IQR 24–35), including 119 (51%) women, 92% had documented HIV diagnosis at birth. At last follow‐up, median CD4 count was 637 cells/mm^3^ (IQR 451–843) and 198 (86%) had plasma HIV‐RNA <50 copies/mL. Characteristics and ART history are detailed in Table 1. Overall, 46% harboured genotypic resistance to at least one recent drug, including 24% to TDF/TAF, 32% to RPV, 25% to DOR, 6.9% to DRV QD and 14% to CAB/BIC/DTG QD. MDR was identified in 59 (25%) of APAH and 43 (18%) had a genotypic susceptibility score (GSS) <2 to their current ART. Compared to APAH without MDR, those with MDR were less likely to receive INSTI‐based three‐drug regimens (17% vs. 67%, *p* < 0.001) and to be treated with single‐tablet regimens (32% vs. 88%, *p* < 0.001). They were more frequently receiving two‐drug regimens (54% vs. 18%, *p* < 0.001) and boosted PI‐based regimens (47% vs. 8.6%, *p* < 0.001). They were more likely to have a GSS <2 (53% vs. 7%, *p* < 0.001). However, the proportion of APAH with last plasma HIV‐RNA ≥50 copies/mL did not differ in case of MDR versus no MDR (10% vs. 13%, *p* = 0.62).

**Conclusions**: One quarter of APAH harboured MDR HIV, yet virological suppression remained high. This success relies on more complex treatment strategies, highlighting the need for treatment simplification and access to novel antiretrovirals in this young population with a lifelong need for effective therapy.

**TABLE 1:**
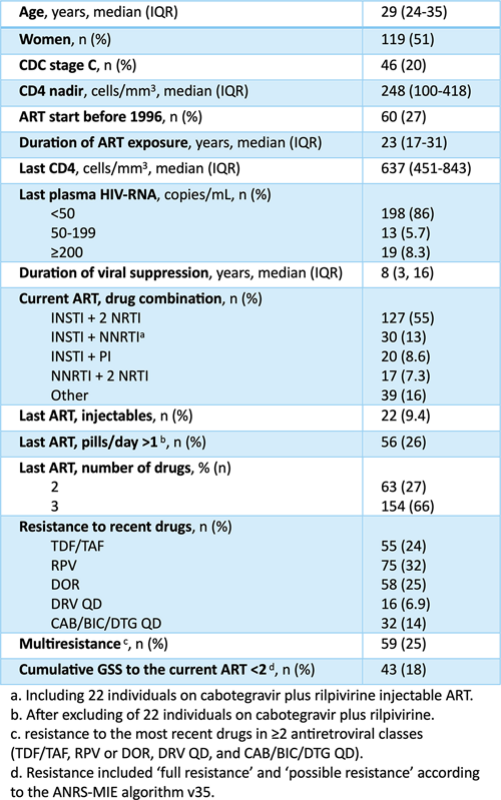
O39.1 | Participant characteristics (*N* = 233).

### O39.2 At the Margins of the Fringe: Intersectional and Structural Inequities in HIV Testing and Healthcare Linkage Among Migrants Across 10 European Countries—A Community‐Led Bio‐Behavioural Cross‐Sectional Study

Haoyi Wang ^1,2^, Hala Majdoub^3^, Anne Flaherty‐Gupta^3^, Kim Isip^4^, Pedro Barrios Sanchez^5^, Francesco Bellissimo^6^, Yiannis Charilaou^7^, Christos Krasidis^7^, Rosaline M'Bayo^8^, Stephen Amoah^8^, Trésors Kouadio^9^, Robert Ian Kibet Ouko Babu^10^, Ann Piercy^11^, Abimbola Ajomale^12^, Moro Yapha^13^, Etinosa Erevbenagie‐Johnbull^14^, Letonde Hermine Gbedo^15^, Kai Jonas^1^, Hanne Zimmermann^1^, Denis Onyango^3^

^1^Department of Work and Social Psychology, Maastricht University, Maastricht, Netherlands; ^2^Viroscience Department, Rotterdam, Netherlands; ^3^Africa Advocacy Foundation, London, UK; ^4^Association PASTT, Paris, France; ^5^Asociacion Karibu, Madrid, Spain; ^6^LHIVE Diritti e Prevenzione, Catania, Italy; ^7^AIDS Solidarity Movement, Nicosia, Cyprus; ^8^Afrikaherz (VIA e.V.), Berlin, Germany; ^9^Plateforme Prévention SIDA, Brussels, Belgium; ^10^Open Paths Athens, Athens, Greece; ^11^HIV Ireland, Dublin, Ireland; ^12^Noaks Ark Mosaik, Malmö, Sweden; ^13^Fixpunkt e.V., Berlin, Germany; ^14^Generation for Change CY, Nicosia, Cyprus; ^15^Comitato per i Diritti Civili delle Prostitute, Trieste, Italy

**Background**: Migrants in Europe face persistent structural barriers to HIV testing and care. We assessed HIV testing history, screening outcomes and linkage to healthcare among migrants attending community‐based outreach, and examined intersectional inequalities in prior HIV testing history.

**Methods**: We analysed bio‐behavioural data collected between September 2024 and October 2025 across 10 European countries. Rapid HIV testing and surveys were delivered by community‐based organizations in migrant and refugee centres, faith‐based organizations and community events. Outcomes included prior HIV testing, reactive screening results and linkage to confirmatory testing or other healthcare services. Univariable logistic regression examined associations between migrant status and prior testing. Among migrants, Multilevel Analysis of Individual Heterogeneity and Discriminatory Accuracy (MAIHDA) models were fitted across 56 intersectional strata defined by legal status, key population, sex work and injection drug use (IDU), adjusting for residence duration and national testing policies.

**Results**: Among 2535 participants, 88.6% were migrants, of whom 1009 (44.9%) had insecure legal status (525 asylum seekers, 163 refugees, 321 undocumented migrants). Only 52.4% (95% CI: 50.5–54.4) reported prior HIV testing; migrants were significantly less likely to have tested than non‐migrants (50.6% vs. 66.2%; OR 0.52, 95% CI: 0.40–0.68). By legal status, testing was lowest among asylum seekers (45.0%); by key population, highest among MSM (86.6%). Thirty‐seven reactive results were identified (1.5%, 95% CI: 1.0–1.9); by legal status, reactivity was highest among undocumented migrants (2.5%) and by key population, among MSM (5.7%). Of those with reactive results, 86.5% were linked to confirmatory testing, including 87.5% of undocumented migrants. Migrants with non‐reactive results were more frequently referred to additional healthcare than non‐migrants (47.6% vs. 35.0%, Figure 1). MAIHDA revealed intersectional strata explained 40.9% of variance in prior testing (AUC—ROC = 0.730), fully accounted for by additive effects. Undocumented heterosexual men who were non‐sex workers and non‐IDU had the lowest predicted testing probability; MSM asylum seekers/refugees who were sex workers and IDU had the highest (Table 1).

**Conclusions**: Persisting inequalities in HIV testing are driven by behavioural, legal and policy‐level determinants. Yet, community‐based outreach achieved strong linkage to confirmatory testing and broader healthcare among structurally marginalized migrants. Expanding inclusive national testing policies and migrant‐centred outreach is essential to closing Europe's HIV testing gap.

**FIGURE 1:**
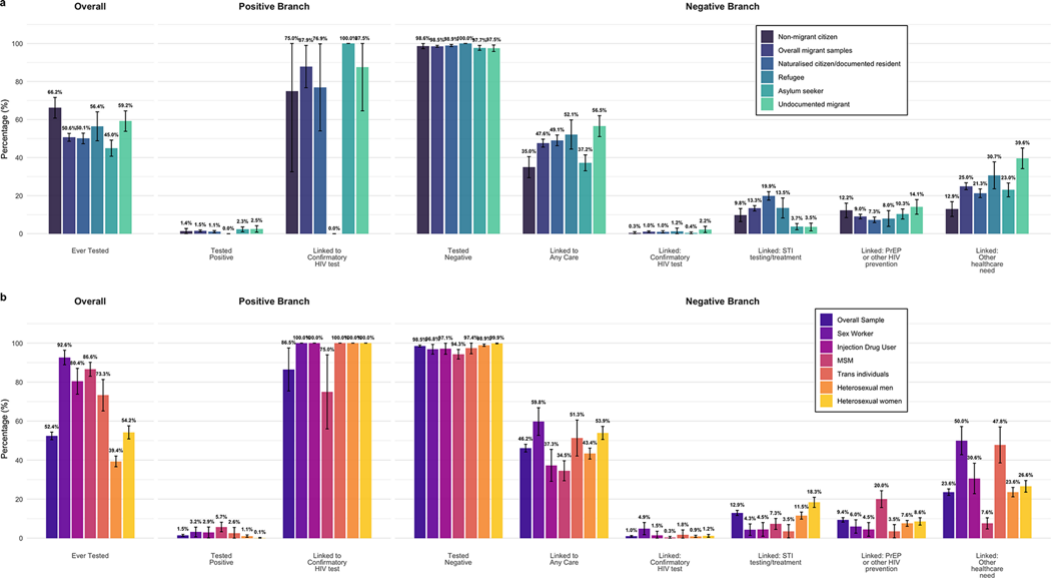
O39.2 | Prior HIV testing history and linkage to healthcare based on testing results across (a) migrant status and legal status and (b) key populations.

**TABLE 1:**
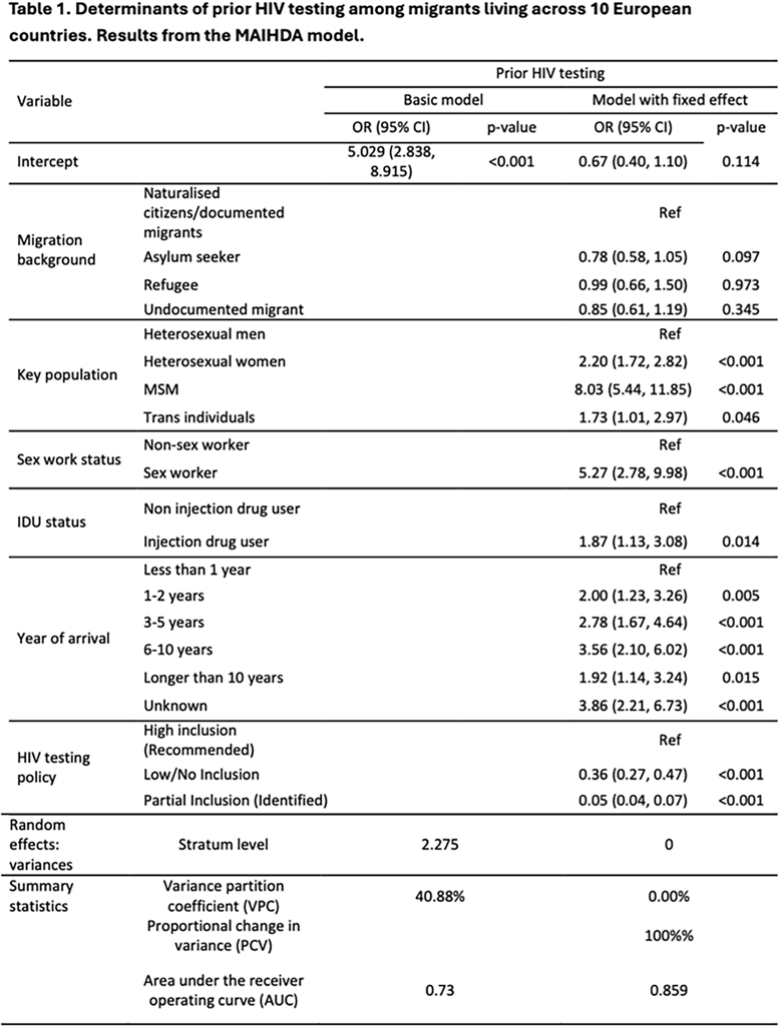
O39.2 | Determinants of a prior HIV testing among migrants living across 10 European countries; results from the MAIHDA models.

### O39.3 Addressing the Data Gap on Recent Migrants and HIV: Understanding the Experiences and Long‐Acting Treatment Preferences of Recent Migrants Living With HIV From the Ask Us Europe Survey

Megan Devonald ^1^, Chloé Pasin^1^, Melanie Smuk^1^, Rebecca Mbewe^1^, Angelina Namiba^2^, Marcel Dams^3^, Matthew Hodson^4^, Nicoletta Policek^5^, Alain Volny Anne^6,7^, Luís Mendão^8,9^, Magdalena Ankiersztejn‐Bartczak^10^, Juddy Otti^11^, Casper Rokx^12^, Frederico Duarte^13^, Sophie Strachan^1,11^, Marc van der Valk^14^, David Jilich^15,16^, Bogusz Aksak‐Wąs^17^, Christophe Rouquette^18^, Sarah Brunel^18^, Thierry Tran^18^, Ann Piercy^19^, Simona Saluzzo^20^, Chloe Orkin^1^, on behalf of Ask Us Steering Group

^1^Queen Mary University of London, London, UK; ^2^4 M Mentor Mothers, London, UK; ^3^Independent Sexual Counsellor and Sexual Educator, Berlin, Germany; ^4^Independent, London, UK; ^5^European Community Led Observatory on Ageing and HIV, Edinburgh, UK; ^6^Ask Us Steering Group, Paris, France; ^7^EATG, Paris, France; ^8^Ask Us Steering Group, Lisbon, Portugal; ^9^GAT Portugal, Lisbon, Portugal; ^10^Foundation for Social Education, Warsaw, Poland; ^11^Sophia Forum, London, UK; ^12^Erasmus University Medical Center, Rotterdam, Netherlands; ^13^Hospital Pedro Hispano, Matosinhos, Portugal; ^14^Amsterdam UMC, Amsterdam, Netherlands; ^15^Charles University, Prague, Czechia; ^16^University Hospital Bulovka, Prague, Czechia; ^17^Pomeranian Medical University, Szczecin, Poland; ^18^TRT‐5 CHV, Paris, France; ^19^HIV Ireland, Dublin, Ireland; ^20^Medical University of Vienna, Vienna, Austria

**Background**: Recent migrants (<5 years) are disproportionately affected by HIV and can face significant disparities in accessing healthcare. We explored treatment optimization discussions and awareness/interest in long‐acting treatments including long‐acting injectable cabotegravir and rilpivirine (CAB‐RPV).

**Methods**: Between 22 July 2025 and 20 May 2026, 1935 people on antiretroviral therapy completed an online equity‐focused, co‐produced survey in 18 European countries and languages, developed with 18 community members, the European AIDS Treatment Group and the UK Community Advisory Board. Dissemination was via social media and community/clinical networks. We compared participants by migration status (recent migrants <5 years in new country vs. non‐migrants) using descriptive statistics.

**Results**: Out of 1658 participants reporting migration status, 167 (10.1%) were recent migrants, including 70 (44%) from the European Region, 52 (33%) from Americas Region (Table 1). Compared to the non‐migrants (1203), recent migrants were more likely to be recently diagnosed (<7 years) (36% vs. 15%), aged <45 (74% vs. 30%), transgender, non‐binary or gender diverse (11% vs. 7%), financially unstable (49% vs. 24%), in shared/insecure housing (28% vs. 4.2%), neurodivergent (13% vs. 8%), had a university degree (63% vs. 49%) and had missed a dose unintentionally (37% vs. 19%). 72% received or needed tailored specialist support (e.g. housing, meal/foodbank services, domestic violence) in the past year (vs. 44%). 86% of recent migrants were clinically eligible for CAB‐RPV. 15% had ever received CAB‐RPV versus 19% non‐migrants. 29% never had a treatment optimization discussion with their HCP (vs. 17%). Among those who had these discussions, CAB‐RPV discussion rates were similar (43%). Recent migrants more frequently first heard of CAB‐RPV through news/social media (42% vs. 26%) and less frequently from their HCP (25% vs. 36%). Recent migrants reported higher interest in CAB‐RPV (80% vs. 53%) and long‐acting injectable 4–6 months (92% vs. 74%), and similar interest in weekly pills (54% vs. 55%) (Figure 1).

**Conclusions**: Recent migrants were less likely to have treatment optimization discussions with healthcare providers and more likely to learn about CAB‐RPV through non‐clinical sources, highlighting persistent inequities. They reported more missed doses and showed high interest in long‐acting injections. Some results might be confounded by factors such as age, housing, financial stability.

**FIGURE 1:**
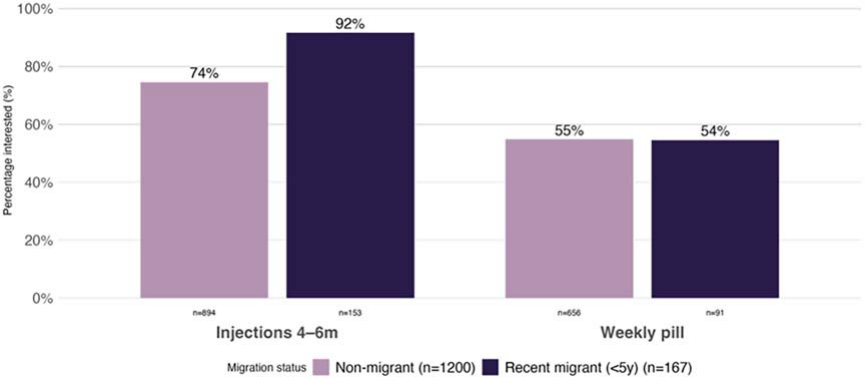
O39.3 | Percentage of participants interested in future long‐acting modalities by migration status.

**TABLE 1:**
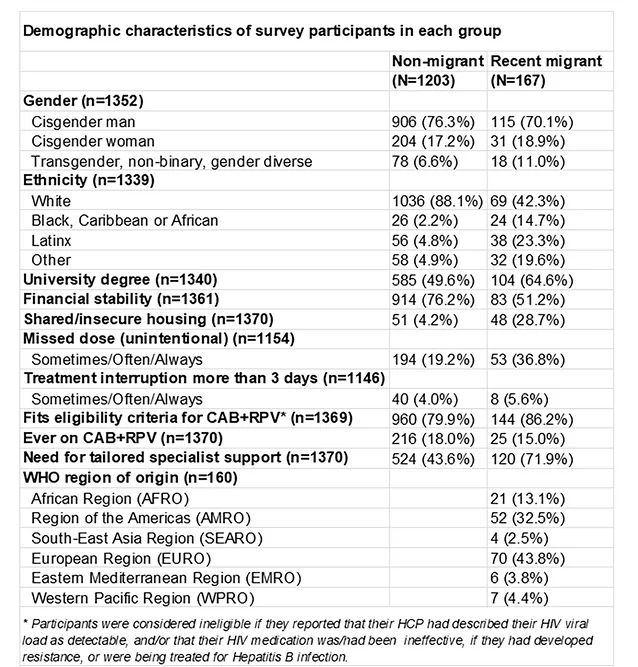
O39.3 | Demographic characteristics of survey participants in each group.

## Hot Topics

### O41 Abstract Withdrawn

### O42 Dolutegravir Resistance Among People With Repeated Viraemia in a Prospective Routine HIV Care Cohort in Lesotho

Anna Klicpera^1^, Maren Röhl^1^, Blaise Lukau^2^, Giuliana Sanchez Samaniego^1^, Moleboheng Mokebe^2^, Lipontso Motaboli^2^, Irene Ayakaka^2^, Roger Kouyos^3,4^, Gioia Wick^3,4^, Tom Loosli^3,4^, Richard Lessells^5,6^, Shirelle Janine Naidoo^6^, Jennifer Giandhari^6^, Nadine Tschumi^1^, Jennifer A. Brown^1,3^, Niklaus D. Labhardt ^1^

^1^Department of Clinical Research, University Hospital Basel, Basel, Switzerland; ^2^SolidarMed Lesotho, Maseru, Lesotho; ^3^Department of Infectious Diseases and Hospital Epidemiology, University Hospital Zurich, Zurich, Switzerland; ^4^Institute of Medical Virology, University of Zurich, Zurich, Switzerland; ^5^Centre for the AIDS Programme of Research in South Africa (CAPRISA), University of KwaZulu‐Natal, Durban, South Africa; ^6^KwaZulu‐Natal Research Innovation and Sequencing Platform, University of KwaZulu‐Natal, Durban, South Africa

**Background**: Dolutegravir is the cornerstone of antiretroviral therapy across Africa, but emerging resistance threatens its long‑term effectiveness [1]. Published reports often lack clear denominators, hindering reliable resistance prevalence estimates. We aimed to estimate the prevalence of dolutegravir resistance and identify its correlates among people with repeated viraemia in routine HIV care in Lesotho and assess whether it differs from the prevalence found in 2023 in the same cohort [2].

**Methods**: The Viral Load Cohort North‑East Lesotho (VICONEL) prospectively enrols all people receiving viral load monitoring in three districts of Lesotho [3]. This study includes VICONEL participants who fulfilled the following criteria to trigger genotypic resistance testing (GRT): written informed consent for further use of biobanked samples, and two or more viral loads ≥50 copies/mL, of which at least one was ≥500 copies/mL, with samples taken > 90 days after dolutegravir initiation. Next‑generation sequencing of the integrase gene was performed; drug‑resistance mutations (DRMs) present at ≥5% frequency were interpreted using the Stanford HIV Drug Resistance Database v10.2. We further analysed the prevalence of dolutegravir resistance in the subgroup meeting World Health Organization (WHO) criteria for virological failure (two consecutive viral loads ≥1000 copies/mL).

**Results**: By 4 June 2026, 27,373 participants had received dolutegravir and been followed in VICONEL for ≥12 months; 1007 met GRT criteria and integrase gene sequences are available from 303 (30.1%). The prevalence of at least low‐level dolutegravir resistance was 7.9% (24/303, 95% CI: 5.2–11.6). Table 1 presents characteristics of participants with and without dolutegravir resistance. Participants with dolutegravir resistance tended to be on a non‐tenofovir containing regimen, to have a history of viraemia on preceding regimens and to have resistance in the reverse transcriptase gene. DRM locations are summarized in Figure 1. Among 142 participants meeting WHO virological failure criteria, 14 (9.9%, 95% CI: 5.5–16.1) had at least low‑level dolutegravir resistance.

**Conclusions**: In this routine care cohort, dolutegravir resistance was detected in 7.9% of people with repeated viraemia and in 9.9% of people meeting WHO virological failure criteria. These findings do not indicate an increase in dolutegravir resistance relative to our earlier analysis with data closure in 2023 [2].

**References**

1. Loosli T, Han N, Hauser A, Josi J, Ingle SM, van Sighem A, Wittkop L, Vehreschild J, Ceccherini‐Silberstein F, Maartens G, Gill MJ, Sabin CA, Johnson LF, Lessells R, Günthard HF, Egger M, Kouyos RD. Predicted dolutegravir resistance in people living with HIV in South Africa during 2020−35: a modelling study. Lancet Glob Health. 2025 Apr;13(4):e698‐e706. doi: 10.1016/S2214‐109X(24)00553‐9.

2. Tschumi N, Lukau B, Tlali K, Motaboli L, Kao M, Kopo M, Haenggi K, Mokebe M, Naegele K, Ayakaka I, Leuzinger K, Brown JA, Labhardt ND. Emergence of acquired dolutegravir resistance in treatment‐experienced people with HIV in Lesotho. Clin Infect Dis. 2024 Nov 22;79(5):1208‐1222. doi: 10.1093/cid/ciae185.

3. Brown JA, Motaboli L, Lerotholi M, Kohler M, Haenggi K, Mokete M, Chakela MG, Kao M, Kopo M, Mokebe M, Urda L, Nsakala BL, Lukau B, Ayakaka I, Amstutz A, Ehmer J, Klicpera A, Klimkait T, Glass T, Muhairwe J, Chammartin F, Tschumi N, Labhardt ND. Cohort profile: the Viral load Cohort North‐East Lesotho (VICONEL) from 2016 to 2023 ‐ cohort description, test volumes, predictors of viraemia and the road ahead. BMJ Open. 2025 Feb 6;15(2):e085404. doi: 10.1136/bmjopen‐2024‐085404.

**FIGURE 1:**
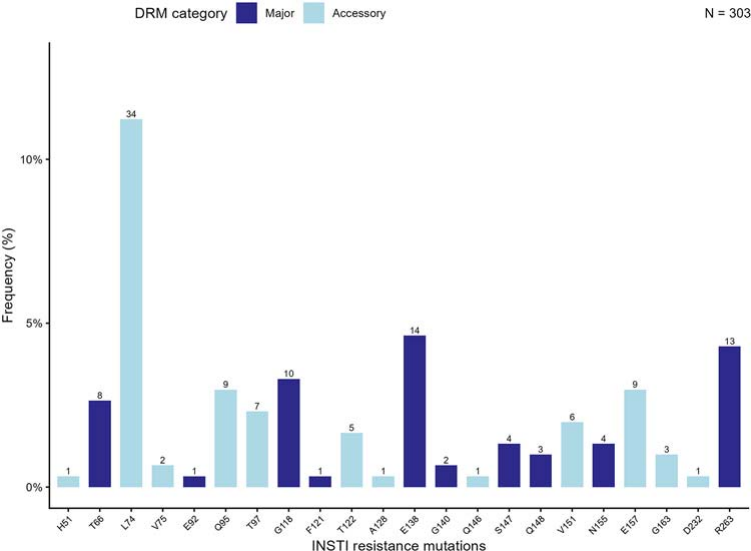
O42 | Frequency of integrase strand transfer inhibitor (INSTI) resistance‐associated mutations (RAM) at each codon position across the 24 participants with dolutegravir resistance.

**TABLE 1:**
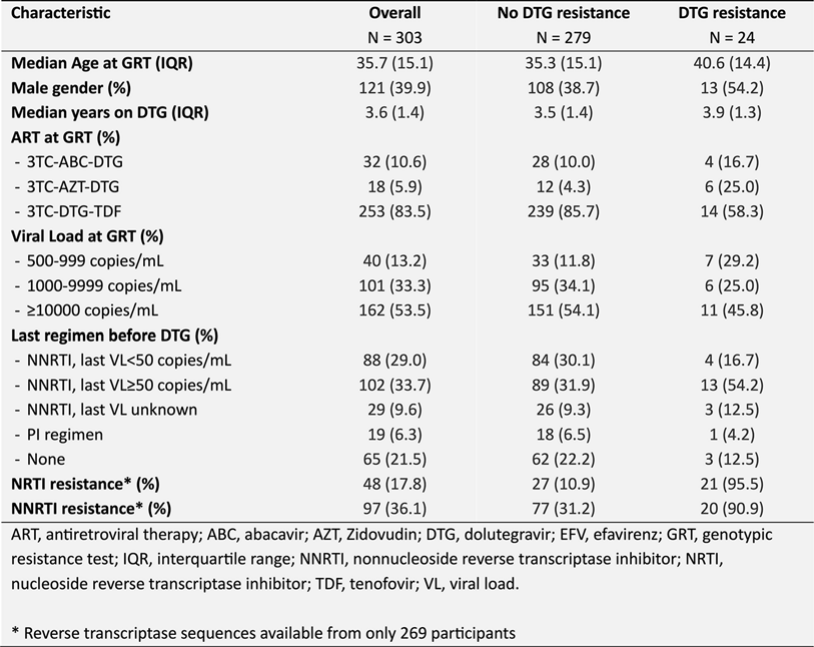
O42 | Participant characteristics at time of genotypic resistance test (GRT), stratified by dolutegravir resistance. ABC, abacavir; ART, antiretroviral therapy; AZT, zidovudine; DTG, dolutegravir; EFV, efavirenz; IQR, interquartile range; NNRTI, non‐nucleoside reverse transcriptase inhibitor; NRTI, nucleoside reverse transcriptase inhibitor; TDF, tenofovir; VL, viral load.

### O43 Pharmacokinetics of Long‐Acting Injectable Cabotegravir in Pregnant and Postpartum Women in South Africa

Dvora Joseph Davey^1,2^, Lauren Jennings^3^, Rufaro Mvdudu^3^, Chantel Schreuder^3^, Elton Mukonda^4^, Nyiko Mashele^3^, Lubbe Wiesner^4^, Landon Myer^4^, Saye Khoo^5^, Catriona Waitt^5^, Linda‐Gail Bekker^3^, Catherine Orrell ^3,6^

^1^Infectious Disease Medicine, Los Angeles, CA, US; ^2^Epidemiology and Biostatistics, Cape Town, South Africa; ^3^Desmond Tutu Health Foundation, Cape Town, South Africa; ^4^University of Cape Town, Cape Town, South Africa; ^5^University of Liverpool, Liverpool, UK; ^6^South Africa Medical Research Centre, Durban, South Africa

**Background**: Pregnant and postpartum women in sub‐Saharan Africa are at elevated risk of HIV acquisition. Acute maternal HIV infection increases the risk of vertical transmission. Pharmacokinetic (PK) data in non‐pregnant women and those initiating long‐acting cabotegravir (CAB‐LA) before pregnancy may not reflect data of those who initiate in later pregnancy. Muscle blood flow and body mass index (BMI) are altered in pregnancy which may impact the pharmacokinetics of CAB‐LA.

**Methods**: We enrolled a cohort of pregnant participants who elected to initiate CAB‐LA. Eligible participants (> 18 years, confirmed HIV‐negative, confirmed 8−20 weeks’ gestation via ultrasound, without contraindications to CAB‐LA, willing to participate in study) were followed over a period of ∼52 weeks from enrolment to 20−32 weeks postpartum (including peripartum delivery time). Maternal samples were collected before injection 2 (week 4) and at 1, 4, 8 weeks after injection 3, injection 4 (if still pregnant) and injection 7 postpartum. Samples were analysed with validated LC‐MS/MS methods. We evaluated the area under the curve and differences between pregnancy and postpartum in same women via geometric mean ratio (GMR).

**Results**: We enrolled 40 women in the study. Mean age was 24 years (SD 3.4), gestational age was 15 weeks (SD 4), BMI was 27 (SD 7.4; 23% with > 30 BMI). Overall, 35% enrolled in first‐trimester; 55% were primigravid; 48% did not know their partner's serostatus; 10% reported recent condom use; 25% previously used oral PrEP. Two women refused baseline CAB‐LA injection, one withdrew after first injection and were excluded from PK analysis. Overall, 78.4% (29/37) completed study follow‐up. Six women did not have postpartum samples drawn. Week 4 trough was 0.693 µg/mL (CAB minimum effective concentration, MEC, 0.644 µg/mL). C_max_, C_trough_ and AUC₀_‐_₈_x_ in pregnancy were 1.53 µg/mL, 1.02 µg/mL and 9.57 µg/mL weeks, compared to 3.49 µg/mL, 2.70 µg/mL and 23.0 µg/mL weeks, respectively, in postpartum. Pregnancy drug concentrations were 40% lower than postpartum (AUC₀_‐_₈_x_ GMR 0.43, 90% CI 0.37–0.51) (Figure 1).

**Conclusions**: CAB‐LA concentrations were lower in pregnancy than postpartum but remained above the MEC. Although adherence to dose is critical with long‐acting formulations due to the long pharmacokinetic “tail,” retention even in our clinical trial setting was moderate, highlighting need for targeted PrEP‐delivery in postpartum.

**FIGURE 1:**
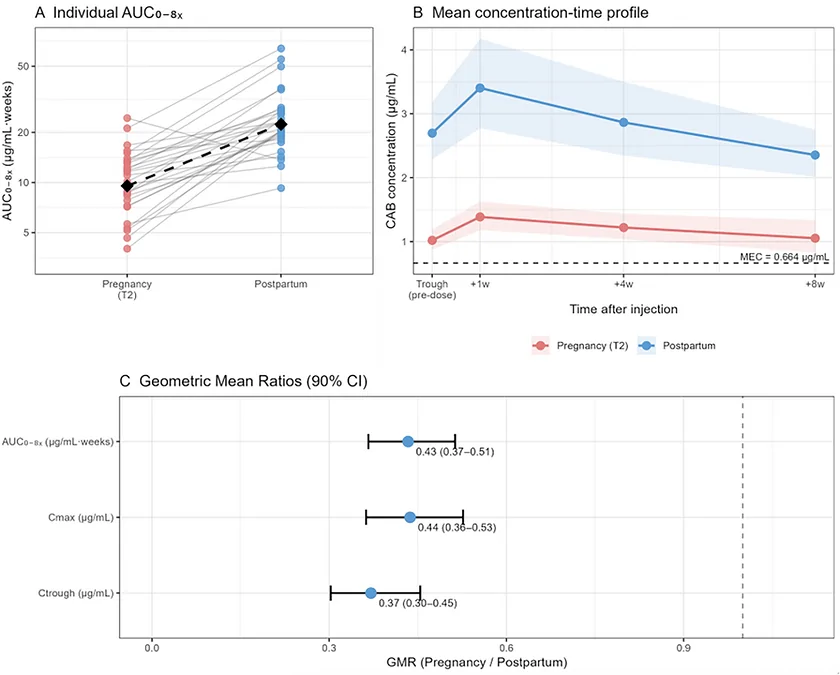
O43 | Cabotegravir pharmacokinetic parameters in pregnancy and post‐partum.

### O44 Real‐World Effectiveness of Long‐Acting Cabotegravir and Rilpivirine in People With HIV‐1 Subtype A6: Preliminary Results From the Multinational LACRIS Study

Miłosz Parczewski ^1,2^, Aleksandra Szymczak^3^, Iwona Cielniak^4,5^, Ewa Siwak^4,6^, Amelie Berner^7^, David Jilich^8^, Zuzana A. Lakatošová^8^, Wiktor Łukasik^3^, Maria Hlebowicz^9^, Anita Olczak^10^, Bartosz Szetela^3,11^, Bogusz Aksak‐Wąs^1^, Elżbieta Mularska^12^, Paweł Jakubowski^13^, Karolina Sorbian‐Gajewska^14^, Anders Boyd^15,16^, Pavel Khaykin^7^

^1^Chair and Department of Infectious Diseases, Pomeranian Medical University, Szczecin, Poland; ^2^Regional Centre for Digital Medicine, Szczecin, Poland; ^3^All Saint's Clinic, Wrocławskie Centrum Zdrowia SP ZOZ, Wrocław, Poland; ^4^Hospital for Infectious Diseases, Warsaw, Poland; ^5^Faculty of Medicine, Collegium Medicum, Cardinal Stefan Wyszyński University, Warsaw, Poland; ^6^Department of Infectious and Tropical Diseases and Hepatology, Warsaw Medical University, Warsaw, Poland; ^7^MainFachArzt, Frankfurt on Main, Germany; ^8^Department of Infectious Diseases, first, second and third Medical Faculty, Charles University and Faculty Hospital Bulovka, Prague, Czechia; ^9^Department of Family Medicine and Infectious Diseases, University of Warmia and Mazury, Olsztyn, Poland; ^10^Department of Infectious Diseases and Hepatology, Faculty of Medicine, Ludwik Rydygier Collegium Medicum, Nicolaus Copernicus University, Bydgoszcz, Poland; ^11^Department of Infectious Diseases, Liver Diseases and Acquired Immune Deficiencies, Wrocław Medical University, Wrocław, Poland; ^12^Department of Infectious Diseases, Regional Hospital Chorzów, Chorzów, Poland; ^13^Szpitale Pomorskie, Gdańsk, Poland; ^14^Clinical Trials Support Center, Pomeranian Medical University, Szczecin, Poland; ^15^Department of Infectious Diseases, Inselspital, Bern University Hospital, University of Bern, Bern, Switzerland; ^16^Department of Infectious Diseases, Amsterdam Institute for Infection & Immunity (AII) Amsterdam UMC, University of Amsterdam, Amsterdam, Netherlands

**Background**: HIV‐1 subtype A6 has been identified as a potential risk factor for confirmed virological failure (CVF) in pooled analyses of registrational clinical trials (FLAIR/ATLAS/ATLAS‐2 M), but real‐world evidence remains limited. The prevalence of subtype A6 in Europe is increasing, driven by ongoing transmission and migration from Eastern Europe, emphasizing the need for evidence on the effectiveness of long‐acting cabotegravir/rilpivirine (CAB/RPV LA) in this population. We present interim results from LACRIS, a multinational study evaluating CAB/RPV LA outcomes in people with subtype A6 case‐matched to subtype B.

**Methods**: LACRIS (NCT07568730) is an ongoing, multicentre, retrospective real‐world study in Poland, Germany and the Czech Republic including people with subtype A6 or B who received ≥1 CAB/RPV LA injection. Data on demographic and clinical characteristics, treatment exposure, injection schedule adherence, virological and clinical outcomes, and resistance patterns at CVF were collected.

**Results**: Overall, 69 people with subtype A6 and 140 subtype B initiated CAB/RPV LA. People with A6 versus B subtype, respectively, were more often women (23.2% vs. 8.6%, *p* = 0.004) and had migrated from Ukraine (29.0% vs. 1.5%, *p* < 0.001) (Table 1). Median numbers of CAB/RPV doses received were 8 (interquartile range [IQR] 6–16) and 12 (IQR 5–17), respectively (*p* = 0.18). Overall, 30 (14.4%) discontinued CAB/RPV LA, with no difference between subtypes (A6, 14.5% vs. B, 14.3%; *p* = 0.23). During a median follow‐up of 19.4 months (IQR 8.6–31.5), 5 (7.2%) people with subtype A6 and 5 (3.6%) with subtype B experienced CVF (incidence rate 4.5/100 person‐years, 95% confidence interval [CI] 1.9–10.9 and 2.0/100 person‐years, 95% CI: 0.8–4.7, respectively; log‐rank test, *p* = 0.16). The cumulative percent without CVF at 12 and 24 months, respectively, were 96.7% (95% CI: 87.2–99.2) and 91.3% (95% CI: 77.9–96.8) for A6, versus 97.1% (95% CI: 91.3–99.1) and 95.7% (95% CI: 88.9–98.4) for B (Figure 1). All participants with CVF had resistance to CAB and/or RPV at failure; 80% had dual‐class resistance. No clear subtype‐specific resistance patterns were observed, apart from the predominance of L74I in A6 (Figure 1).

**Conclusions**: Our preliminary results suggest the negative effect of A6 on the utility of CAB/RPV may be considerably less substantial than previously reported but prospective long‐term observational data are needed. These data are important for optimal care of the growing subtype A6 population in Europe.

**FIGURE 1:**
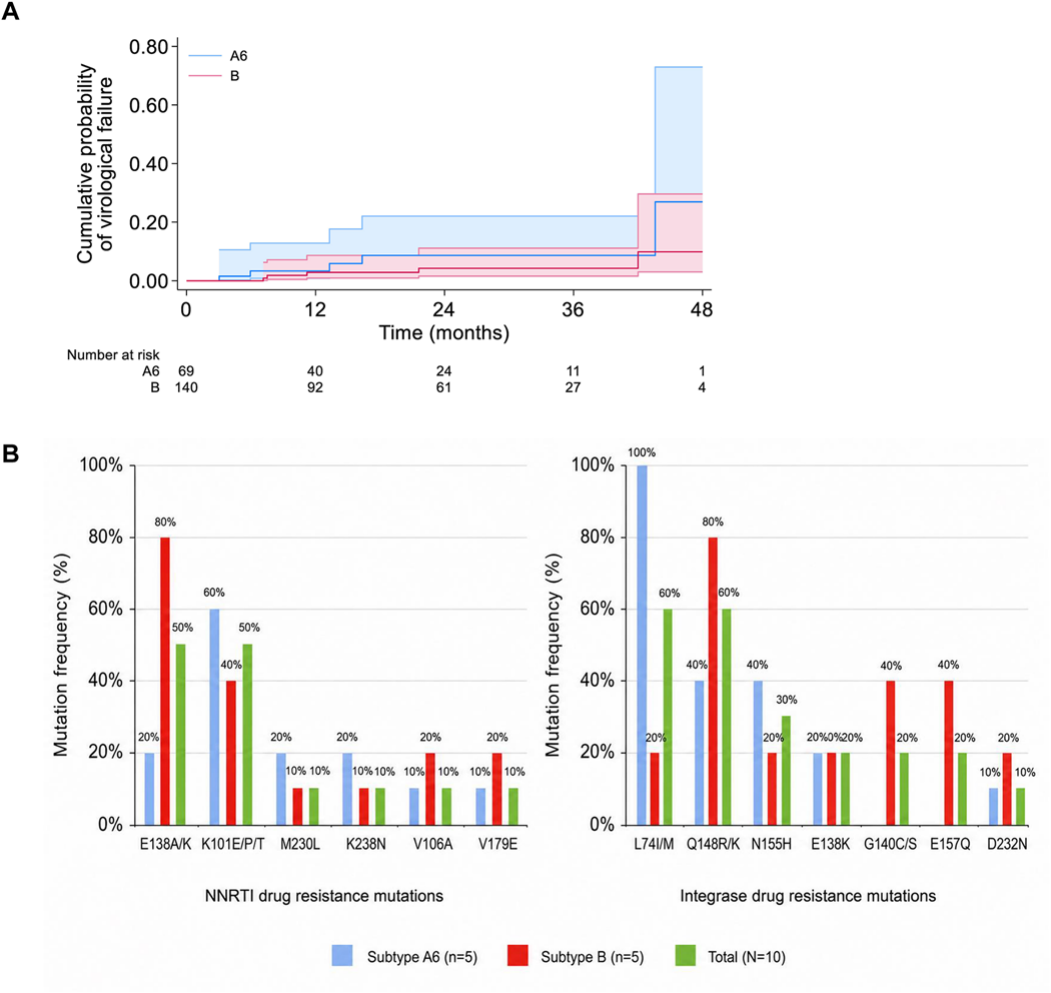
O44 | (A) Kaplan–Meier estimate for cumulative probability of virological failure by subtype. Numbers at risk are provided below the figure. (B) Drug resistance mutations in virologically failing patients by antiretroviral drug class and subtype.

**TABLE 1:**
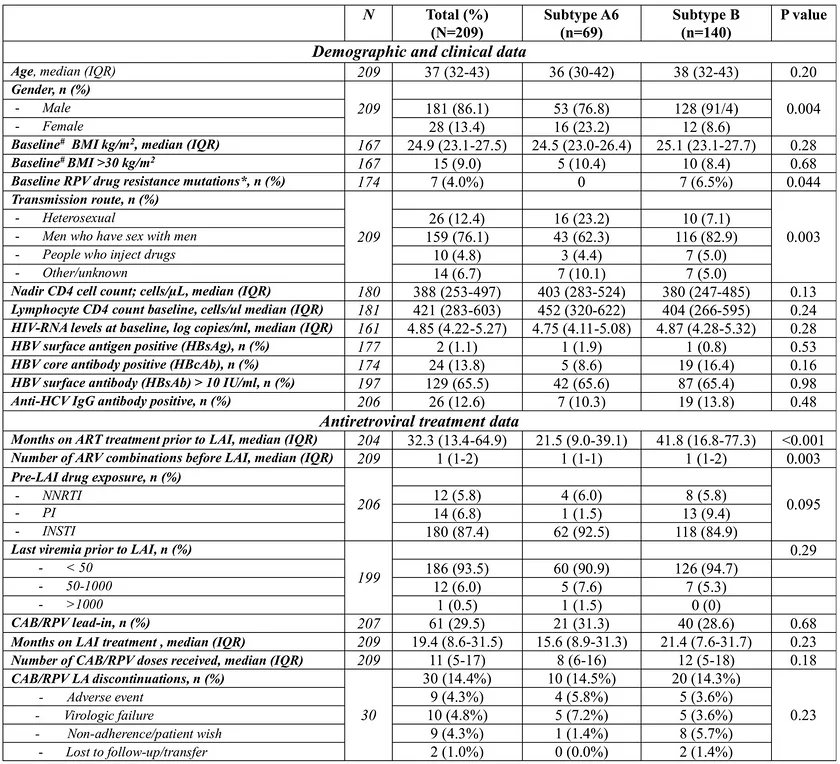
O44 | Clinical characteristics of the study group by subtype.

## Poster Presentations

## ARV‐Based Prevention: Vertical Transmission and PEP

### P001 Near‐Elimination of Vertical HIV Transmission in England: Successes and Remaining Challenges, 2014–2023

Helen Peters, Kate Francis, Claire Thorne, on behalf of NHS England Infectious Diseases in Pregnancy HIV Clinical Expert Review Panel

Integrated Screening Outcomes Surveillance Service, UCL Great Ormond Street Institute of Child Health, London, UK

**Background**: Pregnant women are a key population in efforts to eliminate new HIV transmissions by 2030. In England, antenatal HIV screening coverage is 99.8% and approximately 90% of pregnant women living with HIV (WLWH) are diagnosed before pregnancy.

**Methods**: The Integrated Screening Outcomes Surveillance Service, within NHS England Infectious Diseases in Pregnancy Screening Programme, monitors all pregnancies to diagnosed WLWH and their infants in England. Infants are classified as HIV‐negative if negative ≥18‐month antibody test and/or ≥2 negative HIV PCRs at > 1 month of age. Enhanced data collection is undertaken for all diagnosed children born in England. A Clinical Expert Review Panel (CERP) establishes factors contributing to vertical transmission (VT). We describe VTs among women diagnosed by delivery in 2014–2023 and the most recent transmission rate (2022–2023).

**Results**: The VT rate remained < 0.4% throughout 2014–2023. In 2022–2023, there were 998 livebirths to WLWH, 87% (864/998) of women were diagnosed pre‐pregnancy, 82% (793/969) conceived on antiretroviral therapy (ART) and 92% (757/819) had delivery viral load <50 copies/mL (30 days before to 7 days post‐delivery). Infant tests were available for 93% (928/998): 98% (911/928) of infants were confirmed HIV‐negative. To date, there was one transmission in infants born 2022–2023 (VTR 0.11%, 95% CI: 0.003%, 0.604%) (Figure 1). Of 16 VTs in infants born 2014–2023, 12 were to women diagnosed pre‐pregnancy (8/12 on ART from conception). 3/12 were postnatal transmissions likely due to (unsupported) breastfeeding; 3/12 were in utero transmissions: 2/3 women had engagement and/or adherence issues and 1/3 booked late for antenatal care and delivered prematurely. In 4/12, there was a combination of adherence/engagement issues plus delivery complications, transmission timing unclear. For 2/12, infants timing of transmission/contributing factors were unclear. Among four infants born to women diagnosed during pregnancy: seroconversion during pregnancy (2), likely (unsupported) breastfeeding (1) and late booking (1) were factors.

**Conclusions**: The sustained low VT rate in England reflects the effectiveness of current prevention strategies. However, a small number of transmissions continue to occur, particularly among women facing complex social circumstances and challenges with engagement and treatment adherence. The CERP process provides vital insights to inform guidelines.

**FIGURE 1:**
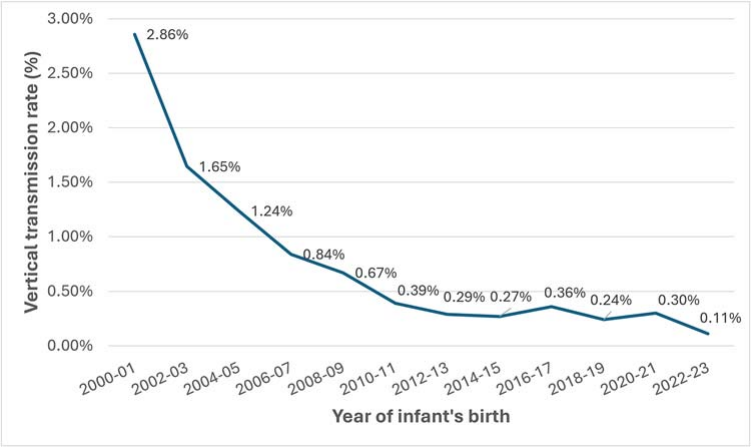
P001 | Vertical HIV transmission rate among women diagnosed by delivery in England, 2000–2023.

### P002 Characteristics and Outcomes of Infants Born to Women Living With HIV in England Receiving Three‐Drug Postnatal Prophylaxis, 2020–2025

Helen Peters ^1^, Kate Francis^1^, Rebecca Sconza^1^, Charlie Gorman^1^, Charlotte Roberts^1^, Alasdair Bamford^2,3^, Claire Thorne^1^

^1^Integrated Screening Outcomes Surveillance Service, UCL Great Ormond Street Institute of Child Health, London, UK; ^2^UCL Great Ormond Street Institute of Child Health, London, UK; ^3^Great Ormond Street Hospital for Children NHS Foundation Trust, London, UK

**Background**: British HIV Association (BHIVA) guidelines recommend risk‑stratified infant postnatal prophylaxis (PNP), with three‐drug PNP for infants classified as being at “high‑risk” of HIV acquisition.

**Methods**: The Integrated Screening Outcomes Surveillance Service (ISOSS), part of the NHS Infectious Diseases in Pregnancy Screening Programme, conducts population‐based surveillance of pregnancies in women living with HIV and their infants in England. Our aim was to describe three‐drug PNP use and outcomes in infants born 01/01/2020–31/12/2025 reported by 31/03/2026.

**Results**: Nearly all infants (99.9%, 3135/3136) received PNP; 5.5% (172/3136) received three‐drug PNP (no time trend, *p* = 0.088). Timing of maternal diagnosis and antiretroviral treatment (ART) for the 172 infants was: 90 (52.3%) on ART at conception, 22 (12.8%) previously diagnosed but started ART in pregnancy (at median 19 weeks), 56 (32.6%) with antenatal diagnosis and ART initiation (median 21 weeks), three pre‐pregnancy/antenatal diagnosis and no antenatal ART and one intrapartum diagnosis. Three infants had mothers who acquired HIV during pregnancy (negative at booking). Six infants were born to mothers without antenatal care (ANC). ANC booking occurred at > 10 weeks in 70.3% (121/172) and > 20 weeks in 21.0% (55/172). Thirty‐three (19.2%) were preterm (<37 weeks), median 33 weeks (range 27–36 weeks). Delivery HIV viral load (VL) was > 50 copies/mL in 64.9% (109/168). In 48.3% (83/172), additional ART was given to the mother at delivery (15/83 preterm). Over half (54.7% [94/172]) of infants were delivered by elective caesarean section. Most infants (86.0%, 147/171) received zidovudine/lamivudine/nevirapine; 11.1% (19) received zidovudine/lamivudine/raltegravir. Most (95.9%, 141/147) completed 4 weeks PNP; 16 subsequently stepped down to zidovudine alone following delivery day VL result of <50 copies/mL. There were four transmissions (3 in utero transmissions with positive PCR at birth where contributing factors were adherence issues [2] and seroconversion [1]); 151 infants were confirmed HIV‐negative (>18 month negative antibody), four were lost‐to‐follow‐up, two died with congenital conditions and 11 still in follow‐up.

**Conclusions**: Three‐drug PNP use remained uncommon over time in England, reflecting the high proportion of pregnant women on suppressive ART. Its use in high transmission risk scenarios is reflected in the transmission rate in this sub‐group.

### P003 Real‐World Safety, Tolerability and Treatment Completion of Bictegravir/Emtricitabine/Tenofovir Alafenamide (B/F/TAF) as Post‐Exposure Prophylaxis in a Large STI Clinic

Flavia Passini^1^, Nicolò Capra^2^, Riccardo Lolatto^2^, Angelo Roberto Raccagni^2^, Tommaso Clemente^2^, Elena Bruzzesi^2^, Anna Danise^2^, Gaia Catalano^1,2^, Davide Beretta^3^, Paola Rienzo^3^, Federico Furlan^3^, Antonella Castagna^1,2^, Silvia Nozza ^1,2^

^1^Vita‐Salute San Raffaele University, Milan, Italy; ^2^Infectious Diseases Unit, IRCCS San Raffaele Scientific Institute, Milan, Italy; ^3^ER Unit, IRCCS San Raffaele Scientific Institute, Milan, Italy

**Background**: Post‐exposure prophylaxis (PEP) is an effective strategy to prevent HIV acquisition following high‐risk sexual exposure. Bictegravir/emtricitabine/tenofovir alafenamide (B/F/TAF), administered as a once‐daily single‐tablet regimen, may improve treatment completion and tolerability in routine clinical practice. However, real‐world data on its use as PEP remain limited. We evaluated treatment completion, safety and virological outcomes among individuals receiving B/F/TAF‐based PEP at a tertiary sexually transmitted infection clinic.

**Methods**: We conducted an observational cohort study including consecutive adults who initiated a 28‐day course of B/F/TAF as PEP following sexual exposure at a tertiary STI clinic in Milan between November 2019 and January 2025. Data were extracted from routinely collected electronic medical records. Outcomes included treatment completion, follow‐up attendance, adverse events, laboratory parameters (alanine aminotransferase [ALT], aspartate aminotransferase [AST] and creatinine) and HIV seroconversion. HIV testing was performed according to clinical practice during follow‐up (weeks 4 and 12). Descriptive statistics were used for analysis.

**Results**: A total of 199 individuals initiated B/F/TAF‐based PEP (median age 35.4 years [IQR 30.1–41.0]; 95.9% male). Overall, 161/199 participants (80.9%) completed the full 28‐day regimen and attended the week‐4 follow‐up visit. Follow‐up HIV testing was available for 178/199 individuals and no HIV seroconversions were observed. Adverse events occurred in 10/199 participants (5.0%) and were generally mild, most commonly gastrointestinal symptoms (diarrhoea 4 [2.0%], nausea 3 [1.5%], abdominal discomfort 2 [1.0%]), headache (3 [1.5%]) and rash (1 [0.5%]). Treatment discontinuation or switch due to intolerance occurred in 3/199 cases (1.5%), while 90.0% reported no side effects. Laboratory parameters remained stable over follow‐up, with no clinically relevant changes in ALT (28 [21–36] vs. 27 [20–38], *p* = 0.319), AST (27 [22–31] vs. 25.5 [22–32], *p* = 0.854) or creatinine (1.01 [0.93–1.12] vs. 1.05 [0.94–1.15], *p* = 0.126) (Table 1).

**Conclusions**: In this large real‐world cohort, B/F/TAF‐based PEP was well tolerated and associated with high treatment completion, low rates of mild adverse events and stable laboratory parameters. No HIV seroconversions were observed among individuals with available follow‐up testing. These findings support B/F/TAF as a simple and effective option for HIV post‐exposure prophylaxis in routine clinical practice.

**TABLE 1:**
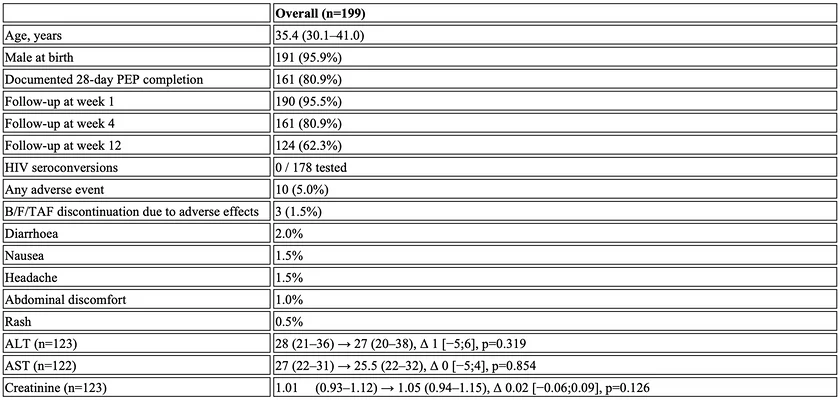
P003 | Clinical characteristics, outcomes and laboratory parameters in individuals receiving B/F/TAF as PEP.

### P004 Referral From Post‐Exposure Prophylaxis to Pre‐Exposure Prophylaxis in the US, 2016–2025: An Analysis of Real‐World Claims Data

Kenneth Mayer ^1^, Travis Lim^2^, Yi‐Han Hu^3^, Wenyi Wang^2^, Laura Mann^4^, Jason Hindman^5^, Lillian Brown^5^

^1^Fenway Health, Boston, MA, US; ^2^Real World Evidence Virology, Gilead Sciences, Inc, Foster City, CA, US; ^3^Syneos Health, Morrisville, NC, US; ^4^IQVIA, Atlanta, GA, US; ^5^Clinical Development, Gilead Sciences, Inc, Foster City, CA, US

**Background**: Both pre‐ and post‐exposure prophylaxis (PrEP and PEP, respectively) are important biomedical HIV prevention options for people at risk of HIV infection. However, uptake and consistent use of PrEP remains a challenge. Encounters with people seeking PEP after a sexual risk exposure provide opportunities to screen and refer people for PrEP. We assessed PrEP initiation rates after PEP episodes in a secondary claims database.

**Methods**: This study analysed retrospective observational closed claims data from the HealthVerity Marketplace from April 2016 to June 2025. We indexed PEP episodes as claims for raltegravir (RAL)+emtricitabine/tenofovir disoproxil fumarate (F/TDF), dolutegravir (DTG)+F/TDF or bictegravir/emtricitabine/tenofovir alafenamide (B/F/TAF). Eligible regimens had 28 or 30 days supply, plus a sexual risk exposure diagnosis code or STI laboratory test for syphilis or gonorrhoea in a medical claim within ≤72 h prior. Exclusion criteria were evidence of prior HIV, prior ART for treatment or intercurrent ARV use. A claims‐based proxy for referral was defined as initiation of oral PrEP (emtricitabine+tenofovir alafenamide [F/TAF] or emtricitabine+tenofovir disoproxil fumarate [F/TDF]) or cabotegravir injection (CAB) within 60 days of completing a PEP episode.

**Results**: Of the 19,102 PEP episodes identified, 3800 (19.9%) were followed by PrEP initiation. PrEP initiation was more common in males (24.0% of episodes), those commercially insured (21.1% of episodes) and those with past PrEP experience (33.5% of episodes; Table 1, *p* < 0.0001 for all comparisons). Referral increased through 2021, then decreased through 2025 (Table 1). Median time to initiating PrEP after PEP completion was 11 days, with most starting oral PrEP (Table 2).

**Conclusions**: Among PEP episodes identified in US claims data, approximately one in five were followed by initiation of PrEP within 60 days. Initiation was more common among males, those with prior PrEP experience and commercially insured individuals. A substantial proportion did not initiate PrEP following PEP, which may reflect missed opportunities as well as differences in risk assessment or patient preference. Further study is needed to better characterize recurring risk and optimize transitions from PEP to PrEP. A limitation of claims data is the potential for incomplete reporting or misclassification of PrEP use, PEP use and risk behaviour.

**TABLE 1:**
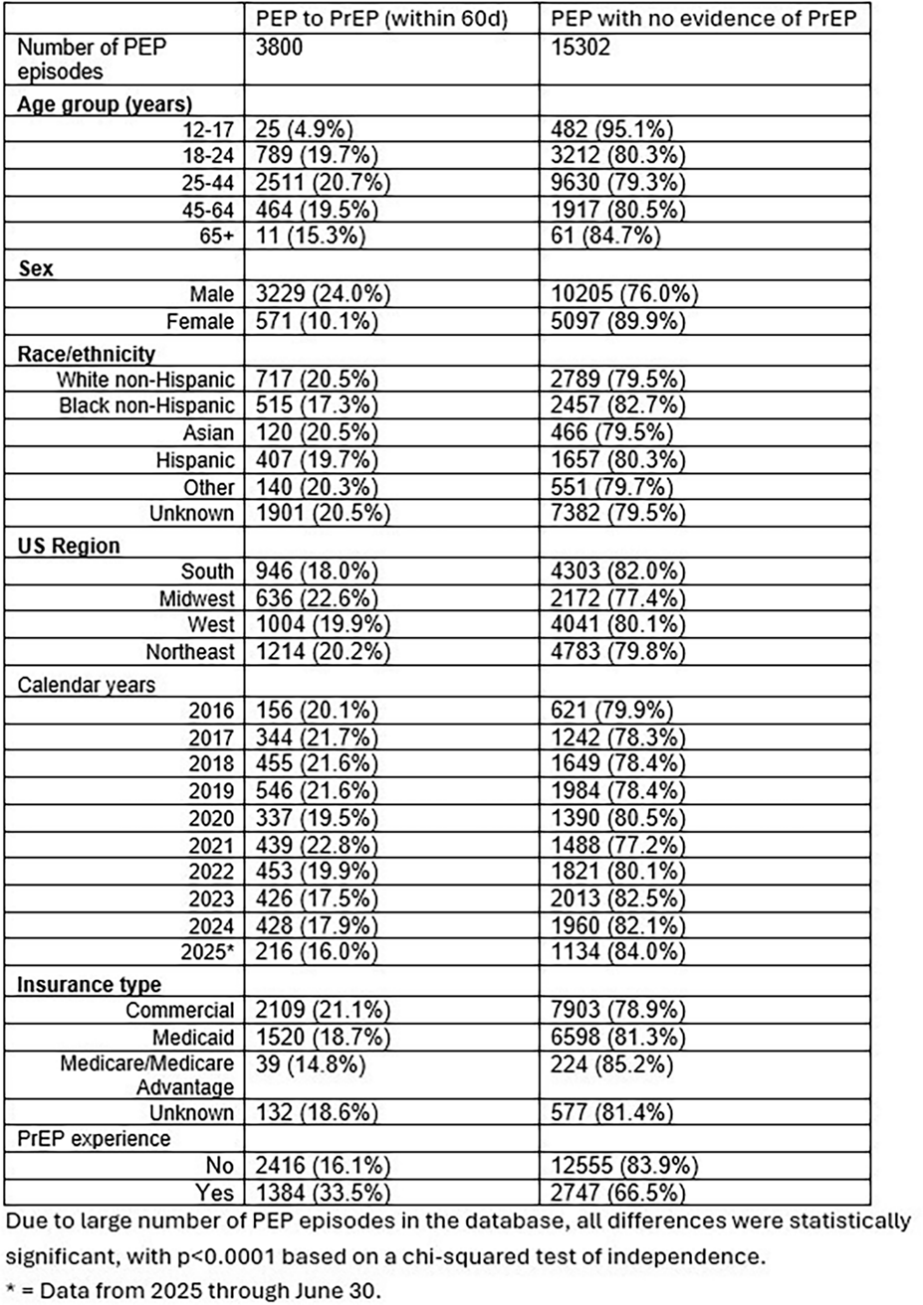
P004 | Characteristics of people who received PEP, with or without subsequent evidence of PrEP initiation within 60 days, among those with a known sexual risk exposure.

**TABLE 2:**
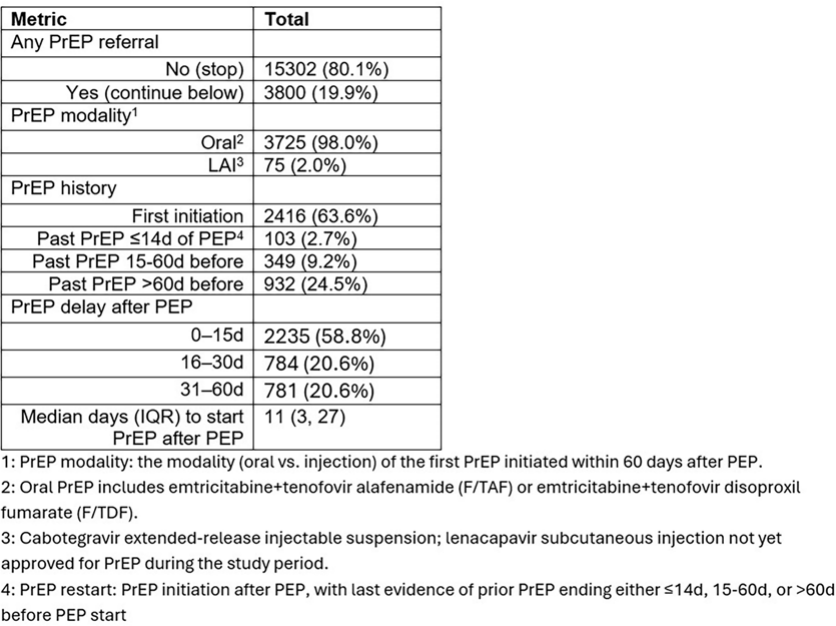
P004 | Description of the timing and type of PrEP initiation following PEP, among people who received both.

### P005 Dual Antiretroviral Therapy in Pregnant Women Living With HIV in Belgium

Marie Aractingi ^1^, Christine Gilles^2^, Severine Caluwaerts^3^, Sophie Henrard^4^, Marc Delforge^1^, Charlotte Martin^1^, Deborah Konopnicki^1^

^1^Infectious Diseases, Saint‐Pierre University Hospital, Brussels, Belgium; ^2^Gynaecology and Obstetrics, Saint‐Pierre University Hospital, Brussels, Belgium; ^3^HIV Clinic, Institute of Tropical Medicine, Antwerp, Belgium; ^4^HIV Reference Center and Immunodeficiency Unit, HUB Erasme, Brussels, Belgium

**Background**: Guidelines to prevent perinatal HIV recommend sustained undetectable viral load (VL) during pregnancy by using a triple antiretroviral drug regimen. However, modern dual antiretroviral therapy (ART) is as effective in achieving or maintaining VL suppression in persons with HIV and could reduce maternal and foetal drug exposure. This study characterizes dual ART use during pregnancy.

**Methods**: Multicentric ongoing retrospective study including 44 (ongoing or ended) pregnancies in 39 women receiving dual ART at any time during pregnancy in HIV reference centres in Belgium between 2019 and March 2026. Demographic characteristics, VL during pregnancy and delivery and delivery and infant outcomes were collected from medical file review. Viral outcomes were compared to the ones from 92 pregnancies under triple ART during whole pregnancy.

**Results**: At time of conception, women under dual ART had sub‐Saharan African origin in 88%, median age 31 years, median CD4+ count 732 cells/µL and 88% had undetectable VL (≤50 cp/mL); dual therapy consisted of dolutegravir‐containing regimens in 31/44 (70%) pregnancies, injectables cabotegravir/rilpivirine in 3 (7%). Switch from triple to dual therapy during pregnancy occurred in 8 (18%) pregnancies mostly to discontinue cobicistat‐containing regimens. Seven (16%) women were switched from dual to triple therapy mostly (72%) to follow guidelines. Most women (83.8%) were virologically suppressed all throughout pregnancy. Blips (unconfirmed VL > 50 cp/mL) and failure (confirmed VL > 50 cp/mL) rates were, respectively, 15.4% and 5% and occurred either after treatment interruption or switch or were receiving injectables (1 failure). In 37 pregnancies that reached delivery, 35 (94.6%) were undetectable around 36 gestational weeks. Compared to women under triple ART during whole pregnancy, there were less failure under dual therapy (5% vs. 18.5%, *p* = 0.0575) and more VL ≤50 cp/mL at week 36 (94.6% vs. 76%, *p* = 0.04) and delivery (91.2 vs. 80.4%, *p* = 0.08). No adverse outcome (including HIV infection) was diagnosed in the 37 infants.

**Conclusions**: Preliminary results show viral suppression before delivery in 94.6% of women under dual ART, mainly dolutegravir‐based, which compares favourably to triple therapy, suggesting that dolutegravir‐based dual regimen could be included in the pregnancy guidelines. One failure occurred among three patients under injectable cabotegravir/rilpivirine, which calls for strong caution and further assessment.

### P006 Exploration of Surgical Procedures With the Highest Risk for Potential HIV Transmission

David Custers ^1^, Jessica van der Mannen^2^, Kees Brinkman^2^, on behalf of HIV team OLVG, Amsterdam

^1^Faculty of medicine, University of Amsterdam, Amsterdam, Netherlands; ^2^Internal medicine department, OLVG, Amsterdam, Netherlands

**Background**: Globally, a substantial number of needle stick injuries (NSI) occur during surgical procedures, putting healthcare workers at risk for acquiring infectious diseases. Although most research in this area only considers general factors, such as the duration of procedures, the equipment used and the experience of the personnel involved, as risk factors for NSI incidence, little is known about the incidence of NSIs between different surgical specialties and between different surgical procedures. In this study, we explored which surgical procedures carry the highest risk for NSIs and, therefore, the highest risk for HIV transmission in order to design a more targeted intervention for the prevention of HIV transmission.

**Methods**: For this explorative retrospective study, we used the needle stick accidents database of OLVG hospital, which contains information of all incidents with a risk for blood‐borne transmissions that occurred in the hospital. All surgical procedures in 2023 in the OLVG were filtered with CTcue with data on the specialty of and type of surgical procedure. The distribution of NSIs across surgical specialties was calculated as the primary outcome measure. We used a Fisher's exact test to evaluate the risk of NSIs per surgical specialty. We calculated the odds ratios of the NSIs risks for specialties with > 5 NSIs and the procedures of the specialty with the highest proportion of NSIs.

**Results**: Our results show 253 reported NSIs in 2023 at OLVG hospital (Figure 1), with 111 NSIs occurring during 23,645 surgical procedures. The results highlight the high proportion of NSIs in cardiothoracic surgery 40.5% (45/111) with a corresponding high risk 9.11% (45/449) and on OR 38.29 (CI 22.96–63.88, *p* < 0.001) compared to general surgery 0.26% (23/8786). The cardiothoracic surgery procedure with the highest incidence was cardiac valve surgery by means of video‐assisted thoracoscopic surgery (VATS) 26.2% (11/31).

**Conclusions**: Our results show large differences in incidence of NSIs between surgical specialties. Cardiothoracic surgery had a significantly higher proportion and risk of NSIs in this study. Further research is necessary to confirm these findings, but the results warrant further exploration of more targeted preventive approaches for these high‐risk procedures.

**FIGURE 1:**
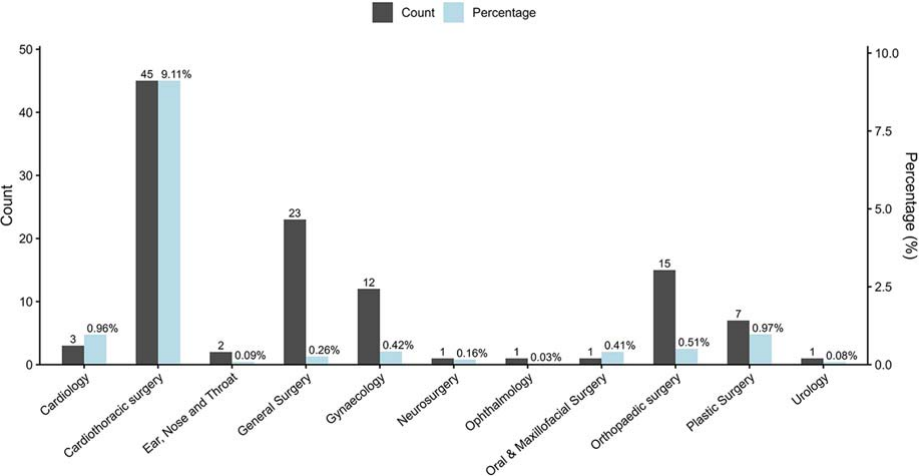
P006 | The number of needle stick injuries (count) per surgical specialism and the percentage of procedures with needle stick injuries of the total amount of procedures performed at the OLVG in 2023.

### P007 Enhancing nPEP Adherence and Mental Health via a LINE‐Based Digital Intervention: A Randomized Controlled Trial, a First 52‐Weeks Result

Jen‐Chung Chu, Shu‐Yuan Li, Che‐Yu Kuo

Comprehensive AIDS Care Center, Taoyuan General Hospital, Taoyuan City, Taiwan

**Background**: Real‐world efficacy of non‐occupational post‐exposure prophylaxis (nPEP) is often hindered by severe psychological distress, stigma and poor adherence. This randomized controlled trial (RCT) conducted at Taoyuan General Hospital evaluated whether a smartphone‐based support system integrated into the LINE messaging application could improve clinical adherence and mental health outcomes.

**Methods**: The study enrolled 55 participants from June 2025 to June 2026 (npep001 to npep055) seeking care after their first non‐occupational sexual exposure. Demographics were predominantly MSM (45.4%) and MSW (54.6%), with 95.8% holding a college degree or higher. Control group received standard of care (SoC) only. Intervention group received SoC plus access to a customized LINE plugin developed with Yuan Ze University, featuring automated medication reminders, follow‐up notifications, positive reinforcement and educational content.

**Results**: Adherence and Retention: Although 5 participants discontinued early during the acute phase (weeks 1–2), users on the digital platform exhibited significantly higher protocol completion rates and medication adherence (measured via pill counts) than the control group.

Mental Health Trajectories: The intervention group showed a significantly faster decline in acute psychological distress (measured via BAI/BDI and HADS) from baseline. The automated features served as a “digital buffer,” effectively mitigating acute anxiety and exposure‐related stigma. PrEP Transition: The intervention group reported substantially higher healthcare satisfaction. Remarkably, 49.1% (27 out of 55) of the total cohort successfully transitioned to the Public Health PrEP Programme immediately after either completing their 12 or 24 weeks follow‐up (Figures 1 and 2).

**Conclusions**: Integrating a customized mHealth tool into a ubiquitous platform like LINE is a highly feasible, scalable strategy for nPEP case management, especially when it comes at the first time for unsafe sexual encounter. By bridging functional clinical reminders with real‐time psychological reinforcement, this dual‐benefit intervention simultaneously optimizes pharmacological compliance and addresses mental health vulnerabilities, supporting its broader integration into comprehensive HIV prevention services.

**FIGURE 1:**
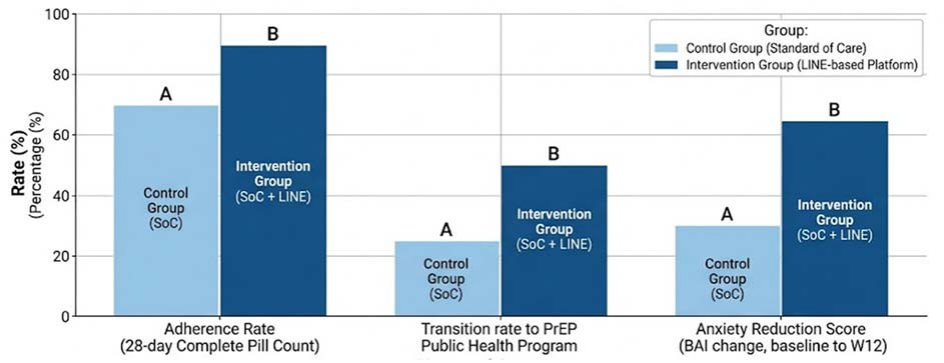
P007 | Comparison of overtime nPEP adherence and clinical transitions between standard care and LINE‐digital intervention.

**FIGURE 2:**
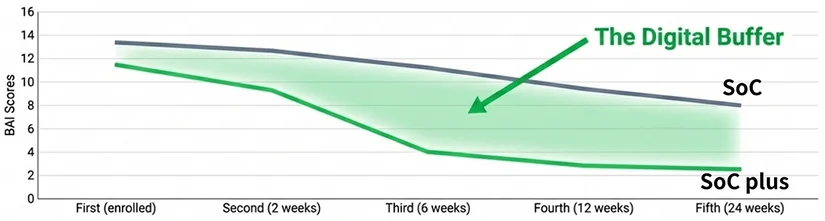
P007 | Beck index anxiety inventory score overtime.

### P008 Vertical Transmission of HIV Among ART‐Treated HIV‐Positive Pregnant Women in Burundi: A Multi‐Site Cohort Study Across 12 Clinics

Emmanuel Nkengurutse, Nadine Uwimana, Desire Ramadhan

University of Burundi, Bujumbura, Burundi

**Background**: Despite widespread ART‐based PMTCT strategies, residual vertical transmission persists in Burundi. We estimated the rate of mother‐to‐child HIV transmission among newborns born to HIV‐positive pregnant women on ART and identified maternal, infant and programmatic factors associated with transmission in a multi‐site cohort study.

**Methods**: We followed 2756 HIV‐positive pregnant women on ART who delivered in 12 Burundi HIV clinics collecting data from PMTCT registers and records, including maternal ART regimen and duration, adherence proxies, regional viral load data when available, CD4 counts, maternal and infant prophylaxis, mode of delivery, infant feeding practices, timing of infant HIV testing and infant HIV status. We characterized transmission rates by context and used multivariable logistic regression accounting for clustering by site to identify factors associated with transmission. ART regimens were categorized per guidelines (Option B+; DTG‐ or EFV‐based regimens; TDF/3TC/ABC or TDF/3TC/EFV‐equivalents where available).

**Results**: The overall vertical transmission rate was 8.3% (229/2756). Transmission risk demonstrated substantial heterogeneity across sites and subgroups. In analyses adjusted for clustering, suboptimal maternal viral suppression near delivery (defined as detectable plasma RNA) markedly increased transmission risk (aOR 3.2; 95% CI: 2.4–4.3). Earlier ART initiation (at conception or early pregnancy) and longer duration of sustained viral suppression were independently protective (aOR per 4‐week advancement in suppression onset: 0.88; 95% CI: 0.82–0.95). Among ART regimens, DTG‐based or EFV‐based first‐line backbones with tenofovir‐emtricitabine achieved higher suppression rates than some older NNRTI‐based schemes (aOR for transmission with DTG‐based vs. EFV‐based: 0.71; 95% CI: 0.54–0.93), though data were limited by incomplete viral load monitoring in peri‐urban clinics. Infant factors associated with lower risk included exclusive formula feeding where feasible and timely infant HIV testing within 6 weeks of birth, linked to prompt initiation of post‐exposure prophylaxis where indicated. Mode of delivery and breastfeeding practices aligned with local PMTCT guidance showed context‐dependent associations with transmission risk. The majority of women received regimens compatible with Option B+ and a substantial subset achieved virologic suppression by late pregnancy or at delivery when data permitted.

**Conclusions**: Vertical transmission remained 8.3% in Burundi. Rapid ART initiation, regimen optimization (DTG‐based), adherence support and comprehensive viral load monitoring alongside timely infant testing and prophylaxis are major strategies to reduce transmission.

## ARV‐Based Prevention: PrEP

### P009 High Persistence, Adherence and Tolerability of Long‐Acting Cabotegravir for HIV Pre‐Exposure Prophylaxis: National Implementation Experience From the Italian PrIDE Cohort

Davide Moschese ^1,2^, Gianluca Frate^3^, Damiano Farinacci^4^, Chiara Fusetti^2^, Angelo Roberto Raccagni^5,6^, Francesco Barbaro^7^, Nicholas Brian Bana^8^, Virginia Barchi^4^, Valentina Sala^9^, Vito Leonardo Del Negro^10^, Enrico Caruso^11^, Samuel Lazzarin^2^, Gabriele Loi^12^, Antonella Castagna^5,6^, Andrea Gori^13,14^, Andrea Antinori^4^, Silvia Nozza^5,6^, Valentina Mazzotta^4^

^1^Department of Clinical and Biomedical Sciences—Università degli Studi di Milano, Milan, Italy; ^2^Department of Infectious Diseases—Luigi Sacco University Hospital, Milan, Italy; ^3^ICONA Foundation, Milan, Italy; ^4^National Institute for Infectious Diseases Lazzaro Spallanzani IRCCS, Rome, Italy; ^5^Vita Salute San Raffaele University, Milan, Italy; ^6^Infectious Diseases Unit—IRCCS San Raffaele Scientific Institute, Milan, Italy; ^7^Infectious and Tropical Diseases Unit—Azienda Ospedale‐Università Padova, Padua, Italy; ^8^ASST Grande Ospedale Metropolitano Niguarda, Milan, Italy; ^9^ASST Santi Paolo e Carlo, Milan, Italy; ^10^Checkpoint Plus Roma, Rome, Italy; ^11^Milano Checkpoint ETS, Milan, Italy; ^12^5.Vita Salute San Raffaele University, Milan, Italy; ^13^Department of Biomedical and Clinical Sciences—Università degli Studi di Milano, Milan, Italy; ^14^Department of Infectious Diseases, Luigi Sacco University Hospital, Milan, Italy

**Background**: Long‐acting cabotegravir (CAB‐LA) is highly effective for HIV PrEP, but data describing national implementation programmes remain limited, particularly in Europe [1−3]. We evaluated persistence, adherence and tolerability outcomes of CAB‐LA PrEP within the PrIDE cohort.

**Methods**: Prospective observational study including individuals initiating CAB‐LA PrEP between December 2024 and January 2026. CAB‐LA was administered without an oral lead‐in. Outcomes included persistence in PrEP during follow‐up assessed using Kaplan–Meier methods, adherence to the injection schedule (rate of on‐time and delayed injection), rates and reasons for discontinuations, systemic and injection‐site adverse events and incident HIV infections.

**Results**: Overall, 529 individuals received CAB‐LA PrEP, accounting for 3406 injections. Median age was 40 years (IQR 34–48); 96.2% were assigned male sex at birth and 94.5% were gay, bisexual and other men who have sex with men. Access was based on user preference (25.7%) or predefined eligibility criteria (74.3%), including clinical indications and membership of a key population. Median follow‐up was 44.6 weeks (IQR 36.7–47.7). Persistence probability at week 44 was 90% (95% CI: 87%–93%). Among 2877 scheduled follow‐up injections, 2705 (95.8%) were administered within the predefined dosing window, 113 (4%) were delayed and 8 (0.3%) required re‐initiation following prolonged interruption (Figure 1A). Overall, 51 participants (9.6%) discontinued CAB‐LA PrEP. Twenty discontinuations (3.8%) were unrelated to treatment, while 31 (5.9%) were treatment‐related (Table 1). No HIV breakthrough infections were observed. One discordant HIV screening (Ag/AB) result was investigated and subsequently confirmed as a false positive. Among participants with pain assessments, the proportion reporting no injection‐site pain increased from 36.5% after the first injection to 58.0% after the sixth injection, while grade 3 pain decreased from 15.6% to 8.2%. Systemic adverse events were uncommon. Despite frequent early injection‐site reactions, pain‐related discontinuation occurred in only 11 participants (2.1%) (Figure 1B).

**Conclusions**: Across more than 3400 CAB‐LA injections, persistence and adherence to the dosing schedule remained high and no HIV infections occurred. Tolerability improved over time, with very few discontinuations related to injection‐site reactions. These real‐world data support large‐scale implementation of CAB‐LA PrEP as a highly effective and acceptable HIV prevention strategy.

**References**

1. Landovitz RJ, Hanscom BS, Clement ME, Tran HV, Kallas EG, Magnus M, Sued O, Sanchez J, Scott H, Eron JJ, Del Rio C, Fields SD, Marzinke MA, Eshleman SH, Donnell D, Spinelli MA, Kofron RM, Berman R, Piwowar‐Manning EM, Richardson PA, Sullivan PA, Lucas JP, Anderson PL, Hendrix CW, Adeyeye A, Rooney JF, Rinehart AR, Cohen MS, McCauley M, Grinsztejn B; HPTN 083 Study Team. Efficacy and safety of long‐acting cabotegravir compared with daily oral tenofovir disoproxil fumarate plus emtricitabine to prevent HIV infection in cisgender men and transgender women who have sex with men 1 year after study unblinding: a secondary analysis of the phase 2b and 3 HPTN 083 randomised controlled trial. Lancet HIV. 2023 Dec;10(12):e767‐e778. doi: 10.1016/S2352‐3018(23)00261‐8. Epub 2023 Nov 9. PMID: 37952550; PMCID: PMC11375758.

2. Delany‐Moretlwe S, Hughes JP, Bock P, Ouma SG, Hunidzarira P, Kalonji D, Kayange N, Makhema J, Mandima P, Mathew C, Spooner E, Mpendo J, Mukwekwerere P, Mgodi N, Ntege PN, Nair G, Nakabiito C, Nuwagaba‐Biribonwoha H, Panchia R, Singh N, Siziba B, Farrior J, Rose S, Anderson PL, Eshleman SH, Marzinke MA, Hendrix CW, Beigel‐Orme S, Hosek S, Tolley E, Sista N, Adeyeye A, Rooney JF, Rinehart A, Spreen WR, Smith K, Hanscom B, Cohen MS, Hosseinipour MC; HPTN 084 study group. Cabotegravir for the prevention of HIV‐1 in women: results from HPTN 084, a phase 3, randomised clinical trial. Lancet. 2022 May 7;399(10337):1779‐1789. doi: 10.1016/S0140‐6736(22)00538‐4. Epub 2022 Apr 1. Erratum in: Lancet. 2022 May 7;399(10337):1778. doi: 10.1016/S0140‐6736(22)00783‐8. PMID: 35378077; PMCID: PMC9077443.

3. Moschese D, Esvan R, Fusetti C, Barchi V, Cossu MV, Giacinta A, Lazzarin S, Terracini G, Caruso F, Micheli G, Maddalena D, Del Duca G, Petri F, Frate G, Matone M, Farinacci D, Giacomelli A, Faccendini P, Visigalli D, Passacantilli S, Riva A, Antinori A, Gori A, Mazzotta V. Early results from implementation of HIV pre‐exposure prophylaxis (PrEP) with long‐acting injectable cabotegravir for people with barriers to oral strategies, Italy, December 2024 to August 2025. Euro Surveill. 2025 Oct;30(39):2500739. doi: 10.2807/1560‐7917.ES.2025.30.39.2500739. PMID: 41040064; PMCID: PMC12495377.

**FIGURE 1:**
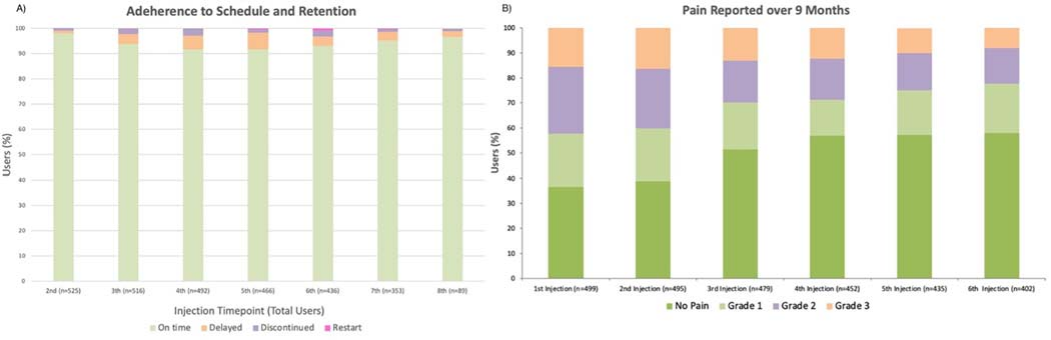
P009 | Persistence, adherence and tolerability of CAB‐LA PrEP across more than 3400 injections in Italy.

**TABLE 1:**
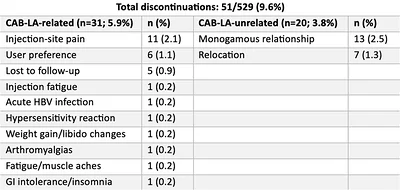
P009 | Reasons for CAB‐LA discontinuation.

### P010 Early Multicompartment Distribution of Long‑Acting Cabotegravir After the First Intramuscular Injection for HIV Pre‐Exposure Prophylaxis: Preliminary Results From the TUTT*E Study

Massimo Tempestilli^1^, Davide Moschese^2^, Damiano Farinacci^3^, Sara Soloperto^4^, Maria Maddalena Plazzi^3^, Simona Gili^1^, Samuel Lazzarin^2^, Virginia Barchi^3^, Elisa Martina^4^, Francesco Caruso^2^, Rebecca Jo Steiner^3^, Eleonora Cimini^1^, Andrea Gori^2^, Andrea Antinori^5^, Antonio D'Avolio^4^, Valentina Mazzotta ^3^

^1^Cellular Immunology and Pharmacology Laboratory, National Institute for Infectious Diseases “Lazzaro Spallanzani” IRCCS, Rome, Italy; ^2^Department of Infectious Diseases, Luigi Sacco University Hospital, ASST Fatebenefratelli Sacco, Milan, Italy; ^3^Regional HIV/ AIDS Reference Center (CRR‐AIDS), National Institute for Infectious Diseases Lazzaro Spallanzani IRCCS, Rome, Italy; ^4^Laboratory of Clinical Pharmacology and Pharmacogenetics, Department of Medical Sciences, University of Turin, Turin, Italy; ^5^Health Direction, National Institute for Infectious Diseases Lazzaro Spallanzani IRCCS, Rome, Rome, Italy

**Background**: Tissue‐to‐plasma ratios are relevant for understanding early exposure to long‐acting cabotegravir (CAB‐LA) administered as pre‐exposure prophylaxis (PrEP), especially when CAB‐LA is started without an oral lead‐in. Available multicompartment pharmacokinetic data have been derived mainly from controlled clinical studies and model‐based analyses, using procedures not easily transferable to routine care [1,2]. We investigated CAB‐LA concentrations in plasma (PL) and anatomical compartments relevant to sexual HIV‐1 transmission for men who have sex with men (MSM) up to 28 days after the first intramuscular (IM) injection in a real‐life setting.

**Methods**: CAB concentrations were measured in PL, seminal plasma (SP) and rectal fluid (RF) on days 3, 7 and 28 after the first 600‐mg CAB‐LA IM injection without an oral lead‐in. RF samples were collected using volumetric absorptive microsampling (Mitra) via anoscopy [3]. CAB concentrations were quantified using a validated liquid chromatography coupled with mass‐spectrometry. Predefined CAB thresholds were the proposed prevention‐effective target of 664 ng/mL (4×protein‐adjusted 90% inhibitory concentration, 4×PA‐IC90) for PL [4] and 16.6 ng/mL (IC90) for SP and RF. Exploratory univariable linear regression was used to assess associations between CAB concentrations and baseline characteristics.

**Results**: 23 cisgender men were enrolled; median age 49 years (IQR 38–54). Median CAB concentrations exceeded 4xPA‐IC90 in PL and IC90 in SP and RF at all timepoints (Figure 1). PL concentrations above 4×PA‐IC90 were observed in 87% of participants at all timepoints, while SP concentrations above IC90 were observed in 74% at day 3 and 83% at days 7 and 28; corresponding RF proportions above IC90 were 85% (17/20 quantifiable samples), 87% and 83% (20/23 and 19/23 samples) at days 3, 7 and 28, respectively. PL concentrations were associated with time‐matched SP concentrations but not significantly associated with RF at any time point. Higher BMI was consistently associated with lower PL and SP CAB concentrations (Table 1).

**Conclusions**: Our findings showed that most participants achieved PL CAB concentrations above the proposed prevention‐effective target by day 3 after the first CAB‐LA injection without oral lead‐in. Early multicompartment CAB distribution suggests potentially protective pharmacological exposure as early as the third day after initiation of CAB‐LA PrEP.

**References**

1. Shaik JS, Weld ED, Edick S, Fuchs E, Riddler S, Marzinke MA, D'Amico R, Bakshi K, Lou Y, Hendrix C, Han K, Ford SL, Margolis D, Spreen W, Patel P. Multicompartmental pharmacokinetic evaluation of long‐acting cabotegravir in healthy adults for HIV pre‐exposure prophylaxis. Br J Clin Pharmacol. 2022;88(4):1667‐1678.

2. Han K, Patel P, McCallister S, Rinehart AR, Gandhi Y, Spreen W, Landovitz RJ, Delany‐Moretlwe S, Marzinke MA, McKeon T, Budnik P, van Wyk J, Ford SL. Long‐acting cabotegravir pharmacokinetics with and without oral lead‐in for HIV PrEP. Antimicrob Agents Chemother. 2024;68(6):e0147523.

3. Protti M, Mandrioli R, Mercolini L. Tutorial: Volumetric absorptive microsampling (VAMS). Anal Chim Acta. 2019;1046:32‐47.

4. Hanscom B, Marzinke MA, Li X, Donnell D, Bies R, Hendrix CW, Wang Z, Acuipil C, Rinehart A, Collins JW, Rooney JF, Soto Torres L, Richardson P, Chariyalerstsak S, Valdez Ramalho Madruga J, Hurt CB, Magnus M, Frank I, McCauley M, Grinsztejn B, Landovitz RJ. Estimation of prevention‐effective CAB‐LA concentrations among men who have sex with men (MSM) and transgender women (TGW) in HPTN 083. J Infect Dis. 2026;233:e454‐e461.

**FIGURE 1:**
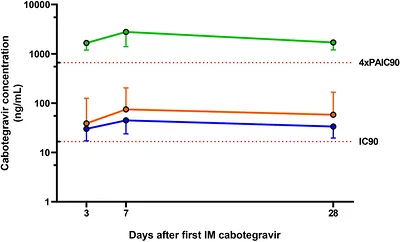
P010 | Median cabotegravir concentrations and 95% confidence intervals, shown on a logarithmic scale, in plasma (green line), seminal plasma (blue line) and rectal fluid (orange line) after the first intramuscular long‐acting cabotegravir injection. Red dashed lines indicate IC90 (90% inhibitory concentration) = 16.6 ng/mL and 4×PA‐IC90 = 664 ng/mL.

**TABLE 1:**
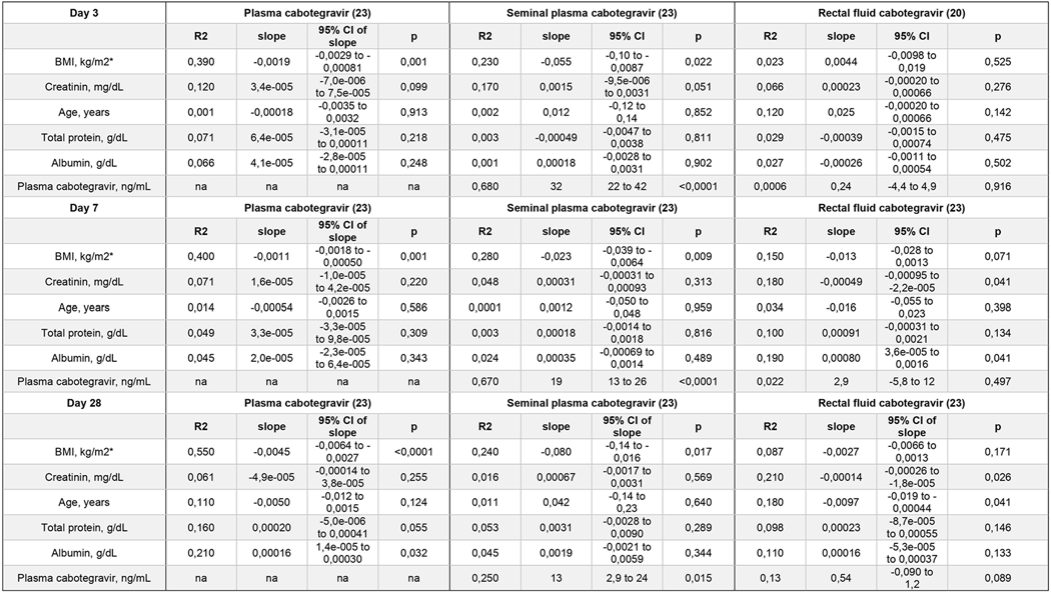
P010 | Linear regression analysis of key factors associated with cabotegravir concentrations in plasma (PL), seminal plasma (SP) and rectal fluid (RF) during the first 28 days after the initial CAB‐LA injection.

### P011 Potential Target Populations for Lenacapavir PrEP in Italy: A Real‐World Analysis From the PrIDE Cohort (Epi‐PrEP Study)

Valentina Mazzotta ^1^, Gianluca Frate^2^, Anna Lucchini^3^, Chiara Fusetti^4^, Giulia Micheli^1^, Vito Leonardo Del Negro^5^, Giulia C Marchetti^6,7^, Annamaria Cattelan^8^, Roberto Caldera^9^, Gabriele Forcina^9^, Massimo Cernuschi^10^, Antonella Castagna^11,12^, Silvia Nozza^11,12^, Davide Moschese^4,13^, Andrea Antinori^14^, on behalf of PrIDE/Epi‐PrEP Study Group

^1^Counseling, Test and HIV Prophylaxis and STI Unit, Regional AIDS Reference Center, National Institute for Infectious Diseases Lazzaro Spallanzani IRCCS, Rome, Italy; ^2^ICONA Foundation, Milan, Italy; ^3^Multidisciplinary Center for Sexual Health (Ce.Mu.S.S.), Ospedale Oftalmico “Casimiro Sperino”, ASL Città di Torino, Turin, Italy; ^4^Department of Infectious Diseases, Luigi Sacco University Hospital, Milan, Italy; ^5^Checkpoint Plus Roma, Rome, Italy; ^6^Clinic of Infectious Diseases, San Paolo Hospital, ASST Santi Paolo e Carlo, University of Milan, Milan, Italy; ^7^Department of Health Sciences, University of Milan, Milan, Italy; ^8^Infectious and Tropical Diseases Unit, Padua University Hospital, Padua, Italy; ^9^Gilead Sciences S.r.l., Milan, Italy; ^10^Milano Checkpoint ETS, Milan, Italy; ^11^Infectious Diseases Unit, Vita‐Salute San Raffaele University, Milan, Italy; ^12^Infectious Diseases Unit, IRCCS San Raffaele Scientific Institute, Milan, Italy; ^13^Department of Clinical and Biomedical Sciences, University of Milan, Milan, Italy; ^14^Clinical and Research Infectious Diseases Department, National Institute for Infectious Diseases Lazzaro Spallanzani IRCCS, Rome, Italy

**Background**: Twice‐yearly lenacapavir (LEN) may expand HIV pre‐exposure prophylaxis (PrEP) options by reducing administration frequency and improving adherence. Its implementation value depends on identifying people whose prevention needs are not fully met by oral PrEP or 2‐monthly intramuscular long‐acting cabotegravir (CAB‐LA). We aimed to identify potential LEN target populations in the national PrIDE cohort.

**Methods**: Exploratory analysis of PrEP users enrolled in the Italian observational PrIDE cohort in 2024–2026, classified into two groups: CAB‐LA‐experienced users and oral PrEP users with no prior CAB‐LA exposure. Among CAB‐LA‐experienced users, LEN target criteria were predefined to capture barriers to 2‐monthly CAB‐LA delivery, tolerability or route of administration: recurrent delayed injections, loss to follow‐up, discontinuation for logistical reasons or adverse events, ≥2 injections associated with grade 3 pain and BMI > 30 kg/m^2^ as a potential barrier to intramuscular injection. Among oral users, criteria identified underserved populations whose access to PrEP could improve with twice‐yearly subcutaneous administration, including mental health disorders, sex workers and cis/trans women. Among remaining users with any clinical criteria for long‐acting PrEP, community‐based setting (CBS) follow‐up was explored as a proxy for users potentially facing barriers to hospital‐based PrEP delivery; in this subgroup, twice‐yearly subcutaneous LEN may offer a more feasible alternative to 2‐monthly intramuscular CAB‐LA. Users could meet more than one criterion.

**Results**: Overall, 559/5427 PrEP users (10.3%) met at least one potential LEN target criterion (Figure 1). Combinations of criteria are in Table 1. Among 567 CAB‐LA‐experienced users, 153 (27.0%) met criteria: repeated grade 3 injection‐site pain (93, 16.4%), delayed injections (43, 7.6%), BMI > 30 kg/m^2^ (22, 3.9%), discontinuation for adverse events (3, 0.5%), loss to follow‐up (3, 0.5%) or logistical reasons (2, 0.4%). 295/4860 (6.1%) oral users met underserved‐population criteria, including sex work (150, 3.1%), mental health disorder (92, 1.9%), transgender women (84, 1.7%) and cisgender women (48, 1.0%). An additional 111 oral users (2.3%) eligible for long‐acting due to clinical issues with oral PrEP met the CBS proxy.

**Conclusions**: Potential LEN target populations accounted for 10% of PrIDE users, including one in four CAB‐LA‐experienced users. LEN may expand PrEP access beyond users adequately served by currently available oral and bimonthly injectable options.

**FIGURE 1:**
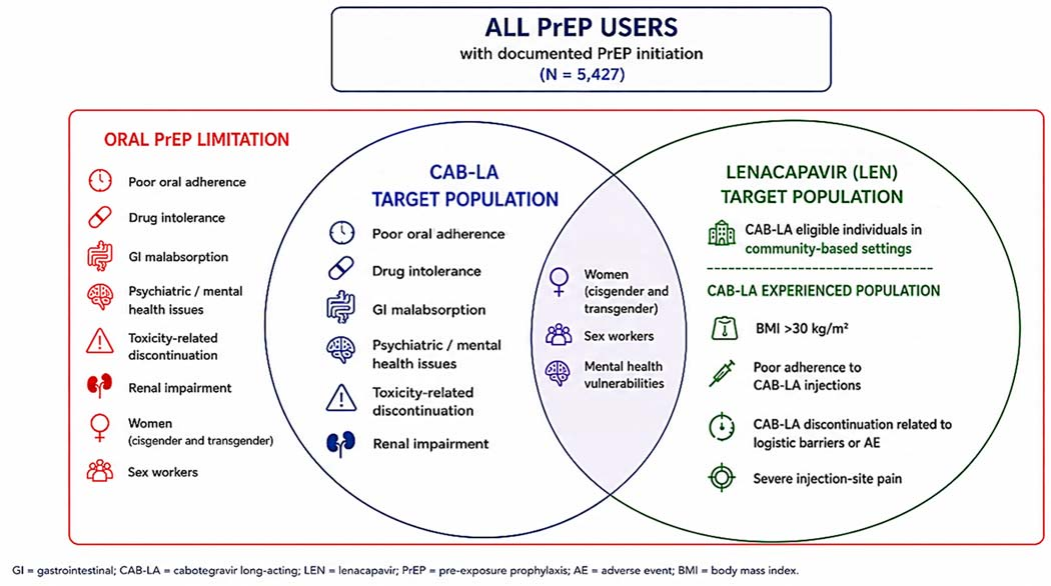
P011 | LEN flowchart.

**TABLE 1:**
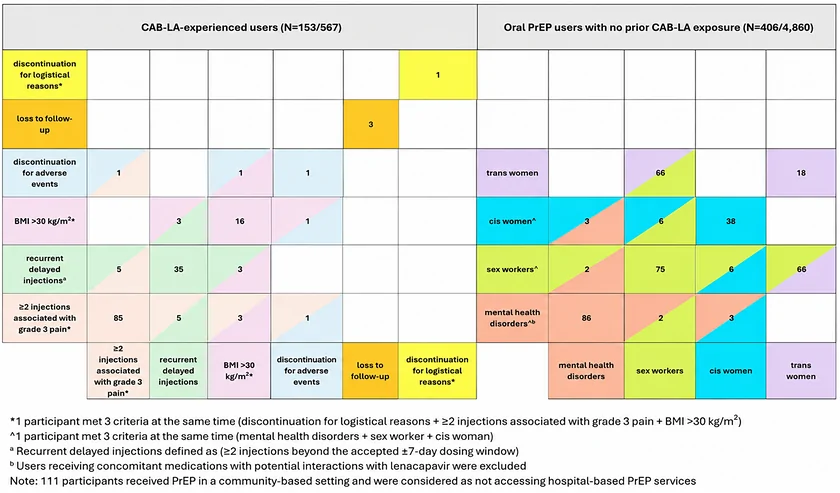
P011 | Predefined LEN target criteria to capture barriers to CAB‐LA or oral PrEP, alone or in double combination (overall cohort; *N* = 5427).

### P012 Phenotypic Analyses of HIV Clinical Isolates With Capsid Substitutions Observed in the PURPOSE 1 and PURPOSE 2 Studies

Matthew R. Hendricks ^1^, Kristen Andreatta^1^, Jiani Li^2^, Christoph C. Carter^3^, Christian Callebaut^1^, Stephanie Cox^1^

^1^Clinical Virology, Gilead Sciences, Inc., Foster City, CA, US; ^2^Biostatistics, Gilead Sciences, Inc., Foster City, CA, US; ^3^Clinical Development, Gilead Sciences, Inc., Foster City, CA, US

**Background**: Lenacapavir (LEN) is a first‐in‐class HIV‐1 capsid inhibitor approved for HIV pre‐exposure prophylaxis (PrEP). The pivotal phase 3 PURPOSE 1 (NCT04994509) and PURPOSE 2 (NCT04925752) studies demonstrated that acquisition of HIV while receiving LEN for PrEP was rare. In the primary analyses, zero and two new HIV‐1 cases were detected in participants receiving LEN in PURPOSE 1 and 2, respectively; both participants developed the N74D LEN resistance‐associated mutation (RAM). Additionally, two participants receiving LEN in each study had unrecognized HIV‐1 at baseline and developed N74D. We characterize N74D capsid RAMs, including baseline and background capsid sequence diversity, in clinical isolates and site‐directed mutants (SDMs).

**Methods**: Participant‐derived viral gag sequences and associated SDMs were synthesized, cloned into HIV‐1 infectious molecular clones and produced in 293T cells. Fold changes (FCs) in LEN susceptibility and/or replication capacity (RC) of viruses relative to wild‐type were evaluated using multicycle luciferase reporter assays in T‐cell lines and primary peripheral blood mononuclear cells (PMBCs), plus the single‐cycle PhenoSense Gag‐Pro assay (Monogram Biosciences).

**Results**: Among N74D‐containing viruses tested, a wide range of LEN resistance levels was observed in multicycle luciferase reporter assays (FC range: 14–282), similar to results from the single‐cycle assay. No LEN resistance was observed in participant‐derived baseline samples tested, indicating the absence of baseline resistance to LEN prior to N74D acquisition. In one participant with unrecognized HIV at baseline who received LEN, emergence of Q67Q/R and I73I/V capsid substitutions alongside N74D was observed. In the participant‐derived sequence without N74D, the Q67R, I73V and Q67R + I73V mutations were associated with LEN FCs of 64, 2.5 and 20, respectively, in multicycle assays. The Q67R SDM was non‐infectious in T‐cell lines, similar to Q67K. The RC of all participant‐derived isolates with N74D was low (0.6%–37% of wild‐type) in PBMC assays, demonstrating a significant decrease in viral fitness.

**Conclusions**: The N74D capsid RAM showed variable levels of LEN resistance and was associated with low RC, consistent with prior observations. The infrequency of breakthrough infection with resistance development, combined with the fitness cost of LEN RAMs, has favourable implications for the risk of resistance accumulation in circulating viral populations.

**Funding**: Gilead Sciences, Inc.

### P013 Uptake, Adherence and Effectiveness Over 4 Years of Cabotegravir Long‐Acting Pre‐Exposure Prophylaxis in the US OPERA Cohort

Ricky Hsu^1,2^, Laurence Brunet^3^, Anthony Mills^4^, Kevin Frost^5^, Gerald Pierone Jr ^6^, Michael Sension^7^, Philip Lackey^8^, Karam Mounzer^9^, Michael Wohlfeiler^10^, Jennifer Fusco^3^, Todd McKeon^11^, Piotr Budnik^12^, Adrienne Guignard^13^, Vani Vannappagari^11^, Gregory Fusco^3^

^1^AIDS Healthcare Foundation, New York, NY, US; ^2^NYU Langone Medical Center, New York, NT, US; ^3^Epividian, Raleigh, NC, US; ^4^Men's Health Foundation, West Hollywood, CA, US; ^5^No affiliation, New York, NY, US; ^6^Whole Family Health Center, Vero Beach, FL, US; ^7^CAN Community Health, Fort Lauderdale, FL, US; ^8^No affiliation, Winston‐Salem, NC, US; ^9^Philadelphia FIGHT, Philadelphia, PA, US; ^10^AIDS Healthcare Foundation, Miami, FL, US; ^11^ViiV Healthcare, Durham, NC, US; ^12^ViiV Healthcare, London, UK; ^13^ViiV Healthcare, Wavre, Belgium

**Background**: Cabotegravir long‐acting pre‐exposure prophylaxis (CAB LA PrEP) has been available in the US since 20 December 2021. Its use, adherence and effectiveness are described over the first 4 years of availability.

**Methods**: Adults with ≥1 CAB LA PrEP injection between 20 December 2021 and 30 June 2025 in the US OPERA cohort were followed through 31 December 2025. Initiation was completed if the second injection was received ≤67 days after the first (on time: ≤37 days). Delays (i.e. maintenance injection received 68–127 days after the preceding injection), were considered short (68–97 days) or long/requiring re‐initiation (98–127 days). Incidence rates of HIV testing (antibody/antigen/RNA), sexually transmitted infections (STIs) and HIV acquisition were estimated using univariate Poisson regression.

**Results**: We identified 3021 CAB LA PrEP users, including 4% aged ≥60 years, 12% women, 7% transgender, 39% with a recent history of STIs and 73% with prior oral PrEP use (Table 1). Most (88%) completed the initiation sequence, of whom 2268 (86%) received the second injection on time. Complete initiators were followed for a median 12.8 months (IQR 8.1, 21.9) and received a median 7 injections (IQR 4, 11). Of those with ≥1 maintenance injection, 58% had all injections on time. Of 16,268 total maintenance injections, 14,748 injections (91%) were on time; most (84%) delays were short (median 4 days late; IQR 2, 10). Even after incomplete initiation or discontinuation, 36% (137/377) and 21% (237/1138) subsequently resumed CAB LA PrEP injections, respectively (Figure 1). Among all CAB LA PrEP users, HIV testing was performed at a rate of 5.90 tests per person‐years (5.83, 5.98). There were 45.39 STI diagnoses per 100 person‐years (95% CI: 42.77, 48.18) and 0.17 HIV acquisitions per 100 person‐years (0.08, 0.37).

**Conclusions**: In this large, real‐world US cohort of diverse individuals using CAB LA PrEP, high levels of adherence to the injection schedule were observed and delays, when they occurred, tended to be short. CAB LA PrEP resumption after incomplete initiation or discontinuation suggests continued interest and may reflect the dynamic nature of PrEP needs over time. Importantly, CAB LA PrEP was highly effective for HIV prevention, with a very low rate of HIV acquisition despite ongoing risk demonstrated by high STI rates.

**FIGURE 1:**
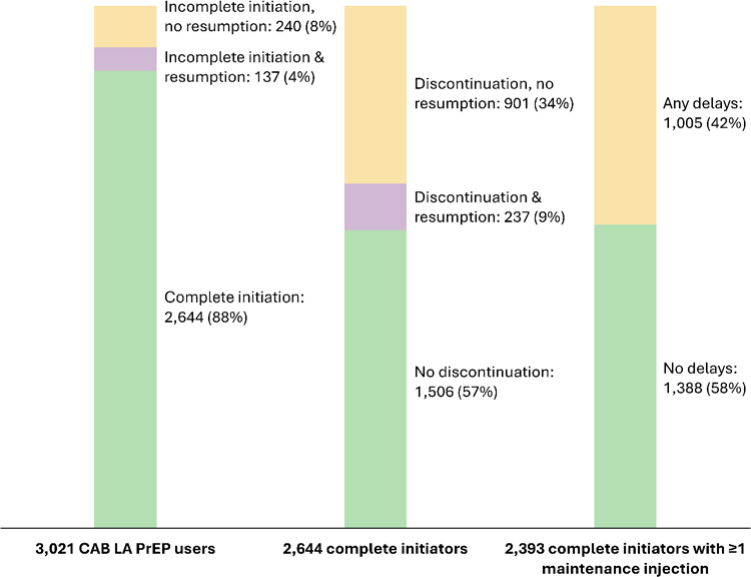
P013 | CAB LA PrEP initiation, discontinuation and adherence to injections.

**TABLE 1:**
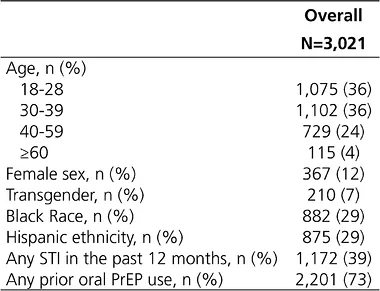
P013 | Characteristics at CAB LA PrEP start, overall and stratified by complete initiation and discontinuation.

### P014 Injection‐Site Reactions Drive Early Discontinuation of Lenacapavir for HIV PrEP in Real‐World Clinical Practice

Joseph Cherabie ^1^, Tommy Schafer^1^, Andrew Crone^1^, Aniruddha Hazra^1,2^

^1^Howard Brown Health, Chicago, IL, US; ^2^University of Chicago, Chicago, IL, US

**Background**: Lenacapavir (LEN), a capsid inhibitor administered by subcutaneous injection every 6 months, was recently approved in the US for HIV pre‐exposure prophylaxis (PrEP). We evaluated early implementation outcomes following LEN initiation at one of the largest PrEP programmes in the US.

**Methods**: We performed a retrospective cohort study of all individuals initiating LEN PrEP at Howard Brown Health through 15 June 2026. Demographic and clinical characteristics were abstracted from the electronic health record. Implementation outcomes included PrEP‐naïve LEN initiation, injection‐site reactions (ISRs) requiring clinical contact (telephone call, patient portal message or office visit) and LEN discontinuation. Logistic regression was used to identify factors associated with these implementation outcomes.

**Results**: During the study period, 310 individuals initiated LEN PrEP, of whom 52 (17.4%) had completed their second injection visit. Cohort characteristics are summarized in Table 1. Overall, 48 individuals (15.5%) were PrEP‐naïve. Compared with PrEP‐experienced LEN initiators, PrEP‐naïve individuals were more likely to be cisgender women (OR 4.06, 95% CI: 1.27–12.91; *p* = 0.004) or non‐binary (OR 4.49, 95% CI: 1.72–11.73; *p* = 0.004). ISRs requiring clinical contact occurred in 33 (10.7%) individuals, with most (24, 72.7%) following abdominal injections. Over 154.8 person‐years of follow‐up, 15 individuals (4.8%) discontinued LEN PrEP. Discontinuation was associated with experiencing an ISR requiring clinical contact (OR 12.34, 95% CI: 4.13–36.86; *p* < 0.0001).

**Conclusions**: Nearly one in six individuals initiating LEN PrEP had never previously received PrEP, suggesting that LEN may expand PrEP uptake beyond traditional populations, particularly among cisgender women and gender‐diverse individuals. Although early discontinuation was uncommon (4.8%), it exceeded that reported in the PURPOSE 1 (0.2%) and PURPOSE 2 (1.2%) trials. ISRs requiring clinical contact were the only factor associated with discontinuation, highlighting injection tolerability as a key determinant of early persistence. These findings suggest that optimizing the injection experience through patient‐centred counselling and proactive management of ISRs may be among the most impactful strategies to maximize long‐term persistence with LEN PrEP.

**TABLE 1:**
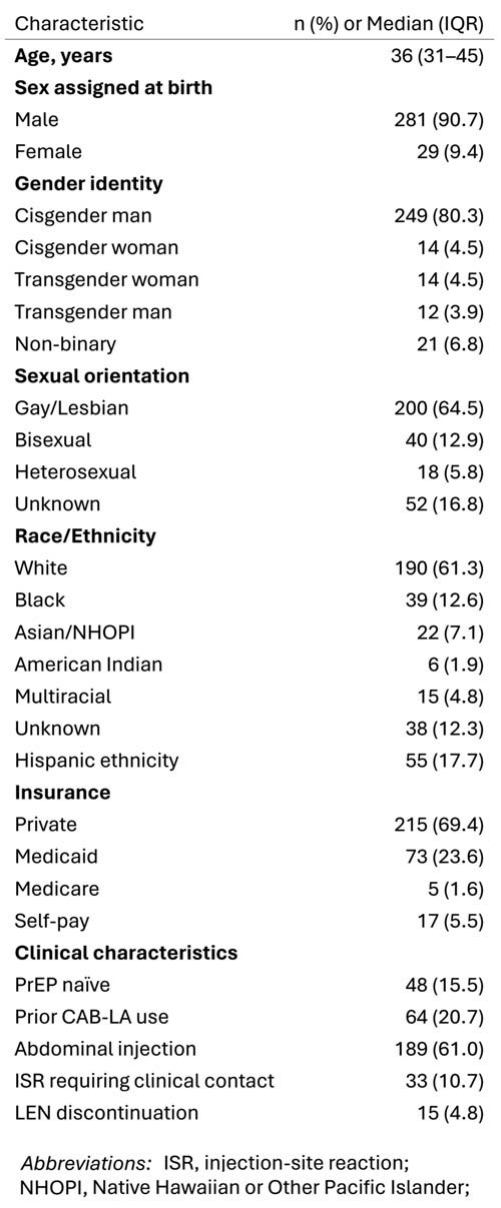
P014 | Baseline characteristics of individuals initiating lenacapavir PrEP (*n* = 310).

### P015 EXPrESSIVE‐11: A Phase 3 Study Evaluating MK‐8527 Oral Once Monthly for HIV Pre‐Exposure Prophylaxis in Cisgender Men Who Have Sex With Men, Transgender Women, Transgender Men and Gender Non‐Binary Individuals Who Are at Greater Likelihood of HIV Exposure

Raphael J. Landovitz^1^, Susan Buchbinder^2^, David V. Glidden^3^, Beatriz Grinsztejn^4^, Jean‐Michel Molina^5^, Fredrick O. Otieno^6^, Nittaya Phanuphak^7^, Adrienne E. Shapiro^8^, Dawn Averitt^9^, Jean Marie Arduino^10^, Lina De Montigny^11^, Jemar Bather^10^, Peggy May Tan Hwang^10^, Barbara Evans^10^, Luisa M. Stamm^10^, Christopher J. Bruno^10^, Rebeca M. Plank ^10^

^1^UCLA Center for Clinical AIDS Research and Education, Los Angeles, CA, US; ^2^San Francisco Department of Health, San Francisco, CA, US; ^3^UC San Francisco, San Francisco, CA, US; ^4^Evandro Chagas National Institute of Infectious Diseases‐Fiocruz, Rio de Janeiro, Brazil; ^5^Université Paris Cité; Assistance Publique‐Hôpitaux de Paris, Hôpital Saint‐Louis and Hôpital Lariboisière; INSERM UMR1342, Paris, France; ^6^Nyanza Reproductive Health Society, Kisumu, Kenya; ^7^Institute of HIV Research and Innovation, Bangkok, Thailand; ^8^University of Washington and Fred Hutch Vaccine Trials Unit, Seattle, WA, US; ^9^The Well Project, Brooklyn, NY, US; ^10^Merck & Co., Inc., Rahway, NJ, US; ^11^Merck Canada Inc., Kirkland, Canada

**Background**: Despite effective HIV pre‐exposure prophylaxis (PrEP), unmet prevention needs remain among cisgender men who have sex with men (MSM), transgender women (TGW), transgender men (TGM) and gender non‐binary (GNB) individuals with male sexual partners. Adherence drives effectiveness of daily oral PrEP; however, access barriers, stigma and daily dosing limit uptake and persistence. Long‐acting injectables may overcome some barriers but present implementation and preference challenges. Long‐acting oral PrEP may address barriers to PrEP uptake and improve adherence. MK‐8527 is a nucleoside reverse transcriptase translocation inhibitor with potent antiretroviral activity against HIV‐1 that reaches predicted protective exposure within 1 h and has a long intracellular half‐life and low potential for clinically relevant drug−drug interactions, supporting development as once‐monthly (qm) oral PrEP. In adults without HIV, single (0.5–200 mg) and multiple (5–40 mg once weekly for 3 weeks in phase 1 and 3–12 mg qm for 6 months in phase 2) doses of MK‐8527 were generally well tolerated. In treatment‐naïve adults living with HIV‐1, single doses as low as 0.5 mg achieved ≥1.0 log_10_ decreases in HIV‐1 RNA 7 days after administration.

**Methods**: EXPrESSIVE‐11 (MK‐8527‐011; NCT07044297) is an ongoing randomized, active‐controlled, double‐blind, multicentre, endpoint‐driven phase 3 study (Figure 1) evaluating the efficacy and safety of MK‐8527 11 mg oral qm versus emtricitabine/tenofovir disoproxil fumarate (FTC/TDF) oral daily for HIV‐1 PrEP. EXPrESSIVE‐11 will enrol ∼4390 MSM, TGW, TGM and GNB individuals aged ≥16 years at increased likelihood of HIV‐1 exposure, at sites in Africa, Asia, Europe, Latin America and North America. Participants are randomized 1:1 to MK‐8527 11 mg qm plus placebo daily or FTC/TDF daily plus placebo qm for ≤30 months. The primary efficacy endpoint is incidence of adjudicated HIV‐1 infection; primary safety endpoints include adverse events and discontinuations due to adverse events. An independent data monitoring committee provides safety oversight. This study was developed with input from investigators and community stakeholders and includes individuals aged ≥16 years and MSM, TGW, TGM and GNB populations. Exploratory assessments include viral resistance analyses, participant‐reported outcomes, renal biomarkers and body mass index. Completion of enrolment is expected by September 2026. Demographics and baseline characteristics will be presented.

**FIGURE 1:**
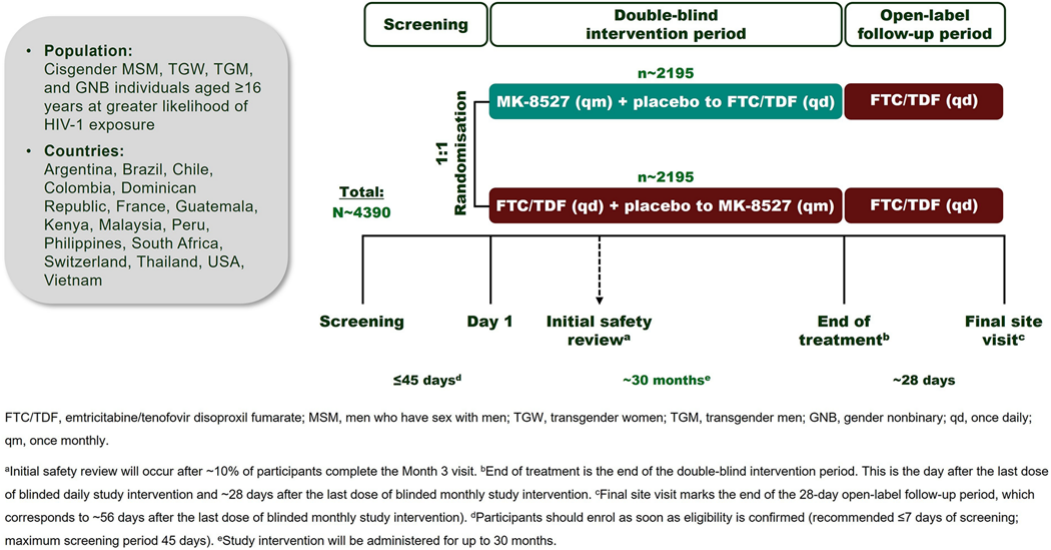
P015 | EXPrESSIVE‐11 phase 3 study design.

### P016 Pre‐Exposure Prophylaxis With Cabotegravir Long‐Acting Versus Daily Oral Tablets: Increased Frequency of Testing for HIV and Sexually Transmitted Infections

Gerald Pierone Jr ^1^, Rachel Palmieri Weber^2^, Laurence Brunet^2^, Anthony Mills^3^, Michael Sension^4^, Philip Lackey^5^, Michael Wohlfeiler^6^, Jennifer Fusco^2^, Supriya Sarakar^7^, Vani Vannappagari^8^, Kimberley Brown^8^, Gregory Fusco^2^

^1^Whole Family Health Center, Vero Beach, FL, US; ^2^Epividian, Inc., Raleigh, NC, US; ^3^Men's Health Foundation, West Hollywood, CA, US; ^4^CAN Community Health, Sarasota, FL, US; ^5^No affiliation, Winston Salem, NC, US; ^6^AIDS Healthcare Foundation, Miami, FL, US; ^7^ViiV Healthcare, Durham, NV, US; ^8^ViiV Healthcare, Durham, NC, US

**Background**: Regular testing for HIV and sexually transmitted infections (STIs) is recommended for people on pre‐exposure prophylaxis (PrEP). More frequent clinic visits for cabotegravir long‐acting (CAB LA) injections may provide opportunities for earlier detection and management of HIV and STIs than with oral PrEP. We compared the timing of HIV and STI testing during CAB LA PrEP versus daily oral PrEP use in the US OPERA cohort.

**Methods**: Adults without HIV who completed CAB LA PrEP initiation between 01JAN2022‐31OCT2023 (Comparison analysis) or switched from oral PrEP between 01FEB2023‐31JAN2024 (Pre/Post analysis) were followed through study end (30APR2025), PrEP discontinuation, death or loss to follow‐up. In the Comparison analysis, new CAB LA PrEP users were matched with replacement on age, sex and location to 1−3 individuals starting new oral PrEP. In the Pre/Post analysis, comparison was made between the periods on oral PrEP (Pre) and on CAB LA PrEP (Post). On‐schedule HIV, syphilis, gonorrhoea and chlamydia testing was determined during CAB LA and oral PrEP use.

**Results**: In the Comparison analysis, CAB LA users (*N* = 880) had comparable characteristics to oral PrEP users (*N* = 2613), although CAB LA PrEP users were more likely to receive Medicaid and to have had a clinical visit in the past year (Table 1). In both the Comparison and the Pre/Post (*N* = 544) analyses, CAB LA PrEP use was associated with a higher proportion of on‐schedule HIV, syphilis and gonorrhoea testing, compared with oral PrEP use, with the greatest difference observed for HIV testing after PrEP initiation. No difference was observed in the frequency of on‐schedule chlamydia testing between CAB LA and oral PrEP in either analysis (Figure 1).

**Conclusions**: In this large, real‐world US study, use of CAB LA PrEP was associated with an overall increase in on‐schedule HIV and STI testing, compared to oral PrEP. Visits to receive CAB LA PrEP injections every 2 months may have the ancillary benefit of facilitating HIV and STI testing in accordance with clinical guidelines. The higher testing frequency associated with CAB LA PrEP injection visits has the potential to result in faster detection and lower transmission rates of HIV and STIs.

**FIGURE 1:**
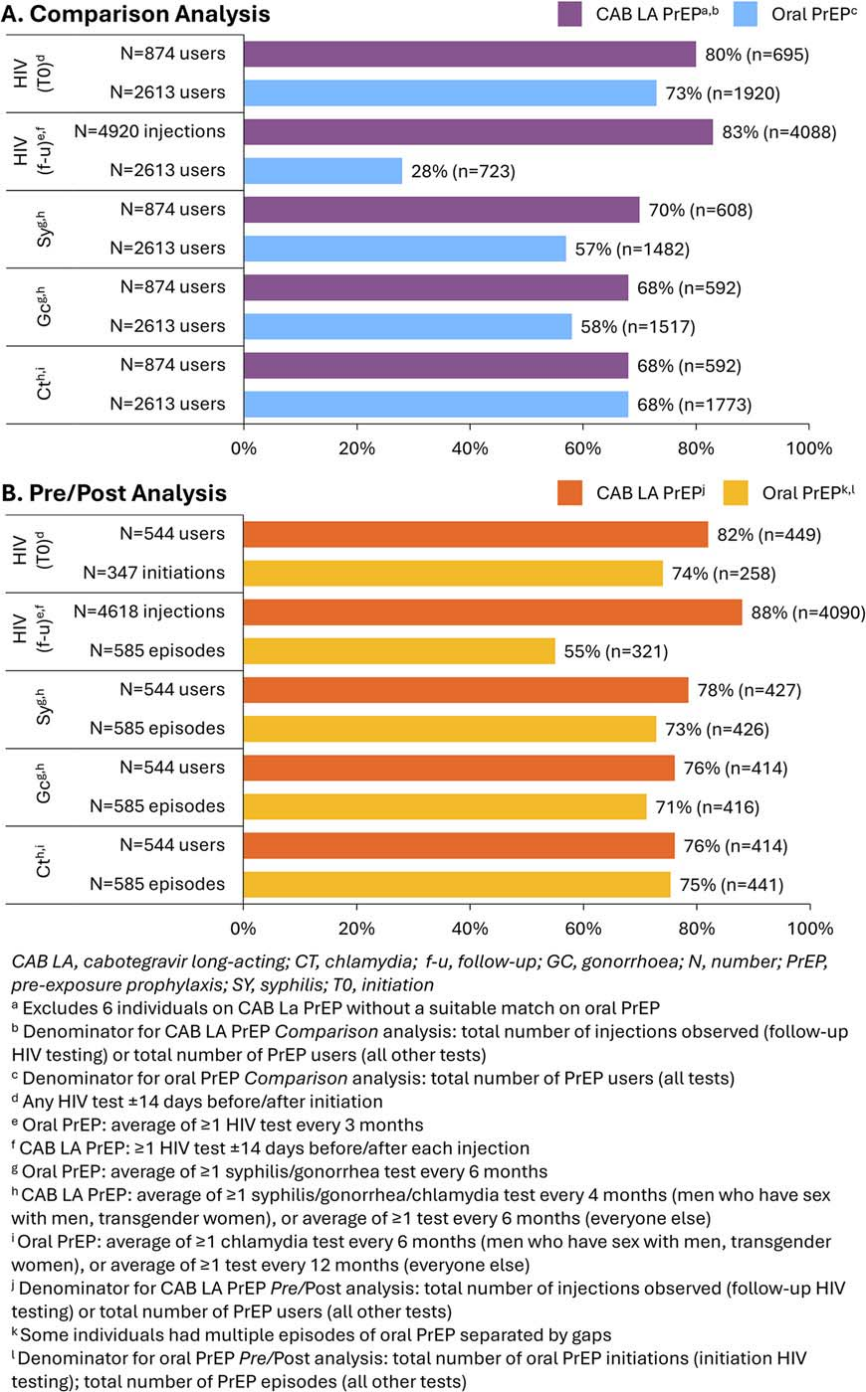
P016 | Proportion of HIV and STI tests on schedule among oral and CAB LA PrEP users in the Comparison and Pre/Post analyses.

**TABLE 1:**
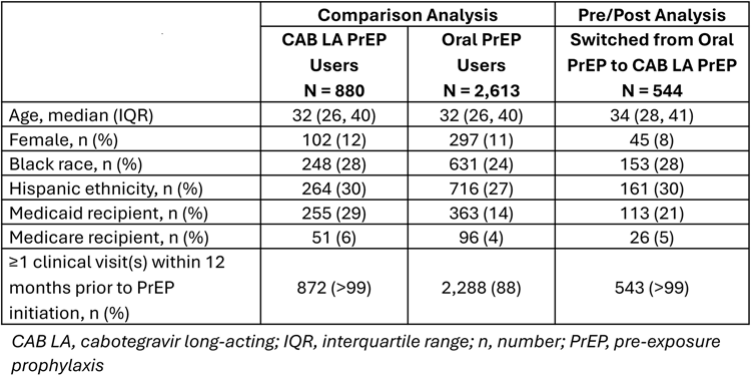
P016 | Population characteristics at CAB LA PrEP start (Comparison and Pre/Post analyses) or at oral PrEP start (Comparison analysis).

### P017 EXPrESSIVE‐10: A Phase 3 Study Evaluating MK‐8527 Oral Once Monthly for HIV Pre‐Exposure Prophylaxis in Cisgender Women Who Are at Greater Likelihood of HIV Exposure

Renee Heffron ^1^, Connie L. Celum^2^, Linda‐Gail Bekker^3^, Elizabeth A. Bukusi^2,4^, Sinead Delany‐Moretlwe^5^, Deborah Donnell^6^, Flavia Matovu Kiweewa^7^, Nelly Mugo^2,8^, Andrew Mujugira^2,9^, Simon Odiwuor Ondiek^10^, Nandisile Sikwana^11^, Elizabeth S. Russell^12^, Patricia Castronuovo^12^, Yash Kapoor^12^, John Love^12^, Anjana Grandhi^12^, Luisa M. Stamm^12^, Cyrus Badshah^12^, Rebeca M. Plank^12^

^1^University of Alabama at Birmingham, Birmingham, AL, US; ^2^Departments of Global Health, Medicine and Epidemiology, University of Washington, Seattle, WA, US; ^3^Desmond Tutu HIV Centre, University of Cape Town, Cape Town, South Africa; ^4^UC San Francisco, San Francisco, CA, US; ^5^Wits RHI, University of the Witwatersrand, Johannesburg, South Africa; ^6^Fred Hutchinson Cancer Center, Seattle, WA, US; ^7^Makerere University‐Johns Hopkins University Research Collaboration, Kampala, Uganda; ^8^Kenya Medical Research Institute (KEMRI), Nairobi, Kenya; ^9^The Infectious Diseases Institute, Makerere University, Kampala, Uganda; ^10^AVAC: Global Advocacy for HIV Prevention, Kisumu, Kenya; ^11^AVAC: Global Advocacy for HIV Prevention, Johannesburg, South Africa; ^12^Merck & Co., Inc., Rahway, NJ, US

**Background**: Despite effective HIV pre‐exposure prophylaxis (PrEP), gaps remain in meeting the prevention needs of African adolescent girls and young women (AGYW). Daily oral PrEP effectiveness is associated with adherence, while long‐acting injectable PrEP may present implementation and preference challenges; all PrEP options are limited by stigma and access barriers. Long‐acting oral PrEP could expand prevention choice and simplify delivery. MK‐8527 is a nucleoside reverse transcriptase translocation inhibitor that reaches predicted protective levels within 1 h and has potent antiretroviral activity against HIV‐1, a long intracellular half‐life and low potential for clinically relevant drug−drug interactions, supporting development as once‐monthly (qm) oral PrEP. In adults without HIV, single (0.5–200 mg) and multiple (5–40 mg once weekly; 3–12 mg qm) doses of MK‐8527 were generally well tolerated. In treatment‐naïve adults living with HIV‐1, single doses as low as 0.5 mg achieved ≥1.0 log_10_ decreases in HIV‐1 RNA after 7 days.

**Methods**: EXPrESSIVE‐10 (MK‐8527‐010; NCT07071623) is an ongoing randomized, active‐controlled, double‐blind, multicentre phase 3 study (Figure 1) evaluating the efficacy and safety of MK‐8527 11 mg oral qm versus emtricitabine/tenofovir disoproxil fumarate (FTC/TDF) oral daily as HIV‐1 PrEP. EXPrESSIVE‐10 will enrol ∼4580 cisgender AGYW aged 16–30 years who are at increased likelihood of HIV‐1 exposure across 30 sites in Kenya, South Africa and Uganda. Participants are randomized 1:1 to MK‐8527 11 mg qm plus daily placebo or FTC/TDF daily plus monthly placebo for up to 24 months. The primary efficacy endpoint is incidence of adjudicated HIV‐1 infection; primary safety endpoints include adverse events and discontinuations due to adverse events. An independent data monitoring committee provides safety oversight.

This endpoint‐driven study was developed with input from investigators and community representatives to ensure participant‐centred implementation. The study includes enrolment of adolescents aged 16–17 years following an interim safety analysis, supporting evidence generation in an underrepresented population, optional continued participation during pregnancy and breastfeeding, maternal/infant follow‐up and evaluation of concomitant hormonal contraceptive use. Exploratory assessments include viral resistance, participant‐reported outcomes, renal biomarkers and body mass index. Enrolment is expected to be completed by September 2026. Demographics and baseline characteristics will be presented.

**FIGURE 1:**
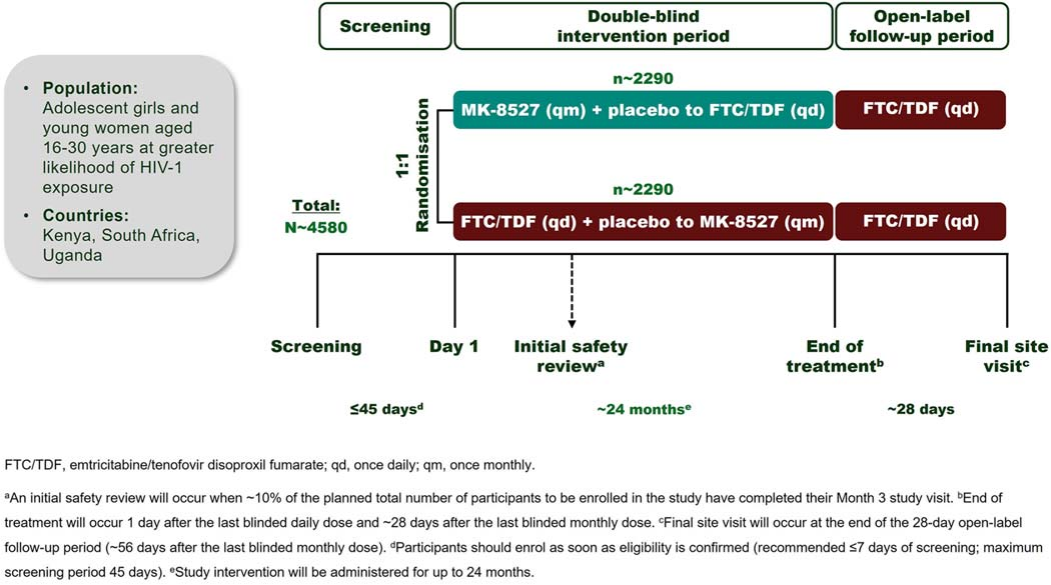
P017 | EXPrESSIVE‐10 phase 3 study design.

### P018 Abstract Withdrawn

### P019 Impact of Follow‐Up Frequency on STI Detection and Prevention Among Men Who Have Sex With Men (MSM) and Women Using HIV PrEP: A Decision‐Analytic Model

Eduard Vidal‐Barraquer^1^, Natalya Danchenko^2^, Sarah‐Jane Anderson^3^, Mairi Ward^1^, Ashley Davis^4^, Heidi Swygard^5^, Aimee Metzner^5^, Barry S. Zingman^6^, Kevin Hatfield^7^, Brittany Marie Williams^8^, Nasser Mohammed^9^, Claire Dewsnap^10^, Juddy Otti^11^, Jenny Scherzer^12^, Laure Dupont Benjamin^2^, Jonathan Tosh ^3^, Sean Robbins^13^, Jean van Wyk^3^

^1^LatiicePoint Consulting, Barcelona, Spain; ^2^ViiV Healthcare, Paris, France; ^3^ViiV Healthcare, London, UK; ^4^RTI Health Solutions, Durham, NC, US; ^5^ViiV Healthcare, Durham, NC, US; ^6^Albert Einstein College of Medicine, Bronx, NY, US; ^7^Optum Care, Seattle, WA, US; ^8^Department of Education, University of Vermont, Burlington, VT, US; ^9^Osra Medical; Alwan Foundation, San Francisco, CA, US; ^10^NHS Consultant, President of BASHH, Sheffield, UK; ^11^Africa Advocacy Foundation, London, UK; ^12^ViiV Healthcare, Berlin, Germany; ^13^LatiicePoint Consulting, Geneva, Switzerland

**Background**: Guidelines recommend sexually transmitted infection (STI) screening every 3−6 months for people on HIV PrEP, with the US CDC recommending quarterly testing for MSM [1−3] to support early detection and prevent onward transmission. As longer‐acting PrEP options emerge, screening frequency may vary, potentially reducing opportunities for STI detection. We assessed individual and population health impact of triannual (3x yearly) versus semiannual (2x yearly) screening.

**Methods**: A decision‐analytic model comprising STI‐specific decision trees for gonorrhoea, chlamydia and syphilis estimated detection, partner tracking, onward transmission and long‐term sequelae with 3x versus 2x yearly screening. A cohort of 10,000 adult PrEP users was modelled over 2 years, including MSM and heterosexual women. Model inputs, including population‐ and STI‐specific incidence probabilities, were informed by published literature. Missed or delayed detection was modelled across screening schedules and STI testing adherence levels, defined as full (i.e. STI screening at every scheduled visit) or moderate (i.e. screening at every other visit). Uncertainty was explored through scenario analyses.

**Results**: 3x yearly screening increased detected cases by 10.9% and reduced delayed/missed detection by 25.0%, with a corresponding 39.9% reduction in the risk of onward transmission, versus 2x yearly. When screening adherence decreased, detection declined across both schedules (5310 to 4018 cases for 3x yearly; 4790 to 3244 for 2x yearly), with a 23.9% higher detection rate for 3x yearly versus 2x yearly, alongside reductions in delayed/missed detection (−21.4%) and onward transmissions (−21.6%). Long‐term sequelae, although infrequent, showed similar patterns. Under full adherence, 3x yearly screening reduced key outcomes by 30.2–36.6% versus 2x yearly (epididymitis: 32 vs. 50 cases per 10,000; pelvic inflammatory disease: 17 vs. 25 per 10,000). With lower screening adherence, prevention of long‐term sequelae declined across both schedules, yet 3x yearly screening maintained a 19.7–20.9% higher detection rate than 2x yearly for both sequelae.

**Conclusions**: Maintaining regular HCP interactions improved STI detection and was associated with reduced risk of onward transmission, supporting individual and population health. 3x yearly STI screening yielded more favourable outcomes than 2x yearly under both adherence scenarios, highlighting the importance of structured, regular STI screening within HIV prevention pathways.

**References**

1. CDC Clinicians’ Quick Guide—Injectable PrEP: Bacterial STI screening at least every 4 months. Accessible at: https://www.cdc.gov/hivnexus/media/pdfs/2024/04/cdc‐lsht‐prevention‐brochure‐clinicians‐quick‐guide‐what‐is‐injectable‐hiv‐prep.pdf Accessed 15/06/2026.

2. BHIVA/BASHH PrEP Guidelines (2025): STI testing included in ongoing monitoring of PrEP users. Accessible at: https://www.bashh.org/_userfiles/pages/files/draft_bashh_bhiva_prep_guidelines_240924_v30_final.pdf Accessed 15/06/2026.

3. EACS Guidelines (2025): HIV testing every 2–4 months; regular STI screening during PrEP use. Accessible at: https://eacs.sanfordguide.com/en/eacs‐hiv/art/eacs‐pre‐exposure‐prophylaxis. Accessed: 15/06/2026.

### P020 Willing but Unable: How Intersecting Vulnerabilities and Structural Barriers Fracture the PrEP Cascade Among Migrants in Europe

Haoyi Wang ^1,2^, Hala Majdoub^3^, Anne Flaherty‐Gupta^3^, Kim Isip^4^, Pedro Barrios Sanchez^5^, Francesco Bellissimo^6^, Yiannis Charilaou^7^, Christos Krasidis^7^, Rosaline M'Bayo^8^, Stephen Amoah^8^, Trésors Kouadio^9^, Robert Ian Kibet Ouko Babu^10^, Ann Piercy^11^, Abimbola Ajomale^12^, Moro Yapha^13^, Etinosa Erevbenagie‐Johnbull^14^, Letonde Hermine Gbedo^15^, Kai Jonas^1^, Hanne Zimmermann^1^, Denis Onyango^3^

^1^Department of Work and Social Psychology, Maastricht University, Maastricht, Netherlands; ^2^Viroscience Department, Rotterdam, Netherlands; ^3^Africa Advocacy Foundation, London, UK; ^4^Association PASTT, Paris, France; ^5^Asociacion Karibu, Madrid, Spain; ^6^LHIVE Diritti e Prevenzione, Catania, Spain; ^7^AIDS Solidarity Movement, Nicosia, Cyprus; ^8^Afrikaherz (VIA e.V.), Berlin, Germany; ^9^Plateforme Prévention SIDA, Brussels, Belgium; ^10^Open Paths Athens, Athens, Greece; ^11^HIV Ireland, Dublin, Ireland; ^12^Noaks Ark Mosaik, Malmö, Sweden; ^13^Fixpunkt e.V., Berlin, Germany; ^14^Generation for Change CY, Nicosia, Cyprus; ^15^Comitato per i Diritti Civili delle Prostitute, Trieste, Italy

**Background**: Despite increasing PrEP availability across Europe, migrants continue to face barriers throughout the PrEP cascade. We examined an extended PrEP cascade among migrants attending community‐based HIV outreach events and assessed intersectional inequalities in cascade engagement.

**Methods**: We analysed bio‐behavioural cross‐sectional data from community‐led outreach HIV test events across 10 European countries (September 2024−October 2025), including 2498 participants with non‐reactive HIV results. PrEP cascade indicators included awareness, intention, previous PrEP seeking from healthcare providers, current uptake and unmet need (intention without current use). Univariable logistic regression was used to compare migrants and non‐migrants. Among migrants, Multilevel Analysis of Individual Heterogeneity and Discriminatory Accuracy (MAIHDA) models were fitted across 56 intersectional strata defined by legal status, key population membership, sex work involvement and injection drug use, adjusting for duration of residence and national PrEP reimbursement policies.

**Results**: Overall, 22.4% (95% CI: 20.8–24.1) reported PrEP awareness, 28.7% (26.9–30.5) intention, 8.5% (7.4–9.6) previous PrEP seeking and 3.5% (2.8–4.2) current use; unmet need was 25.2% (23.5–26.9). Compared with non‐migrants, migrants had higher odds of PrEP intention (OR 1.61, 95% CI: 1.20–2.18), but lower odds of having sought PrEP (OR 0.48, 95% CI = 0.34–0.69) and current use (OR 0.58, 95% CI: 0.34–1.04), resulting in nearly double the unmet need (OR 1.99, 95% CI: 1.44–2.82). Undocumented migrants had the lowest awareness (19.5%), but the highest intention (39.6%) and unmet need (35.1%). Awareness and uptake were highest among MSM, trans* individuals and sex workers, yet these groups also experienced the greatest unmet need (Figure 1). MAIHDA revealed substantial intersectional heterogeneity, particularly for PrEP seeking (VPC 61.3%) and uptake (VPC 53.7%). Although most between‐stratum variance was explained after adjustment (PCV 95%–100%), unmet need retained residual intersectional variance (adjusted VPC 12.5%), suggesting non‐additive inequalities beyond measured individual and structural factors. Restrictive reimbursement policies were strongly associated with lower seeking and uptake but higher unmet need; asylum seekers and undocumented migrants had lower predicted probabilities of early cascade engagement.

**Conclusions**: High PrEP demand among migrants is not translating into access or uptake, particularly among those with insecure legal status. Persistent intersectional inequalities in unmet need suggest that policy reform alone will be insufficient. Inclusive reimbursement frameworks and community‐based PrEP delivery models are urgently needed to convert demand into equitable PrEP uptake.

**FIGURE 1:**
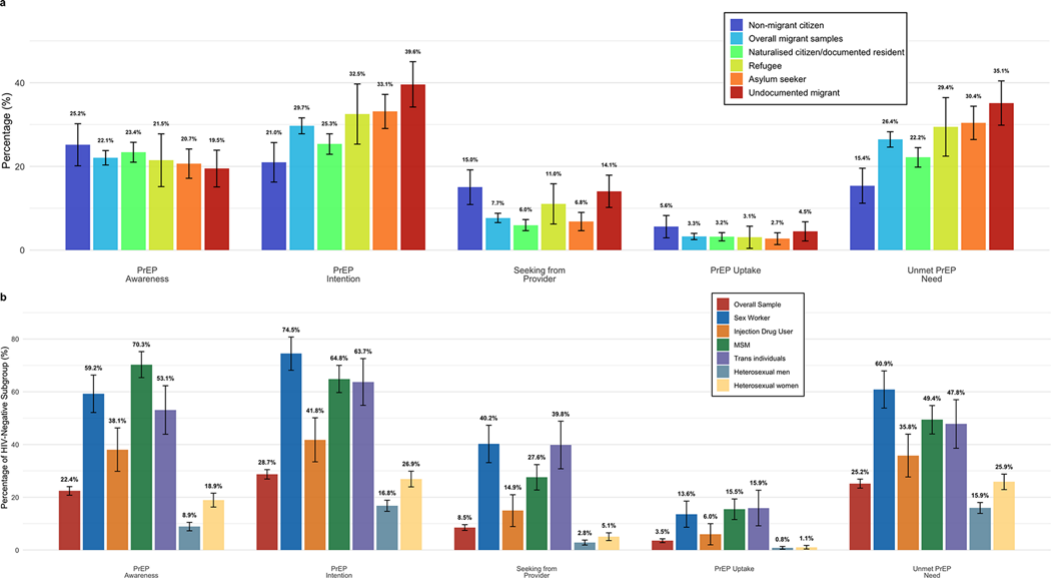
P020 | PrEP cascade across (a) migrant status and legal status and (b) key populations.

### P021 Impact of Doxycycline Post‐Exposure Prophylaxis (Doxy‐PEP) Implementation on Reducing Syphilis Incidence Among HIV PrEP Users: A Real‐World Experience at Taoyuan General Hospital, Taiwan

Jen‐Chung Chu, Che‐Yu Kuo

Comprehensive AIDS Care Center, Taoyuan General Hospital, Taoyuan City, Taiwan

**Background**: While pre‐exposure prophylaxis (PrEP) is highly effective in preventing HIV, the rising incidence of bacterial sexually transmitted infections (STIs), particularly syphilis, remains a significant challenge. Doxycycline post‐exposure prophylaxis (doxy‐PEP) has emerged as a promising strategy. This study evaluates the impact of integrating doxy‐PEP into a government‐funded PrEP programme at Taoyuan General Hospital on the rates of syphilis reinfection and new infections.

**Methods**: A retrospective cohort study was conducted involving individuals enrolled in the government‐funded PrEP programme at Taoyuan General Hospital from January 2025 to December 2025. We tracked the monthly uptake of doxy‐PEP among PrEP users and monitored monthly syphilis screening results. The primary outcomes were the total number of syphilis‐positive cases and the number of newly acquired infections (primary/secondary syphilis) following the introduction and scaling of doxy‐PEP (Figures 1 and 2).

**Results**: Data analysis showed a steady increase in the number of PrEP users opting for doxy‐PEP over the 12‐month period. Concurrently, a downward trend in syphilis infections was observed. Specifically, as doxy‐PEP uptake increased from 46 to 81 users per month, the monthly number of new syphilis infections decreased from 10 to 1 case. Initial findings suggest a significant reduction in recurrence among high‐risk users who consistently applied the doxy‐PEP protocol (200 mg within 72 h of condomless sex).

**Conclusions**: The implementation of doxy‐PEP within a public health PrEP framework in Taiwan significantly reduced the burden of syphilis among at‐risk populations. These real‐world results from Taoyuan General Hospital support the routine integration of doxy‐PEP for PrEP users to mitigate STI rates. Further research is needed to evaluate long‐term antibiotic resistance and cost‐effectiveness in the Asian countries.

**FIGURE 1:**
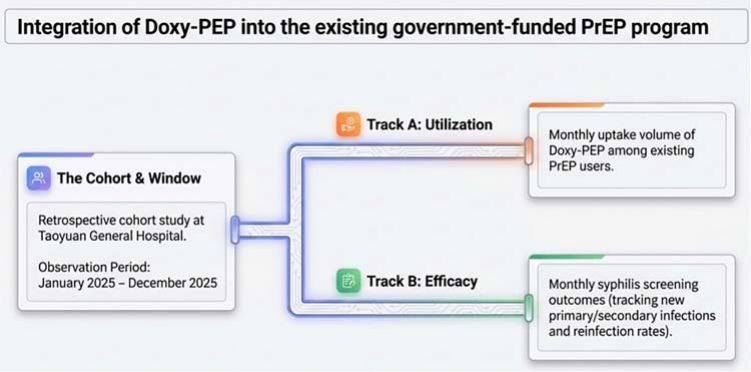
P021 | Integration of doxy‐PEP into the existing government‐funded PrEP programme.

**FIGURE 2:**
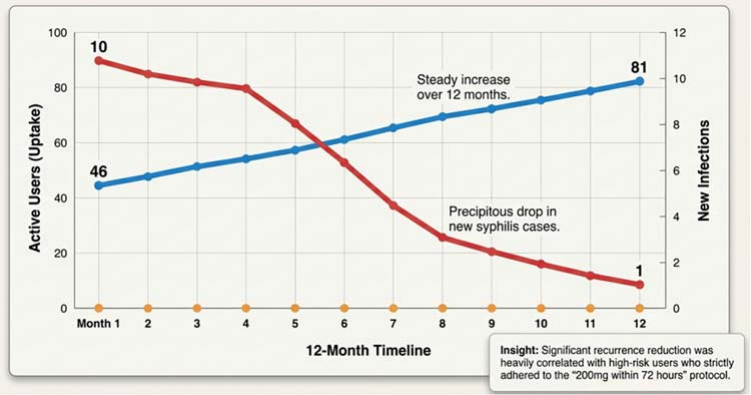
P021 | Correlation between syphilis PEP adherence and infection rates, a 12‐month analysis.

### P022 Estimating Unmet Need for PrEP in France Using National Claims Data and Machine Learning

Marc Tassi^1^, Lauren Inchboard^2^, Martin Siguier^3^, Marine Vierling^2^, Sophie Larrieu^2^, Sparkle Russell‐Puleri^4^, Claire Idelovici‐Marchal^5^, Elias Benabadji ^5^, Romain Palich^6^, Olivier Robineau^7^

^1^CHUR Tours, Tours, France; ^2^HORIANA, Bordeaux, France; ^3^Hôpital Tenon, APHP, Paris, France; ^4^Gilead Sciences, Foster City, CA, US; ^5^Gilead Sciences, Puteaux, France; ^6^Hôtel‐Dieu, AP‐HP, Paris, France; ^7^CHU Lille, Lille, France

**Background**: The population at risk of HIV acquisition in France remains difficult to quantify using conventional epidemiological approaches alone. We aimed to estimate the size and characteristics of individuals at high risk of HIV acquisition using nationwide claims data and machine‐learning methods.

**Methods**: Epi‐PrEP is a retrospective study using the French national claims database covering 99% of the population and its representative 2% sample. First, to train sex‐specific machine‐learning models predicting incident HIV diagnosis, individuals newly diagnosed with HIV between 2016 and 2023 were matched with HIV‐negative controls not using PrEP. Candidate predictors included demographics, hospital diagnoses, laboratory tests, prescriptions and healthcare‐use indicators over the previous 3 years. Second, the models were applied to the representative sample to estimate the prevalence of individuals with healthcare‐consumption profiles consistent with elevated HIV acquisition risk (threshold defined graphically as the inflection point where relative risk rose sharply).

**Results**: Of 553 candidate predictors, 313 and 266 were selected for men and women, respectively. Overall, 0.35% of women and 0.58% of men were classified as high risk, corresponding nationally to 99,135 women and 149,825 men (248,960 individuals). This population was almost four times larger than the number of PrEP users in France (67,505 [1]). High‐risk individuals were younger and more frequently located in the Paris region than low‐risk individuals (Table 1). Profiles differed markedly by sex: high‐risk men had substantially higher recent HIV testing than low‐risk men (79.4% vs. 5.8%), whereas high‐risk women showed lower healthcare utilization, including fewer GP visits (median 1.0 vs. 10.0), more frequent residence in overseas territories (18.0% vs. 2.8%) and greater social vulnerability, including higher coverage by low‐income insurances (53.4% vs. 11.4%) and state‐funded healthcare for migrants (2.3% vs. 0.1%).

**Conclusions**: This study provides one of the first estimates of the population potentially eligible for PrEP in France. The consistency of sex‐specific model outputs with known differences in HIV risk profiles between men and women supports the clinical plausibility of the approach. The estimated PrEP‐eligible population markedly exceeds current uptake, highlighting a major unmet prevention need. Claims‐based machine learning can complement epidemiological approaches to quantify PrEP need, while community‐based data may help capture underserved populations.

**References**

1. Billioti de Gage S, Desplas D, Dray‐Spira R. *Suivi de l'utilisation de Truvada ou génériques pour une PrEP au VIH: actualisation au 30 juin 2025*. EPI‐PHARE; 2025. Accessed 27 May 2026. Available from: https://www.epi‐phare.fr/rapports‐detudes‐et‐publications/suivi‐prep‐vih‐2025/

**TABLE 1:**
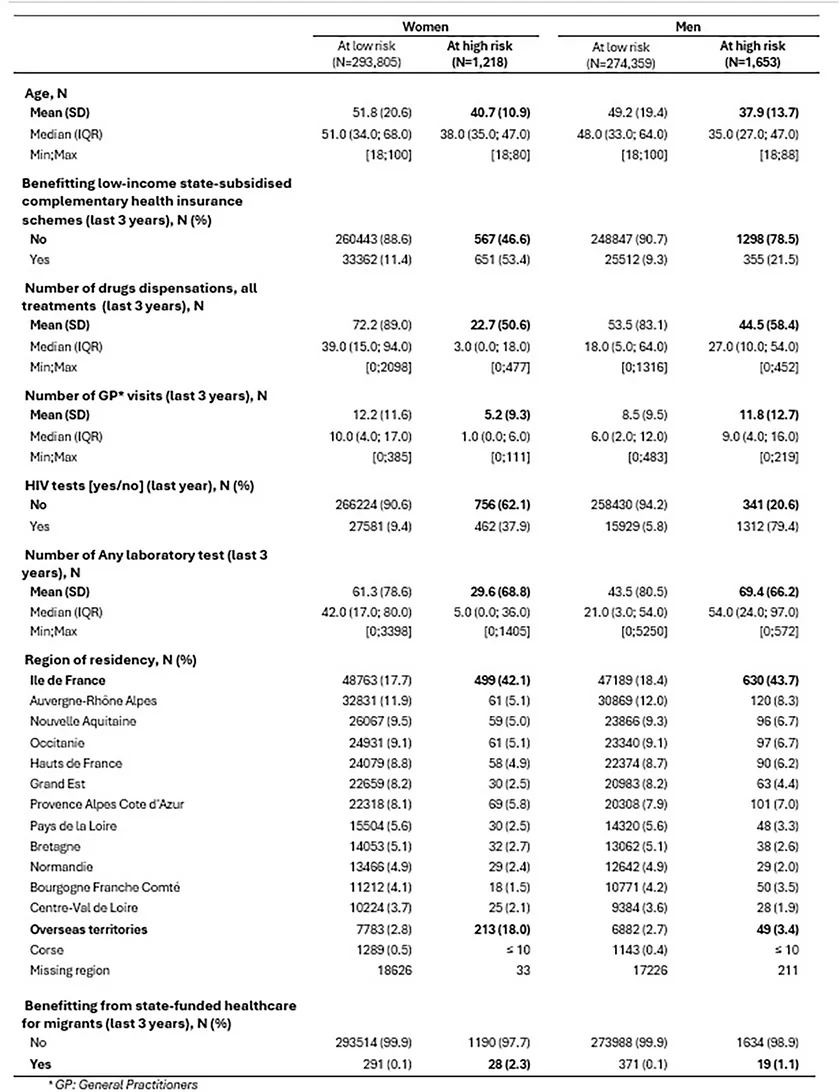
P022 | Patient characteristics and healthcare utilization by sex and predicted HIV acquisition risk, based on top‐ranked model predictors.

### P023 Relationships of Pre‐Exposure Prophylaxis (PrEP) Side Effect Concerns and Modality Preference Among American Gay, Bisexual and Other Men Who Have Sex With Men (GBMSM)

Katie B. Biello ^1,2^, Iju Shakya^1^, Ronnie M. Gravett^3^, Jean Marie Arduino^4^, Katia J. Bruxvoort^5^, Yohance Whiteside^4^, Megan E. McCabe^6^, Valerie Raziano^4^, Kenneth Mayer^2^

^1^Department of Epidemiology, School of Public Health, Brown University, Providence, RI, US; ^2^Fenway Health, The Fenway Institute, Boston, MA, US; ^3^Division of Infectious Diseases, Department of Medicine, Heersink School of Medicine, University of Alabama at Birmingham, Birmingham, AL, US; ^4^Merck & Co., Inc, Rahway, NJ, US; ^5^Department of Epidemiology, School of Public Health, University of Alabama at Birmingham, Birmingham, AL, US; ^6^Department of Biostatistics, School of Public Health, University of Alabama at Birmingham, Birmingham, AL, US

**Background**: With a growing number of PrEP modalities, understanding specific side effect concerns and how they correlate with PrEP preferences may help improve clinical communication and uptake among GBMSM.

**Methods**: In 2023, we mounted an online survey of American GBMSM, asking, “Which side effects would prevent you from using PrEP?”, focusing on 10 common side effects. Participants also ranked seven PrEP modalities as most or least preferred and had the option of indicating whether side effects affected their rankings. We categorized side effect concerns into systemic only (e.g. mild nausea), local only (e.g. injection site nodule) or both and fit a multinomial logistic model with the following explanatory variables: age, combined race/ethnicity, education and PrEP experience. We examined associations between selecting the least preferred PrEP modality due to side effects and type of side effect concerns (i.e. systemic, local, both) using separate two‐sample proportion tests.

**Results**: Among participants with PrEP indications (*n* = 686), 65.9% were PrEP‐experienced. The most common side effect concerns (> 30%) were nausea, diarrhoea and/or a small nodule. A majority (55.8%) of participants were concerned about systemic effects only, 21.7% about local effects only and 22.4% about both. In the multivariable analyses, compared to White, non‐Hispanic GBMSM, Black, non‐Hispanic (aOR 0.34; 95% CI: 0.18–0.67) and Hispanic or Latino (aOR 0.52; 95% CI: 0.30–0.90) GBMSM had lower odds of local effect concerns than systemic concerns. GBMSM with higher education had greater odds of being concerned about local effects versus systemic effects compared to those with less than a bachelor's degree (aOR 1.63; 95% CI: 1.06–2.51). Participants who selected oral PrEP modality as least preferred were more concerned about systemic effects than those who selected injectables or implants (74.7% vs. 43.1%, *p* < 0.01).

**Conclusions**: Black, Hispanic and GBMSM with less education were more likely to be concerned about PrEP‐related systemic (e.g. nausea) than local effects (e.g. injection site reactions). Concerns about nausea, diarrhoea and other systemic effects were more common among those who indicated oral PrEP as least preferred. As PrEP modality options expand, clinical communication approaches regarding anticipated side effects and their management may help mitigate concerns and improve shared decision‐making, while centring GBMSM autonomy and experiences.

### P024 HIV‐1 Molecular Characteristics and Transmitted Drug Resistance Mutations Epidemiology Affecting Pre‐Exposure Prophylaxis in the HIV‐1 A6 Epidemic in Poland—A Cross‐Sectional Study

Andrzej Załęski ^1,2^, Agnieszka Lembas^1,2^, Tomasz Dyda^3^, Justyna Orzechowska^4^, Aldona Kot^5^, Joanna Osińska^6^, Justyna Stempkowska‐Rejek^7^, Marcin Dręczewski^8^, Marta Dobrzyńska^1,2^, Michał Dowiatt^9^, Alicja Wiercińska‐Drapało^1,2^

^1^Hospital for Infectious Diseases in Warsaw, Warsaw, Poland; ^2^Department of Infectious Diseases, Tropical Diseases and Hepatology, Medical University of Warsaw, Warsaw, Poland; ^3^Molecular Diagnostics Laboratory, Hospital for Infectious Diseases in Warsaw, Warsaw, Poland; ^4^Clinical Department of Infectious Diseases, Medical Center in Łańcut, College of Medical Sciences, University of Rzeszów, Rzeszów, Poland; ^5^Department of Infectious Diseases and Hepatology, Medical University of Białystok, Białystok, Poland; ^6^Infectious Diseases Clinical Ward in Ostróda, Department of Family Medicine and Infectious Diseases, University of Warmia and Mazury in Olsztyn, Olsztyn, Poland; ^7^Department of Infectious Diseases and Hepatology, Medical University of Lublin, Lublin, Poland; ^8^Department of Infectious Diseases, Faculty of Medicine, Medical University of Gdańsk, Gdańsk, Poland; ^9^Students’ Scientific Society of the Department of Infectious Diseases, Tropical Diseases and Hepatology, Medical University of Warsaw, Warsaw, Poland

**Background**: The emergence and transmission of HIV variants harbouring resistance mutations or subtype‐specific polymorphisms may compromise the effectiveness of pre‐exposure prophylaxis (PrEP). The aim of the study was to assess the frequency of HIV DRMs to drugs used in PrEP.

**Methods**: In this multicentre cross‐sectional study (2023–2026), the frequency of HIV‐1 resistance mutations to PrEP: tenofovir disoproxil fumarate (TDF), emtricitabine (FTC) and cabotegravir (CAB) was assessed in treatment‐naïve individuals.

**Results**: Subtype‐specific analyses were conducted for subtype B and sub‐subtype A6. A total of 606 individuals were included; 47.19% with subtype B and 45.87% with subtype A, with A6 accounting for 94.24% of subtype A infections. The L74I polymorphism was detected in 88.07% of A6 cases. Overall, 4.13% of patients harboured transmitted resistance mutations to TDF/FTC and 2.48% to CAB (*p* > 0.05). Table 1 presents the prevalence of DRMs to drugs used in PrEP. Major resistance mutations were identified in 1.65%, 1.16% and 0.83% for TDF, FTC and CAB, respectively. Figure 1 shows the DRMs associated with PrEP resistance in the study population. Phenotypically relevant resistance (score > 30) was observed in 0.99% for TDF/FTC and 0.16% for CAB. No statistically significant differences between subtype A6 and B were observed (*p* > 0.05), except for the higher prevalence of L74I mutation in A6 (*p* < 0.05).

**Conclusions**: The frequency of HIV resistance mutations to PrEP agents remains low; however, the high prevalence of the L74I mutation in A6 highlights the need for continued molecular surveillance in regions with expanding A6 epidemics.

**FIGURE 1:**
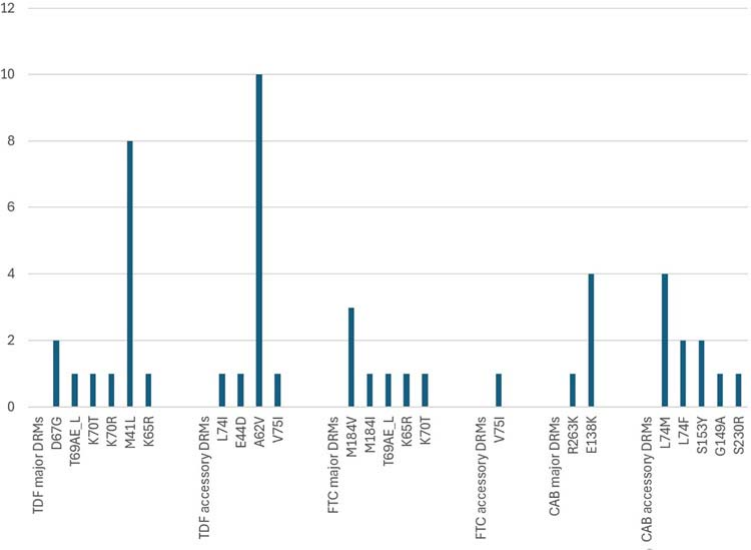
P024 | Detected DRMs to TDF, FTC and CAB. L74I excluded from analysis.

**TABLE 1:**
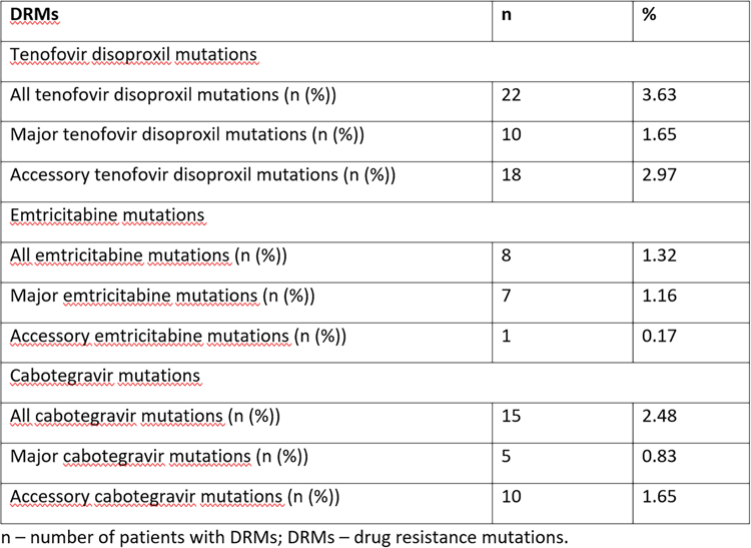
P024 | The number of patients with DRMs to drugs used in PrEP.

### P025 Adherence to European Standards of HIV Prevention and Care of Pre‐Exposure Prophylaxis (PrEP) in Italy: Results From the PrIDE Cohort

Giulia Micheli ^1^, Gianluca Frate^2^, Sara Leone^3^, Enrico Caruso^4^, Angelo Roberto Raccagni^5,6^, Marco Tutone^7^, Daniele Tesoro^8^, Vito Leonardo Del Negro^9^, Anna Lucchini^7^, Andrea Santoro^8,10^, Andrea Antinori^11^, Davide Moschese^12,13^, Silvia Nozza^5,6^, Massimo Cernuschi^4^, Valentina Mazzotta^1^

^1^Counseling, Test and HIV Prophylaxis and STI Unit, Regional AIDS Reference Center, National Institute for Infectious Diseases Lazzaro Spallanzani IRCCS, Rome, Italy; ^2^ICONA Foundation, Milan, Italy; ^3^Clinical Epidemiology and Biostatistics Unit, Department of Epidemiology, Preclinical Research and Advanced Diagnostics, National Institute for Infectious Diseases Lazzaro Spallanzani IRCCS, Rome, Italy; ^4^Milano Checkpoint ETS, Milan, Italy; ^5^Infectious Diseases Unit, Vita‐Salute San Raffaele University, Milan, Italy; ^6^Infectious Diseases Unit, IRCCS San Raffaele Scientific Institute, Milan, Italy; ^7^Multidisciplinary Center for Sexual Health (Ce.Mu.S.S.), Ospedale Oftalmico “Casimiro Sperino”, ASL Città di Torino, Turin, Italy; ^8^Department of Infectious Diseases, ASST Santi Paolo e Carlo, Milan, Italy; ^9^Checkpoint Plus Roma, Rome, Italy; ^10^Department of Health Sciences, Università degli Studi di Milano, Milan, Italy; ^11^Health Direction, National Institute for Infectious Diseases, Lazzaro Spallanzani IRCCS, Rome, Italy; ^12^Department of Clinical and Biomedical Sciences, Università degli Studi di Milano, Milan, Italy; ^13^Department of Infectious Diseases, Luigi Sacco University Hospital, Milan, Italy

**Background**: As European PrEP implementation still falls short of WHO regional goals [1], ECDC PrEP Standards of Care (SoC) have been developed to better monitor PrEP programmes in Europe and improve service access and quality [2]. The first audit was recently published with limited participation from very few clinics across Europe and discrepant results [3]. The aim of our study was to evaluate adherence to SoC indicators in the PrIDE network, a multicentre prospective cohort involving several hospital‐ and community‐based services in Italy.

**Methods**: We evaluated the performance of ECDC/EACS SoC indicators among new PrEP users (PrUs) enrolled in the PrIDE cohort by centre, focusing on PrEP initiation, the continuum of PrEP care, PrEP care delivery, integrated services and combination prevention. All centres with laboratory data available were included in the analysis (15/09/2024–31/12/2025). Performance was measured as the percentage of PrUs receiving SoC‐adherent care, stratified by centre and geographical area, as well as overall target achievement.

**Results**: Data from 4544 PrUs among 19 sites were analysed. Overall, the percentage of PrUs with reliable exclusion of HIV infection before PrEP initiation was 94.8%, with two centres below the 95% target. 92.1% of PrUs were tested for HIV at least every 6 months (target 80%). One HIV infection occurred over 2865 PYFU (incidence 0.03/100PYFU, 95% CI: 0–0.19); ART was initiated after resistance testing. At least annual STI testing was performed in 99.3% of PrUs (target 80%). Among PrUs with verified HBV status, 83.6% of HBsAb‐negative PrUs were addressed to vaccination (target 80%). Kidney function was monitored at least annually in 86.9% of PrUs aged >50 years with baseline eGFR < 90 mL/min/1.73 m^2^, with 3 centres below the 90% target. Adherence to SoC indicators by centre is detailed in Table 1. Selected SoC indicator performance for the network and anonymized centre benchmarking are shown in Figure 1.

**Conclusions**: The Italian PrIDE network demonstrates that high adherence to newly established European PrEP Standards of Care is achievable at national scale. Benchmarking identified HBV prevention and kidney monitoring as the principal remaining quality gaps, providing actionable targets for future quality improvement.

**References**

1. European Centre for Disease Prevention and Control. Pre‐exposure prophylaxis for HIV prevention in Europe and Central Asia. SE: European Centre for Disease Prevention and Control; 2025. https://doi.org/10.2900/2936869.

2. European Centre for Disease Prevention and Control. European standards of HIV prevention and care: module on pre exposure prophylaxis. LU: Publications Office; 2025. https://doi.org/10.2900/4677371.

3. European Centre for Disease Prevention and Control. Final report from the audit of the HIV pre‐exposure prophylaxis standards of care: September 2025. LU: Publications Office; 2025. https://doi.org/10.2900/4238499.

**FIGURE 1:**
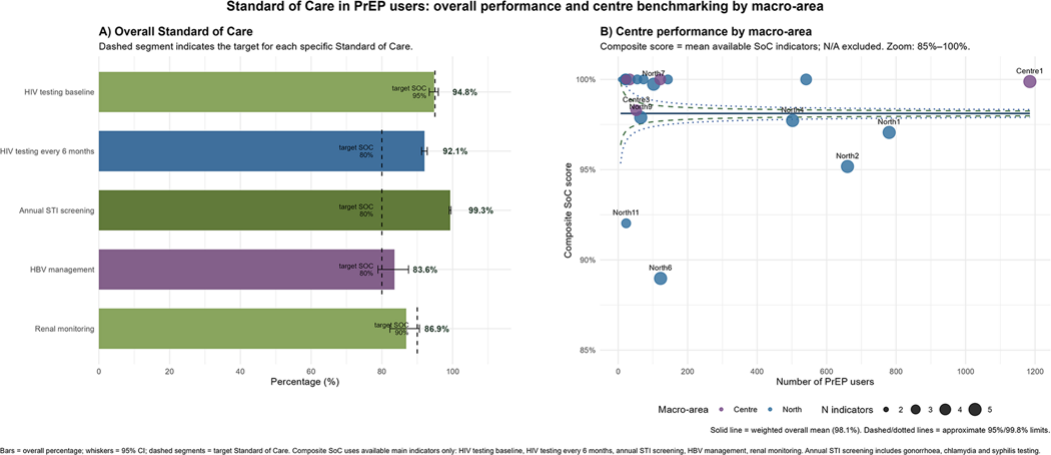
P025 | Standard of care selected indicators overall performance and anonymized centre benchmarking.

**TABLE 1:**
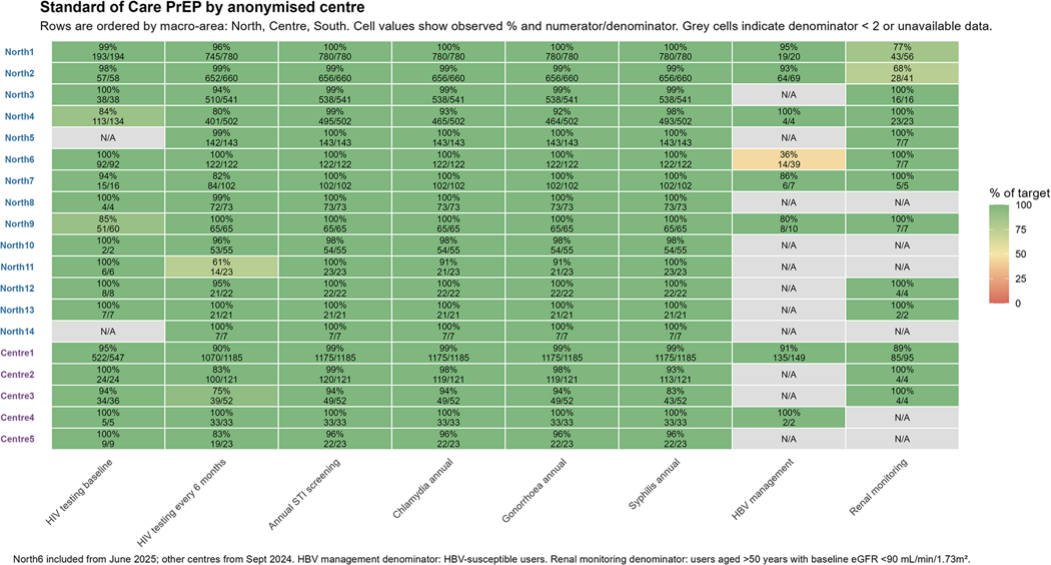
P025 | Standard of care selected indicators adherence by anonymized centre of PrIDE cohort.

### P026 Factors Associated With Hepatitis B (HBV) Status and HIV Pre‐Exposure Prophylaxis (PrEP) Use Among Gay, Bisexual and Other Men Who Have Sex With Men (GBM) in Vancouver, Toronto and Montreal, Canada

Mark Hull ^1,2^, Aki Gormezano^2,3^, Lu Wang^2^, Darrell Tan^4^, Nathan Lachowsky^2,3^, Daniel Grace^5^, Jason Trigg^2^, Justin Barath^2^, Alan Lal^2^, Joseph Cox^6^, David Moore^1,2^

^1^Department of Medicine, University of British Columbia, Vancouver, British Columbia, Canada; ^2^BC Centre for Excellence in HIV/AIDS, Vancouver, British Columbia, Canada; ^3^School of Health Sciences, University of Northern British Columbia, Vancouver, British Columbia, Canada; ^4^Division of Infectious Diseases, St. Micheal's Hospital, Toronto, Ontario, Canada; ^5^Dalla Lana School of Public Health, University of Toronto, Toronto, Ontario, Canada; ^6^Research Institute of the McGill University Health Centre, Montreal, Quebec, Canada

**Background**: HIV PrEP is standard of care among GBM at risk for HIV. Hepatitis B (HBV) status may impact prescribing of tenofovir‐based PrEP as additional monitoring of HBV parameters is needed and HBV may flare after discontinuation. HBV status impacts PrEP therapeutic options; understanding local HBV prevalence may guide clinical decision tools.

**Methods**: Sexually active GBM aged 16+ were recruited through respondent‐driven sampling from February 2017 to August 2019. Participants completed HBV serologic testing and a computer‐assisted self‐interview, which included questions about healthcare, substance use, mental health history and self‐reported HIV PrEP use. We measured the proportion of individuals with evidence of current and previous HBV (HBV positive surface antigen/HBV positive core antibody) and used multivariable generalized linear mixed modelling to examine sociodemographic, behavioural and clinical factors associated with HBV status and with PrEP use.

**Results**: Of 2405 GBM participants, a majority (95.2%) reported they were unaware of their HBV status, while 1.4% were HBV surface antigen positive and 10.9% had positive HBV core antibody. Those with chronic/prior HBV were more likely to be older ([age 30–44 years] aOR 2.88, 95% CI: 1.67–4.96; [> 44 years] aOR 12.44, 7.46–20.75), born in Asia (aOR 5.36, 2.66–10.82) or Africa (aOR 5.02, 2.29–11.02) and be living with HIV (aOR 2.78, 1.90–4.06). Among *n* = 1115 PrEP‐eligible GBM (per Canadian clinical guidelines), 4.4% (*n* = 14) of those reporting current PrEP use (27% of eligible GBM) had chronic/prior HBV. For PrEP‐eligible GBM, use of oral PrEP was associated with HIV risk indicators (HIRI‐MSM aOR 1.15, 1.12–1.19; prior bacterial STI 2.03, 1.38–2.98, substance use 1.67, 1.24–2.26), city of residence (Toronto aOR 1.85, 1.16–2.95; Vancouver aOR 2.36, 1.54–3.62), engagement with primary care (aOR 1.78, 1.20–2.63), education level (> high school aOR 2.01, 1.01–4.00) but not chronic/prior HBV status (OR 0.56, 0.22–1.44 in the univariate model).

**Conclusions**: Among GBM in urban Canada, HBV was uncommon and was associated with older age, coming from higher‐prevalence regions and HIV status. Use of PrEP among those with chronic/prior HBV was low, but HBV status did not appear to affect PrEP uptake. As non‐tenofovir PreP options increase, HBV status may limit uptake of long‐acting PreP agents.

### P027 Injectable Cabotegravir for HIV PrEP: Early Real‐World NHS Implementation in a North West England Sexual Health Service

Martyn Wood, Emily Clarke, Sarah Keegan, Mark Lawton

Axess Sexual Health, University Hospitals of Liverpool Group, Liverpool, UK

**Background**: Long‐acting injectable cabotegravir (CAB‐LA) provides an alternative HIV pre‐exposure prophylaxis (PrEP) option for individuals who cannot use or do not tolerate oral PrEP. CAB‐LA was commissioned in England in November 2025. Implementation represents a complex service delivery intervention requiring adaptation of clinic pathways, workforce skills and eligibility assessment. We report early real‐world service outcomes, focusing on uptake, tolerability and delivery challenges.

**Methods**: We undertook a retrospective descriptive service evaluation of all individuals initiating or continuing intramuscular CAB‐LA PrEP within a large northwest England sexual health service. Routinely collected clinical data were analysed, including demographics, prior oral PrEP use, indication for CAB‐LA, oral lead‐in use, cumulative injections, delayed dosing, discontinuation and reported adverse effects. Seven months of data since implementation were reviewed.

**Results**: Thirty‐five individuals received NHS commissioned CAB‑LA PrEP from first November 2025. Most were cisgender men (33/35, 94.3%), with lower representation from transgender individuals (2/35, 5.7%). Median age was 34 years (IQR 28–40). The majority were White British (26/35, 74.3%) and most identified as gay or bisexual (30/35, 85.7%). Most had previously used oral PrEP (30/35, 85.7%). The main indication for switching was gastrointestinal side effects or intolerance (19/35, 54.3%), followed by psychological barriers to tablet taking (6/35, 17.1%) and difficulty maintaining recommended dosing (4/35, 11.4%). Oral lead‐in was used in 3/35 (8.6%). Median injections received were 4 (IQR 3–4). Delayed dosing occurred in 3/35 (8.6%), with a median delay of 24 days (11–33 days) and discontinuation in 2/35 (5.7%). Adverse events were reported in 18/35 (51.4%), most commonly mild injection‐site pain (48.6%) and myalgia (17.1%); other side effects were infrequent.

**Conclusions**: In this early real‐world cohort, injectable cabotegravir PrEP was predominantly initiated by individuals with prior oral PrEP experience, most commonly due to intolerance or challenges with consistent use. These early data suggest CAB‐LA demand is likely to arise primarily from people already engaged with PrEP services, with implications for clinic capacity, injection delivery pathways and workforce planning. Early implementation suggests CAB‐LA may reinforce existing PrEP use rather than expand coverage, highlighting the need for pathway redesign and targeted strategies for equitable provision.

### P028 Real‐World Barriers to PrEP Access and HIV Knowledge Among First‐Time PrEP Users in Portugal: The POSITIVE CARE Study

Raquel Borges de Pinho^1^, Inês Maury^2^, Patrícia Pacheco^3^, Kamal Mansinho^4^, Ana Cláudia Miranda^4^, Cláudia Franco^5^, Cátia Caldas^6^, Fernando Maltez^7,8^, Maria José Manata^7^, Josefina Méndez^9^, Laura Paixão ^10^

^1^Internal Medicine Department, Hospital de Portimão, ULS Algarve, Portimão, Portugal; ^2^Infectious Diseases Department, Hospital Santa Maria, ULS Santa Maria, Lisboa, Portugal; ^3^Infecciology Department, Hospital Prof. Dr. Fernando da Fonseca, ULS Amadora Sinta, Amadora, Portugal; ^4^Infectious Diseases Department, Hospital Egas Moniz, ULS Lisboa Ocidental, Lisboa, Portugal; ^5^Infecciology Department Hospital Garcia de Orta, ULS Almada Seixal, Almada, Portugal; ^6^Infectious Diseases Department, Hospital de São João, ULS de São João, Porto, Portugal; ^7^Infectious Diseases Department, Hospital Curry Cabral, ULS São José, Lisboa, Portugal; ^8^Instituto de Saúde Ambiental da Faculdade de Medicina de Lisboa, Lisboa, Portugal; ^9^Infectious Diseases Department, Hospital de Santo António, ULS Santo António, Porto, Portugal; ^10^MSD Portugal, Paço de Arcos, Portugal

**Background**: Despite the efficacy of pre‐exposure prophylaxis (PrEP) in preventing HIV (human immunodeficiency virus) transmission, uptake remains suboptimal. The POSITIVE CARE study evaluated barriers to PrEP access and HIV knowledge in first time PrEP users in Portugal.

**Methods**: Observational, prospective study in 8 Portuguese hospitals including people without HIV initiating PrEP (12/2022–05/2024), with follow‐up until 07/2025. At enrolment, participants completed self‐administered questionnaires assessing barriers to PrEP access and HIV knowledge.

**Results**: The study included 202 individuals (cisgender men: 91.6%; gay/lesbian: 76.7%, aged 18–39: 79.2%; higher education: 61.5%). At PrEP initiation, 19.8% of individuals had at least one comorbidity, mainly neuropsychiatric (10.4%) and 23.3% had an STI. Most users accessed PrEP care following referral (67.8%), of whom 67.9% via a non‐governmental organization (NGO); among those accessing PrEP without a formal referral pathway, 83.1% learned about PrEP through friends. Only a minority of PrEP users considered embarrassment when attending a PrEP appointment (9.0%), stigma from friends/family (16.5%) or stigma from healthcare professionals (9.0%) as perceived barriers to PrEP access, whereas 38.7% of individuals identified low perceived HIV risk as barrier to uptake. Lack of knowledge regarding how to schedule a PrEP appointment and waiting times exceeding 1 month were considered logistical constraints to accessing PrEP care by 47.4% and 52.8% of individuals, respectively. HIV knowledge was good (score ≥10 points, max 18), with a median (P25; P75) score of 15.0 (13.0; 16.0). HIV knowledge differed significantly by age (*p* = 0.0396) and educational attainment (*p* = 0.0001), with higher scores in individuals aged 30–39 (16.0 [14.0; 17.0]) than 18–29 (14.0 [13.0; 16.0]) and among those with university (16.0 [15.0; 17.0]) compared with primary (12.5 [11.2; 15.2]) and secondary or professional education (14.0 [12.0; 15.0]).

**Conclusions**: PrEP uptake in Portugal is limited not by perceived stigma or HIV knowledge gaps, but by low‐risk perception and structural access constraints. Closing this gap requires shifting from awareness to action—enhancing risk perception among key groups, leveraging community pathways and simplifying access to care.

### P029 Abstract Withdrawn

### P030 Key Stakeholder Perspectives on the Barriers and Facilitators for HIV Prevention Services Among People Experiencing Homelessness

Erica Pool ^1^, Alison Rodger^1^, Shema Tariq^1^, Andrew Hayward^2^, Charles Witzel^1^

^1^Institute for Global Health, UCL, London, UK; ^2^UKHSA, London, UK

**Background**: In the UK, > 300,000 people are experiencing homelessness. One study reported an HIV prevalence in this group at 3.2%, 20 times higher than the general population prevalence (0.16%). Although effective HIV prevention methods are available, uptake of pre‐exposure prophylaxis (PrEP), post exposure prophylaxis (PEP), HIV testing, condoms and needle exchange remains suboptimal in this population. This qualitative study aims to explore stakeholders’ perspectives on barriers to and facilitators of HIV prevention among people experiencing homelessness.

**Methods**: Eight semi‐structured interviews were conducted with key stakeholders working in HIV prevention and/or homelessness sectors. Purposive sampling was used to ensure a range of occupations and roles. Interviews were analysed using a thematic framework approach, informed by the Høj integrated framework.

**Results**: Participant's job roles were clinicians (*n* = 4, primary care, inclusion health and HIV), a local authority commissioner (*n* = 1) and in the third sector (*n* = 3, HIV and homelessness sectors), based in England, Wales and Scotland. Participants described how high unmet need (housing, food, mental health and substance use‐related) constrained engagement with HIV prevention. HIV was not seen as a priority for most people experiencing homelessness, except in periods of heighted HIV awareness, for example an outbreak or diagnosis of a peer. Clinicians and commissioners may fail to identify this group as candidates for HIV prevention as they are often not perceived to fit traditional “key population” categories. Service‐level barriers included complicated care pathways, inaccessible locations and online booking systems that were challenging to navigate. At the individual level, lack of private domestic space, stigma and physical disability further limited access. Peer workers were seen as key facilitators, supporting engagement, fostering trust and showing solidarity. Despite questions related to all modes of HIV prevention, participants predominantly focused on oral and injectable PrEP. Many highlighted the potential for injectable PrEP to reduce barriers associated with oral PrEP, for example lack of privacy, storage and memory challenges limiting daily adherence (Table 1).

**Conclusions**: Injectable PrEP offers a potentially transformative opportunity for HIV prevention among people experiencing homelessness. Further work is needed to establish the preferences and priorities of this population to inform equitable and accessible HIV prevention care.

**TABLE 1:**
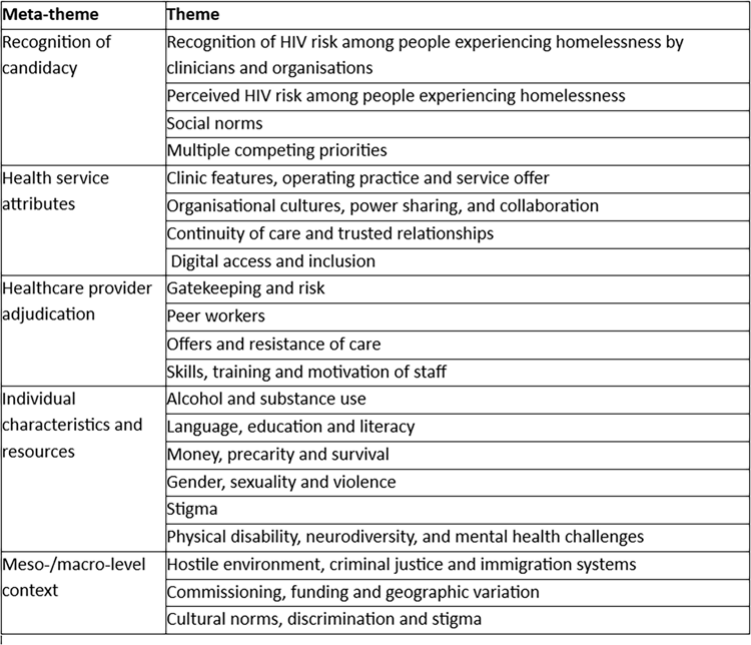
P030 | Summary of themes.

### P031 Abstract Withdrawn

### P032 Regional Reimbursement of STI Screening and PrEP Initiation in Italy: An Ecological Analysis From the PrIDE Cohort (Epi‐PrEP Study)

Sara Leone ^1^, Davide Moschese^2,3^, Gianluca Frate^4^, Enrico Caruso^5^, Angelo Roberto Raccagni^6,7^, Samuel Lazzarin^2^, Anna Lucchini^8^, Giulia C. Marchetti^9,10^, Annamaria Cattelan^11^, Salvio Cecere^12^, Vito Leonardo Del Negro^13^, Marianna Menozzi^14^, Roberto Caldera^15^, Gabriele Forcina^15^, Andrea Antinori^16^, Silvia Nozza^7,17^, Massimo Cernuschi^5^, Valentina Mazzotta^18^, on behalf of PrIDE/Epi‐PrEP Study Group

^1^Clinical Epidemiology and Biostatistics Unit, National Institute for Infectious Diseases Lazzaro Spallanzani IRCCS, Rome, Italy; ^2^Department of Infectious Diseases, Luigi Sacco University Hospital, ASST Fatebenefratelli Sacco, Milan, Italy; ^3^Department of Clinical and Biomedical Sciences—University of Milan, Milan, Italy; ^4^ICONA Foundation, Milan, Italy; ^5^Milano Checkpoint ETS, Milan, Italy; ^6^Infectious Diseases, Vita‐Salute San Raffaele University, Milan, Italy; ^7^Infectious Diseases Unit, IRCCS San Raffaele Scientific Institute, Milan, Italy; ^8^Multidisciplinary Center for Sexual Health (Ce.Mu.S.S.), Ospedale Oftalmico “Casimiro Sperino”, ASL “Città di Torino”, Turin, Italy; ^9^Clinic of Infectious Diseases, San Paolo Hospital, ASST Santi Paolo e Carlo, University of Milan, Milan, Italy; ^10^Department of Health Science, University of Milan, Milan, Italy; ^11^Infectious and Tropical Diseases Unit, Padua University Hospital, Padua, Italy; ^12^Bologna Checkpoint, Bologna, Italy; ^13^Checkpoint Plus Roma, Rome, Italy; ^14^Department of Infectious Diseases, Azienda Ospedaliero‐Universitaria of Modena, Modena, Italy; ^15^Gilead Sciences srl, Milan, Italy; ^16^Clinical and Research Infectious Diseases Department, National Institute for Infectious Diseases Lazzaro Spallanzani, IRCCS, Rome, Italy; ^17^Infectious Diseases Unit, Vita‐Salute San Raffaele University, Milan, Italy; ^18^Counseling, Test and HIV Prophylaxis and STI Unit, Regional AIDS Reference Center, National Institute for Infectious Diseases, Lazzaro Spallanzani IRCCS, Rome, Italy

**Background**: Financial barriers to sexually transmitted infection (STI) screening may limit access to HIV pre‐exposure prophylaxis (PrEP). Comprehensive STI screening is recommended before and during PrEP use, but its reimbursement varies across Italian regions and may influence access to PrEP within the National Health Service pathway. We evaluated whether regional STI screening reimbursement was associated with PrEP initiation using data from PrIDE, the largest multicentre PrEP cohort in Italy.

**Methods**: We conducted an ecological analysis of PrEP initiations recorded in the PrIDE cohort between September 2024 and December 2025. The outcome was the number of PrEP initiations aggregated by region, sex and age group. Regional populations aged 18–64 years were obtained from the Italian National Institute of Statistics. The population at risk was defined as the regional population minus the estimated number of people living with HIV, based on national surveillance data. Person‐years (PY) were calculated by weighting the population at risk according to the observation time of participating centres. Crude incidence rates (CIRs) were calculated overall and by STI screening reimbursement. Association between STI screening reimbursement and PrEP initiation was assessed using negative binomial regression, with PY as an offset. The model was adjusted for potential confounders, including sex, age group, regional HIV incidence and the density of LGBTQ+ associations.

**Results**: A total of 5357 PrEP initiations were recorded across eight Italian regions, five of which fully reimbursed STI screening. The overall CIR of PrEP initiation was 26.40 per 100,000 PY (Table 1). CIRs were 26.70 and 25.69 per 100,000 PY in regions with and without reimbursement, respectively. After adjustment, STI screening reimbursement was associated with more than two‐fold higher rate of PrEP initiation (aIRR 2.13, 95% CI: 1.40–3.25; *p* < 0.001) (Figure 1).

**Conclusions**: Regions providing reimbursed STI screening had higher rates of PrEP initiation than regions without reimbursement. While the ecological design does not allow causal inference and residual confounding by regional healthcare organization and prevention infrastructure cannot be excluded, the observed association suggests that financial barriers to STI testing and monitoring may influence access to PrEP. Further evaluation of policies providing free STI screening is warranted.

**Funding**: The Epi‐PrEP Study is sponsored and funded by Gilead Sciences.

**FIGURE 1:**
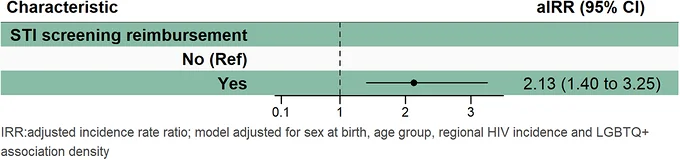
P032 | Reimbursement of STI screening and PrEP initiation: adjusted negative binomial regression.

**TABLE 1:**
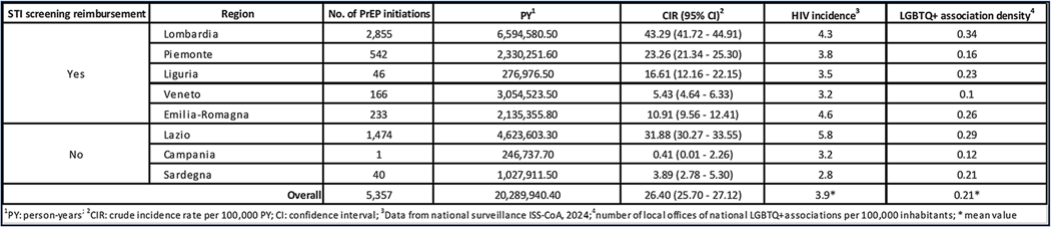
P032 | Characteristics of the Italian regions included in the study.

### P033 Factors Associated With Early Losses Along the PrEP Care Cascade Following Referral to a Hospital‐Based PrEP Unit

Lucio Jesus García Fraile Fraile ^1,2^, Inmaculada Jarrín Vera^2,^ ^3,^, Violeta Sampériz Rubio^4^, Ángela Gutiérrez Liarte^4^, Marta De Miguel Montero^5^, María López Cabanillas^6^, Ignacio De los Santos Gil^2,4^

^1^Infectious Diseases Department, Hospital Universitario de La Princesa, Madrid, Spain; ^2^CIBERINFEC, Instituto de Salud Carlos III, Madrid, Spain; ^3^Centro Nacional de Epidemiología, Instituto de Salud Carlos III, Madrid, Spain; ^4^Hospital Universitario de La Princesa, Madrid, Spain; ^5^Fundación SEIMC‐GESIDA, Madrid, Spain; ^6^HIV Medical Department Spain & Portugal, Gilead Sciences, Madrid, Spain

**Background**: HIV PrEP in our country has largely been delivered through a hospital‐centred model that may create barriers to access and engagement in care. We aimed to describe the referral, assessment and PrEP initiation process in a hospital‐based unit and to identify factors associated with losses before PrEP engagement.

**Methods**: Cross‐sectional study of individuals eligible for PrEP at a tertiary hospital in Madrid between April 2022 and April 2024. Early losses were classified as occurring during referral or between assessment and PrEP initiation. Factors associated with failure to initiate PrEP despite clinical indication were assessed using multivariable logistic regression.

**Results**: The study included 512 individuals eligible for PrEP. The PrEP care cascade is shown in Figure 1. Participants aged ≥30 years accounted for 74.6% of the cohort. 99.4% were male at birth, 98.8% identified as cis men and 97.7% as GBMSM. 60.5% were born in Spain and 30.5% in Latin America. Referrals originated from primary care in 55.9% of cases and from infectious diseases clinics in 38.5%. Primary care accounted for 55.9% of referrals and infectious diseases clinics for 38.5%. Overall, 8.0% of individuals were lost before PrEP initiation. During referral, 2.3% were lost before the first assessment visit. Those lost during referral were younger and more frequently referred through non‐standard pathways. Among attendees, 95.0% met criteria for PrEP initiation. Sexualized drug use was reported by 21.3%. Among individuals with a PrEP indication, 446 (93.9%) initiated PrEP and 29 (6.1%) did not. Failure to initiate PrEP was independently associated with age < 30 years (aOR 4.63; 95% CI: 2.12–10.13), LA origin (aOR 2.33; 95% CI: 1.02–5.31) and unawareness of HAV/HBV vaccination status (aOR 2.41; 95% CI: 1.02–5.72). Among individuals with a PrEP indication, 93.9% initiated PrEP and 6.1% did not. In multivariable analysis, age < 30 years (aOR 4.63; 95% CI: 2.12–10.13; *p* < 0.001), being born in Latin America (aOR 2.33; 95% CI: 1.02–5.31) and being unaware of HAV/HBV vaccination status (aOR 2.41; 95% CI: 1.02–5.72) were independently associated with failure to initiate PrEP.

**Conclusions**: Younger age was associated with a higher risk of early loss during the PrEP initiation process. Individuals of Latin America origin and those with limited knowledge of their own health history were more likely not to initiate PrEP despite clinical indication. Targeted interventions during referral and assessment could improve PrEP uptake in these groups.

**FIGURE 1:**
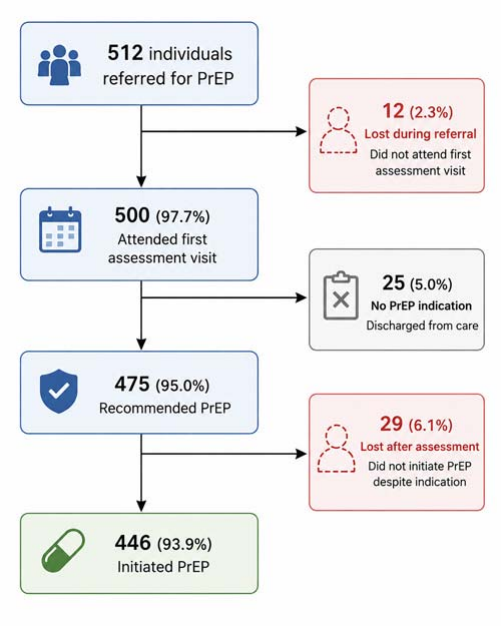
P033 | Flowchart of early losses before PrEP initiation.

### P034 Poke or Pill? Real‐World Adherence, Persistence and Discontinuation Data From the First Year of Introducing Injectable PrEP at a Toronto Community Sexual Health Clinic

Kevin Woodward ^1^, Tim Guimond^2^, Hommood Alrowais^3^, Victor Monroy^4^, Lucas Gergyek^5^

^1^Executive & Medical Director, HQ Health Hub, Toronto, Canada; ^2^Mental Health Director, HQ Health Hub, Toronto, Canada; ^3^Data Scientist & Privacy Officer, HQ Health Hub, Toronto, Canada; ^4^Research Analyst & Software Developer, HQ Health Hub, Toronto, Canada; ^5^Research Coordinator, HQ Health Hub, Toronto, Canada

**Background**: Long‐acting injectable cabotegravir (CAB‐LA) offers a highly effective and convenient alternative to daily oral pre‐exposure prophylaxis (PrEP). Barriers to access and uptake of oral PrEP are especially pronounced among marginalized communities, including Black, Indigenous and other People of Colour (IBPOC) [1]. Understanding who chooses injectable versus oral PrEP is essential for minimizing barriers to CAB‐LA while enhancing PrEP access, uptake and equity.

**Methods**: We conducted a retrospective chart review of 2293 individuals who initiated oral PrEP and 332 who initiated or switched to injectable PrEP at HQ, a community sexual health clinic in Toronto, Ontario between 31 March 2025 and 1 June 2026 to assess factors associated with injectable versus oral PrEP. Injectable versus oral PrEP was the binary outcome and predictors included sexual orientation, gender identity, ethnoracial identity, sexual practices, housing, immigration status, insurance coverage, STBBI history, substance use and mental health support needs. Multivariate logistic regression was applied to identify demographic and behavioural predictors.

**Results**: Overall, injectable PrEP uptake was 12.6%. In the fully adjusted model, Black (aOR 2.22, 95% CI: 1.31–3.76), Indigenous (aOR 5.02, 95% CI: 1.71–14.76) and Latinx (aOR 2.00, 95% CI: 1.20–3.34) people were significantly more likely to choose injectable PrEP than white people; Black and Indigenous associations survived FDR correction (*q* = 0.039). Insurance coverage was not an independent predictor and ethnicity effects were consistent across coverage types (interaction *p* = 0.40). Exploratory associations included syphilis history (aOR 1.59, 95% CI: 1.11–2.28) and mental health support requests (aOR 1.49, 95% CI: 1.09–2.03).

**Conclusions**: In our sample, ethnicity independently predicted injectable PrEP modality choice regardless of insurance coverage. IBPOC people were significantly more likely to choose injectable PrEP across both coverage types, suggesting greater acceptability of injectable PrEP among these communities. Tailored interventions that continue to encourage injectable PrEP uptake among IBPOC communities are paramount for continued engagement in care. Raising awareness and promoting access are important programme targets moving forward to encourage injectable PrEP uptake for those currently underreached.

**Reference**

1. Ajiboye W, Tharao W, Owino M, Soje L, Tian JMLH, Ly A, Piyesis MK, McLeod A, Fleury M, McBain K, Massaquoi N, Mosugu T, Swidrovich J, Tan DHS, Nelson L, Rourke SB. Racial disparities in HIV pre‐exposure prophylaxis (PrEP) awareness and uptake among white, Black and Indigenous men in Canada: analysis of data from the I'm Ready national HIV self‐testing study. Can J Public Health. 2025 Apr;116(2):243‐253. doi: 10.17269/s41997‐025‐01009‐5. Epub 2025 Mar 19. PMID: 40106208; PMCID: PMC12075050.

### P035 Beyond the Pill: First‐Year Real‐World Outcomes of Injectable PrEP at a Toronto Community Sexual Health Clinic

Kevin Woodward ^1^, Tim Guimond^2^, Hommood Alrowais^3^, Victor Monroy^4^, Lucas Gergyek^5^

^1^Executive & Medical Director, HQ Health Hub, Toronto, Canada; ^2^Mental Health Director, HQ Health Hub, Toronto, Canada; ^3^Data Scientist & Privacy Officer, HQ Health Hub, Toronto, Canada; ^4^Research Analyst & Software Developer, HQ Health Hub, Toronto, Canada; ^5^Research Coordinator, HQ Health Hub, Toronto, Canada

**Background**: Long‐acting injectable cabotegravir (CAB‐LA) is the first injectable HIV pre‐exposure prophylaxis (PrEP). Real‐world data on its adherence and persistence and how these compare with oral PrEP remain limited, making it challenging to determine where opportunities for programme refinement exist.

**Methods**: We conducted a retrospective chart review of 321 individuals who initiated injectable PrEP and 2445 who initiated oral PrEP at HQ, a community sexual health clinic in Toronto, Ontario during the period of 31 March 2025 and 22 May 2026. For injectable PrEP, adherence was defined as receiving all injections within ± 7 days of the expected date. For oral PrEP, adherence was defined as obtaining all PrEP refills within a ± 14‐day window of the expected date. For both injectable and oral, persistence was defined as continuous engagement in care without a ≥6‐month lapse or documented discontinuation. Predictors of persistence and adherence were examined by univariate and forward‐AIC multivariable logistic regression per cohort and a pooled model with a modality indicator (adjusted for age, gender, orientation, sexual practices) was conducted.

**Results**: For injectable PrEP, 321 individuals received 1183 injections; 87.8% were on‐time and 70.0% had every injection on time. Kaplan–Meier persistence (Figure 1) was 84.2% (95% CI: 79.3–89.4) at 6 months and 76.2% (68.0–85.5) at 12 months. 12.5% discontinued (13 switched to oral PrEP and 27 stopped before 6 months). During the same period, 2445 individuals initiated oral PrEP. Injectable persistence was higher (84.2%/76.2% vs. 73.0%/65.4% at 6/12 months; log‐rank *p* = 0.015). On native adherence metrics, 70.0% of individuals on injectable PrEP had every injection on time (± 7 days) versus oral PDC ≥0.8 in 65.0% (all refills within ± 14 days in 46.3%). In a pooled model, injectable (vs. oral) independently predicted persistence (adjusted OR 5.21, 95% CI: 3.72–7.48; *p* < 0.001). Demographic predictors were weak for oral outcomes (AUC ≈ 0.53–0.59) and modest for injectable (persistence AUC 0.79; adherence 0.70), with younger age and substance use associated with lower persistence.

**Conclusions**: Injectable CAB‐LA achieved high on‐time adherence and substantially better persistence than oral PrEP, while in‐window adherence was comparable. Observed cases of injectable discontinuations were minimal and often involved transitions to oral PrEP rather than cessation. Our findings suggest that injectable PrEP may improve sustained protection and retention over time.

**FIGURE 1:**
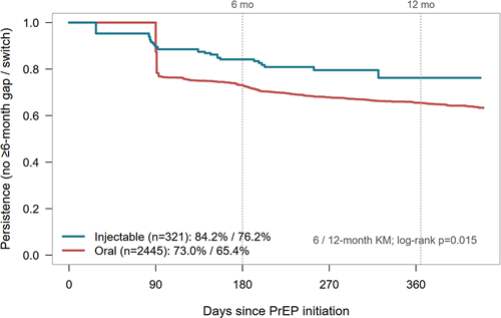
P035 | Persistence on PrEP: injectable versus oral cohort.

### P036 Barriers to PrEP Access Among Underserved Groups in the UK: A Co‐Produced Critical Interpretive Synthesis

Elizabeth Eithne McKeand^1^, Sara Paparini^1^, Vian Rajabzadeh^1^, Sophie Strachan^1,2^, Melanie Smuk^1^, Laura Leigh Vetters^1^, Nishat Halim^1^, Caro Camargo^1,2^, Chloé Pasin^1^, Anqa Awan^2^, Taranjit Chana^3^, Kelsea Delatango^4^, Vimbai Mandaza^1^, Pamela Menzies‐Banton^3^, Zoch Nwaosu^3^, Juddy Otti^2,5^, Kyle Ring^1^, Chloe Orkin^1^, Megan Devonald ^1^, PrEP‐Position Steering Group Member^3^, on behalf of PrEP‐Position Steering Group

^1^Queen Mary University of London, London, UK; ^2^Sophia Forum, London, UK; ^3^The PrEP‐Position Steering Group, London, UK; ^4^Bare Radicals, London, UK; ^5^Africa Advocacy Foundation, London, UK

**Background**: Inequalities in access to and engagement with pre‐exposure prophylaxis (PrEP) persist among underserved populations. This review analyses the evidence‐base on highly underserved populations in PrEP care in the UK (women, racially minoritized communities, recent migrants, people who use drugs, unhoused people, transgender or non‐binary individuals and sex workers) and offers new insights to support more inclusive research and PrEP equity efforts.

**Methods**: A co‐produced, critical interpretive synthesis (CIS) approach was used to support the generation of higher‐order new interpretations from existing secondary data. Electronic databases (Web of Science, Scopus, Medline, Google Scholar) and informal methods (reference chaining, including grey literature) were used for the initial search (November 2024–January 2025). Evidence quality was assessed using the Dixon−Woods checklist. The evidence synthesis was presented to a Steering Group of 12 members representing various communities underserved by sexual health to enhance interpretation of the findings and update search terms. An additional search was conducted up to December 2025. Papers were included if they focused: on PrEP care in the UK; one or more underserved group; studies conducted after 2010. 23 papers were included.

**Results**: Barriers to PrEP access for underserved groups were identified at the individual (low perceived PrEP need, limited PrEP knowledge and competing life priorities), community (stigma and social norms) and structural (institutional discrimination, practical barriers such as long waiting lists) level (Figure 1). Existing literature often frames experiences through fixed and singular identity categories, overlooking the dynamic and intersecting factors shaping PrEP engagement. The community input and CIS highlighted underexplored interrelated concepts: intersectional identities, the influence of place and context and the changing nature of PrEP needs over time. A lack of disaggregated data on recent migrants, unhoused people, sex workers, non‐binary people and those in rural areas also hampers current understandings (Table 1). These will be needed for any impactful analysis of PrEP need for underserved group to obtain a complete picture for developing equitable services.

**Conclusions**: Time and place‐specific contexts are crucial for understanding how intersecting identities and practices shape PrEP experiences for different groups. More disaggregated, intersectional and longitudinal research is urgently needed to address gaps in the evidence‐base among underserved groups.

**References**

1. Coukan F, Thamm W, Afolabi F, Murray KK, Rathbone AP, Saunders J, et al. Co‐designing interventions with multiple stakeholders to address barriers and promote equitable access to HIV pre‐exposure prophylaxis (PrEP) in Black women in England. BMC Public Health. 2025;25(1):1831.

2. Nakasone SE, Young I, Estcourt CS, Calliste J, Flowers P, Ridgway J, et al. Risk perception, safer sex practices and PrEP enthusiasm: barriers and facilitators to oral HIV pre‐exposure prophylaxis in Black African and Black Caribbean women in the UK. Sex Transm Infect. 2020;96(5):349‐54.

3. Witzel TC, Nutland W, Bourne A. What are the motivations and barriers to pre‐exposure prophylaxis (PrEP) use among black men who have sex with men aged 18−45 in London? Results from a qualitative study. Sex Transm Infect. 2019;95(4):262‐6.

4. Witzel TC, Nutland W, Bourne A. What qualities in a potential HIV pre‐exposure prophylaxis service are valued by black men who have sex with men in London? A qualitative acceptability study. Int J STD AIDS. 2018;29(8):760‐5.

5. Maund O MR and Wain, O “There needs to be care throughout” Exploring the access of non‐binary people, trans men and trans women to sexual health services in Scotland. Edinburgh: Waverley Care and Scottish Trans Alliance; 2020.

6. Pyper T WA and Pyper C. Towards Equity: Reimagining HIV Prevention for London's Black Communities. London: Do it London; 2025.

7. Smith M, Elliott L, Hutchinson SJ, Metcalfe R, Flowers P, McAuley A. Perspectives on pre‐exposure prophylaxis for people who inject drugs in the context of an HIV outbreak: a qualitative study. Int J Drug Policy. 2021;88:103033.

8. Di Giuseppe K, Kasoka K, Dunkley Y. Investigating pre‐exposure prophylaxis (PrEP) uptake among Black African communities in East London and Hertfordshire: a brief report of a mixed methods study. International Journal of Sexual Health. 2019;31(3):291‐6.

9. Jaspal R, Daramilas C. Perceptions of pre‐exposure prophylaxis (PrEP) among HIV‐negative and HIV‐positive men who have sex with men (MSM). Cogent Medicine. 2016;3(1):1256850.

10. Grimshaw C, Boyd L, Smith M, Estcourt CS, Metcalfe R. Evaluation of an inner city HIV pre‐exposure prophylaxis service tailored to the needs of people who inject drugs. HIV Med. 2021;22(10):965‐70.

11. Coukan F, Sullivan A, Mitchell H, Jaffer S, Williams A, Saunders J, et al. Impact of national commissioning of pre‐exposure prophylaxis (PrEP) on equity of access in England: a PrEP‐to‐need ratio investigation. Sexually Transmitted Infections. 2024;100(3):166.

12. Maxwell S, Shahmanesh M, Gafos M. Pre‐exposure prophylaxis (PrEP) uptake and adherence experiences of gay and bisexual men who engage in chemsex: a qualitative study. Int J Drug Policy. 2022;103:103630.

13. Rathbone A, Cartwright N, Cummings L, Noble R, Budaiova K, Ashton M, et al. Exploring young people's attitudes to HIV prevention medication (PrEP) in England: a qualitative study. BMJ Open. 2024;14(3):e077733.

14. Wolton A J CR, Ross‐Turner M, Suchak T. Trans:Mission A community‐led HIV testing initiative for transpeople and their partners at a London sex‐on‐premises venue. HIV Nursing. 2018;18(2):24–9.

15. Young I, Flowers P, McDaid LM. Barriers to uptake and use of pre‐exposure prophylaxis (PrEP) among communities most affected by HIV in the UK: findings from a qualitative study in Scotland. BMJ Open. 2014;4(11):e005717.

16. Weil BN, W. Understanding the PrEP and sexual health needs and experiences of young (18−25) GBMSM in London. Qualitative research report. London: The Love Tank; 2024.

**FIGURE 1:**
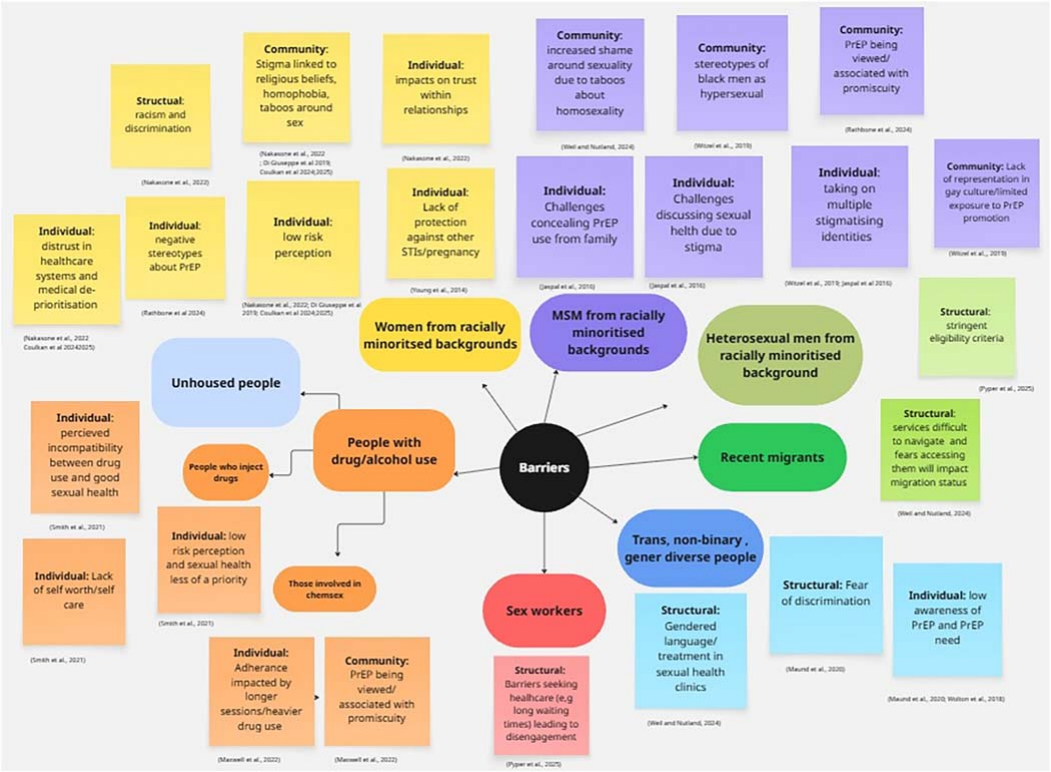
P036 | Overview of PrEP barriers for underserved groups in the UK [1−9, 11−16].

**TABLE 1:**
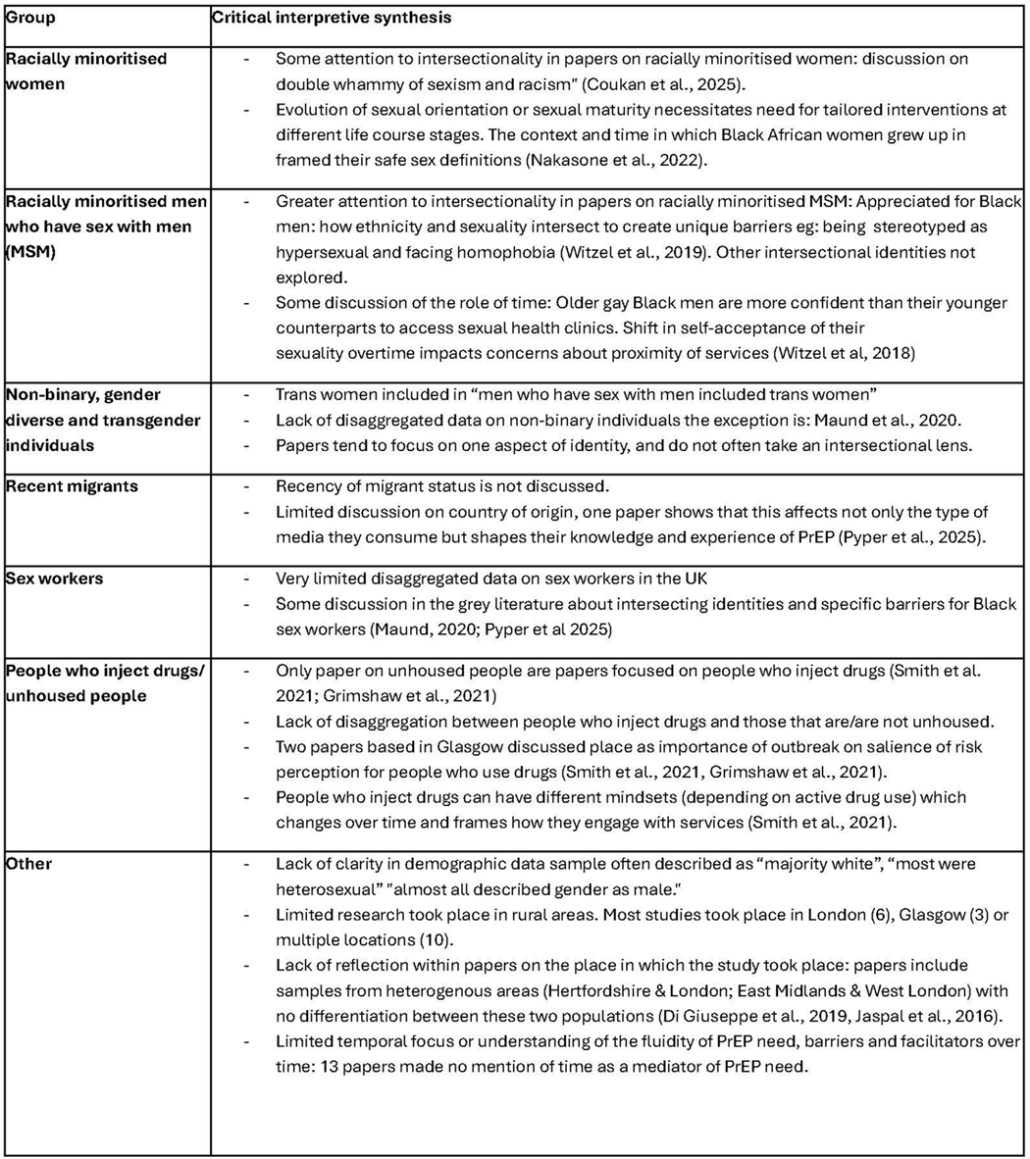
P036 | Overview of critical interpretive synthesis [1−10].

### P037 Pre‐Exposure Prophylaxis in Men Who Have Sex With Men and New HIV Infections in Croatia

Šime Zekan ^1,2^, Vanja Romih Pintar^1^, Marija Santini^1,2^, Josip Begovac^1^

^1^University Hospital for Infectious Diseases, Zagreb, Croatia; ^2^School of Medicine, University of Zagreb, Zagreb, Croatia

**Background**: A fully reimbursed, centralized pre‐exposure prophylaxis (PrEP) service for men who have sex with men (MSM) started in Zagreb, Croatia, in September 2018. MSM have remained the population at highest risk and account for most new HIV infections in Croatia. Between 2015 and 2022, we observed a decline in new HIV infections overall and among MSM, with a more pronounced decrease from 2020 onwards. It was unclear whether this reflected disruption of services and reduced HIV testing during and after the COVID‐19 pandemic or increased uptake of PrEP.

**Methods**: We retrospectively collected data on new HIV infections from the centralized databases of the single Croatian HIV centre and data on PrEP users from the largest PrEP service in Croatia (Zagreb). We analysed data from the HIV centre from January 2015 to December 2025 (11 years in total) and data from the PrEP service from September 2018 to December 2025 (7 years and 4 months in total).

**Results**: New HIV infections among MSM entering HIV care were 57% lower in 2025 than in 2015 and 46% lower for all new HIV infections. The number of PrEP users attending at least once per year increased several‐fold between 2018 and 2025. In the last 3 years (2023–2025), the number of new HIV infections among MSM was strikingly similar (Figure 1). We assessed prevention exposure among MSM presenting with new HIV infection in 2025. Most (> 70%) had tested very rarely or never previously and only 2 have ever used PrEP (Table 1).

**Conclusions**: The reasons for the decline in new HIV infections among MSM entering single‐centre HIV care in Zagreb, Croatia—where same‐day ART initiation is practiced—are still likely to be multifactorial. However, we believe that PrEP significantly contributed to this decline, consistent with findings from other urban settings worldwide where HIV epidemics are concentrated among MSM. Our findings provide proof of concept for PrEP in a more stigmatized Central and Eastern European context. Further efforts to decentralize and promote PrEP services and HIV testing could help reach MSM who are still not using prevention tools, thereby reducing new HIV infections in Croatia.

**FIGURE 1:**
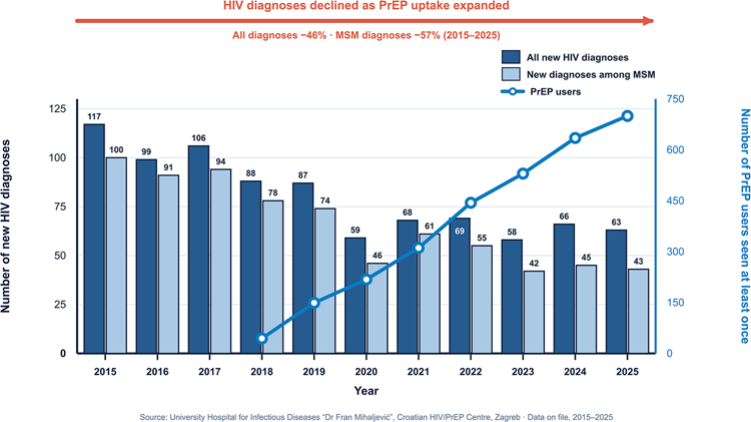
P037 | New HIV diagnoses (2015–2025) and PrEP uptake (2018–2025) in Zagreb, Croatia.

**TABLE 1:**
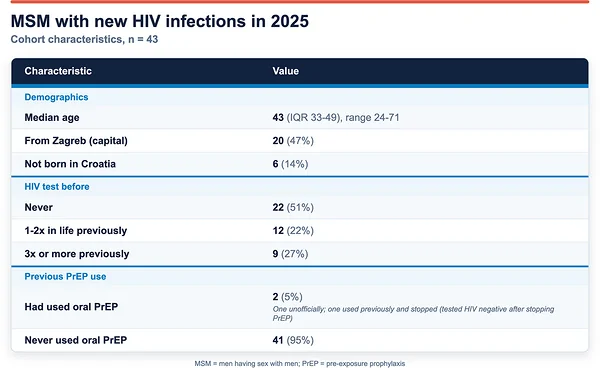
P037 | MSM diagnosed with new HIV infections in a single HIV centre in Zagreb, Croatia, in 2025.

## Treatment Strategies: Novel Therapeutic Targets (Phase I and II)

### P038 Once‐Weekly Ulonivirine Provides a Week of Forgiveness After a Missed Dose

Michelle M. Pham ^1^, Jingcheng Xiao^1^, Shuang Xu^2^, Uche Okoro^1^, Yayun Xu^1^, Sandy Rawlins^1^, Mengchun Li^1^, Bill Poland^2^, Vincent Dubois^1^, Ryan C.Vargo^1^, Randolph P. Matthews^1^

^1^Merck & Co., Inc., Rahway, NJ, US; ^2^Certara, Radnor, PA, US

**Background**: Ulonivirine (ULO) is a non‐nucleoside reverse transcriptase inhibitor (NNRTI) being investigated at a once‐weekly (qw) dose of 200 mg in combination with islatravir 2 mg in a phase 2b study in virologically suppressed adults living with HIV‐1 (MK‐8591B‐060 [P060]). Data from P060 were used to update a population pharmacokinetic (popPK) model describing the pharmacokinetics (PK) of ULO. This analysis, together with adherence data, demonstrated ULO forgiveness and supported missed‐dose recommendations.

**Methods**: Plasma ULO samples were analysed from adults with and without HIV‐1 across six phase 1 and two phase 2 studies, including P060, through week 24. A popPK model was developed and adherence data from P060 were used to assess and quantify the degree of forgiveness following missed ULO doses.

**Results**: This analysis included 4428 ULO PK samples from 354 participants. The PK of ULO was best characterized using a 2‐compartment model with first‐order absorption and elimination. Apparent clearance was higher in participants assigned male at birth and increased with increasing body weight and estimated glomerular filtration rate, as estimated by the Chronic Kidney Disease Epidemiology Collaboration 2021 equation; apparent central and peripheral volumes also showed a positive association with body weight. While most of the participants demonstrated weekly adherence (1642/1808 doses taken every 7 days; 91%), 5% of doses were taken at intervals of 8 days or more. Notably, all participants in P060 maintained virological suppression. Simulations using individual empirical Bayes estimates of PK from P060 demonstrated that 90% of participants maintained concentrations above the wild‐type PK threshold (70 nM) 1 week after a missed dose (Table 1). Additional simulations conducted with typical‐value participants (*n* = 1000) receiving ULO 200 mg qw and incorporating between‐subject variability demonstrated adequate ULO concentrations and rapid return to efficacious exposures among participants whose concentrations dropped below the threshold in scenarios of missed doses (Figure 1).

**Conclusions**: The popPK model adequately described ULO PK. Weekly ULO dosing maintained efficacious concentrations for virologically suppressed adults living with HIV‐1 for up to 1 week after a missed dose, providing a week of forgiveness.

**FIGURE 1:**
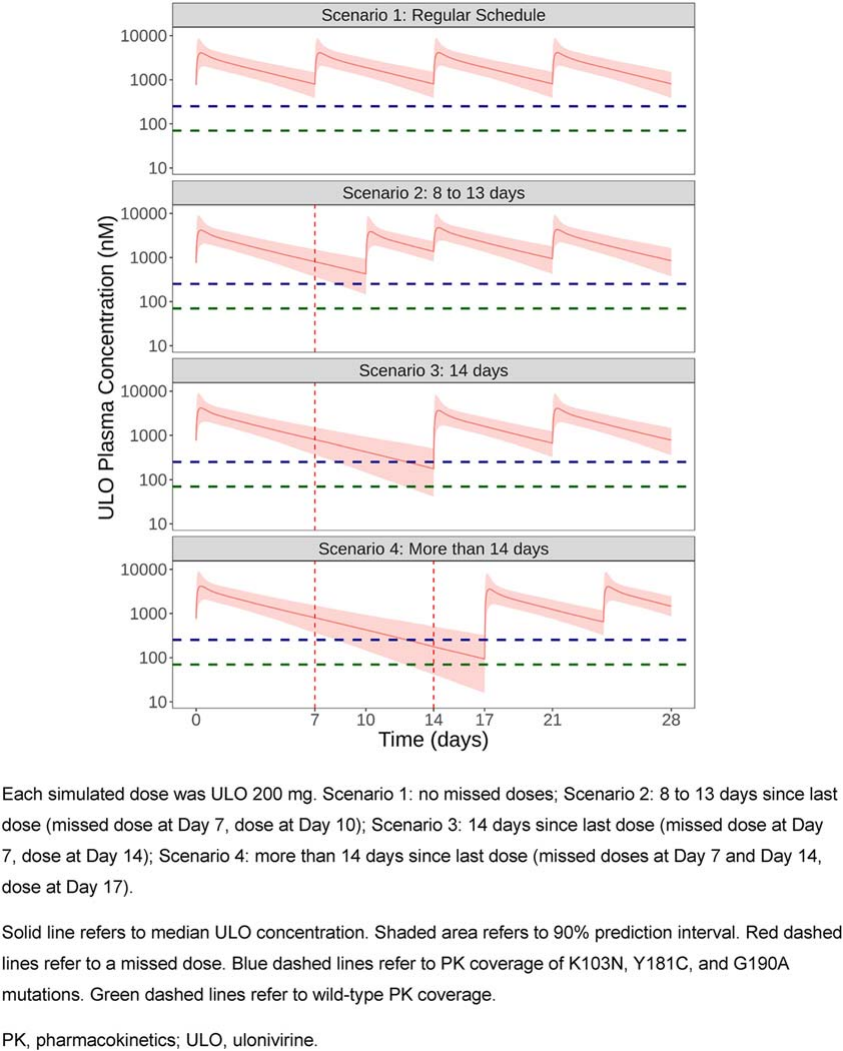
P038 | Simulated ULO concentration‐time profiles following ULO 200 mg for missed‐dose scenarios.

**TABLE 1:**
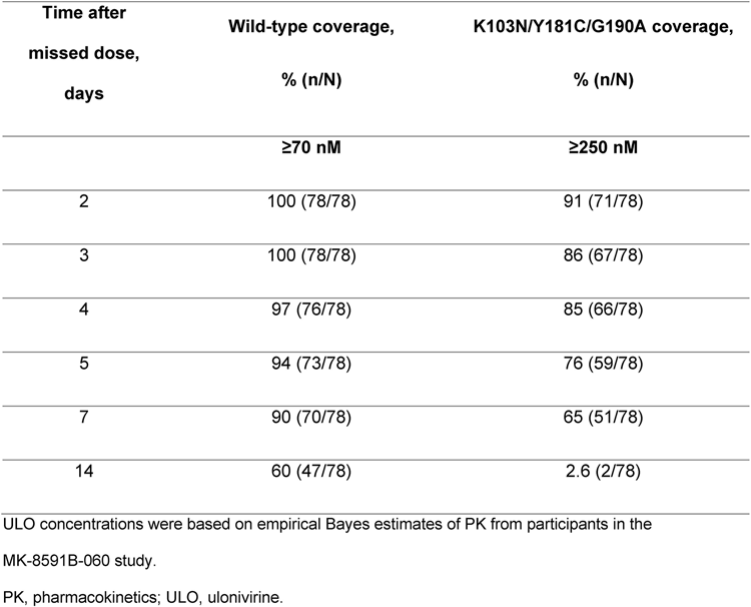
P038 | Proportion of participants maintaining wild‐type and mutant coverage after a missed dose of ULO.

### P039 Phase 1 Results for the Selection of a New Long‐Acting Rilpivirine Formulation to Support Dosing Three Times a Year

Eileen Castronova ^1^, Ronald D'Amico^2^, Jörg Sievers^3^, Randa Hareedy^4^, Gilda Bontempo^5^, Lisa Morgan^1^, Shawn Searle^6^, Katherine Sides^7^, David Dorey^8^, Herta Crauwels^9^, Kati Vandermeulen^9^, Lorant Leopold^10^, Eileen Birmingham^11^, Rodica van Solingen‐Ristea^9^, Lionel Tan^3^

^1^GSK, Collegeville, PA, US; ^2^ViiV Healthcare, Durham, NC, US; ^3^ViiV Healthcare, London, UK; ^4^GSK, London, UK; ^5^ViiV Healthcare, Branford, CT, US; ^6^Trialmed, Las Vegas, NV, US; ^7^Trialmed, Austin, TX, US; ^8^GSK, Mississauga, Canada; ^9^Johnson & Johnson, Beerse, Belgium; ^10^Johnson & Johnson, Titusville, NJ, US; ^11^Johnson & Johnson, Raritan, NJ, US

**Background**: Long‐acting (LA) cabotegravir (CAB) co‐administered with LA rilpivirine (RPV) provides a complete LA HIV‐1 treatment regimen administered monthly or every 2 months. New CAB and RPV formulations have been developed to explore extending the dosing interval. We previously reported safety and pharmacokinetics (PK) of a CAB formulation that supports dosing every 4 months (3 times a year). Here, we present the safety, PK, modelling and simulation of new RPV formulations that support every‐4‐month dosing.

**Methods**: Adults without HIV received single or repeat intramuscular injections of 2 RPV formulations in 7 cohorts in this ongoing phase 1 study (NCT05418868). Participants received Formulation 1 as single injections at 2 dose levels (12 per cohort) or repeat injections at 2 dose levels (6 per cohort) or Formulation 2 as single injections at 2 dose levels (12 per cohort). Results of this phase 1 study and an RPV population PK (PopPK) model were leveraged to simulate RPV PK profiles to support 3‐times‐a‐year dose selection for a phase 3 study.

**Results**: To date, 72 participants received 84 RPV injections. Maximum RPV concentration was typically reached within 6 weeks post‐dose (Table 1). Injections were generally well tolerated; optimization of needle length in later cohorts led to fewer and lower‐grade injection site reactions (Table 2). Systemic safety outcomes were consistent with the established RPV LA monthly and every‐2‐month safety profile. Observed concentrations are supportive of every‐4‐month dosing.

**Conclusions**: Safety and PK results obtained in this phase 1 study support further testing of RPV in combination with CAB as part of a complete HIV‐1 treatment regimen dosed 3 times a year. This regimen will be assessed in a phase 3 study evaluating the efficacy, safety and tolerability of CAB+RPV dosed 3 times a year in people with HIV‐1.

**TABLE 1:**
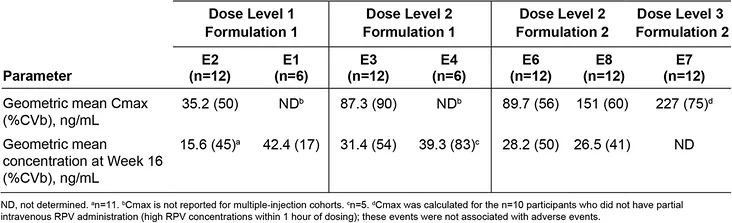
P039 | Pharmacokinetics of RPV formulations.

**TABLE 2:**
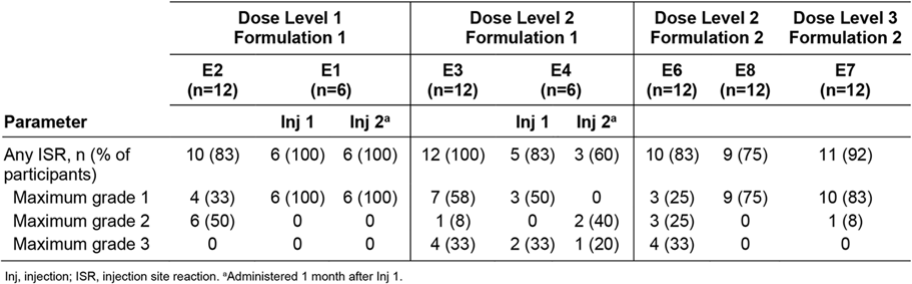
P039 | ISR summary of RPV formulations.

### P040 Single‐Dose Escalation Study of TMB‐365 in People Living With HIV: Safety, Pharmacokinetics and Antiviral Activity

Edwin DeJesus^1^, Jacob P. Lalezari^2^, Moti Ramgopal^3^, Javier Morales‐Ramirez^4^, Leandro Mena^5^, Kuei‐Ling Kuo^6^, Ying‐An Lai^6^, Martin Markowitz ^7^

^1^Orlando Immunology Center, Orlando, FL, US; ^2^Quest Clinical Research, San Francisco, CA, US; ^3^Midway Special Care, Fort Pierce, FL, US; ^4^Clinical Research PR, San Juan, Puerto Rico, US; ^5^Emory University, Atlanta, GA, US; ^6^TaiMed Biologics Inc., Taipei City, Taiwan; ^7^TaiMed Biologics Inc., Irvine, CA, US

**Background**: TMB‐365 is a second‐generation post‐attachment inhibitor based on ibalizumab that binds similarly to domain 2 of CD4. It incorporates Fc receptor (FcRn) mutations to reduce effector function, histidine substitutions to enhance PK and glycan modifications to improve breadth. Here, we present safety, PK and antiviral activity results of single‐dose intravenous (IV) TMB‐365 in people living with HIV (PLWH); NCT04027387.

**Methods**: In this phase 1, randomized, placebo‐controlled, sequential single dose escalation study, three groups (*n* = 8–9 each) received TMB‐365 at 400, 800 and 1600 mg IV or placebo (6:2 ratio) and followed for 10 weeks. Study drug was diluted in an IV bag and infused over 60 min with participants observed for ≥60 min after. Dose escalation was sequential and approved by an independent data monitoring committee. Participants in the 400 and 800 mg cohorts were viraemic, antiretroviral therapy (ART)‐interrupted PLWH (off ART ≥4 weeks; HIV‐1 RNA 1000–100,000 copies/mL). The 1600 mg cohort included virologically suppressed PLWH on stable combination antiretroviral therapy (cART). Participants in the lower‐dose cohorts were encouraged to reinitiate ART ≥6 weeks post‐dose; those in the 1600 mg cohort continued cART. Safety, PK and virologic endpoints were assessed through week 10.

**Results**: Twenty‐five participants were treated with study drug or placebo. No serious adverse events, grade 3/4 treatment emergent adverse events (TEAEs) or acute infusion events were observed. All TEAEs (*N* = 23) were mild to moderate. Six were attributed to TMB‐365 in 3 participants; 1 in each cohort, including headache, pyrexia, oropharyngeal pain, periorbital oedema, wheezing and urticaria. No anti‐drug antibodies were observed. TMB‐365 exhibited capacity‐limited elimination PK. Apparent half‐life was ∼6–11 days during the CD4‐saturated phase and ∼2–4 days during the terminal unsaturated phase. PK simulations supported dosing intervals of up to 12 weeks with higher doses. In viraemic participants, single doses of 400 and 800 mg produced mean HIV‐1 RNA reductions of ∼1.6 log₁₀ copies/mL by day 14 (Figure 1). Loss of antiviral activity correlated with reduction in TMB‐365 levels.

**Conclusions**: Single‐dose TMB‐365 up to 1600 mg was well tolerated and demonstrated robust antiviral activity. Its PK profile supports the feasibility of extended dosing intervals, including every 8–12 weeks.

**FIGURE 1:**
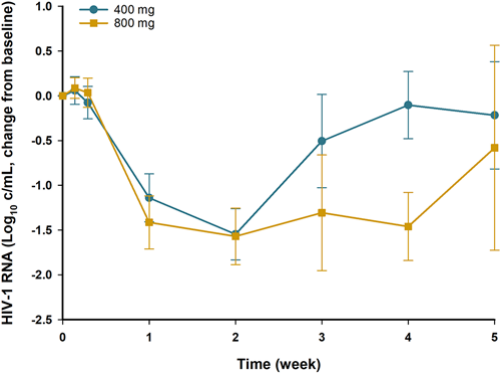
P040 | Mean change in HIV‐1 RNA from baseline.

### P041 Safety, Tolerability and Acceptability of HIV‐1 Capsid Inhibitor VH4011499 Injectable Formulation

Bronagh Shepherd^1^, Veronica Bainbridge^1^, Jose R. Castillo‐Mancilla ^2^, Nilay Thakkar^3^, Gilda Bontempo^4^, Adelaide Jewell^5^, Jia Chang^6^, Shawn Searle^7^, Noopur Singh^3^, Lisa Morgan^8^, Babafemi Taiwo^2^, Paul Benn^5^

^1^GSK, London, UK; ^2^ViiV Healthcare, Durham, NC, US; ^3^GSK, Collegeville, PA, US; ^4^ViiV Healthcare, Branford, CT, US; ^5^ViiV Healthcare, London, UK; ^6^Trialmed, Austin, TX, US; ^7^Trialmed, Las Vegas, NV, US; ^8^GSK, Amersfoort, Netherlands

**Background**: VH4011499 (VH‐499) is a new HIV‐1 capsid inhibitor in development for treatment of HIV‐1. Oral VH‐499 monotherapy demonstrated highly potent antiviral activity in people with HIV‐1 and has been well tolerated across all studies to date. Injectable VH‐499 formulations showed potential for twice‐yearly dosing in a first‐time in‐human study; interim safety, pharmacokinetic (PK), tolerability and acceptability findings are presented.

**Methods**: In this phase 1, double‐blind (sponsor‐unblind), ongoing study (NCT06724640), adults without HIV were randomized to receive single ascending doses (100–1800 mg) administered subcutaneously (SC; abdomen) or intramuscularly (IM; *gluteus medius*) or repeat doses (600 mg IM on day 1; 1200 mg IM on day 29) of VH‐499 or placebo. Pre‐injection ice packs were used for cohorts dosed ≥800 mg. Safety, tolerability, PK and participant‐reported outcomes were assessed. Single‐dose PK parameters using available data were evaluated by non‐compartmental analyses.

**Results**: Overall, 122 participants received VH‐499 or placebo via SC (*n* = 48), single IM (*n* = 49) or repeat IM (*n* = 25) injection. Eighty‐one percent of participants were assigned male sex at birth and 48% identified as non‐white. Most adverse events (AEs) were grade 1 or 2 in severity. The primary VH‐499‐related AEs were injection‐site reactions (ISRs), with injection‐site pain (84% [77/92]) being most common. Median overall ISR event duration ranged from 1 to 5 days across all cohorts. There were no withdrawals due to AEs and no serious VH‐499‐related AEs. No clinically significant trends in laboratory tests, vital signs or electrocardiogram parameters were observed. VH‐499 had a long half‐life following SC and IM administration. Most participants rated post‐injection pain and local reactions as totally/very acceptable; self‐reported pain was mild to moderate and transient.

**Conclusions**: Both SC and IM VH‐499 formulations demonstrated a favourable safety and tolerability profile with no VH‐499‐related serious AEs or discontinuations; post‐injection pain was transient and generally acceptable. VH‐499 demonstrated a long half‐life following SC and IM administration, supporting the goal of twice‐yearly dosing. These findings support further evaluation of injectable long‐acting VH‑499.

### P042 Antiviral Activity, Pharmacokinetics and Safety of Oral GS‐3242 for HIV‐1 Treatment

Samir Gupta^1^, Mezgebe Berhe^2^, Anchalee Avihingsanon^3^, Nan Zhang^4^, Aobo Wang^4^, Kristen Andreatta^4^, Ramya Rao^4^, Kwad Mponponsuo^4^, Peter Sklar ^4^, Gary Sinclair^5^

^1^Indiana University School of Medicine, Indianapolis, IN, US; ^2^Baylor University Medical Center, Dallas, TX, US; ^3^HIVNAT, Thai Red Cross AIDS and Infectious Diseases Research Centre and Center of Excellence in Tuberculosis, Faculty of Medicine, Chulalongkorn University, Bangkok, Thailand; ^4^Gilead Sciences, Inc., Foster City, CA, US; ^5^Prism Health North Texas, Dallas, TX, US

**Background**: Long‐acting antiretrovirals for HIV‐1 treatment, both injectable and oral, may help to address barriers to suboptimal adherence. GS‐3242 is an integrase strand transfer inhibitor (INSTI) with potent anti‐HIV‐1 activity and physiochemical properties well suited for long‐acting formulations. Pharmacokinetics of injectable GS‐3242 in humans have been previously presented (CROI 2026 #174).

**Methods**: This open‐label, phase 1b, multicentre study enrolled participants with HIV‐1 who were INSTI‐naïve and had not received any antiretrovirals within 12 weeks prior to screening. Participants received oral GS‐3242 once daily on days (D) 1 and 2 (fasted) in four dose cohorts (75, 175, 450 or 900 mg). The primary endpoint was change in plasma HIV‐1 RNA from baseline to D11. Secondary endpoints included the pharmacokinetics, pharmacokinetic–viral dynamic relationships of HIV‐1 and safety.

**Results**: A total of 26 participants (*n* = 6, *n* = 6, *n* = 6 and *n* = 8 in the 75, 175, 450 and 900 mg cohorts, respectively) were enrolled and dosed. Median (range) age was 29 (18–65) years; 84.6% were male. Mean (95% CI) changes in HIV‐1 RNA from baseline to D11 were −2.11 (–2.45; −1.78), −2.01 (–2.44; −1.59), −2.29 (–2.83; −1.76) and −2.41 (–2.68; −2.14) log_10_ copies/mL in the 75, 175, 450 and 900 mg cohorts, respectively (Figure 1). Median terminal half‐life ranged from 5.59 to 6.47 days across cohorts. Mean GS‐3242 concentrations on D11 were 1.71, 3.37, 6.48 and 10.2 µg/mL in 75, 175, 450 and 900 mg cohorts, respectively. Across the entire individual exposure range at D11 (from 0.88‐ to 16.0‐fold the protein‐adjusted EC_95_ of 0.957 µg/mL), robust anti‐viral activities were observed. All treatment‐related adverse events were grade 1.

**Conclusions**: GS‐3242 demonstrated robust antiviral activity across a wide range of GS‐3242 exposures with favourable pharmacokinetic and safety profiles. These findings support further development of GS‐3242 as part of an oral or injectable long‐acting HIV‐1 treatment regimen.

**FIGURE 1:**
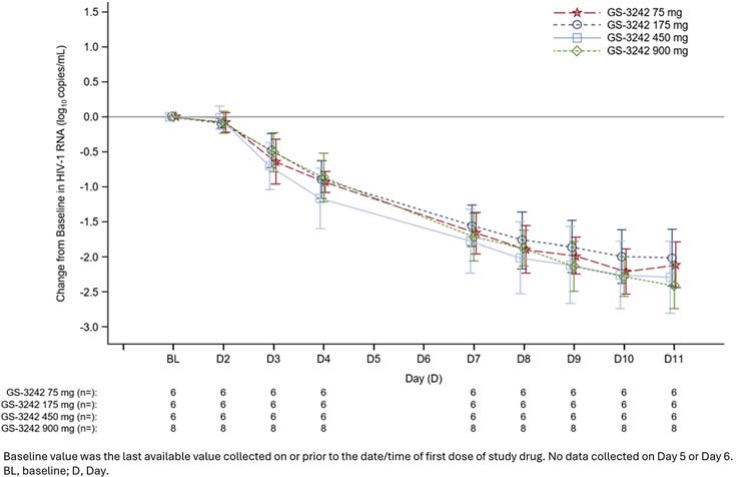
P042 | Mean (95% CI) HIV‐1 RNA (log_10_ copies/mL) change from baseline to day 11 by visit day.

### P043 The Evolution of Integrase Strand Transfer Inhibitors: VH‐184, a Third‐Generation Inhibitor With an Enhanced Resistance Profile

Jerry Jeffrey^1^, Jon Collins^1^, Luise Rogg^1^, Joseph Horton^2^, Sherene Min^1^, Cynthia Donovan^1^, Turner Overton ^1^, Jorge Villacian^3^

^1^ViiV Healthcare, Durham, NC, US; ^2^Parexel International, Durham, NC, US; ^3^ViiV Healthcare, London, UK

**Background**: Integrase strand transfer inhibitors (INSTIs) have transformed antiretroviral therapy, with INSTIs improving potency and tolerability and reducing treatment‐emergent resistance. Even with INSTIs, durability can be challenged by virus heterogeneity and selective pressure. Continued innovations in INSTIs should enable a higher barrier to resistance. VH4524184 (VH‐184) is a third‐generation INSTI with an enhanced resistance profile.

**Methods**: We present data showing the evolution of the INSTI resistance barrier. In vitro studies evaluated the resistance profile of dolutegravir (DTG) against clinical isolates and site‐directed mutants with first‐generation INSTI resistance. Furthermore, twice‐daily DTG 50 mg was evaluated for clinical efficacy in VIKING‐3 and −4 in participants with INSTI‐resistant virus. VH‐184 was also evaluated in vitro against a panel of second‐generation INSTI‐resistant HIV‐1 pseudotyped viruses from participants in SAILING (*n* = 7; week [W] 132) and DAWNING (*n* = 7; W216; Table 1). Wild‐type‐level activity was defined as a half‐maximal inhibitory concentration fold change ≤2.

**Results**: DTG demonstrated an improved in vitro resistance profile versus first‐generation INSTIs, showing activity against clinical isolates and site‐directed mutants with signature raltegravir resistance‐associated mutations. In VIKING‐3 and −4, participants with Q148 + ≥2 secondary mutations had lower virologic suppression rates compared with the overall cohorts (VIKING‐3, 29% [6/21] vs. 63% [116/183]; VIKING‐4, 25% [1/4] vs. 40% [12/30]). In vitro, VH‐184 demonstrated potent antiviral activity and retained activity against viruses with Q148R + ≥2 secondary substitutions, R263K alone and in combination with most other substitutions and G118R in combination with most other substitutions (Figure 1).

**Conclusions**: In vitro data demonstrate the enhanced resistance profile of VH‐184, showing activity against first‐ and second‐generation INSTI‐resistant strains. VH‑184 represents the next step in the evolution of the INSTI class, with an enhanced resistance profile intended to support long‐term treatment durability and the potential for longer dosing intervals, including twice‐a‐year dosing, as clinical development progresses.

**FIGURE 1:**
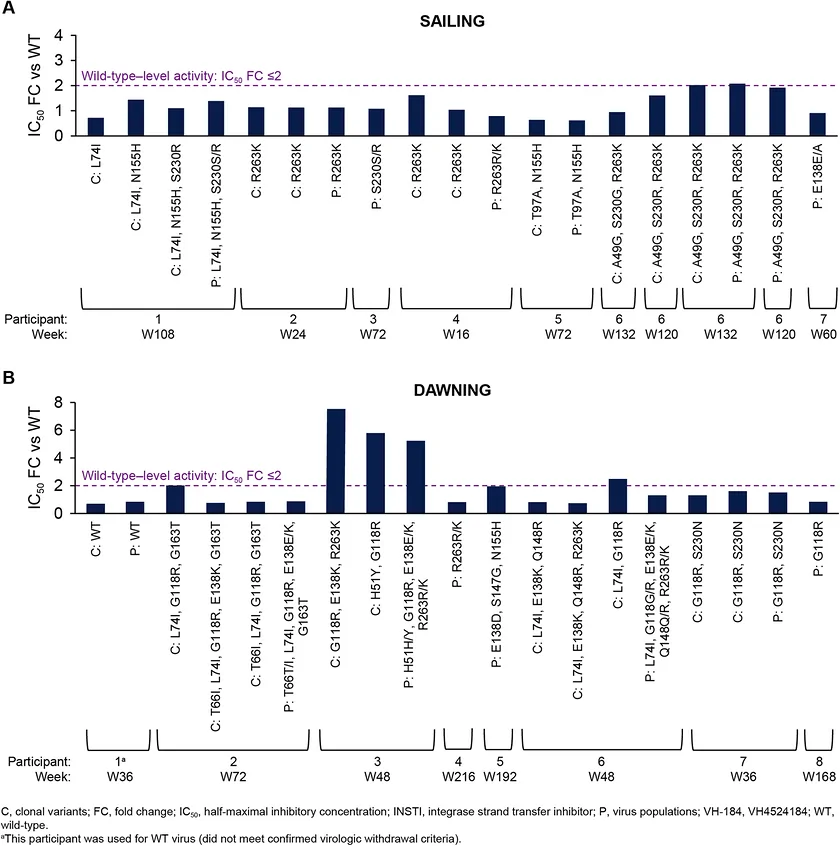
P043 | VH‐184 antiviral activity against INSTI‐resistant pseudotyped virus populations and clonal variants from (A) SAILING and (B) DAWNING.

**TABLE 1:**
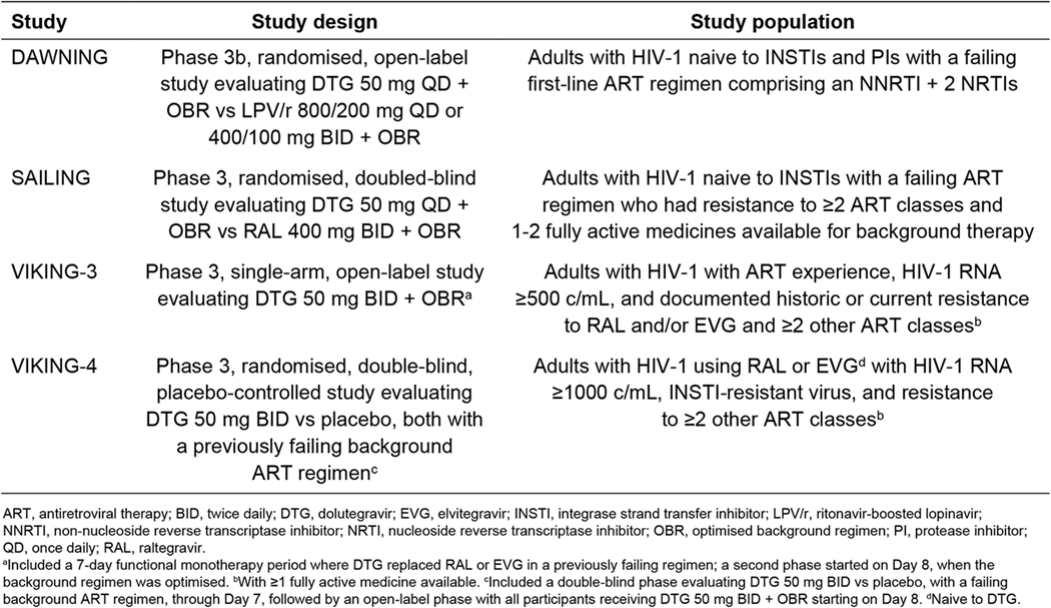
P043 | Key clinical trials informing resistance profiles of second‐generation INSTIs.

### P044 Therapeutic Development of Novel Proteolysis‐Targeting Chimeras for Degradation of HIV‐1 Integrase

Faisal Abedin^1^, Xianyou Peng^2^, Madhusoodanan Mottamal^2^, Olaf Kutsch^3^, Guangdi Wang^2^, Thomas Huckaba ^1^

^1^Department of Biology, Xavier University of Louisiana, New Orleans, LA, US; ^2^Department of Chemistry, Xavier University of Louisiana, New Orleans, LA, US; ^3^Department of Medicine, Division of Infectious Diseases, University of Alabama at Birmingham, Birmingham, AL, US

**Background**: HIV‐1 integrase is a crucial therapeutic target. Five IN strand transfer inhibitors (INSTIs) have been approved for treatment and prevention of viral infection, but the emergence of INSTI‐resistant mutant forms of integrase contributes to virological failure. To address this limitation of inhibitor therapy, we have optimized a series of proteolysis‐targeting chimeras (PROTACs) to induce polyubiquitylation and subsequent proteasome‐dependent degradation of the integrase protein (see Figure 1). Since PROTACs do not require long‐lived interactions with their target to induce degradation, they can retain efficacy even in the presence of inhibitor‐resistant mutations.

**Methods**: We performed molecular docking studies to identify the solvent‐exposed portion of the INSTI dolutegravir molecule when bound to HIV‐1 integrase. The solvent‐facing oxazine ring was then conjugated to a series of short linkers and CRBN E3 ligase‐recruiting moieties to form 18 candidate IN‐targeting PROTACs. To test the ability of these compounds to block HIV‐1 infection, we performed infection assays with the dual‐tropic HIV‐1 GFP reporter virus 89ENG to infect Jurkat T cells. Infection levels and cytotoxicity were determined using flow cytometry, followed by cell lysis and western blots to assay for IN degradation. Pharmacokinetic analysis was performed to determine the oral bioavailability of the lead candidate PROTAC molecules.

**Results**: In Jurkat T cell HIV‐1 infection assays, our two lead candidate PROTACs showed IC_50_ values of 0.5 and 0.6 nanoMolar (nM), comparable to the 0.5 nM IC_50_ for dolutegravir alone. Western blot analysis of the assayed cells showed that the concentration of PROTAC that induced degradation of 50% of the integrase protein (DC_50_) was 0.01 and 1.62 nM for our two lead PROTACs. Pharmacological blockade of the ubiquitin‐proteasome system abolished the ability of the PROTACs to induce integrase degradation, although they still retained significant infection inhibition. Pharmacokinetic profiling showed oral bioavailability, with an oral dose of 10 mg/kg inducing peak plasma concentration of 62–85 ng/mL and plasma clearance rates of 6.4–7.1 mL/min/kg.

**Conclusions**: To our knowledge, these compounds represent a first in class integrase degrader with effective inhibition and degradation profiles. Our ongoing studies are testing the ability of these lead compounds to degrade IN and inhibit the infection of HIV harbouring INSTI‐resistant mutations.

**FIGURE 1:**
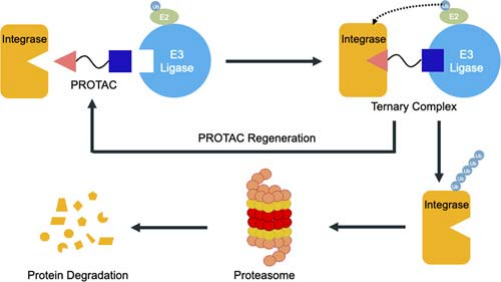
P044 | Model of PROTAC‐mediated integrase degradation via targeting to the ubiquitin‐proteasome system.

### P045 Switching From Standard‐of‐Care Antiretroviral Therapy (SoC ART) to Dual Therapy Lotivibart (LVB) Plus Cabotegravir (CAB) Is Associated With a Modest Reduction in Chronic Innate Immune Activation in Virologically Suppressed Adults With HIV

Michael Keegan ^1^, Xiaowen Chen^2^, Wilson Chen^2^, Kumar Chandan^3^, Peter Leone^2^, Jan Losos^2^, Richard Dunham^2^

^1^ViiV Healthcare, London, UK; ^2^ViiV Healthcare, Durham, NC, US; ^3^GSK, Bangalore, India

**Background**: HIV‐1‐specific broadly neutralizing antibodies (bNAbs) are being investigated for their antiviral activities through neutralization of the HIV Env protein, preventing interaction with cellular receptors and infection of new cells. bNAbs also mediate important immunological effects and may affect immune activation beyond direct antiviral activity. LVB (N6LS) is a bNAb in phase 2b clinical trials for maintenance of HIV‐1 suppression. In this exploratory analysis in virologically suppressed adults from the phase 2b EMBRACE study (NCT05996471), we used serum proteomics to assess the impact of switching from SoC ART to LVB plus CAB.

**Methods**: In EMBRACE, participants (*N* = 125; median [range] baseline CD4+ cell count, 383 [179–850] cells/mm^3^) either continued their SoC ART (*n* = 25) or switched to LVB administered 3 times per year intravenously (*n* = 50) or subcutaneously with recombinant hyaluronidase (rHuPH20) Q4M (*n* = 50), in combination with CAB intramuscular injection Q1M. Serum proteins were measured by Olink REVEAL. Differentially expressed protein (DEP) analysis was performed using limma on proteins with NPX values greater than the limit of detection in ≥50% of samples. The differential expression cutoff was a fold change of 1.2; a Benjamini–Hochberg (BH)‐adjusted *p* value of ≤0.05 was used. Findings were confirmed using per‐protein pairwise *t* tests using an adjusted (BH) *p* value set at 0.05. DEPs at each follow‐up time point up to month 12 were compared with day 1 baseline within each arm. Pathway enrichment analysis was performed using R package clusterProfiler based on GO‐BP, KEGG and Reactome databases.

**Results**: Table 1 details the DEPs at each time point by study arm. The main biological pattern observed in the LVB arms was a modest fall in serum markers of chronic monocyte (FGR, ARG1) and neutrophil activation (ARG1, CEACAM3, PADI4, RAB44), NF‐κB‐related proteins (CHUK, IKBKG) and cellular stress markers (BCL2L15, NAGK, ANP32E, APEX1, CFDP1). The proteomic signature in the SoC arm was stable across 12 months of treatment.

**Conclusions**: Switching from SoC ART to a dual regimen of LVB plus CAB was associated with a serum proteomic profile consistent with a modest reduction in chronic innate immune activation in virologically suppressed people with HIV‐1.

**TABLE 1:**
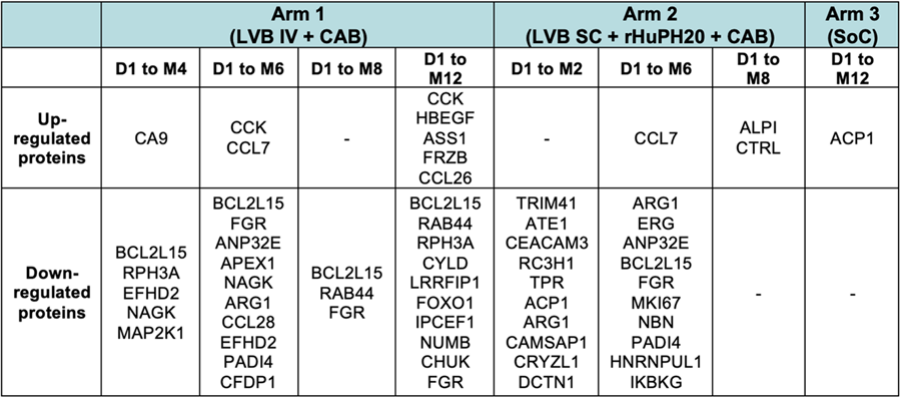
P045 | DEPs by adjusted *p* value (limma).

### P046 Positive Experiences of Healthcare Staff With Administration of Twice‐Yearly Intravenous Lotivibart for HIV‐1 Treatment in the EMBRACE Study

Cassidy A. Gutner ^1^, Irina Kolobova^2^, Peter Leone^3^, Riccardo D'Agostino^4^, Michael Warwick‐Sanders^4^, Viviana Wilches^5^, Owen Cooper^6^, Jack Brouwer^7^, Jennifer N. Hill^8^, Jan Losos^3^

^1^Global Implementation Science, ViiV Healthcare, Durham, NC, US; ^2^Global Health Outcomes, ViiV Healthcare, Durham, NC, US; ^3^Research and Development, ViiV Healthcare, Durham, NC, US; ^4^Safety Evaluation and Risk Management, GSK, London, UK; ^5^Research and Development, GSK, Collegeville, PA, US; ^6^Patient Centered Solutions, IQVIA, London, UK; ^7^Patient Centered Solutions, IQVIA, Amsterdam, Netherlands; ^8^Patient Centered Solutions, IQVIA, New York, NY, US

**Background**: As HIV treatment expands to include long‐acting (LA) regimens, understanding healthcare staff experiences with treatment administration is important for successful real‐world implementation. Part 2 of the phase 2b EMBRACE study evaluated efficacy, safety and tolerability of the broadly neutralizing antibody lotivibart (LVB, N6LS, VH3810109) administered intravenously (IV) 2 times a year plus intramuscular (IM) cabotegravir LA 6 times a year versus oral standard‐of‐care antiretroviral therapy. We explored healthcare staff experiences with twice‐yearly lotivibart administration in EMBRACE.

**Methods**: Semi‐structured qualitative interviews were conducted at month 6 with a subset (*N* = 21) of EMBRACE study staff from US sites administering lotivibart. Staff included both lotivibart administrators and individuals responsible for oversight of administration. Interview responses were thematically coded and analysed descriptively.

**Results**: Most (90% [18/20]) staff felt enthusiastic about twice‐yearly lotivibart (Table 1). A majority reported staff‐perceived patient benefits, including not having to take daily pills (62% [13/21]) and positive views on extended dosing intervals (57% [12/21]). Many (72% [13/18]) staff also perceived the potential for improved adherence with twice‐yearly lotivibart compared with daily oral therapy, noting that patients consistently attended scheduled study visits. Staff perceived less frequent clinic visits and reduced day‐to‐day treatment burden as beneficial to patient quality of life. From a clinical perspective, over half (52% [11/21]) of respondents identified IV infusion preparation as complex in the study setting; this was discussed as logistical challenges rather than as barriers to acceptability. Despite this, 11 staff discussed benefits of this regimen, with 73% (*n* = 8) of those reporting that patients were receptive to the dual‐modality (IV+IM) regimen (Table 1) and many staff emphasized that workflow impacts were manageable with planning. Comparisons with existing LA IM regimens were discussed by 15 staff and some (40% [*n* = 6]) noted no major differences observed in overall patient experience between regimens.

**Conclusions**: Healthcare staff expressed strong enthusiasm and perceived acceptability for twice‐yearly lotivibart. Extended dosing builds on existing LA regimens by further minimizing treatment burden, supporting its feasibility and patient‐centred value as a next step in HIV care.

**TABLE 1:**
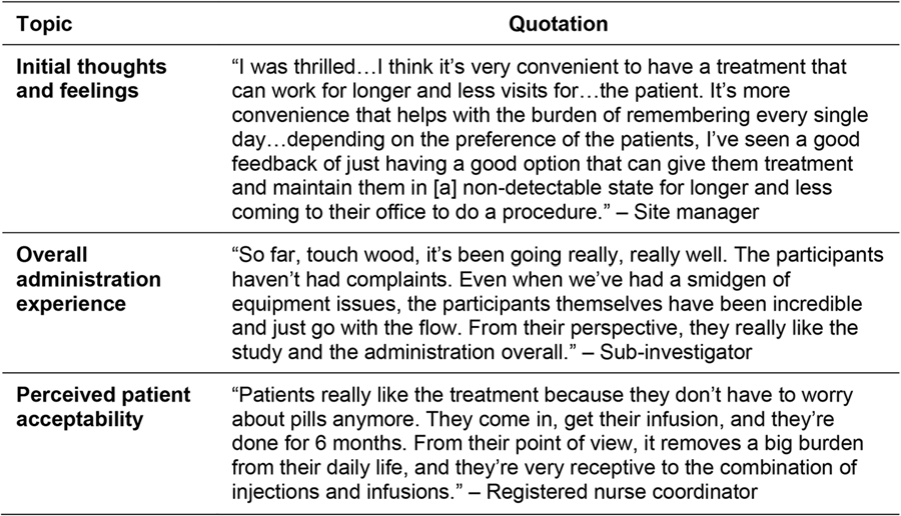
P046 | Healthcare staff perspectives of twice‐yearly lotivibart administration.

### P047 Chidamide Synergizes With NKG2D CAR‐NK Cells to Clear HIV Latency by Upregulating Ligand Expression

Biao Zhu, Zhikai Wan, Xiaotang Zhou, Xueling Zhu, Bohao Dai

The Department of Infectious Diseases, State Key Laboratory for Diagnosis and Treatment of Infectious Diseases, National Clinical Research Center for Infectious Diseases, the First Affiliated Hospital, School of Medicine, Hangzhou, Zhejiang, China

**Background**: Eliminating HIV latently infected cells remains a pivotal challenge for achieving a functional cure for AIDS. The “shock and kill” strategy aims to induce viral antigen expression using latency‐reversing agents (LRAs), followed by targeted clearance through immune effector cells. This study investigates the synergistic mechanism between the histone deacetylase inhibitor chidamide (an LRA) and NKG2D ligand‐targeting CAR‐NK cell therapy, focusing on whether chidamide can upregulate NKG2D ligands on latently infected cells and thereby enhance CAR‐NK recognition and killing.

**Methods**: Two well‐established HIV‐1 latency cell models—J‐LAT 10.6 and J‐LAT C11—were employed. Surface expression of NKG2D ligands (MICA, MICB, ULBP‐1, ULBP‐2/5/6) was assessed by flow cytometry at baseline and after 48‐h treatment with chidamide. To evaluate functional synergy, target cells pretreated with chidamide or solvent control were co‐cultured with NKG2D CAR‐NK cells at varying effector‐to‐target (E:T) ratios (from 1:1 to 20:1). Cytotoxicity was measured using a short‐term Europium release assay.

**Results**: Baseline expression profiles of NKG2D ligands differed between the two cell lines: ULBP‐1 and ULBP‐2/5/6 were moderately expressed in J‐LAT C11, whereas MICA and MICB were low; in contrast, J‐LAT 10.6 exhibited higher levels of MICB and ULBP family members. Chidamide treatment significantly upregulated the expression of MICA and MICB on both cell lines (Figure 1). CAR‐NK cells effectively lysed both target cell types and chidamide pretreatment further enhanced cytotoxicity. This enhancement was significant across all E:T ratios in J‐LAT C11, particularly at lower ratios and was also statistically significant at a low E:T ratio (1:1) in J‐LAT 10.6 (Figure 2).

**Conclusions**: This study demonstrates in vitro that chidamide not only reverses HIV latency but also upregulates specific NKG2D ligands (MICA/MICB) on latently infected cells, thereby enhancing their recognition and elimination by NKG2D CAR‐NK cells. These findings provide mechanistic support for the “chidamide plus CAR‐NK” strategy as a promising approach towards HIV functional cure, warranting further validation in primary cell models and more clinically relevant systems.

**FIGURE 1:**
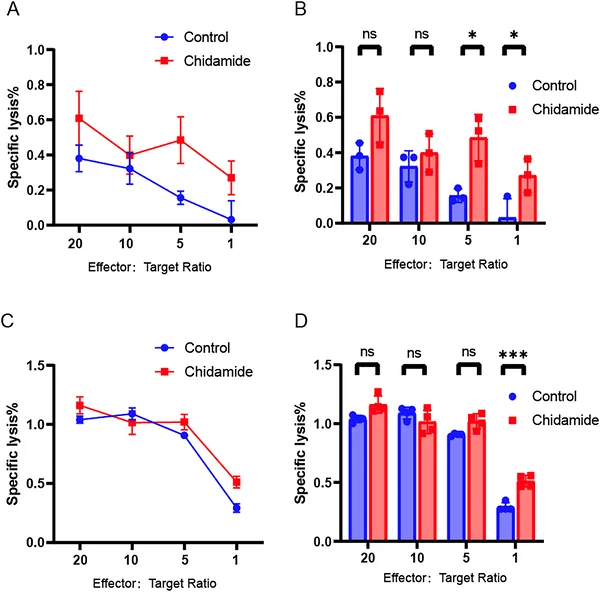
P047 | Chidamide upregulates surface expression of MICA/B in HIV‑1 latently infected J‑LAT cell lines. (A, B) Representative flow cytometry histograms showing surface expression of MICA (A) and MICB (B) on J‑LAT C11 cells after 48 h treatment with chidamide or solvent control; (C, D) representative histograms showing surface expression of MICA (C) and MICB (D) on J‑LAT 10.6 cells under the same treatment conditions.

**FIGURE 2:**
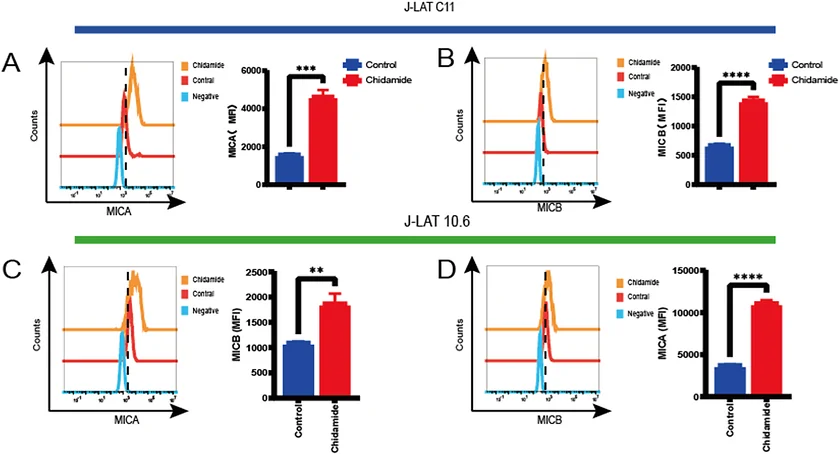
P047 | Chidamide pretreatment enhances NKG2D CAR‑NK‑mediated killing of HIV‑1 latently infected cells. (A, B) Cytotoxicity against J‑LAT C11 cells. The killing activity of NKG2D CAR‑NK cells was measured using a DELFIA EuTDA release assay (2 h co‑culture) at effector‑to‑target (E:T) ratios ranging from 1:1 to 20:1. (C, D) Cytotoxicity against J‑LAT 10.6 cells under the same experimental conditions.

## Treatment Strategies: RCTs—Oral and Injectable Therapy in First‐Line and Suppressed Switch Populations

### P048 Bictegravir/Lenacapavir: Baseline and Week 48 Resistance in the ARTISTRY‐2 Trial

Michelle L. D'Antoni ^1^, Kristen Andreatta^1^, Cameron Soulette^2^, Priyanka Arora^3^, Eric Meissner^4^, Kwad Mponponsuo^5^, Peter Sklar^5^, Nicolas Margot^1^, Christian Callebaut^1^

^1^Clinical Virology, Gilead Sciences Inc., Foster City, CA, US; ^2^Research Data Sciences, Gilead Sciences Inc., Foster City, CA, US; ^3^Clinical Pharmacology, Gilead Sciences Inc., Foster City, CA, US; ^4^Department of Medicine, Medical University of South Carolina, Charleston, SC, US; ^5^Clinical Development, Gilead Sciences Inc., Foster City, CA, US

**Background**: ARTISTRY‐2 (NCT06333808) is a phase 3 trial evaluating the efficacy and safety of switching to bictegravir/lenacapavir (BIC/LEN) single‐tablet regimen (STR) versus continuing bictegravir/emtricitabine/tenofovir alafenamide (B/F/TAF) in people with HIV (PWH) with virologic suppression (VS). BIC/LEN maintained high VS levels and was non‐inferior to B/F/TAF at week (W) 48. We report the resistance profile of participants at baseline through W48.

**Methods**: Pre‐existing resistance‐associated mutations (RAMs) in protease, reverse transcriptase (RT) and integrase were determined using historical genotypic reports and retrospective proviral DNA genotyping of baseline samples. Effects of baseline RAMs on W48 efficacy (HIV‐1 RNA <50 copies/mL; US FDA Snapshot algorithm) were evaluated. Post‐baseline resistance testing (capsid, protease, RT, integrase) and pharmacokinetic analyses (BIC/LEN only) were performed for participants who experienced virologic failure (confirmed virologic rebound or last visit with HIV‐1 RNA ≥200 copies/mL). The final resistance analysis population (RAP) excluded participants who resuppressed by W48.

**Results**: Baseline resistance was determined for 511/574 (89.0%) participants. Primary nucleos(t)ide RT inhibitor (NRTI), non‐nucleoside RT inhibitor (NNRTI), protease inhibitor (PI) and integrase strand transfer inhibitor (INSTI) RAMs were detected in 18.0%, 23.3%, 9.4% and 2.6% of participants, respectively (Table 1). The most prevalent RAMs detected were M184I/V (11.5%) and K103N/S (10.4%) in RT. Baseline RAMs did not affect BIC/LEN or B/F/TAF efficacy at W48 (NRTI‐resistance [R], NNRTI‐R, PI‐R, INSTI‐R; all *p* > 0.05). The final RAP included four participants (BIC/LEN = 2; B/F/TAF = 2); no resistance to study drugs was detected through W48 in three participants. In one participant in the BIC/LEN group with prior INSTI treatment, R263K in integrase was detected at W36; phenotypic susceptibility to BIC was maintained (IC_50_ fold‐change from wild‐type 2.0), no capsid RAMs were detected and BIC and LEN plasma levels were within the overall observed range.

**Conclusions**: BIC/LEN STR maintained high VS rates regardless of preexisting RAMs. Genotypic analysis demonstrated treatment‐emergent RAMs were rare, occurring in 1/383 (0.3%) participants receiving BIC/LEN, with phenotypic susceptibility to study drugs maintained. These data underscore the potential of BIC/LEN STR as a treatment option for PWH with VS.

**TABLE 1:**
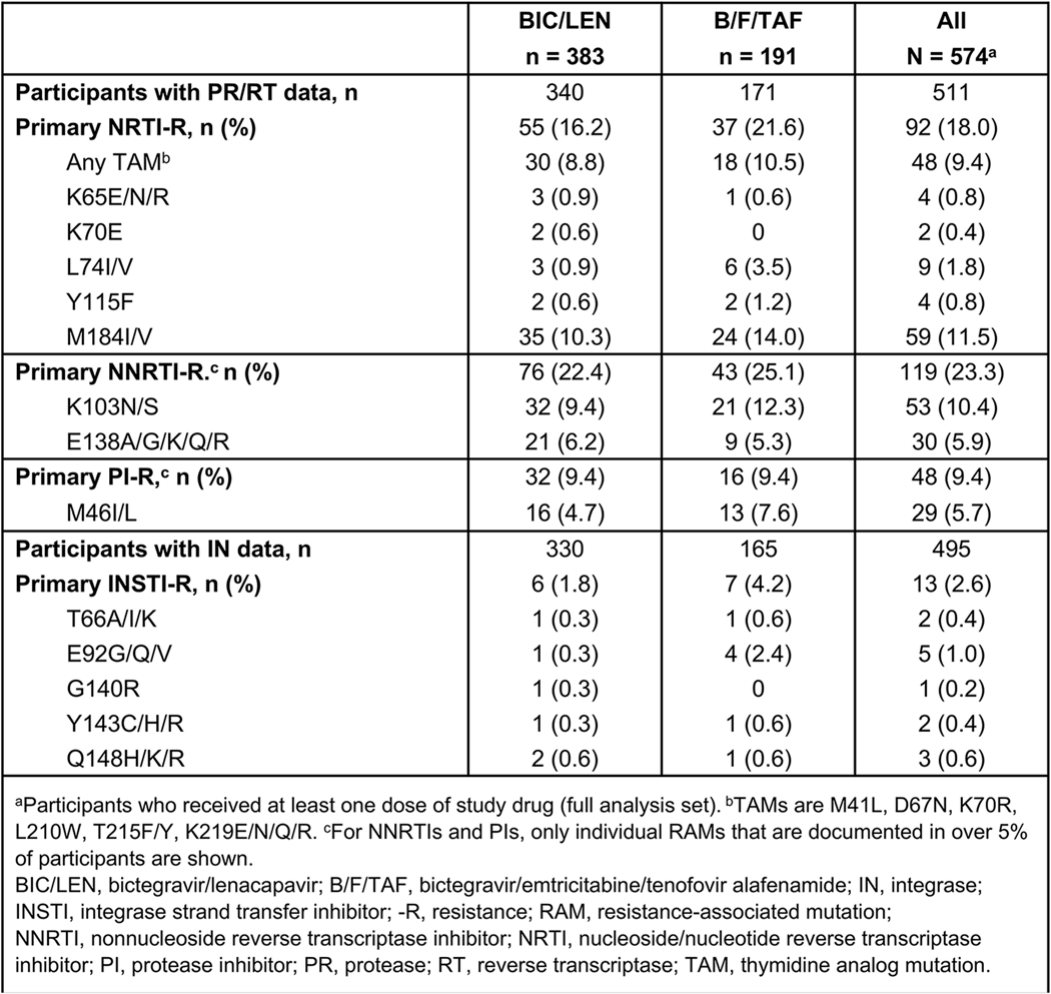
P048 | Pre‐existing NRTI, NNRTI, PI and INSTI RAMs.

### P049 Efficacy and Safety of Once‐Weekly Oral Islatravir/Lenacapavir (ISL/LEN) Versus Daily Standard of Care (SOC) Across Demographic and Clinical Subgroups Through Week 48 in the Phase 3 ISLEND‐2 Trial

Miłosz Parczewski ^1^, Patricia Echeverría^2^, Luis Fernando López Cortés^3^, Georg M. Behrens^4^, Anchalee Avihingsanon^5^, Alexandra Calmy^6^, Julie Fox^7^, Marcelo H. Losso^8^, Bartosz Szetela^9^, María De Lagarde Sebastián^10^, Olayemi Osiyemi^11^, Joel Gallant^12^, Richard Kaplan^13^, Thomas B. Campbell^14^, Hongyuan Wang^15^, Ke Chen^16^, Fadi Shihadeh^15^, Tracy L. Diamond^16^, Elena Bekerman^15^, Hadas Dvory‐Sobol^15^, Cyril Llamoso^16^, Devi SenGupta^15^, Amy E. Colson^17,18^, on behalf of ISLEND‐2 Study Team

^1^Pomeranian Medical University, Szczecin, Poland; ^2^Hospital Universitari Germans Trias i Pujol, Badalona, Spain; ^3^Hospital Universitario Virgen del Rocío, PPDS, Seville, Spain; ^4^Medizinische Hochschule Hannover, Hannover, Germany; ^5^HIV Netherlands Australia Thailand Research Collaboration (HIV‐NAT), Thai Red Cross AIDS Research Centre, Bangkok, Thailand; ^6^Hôpitaux Universitaires de Genève, Geneva, Switzerland; ^7^Guy's Hospital, London, UK; ^8^Hospital General de Agudos Dr. José María Ramos Mejía, Buenos Aires, Argentina; ^9^Wroclaw Medical University PLUS Wrocławskie Centrum Zdrowia SP ZOZ, Wrocław, Poland; ^10^Hospital Universitario 12 de Octubre, imas12, Madrid, Spain; ^11^Triple O Research Institute PA, West Palm Beach, FL, US; ^12^AXCES Research Group, Santa Fe, NM, US; ^13^Groote Schuur Hospital, Cape Town, South Africa; ^14^University of Colorado Anschutz Medical Campus, Aurora, CO, US; ^15^Gilead Sciences, Inc., Foster City, CA, US; ^16^Merck & Co., Inc., Rahway, NJ, US; ^17^Community Resource Initiative, Boston, MA, US; ^18^Cambridge Health Alliance, Cambridge, MA, US

**Background**: Long‐acting oral HIV‐1 treatments with reduced dosing frequency are desirable to address unmet needs and broaden treatment options for people seeking alternatives to daily oral or long‐acting injectable antiretroviral therapy (ART). In the phase 3 ISLEND‐2 trial, switching to once‐weekly oral ISL/LEN was non‐inferior to continuing daily oral SOC in maintaining high rates of virologic suppression through week 48 and was well tolerated in a diverse population of people with HIV‐1 (PWH). Here, we present week 48 analyses by subgroups based on baseline demographic and clinical characteristics.

**Methods**: ISLEND‐2 (NCT06630299) is a global, open‐label non‐inferiority trial. Adult PWH who were virologically suppressed on guideline‐recommended oral SOC ART were randomized 1:1 to switch to once‐weekly oral ISL/LEN (2/300 mg) or continue daily oral SOC for 96 weeks. The primary efficacy endpoint was the proportion of participants with HIV‐1 RNA ≥50 copies/mL at week 48 (US FDA Snapshot algorithm), with a non‐inferiority margin of 4%. Analyses were conducted in subgroups stratified by ethnicity and baseline M184V/I status (efficacy analyses only), as well as by sex at birth, age, race, geographic region, baseline CD4+ T‐cell count and baseline ART.

**Results**: In the overall population, ISL/LEN was non‐inferior to SOC at week 48: 1/314 (0.3%) ISL/LEN and 4/312 (1.3%) SOC participants had HIV‐1 RNA ≥50 copies/mL (difference: −1.0%; 95.002% CI: −3.0, 1.1%), while 299 (95.2%) and 298 (95.5%) had HIV‐1 RNA <50 copies/mL (difference: −0.3%; 95% CI: −3.9%, 3.2%), respectively. Virologic suppression was high and generally comparable between treatment groups across subgroups (Figure 1), including in participants who were older or assigned female at birth and regardless of baseline CD4+ T‐cell count. Efficacy was also consistent regardless of baseline M184V/I status or baseline ART. The incidence of adverse events was generally similar across treatment groups and subgroups, with no new safety signals (Table 1).

**Conclusions**: In the diverse population enrolled in ISLEND‐2, once‐weekly oral ISL/LEN demonstrated consistent efficacy and safety across participant subgroups. These data support the potential for ISL/LEN to be the first complete once‐weekly oral single‐tablet regimen option for PWH.

**Funding**: Gilead Sciences, Inc. and Merck Sharp & Dohme LLC.

**FIGURE 1:**
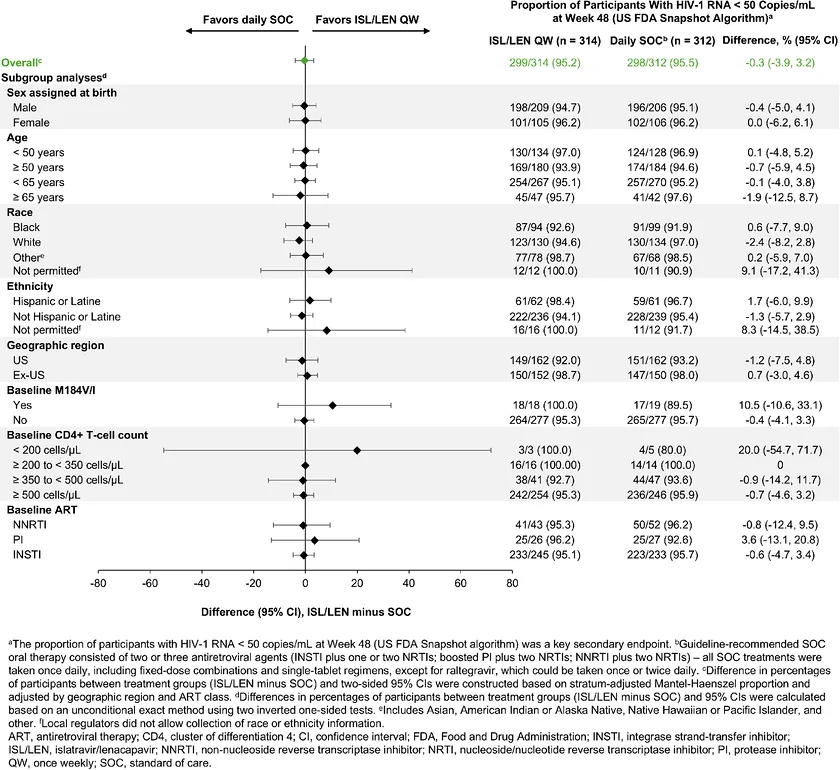
P049 | Maintenance of virologic suppression at week 48 in ISLEND‐2: subgroup analysis.

**TABLE 1:**
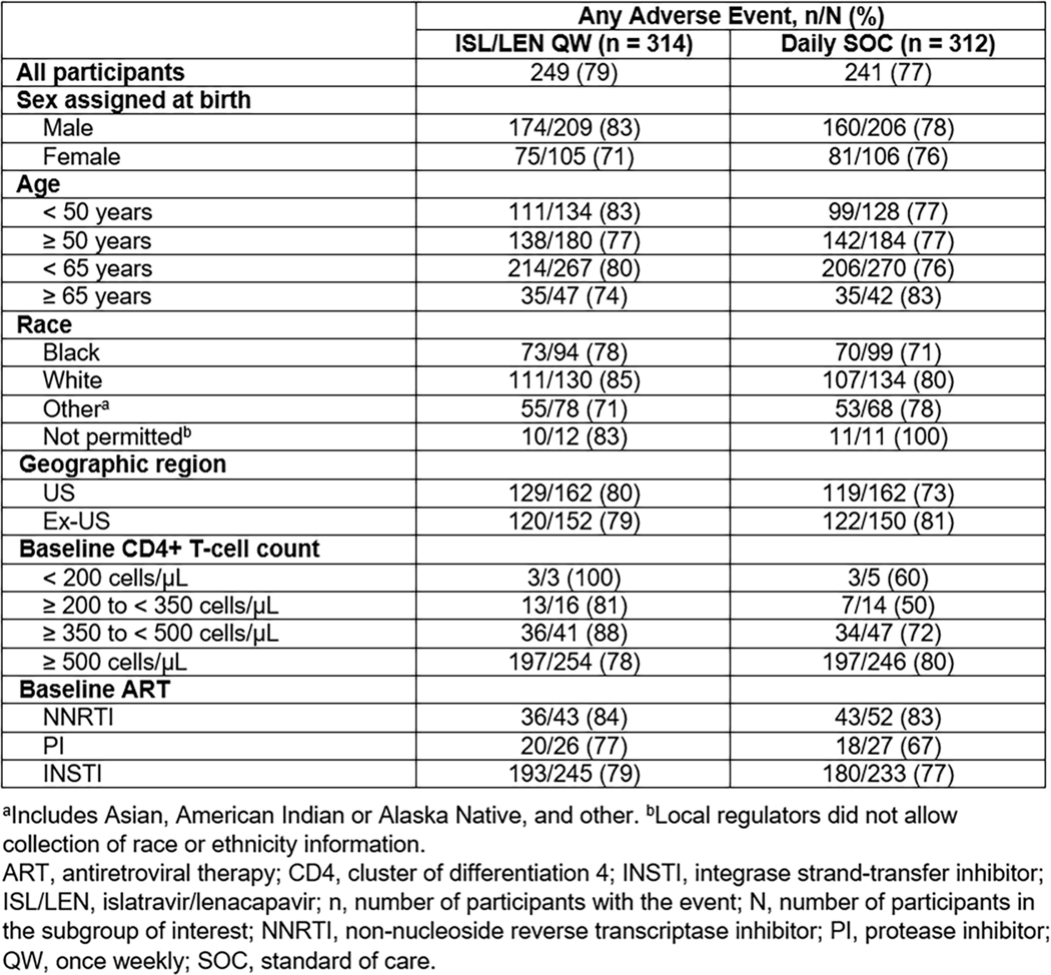
P049 | Safety profile by subgroup through week 48 in ISLEND‐2.

### P050 Once‐Weekly Oral Islatravir/Lenacapavir (ISL/LEN) Versus Daily Bictegravir/Emtricitabine/Tenofovir Alafenamide (B/F/TAF) in Virologically Suppressed Adults With HIV‐1: Week 48 Subgroup Analyses of the Phase 3 ISLEND‐1 Trial

Jürgen Rockstroh^1^, Anthony Mills^2^, Benoit Trottier^3^, Shawn Hassler^4^, Manoj Dalmia^5^, Bertrand Lebouché^6^, Cynthia Gay^7^, Alvaro Mena de Cea^8^, Clara Lehmann^9^, Hartmut Stocker^10,11^, Javier Morales‐Ramirez^12^, Michael Sension^13^, Marta Boffito^14^, Michinori Shirano^15^, Shan‐Yu Liu^16^, Ke Chen^17^, Fadi Shihadeh^16^, Jason Kim^17^, Renee‐Claude Mercier^16^, Hadas Dvory‐Sobol^16^, Cyril Llamoso^17^, Devi SenGupta^16^, Chloe Orkin ^18^, on behalf of ISLEND‐1 Study Team

^1^University Hospital Bonn, Bonn, Germany; ^2^Mills Clinical Research at Men's Health Foundation, Los Angeles, CA, US; ^3^Clinique de Médecine Urbaine du Quartier Latin, Montreal, QC, Canada; ^4^Optimus Medical, San Francisco, CA, US; ^5^Brooklyn Clinical Research, Brooklyn, NY, US; ^6^McGill University Health Centre, Montreal, QC, Canada; ^7^University of North Carolina at Chapel Hill, Chapel Hill, NC, US; ^8^Hospital Universitario A Coruña, A Coruña, Spain; ^9^Faculty of Medicine and University Hospital Cologne, University of Cologne, Cologne, Germany; ^10^Epimed GmbH, Berlin, Germany; ^11^ St. Joseph Krankenhaus Berlin‐Tempelhof, Berlin, Germany; ^12^Clinical Research Puerto Rico Inc., San Juan, Puerto Rico, US; ^13^CAN Community Health Care, Inc., Fort Lauderdale, FL, US; ^14^Chelsea and Westminster Hospital—PPDS, London, UK; ^15^Osaka City General Hospital, Osaka, Japan; ^16^Gilead Sciences, Inc., Foster City, CA, US; ^17^Merck & Co., Inc., Rahway, NJ, US; ^18^SHARE Collaborative, Blizard Institute, Faculty of Medicine and Dentistry, Queen Mary University of London, London, UK

**Background**: Despite high efficacy, daily oral antiretroviral therapy can be challenging for some people with HIV‐1, driving interest in less frequent dosing strategies. In the phase 3 ISLEND‐1 trial, switching to once‐weekly oral ISL/LEN was non‐inferior to once‐daily oral B/F/TAF in maintaining virologic suppression through week 48 and was well tolerated in a broad population of people with HIV‐1. Here, we present week 48 analyses by subgroups based on baseline demographic and clinical characteristics.

**Methods**: ISLEND‐1 (NCT06630286) is a global, double‐blind non‐inferiority trial, in which adults with HIV‐1 virologically suppressed on B/F/TAF were randomized 1:1 to switch to once‐weekly oral ISL/LEN (2/300 mg; *n* = 304) or continue once‐daily B/F/TAF (50/200/25 mg; *n* = 303), plus matching placebos, for 96 weeks. The primary efficacy endpoint was the proportion of participants with HIV‐1 RNA ≥50 copies/mL at week 48 (US FDA Snapshot algorithm), with a non‐inferiority margin of 4%. Subgroups were defined according to ethnicity and the presence of the M184V/I resistance‐associated mutation at baseline (efficacy analyses only), as well as sex at birth, age, race, geographic region and baseline CD4+ T‐cell count.

**Results**: In the overall population, ISL/LEN was non‐inferior to B/F/TAF at week 48: none of the 304 ISL/LEN participants and 1 of 303 (0.3%) B/F/TAF participants had HIV‐1 RNA ≥50 copies/mL (difference: −0.3%; 95.002% CI −1.4%, 0.8%); correspondingly, 284 (93.4%) and 280 (92.4%) participants had HIV‐1 RNA <50 copies/mL (difference: 1.0%; 95% CI: −3.2%, 5.2%). Efficacy results were consistent across subgroups (Figure 1). The incidence of adverse events was generally similar across treatment groups and subgroups, with no new safety signals (Table 1).

**Conclusions**: In the diverse population enrolled in ISLEND‐1, once‐weekly oral ISL/LEN had a consistent efficacy and safety profile across participant subgroups. These data support ISL/LEN as a potential first complete once‐weekly oral single‐tablet regimen option for PWH.

**Funding**: Gilead Sciences, Inc. and Merck Sharp & Dohme LLC.

**FIGURE 1:**
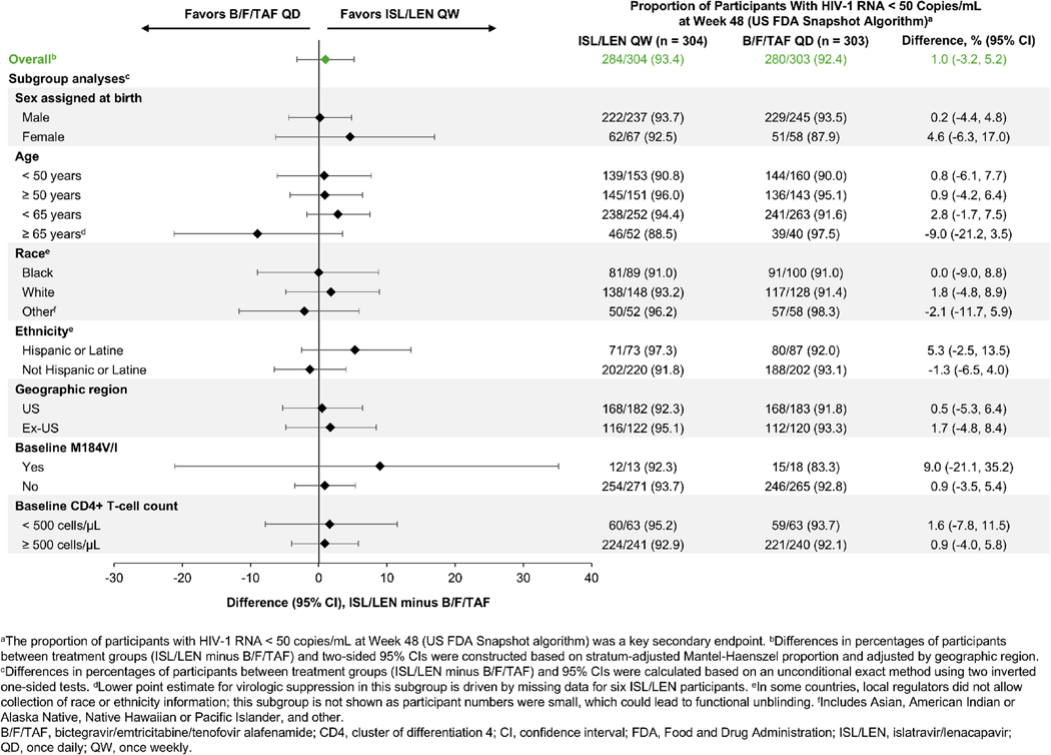
P050 | Maintenance of virologic suppression at week 48 in ISLEND‐1: subgroup analysis.

**TABLE 1:**
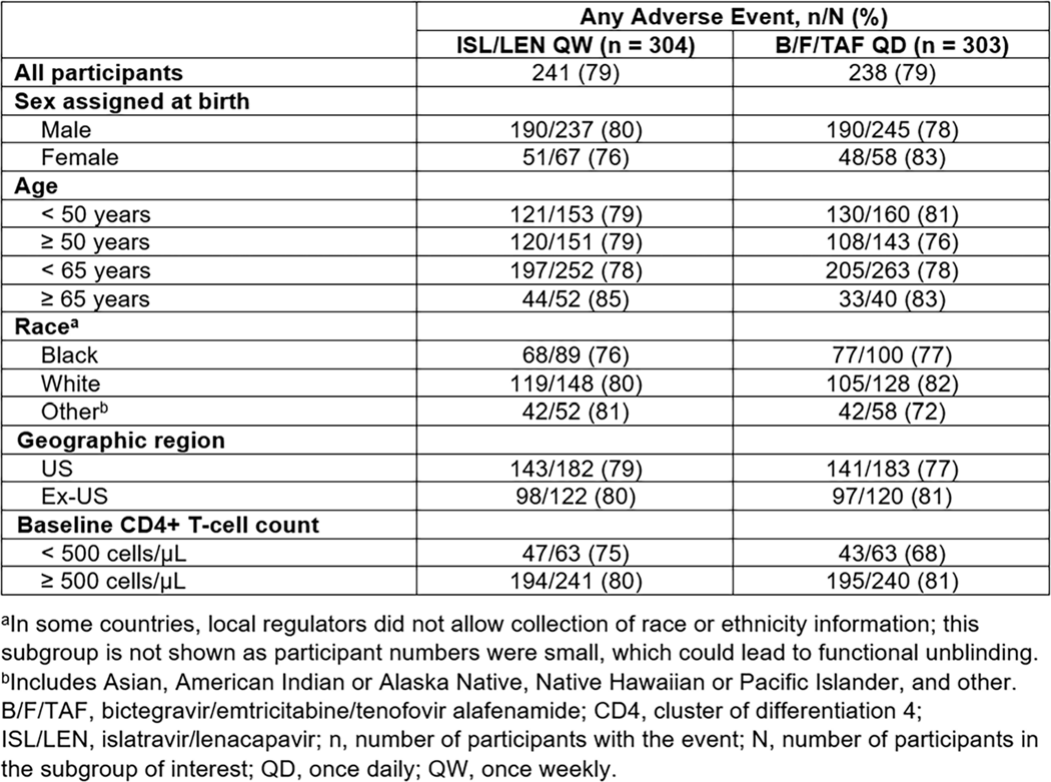
P050 | Safety profile by subgroup through week 48 in ISLEND‐1.

### P051 VOGUE: Consistent Efficacy of DTG/3TC Versus BIC/FTC/TAF Across Clinically Relevant Subgroups in Adults Initiating ART

María de Lagarde Sebastián ^1,2^, Marcelo D. Martins^3^, Dai Watanabe^4^, Kehui Wang^5^, Richard Grove^6^, Ruolan Wang^7^, Adelaide Jewell^8^, Emilie Elliot^9^, Rebecca Borsi^7^, Parul Patel^7^, Michelle Kisare^8^, Piotr Budnik^8^, Jean van Wyk^8^

^1^Hospital Universitario 12 de Octubre, imas12, Madrid, Spain; ^2^CIBERINFEC, Madrid, Spain; ^3^Instituto Oulton, Córdoba, Argentina; ^4^AIDS Medical Center, NHO Osaka National Hospital, Osaka, Japan; ^5^GSK, Collegeville, US; ^6^GSK, London, UK; ^7^ViiV Healthcare, Durham, US; ^8^ViiV Healthcare, London, UK; ^9^ViiV Healthcare, Madrid, Spain

**Background**: In VOGUE, dolutegravir/lamivudine (DTG/3TC) demonstrated non‐inferior efficacy to bictegravir/emtricitabine/tenofovir alafenamide (BIC/FTC/TAF) as first‐line antiretroviral therapy (ART) at week 48, with no treatment‐emergent resistance identified. Week 48 efficacy was assessed across subgroups based on demographics and baseline viral load (VL) and CD4+ cell count randomization strata.

**Methods**: VOGUE (NCT05979311) is an ongoing phase 3b, randomized, open‐label, non‐inferiority study in adults initiating ART prior to availability of resistance testing results and without baseline VL or CD4+ cell count restrictions. The primary endpoint was the proportion of participants with HIV‐1 RNA <50 c/mL at week 48 (Snapshot algorithm; −10% non‐inferiority margin). Treatment differences in virologic suppression rates (HIV‐1 RNA <50 c/mL) and time to virologic suppression (Kaplan–Meier methods) across subgroups were assessed as exploratory analyses.

**Results**: Among 509 randomized participants (DTG/3TC, *n* = 254; BIC/FTC/TAF, *n* = 255), median age was 33 years (range, 18–84) and 16% were assigned female at birth. At baseline, 47% of participants had VL ≥100,000 c/mL and 16% had CD4+ cell count < 200 cells/mm^3^. Across all subgroups, virologic suppression rates were consistently high and comparable between treatment groups (Figure 1). Among participants with baseline VL ≥100,000 c/mL, 85% receiving DTG/3TC and 89% receiving BIC/FTC/TAF achieved HIV‐1 RNA <50 c/mL at week 48 (adjusted difference, −3% [95% credible interval, −11%, 4%]). Median time to virologic suppression was 4.1 weeks in both treatment groups overall and in participants with baseline VL < 100,000 c/mL; adjusting for randomization strata, the hazard ratios showed a statistically significantly higher suppression rate with DTG/3TC (Table 1). For participants with baseline VL ≥100,000 c/mL, median time to virologic suppression was similar between treatment groups (DTG/3TC, 7.7 weeks; BIC/FTC/TAF, 8.1 weeks). Adverse events were comparable between treatment groups.

**Conclusions**: DTG/3TC demonstrated non‐inferior efficacy versus BIC/FTC/TAF at week 48, with no treatment‐emergent resistance identified. Efficacy was high and similar across demographic and baseline subgroups, including high VL and/or low CD4+ cell count. Time to virologic suppression was rapid and similar between treatment groups. These findings support DTG/3TC as an effective, first‐line, 2‐drug regimen for a broad population initiating ART in a test‐and‐treat approach.

**FIGURE 1:**
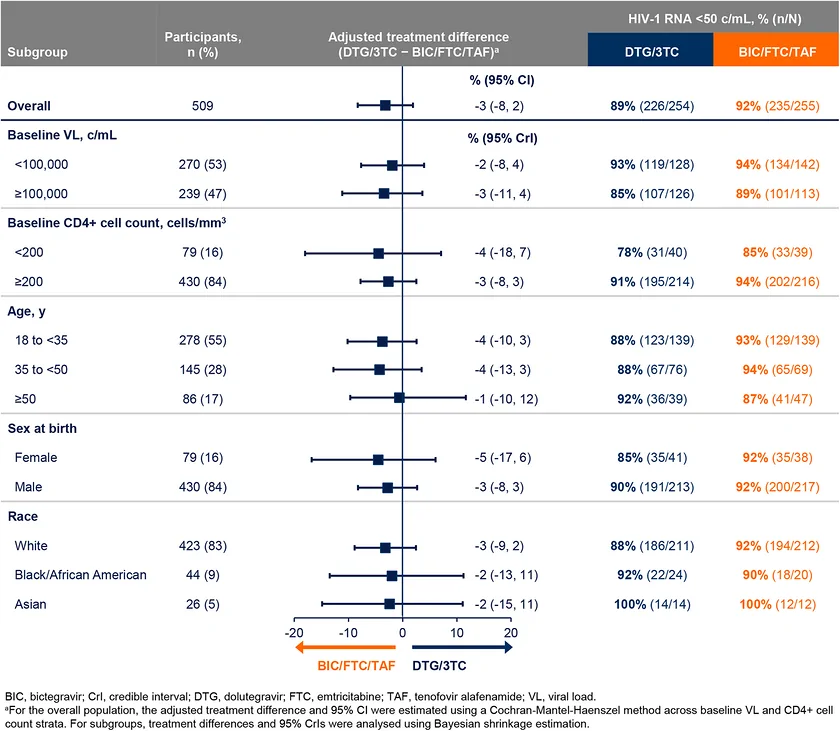
P051 | Consistent virologic suppression across clinically relevant subgroups with DTG/3TC versus BIC/FTC/TAF at week 48 (Snapshot algorithm).

**TABLE 1:**
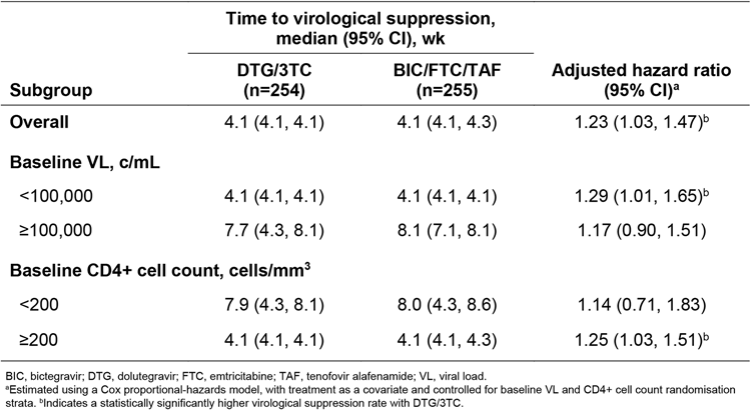
P051 | Time to virologic suppression across baseline VL and CD4+ cell count randomization strata with DTG/3TC versus BIC/FTC/TAF.

### P052 Longitudinal Dynamics of Viraemic Events: A Multi‐State Modelling Analysis of Dolutegravir/Lamivudine Versus 3‐Drug Regimens

Richard Grove ^1^, Pedro Cahn^2^, Josep M. Llibre^3^, Avishek Chatterjee^4^, Lijina Kaiprath^4^, Gustavo Verdier^5^, Piotr Budnik^6^, Michelle Kisare^6^

^1^GSK, London, UK; ^2^Fundación Huésped, Buenos Aires, Argentina; ^3^CIBER Epidemiology and Public Health (CIBERESP), Barcelona, Spain; ^4^GSK, Bangalore, India; ^5^ViiV Healthcare, Montreal, Canada; ^6^ViiV Healthcare, London, UK

**Background**: Viraemic events (VEs) after virological suppression occur with variable outcomes and are not always fully characterized by static endpoints in clinical trials. Prior pooled analyses showed similar VE incidences and outcomes with dolutegravir/lamivudine (DTG/3TC) and 3‐drug regimens (3DRs). Multi‐state modelling evaluated longitudinal transitions between virological states in a pooled analysis of 4 randomized trials (GEMINI‐1/‐2 [treatment‐naïve]; TANGO and SALSA [treatment‐experienced]).

**Methods**: A multi‐state model analysed transitions between virological states through Week 48, accounting for recurrent VEs. A 3‐state continuous‐time Markov model was specified, comprising pre‐first suppression viraemia (HIV‐1 RNA ≥50 c/mL before initial suppression), transitioning to suppression (HIV‐1 RNA <50 c/mL) and allowing transitions to/from post‐suppression viraemia (HIV‐1 RNA ≥50 c/mL after prior suppression). Participants with baseline viral load ≥50 c/mL entered in the pre‐first suppression viraemia state, whereas those with < 50 c/mL entered in the suppression state. Transition intensities were estimated using an Andersen−Gill model. Marginal state occupation probabilities were estimated using G‐computation to quantify the proportion of individuals in each state under each treatment over time.

**Results**: The analysis included 2648 individuals (DTG/3TC, *n* = 1320; 3DR, *n* = 1328). Through Week 48, the hazard of transitioning from suppression to post‐suppression viraemia was lower with DTG/3TC compared with 3DRs (hazard ratio, 0.77 [95% CI: 0.61, 0.96]), whereas rates of suppression following a VE were similar between groups (Table 1). Overall, the probability of being in each state was similar between DTG/3TC and 3DRs at week 48 (Figure 1), with a low probability of post‐suppression viraemia (2% in both groups). The estimated average time spent in the post‐suppression viraemia state was low and similar between groups (treatment difference, −0.18 [95% CI: −0.37, 0.00] weeks).

**Conclusions**: Virological event longitudinal dynamics were comparable between DTG/3TC and 3DRs, including similar rates of suppression following a VE and similar expected time spent in viraemia. A lower hazard of transitioning from suppression to viraemia was observed with DTG/3TC. These findings indicate stable and durable virological control with DTG/3TC across treatment‐naïve and treatment‐experienced populations.

**FIGURE 1:**
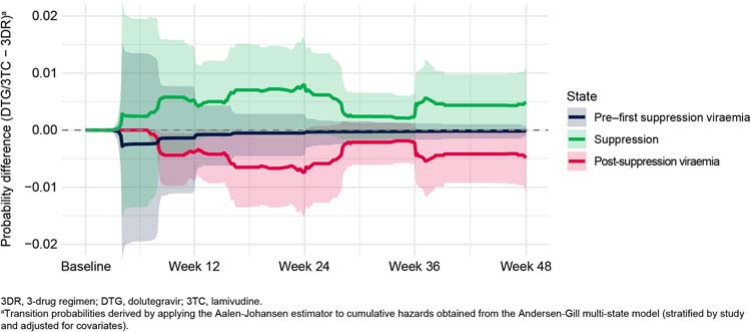
P052 | Difference in marginal state occupation probability between DTG/3TC and 3DRs through week 48.

**TABLE 1:**
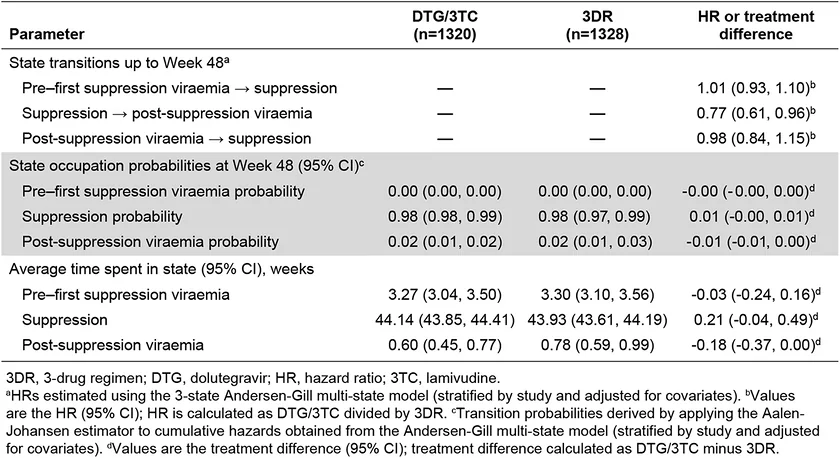
P052 | Summary of model‐based probabilities at week 48.

### P053 Comparable Efficacy of Dolutegravir/Lamivudine Versus Second‐Generation Integrase Strand Transfer Inhibitor‐Based 3‐Drug Regimens Across the Baseline Viral Load Continuum: A Participant‐Level Meta‐Analysis

Pedro Cahn ^1^, Carlos Brites^2^, Ezequiel Córdova^3^, Maria Ines Figueroa^1^, Jade Ghosn^4,5^, Belinda Ha^6^, Lijina Kaiprath^7^, Richard Grove^8^, Muhammed V. R. Murshid^7^, Jamile Ballivian^3^, Michelle Kisare^9^, Gustavo Verdier^10^, Piotr Budnik^9^, Parul Patel^6^

^1^Fundación Huesped, Buenos Aires, Argentina; ^2^Universidade Federal da Bahia, Salvador, Brazil; ^3^Fundación IDEAA, Buenos Aires, Argentina; ^4^Université Paris Cité, INSERM UMR 1137 IAME, F‐75018, Paris, France; ^5^Service des Maladies Infectieuses, Assistance Publique, Hôpitaux de Paris Nord, Hôpital Bichat‐Claude Bernard, F‐75018, Paris, France; ^6^ViiV Healthcare, Durham, NC, US; ^7^GSK, Bangalore, India; ^8^GSK, London, UK; ^9^ViiV Healthcare, London, UK; ^10^ViiV Healthcare, Montreal, Canada

**Background**: A prior meta‐analysis among individuals naïve to antiretroviral therapy (ART) demonstrated comparable week 48 (W48) efficacy of dolutegravir/lamivudine (DTG/3TC) versus DTG‐based 3‐drug regimens (3DRs) across the baseline (BL) viral load (VL) continuum. VOGUE, a head‐to‐head study of DTG/3TC versus bictegravir (BIC)/emtricitabine/tenofovir alafenamide in people naïve to ART with no BL VL restriction, adds evidence for those with very high BL VL (≥500,000 copies/mL). We conducted a participant‐level Bayesian meta‐analysis with VOGUE data to evaluate W48 efficacy and non‐inferiority of DTG/3TC versus DTG‐ and BIC‐based 3DRs irrespective of BL VL level, including among participants with BL VL ≥500,000 copies/mL.

**Methods**: Participant‐level data from the GEMINI‐1/‐2, STAT, DOLCE, D2ARLING and VOGUE trials were pooled and analysed using a Bayesian hierarchical logistic regression model, including adjustments for participant and study characteristics. W48 virologic suppression (HIV‐1 RNA <50 copies/mL; FDA Snapshot) was evaluated across BL VL as a continuous variable. Model‐estimated W48 risk differences, 95% credible intervals (CrIs) and posterior probabilities of non‐inferiority (10% margin) were estimated.

**Results**: The pooled analysis included 2314 participants, including 192 with BL VL ≥500,000 copies/mL. No treatment‐emergent resistance through W48 was reported in any study. W48 virologic suppression remained high and comparable for DTG/3TC versus DTG‐ and BIC‐based 3DRs regardless of BL VL. Over the BL VL range, treatment differences remained close to zero, with 95% CrIs including zero throughout the modelled continuum (Figure 1). Among participants with BL VL ≥500,000 copies/mL, adjusted mean W48 virologic suppression remained high at 83.6% for DTG/3TC and 81.6% for DTG‐ and BIC‐based 3DRs. The estimated mean (95% CrI) risk difference (3DRs−DTG/3TC) was −41.7% (−9.6%, 6.0%), corresponding to a 99.8% posterior probability of non‐inferiority.

**Conclusions**: This participant‐level meta‐analysis supports comparable W48 efficacy of DTG/3TC versus second‐generation integrase strand transfer inhibitor–based 3DRs across the BL VL continuum, including among people initiating ART with BL VL ≥500,000 copies/mL. No treatment‐emergent resistance was observed through W48. The expanded dataset and Bayesian modelling demonstrated a 99.8% probability of non‐inferiority for DTG/3TC versus DTG‐ and BIC‐based 3DRs using a stringent 10% margin, providing the strongest evidence to date supporting DTG/3TC efficacy among people initiating ART with BL VL ≥500,000 copies/mL.

**FIGURE 1:**
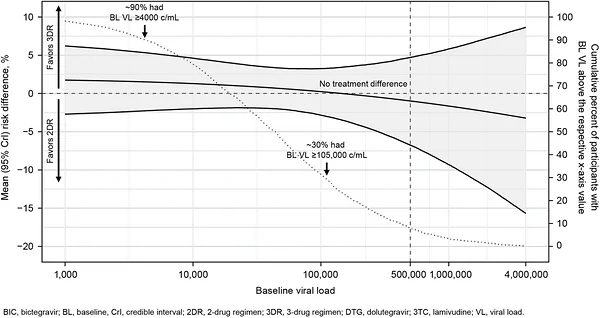
P053 | Mean (95% CrI) risk difference in week 48 virologic suppression (DTG‐ and BIC‐based 3DRs minus DTG/3TC) across the baseline viral load continuum.

### P054 Efficacy of Doravirine Plus Dolutegravir Dual Therapy in People With HIV RNA Below 50 Copies/mL: A Randomized Open‐Label Study (DORDOL)

Jesal Gohil ^1,2^, Sarah Pett^3,4^, Alan Winston^1^, Andrew Hill^5^, Roya Movahedi^2^, Darwin Matila^2^, Ebunoluwa Taiwo^3,4^, Deirdre Sally^3,4^, Merle Henderson^1^, Bryony Simmons^6^, Kaitlyn McCann^7^, Manisha Joshi^2^, Andrea Calcagno^8^, Marta Boffito^2^

^1^Department of Infectious Diseases, Imperial College London, London, UK; ^2^Department of HIV Medicine, Chelsea and Westminster Hospital, London, UK; ^3^Centre for Clinical Research in Infection & Sexual Health, Institute for Global Health, University College London, London, UK; ^4^Mortimer Market Centre, Central and North‐West London NHS Foundation Trust, London, UK; ^5^Department of Pharmacology and Therapeutics, University of Liverpool, Liverpool, UK; ^6^LSE Health, London School of Economics and Political Science, London, UK; ^7^School of Public Health, Imperial College London, London, UK; ^8^Department of Translational Medicine, University of Piemonte Orientale, Novara, Italy

**Background**: Dual antiretroviral therapy (ART) is increasingly supported as a means of reducing drug exposure and is now included in international treatment guidelines. However, prospective clinical trial data on doravirine (DOR) plus dolutegravir (DTG) remain scarce.

**Methods**: DORDOL (NCT04892654) is an ongoing open label, randomized, non‐inferiority switch study across four UK and Italian sites. Participants with HIV‐RNA ≤50 copies/mL on triple ART and no NNRTI or INSTI resistance were randomized 2:1 to DOR (300 mg) plus DTG (50 mg), both once daily (immediate switch, IS) or triple ART continuation for 48 weeks (delayed switch, DS). The primary outcome was week 48 viral load ≤50 copies/mL by FDA snapshot algorithm (non‐inferiority margin 10%) in the intention‐to‐treat population. This interim analysis covers data to week 48; the trial continues to 96 weeks.

**Results**: Between 08/09/2022 and 23/06/2025, 109 participants were randomized; data are presented on 107 (97 [91%] male; 81 [75%] White; median [IQR] age 55 [46–62]; time since HIV diagnosis 16 [10–23] years). At baseline, 63% were on tenofovir disoproxil fumarate (TDF), 7% on boosted protease inhibitors (PI/b) and 3% had major resistance mutations. Week 24 HIV RNA ≤50 copies/mL was observed in 68/70 (97%) and 36/37 (97%) participants in IS versus DS (difference −0.2%; 95% CI: −7.6 to 11.3). Week 48 HIV RNA ≤50 copies/mL was observed in 66/70 (94%) and 37/37 (100%) in IS versus DS (difference, −5.7%; 95% CI: −13.8 to 4.0) (Figure 1). Only one participant had a VL 50–200 copies/mL, at week 48 in IS; all remaining Snapshot non‐responders had missing data within the visit window. Serious adverse events (SAEs) occurred in two (3%) participants on DOR+DTG and two (5%) in DS; all SAEs were unrelated/unlikely related. Median weight gain at week 48 was 1.1 kg in IS versus 0.5 kg in DS (*p* = 0.027). Total and LDL cholesterol increased in IS to week 24 but not by week 48; HbA1c showed a small increase in IS with a between‐arm difference at week 48 (*p* = 0.018).

**Conclusions**: Dual ART with DOR and DTG showed high rates of sustained virological suppression and a comparable safety profile to triple ART in this virologically suppressed population.

**FIGURE 1:**
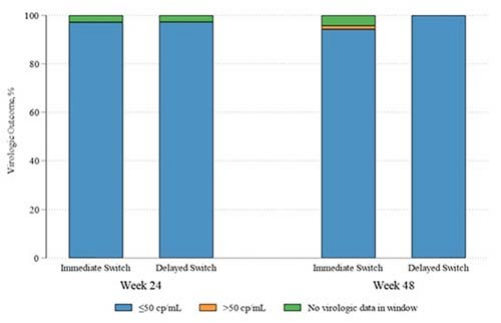
P054 | Week 24 and week 48 virological outcomes in immediate switch versus delayed switch.

### P055 Efficacy and Safety of Early Switch to Dolutegravir/Lamivudine From Integrase Inhibitors‐Based 3‐Drug Regimens in HIV‐1‐Infected Adults: 48‐Week Results of the EDOLAS Randomized Trial

Roberta Gagliardini ^1^, Alessandro Cozzi‐Lepri^2^, Laura Comi^3^, Lucia Taramasso^4^, Maria Maddalena Plazzi^1^, Sandrine Ottou^1^, Giuseppe Lapadula^5^, Silvia Nozza^6^, Annamaria Cattelan^7^, Massimiliano Fabbiani^8^, Giulia C. Marchetti^9^, Maria Vittoria Cossu^10^, Alessandro Bartoloni^11^, Marianna Menozzi^12^, Carlo Torti^13^, Federico De Zottis^1^, Rita Bellagamba^1^, Jessica Paulicelli^1^, Diego Ripamonti^3^, Paolo Bonfanti^5^, Antonio Di Biagio^4^, Valentina Mazzotta^1^, Antonella Castagna^14^, Andrea Gori^10^, Cristina Mussini^12^, Franco Maggiolo^15^, Andrea Antinori^1^, on behalf of EDOLAS Study Group

^1^INMI L. Spallanzani IRCCS, Rome, Italy; ^2^Centre for Clinical Research, Epidemiology, Modelling and Evaluation, Institute for Global Health, University College London, London, UK; ^3^Malattie Infettive, ASST Papa Giovanni XXIII, Bergamo, Italy; ^4^Infectious Diseases Unit, Policlinico San Martino Hospital—IRCCS, Genoa, Italy; Department of Health Sciences (DISSAL), University of Genoa, Genoa, Italy; ^5^IRCCS Fondazione San Gerardo, University of Milano Bicocca, Milano, Italy; ^6^Infectious Diseases Unit, IRCCS San Raffaele Scientific Institute, Vita‐Salute San Raffaele University, Milano, Italy; ^7^Infectious and Tropical Diseases Unit, Padua University Hospital, Padua, Italy; ^8^Department of Medical Biotechnologies, University of Siena, Siena, Italy; ^9^ASST Santi Paolo e Carlo, Department of Health Science, University of Milan, Milan, Italy; ^10^Department of Infectious Diseases, ASST Fatebenefratelli Sacco, Milan, Italy; ^11^Department of Experimental and Clinical Medicine, University of Florence, Florence, Italy; ^12^Infectious Diseases Unit, Azienda Ospedaliera‐Universitaria of Modena, University of Modena and Reggio Emilia, Modena, Italy; ^13^Department of Medical and Surgery Sciences, Fondazione Policlinico Universitario Agostino Gemelli IRCCS, Rome, Italy; ^14^Infectious Diseases Unit, IRCCS San Raffaele Scientific Institute, Vita‐Salute San Raffaele University, Milan, Italy; ^15^No affiliation, Fabro, Italy

**Background**: Dolutegravir (DTG)/lamivudine (3TC) has shown non‐inferiority in people with HIV (PWH) switching from three‐drug regimens after prolonged viral suppression (VS). However, the safety and efficacy of an early switch shortly after achieving VS have not been assessed. To our knowledge, this is the first randomized trial addressing this question.

**Methods**: This multicentre, randomized, open‐label, phase 3 non‐inferiority trial was conducted at 26 centres in Italy (EudraCT 2019‐004241‐32). PWH on first‐line INSTI‐based three‐drug regimens for <18 months, with HIV‐RNA ≤50 copies/mL for <12 months, no prior virological failure and no HBV infection, were randomized 1:1 to switch to DTG/3TC or continue 3DR. Randomization was stratified by duration of VS (<6 vs. ≥6 months). Enrolment was stopped before reaching the prespecified sample size by the DSMB due to slow recruitment. The primary endpoint was virological rebound (VR) at week 48 (HIV‐RNA ≥50 copies/mL, discontinuation with HIV‐RNA ≥50 or missing = failure; snapshot algorithm; ITT 4% non‐inferiority margin). Secondary on‐treatment endpoints were also examined. All binary outcomes were assessed using a Farrington–Manning approach on the risk difference (RD) scale. Secondary analyses used logistic regression to explore whether inferiority could be demonstrated.

**Results**: Of 307 screened participants, 284 were randomized (DTG/3TC *n* = 143; 3DR *n* = 141). Participants were 11% female, median age 42 years and 54.6% had zenith HIV‐RNA > 100,000 copies/mL. Median duration of suppression before randomization was 5 months (IQR 2.8–7.9) (Table 1). At week 48, VR (ITT primary analysis) occurred in 19 (13.3%) in the DTG/3TC arm and 9 (6.4%) in the 3DR arm (RD 6.9%; 95% confidence interval [CI] 1.18–12.6). Confirmed VR (2 HIV‐RNA ≥50) was seen in 6/143 (4.2%) versus 5/141 (3.6%, Figure 1). Logistic regression for inferiority yielded an odds ratio of VR ≥50 copies/mL for switching to DTG/3TC versus remaining on 3DR of 2.25 (95% CI: 0.98–5.15; *p* = 0.06).

**Conclusions**: In this trial of early switch to DTG/3TC, virologic rebound was more frequent with the switch strategy, although the number of events was small due to under‐recruitment and uncertainty remains. Additional evidence is needed to better define the safety and efficacy of treatment deintensification after early viral suppression.

**FIGURE 1:**
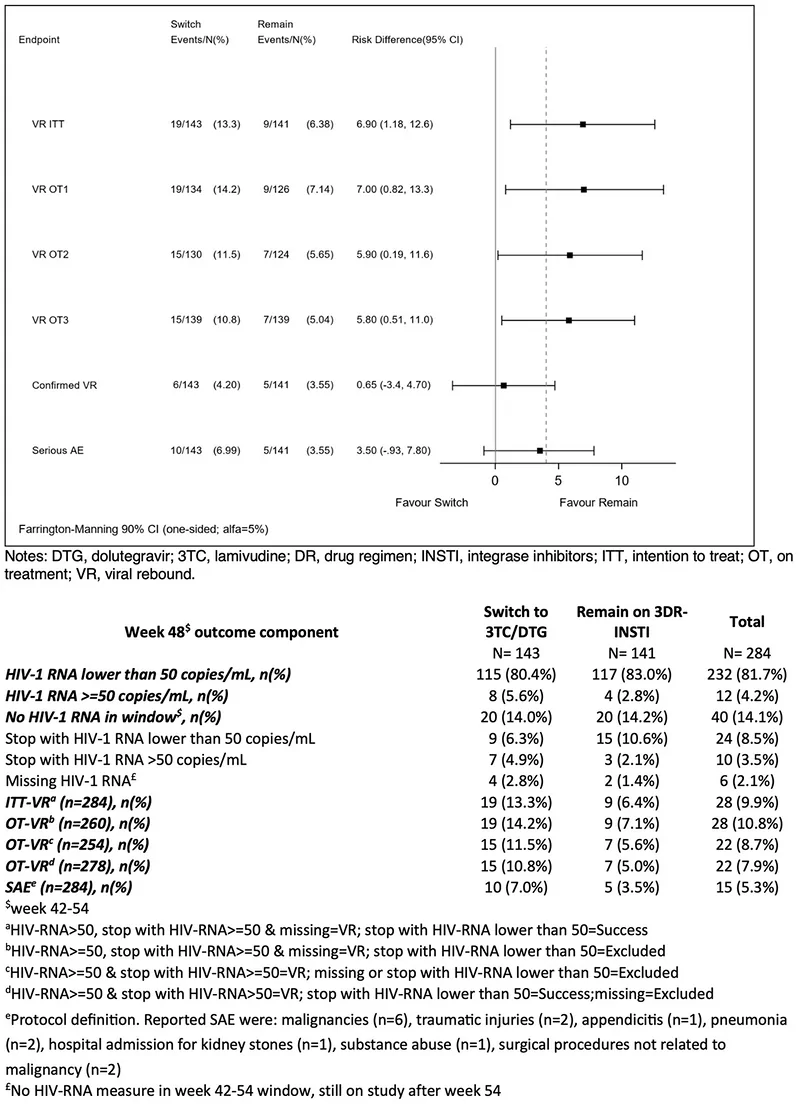
P055 | Forest plot of the primary and secondary endpoints and outcome components at week 48.

**TABLE 1:**
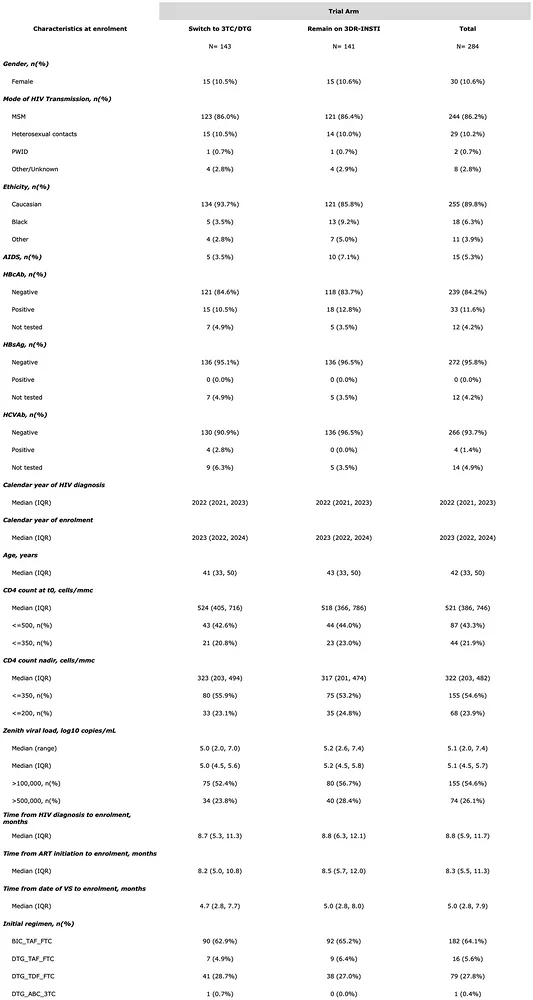
P055 | Participants characteristics (*n* = 284). Abbreviations: ABC, abacavir; ART, antiretroviral therapy; BIC, bictegravir; BL, baseline; DTG, dolutegravir; DR, drug regimen; FTC, emtricitabine; INSTI, integrase inhibitors; IQR, interquartile range; MSM, men who have sex with men; PWID, people who inject drugs; TAF, tenofovir alafenamide; TDF, tenofovir disoproxil fumarate; 3TC, lamivudine.

### P056 Understanding Treatment Experiences With Weekly Oral Islatravir/Lenacapavir in the Phase 3 ISLEND‐2 Study: Participant and Provider Interviews

Chloe Orkin ^1^, Peter J. Ruane^2^, Ploenchan Chetchotisakd^3^, Winai Ratanasuwan^4^, Mark O'Reilly^5^, Ronald Nahass^6^, Maria Lagarde^7^, Patricia Echeverría^8^, Frank Post^9^, Alejandro Arenas‐Pinto^10^, David Baker^5^, Ei Kinai^11^, Hideta Nakmura^12^, Amy E. Colson^13^, Melissa Shaugnessy^14^, Keith Dunn^15^, James Jarrett^15^, Ke Chen^14^, Arman P. Sali^15^, Devi SenGupta^15^, Cyril Llamoso^14^, Hadas Dvory‐Sobol^15^, Onyema Ogbuagu^16^, William You^17^, Claire Graziani‐Taugeron^18^

^1^SHARE Collaborative, Blizard Institute, Queen Mary University of London, London, UK; ^2^Ruane Clinical Research, Los Angeles, CA, US; ^3^Srinagarind Hospital, Khon Kaen, Thailand; ^4^Mahidol University, Bangkok, Thailand; ^5^Taylor Square Private Clinic, Sydney, Australia; ^6^IDCare, Hillsborough, NJ, US; ^7^Hospital Universitario 12 de Octubre, Madrid, Spain; ^8^Hospital Universitari Germans Trias i Pujol, Badalona, Spain; ^9^King's College Hospital NHS Foundation Trust, London, UK; ^10^Centre for Clinical Research in Infection, Institute for Global Health, University College London, London, UK; ^11^Tokyo Medical University Hospital, Tokyo, Japan; ^12^University of Rykyus Graduate School of Medicine, Okinawa, Japan; ^13^Cambridge Health Alliance, Cambridge, MA, US; ^14^Merck & Co., Inc., Rahway, NJ, US; ^15^Gilead Sciences, Inc., Foster City, CA, US; ^16^Yale School of Medicine, New Haven, CT, US; ^17^Oracle Life Sciences, Austin, TX, US; ^18^Oracle Life Sciences, Paris, France

**Background**: Islatravir/lenacapavir (ISL/LEN) is the first once‐weekly oral combination for the treatment of HIV‐1 and was shown to be non‐inferior to daily oral standard of care for maintenance of virological suppression in a phase 3 study (NCT06630299). This study also explored the participant experience, with 78% of participants reporting greater satisfaction with ISL/LEN than daily oral pills and 64% found ISL/LEN less of a burden.

**Methods**: In addition to previously reported patient‐reported outcomes (PROs), we conducted in‐depth, 60‐min, semi‐structured interviews with participants and healthcare providers (HCPs) across Australia, UK and USA. Interviews were conducted at baseline, week 12 and week 48 with 20, 17 and 18 participants and 10, 10 and 9 HCPs, respectively, to better understand patient and HCP perspectives with once‐weekly oral ISL/LEN with a loading dose. Interview topics focused on participant perspectives on ISL/LEN initiation and impact on quality of life (QoL) and HCP perspectives on ISL/LEN use. Content and thematic analysis of interview data were performed.

**Results**: At baseline, most (15/20) participants reported preferring treatments taken less frequently than daily therapy (Table 1). All participants (20/20) expected ISL/LEN to have similar or superior efficacy to their prior treatment. No participants reported issues taking the loading dose. Across weeks 12 and 48, participants consistently described positive benefits of ISL/LEN across multiple domains, including emotional and logistical QoL, treatment convenience and reduced daily reminder of HIV‐1 status (Table 1). Participants used various reminder strategies to maintain adherence, including utilizing a pill organizer, taking it with other medications, smartphone‐based app or alarms. All HCPs had positive impressions of ISL/LEN post‐baseline, specifically regarding improved tolerability and good adherence. Most HCPs felt weekly ISL/LEN would be straightforward to implement in clinical practice, because it would not require additional staff or clinic capacity.

**Conclusions**: Building on PRO findings favourable to ISL/LEN, interview participants reported positive impacts of once‐weekly oral ISL/LEN on QoL, treatment convenience and reduced daily reminder of HIV‐1 status. HCPs noted a positive impact on adherence and ease of implementation in clinical practice. These results support the potential of ISL/LEN to improve QoL among people with HIV‐1 taking daily oral therapy.

**TABLE 1:**
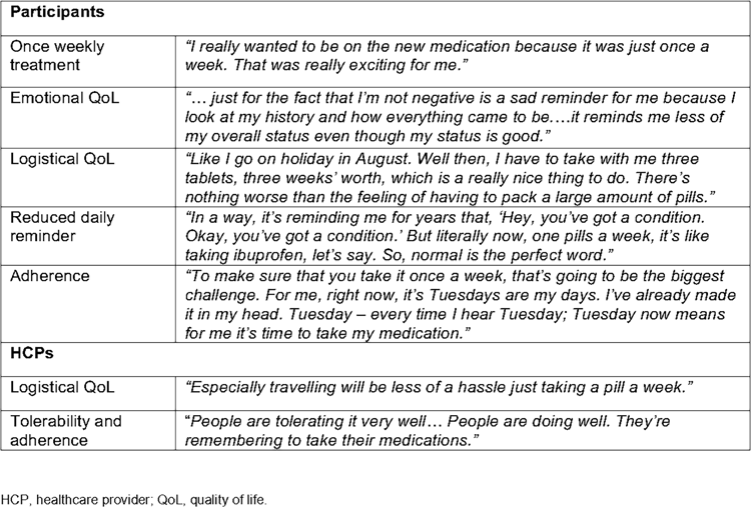
P056 | Key supporting quotes from study participants and HCPs at baseline, week 12 and week 48.

### P057 Quality of Life and Treatment Preference With Weekly ISL/LEN Versus Standard of Care: Week 48 Patient‐Reported Outcome Analysis of the Phase 3 ISLEND‐2 Study

Moti Ramgopal^1^, Peter J. Ruane^2^, Ploenchan Chetchotisakd^3^, Winai Ratanasuwan^4^, Afaaf Liberty^5^, Princy Kumar^6^, Ronald Nahass^7^, Mark O'Reilly^8^, Charlotte‐Paige Rolle^9^, William J. Towner^10^, Patricia Echeverría^11^, Maria Lagarde^12^, Alejandro Arenas‐Pinto^13^, Frank Post^14^, Georg M. Behrens^15^, Isabel Cassetti^16^, Alexandra Calmy^17^, Hideta Nakamura^18^, Amy E. Colson^19^, Hongyuan Wang^20^, Hadas Dvory‐Sobol^20^, Cyril Llamoso^21^, Devi SenGupta^20^, Yohance Whiteside^21^, James Jarrett^20^, Todd L. Saretsky^21^, Arman P. Sali^20^, Miłosz Parczewski ^22^

^1^Midway Immunology & Research Center, Fort Pierce, FL, US; ^2^Ruane Clinical Research, Los Angeles, CA, US; ^3^Srinagarind Hospital, Khon Kaen University, Khon Kaen, Thailand; ^4^Siriraj Hospital, Mahidol University, Bangkok, Thailand; ^5^University of the Witwatersrand, Johannesburg, South Africa; ^6^Georgetown University School of Medicine, Washington, DC, US; ^7^IDCare, Hillsborough, NJ, US; ^8^Taylor Square Private Clinic, Sydney, US; ^9^Orlando Immunology Center, Orlando, FL, US; ^10^Kaiser Permanente Department of Research and Evaluation, Pasadena, CA, US; ^11^Hospital Universitari Germans Trias i Pujol, Badalona, Spain; ^12^Hospital Universitario 12 de Octubre, Madrid, Spain; ^13^Institute for Global Health, University College London, London, US; ^14^King's College Hospital NHS Foundation Trust, London, US; ^15^Hannover Medical School, Hannover, Germany; ^16^Helios Salud, Buenos Aires, Argentina; ^17^Geneva University Hospitals, Geneva, Switzerland; ^18^University of Rykyus Graduate School of Medicine, Okinawa, Japan; ^19^Cambridge Health Alliance, Cambridge, MA, US; ^20^Gilead Sciences, Inc., Foster City, CA, US; ^21^Merck & Co., Inc., Rahway, NJ, US; ^22^Pomeranian Medical University, Szczecin, Poland

**Background**: In the phase 3 ISLEND‐2 study (NCT06630299), once‐weekly islatravir (ISL; nucleoside reverse transcriptase translocation inhibitor) and lenacapavir (LEN; capsid inhibitor) maintained virologic suppression for 48 weeks in 95.2% participants versus 95.5% continuing daily oral standard of care (SoC) regimens. We describe week (W) 48 patient‑reported outcomes (PROs) from ISLEND‐2.

**Methods**: In this ongoing phase 3, randomized, open‐label study, virologically suppressed participants were randomized 1:1 to receive once‐weekly fixed‐dose ISL/LEN or to continue baseline SoC for up to 96 weeks. PROs assessed at W48 included a 2‑item preference questionnaire (assesses participant preference of treatment modality [ISL/LEN group only]), HIV Patient Perspective of Regimen (HIV‐PP‐R; explores perceptions of current regimen [both groups]) and Regimen Change (HIV‐PP‐RC; explores perceptions of a switch from daily to weekly oral therapy [ISL/LEN group only]), the shortened HIV/AIDS‐Targeted Quality of Life questionnaire (HAT‐QoL; measures HIV‐specific quality of life [both groups]) and the EQ‑5D‐5L (including the visual analogue scale [EQ‐5D VAS]; measures overall health [both groups]). Results were summarized using descriptive statistics.

**Results**: Data are reported for the 314 and 312 participants who received ≥1 dose of ISL/LEN or SoC, respectively. At W48, 72.0% participants strongly preferred ISL/LEN compared with their prior treatment. Select HIV‐PP‐R items are reported in Figure 1. For HIV‐PP‐RC at W48, 81.8% participants considered ISL/LEN a more convenient lifestyle fit than their prior regimen and 78.0% were more/much more satisfied with the ISL/LEN regimen; additionally, 63.7% experienced greater treatment burden with their prior regimen than ISL/LEN. Mean (SD) changes in HAT‐QoL dimension scores from baseline to W48 were more favourable in the ISL/LEN group versus the SoC group, including, respectively: medication worries, 4 (13.8) versus −1 (12.9); disclosure worries, 7 (25.5) versus 0 (21.9). At W48, there was no between‐group difference in EQ‐5D VAS least‐squares mean change from baseline.

**Conclusions**: Most participants receiving once‐weekly oral ISL/LEN preferred ISL/LEN over their prior daily oral treatment and reported better lifestyle fit, greater treatment satisfaction, reduced treatment burden and higher HIV‐related quality of life than those continuing SoC. These results suggest that ISL/LEN may offer a treatment option for people with HIV‐1 aligned with their diverse preferences for dosing frequency.

**FIGURE 1:**
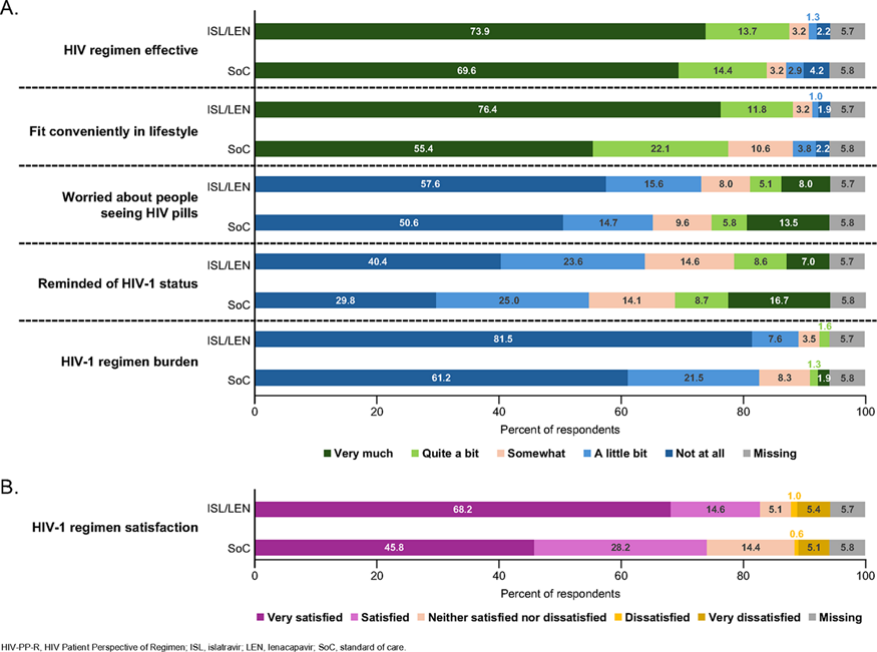
P057 | HIV‐PP‐R questionnaire at week 48 for participants receiving ISL/LEN (*n* = 314) and standard of care (*n* = 312).

### P058 Efficacy of Long‐Acting Cabotegravir Plus Rilpivirine Immediately Post‐Suppression on DTG/3TC in Prior High Viraemic, Low CD4 HIV‐1 Subgroups: 11‐Month VOLITION Results

Chinyere Okoli ^1^, Patricia De los Rios^2^, Charlotte‐Paige Rolle^3^, Paul Benson^4^, Pedro Cahn^5^, Teresa Aldamiz‐Echevarria^6^, Joaquín Bravo Urbieta^7,8^, Andrea Antinori^9^, Rosario Palacios^10^, Kai Hove^1^, Richard Grove^11^, Avishek Chatterjee^12^, Kehui Wang^13^, Samia Dakhia^1^

^1^ViiV Healthcare, London, UK; ^2^ViiV Healthcare, Montreal, QC, Canada; ^3^Orlando Immunology Center, Orlando, FL, US; ^4^Be Well Medical Center, Berkley, MI, US; ^5^Fundación Huésped, Buenos Aires, Argentina; ^6^CIBERINFEC, Madrid, Spain; ^7^Infectious Diseases Unit, Internal Medicine Department, J. M. Morales Meseguer General University Hospital, Murcia, Spain; ^8^Infectious Diseases and Artificial Intelligence Group, Biomedical Research Institute of Murcia Pascual Parrilla‐IMIB, Murcia, Spain; ^9^National Institute for Infectious Diseases Lazzaro Spallanzini IRCCS, Rome, Italy; ^10^Infectious Diseases Unit, Hospital Virgen de la Victoria, IBIMA, Málaga, Spain; ^11^GSK, London, UK; ^12^GSK CCC, Bengaluru, Karnataka, India; ^13^GSK, Collegeville, PA, US

**Background**: People with HIV‐1 initiating antiretroviral therapy (ART) with high viral load (VL; ≥100,000 copies/mL) and low CD4 count (<350 cells/mm^3^) face elevated virological failure in the first year of therapy. Dolutegravir/lamivudine (DTG/3TC) provides an effective daily oral option, whereas long‐acting cabotegravir plus rilpivirine (CAB+RPV LA), dosed 6 times a year, reduces daily dosing burden. However, CAB+RPV LA efficacy data from individuals with high baseline VL or low CD4 count remain limited, leaving a gap in guidance for this population considering an early switch.

**Methods**: VOLITION (NCT05917509), a phase 3b study, evaluated suppression with daily DTG/3TC among people naïve to ART, for 4−16 weeks, followed by participant‐determined optional switch to CAB+RPV LA post‐suppression for 11 months. Primary endpoints were time to suppression with DTG/3TC and month 11 (M11) virological suppression (HIV‐1 RNA <50 copies/mL) on CAB+RPV LA. Descriptive results of participants’ outcomes were analysed by subgroup, namely baseline VL and CD4 count.

**Results**: Of eligible participants, 85% (*n* = 129/151) chose to switch to CAB+RPV LA. Participants with baseline VL ≥100,000 copies/mL (*n* = 41) and < 100,000 copies/mL (*n* = 88) had consistent median DTG/3TC suppression time (4.29 and 4.14 weeks) and M11 CAB+RPV LA suppression rates (85%, 35/41; 89%, 78/88). Participants with CD4 count < 350 cells/mm^3^ (*n* = 53) and ≥350 cells/mm^3^ (*n* = 75) had similar DTG/3TC median suppression times (both 4.14 weeks) and M11 CAB+RPV LA suppression rates (89%, 47/53; 87%, 65/75). Median DTG/3TC suppression times were 4.86 weeks for individuals with ≥100,000 copies/mL and < 350 cells/mm^3^ (*n* = 27) and 4.14 weeks for individuals with < 100,000 copies/mL and > 350 cells/mm^3^ (*n* = 61), with M11 CAB+RPV LA suppression rates of 85% (23/27) and 87% (53/61), respectively (Figure 1). Adverse events and discontinuation rates were consistent across all subgroups. No confirmed virological failures with treatment‐emergent resistance‐associated mutations were observed in the high baseline VL, low CD4 or combined high‐risk subgroups.

**Conclusions**: Early switch to CAB+RPV LA immediately after suppression on DTG/3TC is a robust strategy for people with HIV‐1, irrespective of baseline VL or CD4 count. Virological suppression and tolerability were maintained across all subgroups, supporting early switch to CAB+RPV LA regardless of baseline immunovirological profile.

**FIGURE 1:**
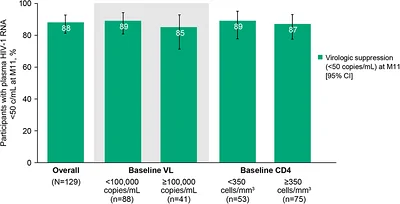
P058 | Durable M11 viral load suppression after dolutegravir/lamivudine suppression and long‐acting cabotegravir plus rilpivirine maintenance by viral load and CD4 status at baseline.

### P059 Virological Outcomes After Late CAB+RPV LA Injections in Newly Suppressed People With HIV‐1: 11‐Month Results From the VOLITION Study

Chinyere Okoli ^1^, Patricia De los Rios^2^, Charlotte‐Paige Rolle^3^, Laurence Slama^4,5^, Andrea Antinori^6^, Guillermo Cuevas Tascon^7^, Jose Rafael Granados Monzon^8^, Michele Hernandez Cabrera^9,10^, Celia Jonsson‐Oldenbüttel^11^, Kai Hove^1^, Richard Grove^12^, Avishek Chatterjee^13^, Kehui Wang^14^, Samia Dakhia^1^

^1^ViiV Healthcare, London, UK; ^2^ViiV Healthcare, Montreal, QC, Canada; ^3^Orlando Immunology Center, Orlando, FL, US; ^4^Service d'immuno‐infectiologie, Hôtel Dieu, APHP, Paris, France; ^5^Université Paris Cité and Université Sorbonne Paris Nord, Inserm, INRAE, Center for Research in Epidemiology and Statistics (CRESS), F‐75004, Paris, France; ^6^National Institute for Infectious Diseases Lazzaro Spallanzini IRCCS, Rome, Italy; ^7^Department of Internal Medicine, Hospital Universitario Infanta Leonor, Madrid, Spain; ^8^Infectious Diseases Unit, University Hospital of Gran Canaria Dr. Negrín, Las Palmas de Gran Canaria, Spain; ^9^Unidad de Enfermedades Infecciosas y Medicina Tropical, Hospital Universitario Insular de Gran Canaria, Las Palmas de Gran Canaria, Spain; ^10^Departamento de Ciencias Médicas y Quirúrgicas, Universidad de Las Palmas de Gran Canaria, Las Palmas de Gran Canaria, Spain; ^11^MVZ CARE, Munich, Germany; ^12^GSK, London, UK; ^13^GSK, Bengaluru, Karnataka, India; ^14^GSK, Collegeville, PA, US

**Background**: Long‐acting cabotegravir plus rilpivirine (CAB+RPV LA) is an injectable regimen associated with high adherence and efficacy. However, some scheduling variability is inevitable and concerns about late injection impact on virological outcomes remain a potential barrier to broader implementation. Clinical outcomes data on infrequent CAB+RPV LA dosing delays in individuals who are recently virologically suppressed are needed to support shared decision‐making.

**Methods**: VOLITION (NCT05917509) is a phase 3b study on participants achieving virological suppression on dolutegravir/lamivudine (DTG/3TC) followed by a participant‐determined optional switch to CAB+RPV LA administered 6 times a year or to continue DTG/3TC. This subgroup analysis included participants who chose to switch to CAB+RPV LA after achieving initial virological suppression on oral DTG/3TC. Participants were categorized according to injection adherence: “early/on‐time” (no injections > 7 days late, no missed injections, no oral bridging) or “late” (≥1 injection > 7 days late, ≥1 missed injection or oral bridging therapy required). Virological snapshot outcomes (HIV‐1 RNA <50 copies/mL), confirmed virological failure (CVF) and safety outcomes were evaluated at month 11 (M11).

**Results**: Adherence to injection schedule was high; of 729 injection visits from months 1 to 11, 88% (643/729) occurred on time (within the ± 7‐day window) or earlier (1%, 9/729). Overall, 42% of participants (54/129) experienced ≥1 “late” injection during the 11‐month period. Delays were infrequent, with a median of 9 days beyond the scheduled injection date (IQR 8.0–10.0 days). Despite these occasional delays, 96% (*n* = 52/54) of these participants maintained virological suppression (<50 copies/mL) at M11, showing no adverse impact relative to 81% (*n* = 61/75) in the “early/on time” subgroup (Figure 1). No CVFs occurred in the “late” subgroup, compared with 4 (3%) in the “early/on‐time” subgroup. Adverse event and treatment discontinuation rates were similar between subgroups. One participant in the “late” subgroup required oral bridging (DTG/3TC).

**Conclusions**: Short, infrequent delays in CAB+RPV LA administration beyond the ± 7‐day window were not associated with reduced virological suppression at month 11, with no CVFs or notable safety outcomes observed. These findings are consistent with prior data on CAB+RPV LA pharmacology but do not diminish scheduling adherence importance; real‐world evidence in broader populations is warranted.

**FIGURE 1:**
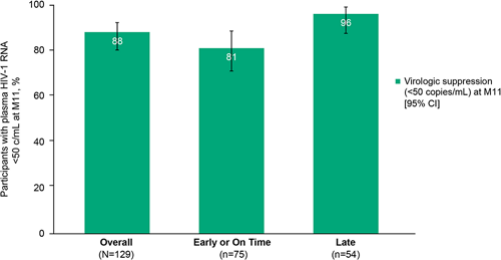
P059 | Durable month 11 virological suppression with CAB+RPV LA by adherence subgroup.

### P060 Resistance Analysis at Week 48 of Daily Doravirine/Islatravir (100/0.25 mg) Versus Bictegravir/Emtricitabine/Tenofovir Alafenamide in a Blinded Phase 3 Study in Adults Initiating Treatment for HIV‐1

Tracy L. Diamond, Uchechukwu Nwoke, Tarak Patel, Yayun Xu, Stephanie Klopfer, Mary Vesnesky, Luisa M. Stamm, Michelle C. Fox, Mary Pisculli, Ernest Asante‐Appiah

MRL, Merck & Co., Inc., Rahway, NJ, US

**Background**: Doravirine/islatravir (DOR/ISL [100/0.25 mg]) is a once‐daily single tablet regimen of DOR, a non‐nucleoside reverse transcriptase inhibitor (NNRTI) and ISL, a nucleoside reverse transcriptase translocation inhibitor (NRTTI) available in the US and Japan for HIV‐1 treatment in virologically suppressed people. DOR/ISL demonstrated non‐inferior efficacy compared to bictegravir/emtricitabine/tenofovir alafenamide (BIC/FTC/TAF) at week (W) 48 in a blinded phase 3 study (P053: NCT05705349) in treatment‐naïve adults with HIV‐1 RNA ≥500 copies/mL. Here, we assessed prevalence and impact of baseline resistance‐associated mutations (RAMs) on virologic outcomes.

**Methods**: Resistance analysis at screening was used to identify exclusionary RAMs for DOR or BIC/FTC/TAF. Viraemia was monitored at study visits using the Cobas HIV assay. Post‐baseline resistance analysis was performed for participants with 2 consecutive HIV‐1 RNA ≥200 copies/mL 3–5 weeks apart with virological rebound or incomplete virological response or for participants discontinued with HIV‐1 RNA ≥200 copies/mL.

**Results**: Of 756 participants screened, 21 were ineligible due to pre‐existing resistance (13 associated with DOR [5 with V106M, 2 each with V108I, Y188L, H221Y or P225H], 8 associated with BIC/FTC/TAF [7 with M184V in reverse transcriptase, 1 with R263K in integrase] and 6 due to resistance assay failure). Of the 536 treated participants (DOR/ISL: 269; BIC/FTC/TAF: 267), similar proportions of participants achieved HIV‐1 RNA <50 copies/mL (virologic suppression) at W48 in both groups irrespective of non‐exclusionary baseline RAMs. Initial virological suppression was achieved for 236 DOR/ISL (88%) and 251 BIC/FTC/TAF (94%) participants by W24; by W36, 259 (96%) and 259 (97%) participants in each group, respectively; and by W48, 260 (97%) in each group. Among 32 DOR/ISL participants with baseline major NNRTI RAMs (including K101E, K103N and E138A/K), 30 achieved virologic suppression by W48, 1 was lost‐to‐follow‐up at W4 and 1 discontinued with treatment‐emergent resistance. Overall, 2 participants had treatment‐emergent resistance to DOR with reduced susceptibility to ISL; both had baseline RAMs and pre‐treatment HIV‐1 RNA > 1,000,000 copies/mL and discontinued by W24.

**Conclusions**: DOR/ISL demonstrated similar efficacy to BIC/FTC/TAF at achieving HIV‐1 RNA <50 copies/mL through W48 in adults initiating treatment for HIV‐1 including in the presence of baseline RAMs.

### P061 Long‐Term Outcomes of Cabotegravir Plus Rilpivirine Long‐Acting Every 2 Months in a Real‑World Setting: Effectiveness, Adherence and Patient‐Reported Outcomes From People With HIV‐1 in the CARLOS Study 3 Years Post‐Switch

Sebastian Noe^1^, Celia Jonsson‐Oldenbüttel^1^, Christoph Wyen^2^, Jan Borch^3^, Kevin Ummard‐Berger^4^, Christiane Cordes^5^, Stefan Scholten^6^, Niels Schübel^7^, Alexa Pfauth^8^, Bernd Westermayer^9^, Jenny Scherzer ^8^

^1^MVZ München am Goetheplatz, Munich, Germany; ^2^Praxis am Ebertplatz, Köln, Germany; ^3^Praxis Goldstein, Berlin, Germany; ^4^UBN/Praxis, Berlin, Germany; ^5^Infektionsmedizin Berlin‐Friedrichshain, MVZ MDI Limbach Berlin, Berlin, Germany; ^6^Praxis Hohenstaufenring, Cologne, Germany; ^7^MVZ Klinikum Osnabrück, Osnabrück, Germany; ^8^ViiV Healthcare, Munich, Germany; ^9^GSK, Munich, Germany

**Background**: Injectable long‐acting (LA) cabotegravir (CAB) plus rilpivirine (RPV) offers an alternative mode of administration with less frequent dosing than daily oral antiretroviral therapy (ART). CARLOS is a non‐interventional, 3‐year, multicentre study including people with HIV‐1 on suppressive daily oral ART who switched to CAB+RPV LA dosed every 2 months in routine care in Germany. Here, we describe outcomes at year 3.

**Methods**: Year 3 effectiveness outcomes included virologic suppression (HIV‐1 RNA <50 copies/mL), virologic non‐response (HIV‐1 RNA ≥50 copies/mL) and protocol‐defined virologic failure (PDVF; 2 consecutive HIV‐1 RNA ≥200 copies/mL or a single HIV‐1 RNA ≥200 copies/mL followed by discontinuation for any reason). Additional outcomes included adherence to the injection window and change in HIV Treatment Satisfaction Questionnaire status version (HIVTSQs) score. Analyses were performed in the intention‐to‐treat population.

**Results**: In total, 351 people with HIV‐1 were included in the year 3 analysis. Overall, 94% (*n* = 4935/5246) of CAB+RPV LA injections were administered before/within the ± 7‐day injection window and 6% (*n* = 311/5246) occurred late. At year 3, 70% (*n* = 246/351) of participants were virologically suppressed and 26% (*n* = 91/351) discontinued for any reason while being virologically suppressed. Seven participants (2%) met PDVF through 24 months; no additional cases of PDVF occurred through year 3 (Figure 1, see also for full virologic outcomes). There was a statistically significant mean change in treatment satisfaction (+6.1; *p* < 0.001) that also met the threshold for a minimum clinically important difference (MCID; greater than half of the baseline standard deviation [10.1]) among participants completing both baseline (mean score [standard deviation], 54.8/66 [10.1]) and year 3 (mean score [SD], 60.9/66 [6.1]) HIVTSQs (*n* = 214/351).

**Conclusions**: In this real‐world study, CAB+RPV LA maintained high rates of virologic suppression 3 years after switch, with a low virologic failure rate. There was a low rate of discontinuation due to tolerability reasons and most injections were administered before or within the ± 7‐day injection window. Despite high baseline scores on oral ART, the significant increase in treatment satisfaction for participants remaining on CAB+RPV LA that was previously reported at 12 and 24 months after switch was maintained 3 years post switch.

**FIGURE 1:**
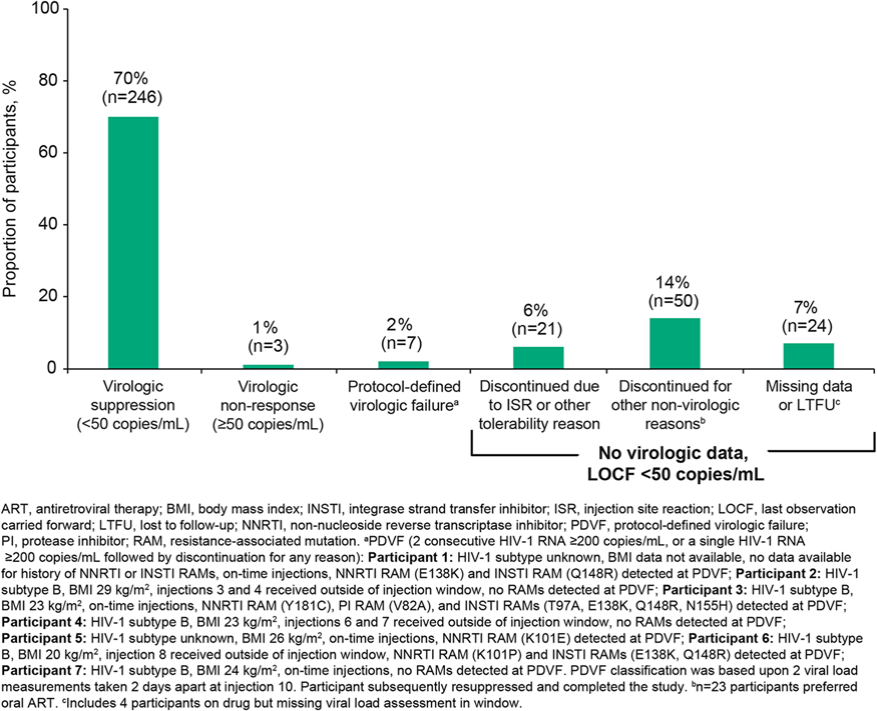
P061 | Intention‐to‐treat virologic outcomes at year 3.

### P062 Characterizing Viraemic Events in the ARTISTRY‐1 and ARTISTRY‐2 Trials

Peter J. Ruane^1^, Eric Meissner^2^, Edwin DeJesus^3^, Dai Watanabe^4^, Ying Guo^5^, Kwad Mponponsuo^6^, Jairo M. Montezuma‐Rusca^6^, Peter Sklar^6^, Chloe Orkin ^7^

^1^Ruane Clinical Research Group, Los Angeles, CA, US; ^2^Department of Medicine, Medical University of South Carolina, Charleston, SC, US; ^3^Orlando Immunology Center, Orlando, FL, US; ^4^AIDS Medical Center, NHO Osaka National Hospital, Osaka, Japan; ^5^Biostatistics, Gilead Sciences, Inc., Foster City, CA, US; ^6^Clinical Development, Gilead Sciences, Inc., Foster City, CA, US; ^7^SHARE Collaborative, Blizard Institute, Faculty of Medicine and Dentistry, Queen Mary University of London, London, UK

**Background**: In ARTISTRY‐1 (NCT05502341) and ARTISTRY‐2 (NCT06333808), switching to a single‐tablet regimen of bictegravir/lenacapavir (BIC/LEN) was non‐inferior to continuing complex antiretroviral regimens (CRs) and bictegravir/emtricitabine/tenofovir alafenamide (B/F/TAF), respectively, in people with HIV (PWH) who were virologically suppressed. Here, we present data from participants in ARTISTRY‐1 and ARTISTRY‐2 who experienced viraemia post‐treatment.

**Methods**: In phase 3 of the open‐label ARTISTRY‐1 trial, PWH who were virologically suppressed on CRs were randomized to switch to once‐daily oral BIC/LEN or continue on CRs. In the double‐blind ARTISTRY‐2 trial, PWH virologically suppressed on B/F/TAF were randomized to switch to BIC/LEN or continue on B/F/TAF. Randomized participants who took ≥1 dose of study treatment were included in this analysis. Viraemia was defined as having at least one HIV‐1 RNA result ≥50 copies/mL at post‐baseline visits after being suppressed on BIC/LEN or comparator. Viraemia with HIV‐1 RNA ≥200 copies/mL was also summarized. Although not trial mandated, all participants could change regimen at investigator's discretion or per local guidelines.

**Results**: In ARTISTRY‐1, 557 PWH were randomized to switch to BIC/LEN (*n* = 371) or continue on CRs (*n* = 186). In ARTISTRY‐2, 574 PWH were randomized to switch to BIC/LEN (*n* = 383) or continue on B/F/TAF (*n* = 191). Overall, 51 (9%) participants in ARTISTRY‐1 and 46 (8%) participants in ARTISTRY‐2 had HIV‐1‐RNA ≥50 copies/mL post‐baseline (Table 1). In total, 21 (4%) participants in ARTISTRY‐1 and 18 (3%) participants in ARTISTRY‐2 experienced HIV‐1‐RNA ≥200 copies/mL. Most participants who experienced viraemic events resuppressed to HIV‐1‐RNA <50 copies/mL without regimen change (Table 1).

**Conclusions**: The majority of participants had no viraemic events in ARTISTRY‐1 and ARTISTRY‐2. Most events that occurred resolved while continuing study drug.

**TABLE 1:**
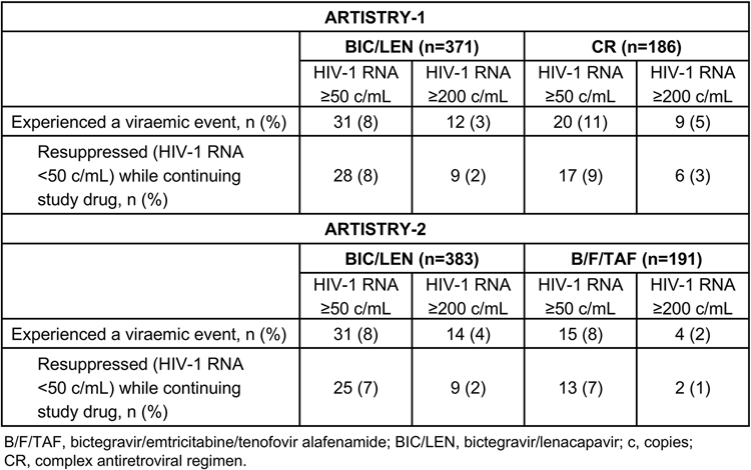
P062 | Viraemic events in ARTISTRY‐1 and ARTISTRY‐2.

### P063 Patient‐Reported Outcomes With Doravirine/Islatravir (100/0.25 mg) Once Daily Versus Bictegravir/Emtricitabine/Tenofovir Alafenamide in Virologically Suppressed Adults Living With HIV‐1: Phase 3 Results Through Week 96

Gary Sinclair ^1^, Amy E. Colson^2,3^, Moti Ramgopal^4^, Craig A. Dietz^5^, Anthony LaMarca^6^, Alexandra W. Dretler^7^, Chloe Orkin^8,9^, Philip Bright^10^, Andrew Carr^11^, Takuma Shirasaka^12^, Chenguang Zhang^13^, Michelle C. Fox^13^, Mengchun Li^13^, Girish Prajapati^13^

^1^Prism Health North Texas, Dallas, TX, US; ^2^Community Resource Initiative, Boston, MA, US; ^3^Cambridge Health Alliance, Cambridge, MA, US; ^4^Midway Immunology and Research Center, Fort Pierce, FL, US; ^5^Kansas City CARE Health Center, Kansas City, MO, US; ^6^Therafirst Medical Center, Fort Lauderdale, FL, US; ^7^Metro Infectious Disease Consultants, Decatur, GA, US; ^8^Queen Mary UPniversity of London, London, UK; ^9^Barts Health NHS Trust, London, UK; ^10^North Bristol NHS Trust, Bristol, UK; ^11^ St Vincent's Hospital, Sydney, Australia; ^12^NHO Osaka National Hospital, Osaka, Japan; ^13^Merck & Co., Inc., Rahway, NJ, US

**Background**: In a phase 3 study (MK‐8591A‐052; NCT05630755) in people living with HIV‐1 (PLWH), switching to doravirine/islatravir (DOR/ISL 100/0.25 mg) oral once daily was non‐inferior for efficacy to continuing bictegravir/emtricitabine/tenofovir alafenamide (BIC/FTC/TAF) at week (W) 48; high rates of virologic suppression were maintained and DOR/ISL was well tolerated through W96. We report patient‐reported outcomes (PROs), including health status, treatment satisfaction and symptom burden.

**Methods**: Adults with HIV‐1 RNA <50 copies/mL for ≥3 months on BIC/FTC/TAF were randomized (2:1) to switch to DOR/ISL (100/0.25 mg) or continue BIC/FTC/TAF. PROs were assessed at baseline and W4, W12, W48 and W96 using EQ‐5D‐5L (EQ VAS), HIV Treatment Satisfaction Questionnaire‐status version (HIVTSQs) and HIV Symptom Index. Analyses were exploratory and conducted using descriptive statistics; within‐group changes from baseline and between‐group differences are summarized with nominal 95% CIs.

**Results**: Overall, 342 participants switched to DOR/ISL and 171 continued BIC/FTC/TAF. Baseline demographics were similar between groups. PRO completion and compliance were high (> 88%, with minimal missingness) and comparable across groups through W96. At baseline, participants reported high overall health status and treatment satisfaction, which were maintained across groups (Table 1). Mean changes in EQ VAS scores were minimal at W48 and W96. HIVTSQs scores remained stable, with minor changes from baseline across groups. The most common bothersome symptoms at baseline were fatigue/loss of energy, changes in body composition/weight gain and difficulty sleeping. Bothersome symptom burden was low at baseline with minimal changes through W48 and W96 across groups. No meaningful between‐group differences in percent change from baseline were observed for any bothersome symptoms at W48 (Figure 1); W96 findings were generally similar. Differences for individual symptoms at W96 were small and inconsistent across time points.

**Conclusions**: In virologically suppressed PLWH, PROs following switch to DOR/ISL were consistent with those observed with continued BIC/FTC/TAF through W96. Health status and treatment satisfaction remained high, while symptom burden remained low over time. Outcomes were generally comparable between groups, with no consistent or clinically meaningful differences identified. These findings support the tolerability of DOR/ISL and a positive treatment experience among PLWH as a switch option.

**FIGURE 1:**
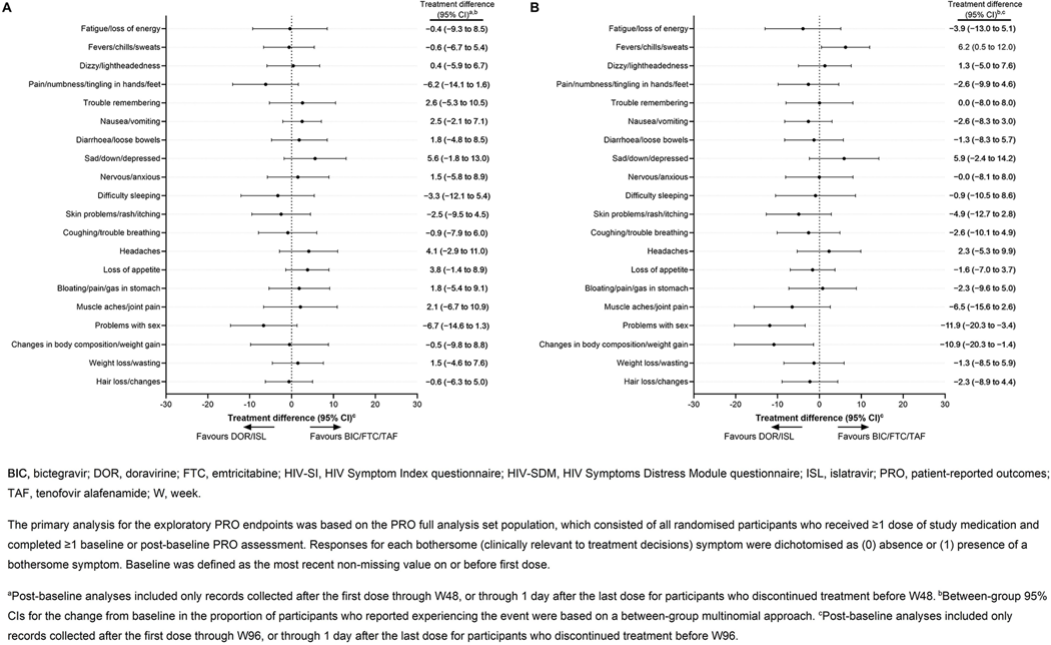
P063 | Percentage of participants with HIV‐SI/HIV‐SDM bothersome symptoms at (A) W48 and (B) W96.

**TABLE 1:**
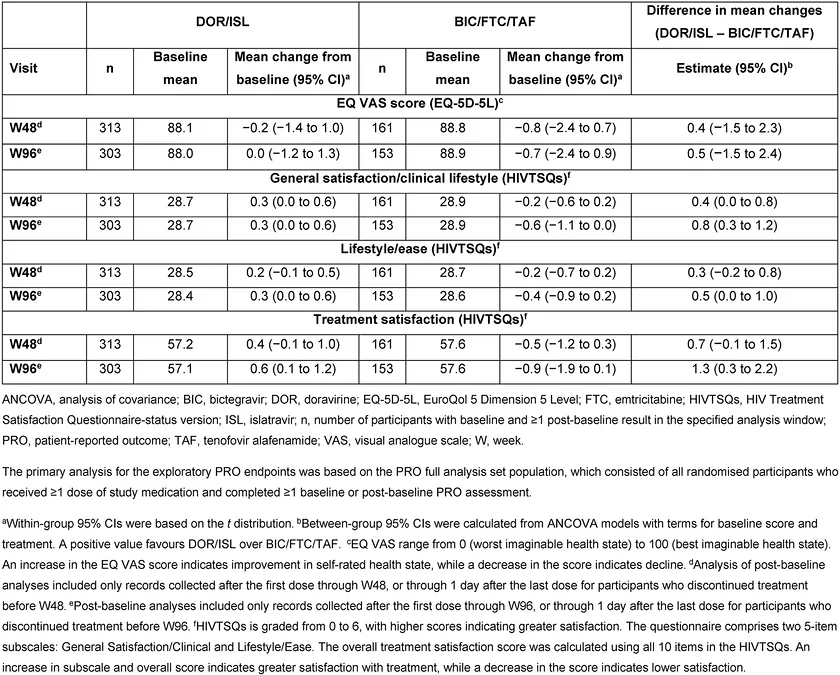
P063 | Mean change from baseline in EQ VAS and HIVTSQs scores.

### P064 Comparative Efficacy and Safety of Doravirine/Islatravir Versus Contemporary ART Regimens in Virologically Suppressed People Living With HIV‐1 at 48 Weeks: A Systematic Review and Network Meta‐Analysis

Alex Karagiannis ^1^, Girish Prajapati^2^, Abdel Hmissi^1^, Kate Young^3^, Nick Halfpenny^4^, Megha Garg^5^, Sean Fleming^6^

^1^MSD, Zurich, Switzerland; ^2^Merck & Co., Inc., Rahway, NJ, US; ^3^Merck & Co., Inc., West Point, PA, US; ^4^OPEN Health HEOR & Market Access, Rotterdam, Netherlands; ^5^OPEN Health HEOR & Market Access, London, UK; ^6^Merck & Co., Inc., Philadelphia, PA, US

**Background**: Doravirine/islatravir (DOR/ISL 100/0.25 mg) demonstrated non‐inferior efficacy and comparable safety to bictegravir/emtricitabine/tenofovir alafenamide (BIC/FTC/TAF) in phase 3 studies. We conducted a systematic literature review (SLR) and network meta‐analysis (NMA) to evaluate the relative efficacy and safety of DOR/ISL versus other antiretroviral therapy (ART) regimens in virologically suppressed people living with HIV (PLWH).

**Methods**: An SLR (22 April 2026) identified 105 publications reporting 62 randomized clinical studies in virologically suppressed PLWH on stable ART. Outcomes of interest for this NMA included virologic outcomes at week 48 (HIV RNA ≥50 and < 50 copies/mL), any adverse events (AEs), drug‐related AEs (DRAEs) and discontinuation through week 48. NMA feasibility assessment based on study similarities and a strict common comparator definition identified a network anchored on BIC/FTC/TAF linking DOR/ISL, dolutegravir/lamivudine (DTG/3TC), bictegravir/lenacapavir (BIC/LEN) and cabotegravir/rilpivirine (CAB/RPV). A fixed‐effects Bayesian NMA was used to estimate the relative treatment effect as odds ratios (OR) and 95% credible intervals (CrI) for all outcomes.

**Results**: Four randomized studies in virologically suppressed PLWH informed the network: MK‐8591A‐052 (DOR/ISL), DYAD (DTG/3TC), ARTISTRY‐2 (BIC/LEN) and SOLAR (CAB/RPV), each versus BIC/FTC/TAF; SOLAR and DYAD were open‐label studies. No differences were observed in virologic outcomes between DOR/ISL and BIC/FTC/TAF, DTG/3TC, BIC/LEN or CAB/RPV (Table 1). For safety outcomes, DOR/ISL was associated with lower odds of any AEs versus CAB/RPV (OR, 0.44; 95% CrI 0.25–0.80) and lower odds of DRAEs versus CAB/RPV (OR, 0.00*; 95% CrI 0.00*–0.02; *rounded, actual estimates < 0.01) and versus DTG/3TC (OR, 0.12; 95% CrI 0.02–0.56). AEs and DRAEs include injection site reactions for CAB/RPV. No differences were observed for serious AEs, serious DRAEs or discontinuations across regimens.

**Conclusions**: This NMA of randomized switch studies in virologically suppressed PLWH demonstrated that DOR/ISL has similar efficacy and safety to DTG/3TC, BIC/LEN and CAB/RPV. These findings support DOR/ISL as a relevant switch option in the absence of direct comparative evidence, although it is based on a small evidence network and wide credible intervals.

**TABLE 1:**
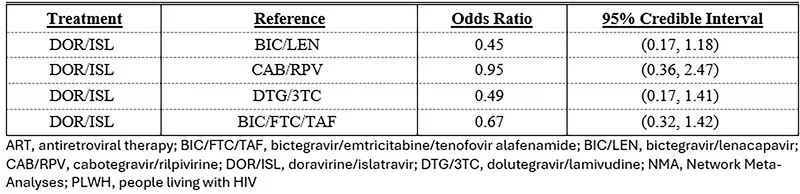
P064 | NMA results for Week 48 HIV‐1 RNA <50 copies/mL: DOR/ISL versus other ART regimens in virologically suppressed PLWH.

### P065 Abstract Withdrawn

### P066 Home‐Based Versus Hospital‐Based Long‐Acting Cabotegravir/Rilpivirine: Results From the Randomized ALADDIN Trial

Silvia Nozza ^1,2^, Maria Vittoria Bufali^3^, Angelo Roberto Raccagni^1,2^, Nicolò Capra^1^, Riccardo Lolatto^1^, Valentina Lovato^1^, Flavia Badalucco^1^, Elena Bruzzesi^1^, Tommaso Clemente^1^, Vincenzo Spagnuolo^1,2^, Antonella Castagna^1,2^, Oriana Ciani^3^

^1^Infectious Diseases Unit, IRCCS San Raffaele Scientific Institute, Milan, Italy; ^2^Vita‐Salute San Raffaele University, Milan, Italy; ^3^Bocconi University, SDA Bocconi School of Management, Milan, Italy

**Background**: Although long‐acting cabotegravir plus rilpivirine (CAB+RPV LA) offers an alternative to daily oral ART, the need for hospital administration may limit its scalability. Aim was to compare patient‐reported implementation outcomes (PROs) of home‐based versus hospital‐based CAB+RPV LA administration.

**Methods**: ALADDIN (Antiviral Long‐Acting Drugs Landing in people living with HIV; NCT06468995) was a single‐centre, prospective, randomized, open‐label, implementation‐effectiveness hybrid type III trial enrolling people with HIV receiving oral ART with HIV‐RNA <50 copies/mL at IRCCS San Raffaele Scientific Institute, Milan, Italy. Participants were randomized (1:1) to hospital‐based or home‐based CAB+RPV LA administration for 11 months. The primary endpoint was the proportion of people with a score ≥4 in Feasibility of Implementation Measure (FIM), Acceptability of Intervention Measure (AIM), Intervention Appropriateness Measure (IAM) scales and qualitative insights at month 11 (M11). Secondary endpoints were treatment discontinuations and PROs (EQ‐5D, HAT‐QoL, HIV‐TSQ) at M11. Intention‐to‐treat (ITT) and per‐protocol (PP) analyses are presented. The study was partially supported by ViiV Healthcare.

**Results**: A total of 110 participants were screened and 100 randomized to home‐based (*n* = 50) or hospital‐based (*n* = 50) CAB+RPV LA administration. Baseline demographic, immunological and treatment characteristics were balanced between study arms (Table 1). Implementation outcomes at M11 were consistently high at ITT and PP analyses. Overall, 88.9%, 90% and 85.6% of participants achieved AIM, IAM and FIM scores ≥4, without differences between groups at PP. Acceptability was higher at ITT analysis in the hospital‐based group (Figure 1). Qualitative insights highlighted that home‐based administration was associated with privacy, comfort and convenience, while hospital‐based with clinical safety and organizational support, with positive care experience and staff relationships across both settings. Secondary PROs at M11 highlighted high treatment satisfaction (HIV‐TSQ ≥50 = 75, 83.3%) and quality of life (EQ‐5D VAS = 85.0 [70.0; 92.0]; HAT‐QoL provider trust = 100 [75; 100]), without differences between groups at PP. Discontinuation occurred in 10 participants (7/10 for injection‐site reaction), more frequently in the home‐based arm (8 vs. 2).

**Conclusions**: CAB+RPV LA administration was highly feasible, acceptable and appropriate in both settings, with a better trend of acceptability in hospital. Additional benefits of home‐based administration included privacy, comfort and convenience.

**FIGURE 1:**
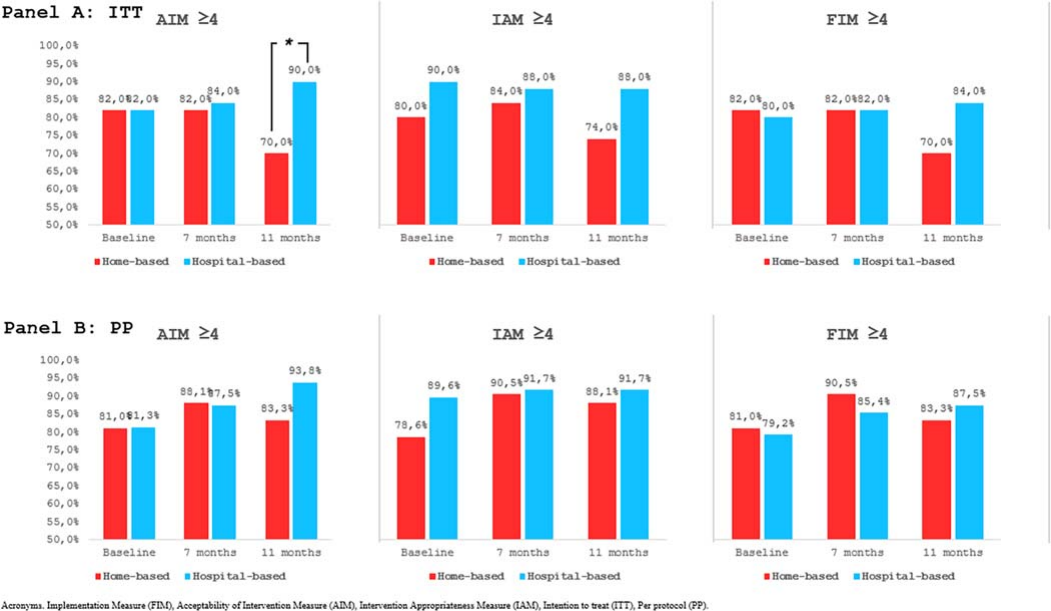
P066 | Proportion of people receiving CAB/RPV with an AIM, IAM and FIM score ≥4 in home‐based or hospital‐based settings.

**TABLE 1:**
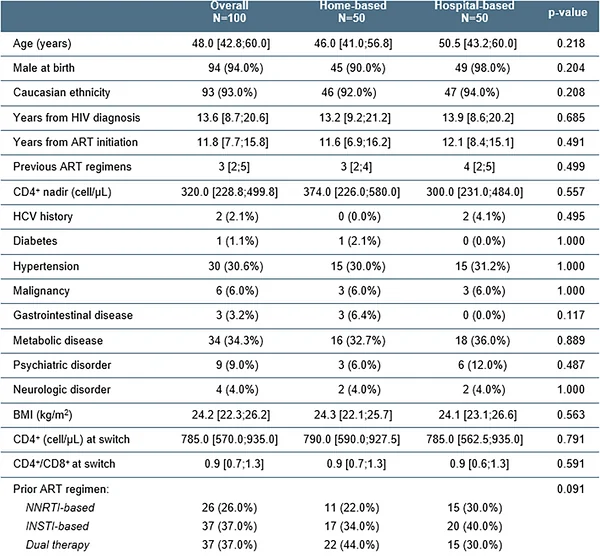
P066 | Baseline demographic and clinical characteristics of participants receiving CAB+RPV LA in the home‐based and hospital‐based settings.

### P067 Case Study of a Patient on Subcutaneous Long‐Acting Cabotegravir and Rilpivirine

Arno Beer ^1^, Stefan Lung^2^, Iris Kremser^3^, Alexander Egle^1^

^1^Universitätsklinik für Innere Medizin III, mit Hämatologie, internistischer Onkologie, Hämostaseologie, Infektiologie, Rheumatologie und Onkologisches Zentrum, Salzburg, Austria; ^2^Institute of Virology Innsbruck, Innsbruck, Austria; ^3^UI f. Med‐Chem. Labordiagnostik (Zentrallabor‐LKH), Salzburg, Austria

**Background**: Injectable cabotegravir (CAB) and rilpivirine (RPV) is the only and well‐established long‐acting (LA) antiretroviral therapy (ART) in HIV treatment. LA intramuscular (IM) injections are often well tolerated and injection site reactions (ISRs) are few. Still, there is a small number of patients who cannot tolerate the ISRs mainly due to preexisting conditions (e.g. lumboischialgia). Nevertheless, data exist for subcutaneous (SC) CAB/RPV, mainly from phase 2 and 3 clinical trials. This mode of delivery was not pursued further by ViiV Healthcare after phase 3 due to injection site reactions (ISRs), for example nodules, redness and swelling.

**Methods**: We report a 56‐year‐old male patient, who was diagnosed with HIV in 2006. He was started on antiretroviral therapy (ART) in 2016 (F, T and DTG). This treatment was highly effective and well tolerated; however, when LA‐ART became available, the patient was switched to CAB/RPV in July of 2023 (patient's wish), which was equally effective; however, over the course of 18 months, ISRs became increasingly prominent, ultimately prompting consideration of a switch back to oral ART. After discussing the available data on SC administration and the off‐label nature of this approach, the patient agreed to switch to SC every 2 month (q2m) with the prerequisite of close therapeutic drug monitoring (TDM).

**Results**: SC administration of LA‐CAB/RPV was started at the end of 2024 and remains ongoing. Close viral load (VL) monitoring confirmed continuous complete virologic suppression. Furthermore, TDM demonstrated not only excellent peak and trough levels, but also very good interim levels for both CAB and RPV always staying well above the respective PA‐IC90 (protein‐adjusted 90% inhibitory concentration). Concerning viral control, no proviral DNA was detected in peripheral blood or in the gastric and anal mucosa sampled during routine endoscopy. Moreover, direct Oxford Nanopore sequencing (SQK‐NBD114.96) of the genomic DNA identified no proviral reads. ISRs were much better tolerable when compared to i.m. injections with a predictable course of some redness, pain and swelling for a few days.

**Conclusions**: We conclude that in selected patients who cannot tolerate IM injections, SC CAB/RPV LA‐ART administration could be an option provided close TDM, VL and ISR monitoring is available.

### P068 Two‐Year Follow‐Up of People Living With HIV Receiving Long‐Acting CAB/RPV: Impact on Wellbeing?

Anna Carraro ^1^, Vanessa Bertocchi^2^, Raffaella Marocco^3^, Lorenzo Ansaldo^3^, Vito Speciale^4^, Giulia Mancarella^3^, Cosmo Del Borgo^3,4^, Miriam Lichtner^1,5^

^1^Infectious Diseases Unit, Sant'Andrea University Hospital, Sapienza University of Rome, Rome, Italy; ^2^Sapienza University of Rome, Rome, Italy; ^3^Infectious Diseases Unit, S. M. Goretti Hospital, Polo Pontino Sapienza University of Rome, Latina, Italy; ^4^Department of Public Health and Infectious Diseases, Sapienza University of Rome, Rome, Italy; ^5^Department of Neuroscience, Mental Health and Sense Organs, Sapienza University of Rome, Rome, Italy

**Background**: Registration studies of CAB/RPV have shown how much people starting this type of treatment appreciate long‐acting injectable therapy; furthermore, long‐acting therapy was developed with the aim of improving the patient−treatment relationship, but this impact has not yet been demonstrated.

**Methods**: This is a real‐world, multicentre study (Latina AIDS Reference Centre and the Infectious Diseases Clinic at Sant'Andrea University Hospital—Rome, Italy) in which we administered several patient‐reported outcomes (PROs) to people living with HIV (PLWH) on long‐acting cabotegravir/rilpivirine (LA‐CAB/RPV) at time 0 (at the start of CAB/RPV treatment), after 28 weeks, after 52 weeks and after 100 weeks. Here, we focus on the HIV Stigma Scale, the Patient Health Questionnaire‐9 (PHQ‐9; mental health assessment), the Generalized Anxiety Disorder‐7 (GAD‐7) and the International Index of Erectile Function Questionnaire (IIEF‐5).

**Results**: 85 patients have started CAB/RPV, but only 41% completed the questionnaire via their own mobile phone. Adherence was 41% and was higher among males. The HSS confirms that stigma is still very much present among these individuals, highlighting that, in particular, the domains “disclosure concerns” and “concern with public attitudes” had the greatest impact on stigma (*p* < 0.0001). HSS scores do not change over time; however, when analysing the score for each item, a reduction in the score relating to disclosure is observed (*p* = 0.07). The GAD‐7 and PHQ‐9 scales indicated high levels of anxiety and depression in our cohort; 42% and 49% of respondents, respectively, had scores consistent with these disorders. No reduction in these scores was observed up to T100. The results of each questionnaire correlate with one another (*r* = 0.71, *p* = 0.0002). Focusing on people with penises, the IIEF‐5 score also remained unchanged. The IIEF‐2 scores are not correlated with the perceived stigma score.

**Conclusions**: Although it is very common to hear people praising this new treatment option, it does not actually seem to do much to reduce their anxiety or the distress caused by stigma. Long‐acting therapy is a first step and can help, but it is not enough: social and cultural initiatives need to be put in place.

### P069 Abstract Withdrawn

## Treatment Strategies: Real‐World and Implementation Science Studies, Oral and Injectable Therapy

### P070 High Virologic Suppression and Adherence With Rare Virologic Failure in Treatment Experienced Virologically Suppressed Individuals on Cabotegravir+Rilpivirine: Results From the COMBINE‐2 European Cohort

Anton Pozniak^1^, Gayathri Sridhar ^2^, Lambert Assoumou^3,4^, Ana González‐Cordón^5^, Eugènia Negredo^6^, Nathalie De Castro^7^, Myriam Diemer^8^, Leigh Ragone^2^, Ajay Shah^9^, Jean van Wyk^10^, Vani Vannappagari^2^

^1^Chelsea and Westminster NHS Foundation, London, UK; ^2^ViiV Healthcare, Durham, NC, US; ^3^The European Treatment Network For HIV, Hepatitis and Global Infectious Diseases (NEAT‐ID), London, UK; ^4^Sorbonne Université, INSERM, Institut Pierre Louis d'Épidémiologie et de Santé Publique, Paris, France; ^5^Hospital Clinic Barcelona, IDIBAPS, Barcelona, Spain; ^6^Hospital Germans Trias I Pujol, Fundació Lluita contra les Infeccions, Barcelona, Spain; ^7^Hospital Saint Louis, Paris, France; ^8^Hospital Lariboisiere, Paris, France; ^9^GlaxoSmithKline, London, UK; ^10^ViiV Healthcare, London, UK

**Background**: Cabotegravir (CAB)+rilpivirine (RPV) is the first complete long‐acting (LA) regimen for people with HIV who are virologically suppressed (viral load [VL] < 50 copies/mL) on a stable antiretroviral regimen, with no prior virologic failure or known resistance to NNRTI or INSTI classes. Clinical outcomes were evaluated among individuals initiating CAB+RPV LA in routine clinical practice across Europe.

**Methods**: Treatment‐experienced, virologically suppressed adults initiating CAB+RPV LA were enrolled at NEAT‐ID Network sites in seven European countries between December 2020 and September 2025. VLs were assessed from the first injection through discontinuation or time of analysis. Confirmed virologic failure (CVF) was defined as either 2 consecutive VLs ≥200 copies/mL or 1 VL ≥200 copies/mL followed by discontinuation.

**Results**: Among 1022 participants initiating CAB+RPV LA, median age was 45 years (IQR 37–53), 85% were male, 64% were white Caucasian and 10% had BMI ≥30 kg/m^2^ at initiation. Adherence was high, with 94% of participants receiving injections on time, 4% delayed doses and 1% missed injections. Overall, 885 individuals (87%) remained on CAB+RPV LA with a median follow‐up of 23 months (IQR 21.2, 23.6). The most common reasons for discontinuation included individual decision (3.3%), loss to follow‐up (1.8%) and injection site reaction (1.4%). Among the 1007 participants with follow‐up VL data, 98% had a last VL < 50 copies/mL and 91% had sustained virologic suppression with all follow‐up VLs < 50 copies/mL. At 6, 12 and 24 months, 96%, 94% and 88% of participants, respectively, had VL < 50 copies/mL at the time point (Table 1). Participants with BMI < 30 and ≥30 kg/m^2^ had similar virologic suppression (98% with last VL < 50 copies/mL). Seven participants (0.7%) experienced CVF. All were adherent to the regimen, three had NNRTI and/or INSTI resistance mutations at failure and all were suppressed after switching to a PI (*n* = 5) or INSTI‐based (*n* = 2) regimen.

**Conclusions**: In this real‐world European cohort, CAB+RPV LA achieved high rates of virologic suppression, strong adherence and low CVF. Virologic outcomes were consistent across BMI categories and all individuals with CVF resuppressed after switching regimens. These findings reinforce CAB+RPV LA as a highly effective and durable treatment strategy in routine clinical practice.

**TABLE 1:**
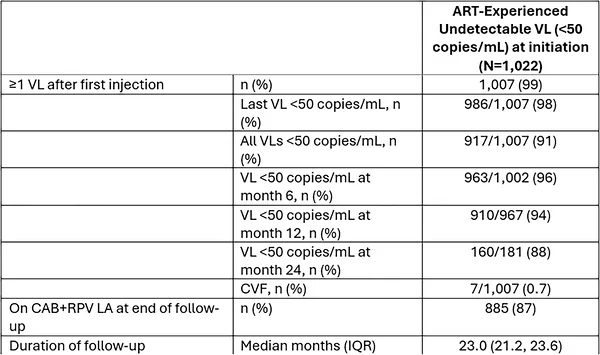
P070 | Virologic outcomes among individuals initiating CAB+RPV LA regimen.

### P071 Real‐World Data of Effectiveness of Dolutegravir Plus Lamivudine in Treatment‐Naïve People Living With HIV: The ANA‐DOLU Cohort Study

Ferit Kuşcu ^1^, Aslıhan Candevir^1^, Ongun Yeniceri^1^, Mustafa Kemal Çelen^2^, Süheyla Kömür^1^, Tuba Damar Çakırca^3^, Damla Ertürk^1^, Çiğdem Mermutluoğlu^2^, Ayşe Seza İnal^1^, Mehmet Cabalak^4^, İlkay Karaoğlan^5^, Elif Sahin Horasan^6^, Ayşe Özlem Mete^5^, Tugce Simsek Bozok^6^, Behice Kurtaran^7^, Yeşim Taşova^1^

^1^Cukurova University Faculty of Medicine, Dept. of Infectious Diseases and Clinical Microbiology, Adana, Türkiye; ^2^Dicle University Faculty of Medicine, Dept. of Infectious Diseases and Clinical Microbiology, Diyarbakir, Türkiye; ^3^Harran University Faculty of Medicine, Dept. of Infectious Diseases and Clinical Microbiology, Sanliurfa, Türkiye; ^4^Mustafa Kemal University Faculty of Medicine, Dept. of Infectious Diseases and Clinical Microbiology, Hatay, Türkiye; ^5^Gaziantep University Faculty of Medicine, Dept. of Infectious Diseases and Clinical Microbiology, Gaziantep, Türkiye; ^6^Mersin University Faculty of Medicine, Dept. of Infectious Diseases and Clinical Microbiology, Mersin, Türkiye; ^7^Acıbadem University Adana Hospital, Dept. of Infectious Diseases and Clinical Microbiology, Adana, Türkiye

**Background**: Dolutegravir plus lamivudine (DTG+3TC) dual therapy is a recommended first‐line regimen for antiretroviral‐naïve people living with HIV (PLWH), offering efficacy comparable to triple‐drug combinations while minimizing long‐term toxicity. However, real‐world evidence in patients with very high viral loads and advanced immunosuppression remains limited.

**Methods**: We conducted a multicentre, retrospective cohort study across six centres, including 149 ART‐naïve PLWH who initiated DTG+3TC. The primary endpoint was virological suppression (HIV‐1 RNA <50 copies/mL) at months 1, 3, 6 and 12. Secondary endpoints included CD4+ T‐cell recovery, CD4/CD8 ratio, metabolic and renal outcomes.

**Results**: The median age of the patients was 37 years; 89.3% were male. At baseline, median HIV‐1 RNA was 74,900 copies/mL (IQR 25,689–278,900) and median CD4+ T cell counts was 330 cells/mm^3^ (IQR 200–466); 24.8% had CD4+ T cell counts < 200 cells/mm^3^. Overall virological suppression rates were 48.5%, 78.9%, 87.4% and 93.8% at months 1, 3, 6 and 12, respectively. Patients with baseline HIV RNA ≥500,000 copies/mL showed delayed early suppression (5.9% at month 1), but reached 94.4% by month 12 (Figure 1). Similarly, suppression was initially lower among patients with CD4+ T cell counts < 200 cells/mm^3^ (20.0% vs. 53.6% at month 1, *p* = 0.017), yet all achieved suppression at month 12. No patients discontinued treatment due to adverse events. Small increases in BMI and HDL cholesterol and a modest decline in eGFR were observed.

**Conclusions**: The ANA‐DOLU cohort study provides compelling real‐world evidence that the DTG+3TC two‐drug regimen achieves rapid, durable virological suppression and robust immunological recovery in ART‐naïve PLWH. Most importantly, the regimen demonstrated remarkable long‐term efficacy in historically challenging subgroups, including late presenters with advanced immunosuppression (CD4+ < 200 cells/mm^3^) and those with very high baseline viraemia (≥500,000 copies/mL). Although early virological suppression was slightly delayed in these high‐risk groups, the 12‐month outcomes equalled those of patients with lower baseline viral loads and higher CD4+ counts. Furthermore, DTG+3TC demonstrated excellent clinical tolerability. These findings extend the external validity of pivotal clinical trials to real‐world, middle‐income settings, firmly supporting DTG+3TC as an optimal, safe and highly effective first‐line therapy across the full spectrum of HIV‐1 disease stages.

**FIGURE 1:**
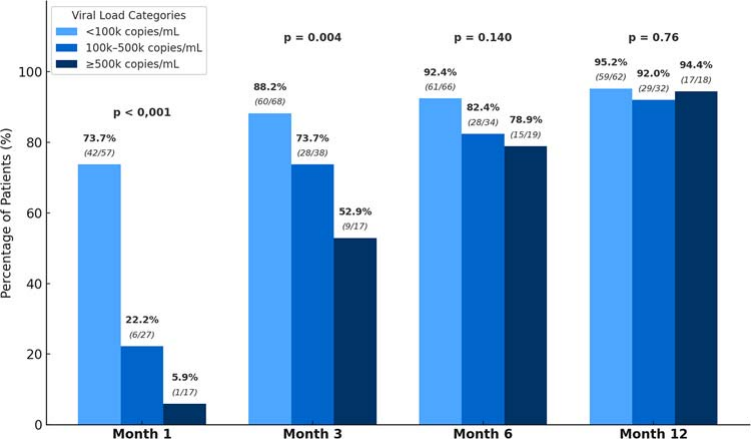
P071 | Virological suppression rates (<50 copies/mL) stratified by baseline HIV RNA levels at months 1, 3, 6 and 12.

### P072 Real‐World Virological Effectiveness and Hepatitis B Safety of Switching to Long‐Acting Injectable Cabotegravir and Rilpivirine (LANTERN): A Multicentre Observational Cohort Study in Asia

Chien‐Yu Cheng ^1^, Hsin‐Yen Ku^2^, Jui‐Hsuan Yang^1^, Shu‐Yuan Lee^1^, Cheng‐Pin Chen^1^, Shu‐Hsing Cheng^1^, Jui‐Hsing Wang^3^, Chia‐Jui Yang^4^, Tsung‐Chia Chen^5^, Tung‐Che Hung^6^, Andrew Po‐Liang Chen^7^, Linghua Li^8^, Wang‐Huei Sheng^9^, on behalf of LANTERN Study Group

^1^Department of Infectious Diseases, Taoyuan General Hospital, Ministry of Health and Welfare, Taoyuan City, Taiwan; ^2^Department of Nursing, Taoyuan General Hospital, Ministry of Health and Welfare, Taoyuan City, Taiwan; ^3^Division of Infectious Disease, Department of Internal Medicine, Taichung Tzu Chi Hospital, Buddhist Tzu Chi Medical Foundation, Taichung City, Taiwan; ^4^Department of Internal Medicine, Far Eastern Memorial Hospital, New Taipei City, Taiwan; ^5^Department of Infectious Diseases, Taichung Hospital, Ministry of Health and Welfare, Taichung City, Taiwan; ^6^Department of Infectious Diseases, Ditmanson Foundation Chiayi Christian Hospital, Chiayi City, Taiwan; ^7^Division of Infectious Diseases, Department of Medicine, Taipei Veterans General Hospital, Taipei City, Taiwan; ^8^Department of Infectious Diseases, Guangzhou Medical University Affiliated Eighth Hospital, Guangzhou City, China; ^9^Department of Infectious Diseases, National Taiwan University Hospital, Taipei City, Taiwan

**Background**: Long‐acting cabotegravir plus rilpivirine (CAB/RPV‐LA) has been available in Taiwan and China for intramuscular administration every 2 months since May 2024. This is the first study to evaluate its virological effectiveness and hepatitis B outcomes in this population [1].

**Methods**: We conducted a prospective, multicentre, observational cohort study of people living with HIV (PLWH) who switched to CAB/RPV‐LA at eight HIV care‐designated hospitals in Taiwan and China between May 2024 and May 2026 [2,3]. The primary endpoint was conversion from HBsAg‐negative to HBsAg‐positive or detectable HBV DNA, among anti‐HBc‐positive/HBsAg‐negative participants. The secondary endpoint was the proportion maintaining HIV RNA < 50 copies/mL.

**Results**: Among 394 patients, median age was 40.0 years (mean 41.6 ± 10.0), 95.4% were male and 86.0% reported MSM as the transmission risk factor. Median baseline CD4+ count was 701 cells/µL (Q1–Q3: 522–909); 51% had vaccine‐induced immunity, 25% had prior HBV exposure (anti‐HBc‐positive, including 6% isolated anti‐HBc) and median BMI was 24.7 kg/m^2^ (Q1–Q3: 22.5–26.7). Prior regimens were mainly 3TC/DTG (43.4%) and BIC/FTC/TAF (30.7%). After 13 months, no HBsAg seroconversion occurred; one patient had a transient HBsAg blip (13 IU/mL) at month 13 that resolved within 2 months (Table 1). Virological suppression (HIV‐RNA <50 copies/mL) was maintained in 97.2% of patients, with a low discontinuation rate of 4.3% (17/394) (Figure 1). Discontinuation causes included injection site reactions/pain (*n* = 6, 1.5%), systemic adverse events/intolerance (*n* = 5, 1.3%, including fever, insomnia and one unrelated sudden cardiac death), transfer of care (*n* = 5, 1.3%) and virological failure (*n* = 1, 0.25%).

**Conclusions**: CAB/RPV‐LA appears safe in patients with prior HBV exposure, including isolated anti‐HBc and demonstrated sustained virological efficacy over 13 months in real‐world use. Comprehensive HBV screening and serological monitoring remain essential.

**References**

1. Foncillas A, Ambrosioni J, Sempere A, et al. Hepatitis B outcomes after switching to long‐acting cabotegravir/rilpivirine in people with HIV: reactivation, incident infection and liver safety across diverse serological profiles. Clin Infect Dis. 2026 Jan 14:ciag023.

2. Jongen VW, Wit FWNM, Boyd A, et al. Effectiveness of bi‐monthly long‐acting injectable cabotegravir and rilpivirine as maintenance treatment for HIV‐1 in the Netherlands: results from the Dutch ATHENA national observational cohort. Lancet HIV. 2025;12(1):e40‐e50.

3. Orkin C, Oka S, Philibert P, et al. Long‐acting cabotegravir plus rilpivirine for treatment in adults with HIV‐1 infection: 96‐week results of the randomised, open‐label, phase 3 FLAIR study. Lancet HIV. 2021;8(4):e185‐e196.

**FIGURE 1:**
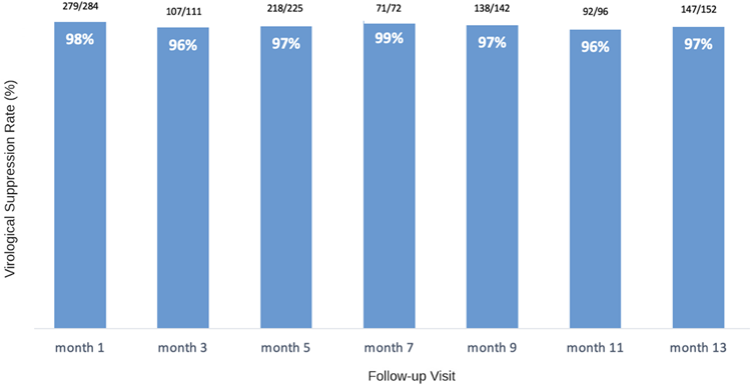
P072 | The proportion of virological suppression over time.

**TABLE 1:**
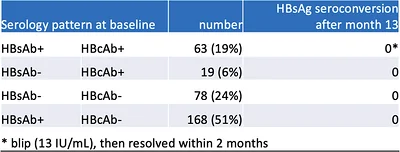
P072 | The serology pattern of hepatitis B and the outcome of safety over 13 months.

### P073 Polypharmacy in People With HIV Over 50 Compared to a Matched Control Population and Drug Interactions With ART: Results of the French Polypills Study

Clotilde Allavena ^1^, Rocco Collarino^2^, Philippe Mariot^3^, Haydar Benachir^3^, Stephanie Dousa^3^, Aurelien Carlicchi^4^, Augustin‐Martin Gaigne^4^, Patrice Muret^5^

^1^CHU de Nantes, Nantes, France; ^2^CHU Kremlin‐Bicêtre, Le Kremlin‐Bicêtre, France; ^3^MSD, Puteaux, France; ^4^CEMKA, Bourg‐la‐Reine, France; ^5^CHU de Besançon, Besançon, France

**Background**: Polypharmacy has emerged as a challenge in the clinical management of ageing people with HIV (PWH), particularly due to the accumulation of comorbidities. The objectives of this study were to describe the polypharmacy in PWH over 50 years and compare it to a control population without HIV and assess the risk of drug/drug interactions (DDIs) between antiretroviral therapy (ART) and concomitant medications.

**Methods**: Polypills draws upon anonymized records from a 2% representative sample of the French national health database (SNDS), which includes > 90% of the French population. PWH were identified based on ICD‐10 HIV‐diagnoses, HIV‐specific laboratory tests and/or prescription of antiretrovirals. Controls are persons without HIV matched to PWH (1:3) according to year of birth and gender. Drugs and comorbidities were assessed between 1 January 2024 and 31 December 2024 in PWH and controls. DDIs were identified using the Liverpool HIV Drug Interactions database.

**Results**: 1914 PWH and 5742 controls were identified (mean age 60.9 years; SD 7.6), 70.6% were male. Polypharmacy was significantly more prevalent in PWH than in controls at all thresholds (*p* < 0.0001): ≥3 non‐ART: 56.4% versus 44.7%; ≥4 non‐ART: 42.2% versus 32.5%; ≥5 non‐ART: 29.6% versus 23.6%. The main non‐ART therapeutic classes among PWH and controls are reported in Table 1. The most frequent (>5%) comorbidities among PWH and more prevalent than in controls were dyslipidaemia (39.3% for PWH/30.0% for controls), hypertension (25.2%/18.2%), ischemic heart disease (11.2%/8.2%), cancers (10.8%/8.4%), disorders due to the use of psychoactive substances (9.9%/4.8%), depression (9.8%/5.7%), anxiety (6.6%/3.6%) and chronic obstructive pulmonary disease (6.1%/2.9%). Obesity (6.8%/7.3%) and diabetes (12.2%/12.5%) were frequent but not significantly different between the 2 groups. Among the 1645 PWH on ART and non‐ART comedications (85.9%), 49.9% (*N* = 821) had at least one potential DDI (see Figure 1): moderate risk for DDI: 28.4% (*N* = 467); major risk for DDI: 34.6% (*N* = 569); contraindicated interaction: 9.7% (*N* = 160).

**Conclusions**: Older PWH were significantly more exposed to polypharmacy than matched controls. About 10% of treated PWH were exposed to contraindicated interaction, underlining the importance of systematic medication review and DDI monitoring in this ageing population.

**FIGURE 1:**
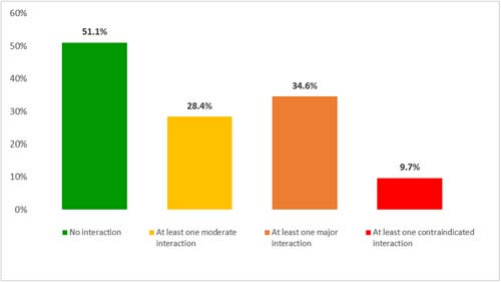
P073 | Proportions of PWH over 50‐year‐old with potential DDI between ART and non‐ART according to the Liverpool HIV Drug Interactions database.

**TABLE 1:**
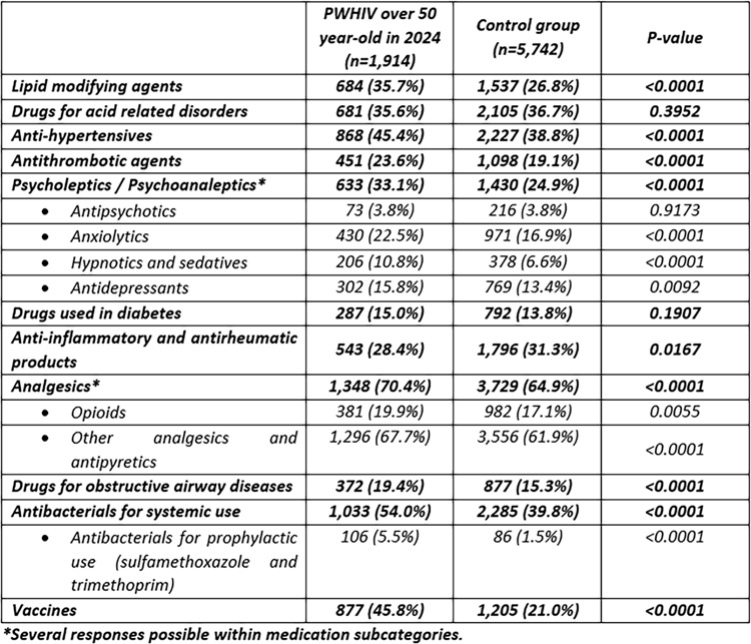
P073 | Concomitant medications among PWH over 50‐year‐old and control group.

### P074 Cabotegravir Plus Rilpivirine in Real‐World Evidence: A Systematic Literature Review of Outcomes Following Virological Failure, Including Resistance and Subsequent Treatment Options

Inês Augusto ^1^, John Thornhill^2^, Ian Jacob^2^, Patricia De los Rios^3^, Bryn Jones^2^

^1^ViiV Healthcare, Algés, Portugal; ^2^ViiV Healthcare, London, UK; ^3^ViiV Healthcare, Montreal, Canada

**Background**: Cabotegravir (CAB) + rilpivirine (RPV) is the first and only complete long‐acting (LA) injectable regimen approved for people with HIV‐1 who are virologically suppressed. While phase 3 trials report switching to standardized protease inhibitor (PI)‐based regimens after CAB+RPV LA virological failure (VF), real‐world evidence (RWE) suggests treatments are more diverse, including second‐generation integrase strand transfer inhibitors.

**Methods**: An ongoing systematic literature review of RWE studies for CAB+RPV LA was performed (PRISMA guidelines; Embase, PubMed, Cochrane Library and international congresses; January 2020–October 2025). Publications reporting VF and related outcomes from discrete cohorts in ≥100 people virologically suppressed (<50 c/mL) at CAB+RPV LA initiation were synthesized descriptively to characterize patterns across a large and diverse RWE base. Across publications, VF definitions varied based on viral load thresholds and single versus confirmatory testing, reflecting diverse clinical practices and study designs.

**Results**: A total of 432 publications, representing 121 unique cohorts and 39,434 people with HIV‐1, reported CAB+RPV LA use. Of these, 20 publications described VF outcomes among 16,172 people who were virologically suppressed at CAB+RPV LA initiation and are included in this analysis. Virological failure was described in 183 individuals, with rates of 1%–3% across individual cohorts; 4 publications (*n* = 778) reported 0 VF cases. Ten publications (*n* = 10,220) reported genotypic resistance after VF; integrase inhibitor (INI) and non‐nucleoside reverse transcriptase inhibitor resistance‐associated mutations were described, respectively, in 43 and 44 individuals, with publication‐reported rates of < 1%–1%. Post‐VF ART regimens were described for 54 individuals, with most switching to single‐tablet regimens (89%, 48/54); over one‐third (37%, 20/54) transitioned to INI‐based oral therapy or continued CAB+RPV LA. Post‐VF treatments by ART class from phase 3 trials and this RWE analysis are shown in Figure 1. Re‐suppression outcomes following regimen switch were available for 35 individuals across 5 publications, with publication‐reported data cut‐off rates of 60%–100%. Table 1 provides a comprehensive summary of results.

**Conclusions**: Real‐world evidence consistently supports low VF rates with CAB+RPV LA and low incidence of INI resistance at VF. After VF, various treatment strategies, including INI‐based therapies, were used in clinical practice, most commonly single‐tablet regimens, demonstrating the availability of multiple effective treatment pathways.

**FIGURE 1:**
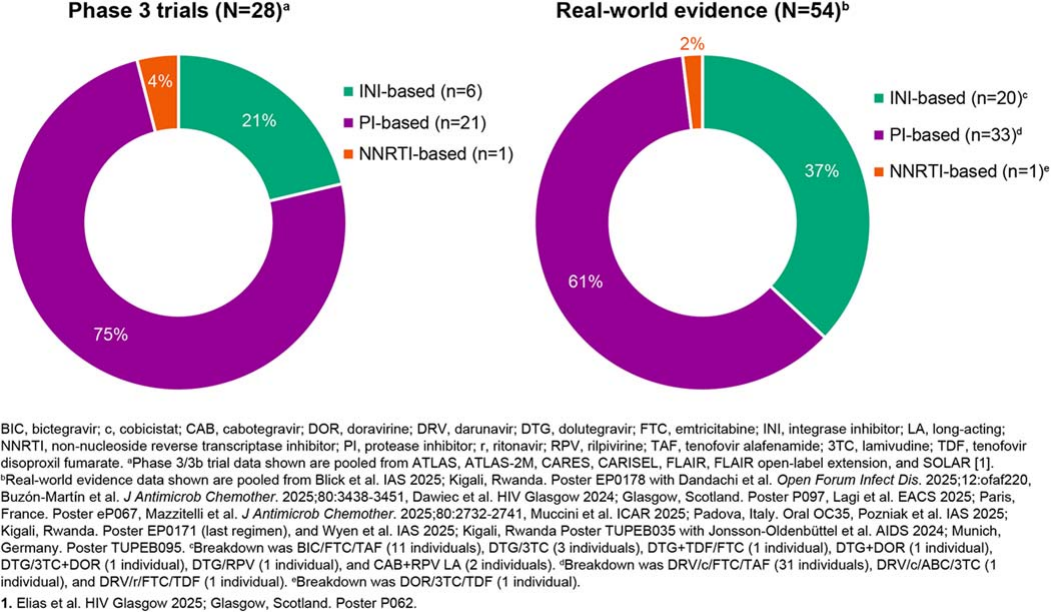
P074 | Post‐virological failure regimen utilization in phase 3 trials and real‐world evidence studies.

**TABLE 1:**
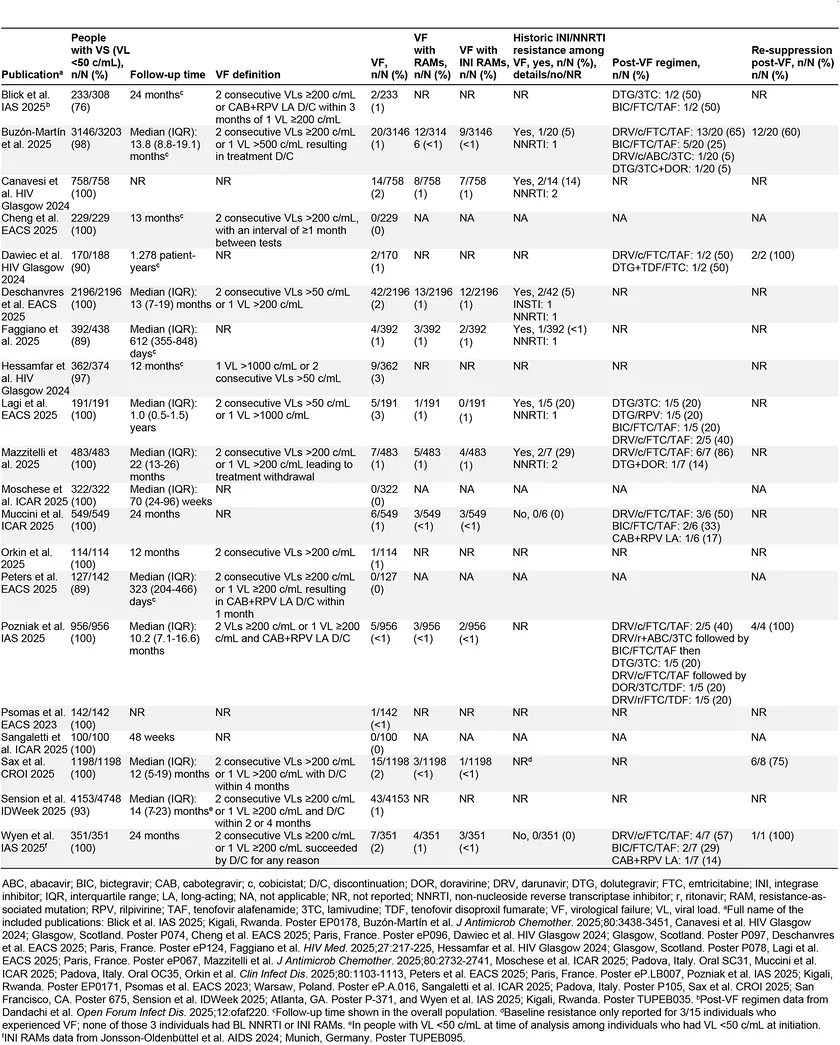
P074 | Virological failure after CAB+RPV LA treatment, resistance‐associated mutations and regimens used after virological failure.

### P075 Abstract Withdrawn

### P076 Patient‐Reported Burden of Daily Antiretroviral Therapy and the Lived Experience of Switching to Cabotegravir+Rilpivirine Long‐Acting: Mixed‐Methods Findings From the VOLITION Trial

Cassidy A. Gutner ^1^, Ian Jacob^2^, Julie Priest^1^, Rekha Trehan^2^, Anoushka Dugal^3^, Avishek Chatterjee^3^, Kehui Wang^4^, Miguel Pascual‐Bernaldez^5^, Patricia De los Rios^6^, Monika Bui^3^, Kai Hove^2^, Cheryl McDonald^7^, Mezgebe Berhe^8^, Jean van Wyk^2^, Bryn Jones^2^

^1^ViiV Healthcare, Durham, NC, US; ^2^ViiV Healthcare, London, UK; ^3^GSK, London, UK; ^4^GSK, Collegeville, PA, US; ^5^ViiV Healthcare, Madrid, Spain; ^6^ViiV Healthcare, Montreal, QC, Canada; ^7^Tarrant County Infectious Diseases Associates, Fort Worth, TX, US; ^8^North Texas Infectious Diseases Consultants, Dallas, TX, US

**Background**: Daily oral antiretroviral therapy (ART) can impose burden on people with HIV‐1. In the VOLITION phase 3b study, ART‐naïve participants initiated dolutegravir/lamivudine; upon achieving virologic suppression, the study evaluated participant‐perceived treatment burden following an optional switch to cabotegravir+rilpivirine long‐acting (CAB+RPV LA).

**Methods**: Participants (*n* = 167) completed questionnaires at day of choice (DoC), month 5/6 and month 11/12 assessing adherence anxiety, HIV status reminders, stigma and mood. A subgroup participated in qualitative interviews at DoC (*n* = 43 interviewed; 40 analysed within a 2‐week window) and month 11/12 (*n* = 28) to explore how treatment modality affected daily life and psychological burden.

**Results**: Before switching, among participants using CAB+RPV LA (*n* = 129; 127 complete surveys), 85% (108/127) reported adherence‐related anxiety, 87% (110/127) reported their medication was a recurring HIV reminder and 73% (93/127) reported any concern about others unintentionally discovering their HIV status through their treatment (including “rarely” [5‐point scale ranging from “never” to “always”]). From DoC to month 11/12, the proportion of participants using CAB+RPV LA completing the survey (*n* = 110) reporting any adherence‐related anxiety decreased to 60% (66/110) and those reporting their medication was a recurring HIV reminder decreased to 79% (87/110). On a 5‐point scale of overall HIV‐related worry, the proportion “not worried at all” about their diagnosis increased from 33% (42/127) at DoC to 52% (60/115) at month 11/12. Qualitative interviews (Table 1) identified the mechanisms underlying these shifts. At DoC, more than half of interviewed participants (21/40) discussed concern about unintentional disclosure of their HIV status. Of participants who chose to switch to CAB+RPV LA, 69% (27/39) discussed no longer needing to remember a daily pill as a key advantage of CAB+RPV LA and 69% (18/26) cited adherence concerns as a primary reason for switching. At DoC, among those who discussed advantages of CAB+RPV LA (*n* = 39), 69% (27/39) anticipated no longer needing to remember a daily pill. Among switchers, 62% (16/26) anticipated fewer daily HIV status reminders and 88% (23/26) a strong alignment with lifestyle.

**Conclusions**: CAB+RPV LA reduced burden of daily oral ART by easing pill‐taking anxiety and disclosure concerns, supporting routine offer of long‐acting therapy as a patient‐centred treatment option.

**TABLE 1:**
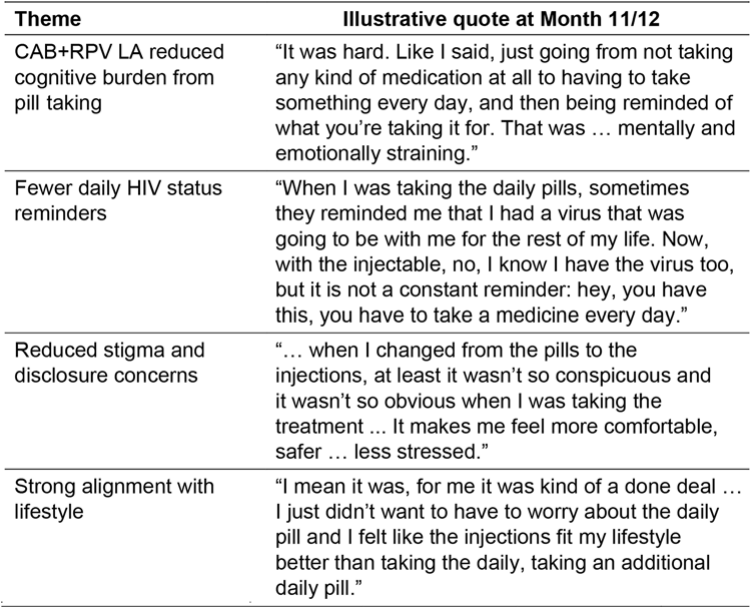
P076 | Supporting qualitative quotes.

### P077 Staff Perspectives on the Burden of Daily Oral Antiretroviral Therapy and Clinical Implementation of Switching to Cabotegravir+Rilpivirine Long‐Acting: Mixed‐Methods Findings From the VOLITION Trial

Cassidy A. Gutner ^1^, Ian Jacob^2^, Julie Priest^1^, Rekha Trehan^2^, Anoushka Dugal^3^, Avishek Chatterjee^3^, Kehui Wang^4^, Miguel Pascual‐Bernaldez^5^, Patricia De los Rios^6^, Monika Bui^3^, Kai Hove^2^, Cheryl McDonald^7^, Mezgebe Berhe^8^, Jean van Wyk^2^, Bryn Jones^2^

^1^ViiV Healthcare, Durham, NC, US; ^2^ViiV Healthcare, London, UK; ^3^GSK, London, UK; ^4^GSK, Collegeville, PA, US; ^5^ViiV Healthcare, Madrid, Spain; ^6^ViiV Healthcare, Montreal, QC, Canada; ^7^Tarrant County Infectious Diseases Associates, Fort Worth, TX, US; ^8^North Texas Infectious Diseases Consultants, Dallas, TX, US

**Background**: Healthcare staff observe that daily pill taking remains a burden for people with HIV‐1, affecting adherence, psychosocial well‑being and daily life. The VOLITION study evaluated staff study participant (SSP) perspectives on treatment burden and care optimization when offering early switch to injectable cabotegravir+rilpivirine long‐acting (CAB+RPV LA) after rapid virologic suppression with daily oral dolutegravir/lamivudine (DTG/3TC) in antiretroviral therapy (ART)‐naïve patients.

**Methods**: SSPs (*n* = 102) completed validated quantitative assessments evaluating perceptions of HIV‐1 treatment, acceptability (Acceptability of Intervention Measure; AIM) and feasibility (Feasibility of Intervention Measure; FIM) of CAB+RPV LA at day 1, day of choice (DoC), month 5/6 (M5/6) or month 11/12 (M11/12). A subset (*n* = 64) participated in qualitative interviews at DoC and M11/12 to explore SSP‐observed treatment burden, unmet needs and perceived treatment impact.

**Results**: Daily adherence challenges were the most reported potential disadvantage of DTG/3TC among responding staff at day 1 (73%; 69/95), M5/6 (73%; 63/86) and M11/12 (64%; 46/72). Staff insights on oral DTG/3TC highlighted that patients may prefer alternatives to daily oral therapy at M5/6 (67%; 58/86) and M11/12 (58%; 42/72). Conversely, for patients achieving virologic suppression who chose to switch, staff rated CAB+RPV LA highly acceptable (mean AIM, 4.4 [standard deviation {SD}, 0.79] at DoC; 4.2 [SD, 0.84] at M11/12) and highly feasible (mean FIM, 4.3 [SD, 0.84] at DoC; 4.2 [SD, 0.93] at M11/12) on a 1−5 scale. In DoC qualitative interviews, 97% of SSPs (71/73) expressed positive perceptions of CAB+RPV LA, citing reduced pill‐taking burden, lower adherence‐related anxiety and decreased HIV stigma. At M11/12, 91% of SSPs (58/64) reported CAB+RPV LA addressed previously unmet patient needs, most commonly by reducing adherence‐related anxiety (30/58), daily medication‐associated stigma (26/58) and the daily reminder of HIV status (21/58).

**Conclusions**: Staff in VOLITION viewed CAB+RPV LA as addressing the persistent burden of daily oral ART by providing a highly acceptable and feasible option that optimizes HIV care by addressing adherence‑related challenges and unmet patient needs, consistent with findings from the paired VOLITION patient analysis. Together, these findings support integrating early‐switch CAB+RPV LA protocols immediately following virologic suppression to actively reduce daily treatment burden and stigma.

### P078 Consistently High Cabotegravir+Rilpivirine Long‐Acting Persistence and Effectiveness Across Ages and Race/Ethnicity in the US OPERA Cohort

Ricky Hsu^1,2^, Michael Sension^3^, Jennifer Fusco ^4^, Laurence Brunet^4^, Quateka Cochran^5^, Gayathri Sridhar^6^, Vani Vannappagari^6^, Kimberley Brown^6^, Jean van Wyk^7^, Michael Wohlfeiler^8^, Gregory Fusco^4^

^1^AIDS Healthcare Foundation, New York, NY, US; ^2^NYU Langone Medical Center, New York, NY, US; ^3^CAN Community Health, Fort Lauderdale, FL, US; ^4^Epividian, Inc., Raleigh, NC, US; ^5^AIDS Healthcare Foundation, Fort Lauderdale, FL, Uruguay; ^6^ViiV Healthcare, Durham, NC, US; ^7^ViiV Healthcare, London, England, UK; ^8^AIDS Healthcare Foundation, Miami, FL, US

**Background**: Long‐acting (LA) antiretroviral formulations are increasingly used across diverse age and race/ethnicity groups offering sustained therapeutic coverage, fewer drug interactions and an alternative that may help reduce stigma. Cabotegravir+rilpivirine (CAB+RPV) LA is the only complete LA regimen for HIV‐1 treatment of virologically suppressed individuals in the US. We described persistence and virologic outcomes by age and race/ethnicity among CAB+RPV LA users in routine clinical care.

**Methods**: Adults with virologically suppressed (viral load [VL] < 50 copies/mL) HIV in the OPERA Cohort switching to CAB+RPV LA (21 January 2021–31 December 2025) were followed through 29 February 2026. Among complete initiators (first 2 injections ≤67 days apart), we assessed persistence (on CAB+RPV LA at study end), maintenance of suppression (all VLs < 50 copies/mL) and confirmed virologic failure (2 VLs > 200 copies/mL or 1 VL > 200 copies/mL + discontinuation). Outcomes were assessed overall and stratified by age (18–49, 50–64, ≥65 years) and race/ethnicity (Hispanic, non‐Hispanic Black, non‐Hispanic white) at initiation.

**Results**: 6955 adults started CAB+RPV LA while virologically suppressed in OPERA. Older individuals had more VL testing, had been on the previous regimen for longer, but were less likely to switch from an INSTI‐based regimen than younger individuals. The highest proportion of women was observed among non‐Hispanic Black individuals; other characteristics were somewhat balanced across race/ethnicity groups (Table 1). At study end, 76%–83% of complete initiators alive and in care remained on CAB+RPV LA for a median of 18–23 months, with minimal variation across groups. Virologic effectiveness was high, with 94% suppressed at last follow‐up. A lower proportion of individuals aged ≥65 years maintained continuous suppression without blips (71%) compared with those aged 18–49 years (82%) (Figure 1). Confirmed virologic failure was rare across all groups (<1%–2%).

**Conclusions**: In this large real‐world cohort of virologically suppressed adults in the US, CAB+RPV LA showed high persistence and consistently high effectiveness across age and race/ethnicity groups. Most individuals maintained virologic suppression and confirmed virologic failure was rare across all groups. Although adults aged ≥65 years appeared to experience more virologic blips, this was not associated with a higher risk of virologic failure and may be attributable to increased monitoring.

**FIGURE 1:**
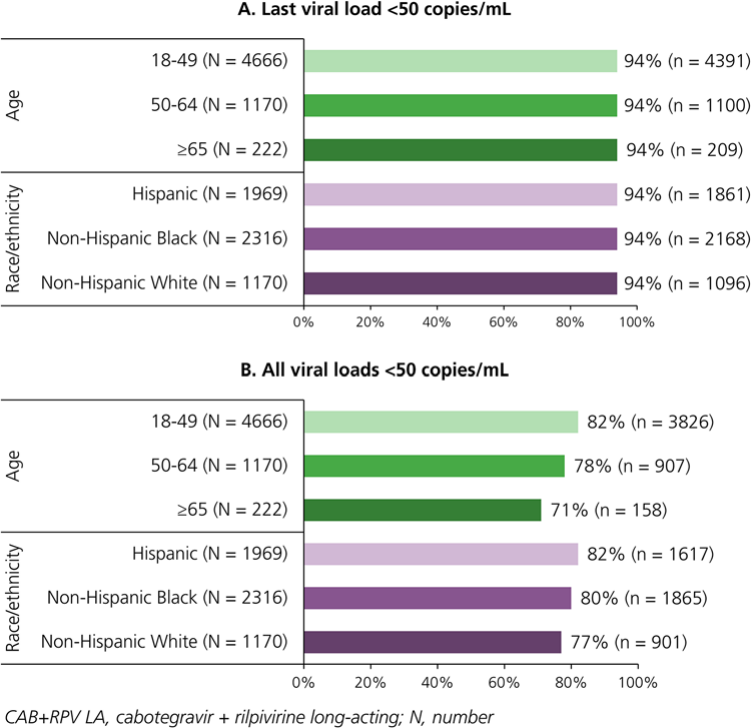
P078 | CAB+RPV LA virologic effectiveness among complete initiators with ≥1 follow‐up viral load.

**TABLE 1:**
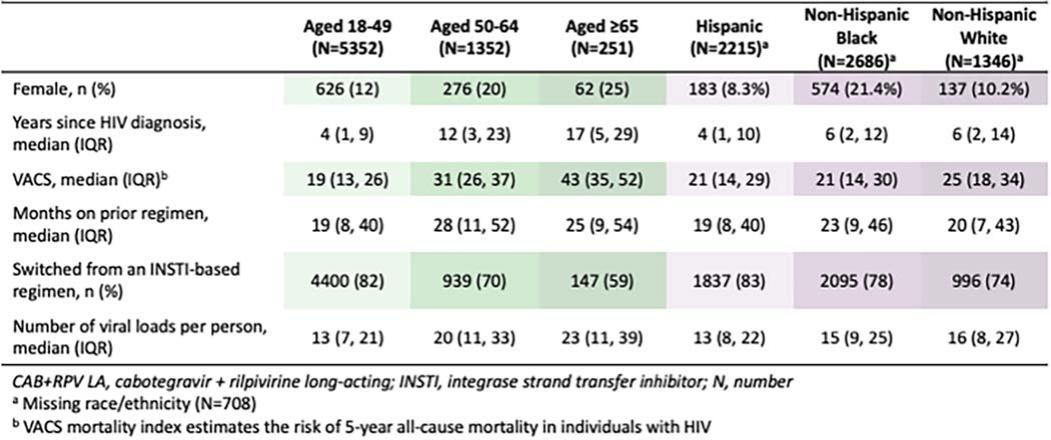
P078 | Demographic and clinical characteristics at the first CAB+RPV LA injection.

### P079 Beyond Viral Suppression: Adherence Challenges and Unmet Needs in People With HIV in the UK (Positive Perspectives 3 Study)

Garry Brough ^1^, Mercy Shibemba^2^, Liz Foote^3^, Abdul Halim^3^, Thomas Bendall^3^, Ta‐Fen Hwang^4^, Chinyere Okoli^3^, Nneka Nwokolo^5^, Rickesh Patel^6^, Ama Appiah^3^

^1^Transformation Partners in Health and Care, London, UK; ^2^Independent Consultant, Manchester, UK; ^3^ViiV Healthcare, London, UK; ^4^ViiV Healthcare, Singapore, Singapore; ^5^ St Stephen's Centre, Chelsea and Westminster Hospital, London, UK; ^6^GSK, London, UK

**Background**: As people with HIV require lifelong antiretroviral therapy (ART), shared decision‐making empowers participation in treatment decisions, driving treatment satisfaction and adherence, which influence health‐related quality of life. Yet, unmet needs in person‐centred care persist. The Positive Perspectives 3 (PP3) study is a global, cross‐sectional survey co‐designed with community representatives, aiming to capture experiences of people with HIV across 29 geographic areas. Here, we explore perspectives and needs of people with HIV in the UK.

**Methods**: Self‐reported data from adults with HIV in the UK receiving ART, including treatment adherence, treatment satisfaction and relationships with HIV care providers (HCPs), were analysed. Suboptimal treatment adherence was defined as reporting any non‐adherence behaviour (mistakenly or intentionally missing a dose, not following prescription or taking extra medication) sometimes, often or always. Descriptive data were summarized as percentages.

**Results**: Among 349 participants, 40% (138/349) were female sex at birth and 26% (89/349) identified as gay; median (range) age was 36 (22–107) years (Table 1). Over half (57% [183/323]) worried about their health; 54% (172/319) reported “fair to very poor” for overall and mental health and 51% (164/319) reported “fair to very poor” for sexual health. Approximately half stated their medication was an uncomfortable reminder of their HIV status (48% [154/321]) and that managing HIV wears them out (45% [146/323]). Most (95% [330/349]) used oral ART, with only 42% (139/330) taking a once‐daily single‐tablet regimen. Over half reported suboptimal adherence in the previous year (58% [203/349]); 28% (98/346) intentionally missed doses (Figure 1). Only 65% (213/329) had been told by their HCP that they were currently virologically suppressed. Although 73% (238/328) were satisfied with their medication, 53% (173/328) believed it could be improved and 34% (112/328) experienced side effects, with 25% (78/315) reporting that their HCP had not recently asked about side effects. Only 65% (205/315) were satisfied with their involvement in treatment decisions.

**Conclusions**: These findings highlight ongoing unmet needs for people with HIV in the UK: widespread suboptimal adherence across demographic groups, substantial health burden and treatment concerns. Increased shared decision‐making is crucial for ensuring person‐centred care. Further analysis of non‐adherence is in progress.

**FIGURE 1:**
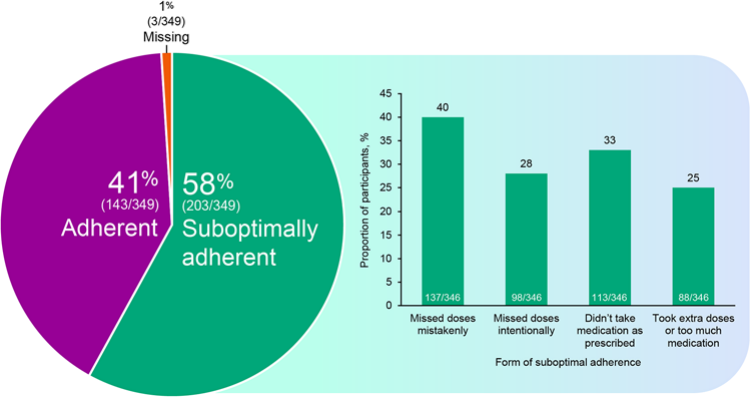
P079 | Suboptimal adherence to HIV medication in the previous 12 months.

**TABLE 1:**
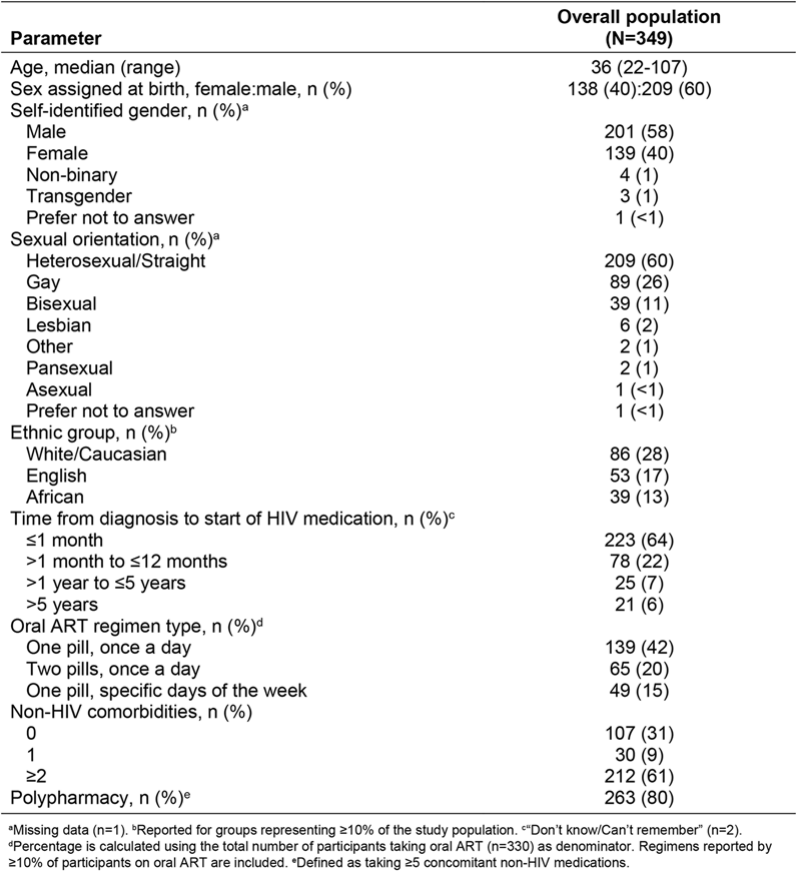
P079 | Baseline demographics and characteristics.

### P080 Not All Dual INSTI‐Based Regimens Are Equal: Real‐World Durability and Virological Outcomes of DTG/3TC Versus CAB/RPV

Camilla Muccini ^1^, Nicholas Brian Bana^1^, Giulia Burastero^1^, Leonardo Francesco Rezzonico^1^, Andrea Forni^2^, Elisa Di Gennaro^1^, Maria Cristina Moioli^1^, Massimo Puoti^1,2^

^1^ASST Grande Ospedale Metropolitano Niguarda, Milan, Italy; ^2^University of Milano‐Bicocca, Milan, Italy

**Background**: Oral dolutegravir/lamivudine (DTG/3TC) and long‐acting cabotegravir/rilpivirine (CAB/RPV) are INSTI‐based regimen adopted as switch strategy for people with HIV (PWH). Aim of the study was to assess and compare treatment durability and virological outcomes in PWH switching to DTG/3TC or CAB/RPV.

**Methods**: Longitudinal cohort of PWH who switched to DTG/3TC or CAB/RPV from July 2022, when long‐acting therapy became available in Italy. Follow‐up was censored at regimen discontinuation for any cause or at 31 December 2025, whichever occurred first. Participants with < 6 months of follow‐up were excluded. Virological failure (VF) was defined as two consecutive HIV‐RNA > 200 copies/mL or a single > 1000 copies/mL, blip as a single HIV‐RNA > 50 and < 1000 copies/mL, low‐level viraemia (LLV) as persistent HIV‐RNA between 50 and 200 copies/mL. Treatment retention was assessed using Kaplan–Meier survival analysis.

**Results**: We included 375 PWH (183 in DTG/3TG and 192 in CAB/RPV): 311 (82.9%) were male, with a median age of 50 (41–59) years (Table 1). Overall, 35 participants discontinued their regimen: 11/183 (6.0%) in the DTG/3TC group and 24/192 (12.5%) in the CAB/RPV group (*p* = 0.034) over a median time of 13.2 (7.7–18.5) and 10.3 (4.3–15.6) months (*p* = 0.311), respectively. Kaplan–Meier estimated retention at 24 months was 93.1% in the DTG/3TC group and 88.5% in the CAB/RPV group (log‐rank *p* = 0.084, Figure 1). Throughout the follow‐up, 161/183 (88.0%) in DTG/3TC and 147/192 (76.6%) in CAB/RPV maintained virological suppression at each HIV‐RNA measurement (*p* = 0.005). VF occurred in 6 participants in both groups (3.3% vs. 3.1%), with comparable incidence rates (1.64 vs. 1.31 per 100 person‐years). Resistance‐associated mutations were documented in 4/6 CAB/RPV versus none in DTG/3TC (*p* = 0.061). Blips were more frequent in the CAB/RPV group (15.6% vs. 7.1%; *p* = 0.014), while LLV was observed in 9/192 (4.7%) PWH receiving CAB/RPV and in 2/183 (1.1%) on DTG/3TC (*p* = 0.062). Virological suppression at the last available measurement was equivalent (96.4% for CAB/RPV vs. 96.7% for DTG/3TC).

**Conclusions**: DTG/3TC and CAB/RPV demonstrated comparable efficacy with distinct durability profiles. Despite similar rates of VF, DTG/3TC was associated with fewer treatment discontinuations and resistance‐associated mutations, whereas CAB/RPV showed a higher frequency of blips and LLV.

**FIGURE 1:**
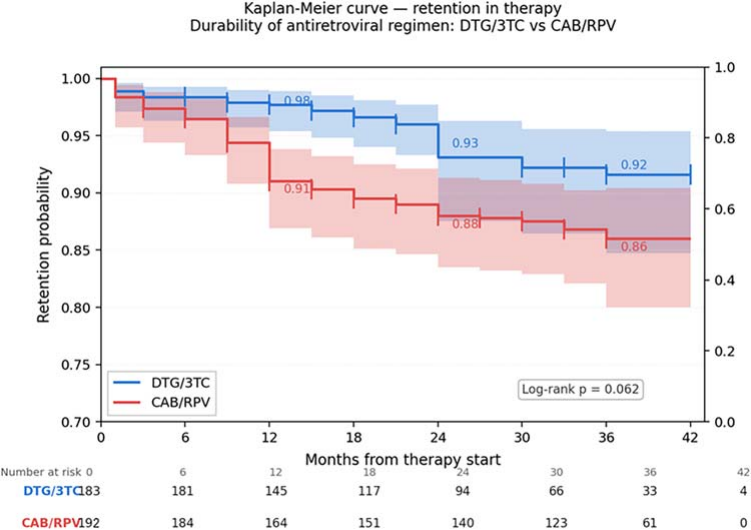
P080 | Kaplan–Meier curves for retention in therapy in people with HIV on dolutegravir/lamivudine (DTG/3TC) versus cabotegravir/rilpivirine (CAB/RPV).

**TABLE 1:**
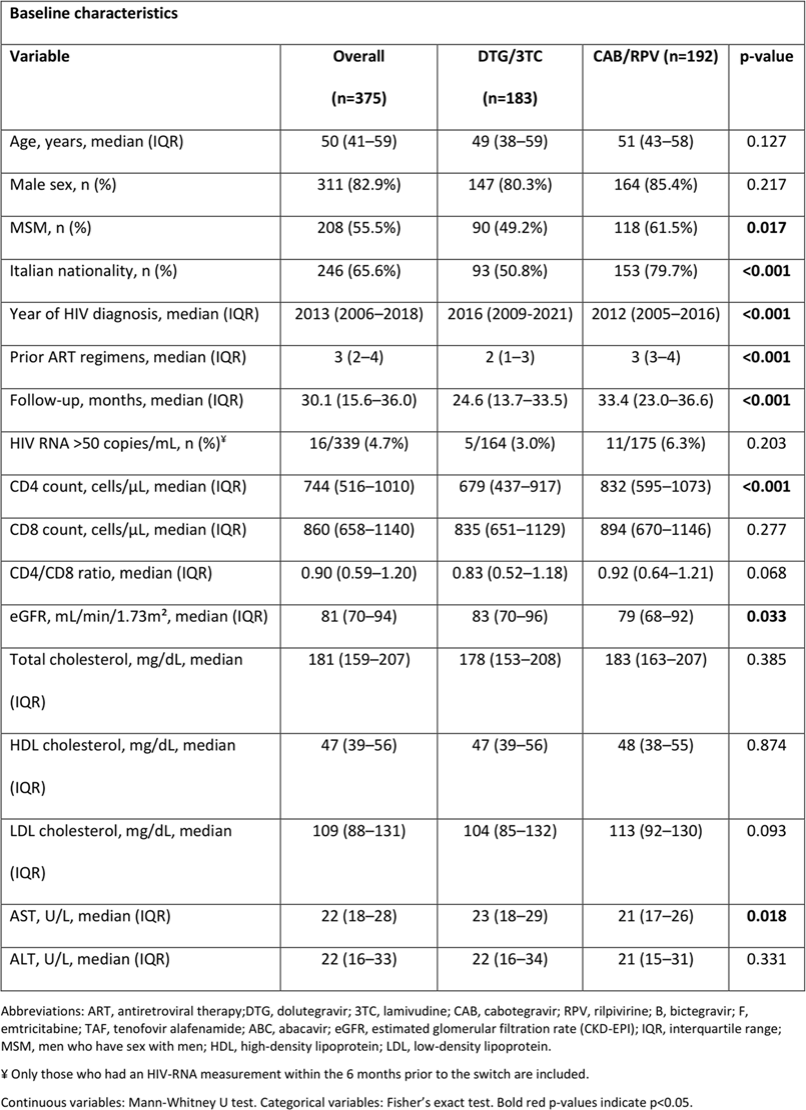
P080 | Baseline characteristics of people with HIV on dolutegravir/lamivudine (DTG/3TC) versus cabotegravir/rilpivirine (CAB/RPV).

### P081 Non‐RPV NNRTI RAMs Are Associated With Virological Failure in People With HIV Receiving Cabotegravir/Rilpivirine

Camilla Muccini ^1^, Nicholas Brian Bana^1^, Andrea Carbone^2,3^, Simona Di Giambenedetto^2,3^, Carlo Torti^2,3^, Paolo Bonfanti^4,5^, Fiore Artelli^5^, Diego Ripamonti^6^, Paola Vitiello^7^, Clara Abeli^7^, Giorgio Tiecco^8^, Eugenia Quiros‐Roldan^8^, Massimo Puoti^1,5^, Roberto Rossotti^9^

^1^ASST Grande Ospedale Metropolitano Niguarda, Milan, Italy; ^2^IRCCS Fondazione Policlinico Universitario A. Gemelli, Rome, Italy; ^3^Catholic University of the Sacred Heart, Rome, Italy; ^4^IRCCS Fondazione San Gerardo dei Tintori, Monza, Italy; ^5^University of Milano‐Bicocca, Milan, Italy; ^6^ASST Papa Giovanni XXIII, Bergamo, Italy; ^7^ASST Valle Olona, Ospedale di Circolo, Busto Arsizio, Italy; ^8^Department of Clinical and Experimental Sciences, Unit of Infectious and Tropical Diseases, University of Brescia and ASST Spedali Civili di Brescia, Brescia, Italy; ^9^University of Verona, Verona, Italy

**Background**: Eligibility criteria for long‐acting cabotegravir/rilpivirine (CAB/RPV) differ between regulatory agencies: while EMA excludes people with HIV (PWH) harbouring NNRTI resistance‐associated mutations (RAMs), FDA restricts exclusion only to RPV‐specific RAMs, considering non‐RPV non‐nucleoside reverse transcriptase inhibitors (NNRTI) resistance‐associated mutations (RAMs) acceptable for treatment switch. We aimed to evaluate the impact of non‐RPV NNRTI RAMs on virological failure (VF) among PWH receiving CAB/RPV.

**Methods**: A retrospective multicentre observational study was conducted on PWH switching to CAB/RPV. Non‐RPV NNRTI RAMs were identified using the Stanford University HIV Drug Resistance Database. The association between non‐RPV NNRTI RAMs and VF, defined as confirmed HIV‐RNA > 200 copies/mL, was assessed by logistic regression and inverse probability of treatment weighting using propensity scores derived from the following baseline covariates: HIV transmission risk, HCV coinfection, nadir CD4 count, years from HIV diagnosis, previous dual therapy, number of prior antiretroviral regimens and baseline HIV‐RNA.

**Results**: Overall, 678 PWH were included: 506 with no available cumulative historical genotypic resistance data, 140 without and 32 with non‐RPV NNRTI RAMs. The study population was predominantly male (553, 81.6%), with a median age of 48 (interquartile range, IQR 40–57) years. PWH with non‐RPV NNRTI RAMs had a significantly longer history of HIV infection (17.4 vs. 10.3 years; *p* < 0.001), lower nadir CD4 count (304 vs. 341 cells/mcL; *p* < 0.001), higher rates of HCV coinfection (21.9% vs. 6.4%; *p* = 0.026) and more frequent injection drug user as HIV transmission risk (18.8% vs. 4.3%; *p* < 0.001) compared to those without non‐RPV NNRTI RAMs. K103N was the most detected RAM (19/32, 59.4%). Over a median follow‐up of 1.62 (IQR 0.70–2.38) years, VF occurred in 13 PWH (1.9%), with a higher rate among those with non‐RPV NNRTI RAMs (12.5%) than those without (1.4%; *p* < 0.001). Participants’ characteristics are reported in Table 1. On logistic regression, PWH harbouring non‐RPV NNRTI RAMs were more likely to experience VF than those with unknown resistance data (odds ratio [OR] 10.18, 95% CI: 2.81–36.85; *p* < 0.001). After propensity score weighting, the association remained statistically significant (OR 9.61, 95% CI: 1.91–48.38; *p* = 0.006).

**Conclusions**: Non‐RPV NNRTI RAMs were independently associated with VF in PWH receiving CAB/RPV, supporting a more cautious people selection approach.

**TABLE 1:**
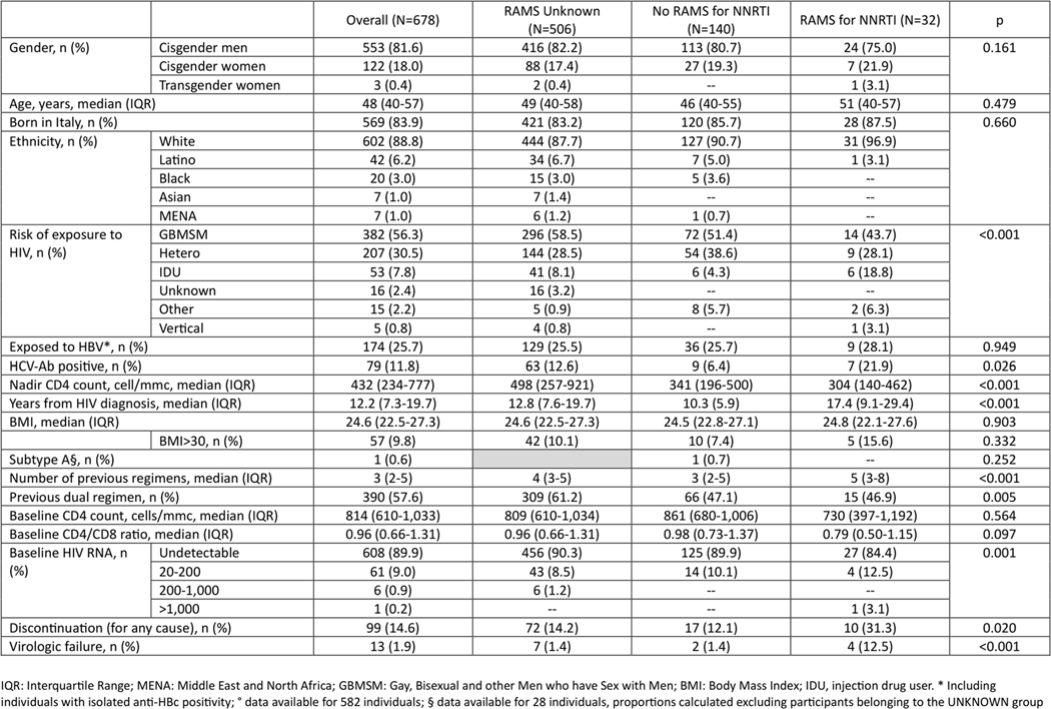
P081 | Demographic and clinical features of the study population.

### P082 Impact of Virological Suppression Status on Immune Recovery After Switch to Long‐Acting CAB/RPV

Camilla Muccini ^1^, Nicholas Brian Bana^1^, Giulia Burastero^1^, Francesco Leonardo Rezzonico^1^, Paola Vitiello^2^, Clara Abeli^2^, Gaia Cologgi^3^, Giorgio Tiecco^4^, Eugenia Quiros‐Roldan^4^, Laura Comi^5^, Giuseppe Lapadula^6,7^, Paolo Bonfanti^6,7^, Massimo Puoti^1,7^, Roberto Rossotti^8^

^1^ASST Grande Ospedale Metropolitano Niguarda, Milan, Italy; ^2^ASST Valle Olona, Ospedale di Circolo, Busto Arsizio, Italy; ^3^IRCCS Ospedale Policlinico San Martino, Genova, Italy; ^4^Department of Clinical and Experimental Sciences, Unit of Infectious and Tropical Diseases, University of Brescia and ASST Spedali Civili di Brescia, Brescia, Italy; ^5^ASST Papa Giovanni XXIII, Bergamo, Italy; ^6^IRCCS Fondazione San Gerardo dei Tintori, Monza, Italy; ^7^University of Milano‐Bicocca, Milan, Italy; ^8^University of Verona, Verona, Italy

**Background**: Long‐acting cabotegravir/rilpivirine (CAB/RPV) is increasingly adopted as a treatment‐switch strategy in people with HIV (PWH). We aimed to evaluate whether virological suppression status at the time of switching is associated with subsequent trajectories of CD4/CD8 ratio following the switch.

**Methods**: A retrospective, multicentre cohort study was conducted in PWH who switched to CAB/RPV. Participants were stratified by baseline virological status: target not detected (TND: HIV‐RNA < 1 copy/mL), versus detectable viraemia, including those with viraemia below the limit of quantification (BLQ: HIV‐RNA 1–20 copies/mL). Longitudinal changes in CD4/CD8 ratio were analysed using a linear mixed‐effects model with repeated measures. Time since switch, baseline virological status and their interaction (TNDxtime) was included as a fixed effect. Random intercepts and random slopes at the individual patient level were incorporated in the model to account for within‐subject correlation. Models were adjusted for gender, age, CD4 nadir, duration of HIV infection, prior dual‐regimen use and on‐time administration of CAB/RPV.

**Results**: We included 546 PWH: 453 (83.0%) were male with a median age of 48 (interquartile range [IQR] 40–57) years; 415 (76%) were virologically suppressed with TND and 131 (24%) had detectable viraemia at baseline, including 64 with viraemia BLQ. The baseline CD4/CD8 ratio was significantly higher (0.99 vs. 0.89, *p* = 0.002) in PWH starting CAB/RPV with TND compared with those with detectable viraemia (Table 1). In the adjusted mixed‐effects model, TND at baseline was independently associated with a higher CD4/CD8 throughout follow‐up (+0.136, 95% CI: 0.041–0.231, *p* = 0.005). Over a median follow‐up time of 1.89 (IQR 0.86–2.39) years, the CD4/CD8 ratio increased progressively in both groups, with a statistically significant estimated gain of +0.048/year. Despite the baseline CD4/CD8 gap, both groups improved at the same rate over time, with no evidence of trajectory divergence (*p* = 0.776, Figure 1). Male sex was associated with lower CD4/CD8 (−0.280, 95% CI: −0.39; −0.17, *p* < 0.001), while a higher CD4 nadir was associated with a higher CD4/CD8 ratio throughout follow‐up (+0.086 per 100 cells/µL increase, 95% CI: 0.068–0.103; *p* < 0.001).

**Conclusions**: PWH switching to CAB/RPV showed sustained CD4/CD8 ratio increase over follow‐up. Longitudinal CD4/CD8 patterns were similar across baseline virological status groups, with no evidence of differential evolution over time.

**FIGURE 1:**
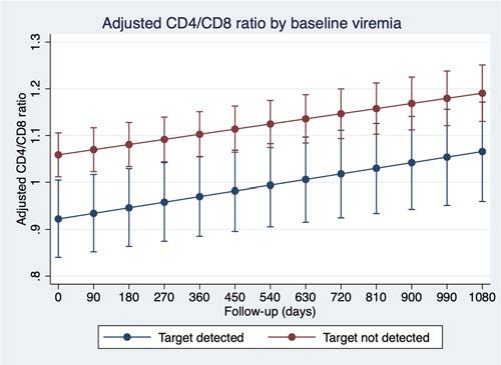
P082 | Adjusted CD4/CD8 ratio over 3‐year follow‐up according to baseline viraemia status.

**TABLE 1:**
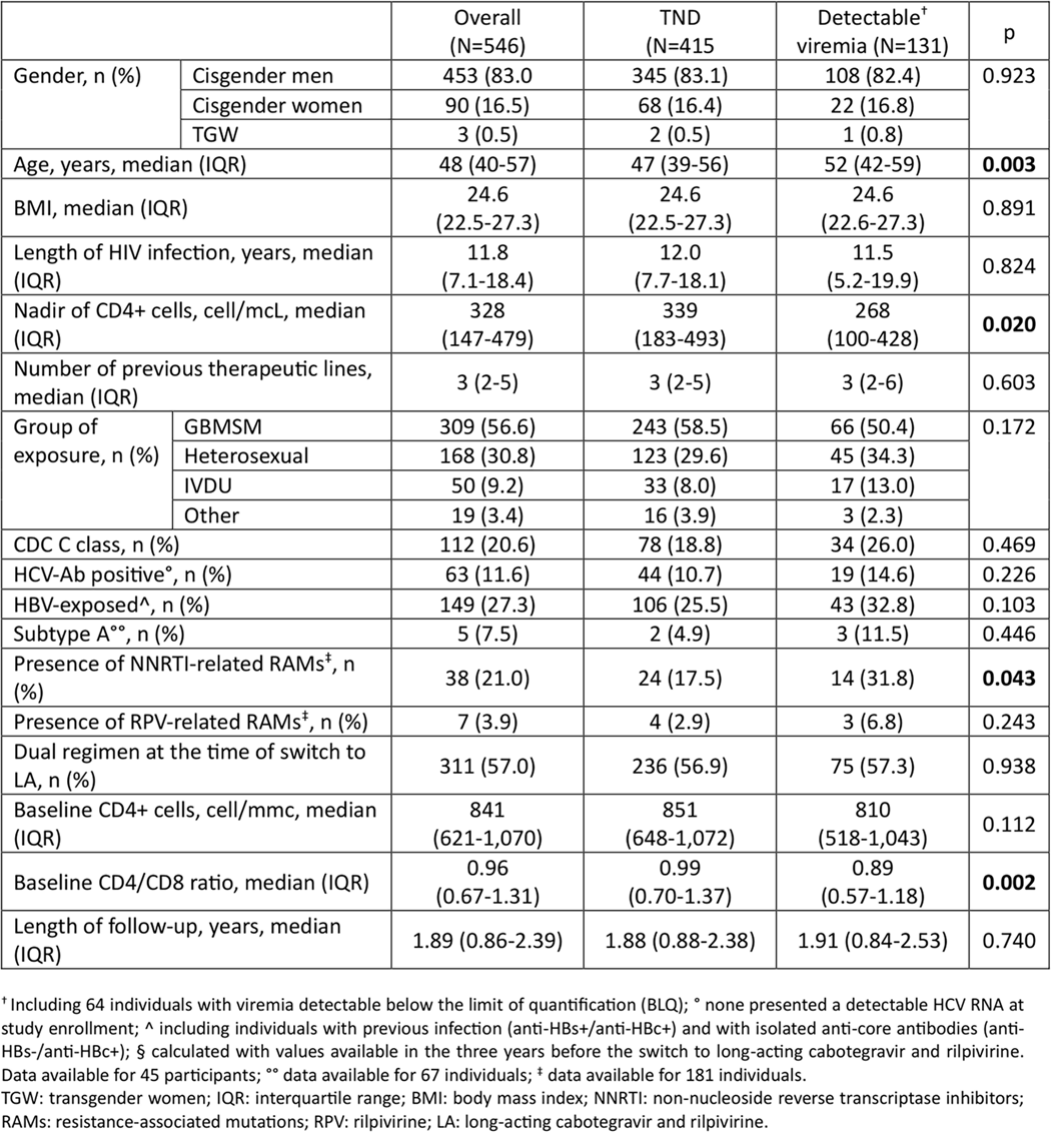
P082 | Demographic, virologic and clinical features of the study population.

### P083 Comparison of Virological Success and Immunological Recovery of LAI CAB/RPV Versus DOR/3TC/TDF in Virologically Suppressed People With HIV

Arturo Ciccullo ^1^, Gianmaria Baldin^2^, Adriana Cervo^3^, Valentina Iannone^4^, Sasha Trevisan^5^, Pierluigi Francesco Salvo^2^, Andrea De Vito^6^, Maddalena Matone^4^, Massimiliano Fabbiani^7^, Gabriella D'Ettorre^8^, Stefano Rusconi^9^, Filippo Lagi^5^, Alberto Borghetti^10^, Chiara Papalini^11^, Giordano Madeddu^6^, Spinello Antinori^4^, Andrea Gori^4^, Cristina Mussini^3^, Simona Di Giambenedetto^2^

^1^UOC Malattie Infettive, L'Aquila, Italy; ^2^Fondazione Policlinico Universitario A. Gemelli IRCCS, Rome, Italy; ^3^Department of Infectious Diseases, Azienda Ospedaliero‐Universitaria of Modena, Modena, Italy; ^4^Department of Infectious Diseases, ASST Fatebenefratelli Sacco, Luigi Sacco Hospital, Milan, Italy; ^5^Department of Experimental and Clinical Medicine, University of Florence, Firenze, Italy; ^6^Unit of Infectious Diseases, Department of Medicine, Surgery and Pharmacy, Sassari, Italy; ^7^Department of Medical Sciences, Infectious and Tropical Diseases Unit, University Hospital of Siena, Siena, Italy; ^8^Department of Public Health and Infectious Diseases, Sapienza University of Rome, Rome, Italy; ^9^Infectious Diseases Unit, Ospedale Civile di Legnano ASST Ovest Milanese, Legnano, Italy; ^10^Infectious Diseases Unit, Department of Clinical and Experimental Medicine, Azienda Ospedaliera Universitaria Pisana, Pisa, Italy; ^11^Department of Medicine, Clinic of Infectious Diseases, “Santa Maria della Misericordia” Hospital, University of Perugia, Perugia, Italy

**Background**: Long‐acting CAB+RPV and DOR/3TC/TDF represent two modern maintenance switch strategies for virologically suppressed people with HIV (PWH), although comparative real‐world data on long‐term effectiveness and immunological outcomes remain limited.

**Methods**: We conducted a multicentre observational study including virologically suppressed PWH switching to either DOR/3TC/TDF or long‐acting CAB+RPV (LAI). The primary endpoint was virological failure (VF), defined as two consecutive HIV‐RNA > 50 copies/mL or a single determination > 1000 copies/mL. Secondary endpoints included changes in CD4+ cell count and CD4/CD8 ratio. Survival analyses were performed using Kaplan–Meier curves and Cox regression models. Exclusion criteria included HBsAg positivity, BMI > 30 kg/m^2^, previous resistance‐associated mutations to study drugs and HIV‐1 subtype A1/A6 infection.

**Results**: Overall, 857 PWH were included: 438 in the DOR group and 419 in the LAI group. Population characteristics are summarized in Table 1. Regarding treatment effectiveness, 21 VF occurred in the DOR group (1.7/100 PYFU) and 5 in the LAI group (0.7/100 PYFU). Estimated probabilities of maintaining virological suppression at weeks 48 and 144 were 97.8% and 92.1% in the DOR group and 99.1% and 98.1% in the LAI group (Figure 1), respectively (log‐rank *p* = 0.002). Compared with LAI, DOR‐based therapy was associated with an increased risk of VF (aHR 3.37, *p* = 0.028), after adjusting for nadir CD4+, baseline CD4+ and CD4/CD8 ratio. Within the DOR group, prior VF was independently associated with increased VF risk (aHR 7.7, *p* < 0.001), whereas longer ARV exposure was protective (aHR 0.88, *p* = 0.044). No significant predictors emerged in the LAI group. Regarding immunological parameters, both groups showed significant improvements in CD4+ cell count and CD4/CD8 ratio throughout follow‐up. At week 144, the DOR group showed a median CD4+ increase of +41 cell/mm^3^ (*p* < 0.001) and a CD4/CD8 ratio increase of +0.1 (*p* < 0.001), whereas the LAI group showed a median CD4+ increase of +48 cell/mm^3^ (*p* = 0.045) and a ratio increase of +0.06 (*p* = 0.011). In adjusted analyses, no significant between‐group differences in immunological recovery were observed.

**Conclusions**: In this large real‐world cohort, LAI CAB+RPV showed greater long‐term virological effectiveness than DOR/3TC/TDF, while both strategies achieved comparable immunological recovery. Prior VF strongly impacted outcomes in individuals receiving DOR‐based therapy, highlighting the importance of careful selection for oral maintenance strategies.

**FIGURE 1:**
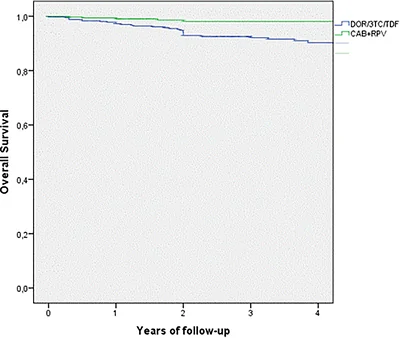
P083 | Kaplan–Meier curves for time to virological failure by treatment group.

**TABLE 1:**
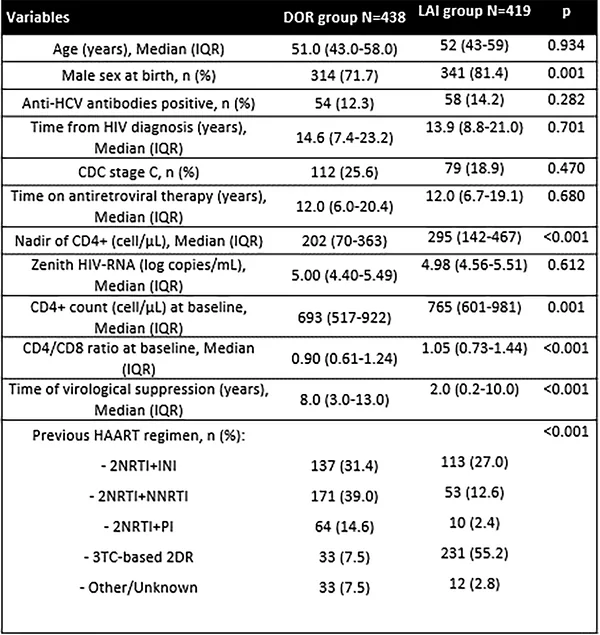
P083 | Population characteristics at baseline.

### P084 Comparative Effectiveness, Durability and Immunological Outcomes of Dolutegravir/Lamivudine Versus Bictegravir/Emtricitabine/Tenofovir Alafenamide in Antiretroviral‐Naïve People With HIV

Arturo Ciccullo ^1^, Gianmaria Baldin^2^, Maria Vittoria Cossu^3^, Alberto Zuppiroli^4^, Adriana Cervo^5^, Francesca Lombardi^2^, Andrea De Vito^6^, Valentina Iannone^3^, Marianna Menozzi^5^, Gabriella D'Ettorre^7^, Maria Aurora Carleo^8^, Alberto Borghetti^9^, Pierluigi Viale^4^, Cristina Mussini^5^, Spinello Antinori^3^, Simona Di Giambenedetto^2^

^1^Infectious Diseases Unit, San Salvatore Hospital, L'Aquila, Italy; ^2^Fondazione Policlinico Universitario A.Gemelli IRCCS, Roma, Italy; ^3^Department of Infectious Diseases, Luigi Sacco University Hospital, ASST Fatebenefratelli Sacco, Milano, Italy; ^4^Department of Medical and Surgical Sciences, Unit of Infectious Diseases, S.Orsola Hospital, Bologna, Italy; ^5^Department of Infectious Diseases, Azienda Ospedaliero‐Universitaria of Modena, Modena, Italy; ^6^Unit of Infectious Diseases, Department of Medicine, Surgery and Pharmacy, Sassari, Italy; ^7^Department of Public Health and Infectious Diseases, Sapienza University of Rome, Roma, Italy; ^8^Infectious Diseases and Gender Medicine Unit, Cotugno Hospital, AO dei Colli, Napoli, Italy; ^9^Università di Pisa, Pisa, Italy

**Background**: Although DTG/3TC and BIC/FTC/TAF are recommended first‐line options for treatment‐naïve people with HIV (PWH), direct real‐world comparisons are scarce. We compared virological effectiveness, treatment durability and immunological recovery between these regimens in routine clinical practice.

**Methods**: We conducted a retrospective cohort study comparing antiretroviral‐naïve PWH initiating dolutegravir/lamivudine (DTG/3TC) or bictegravir/emtricitabine/tenofovir alafenamide (BIC/FTC/TAF). Virological failure (VF) was defined as failure to achieve viral suppression within 24 weeks or confirmed virological rebound after suppression (one HIV‐RNA > 1000 copies/mL or two consecutive measurements > 50 copies/mL). Secondary outcomes included time to treatment discontinuation (TD) and changes in CD4 cell count and CD4/CD8 ratio. Kaplan–Meier methods and Cox proportional hazards models were used to evaluate time to VF and TD and their predictors. Immunological changes were compared using paired‐sample tests within groups and Mann–Whitney U tests between groups.

**Results**: We analysed 595 individuals: 129 initiated DTG+3TC and 466 BIC/FTC/TAF. Baseline characteristics are reported in Table 1. During follow‐up, 9 VF occurred in the DTG group (2.37/100PYFU) and 30 VF in the BIC group (2.75/100PYFU). The estimated probability of remaining free from VF at weeks 48, 144 and 240 was 98.3%, 91.4% and 91.4% with DTG/3TC, and 97.0%, 91.0% and 89.1% with BIC/FTC/TAF, respectively (log‐rank *p* = 0.741; Figure 1). In the BIC group, a higher baseline HIV‐RNA (aHR 1.632, *p* = 0.037) was associated with VF after adjusting for age, CD4+ and CD4/CD8 ratio. We observed 24 TD with DTG (6.2/100PYFU) and 180 with BIC (15.8/100PYFU). The estimated probability of remaining on the initial regimen at week 144 were 85.3% with DTG/3TC versus 66.3% with BIC/FTC/TAF (log‐rank *p* < 0.001). Treatment simplification, either to oral or long‐acting two‐drug regimens, was the main reason for TD in both groups. Both regimens were associated with significant improvements in CD4 count and CD4/CD8 ratio at all time points, with no between‐group differences. At week 144, median CD4 gain was +346 cell/mm^3^ with DTG and +359 cell/mm^3^ with BIC, while median CD4/CD8 ratio increase was +0.43 and +0.40, respectively.

**Conclusions**: In this real‐world cohort of treatment‐naïve PWH, DTG/3TC showed virological effectiveness and immunological recovery comparable to BIC/FTC/TAF, with greater treatment durability during long‐term follow‐up.

**FIGURE 1:**
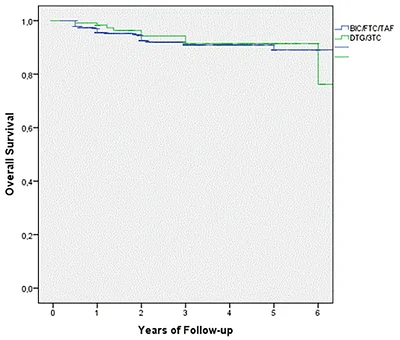
P084 | Kaplan–Meier estimates of time to virological failure by treatment group.

**TABLE 1:**
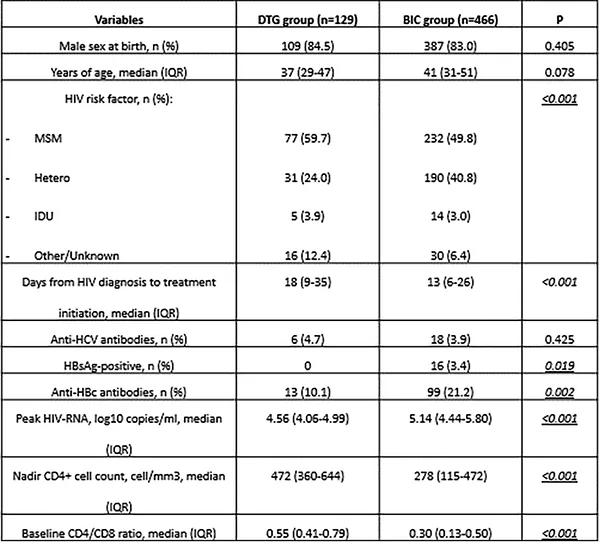
P084 | Population characteristics at baseline.

### P085 Early Dolutegravir/Lamivudine Switch Versus Continued Bictegravir‐Based Therapy Following Virological Suppression in a Real‐Life Cohort

Arturo Ciccullo ^1^, Gianmaria Baldin^2^, Andrea Giacomelli^3^, Adriana Cervo^4^, Virginia Donini^5^, Letizia Oreni^3^, Marianna Menozzi^4^, Vincenzo Esposito^6^, Massimiliano Fabbiani^7^, Annamaria Cattelan^8^, Alberto Zuppiroli^9^, Giordano Madeddu^10^, Stefano Rusconi^11^, Filippo Lagi^5^, Spinello Antinori^3^, Cristina Mussini^4^, Simona Di Giambenedetto^2^

^1^Infectious Diseases Unit, San Salvatore Hospital, L'Aquila, Italy; ^2^Fondazione Policlinico Universitario A. Gemelli IRCCS, Roma, Italy; ^3^Department of Infectious Diseases, ASST Fatebenefratelli Sacco University Hospita, Milano, Italy; ^4^Infectious Diseases Clinic, University Hospital of Modena, Modena, Italy; ^5^Dipartimento di Medicina Sperimentale e clinica, Università degli studi di Firenze, Firenze, Italy; ^6^Infectious Diseases and Gender Medicine Unit, Cotugno Hospital, AO dei Colli, Napoli, Italy; ^7^Department of Medical Sciences, Infectious and Tropical Diseases Unit, University Hospital of Siena, Siena, Italy; ^8^Infectious and Tropical Diseases Unit, Padua University Hospital, Padova, Italy; ^9^Department of Medical and Surgical Sciences, Unit of Infectious Diseases, S.Orsola Hospital, IRCCS Azienda Ospedaliero‐Universitaria di Bologna, Bologna, Italy; ^10^Università degli studi di Sassari, Sassari, Italy; ^11^Struttura Complessa di Malattie Infettive Ospedale Civile di Legnano ASST Ovest Milanese, Legnano, Italy

**Background**: Evidence supporting dolutegravir/lamivudine (DTG/3TC) is well established, but data on early treatment simplification shortly after virological suppression remain limited. We evaluated the effectiveness and durability of an early switch to DTG/3TC versus continued bictegravir/emtricitabine/tenofovir alafenamide (BIC/FTC/TAF).

**Methods**: In this retrospective cohort study, people with HIV (PWH) achieving virological suppression (HIV‐1 RNA <50 copies/mL) on INSTI‐based ART were included. The study group included individuals who switched to DTG/3TC within 12 months of virological suppression, while the comparator group included treatment‐naïve individuals initiating and maintaining BIC/FTC/TAF. Follow‐up started 12 months after virological suppression in the BIC/FTC/TAF group and at the first available visit after switching to DTG/3TC in the switch group. Individuals aged < 18 years, HBsAg‐positive, with resistance‐associated mutations to study drugs or prior virological failure were excluded. Effectiveness and durability were assessed using Kaplan–Meier analyses and adjusted Cox proportional hazards models. Immunological changes were also evaluated.

**Results**: Overall, 397 individuals were included: 113 in the DTG/3TC group and 284 in the BIC/FTC/TAF group (Table 1). Three virological failures (VFs) occurred in the DTG/3TC group (0.8/100 PYFU) and 11 in the BIC/FTC/TAF group (1.7/100 PYFU). The probability of remaining free from VF at weeks 48 and 144 was 99.0% and 97.9% with DTG/3TC and 99.6% and 94.8% with BIC/FTC/TAF, respectively, with no between‐group difference (log‐rank *p* = 0.255, Figure 1). Virological failure rates were consistent across time‐to‐suppression strata within the DTG/3TC group (<24 vs. > 24 weeks; log‐rank *p* = 0.924). No resistance‐associated mutations were detected among individuals with available post‐failure genotypes. Durability differed significantly between groups (log‐rank *p* < 0.001). Probability of maintaining study regimen at weeks 48 and 144 was 97.3% and 86.3% with DTG/3TC, compared with 90.4% and 66.7% with BIC/FTC/TAF. Treatment simplification, including switches to long‐acting regimens or alternative two‐drug regimens, was the leading reason for discontinuation in both groups. Treatment intensification accounted for only two discontinuations in the DTG/3TC group and three in the BIC/FTC/TAF group.

Both groups experienced significant increases in CD4+ count and CD4/CD8 ratio, with no between‐group differences.

**Conclusions**: Early switch to DTG/3TC maintained virological effectiveness comparable to continued BIC/FTC/TAF, with similar immunological outcomes over follow‐up.

**FIGURE 1:**
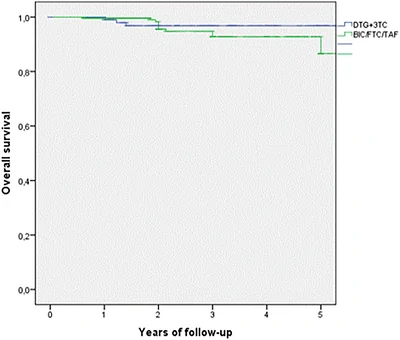
P085 | Probability of remaining free from virological failure.

**TABLE 1:**
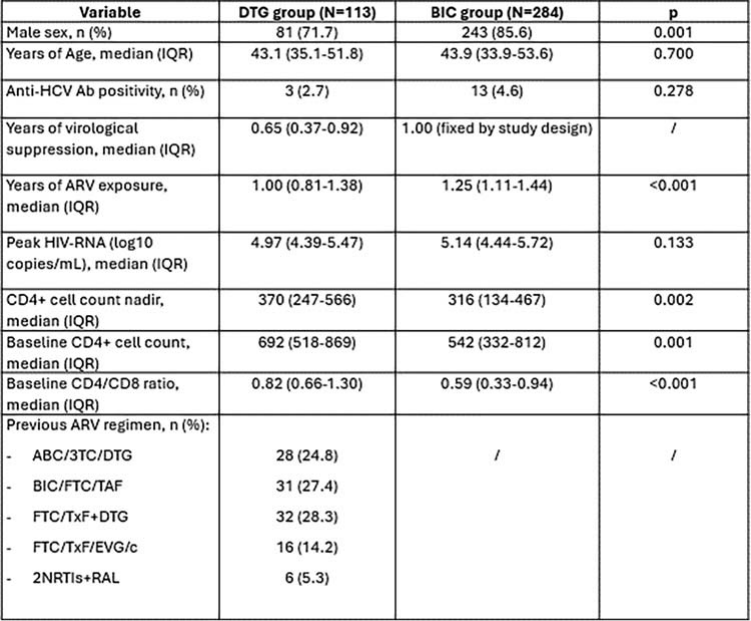
P085 | Population characteristics at baseline.

### P086 Factors Associated With Non‐Treatment With Antiretroviral Therapy Among People Living With HIV COCOVIH2

Catherine Majerholc ^1^, François Prevoteau du Clary^2^, Jean‐Michel Livrozet^3^, David Zucman^1^, Camille Nevoret^4^, Stéphane Bouee^4^, Pierre Leroy^5^

^1^Foch Hospital, Clinical Care, Suresnes, France; ^2^Hôpital La Grave Cité de la santé, Clinical Care, Toulouse, France; ^3^Hôpital Edouard Herriot, Clinical Care, Lyon, France; ^4^CEMKA, Bourg‐la‐Reine, France; ^5^Infectious Diseases, Public Health Department, Groupe Hospitalier Sud Île‐de‐France, Melun, France

**Background**: People living with HIV (PLWH) have benefited for many years from effective treatments allowing stabilization of their disease. However, previous COCOVIH1 results reported that PLWH have an over‐mortality [1,2]. The objective of COCOVIH2 was to report the proportion of PLWH not receiving antiretroviral therapy (ART) and to identify factors associated with the absence of ART.

**Methods**: A cohort of PLWH was identified between 2019 and 2023 using data from the French National Health Data System (SNDS) by using a published and validated algorithm based on ICD‐10 code for HIV, antiretroviral drug specifically indicated for HIV and biological test specific to HIV. Incident patients or those who died during the study period were excluded, as well as individuals without any medication reimbursement in 2023. Untreated subjects for at least 1 given year were defined as individuals with no ART dispensing during at least 1 calendar year between 2019 and 2023.

**Results**: A total of 132,545 PLWH were included in the analysis (mean age 53.8 years, 67% of men). Among them, 8622 (6.5%) had not ART dispensing during at least 1 calendar year between 2019 and 2023. Factors associated with no ART were female sex, younger age, living in the region of Ile de France and overseas, a low socioeconomic status, psychosis and the use of psychotropic substances, lower routine healthcare consumptions but higher emergency healthcare consumptions (Table 1). A multivariate logistic regression led to the same factors.

**Conclusions**: These findings confirm the results of our previous study COCOVIH1 [3] and need specific policy to link these individuals to treatment for HIV infection and to implement public health interventions aimed at improving this situation.

**References**

1. Prevoteau du Clary F, Majerholc C, Zucman D, Livrozet JM, Vallee A, Laurendeau C, Bouee S. Evaluation of HIV antiretroviral treatment adherence in 2019 using anonymized data from the French national health insurance claims data base: the COCOVIH study. HIV Med. 2024 Nov;25(11):1218‐1226. doi: 10.1111/hiv.13690. Epub 2024 Sep 5. PMID: 39234923.

2. Vallée A, Majerholc C, Zucman D, Livrozet JM, Laurendeau C, Bouée S, Prevoteau du Clary F. Mortality and comorbidities in a nationwide cohort of HIV‐infected adults: comparison to a matched non‐HIV adults' cohort, France, 2006−18. Eur J Public Health. 2024 Oct 1;34(5):879‐884. doi: 10.1093/eurpub/ckae031. PMID: 38409963; PMCID: PMC11430913.

3. Majerholc C, et al. Adherence to HIV treatment in 2019: the COCOVIH study using the French national health insurance claims data base [oral presentation]. HIV Glasgow Congress 2022; 23–26 Oct 2022; Glasgow, UK.

**TABLE 1:**
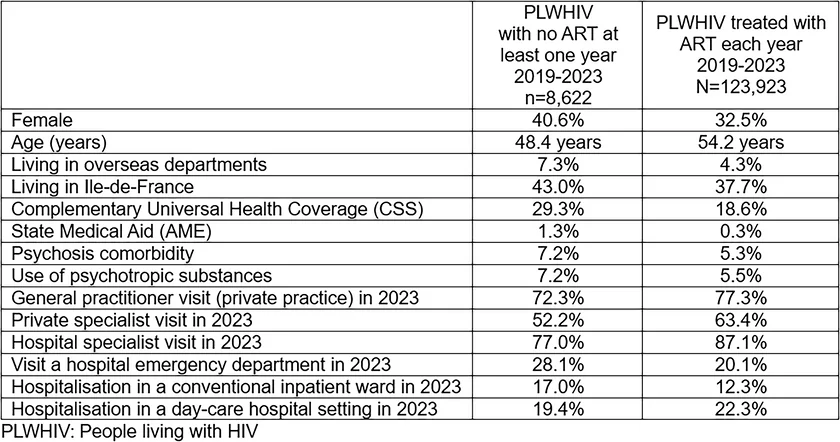
P086 | Main characteristics of both studied populations.

### P087 DISCONT‐LA: Interim Analysis of Discontinuation on Long‐Acting Injectable in Clinical Practice Conditions

Neus Gómez‐Muñoz^1^, Marco Fernández‐Blest ^1^, Ana Moreno Zamora^2^, Santos Del‐Campo‐Terrón^2^, Vicente Estrada‐Pérez^3^, Noemí Cabello‐Clotet^3^, María José Galindo Puerto^4^, Miguel Torralba^5^, Daniel Collado^6^, Marian Fernández‐López^7^, Naya Faro^8^, Jesús Vera‐Méndez^9^, Robert Güerri^10^, Antonio Agustí Adrover^11^, Clara Munoz^12^, Javier Leal Martinez‐Bujanda^12^, Keith Dunn^13^, Miguel García‐Deltoro^1^

^1^Infectious Diseases, General University Hospital of Valencia, Valencia, Spain; ^2^Infectious Diseases, Ramón y Cajal University Hospital, Madrid, Spain; ^3^Internal Medicine, Infectious Diseases, San Carlos Clinical Hospital, Madrid, Spain; ^4^Internal Medicine, University Clinical Hospital of Valencia, Valencia, Spain; ^5^Internal Medicine, Guadalajara University Hospital, Guadalajara, Spain; ^6^Clinical Management Unit for Infectious Diseases, Torrecárdenas University Hospital, Almería, Spain; ^7^HIV Unit (UNITS), Virgen de la Arrixaca University Hospital, El Palmar, Murcia, Spain; ^8^Infectious Diseases, San Cecilio University Hospital, Granada, Spain; ^9^Infectious Diseases, Santa Lucía University General Hospital, Cartagena, Spain; ^10^Infectious Diseases, Hospital del Mar, Barcelona, Spain; ^11^Internal Medicine, Division of Infectious Diseases, Son Llàtzer University Hospital, Palma, Illes Balears, Spain; ^12^Gilead Sciences S.L.U., Madrid, Spain; ^13^Gilead Sciences S.L.U., Boston, MA, US

**Background**: In December 2022, a long‐acting intramuscular injection of cabotegravir+rilpivirine (CAB+RPV) was approved in Spain as a fully suppressive regimen for people living with HIV. Results from clinical trials showed that approximately 7% of participants discontinued CAB+RPV during the first year of therapy. Additionally, there are potential barriers to the implementation and proper monitoring of long‐acting treatment (LA‐ART), such as therapeutic needs, patient preferences and the organization of the healthcare facility. Our aim is to evaluate the cumulative incidence of discontinuation of CAB+RPV, the reasons for discontinuation through real‐world utilization, in order to provide a better care response.

**Methods**: Retrospective, multicentre observational study involving 11 Spanish hospitals. The study included 1200 patients who initiated CAB+RPV between 1 March 2023 and 30 June 2024. Baseline demographic and clinical characteristics were assessed. At 48 weeks, participants were classified as those who discontinued treatment (study group) or those who remained on therapy (control group), with both cohorts followed for an additional 6 months. An interim analysis of baseline data and data after 48 weeks was performed to estimate the cumulative incidence of CAB+RPV discontinuation and its main reasons. Statistical analyses were carried out using the SAS statistical package, V9.4.

**Results**: Table 1 shows demographic and clinical characteristics of the interim analysis. It is noteworthy that at the time of discontinuation, a higher proportion of patients in the study group had a detectable HIV viral load compared with the control group at 48 weeks (18% vs. 8%). The cumulative incidence of LA‐ART discontinuation after 48 weeks was 10% (*n* = 120 patients). In 42% of cases, the reason for discontinuation was the patient's decision, notably transfer to other healthcare facilities (38%), medication‐related adverse effects (24%) such as pain at the injection site or muscle pain; and logistical problems (10%) related to hospital organization. 7.6% of patients discontinued treatment due to virological failure, representing 0.8% of the total sample.

**Conclusions**: In conclusion, the implementation of CAB+RPV requires careful patient selection, adequate logistical management by the hospital and close monitoring of the cohort receiving CAB+RPV.

**TABLE 1:**
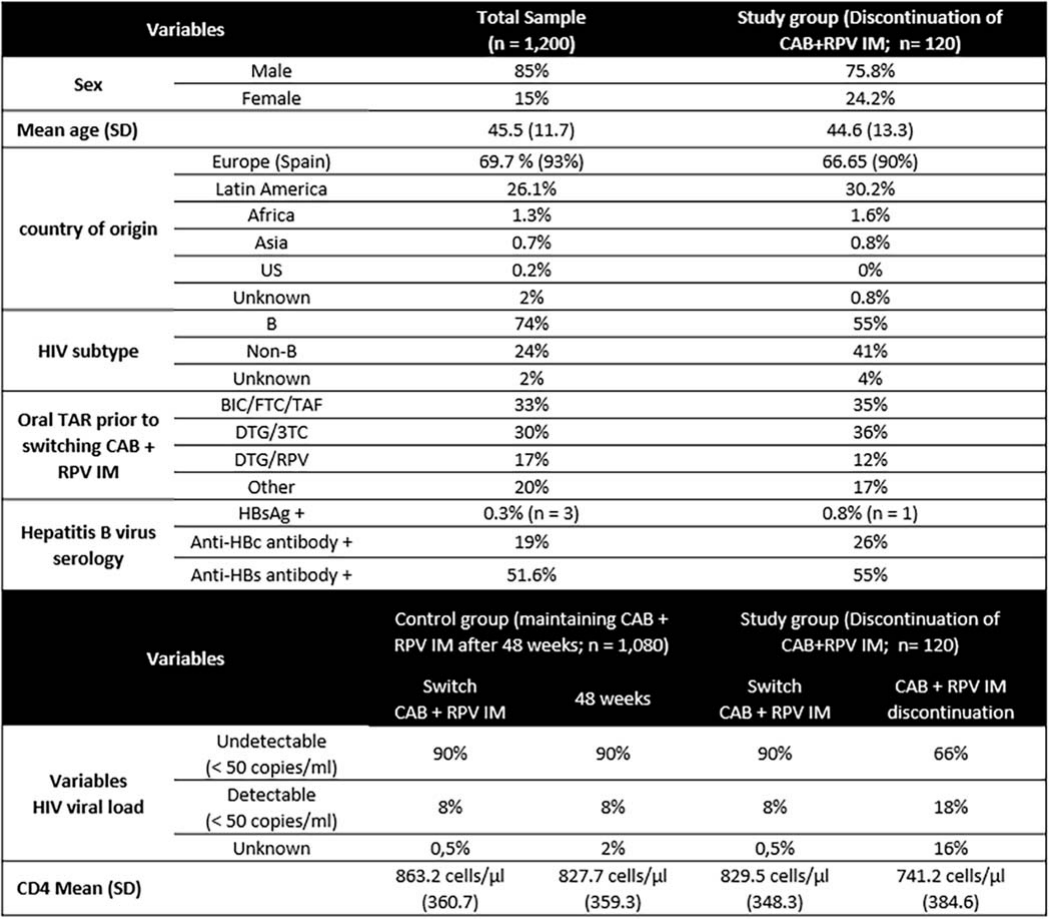
P087 | Demographic and clinical data of the patients included in the study. 3TC, lamivudine; ART‐LA, long‐acting antiretroviral therapy; BIC, bictegravir; DTG, dolutegravir; FTC, emtricitabine; SD, standard deviation; TAF, tenofovir alafenamide; RPV, rilpivirine.

### P088 Real‐World Outcomes of Patients Living With HIV and M184 Mutations Switched to Bictegravir/Emtricitabine/Tenofovir Alafenamide (BIC/FTC/TAF)

Dominic Haigh ^1,2^, Jacob Smiles^1,3^, Nathan Jacobs^1^, Anna Garner^1,3^, Orla McQuillan^1,3^, Ashley McEwan^1,2^, Peter Tilston^1,2^, Shazaad Ahmad^1,2^

^1^Manchester University NHS FT, Manchester, UK; ^2^UK Health Security Agency, Manchester, UK; ^3^The Northern Contraception, Sexual Health & HIV Service, Manchester, UK

**Background**: BHIVA guidelines recommend integrase strand transfer inhibitor (INSTI)‐based antiretroviral therapy. Mutations at codon 184 (M184V/I) are the most common HIV‐1 resistance markers; they confer resistance to cytidine analogues (FTC/3TC) but incur a viral fitness cost. Real‐world data demonstrates good outcomes for patients on BIC/FTC/TAF with an M184V/I mutation [1].

**Methods**: A retrospective database review was conducted across Manchester University NHS Foundation Trust. Historical HIV‐1 resistance genotypes were screened to identify people with M184V, M184I or mixed 184 mutations at any time. Electronic records were analysed for treatment‐experienced transitions to single‐tablet BIC/FTC/TAF monotherapy, pre‐switch viral loads (VLs), treatment duration and reasons for transition. People using BIC/FTC/TAF concurrently with other antiretrovirals or those with incomplete electronic records were excluded. Complex regimen transitions for people with historical resistance mutations are managed via multidisciplinary team (MDT) pathways.

**Results**: Screening of 20,388 records identified 70 unique patients (median age 51 years [IQR 42–60]; 34% female; 44% Black ethnicity). The most common prior regimen was TAF/FTC/DRV/r (40%). Total follow‐up on BIC/FTC/TAF encompassed 34,268 days, with an extensive cumulative cohort exposure of ∼93.7 person‐years with a median follow‐up of 456 days per patient (IQR 13 [SA4] 6−822 days). Primary reasons for switching included commissioning/cost (*n* = 17), adherence/intolerance (*n* = 16), single‐tablet simplification (*n* = 14) and prior virological failure/resistance (*n* = 14).

At their most recent follow‐up, 90% (*n* = 60/70 [SA6]) of patients maintained virological control with a VL < 200 c/mL (60% [*n* = 40] < 50 c/mL; 30% [*n* = 20] 50–199 c/mL). Only four patients (6%) had a VL > 200 c/mL: one had a resolving high baseline VL (> log_10_ 5); one had very early follow‐up (<1 week); one was not in care and unfortunately died from complications related to advanced HIV infection; and one experienced virological failure following a rapid start, developing an emergent T69del mutation. No serious adverse events were caused by BIC/FTC/TAF. One patient with an M184 mutation acquired during TDF/FTC PrEP was successfully maintained.

**Conclusions**: In this real‐world cohort, BIC/FTC/TAF maintained virological control in the vast majority of treatment‐experienced patients with archived M184 mutations and was well tolerated over prolonged follow‐up.

**Reference**

1. Sax P et al. High efficacy of switching to bictegravir/emtricitabine/tenofovir alafenamide in people with suppressed HIV and preexisting M184V/I. AIDS. 2022 Apr 23;36(11):1511‐1520.

### P089 Estimating the Prevalence of Non‐Suppressible Viraemia in Adults Living With HIV‐1 Using a Novel Diagnostic Screenings Assay

Jolanda Voermans ^1^, Jolieke van Osch^1^, Robin Huisman^1^, Laura van Schouwenburg^1^, Bart Rijnders^2,3^, Adam Anas^2,3^, Hannelore Bax^2,3^, Dorine De Vries‐Sluijs^3^, Karin Schurink^3^, Jan Nouwen^3^, Eric van Gorp^1^, Mariana de Mendonca Melo^3^, Lennert Slobbe^3^, Els van Nood^3^, Widia Soochit^1,2^, Daphne Mulders^1^, Carlos Grasa^4^, Pieter Fraaij^1^, Casper Rokx^2,3^, David van de Vijver^1^, Rob Gruters^1^, Thibault Mesplede^1^, Jeroen van Kampen^1^

^1^Department of Viroscience, Erasmus University Medical Center, Rotterdam, Netherlands; ^2^Department of Internal Medicine, Section of Infectious Diseases, Erasmus University Medical Center, Rotterdam, Netherlands; ^3^Department of medical Microbiology and Infectious Diseases, Erasmus University Medical Center, Rotterdam, Netherlands; ^4^Hospital Materno Infantil 12 de Octubre, Hospital Universitario la Paz, IdiPAZ, CIBERINFEC, Madrid, Spain

**Background**: Clonal expansion of defective proviruses can result in the production of non‐infectious particles containing HIV RNA, leading to detectable plasma HIV‐1 viral loads (> 50 copies/mL) despite suppressive antiretroviral therapy (ART), a phenomenon known as non‐suppressible viraemia (NSV) [1]. Defects in the 5’ leader region of the HIV genome are a major cause of NSV. The prevalence of NSV caused by 5’‐leader defects or its clinical implications remain unclear. Therefore, we developed a rapid and sensitive dual probe qPCR assay to screen for 5’‐leader defects in plasma followed by sequencing for confirmation. In this study, we present the prevalence of NSV caused by 5’‐leader defects in people living with HIV (PLWH) who experienced virological rebound or incomplete virological response in a large HIV treatment centre in the Netherlands.

**Methods**: To estimate the prevalence, we performed a systematic analysis on plasma samples collected for routine clinical care between January 2005 and September 2025. We included materials of HIV‐1 infected adults who experienced virological rebound or incomplete virological response while on antiretroviral therapy (ART) (for definitions, see Table 1). Samples that were positive, that is suspected for 5’‐leader defects, in the dual‐probe qPCR screenings assay (log difference ≥0 between the two probes) were confirmed by amplicon‐based next‐generation nanopore sequencing of the qPCR product.

**Results**: A total of 827 PLWH (1046 samples) were included in the study (Figure 1). 74.5% of the samples tested negative for suspected 5’‐leader defects and 8.3% tested positive in the screening assay. Of the positive samples, 78.2% were confirmed by NGS to be proven NSV (32.2%) or possible NSV (46.0%) (for definitions, see Figure 1). The prevalence of NSV (proven and possible) caused by 5’‐leader defects is 6.1% of PLWH in the incomplete virological response group, 6.5% in the virological rebound group and 11.2% in the possible virological rebound group.

**Conclusions**: Our new two‐step approach allows for rapid and sensitive detection of NSV caused by 5’‐leader defects in PLWH. This new approach may facilitate the treatment management of PLWH in selected populations.

**Reference**

1. White JA, Wu F, Yasin S, Moskovljevic M, Varriale J, Dragoni F, Camilo‐Contreras A, Duan J, Zheng MY, Tadzong NF, Patel HB, Quiambao JMC, Rhodehouse K, Zhang H, Lai J, Beg SA, Delannoy M, Kilcrease C, Hoffmann CJ, Poulin S, Chano F, Tremblay C, Cherian J, Barditch‐Crovo P, Chida N, Moore RD, Summers MF, Siliciano RF, Siliciano JD, Simonetti FR. Clonally expanded HIV‐1 proviruses with 5’‐leader defects can give rise to nonsuppressible residual viremia. J Clin Invest. 2023 Mar 15;133(6):e165245. doi: 10.1172/JCI165245. PMID: 36602866; PMCID: PMC10014112.

**FIGURE 1:**
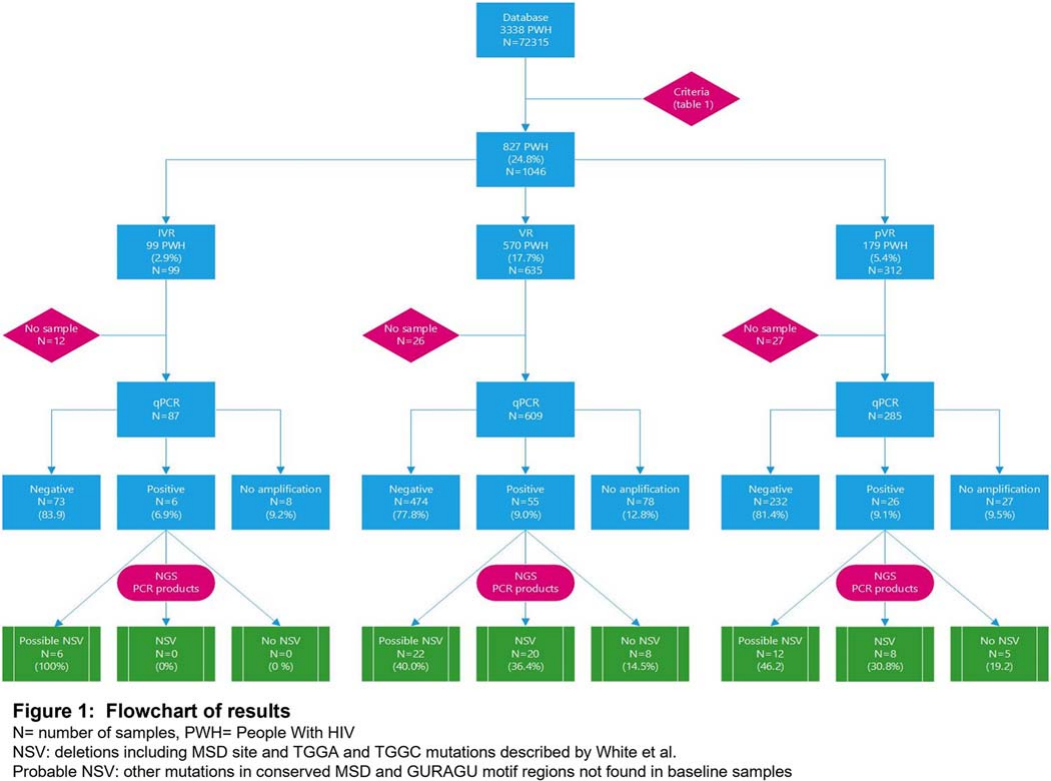
P089 | Flowchart of results.

**TABLE 1:**
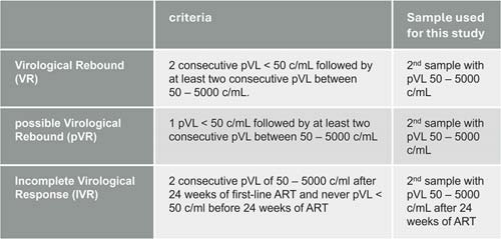
P089 | Inclusion criteria (pVL, viral load in plasma in c/mL).

### P090 Characterizing Resistance‐Associated Mutations and Virological Status Among People Living With HIV Receiving Regimens Containing Bictegravir, Cabotegravir or Dolutegravir

Sara N. Levintow, Paul McDwyer, Ziwei Yin, Wenyi Wang, Michelle L. D'Antoni, Travis Lim

Gilead Sciences, Inc., Foster City, CA, US

**Background**: The risk of resistance is low for regimens containing newer integrase strand transfer inhibitors (INSTIs). In rare instances where resistance‐associated mutations (RAMs) are detected, clinical relevance varies depending on the drug classes affected and levels of resistance conferred. Using real‐world data, we identified RAMs and characterized potential impacts among people living with HIV (PLWH) receiving INSTI‐based regimens.

**Methods**: This study used US insurance claims linked to commercial laboratory records from HealthVerity Marketplace. We included PLWH initiating one of the following regimens between February 2018 and November 2024 regardless of treatment experience: bictegravir/emtricitabine/tenofovir alafenamide (B/F/TAF), cabotegravir/rilpivirine (CAB/RPV), dolutegravir/lamivudine (DTG/TC), dolutegravir/abacavir/lamivudine (DTG/ABC/3TC) or a dolutegravir multi‐tablet regimen. Follow‐up continued until the RAM outcome, regimen switch, insurance disenrolment or end of data. Using genotyping reports and the Stanford HIV Drug Resistance Database (HIVdb), we defined the outcome as the first detection of RAMs to antiretroviral (ARV) components of the regimen ≥90 days after initiation (proxy for on‐treatment emergence). HIVdb genotypic susceptibility scores were assigned based on the individual RAMs and used to classify resistance levels to ARVs. Analyses summarized drug classes impacted, resistance levels and HIV RNA ± 90 days from RAM detection.

**Results**: We included 151,685 PLWH who initiated study regimens (B/F/TAF: *n* = 108,721, CAB/RPV: *n* = 3172, DTG‐based regimens: *n* = 39,792). PLWH were followed for a median 805 (interquartile range: 360–1558) days and 388 (<0.3%) had the RAM outcome. Of these 388, dual‐class resistance was detected concurrently in 38/280 (14%) on B/F/TAF, 28/97 (29%) on DTG‐based regimens and 8/11 (73%) on CAB/RPV (Table 1). Among those with RAMs, high‐level resistance was common for 3TC (93%), FTC (84%), CAB (67%) and RPV (60%) (Figure 1). All 11 with RAMs on CAB/RPV, 83/97 (86%) on DTG‐based regimens and 215/280 (77%) on B/F/TAF had HIV RNA ≥200 copies/mL (Table 1).

**Conclusions**: RAMs were rare in this large real‐world cohort. Where detected, many RAMs conferred high‐level resistance and were frequently associated with viraemia. Findings suggested lesser clinical impacts of RAMs observed on B/F/TAF and DTG‐based regimens compared with CAB/RPV; however, interpretation is limited by small sample sizes, particularly for CAB/RPV.

**FIGURE 1:**
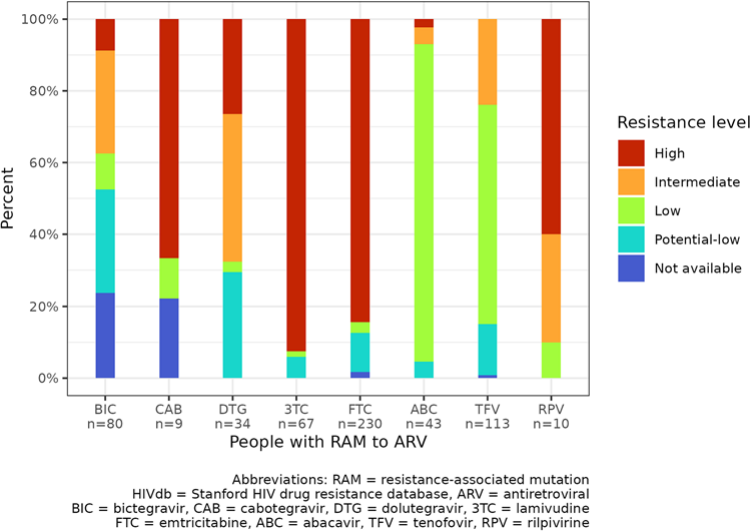
P090 | Resistance classification for people with RAMs from HIVdb genotypic susceptibility score.

**TABLE 1:**
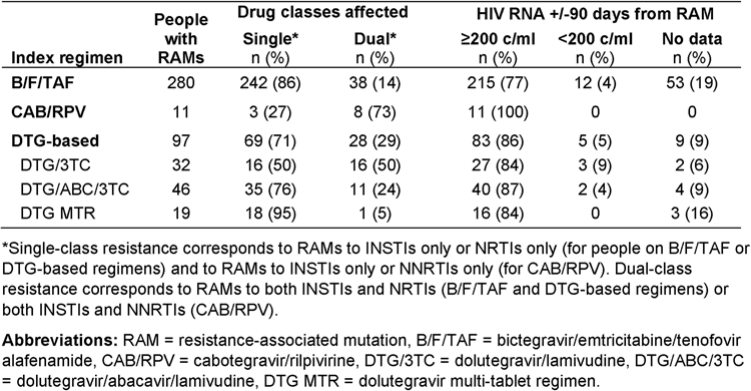
P090 | Drug class resistance and documented viraemia among 388 people with the RAM outcome after initiation of B/F/TAF, CAB/RPV or DTG‐based regimens in 2018–2024.

### P091 Understanding Perspectives and Experiences of Older People Living With HIV: Survey Findings From the Ask Us Europe Study

Chloé Pasin ^1^, Megan Devonald^1^, Melanie Smuk^1^, Rebecca Mbewe^1^, Angelina Namiba^2^, Marcel Dams^3^, Matthew Hodson^4^, Nicoletta Policek^5^, Alain Volny Anne^6,7^, Luís Mendão^8^, Marta Boffito^9^, Simon Collins^10^, Tristan Barber^11,12^, Giovanni Guaraldi^13^, Alexandra Calmy^14^, Stefan Scholten^15^, Silvia Nozza^16^, Ben Collins^17^, Christophe Rouquette^18^, Sarah Brunel^18^, Thierry Tran^18^, Chloe Orkin^1^, on behalf of Ask Us Steering Group

^1^Queen Mary University of London, London, UK; ^2^4 M Mentor Mothers, London, UK; ^3^Independent Sexual Counsellor and Sexual Educator, Cologne, Germany; ^4^Independent, London, UK; ^5^European Community Led Observatory on Ageing and HIV, Edinburgh, UK; ^6^European AIDS Treatment Group, Paris, France; ^7^Ask Us Steering Group, Paris, France; ^8^Ask Us Steering Group, Lisbon, Portugal; ^9^Chelsea and Westminster Hospital NHS Trust, London, UK; ^10^HIV i‐Base, London, UK; ^11^Royal Free London NHS Foundation Trust, London, UK; ^12^Institute for Global Health, University College London, London, UK; ^13^University of Modena and Reggio Emilia, Modena, Italy; ^14^Division of Infectious Diseases, HUG, Geneva, Switzerland; ^15^Praxis Hohenstaufenring, Cologne, Germany; ^16^University Vita Salute San Raffaele Scientific Institute, Milan, Italy; ^17^Reshape, London, UK; ^18^TRT‐5 CHV, Paris, France

**Background**: Older adults with HIV experience complex health concerns yet data in those over 55 is extremely limited. We explored perceptions of treatment optimization discussions and awareness/interest in long‐acting treatment modalities including injectable cabotegravir+rilpivirine (CAB‐RPV).

**Methods**: Between 22 July 2025 and 20 May 2026, people on antiretroviral therapy completed an equity‐focused, co‐produced online survey in 18 European countries and languages developed with 18 community members, European AIDS Treatment Group and the UK Community Advisory Board. Dissemination was via social media and community/clinical networks. We performed descriptive statistics to compare across different age groups and logistic regressions adjusted for confounders for pre‐specified outcomes: CAB‐RPV discussions with HCP and interest (adjusted odds‐ratio [aOR], 95% confidence interval [CI]).

**Results**: Of 1935 participants, 755 (39.0%) were ≥55, including 516 (26.7%) aged 55–64, 194 (10.0%) 65–74 and 45 (2.3%) > 75 (Table 1). Compared to <55, those ≥55 were more likely to be cisgender women (21.6% vs. 16.3%), White (81.1% vs. 70.5%) and financially stable (75.8% vs. 68.4%), with 36.8% diagnosed > 30 years ago; median time on HIV treatment was 24 years (interquartile range [IQR] 16–30). More older participants were taking multi‐pill regimens (23.9% vs. 8.6% in < 55) and fewer reported missed doses (18% vs. 27.7%). 72.7% of ≥55 were clinically eligible for CAB‐RPV (85.1% of < 55). CAB‐RPV uptake decreased with age: 19.2%, 10.1%, 8.8% in < 55, 55–64, ≥65's, respectively. Among those not on CAB‐RPV, older participants were less likely to have discussed CAB‐RPV with their HCP (aOR 0.66, 95% CI: 0.51–0.87) but were no less interested in it (aOR 0.80, 95% CI: 0.62–1.02). Older participants more frequently first heard about CAB‐RPV through HIV community organizations (20.6%, 22.9%, 32.3%) or HIV‐specific newsletters (9.9%, 14.9%, 18.4%); younger participants through news/social media (33.0%, 23.6%, 22.4%) or another person living with HIV (21.1%, 15.4%, 13.8%). Interest in long‐acting Q4‐6 M injectables trended down with age (83%, 71%, 62%), while interest in weekly pills was similar (55%, 56%, 62%) (Figure 1).

**Conclusions**: Older participants were more likely to take multi‐pill regimens. Despite lower uptake of CAB‐RPV and fewer treatment discussions, adjusted interest was high and comparable. Interest in Q4‐6 M injectable therapies decreased with age, while interest in weekly pills was highest in ≥65's. Community organizations play a crucial role in information‐sharing for this population.

**FIGURE 1:**
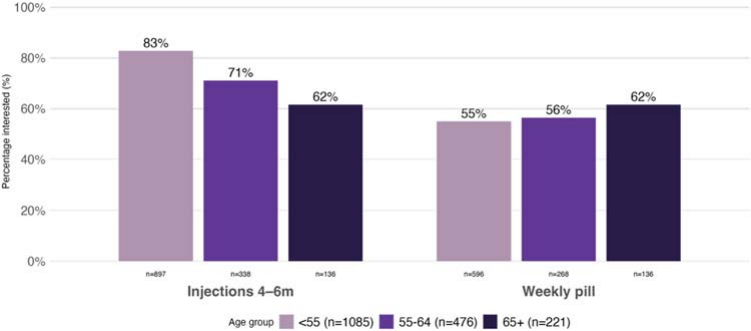
P091 | Percentage of participants interested in future long‐acting modalities by age group.

**TABLE 1:**
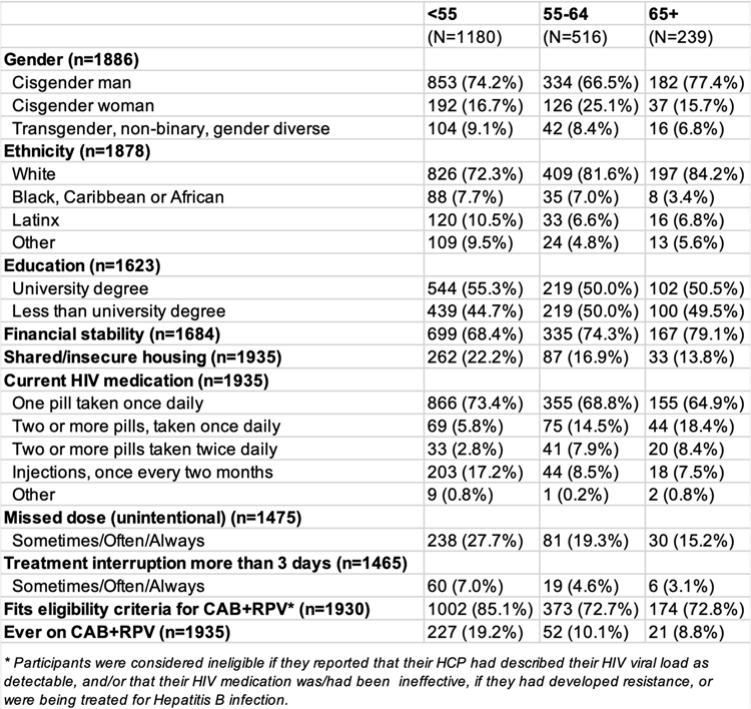
P091 | Demographic characteristics of survey participants in each category.

### P092 96‐Week Effectiveness and Tolerability of Long‐Acting Injectable Cabotegravir Plus Rilpivirine in Young Adults With Perinatally Acquired HIV: A Substudy of the RELATIVITY Cohort

Alejandro De Gea Grela ^1^, Noemí Cabello‐Clotet^2^, María José Galindo Puerto^3^, Jesús Troya García^4^, Rosa María Martínez Álvarez^5^, María Remedios Alemán^6^, María De Lagarde Sebastián^7^, Miguel Torralba^8^, Luis Buzón^9^, María José Crusells Canales^10^, Francisco Fanjul^11^, Adrián Rodríguez^12^, Raúl Parra Fariñas^13^, Mireia Cairó^14^, Maria del Carmen Navarro Sáez^15^, Ignacio De los Santos Gil^16^, Carmen Hidalgo‐Tenorio^17^, Luis Enrique Morano Amado^18^, Enrique Bernal^19^, David Vinuesa^20^, María Josefa De la Asunción^21^, Lucia Fito^22^, Hadrián Pernas Pardavila^23^, Maria Jesus Vivancos^24^, José Ignacio Bernardino^1^, on behalf of RELATIVITY Cohort Study Group

^1^Hospital Universitario la Paz, Madrid, Spain; ^2^Hospital Clínico San Carlos, Madrid, Spain; ^3^Hospital Clínico Universitario, Valencia, Spain; ^4^Hospital Universitario Infanta Leonor, Madrid, Spain; ^5^Hospital Universitario Miguel Servet, Zaragoza, Spain; ^6^Hospital Universitario de Canarias, Tenerife, Spain; ^7^Hospital 12 de Octubre, Madrid, Spain; ^8^Hospital Universitario de Guadalajara, Guadalajara, Spain; ^9^Hospital Universitario de Burgos, Burgos, Spain; ^10^Hospital Universitario Lozano Blesa, Zaragoza, Spain; ^11^Hospital Universitari Son Espases, Palma de Mallorca, Spain; ^12^Hospital Universitari Son Llatzer, Palma de Mallorca, Spain; ^13^Hospital Universitario Marqués de Valdecilla, Santander, Spain; ^14^Hospital Universitario Mutua de Terrasa, Terrassa, Spain; ^15^Hospital Universitario Parc Taulí, Sabadell, Spain; ^16^Hospital Universitario la Princesa, Madrid, Spain; ^17^Hospital Universitario Virgen de las Nieves, Granada, Spain; ^18^Hospital Universitario Álvaro Cunqueiro, Vigo, Spain; ^19^Hospital General Universitario Reina Sofía, Murcia, Spain; ^20^Hospital Clínico San Cecilio, Granada, Spain; ^21^Hospital de Denia Marina Salud, Denia, Spain; ^22^Hospital Universitario de Torrejón, Torrejón de Ardoz, Spain; ^23^Complejo Hospitalario Universitario de Pontevedra, Pontevedra, Spain; ^24^Hospital Universitario Ramón y Cajal, Madrid, Spain

**Background**: Adolescents and young adults with perinatally acquired HIV (PaHIV) have lifelong antiretroviral therapy (ART) exposure, frequent histories of virological failure and archived resistance mutations, factors linked to poorer outcomes. Long‐term real‐world evidence on long‐acting injectable (LAI) cabotegravir (CAB) plus rilpivirine (RPV) in this group is scarce. We evaluated the 96‐week effectiveness and safety of LAI CAB+RPV in PaHIV within a national cohort.

**Methods**: We conducted an ambispective, observational, multicentre study nested within the RELATIVITY cohort across 58 Spanish hospitals. Adults with PaHIV were compared directly with adults without documented perinatal HIV acquisition (non‐PaHIV). Virological effectiveness, immunological evolution, injection adherence, safety and treatment discontinuation were assessed over 96 weeks of follow‐up.

**Results**: Twenty‐eight of 3349 participants had PaHIV and 3321 were non‐PaHIV (comparison group); the median follow‐up was 23.7 months in PaHIV and 25.1 months in non‐PaHIV. Adults with PaHIV were younger, predominantly women and had markedly longer cumulative ART exposure, more previous virological failure and more archived nucleoside (NRTI) and non‐nucleoside reverse transcriptase inhibitor (NNRTI) resistance‐associated mutations (Table 1). The most frequent reasons for switching were improved comfort or quality of life and patient preference. Despite this less favourable profile, detectable HIV‐1 RNA > 50 copies/mL was rare and treatment injection adherence was similar between groups. At month 25, none of the PaHIV participants (0/4) had HIV‐1 RNA > 50 copies/mL, compared with 29/738 (3.9%) in the non‐PaHIV group (*p* = 1.000). Only one virological failure occurred (3.6%), which re‐suppressed on a boosted regimen. Permanent discontinuation occurred in seven adults with PaHIV (25.0%): one for virological failure and six for non‐virological reasons.

**Conclusions**: In this real‐world cohort, LAI CAB+RPV effectiveness was comparable despite the more complex historical treatment profiles in PaHIV. Toxicity was uncommon and although permanent discontinuation was more frequent, it was mainly driven by non‐virological reasons rather than injection‐site reactions. Clinicians should consider LAI CAB+RPV as part of an individualized treatment strategy for young adults with PaHIV, supported by proactive counselling around pregnancy planning and flexible, accessible injection scheduling to sustain therapy.

**TABLE 1:**
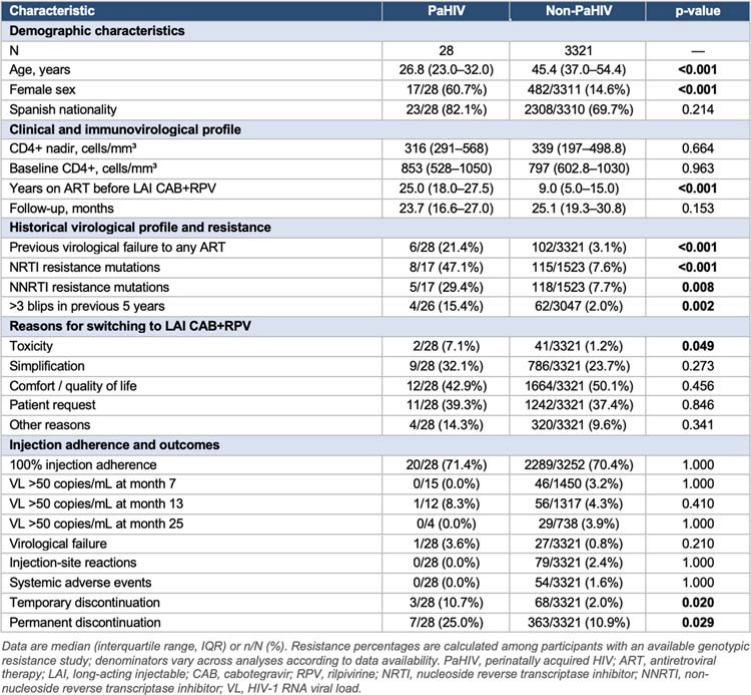
P092 | Baseline characteristics and follow‐up outcomes in adults with PaHIV versus non‐PaHIV individuals.

### P093 Detected but Not Predictive? Baseline NGS Resistance and Virological Suppression With B/F/TAF in Treatment‐Naïve People With HIV

Giovanni Massobrio^1^, Marianna Giurco ^1^, Vincenzo Paolozzi^2^, Eleonora Pratolongo^1^, Valentina Ricucci^2^, Federica Stefanelli^2^, Nadia Randazzo^2^, Matteo Bassetti^1,3^, Lucia Taramasso^3^, Antonio Di Biagio^1,3^

^1^Department of Health Sciences (DISSAL), University of Genoa, Genoa, Italy; ^2^Hygiene Unit, IRCCS Azienda Ospedaliera Metropolitana, Genoa, Italy; ^3^Department of Specialist Medicine, Infectious Disease Clinic, IRCCS Azienda Ospedaliera Metropolitana, Genoa, Italy

**Background**: Bictegravir/emtricitabine/tenofovir alafenamide (B/F/TAF) is a preferred first‐line regimen in treatment‐naïve people living with HIV (PLWH), combining a high genetic barrier with strong virological efficacy. Next‐generation sequencing (NGS) detects resistance variants present below the 20% prevalence threshold of Sanger sequencing, but their clinical relevance in this setting remains debated.

**Methods**: Retrospective monocentric cohort study of treatment‐naïve PLWH initiating B/F/TAF (2022–2025) with baseline NGS resistance testing. HIV‐RNA was measured at baseline, weeks 4, 16, 24 and 48 (suppression < 30 copies/mL). Viral load kinetics were analysed by Kaplan–Meier/log‐rank and Mann–Whitney U‐tests; a multivariate Cox model assessed CD4 count, HIV‐RNA category and resistance mutations as predictors.

**Results**: Among 133 treatment‐naïve PLWH identified, 121 with complete follow‐up data were included: 87 male; median age 40 years (IQR 32–53), median CD4 count 224 cells/µL (IQR 97–386) and median baseline HIV‐RNA 230,000 copies/mL (IQR 46, 300–880,000). Virological suppression rates were 44%, 79%, 84% and 92% at weeks 4, 16, 24 and 48, respectively. Baseline NGS detected resistance mutations in 39 participants (32%). In 7 participants (6%), at least one mutation was present below the 20% threshold, including INSTI mutations (E138K and Q148H). Resistance mutations showed only a borderline association with suppression kinetics, reflecting a trend towards slower early suppression at week 4 (*p* = 0.054; log‐rank *p* = 0.081), with convergence by week 12. No significant difference was observed across groups defined by mutation prevalence threshold: wild type, < 20% and ≥20% (log‐rank *p* = 0.21). In multivariable analysis, baseline HIV‐RNA was the only independent predictor of time to suppression (Figure 1). CD4 count and resistance mutations were no longer significant after adjustment for viraemia, suggesting that the univariable CD4 association was explained by confounding HIV‐RNA (Table 1).

**Conclusions**: Baseline NGS uncovered resistance signals invisible to Sanger sequencing, but these did not translate into clinically meaningful differences in virological suppression with B/F/TAF. In this cohort, baseline viraemia—not CD4 count, transmitted resistance or mutation prevalence threshold—was the main determinant of viral suppression kinetics. These data suggest that, in treatment‐naïve PLWH starting B/F/TAF, low‐prevalence resistance detected by NGS may not be decisive for early viral suppression outcome. Larger studies are needed to confirm these findings, particularly for sub‐20% mutations.

**FIGURE 1:**
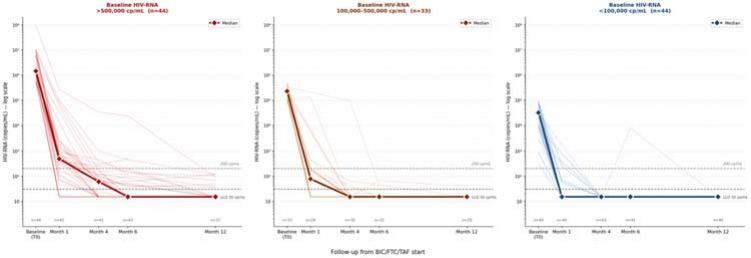
P093 | HIV‐RNA decay kinetics in treatment‐naïve patients on BIC/FTC/TAF by baseline HIV‐RNA category.

**TABLE 1:**
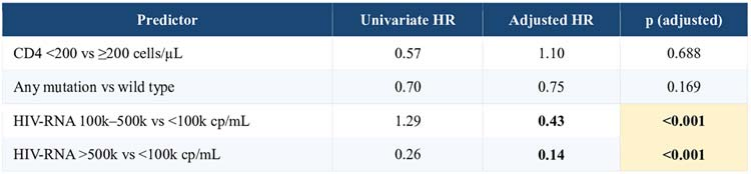
P093 | Predictors of time to virological suppression (<30 cp/mL).

### P094 Patient‐Reported Outcomes in Treatment‐Naïve People With HIV Initiating B/F/TAF: A Pooled Analysis of the BICSTaR Cohort

Benoit Trottier^1^, Ayşe Deniz Gökengin^2^, Diana Canetti^3^, Tomoyuki Endo^4^, Shu‐Hsing Cheng^5^, Eynat Kedem^6^, Antonio Antela López^7^, Jan Den Hollander^8^, Daniel Beer^9^, Arthur Jackson^10^, Arnaud Desclaux^11^, David Thorpe^12^, Andrea Marongiu ^13^, Rebecca Harrison^14^, Mo Hu^15^, Yeon‐Sook Kim^16^

^1^Département de Recherche, Clinique de Médecine Urbaine du Quartier Latin, Montreal, QC, Canada; ^2^Department of Infectious Diseases and Clinical Microbiology, Ege University, Izmir, Türkiye; ^3^Department of Infectious Diseases, IRCCS San Raffaele Scientific Institute, Milan, Italy; ^4^Department of Infection Control and Prevention, Hokkaido University Hospital, Sapporo, Japan; ^5^Department of Infectious Diseases, Taoyuan General Hospital, Taoyuan City, Taiwan; ^6^HIV Clinic, Rambam Healthcare Campus, Haifa, Israel; ^7^Infectious Diseases Unit, University Clinical Hospital of Santiago, University of Santiago de Compostela, Santiago de Compostela, Spain; ^8^Department of Internal Medicine and Infectious Diseases, Maasstad Hospital, Rotterdam, Netherlands; ^9^PZB Aachen, Praxis/Labor Dr. med. Heribert Knechten, Aachen, Germany; ^10^Consultant in Infectious Diseases, Cork University Hospital, Cork, Ireland; ^11^Service des Maladies Infectieuses et Tropicales, CHU de Bordeaux, Bordeaux, France; ^12^Medical Affairs Research, Gilead Sciences Europe Ltd, Uxbridge, UK; ^13^Real World Evidence Virology Department, Gilead Sciences Europe Ltd, Uxbridge, UK; ^14^Real World Evidence, IQVIA, London, UK; ^15^Real World Evidence, IQVIA, Chicago, IL, US; ^16^Division of Infectious Diseases, Department of Internal Medicine, Chungnam National University School of Medicine, Daejeon, Republic of Korea

**Background**: Observational patient‐reported outcome (PRO) data in newly diagnosed people with HIV (PWH) are limited, but could provide insights into health and quality of life. BICSTaR, a large, multicountry, observational study evaluated effectiveness, safety and PROs of B/F/TAF in PWH during routine clinical practice. We report PROs in treatment‐naïve participants.

**Methods**: Mental/physical health (24 months [M]), HIV symptom burden (12 M) and treatment satisfaction (3 M) were assessed using the 36‐Item Short Form Health Survey (mental/physical component summary [MCS/PCS]), HIV Symptom Index (HIV‐SI) and HIV Treatment Satisfaction Questionnaire status (HIVTSQs), respectively. Descriptive statistics and logarithmic mixed models, adjusted for confounding and interaction, were used to evaluate changes in outcomes over 24 M and by baseline CD4 count, viral load (VL) and rapid initiation status. Model interaction terms were retained if *p* < 0.1.

**Results**: Among 482 participants (median age 36 years, 73% White, 91% male), 407 had MCS/PCS data for modelling, 334 had HIV‐SI data at baseline and 12 M and 356 had HIVTSQs data at 3 M. At baseline, 48% had VL > 100,000 copies/mL and 27% had CD4< 200 cells/µL; 116 (27%) initiated ART within 7 days of HIV diagnosis (rapid initiation). Mean (95% CI) unadjusted MCS and PCS scores increased over 24 M by +5.8 (4.1–7.1) and +3.3 (2.4–4.2), respectively. Baseline adjusted MCS scores were lowest in participants with rapid initiation and CD4< 200 cells/µL and increased quickest through 24 M versus other groups (+9.8 [95% CI: 6.9–13.1]) (Figure 1, panel A). Improvements in adjusted PCS scores were more pronounced (albeit from lower baseline) in participants with more advanced HIV (CD4< 200 cells/µL and VL > 100,000 c/mL) versus other groups (+7.7 [95% CI: 5.7–9.7]) (Figure 1, panel B). Median (IQR) baseline HIV‐SI score (possible range: 0–20) was 5 (2–9) at baseline with a median (IQR) decrease of −2 (−5 to 0) at 12 M. Median (IQR) HIV‐SI change at 12 M in participants with rapid initiation and non‐rapid initiation was −2 (−6 to 0) and −1 (−5 to 0), respectively. Median (IQR) HIV treatment satisfaction score was high at 3 M (55 [50–59]; *n* = 356).

**Conclusions**: PROs improved over 24 M following initiation of B/F/TAF in treatment‐naïve PWH.

**FIGURE 1:**
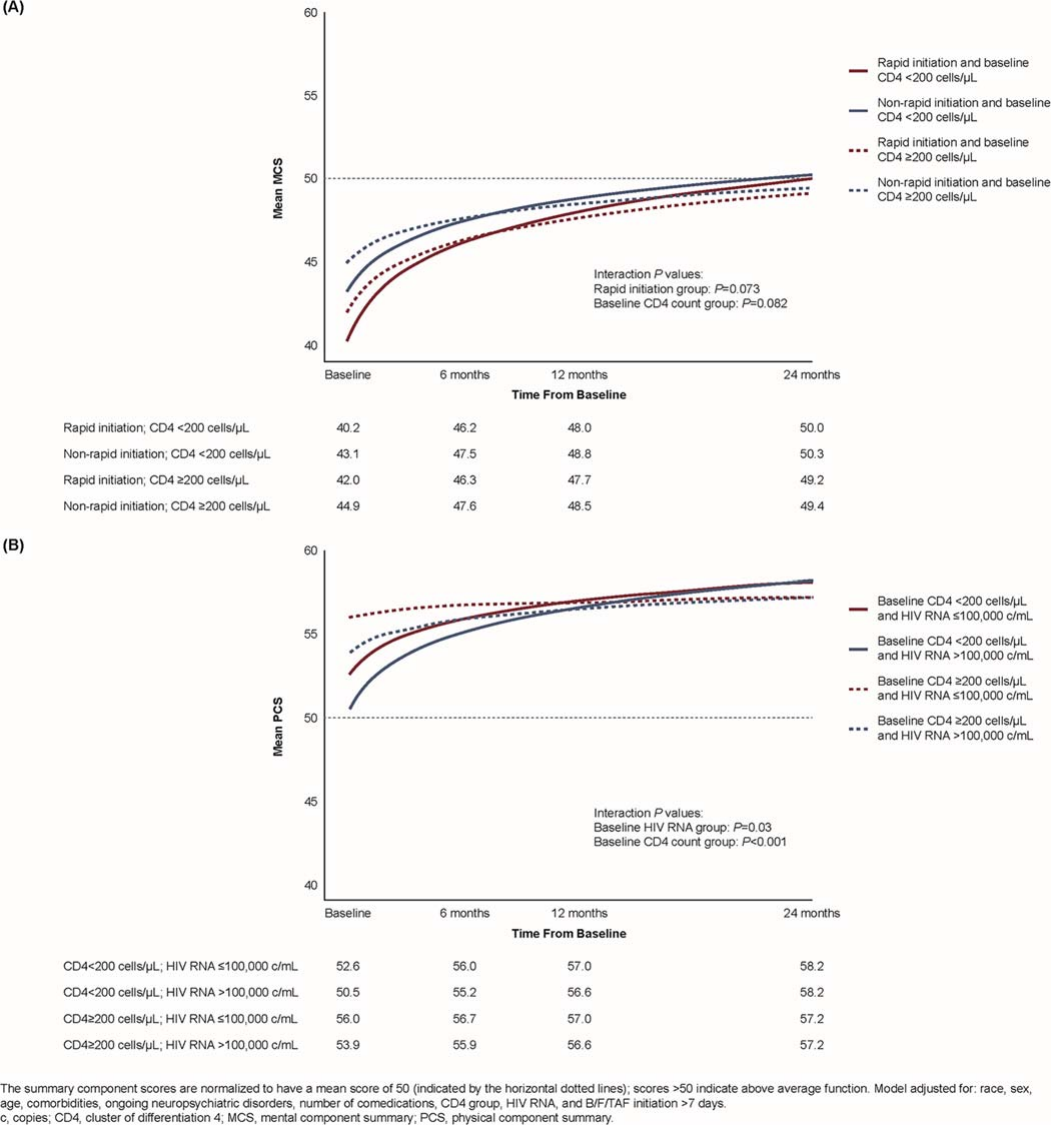
P094 | BICSTaR adjusted (A) MCS and (B) PCS scores (log time model).

### P095 BICSTaR: 24‐Month Effectiveness and Tolerability of B/F/TAF by HIV Diagnosis Era

Itzchak Levy^1,2^, Katsuji Teruya^3^, Marta Boffito ^4^, Chia‐Jui Yang^5,6^, Giulia C. Marchetti^7^, Miguel Garcia Deltoro^8^, Malcolm Hedgcock^9^, Roger Vogelmann^10,11^, Laurent Hocqueloux^12^, Arthur Jackson^13^, Shin‐Woo Kim^14^, Chiaw Yee Choy^15,16^, Ahmet Çağkan İnkaya^17^, David Thorpe^18^, Andrea Marongiu^19^, Tali Cassidy^19^, Berend van Welzen^20^

^1^Infectious Disease Unit, Sheba Medical Center, Tel Aviv, Israel; ^2^Gray School of Medicine, Tel Aviv University, Tel Aviv, Israel; ^3^AIDSClinical Center, National Center for Global Health and Medicine, Tokyo, Japan; ^4^Chelsea and Westminster Hospital NHS Foundation Trust, London, UK; ^5^Department of Internal Medicine, Far Eastern Memorial Hospital, New Taipei City, Taiwan; ^6^School of Medicine, National Yang Ming Chiao Tung University, Taipei, Taiwan; ^7^Department of Health Sciences, University of Milan, Milan, Italy; ^8^Infectious Diseases Department, Consorcio General University Hospital, Valencia, Spain; ^9^Spectrum Health, Vancouver, BC, Canada; ^10^Mannheimer Onkologie‐Praxis, Mannheim, Germany; ^11^Department of Medicine II, Medical Faculty Mannheim, University Medical Center Mannheim, Heidelberg University, Mannheim, Germany; ^12^Division of Tropical and Infectious Diseases, University Hospital of Orléans, Orléans, France; ^13^Department of Infectious Diseases, Cork University Hospital, Cork, Ireland; ^14^Division of Infectious Diseases, Department of Internal Medicine, Kyungpook National University Hospital, Daegu, Republic of Korea; ^15^Department of Infectious Diseases, National Centre for Infectious Diseases, Singapore, Singapore; ^16^Department of Infectious Diseases, Tan Tock Seng Hospital, Singapore, Singapore; ^17^Department of Infectious Diseases and Clinical Microbiology, Hacettepe University Faculty of Medicine, Ankara, Türkiye; ^18^Global HIV Medical Affairs, Gilead Sciences Europe Ltd, Uxbridge, UK; ^19^Real World Evidence, Gilead Sciences Europe Ltd, Uxbridge, UK; ^20^Department of Infectious Diseases, University Medical Centre Utrecht, Utrecht, Netherlands

**Background**: People who have lived with HIV for many years and were diagnosed in earlier treatment eras may have longer exposure to antiretroviral therapy (ART), greater cumulative viraemia, more complex treatment histories and a higher comorbidity burden compared with those who were more recently diagnosed. We evaluated the 24‐month effectiveness and tolerability of bictegravir/emtricitabine/tenofovir alafenamide (B/F/TAF) among treatment‐experienced (TE) participants in the prospective, observational BICSTaR study who were diagnosed pre‐ or post‐introduction of highly active ART (HAART).

**Methods**: TE participants were stratified by diagnosis era: pre‐HAART (pre‐1996), post‐HAART/pre‐START (Strategic Timing of Antiretroviral Treatment) study (1996–2014) and post‐START (≥2015). 24‐month outcomes included: virologic suppression (HIV‐1 RNA <50 copies/mL, missing = excluded), changes in CD4 cell count and CD4/CD8 ratio, drug‐related adverse events (DRAEs) and discontinuations. Diagnosis era was used descriptively to reflect correlated factors including age, time living with HIV and ART experience; analyses were not intended to estimate causal effects.

**Results**: Overall, 1580 TE participants from 14 countries were included (171 pre‐HAART, 1037 post‐HAART/pre‐START, 372 post‐START). Those diagnosed pre‐HAART had a higher median age and higher median numbers of prior ART regimens, concomitant medications and comorbidities/coinfections at B/F/TAF initiation compared with those diagnosed post‐HAART/pre‐START and post‐START (Table 1). At 24 months, of those diagnosed pre‐HAART, post‐HAART/pre‐START and post‐START, virologic suppression was achieved by 97.9% (139/142), 95.7% (783/818) and 96.1% (270/281), respectively (Figure 1). Median (Q1, Q3) changes in CD4 count were +16.0 (–87.5, 136.0), +38.0 (–90.0, 144.0) and +52.0 (–22.0, 185.0) cells/µL, respectively; and changes in CD4/CD8 ratio were +0.03 (–0.08, 0.16), +0.05 (–0.06, 0.16) and +0.12 (0.00, 0.27), respectively. DRAEs occurred in 11.7% (20/171), 11.6% (120/1037) and 15.6% (58/372) of participants, respectively, with one serious DRAE reported (depression; post‐HAART/pre‐START group); discontinuation due to DRAEs occurred in 3.5% (6/171), 5.0% (52/1037) and 7.0% (26/372) of participants, respectively. No treatment‐emergent resistance was reported among those assessed.

**Conclusions**: B/F/TAF demonstrated high rates of virologic suppression and was well tolerated across diagnosis eras, including among participants who were diagnosed pre‐1996, who were older and more treatment experienced.

**FIGURE 1:**
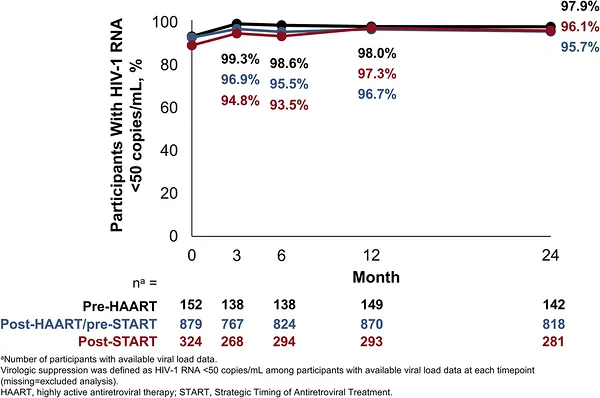
P095 | Virologic effectiveness through 24 months.

**TABLE 1:**
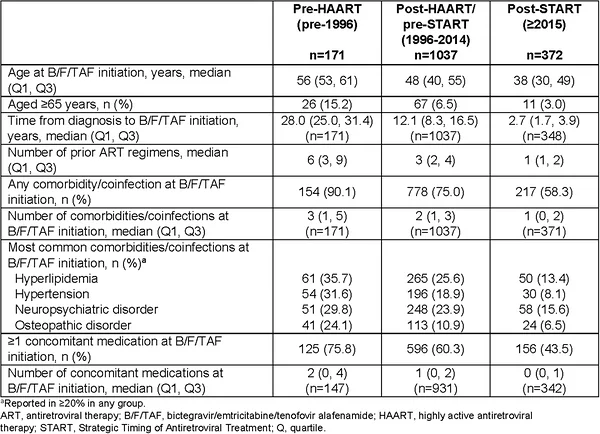
P095 | Baseline demographic and clinical characteristics by HIV diagnosis era.

### P096 Effectiveness of Using a Multidisciplinary Care Model for Engaging Unstable Individuals in HIV Treatment With Bictegravir/Emtricitabine/Tenofovir Alafenamide

Christina Wiesmann^1,2^, Shana Yi^2^, David Truong^2^, Brian Conway ^1,2^

^1^Simon Fraser University, Burnaby, Canada; ^2^Vancouver Infectious Diseases Centre, Vancouver, Canada

**Background**: Human immunodeficiency virus (HIV) infection continues to represent one of the most significant public health challenges worldwide. Recent data indicates that close to 60% of PWID living in BC have an unsuppressed HIV infection. Bictegravir/emtricitabine/tenofovir alafenamide (B/F/TAF) is one of the most effective and tolerable single tablet regimens (STRs) available.

**Methods**: This study was launched as a prospective observational study to assess the feasibility, scalability and operational success of using an integrated, multidisciplinary model of HIV care among inner‐city residents with a history of substance use. Participant recruitment, demographics and programme feasibility metrics are included in this interim analysis of the study.

**Results**: From January 2024 until June 2025, we had enrolled a total of 60 individuals in the study. 13 of these individuals were identified and recruited at community pop‐up clinics and the remaining 47 individuals were already receiving care through the Vancouver Infectious Diseases Centre (VIDC), the study location. At baseline, 83% of individuals (39) had an undetectable HIV viral load; one individual had low level viraemia, with a viral load of 52 copies/mL and 7 individuals were experiencing virological failure (Table 1). Following study baseline visits, we have had good success keeping individuals engaged in care (60/65). In general, we see that the majority of study visits are completed on time and that relatively few study visits are missed (Figure 1).

**Conclusions**: The ability to steadily recruit new participants every month and high rates of retention in care demonstrates the ongoing need for low‐barrier access to programmes that help individuals improve their engagement in HIV treatment.

**FIGURE 1:**
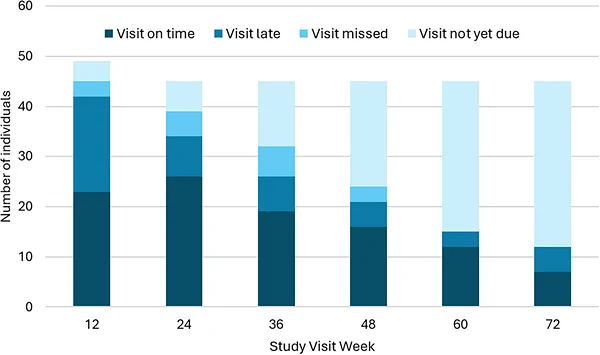
P096 | Summary of visit timeline projections and status (on time, late, missed) of study visits completed until the end of June 2025.

**TABLE 1:**
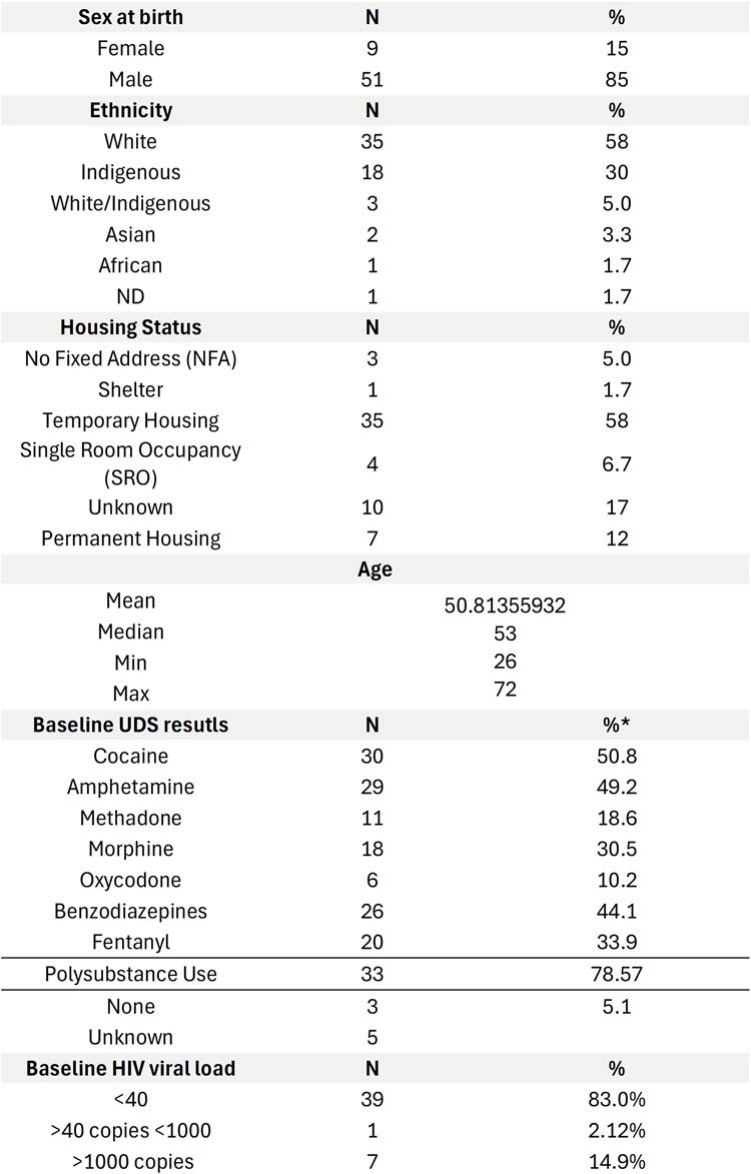
P096 | Demographics and lab results of study participants at baseline.

### P097 Patient‐Reported HIV Symptoms Distress Decreases After Switching to Bictegravir/Emtricitabine/Tenofovir Alafenamide (B/F/TAF) in HIV‐1 Infected Marginalized Individuals Living in Vancouver, Canada

Christina Wiesmann^1,2^, David Truong^2^, Brian Conway ^1^

^1^Simon Fraser University Faculty of Health Sciences, Burnaby, Canada; ^2^Vancouver Infectious Diseases Centre, Vancouver, Canada

**Background**: The single tablet combination of bictegravir, emtricitabine and tenofovir alafenamide (B/F/TAF) has been used as a switch strategy among patients experiencing dose‐limiting toxicities of their current regimens or wanting to simplify these regimens, to improve tolerability and adherence. Assessing improvements in patient‐reported outcomes (PROs) after switching to B/F/TAF is important for understanding patient satisfaction following the switch.

**Methods**: We conducted a prospective evaluation of the safety, efficacy and tolerability of B/F/TAF among 45 HIV‐infected PWUD who had experienced incomplete virologic suppression or low adherence on previous therapy, with B/F/TAF being delivered in the context of a multidisciplinary programme of care to support adherence to medications and engagement in care. To determine participants’ overall satisfaction and quality of life while on B/F/TAF, we used the HIV systems distress module (HIV‐SDM), a validated tool which measures 20 different HIV symptoms and overall quality of life. A paired *t*‐test was used to determine if the average score changed after study enrolment relative to baseline. We also calculated the proportion of individuals experiencing each symptom at baseline and at weeks 36 and 72.

**Results**: We saw a significant reduction (5.4 point [25%] decrease, from 21.6 to 16.2) in average total scores after study enrolment (*p* = 0.007), indicating that overall average HIV symptom distress decreased following study enrolment. We saw a consistent reduction (i.e. lower percentage of individuals reporting to experience each symptom at both weeks 36 and 72 relative to baseline) in the number of individuals experiencing hair loss, weight loss, changes in the way their body looks (body image), sex problems, bloating/gas, headaches, skin problems, anxiety, sadness, diarrhoea, nausea, dizziness, fevers and fatigue (14/20 symptoms) (Figure 1).

**Conclusions**: Our results demonstrated that switching to B/F/TAF and engagement in the multidisciplinary care model offered by VIDC leads to a clear and consistent reduction in HIV symptom distress among marginalized individuals. These results emphasize the importance of ensuring that all individuals have access to improved HIV treatment regimens as they become available to increase client satisfaction and ultimately improve medication adherence, viral suppression and overall quality of life.

**FIGURE 1:**
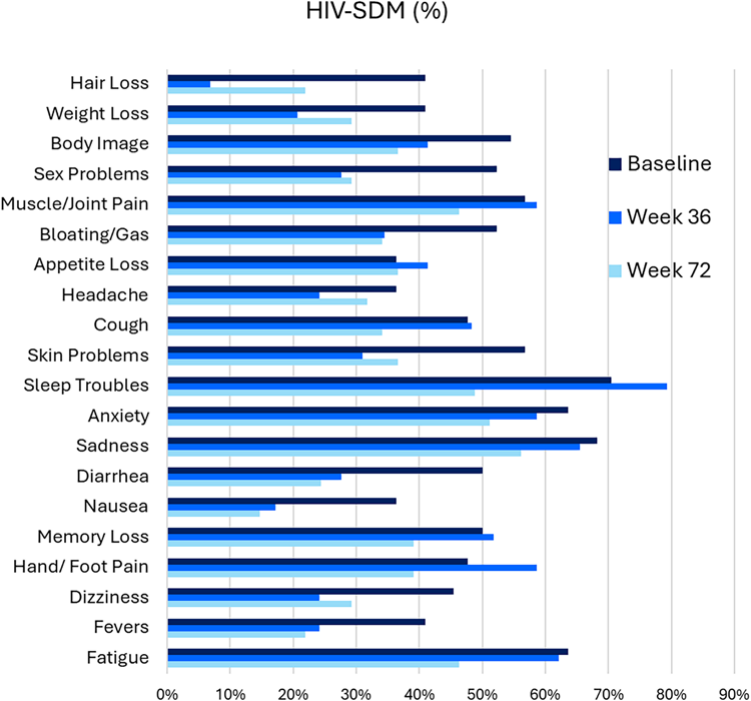
P097 | Percentage of questionnaire respondents reportedly experiencing each symptom (i.e. giving a score between 1 and 4) at baseline and weeks 36 and 72.

### P098 Investigation of the Effectiveness of a Once‐a‐Day Bictegravir/Emtricitabine/Tenofovir Alafenamide Treatment Regimen in Combination With a Multidisciplinary Model of Care for Improving HIV Viral Suppression

Christina Wiesmann^1,2^, David Truong^2^, Brian Conway ^1,2^

^1^Simon Fraser University, Burnaby, Canada; ^2^Vancouver Infectious Diseases Centre, Vancouver, Canada

**Background**: Providing healthcare to vulnerable inner‐city populations, characterized by unstable housing and high rates of infections, requires innovative care models to address their complex medical and social needs. Traditional healthcare systems have proven inadequate for this population, particularly when it comes to ensuring consistent antiretroviral therapy (ART) adherence, as well as ensuring that individuals are prescribed an HIV medication which effectively suppresses their infection. The Vancouver Infectious Diseases Centre (VIDC) has developed a multidisciplinary care model centred on addressing social, psychological and addiction‐related needs in addition to medical care.

**Methods**: This is a single‐arm, prospective, observational study. All participants enrolled in the study have previous indication of non‐adherence to their HIV medication and/or a detectable viral load in the past year. The primary endpoint is virologic suppression (HIV plasma viral load < 200 copies/mL) at 24 weeks.

**Results**: As of June 2026, a total of 56 individuals had been enrolled in the study for 24 weeks and are included in this analysis. At baseline, 11/56 individuals (19.6%) were viraemic (viral load [VL] above 200 copies/mL). 51 individuals have had week 12 bloodwork and 5/51 (10%) were viraemic (VL > 200 copies/mL), while 39 individuals have completed their week 24 visit and 6/39 (15%) were viraemic (Figure 1). We surveyed individuals with virologic failure to determine reasons for this including competing priorities, dementia, inability to go to the pharmacy due to other health conditions, losing pills and switching pharmacies without having medication switched to the new pharmacy, among others.

**Conclusions**: Many individuals living in Vancouver's inner‐city have an unsuppressed HIV viral load and/or are non‐adherent to their medication. Prescribing B/F/TAF in combination with a multidisciplinary care model may help to achieve increased rates of HIV suppression among this vulnerable population. Individualized care plans are required for optimizing treatment adherence; however, there are many systemic and individual factors which can lead to poor adherence.

**FIGURE 1:**
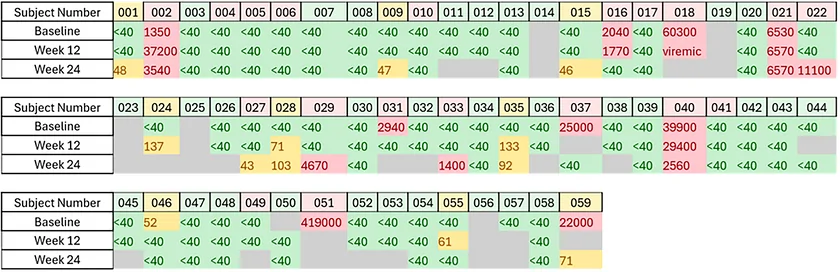
P098 | Participant viral loads up to 24 weeks. Viral loads are measured in copies/mL. Grey cells indicate that the viral load was not measured at the indicated study time point.

### P099 Novel Bioinformatic Pipeline Identifies INSTI Resistance Mutation G140R as a Minority Variant in HIV‐1 Sub‐Subtype A6 and Distinguishes Between APOBEC Hypermutation and True DRM

Anna Weibull Wärnberg ^1,2^, Bryant Oh Zhi Yang^2,3^, Annie Hsieh^4^, Piotr Nowak^1,2^, Anders Sönnerborg^2,4^, Emmi Andersson^2,4^, Maarten van de Klundert^2^

^1^Department of Infectious Diseases, Karolinska University Hospital, Stockholm, Sweden; ^2^Department of Medicine Huddinge, Karolinska Institutet, Stockholm, Sweden; ^3^School of Biological Sciences, Nanyang Technological University, Singapore, Sweden; ^4^Department of Clinical Microbiology, Karolinska University Hospital, Stockholm, Sweden

**Background**: HIV‐1 sub‐subtype A6 (HIV‐1A6) has emerged as the predominant variant in the WHO Eastern European region and is increasing in Western Europe, mostly due to migration from Ukraine since 2022 [1]. HIV‐1A6 is associated with an increased risk of virological failure on long‐acting injectable integrase strand inhibitor (INSTI) cabotegravir co‐administered with rilpivirine (LA‐CAB/RPV) [2]. Archived drug resistance mutations (DRMs) are a risk factor for virological failure and can be detected by proviral DNA sequencing. However, interpretation is complicated by hypermutated sequences containing G‐to‐A mutations introduced by the antiviral defence protein APOBEC and can be difficult to distinguish from clinically relevant DRMs [3]. The INSTI resistance mutation G140R strongly reduces HIV‐1 replication capacity, but the fitness cost is lower in HIV‐1A6, especially in the presence of L74I, which is a characteristic polymorphism in HIV‐1A6 [4].

**Methods**: HIV‐1 DNA from 24 people with HIV‐1A6 and 36 people with non‐A6 HIV‐1 variants were sequenced by NGS (Table 1). DRMs were detected using a novel in‐house bioinformatic pipeline developed to identify minority variants. The pipeline distinguishes between APOBEC‐induced G‐to‐A mutations (AGA) and non‐APOBEC‐related G‐to‐A mutations (NAGA) by analysing G‐to‐A mutations co‐occurring in the same reads in order to define G‐to‐A mutations as true DRM or not.

**Results**: Excluding L74I, a total of 85 potential DRMs were identified in 12 (50%) people with HIV (PWH)‐1A6 and 15 (42%) PWH non‐A6 HIV‐1 (Figure 1). Of these, 46 (54%) resulted from G‐to‐A mutations: 37 (80%) AGA and 9 (20%) NAGA. The INSTI resistance mutation G140R, which can be APOBEC‐induced, was detected as a minority variant in 8/60 (13%) sequences; 4 of these were NAGA, all in HIV‐1A6. Non‐APOBEC‐related minority variant DRMs were detected in HIV‐1A6 (*n* = 16) but not in non‐A6 HIV‐1.

**Conclusions**: DRMs occurring as minority variants, particularly G140R, were more common in HIV‐1A6 proviral DNA and may contribute to virological failure on LA‐CAB/RPV. Our bioinformatic pipeline efficiently distinguished clinically relevant DRMs from sequencing artefacts and APOBEC‐induced hypermutations, underscoring the value of dedicated pipelines for detection of minority variants in HIV‐1 proviral DNA.

**References**

1. Weibull Wärnberg A, Brännström J, Elvstam O, Gisslén M, Månsson F, Sönnerborg A, et al. The molecular epidemiology of HIV‐1 in Sweden 1996 to 2022 and the influence of migration from Ukraine. Eurosurveillance. 2023 Nov 30;28(48). doi:10.2807/1560‐7917.ES.2023.28.48.2300224

2. Cutrell AG, Schapiro JM, Perno CF, Kuritzkes DR, Quercia R, Patel P, et al. Exploring predictors of HIV‐1 virologic failure to long‐acting cabotegravir and rilpivirine: a multivariable analysis. AIDS. 2021 Jul 15;35(9):1333–42. doi:10.1097/QAD.0000000000002883

3. Wensing AM, Charpentier C, Calvez V, Ceccherini‐Silberstein F, Günthard HF, Jacobsen DM, et al. Utilizing HIV proviral DNA to assess for the presence of HIV drug resistance. Clin Infect Dis. 2025 Nov 6;81(4):e132–6. doi:10.1093/cid/ciaf161

4. Hu Z, Cordwell T, Nguyen H, Li J, Jeffrey JL, Kuritzkes DR. Effect of the L74I polymorphism on fitness of cabotegravir‐resistant variants of human immunodeficiency virus 1 subtype A6. J Infect Dis. 2023 Nov 11;228(10):1352–6. doi:10.1093/infdis/jiad291

**FIGURE 1:**
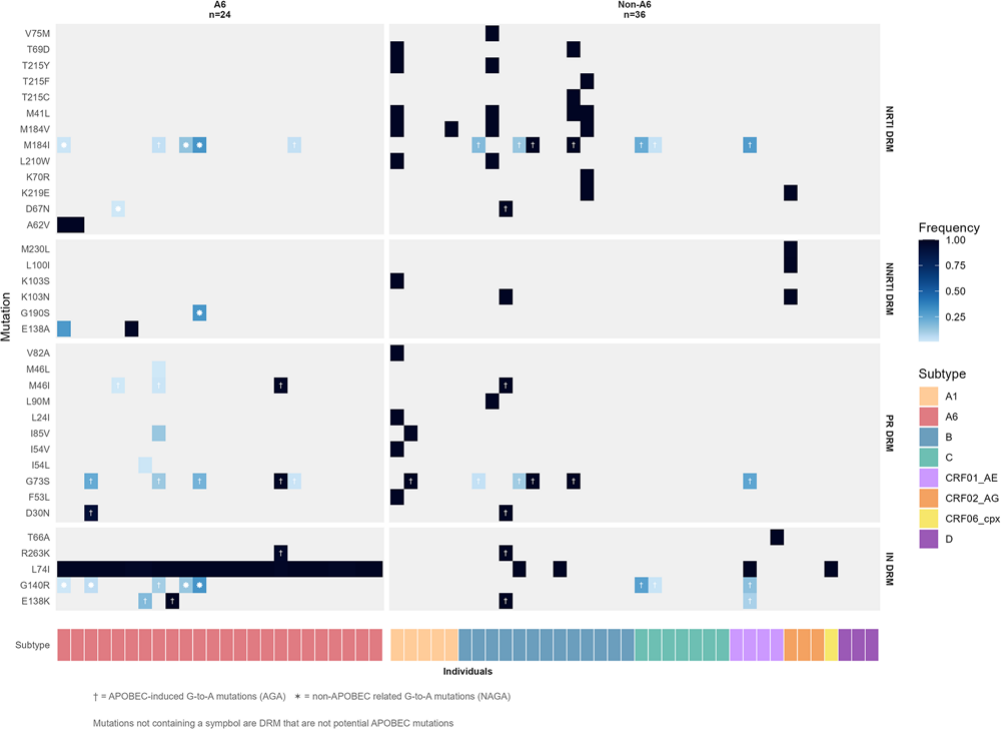
P099 | Heatmap of potential DRM, including L74I, by HIV‐1 subtype.

**TABLE 1:**
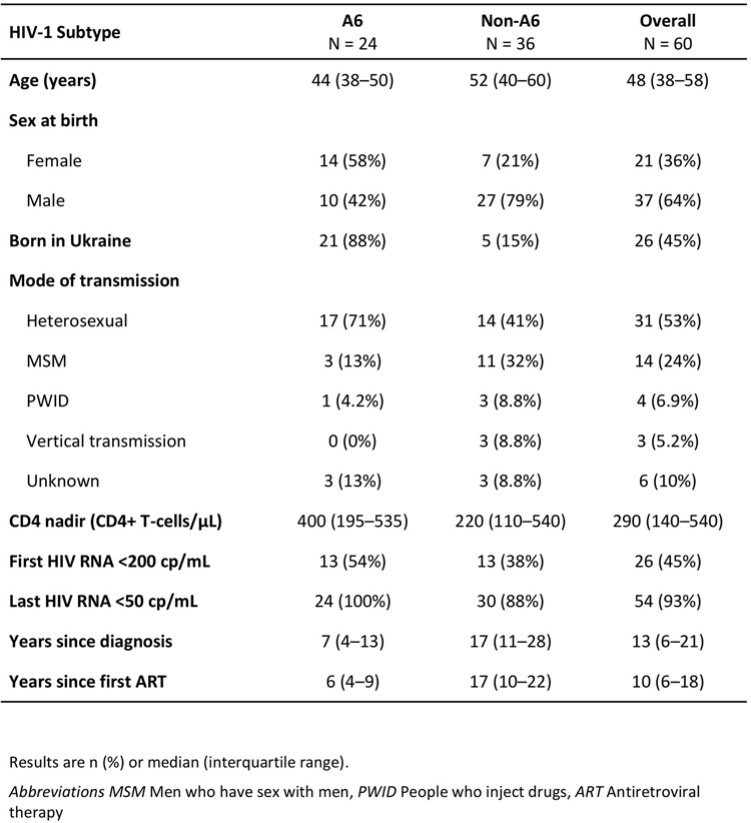
P099 | Cohort characteristics.

### P100 Long‐Acting Cabotegravir Plus Rilpivirine in a Large Single‐Centre Cohort: High Virological Efficacy Including in People Without Baseline Resistance Testing

Miguel Araújo Abreu, Rita De Almeida Teixeira, Carolina Costa, João Matos, Joana Vaz Cardoso, Josefina Méndez

Serviço de Doenças Infeciosas da Unidade Local de Saúde Santo António, Porto, Portugal

**Background**: Long‐acting injectable cabotegravir plus rilpivirine (LA‐CAB/RPV) is an effective maintenance strategy, but real‐world data from large single‐centre cohorts remain limited, particularly regarding outcomes in people initiated without baseline resistance testing. We report virological effectiveness, durability and resistance outcomes in a real‐world cohort.

**Methods**: Retrospective observational study of all adults with HIV‐1 who initiated LA‐CAB/RPV every 2 months at a single centre. Demographics, prior treatment, baseline genotyping and longitudinal HIV‐1 RNA were extracted. Virological suppression was assessed by snapshot of the last available viral load (intention‐to‐treat, ITT) and per‐protocol (PP; individuals remaining on treatment). Confirmed virological failure (CVF) was defined as two consecutive HIV‐1 RNA ≥200 copies/mL or treatment‐emergent resistance.

**Results**: 338 individuals (340 treatment courses; 1944 injections) were included. Median age was 42 years (IQR 34–52); 31% were aged ≥50, 15% were cis women and 44% were non‐Portuguese nationals. At initiation, 99% were virologically suppressed; median CD4 was 726 cells/µL (IQR 542–923); 93% had prior integrase‐inhibitor and 42% prior NNRTI exposure. Notably, 39% (133/340) had no baseline resistance test. Median follow‐up was 9 months (maximum 19) (Table 1). Virological suppression to <50 copies/mL was 97.5% (313/321) by ITT‐snapshot and 98.3% (281/286) per‐protocol; suppression to <200 copies/mL was 98.8% and 99.7%, respectively. Three CVF occurred (0.9%), all with treatment‐emergent resistance; all three were subsequently managed with and re‐suppressed on, a single‐tablet oral regimen. All three CVF occurred among individuals with baseline genotyping; no CVF occurred among the 133 individuals without baseline testing. 38 individuals (11%) discontinued, most commonly for scheduling/logistical reasons (*n* = 15) and injection‐related pain or local reactions (*n* = 8).

**Conclusions**: In this large single‐centre cohort, LA‐CAB/RPV achieved high virological efficacy, with only three CVF, each remaining switchable to an oral single‐tablet regimen. The absence of baseline resistance testing in a substantial proportion of suppressed individuals was not associated with virological failure, supporting the feasibility of LA‐CAB/RPV initiation where baseline genotyping is unavailable.

**TABLE 1:**
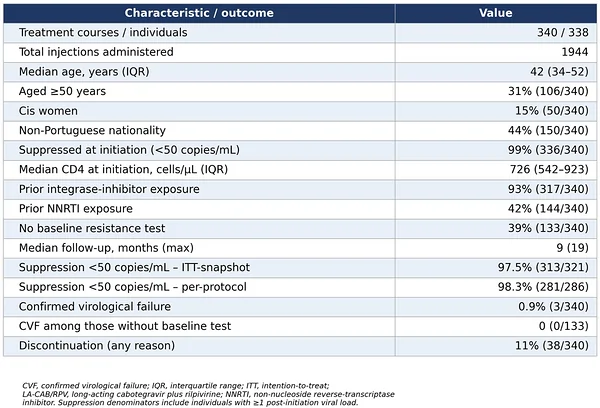
P100 | Baseline characteristics and virological outcomes of the LA‐CAB/RPV cohort (*N* = 340 treatment courses).

### P101 Outpatient or Hospital‐Based Care for Long‐Acting Injectable Antiretroviral Therapy: Different Preferences, Different Reasons, but Equal Satisfaction—The Real‐Life PANTER Study

Tristan Alain^1^, Fabienne Marcellin^2^, Saleh Ali^1^, Carole Abeguille^1^, Claudine Duvivier ^3^, Valérie Pourcher^4^, Pascale Perfezou^5^, Wiem Loghmari Bouchneb^6^, Anais Lesourd^7^, Gwenael Le Moal^8^, Martin Duracinsky^1^

^1^Patient‐Reported Outcomes Research (PROQOL), Health Economics Clinical Trial Center (URC‐ECO), Hôtel‐Dieu Hospital, AP‐HP, Paris, France; ^2^Aix Marseille Univ, Inserm, IRD, SESSTIM, Sciences Economiques & Sociales de la Santé & Traitement de l'Information Médicale, ISSPAM, Marseille, France; ^3^Infectious Diseases Department, Paris Cité University, INSERM 1016, Cochin Institute, CNRS 8104, IHU Imagine, AP‐HP, Necker‐Enfants Malades Hospital, Pasteur Institute, Medical Center, Paris, France; ^4^Infectious Diseases Department, Pitié‐Salpêtrière Charles Foix University Hospitals, AP‐HP, Paris, France; ^5^Infectious Diseases Department, Cornouaille University Hospital, Quimper, France; ^6^Department of Infectious Diseases and Immunology, Hôtel Dieu Hospital, Assistance Publique‐Hôpitaux de Paris, Université de Paris Cité, Paris, France; ^7^Infectious Diseases Department, Rouen University Hospital, Rouen, France; ^8^Infectious Diseases Department, Poitiers University Hospital, Poitiers, France

**Background**: Long‐acting (LA) injectable antiretroviral therapy (ART) could significantly impact the treatment experience and disease management for people living with HIV (PLWH). However, its increasing use raises challenges for the management of HIV care, highlighting the need to document PLWH preferences for treatment delivery and follow‐up settings to support patient‐centred implementation of this new therapeutic option.

**Methods**: The PANTER observational, multicentre, real life, study assesses patient‐reported outcomes in PLWH followed‐up in French hospitals and switching from oral ART to cabotegravir/rilpivirine (LA‐CAB/RPV). We described PLWH's characteristics and pain experience at treatment switch, as well as their satisfaction and preferences for hospital or outpatient delivery and care 9 months after switch. Means with standard deviations were for continuous variables, frequencies and percentages for categorical variables and Pearson chi‐squared or Fisher's exact tests for variables comparisons.

**Results**: From August 2023 to May 2025, 300 PLWH (66% male; mean [SD] age: 44 [12] years) were included in 22 centres. At enrolment, mean ART duration was 12 (9) years, 46% of PLWH had at least one comorbidity and 96% were receiving daily ART. Fifty‐two percent of PLWH (*N* = 255/300) reported being worried before injection (a little: 31%, moderately: 10%, a lot or extremely: 11%). Finally, 71% experienced pain or discomfort at injection site (45%, 18%, 8%, respectively). At month 9 (*n* = 228), 93% of PLWH were highly or completely satisfied with the overall injectable treatment pathway, with no difference between hospital, outpatient nurse and home delivery (*p* = 0.11), which accounted for 56%, 33% and 11% of PLWH, respectively (*N* = 219/300). However, the reported reasons for preferences among PLWH varied highly between places of treatment delivery: easier appointments and saving time were associated with a preference for outpatient nurse delivery (79% and 67%, respectively) and home delivery (70% and 70%, respectively), while better discussions with healthcare professionals (61%), better injection administration (60%) and protection of anonymity (52%) were associated with preference for hospital delivery (*p* < 0.001).

**Conclusions**: The high satisfaction with LA‐CAB/RPV among PLWH is accompanied by place preferences that should be taken into account to facilitate its implementation in community settings. Strengthening coordination between hospitals and outpatient care could help improve continuity of care.

### P102 Comparison of Dolutegravir/Lamivudine and Bictegravir/Emtricitabine/Tenofovir Alafenamide in Antiretroviral Therapy‐Naïve Patients With High Viral Load: A Multicentre Retrospective Study

Joon Young Song^1^, Jun Yong Choi^2^, Dae Won Park^3^, Shin‐Woo Kim^4^, Ji Young Rhee^5^, Dong Hoon Shin^6^, Sun Hee Lee^7^, Tae Hyong Kim^8^, Eun Jin Kim^9^, Jihyeon Baek^10^, Kyung‐Hwa Park^11^, Yeon‐Sook Kim ^12^, on behalf of Rose of Sharon Study Group

^1^Division of Infectious Diseases, Department of Internal Medicine, Korea University Guro Hospital, Korea University College of Medicine, Seoul, Republic of Korea; ^2^Division of Infectious Diseases, Department of Internal Medicine, Yonsei University College of Medicine, Seoul, Republic of Korea; ^3^Division of Infectious Diseases, Department of Internal Medicine, Korea University Ansan Hospital, Korea University College of Medicine, Ansan, Republic of Korea; ^4^Division of Infectious Diseases, Department of Internal Medicine, School of Medicine, Kyungpook National University, Daegu, Republic of Korea; ^5^Division of Infection Control, Dankook University Hospital, Dankook University College of Medicine, Cheonan, Republic of Korea; ^6^Department of Internal Medicine, Seoul Metropolitan Government‐Seoul National University Boramae Medical Center, Seoul, Republic of Korea; ^7^Division of Infectious Diseases, Department of Internal Medicine, Pusan National University School of Medicine, Pusan National University Hospital, Busan, Republic of Korea; ^8^Division of Infectious Diseases, Department of Internal Medicine, Soonchunhyang University Seoul Hospital, Seoul, Republic of Korea; ^9^Department of Infectious Disease, Ajou University School of Medicine, Suwon, Republic of Korea; ^10^Division of Infectious Diseases, Department of Internal Medicine, Inha University Medical College, Incheon, Republic of Korea; ^11^Department of Infectious Diseases, Chonnam National University Medical School, Gwangju, Republic of Korea; ^12^Division of Infectious Diseases, Department of Internal Medicine, Chungnam National University School of Medicine, Daejeon, Republic of Korea

**Background**: Treatment with effective antiretroviral therapy (ART) is important to reduce the clinical impact of high viral load (VL) in patients with HIV (PWH). There are limited data comparing the treatment outcomes of dolutegravir/lamivudine (DTG/3TC) or bictegravir/emtricitabine/tenofovir alafenamide (BIC/FTC/TAF) in ART‐naïve patients with high VL (> 100,000 copies/mL).

**Methods**: We conducted a retrospective study to compare DTG/3TC and BIC/FTC/TAF in ART‐naïve patients with high VL. Our study compared virologic and immunologic outcomes at 48 weeks of ART by per‐protocol analysis and time to virologic suppression by Kaplan–Meier survival analysis.

**Results**: A total of 129 patients with high VL (77 in DTG/3TC and 52 in BIC/FTC/TAF group) was enrolled from 12 centres in South Korea. The proportion of patients who achieved viral suppression (VL < 50 copies/mL) at week 48 was 52 of 77 (80.0%) in DTG/3TC and 35 of 52 (79.5%) in BIC/FTC/TAF group, respectively (*p* = 0.954). VL > 200 copies/mL at week 48 was observed in 4 (5.19%) of DTG/3TC and 2 (3.8%) of BIC/FTC/TAF group (*p* = 0.537). The median time to virologic suppression was 22 weeks (IQR, 11–44 weeks) and 17 weeks (IQR, 10–39 weeks) in DTG/3TC and BIC/FTC/TAF group (*p* = 0.867). The CD4 cell count significantly increased from 200.8 ± 172.6 cells/mL to 484.7 ± 303.6 cells/mL in DTG/3TC and from 178.4 ± 150.5 cells/mL to 410.5 ± 245.8 cells/mL in BIC/FTC/TAF group at week 48 (*p* = 0.135). Subgroup analysis for PWH with VL > 500,000 copies/mL (24 in DTG/3TC and 16 in BIC/FTC/TAF group) was performed. Viral suppression was achieved in 16 (80%) of DTG/3TC and 9 (64.3%) of BIC/FTC/TAF group (*p* = 0.264). VL > 200 copies/mL at week 48 was not observed in either DTG/3TC or BIC/FTC/TAF group.

**Conclusions**: Our study demonstrated that DTG/3TC showed similar virologic and immunologic outcomes to BIC/FTC/TAF at week 48 in PWH with high VL. These data support that high VL needs not be a limiting factor for choosing between 2‐drug and 3‐drug ART regimens.

### P103 Tolerability Following the First Injection Satisfaction of Long‐Acting Cabotegravir Plus Rilpivirine in Virologically Suppressed People With HIV Aged ≥60 Years: The LOVER60 Trial

Salvador Valero Cifuentes ^1,2^, Helena Albendin Iglesias^3^, Francisco Vera Méndez^4^, Joaquín Bravo Urbieta^2,5^, Sergio Padilla^6,7^, Sergio Reus^8^, Concepción Gil Anguita^9^, Raque Garijo^10^, Antonia Alcaraz Garcia^1,2^, Antonia Castillo Navarro^3^, Onofre Martínez Madrid^4^, Isabel Carpena Martínez^5^, Javier Garcia Abellan^6,7^, Livia Giner Oncina^8^, Isabel Ribes Mengual^9^, Angeles Muñoz Perez^1,2^, Maria Angeles Fernandez Lopez^3^, Lorena Martinez^4^, Maria Dolores Hernandez Lorente^1,2^, Paula Mascarell^6,7^, Jimena Teran^8^, Cristina Tomas Jimenez^1,2^, Carlos Galera Peñaranda^3^, Rosa Blazquez Garrido^5^, Esperanza Merino^8^, Enrique Bernal Morell^1,2^

^1^Infectious Diseases Unit, Internal Medicine Department, Reina Sofía University General Hospital, Murcia, Spain; ^2^Research Group on Medicine and Artificial Intelligence, Murcian Institute of Biosanitary Research Pascual Parrilla, IMIB, Murcia, Spain; ^3^Infectious Diseases Unit, Virgen de la Arrixaca University General Hospital, Murcia, Spain; ^4^Infectious Diseases Unit, Santa Lucía University General Hospital, Cartagena, Spain; ^5^Infectious Diseases Unit, Internal Medicine Department, Morales Meseguer University General Hospital, Murcia, Spain; ^6^Infectious Diseases Unit, Elche University General Hospital, Alicante, Spain; ^7^CIBERINFEC, ISCIII, Madrid, Spain; ^8^Infectious Diseases Unit, Dr. Balmis General University Hospital, ISABIAL, Alicante, Spain; ^9^Infectious Diseases Unit, Marina Baixa University General Hospital in Villajoyosa, Alicante, Spain; ^10^ViiV Healthcare, Tres Cantos, Madrid, Spain

**Background**: Older people with HIV (PWH) remain underrepresented in pivotal trials of long‐acting cabotegravir plus rilpivirine (CAB+RPV LA) and real‐world data in those aged ≥60 years are limited. We aimed to describe the clinical profile, prior antiretroviral therapy (ART), baseline treatment satisfaction and early tolerability after switching to CAB+RPV LA in virologically suppressed older PWH.

**Methods**: LOVER60 is a phase 4, multicentre, 24‐month clinical trial conducted in Spain including PWH aged ≥60 years with sustained virological suppression on oral ART who switched to intramuscular CAB+RPV LA according to the product label. Baseline demographic, clinical, immunovirological and historical resistance data (when available) were collected. Treatment satisfaction was assessed using the HIVTSQs questionnaire. Injection‐related adverse events (AEs) after the first dose and patient experience using the PIN questionnaire were prospectively evaluated. Descriptive statistics were used.

**Results**: A total of 120 participants were enrolled (mean age 65.7 ± 5.3 years; 77.5% male). Mean time since HIV diagnosis was 21.1 years, 31.7% had a prior AIDS‐defining condition, mean CD4 count was 778 cells/µL and mean CD4/CD8 ratio was 1.12. At baseline, 66.7% had undetectable viral load, 24.2% had viraemia < 50 copies/mL and 9.2% had viral blips (50–200 copies/mL). Historical resistance testing was available for 30.0%. Previous ART consisted of 2‐drug (55%) or 3‐drug regimens (45%), predominantly integrase inhibitor‐based (91.7%). After the first injection, 40.0% experienced ≥1 injection‐related AE, mainly injection‐site pain (83.3%), followed by flu‐like symptoms (14.6%), asthenia (6.3%), headache (4.2%), flushing/generalized pruritus (4.2%) and isolated cases of insomnia, rhinoconjunctivitis syndrome, hepatic AEs and suicidal ideation (2.1% each). Most AEs were mild (79.2%), with permanent discontinuation in 4.2% of participants. Baseline treatment satisfaction was high (mean HIVTSQs 55.5/60; 85.8% scored ≥50). In the PIN questionnaire, 80.8% rated local reactions and 76.7% pain as totally acceptable; 83.3% were very satisfied with the injection system and 84.2% would definitely choose injectable treatment outside the trial.

**Conclusions**: In older PWH with long‐standing HIV infection and high satisfaction with oral ART, switching to CAB+RPV LA showed good early tolerability. Injection‐related AEs were predominantly mild, discontinuation was uncommon and patient‐reported acceptability was high, supporting CAB+RPV LA as a well‐tolerated option for older PWH.

### P104 Hepatic Steatosis and Fibrosis in People With HIV Aged ≥60 Years Eligible for Long‐Acting Cabotegravir Plus Rilpivirine: Baseline Findings From the LOVER60 Trial

Salvador Valero Cifuentes ^1,2^, Helena Albendin Iglesias^3^, Francisco Vera Méndez^4^, Joaquín Bravo Urbieta^2,5^, Sergio Padilla^6^, Sergio Reus^7^, Concepción Gil Anguita^8^, Jose Martin‐Herrero^9^, Eva Garcia Villalba^1,2^, Antonia Castillo Navarro^3^, Onofre Martínez Madrid^4^, Sergio Aleman^5^, Javier Garcia Abellan^6^, Livia Giner Oncina^7^, Isabel Ribes Mengual^8^, Jose Miguel Gomez Verdu^1,2^, Maria Angeles Fernandez Lopez^3^, Lorena Martinez^4^, Monica Martinez Martinez^5^, Paula Mascarell^6^, Jimena Teran^7^, Rodrigo Martinez Rodriguez^1,2^, Carlos Galera Peñaranda^3^, Antonio Sanchez Guirao^5^, Enrique Bernal Morell^1,2^

^1^Infectious Diseases Unit, Internal Medicine Department, Reina Sofía University General Hospital, Murcia, Spain; ^2^Research Group on Medicine and Artificial Intelligence, Murcian Institute of Biosanitary Research Pascual Parrilla, IMIB, Murcia, Spain; ^3^Infectious Diseases Unit, Virgen de la Arrixaca University General Hospital, Murcia, Spain; ^4^Infectious Diseases Unit, Santa Lucía University General Hospital, Cartagena, Spain; ^5^Infectious Diseases Unit, Internal Medicine Department, Morales Meseguer University General Hospital, Murcia, Spain; ^6^Infectious Diseases Unit, Elche University General Hospital, Alicante, Spain; CIBERINFEC, ISCIII, Madrid, Elche, Spain; ^7^Infectious Diseases Unit, Dr. Balmis General University Hospital, ISABIAL, Alicante, Spain; ^8^Infectious Diseases Unit, Marina Baixa University General Hospital in Alicante, Villajoyosa, Spain; ^9^ViiV Healthcare, Tres Cantos, Madrid, Spain

**Background**: Older people with HIV (PWH) are at increased risk of metabolic dysfunction‐associated steatotic liver disease (MASLD) and liver fibrosis. One objective of the LOVER60 trial is to evaluate the long‐term metabolic effects of switching to long‐acting cabotegravir plus rilpivirine (CAB+RPV LA), based on the hypothesis that this regimen may provide hepatic benefits, potentially mediated by rilpivirine. This analysis describes the baseline hepatic status of a LOVER60 sub‐cohort before switching, assessing the prevalence of hepatic steatosis and associated clinical and laboratory factors and establishing a reference for future evaluation of liver outcomes.

**Methods**: LOVER60 is an open‐label, phase 4, multicentre, single‐arm, 24‐month trial conducted in Spain in PWH aged ≥60 years with sustained virological suppression on oral ART who switched to CAB+RPV LA. Among 120 participants, 103 underwent baseline FibroScan with controlled attenuation parameter (CAP). CAP was used to grade steatosis and liver stiffness to stage fibrosis. Anthropometric, metabolic, immunological and virological variables were compared between participants with steatosis grade < 2 and ≥2 (CAP > 248 dB/m) using Student's *t* test.

**Results**: The 103 participants had a mean age of 65.8 ± 5.3 years and 77.6% were male. Clinically relevant steatosis (grade ≥2) was present in 31.1% (32/103), while liver fibrosis was detected in 30.1% (31/103). Mean CAP was 244.7 dB/m. Compared with participants with steatosis < 2, those with steatosis ≥2 had significantly higher BMI (30.05 vs. 25.00 kg/m^2^; *p* < 0.001), waist circumference (106.83 vs. 94.44 cm; *p* < 0.001), abdominal circumference (106.16 vs. 97.80 cm; *p* < 0.001), TyG index (8.95 vs. 8.54; *p* = 0.001) and fasting glucose (119.0 vs. 104.0 mg/dL; *p* = 0.036). HOMA‐IR was numerically higher (9.33 vs. 6.41; *p* = 0.17), whereas ALT, LDL cholesterol, insulin, CD4 count, age and CD4/CD8 ratio did not differ significantly. Mean CAP (240.3 vs. 250.3 dB/m; *p* = 0.38) and steatosis prevalence (29.8% vs. 32.6%; *p* = 0.93) were similar between dual‐ and triple‐therapy recipients.

**Conclusions**: Approximately one‐third of virologically suppressed older PWH had clinically relevant steatosis and liver fibrosis despite preserved immune status. Steatosis was associated with higher BMI, central adiposity and TyG index, but not ART regimen, providing a robust baseline for evaluating the long‐term hepatic impact of CAB+RPV LA in LOVER60.

### P105 Comorbidity and Frailty Assessed by the FRAIL Scale in People With HIV Aged ≥60 Years Switching to Long‐Acting Cabotegravir Plus Rilpivirine: Baseline Results From the LOVER60 Trial

Salvador Valero Cifuentes ^1,2^, Helena Albendin Iglesias^3^, Francisco Vera^4^, Joaquín Bravo Urbieta^2,5^, Sergio Padilla^6^, Sergio Reus^7^, Concepción Gil Anguita^8^, Maria Jesus Vazquez^9^, Rebeca Mondaray‐Marin^1,2^, Antonia Castillo Navarro^3^, Onofre Martínez Madrid^4^, Isabel Carpena^5^, Javier Garcia Abellan^6^, Livia Giner Oncina^7^, Isabel Ribes Mengual^8^, Antonia Alcaraz Garcia^1,2^, Maria Angeles Fernandez Lopez^3^, Lorena Martinez^4^, Maria Dolores Hernandez Lorente^1,2^, Paula Mascarell^6^, Jimena Teran^7^, Angeles Muñoz Perez^1,2^, Carlos Galera Peñaranda^3^, Rosa Blazquez Garrido^5^, Enrique Bernal Morell^1,2^

^1^Infectious Diseases Unit, Internal Medicine Department, Reina Sofía University General Hospital, Murcia, Spain; ^2^Research Group on Medicine and Artificial Intelligence, Murcian Institute of Biosanitary Research Pascual Parrilla, IMIB, Murcia, Spain; ^3^Infectious Diseases Unit, Virgen de la Arrixaca University General Hospital, Murcia, Spain; ^4^Infectious Diseases Unit, Santa Lucía University General Hospital, Cartagena, Spain; ^5^Infectious Diseases Unit, Internal Medicine Department, Morales Meseguer University General Hospital, Murcia, Spain; ^6^Infectious Diseases Unit, Elche University General Hospital, Alicante, Spain; CIBERINFEC, ISCIII, Madrid, Spain; ^7^Infectious Diseases Unit, Dr. Balmis General University Hospital, ISABIAL, Alicante, Spain; ^8^Infectious Diseases Unit, Marina Baixa University General Hospital in Villajoyosa, Villajoyosa, Spain; ^9^ViiV Healthcare, Tres Cantos, Madrid, Spain

**Background**: Ageing people with HIV (PWH) have a high burden of multimorbidity and are at increased risk of frailty, which is associated with disability, healthcare utilization and mortality. The FRAIL scale is a simple 5‐item screening tool classifying individuals as robust, pre‐frail or frail. We assessed baseline multimorbidity and frailty in older PWH enrolled in the LOVER60 trial of long‐acting cabotegravir plus rilpivirine (CAB+RPV LA).

**Methods**: LOVER60 is an open‐label, single‐arm, phase 4 multicentre trial in Spain including virologically suppressed PWH aged ≥60 years who switched from oral ART to CAB+RPV LA. Baseline demographics, HIV history and comorbidities were systematically collected, including hypertension, diabetes, cardiovascular, kidney, liver and lung disease, cancer, obesity, osteopenia/osteoporosis, arthritis and asthma. Frailty was assessed using the FRAIL scale. Descriptive statistics summarized comorbidity burden and frailty status.

**Results**: A total of 120 participants were included (mean age 65.7 ± 5.3 years; mean time since HIV diagnosis 21.1 ± 10.6 years). The most common comorbidities were hypertension (40.8%), chronic liver disease (26.7%), obesity (19.2%), diabetes (18.3%), osteopenia/osteoporosis (17.5%), cardiovascular disease (12.5%), non‐skin cancer (9.2%), chronic lung disease (8.3%), chronic kidney disease (7.5%), asthma (2.5%), myocardial infarction (5.0%), angina (3.3%), heart failure (1.7%) and stroke (3.3%). Overall, 7.5% had three or more cardiometabolic comorbidities. Regarding FRAIL components, 11.7% reported fatigue, 10.8% had difficulty climbing 10 steps, 6.7% had difficulty walking 100 m and 10.0% reported ≥5% unintentional weight loss during the previous year. No participant met the FRAIL criterion of five or more chronic conditions. Overall, 71.7% were classified as robust, 24.2% as pre‐frail and 4.2% as frail, indicating a predominantly robust but substantially comorbid ageing HIV population.

**Conclusions**: Older virologically suppressed PWH eligible for CAB+RPV LA had a high burden of comorbidities, particularly hypertension, liver disease, obesity and bone disorders, but most remained robust, with one quarter classified as pre‐frail and few as frail. These findings support routine FRAIL screening and identify a subgroup that may benefit from regular follow‐up during CAB+RPV LA administration.

### P106 Resistance Burden and Treatment Optimization Potential in Heavily Treatment‐Experienced People Living With HIV: A Real‐World Genotypic Analysis

Alejandro De Gea Grela^1^, José Ignacio Bernardino^1^, Jara Llenas‐García^1^, Luz Martín‐Carbonero^1^, Maria Luisa Montes^1^, María del Mar Arcos Rueda^1^, Rosa De Miguel‐Buckley^1^, Carmen Busca^1^, Rafael Mican^1^, Juan Gonzalez‐Garcia^1^, Jose R. Arribas^1^, Rocio Montejano ^1,2^

^1^Hospital Universitario La Paz, IdiPAZ, Madrid, Spain; ^2^CIBERINFEC, Madrid, Spain

**Background**: People living with HIV (PLWH) who are heavily treatment‐experienced (HTE) represent a particularly vulnerable group in whom accumulated multi‐class resistance may constrain antiretroviral optimization. We aimed to characterize the HTE population within a large tertiary HIV cohort and to evaluate the genotypic susceptibility of their current antiretroviral regimens.

**Methods**: Cross‐sectional analysis of the HIV cohort at Hospital Universitario La Paz (Madrid, Spain), comprising 3995 PLWH under active follow‐up. HTE status was defined as the presence of resistance‐associated mutations conferring high‐level resistance (Stanford HIVdb score >15) to ≥3 of 4 drug classes (NRTI, NNRTI, PI, INSTI). Genotypic susceptibility scores (GSS) for current regimens were calculated per drug using the Stanford University HIV Drug Resistance Database (HIVdb, v9.x) algorithm, with individual drug scores weighted as: susceptible (score ≤10) = 1.0, low‐level resistance (11–30) = 0.5–0.75 and high‐level resistance (> 60) = 0, following the methodology described by Wensing et al. [1]. Total GSS per regimen was the sum of individual drug GSS values.

**Results**: Of 1701 patients with available resistance genotyping data, 132 (7.8% of genotyped patients; 3.3% of the overall cohort) met HTE criteria. The cohort was predominantly male (75.8%), median age 61 years (IQR 57–65) (Table 1). Resistance class distribution showed universal NRTI resistance (100%), near‐universal NNRTI resistance (98.5%), high‐level PI resistance (87.1%) and INSTI resistance in 21.2%. The predominant resistance pattern was triple‐class NRTI+NNRTI+PI failure (78.8%). Despite this resistance burden, 90.2% of HTE patients were virologically suppressed (plasma HIV‐RNA <50 copies/mL) at the time of analysis, median CD4 count 618 cells/µL (66.2% ≥500 cells/µL). The median GSS of current regimens was 1.5 (mean 1.50, range 0.2–3.0). Only 34.8% of patients had a GSS ≥2 (fully suppressive potential), while 65.2% had a GSS < 2, including 8 patients (6.1%) with a GSS < 1. Among newer agents, DTG, BIC and CAB retained susceptibility (score ≤10) in 89.4% of patients; DOR in 72.0%; and DRV in 48.5%.

**Conclusions**: HTE patients represent a small but clinically complex subgroup, characterized by long‐standing HIV infection and predominantly triple‐class resistance. Despite low regimen GSS, most remain virologically suppressed, highlighting opportunities for optimization using newer active agents.

**Reference**

1. Wensing AM, Calvez V, Ceccherini‐Silberstein F. 2019 update of the drug resistance mutations in HIV‐1. Top Antivir Med. 2019 Sep;27(3):111‐121.

**TABLE 1:**
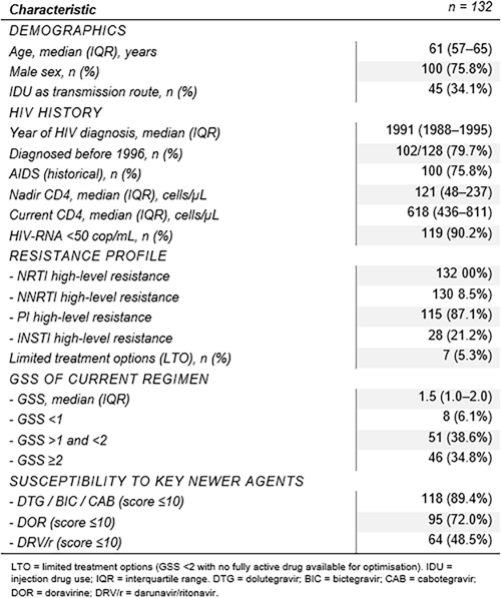
P106 | Baseline characteristics of HTE.

### P107 Cardiometabolic and Body‐Composition Outcomes in People With HIV Switching to Long‐Acting Cabotegravir Plus Rilpivirine Versus Oral Two‐Drug Regimens: A Matched Cohort Study

Filippo Calandra Buonaura ^1,2^, Jovana Milić^1,2^, Uberto Calvi^1,2^, Filippo Buffagni^1,2^, Eugenio Carriero^1,2^, Cristina Mussini^1,2^, Giovanni Guaraldi^1,2^

^1^Department of Surgical, Medical, Dental and Morphological Sciences, University of Modena and Reggio Emilia, Modena, Italy; ^2^Modena HIV Metabolic Clinic, University of Modena and Reggio Emilia, Modena, Italy

**Background**: Long‐acting injectable cabotegravir plus rilpivirine (LA CAB+RPV) is increasingly used for HIV maintenance, but real‐world data on its cardiometabolic and body‐composition impact relative to oral two‐drug regimens (2DR) are limited. We compared longitudinal cardiometabolic and dual‐energy X‐ray absorptiometry (DEXA) body composition outcomes between people with HIV (PWH) switching to LA CAB+RPV and matched PWH switching to or remaining on, oral 2DR.

**Methods**: In a retrospective longitudinal single‐centre cohort, PWH switching to LA CAB+RPV were matched 1:2 by propensity score (calliper 0.1), exactly on prior regimen class and balanced on antiretroviral‐therapy duration and follow‐up time, to PWH on oral 2DR (DTG/3TC, DTG/RPV, DOR+DTG, DOR+RAL or RPV+RAL). Baseline and follow‐up were the last pre‐ and post‐switch assessments. Outcomes were change in DEXA visceral adipose tissue (VAT), total lean and fat mass, trunk fat, femoral and lumbar bone mineral density (BMD), weight, BMI, HOMA‐IR and lipids, plus incident diabetes and hypertension (EACS). Change was assessed by paired and between‐group tests and unadjusted linear mixed models.

**Results**: We analysed 106 matched PWH (39 LA CAB+RPV, 67 oral 2DR). Mean age was 52.9 years, 71.7% were male, mean time since HIV diagnosis was 18.8 years and 98.1% had suppressed HIV‐RNA. Median follow‐up was 1.59 years in the LA group and 1.36 years in the oral 2DR group (*p* = 0.487). Baseline characteristics were well balanced after matching (Table 1). No significant between‐group differences were observed in the change in VAT, total lean or fat mass, trunk fat, femoral or lumbar BMD, weight, BMI or HOMA‐IR (all *p* > 0.05; Figure 1). LDL cholesterol decreased significantly in both groups (oral 2DR 126→114 mg/dL, *p* = 0.009; LA 112→96 mg/dL, *p* = 0.005). Statin use increased over the same period (oral 2DR 25→48%, LA 33→62%). Incident diabetes, hypertension and insulin resistance were infrequent and similar between arms.

**Conclusions**: In this matched real‐world cohort, switching to LA CAB+RPV was not associated with adverse cardiometabolic or body‐composition changes compared with oral 2DR, with no significant between‐group differences. The LDL reductions seen in both arms were most likely driven by concurrent statin initiation rather than the antiretroviral regimen.

**TABLE 1:**
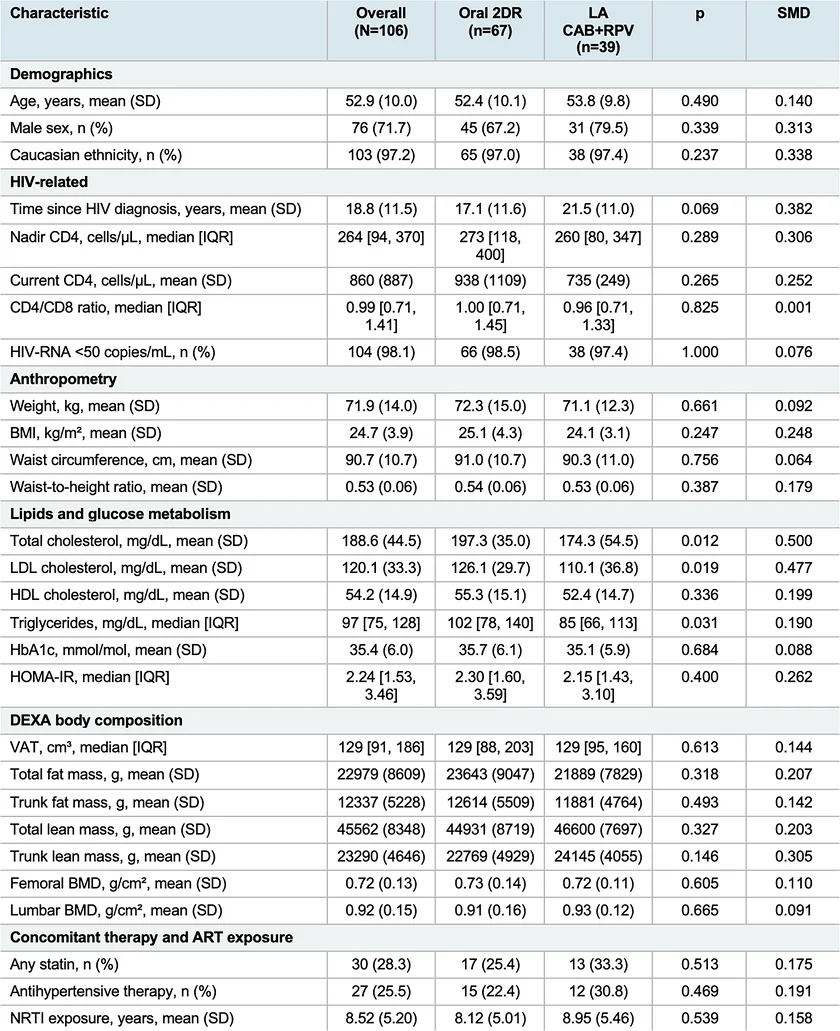
P107 | Baseline characteristics of the matched cohort, overall and by group. Data are mean (SD), median (IQR) or *n* (%). *p*, between‐group comparison; SMD, standardized mean difference (<0.10 indicates good balance). LA, long‐acting; CAB, cabotegravir; RPV, rilpivirine; 2DR, two‐drug regimen; VAT, visceral adipose tissue; BMD, bone mineral density; ASMI, appendicular skeletal muscle mass index; BP, blood pressure; NRTI, nucleoside reverse‐transcriptase inhibitor.

**FIGURE 1:**
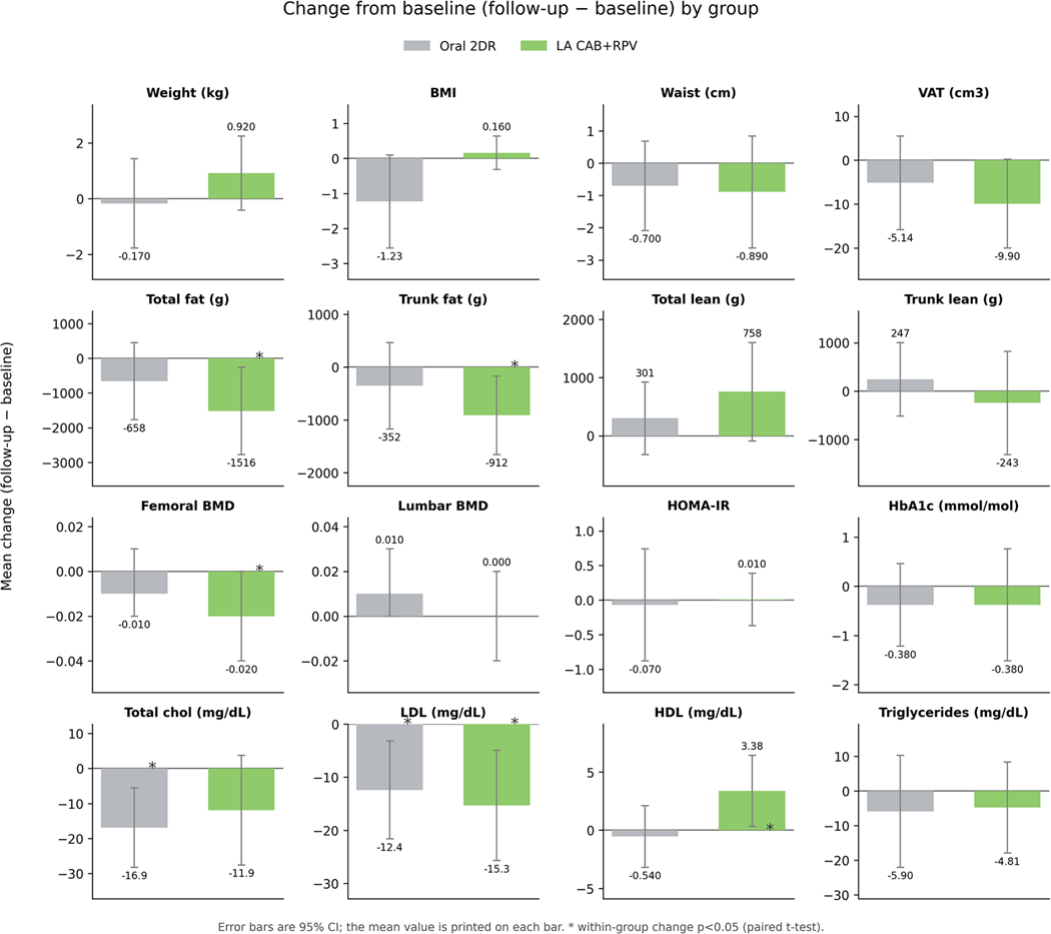
P107 | Mean change from baseline (follow‐up−baseline) in anthropometric, body‐composition, bone and metabolic parameters, by treatment group, in the matched cohort (39 LA CAB+RPV, 67 oral 2DR). Each panel shows one variable; bars represent the group mean change (grey = oral 2DR, green = LA CAB+RPV) and rise above or fall below the zero line for increases or decreases, respectively. The mean value is printed on each bar, and error bars denote 95% confidence intervals. Asterisks (*) indicate a statistically significant within‐group change from baseline to follow‐up (paired *t*‐test, *p* < 0.05). There were no significant between‐group differences in the change of any variable (all *p* > 0.05). DEXA‐derived fat and lean masses are expressed in grams. 2DR, two‐drug regimen; BMD, bone mineral density; CAB, cabotegravir; HOMA‐IR, homeostatic model assessment of insulin resistance; LA, long‐acting; LDL/HDL, low‐/high‐density lipoprotein cholesterol; RPV, rilpivirine; VAT, visceral adipose tissue.

### P108 Safety and Effectiveness Results of Post‐Marketing Surveillance Studies of B/F/TAF in China, Japan and Republic of Korea

Jami Peters^1^, Jennifer Davidson^2^, Margarida Serejo^3^, Keith Aizen^3^, Nao Taguchi^4^, Jeong‐a Lee^5^, Yixiao Lin^6^, Mellissa Williamson ^1^

^1^Gilead Sciences, Foster City, CA, US; ^2^Evidera, London, UK; ^3^Gilead Sciences, Cambridge, UK; ^4^Gilead Sciences, Tokyo, Japan; ^5^Gilead Sciences, Seoul, Republic of Korea; ^6^Gilead Sciences, Shanghai, China

**Background**: B/F/TAF is a once‐daily single tablet regimen used to treat people with HIV‐1 (PWH). Post marketing surveillance (PMS) studies are a requirement in several countries in East Asia.

**Methods**: Three large, multicentre observational PMS studies were conducted among PWH treated with B/F/TAF to assess its safety and effectiveness. The observation period was ≥52 weeks in all three studies. Study data were extracted from medical records.

**Results**: The studies in China, Japan and Republic of Korea (ROK) included 2982 PWH across 34 sites (May 2021–June 2023), 1496 PWH across 24 sites (April 2019–February 2025) and 2761 PWH across 45 sites (July 2019–November 2023), respectively (Figure 1). Across the studies, the mean (±SD) age ranged from 38.8 to 43.7 years (±11.8–13.7). The majority of participants were male (91.9–96.5%) and antiretroviral treatment–experienced (TE) (64.6–81.5%). Adverse drug reactions (ADRs) were reported in 5.4–17.1% of participants. Serious ADRs were infrequent, occurring in 0.01–1.7% of participants (Table 1). In all three countries, less than 3% of participants reported ADRs leading to discontinuation of B/F/TAF. In China, ADRs ≥1% by preferred term (PT) were: weight increased (5.3%), hyperuricemia (4%), hyperlipidaemia (3.5%), hepatic function abnormal (2.2%) and renal impairment (1.5%). In Japan, ADRs ≥1% by PT were: weight increased (2.1%), insomnia (1.2%), syphilis (1.1%) and low‐density lipoprotein increased (1.1%). In ROK, there were no ADRs with a frequency of ≥1%. At approximately 52 weeks (36–64 weeks), mean CD4^+^ T‐cell counts increased across three studies in both antiretroviral treatmentnaïve (TN) and TE patients. Mean (±SD) increases were China: +65.4 ± 212.8 (TE) and +230.6 ± 229.4 cells/mm^3^ (TN); Japan: +14.9 ± 149.9 (TE) and +206.8 ± 128.0 cells/mm^3^ (TN) and ROK: +20.7 ± 211.1 (TE) and +277.5 ± 258.5 cells/mm^3^ (TN). Viral suppression rates exceed 97% in TN and 93% in TE.

**Conclusions**: No new safety signals were identified; observed safety and effectiveness were consistent with the established profile of B/F/TAF. The findings from these > 7000 patients support the tolerability and effectiveness of B/F/TAF in routine clinical practice among PWH in East Asia.

**FIGURE 1:**
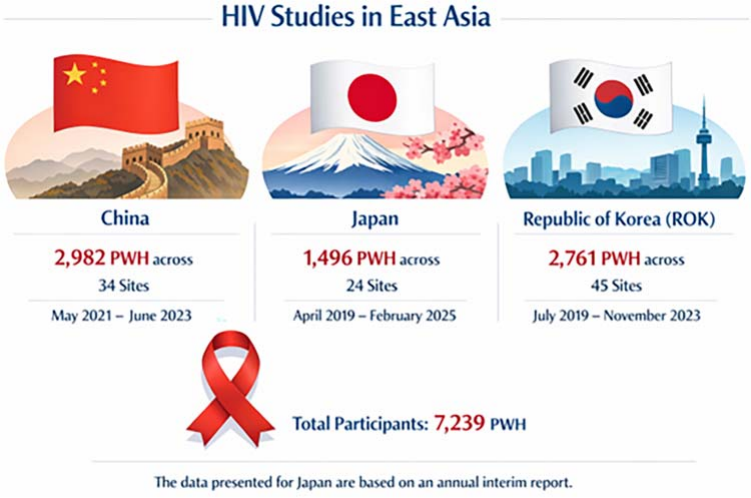
P108 | Overview of post‐marketing surveillance studies of B/F/TAF in China, Japan and Republic of Korea.

**TABLE 1:**
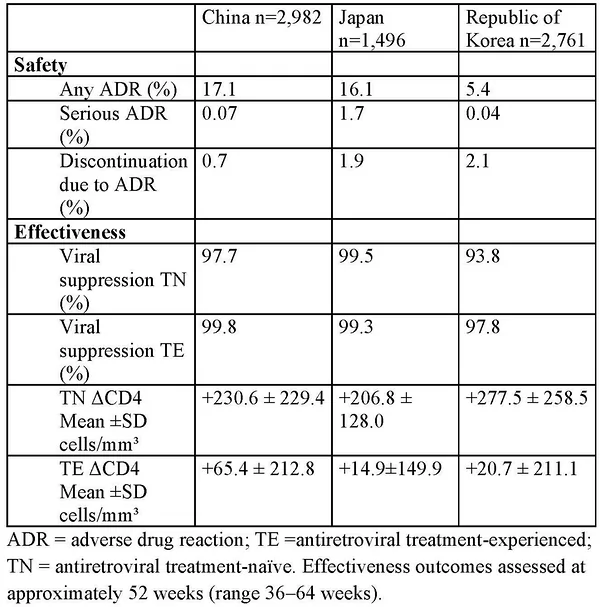
P108 | Key safety and effectiveness outcomes.

### P109 Real‐World Outcomes Following Switch From Boosted Protease Inhibitor Based Antiretroviral Therapy Regimens to Dolutegravir/Lamivudine

Thomas Swaine ^1,2^, Alan Hunter^1^, Shenal Gohil^1^, Fiona Burns^1,2^, Tristan Barber^1,2^

^1^Department of HIV Medicine, Royal Free London NHS Trust, London, UK; ^2^UCL Institute for Global Health, London, UK

**Background**: Dolutegravir/lamivudine (DTG/3TC) is an efficacious complete ART regimen with minimal toxicities, licensed in the UK since 2019. While the initial DTG/3TC switch trials included relatively few participants on boosted protease inhibitor (bPI) regimens [1], switch away from bPI‐based regimens is of increasing interest in people with HIV (people) due to concerns about long‐term toxicities and drug−drug interactions.

**Methods**: Single‐centre retrospective study of people who switched from bPI‐based ART to DTG/3TC between 11 January 2019 and 06 May 2026 and completed at least 12 months of treatment. Data collected included demographic data, ART prescription, HIV‐1 viral loads and clinical outcomes. BMI, renal function, lipids and HbA1c pre‐switch and closest to data censor were compared in those with paired measurements taken at least 12 months apart. Results are presented as absolute number (%) or median (IQR).

**Results**: 142 people were included; baseline demographic data presented below (Table 1). Median exposure to DTG/3TC was 45 months (35.25–49). 133 (93.7%) continued DTG/3TC to the end of the observation period, of whom 130 (97.7%) had an undetectable HIV‐1 RNA (<50 copies/mL). 9 (6.3%) people discontinued DTG/3TC: 3 due to preference for long‐acting injectable ART, 5 for side effects (3 = weight gain, 1 = sleep disturbance, 1 = anxiety) and 1 for low level viraemia attributed to drug−drug interactions (subsequently suppressed on BIC/TAF/FTC). A small increase in BMI (+0.5 kg/m^2^; 26.64 vs. 27.14; 73/143 participants) with no change in HbA1c (35 vs. 36 mmol/mol; 93/142) was observed. A small decrease in eGFR was observed (−5 mL/min/1.73 m^2^; IQR −13–+0; 133/142 participants) below the expected artefactual impact associated with INSTIs; in those with baseline eGFR < 60, a small improvement was noted (+3 mL/min/1.73 m^2^; IQR −3.25–+9.75; 14/142 participants). A significant decrease in non‐HDL cholesterol (−24.7%; 3.85 vs. 2.9 mmol/L; 126/142) was observed, persisting even following the exclusion of people newly prescribed a statin during the observation period (−19.4%; 3.6 vs. 2.9 mmol/L, 67/142).

**Conclusions**: Switch to DTG/3TC was well tolerated and maintained excellent rates of virological control. We observed significant improvements in lipids independent of statins and only minor increases in weight. Switching from bPI‐based regimens to DTG/3TC represents an attractive strategy for optimizing people's metabolic health.

**Reference**

1. Osiyemi O, De Wit S, Ajana F et al. Efficacy and safety of switching to dolutegravir/lamivudine versus continuing a tenofovir alafenamide‐based 3‐ or 4‐drug regimen for maintenance of virologic suppression in adults living with human immunodeficiency virus type 1: results through week 144 from the phase 3, noninferiority TANGO randomized trial. Clin Infect Dis. 2022 Sep 29;75(6):975‐986.

**TABLE 1:**
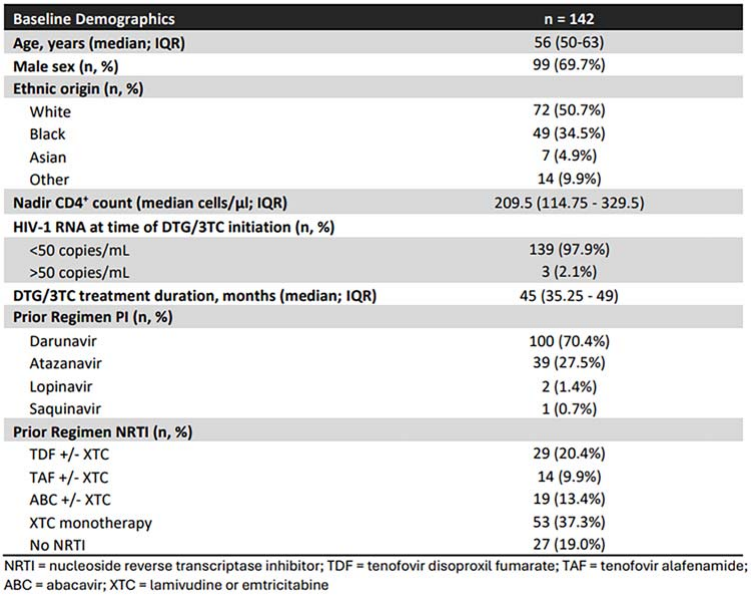
P109 | Participant baseline demographics.

### P110 The Prevalence of Risk Factors for Long‐Acting Injectable Antiretroviral Therapy (CAB+RPV) Failure in a Polish Multicentre Cohort

Andrzej Załęski ^1^, Michał Dowiatt^2^, Agnieszka Lembas^1^, Marta Dobrzyńska^1^, Justyna Orzechowska^3^, Aldona Kot^4^, Justyna Stempkowska‐Rejek^5^, Joanna Osińska^6^, Marcin Dręczewski^7^, Tomasz Dyda^8^, Alicja Wiercińska‐Drapało^1^

^1^Department of Infectious Diseases, Tropical Diseases and Hepatology, Medical University of Warsaw, Warsaw, Poland; ^2^Students’ Scientific Society of the Department of Infectious and Tropical Diseases and Hepatology, Medical University of Warsaw, Warsaw, Poland; ^3^Clinical Department of Infectious Diseases, Medical Center in Łańcut, College of Medical Sciences, University of Rzeszów, Łańcut, Poland; ^4^Department of Infectious Diseases, Medical University of Bialystok, Białystok, Poland; ^5^Department of Infectious Diseases and Hepatology, Medical University of Lublin, Lublin, Poland; ^6^Infectious Diseases Clinical Ward in Ostróda, Department of Family Medicine and Infectious Diseases, University of Warmia and Mazury in Olsztyn, Olsztyn, Poland; ^7^Department of Infectious Diseases, Faculty of Medicine, Medical University of Gdańsk, Gdańsk, Poland; ^8^Molecular Diagnostics Laboratory, Hospital for Infectious Diseases in Warsaw, Warsaw, Poland

**Background**: New therapeutic regimen with long‐acting injectable (LAI) antiretroviral therapy (ART) using cabotegravir (CAB) and rilpivirine (RPV) offers an alternative to daily oral regimens and may improve adherence and efficacy. Nevertheless, recent trials have demonstrated that the presence of ≥2 baseline factors: A6 sub‐subtype, CAB/RPV drug resistance mutations (DRMs) and/or body mass index (BMI) ≥30 kg/m were associated with increased confirmed virologic failure.

**Methods**: In this multicentre, cross‐sectional study, results were obtained from the database of six clinical centres in Poland (ResiPol Group). The inclusion criteria were: HIV infection, age ≥18 years, no previous exposure to ART (genotyping in ART‐naïve individuals) and availability of BMI measurements. HIV subtype, the presence of DRMs for CAB and RPV and patients’ BMI were evaluated. HIV DRMs were interpreted according to the Stanford University HIV Drug Resistance Database. DRMs with positive penalty scores were included in the analysis. Clinical Trials registration number: NCT06661317.

**Results**: A total of *n* = 377 individuals were included in the study. In the study group, HIV subtype A6 was present in 46.68% of patients (*n* = 176/377), with L74I polymorphism in 140 patients (37.14% of the whole cohort). DRMs for RPV in 14.06% (*n* = 53/377) were more frequent (*p* < 0.001) than those for CAB (2.12%, *n* = 8/377) (Figure 1). No patients with combined DRMs to both CAB and RPV were detected. There were 5 individuals (1.33%) with RPV DRMs with penalty score ≥30 and 1 patient (0.27%) with CAB DRMs with score ≥30 (Table 1). BMI ≥30 kg/m^2^ was observed in 7.16% of patients (*n* = 27/377). In total, 5.31% of patients (*n* = 20/377) had 2 risk factors, there were no patients with more than 2 risk factors.

**Conclusions**: The predominance of HIV subtype A6, identified in nearly half of patients, highlights the specific epidemiological profile of the study population and underscores the need for careful pre‐treatment risk stratification. Approximately 5% of patients in cohort had 2 risk factors for LAI CAB/RPV failure, which proves the relative safety of this treatment strategy.

**FIGURE 1:**
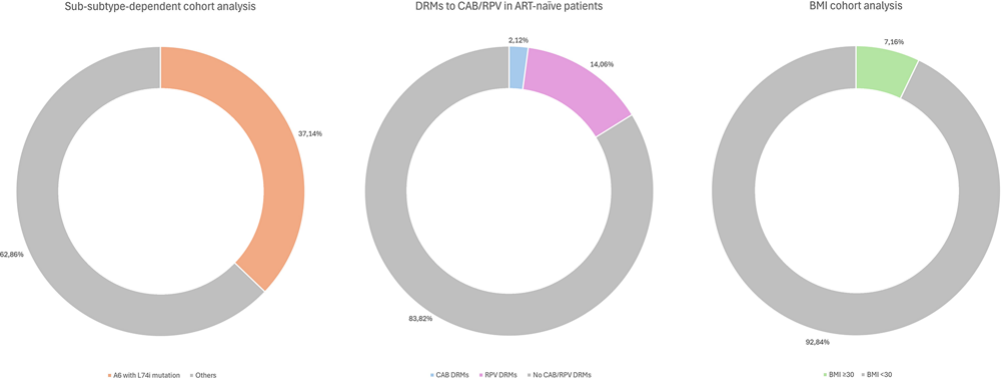
P110 | Prevalence of risk factors for LAI CAB/RPV failure in the study population.

**TABLE 1:**
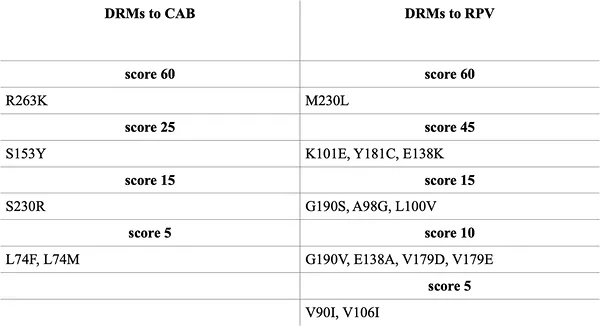
P110 | Drug resistance mutations associated with CAB and RPV, with penalty scores.

### P111 Why Different Individuals Develop Resistance to Different Drugs on Dolutegravir‐Based HIV Therapy

Germander Soothill ^1,2^, Lanxin Zhang^3^, Sara Iannuzzi^3^, Tom Loosli^4,5^, Matthias Egger^6,7^, Roger Kouyos^4,5^, Max von Kleist^3,8^, Andrew Leigh Brown^2^, Katherine E. Atkins^1,9^

^1^Centre for Global Health, School of Population Health Sciences, University of Edinburgh, Edinburgh, UK; ^2^Institute of Evolutionary Biology, University of Edinburgh, Edinburgh, UK; ^3^Project group 5 ‘Systems Medicine of Infectious Diseases’, Robert Koch Institute, Berlin, Germany; ^4^Institute of Medical Virology, University of Zurich, Zurich, Switzerland; ^5^Department of Infectious Diseases and Hospital Epidemiology, University Hospital Zurich, Zurich, Switzerland; ^6^Institute of Social and Preventive Medicine, University of Bern, Bern, Switzerland; ^7^Centre for Infectious Disease Epidemiology and Research, Faculty of Health Sciences, University of Cape Town, Cape Town, South Africa; ^8^Department of Mathematics and Computer Science, Freie Universität Berlin, Berlin, Germany; ^9^Centre for Mathematical Modelling of Infectious Diseases and Department of Infectious Disease Epidemiology and Dynamics, Faculty of Epidemiology and Population Health, London School of Hygiene and Tropical Medicine, London, UK

**Background**: Dolutegravir (DTG)‐based first‐line HIV combination therapy is highly effective and DTG's high genetic barrier to resistance promised to circumvent the resistance burden of earlier regimens. Since WHO approval as the first‐line treatment in 2019, however, resistance to both DTG and its partner drugs is now emerging globally. Why resistance emerges and why the drug to which resistance emerges varies between individuals, remains unknown.

**Methods**: We collated empirical data from 146 treatment‐naïve individuals experiencing treatment failure on DTG with lamivudine or emtricitabine (XTC) and tenofovir (TDF/TAF). We developed a mathematical modelling framework incorporating variability in individual pharmacokinetics and mutation phenotype and simulated 300,000 individuals treated with this regimen for 1 year, validated against the empirical resistance frequencies. During suboptimal adherence, if two of three drugs fall below their suppressive threshold, the third enters a period of functional monotherapy during which resistance may emerge. Logistic regression incorporating functional monotherapy duration and mutation phenotype was used to predict resistance risk.

**Results**: In the empirical dataset, there were 14 cases of treatment failure with resistance: XTC resistance was most common (*n* = 9), followed by DTG (*n* = 3) and dual XTC and DTG (*n* = 2), with no tenofovir resistance. In our simulated cohort, 388 individuals developed treatment failure with drug‐specific resistance proportions consistent with those observed (Figure 1). This pattern is explained by the interplay of two sources of variability. The first source, pharmacokinetic variability, means that even individuals with identical adherence may experience different drugs as functional monotherapy, with the drug experiencing the longest such period at greatest risk of resistance. The second, mutation phenotype variability, determines how likely resistance is to be selected during functional monotherapy. Resistance risk is greatest when a permissive mutation phenotype coincides with prolonged functional monotherapy. This joint condition is rare, so risk is concentrated in a small, distinguishable subset of individuals (Table 1).

**Conclusions**: Our findings provide an explanation for why different individuals develop resistance to different drugs and reframe resistance risk on DTG‐based regimens not as a uniform population‐level threat but an individual‐level one. Given the scale of DTG‐based regimen use, this supports resistance monitoring and improved adherence to preserve first‐line efficacy and suggests that those at greatest risk may be identifiable.

**FIGURE 1:**
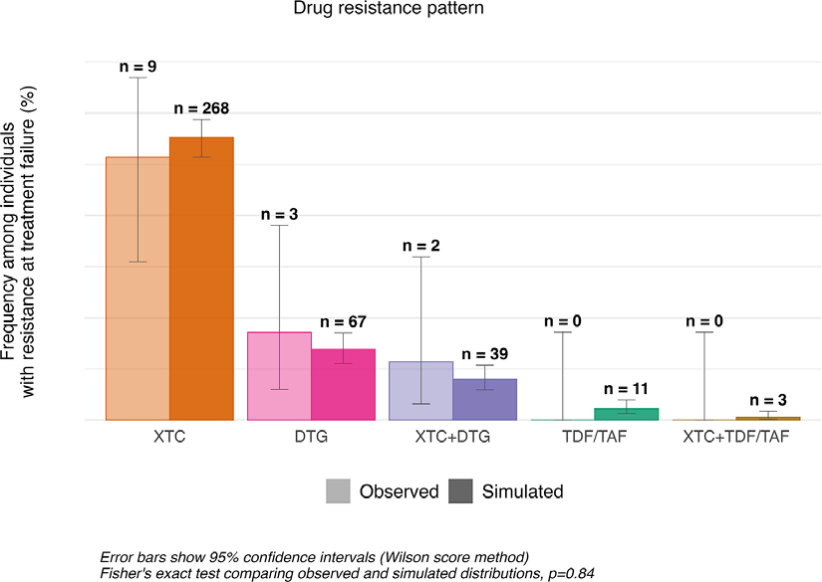
P111 | Simulated resistance frequencies are consistent with observed clinical data.

**TABLE 1:**
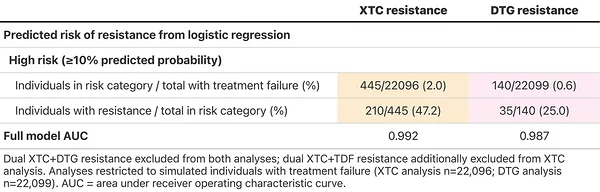
P111 | Joint condition of functional monotherapy and mutation phenotype skews resistance risk.

### P112 Community Pharmacy Readiness for Delivery of Long‐Acting Injectable HIV Pre‐Exposure Prophylaxis in Uganda

Jonathan Derrick Lukubuya ^1^, Eva Agnes Laker Odongpiny^2,3^, Elizabeth B. Katana^4^, Joseph Owori^2^, Ivan Segawa^2^, Freddy Eric Kitutu^1^, Kalidi Rajab^1^, Timothy R. Muwonge^5^, Katrina F. Ortblad^6^, Andrew Mujugira^5,7^

^1^School of Pharmacy, College of Health Sciences, Makerere University, Kampala, Uganda; ^2^The Infectious Diseases Institute, College of Health Sciences, Makerere University, Kampala, Uganda; ^3^Division of Infection and Global Health, School of Medicine, University of St Andrews, St Andrews, UK; ^4^Uganda National Institute of Public Health, Ministry of Health, Kampala, Uganda; ^5^The Infectious Diseases Institute, College of Health Sciences, Makerere University, Kampala, US; ^6^Public Health Sciences Division, Fred Hutchinson Cancer Center, Seattle, WA, Uganda; ^7^Department of Global Health, University of Washington, Seattle, WA, US

**Background**: Community pharmacies could expand decentralized access to long‐acting injectable pre‐exposure prophylaxis (LAI‐PrEP), including 2‐monthly cabotegravir and 6‐monthly lenacapavir. LAI‐PrEP offers alternatives to daily oral PrEP and may reduce adherence, stigma and access barriers. Although some pharmacies deliver oral PrEP, their readiness to deliver LAI‐PrEP remains unclear.

**Methods**: A cross‐sectional study was conducted among licensed professional pharmacy staff (≥18 years) at registered community retail pharmacies in Kampala, Uganda. One respondent per pharmacy completed an interviewer‐administered questionnaire. Readiness was assessed as a composite score (8–40) using Acceptability of Intervention Measure, Intervention Appropriateness Measure and Feasibility of Intervention Measure; rated on a 5‐point Likert scale, with higher scores indicating better readiness. Staff characteristics, pharmacy practices and LAI‐PrEP delivery views influencing readiness were assessed using the Consolidated Framework for Implementation Research. Modified Poisson regression estimated prevalence ratios (PRs) and 95% confidence intervals (CIs) for high readiness (≥32/40).

**Results**: During May 2024–May 2025, 150 staff completed surveys. Median age was 27 years (IQR 25–29); most were male (59%, 89/150), diploma‐trained (80%, 118/150) and 34% (51/150) had oral PrEP training. Mean acceptability (3.8, SD 0.6), appropriateness (3.7, SD 0.5) and feasibility (3.7, SD 0.6) scores were high; 57% (86/150) were ready to deliver LAI‐PrEP. Most lacked injection experience (99%; 148/150), but were willing to train on all PrEP formulations (99%, 149/150), particularly initiation and refill (97%, 144/149). Unprompted client PrEP inquiries were common (90%; 135/150). Few pharmacies administered injections (1.3%, 2/150), had injection resources (2.0%, 3/150) or were integrated into antiretroviral refill systems (1.3%, 2/150). Regulations limited injection delivery (55.6%, 35/63) (Figure 1). Certificate‐level staff showed higher readiness (aPR 4.58; 95% CI: 1.01–20.8; *p* = 0.048).

**Conclusions**: Over half of pharmacies were ready to deliver LAI‐PrEP. Staff perceived this approach as feasible, appropriate and acceptable. Regulatory, training and infrastructure barriers should be addressed for scale‐up.

**FIGURE 1:**
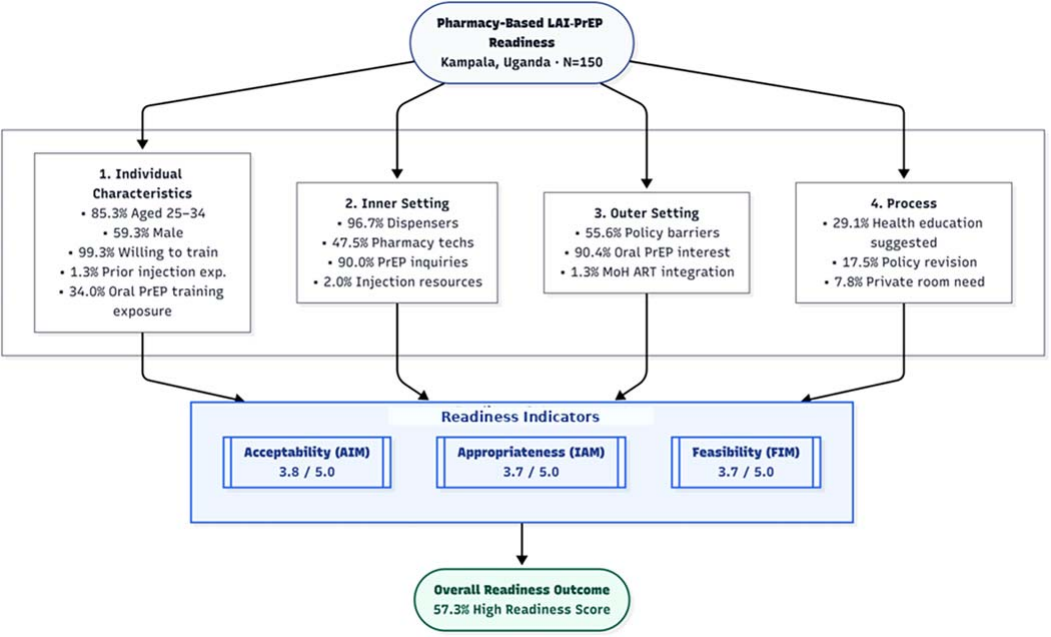
P112 | CFIR domains and readiness indicators for pharmacy‐based delivery of LAI‐PrEP.

### P113 Preferred HIV PrEP Formulation and Delivery Model for Adolescent Girls and Young Women in Fort Portal City: A Mixed Methods Study in Uganda

Jonathan Derrick Lukubuya ^1^, Eva Agnes Laker Odongpiny^2,3^, Elizabeth B. Katana^4^, Joseph Owori^2^, Ivan Segawa^2,5^, Richard Muhindo^6^, Stephen Okoboi^2^, Katrina F. Ortblad^7^, Andrew Mujugira^2,8^, Timothy R. Muwonge^2^

^1^School of Pharmacy, College of Health Sciences, Makerere University, Kampala, Uganda; ^2^The Infectious Diseases Institute, College of Health Sciences, Makerere University, Kampala, Uganda; ^3^Division of Infection and Global Health, School of Medicine, University of St Andrews, St Andrews, UK; ^4^Uganda National Institute of Public Health, Ministry of Health, Kampala, Uganda; ^5^Clinical Epidemiology Unit, College of Health Sciences, Makerere University, Kampala, Uganda; ^6^Department of Nursing, Makerere University College of Health Sciences, Kampala, Uganda; ^7^Public Health Sciences Division, Fred Hutchinson Cancer Center, Seattle, WA, US; ^8^Department of Global Health, University of Washington, Seattle, WA, US

**Background**: Engagement in clinic‐based HIV oral pre‐exposure prophylaxis (PrEP) services among adolescent girls and young women (AGYW, 15–24 years)—who account for roughly a third new infections in Uganda—remains low. Identifying their preferred PrEP formulations and delivery approaches is critical to improving PrEP uptake and adherence. We assessed PrEP preferences among AGYW in Fort Portal, Uganda, where HIV prevalence (∼14%) is nearly three times the national average (∼5%).

**Methods**: We conducted an explanatory sequential mixed‐methods study among community‐ and facility‐based AGYW. Through surveys, participants ranked preferences for PrEP formulations (multipurpose prevention [PrEP+contraceptives], 6‐ and 2‐monthly injections, implant, daily oral pills and monthly vaginal ring) and service delivery models (facility‐based services, online/home delivery, community pharmacies, collection points, peer‐led or youth clinics, mobile refills and school‐based services). Furthermore, participants selected the most and least preferred levels of service delivery attributes (privacy, cost, waiting time, provider, follow‐up, HIV monitoring and location). Outcomes were descriptively analysed. Twenty‐four purposively selected AGYW participated in four focus group discussions, which were analysed thematically.

**Results**: In November 2025, 208 AGYW completed surveys (median age 21 years, interquartile range 19–23 years). Among the PrEP formulations assessed, multipurpose and long‑acting formations were most preferred and the vaginal ring was least preferred. Among delivery models, facility‐based services were most preferred, followed by home‐ and pharmacy‐based services (Figure 1). Participants most preferred services that were free (87.5%), offered in a private room (91.4%), completed within 30 min (75.5%) and delivered by a nurse (54.8%) (Table 1). In FGDs, participants reported liking long‐acting products because of their convenience and discretion: “no one sees anything and it lasts for two months”; they reported disliking the vaginal ring due to comfort concerns: “you feel something that is not supposed to be there.” Participants reported liking healthcare facilities for their reliable counselling and side‐effect management and pharmacies for their fast and private services.

**Conclusions**: PrEP rollout for AGYW should prioritize multipurpose and long‐acting products through facility‐centred services, while scaling up trusted online and home delivery and physical community pharmacies and emphasizing privacy, affordability, short wait times and support from trained health workers.

**FIGURE 1:**
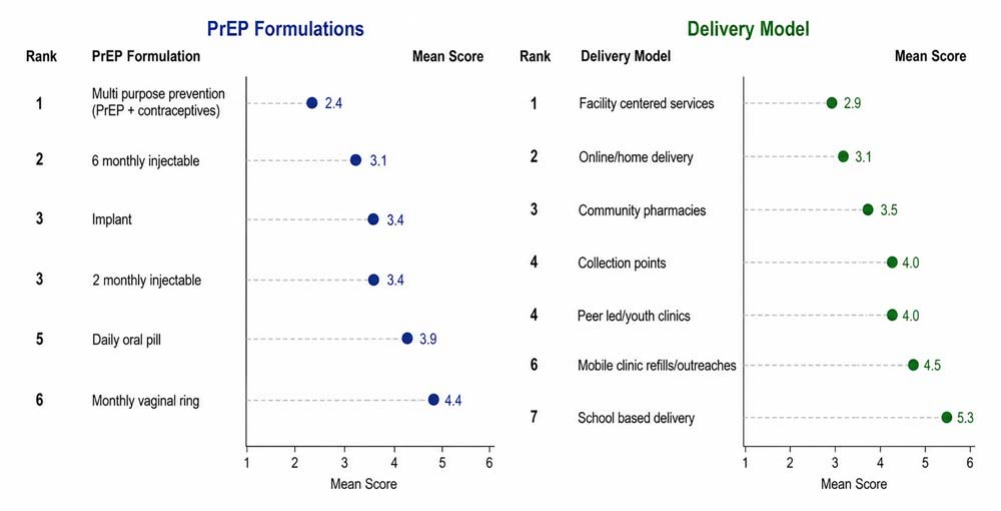
P113 | Ranked preferences for PrEP formulations and delivery models.

**TABLE 1:**
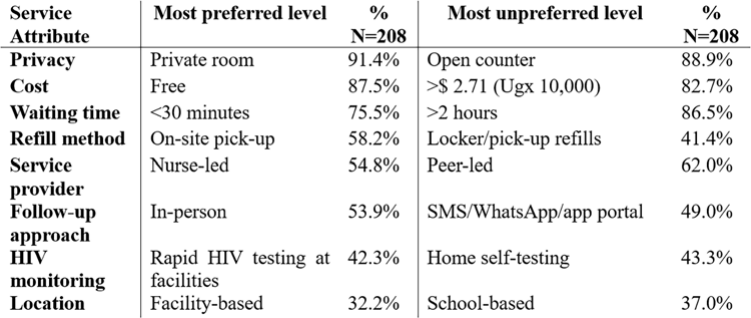
P113 | Preferences for PrEP service delivery attributes.

### P114 Immunovirological Outcomes in a Cohort of Migrants Living With HIV: The Impact of Diagnosis Setting and Social Vulnerability

Lara Neto, Constança Azeredo, Cristina Soeiro, Clara Batista, Eduarda Pena, Isabel Neves, Maria João Gonçalves, Mário Guimarães, Sofia Jordão, Sónia Duarte Rocha, Ricardo Correia de Abreu, Frederico Duarte

Infectious Diseases Department, Local Health Unit of Matosinhos, Matosinhos, Portugal

**Background**: Migration flows to Portugal have expanded substantially, bringing growing numbers of people living with HIV (PLWH) into care under conditions of social vulnerability, including precarious legal and economic status, mental health challenges and disrupted care continuity. We describe the immunovirological profile, vulnerability burden and antiretroviral treatment (ART) outcomes of a cohort of migrant PLWH (MLWH) followed in Portugal (2020–2026).

**Methods**: Retrospective cohort analysis of MLWH initiating follow‐up between October 2020 and March 2026. MLWH were stratified into newly diagnosed (naïve) and those with a prior diagnosis from their country of origin (prior‐HIV). Virological suppression (VS) was defined as plasma HIV‐1 RNA <50 copies/mL. Vulnerability indicators were recorded prospectively. Mann–Whitney U and Fisher's exact tests were used.

**Results**: 199 MLWH were included: 71 naïve (35.7%) and 128 prior‐HIV (64.3%). The cohort predominantly originated from Latin America (*n* = 127, 63.8%), mainly Brazil (*n* = 117, 58.8%) and sub‐Saharan Africa (*n* = 53, 26.6%), mainly Mozambique (*n* = 31, 15.6%). Median age was 36 years in both groups. Naïve patients presented with significantly lower baseline CD4 counts (median 373 vs. 657 cells/µL, *p* < 0.001) and high HIV‐1 RNA at diagnosis (median 109,170 copies/mL). VS at 6 months was achieved in 94.3% (50/53) of naïve versus 95.6% (87/91) of prior‐HIV patients (*p* = 0.71). Social vulnerability was prevalent across both groups: drug use 11.3% versus 15.6%; psychiatric diagnosis 15.5% versus 11.7%; chemsex 1.4% versus 3.9%; sexually transmitted infections (STIs) 16.9% versus 13.3%; prior AIDS‐defining illness 11.3% versus 10.9%. Two deaths occurred, a 29‐year‐old man (prior‐HIV) and a 64‐year‐old woman (naïve). Of 24 patients (12.1%) lost to follow‐up, emigration from Portugal was the predominant reason (*n* = 13, 54.2%).

**Conclusions**: Excellent virological outcomes (> 94% VS at 6 months) were achievable in vulnerable MLWH within a structured follow‐up model, despite high social vulnerability burden. Naïve patients present with more advanced immunodeficiency, highlighting the urgent need for proactive screening, rapid linkage to care and integrated psychosocial support tailored to this population's unique challenges.

### P115 Four‐Year Experience With Long‐Acting Cabotegravir–Rilpivirine in a Multicentre Cohort in Italy: Update From the LONGITUDE Study

Francesco Vladimiro Segala ^1^, Lolita Sasset^1^, Marina Malena^2^, Alberto Zuppiroli^3^, Silvia Cretella^3^, Daniela Piacentini^4^, Emanuela Lattuada^5^, Stefano Nicolé^6^, Giuliana Battagin^6^, Fabio Rigo^6^, Giada Fasani^7^, Anna Rigo^7^, Cinzia Puzzolante^8^, Lorenzo Badia^9^, Maria Mazzitelli^10^, Mariacristina Rossi^11^, Massimiliano Lanzafame^12^, Leonardo Calza^3^, Cristina Mussini^8^, Annamaria Cattelan^13^, on behalf of LONGITUDE Study Group

^1^Infectious and Tropical Diseases Unit, Padua University Hospital, Padua, Italy; ^2^Unità Operativa Malattie Infettive e Tropicali di Rovigo, Rovigo Hospital, Rovigo, Italy; ^3^Unità Operativa Malattie Infettive, Policlinico S. Orsola, IRCCS Azienda Ospedaliero‐Universitaria di Bologna, Bologna, Italy; ^4^Unità Operativa Malattie Infettive e Tropicali di Belluno, Belluno Hospital, Belluno, Italy; ^5^Unità Operativa Malattie Infettive e Tropicali di Trento, Trento Hospital, Trento, Italy; ^6^Unità Operativa Malattie Infettive e Tropicali di Vicenza, Vicenza Hospital, Vicenza, Italy; ^7^Unità Operativa Malattie Infettive e Tropicali di Verona, Azienda Ospedaliera Universitaria di Verona, Verona, Italy; ^8^Unità Operativa Malattie Infettive e Tropicali di Modena, University of Modena and Reggio Emilia, Modena, Italy; ^9^Infectious Diseases Outpatient Clinic, Azienda USL of Imola, Imola, Italy; ^10^Dipartimento di Sicurezza e Bioetica—Sezione di Malattie Infettive, Università Cattolica del Sacro Cuore, Roma, Italy; ^11^Unità Operativa Malattie Infettive e Tropicali di Treviso, Treviso Hospital, Treviso, Italy; ^12^Unità Operativa Malattie Infettive e Tropicali di Trento, Trento, Italy; ^13^Department of Molecular Medicine, University of Padua, Padua, Italy

**Background**: Since 2022, long‐acting (LA) antiretroviral therapies have emerged as potential game‐changers in supporting a long and healthy life in PLWH. Cabotegravir/rilpivirine (CAB/RPV) is the first combination approved in Italy for virologically suppressed PLWH with no known mutations or failure to NNRTI and INSTI. However, prospective, real‐life data about long‐term effectiveness and tolerance of CAB/RPV‐LA are lacking.

**Methods**: This is a multicentre, prospective cohort study within the established LONGITUDE cohort study in Italy. All PLWH who received at least one dose of CAB/RPV‐LA since its commercialization were eligible for inclusion. Clinical and laboratory data were collected to assess incidence and risk factors of treatment discontinuation (TD) and virological failure (VF), defined as overt viraemia (≥200 copies/mL) leading to treatment withdrawal. Survival analysis, Fine–Gray and Cox proportional hazards modelling were applied to investigate probability and predictors of TD.

**Results**: Among the 476 patients included in this updated 4‐year cohort, most were males (*n* = 336, 78%), MSM (*n* = 239, 62%) with a median age of 48 years (Table 1). Treatment discontinuation occurred in 64 people (13.3%) at an incidence rate of 0.642 per 100 person‐months (95% CI: 0.49–0.82), after a median follow up of 21.2 months (Q1–Q3 11–29), but reaching 20% at 42 months (Figure 1A). Virological failure was documented in four cases: all were switched to oral therapy with DRV/c/FTC/TAF and subsequently achieved viral suppression. Main reasons for TD were adverse events/injection site reactions (*n* = 36, 57%) and patient decision (*n* = 10, 15.9%). At multivariable Cox hazard analysis, the only predictor for TD was a longer HIV history (aHR 1.04, *p* = 0.011), which remained significant after filtering for competing events (2 deaths and 1 transfer to different region). Body weight, kidney function and liver enzymes remained stable during treatment (Figure 1B).

**Conclusions**: In our cohort, over a 4‐year of observation, TD risk increased steadily over time, was primarily driven by local side effects and was more likely in people with a longer HIV history. Virological failure was rare and no effect was evident on body weight, liver and kidney function.

**FIGURE 1:**
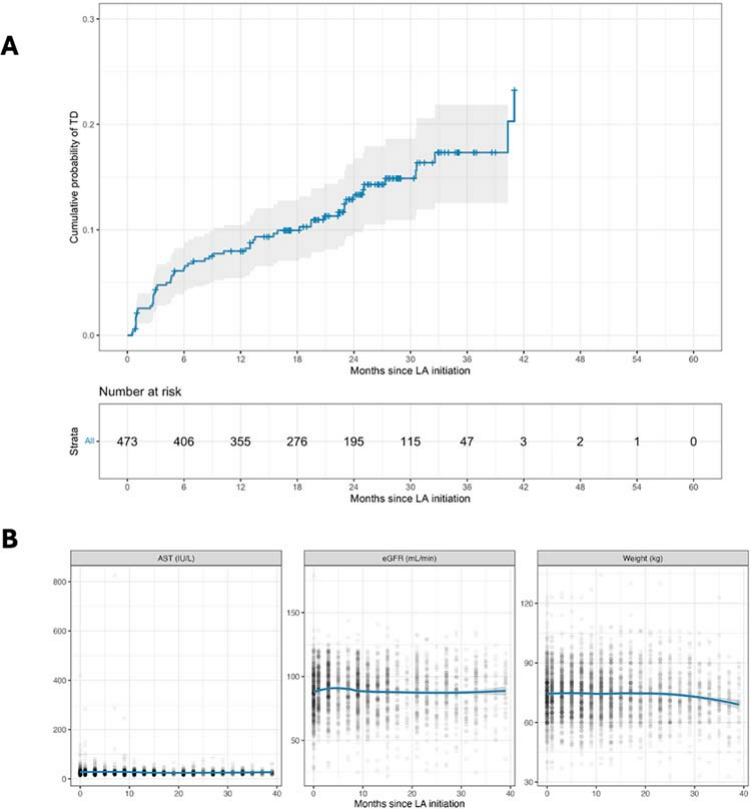
P115 | (A) Kaplan–Meier curve for treatment discontinuation and (B) AST, eGFR and weight trajectories over follow‐up.

**TABLE 1:**
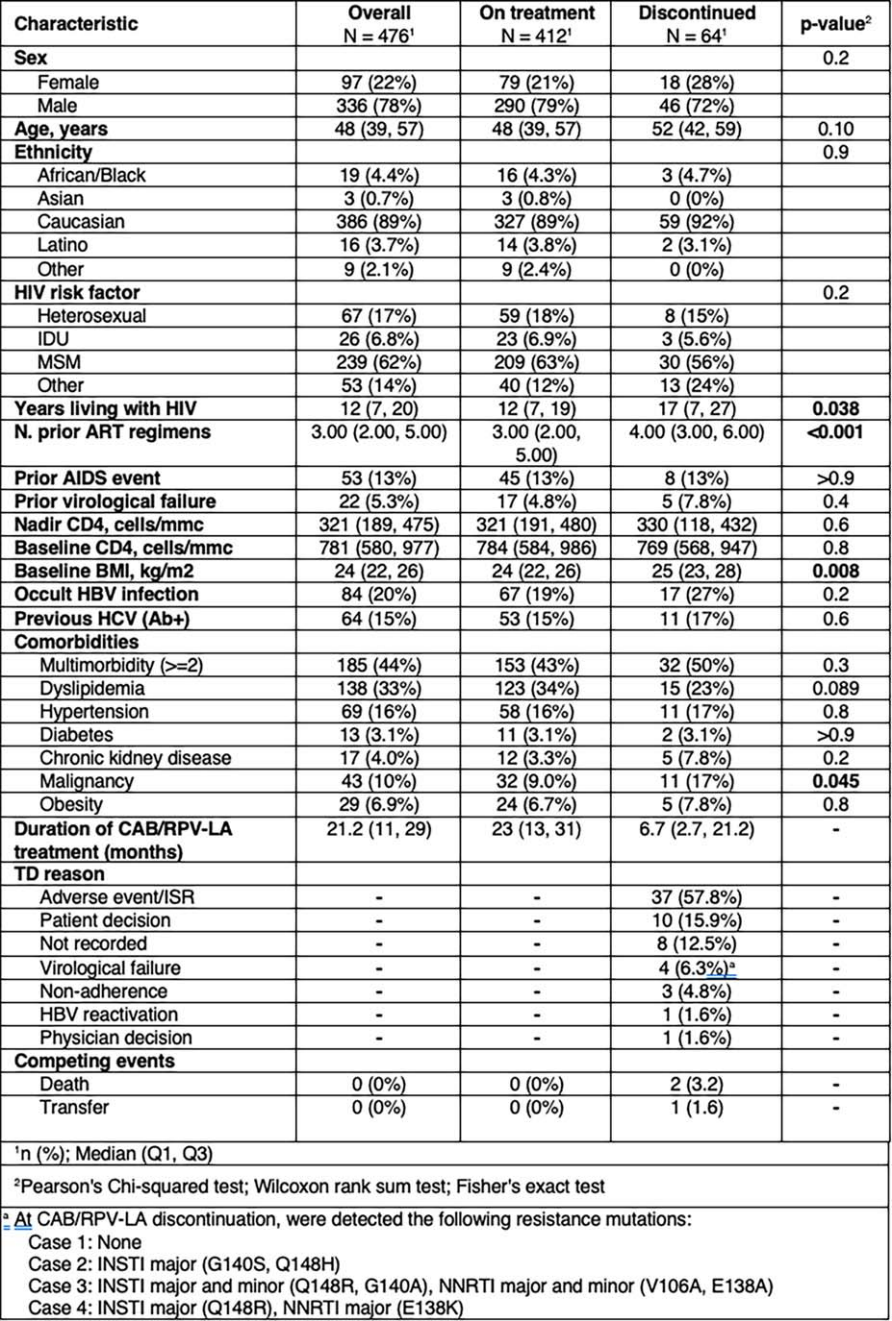
P115 | Baseline characteristics, overall and by treatment discontinuation.

### P116 Long‐Acting Cabotegravir and Rilpivirine in Routine Clinical Practice: Virological Outcomes and Patient‐Reported Reasons for Switching From Oral Antiretroviral Therapy

Tiago Neves, Ana Jorge Martins, Gonçalo Cruz, Helena Alves, Sandra Tejo, Cristina Valente

Serviço de Doenças Infeciosas da Unidade Local de Saúde de Coimbra, Coimbra, Portugal

**Background**: Long‐acting injectable cabotegravir plus rilpivirine (CAB+RPV‐LA) provides an alternative to daily oral antiretroviral therapy (ART). This study aimed to characterize a real‐world cohort of people living with HIV‐1 who initiated CAB+RPV‐LA, evaluating virological outcomes and patient‐reported reasons for switching from oral ART.

**Methods**: An observational study was conducted including 166 people living with HIV‐1 who initiated CAB+RPV‐LA at a tertiary hospital between October 2024 and May 2026. Demographic and clinical data were collected through medical record review. A questionnaire assessed satisfaction with oral ART and potential motivations for switching, including concerns regarding HIV status disclosure, treatment‐related reminders of HIV infection and missed oral doses.

**Results**: Among the 166 participants, 80.7% were cisgender men and the median age was 49 years (20–79; IQR 36–59). Most participants were Portuguese (62.7%). HIV acquisition occurred predominantly among men who have sex with men (54.9%) and through heterosexual transmission (38.9%). Median ART duration before switching was 10 years (IQR 5–16). The most common previous regimens were dolutegravir/lamivudine (45.2%), dolutegravir plus emtricitabine/tenofovir disoproxil fumarate (13.9%), followed by rilpivirine‐based regimens (13.2%). Up to 145 participants completed the questionnaire before receiving their first long‐acting ART injection. Overall, 88.3% reported satisfaction with oral ART. Nevertheless, 66.7% reported missed doses at least occasionally, 66.2% reported concerns regarding disclosure of their HIV status and 60.3% reported that daily treatment reminded them of their HIV infection. Before switching, 95.8% had HIV RNA <50 copies/mL. Virological outcomes were assessed in 150 participants who remained on treatment and had received at least two injections by the end of the study period. Among evaluable participants, 95.3% maintained HIV RNA <50 copies/mL, while 4.7% had 50–199 copies/mL. A total of 11 participants discontinued or interrupted treatment for various reasons, most notably due to virological failure (*n* = 2) and adverse events (*n* = 2).

**Conclusions**: CAB+RPV‐LA demonstrated high effectiveness in routine clinical practice, with 95.3% of evaluable participants maintaining HIV RNA <50 copies/mL. Despite high satisfaction with oral ART, concerns regarding adherence, HIV status disclosure and the psychological burden of daily treatment remained common. Long‐acting injectable ART may address quality‐of‐life and privacy‐related needs beyond virological control.

### P117 Geographic and Socioeconomic Determinants of Long‐Acting Cabotegravir Plus Rilpivirine Access and Outcomes: A Real‐World Cohort Study From Southern Spain

Claudia Ferreira‐Tata, Laura Ruiz‐Torres, Diana Corona‐Mata, Angela Camacho, Marina Gallo, Andres Baumela, Isabel Carmona, Antonio Doblas, Antonio Rivero, Ignacio Pérez‐Valero

Infectious Diseases Department, Reina Sofia University Hospital, Maimonides Institute for Biomedical Research of Cordoba (IMIBIC), University of Cordoba, Cordoba, Spain

**Background**: Long‐acting cabotegravir plus rilpivirine (CAB+RPV LA) requires bimonthly in‐clinic injections. Centralized delivery models may create geographic access barriers. In Córdoba province, Spain (17,141 km^2^), administration is restricted to a single tertiary hospital (HURS). We evaluated whether geographic distance and neighbourhood socioeconomic status (SES) influence treatment uptake and outcomes.

**Methods**: Retrospective open cohort study. Multivariable logistic regression compared 153 CAB+RPV LA recipients with 1478 non‐recipients. Geographic access was operationalized as residence outside Córdoba city (binary) and estimated driving time to HURS (continuous). Neighbourhood SES was estimated using 2022 Spanish Tax Agency gross income by postal code. Among recipients, associations between geo‐SES variables and time to discontinuation (Cox regression), missed injections and virological suppression were analysed.

**Results**: Compared with non‐recipients, people with HIV (PWH) on CAB+RPV LA were younger (median 47 vs. 57 years), had higher CD4 nadir (332 vs. 168 cells/µL) and lower prior AIDS history (30.9% vs. 50.5%; all *p* < 0.001). In multivariable analysis, residing outside Córdoba city independently predicted lower uptake (adjusted OR 0.560, 95% CI: 0.387–0.809; *p* = 0.002), whereas driving time (adjusted OR 1.013/10 min; *p* = 0.690) and income (OR 1.023/€1000; *p* = 0.348) did not—demonstrating a binary urban/provincial barrier rather than a continuous gradient (Figure 1). Among 153 recipients (median follow‐up 567 days; 247.5 patient‐years), 28 (18.3%) discontinued treatment (11.3/100 patient‐years; 12‐month Kaplan‒Meier retention 86.9%). Virological suppression was 91.5% at last follow‐up. The overall missed‐injection rate was 4.5% (74/1651 expected doses). Neither driving time, distance, nor income predicted discontinuation, missed injections or virological suppression (91.5% suppressed; all *p* > 0.20). Paradoxically, PWH residing > 60 min from HURS had lower discontinuation rates than those ≤15 min (8.3% vs. 22.2%). Among 45 PWH with ≥1 missed injection, greater driving time correlated with fewer total missed doses (Spearman ρ = −0.349; *p* = 0.016; Figure 1).

**Conclusions**: CAB+RPV LA access is constrained by a binary urban/provincial barrier—not a continuous distance gradient—and is unrelated to SES. A missed‐injection rate of 4.5% confirms high real‐world adherence. Once in treatment, neither geography nor income impairs retention or virological outcomes; longer‐distance PWH paradoxically showed stronger adherence motivation. These findings support decentralizing LA‐ART to satellite sites to close the access gap without compromising outcomes.

**FIGURE 1:**
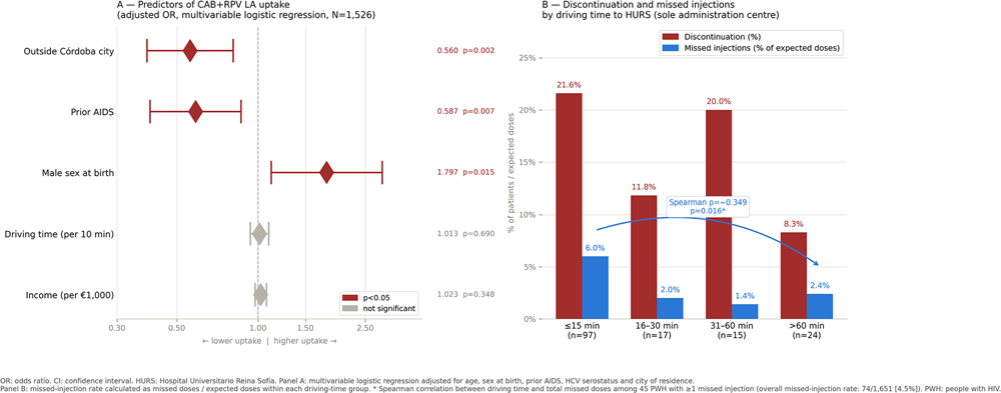
P117 | Geographic determinants of CAB+RPV LA uptake and outcomes.

### P118 Real‐World Outcomes With Long‐Acting Cabotegravir Plus Rilpivirine in People With HIV‐1 Aged ≥50 Years: A Systematic Literature Review

Andrea Calcagno ^1^, Kristine Erlandson^2^, Inês Augusto^3^, Ian Jacob^4^, Patricia De los Rios^5^, Santiago Cenoz Gomis^6^, Ta‐Fen Hwang^7^, Bryn Jones^4^

^1^Department of Translational Medicine, University of Eastern Piedmont, Novara, Italy; ^2^Department of Medicine, University of Colorado Anschutz, Aurora, CO, US; ^3^ViiV Healthcare, Algés, Portugal; ^4^ViiV Healthcare, London, UK; ^5^ViiV Healthcare, Montreal, Canada; ^6^ViiV Healthcare, Madrid, Spain; ^7^ViiV Healthcare, Singapore, Singapore

**Background**: By 2030, over 70% of people with HIV are expected to be ≥50 years, a demographic facing increased multimorbidity and polypharmacy. Guidelines recommend treatment simplification to minimize drug interactions and cumulative toxicities while maintaining viral suppression. Cabotegravir (CAB) + rilpivirine (RPV) is the first and only complete long‐acting (LA) injectable regimen approved for people with HIV‐1 who are virologically suppressed. Real‐world evidence (RWE) is vital to better characterize clinical outcomes in this ageing population using CAB+RPV LA.

**Methods**: An ongoing systematic literature review of RWE studies for CAB+RPV LA was performed (PRISMA guidelines; Embase, PubMed, Cochrane Library and international congresses; January 2020–October 2025). Publications reporting outcomes in ≥10 people aged ≥50 years were synthesized descriptively.

**Results**: A total of 432 publications, representing 121 unique cohorts and 39,434 people with HIV‐1, reported CAB+RPV LA use. Of these, 27 publications (20 discrete cohorts) described outcomes for 5009 people aged ≥50 years; 12 with ≥10 people are included here. At baseline, 2 publications reported high rates of polypharmacy (65%–68%) and multimorbidity (35%–68%). Six publications reported effectiveness, 3 treatment persistence, 3 tolerability, 5 adherence and 2 patient experience. Three publications (*n* = 1518) reported virological suppression (VS) rates of 93%−98%; Figure 1 reports VS rates by age groups. Six publications (*n* = 2640) reported a total of 25 virological failures (VFs) (follow‐up time 13–24 months), of which 3 reported 0 VFs and 3 reported rates of 1%−2%. Three publications (*n* = 1498) reported persistence rates of 77%−89%. Three publications (*n* = 1471) described treatment discontinuation rates of 8%−16% due to tolerability; two publications reported injection site reaction rates of 2% (19/1156) and 9% (7/81) as discontinuation reasons. Adherence (various definitions) was high in people aged ≥50 years (Table 1). One publication (*n* = 1156) reported 48% of people indicated comfort/quality of life as the main reason for switching to CAB+RPV LA; another (*n* = 113) reported increased treatment satisfaction with CAB+RPV LA over 12 months.

**Conclusions**: Real‐world evidence supports the use of CAB+RPV LA in people aged ≥50 years with high multimorbidity and polypharmacy prevalence, demonstrating high VS, persistence and adherence, with low VF rates and improved treatment satisfaction in this increasing demographic.

**FIGURE 1:**
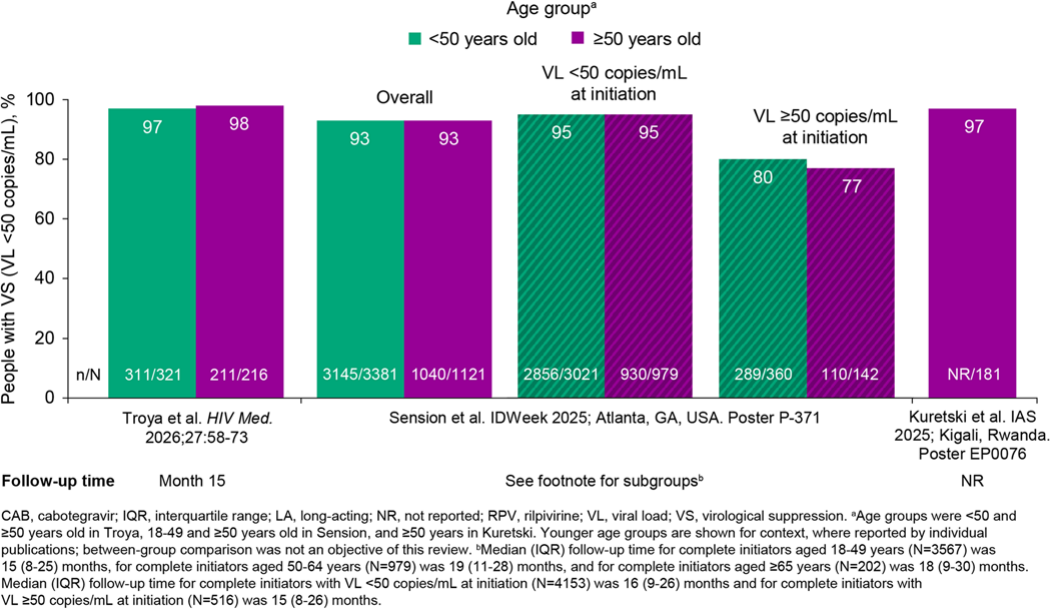
P118 | Virological suppression rates in people using CAB+RPV LA by age groups.

**TABLE 1:**
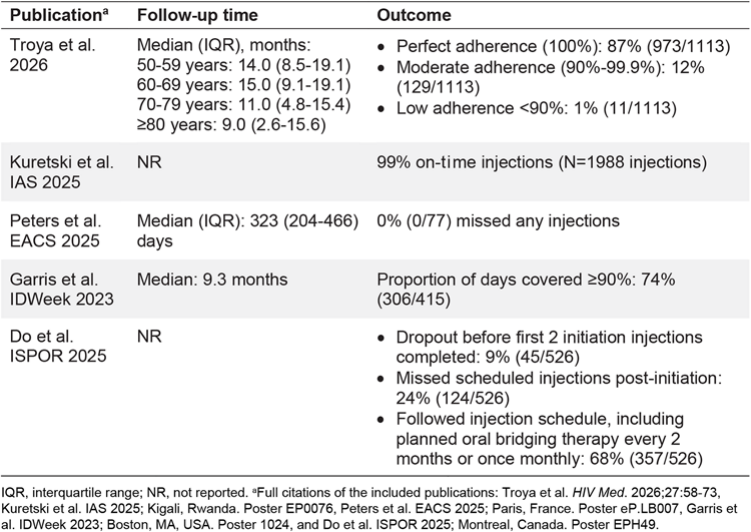
P118 | Adherence outcomes among people aged ≥50 years.

### P119 CABO‐CHANCE: Long‐Acting Cabotegravir Plus Rilpivirine in ART‐Experienced People With HIV (24‐Month Real‐World Outcomes)

Carmen Hidalgo‐Tenorio ^1^, María Aguilera García^2^, Ignacio De los Santos Gil^2^, Andres Ruiz‐Sancho^3^, Antonio Rivero^4^, Ana Lopez Lirola^5^, Mohamed Omar^6^, Alberto Romero^7^, Enrique Bernal^8^, Onofre Martínez Madrid^9^, Teresa Lopez^10^, Patricia Sorni^11^, Paula Garcia Ocaña^12^, Santiago Moreno^13^, Isabel SanJoaquin^14^, Jose Ramón Blanco^15^, Coral Garcia Vallecillos^1^

^1^Infectious Diseases Service, Hospital Universitario Virgen de las Nieves, Granada, Spain; ^2^Infectious Diseases Service, Hospital Universitario de La Princesa, Madrid, Spain; ^3^Infectious Diseases Service, Hospital Universitario San Cecilio, Granada, Spain; ^4^Infectious Diseases Department, Hospital Universitario Reina Sofia, Cordoba, Spain; ^5^Unit of Infectious Diseases, Hospital Universitario de Canarias, San Cristóbal de La Laguna, La Laguna, Spain; ^6^Infectious Diseases Unit, Hospital Universitario de Jaén, Jaen, Spain; ^7^Infectious Diseases Unit, Hospital Universitario de Puerto Real, Cadiz, Spain; ^8^Infectious Diseases Unit, Hospital Universitario Reina Sofia, Murcia, Spain; ^9^Infectious Disease Unit, Hospital General Universitario Santa Lucía, Cartagena, Spain; ^10^Infectious Diseases Unit, Hospital Santa Ana, Motril, Spain; ^11^Unit of Infectious Diseases Hospital Son Llàtzer, Mallorca, Spain; ^12^Infectious Diseases Unit, Hospital Universitario de Jerez, Cadiz, Spain; ^13^Infectious Diseases Service, Hospital Universitario Ramón y Cajal, Madrid, Spain; ^14^Unit of Infectious Diseases, Hospital Lozano Blesa, Zaragoza, Spain; ^15^Hospital Universitario San Pedro, Logroño, Spain

**Background**: CABO‐CHANCE study evaluated long‐term effectiveness, safety and impact on inflammatory markers, quality of life, sleep, health satisfaction and stigma experience over 2 years of CAB+RPV‐LAI in ART‐experienced people with HIV (PWH).

**Methods**: A prospective, longitudinal, multicentre study was conducted in 15 Spanish hospitals (recruitment: June 2023−January 2024). It included virologically suppressed PWH (HIV‐1 RNA <50 c/mL for ≥6 months) switched from oral ART to CAB+RPV‐LAI. Anthropometrics, CD4 and CD8 counts, viral load, creatinine clearance, lipid profile and inflammatory markers were recorded at baseline and 3, 6, 12, 18 and 24 months. As well as patient‐reported outcomes using the WHOQOL‐HIV‐BREF, HIV Stigma Scale for use in Spain, Pittsburgh Sleep Quality Index and questionnaires on health satisfaction. Adverse events were gathered in all study visits. Virological effectiveness at 24 M was primarily described according to the proportion of evaluable participants with HIV‐1 RNA > 50 copies/mL according to the FDA Snapshot. Quantitative longitudinal endpoints were re‐analysed using a repeated‐measures approach to account for within‐subject correlation; generalized estimating equations were used as the primary framework, with pairwise contrasts versus baseline considered secondary and adjusted for multiplicity.

**Results**: Among 269 PWH (mean age 45.6 years; 88.8% male), 210 completed 24 M follow‐up, 4 (15%) were lost to follow‐up and15 (5.5%) has discontinued. mITT effectiveness was 7.0% of participants (15/210) with HIV‐1 RNA ≥50 copies/mL, all low‐level viraemias (VL from 54 to 161 copies/mL) and 3 (1.1%) had virological failure (two consecutive HIV‐1 RNA VLs ≥200 copies/mL). HIV‐1 RNA ≤50 copies/mL was observed in 73.9% by ITT and 94.7% by PP. For 24 M, no oral bridging was required. In the repeated‐measures analysis, the CD4/CD8 ratio continued to improve over follow‐up (median 1.01−1.07; *p* = 0.036), and renal function (CKD‐EPI median 90−93 mL/min/1.73 m^2^; *p* < 0.0001). Among inflammatory markers, D‐dimer (*p* < 0.0001) and fibrinogen (*p* = 0.001) decreased over follow‐up. Regarding lipid profile, HDL cholesterol increased (median 46−48 mg/dL; *p* = 0.003) and the total cholesterol/HDL ratio decreased (*p* = 0.026). Global quality of life improved from baseline to M24 (adjusted *p* = 0.037) (Table 1).

**Conclusions**: In this multicentre RWD, CAB+RPV LAI demonstrated durable virological effectiveness, with sustained immunological benefit, improvements in inflammatory markers and favourable changes in renal function and lipid parameters, and quality of life.

**TABLE 1:**
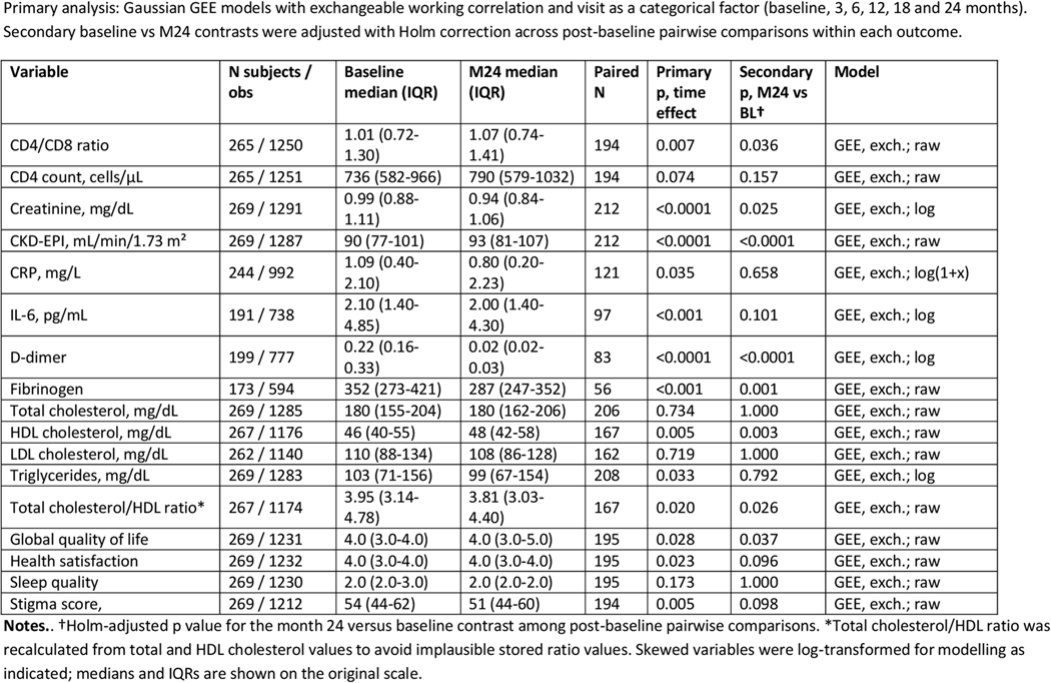
P119 | Longitudinal repeated‐measures analysis through month 24.

### P120 Real‐World Virologic Effectiveness Outcomes With Long‐Acting Cabotegravir Plus Rilpivirine in Women: A Systematic Literature Review

María José Galindo Puerto ^1^, William Short^2^, Inês Augusto^3^, Ian Jacob^4^, Emilio Letang^5^, Patricia De los Rios^6^, Chinyere Okoli^4^, Bryn Jones^4^

^1^Unit of Infectious Diseases, Hospital Clínico Universitario de Valencia, Instituto de Investigación Clinica INCLIVA, Valencia, Spain; ^2^Division of Infectious Diseases, Department of Medicine, Perelman School of Medicine, University of Pennsylvania, Philadelphia, PA, US; ^3^ViiV Healthcare, Algés, Portugal; ^4^ViiV Healthcare, London, UK; ^5^ViiV Healthcare, Madrid, Spain; ^6^ViiV Healthcare, Montreal, Canada

**Background**: Although > 50% of people with HIV‐1 are women, they remain historically underrepresented in clinical research, limiting evidence to inform their care. Cabotegravir (CAB) plus rilpivirine (RPV) is the first and only complete long‐acting (LA) injectable regimen for people with HIV‐1 who are virologically suppressed, with real‐world evidence (RWE) demonstrating high effectiveness and good tolerability in diverse populations. Evaluating RWE is fundamental to better characterize clinical outcomes among women in routine clinical practice.

**Methods**: An ongoing systematic literature review of RWE studies for CAB+RPV LA was performed (PRISMA guidelines; Embase, PubMed, Cochrane Library and international congresses; January 2020–July 2025). Publications reporting outcomes in ≥10 cisgender and transgender women were synthesized descriptively.

**Results**: Overall, 432 publications, representing 121 unique cohorts and 39,434 people with HIV‐1, reported CAB+RPV LA use. Of these, 78 publications (47 discrete cohorts) described outcomes for 3518 cisgender women and 4 described outcomes for 60 transgender women; 34 publications with ≥10 people are included here. Eighteen publications reported effectiveness, 3 persistence, 5 adherence and 1 patient experience. Additionally, 3 publications described virological and neonatal outcomes in women receiving CAB+RPV LA during pregnancy. Virological suppression (VS) rates were 82%−100% across 5 publications (*n* = 551); 3 of which reported 100%. Importantly, VS rates were similar between women and men (Figure 1). Thirteen publications (*n* = 1504) reported 27 virological failures (VFs), with VF rates ranging from 0% to 4% across publications; 6 of these reported 0% VF rate. Five publications (*n* = 327) reported resistance at VF; only 1 described integrase strand transfer inhibitor and non‐nucleoside reverse transcriptase inhibitor resistance in 2/64 women. Of 3 publications reporting persistence (*n* = 975), 1 showed 78% of people remained on CAB+RPV LA and 2 reported discontinuation rates of 20% and 8%; the latter stated local injection reactions (2%) and systemic adverse events (2%) as reasons. Table 1 reports adherence outcomes. One publication identified inconvenience of carrying oral medication or better alignment of long‐acting injectables with daily lifestyle as the main reasons for switching to CAB+RPV LA.

**Conclusions**: Real‐world evidence supports the effectiveness of CAB+RPV LA in women, consistent with findings from clinical trials, with low VF rates and similar outcomes to men.

**FIGURE 1:**
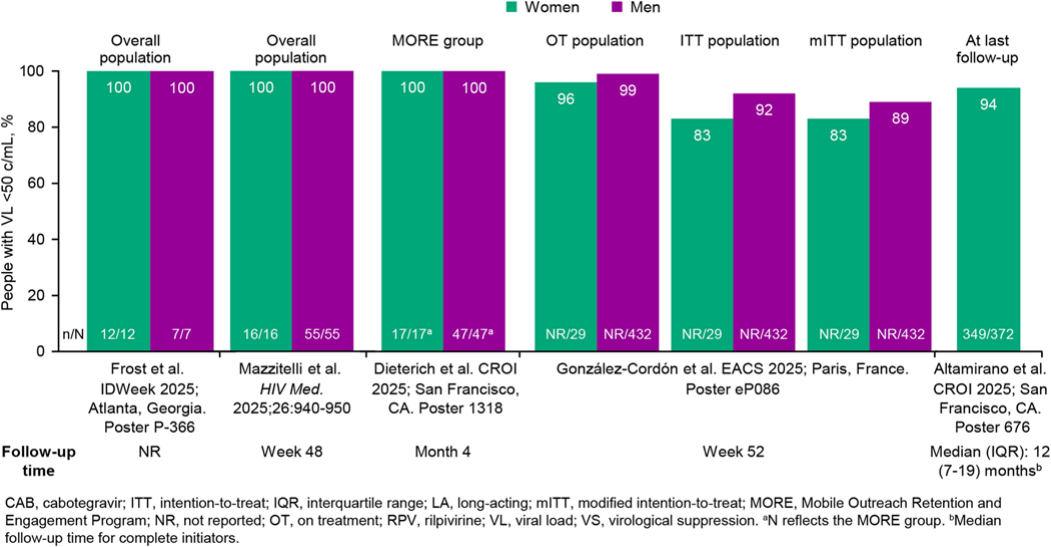
P120 | Virological suppression rates in women and men receiving CAB+RPV LA.

**TABLE 1:**
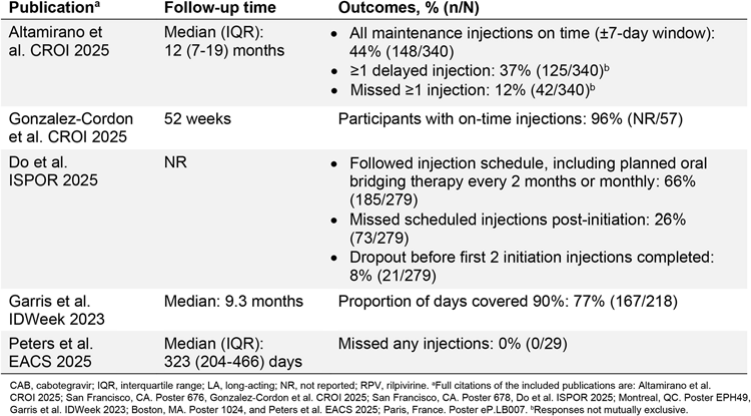
P120 | CAB+RPV LA adherence outcomes in women.

### P121 Real‐World Implementation of Long‐Acting Cabotegravir and Rilpivirine in UK NHS HIV Clinics

Claire Norcross^1,2^, Clea Tanner^1^, Kate Alford^1^, Kyle Ring^3,4^, Ankur Gupta‐Wright^5,6^, Bazga Ali^7^, Dan Clutterbuck^8^, Julie Fox^9^, Emily Clarke^10^, Alison Uriel^11^, Jill Dixon^12^, Paola Cicconi^13^, Jaime Vera ^1,2^, Caroline Sabin^14^, Fiona Cresswell^1,2^

^1^Brighton & Sussex Medical School, Brighton, UK; ^2^University Hospitals Sussex NHS Foundation Trust, Brighton, UK; ^3^Barts Health NHS Trust, London, UK; ^4^Queen Mary University of London, London, UK; ^5^Bristol NHS Foundation Trust, Bristol, UK; ^6^Imperial College London, London, UK; ^7^Cardiff and Vale University Health Board, Cardiff, UK; ^8^NHS Lothian, Edinburgh, UK; ^9^Guy's & St Thomas’ NHS Foundation Trust, London, UK; ^10^Liverpool University Hospitals NHS Foundation Trust, Liverpool, UK; ^11^Manchester University NHS Foundation Trust, Manchester, UK; ^12^Newcastle upon Tyne Hospitals NHS Foundation Trust, Newcastle, UK; ^13^Oxford University Hospitals NHS Foundation Trust, Oxford, UK; ^14^University College London, London, UK

**Background**: Long‐acting cabotegravir and rilpivirine (LA CAB/RPV) offers an alternative to daily oral antiretroviral therapy (ART) and represents a paradigm shift in HIV management. Routine use of long‐acting therapy as part of UK government‐approved care began in 2022. More data on the successes and challenges of implementation of LA CAB/RPV in routine care settings are needed.

**Methods**: At nine UK NHS HIV services, adults prospectively switching to LA CAB/RPV or switched in the prior 6 months were invited to join an observational cohort study. A control group continuing oral ART (matched for age, sex, ethnicity, sexual orientation, ART duration) was also recruited. Baseline demographics, treatment switches, missed/late injections and virological outcomes at 12 months of follow‐up are described by treatment group.

**Results**: A total of 167 participants were included, 114 on LA CAB/RPV and 53 on oral ART, of whom 77% (129/167) were men (Table 1). Median age was 46 years (IQR 38–54), 52% (86/167) were of white ethnicity, median time since HIV diagnosis was 14 years (IQR 9−20) and 95% (159/167) had HIV RNA <50 c/mL at baseline. Among those receiving LA CAB/RPV, none had evidence of prior resistance to integrase inhibitors, but one had known non‐nucleoside reverse transcriptase inhibitor resistance (K103N). After 12 months of follow up, 17/114 (14.9%) on LA CAB/RPV and 4/53 (7.5%) on oral ART had at least one HIV RNA 50–199 c/mL. No HIV RNA readings ≥200 c/mL were observed. Three (3/114, 2.6%) individuals had switched off LA CAB/RPV, primarily due to injection site reactions. Four (4/53, 9.4%) individuals in the control arm had switched treatment, citing drug−drug interactions and adherence challenges. Oral bridging was provided on three occasions to cover missed injections. Nine out of 736 (1.2%) injections administered during the follow‐up period were administered out of window, of which five were late. No virological failure was observed at 12 months of follow‐up. No drug‐related serious adverse events were reported.

**Conclusions**: At 12 months of LA CAB/RPV, there were no episodes of virological failure; however, viral blips were common (14.9% of participants). The regimen was well tolerated with 97.4% (111/114) continuing LA CAB/RPV and all missed injections managed with oral bridging.

**TABLE 1:**
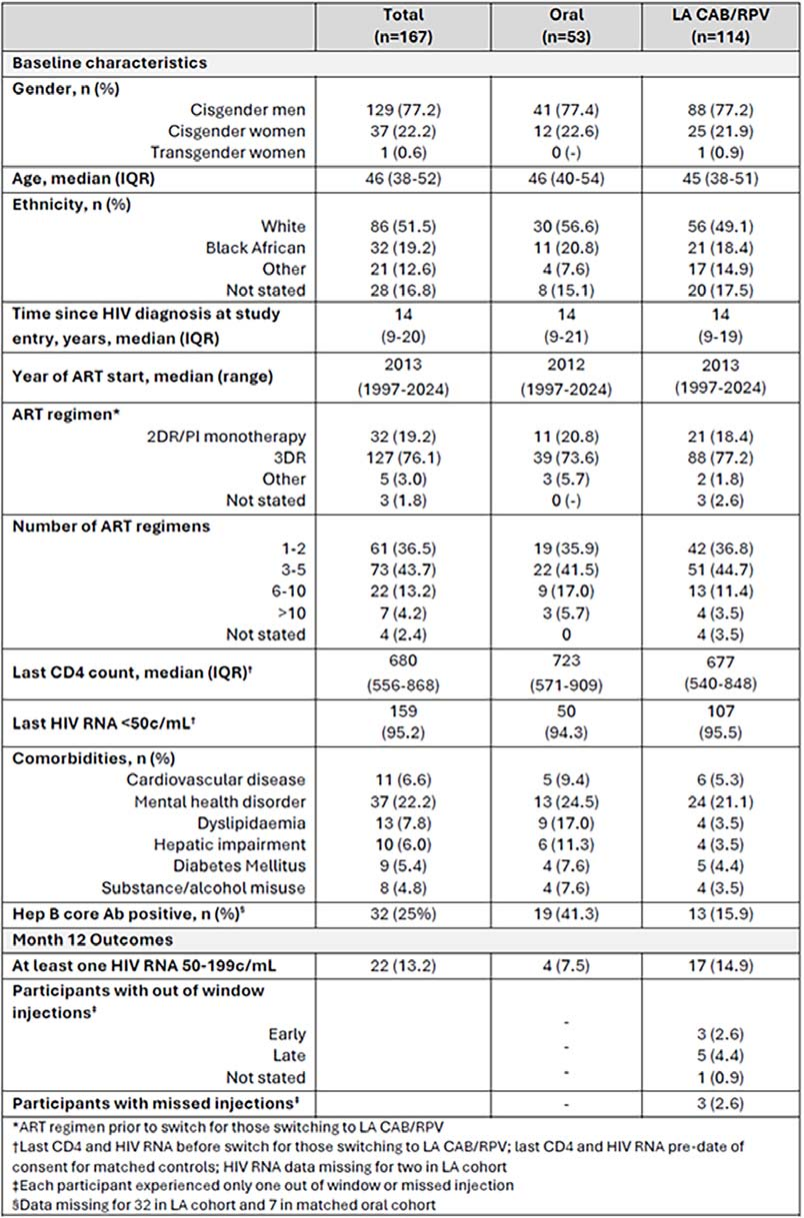
P121 | Baseline characteristics and month 12 outcomes.

### P122 HBV Surveillance After Switch to Long‐Acting Injectable Antiretroviral Therapy in People Living With HIV. Are We Monitoring Adequately?

Luísa Lopes, Gonçalo Silva, Inês Gonçalves, Filipe Medeiros, Hugo Graça, Joana Marinho, Cristina Valente

Infectious Diseases, Local Health Unit of Coimbra, Coimbra, Portugal

**Background**: HIV and hepatitis B virus (HBV) coinfection is associated with accelerated liver disease progression, cirrhosis, hepatocellular carcinoma and liver‐related mortality compared with HBV monoinfection. HBV reactivation, defined as detection of HBV DNA and/or HBsAg in individuals with previously undetectable markers, may lead to hepatic flare, acute hepatitis or liver failure. The increasing use of long‐acting injectable antiretroviral therapy raises concerns regarding HBV monitoring after withdrawal of HBV‐active agents.

**Methods**: A retrospective observational study was conducted at a tertiary care hospital, including people living with HIV with previous HBV exposure (anti‐HBc positivity) who switched from oral to long‐acting injectable antiretroviral therapy and were followed until May 2026. Demographic, clinical, biochemical and virological data, including sex, HBV serology, HBV DNA, AST, ALT and hepatic symptoms, were collected from electronic medical records. The primary outcome was HBV reactivation after treatment switch.

**Results**: A total of 163 patients were included, 97.0% (*n* = 158) male. Mean follow‐up was 4.6 years (maximum 7 years). Previous HBV exposure (anti‐HBc positivity) was identified in 17.2% (*n* = 28), of whom 26 (92.9%) were anti‐HBs positive. The median CD4+ T‐cell count was 548 cells/µL (IQR 489–887) and only two patients (7.1%) had CD4 counts between 200 and 349 cells/µL. All anti‐HBc‐positive patients underwent AST and ALT monitoring at least every 6 months after the switch, while HBsAg was monitored in 46.4% (*n* = 13). No clinical symptoms suggestive of HBV reactivation or AST/ALT elevations above the upper limit of normal were observed. However, post‐switch HBV DNA testing was not performed.

**Conclusions**: Monitoring practices were heterogeneous and did not fully align with current recommendations. Nevertheless, no clinical or biochemical evidence of HBV reactivation was identified during follow‐up among patients with previous HBV exposure receiving long‐acting injectable antiretroviral therapy. Although post‐switch HBV DNA testing was not performed, precluding exclusion of asymptomatic virological reactivation, the optimal HBV monitoring strategy and the true risk of reactivation in patients with good immune reconstitution and/or protective anti‐HBs levels remain to be established.

### P123 Immunological and Neuroinflammatory Profile of Long‐Acting Cabotegravir/Rilpivirine in People With HIV: A 24‐Month Study

Maria Antonella Zingaropoli^1,2^, Mariasilvia Guardiani^1^, Anna Carraro^1^, Eeva Tortellini^1^, Federica Dominelli^1^, Michele Antonacci^1^, Cosmo Del Borgo^3^, Raffaella Marocco^4^, Giulia Mancarella^1^, Lorenzo Ansaldo^4^, Ombretta Turriziani^5^, Maria Rosa Ciardi^1^, Claudio Maria Mastroianni^1^, Serena Vita^5^, Miriam Lichtner ^5^

^1^Department of Public Health and Infectious Diseases, Sapienza University of Rome, Rome, Italy; ^2^Microbiology and Virology Unit, University Hospital Policlinico Umberto I, Sapienza University of Rome, Rome, Italy; ^3^Infectious Diseases Unit, SM Goretti Hospital, Sapienza University of Rome, Rome, Italy; ^4^Infectious Diseases Unit, SM Goretti Hospital, Sapienza University of Rome, Latina, Italy; ^5^Department of Molecular Medicine, Sapienza University of Rome, Rome, Italy

**Background**: This single‐centre study involved an in‐depth longitudinal immunological evaluation of people with HIV (PWH) switching to long‐acting injectable cabotegravir plus rilpivirine (LA‐CAB/RPV). The aim was to comprehensively assess systemic immune activation and central nervous system injury biomarkers over a 2‐year follow‐up period.

**Methods**: Three time points were considered: before LA‐CAB/RPV (T0), after 12 (T1) and 24 months (T). Plasma HIV‐1 RNA and total cell‐associated HIV‐1 DNA levels were evaluated. Monocyte/macrophage subsets were analysed by flow cytometry. Plasma biomarkers of neuronal (NfL) and astrocyte (GFAP) damage, monocyte/macrophage activation (sCD163, sCD14) and BBB permeability (MMP‐9, TIMP‐1) were investigated using microfluidic next‐generation ELISA. Each time point was compared to healthy donors (HDs).

**Results**: Thirty‐eight ART‐experienced, aviraemic PWH and 32 HDs were enrolled. At T2, PWH higher classical, intermediate and non‐classical monocyte counts compared to HD. Lower pDC and slanDC counts were observed, alongside higher sCD163, sCD14, NfL, GFAP, MMP‐9 and TIMP‐1 plasma levels at both time points compared to HD (see Table 1). The longitudinal evaluation revealed no virological failure, nor any differences in the total cell‐associated HIV‐1 DNA levels. At T2, significant decreases were observed in intermediate and non‐classical monocyte counts, as well as in the sCD14, GFAP and TIMP‐1 plasma levels compared to T0 (see Table 1). Positive correlations were found between plasma levels of TIMP‐1 and intermediate monocyte count (ρ = 0.3437, *p* < 0.0001), GFAP (ρ = 0.3918, *p* < 0.0001), sCD163 (ρ = 0.3517, *p* < 0.0001) and sCD14 (ρ = 0.2397, *p* = 0.0016). No correlations were found between total cell‐associated HIV‐1 DNA and monocyte/macrophage subsets nor with CNS inflammation/damage blood biomarkers.

**Conclusions**: After starting LA‐CAB/RPV, an improvement of monocyte/macrophage activation markers and TIMP‐1 levels was observed, indicating a state of reduced systemic inflammation, likely due to the effective ART and viral suppression. The reduction in plasma GFAP levels suggests a potential reduction in CNS inflammation advocating the safety of LA‐CAB/RPV on CNS damage/inflammation, given also the absence of fluctuations in other inflammation‐linked or neuronal damage biomarkers. The observed correlations likely reflect causal pathways rather than epiphenomena: peripheral myeloid activation, orchestrated by TIMP‐1, drives systemic inflammation that fuels a neuroinflammatory environment, culminating in astrocytic and axonal injury. TIMP‐1 serves as a central hub, coordinating myeloid responses, tissue remodelling and CNS signalling.

**TABLE 1:**
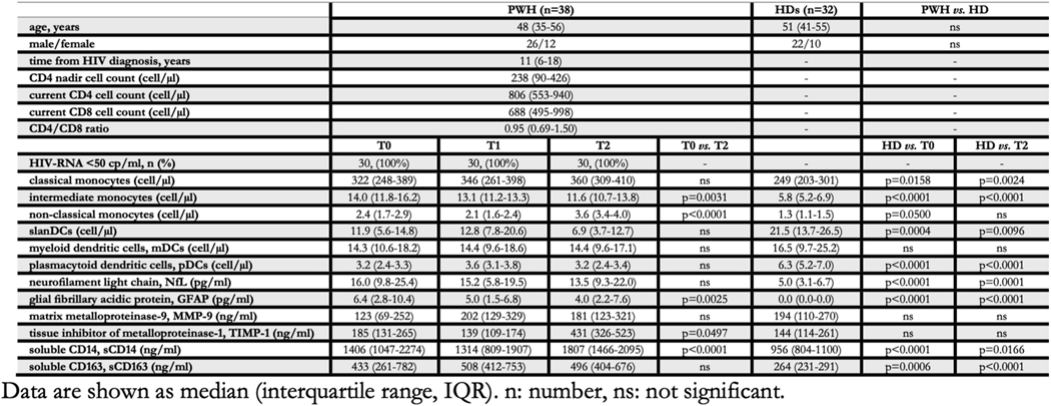
P123 | Study population.

### P124 Virological Failure Classification Under the CONSENSUS‐LAI Definition: Real‐World Data From the Multicentre LAHIV Cohort

Filippo Lagi^1^, Barbara Rossetti^2^, Sasha Trevisan^3^, Massimiliano Fabbiani^4^, Matteo Piccica^5^, Alessio Pampaloni^6^, Sara Modica^7^, Beatrice Menichini^8^, Silvia Costarelli^9^, Claudia Bianco^10^, Giovanni Sarteschi^11^, Michele De Gennaro^7^, Martina Turco^12^, Virginia Donini^3^, Martina Maruotto^3^, Paola Corsi^1^, Marco Pozzi^1^, Mario Tumbarello^4^, Daniela Messeri^8^, Cecilia Costa^5^, Beatrice Anna Adriani^6^, Cesira Nencioni^2^, Danilo Tacconi^10^, Spartaco Sani^9^, Antonella Vincenti^11^, Luigi Pisano^12^, Filippo Bartalesi ^5^, Alessandro Bartoloni^1^

^1^Infectious and Tropical Diseases Unit, Careggi University Hospital, Florence, Italy; ^2^Infectious Diseases Unit, Grosseto Hospital, USL Toscana Sud‐Est, Grosseto, Italy; ^3^Department of Experimental and Clinical Medicine, University of Florence, Florence, Italy; ^4^Infectious Diseases Unit, Siena University Hospital, University of Siena, Siena, Italy; ^5^Infectious Diseases Unit, Santa Maria Annunziata Hospital, USL Toscana Centro, Florence, Italy; ^6^Infectious Diseases Unit, Prato Hospital, USL Toscana Centro, Prato, Italy; ^7^Infectious Diseases Unit, Lucca Hospital, USL Toscana Nord‐Ovest, Lucca, Italy; ^8^Infectious Diseases Unit, Pistoia Hospital, USL Toscana Centro, Pistoia, Italy; ^9^Infectious Diseases Unit, Livorno Hospital, USL Toscana Nord‐Ovest, Livorno, Italy; ^10^Infectious Diseases Unit, Arezzo Hospital, USL Toscana Sud‐Est, Arezzo, Italy; ^11^Infectious Diseases Unit, Nuovo Ospedale Apuane, USL Toscana Nord‐Ovest, Massa, Italy; ^12^Section of Dermatology, Health Sciences Department, University of Florence, Florence, Italy

**Background**: Definitions of virological failure (VF) for long‐acting cabotegravir plus rilpivirine (CAB+RPV) remain inconsistent across real‐world cohorts. The CONSENSUS‐LAI Delphi process [1] established a shared definition: two consecutive HIV‐RNA ≥200 copies/mL 2–4 weeks apart, a single > 1000 copies/mL, emergent resistance or inability to suppress below 200 copies/mL. We evaluated its impact on VF classification and whether its September 2025 publication influenced prescriber behaviour.

**Methods**: Retrospective analysis of virologically suppressed adults initiating CAB+RPV across 11 centres in Tuscany, Italy (2022–2026). All physician‐initiated switches for virological reasons were reviewed and reclassified according to the CONSENSUS‐LAI definition. Sub‐threshold switches—defined as VF with HIV‐RNA between 50 and 200 copies/mL—were analysed separately. VF rates were calculated per 100 person‐years (100py). Switches before and after September 2025 were compared using Fisher's exact test.

**Results**: Among 297 participants contributing 418 person‐years, 11 switches were attributed to VF by treating physicians (2.63/100py, 95% CI: 1.31–4.71). Applying the CONSENSUS‐LAI definition retrospectively, only 6 met criteria (1.44/100py, 95% CI: 0.53–3.12): 5 with HIV‐RNA > 1000 copies/mL and 1 CSF viral escape with undetectable plasma HIV‐RNA, included conservatively given compartmental replication. Pre‐existing NNRTI resistance mutations unrecognized at baseline were identified in one of these cases only at VF. Resistance mutations emerged at failure in 2 of the 6. The remaining 5 were sub‐threshold switches (HIV‐RNA 50–200 copies/mL); none developed resistance mutations and all achieved viral re‐suppression below 50 copies/mL. Plasma re‐suppression was also achieved in 4 cases meeting CONSENSUS‐LAI criteria, with one outcome pending. Before September 2025, 1 of 6 virological switches (17%) was sub‐threshold; after publication, this rose to 4 of 5 (80%), though not statistically significant (*p* = 0.080), likely due to the small number of events (Figure 1).

**Conclusions**: Retrospective application of the CONSENSUS‐LAI definition nearly halves the estimated VF rate. Reassuringly, no resistance mutations emerged in sub‐threshold cases and re‐suppression was universally achieved. Despite international consensus publication, most post‐consensus virological switches remained sub‐threshold, suggesting limited real‐world penetration within the first year.

**Reference**

1. Orkin C, Paterson A, Elias A, et al. Establishing shared definitions of virological failure and discontinuation for long‐acting injectable cabotegravir and rilpivirine therapy (the CONSENSUS‐LAI Study): an international survey and Delphi process. Lancet HIV. 2025;12(9):e649–e659.

**FIGURE 1:**
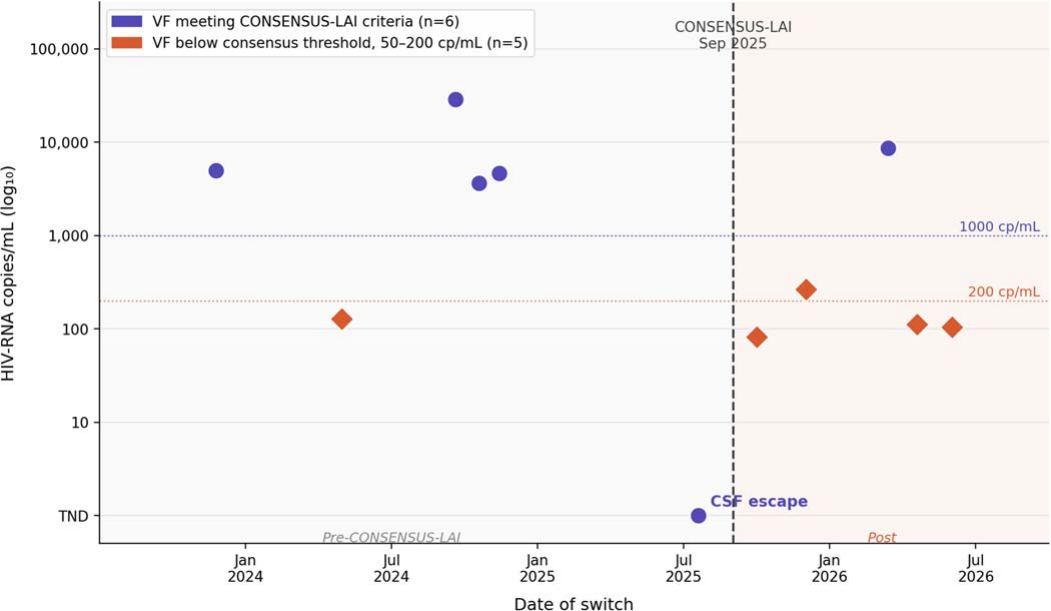
P124 | HIV‐RNA levels in plasma at the time of virological switch in 11 CAB+RPV‐treated patients. Circles (purple): cases meeting CONSENSUS‐LAI criteria; diamonds (orange): low‐level viraemia 50–200 copies/mL, below consensus threshold. Dashed vertical line: CONSENSUS‐LAI publication (September 2025). CSF escape case plotted at 1 copy/mL for graphical purposes.

### P125 Real‐World Durability and Discontinuation Patterns of Long‐Acting Cabotegravir/Rilpivirine in Tuscany: 3‐Year Data From the LAHIV Multicentre Cohort

Filippo Lagi^1^, Sasha Trevisan^2^, Massimiliano Fabbiani^3^, Barbara Rossetti^4^, Matteo Piccica^5^, Alessio Pampaloni^6^, Sara Modica^7^, Beatrice Menichini^8^, Silvia Costarelli^9^, Claudia Bianco^10^, Giovanni Sarteschi^11^, Michele De Gennaro^7^, Martina Turco^12^, Virginia Donini^2^, Maruotto Martina^2^, Paola Corsi^1^, Marco Pozzi^1^, Mario Tumbarello^3^, Daniela Messeri^8^, Cecilia Costa ^5^, Beatrice Anna Adriani^6^, Cesira Nencioni^4^, Danilo Tacconi^10^, Spartaco Sani^9^, Vincenti Antonella^11^, Luigi Pisano^12^, Filippo Bartalesi^5^, Alessandro Bartoloni^1^

^1^Infectious and Tropical Diseases Unit, Careggi University Hospital, Florence, Italy; ^2^Department of Experimental and Clinical Medicine, University of Florence, Florence, Italy; ^3^Infectious Diseases Unit, Siena University Hospital, University of Siena, Siena, Italy; ^4^Infectious Diseases Unit, Grosseto Hospital, USL Toscana Sud‐Est, Grosseto, Italy; ^5^Infectious Diseases Unit, Santa Maria Annunziata Hospital, USL Toscana Centro, Florence, Italy; ^6^Infectious Diseases Unit, Prato Hospital, USL Toscana Centro, Prato, Italy; ^7^Infectious Diseases Unit, Lucca Hospital, USL Toscana Nord‐Ovest, Lucca, Italy; ^8^Infectious Diseases Unit, Pistoia Hospital, USL Toscana Centro, Pistoia, Italy; ^9^Infectious Diseases Unit, Livorno Hospital, USL Toscana Nord‐Ovest, Livorno, Italy; ^10^Infectious Diseases Unit, Arezzo Hospital, USL Toscana Sud‐Est, Arezzo, Italy; ^11^Infectious Diseases Unit, Nuovo Ospedale Apuane, USL Toscana Nord‐Ovest, Massa, Italy; ^12^Section of Dermatology, Health Sciences Department, University of Florence, Florence, Italy

**Background**: Long‐acting cabotegravir plus rilpivirine (CAB+RPV) is the first intramuscular antiretroviral regimen approved for maintenance of HIV‐1 virological suppression. Real‐world data on treatment durability and discontinuation patterns across diverse clinical settings remain limited.

**Methods**: Multicentre observational study of virologically suppressed adults (HIV‐RNA <50 copies/mL) initiating CAB+RPV across 11 centres in Tuscany, Italy. Discontinuation (TD) was classified a posteriori as virological failure (VF), toxicity or patient‐ and physician‐initiated decision. VF required TD and two consecutive HIV‐RNA > 50 copies/mL or a single > 1000 copies/mL. Kaplan–Meier analyses estimated treatment persistence; Cox regression identified predictors of TD.

**Results**: We included 297 participants (median age 51.5 years, 77.8% male) contributing 418 person‐years (median follow‐up 1.4 years, IQR 0.6–2.2) (Table 1). Thirty‐six (12.1%) discontinued CAB+RPV. Treatment persistence was 91.0% at 1 year, 85.5% at 2 years and 78.3% at 3 years. Discontinuations were attributable to patient‐ and physician‐initiated decisions (*n* = 14; 4.7%), adverse events (*n* = 11; 3.7%) and VF (*n* = 11; 3.7%). VF presentations were heterogeneous, ranging from low‐level viraemia to high‐level rebound; INSTI and NNRTI resistance mutations emerged at failure in 2 cases and pre‐existing NNRTI mutations unrecognized at baseline were identified retrospectively in 1 case. All VFs occurred despite adherence to scheduled injections. Shorter prior virological suppression was independently associated with TD. Discontinuation risk declined significantly with calendar year of initiation: compared with 2022, hazard was lower in 2023 (HR 0.35, 95% CI: 0.14–0.88) and 2024 (HR 0.15, 95% CI: 0.05–0.51), driven largely by a reduction in adverse event‐related stops (Figure 1).

**Conclusions**: CAB+RPV demonstrated good real‐world durability across academic and non‐academic centres. The marked decline in discontinuation rates over calendar time suggests a learning‐curve effect during early implementation of long‐acting therapy.

**FIGURE 1:**
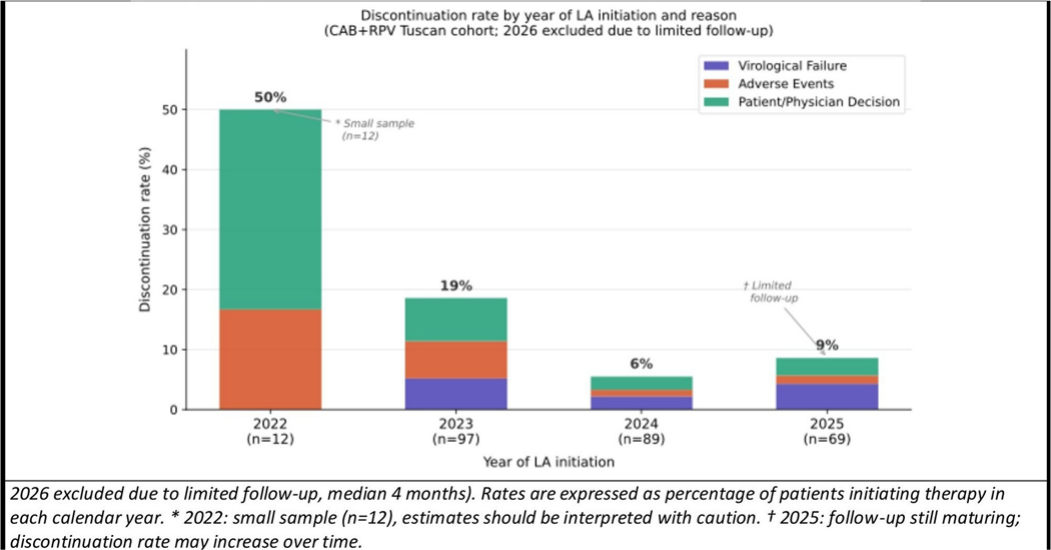
P125 | Discontinuation percentage by year of CAB+RPV initiation and reason, in virologically suppressed adults with HIV‐1 switching to long‐acting cabotegravir plus rilpivirine across 11 centres in Tuscany, Italy (2022–2025).

**TABLE 1:**
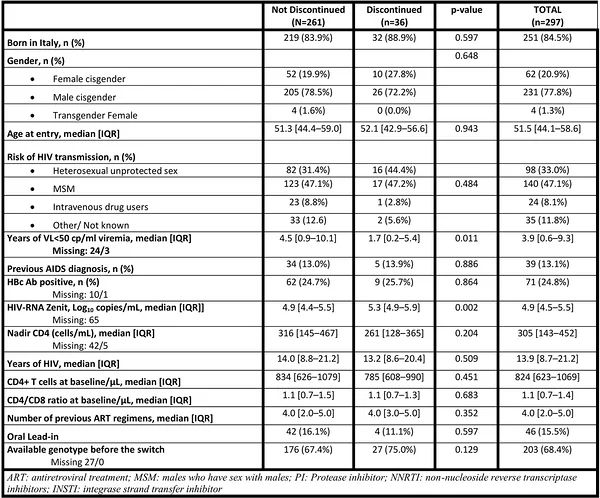
P125 | Clinical/demographic characteristics of adults with HIV‐1, ART experienced with HIV‐RNA level < 50 copies/mL at switch to RPV+CAB in Tuscany, Italy.

### P126 Two‐Year Metabolic and Immunological Profile of Long‐Acting Cabotegravir/Rilpivirine in Real‐World Practice: Data From the LAHIV Cohort

Filippo Lagi^1^, Martina Maruotto^2^, Barbara Rossetti^3^, Matteo Piccica^4^, Giovanni Sarteschi^5^, Alessio Pampaloni^6^, Beatrice Menichini^7^, Margherita Sambo^8^, Silvia Costarelli^9^, Claudia Bianco^10^, Michele De Gennaro^11^, Sara Modica^11^, Paola Corsi^1^, Marco Pozzi^1^, Martina Turco^12^, Mario Tumbarello^8^, Sasha Trevisan^2^, Cecilia Costa^4^, Beatrice Anna Adriani^6^, Cesira Nencioni^3^, Daniela Messeri^7^, Danilo Tacconi^10^, Spartaco Sani^9^, Antonella Vincenti^5^, Luigi Pisano^12^, Filippo Bartalesi ^4^, Alessandro Bartoloni^1^

^1^Infectious and Tropical Diseases Unit, Careggi University Hospital, Florence, Italy; ^2^Department of Experimental and Clinical Medicine, University of Florence, Florence, Italy; ^3^Infectious Diseases Unit, Grosseto Hospital, USL Toscana Sud‐Est, Grosseto, Italy; ^4^Infectious Diseases Unit, Santa Maria Annunziata Hospital, USL Toscana Centro, Florence, Italy; ^5^Infectious Diseases Unit, Nuovo Ospedale Apuane, USL Toscana Nord‐Ovest, Massa, Italy; ^6^Infectious Diseases Unit, Prato Hospital, USL Toscana Centro, Prato, Italy; ^7^Infectious Diseases Unit, Pistoia Hospital, USL Toscana Centro, Pistoia, Italy; ^8^Infectious Diseases Unit, Siena University Hospital, University of Siena, Siena, Italy; ^9^Infectious Diseases Unit, Livorno Hospital, USL Toscana Nord‐Ovest, Livorno, Italy; ^10^Infectious Diseases Unit, Arezzo Hospital, USL Toscana Sud‐Est, Arezzo, Italy; ^11^Infectious Diseases Unit, Lucca Hospital, USL Toscana Nord‐Ovest, Lucca, Italy; ^12^Section of Dermatology, Health Sciences Department, University of Florence, Florence, Italy

**Background**: Long‐acting cabotegravir plus rilpivirine (CAB+RPV), administered every 2 months, became the first available long‐acting antiretroviral regimen for maintenance of HIV‐1 virological suppression following approval in Italy in June 2022. Real‐world data on its longitudinal metabolic and immunological impact remain limited. This study assessed changes in creatinine, total cholesterol, triglycerides and CD4/CD8 parameters from baseline to weeks 44 and 96 in a multicentre Tuscan cohort.

**Methods**: Multicentre observational study across 11 HIV centres in Tuscany, including academic and non‐academic settings. Eligible participants were virologically suppressed adults (HIV‐RNA <50 copies/mL) initiating CAB+RPV with available laboratory measurements at baseline and at weeks 44 and/or 96. Within‐patient changes were assessed using Wilcoxon signed‐rank test. Dyslipidaemia was defined as total cholesterol > 200 mg/dL and/or triglycerides > 150 mg/dL.

**Results**: At week 44, 136 participants were included (79.4% male, median age 51 years); 89 had data at week 96 (Table 1). Total cholesterol, triglycerides and creatinine showed no clinically meaningful changes at either time point, despite a small but statistically significant creatinine reduction (Δ −0.08 mg/dL at W44, Δ −0.06 mg/dL at W96, *p* < 0.001). A proportional decline in both CD4 and CD8 counts was observed at week 96, with no significant change in the CD4/CD8 ratio overall (Δ +0.01, *p* = 0.325), probably consistent with reduced immune activation following the switch rather than immunological deterioration (Figure 1). Among participants with a CD4/CD8 ratio < 1 at baseline, a modest but significant improvement was observed at week 44 (*n* = 57, Δ +0.030, *p* = 0.015) and confirmed at week 96 (*n* = 34, Δ +0.032, *p* = 0.050), with no comparable change in those with ratio ≥1. Statin use was stable throughout follow‐up (23.5% at baseline, 27.0% at W96); cholesterol change did not differ between statin users and non‐users at either time point (*p* = 0.09 at W44, *p* = 0.55 at W96), arguing against a confounding effect of statin intensification.

**Conclusions**: Switching to CAB+RPV maintains a metabolically neutral profile over 2 years of follow‐up in routine clinical practice. The observed reduction in creatinine is of uncertain clinical significance. A consistent and significant improvement in CD4/CD8 ratio was detected in immunologically suboptimal patients across both time points, suggesting a potential immunological benefit of long‐acting therapy in this subgroup.

**FIGURE 1:**
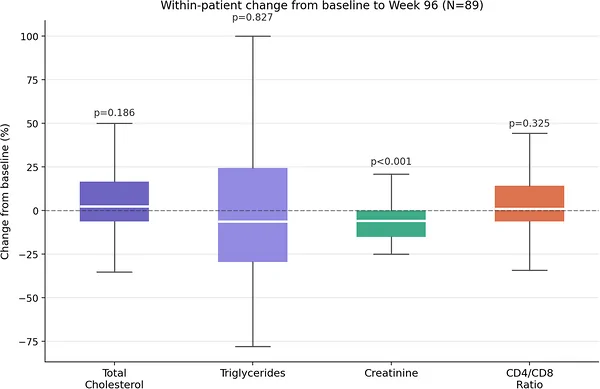
P126 | Longitudinal changes in creatinine, triglycerides, total cholesterol and CD4/CD8 ratio from baseline to weeks 44 and 96 in virologically suppressed adults with HIV‐1 switching to long‐acting cabotegravir plus rilpivirine across 11 centres in Tuscany, Italy.

**TABLE 1:**
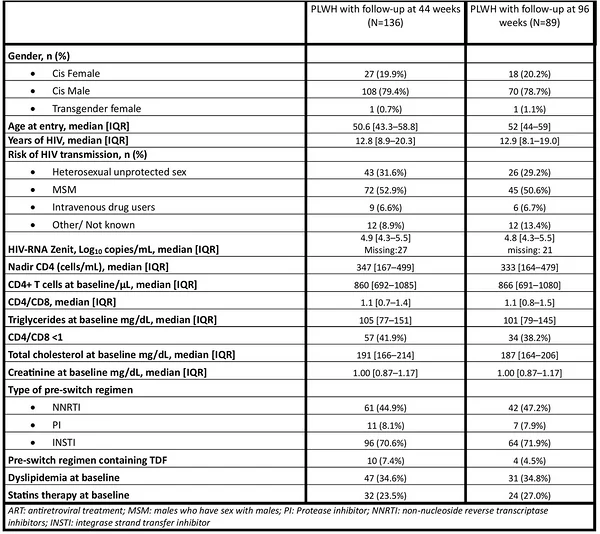
P126 | Clinical/demographic characteristics at baseline of adults with HIV‐1, switching to RPV+CAB, with laboratory tests available at the 44 and 96th week of follow‐up in 11 centres in Tuscany, Italy.

### P127 Examining the Healthcare Burden of Antiretroviral Therapy Resistance Among People With HIV in the US

Hansel Tookes^1^, Garrett Gremel^2^, Uche Mordi^2^, Mary Christoph^2^, Michelle L. D'Antoni^2^, Sara N. Levintow^2^, Eva Culakova^2^, Woodie Zachry ^2^, Joshua Cohen^3^

^1^Department of Medicine, University of Miami Liller School of Medicine, Miami, FL, US; ^2^Gilead Sciences, Inc., Foster City, CA, US; ^3^Independent Healthcare Analyst (Former Research Associate Professor, Tufts University), Boston, MA, US

**Background**: Resistance reduces antiretroviral therapy (ART) effectiveness and remains a barrier to ending the HIV epidemic. This study aimed to examine the clinical outcomes and healthcare resource utilization (HCRU) associated with antiretroviral resistance in the US.

**Methods**: This retrospective, observational analysis used medical and pharmacy claims from HealthVerity's database to identify treatment‐naïve or treatment‐experienced people with HIV (PWH) with resistance (≥1 primary resistance‐associated mutation [RAM] documented during 2023) who were matched 1:2 to PWH without suspected resistance or evidence of resistance testing (non‐RAM; proxy for no resistance). Matching criteria were age, sex, payer type, treatment history and Charlson Comorbidity Index (CCI). Index date was the earliest detection of primary RAM. The non‐RAM cohort inherited the index date of their matched counterpart. Included individuals were required to have at least 12 months of continuous enrolment pre‐ and post‐index. Baseline characteristics included adherence (proportion of days covered [PDC] by a guideline‐recommended ART) in the 12 months prior to index and laboratory values (measured within 90 days of index). Opportunistic infections (OIs) and HIV‐related HCRU were evaluated over the 12‐month follow‐up period, controlling for baseline matched characteristics. Differences between primary and non‐RAM groups were tested using generalized estimating equation method.

**Results**: Of those meeting inclusion criteria, 1330 had a primary RAM and 2660 were matched to the non‐RAM cohort (Table 1). For treatment‐experienced PWH, baseline adherence was lower in the RAM cohort (mean PDC 0.64 vs. 0.75, *p* < 0.05). Among those with available data, mean CD4 counts were higher in non‐RAM versus RAM cohort (*p* < 0.05) noting limited and imbalanced availability of lab values across cohorts. Proportion with an undetectable viral load (<200 copies/mL) was higher among non‐RAM versus RAM cohort (*p* < 0.05). Post‐index, OIs were higher in RAM versus non‐RAM cohort (5.3% vs. 2.5%; absolute difference 2.8%, *p* < 0.05). HIV‐related inpatient (19.4% vs. 14.2%), emergency department (8.3% vs. 5.7%) and ambulatory visits (84.2% vs. 78.5%) during follow‐up were higher in RAM versus non‐RAM cohort (*p* < 0.05 for all).

**Conclusions**: Findings suggest that ART resistance is associated with poorer clinical outcomes and greater healthcare system utilization.

**TABLE 1:**
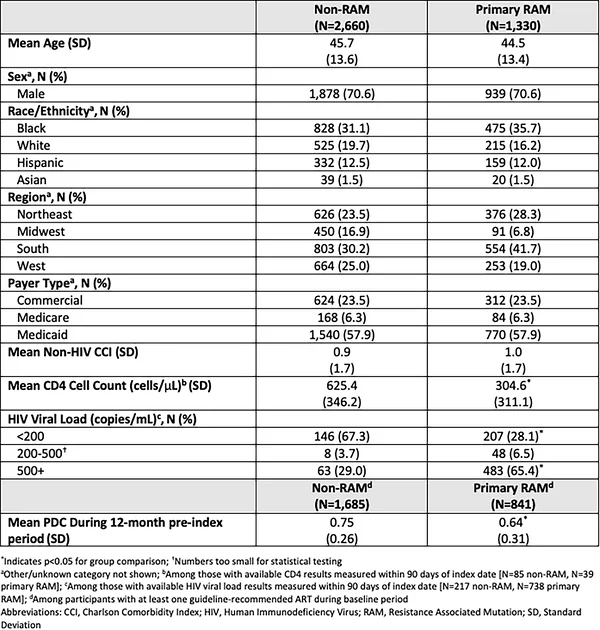
P127 | Baseline demographics and clinical characteristics.

### P128 CD27 and PD‐1 Expression Patterns and Ratio‐Based Assessment of Immune Exhaustion in People Living With HIV

Veysel Akca ^1,2^, Burak Ekrem Çitil^3^, Turhan Togan^2^

^1^Department of Infectious Diseases and Clinical Microbiology, Sandıklı, Türkiye; ^2^Department of Infectious Diseases and Clinical Microbiology, Muğla, Türkiye; ^3^Department of Medical Microbiology, Muğla, Türkiye

**Background**: Persistent immune activation and T‐cell exhaustion may continue in people living with HIV (PLWH) despite antiretroviral therapy (ART). We evaluated CD27 and PD‐1 expression and ratio‐based indices representing the balance between T‐cell differentiation and exhaustion.

**Methods**: This prospective, single‐centre study included 199 PLWH categorized as BIC/FTC/TAF (*n* = 57), DTG/TDF/FTC (*n* = 51), DTG/3TC (*n* = 39), viraemic on ART (*n* = 22) and treatment‐naïve (*n* = 30). Demographic and HIV‐related characteristics are summarized in Table 1. CD27 and PD‐1 expression on CD4^+^ and CD8^+^ T cells was assessed by flow cytometry. CD4^+^CD27^+^/CD4^+^PD‐1^+^ and CD8^+^CD27^+^/CD8^+^PD‐1^+^ ratios were calculated.

**Results**: Clinical characteristics were generally comparable across groups, although ART‐related treatment intervals differed significantly (Table 1A). Immunological parameters differed across groups, including CD4^+^ T‐cell count (*p* < 0.001), CD8^+^ T‐cell count (*p* = 0.041), CD4^+^PD‐1^+^ (*p* = 0.022), CD8^+^CD27^+^ (*p* = 0.004) and CD8^+^PD‐1^+^ percentages (*p* = 0.001) (Table 1B). Both CD4^+^CD27^+^/CD4^+^PD‐1^+^ (*p* = 0.019) and CD8^+^CD27^+^/CD8^+^PD‐1^+^ ratios (*p* = 0.010) differed significantly among groups, with the highest values in the DTG/3TC group and lowest values in treatment‐naïve participants (Table 1B). Both ratios correlated inversely with age, diagnostic delay and HIV RNA at diagnosis and positively with baseline CD4/CD8 ratio (Table 1C). Their associations with HIV RNA and baseline CD4/CD8 ratio are illustrated in Figure 1.

**Conclusions**: Higher CD27/PD‐1 ratios were associated with more favourable immunological profiles and preserved immune balance. These ratio‐based indices may provide practical markers of immune restoration and chronic immune exhaustion in PLWH and warrant further validation in prospective studies.

**FIGURE 1:**
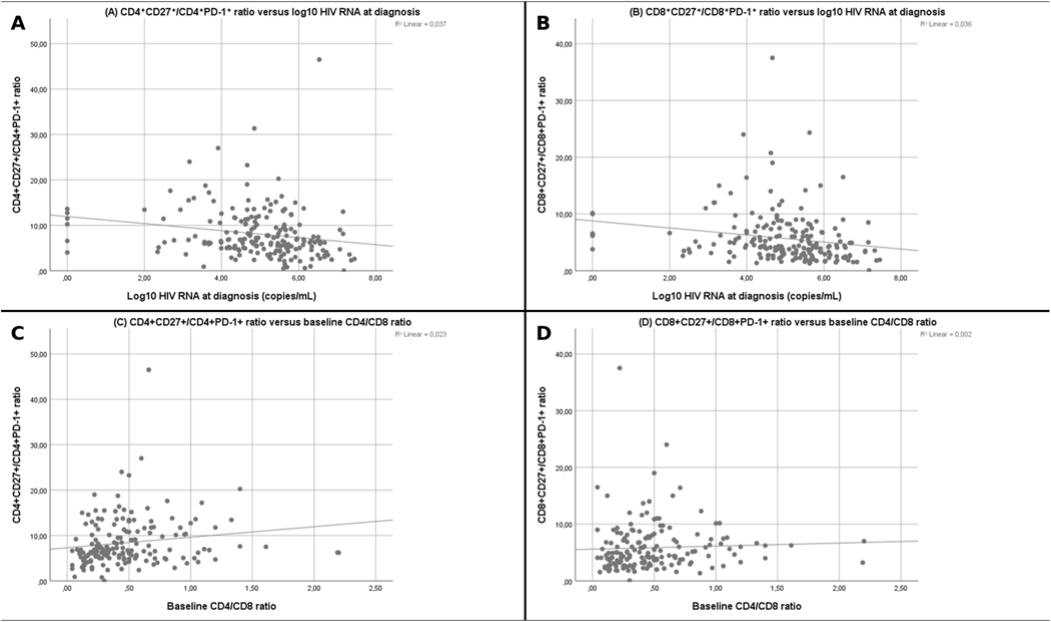
P128 | Correlations between CD27/PD‐1 ratios and selected virological and immunological parameters. Scatter plots showing the relationships between the CD4^+^CD27^+^/CD4^+^PD‐1^+^ and CD8^+^CD27^+^/CD8^+^PD‐1^+^ ratios and selected virological and immunological parameters. (A) CD4^+^CD27^+^/CD4^+^PD‐1^+^ ratio versus log_10_ HIV RNA at diagnosis. (B) CD8^+^CD27^+^/CD8^+^PD‐1^+^ ratio versus log_10_ HIV RNA at diagnosis. (C) CD4^+^CD27^+^/CD4^+^PD‐1^+^ ratio versus baseline CD4/CD8 ratio. (D) CD8^+^CD27^+^/CD8^+^PD‐1^+^ ratio versus baseline CD4/CD8 ratio.

**TABLE 1:**
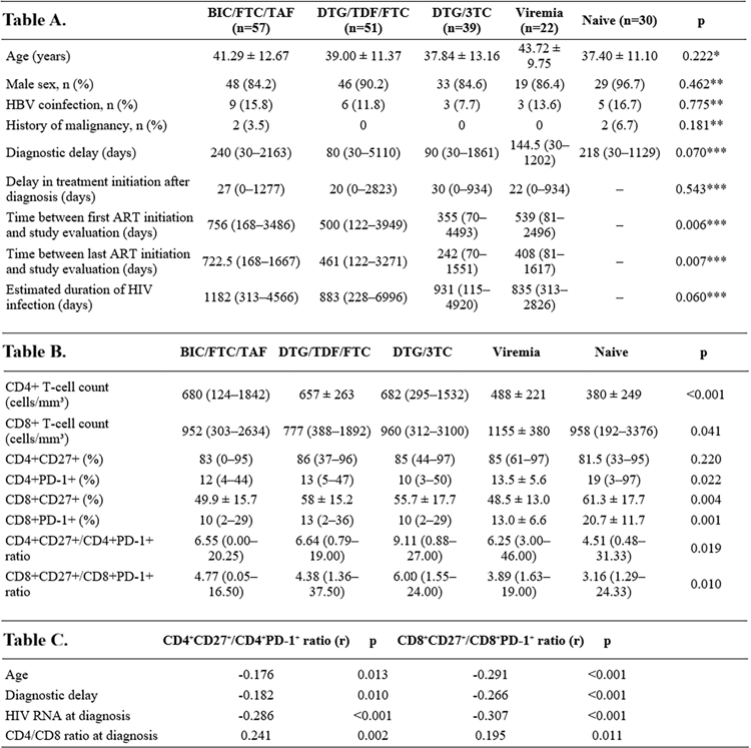
P128 | (A) Demographic, clinical characteristics and HIV‐related time intervals. (B) Distribution of immunological parameters and CD27/PD‐1 ratios. (C) Correlation analysis of CD4^+^CD27^+^/CD4^+^PD‐1^+^ and CD8^+^CD27^+^/CD8^+^PD‐1^+^ ratios with selected clinical and immunological parameters. (A, B) Data are presented as mean ± standard deviation (SD), median (minimum–maximum) or *n* (%), as appropriate. Comparisons were performed using one‐way analysis of variance (ANOVA), Pearson's chi‐square test or the Kruskal–Wallis test, as appropriate. (C) Data are presented as Spearman correlation coefficients (*r*) and corresponding *p* values. Positive coefficients indicate direct correlations, whereas negative coefficients indicate inverse correlations.

### P129 Long‐Acting Injectable Cabotegravir/Rilpivirine in Migrant People Living With HIV: Uptake, Barriers and Social Vulnerability as a Driver of Adoption

Lara Neto, Cristina Soeiro, Clara Batista, Eduarda Pena, Maria João Gonçalves, Sofia Jordão, Ricardo Correia de Abreu, Frederico Duarte

Infectious Diseases Department, Local Health Unit of Matosinhos, Matosinhos, Portugal

**Background**: Long‐acting injectable cabotegravir/rilpivirine (CAB/RPV LA) holds particular promise for migrant people living with HIV (MLWH), a population in whom adherence to daily oral antiretroviral therapy (ART) can be undermined by social instability, precarious living conditions and stigma. Data on uptake and barriers in this population are scarce. We report on CAB/RPV LA uptake, recipient profiles and real‐world barriers in a cohort of MLWH followed in Portugal.

**Methods**: We performed a cross‐sectional analysis of patients receiving CAB/RPV LA within a retrospective cohort of 199 MLWH (October 2020–March 2026). Social vulnerability indicators and reasons for non‐uptake were systematically recorded.

**Results**: 44/199 patients (22.1%) were receiving CAB/RPV LA, a notably high adoption rate for a migrant cohort. Recipients were predominantly male (79.5%), with a median age of 32 years, largely of Brazilian nationality (72.7%). A further 18 patients (9%) expressed interest in initiating CAB/RPV LA. Social vulnerability indicators were prevalent: drug use 6.8%, psychiatric comorbidity 4.5% and prior sexually transmitted infections (STIs) 34.1%. Barriers to CAB/RPV LA were identified in 14 patients: no reason explicitly stated (*n* = 6, 42.9%), logistical and scheduling constraints (*n* = 4, 28.6%), preference for oral medication (*n* = 3, 21.4%) and stigma related to clinic attendance for injections (*n* = 1, 7.1%). Genotypic resistance testing (GRT) was available in only 14/44 (31.8%) patients on CAB/RPV LA. However, all patients without GRT had fully documented prior ART histories based on high‐barrier integrase inhibitors (76%) or efavirenz‐based regimens (24.0%), which provided sufficient clinical reassurance of viral susceptibility. No virological failure was recorded.

**Conclusions**: A CAB/RPV LA uptake of 22.1% in a socially vulnerable migrant population challenges assumptions about feasibility in this group. Stigma related to clinic attendance emerged as a discrete actionable barrier. When formal GRT is unavailable, a well‐documented treatment history combined with sustained virological suppression is sufficient to support CAB/RPV LA eligibility.

### P130 Five‐Year Persistence and Reasons for Discontinuation of Dolutegravir/Lamivudine in a Multicentre Real‐World Cohort of People Living With HIV

Emma Vallribera Olmos ^1^, Marta Escura Esteban^1^, Maria del Carmen Navarro Sáez^2^, Sonia Calzado Isbert^2^, Marc Pedrosa Aragon^2^, Lizza Maria Macorigh Mantilla^3^, Elisabet Deig Comerma^3^, Julia Gutierrez Sanjuan^4^, Rodrigo Alonso Navarro^4^, Oscar Del Rio Perez^5^, Silvia Valero Rovira^5^, Marta Navarro Vilasaró^2^

^1^Parc Taulí Hospital Universitari, Institut d'Investigació i Innovació Parc Taulí (I3PT‐CERCA), Universitat Autònoma de Barcelona, Internal Medicine, Sabadell, Spain; ^2^Parc Taulí Hospital Universitari, Institut d'Investigació i Innovació Parc Taulí (I3PT‐CERCA), Universitat Autònoma de Barcelona, Infectious diseases, Sabadell, Spain; ^3^Hospital General de Granollers, Internal Medicine, Granollers, Spain; ^4^Consorci Sanitari del Maresme, Hospital Universitari de Mataró, Internal Medicine, Mataró, Spain; ^5^Hospital Comarcal Sant Jaume de Calella, Internal Medicine, Calella, Spain

**Background**: Dolutegravir/lamivudine (DTG/3TC; Dovato) is a two‐drug regimen for people living with HIV (PLWH). Although efficacy has been demonstrated in randomized trials, long‐term real‐world data beyond 3 years remain limited. We evaluated treatment persistence, discontinuation and virological outcomes over 5 years following DTG/3TC implementation in routine clinical practice in Spain.

**Methods**: A retrospective multicentre cohort study including adult PLWH from four hospitals in Catalonia, Spain (Hospital Parc Taulí, Hospital Calella, Hospital Granollers, Hospital Mataró), who initiated DTG/3TC from its introduction into clinical practice until March 2026. Treatment‐naïve and treatment‐experienced individuals were included. Data were collected from electronic medical records using REDCap. The primary endpoint was treatment persistence; secondary endpoints included reasons for discontinuation, virological suppression, blips and confirmed virological failure (VF).

**Results**: A total of 608 PLWH were included: 10 (16.9%) treatment‐naïve and 505 (83.1%) treatment‐experienced. Median follow‐up was 43 months. At data cut‐off, 447/595 (75.1%) remained on DTG/3TC; 95 had discontinued (16%), 8 (1.3%) had died and 45 (7.6%) were lost to follow‐up or transferred (Table 1). Discontinuation rates were significantly higher in treatment‐naïve than treatment‐experienced individuals (24.3% vs. 15.4%; *p* < 0.05). The main reason for discontinuation was a strategic switch to long‐acting cabotegravir/rilpivirine (57/112, 50.9%), followed by toxicity/tolerability, patient preference and virological concern/blips (Figure 1). Virological suppression was maintained in 491/591 (83%) of PLWH; transient blips occurred in 50 (8.4%). Baseline resistance data were available for 377/608 patients (62.0%). Among these, 122 (32.4%) harboured resistance‐associated mutations, mainly NNRTI mutations and protease polymorphisms. Mutations potentially impacting DTG/3TC activity were rare and were not associated with VF. Overall, three patients experienced VF during follow‐up (0.6%), without documented baseline resistance mutations; two subsequently developed emergent resistance mutations while receiving treatment (having previous programmed therapeutic stops).

**Conclusions**: DTG/3TC demonstrated high long‐term persistence and excellent virological effectiveness. Confirmed VF was exceptionally rare and most discontinuations reflected treatment strategy change (transitions to long‐acting) rather than loss of efficacy or tolerability. Baseline resistance mutations potentially relevant to DTG/3TC were uncommon and were not associated with VF. It is noteworthy that VF occurred in patients without documented baseline resistance mutations and with programmed therapeutic stops in the past.

**FIGURE 1:**
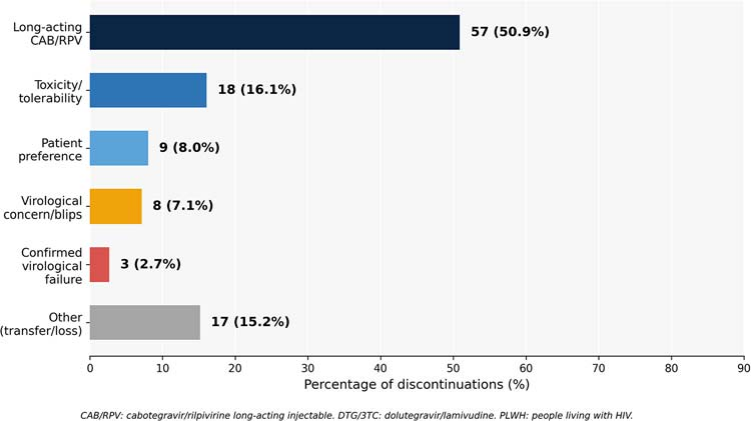
P130 | Reason for DTG/3TC discontinuation (*n* = 112 PLWH, median follow‐up 43 months).

**TABLE 1:**
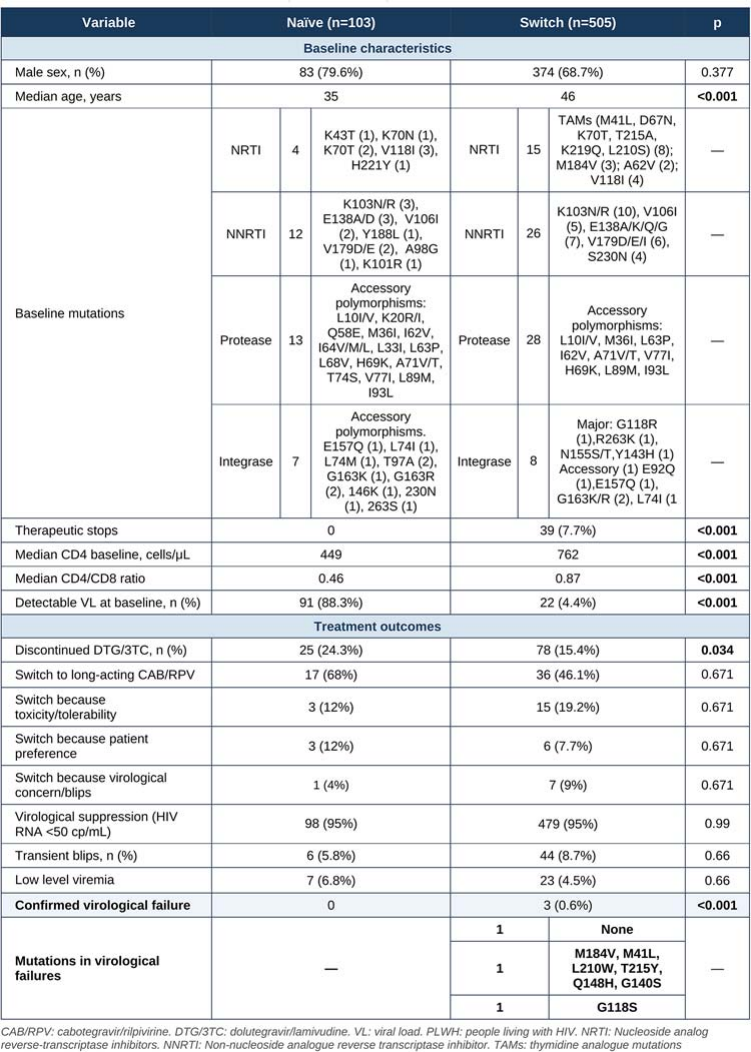
P130 | Key outcomes by treatment status.

### P131 Residual Viraemia in Adults Receiving Contemporary Antiretroviral Therapy: A Real‐World Brazilian Cohort

Pietra Stanicki, Karen Ingrid Tasca, Alexandre Naime Barbosa

UNESP – Faculdade de Medicina de Botucatu, Departamento de Infectologia, Botucatu, Brazil

**Background**: As people living with HIV age, long‐term exposure to antiretroviral therapy (ART) and treatment optimization have become central challenges in routine care. Residual viraemia is increasingly detected in individuals receiving contemporary ART; however, its epidemiology and clinical significance remain incompletely understood, particularly in middle‐income settings with universal treatment access.

**Methods**: This observational cohort study included 1018 adults living with HIV receiving ART at a public reference service in Brazil between January 2020 and July 2022. Participants were stratified by age (18–49 vs. ≥50 years). Virological outcomes were categorized as undetectable viral load (<40 copies/mL), residual viraemia (40–500 copies/mL) and virological failure (> 500 copies/mL). Demographic characteristics, CD4 T‐cell counts, ART duration and treatment regimens were analysed. Multivariable logistic regression identified predictors of sustained viral suppression.

**Results**: Among participants, 442 (43.4%) were aged ≥50 years. Older adults more frequently had ART duration ≥11 years (47.1% vs. 39.2%; *p* = 0.010), fewer CD4 T‐cell counts < 350 cells/mm^3^ (7.0% vs. 13.3%; *p* = 0.0012), higher rates of viral suppression (89.8% vs. 83.3%; *p* < 0.0001) and lower rates of virological failure (2.5% vs. 10.1%; *p* < 0.0001). Residual viraemia was observed in approximately 7% of participants and did not differ between age groups. Overall, 41.2% received TDF/3TC/DTG, while 32.4% received dolutegravir/lamivudine, which was prescribed more frequently among older adults (37.8% vs. 28.3%; *p* = 0.003). In multivariable analysis, ART duration ≥11 years (OR 1.57; 95% CI: 1.02–2.42) and receipt of dolutegravir/lamivudine were independently associated with sustained viral suppression, whereas age and sex were not.

**Conclusions**: Residual viraemia affected approximately 1 in 14 treated individuals and occurred at similar frequencies across age groups. Older adults demonstrated higher rates of viral suppression despite comparable rates of residual viraemia. Longer ART duration and receipt of dolutegravir/lamivudine were independently associated with sustained viral suppression. These findings support the effectiveness of contemporary dolutegravir‐based ART in routine clinical practice while highlighting the importance of continued monitoring of residual viraemia during long‐term treatment.

### P132 Towards Elimination of HIV, Syphilis and HBV Mother‐to‐Child Transmission in the Gambia and Burkina Faso: The Triple Elimination Model of Mother‐to‐Child Transmission (TRI‐MOM) Programme

Abdoulie E. Jallow

MRCG@LSHTM, Fajara, Banjul, Gambia

**Background**: West Africa faces significant challenges with mother‐to‐child transmission (MTCT) of HIV, syphilis and hepatitis B virus (HBV) infections. Despite this burden, MTCT of these infections can be effectively prevented with straightforward interventions. The World Health Organization (WHO) has advocated for a “triple elimination” model, integrating the prevention of these infections into maternal care services to accelerate their control and elimination. The TRI‐MOM project aims to evaluate the feasibility of integrating the triple elimination model in two West African countries.

**Methods**: At four urban and rural health facilities, healthcare workers received training and pregnant women were routinely sensitized about integrated screening. All women attending their first antenatal visit were offered rapid tests for HIV, syphilis and HBV. HIV and syphilis infections were managed through a test‐and‐treat approach. HBV‐positive women with viral loads > 200,000 IU/mL received tenofovir (TDF) prophylaxis at three sites equipped with GeneXpert; one site implemented TDF treatment without viral load assessment. The primary endpoint was uptake of TDF prophylaxis among eligible HBV‐positive pregnant women.

**Results**: Between January and March 2024, HCWs in government facilities were trained. Simplified screening and treatment/prophylaxis SOPs were developed and adapted per health facility. Between 18 March 2024 and 23 October 2025, 13,025 (99.5%) pregnant women were screened for the 3 infections. Among screened women, prevalence was 434 (67.4%) for HBV, 164 (25.5%) for HIV, 29 (4.5%) for syphilis and 12 (1.9%) for HIV‐HBV co‐infection and HIV–syphilis. At GeneXpert‐equipped sites, HBV viral load testing was conducted for 280 HBsAg‐positive women; 14 (4.9%) had viral loads > 200,000 IU/mL. Treatment initiation rates were high: HBV (68/70; 8 by viral load), HIV (94/98) and syphilis (19/29). Complete adherence occurred in 56/68 HBV‐treated and 107/164 HIV‐treated women; 19/29 syphilis‐positive women completed the full penicillin regimen. The HBV vaccine was given within 24 h to 276/376 (72.6%) infants. No maternal or infant deaths occurred in phase 1. Phase 2 enrolled 360/389 mother−infant pairs; one HBV MTCT occurred. 3 HIV transmission was observed among 71 infants.

**Conclusions**: Simplified integrated triple screening and treatment is feasible, informing strategies towards triple MTCT elimination in West Africa. Ongoing follow‐up will further assess effectiveness.

### P133 Bictegravir/Emtricitabine/Tenofovir Alafenamide in Treatment‐Naïve People With HIV: 48‐Week Real‐World Outcomes Including Very High Baseline Viraemia

Aysin Kilinc Toker, Hande Saglam Eslik, Merve Sultan Akpinar, Zehra Gul Koca, Aysenur Kefkir, Ahmed Hatip

Department of Infectious Diseases and Clinical Microbiology, Kayseri City Training and Research Hospital, Kayseri, Türkiye

**Background**: Bictegravir/emtricitabine/tenofovir alafenamide (BIC/FTC/TAF) is widely used as initial antiretroviral therapy in treatment‐naïve people with HIV. Although randomized trials have established its efficacy, real‐world data remain important in heterogeneous populations, particularly individuals with very high baseline viraemia or low CD4+ T‐cell counts. We evaluated 48‐week virological and immunological outcomes of BIC/FTC/TAF in routine clinical practice.

**Methods**: This retrospective, single‐centre, real‐world cohort study was conducted at Kayseri City Hospital, a regional tertiary care centre. Treatment‐naïve adults with HIV initiating BIC/FTC/TAF were included. Baseline characteristics and HIV RNA results at weeks 4, 24 and 48 were evaluated. Very high baseline viraemia was defined as HIV RNA > 1,000,000 copies/mL. Non‐normally distributed repeated measurements were analysed using the Friedman test, followed by paired Wilcoxon signed‐rank tests.

**Results**: Overall, 210 treatment‐naïve people with HIV were included; median age was 37 years (IQR 30–49; range 18–75) and 185 (88.1%) were male. Baseline median CD4+ T‐cell count was 363.1 cells/mm^3^ (IQR 237.6–526.8), CD4/CD8 ratio 0.35 (IQR 0.22–0.58) and HIV RNA 300,018 copies/mL (IQR 44,076.5–1,141,345.8). Sixty patients (28.6%) had HIV RNA > 1,000,000 copies/mL. Week 48 HIV RNA data were available for 194/210 (92.4%). HIV RNA <50 copies/mL was achieved in 100/196 (51.0%), 145/197 (73.6%) and 182/194 (93.8%) at weeks 4, 24 and 48, respectively; corresponding rates for HIV RNA < 200 copies/mL were 128/196 (65.3%), 164/197 (83.2%) and 185/194 (95.4%). Among patients with baseline HIV RNA > 1,000,000 copies/mL, 44/56 (78.6%) achieved HIV RNA <50 copies/mL and 47/56 (83.9%) achieved < 200 copies/mL at week 48 (Table 1). The median CD4+ T‐cell count increased from 355.2 to 657.6 cells/mm^3^ and the median CD4/CD8 ratio from 0.35 to 0.79 by week 48; both changes were significant over time (Figure 1). No treatment discontinuation or regimen switch due to documented adverse events, tolerability issues or other clinical reasons was observed.

**Conclusions**: In this regional single‐centre cohort, BIC/FTC/TAF was associated with high virological suppression, immunological recovery and maintained treatment persistence through week 48. Although suppression was slower with very high baseline viraemia, most patients achieved virological response by week 48.

**FIGURE 1:**
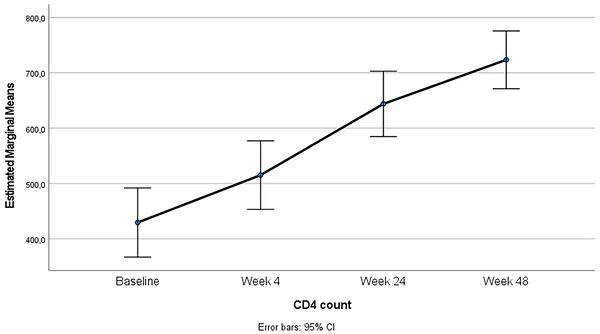
P133 | CD4+ T‐cell counts from baseline to week 48.

**TABLE 1:**
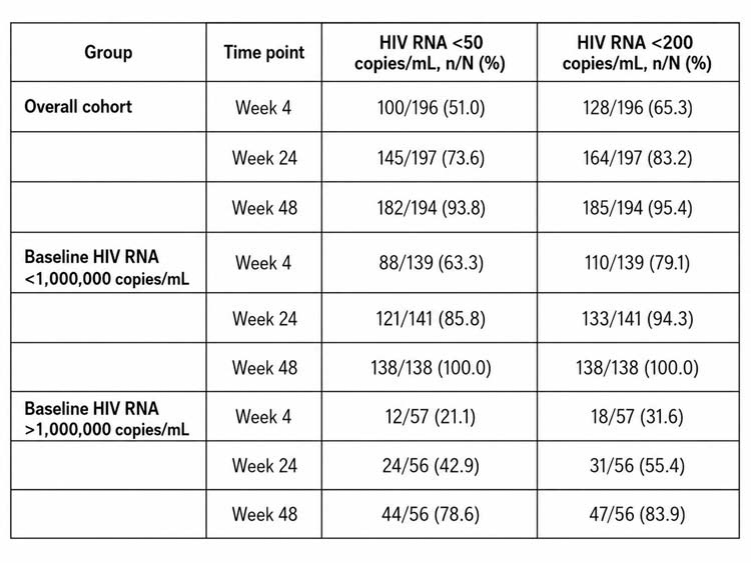
P133 | Virological outcomes by baseline HIV RNA category.

### P134 Effectiveness of DTG/3TC as Simplification Strategy in a Multicentric Virologically Suppressed Cohort of People With HIV With a Current CD4 Count Below 350 cells/mmc

Gianmaria Baldin ^1^, Francesca Lombardi^1^, Adriana Cervo^2^, Alberto Borghetti^3^, Massimiliano Fabbiani^4^, Arturo Ciccullo^5^, Filippo Lagi^6^, Letizia Oreni^7^, Francesco Bassani^8^, Stefano Rusconi^8^, Andrea Gori^7^, Cristina Mussini^2^, Simona Di Giambenedetto^1,9^, Andrea Giacomelli^7^

^1^Fondazione Policlinico Universitario A. Gemelli, Roma, Italy; ^2^Department of Infectious Diseases, Azienda Ospedaliero‐Universitaria of Modena, Modena, Italy; ^3^Infectious Diseases Unit, Department of Clinical and Experimental Medicine, Azienda Ospedaliera Universitaria Pisana, Pisa, Italy; ^4^Department of Medical Sciences, Infectious and Tropical Diseases Unit, University Hospital of Siena, Siena, Italy; ^5^Infectious Diseases Unit, Ospedale San Salvatore, L'Aquila, Italy; ^6^Department of Experimental and Clinical Medicine, University of Florence, Firenze, Italy; ^7^Department of Infectious Diseases, ASST Fatebenefratelli Sacco University Hospital, Milano, Italy; ^8^SC Malattie Infettive, ASST Ovest Milanese, Ospedale di Legnano (MI), Legnano, Italy; ^9^Università Cattolica del Sacro Cuore, Roma, Italy

**Background**: Dolutegravir/lamivudine (DTG/3TC) is widely used as simplification strategy, although few evidence exist for people with HIV (PWH) with low CD4 counts. This study aimed to assess DTG/3TC effectiveness in PWH in virological suppression (VS) and with CD4< 350 cells/mmc at switch, compared with triple regimens (3DR).

**Methods**: This was a multicentric cohort study including PWH in VS switching from any triple regimen to DTG/3TC or to a DTG or BIC‐based 3DR between 2015 and 2025 and with current CD4< 350 cells/mmc at switch. Virological failure (VF) was defined as 2 consecutive HIV‐RNA > 50 cps/mL or a single HIV‐RNA > 1000 cps/mL. Probability of maintaining versus was estimated by Kaplan–Meier curves. A propensity score (PS) for switching to DTG/3TC was estimated by including baseline factors that were unevenly distributed between treatment‐groups (at a *p*‐value < 0.05), into a logistic regression model. A Cox proportional hazards model, adjusted for PS, was then fitted to estimate the association between treatment strategy and VF. A sub‐analysis included PWH switching with CD4< 200 cells/mmc.

**Results**: A total of 282 PWH were included: 115 in DTG/3TC and 167 in 3DR. Baseline characteristics and difference between groups are presented in Table 1. Eleven VF occurred: 4 in DTG/3TC and 7 in 3DR. The estimated probabilities of maintaining VS at 144 weeks were 94.5% (95% CI: 85.6–97.9) for DTG/3TC and 94.7% (95% CI: 89.0–97.4) for 3DR (*p* = 0.992) (Figure 1). In the PS‐adjusted Cox model (HIV‐RNA zenith, time on ART, prior ART, prior VF), switching to DTG/3TC was not associated with VF compared with 3DR (aHR 0.84, 95% CI: 0.20–3.58; *p* = 0.817). Both groups showed significant CD4 gains from baseline to 144 weeks: in DTG/3TC +94 cells/mmc (*p* < 0.001); in 3DR +87 cells/mmc (*p* < 0.001). No between groups difference in immunological recovery were observed (*p* = 0.493). In the subpopulation with CD4< 200 cells/mmc, we found 1 VF in DTG/3TC and 2 VF in 3DR (*p* = 0.954) and a significant CD4 gains from baseline to 144 weeks: in DTG/3TC +76 cells/mmc (*p* = 0.003); in 3DR +66 cells/mmc (*p* < 0.001). No between groups difference in immunological recovery were observed (*p* = 0.450).

**Conclusions**: DTG/3TC showed good effectiveness with comparable immunological recovery to 3DR in a population of VS PWH switching with low CD4 counts.

**FIGURE 1:**
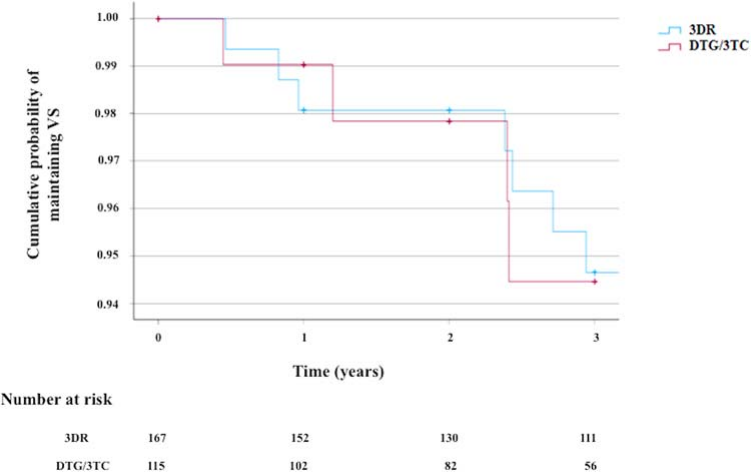
P134 | KM curves for the probability of maintaining virological suppression (VS) according to the 3DR and the DTG/3TC group.

**TABLE 1:**
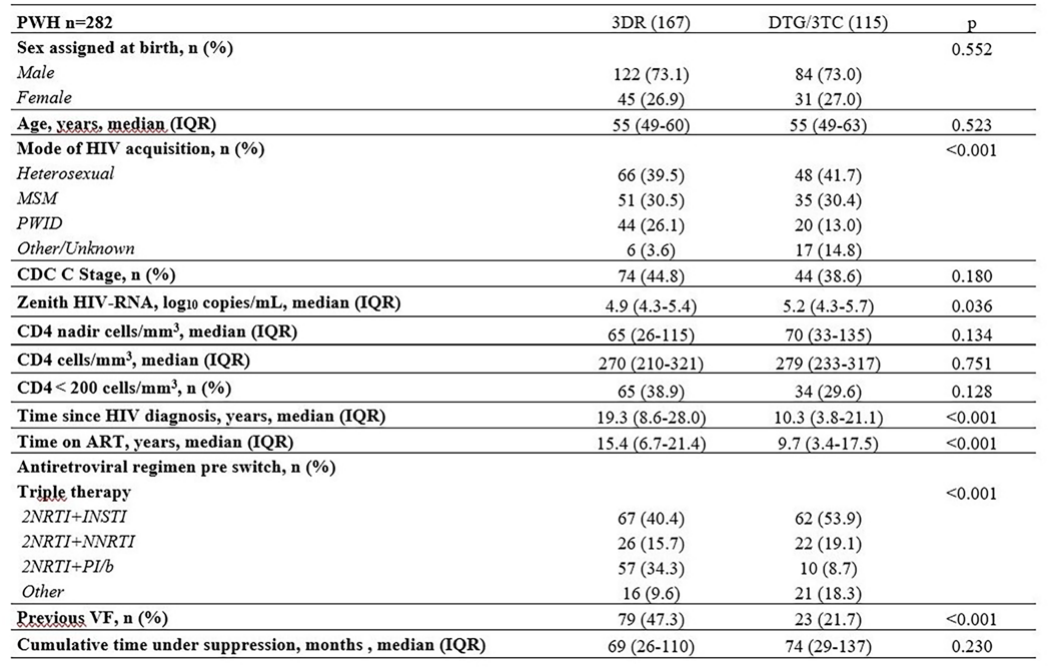
P134 | Baseline characteristics and difference between groups.

### P135 Long‐Term Viral Reservoir, T‐Cell Homeostasis, Inflammation and Mitochondrial Damage in People With HIV Switching to Long‐Acting Injectable Cabotegravir/Rilpivirine

Matteo Augello ^1^, Valeria Bono^1^, Chiara Orlandi^2^, Roberta Rovito^1^, Valentina Sala^1^, Camilla Tincati^1^, Anna Casabianca^2^, Giulia C. Marchetti^1^

^1^Clinic of Infectious Diseases and Tropical Medicine, San Paolo Hospital, ASST Santi Paolo e Carlo, Department of Health Sciences, University of Milan, Milan, Italy; ^2^Department of Biomolecular Sciences, University of Urbino “Carlo Bo”, Urbino, Italy

**Background**: Long‐acting injectable (LAI) antiretroviral therapy containing cabotegravir (CAB) and rilpivirine (RPV) have shown solid results in terms of virologic suppression. However, data on the impact on HIV reservoir, T‐cell homeostasis, inflammation and mitochondrial damage are limited. We, therefore, aimed to assess these parameters in a cohort of people with HIV (PWH) switching to LAI‐CAB/RPV from either an oral 3‐drug (3DR) or 2‐drug regimen (2DR).

**Methods**: The following parameters were measured at baseline (T0) and 12 months following the switch (T12): total/integrated/unintegrated HIV‐DNA in PBMCs (Real‐Time PCR); plasma soluble p24 as a measure of the translationally competent reservoir (Simoa); CD4/CD8 ratio, functionally competent CD4 T‐cells (CD4+CD127+), activated (CD8+CD38+) and primed‐activated (CD8+CD45RO+CD38+) CD8 T‐cells in whole blood (flow cytometry); plasma IP‐10 and IFN‐α (Ella); circulating cell‐free mitochondrial DNA (ccf‐mtDNA) in plasma (real‐time PCR, *ND‐1*gene).

**Results**: Sixty‐four virologically suppressed PWH were enrolled: 30 switching from 3DRs, 34 from 2DRs (87.5% males; median [IQR] age: 47 [40–53] years, CD4 count: 745 [586–943] cells/µL; Table 1). While no changes in HIV tDNA/iDNA/uDNA were observed overall (Figure 1A), PWH‐2DR had higher uDNA levels at T0, with reduction at T12 reaching levels comparable to PWH‐3DR (Figure 1B). No changes in plasma p24 were detected overall (Figure 1C) and according to the pre‐switch regimen. CD4/CD8 ratio and activated CD8 T‐cells did not differ between the two time‐points in both groups (Figure 1D‐G), yet a slight reduction in CD4+CD127+ cells was observed, especially in PWH‐2DR (Figure 1F). No changes were registered in IP‐10 both overall and according to the pre‐switch regimen, while IFN‐α declined in PWH‐3DR (Figure 1H,I). Lastly, ccf‐mtDNA was stable in both groups (Figure 1J).

**Conclusions**: In virologically suppressed PWH, switching to LAI‐CAB/RPV was not associated with major 12‐month changes in HIV reservoir size or expression, T‐cell homeostasis, inflammation or mitochondrial damage, altogether supporting the long‐term viro‐immunological stability of LAI‐CAB/RPV. Notably, PWH‐2DR exhibited higher baseline levels of uDNA, which declined after the switch, suggesting a potential beneficial effect of LAI‐CAB/RPV on residual intracellular HIV replication in this subgroup. Furthermore, PWH‐3DR showed a modest reduction in IFN‐α, whereas PWH‐2DR experienced a slight decrease in CD4+CD127+ cells, highlighting subtle differences in immunological responses according to the pre‐switch regimen.

**FIGURE 1:**
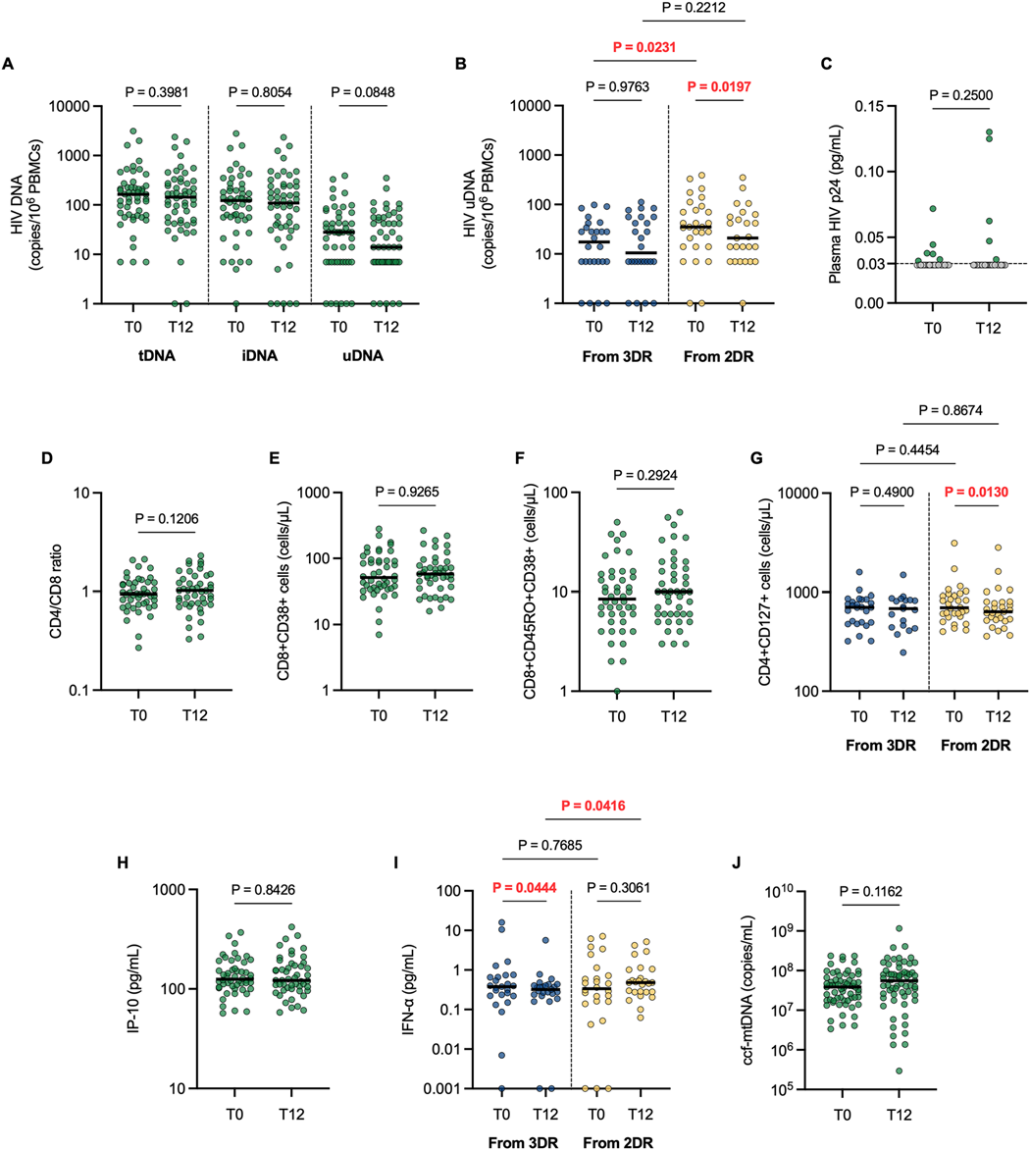
P135 | HIV DNA (A, B), plasma HIV p24 (C), CD4/CD8 ratio (D), activated CD8 T‐cells (E, F), functionally competent CD4 T‐cells (G), plasma IP‐10 (H), plasma IF‐alpha (I), circulating cell‐free mitochondrial DNA (J).

**TABLE 1:**
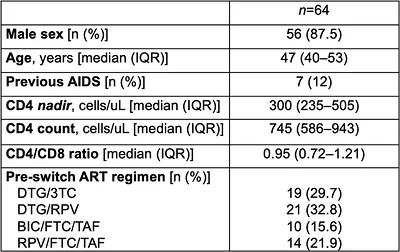
P135 | Demographic and clinical characteristics of the study population.

### P136 Real‐World Effectiveness and Tolerability of Bictegravir/Emtricitabine/Tenofovir Alafenamide in People Living With HIV in Belgium: A 144‐Week Analysis

Rakan Nasreddine^1^, Maartje van Frankenhuijsen^2^, Jean Cyr Yombi ^3^, Marie‐Angélique De Scheerder^4^, Peter Messiaen^5^, Gilles Darcis^6^, Nathalie Ausselet^7^, Jens Van Praet^8^, Sabine D. Allard^9^, Rémy Demeester^10^, Marc Delforge^1^, Stéphane De Wit^1^, on behalf of Belgian Research on AIDS & HIV Consortium

^1^Saint‐Pierre University Hospital, Brussels, Belgium; ^2^Institute of Tropical Medicine, Antwerp, Belgium; ^3^Cliniques Universitaires Saint‐Luc, Brussels, Belgium; ^4^Ghent University Hospital, Ghent, Belgium; ^5^Jessa Hospital, Hasselt, Belgium; ^6^Liège University Hospital, Liège, Belgium; ^7^UCL University Hospital Namur‐Godinne, Yvoir, Belgium; ^8^AZ Sint‐Jan Brugge AV, Brugge, Belgium; ^9^Universitair Ziekenhuis Brussel, Brussels, Belgium; ^10^University Hospital of Charleroi, Lodelinsart, Belgium

**Background**: Bictegravir/emtricitabine/tenofovir alafenamide (BIC/FTC/TAF) is recommended for treatment‐naïve (TN) and experienced (TE) people living with HIV (PLWH). The 48‐week Belgian Research on AIDS and HIV Consortium (BREACH) real‐world study showed BIC/FTC/TAF to be highly effective and well tolerated, with rare occurrence of emergent resistance [1]. Long‐term real‐world data are needed to evaluate for continued efficacy. The aim of this study was to assess the long‐term effectiveness and tolerability of BIC/FTC/TAF within the BREACH cohort.

**Methods**: The BREACH study was a Belgian retrospective, multicentre study, including adult TN and TE PLWH treated with BIC/FTC/TAF between 1 January 2019 and 30 September 2020. Participants from that study, who remained on BIC/FTC/TAF and were in follow up at week 48, were included in this analysis (no new participants were added). Primary endpoint was viral suppression (plasma HIV‐1 viral load [VL] < 50 copies/mL; on‐treatment analysis) at week 144. Main secondary endpoints (week 144) included loss of viral suppression (LVS; 2 consecutive HIV‐1 VLs of > 200 copies/mL after initially being suppressed); treatment failure (TF; LVS with discontinuation of BIC/FTC/TAF); emergent resistance‐associated mutations (RAMs) at TF; treatment discontinuation; and weight gain.

**Results**: Overall, 1369 participants were included. Baseline characteristics: age ≥50 years (42.4%), Black (30.2%), women (sex at birth; 34.8%), TN (22.7%), CD4^+^ < 500 cells/µL (40.1%) and non‐suppressed (> 50 copies/mL; 32.4%). At 144 weeks, overall viral suppression was 94.2% with similar results observed among: age ≥50 years (94.3%), women (94.3%), Black (91%), TN (94.6%), TE (94.1%), CD4^+^ < 500 cells/µL (93.2%) and non‐suppressed at baseline (88%). Eighteen (1.3%) participants experienced LVS, all resuppressed without treatment change. TF was observed in 5 (0.37%) participants: 2 (0.15%) had VLs too low for resistance testing, 2 (0.15%) did not develop RAMs, while 1 (0.07%) had emergent 184 V and 263K mutations. Of the 169 (12.3%) treatment discontinuations, most common cause was switch to a two‐drug regimen (31/169; 18.3%). Median (IQR) weight gain was 2.8 kg (−1.3 to 7.9).

**Conclusions**: In this long‐term, real‐world analysis of BIC/FTC/TAF, treatment maintained a high rate of viral suppression, was well tolerated and continued to show a high barrier to resistance.

**Reference**

1. Nasreddine R, Florence E, Yombi JC, Henrard S, Darcis G, Van Praet J, et al. Efficacy, durability and tolerability of bictegravir/emtricitabine/tenofovir alafenamide for the treatment of HIV in a real‐world setting in Belgium. HIV Med. 2023;24(8):914‐924.

### P137 Abstract Withdrawn

### P138 Beyond Viral Suppression: Sustained Reductions in T‐Cell Activation and Senescence After Switching to Long‐Acting Cabotegravir/Rilpivirine Over 36 Months in People Living With HIV

Martina Proietti ^1^, Mariasilvia Guardiani^1^, Federica Dominelli^1^, Anna Carraro^1^, Eeva Tortellini^1^, Lorenzo Ansaldo^2^, Maria Antonella Zingaropoli^1^, Michele Antonacci^1^, Vito Speciale^1^, Fabio Mengoni^1^, Giulia Mancarella^1^, Andrea Gasperin^1^, Marco De Masi^1^, Serena Vita^3^, Claudio Maria Mastroianni^1^, Raffaella Marocco^2^, Cosmo Del Borgo^2^, Miriam Lichtner^3^

^1^Department of Public Health and Infectious Diseases, Sapienza University, Rome, Italy; ^2^Infectious Diseases Unit, SM Goretti Hospital, Rome, Italy; ^3^Department of Neurosciences, Mental Health and Sense Organs, NESMOS, Sapienza University of Rome, Rome, Italy

**Background**: Although combination antiretroviral therapy (ART) has dramatically improved the prognosis of people with HIV (PWH), persistent chronic inflammation, immune activation and accelerated cellular senescence remain hallmarks of HIV treatment. Data regarding the long‐term immunoinflammatory impact of long‐acting cabotegravir plus rilpivirine (CAB/RPV‐LA) are still limited. Therefore, we evaluated longitudinal changes in T‐cell activation and senescence over 36 months in PWH switching to CAB/RPV‐LA.

**Methods**: PWH switching to CAB/RPV‐LA (LA) and an oral ART control group (CG) were recruited for this study. T‐cell activation (HLA‐DR^+^CD38^+^) and senescence (CD28^−^CD57^+^) in CD4^+^ and CD8^+^ subsets were longitudinally assessed at baseline (T0) and at 12 (T12), 24 (T24) and 36 (T36) months post‐switch. The CG were evaluated at T0, T12 and T24 months (Figure 1).

**Results**: Thirty‐eight LA (female/male: 13/25, age 48 [35–56] years) and 16 CG (female/male: 5/11, age 56 [34–63] years) were enrolled (Table 1). Total CD4+ increased at T36 versus T0 (*p* = 0.0370). The CD4/CD8 ratio remained stable in LA over time. To date, 25 LA had completed 24 months of follow‐up. Compared to baseline, percentages of CD4+ HLA‐DR^+^CD38^+^ T‐cell significantly declined at T12 (*p* = 0.0218), whereas percentages of CD8+ HLA‐DR^+^CD38^+^ T‐cell slightly increased at T24 (*p* = 0.0678). Percentages of CD4+ and CD8+ CD28−CD57^+^ T‐cells decreased at T36 compared to T0 (*p* < 0.0001; *p* < 0.0001). Percentages of CD4+ HLA‐DR^+^CD38^+^ T‐cell decreased at T36 compared to T0 (*p* = 0.0156), while CD8+ compartment remained stable. CD4+ CD28−CD57^+^ decreased significantly at T36 (*p* < 0.0004) and CD8+ CD28−CD57^+^ T‐cell percentages significantly decreased at T24 compared to T0 (*p* = 0.0219). No significant longitudinal changes in activation or senescence markers were observed in the CG at any follow‐up time point (all adjusted *p* > 0.9999). Compared with CG, LA showed higher percentages of CD4^+^ HLA‐DR^+^CD38^+^ T‐cells only at T12 (*p* = 0.0196) with no between‐group differences in CD8^+^ HLA‐DR^+^CD38^+^ T‐cell percentages at any time‐point.

**Conclusions**: Over 36 months, CAB/RPV‐LA was associated with reduced T‐cell senescence and CD4+ T‐cell activation, while maintaining a stable CD4/CD8 ratio. No comparable changes were observed with oral ART. These differences may reflect the more stable pharmacokinetic profile of CAB/RPV‐LA, potentially reducing immune activation by limiting drug level fluctuations and adherence‐related variability. Overall, these findings suggest a favourable long‐term immunological profile beyond virological suppression.

**FIGURE 1:**
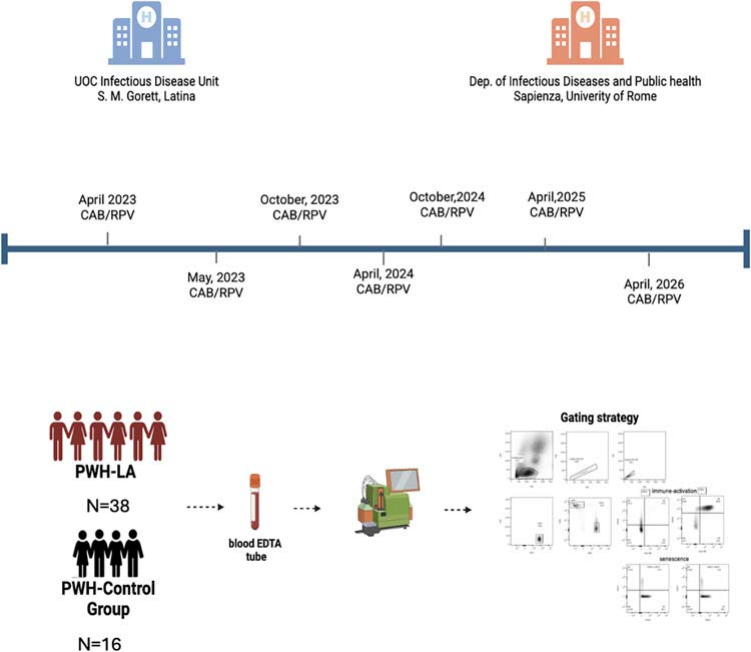
P138 | Study design. Longitudinal peripheral blood samples were collected from people with HIV receiving long‐acting cabotegravir/rilpivirine (PWH‐LA, *n* = 38) between April 2023 and April 2026. A control group receiving oral ART (PWH‐control, *n* = 16) was included for comparison. Immune phenotyping was performed by flow cytometry to evaluate T‐cell activation and senescence markers.

**TABLE 1:**
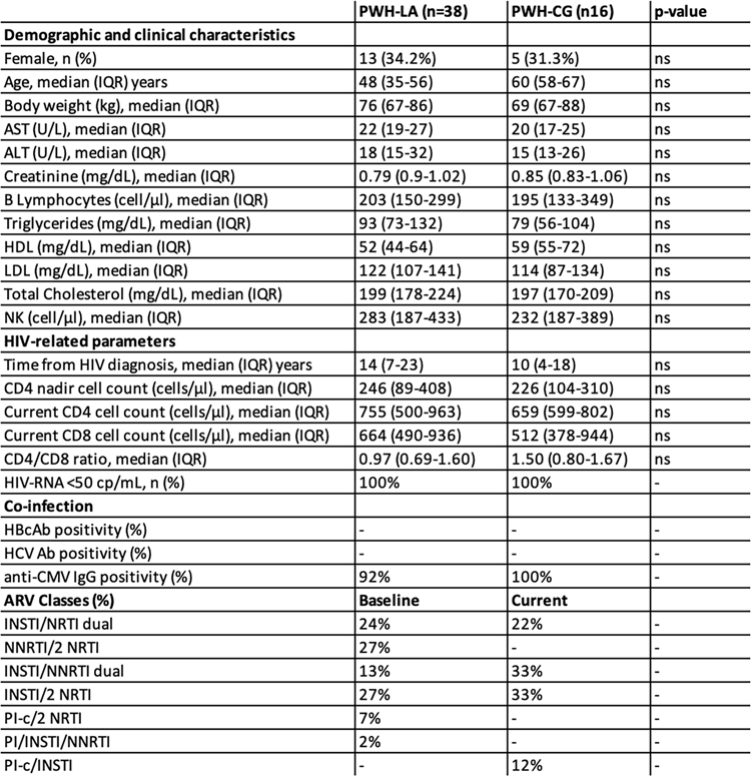
P138 | Demographic and clinical characteristics of the study population. Data are shown as median (interquartile range, IQR); *n*, number, PWH‐LA, people with HIV, PWH‐CG, control group.

### P139 Abstract Withdrawn

### P140 Real‐World Effectiveness and Patient‐Reported Outcomes of Long‐Acting Cabotegravir–Rilpivirine in People With HIV in South Korea: A Prospective Observational Study

Hyeongseok Jeong, Shinhye Cheon, Younghee Jung, Yeon‐Sook Kim

Division of Infectious Diseases, Department of Internal Medicine, Chungnam National University School of Medicine, Daejeon, Republic of Korea

**Background**: Long‐acting injectable (LAI) cabotegravir–rilpivirine offers an alternative to daily oral antiretroviral therapy (ART), with the potential to enhance long‐term treatment adherence. However, real‐world data regarding treatment implementation and patient‐reported outcomes (PROs) in South Korea remain limited. This study aimed to evaluate the real‐world effectiveness, adherence and PROs among people with HIV switching to LAI therapy in South Korea.

**Methods**: This single‐centre, prospective observational study enrolled people with HIV switching to LAI cabotegravir–rilpivirine from May 2025 to March 2026 at a tertiary referral university hospital. The primary outcome was virological suppression (HIV‐1 RNA <50 copies/mL) at 3 months post switch. Secondary outcomes included changes in CD4+ T‐cell counts, adherence (attendance vs. scheduled visits) and PRO assessed with a structured 12‐item questionnaire and evaluated post‐switch using a 7‐point scale (−3 to +3), with higher scores indicating favourability towards LAI treatment.

**Results**: A total of 114 people with HIV were evaluated. The overall study population had a median age of 54 years (interquartile range [IQR] 43–64) and was predominantly male (108/114, 94.7%). At 3 months post‐switch, virological suppression was maintained in 100/103 (97.1%) participants with available laboratory data, showing no significant change from baseline (99/103, 96.1%; *p* = 0.85). The median CD4+ T‐cell count remained stable, shifting from 770 cells/mm^3^ (IQR 514–1025) at baseline to 758 cells/mm^3^ (IQR 505–942) (*p* = 0.23). Over a median follow‐up period of 9.7 months (IQR 8.3–11.1), 105 participants (92.1%) maintained the LAI therapy with 100% adherence. Conversely, 9 (7.9%) discontinued LAI and resumed oral therapy for reasons including adverse events (*n* = 4; injection‐site pain and constitutional symptoms), logistical or financial barriers (*n* = 4; hospital visit constraints, relocation or insurance issues) and death unrelated to treatment (*n* = 1). PRO analysis among 76 participants demonstrated high overall satisfaction and convenience (overall mean score 2.3 ± 1.1), with domain‐specific heterogeneity, particularly regarding treatment‐related discomfort and requirements (Figure 1).

**Conclusions**: LAI cabotegravir–rilpivirine maintained virological suppression with high patient satisfaction in a real‐world South Korean cohort.

**FIGURE 1:**
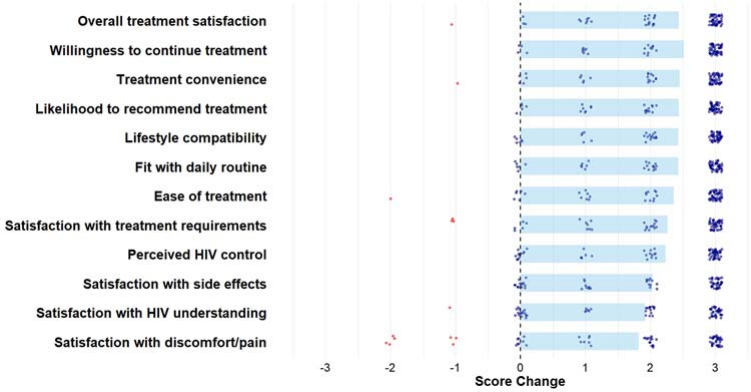
P140 | Changes in patient‐reported outcome (PRO) scores. Individual questionnaire responses are shown as scatter points and mean change scores represented by shaded bars.

### P141 Efficacy and Safety of Bictegravir/Emtricitabine/Tenofovir Alafenamide (BIC/FTC/TAF) in Older People Living With HIV: Real‐World Evidence From Saudi Arabia

Roaa Alosaimi, Batool Ali, Tala Galai, Ibrahim Alshehri, Alaa Malki, Osama Alghamdi

Jeddah First Health Cluster, East Jeddah Hospital, Jeddah, Saudi Arabia

**Background**: The growing population of older people living with HIV (PLWH) requires antiretroviral regimens that provide durable virological suppression while maintaining favourable tolerability and minimizing the risk of drug–drug interactions. We evaluated the real‐world effectiveness and safety of bictegravir/emtricitabine/tenofovir alafenamide (BIC/FTC/TAF) [1,2] in routine clinical care in Saudi Arabia.

**Methods**: This retrospective observational study included PLWH aged ≥50 years receiving care at East Jeddah Hospital, Saudi Arabia, who either initiated BIC/FTC/TAF as first‐line antiretroviral therapy (treatment‐naïve) or switched from another regimen (treatment‐experienced). Demographic, clinical, immunological and virological data were extracted from electronic medical records. The primary endpoint was virological suppression (HIV RNA <50 copies/mL or undetectable) at the last follow‐up. Safety outcomes included treatment discontinuation and adverse events.

**Results**: A total of 528 patients were included, comprising 42 treatment‐naïve and 486 treatment‐experienced individuals. In the treatment‐naïve cohort, the mean baseline HIV RNA was 175,450 ± 273,566 copies/mL and 39/42 patients (92.9%) achieved virological suppression at the last follow‐up. In the treatment‐experienced cohort, 478/484 evaluable patients (98.8%) maintained or achieved virological suppression after switching to BIC/FTC/TAF (Figure 1). Treatment discontinuation was infrequent (15/482, 3.1%), with reduced renal function (creatinine clearance < 30 mL/min) accounting for 12/15 (80.0%) discontinuations and rash for 3/15 (20.0%). No unexpected treatment‐emergent serious adverse events were observed.

**Conclusions**: BIC/FTC/TAF demonstrated excellent real‐world effectiveness and tolerability in older PLWH. High rates of virological suppression and a low discontinuation rate support its use as a preferred antiretroviral regimen for older adults living with HIV.

**Funding**: This study received an unrestricted educational grant from Gilead Sciences Arabia Limited LLC to support publication. The sponsor had no role in the study design, data collection, data analysis, interpretation of the results, manuscript preparation or the decision to submit the work for publication. No employees or representatives of Gilead Sciences are listed as co‐authors.

**References**

1. Rolle CP, Berhe M, Singh T, et al. Real‐world efficacy and safety of switching to bictegravir/emtricitabine/tenofovir alafenamide in people with HIV. Infect Dis Ther. 2021;10(3):1467‐1478.

2. Esser S, Stellbrink HJ, Llibre JM, et al. Twelve‐month effectiveness and safety of bictegravir/emtricitabine/tenofvir alafenamide in treatment‐naïve and treatment‐experienced people with HIV. Presented at the International AIDS Conference; 2024.

**FIGURE 1:**
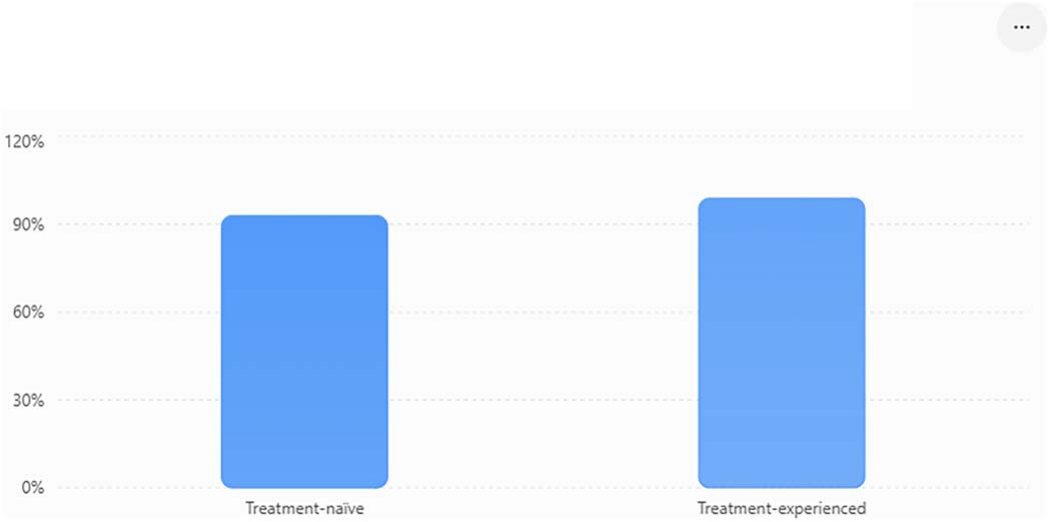
P141 | Virological suppression with BIC/FTC/TAF in older PLHIV. Virological suppression (HIV RNA <50 copies/mL or undetectable) at last follow‐up in treatment‐naïve and treatment‐experienced patients.

### P142 Supporting Individualized Treatment Strategies With NGS: Eligibility for Islatravir/Doravirine in ART‐Naïve Individuals

Marianna Giurco ^1^, Giovanni Massobrio^1^, Vincenzo Paolozzi^1^, Valentina Ricucci^2^, Federica Stefanelli^2^, Nadia Randazzo^2^, Lucia Taramasso^3^, Matteo Bassetti^1,3^, Antonio Di Biagio^1,3^

^1^Department of Health Sciences (DISSAL), University of Genoa, Genoa, Italy; ^2^Hygiene Unit, IRCCS Azienda Ospedaliera Metropolitana, Genoa, Italy; ^3^Infectious Disease Clinic, IRCCS Azienda Ospedaliera Metropolitana, Genoa, Italy

**Background**: The encouraging preliminary results from the phase 3 MK‐8591A‐053 trial suggest that the once‐daily two‐drug regimen islatravir plus doravirine (ISL/DOR) may emerge as a viable treatment option for antiretroviral therapy (ART)‐naïve people living with HIV (PLWH), offering an integrase strand transfer inhibitor (INSTI)‐sparing strategy. Treatment individualization is facilitated by the use of next‐generation sequencing (NGS) at HIV diagnosis, which broadens the detection of clinically relevant viral variants.

**Methods**: This monocentric, retrospective, observational study, conducted from 2022 to 2025, sought to assess the feasibility of ISL/DOR as first‐line ART in a real‐world population. Our cohort included 133 ART‐naïve individuals (Table 1). All subjects underwent NGS of blood samples at diagnosis and RAMs with variant allele frequencies (VAFs) ≥5% were identified. Data about the population's characteristics, viral subtype and HBV serologies at diagnosis were collected. ISL/DOR eligibility criteria included: (i) absence of ISL‐related RAMs; (ii) absence of DOR‐related RAMs; (iii) HbsAg negativity. Descriptive statistics were used to analyse the data.

**Results**: Individuals with incomplete HBV serology (*N* = 13) were excluded from subsequent analysis (Figure 1). Among the study cohort (*N* = 120), 80 individuals harboured wild‐type (WT) HIV variant. Within this subgroup, 18 PLWH were HBcAb‐positive, of whom 2 were also HBsAg‐positive. Among the 40 non‐WT HIV infections, one had an M184I mutation at a VAF of 5% and four had an A98G mutation, all with VAFs ≥97%. None of the other detected mutations was associated with increased resistance to ISL or DOR. Within the non‐WT subgroup, HbsAg positivity was recorded in 2 out of 10 HbcAb‐positive individuals. Overall, 111 PLWH, corresponding to 93% of the cohort, were eligible for ISL/DOR at the time of infection diagnosis.

**Conclusions**: The choice of ISL/DOR in ART‐naïve individuals may offer advantages in selected cases by avoiding INSTI‐specific drug interactions, particularly those involving polyvalent cation‐containing products and in PLWH with a medical history of neuropsychiatric conditions. The implementation of NGS at HIV diagnosis, combined with the collection of medical history and serological data, supports individualized treatment strategies. The majority of our cohort was eligible for ISL/DOR, underlining its potential use in this real‐life setting.

**FIGURE 1:**
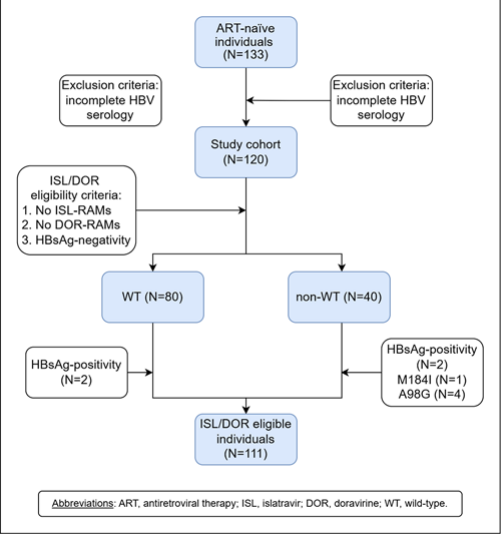
P142 | Study design and results.

**TABLE 1:**
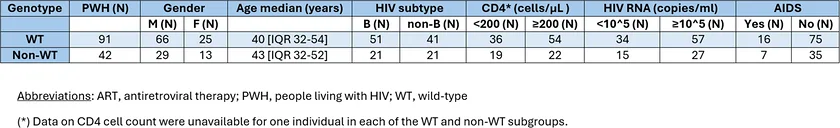
P142 | Cohort of ART‐naïve individuals from 2022 to 2025.

### P143 Albuvirtide Enhances Immune Recovery in Treatment‐Naïve Patients With Advanced HIV Infection Receiving Bictegravir/Emtricitabine/Tenofovir Alafenamide: A Retrospective Propensity Score‐Matched Cohort Study

Yingchun Zhu ^1^, Huanxia Liu^2^, Yin Wang^1^, Zongzheng Wang^1^, Lin Cai^1^

^1^Clinical Section 2, Department of Infectious Disease, Chengdu, China; ^2^Clinical Section 1, Department of Infectious Disease, Chengdu, China

**Background**: Treatment‐naïve patients with advanced HIV infection may experience incomplete immune reconstitution despite standard antiretroviral therapy (ART). This study assessed whether intensification of bictegravir/emtricitabine/tenofovir alafenamide (B/F/TAF) with the fusion inhibitor albuvirtide (ABT) was associated with improved short‐term immune recovery and acceptable safety signals.

**Methods**: This was a single‐centre retrospective cohort study enrolling consecutive patients initiating antiretroviral therapy (ART) at Chengdu Public Health Clinical Medical Center between January 2023 and January 2025. Eligible participants were treatment‐naïve adult patients with advanced HIV infection presenting with baseline CD4^+^ T‐cell count < 200 cells/µL. To mitigate selection bias, propensity score matching (PSM) with a maximum matching ratio of 1:2 was performed to balance baseline covariates. The primary endpoint was the immune recovery rate at week 24, defined as a CD4^+^ T‐cell increment > 150 cells/µL alongside plasma HIV RNA <50 copies/mL. Secondary endpoints consisted of virological suppression rate (HIV RNA < 20 copies/mL) and the incidence of adverse events.

**Results**: At week 24, the immune recovery rate was significantly higher in the ABT+B/F/TAF group than in the B/F/TAF group (45.5% vs. 22.6%, *p* = 0.027). Virological suppression rate in the ABT+B/F/TAF group was numerically higher than that in the B/F/TAF group, with a marginally significant between‐group difference (75.8% vs. 54.7%, *p* = 0.050). To overcome the sample size reduction limitation inherent in the PSM process and to further verify the independent effect of the intervention, unadjusted and multivariable‐adjusted logistic regression models were constructed in the complete pre‐matching cohort (*N* = 124). The multivariate analysis demonstrated that albuvirtide combination therapy and baseline CD4^+^ T‐cell count were independent protective factors for immune recovery (adjusted OR [aOR] 6.872, 95% CI: 1.636–28.864, *p* = 0.008; aOR 1.018, 95% CI: 1.002–1.034, *p* = 0.031). Over 24 weeks, adverse event rates were 54.55% (albuvirtide+B/F/TAF) and 50.94% (B/F/TAF monotherapy), with no between‐group difference (*p* = 0.732).

**Conclusions**: Intensification of B/F/TAF with albuvirtide was associated with a higher immune recovery rate and numerically superior virological suppression at week 24 in treatment‐naïve patients with advanced HIV infection.

### P144 Demographics, Clinical Characteristics and Treatment Patterns of People With HIV Who Are Virologically Suppressed Receiving Complex Regimens in Japan: A Real‐World Database Study Using Electronic Medical Records

Soodi Navadeh ^1^, Nao Taguchi^2^, Jesse Najarro Cermeño^3^, Reiko Yamaura^4^, Siming Chen^4^, Mellissa Williamson^1^, Peter Sklar^1^, Dai Watanabe^5^

^1^Gilead Sciences, Inc., Foster City, CA, US; ^2^Gilead Sciences K.K., Tokyo, Japan; ^3^Gilead Sciences, Inc., Parsippany, NJ, US; ^4^IQVIA Solutions Japan, Tokyo, Japan; ^5^AIDS Medical Center, NHO Osaka National Hospital, Osaka, Japan

**Background**: Although modern antiretroviral therapy allows most people with HIV (PWH) to achieve virologic suppression (VS) using single‐tablet regimens, a smaller subgroup requires complex regimens (CRs) because of resistance, intolerance, contraindications or drug−drug interactions. These individuals often experience multiple treatment changes and higher treatment burden yet remain poorly characterized. This study seeks to describe the real‐world clinical profile and outcomes of PWH who are VS on CR in Japan.

**Methods**: This retrospective observational study utilized DATuM IDEA electronic medical record database, a nationally representative dataset capturing clinical information from over 1.5 million individuals across 58 major Regional Core Hospitals for HIV Care in Japan between January 2019 and March 2025. Adults (≥18 years) who received ≥1 CR, defined as a regimen consisting of ≥2 core agents (non‐nucleoside reverse transcriptase inhibitor, protease inhibitor or integrase strand transfer inhibitor), a boosted PI–based regimen, twice‐daily dosing or multi‐tablet regimens (MTRs). VS was defined within ± 90 days of CR initiation. Baseline characteristics of PWH who are VS on CR were reported. The baseline period was defined as 6 months before the CR initiation.

**Results**: Of 6609 PWH on treatment, 702 received a CR, of whom 515 had observed VS based on available measurements (Figure 1). Most VS on CR individuals (81.8%) received MTRs and were taking a median of 2.0 (range 1–18) pills per day; 25.0% were treated with two of three core classes and 82.7% received boosted PI‐based regimens. The most frequently observed complex regimen was darunavir+cobicistat+emtricitabine+tenofovir alafenamide (40.6%). The median age of the VS on CR population at the time of CR initiation was 47 years (IQR 41–55). Comorbidity burden was notable with 29.4% having eGFR < 60 mL/min/1.73 m^2^, 33.9% classified as overweight or obese, 8.8% with sexually transmitted infection history, 4.5% with hyperlipidaemia and 10.5% with diabetes (Table 1).

**Conclusions**: Approximately 10.4% of PWH in Japan were VS on CR. Despite achieving virologic control, the majority remained on MTRs with some having comorbidity burden. Given the potential challenges associated with these regimens, this highlights an opportunity to address a persistent unmet clinical need for treatment simplification and optimization.

**FIGURE 1:**
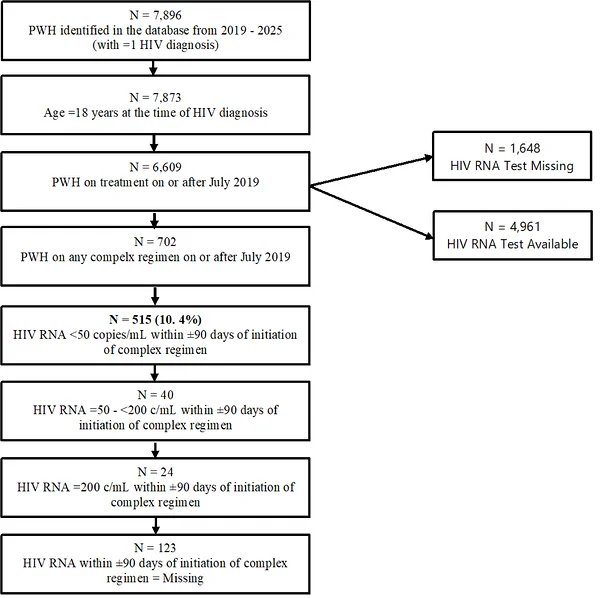
P144 | Cohort summary.

**TABLE 1:**
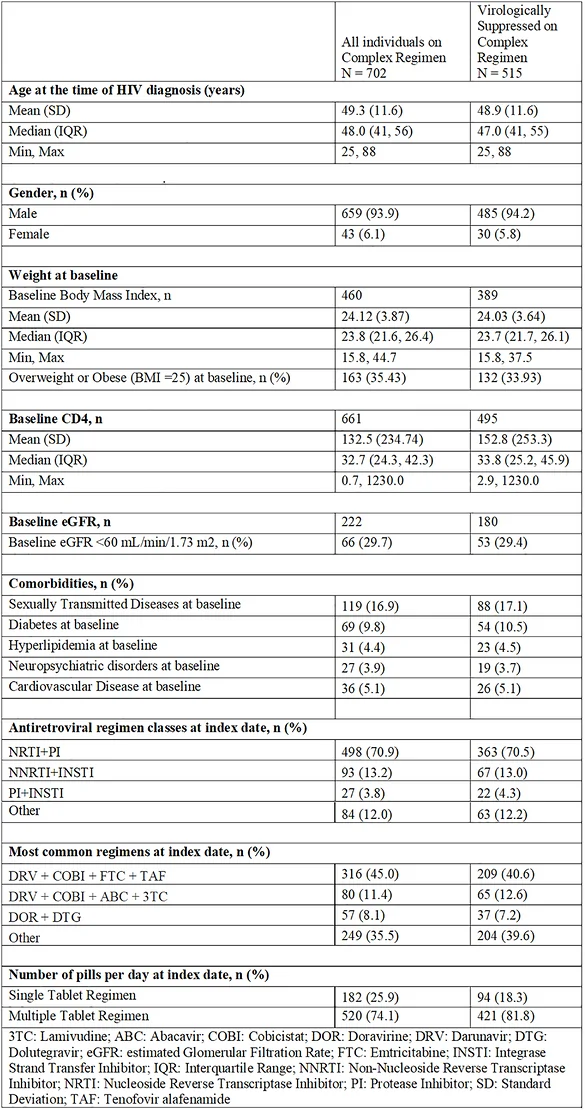
P144 | Demographic and clinical characteristics.

### P145 Patient‐Reported Outcomes Following Switch to Long‐Acting Cabotegravir+Rilpivirine in Nordic Clinical Care: Results From the VALHALLA Study

Liisa Lehtoranta ^1^, Anne‐Mette Lebech^2^, Jussi Sutinen^3^, Inka Aho^3^, Raquel Martin‐Iguacel^4^, Ellen Moseholm^5^, Alex Lund Laursen^6^, Anders Krifors^7,8^, Piotr Nowak^8^, Steffen Leth^9,10^, Jamirah Nazziwa^11^, Hassan Aladdin^12^, Felipe Rogatto^13^, Jenny Scherzer^13^, Melanie Schroeder^13^

^1^GSK/ViiV Healthcare, Helsinki, Finland; ^2^Copenhagen University Hospital‐Rigshospitalet, Copenhagen, Denmark; ^3^Department of Infectious Diseases, Helsinki University Hospital, Helsinki, Finland; ^4^Department of Infectious Disease, Odense University Hospital, Odense, Denmark; ^5^Department of Infectious Diseases, Copenhagen University Hospital, Hvidovre, Denmark; ^6^Department of Infectious Diseases, Aarhus University Hospital, Aarhus, Denmark; ^7^Centre for Clinical Research, Västmanland Hospital Västerås, Region Västmanland‐Uppsala University, Västerås, Sweden; ^8^Department of Medical Sciences, Uppsala University, Uppsala, Sweden; ^9^Department of Infectious Diseases & Internal Medicine, Gødstrup Regional Hospital, Herning, Denmark; ^10^Department of Clinical Medicine, Aarhus University, Aarhus, Denmark; ^11^GSK/ViiV Healthcare, Stockholm, Sweden; ^12^GSK/ViiV Healthcare, Copenhagen, Denmark; ^13^ViiV Healthcare, London, UK

**Background**: Long‐acting injectable cabotegravir+rilpivirine (CAB+RPV LAI) provides an alternative to daily oral antiretroviral therapy (ART) for people with HIV. Real‐world data on the patient‐reported impact on daily life, treatment satisfaction and psychosocial burden remain limited. The VALHALLA study was designed to evaluate patient‐reported outcomes (PROs) following switch to CAB+RPV LAI in the Nordic countries.

**Methods**: VALHALLA is a non‐interventional, multicentre study conducted in Denmark, Sweden and Finland. Adults living with HIV who switched to CAB+RPV LAI were included. PROs were collected at baseline and either at a 12‐ or 18‐month follow‐up using surveys, including the HIV Treatment Satisfaction Questionnaire (HIVTSQ). HIVTSQ comprised 11 items (scored from 0 to 6) and summed to a total score between 0 and 66, with higher scores indicating greater treatment satisfaction.

**Results**: Of 52 participants from nine centres, 50 completed baseline survey and 39 completed follow‐up surveys at either 12 or 18 months (pooled for analysis). At baseline, participants reported psychosocial and practical challenges with oral ART, including hiding medication from others (38%), treatment‐related reminders of HIV status (30%) and difficulties remembering daily ART (30%); 34% reported no problems. At 12 or 18 months following switch to CAB+RPV LAI, participants reported improvements across multiple domains of daily life (Figure 1). Reports of treatment being always/often the source of stigma decreased from 28% to 0%, fear of revealing HIV status from 32% to 3% and interference with travel or social activities from 22% to 3%. Positive perceptions of self and HIV diagnosis improved from 14% to 92%, while reports of treatment‐related low mood decreased from 28% to 3%. Mean (SD) HIVTSQ score increased by 6.5 points from baseline 55.4 (11.4) to 61.9 (5.1) at 12/18 months; driven mainly by treatment “convenience” and “lifestyle fit.” Most participants (*n* = 37/39) preferred to remain on CAB+RPV LAI at follow‐up.

**Conclusions**: In Nordic real‐world cohort, switching to CAB+RPV LAI was associated with favourable patient‐reported changes in treatment satisfaction, treatment‐related quality of life and psychosocial treatment burden over 12–18 months. CAB+RPV LAI was perceived as a better fit for participants’ daily lives, supporting the value of considering PROs when evaluating LAI ART in routine care.

**FIGURE 1:**
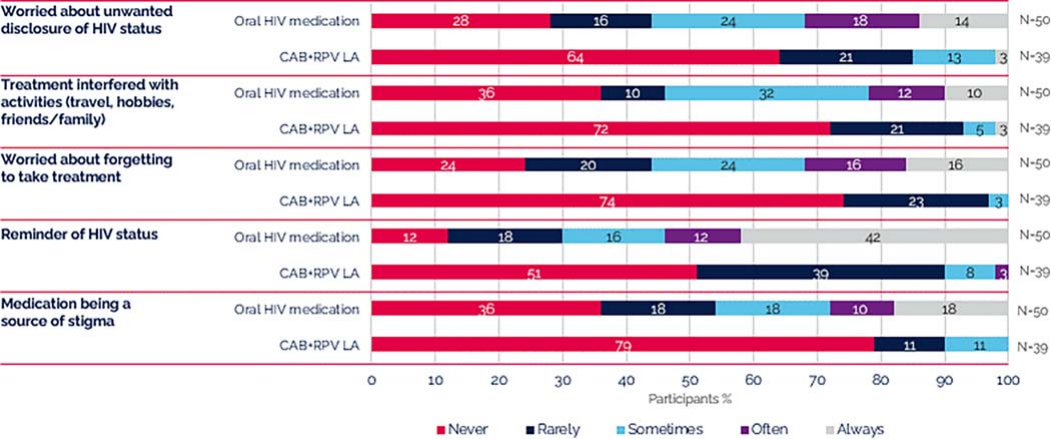
P145 | Reported impact of oral medication at baseline and of CAB+RPV LAI 12/18 months after switch on daily life of people with HIV.

### P146 Real‐World Experience With Long‐Acting Cabotegravir/Rilpivirine in People Living With HIV and Historical Rilpivirine Resistance‐Associated Mutations

Carolina Costa, Rita De Almeida Teixeira, João Matos, Joana Vaz Cardoso, Miguel Araújo Abreu, Josefina Méndez

Serviço Doenças Infecciosas, Unidade Local de Saúde de Santo António, Porto, Portugal

**Background**: Long‐acting injectable cabotegravir/rilpivirine (CAB/RPV‐LA) represents a paradigm shift in the treatment of people living with HIV (PLWH). Although guidelines advise against its use when rilpivirine resistance‐associated mutations (RPV RAMs) are present [1], individual factors—including treatment preference, adherence challenges, stigma and quality of life—may lead clinicians to consider CAB/RPV‐LA despite historical resistance findings or absent baseline resistance testing. As patients with baseline RPV RAMs were excluded from registration trials, evidence in this population remains limited [2]. We assessed virological outcomes of CAB/RPV‐LA in PLWH with historical RPV RAMs.

**Methods**: We conducted a retrospective observational study of PLWH initiating CAB/RPV‐LA between August 2024 and May 2026 at a Portuguese hospital. Patients with RPV RAMs on historical genotyping were included. Mutations were interpreted using the Stanford HIV Drug Resistance Database algorithm (v10.2).

**Results**: Among 338 individuals receiving CAB/RPV‐LA, 208 (61.5%) had a pre‐treatment resistance data, of whom 20 (9.6%) had historical RPV RAMs. Demographic and clinical characteristics are summarized in Table 1. All were virologically suppressed at switch, with a median suppression duration of 3.7 years (IQR 2.4–7.2). Stanford HIVdb interpretation suggested preserved or only limited predicted impact on RPV susceptibility in most cases (19/20, 95%); one had intermediate resistance (5%) and none had high‐level resistance. The most frequent RPV RAM were V106I (7/20, 35%), V90I (5/20, 25%) and E138A (4/20, 20%). BMI > 30 kg/m^2^, a recognized risk factor for virological failure (VF) with CAB/RPV‐LA, was present in 4/20 (20%). At data cut‐off, 10/20 (50%) had more than 12 months of follow‐up after switch. VF occurred in one patient (5%) with no additional recognized risk factors, harbouring baseline E138A with predicted low‐level RPV resistance. At failure, emergent N155H/L74M and M230L were detected, corresponding to intermediate CAB and high‐level RPV resistance, respectively.

**Conclusions**: In this real‐world cohort, most PLWH with historical RPV RAMs maintained virological suppression after switching to CAB/RPV‐LA, suggesting that selected patients with historical RPV RAMs may still benefit from this strategy. However, VF with emergent resistance highlights the need for careful interpretation of historical resistance profiles and further studies to refine patient selection.

**References**

1. Gandhi RT, Landovitz RJ, Sax PE, et al. Antiretroviral drugs for treatment and prevention of HIV in adults: 2024 recommendations of the International Antiviral Society–USA Panel. JAMA. 2025;333(7):572–592.

2. Swindells S, Andrade‐Villanueva JF, Richmond GJ, et al. Long‐acting cabotegravir and rilpivirine for maintenance of HIV‐1 suppression. N Engl J Med. 2020;382(12):1112–1123.

**TABLE 1:**
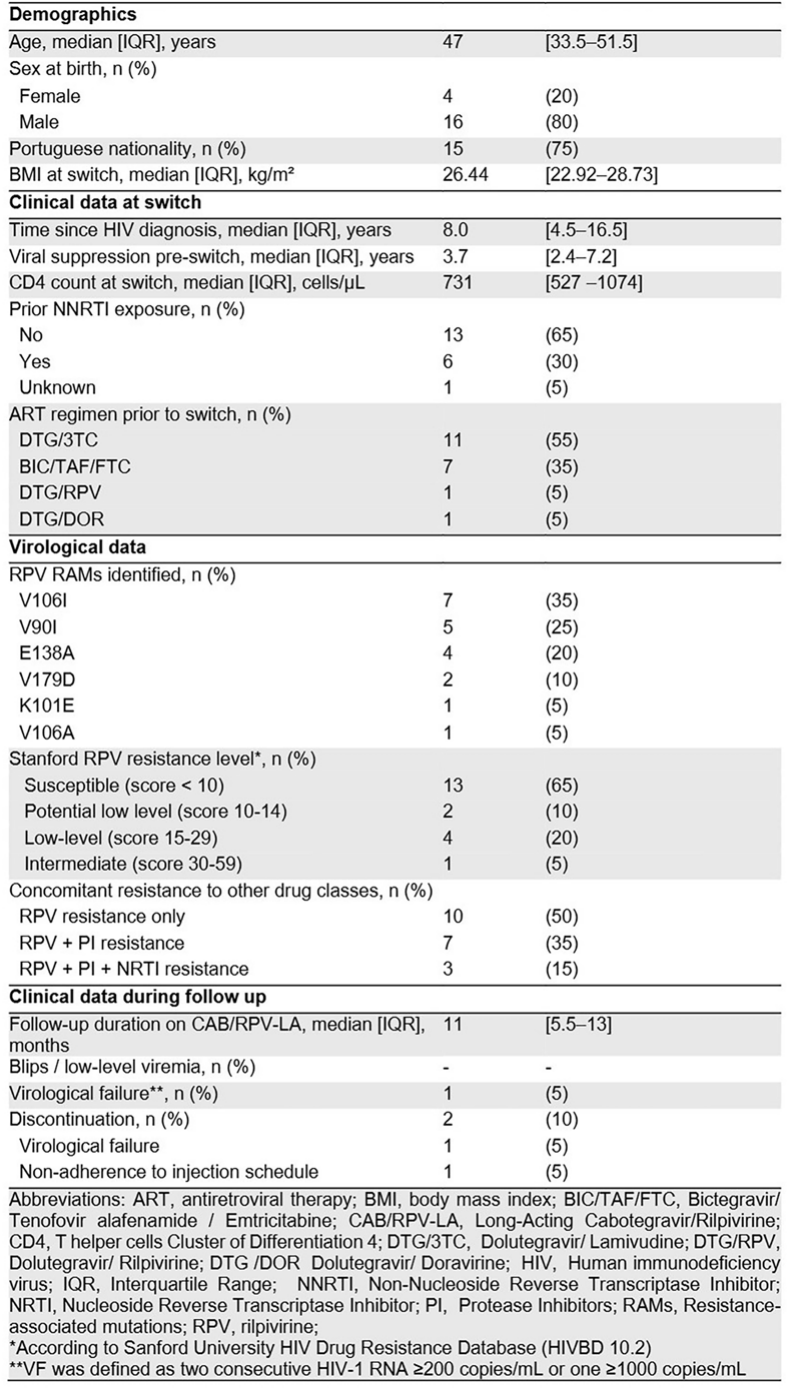
P146 | Characteristics of patients with historical rilpivirine resistance‐associated mutations (*n* = 20).

### P147 Long‐Acting Cabotegravir/Rilpivirine Experience in a Portuguese Hospital: Effectiveness, Tolerability and Hepatitis B Risk Stratification

Margarida Almeida^1^, Constança Antunes^1^, Ana Isabel Duarte^1^, Bruno Rebelo^1^, Margarida Falcão^2^, João Alves^1^, Ana Cláudia Miranda ^1^

^1^Infectious Disease and Tropical Medicine Service; Hospital de Egas Moniz/Unidade Local de Saúde de Lisboa Ocidental, Lisbon, Portugal; ^2^Pharmacy Department; Hospital de Egas Moniz/Unidade Local de Saúde de Lisboa Ocidental, Lisbon, Portugal

**Background**: Long‐acting cabotegravir/rilpivirine (CAB/RPV LA), the first injectable antiretroviral regimen approved as a switch strategy for virologically suppressed people living with HIV‐1 (PLHIV‐1), became available in Portugal in 2024 for administration every 2 months [1,2]. Since CAB/RPV LA lacks activity against hepatitis B virus (HBV), questions remain regarding the monitoring of individuals with prior HBV exposure and the optimal anti‐HBs threshold for booster vaccination [3].

**Methods**: Real‐world, retrospective, observational cohort study included PLHIV‐1 over 18 years old, followed in a Lisbon hospital who initiated CAB/RPV LA between October 2024 and May 2026. Demographic, clinical and laboratory characteristics, reasons for treatment switch, tolerability and self‐reported adverse events were evaluated.

**Results**: One hundred participants initiated CAB/RPV LA (median age 43.5 years, 81% were men). At baseline, the median time since HIV diagnosis was 10.5 years; all participants had HIV‐1 RNA <50 copies/mL and a median CD4+ T‐cell count of 897.5 cells/µL. Most (80%) switched from an integrase inhibitor‐based regimen. Baseline resistance testing results were unavailable for 47% of participants; however, none had a documented history of virological failure. Convenience was the most common reason for switching (69%). Across 495 administrations, injection‐site pain was the most common adverse event (45% of administrations, of these, 55% were mild), followed by fever (3%), myalgia (3%), headache (2%) and injection‐site nodules (1%), including one abscess requiring drainage without treatment discontinuation. One participant died of oesophageal cancer. Three participants discontinued treatment and the reasons were: virological failure without emergent resistance mutations, severe injection‐site pain and recurrent missed scheduled administrations. Among participants remaining on treatment, the median follow‐up was 7 months. Regarding HBV status, 25% of participants were anti‐HBc‐positive, with no cases of HBV reactivation. 18% of participants lacked evidence of prior HBV infection or vaccination. Of these, one developed acute HBV infection while awaiting completion of the vaccination schedule.

**Conclusions**: CAB/RPV LA showed good real‐world tolerability and effectiveness, with one discontinuation due to an adverse event and one case of virological failure (1% of patients for each). The occurrence of acute HBV infection highlights the importance of confirming HBV serological status and ensuring protective HBV vaccination before switching to CAB/RPV LA.

**References**

1. European Medicines Agency. Vocabria. Available at: https://www.ema.europa.eu/en/medicines/human/EPAR/vocabria. Accessed 25/06/2026.

2. European Medicines Agency. Rekambys. Available at: https://www.ema.europa.eu/en/medicines/human/EPAR/rekambys. Accessed 25/06/2026.

3. Geretti AM, Boffito M, Bonora S, Braun P, Charpentier C, Garcia F, et al. Optimizing the use of cabotegravir plus rilpivirine long‐acting therapy in HIV care: evidence, implementation and unanswered questions. Open Forum Infect Dis. 2025 Jul;12(7):ofaf368. doi:10.1093/ofid/ofaf368.

### P148 Long‐Acting Injectable Antiretroviral Therapy Acceptance and Associated Factors in South Korea: A Single‐Centre 1 Year Experience

BumSik Chin, Gayeon Kim, Min‐Kyung Kim, Yeonjae Kim

Division of Infectious Diseases, Department of Internal Medicine, Seoul, Republic of Korea

**Background**: Long‐acting injectable (LAI) antiretroviral therapy (ART) has emerged as a significant alternative to daily oral medications. Despite its clinical benefits, patient‐led adoption rates and specific reasons for treatment preferences in real‐world settings remain under‐explored. This study aimed to evaluate the acceptance rate of LAI ART and identify motivators and deterrents among people living with HIV (PLWH) through a large‐scale retrospective analysis.

**Methods**: A retrospective medical record review was conducted at the National Medical Center in Seoul, South Korea, where 2058 PLWH were registered as May 2026. Following the introduction of LAI ART (cabotegravir and rilpivirine) in South Korea in May 2025, shared decision‐making for LAI was integrated into routine clinical practice. During clinic visits, PLWH were informed of the benefits and drawbacks of LAI ART and their acceptance along with specific reasons for or against the LAI was captured in clinical documentation. Data regarding LAI acceptance and maintenance, documented rationales for acceptance or refusal were extracted from medical records through 26 June 2026.

**Results**: During the observation period, LAI ART was administered to 127 patients (6.2% of the registered cohort). 119 (93.7%) were male and median age was 40 (IQR 34–49). Median observation duration of LAI cohort was 232 days (IQR 154–277). Primary motivators for LAI were disclosure concerns (36.1%) and pill fatigue (31.9%), while the most significant reasons against LAI acceptance were satisfaction with current oral therapy (35.2%) and increased visit frequency (28.4%, Figure 1). During the observation period, 10 (7.9%) discontinued LAI (Table 1) and median maintenance duration was 98 days (IQR 31–213). Injection site reaction (*n* = 3) was most common reason of discontinuation followed by visit frequency burden/relocation (*n* = 2, respectively). Virologic failure was not observed among the LAI cohort.

**Conclusions**: This study reveals a relatively low level of acceptance for switching to LAI ART, primarily reflecting high satisfaction with current oral regimens. Although LAI ART alleviates privacy concerns and pill fatigue, the need for frequent hospital visits is a significant barrier. Although a fraction of patients discontinued LAI therapy, most remained on treatment and no cases of virological failure were observed during the 1‐year period after LAI introduction.

**FIGURE 1:**
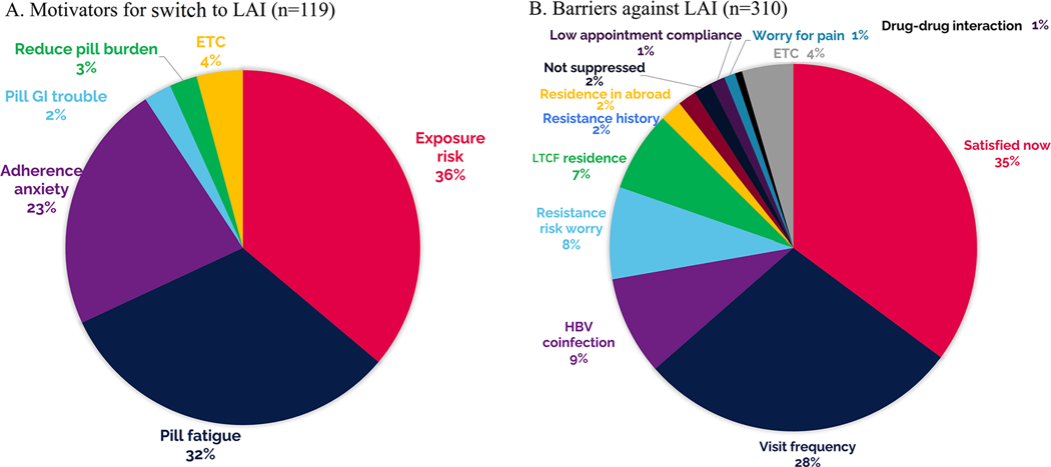
P148 | Motivators and barriers for the long‐acting injectable acceptance.

**TABLE 1:**
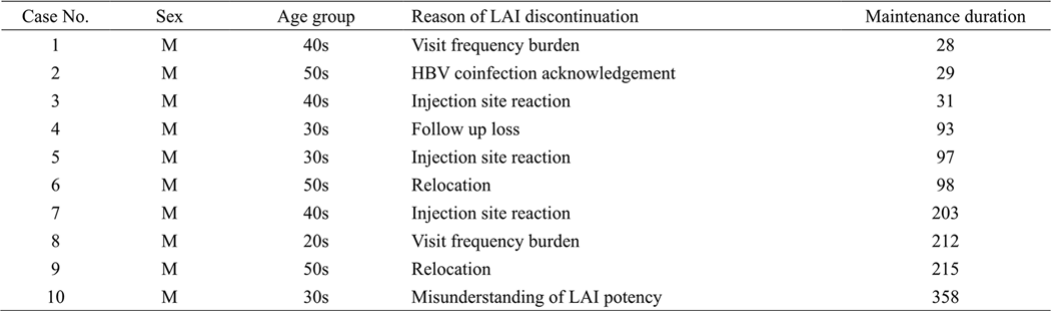
P148 | Characteristics of LAI discontinuation cases.

### P149 Clinical Context and Early Outcomes of Switching the ART Regimen of Migrant People With HIV to B/F/TAF: The ASAP‐Switch Study

David Lessard ^1^, Serge Vicente^2^, Kim Engler^1^, Joel Ishak^1^, Moustafa Laymouna^3^, Kathy Nodzynski^4^, Valérie Martel‐Laferrière^5^, Jean‐Pierre Routy^4^, Sean Yaphe^4,6^, Alexandra De Pokomandy^4^, Bertrand Lebouché^1,7^

^1^Research Institute of the McGill University Health Centre, Montreal, Canada; ^2^Département des Enseignements Généraux, École de Technologie Supérieure, Montreal, Canada; ^3^Department of Family Medicine, McGill University, Montreal, Canada; ^4^Chronic Viral Illness Service, McGill University Health Centre, Montreal, Canada; ^5^Centre de Recherche du Centre Hospitalier de l'Université de Montréal, Montreal, Canada; ^6^Clinique Médicale L'Actuel, Montreal, Canada; ^7^Chronic Viral Illness Service, McGill University Health Centre, Montreal, Canada

**Background**: Many migrants with HIV arrive in Canada on antiretroviral therapy (ART) regimens that cannot be renewed locally (e.g. unavailable, not covered), requiring ART‐regimen switches. Unknown ART histories limit the assessment of prior treatment failure and resistance risk. Tuberculosis treatment, a concern in this population, may further complicate ART selection because of drug–drug interactions. We describe the clinical context and early outcomes of switching ART for migrants with HIV to bictegravir/emtricitabine/tenofovir alafenamide (B/F/TAF), which has a high resistance barrier and no virological restrictions.

**Methods**: Adults with HIV born abroad and settled in the province of Quebec for < 2 years were offered a switch to B/F/TAF at three clinics in Montreal, Canada. Missing clinical information at baseline (VL, CD4‐cell count, ART history and tuberculosis status) is described. Viral suppression (≤50 copies/mL) and CD4‐cell counts at baseline and week 4 were compared between participants with known and unknown ART histories using exact unconditional multinomial (VL) and two‐sample *t*‐tests (CD4).

**Results**: Among 74 participants who switched to B/F/TAF, 30 (40.5%) were women and 48 (64.9%) originated from sub‐Saharan Africa. Mean age ± standard deviation (SD) was 41.8 ± 11.1 years (range 23–67). Missing baseline information included unknown VL (26, 35.1%), CD4 count (30, 40.5%), ART history (30, 40.5%) and tuberculosis status (34, 45.9%). Twelve (16.2%) participants were diagnosed with latent tuberculosis; 4/12 were diagnosed after switching; 10 of them took a 9‐month isoniazid‐based treatment and remained on B/F/TAF, while two preferred a 3‐month rifampicin‐based treatment, which required them to temporarily take another ART regimen. When comparing participants with known and unknown ART histories, the groups did not differ significantly by viral suppression (9/27, 33.3% had an undetectable VL vs. 7/43, 16.3%) and mean CD4‐cell count ± SD (599.4 ± 264.8 vs. 651.9 ± 274.6 cells/mm^3^) and divergences diminished by week 4 (detectable VL: 6/23, 26.1% vs. 6/37, 16.2%; CD4 count: 649.1 ± 278.7 vs. 637.0 ± 217.0 cells/mm^3^). No virologic failure occurred among participants virologically suppressed at baseline.

**Conclusions**: With few exceptions, B/F/TAF can be readily offered to recent ART‐experienced migrants despite gaps in medical history. These findings support ART switching alongside tuberculosis screening and use of tuberculosis treatments compatible with the selected ART regimen.

### P150 Patient Perceptions of Real‐World Use of CAB+RPV LA Therapy in Canada

Arthi Rudak ^1^, Jean‐Francois Fortin^2^, Jonathan Smith^2^, Mistre Alemayehu^3^, Maria Esther Perez Trejo^4^, Dorothée Chopamba^5^, Mona Loufty^6^

^1^Health Economics and Outcomes Research, GlaxoSmithKline Inc., Mississauga, Canada; ^2^Medical Affairs, ViiV Healthcare Canada, Montreal, Canada; ^3^IQVIA Solutions Canada Inc., Mississauga, Canada; ^4^IQVIA Solutions Canada Inc., Montreal, Canada; ^5^Manera Counselling Services, Toronto, Canada; ^6^Research and Innovation Institute, Women's College Hospital, University of Toronto & Maple Leaf Medical Clinic, Toronto, Canada

**Background**: Adherence to antiretroviral therapy (ART) is critical for viral suppression and improved outcomes among people living with HIV. Long‐acting injectable cabotegravir and rilpivirine (CAB+RPV LA) may address challenges with daily oral therapy and improve health‐related quality of life; however, real‐world data on patient perceptions with CAB+RPV LA in Canada are limited.

**Methods**: In this observational study, adults living with HIV in Canada prescribed (*n* = 30 [prospective]) or recently initiated (*n* = 60 [retrospective]) CAB+RPV LA completed online baseline surveys, with ongoing month 4 data collection and months 8 and 12 planned. Surveys assessed demographics, clinical characteristics, treatment perceptions, experiences and satisfaction using the HIV Treatment Satisfaction Questionnaire (HIVTSQ). This interim descriptive analysis presents baseline (*n* = 90) and month 4 (*n* = 43) data.

**Results**: The study enrolled 90 patients, predominantly cisgender men (84.4%, *n* = 76) with mean age of 46 years (SD 11.5; median 45 [IQR 35–55]) and mean HIV duration of 14 years (SD 9.9; median 12 [IQR 7–20]); most (90.8%, *n* = 79) were previously on a single tablet regimen. At month 4, patients on CAB+RPV LA reported substantial improvements in perception outcomes versus baseline oral therapy: 95.3% (*n* = 41) “never/rarely” experienced stigma or discrimination (vs. 72.3% [*n* = 60] on oral therapy) and 88.4% (*n* = 38) “never/rarely” worried about disclosure (vs. 39.8% [*n* = 33] on oral therapy) (Figure 1). Adherence was high, with 100% (*n* = 43) receiving their scheduled injection.

The proportion who “completely agree” with the appropriateness of CAB+RPV LA injections increased from 62.1% (*n* = 54) at baseline to 81.4% (*n* = 35) at month 4; “completely agree” responses for appeal rose from 55.8% (*n* = 48) to 67.4% (*n* = 29). HIVTSQ satisfaction scores were higher at month 4 (mean 60.4, SD 6.8) than baseline (mean 54.1, SD 12.1). All HIVTSQ items except item 3 (side effects satisfaction) and item 12 (discomfort/pain satisfaction) favoured CAB+RPV LA over oral therapy (Table 1).

**Conclusions**: Among people living with HIV, 4 months of CAB+RPV LA treatment was associated with high adherence, improved acceptability over time and greater satisfaction compared to oral therapy. Patient‐reported outcomes improved across multiple domains, including stigma and discrimination, concerns about disclosure, convenience and willingness to continue treatment. These preliminary findings support favourable real‐world patient experiences with long‐acting injectable therapy.

**FIGURE 1:**
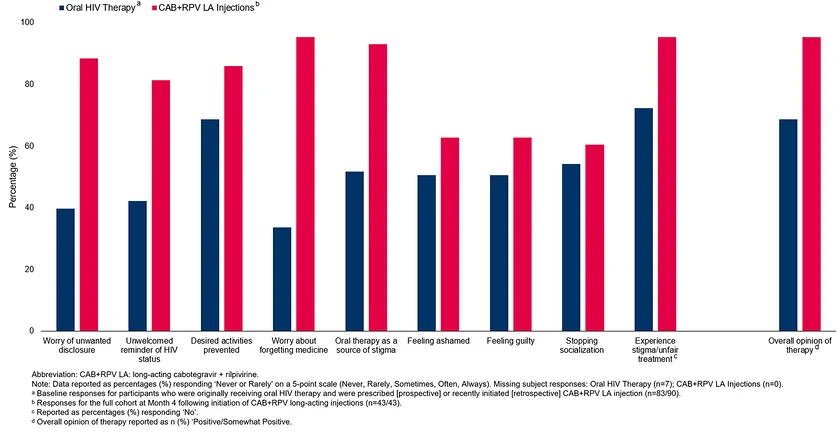
P150 | Patient perceptions of oral HIV therapy compared with CAB+RPV LA injections.

**TABLE 1:**
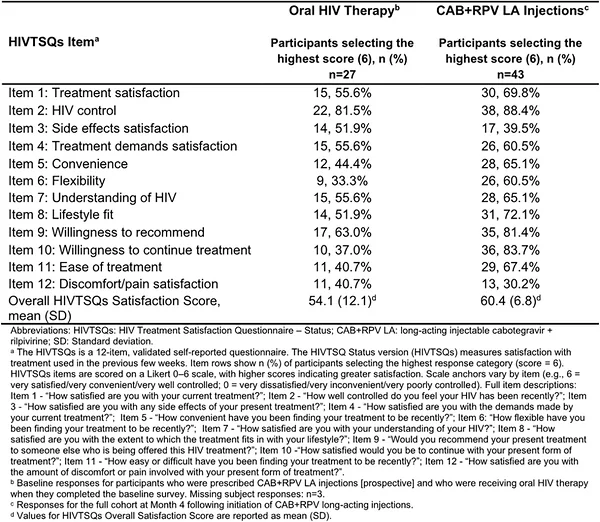
P150 | Comparison of HIVTSQs scores for oral HIV treatment at baseline and CAB+RPV long‐acting injections at month 4.

## Treatment Strategies: Treatment Experienced Adults (Second‐Line and Multi‐Drug Resistance Studies)

### P151 Discordance Between Historical Viral and Proviral Resistance Testing and Its Impact on B/F/TAF Genotypic Susceptibility Scores in the BRACE Study

Eva Wolf ^1^, Martin Daeumer^2^, Markus Bickel^3^, Sebastian Noe^4^, Christoph Wyen^5^, Christoph Stephan^6^, Neel Petersen^7^, Stephan Grunwald^8^, Patrick Braun^9^, Sandra Schreiber^10^, Stefan Esser^11^, Katja Roemer^12^, Claudia Pehlivan^10^, Olaf Degen^13^, Alexander Thielen^2^, Annamaria Balogh^1^, Christian Hoffmann^14^

^1^Eurofins MUC Research, Munich, Germany; ^2^IIG, Institute of Immunology and Genetics, Kaiserslautern, Germany; ^3^Infektio Research, Frankfurt, Germany; ^4^MVZ CARE, Munich, Germany; ^5^Praxis am Ebertplatz, Cologne, Germany; ^6^University Hospital Frankfurt, Frankfurt, Germany; ^7^University Hospital Schleswig‐Holstein, Kiel, Germany; ^8^zibp—Center for Infectiology Berlin Prenzlauer Berg, Berlin, Germany; ^9^PZB Aachen, Aachen, Germany; ^10^Gilead Sciences, Martinsried/Munich, Germany; ^11^University Hospital Essen, Essen, Germany; ^12^Gemeinschaftspraxis Gotenring, Cologne, Germany; ^13^Infectious Diseases Unit, University Medical Centre Hamburg‐Eppendorf, Hamburg, Germany; ^14^ICH Study Center, Hamburg, Germany

**Background**: BRACE is a German retrospective cohort study of people with HIV (PWH) who initiated B/F/TAF (bictegravir/emtricitabine/tenofovir alafenamide) despite a history of major resistance‐associated mutations (RAMs) relevant to ≥1 component of B/F/TAF and detected by genotypic resistance testing of viral RNA. Despite pre‐existing NRTI and/or INSTI RAMs, viral suppression rates were consistently high in this cohort, irrespective of the number of active drug components [1]. We compared cumulative historical RAMs prior to B/F/TAF with results from single time‐point proviral DNA testing performed at study inclusion.

**Methods**: This analysis assessed the re‐detection of historical viral RAMs in proviral DNA (using a 1% cut‐off), as well as the identification of previously undetected RAMs. We compared the B/F/TAF genotypic susceptibility scores (GSS) derived from viral versus proviral results, applying APOBEC‐mediated hypermutation filtering and evaluated the impact of this filtering on resistance interpretation. Only RAMs listed in the Stanford HIVdb output tables (v10.1, 2026‐01‐18) were considered.

**Results**: Paired historical viral and proviral data were available for 147 of 156 participants. The average re‐detection rates at the individual level were 45% for NRTI RAMs (among PWH with previously detected mutations, *n* = 147), 42% for NNRTI RAMs (*n* = 103), 43% for PI RAMs (*n* = 50) and 35% for INSTI RAMs (*n* = 11). Previously undetected RAMs (major, minor or accessory) were identified in 51 (35%), 49 (33%), 20 (14%) and 49 (33%) cases for NRTI, NNRTI, PI and INSTI, respectively; the comparatively high rate of newly detected INSTI RAMs partly reflects the frequent absence of prior integrase genotyping. In 19/147 (13%) participants, neither re‐detected nor newly detected proviral RAMs were observed. The shift in the B/F/TAF GSS based on viral RAMs versus proviral RAMs (after APOBEC filtering) is shown in Figure 1. APOBEC filtering altered resistance interpretation, increasing the B/F/TAF GSS in 19 cases (13%, median change +1.0; IQR +0.25–+1.0, max +1.75).

**Conclusions**: Proviral DNA testing incompletely captured historical viral RAMs, identified previously undetected mutations and resulted in shifts in the B/F/TAF GSS. APOBEC‐associated mutations contributed to these differences in 13% of cases, underscoring the need for careful interpretation of proviral sequences.

**Reference**

1. Wolf E, Bickel M, Wyen C, et al. 26^th^ Int. AIDS Conference; 2026 Jul 26–31; Rio de Janeiro, Brazil. Abstract THPEB129.

**FIGURE 1:**
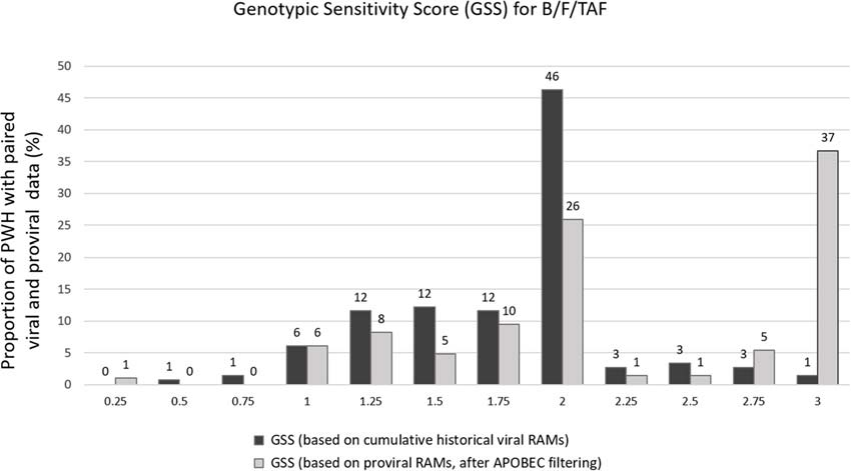
P151 | Distribution of cumulative viral versus proviral GSS for B/F/TAF.

### P152 Legacy ART in 2024−2025: Clinical Necessity, Individual Vulnerability or Prescribing Inertia?

Alessandra Vergori ^1^, Alessandro Tavelli^2,3^, Andrea Giacomelli^4,5^, Stefano Rusconi^5,6^, Silvia Nozza^7^, Valentina Mazzotta^8^, Giuseppe Lapadula^9^, Stefano Bonora^10^, Giordano Madeddu^11^, Roberta Gagliardini^8^, Andrea Antinori^8^, Antonella D'Arminio Monforte^2^, Sergio Lo Caputo^1^, on behalf of ICONA Cohort Study Group

^1^Infectious Diseases Unit, Department of Clinical and Surgical Sciences, University of Foggia, Foggia, Italy; ^2^Icona Foundation, Milan, Italy; ^3^National PhD Programme in One Health approaches to infectious diseases and life science research, Department of Public Health, Experimental and Forensic Medicine, University of Pavia, Pavia, Italy; ^4^III Infectious Diseases Unit, ASST Fatebenefratelli‐Sacco, Milan, Italy; ^5^DIBIC, Università degli Studi di Milano, Milan, Italy; ^6^Infectious Diseases Unit, Ospedale Civile di Legnano, ASST Ovest Milanese, Milan, Italy; ^7^Department of Infectious Diseases, IRCCS San Raffaele Scientific Institute, Vita‐Salute San Raffaele University, Milan, Italy; ^8^Clinical and Research Infectious Diseases Department, National Institute for Infectious Diseases, Lazzaro Spallanzani IRCCS, Rome, Italy; ^9^Department of Infectious Diseases, IRCCS San Gerardo dei Tintori, University of Milano‐Bicocca, Monza, Italy; ^10^Unit of Infectious Diseases, Department of Medical Sciences, University of Turin, Amedeo di Savoia Hospital, Turin, Italy; ^11^Unit of Infectious Disease, Department of Medicine, Surgery and Pharmacy, University of Sassari, Sassari, Italy

**Background**: Despite the widespread adoption of INSTI‐based antiretroviral therapy (ART), a subset of people with HIV (PWH) are receiving regimens far from current treatment standards. We aimed to assess the prevalence of outdated ART regimens, characterize PWH who are treated with them and identify person‐ and centre‐level factors associated with their use.

**Methods**: Retrospective, cross‐sectional study including PWH from ICONA cohort with at least one visit between January 2024 and October 2025. ART regimens were classified as recommended regimens (RecR), outdated regimens (OutR) and complex regimens (CoR, multi‐tablet or ‐class). OutR were further divided into true OutR and acceptable/alternative regimens (AltR) for description. Mixed‐effects logistic regression models with centre‐specific random intercept were used to identify individual‐ and organization‐level factors associated with OutR use. Residual between‐centre heterogeneity was quantified using the median odds ratio (MOR).

**Results**: Among 12,318 PWH under follow‐up, 11,444 were included. Overall, 10,694 (93.5%) were receiving RecR, 176 (1.5%) CoR and 574 (5.0%) OutR. Of these, 299 (2.6%) were true OutR and 275 (2.4%) AltR. Compared with PWH receiving RecR, those on OutR were older (median age 54.0 vs. 49.0 years), more frequently female (29.8% vs. 18.7%), less frequently MSM (32.1% vs. 47.7%) and more often unemployed (16.0% vs. 10.2%) (Table 1). ABC‐based regimens accounted for 42% of all OutR prescriptions, followed by TAF/FTC plus raltegravir (12%) and monotherapy with DRV/cobicistat (5%) (full list of ReC, OutR in Figure 1A‐C). In multivariable analyses, female sex (aOR 1.27, 95% CI: 1.02–1.60), unemployment (1.55, 1.21–2.00), longer ART duration (1.07 per year, 1.05–1.08) and higher total cholesterol levels (1.03 per 10 mg/dL, 1.01–1.05) were independently associated with OutR use (Figure 1D). No association was observed between OutR use and centre size or HIV care model (Figure 1E). However, residual heterogeneity between‐centre on OutR use remained significant (MOR 1.85, 95% CI: 1.56–2.34).

**Conclusions**: In the INSTI era, outdated ART regimens account for a small yet persistent proportion of prescriptions. Their use appears to be associated with social vulnerability, treatment history and long‐term ART exposure rather than with measurable organizational characteristics of HIV centres. Nonetheless, the marked variability across centres suggests that local prescribing practices and other unmeasured factors continue to influence ART choice.

**FIGURE 1:**
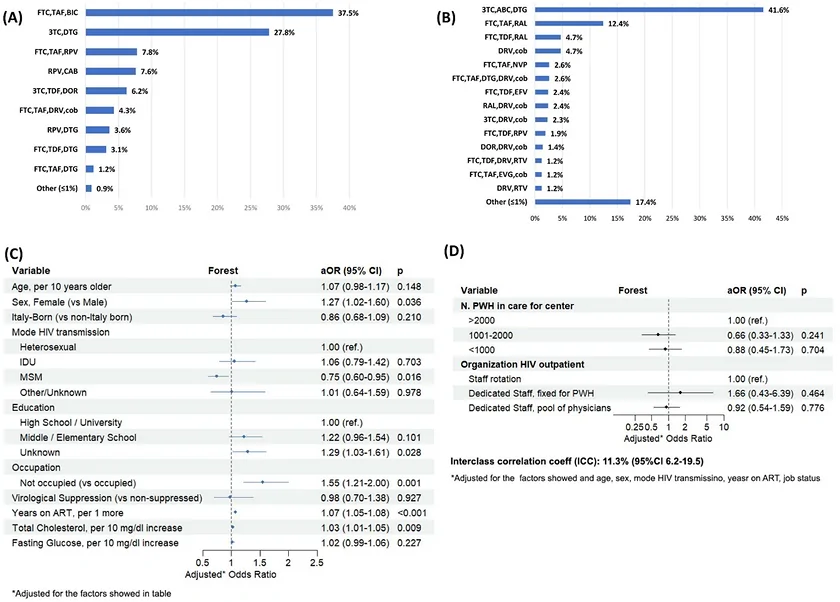
P152 | Distribution of current ART regimens in recommended (RecR) (A), outdated regimens (OutR) (B), individual‐level (C) and centre‐level (D) factors associated with OutR use by mixed‐effects logistic regression models.

**TABLE 1:**
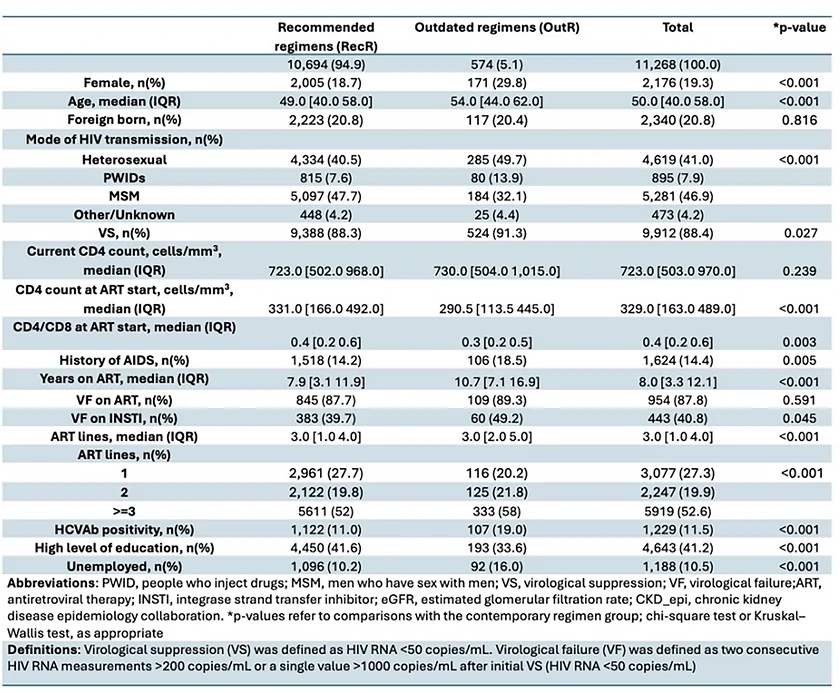
P152 | General characteristics of study population.

### P153 Eligibility for Islatravir‐Containing Regimens in People Living With HIV‐1 Experiencing Virological Failure Under Combined Antiretroviral Therapy: Data From the ARCA Cohort

Marianna Menozzi ^1^, Valeria Micheli^2^, Omar El Khalili^3^, Antonia Bezenchek^4,5^, Valentina Ricucci^6^, Bianca Bruzzone^6^, William Gennari^7^, Ilaria Vicenti^8,9^, Cristina Mussini^1,10^, Franco Maggiolo^11^, Andrea De Vito^12^, Francesca Ceccherini‐Silberstein^3^, Barbara Rossetti^13^, Maria Mercedes Santoro^3^, Daniele Armenia^9^

^1^Clinic of Infectious Diseases, University Hospital of Modena, Modena, Italy; ^2^Clinical Microbiology, Virology and Bioemergencies, ASST Fatebenefratelli Sacco, Luigi Sacco Hospital, Milan, Italy; ^3^Department of Experimental Medicine, University of Rome “Tor Vergata”, Rome, Italy; ^4^IPRO‐InformaPRO S.r.l., Rome, Italy; ^5^EuResist Network GEIE, Rome, Italy; ^6^Hygiene Unit, IRCCS—Policlinico San Martino Hospital, Genoa, Italy; ^7^Clinical Microbiology Unit, University Hospital of Modena, Modena, Italy; ^8^Department of Medical Biotechnologies, University of Siena, Siena, Italy; ^9^Saint Camillus International University of Health Sciences, Rome, Italy; ^10^University of Modena and Reggio Emilia, Modena, Italy; ^11^Unit of HIV‐related Diseases and Experimental Therapies, Azienda Ospedaliera Papa Giovanni XXIII, Bergamo, Italy; ^12^Unit of Infectious Diseases, Department of Medicine, Surgery and Pharmacy, University of Sassari, Sassari, Italy; ^13^Infectious Diseases Unit, Sud‐Est Toscana, Ospedale Della Misericordia, Grosseto, Italy

**Background**: Islatravir (ISL) has shown promising results in clinical trials involving virologically suppressed people living with HIV‐1 (PWH). However, its activity in individuals experiencing virological failure has not been investigated.

**Methods**: PWH experiencing virological failure over 2020–2025 with a subsequent plasma or PBMCs genotypic resistance test (GRT) were included from the ARCA database. ISL activity was evaluated according to the IAS 2026 list and Stanford HIVdb v10.2 as follows: (i) no ISL resistance‐associated mutation (RAM); (ii) no M184V plus ≥1 thymidine analogue mutation (TAM); (iii) HIVdb resistance level < 3. Doravirine (DOR) activity was also assessed. For PWH with previous GRTs, cumulative resistance was evaluated. Logistic regression models were built to identify predictors of ISL activity.

**Results**: Overall, 272 failing PWH were included (median [IQR] viraemia 583 [148–15,079] copies/mL); 230 had ≥1 previous GRT. At failure, 74.6% were receiving triple therapy, 69.1% had a high genetic barrier drug and 85.3% an NRTI backbone, for a median of 2.5 (1.0–4.5) years (Table 1). Cumulative GRTs were more informative than failure GRTs, with absent ISL‐RAMs in 54.8% of individuals compared with 68.4% at failure. PWH with longer history, lower CD4+ nadir and higher viraemia zenith showed higher ISL‐resistance, whereas those receiving high genetic barrier drugs showed less resistance (Table 1). Among ISL‐specific mutations, only I142V was detected; M184V combined with 1–2 TAMs was frequent, especially cumulatively (Figure 1). ISL remained active in > 66% of cumulative GRTs, DOR in > 85% regardless of the analysis and the ISL+DOR combination in 64.7% of individuals (Figure 1). In multivariable analysis, an increased odds ratio of ISL‐susceptibility was associated with high genetic barrier‐based regimens according to failure GRTs (aOR [95% CI] 2.52 [1.17–5.42], *p* = 0.018) and with a cART initiation after 2014 by cumulative GRTs (6.81 [1.13–40.97], *p* = 0.036). An increasing number of previous regimens was associated with reduced ISL susceptibility, both at failure (0.91 [0.82–1.00], *p* = 0.043) and at cumulative GRTs (0.87 [0.78–0.98], *p* = 0.022).

**Conclusions**: ISL was predicted to be fully active in over 65% of individuals recently failing cART. Use of high genetic barrier drugs appeared protective, but review of historical resistance data remains crucial to identify PWH eligible for ISL treatment.

**FIGURE 1:**
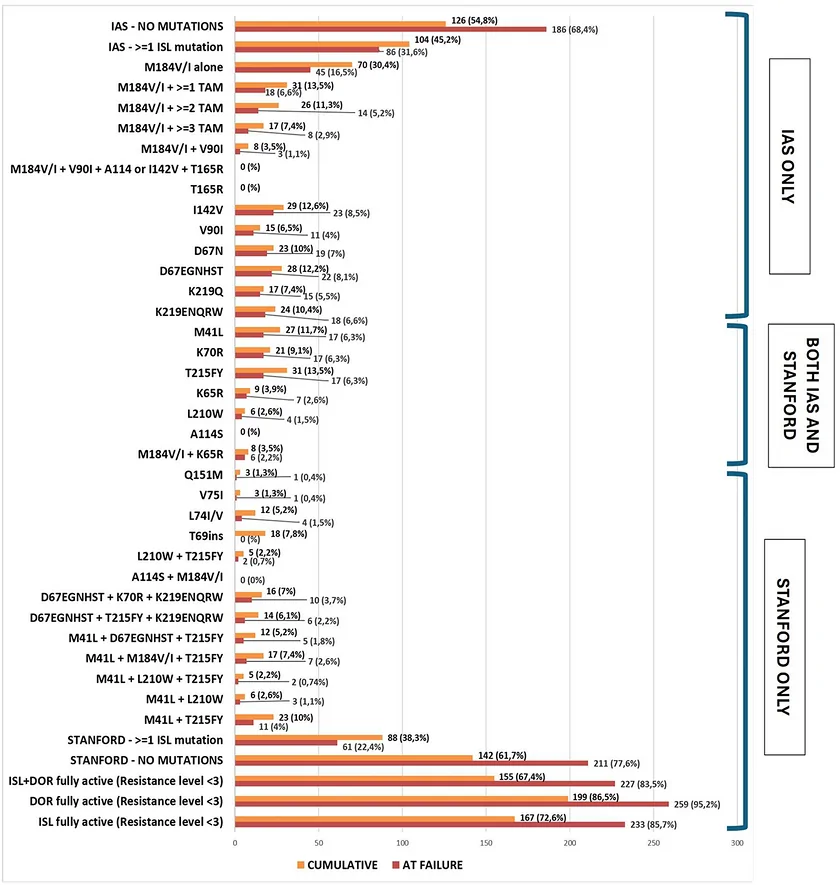
P153 | ISL RAMs distribution according to GRT at failure and cumulative GRT.

**TABLE 1:**
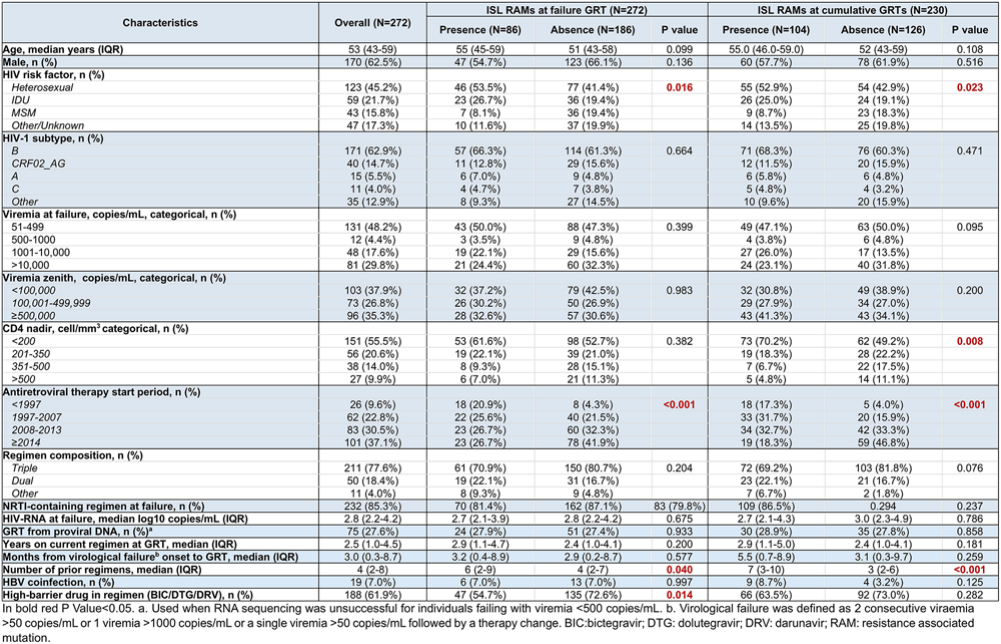
P153 | Characteristics of individuals failing cART in 2020–2025 period according with the presence/absence of ISL resistance.

### P154 Metabolic and Liver Outcomes Through Week 48 Following Switch to Dolutegravir/Lamivudine in Older, ART‐Experienced Adults: The Phase 3b EYEWITNESS Study

Moti Ramgopal^1^, Josefina Méndez^2^, Laurence Slama^3,4^, Manuel Crespo Casal^5^, Gulia C. Marchetti^6^, Bliss Leverette^7^, Kehui Wang^8^, Alex Pitchford^8^, Richard Grove^8^, Riya Moodley^9^, Emilio Letang^10^, Ana Milinkovic^9^, Jean van Wyk^9^, Piotr Budnik ^9^, Parul Patel^7^

^1^Midway Immunology and Research Centre, Fort Pierce, FL, US; ^2^Serviço de Doenças Infecciosas, Unidade Local de Saúde de Santo António, Porto, Portugal; ^3^Department of Infectious Diseases, Hôtel‐Dieu Hospital, University Paris Cité, Paris, France; ^4^Paris Cité and Université Sorbonne Paris Nord, Inserm, INRAE, Center for Research in Epidemiology and Statistics (CRESS), Paris, France; ^5^Infectious Diseases Department, Hospital Universitari de Bellvitge, Barcelona, Spain; ^6^Clinic of Infectious Diseases and Tropical Medicine, San Paolo Hospital, ASST Santi Paolo e Carlo, Department of Health Sciences, University of Milan, Milan, Italy; ^7^ViiV Healthcare, Durham, NC, US; ^8^GSK, London, UK; ^9^ViiV Healthcare, London, UK; ^10^ViiV Healthcare, Tres Cantos, Spain

**Background**: Weight and metabolic effects associated with integrase strand transfer inhibitor‐based and tenofovir alafenamide‐based regimens have been described in people naïve to treatment and are beginning to be described for liver outcomes in people with prior ART experience. However, metabolic and liver outcomes in adults ageing with HIV with increased cardiometabolic risk undergoing ART optimization remain less characterized. In ageing, virologically suppressed, ART‐experienced people, metabolic and hepatic changes were evaluated following switch to dolutegravir/lamivudine (DTG/3TC).

**Methods**: EYEWITNESS is a phase 3b, multicentre, single‑arm study evaluating virologically suppressed adults living with HIV aged ≥50 years with significant cardiometabolic comorbidity switching from bictegravir/emtricitabine/tenofovir alafenamide (BIC/FTC/TAF) to DTG/3TC. Weight change was evaluated longitudinally and prespecified exploratory outcomes assessed changes in body composition and liver outcomes at baseline (BL) and week 48 (W48). Body composition was assessed by dual‑energy X‑ray absorptiometry (DXA) and hepatic steatosis and fibrosis were evaluated using transient elastography, including controlled attenuation parameter (CAP) and liver stiffness measurement (LSM). Liver analyses were restricted to participants with paired BL and W48 assessments.

**Results**: Among 205 participants, median age was 61 years, 42% were female, 37% were non‑White, 44% had ≥20% 10‐year CVD risk (Framingham) and median (interquartile range, IQR) weight was 77.2 kg (IQR, 69, 90.7), with 37% classified as overweight and 30% as obese at BL. At W48, median (IQR) weight change was −0.20 kg (IQR, −2.70, 2.20), with 2% (*n* = 4/179) experiencing > 10% weight gain. Changes in total and regional fat (trunk and limb) and lean mass were minimal via DXA. Distributions of steatosis (CAP) and fibrosis (LSM) were similar at BL and W48, with most participants remaining within the same or adjacent categories (Table 1).

**Conclusions**: In ageing, virologically suppressed people living with HIV with substantial cardiometabolic burden, switching from BIC/FTC/TAF to DTG/3TC was associated with minimal changes in weight and body composition over 48 weeks. Distributions of steatosis and fibrosis at baseline and W48 were similar with no consistent directional shift observed. These findings provide clinically relevant context for interpreting metabolic and liver outcomes following ART modification in ageing, higher‐risk populations.

**TABLE 1:**
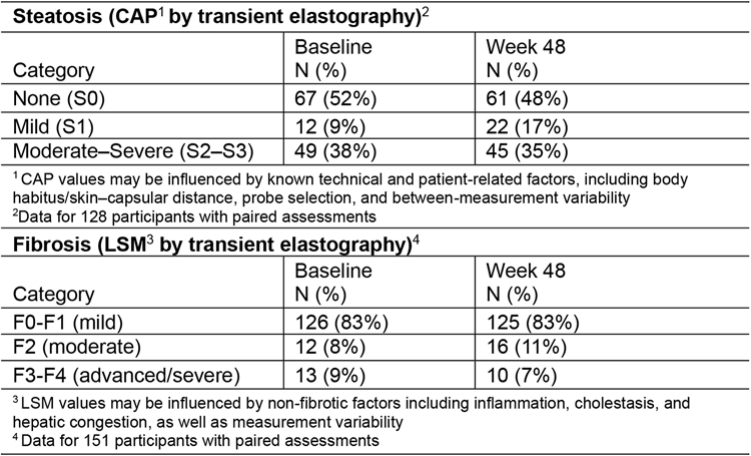
P154 | Steatosis (CAP) and fibrosis (LSM) by transient elastography at baseline and week 48 (paired assessments).

### P155 Outcomes of Doravirine (DOR) Based Regimens in Virologically Suppressed ART‐Experienced Patients: Data From the ICONA Cohort

Alessandro Tavelli ^1,2^, Antonella D'Arminio Monforte^1^, Ilaria Mastrorosa^3^, Roberta Gagliardini^3^, Lucia Taramasso^4^, Nicholas Brian Bana^5^, Nicola Squillace^6^, Teresa Bini^7^, Daniela Segala^8,9^, Eugenia Quiros‐Roldan^10^, Sergio Lo Caputo^11^, Annalisa Saracino^12,13^, Andrea Antinori^3^, on behalf of ICONA Cohort Study Group

^1^ICONA Foundation, Milan, Italy; ^2^National PhD Programme in One Health approaches to infectious diseases and life science research, Department of Public Health, Experimental and Forensic Medicine, Pavia, Italy; ^3^Clinical and Research Infectious Diseases Department, National Institute for Infectious Diseases, Lazzaro Spallanzani IRCCS, Rome, Italy; ^4^Infectious Disease Clinic, IRCCS Ospedale Policlinico San Martino, Genova, Italy; ^5^Department of Infectious Diseases, ASST Grande Ospedale Metropolitano Niguarda, Milan, Italy; ^6^Department of Infectious Diseases, IRCCS San Gerardo dei Tintori, Monza, Italy; ^7^Clinic of Infectious Diseases, ASST Santi Paolo e Carlo, Milan, Italy; ^8^UO Malattie Infettive Territoriali, AUSL Ferrara, Ferrara, Italy; ^9^Department of Translational Medicine and for Romagna, University of Ferrara, Ferrara, Italy; ^10^Department of Clinical and Experimental Sciences, Unit of Infectious and Tropical Diseases, University of Brescia and ASST Spedali Civili di Brescia, Brescia, Italy; ^11^Clinic of Infectious Diseases, Department of Clinical and Surgical Sciences, University of Foggia, Foggia, Italy; ^12^Clinic of Infectious Diseases, Polyclinic of Bari, University Hospital Polyclinic, Bari, Italy; ^13^Department of Precision and Regenerative Medicine and Ionian Area, University of Bari, Bari, Italy

**Background**: We aim to verify in a real‐world setting the outcomes of DOR‐based 3‐drug regimens (3DR) in virologically suppressed people with HIV (PWH).

**Methods**: We included PWH enrolled in ICONA who switched for the first time while virologically suppressed (HIV‐RNA ≤50 cps/mL) from any other regimen to 3DR‐DOR‐based regimen. Primary endpoint, time to treatment failure (TF): virological failure (2 consecutive HIV‐RNA > 50 copies/mL or single > 1000 copies/mL followed by ART change) or DOR discontinuation for toxicity/failure. Secondary endpoints: time to DOR discontinuation for any reason (TD); for toxicity (TDT); time to virological failure (VF); reasons for DOR discontinuation; changes in plasma lipids. Cox regression models were used to evaluate the risk of time to TF, TD, TDT and VF according to selected exposure of interest: age ≥65 years, female sex, Italy‐born, previous regimen NNRTI‐based, CD4< 200 cells/cmm at DOR‐switch and at ART‐initiation. Linear mixed models were used to determine mean lipid changes from DOR switch.

**Results**: A total of 731 PWH included (Table 1). In a median follow‐up of 160 (IQR 89–226) weeks, 51/731 (6.9%) PWH experienced TF (18 VF, 4 TD for failure and 29 TDT). Previous regimens other than NNRTI and marginally CD4< 200 cells/cmm at DOR initiation were predictive of TF (Figure 1A). 18 PWH experienced VF (2.5%): PWH with CD4 counts < 200/cmm at DOR initiation was associated with a higher risk of VF (Figure 1A). 120 PWH discontinued 3DR‐DOR; main causes of TD were simplification (49; 6.7%), other (33; 4.5%), toxicity (29; 4.0%), failure (9; 1.2%). Gastro‐intestinal intolerance and allergic reactions were the main causes of TDT (24% and 13.7% of TDT). Females and PWH with a pre‐DOR regimen other than NNRTI showed a higher risk of TD; females had also higher risk of TDT (Figure 1A). Mean plasma levels of lipids (cholesterol, HDL, LDL, triglycerides) improved after DOR switch (Figure 1B).

**Conclusions**: Switching to 3DR DOR while undetectable is a successful strategy resulting only in 6.4% of treatment failure. The high tolerability along with potency confirms that the regimen is a good option, also for PWH with fragility such as those of advanced age and migrants. The safe profile of DOR on lipid asset was also confirmed.

**FIGURE 1:**
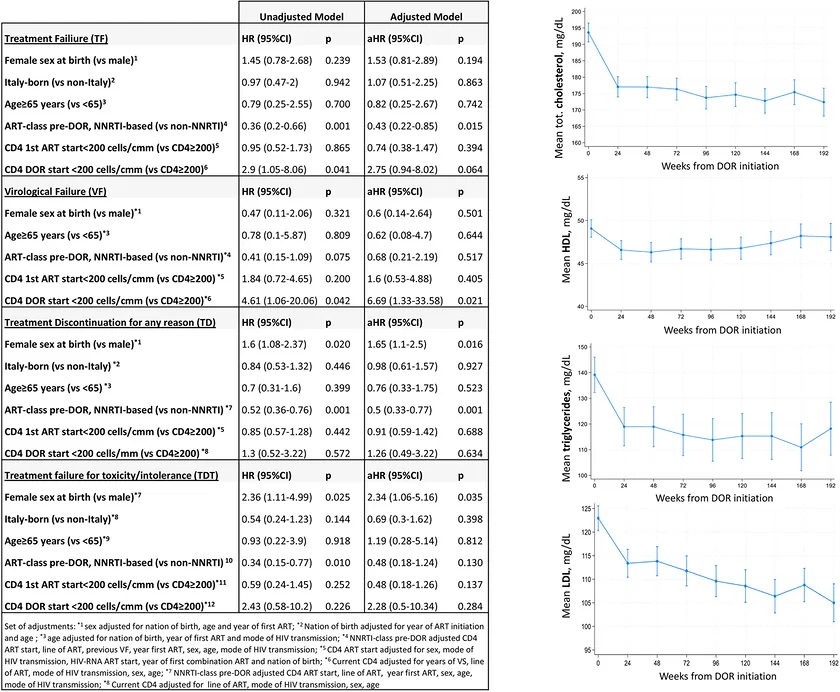
P155 | (A) Unadjusted and adjusted hazard ratios (HR) of TF, VF, TD and TDT by Cox regression model; (B) mean total cholesterol, HDL, LDL and triglycerides after switch to DOR by linear mixed models.

**TABLE 1:**
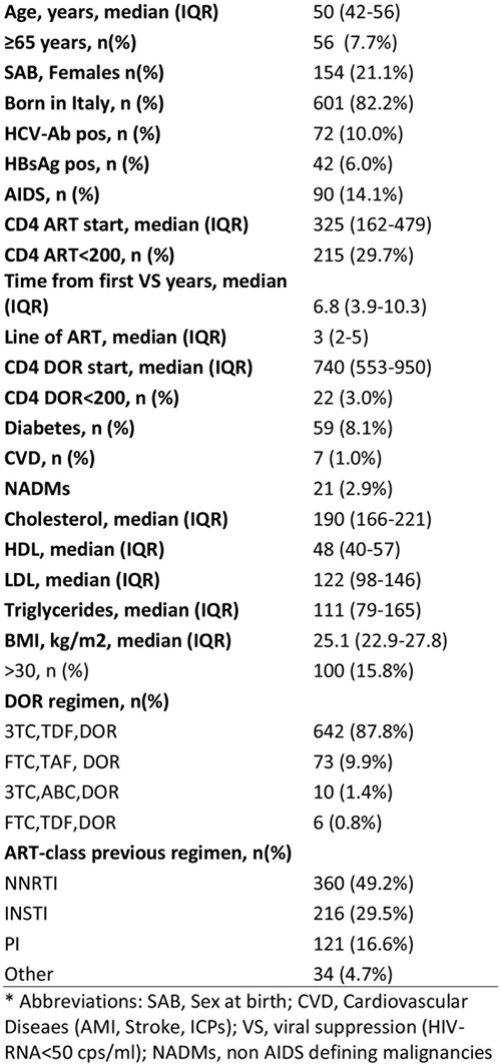
P155 | Characteristics of 731 PWH switching to 3DR DOR‐based.

### P156 Transmitted and Acquired Drug Resistance in Migrants Living With HIV Initiating Care: Prevalence, Patterns and Clinical Implications

Lara Neto, Cristina Soeiro, Ricardo Correia de Abreu, Frederico Duarte

Infectious Diseases Department, Local Health Unit of Matosinhos, Matosinhos, Portugal

**Background**: As migration increases, so does the complexity of HIV care, particularly regarding drug resistance. Migrants living with HIV (MLWH) may carry transmitted drug resistance (TDR) at diagnosis or harbour acquired resistance following fragmented or interrupted antiretroviral treatment (ART) in their countries of origin. We evaluated resistance patterns and their clinical impact in a cohort of MLWH followed in Portugal.

**Methods**: Retrospective analysis of 199 MLWH (between October 2020 and March 2026). Genotypic resistance testing (GRT) was performed in all ART‐naïve patients (naïve group) and in ART‐experienced patients (experienced group) with detectable HIV‐1 RNA. Mutations were classified per the Stanford HIV Drug Resistance Database (v9). Virological outcomes were compared using Fisher's exact test.

**Results**: GRT was requested in all 71 naïve patients (100%), with results available in 57/71 (80.3%) at the time of analysis and 14 (19.7%) still pending. GRT was performed in 22/128 (17.2%) HIV‐experienced patients. Clinically relevant TDR was detected in 21/71 naïve patients (29.6%), well above estimates for the general Portuguese HIV population (∼10%–15%). NNRTI mutations predominated, followed by NRTI and integrase mutations. Two cases of complex multidrug TDR required boosted darunavir‐based regimens. In the experienced group, acquired resistance was identified in 11/22 tested patients (50.0%), including one case of triple‐class resistance (protease: I50L, L33F; NRTI: D67N, K70R, K219E; NNRTI: K101H, E138Q, Y181C, G190A) and multiple cases of NNRTI resistance. Yet, despite this TDR burden, the predominant regimens, dolutegravir/lamivudine (DTG/3TC) and bictegravir/emtricitabine/tenofovir alafenamide (B/F/TAF), conferred high genetic barriers and virological suppression at 6 months exceeded 94% across both groups (*p* = 0.71).

**Conclusions**: A TDR prevalence of 29.6% in newly diagnosed MLWH is a clinically meaningful finding that should inform ART selection, particularly when considering lower‐barrier regimens. High‐barrier integrase inhibitor‐based regimens maintained excellent virological outcomes despite TDR, reinforcing their preferential use in MLWH and the value of universal GRT at diagnosis. Systematic resistance assessment at the point of care transfer remains essential for ART‐experienced migrants.

### P157 Evaluation of Fostemsavir Switch Therapy in Treatment Experienced Patients With HIV: Real‐World Insights

Alison Duncan ^1^, Kate Mackie^1^, James McMahon^2,3^, Rekha Pai Mangalore^2,3^

^1^Pharmacy Department, Alfred Care Group, Bayside Health, Melbourne, Australia; ^2^Department of Infectious Diseases, the Alfred, Bayside Health, Melbourne, Australia; ^3^Monash University, Melbourne, Australia

**Background**: Fostemsavir is a first‐in‐class HIV‐1 attachment inhibitor approved for heavily treatment‐experienced people with multidrug resistant HIV‐1 and limited treatment options. While trial data mainly focusses on virological failure, fostemsavir can also support regimen optimization in virologically suppressed patients with archived resistance, intolerance, co‐morbidity and drug−drug interactions (DDIs). Real‐world data describing switches to a fostemsavir‐containing regimen for indications beyond virological failure are limited.

**Methods**: A retrospective audit was conducted of patients switched to a fostemsavir‐containing regimen (July 2015–October 2025). Baseline demographics, treatment history, reasons for switch, pre‐ and post‐switch regimens, HIV RNA, CD4 count, adverse events and discontinuations were collected. The primary outcome was maintenance of virological suppression at 48 weeks or last follow‐up, defined as maintenance or achievement of HIV RNA < 200 copies/mL without virological rebound or discontinuation for virological failure. Secondary outcomes included HIV RNA < 20 copies/mL, viral blips, treatment persistence, adverse events, CD4 change and changes in regimen complexity. Additionally, we assessed pharmacists’ role in antiretroviral regimen planning.

**Results**: Sixteen men with a median age of 62.5 (IQR 59.3–67.3) years were switched. Patients had been living with HIV for a median of 29 (IQR 22.8–32.0) years; most (13/15, 87%) had CD4 nadir < 200 cells/mm^3^. Patients were highly treatment‐experienced, had frequent prior AIDS‐defining illnesses and high prior treatment failure rates (Table 1). Reasons for switch were multifactorial. Virological suppression was maintained by 15 (94%) patients at last follow‐up (median follow‐up time 32 [IQR 28–39] weeks). Protease inhibitor use decreased from 13 (81%) to 2 (13%) patients post‐switch (*p* = 0.002). The proportion of patients receiving ≥5 antiretroviral tablets/day decreased from 8 (50%) to 4 (25%) (*p* = 0.27). Four patients experienced mild adverse events, none required discontinuation. Pharmacists provided input into identifying patients suitable for switching, managing non‐antiretroviral medications and DDIs (*n* = 15, 94%).

**Conclusions**: Fostemsavir switch was safe and effective in highly treatment‐experienced men with complex resistance and comorbidity profiles. Most maintained virological suppression with substantial reduction in protease inhibitor use and no discontinuations due to adverse events. A pharmacist's input as part of the multidisciplinary team can optimize transition to a fostemsavir‐containing regimen.

**TABLE 1:**
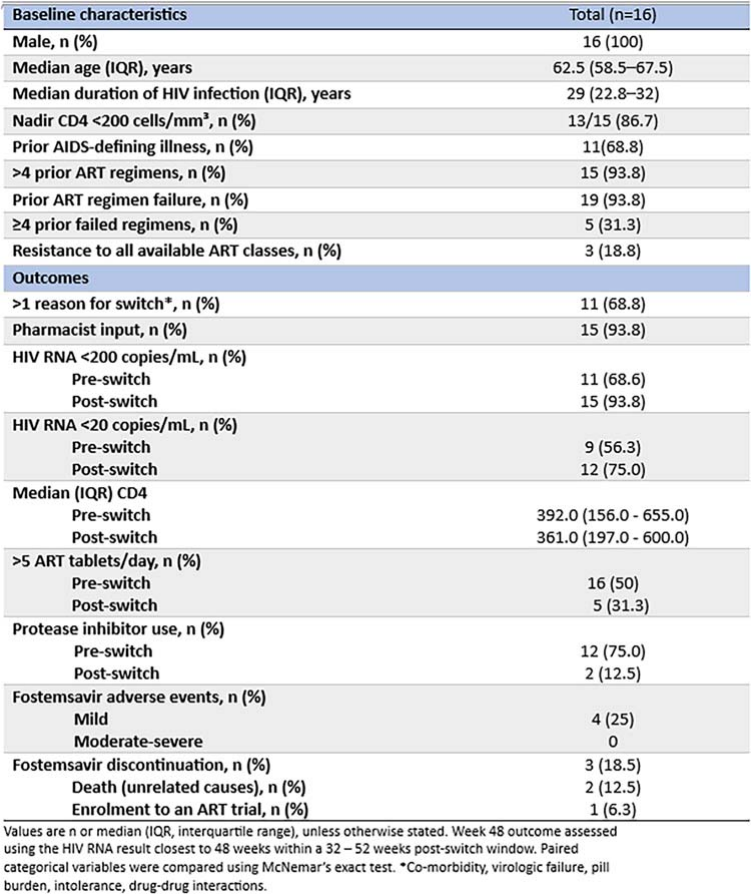
P157 | Baseline characteristics and outcomes of switch.

### P158 Rapid Improvement by Day 8 in CD4/CD8 Ratio With Fostemsavir Treatment in Individuals With Multidrug‐Resistant HIV‐1

Judith Aberg^1^, Michelle Moorhouse^2^, Gustavo Verdier^3^, Hemlata Pant^4^, Neetu Badhoniya^5^, Cynthia Donovan^6^, Scott McCallister^7^, Piotr Budnik^4^, Manyu Prakash ^4^

^1^Division of Infectious Diseases, Icahn School of Medicine at Mount Sinai, New York, US; ^2^ViiV Healthcare, Johannesburg, South Africa; ^3^ViiV Healthcare, Montréal, Canada; ^4^ViiV Healthcare, London, UK; ^5^GSK, London, UK; ^6^ViiV Healthcare, Durham, NC, US; ^7^ViiV Healthcare, Branford, US

**Background**: While viral suppression remains essential, preservation of immune function is a key driver of antiretroviral treatment success. Beyond achieving an undetectable viral load, effective therapy should support immune restoration. Early immune recovery may help re‑establish immune competence and contribute to sustained treatment benefit over time.

**Methods**: BRIGHTE is an ongoing, multi‐national, phase 3 study in adults with multi‐drug‐resistant HIV‐1. In the randomized cohort (RC; *n* = 272), participants with 1 or 2 fully active antiretroviral agents received fostemsavir (FTR; *n* = 203) or placebo (*n* = 69) added to a failing regimen for 8 days, followed by open‑label FTR with optimized background therapy. Immunological parameters, including T‑cell subsets and inflammatory biomarkers, were assessed from baseline through day 8 (D8).

**Results**: Statistically significant improvements (FTR vs. placebo on change from baseline) in circulating immune biomarkers were observed at D8 in the FTR group, including increased CD4 percentage and CD4/CD8 ratio (both *p* < 0.02), along with a statistically significant reduction within the FTR group in soluble CD163 levels (*p* = 0.002) (Table 1). No significant changes were observed in soluble CD14 or D‐dimer levels. Reductions in viral load were previously reported and consistent with prior FTR monotherapy studies, with mean HIV‐1 RNA decreasing by 0.82 log_10_ copies/mL at D8 in the FTR group, compared with 0.17 log_10_ copies/mL in the placebo group.

**Conclusions**: Use of FTR in this population demonstrated by D8 an improvement in immune biomarkers when compared with placebo. These findings highlight the impact of FTR in contributing to an improved immune status within 8 days of treatment initiation.

**TABLE 1:**
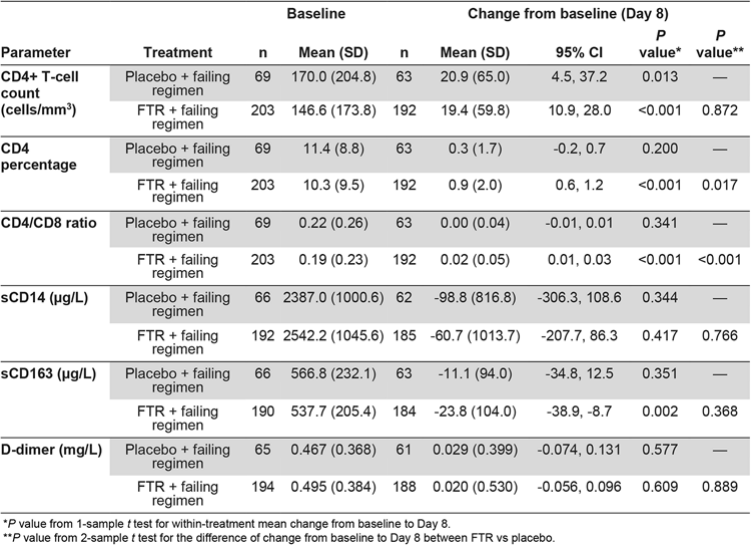
P158 | Change in immune biomarkers from baseline to day 8.

### P159 Identifying and Characterizing People With HIV Who Are Virologically Suppressed and Receiving Complex Regimens in Spain: The CoRIS Cohort

Inmaculada Jarrín Vera^1,2^, Melchor Riera^3^, José Ignacio Bernardino ^4^, Marta Rava^1^, Adrian Curran^5^, Sergio Reus Bañuls^6^, Helena Albendín^7^, Jesse Najarro Cermeño^8^, Javier Leal Martinez‐Bujanda^9^, Neia Prata Menezes^10^, Andrea Marongiu^11^, Santiago Moreno^2^, on behalf of the CoRIS cohort

^1^CoRIS Cohort Coordination Unit, National Center for Epidemiology, Instituto de Salud Carlos III, Madrid, Spain; ^2^CIBERINFEC, Carlos III Health Institute, Madrid, Spain; ^3^Hospital Universitario Son Espases, IDISBA, Palma de Mallorca, Spain; ^4^HIV Unit, Internal Medicine Department, Hospital Universitario La Paz‐Carlos III, IdiPAZ, Madrid, Spain; ^5^Infectious Diseases Department, Hospital Universitari Vall d'Hebron, Vall d'Hebron Research Institute (VHIR), Barcelona, Spain; ^6^Department of Medicine, Hospital General Universitario de Alicante, Alicante, Spain; ^7^HIV Unit, Internal Medicine Department, Hospital Virgen de la Arrixaca, Murcia, Spain; ^8^Gilead Sciences, Inc., Parsippany, NJ, US; ^9^Gilead Sciences, SLU, Madrid, Spain; ^10^Gilead Sciences, Inc., Foster City, CA, US; ^11^Gilead Sciences Ltd, Uxbridge, UK

**Background**: Despite advances in antiretroviral therapy (ART) that have enabled treatment simplification, complex regimens (CR) remain necessary for some people with HIV (PWH) due to prior ART resistance, contraindications or drug−drug interactions. However, little is known about the population size and characteristics of virologically suppressed individuals receiving these regimens in real‐world European settings. We aimed to identify and characterize virologically suppressed PWH on CR in CoRIS.

**Methods**: We conducted a cross‐sectional analysis on 1 January 2022, within CoRIS. We included adults with at least one prior ART regimen and virological suppression (HIV RNA ≤50 copies/mL) during the previous 12 months. CR were defined as regimens containing ≥2 core drug classes (integrase strand transfer inhibitor [INSTI], non‐nucleoside reverse transcriptase inhibitor [NNRTI] and protease inhibitor [PI]), boosted PI‐based (bPI) regimens including once‐daily dosing (QD), single tablet regimens (STRs) or regimens requiring more than QD or multi‐tablet regimens (MTRs) (≥2 pills/day). Regimens with an STR equivalent were not considered complex, regardless of formulation, dosing or anchor class.

**Results**: Among 18,766 PWH in CoRIS, 855 (5%) virologically suppressed, treatment experienced (TE) individuals on ART were receiving CR. Median age was 47.4 years (IQR 40.1–55.2), 81% were male sex at birth and 56% were born in Spain. Median time from HIV diagnosis to initiation of CR was 83.2 months (IQR 42.3–125.6) and individuals had received a median of 2 prior ART regimens (IQR 2–4). These participants had a median CD4 count of 665 cells/mm^3^ (IQR 505–901), median duration of virological suppression was 57.6 months (IQR 25.6–103.9) and median duration of current CR was 41.3 months (IQR 26.3–59.5). Most CR were boosted bPI‐based, particularly bPI+2 NRTIs (48%), bPI+NRTI dual therapy (17%) and bPI monotherapy (9%) (Figure 1); Darunavir was the most prescribed PI (87%). Overall, 54% of regimens were MTRs and 14% required twice‐daily dosing with a median of 2.0 antiviral pills per day (IQR 1.0–3.0).

**Conclusions**: 5% of PWH in CoRIS were virologically suppressed, TE and receiving CR. While most had durable virological suppression and preserved immune function, these findings highlight the need for individualized, flexible treatment strategies, as individuals on CR may still benefit from treatment optimization.

**FIGURE 1:**
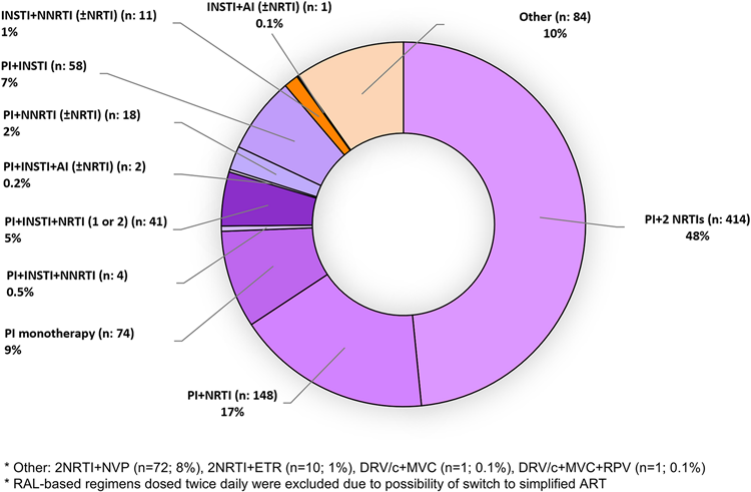
P159 | Distribution of complex regimens among people with HIV who were virologically suppressed and treatment experienced in CoRIS (*n* = 855).

### P160 Abstract Withdrawn

### P161 Characterization of Non‐Canonical Mutations in People With HIV Experiencing Persistent Low‐Level Viraemia

Ilaria Vicenti^1,2^, Ilenia Varasi^1^, Lia Fiaschi^1^, Luca Baldassari^3^, Francesco Saladini^1^, Laura Comi^3^, Sara Soloperto^4^, Maurizio Zazzi^1^, Antonio D'Avolio^4^, Diego Ripamonti ^3^

^1^Department of Medical Biotechnologies, University of Siena, Siena, Italy; ^2^Faculty of Medicine, Saint Camillus International University of Health Sciences, Rome, Italy; ^3^ASST Papa Giovanni XXIII, Bergamo, Italy; ^4^Laboratory of Clinical Pharmacology and Pharmacogenetics, Turin, Italy

**Background**: Despite potent antiretroviral therapy (ART), persistent low‐level viraemia (LLV) may occur in the absence of canonical resistance. Recent evidence suggests that epistatic mutations in non‐canonical genomic regions, including *gag*, *env* and *nef*, may contribute to increased viral fitness and/or resistance. This study aimed to characterize non‐canonical mutations outside the *pol* region in people with HIV (PWH) with persistent LLV.

**Methods**: PWH with persistent LLV (mean 5 ± 3 years) despite adherence to ART were enrolled at ASST Papa Giovanni XXIII. ART adherence was assessed by therapeutic drug monitoring of INSTI concentration. Plasma HIV‐1 RNA was analysed by near full‐length (NFL) sequencing using Illumina platform. When NFL failed, a targeted PCR approach was used to amplify and sequence by NGS pol and the regions possibly involved in epistatic mechanisms (nucleocapsid [NC], *env* and polypurine tract [3’PPT]). Reads were assembled by Geneious Prime 2026.0.2 and species accounting for > 25% were mapped to HXB2.

**Results**: Seven PWH with a mean age of 59 ± 8 and ART experienced (mean ART duration 12 ± 8 years) were included. Median (IQR) HIV‐RNA level was 158 [130–404] copies/mL at last follow‐up. INSTI drug levels were within expected range (Table 1). Amplification was obtained in different genomic regions: NC (*n* = 4), full‐length *gag* (*n* = 4), 3′PPT (*n* = 3), *env* (*n* = 2) and NFL genome (*n* = 1). In 2 participants, the NC N8S and N8G substitutions were detected, associated with INSTI resistance in‐vitro [1] and in vivo [2]. In *env*, D99N + V693I were identified in 2 samples, while V87E and Q363R were detected in one sample; these mutations are related to INSTI resistance in vitro [1,3] and in vivo [4]. Within gp41, T165S and I270T, previously found associated with PI VF [5,6], were detected in one participant. No mutations were detected in 3′PPT. The participant with the highest viraemia showed concurrent substitutions across NC, *env* and gp41. Additional NNRTI canonical resistance mutations were identified, not associated with reduced susceptibility to the ongoing regimen (Table 1).

**Conclusions**: Epistatic mutations in *gag* and *env* were detected in PWH with persistent LLV under INSTI‐based ART, including one case across multiple regions. Larger studies, integrating longitudinal sampling and phenotypic assays, are needed to clarify their biological and clinical relevance.

**References**

1. Hikichi Y, Sharma A, Kanemura Y, et al. Epistatic pathways can drive HIV‐1 escape from integrase strand transfer inhibitors. *Sci Adv*. 2024;10(9):eadn0042.

2. Coetzee N, Delaney KE, Jennings L, et al. Noncanonical resistance mutations in TLD‐treated individuals with unexplained virologic failure. In: Abstracts from the Conference on Retroviruses and Opportunistic Infections (CROI); 2026. *Top Antivir Med*. 2026;34(Suppl 1): ìAbstract 553.

3. Hikichi Y, Penrose KJ, Mathur S, et al. Env mutations are a gateway for acquisition of InSTI resistance. In: Abstracts from the Conference on Retroviruses and Opportunistic Infections (CROI); 2026. *Top Antivir Med*. 2026;34(Suppl 1):Abstract 551.

4. Sudderuddin H, Dang Z, Watson B, et al. Investigation of integrase inhibitor resistance mutations in gp41 in clinical samples. *J Acquir Immune Defic Syndr*. 2025;100(1):e95‐e102.

5. Castain L, Ramière C, Charre C, et al. New mechanisms of resistance in virological failure to protease inhibitors: selection of non‐described protease, Gag and gp41 mutations. *J Antimicrob Chemother*. 2019;74(7):2019‐2023.

6. Perrier M, Charpentier C, Storto A, et al. HIV‐1 protease, Gag and gp41 baseline substitutions associated with virological response to a PI‐based regimen. *J Antimicrob Chemother*. 2019;74(6):1679‐1692.

**TABLE 1:**
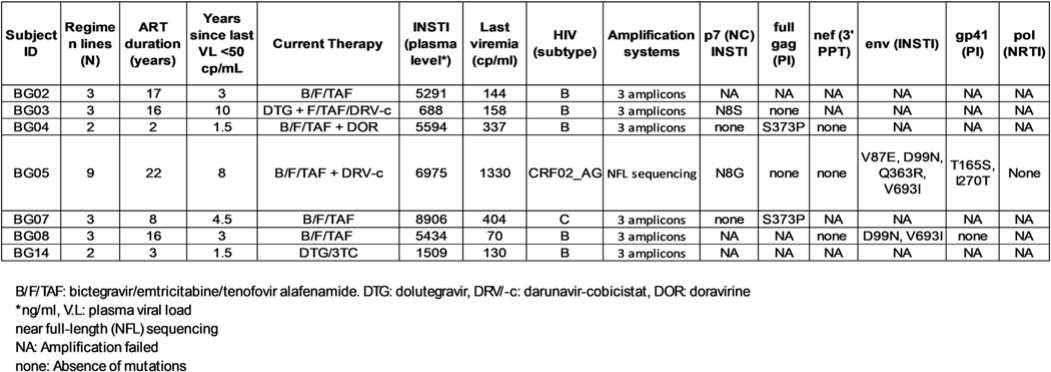
P161 | Clinical and virological characteristics of seven PWH with persistent low‐level viraemia including non‐canonical mutations detected in *gag*, *env* and *gp41*.

### P162 Real‐World Effectiveness of Long‐Acting Cabotegravir Plus Rilpivirine in People Living With HIV With a Long Treatment History and Extensive Prior Antiretroviral Exposure

Rodrigo De Almeida Mota, Cátia Caldas, Carmela Piñeiro, Rosário Serrão, Nélia Neves, Lurdes Santos

Department of Infectious Diseases, Unidade Local de Saúde de São João, Porto, Portugal

**Background**: Long‐acting (LA) cabotegravir (CAB) plus rilpivirine (RPV) offers an alternative to daily oral antiretroviral therapy (ART). Real‐world data in people with a long treatment history and multiple prior regimens remain limited. We describe our experience at a Portuguese tertiary centre, focusing on participants with extensive prior ART exposure.

**Methods**: Retrospective analysis of people with HIV (PWH) initiating CAB+RPV LA. In total, 576 PWH initiated treatment between August 2024 and March 2026. Eligible PWH had ≥12 months of follow‐up since LA initiation, irrespective of early interruption (*n* = 159; 27.6%). We focused on the subset with ≥4 prior ART regimens (*n* = 90; 56.6%). Virological suppression was defined as HIV‐1 RNA <50 copies/mL.

**Results**: In this subset (*n* = 90), median age was 55 years and 70% were assigned male at birth. Median time since HIV diagnosis was 21 years and median time on ART 21.7 years. The main transmission routes were heterosexual contact (43.3%), sex between men (26.7%) and injecting drug use (25.5%). The predominant subtype was B (46%) and resistance genotyping was available for 78%. Median time on CAB+RPV LA was 14 months. Virological suppression was 100% (90/90) at month 1, 97.6% (83/85) at month 6 and 95.1% (77/81) at month 12. No confirmed virological failure was observed. Eleven participants discontinued LA therapy, mainly for logistical/scheduling constraints (*n* = 5), injection‐site pain (*n* = 3), other adverse reactions (*n* = 2) and death from neoplasia (*n* = 1); all had HIV‐1 RNA <50 copies/mL at discontinuation.

**Conclusions**: In a real‐world cohort of PWH with long‐standing infection and extensive prior ART, CAB+RPV LA achieved high and sustained virological suppression through 12 months, with no confirmed virological failure and few discontinuations, all occurring while virologically suppressed. These data support LA therapy as an effective option even in heavily treatment‐experienced populations.

### P163 B‐ALONE: Bictegravir/Emtricitabine/Tenofovir Alafenamide as a Single‐Tablet Regimen Simplification in Virologically Suppressed People Living With 4‐Class Drug‐Resistant HIV: Data From the PRESTIGIO Registry

Tommaso Clemente ^1^, Giovanni Festa^2^, Riccardo Lolatto^1^, Annamaria Cattelan^3^, Antonio Di Biagio^4,5^, Stefano Nardi^6^, Nicolò Capra^1^, Layla Pagnucco^7^, Giancarlo Orofino^8^, Cecilia Costa^9^, Maria Mercedes Santoro^10^, Antonella Castagna^1,2^, on behalf of PRESTIGIO Study Group

^1^Infectious Diseases, IRCCS San Raffaele Scientific Institute, Milan, Italy; ^2^Vita‐Salute San Raffaele University, Milan, Italy; ^3^Infectious and Tropical Diseases Unit, Padua University Hospital, Padua, Italy; ^4^Infectious Disease Unit, AOM San Martino, Genoa, Italy; ^5^DiSSal, Università degli Studi di Genova, Genoa, Italy; ^6^Unit of Infectious Diseases, AULSS 9, Verona, Italy; ^7^Infectious Diseases, Fondazione IRCCS Policlinico San Matteo, Pavia, Italy; ^8^Unit of Infectious Diseases, “Divisione A”, ASL “Città Di Torino”, Turin, Italy; ^9^Infectious Diseases Unit, Santa Maria Annunziata Hospital, Azienda USL Toscana Centro, Florence, Italy; ^10^Department of Experimental Medicine, University of Rome Tor Vergata, Rome, Italy

**Background**: We aimed to evaluate virological outcomes following simplification to bictegravir/emtricitabine/tenofovir alafenamide (B/F/TAF) as the sole antiretroviral regimen in virologically suppressed people with 4‐class drug‐resistant (4DR) HIV.

**Methods**: Cohort study including people with 4DR HIV from the PRESTIGIO Registry [1] who achieved virological suppression (viral load [VL] < 50 copies/mL) and simplified to B/F/TAF single‐tablet regimen (STR). Four‐class drug resistance was defined as resistance to ≥1 nucleoside reverse transcriptase inhibitor (NRTI), ≥1 non‐nucleoside reverse transcriptase inhibitor (NNRTI) and ≥1 protease inhibitor (PI), plus either resistance to ≥1 integrase inhibitor (INSTI) or virological failure on INSTI‐containing regimen. Follow‐up accrued from the date of B/F/TAF initiation (baseline) until B/F/TAF discontinuation, antiretroviral therapy (ART) intensification or last visit. The primary outcome was maintenance of virological suppression. Loss of virological suppression was defined as two consecutive VLs ≥50 copies/mL or a single VL ≥1000 copies/mL. Secondary outcomes included B/F/TAF durability and CD4^+^ changes.

**Results**: Among 272 people with 4‐class drug‐resistant (4DR) HIV from the PRESTIGIO Registry who achieved VL < 50 copies/mL, 10 (3.7%) simplified to B/F/TAF as their sole STR after a median of 4.5 years (interquartile range [IQR], 1.8–8.1) of sustained virological suppression, primarily to reduce pill burden (8/10, 80%). Median age was 58.9 (55.4–60.7) years, 7 (70%) male at birth, median ART duration 27.0 (22.2–27.6) years, 7.3 (4.5–11.1) years since first 4DR evidence. The median genotypic susceptibility score for B/F/TAF was 1.5 (1.0–2.0); NRTI and INSTI resistance‐associated mutations reported in Figure 1. During a median follow‐up of 3.6 (1.8–5.0) years, 6 (60%) remained on B/F/TAF and in follow‐up. All participants (10/10 [100%]) maintained virological suppression (Table 1); one experienced multiple viral blips and underwent ART intensification after 3.5 years. CD4^+^ T‐cell counts remained stable over time (*p* = 0.570).

**Conclusions**: In our small cohort of virologically suppressed people with 4DR HIV who simplified to B/F/TAF, this STR was not associated with loss of virological suppression. Despite a history of extensive drug resistance, careful selection of individuals based on sustained virological suppression and mutation profile may allow treatment simplification with B/F/TAF in selected cases.

**Reference**

1. Clemente T, Galli L, Lolatto R, et al. Cohort profile: PRESTIGIO, an Italian prospective registry‐based cohort of people with HIV‐1 resistant to reverse transcriptase, protease and integrase inhibitors. BMJ Open. 2024 Feb 10;14(2):e080606.

**FIGURE 1:**
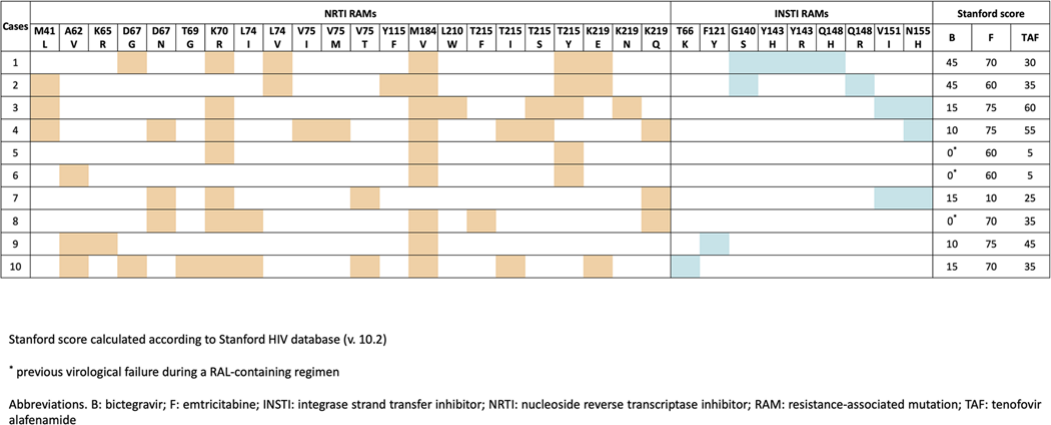
P163 | Cumulative NRTI and INSTI resistance‐associated mutations in virologically suppressed people with 4‐class drug‐resistant HIV who simplified to bictegravir/emtricitabine/tenofovir alafenamide single‐tablet regimen.

**TABLE 1:**
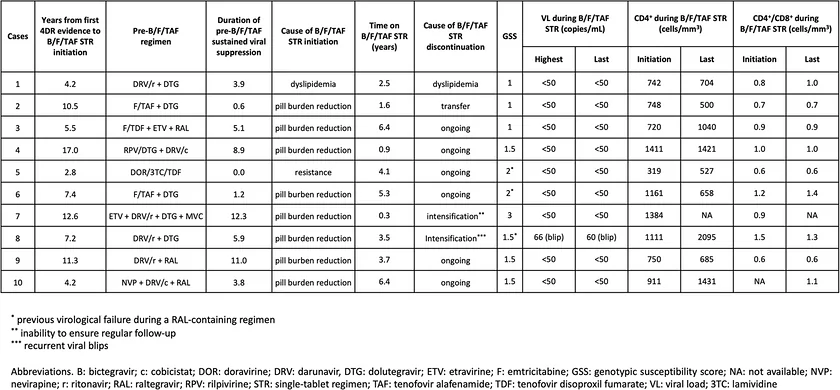
P163 | Characteristics and outcomes of virologically suppressed people with 4‐class drug‐resistant HIV who simplified to bictegravir/emtricitabine/tenofovir alafenamide single‐tablet regimen.

### P164 Proviral DNA Genotyping Reveals Clinically Relevant HIV Drug Resistance Across the Spectrum of Low‐Level Viraemia: A Nationwide Cohort Study

Pablo Ferrer, Alejandro Afani, Verónica Ramos

Medicine, Clinical Hospital University of Chile, Santiago, Chile

**Background**: The clinical relevance of HIV drug resistance detected by proviral DNA in individuals with persistent low‐level viraemia (LLV) remains incompletely understood. We evaluated the prevalence and determinants of archived resistance mutations detected by proviral DNA genotyping in people living with HIV receiving antiretroviral therapy and presenting with plasma HIV‐1 RNA levels below 1000 copies/mL.

**Methods**: We conducted a retrospective nationwide cohort study including 1074 individuals meeting eligibility criteria for proviral DNA genotyping. Reverse transcriptase and protease genotypes were successfully obtained in 953 individuals, while 747 also underwent concomitant integrase genotyping. Resistance was defined according to the Stanford HIV Drug Resistance Database. Associations between clinical variables, viral load and resistance were assessed using logistic regression models.

**Results**: Among 953 interpretable genotypes, 287 (30.1%) harboured at least one resistance‐associated mutation. The most frequent mutations were M184V/I (12.2%) and K103N (8.4%), followed by E138A/G/K/Q/R (5.0%) and V179D/E/L/T/V (4.7%). Resistance prevalence increased progressively across viral load strata, from approximately 25% among individuals with viral loads below 200 copies/mL to 48.4% among those with viral loads between 500 and < 1000 copies/mL. Viral load remained independently associated with resistance in all adjusted models (adjusted OR approximately 1.07 per 100 copies/mL increase). M184V/I and K103N maintained independent associations with increasing viral load (Figure 1). Integrase analysis identified predominantly accessory mutations, whereas major INSTI resistance pathways, including G140, N155H, E92Q/G, Q148H/R and Y143C/R/H, were detected at low frequencies ranging from 2.1% to 3.9%.

**Conclusions**: Archived HIV drug resistance is frequently detected by proviral DNA genotyping in individuals with persistent LLV and becomes increasingly prevalent with higher levels of residual viral replication. Proviral DNA genotyping may provide clinically relevant resistance information when conventional plasma RNA genotyping is not feasible, particularly in patients with persistent low‐level viraemia.

**FIGURE 1:**
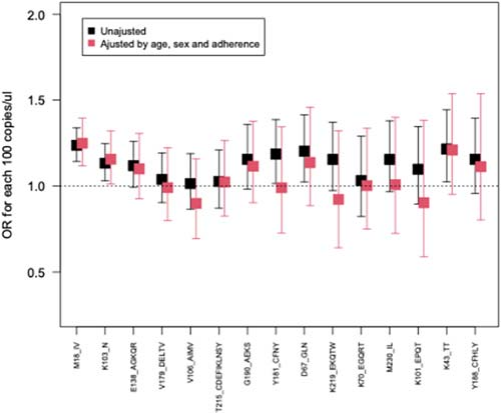
P164 | Association between the 15 most frequent HIV drug resistance mutations and plasma viral load at the time of genotyping. Crude and adjusted odds ratios (ORs) are shown for each 100‐copy/mL increase in HIV‐1 RNA. Adjusted models included age, sex and treatment adherence as covariates. M184V/I and K103N remained significantly associated with higher viral loads after multivariable adjustment, whereas other associations observed in unadjusted analyses were attenuated following adjustment. Horizontal bars represent 95% confidence intervals (95% CIs).

### P165 Efficacy and Safety of Switching to BIC/FTC/TAF Plus DRV/Cob Versus DRV/R, RAL, TDF for Maintaining Virologic Suppression in Heavily Experienced Patients With HIV‐1

Marco Antonio Lopez Hernandez^1^, Juan Pablo Álvarez Mendoza ^1^, Ericka Denise Bustos Angel^2^

^1^CLISIDA, Hospitales Generales Regionales, IMSS, Mexico City, Mexico; ^2^Infectology, Hospital General de México, Mexico City, Mexico

**Background**: Patients living with HIV (PLWH) with longstanding HIV infection and ART who have switched therapy several times because of previous virological failures, drug‐related adverse events or drug–drug interactions due to comorbidities require complex ART with regimens thus hampering adherence to and long‐term success of ART. Data on the combination of BIC/FTC/TAF+darunavir/cobicistat in this scenario are scarce. This study aimed to evaluate the efficacy and safety of this two‐pill regimen in PLWH taking a DRV/, RAL, TDF because of previous virological failure and/or adverse events.

**Methods**: This study was a multicentre, randomized two arms, open‐label, phase 4, pilot trial. Adults with HIV‐1 RNA < 40 copies/mL with previous virologic failure treated with DRV/r, RAL, TDF were randomized 1:1 to switch to once‐daily fixed‐dose combination DRV/cob plus BIC/FTC/TAF or continue with the treatment (primary endpoint: proportion of participants with HIV‐1 RNA ≥40 copies/mL at week 48; Snapshot, intention‐to‐treat‐exposed population, 5% non‐inferiority margin).

**Results**: Overall, 60 adults were randomized to switch to of BIC/FTC/TAF+darunavir/cobicistat (*n* = 30) or continue DRV/r, RAL, TDF (*n* = 30). At week 48, 1 (3%) participant in the DRV/r, RAL, TDF group had HIV‐1 RNA ≥40 copies/mL (Snapshot), demonstrating non‐inferiority. Zero participants met confirmed virologic withdrawal criteria; therefore, no resistance testing was performed. Drug‐related adverse events were more frequent with DRV/r, RAL, TDF (10%) than BIC/FTC/TAF plus DRV/cob (3%) through week 48 but comparable post‐week 24 (6% vs. 2%, respectively). Both groups had generally minimal changes in lipids.

**Conclusions**: Switching to BIC/FTC/TAF plus DRV/cob was non‐inferior to continuing DRV/, RAL, TDF for maintaining virologic suppression at week 48 with no observed resistance, supporting the efficacy, good safety and high barrier to resistance of BIC/FTC/TAF plus DRV/cob.

### P166 Identification of Viraemic Heavily Treatment‐Experienced People With HIV Who May Benefit From Optimized Therapy Using a Clinical Decision Support System

Steven Barnett ^1^, Rachel Palmieri Weber^2^, Jennifer Fusco^2^, Michael Osterman^2^, Courtney Sherman^3^, Cindy Markarian^4^, Mona‐Gekanju Toeque^4^, Ricky Hsu^5,6^, Michael Sension^7^, Adanna Oragwu^8^, Seojin Park^8^, Keith Dunn^8^, Gregory Fusco^2^

^1^CAN Community Health, Tampa, FL, US; ^2^Epividian, Inc., Raleigh, NC, US; ^3^CAN Community Health, Arlington, TX, US; ^4^AIDS Healthcare Foundation, Los Angeles, CA, US; ^5^NYU Langone Medical Center, New York, NY, US; ^6^AIDS Healthcare Foundation, New York, NY, US; ^7^CAN Community Health, Fort Lauderdale, FL, US; ^8^Gilead Sciences, Inc., Foster City, CA, US

**Background**: Heavily treatment‐experienced (HTE) people with HIV with limited treatment options represent a small but important population who might benefit from optimization with novel antiretrovirals (ARVs). Given the low prevalence of HTE, we sought to identify viraemic HTE individuals to their clinical care team via CHORUS, a clinical decision support system (CDSS) and characterize subsequent changes to their antiretroviral therapy (ART).

**Methods**: Adults with HIV in the OPERA cohort with exposure to HTE‐indicative ARVs (Figure 1) who became viraemic were identified in CHORUS (1 June 2025–6 December 2025) and followed through 28 February 2026. Viraemia was defined as the last two viral loads ≥200 copies/mL, with ≥1 occurring ≥4 weeks after initiating the baseline regimen. This information, the individual's baseline regimen, a link to HIV treatment guidelines and informational documentation on fostemsavir (FTR), ibalizumab (IBA) and lenacapavir (LEN) were available in the CDSS for consideration by the provider. Clinic visits and subsequent ART regimen changes over follow‐up were described.

**Results**: Of 194 viraemic HTE individuals, most (*n* = 163; 84%) had a follow‐up visit. Forty‐nine (30%) of those individuals made changes to their ART regimen over a median 32 weeks of follow‐up (interquartile range: 24, 37). Among those, 13 (27%) added FTR, IBA or LEN to a follow‐up regimen (Figure 1). These individuals had less time since HIV diagnosis and since initiation of baseline regimen; they were more likely to be Black, on a regimen with ≥2 anchor classes and have a history of an AIDS‐defining event (Table 1). FTR and LEN were each added by 8 (62%) individuals; fewer than 5 individuals added IBA or more than one of FTR, IBA or LEN. The proportions of individuals who switched to ≥1 regimen containing ≥2 anchor classes over follow‐up were 100% (FTR/IBA/LEN group) and 69% (not FTR/IBA/LEN group).

**Conclusions**: Most viraemic HTE individuals did not make a change to their ART regimen over follow‐up. Those who added FTR, IBA or LEN appear to have different characteristics than individuals whose change(s) did not include these ARVs and may be progressing faster in their HIV infection. Combined, these findings indicate that additional education and intensive intervention to identify and optimize care with novel ARVs may be needed.

**FIGURE 1:**
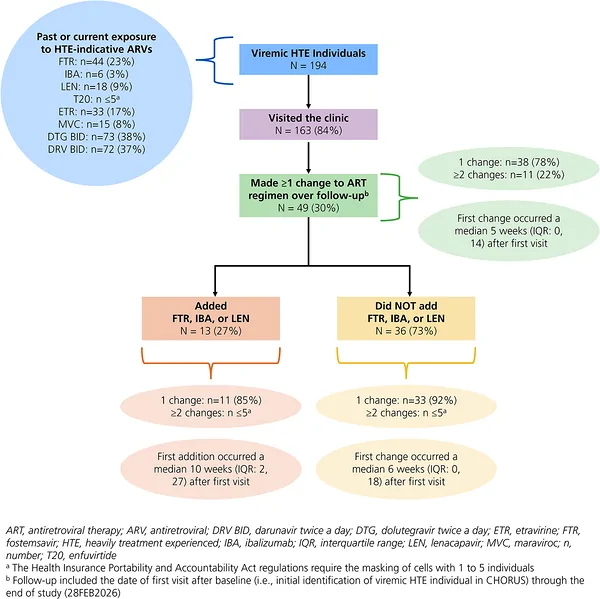
P166 | Viraemic HTE individuals identified in the CHORUS clinical decision support system and their clinical care events that occurred over follow‐up (rectangles) with characteristics of individuals and events (circles/ovals).

**TABLE 1:**
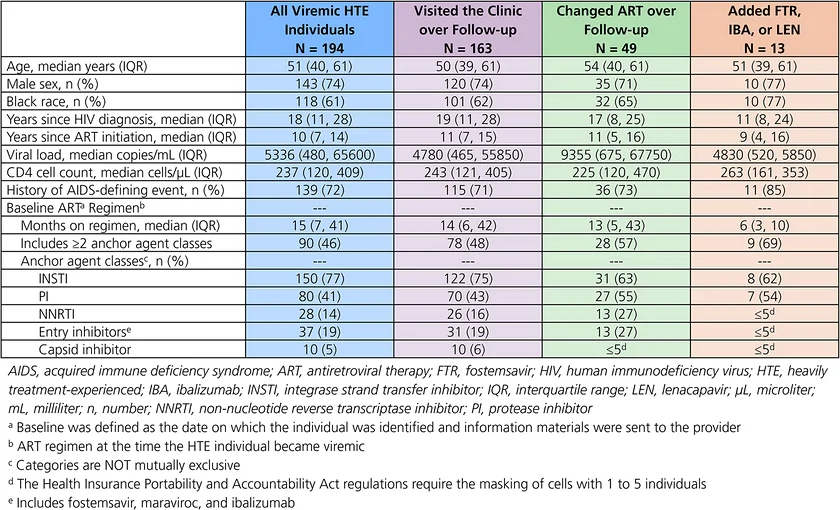
P166 | Baseline characteristics of viraemic HTE individuals, by group.

### P167 Predicted Reduction in Drug−Drug Interactions With Bictegravir/Lenacapavir Compared to Current Complex Regimens Among People With HIV Who Were Virologically Suppressed: An OPERA Cohort Analysis

Anthony Mills^1^, Michael Osterman^2^, Rachel Weber^2^, Gerald Pierone Jr^3^, Karam Mounzer^4^, Michael Sension^5^, Jennifer Fusco ^2^, Neia Prata Menezes^6^, Rebecca Harrison^6^, Priyanka Arora^6^, Rachel Rogers^6^, Bhumi Gandhi‐Patel^6^, Keith Dunn^6^, Gregory Fusco^2^

^1^Men's Health Foundation, West Hollywood, CA, US; ^2^Epividian, Inc., Raleigh, NC, US; ^3^Whole Family Health Center, Vero Beach, FL, US; ^4^Philadelphia FIGHT Community Health Centers, Philadelphia, PA, US; ^5^CAN Community Health, Fort Lauderdale, FL, US; ^6^Gilead Sciences, Inc., Foster City, CA, US

**Background**: Despite advances in antiretroviral therapy (ART), some individuals still require complex regimens for HIV treatment. Complex regimens often include multiple core agents or a pharmacokinetic boosting agent, presenting an increased risk of clinically relevant drug−drug interactions (DDIs) with non‐HIV medications, especially among ageing populations. Bictegravir/lenacapavir (BIC/LEN) is being studied as an investigational single‐tablet regimen (STR) for use among people with HIV that are virologically suppressed. This study aimed to describe the likelihood of encountering DDIs among individuals using complex regimens compared to BIC/LEN.

**Methods**: Virologically suppressed (viral load < 50 copies/mL) adults with HIV in the OPERA cohort using one of the 10 most common complex ART regimens in OPERA on 1 July 2025 were included (Figure 1). Demographic and clinical characteristics, including polypharmacy, were described. Individuals were considered to have a likelihood of encountering DDIs if they used ≥1 medication between 1 January 2025 and 31 December 2025 that was identified as red or amber interaction level by the Liverpool HIV Interactions database for concomitant use with their current complex regimen. To predict likelihood of DDIs with multi‐component regimens, the most severe interaction level across components was used. Likelihood of DDIs and clinical recommendations for management of DDIs with BIC/LEN were validated based on Liverpool interaction levels, prescribing information, Lexicomp and expert opinion (Table 1).

**Results**: There were 2473 virologically suppressed adults using one of the 10 most common complex regimens in OPERA on 1 July 2025 (Figure 1). Median age was 55 years (interquartile range [IQR] 42, 62); they were 77% male, 46% Black and had a median of 9 non‐ART medications (IQR 5, 14) during the 1‐year period (Table 1). Median time since HIV diagnosis was 17 years (IQR 9, 27). When the concomitant medication profiles were evaluated, the predicted likelihood of encountering DDIs decreased from 85% with the current complex regimen to 71% with BIC/LEN. The likelihood of red‐level DDIs decreased from 26% to 7% (Table 1).

**Conclusions**: The predicted likelihood of encountering DDIs was higher with the current complex regimens than with BIC/LEN. The findings suggest that switching from a complex regimen to BIC/LEN may reduce the overall risk of DDIs, particularly for the highest severity (red) interactions.

**FIGURE 1:**
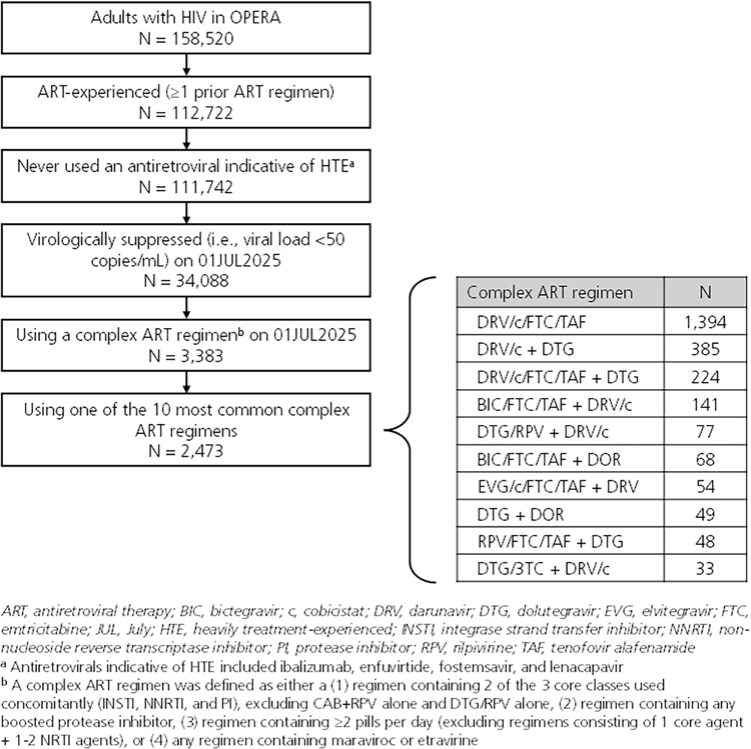
P167 | Identification of people with HIV who were virologically suppressed and using one of the 10 most common complex antiretroviral therapy regimens on 1 July 2025.

**TABLE 1:**
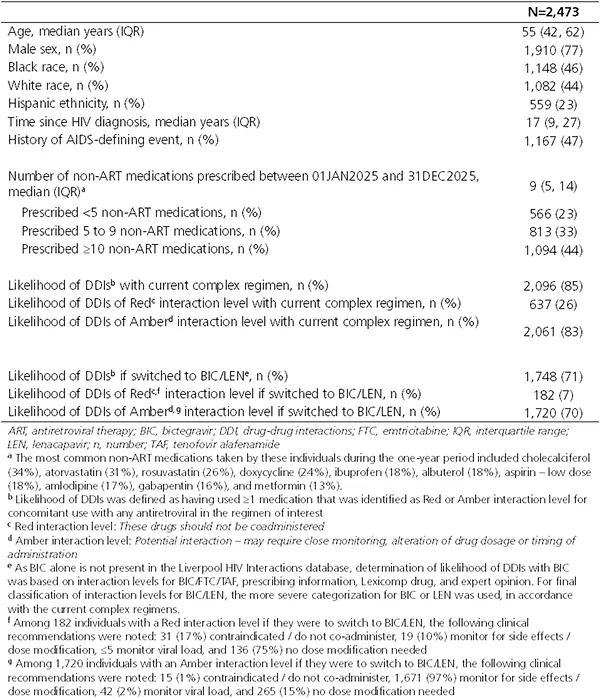
P167 | Characteristics and likelihood of encountering drug−drug interactions, among people with HIV who were virologically suppressed and using one of the 10 most common complex ART regimens in OPERA on 1 July 2025 (*N* = 2473).

### P168 Clinical Outcomes in People With HIV Switching From Oral Dolutegravir‐Based Two‐Drug Regimens to Bictegravir/Emtricitabine/Tenofovir Alafenamide (B/F/TAF): An Analysis From the Italian ICONA Foundation Cohort

Andrea De Vito ^1^, Alessandro Tavelli^2,3^, Andrea Giacomelli^4^, Andrea Costantini^5^, Giancarlo Orofino^6^, Riccardo Serraino^7^, Raffaella Marocco^8^, Roberto Rossotti^9^, Silvia Nozza^10^, Laura Albini^11^, Gabriele Forcina^11^, Alessandra Vergori^12^, Antonio Di Biagio^13^, Antonella D'Arminio Monforte^2^, Giordano Madeddu^14^, on behalf of ICONA Foundation Study Group

^1^Unit of Infectious Diseases, Sassari University Hospital, Sassari, Italy; ^2^ICONA Foundation, Milan, Italy; ^3^National PhD Programme in One Health Approaches to Infectious Diseases and Life Science Research, Department of Public Health, Experimental and Forensic Medicine, University of Pavia, Pavia, Italy; ^4^III Infectious Diseases Unit, ASST FBF‐Sacco, Milan, Italy; ^5^Clinical Immunology Unit, Azienda Ospedaliero Universitaria delle Marche—Università Politecnica delle Marche, Ancona, Italy; ^6^Division I of Infectious and Tropical Diseases, ASL Città di Torino, Turin, Italy; ^7^Dipartimento di Sicurezza e Bioetica—Sezione di Malattie Infettive, Università Cattolica del Sacro Cuore, Rome, Italy; ^8^Infectious Diseases Unit, SM Goretti Hospital, Sapienza University of Rome, Latina, Italy; ^9^Department of Diagnostics and Public Health, University of Verona, Verona, Italy; ^10^Infectious Diseases Unit, IRCCS San Raffaele Scientific Institute, Vita Salute San Raffaele University, Milan, Italy; ^11^Gilead Sciences Srl, Milan, Italy; ^12^Infectious Diseases Unit, A.O.U. Policlinico Foggia, University of Foggia, Foggia, Italy; ^13^Department of Infectious Diseases, University of Genoa, School of Medicine, San Martino Hospital, Genoa, Italy; ^14^Unit of Infectious Disease, Department of Medicine, Surgery and Pharmacy, University of Sassari, Sassari, Italy

**Background**: Evidence on the effectiveness and safety of B/F/TAF in people with HIV (PWH) switching from dolutegravir (DTG)‐based two‐drug regimens (2DRs) is limited.

**Methods**: OPTIMIZE‐BIC (GS‐IT‐380‐7670) is a retrospective study including PWH from ICONA cohort who switched from DTG+3TC or DTG+RPV to B/F/TAF between July 2019 and April 2025, regardless of HIV‐RNA at switch. Participants had ≥1 post‐switch HIV‐RNA measurement and were stratified by HIV‐RNA at switch: < 50 copies/mL (uVL) or ≥50 copies/mL (dVL). The primary endpoint was HIV‐RNA <50 copies/mL at last follow‐up. Secondary endpoints included time to virological failure (VF) among uVL participants, virological suppression (VS) among dVL and treatment discontinuation (TD). Cumulative probabilities were estimated using the Kaplan–Meier method.

**Results**: Overall, 104 PWH were included (Table 1). Most participants switched from DTG+3TC (86.5%); 61 (58.7%) were uVL and 43 (41.3%) were dVL at switch. Main reasons for switching to B/F/TAF were VF (41.3%), toxicity (22.1%), individual decision (14.4%) and simplification to single‐tablet regimen (9.6%). At last follow‐up, 89/104 (85.6%) had HIV‐RNA <50 copies/mL and 100/104 (96.1%) had HIV‐RNA < 200 copies/mL. Among dVL participants, 36/43 dVL participants ever achieved VS (83.7%, 95% CI: 69.3–93.2), with a median time of 3.4 months (95% CI: 2.9–5.7, Figure 1A). At last measurement, after a median follow‐up of 17.6 (6.7–34.1) months, 30/43 (69.8%) dVL PWH had HIV‐RNA <50 copies/mL. Among uVL, 59/61 (96.7%) maintained HIV‐RNA <50 copies/mL at last assessment after a median of 19.4 (9.4–33.4) months; one VF occurred (1.7%), with low‐level viraemia (91 and 77 copies/mL) followed by re‐suppression on B/F/TAF (Figure 1B). Twenty participants discontinued B/F/TAF; cumulative TD probability was 11.0% at 12 months, with no difference by baseline viraemia (Figure 1C). In detail, four PWH discontinued for toxicity (12‐month probability of TDT of 3.4%, 95% CI: 1.1–10.2, Figure 1D); five discontinued for switching to long‐acting, four for failure, two for simplification to less‐drug regimen and five for other reasons.

**Conclusions**: In this observational cohort, most PWH switched from 2DRs to B/F/TAF due to VF or toxicity. Switching to B/F/TAF was associated with high rates of virological control among virologically suppressed individuals and infrequent toxicity‐related discontinuation.

**FIGURE 1:**
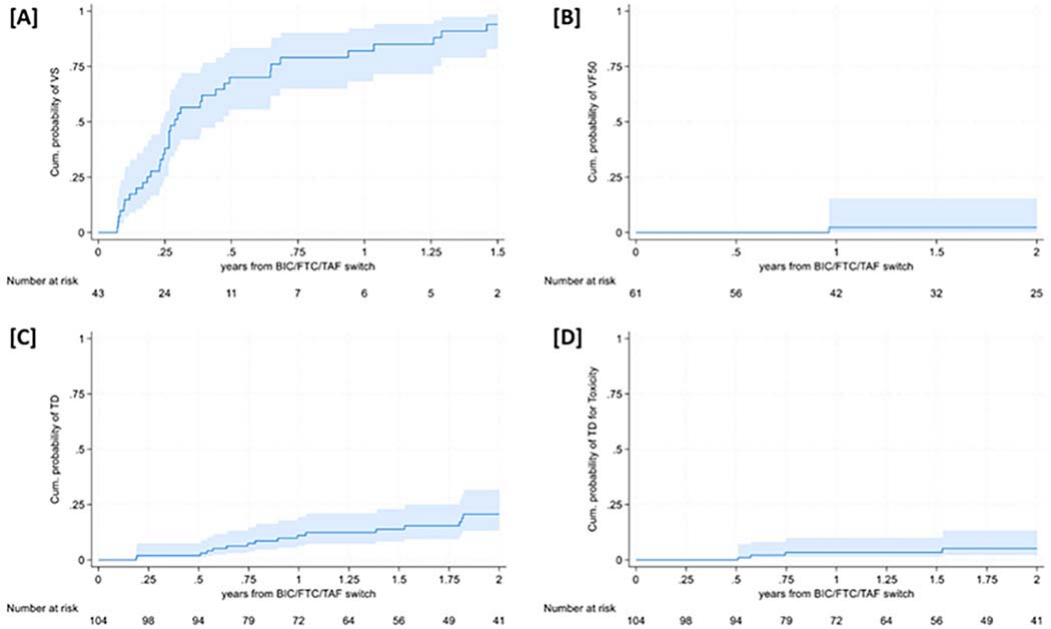
P168 | Kaplan–Meier estimates of the probability of (A) achieving HIV‐RNA <50 copies/mL (VS) among dVL individuals; (B) virological failure (VF50) among uVL individuals; (C) treatment discontinuation for any reason (TD); (D) treatment discontinuation due to toxicity.

**TABLE 1:**
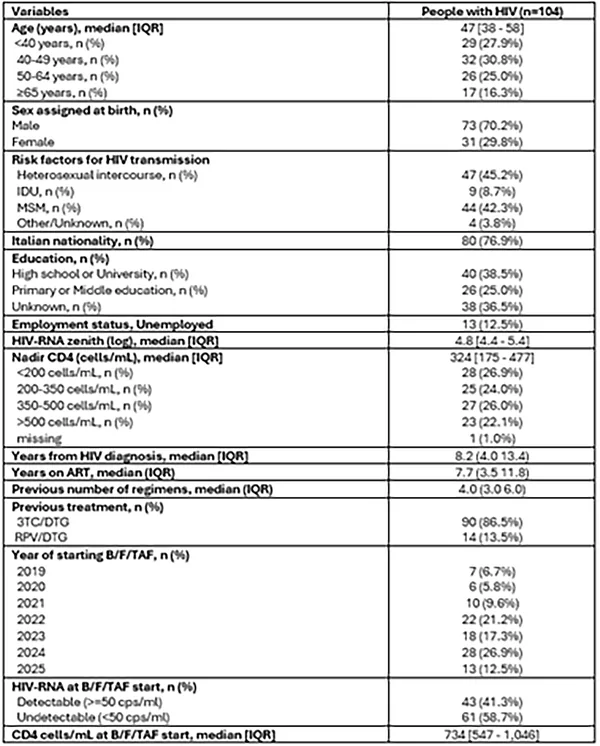
P168 | Baseline epidemiological and clinical characteristics of the study population.

### P169 Abstract Withdrawn

### P170 Long‐Term Effectiveness of Switching to Bictegravir/Emtricitabine/Tenofovir Alafenamide (B/F/TAF) From NNRTI‐Based Antiretroviral Therapy (ART) in Virologically Suppressed People With HIV: A Retrospective Analysis (DRIVE‐SWITCH Study)

Roberta Gagliardini ^1^, Marisa Fusto^1^, Sandrine Ottou^1^, Federica Forbici^1^, Lavinia Fabeni^1^, Giulia Del Duca^1^, Martina Maresca^1^, Alessandra Oliva^1^, Fabrizio Maggi^1^, Ilaria Mastrorosa^1^, Maria Maddalena Plazzi^1^, Federico De Zottis^1^, Rita Bellagamba^1^, Jessica Paulicelli^1^, Carmela Pinnetti^1^, Valentina Mazzotta^1^, Roberto Caldera^2^, Gabriele Forcina^2^, Andrea Antinori^1^

^1^INMI L. Spallanzani IRCCS, Rome, Italy; ^2^Gilead Sciences Srl, Milan, Italy

**Background**: Real‐world data after switching to B/F/TAF from NNRTI‐based ART remain limited, particularly among individuals on rilpivirine (RPV)‐based regimens.

**Methods**: This is a retrospective single‐centre study on people with HIV (PWH) virologically suppressed on NNRTI+2 NRTIs and switched to B/F/TAF. Primary endpoint was the proportion of PWH with HIV‐RNA <50 cp/mL at 12 and 24 months after baseline, defined as switch to B/F/TAF, in the intention‐to‐treat (ITT) analysis. Missing = excluded (M = E) analysis also performed.

**Results**: Overall, 250 PWH were included: median age 59 years (interquartile range, IQR 51–65); 65 (26%) female; 141 (56%) switched from RPV/FTC/TAF, 7 (2.8%) from RPV/FTC/TDF, 96 (38.4%) from EFV/FTC/TDF, 3 (1.2%) from NVP+TDF‐FTC, 2 (0.8%) from DOR‐3TC‐TDF and 1 (0.4%) from NVP+TAF‐FTC (Table 1). At 12 months, 221/250 (88.4%) PWH had HIV‐RNA <50 cp/mL, 19/250 (7.6%) had no viral load (VL) measurement and 10/250 (4.0%) had HIV‐RNA ≥50 cp/mL. In the M = E analysis, 221/231 (95.7%) had HIV‐RNA <50 cp/mL. At 24 months, 187/221 (84.6%) had HIV‐RNA <50 cp/mL, 33/221 (14.9%) had no VL measurement and 1/221 (0.5%) had HIV‐RNA ≥50 cp/mL. In the M = E analysis, 187/188 (99.4%) had HIV‐RNA <50 cp/mL. Fifteen treatment‐discontinuations occurred: simplification (*n* = 4), CNS symptoms (*n* = 4), pregnancy (*n* = 1), epigastric pain (*n* = 1), change of centre (*n* = 1), weight gain (*n* = 1), unknown (*n* = 1) and death (*n* = 2); all but one had HIV‐RNA <50 cp/mL at last determination. Four PWH had virological failure, all due to lack of adherence; all resuppressed viral load without changes and no resistance mutations were detected. Among those switching from RPV‐FTC‐TAF (*n* = 141), at 12 months, 119/141(84.4%) PWH had HIV‐RNA <50 cp/mL, 17/141(12.1%) had no VL measurement and 5/141 (3.5%) had HIV‐RNA ≥50 cp/mL. In the M = E analysis, 119/124 (95.9%) had HIV‐RNA <50 cp/mL. At 24 months, 90/112 (80.4%) PWH had HIV‐RNA <50 cp/mL, 22/112 (19.6%) had no VL measurement and 0 had HIV‐RNA ≥50 cp/mL. In the M = E analysis, 90/90 (100%) had HIV‐RNA <50 cp/mL.

**Conclusions**: In this old treatment‐experienced cohort, switching to B/F/TAF from NNRTI‐based regimens, including RPV/FTC/TAF, maintained virological suppression through 24 months, supporting continued effectiveness in this ageing population. Virological failure was uncommon and treatment discontinuations rare. No resistance mutations detected.

**TABLE 1:**
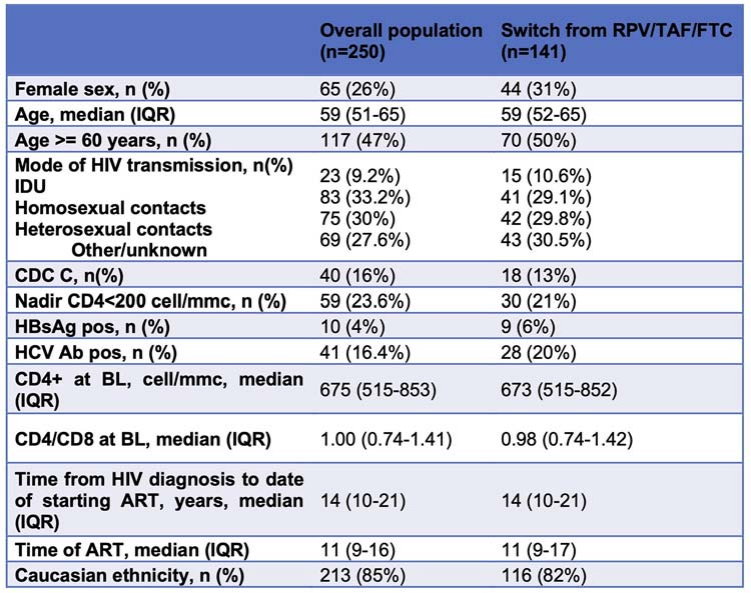
P170 | Main characteristics of PWH included in the study.

### P171 Clustering of HIV‐1 Isolates Based on Index Mutations Detected in Protease and Integrase Region: Results From PRESTIGIO Registry

Sabrina Bagaglio^1^, Nicolò Capra^1^, Riccardo Lolatto^1^, Tommaso Clemente^1^, Stefano Bonora^2^, Maurizio Celesia^3^, Giulia C. Marchetti^4^, Adriana Cervo^5^, Antonio Di Biagio^6^, Giovanni Cenderello^7^, Maria Mercedes Santoro^8^, Maurizio Zazzi^9^, Antonella Castagna^1,10^, Vincenzo Spagnuolo ^1,10^, on behalf of PRESTIGIO Study Group

^1^Unit of Infectious Diseases, IRCCS San Raffaele Hospital, Milan, Italy; ^2^Dept of Medical Sciences, University of Torino Medical School, Turin, Italy; ^3^Unit of Infectious Diseases, Department of Clinical and Experimental Medicine, ARNAS Garibaldi Hospital, Catania, Italy; ^4^Clinic of Infectious Diseases, Department of Health Sciences, San Paolo Hospital, Milan, Italy; ^5^Infectious Diseases Clinic, University Hospital of Modena, Modena, Italy; ^6^Department of Specialist Medicine, Infectious Disease Clinic, IRCCS Ospedale Policlinico San Martino, Genoa, Italy; ^7^Infectious Disease Unit, Sanremo Hospital, Sanremo, Italy; ^8^Department of Experimental Medicine, University of Rome ‘Tor Vergata’, Rome, Italy; ^9^Department of Medical Biotechnology, University of Siena, Siena, Italy; ^10^Vita‐Salute San Raffaele University, Milan, Italy

**Background**: In highly treatment‐experienced people (HTE), HIV therapeutic options are limited by accumulated resistance. RT/PR and integrase sequences from PRESTIGIO Registry including people with HIV (PWH) with resistance to NRTIs, NNRTIs, PIs and INSTIs were analysed to identify clusters not driven by NRTI/NNRTI/PI and INSTI resistance‐associated mutations and assess their association with clinical and immune features.

**Methods**: Clusters were defined identifying index mutations present in most sequences of a group. Continuous variables were expressed as medians (IQR) and compared using non‐parametric tests; categorical as *n* (%) and compared with Fisher's exact or chi‐square. Holm–Bonferroni correction was applied to pairwise comparisons. NRTI/NNRTI/PI and INSTI scores were assigned with Stanford algorithm v9.8.

**Results**: The analysis of RT/PR sequences from 87 PWH allowed to identify 3 sequence clusters: Group A: index mutation = I62V, *n* = 30; Group B: index mutation = Q58E, *n* = 18; Group C, sequences not clustering, *n* = 39. RT/PR analysis compared Group C with the one that included all the other clusters (Group A+, Group B) and no differences emerged. Considering the individual clusters, general differences emerged in CD4 nadir (*p* = 0.008) and marginally CD4 cell count (*p* = 0.064) (Table 1). The analysis of integrase sequences from 89 PWH allowed to identify 5 sequence clusters: Group A: index mutation = S119T/R/P, *n* = 12; Group B, not clustering sequences, *n* = 46; Group C: index mutation = S17N, *n* = 6; Group D: index mutation = V72I, *n* = 15; Group E: index mutation = M154I, *n* = 10. Comparing Group B with the group that included all the other clusters (Group A+, Group C+, Group D+, Group E), lower INSTI resistance scores were observed in Group B (*p* = 0.002), and PWH were more likely to be treated with bictegravir (12% vs. 0%; *p* = 0.05) (Table 1). Breaking down the individual clusters, general differences emerged in CD8 cell count (*p* = 0.038), INSTI resistance scores, the presence of previous major cardiovascular events (*p* = 0.036), the mode of HIV acquisition (*p* = 0.005); results detailed in Table 1.

**Conclusions**: In PRESTIGIO Registry, distinct RT/PR and integrase clusters were identified independently of resistance‐associated mutations. Cluster distribution correlated with immune status and, for integrase, currently used INSTI. ART pressure may drive index mutations not directly tied to resistance, but maintained for fitness, in HTE individuals.

**TABLE 1:**
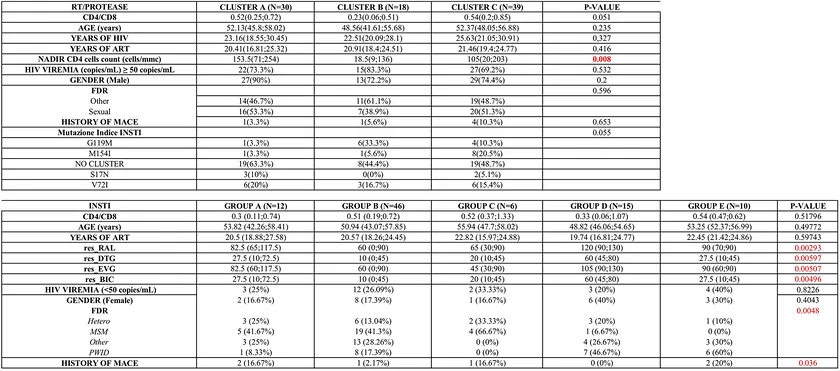
P171 | PWH characteristics according to different clusters detected in RT/protease and integrase regions.

### P172 Real‐World Prescribing in M184V With Additional NRTI Resistance: Heterogeneity in Practice and the Need for Wider Cohort Data

Ciara Hickey^1^, Paul Hine ^2^

^1^University of Liverpool, Liverpool, UK; ^2^Liverpool University Hospitals Group, Liverpool, UK

**Background**: Management of HIV drug resistance is increasingly complex as people living with HIV accumulate historical mutations. BHIVA provides guidance for isolated mutations, but there is limited evidence to guide management of combined nucleoside reverse transcriptase inhibitor (NRTI) resistance, including M184V with additional mutations such as K65R. We evaluated real‐world prescribing and virological outcomes in people living with HIV with M184V and additional NRTI resistance.

**Methods**: We conducted a retrospective service evaluation using electronic patient records from a multi‐site HIV service across Cheshire and Merseyside (cohort ∼2600 patients). We identified patients with documented M184V and at least one additional NRTI mutation, including K65R. We collected demographic, resistance and antiretroviral therapy (ART) data and classified virological outcomes using predefined viral load categories. We performed descriptive analysis using Excel.

**Results**: We identified 26 patients with M184V and additional NRTI resistance. 92% achieved viral suppression. Prescribing varied substantially. Some patients received intensified regimens combining two recycled NRTIs with an INSTI and a boosted PI. Others remained on two‐NRTI plus INSTI regimens without additional agents. A further group received NRTI‐sparing regimens (INSTI with a boosted PI). A small number received individualized approaches, including entry inhibitor‐containing regimens or long‐acting injectable therapy. When stratified by thymidine analogue mutation (TAM) burden (Table 1), regimen choice remained heterogeneous. Similar resistance profiles were managed with different strategies and similar regimens were used across different TAM burdens, suggesting no consistent local approach to retaining, recycling or omitting NRTIs. Some patients with TAMs remained virologically suppressed on two‐NRTI plus INSTI regimens (e.g. bictegravir‐based therapy), although numbers were small and interpretation was limited.

**Conclusions**: Given the limited evidence base, clinicians manage M184V with additional NRTI resistance using a range of antiretroviral strategies, including differing approaches to retaining, recycling or omitting NRTIs. Despite this variability, virological outcomes were generally good. However, current data do not define when two‐NRTI plus integrase inhibitor regimens are sufficient or when intensified or NRTI‐sparing strategies are required. Clarifying this may allow more confident use of simpler regimens and reduce reliance on boosted protease inhibitors where these may not be necessary. Larger UK‐wide real‐world datasets are needed to describe current practice, assess outcomes and inform evidence‐based guidance for people with complex NRTI resistance.

**TABLE 1:**
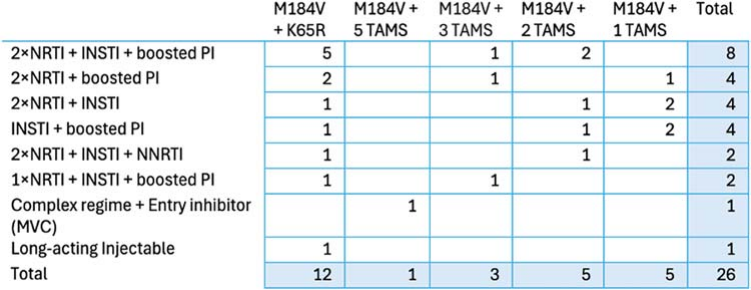
P172 | Antiretroviral regimen by NRTI resistance profile and TAM burden.

## Treatment Strategies: Models of Care for Ageing/Frail Populations Including Virological Failure and Switching

### P173 Healthcare Trajectories Among Ageing People Living With HIV: A Multistate Analysis From the Swiss HIV Cohort Study

Annalisa Marinosci ^1^, Marcel Stoeckle^2^, Matthias Cavassini^3^, Roger Kouyos^4^, Marius Zeeb^4^, Bernard Surial^5^, Andri Rauch^5^, Patrick Schmid^6^, Luigia Elzi^7^, Aurore Thierry^8^, Claire Coutureau^8^, Alexandra Calmy^1^, Maxime Hentzien^9^

^1^HIV Unit, Division of Infectious Diseases, Geneva University Hospitals, Geneva, Switzerland; ^2^Division of Infectious Diseases and Hospital Epidemiology, University Hospital Basel, Basel, Switzerland; ^3^Infectious Diseases Service, Lausanne University Hospital, Lausanne, Switzerland; ^4^Institute of Medical Virology and University Hospital Zurich, Zurich, Switzerland; ^5^Department of Infectious Diseases, Bern University Hospital, Bern, Switzerland; ^6^Division of Infectious Diseases and Hospital Epidemiology, St Gallen, Switzerland; ^7^Division of Infectious Diseases, Regional Hospital Lugano, Lugano, Switzerland; ^8^Methodology Support Unit, University Hospital of Reims, Reims, France; ^9^Department of Infectious Diseases, University Hospital of Reims, Reims, France

**Background**: As the population of people living with HIV (PLWH) ages, understanding ageing‐related healthcare trajectories is critical. We aimed to describe care trajectories among PLWH aged ≥60 years and factors associated with healthcare transitions [1−5].

**Methods**: We analysed participants from the Swiss HIV Cohort Study aged ≥60 years, receiving antiretroviral therapy for > 1 year between 2011 and 2024. Trajectories were reconstructed using a multistate framework including mutually exclusive states (home, hospitalization, institutionalization, death). Cumulative incidence functions were used to describe the first observed event after study entry, considering first hospitalization, institutionalization and death as competing events. Transition‐specific Cox models within a clock‐forward Markov multistate framework identified determinants of selected transitions.

**Results**: Among 3811 participants, mainly men and of white ethnicity, median age at study entry was 60.4 years (Table 1). 1832 participants (48.1%) experienced at least one hospitalization, 172 (4.5%) at least one institutionalization and 457 (12.0%) died. Participants contributed 23,569 person‐years of follow‐up, of which 97.4% were spent at home, 1.8% in institutions and 0.8% in hospital. In competing‐risk analyses, hospitalization was the predominant first healthcare event after study entry with an estimated cumulative incidence of 76.1% at 14 years, compared with 5.3% for death and 2.9% for first institutionalization (Figure 1). In the adjusted model for the home‐to‐hospitalization transition, higher transition intensity was associated with older age (HR per 5 years 1.28, 95% CI: 1.21–1.35), higher Dat'AIDS score (HR per 10 points 1.26, 95% CI: 1.16–1.37), previous hospitalizations (HR per additional hospitalization 1.12, 95% CI: 1.09–1.15), current smoking (HR 1.26, 95% CI: 1.10–1.45) and polypharmacy (HR 1.25, 95% CI: 1.08–1.44), whereas higher education (HR 0.84, 95% CI: 0.75–0.94) and weekly physical activity (HR 0.84, 95% CI: 0.76–0.92) were associated with lower transition intensity. Transition from home to institutionalization was associated with older age (HR per 5 years 1.55, 95% CI: 1.31–1.84), previous hospitalizations (HR per additional hospitalization 1.11, 95% CI: 1.05–1.18), higher Dat'AIDS score (HR per 10 points 1.48, 95% CI: 1.13–1.93) and living alone (HR 1.68, 95% CI: 1.12–2.54).

**Conclusions**: Hospitalization was the predominant first healthcare event after study entry. Older age, comorbidities, healthcare utilization and social vulnerability were major determinants of transitions in healthcare trajectories, supporting targeted preventive interventions.

**References**

1. Gillis J, Loutfy M, Bayoumi AM, Antoniou T, Burchell AN, Walmsley S, et al. A multi‐state model examining patterns of transitioning among states of engagement in care in HIV‐positive individuals initiating combination antiretroviral therapy. J Acquir Immune Defic Syndr. 15 Dec 2016;73(5):531‑9. doi:10.1097/QAI.0000000000001109 PubMed PMID: 27851713; PubMed Central PMCID: PMC5119642.

2. Siddiqi KA, Ostermann J, Zhang J, Khan MM, Olatosi B. Ageing with HIV in the US: changing trends in inpatient hospital stays and comorbidities, 2003−2015. HIV Med. Jan 2023;24(1):93‑103. doi:10.1111/hiv.13325 PubMed PMID: 35674220.

3. Blitz S, Antoniou T, Burchell A, Walmsley S, Light L, Gardner S, et al. The use of multistate models to examine associations of stress and adherence with transitions among HIV care states observed in a clinical HIV cohort. J Acquir Immune Defic Syndr. 1 Nov 2017;76(3):303‑10. doi:10.1097/QAI.0000000000001493 PubMed PMID: 28700406.

4. Fleming J, Berry SA, Moore RD, Nijhawan A, Somboonwit C, Cheever L, et al. U.S. hospitalization rates and reasons stratified by age among persons with HIV 2014−15. AIDS Care. Nov 2020;32(11):1353‑62. doi:10.1080/09540121.2019.1698705 PubMed PMID: 31813269.

5. Hentzien M, Dramé M, Delpierre C, Allavena C, Cabié A, Cuzin L, et al. HIV‐related excess mortality and age‐related comorbidities in patients with HIV aged ≥60: a relative survival analysis in the French Dat'AIDS cohort. BMJ Open. 25 Jan 2019;9(1):e024841. doi:10.1136/bmjopen‐2018‐024841 PubMed PMID: 30782744; PubMed Central PMCID: PMC6352790.

**FIGURE 1:**
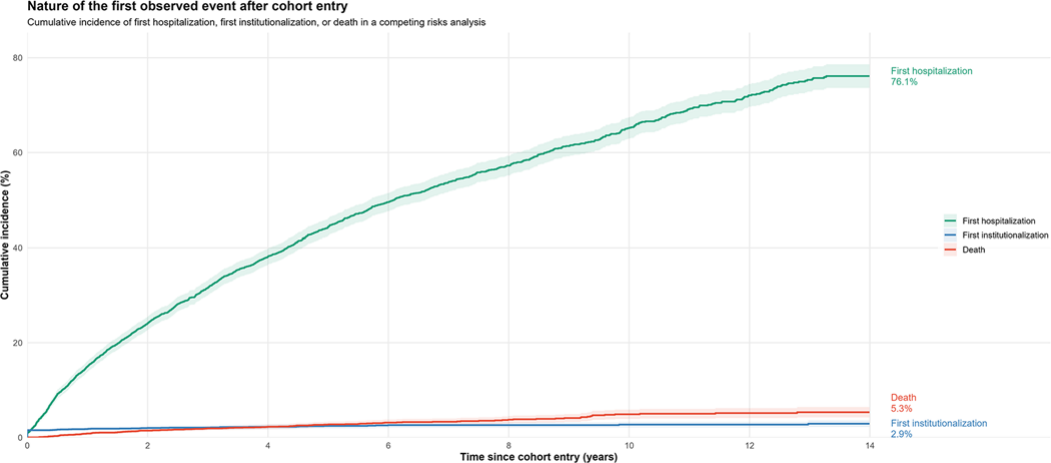
P173 | Cumulative incidence of the first observed event after cohort entry. Curves show the estimated cumulative incidence of first hospitalization, first institutionalization and death, considered as mutually exclusive competing events, among 3811 people living with HIV aged ≥60 years. Shaded areas represent 95% confidence intervals.

**TABLE 1:**
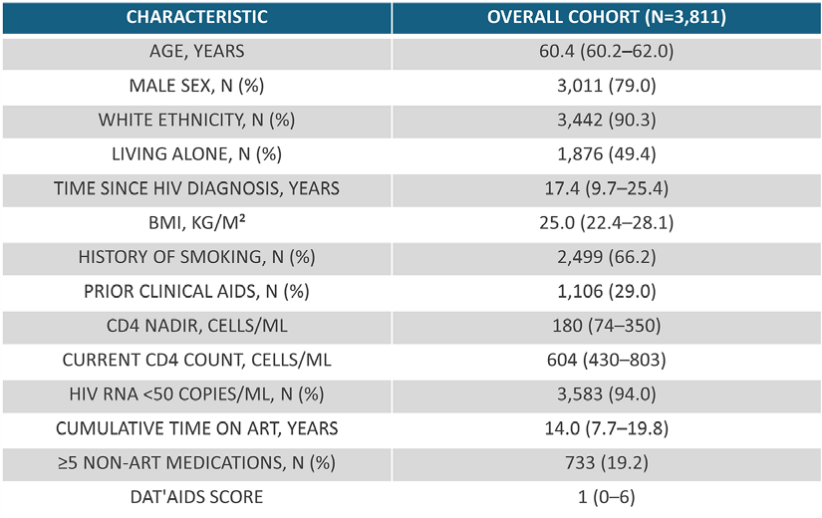
P173 | Baseline characteristics of participants at cohort entry.

### P174 24‐Month Effectiveness and Persistence After Switching to Bictegravir/Emtricitabine/Tenofovir Alafenamide (BIC/FTC/TAF) in People With HIV With Comorbidities and Polypharmacy

Maria Vittoria Cossu ^1^, Andrea Giacomelli^1,2^, Davide Moschese^1,2^, Letizia Oreni^1^, Maddalena Matone^1^, Giacomo Pozza^1^, Giorgia Carrozzo^1^, Annalisa Bergna^1^, Andrea Poloni^1^, Valeria Colombo^1^, Anna Lisa Ridolfo^1^, Agostino Riva^1,2^, Laura Albini^3^, Gabriele Forcina^3^, Cristina Gervasoni^1^, Andrea Gori^1,2^

^1^Department of Infectious Diseases, ASST‐Fatebenefratelli Sacco, Regional Center for Infectious Diseases (CEREMI, Milan, Italy; ^2^Department of Biomedical and Clinical Sciences, University of Milan, Milan, Italy; ^3^Gilead Sciences Italia, Milan, Italy

**Background**: As people living with HIV (PWH) age and accumulate comorbidities and polypharmacy, antiretroviral treatment optimization becomes increasingly challenging. BIC/FTC/TAF, which combines a high genetic barrier with a favourable tolerability profile, may represent a valid option for long‐term regimen optimization. We assessed 24‐month outcomes after switching to BIC/FTC/TAF in this setting.

**Methods**: ESSENTIAL (GS‐IT‐380‐7059) is a single‐centre, retrospective study conducted at ASST Fatebenefratelli Sacco, Milan, Italy. Virologically suppressed (HIV‐RNA <50 copies/mL) PWH switching to BIC/FTC/TAF (July 2019 and April 2024) were included. Month 24 outcomes included maintenance of virological suppression and treatment persistence (time to discontinuation for any reason). Virological outcomes were assessed in participants with available HIV‐RNA at month 24. Persistence was estimated using Kaplan–Meier method in the full cohort (censored at last visit), with exploratory comparisons by baseline regimen using the log‐rank test.

**Results**: A total of 799 PWH were included (median age 53 years, IQR 45–58); 82.6% had ≥1 comorbidity and 34.2% were receiving ≥3 comedications (Table 1). More than half switched from NNRTI‐, boosted PI‐ or abacavir‐based regimens. The main reason for switching was to increase genetic barrier. At month 24, virological suppression was maintained in 97.9% (95% CI: 96.8–99.1; 569/581) of participants with available HIV‐RNA. Median CD4 cell count was 744 cells/mm^3^ (IQR 520–986). Median LDL cholesterol decreased from 124 mg/dL at baseline to 113 mg/dL at month 24. Treatment persistence was 85.7% (95% CI: 83.1–88.3) and was comparable between participants switching from RPV/FTC/TAF and other regimens (87.0% vs. 85.5%, *p* = 0.604). Discontinuations (*n* = 70, 8.8%) were primarily driven by switches to alternative antiretroviral regimens (*n* = 38, 4.8%), particularly long‐acting injectable therapies, whereas discontinuations due to adverse events, drug−drug interactions (DDIs) or virological failure were uncommon (*n* = 8) (Figure 1).

**Conclusions**: In this observational cohort of PWH with comorbidities and polypharmacy, switching to BIC/FTC/TAF from various regimens, including RPV/FTC/TAF, was associated with durable virological suppression and high persistence over 24 months. Discontinuations related to adverse events, DDIs or virological failure were uncommon. These findings suggest that BIC/FTC/TAF may represent a long‐term optimization option in this clinical setting.

**FIGURE 1:**
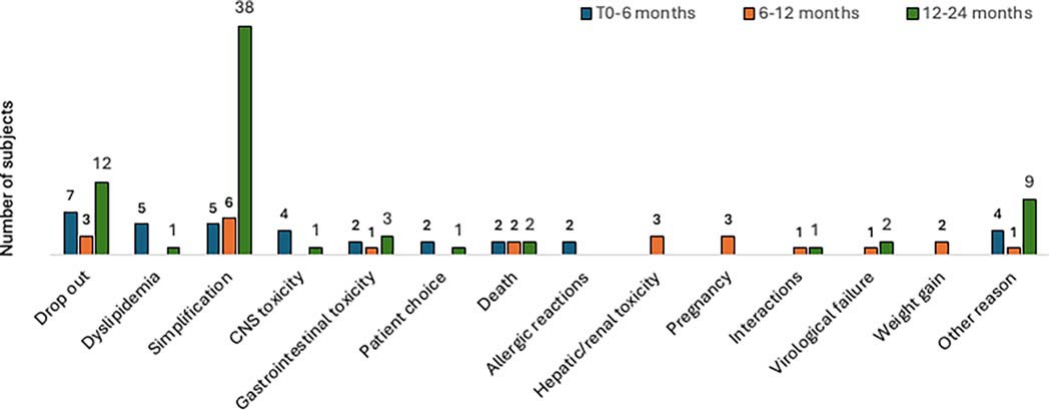
P174 | Causes of BIC/FTC/TAF discontinuation over time, stratified by follow‐up visit (months 6, 12 and 24).

**TABLE 1:**
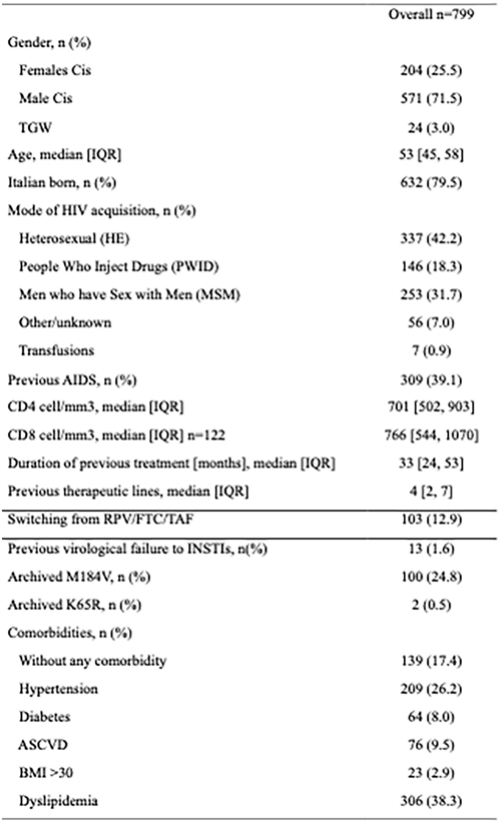
P174 | Baseline characteristics at the time of switch.

### P175 Real‐World Effectiveness of Bictegravir/Emtricitabine/Tenofovir Alafenamide in People Living With HIV Experiencing Socioeconomic Precarity in France

Roland Landman^1,2^, Lambert Assoumou^3^, Aïda Benalycherif^2^, Colin Deschanvres^4^, Marc Digumber^1^, Antoine Asquier Khati^5^, Sylvie Legac^1^, Thomas Jovelin^5^, Aliou Balde^3^, Jade Ghosn^1^, Clotilde Allavena ^5^

^1^IAME, UMR 1137, INSERM, Université Paris Diderot, Sorbonne Paris Cité, AP‐HP, Hôpital Bichat, AP‐HP, Infectious and Tropical Diseases, Paris, France; ^2^Institut de Médecine et d'Epidémiologie Appliquée, IMEA, Hôpital Bichat, Université Paris 7, Paris, France; ^3^Sorbonne Université, INSERM 1136, Institut Pierre Louis d'Epidémiologie et de Santé Publique, Paris, France; ^4^Department of infectious diseases, INSERM U1413, CHU Nantes, Nantes, France; ^5^Department of Infectious Diseases, INSERM U1413, CHU Nantes, Nantes, France

**Background**: Socioeconomic precarity can undermine long‐term antiretroviral therapy (ART) adherence and retention in care, even in settings with universal ART access. In such populations, antiretroviral regimens with a high forgiveness and genetic barrier to resistance are of particular clinical relevance. We assessed real‐world effectiveness of bictegravir/emtricitabine/tenofovir alafenamide (B/F/TAF) among people living with HIV (PLWH) experiencing precarity in France.

**Methods**: Retrospective observational cohort from two French HIV centres. Adults initiating first‑ or second‑line B/F/TAF between 2019 and 2022 were included if covered by France's health insurance schemes for vulnerable populations: C2S (annual income below €10,339 for a single‑person household) or AME (irregular/undocumented migrants). The primary endpoint was HIV‑1 RNA <50 copies/mL at week 48 (± 8 weeks) using the FDA Snapshot approach. Secondary endpoints included confirmed virological failure (CVF; ≥200 copies/mL), low‑level viraemia (LLV; 50– < 200 copies/mL) and treatment discontinuation.

**Results**: Overall, 320 PLWH were included (treatment‑naïve [TN] *n* = 166; virologically suppressed treatment‑experienced [VS‑TE] *n* = 93; virologically unsuppressed treatment‑experienced [VU‑TE] *n* = 61). See Table 1 for demographics. Week‑48 Snapshot suppression was 62.5% overall. Snapshot non‑response was predominantly driven by retention‑related categories, including missing week‑48 viral load (26.9%) and discontinuation for other reasons (22.2%), compared with HIV‑1 RNA ≥50 copies/mL at week 48 (10.6%) and discontinuation due to adverse events (3.4%). Outcomes by treatment subgroup (TN, VS‑TE and VU‑TE) are presented in Figure 1. Among the 234 participants retained on B/F/TAF at week 48, virological suppression was achieved in 200 (85.5%). CVF was uncommon (3.1%) and LLV occurred in 13.1%. Among 10 CVF cases, no emergent resistance to B/F/TAF was observed.

**Conclusions**: In a cohort characterized by migration and high socioeconomic vulnerability, B/F/TAF showed low rates of confirmed virological failure and no emergent B/F/TAF resistance across first‐ and second‐line use, despite anticipated adherence challenges. However, high rates of treatment discontinuation and loss to follow‐up substantially limited Snapshot‐based virological suppression, underscoring that pharmacologically robust regimens must be combined with strategies that improve retention in care in populations experiencing socioeconomic vulnerability.

**FIGURE 1:**
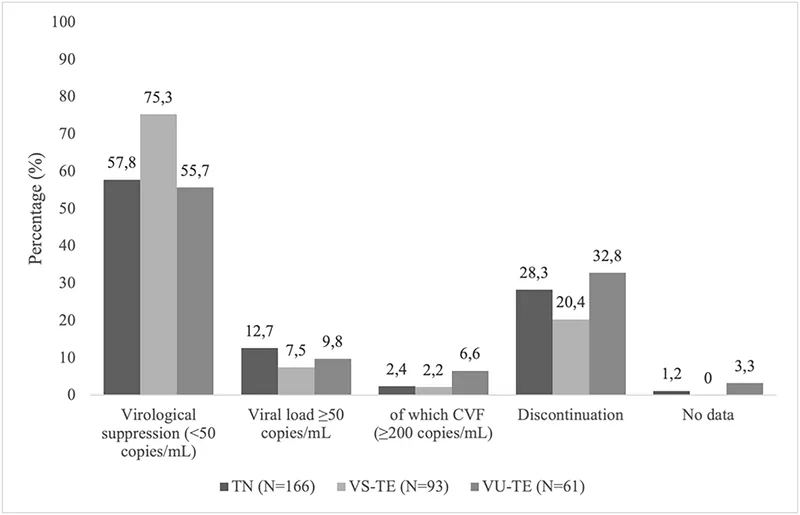
P175 | Outcomes by treatment subgroup (TN, vs. TE and VU TE).

**TABLE 1:**
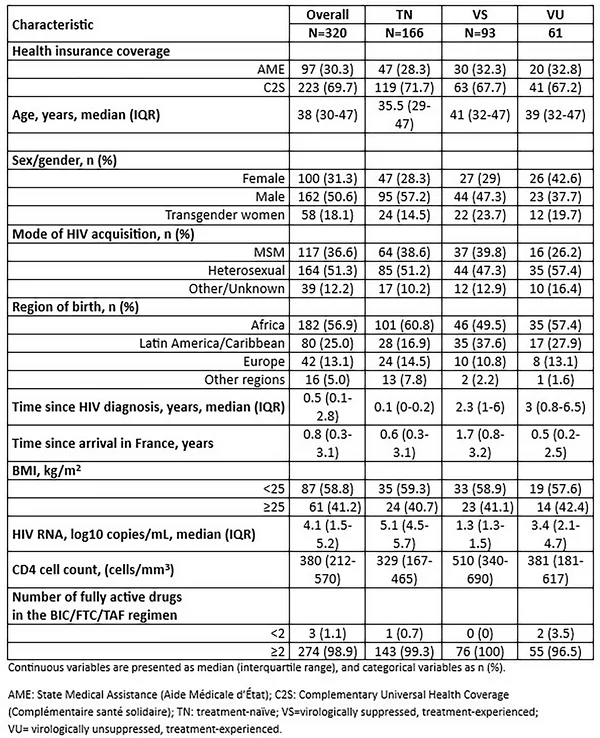
P175 | Demographics and baseline characteristics of the study population.

### P176 Long‑Term Renal Safety of INSTI Single‑Tablet Regimens in Older People Living With HIV

Lolita Sasset ^1^, Francesco Vladimiro Segala^1^, Beatrice Bragato^1^, Anna Ferrari^1^, Eleonora Vania^1^, Silvia Cavinato^1^, Lucrezia Calandrino^1^, Davide Leoni^1^, Annamaria Cattelan^1,2^

^1^Infectious and Tropical Diseases Unit, Padua University Hospital, Padua, Italy; ^2^Department of Molecular Medicine, University of Padua, Padua, Italy

**Background**: In ageing people living with HIV (PLWH), long‑term renal safety is a key consideration when selecting antiretroviral regimens. Both dolutegravir/lamivudine (DTG/3TC) and bictegravir/emtricitabine/tenofovir alafenamide (B/F/TAF) are widely used in older PLWH, but comparative 5‑year real‑world data on creatinine‑based eGFR are limited. We, therefore, evaluated 5‑year eGFRcr changes in older PLWH maintained on unchanged DTG/3TC or B/F/TAF regimens.

**Methods**: We performed a single‐centre retrospective cohort study at Padua University Hospital, Italy in older PLWH maintained on an unchanged single‐tablet regimen with DTG/3TC or B/F/TAF from 2021 to 2026. Renal function was assessed as creatinine‐based eGFR (eGFRcr), at both time points using the race‐free CKD‐EPI 2021 equation. The primary outcome was the 5‐year change in eGFRcr. Baseline variables were compared with the Welch *t*‐test, Wilcoxon rank‐sum or χ^2^/Fisher tests. Between‐regimen ΔeGFRcr was estimated by multivariable linear regression adjusted for age, sex, diabetes, hypertension, chronic kidney disease, BMI, nadir CD4, ART duration and t nephrotoxic co‐medication. Analyses were performed in R (two‐sided α = 0.05).

**Results**: 175 PLWH were studied (88 DTG/3TC, 87 B/F/TAF). Mean age 62 years and 44 (25%) women; comorbidities were balanced between arms (Table 1). Compared with DTG/3TC, the B/F/TAF group had longer ART exposure (median 23 vs. 19 years, *p* = 0.004), lower baseline CD4 (547 vs. 651 cells/µL, *p* = 0.012) and more occult HBV infection (48% vs. 31%, *p* = 0.017); baseline eGFRcr was similar (83 vs. 80 mL/min/1.73 m^2^, *p* = 0.2). By 2026, mean eGFRcr was ≈79 mL/min/1.73 m^2^ in both arms (Figure 1); crude 5‐year changes were small (DTG/3TC −1, B/F/TAF −4 mL/min/1.73 m^2^). Adjusted analysis showed a non‑significant greater decline with B/F/TAF (β −3.1 mL/min/1.73 m^2^; 95% CI: −7.3 to +1.0; *p* = 0.14). No covariate independently predicted ΔeGFRcr and 2026 cystatin C was similar between arms (0.93 vs. 0.95 mg/L, *p* = 0.8).

**Conclusions**: These real‑world data suggest both regimens have acceptable renal safety profiles in older PLWH over a 5‑year follow‐up; the modest, non‑significant greater decline observed with B/F/TAF warrants cautious interpretation given baseline imbalances (ART duration, CD4, occult HBV) and the observational design.

**FIGURE 1:**
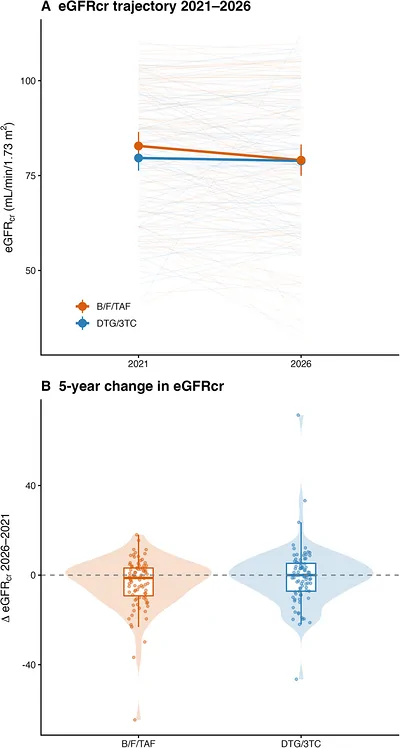
P176 | Estimated creatinine glomerular filtration rate trajectory stratified per study group.

**TABLE 1:**
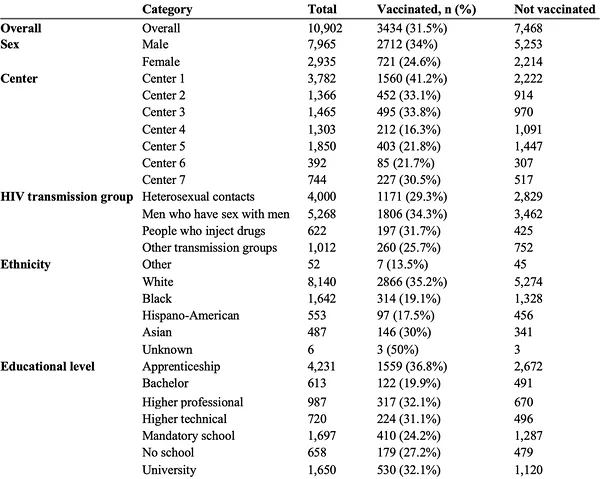
P176 | Baseline characteristics, stratified by regimen.

### P177 Abstract Withdrawn

### P178 Effectiveness of Dolutegravir/Lamivudine According to the Number of Previous Antiretroviral Treatment Lines in People With HIV Older Than 50 Years

Emma Vallribera Olmos ^1^, Marta Escura Esteban^1^, Sonia Calzado Isbert^2^, Maria del Carmen Navarro Sáez^2^, Marc Pedrosa Aragon^2^, Lizza Maria Macorigh Mantilla^3^, Elisabet Deig Comerma^3^, Julia Gutierrez Sanjuan^4^, Rodrigo Alonso Navarro^4^, Oscar Del Rio Perez^5^, Silvia Valero Rovira^5^, Marta Navarro Vilasaró^2^

^1^Parc Taulí Hospital Universitari, Institut d'Investigació i Innovació Parc Taulí (I3PT‐CERCA), Universitat Autònoma de Barcelona, Internal Medicina, Sabadell, Spain; ^2^Parc Taulí Hospital Universitari, Institut d'Investigació i Innovació Parc Taulí (I3PT‐CERCA), Universitat Autònoma de Barcelona, Infectious diseases, Sabadell, Spain; ^3^Hospital General de Granollers, Internal Medicine, Granollers, Spain; ^4^Consorci Sanitari del Maresme, Hospital Universitari de Mataró, Internal Medicine, Mataró, Spain; ^5^Hospital Comarcal Sant Jaume de Calella, Internal Medicine, Calella, Spain

**Background**: People ageing with HIV often accumulate multiple prior lines of antiretroviral therapy (ART) and comorbidities, which may compromise the effectiveness of two‐drug simplification regimens. We assessed whether the number of previous ART lines was associated with persistence, virological effectiveness, co‐medication burden and baseline genotype availability in people aged over 50 years receiving dolutegravir/lamivudine (DTG/3TC, Dovato).

**Methods**: A retrospective multicentre cohort study including adult PLWH from four hospitals in Catalonia, Spain (Hospital Parc Taulí, Hospital Calella, Hospital Granollers, Hospital Mataró), aged over 50 years receiving DTG/3TC (*N* = 281; median follow‐up 43 months), 22 treatment‐naïve and 259 treatment‐experienced, stratified by number of previous ART lines (1 line, *n* = 40; 2–4 lines, *n* = 158; more than 4 lines, *n* = 29). Persistence, sustained virological suppression (always <50 copies/mL vs. blip, persistent viraemia, failure or discontinuation) and comedication burden (comorbidities, concomitant drugs) were compared using chi‐squared and Kruskal–Wallis tests.

**Results**: Persistence on DTG/3TC was similar across stratified groups (87.5% vs. 86.5% vs. 82.8%; *p* = 0.83), as was sustained virological suppression (77.5% vs. 85.7% vs. 82.8%; *p* = 0.43), remaining high even in heavily pre‐treated patients. Co‐medication burden increased progressively with the number of previous lines: median concomitant drugs rose from 1.5 to 3.0 to 5.0 and multimorbidity (2 or more comorbidities) was numerically more frequent with more lines (60.0% vs. 51.9% vs. 65.5%; *p* = 0.31). Baseline genotype was unavailable in an increasing proportion of patients across groups (27.5% vs. 42.4% vs. 55.2%; *p* = 0.06), yet virological suppression was not compromised by missing genotype data (81.4% vs. 86.4%; *p* = 0.37) (Figure 1 and Table 1). Notably, two of the patients who developed integrase resistance mutations (Q148H and G118R were in the 2–4‐line and more‐than‐4‐line groups, respectively) did so only after virological failure on DTG/3TC; neither had documented baseline resistance before switching.

**Conclusions**: In people aged over 50 years, DTG/3TC effectiveness was maintained regardless of the number of previous ART lines, even when baseline genotype was unavailable, supporting its use as a robust simplification strategy in heavily pre‐treated patients. Virological failures were not explained by pre‐existing resistance and greater comedication and comorbidity burden in this ageing population. This warrants comprehensive clinical monitoring beyond virological control.

**FIGURE 1:**
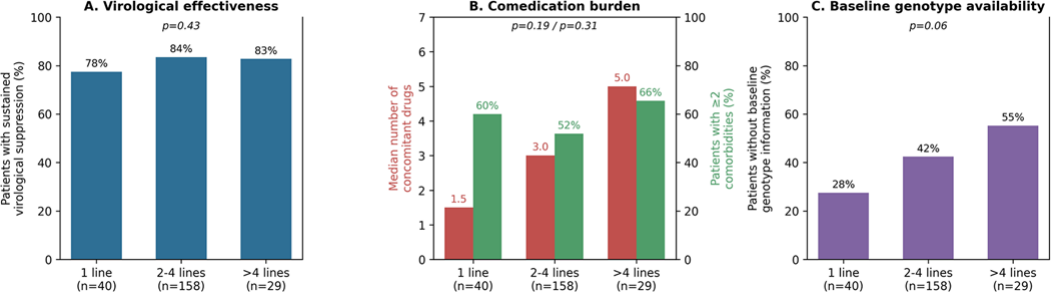
P178 | Virological effectiveness, comedication burden and baseline genotype availability by number of previous ART lines in people aged >50 years receiving DTG/3TC.

**TABLE 1:**
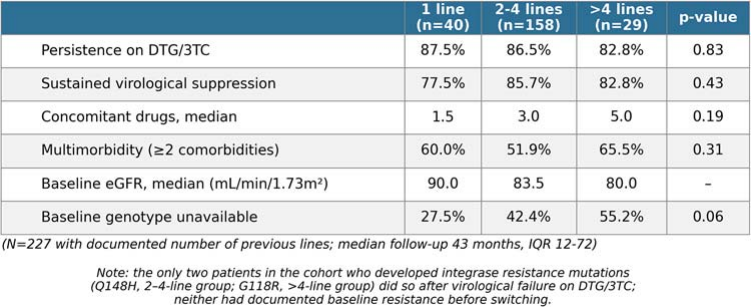
P178 | Outcomes by number of previous ART lines in people aged >50 receiving DTG/3TC.

### P179 Virological and Immunological Outcomes in People Living With HIV and Disabilities: Determinants From a Multicentre Study in China

Lijun Xu ^1^, Ziqing Yuan^2^, Yu He^3^, Ke Hong^4^, Zhen Tang^5^, Jinchun Shi^6^, Li Wang^7^, Guangyong Xu^8^

^1^National Clinical Research Center for Infectious Diseases, the First Affiliated Hospital, Zhejiang University School of Medicine, Hangzhou, China; ^2^Department of Infectious disease, the Second People's Hospital of Fuyang, Fuyang, China; ^3^National Clinical Research Center for Infectious Diseases, The Third People's Hospital of Shenzhen, Shenzhen, China; ^4^Department of Infectious Diseases, JinyinTan Hospital, Wuhan, China; ^5^Department of Infectious Diseases, The Peoples' Third Hospital of Yichang, Yichang, China; ^6^Department of Infectious Diseases II, Xixi Hospital of Hangzhou, Hangzhou, China; ^7^Department of Infectious Diseases, The provincial Hospital of Heilongjiang, Heilongjiang, China; ^8^Qingdao Public Health Clinical Center, Qingdao, China

**Background**: Despite remarkable progress in ART, treatment outcomes among people living with HIV and disabilities have received limited attention, particularly regarding the influence of disability and psychosocial factors on virological suppression and immune recovery.

**Methods**: We conducted a multicentre cross‐sectional study by QR code survey between April and June 2026 in China. Disability, psychological status, social support and quality of life were assessed using the Washington Group Short Set of Questions (WGSS), Patient Health Questionnaire‐4 (PHQ‐4), Social Support Questionnaire (SSQ) and World Health Organization Quality of Life (WHOQOL), respectively. Multivariable logistic regression analyses were performed to identify factors associated with virological failure (HIV RNA ≥50 copies/mL after ≥1 year of ART) and immune recovery (CD4 ≥350 cells/µL after ≥2 years of ART).

**Results**: A total of 345 PLWHD were enrolled, with a median age of 45.0 (IQR, 35.5–62.0) years and a median ART duration of 6.0 (IQR, 3.0–10.0) years. Among participants receiving ART for more than 1 year (*n* = 320), 15.0% exhibited virological failure, 68.1% achieved virological suppression and 16.9% were unaware of HIV‐RNA status. Among those receiving ART for at least 2 years (*n* = 298), 53.0% achieved immune recovery (CD4 ≥350 cells/µL). Multivariable analysis demonstrated that poor ART adherence (β 0.079 [95% CI: 0.017–0.142], *p* = 0.013) and lower WHOQOL environmental scores (β:0.997 [95% CI] 0.994–0.999], *p* = 0.009) were independently associated with virological failure. Better immune recovery was independently associated with lower disability severity of WGSS score (β: 0.974 [95% CI: 0.956–0.992], *p* = 0.005), higher WHOQOL general health scores (β: 1.003 [95% CI: 1.001–1.006], *p* = 0.004) and the absence of mental disorders (β: 0.683 [95% CI: 0.380–0.986], *p* = 0.041).

**Conclusions**: PLWHD exhibited increased virological failure and poor immune recovery rate despite long‐term ART. ART adherence, disability severity, mental health and quality of life independently influenced treatment outcomes. Comprehensive interventions integrating adherence support, disability management, mental healthcare and social support may improve long‐term HIV outcomes in this vulnerable population.

### P180 No Significant Sex Differences in Antiretroviral Treatment Among Ageing People Living With HIV—Results From the NONNA Study

Oliver Schmidt‐Radde ^1^, Markus Bickel^2^, Pavel Khaykin^3^, Gundolf Schuettfort^1^, Christoph Stephan^1^, Annette Haberl^1^

^1^Goethe University Frankfurt, University Hospital, Department of Internal Medicine, Infectious Diseases, Frankfurt am Main, Germany; ^2^Infektiologikum, Frankfurt am Main, Germany; ^3^MainFachArzt, Frankfurt am Main, Germany

**Background**: Ageing with HIV is influenced by numerous factors, including sex and gender‐specific factors such as menopause. Following the DONNA study, which has previously identified significant sex differences in antiretroviral treatment (ART) in people living with HIV aged 18–50 [1], the NONNA study aimed to analyse potential sex differences in ART in persons aged 50 and older.

**Methods**: NONNA is a retrospective cohort study conducted at the HIVCENTER Frankfurt, analysing potential sex‐specific differences in the HIV treatment of women and men aged ≥50 years between 2012 and 2023. Virological response rates and CD4‐cell counts were included in the statistical analyses. Sex‐specific differences in antiretroviral treatment patterns and clinical outcomes were assessed using chi‐square tests and odds ratios (ORs).

**Results**: The mean annual cohort size was 1117, including 927 men (83.0%) and 190 women (17.0%). No consistent sex‐based differences in ART prescribing patterns were observed across calendar years, as annual analyses revealed no significant differences for INSTIs (*p* = 0.69), NNRTIs (*p* = 0.95), NRTIs (*p* = 0.51), PIs (*p* = 0.12) or maraviroc‐containing regimens (*p* = 0.31) (Figure 1). NRTIs remained the backbone of ART, while protease inhibitor use declined and integrase inhibitor use increased markedly from 2016 onwards. NNRTI and maraviroc use remained stable. Clinical outcomes improved in both sexes. The proportion with HIV‐1 RNA <50 copies/mL increased from 88.2% to 94.8% among women and from 86.0% to 95.0% among men from 2012 to 2023 (*p* = 0.6). CD4‐cell counts also improved. The proportion with CD4‐cell counts > 500 cells/µL increased from 65.6% to 82.3% among women and from 56.0% to 75.9% among men (*p* = 0.2), while the proportion with CD4 counts < 200 cells/µL decreased from 5.4% to 1.6% among women and from 5.2% to 1.7% among men (*p* = 0.36).

**Conclusions**: In our cohort of people living with HIV aged 50+, there were no significant sex‐specific differences in ART regimens over the observation period from 2012 to 2023. This finding contrasts with the well‐described sex‐specific differences in ageing‐related comorbidities among people living with HIV, including cardiovascular disease and osteoporosis. Taking this into account, future HIV treatment in the ageing population should be guided by individual risk profiles for comorbidities and thus take greater account of sex‐ and gender‐specific factors.

**Reference**

1. Spitzer S, et al. Significant sex differences in antiretroviral treatment of young people living with HIV – results from the DONNA study, 14th International Workshop on Women & HIV. Washington, USA. 12−13 April 2024.

**FIGURE 1:**
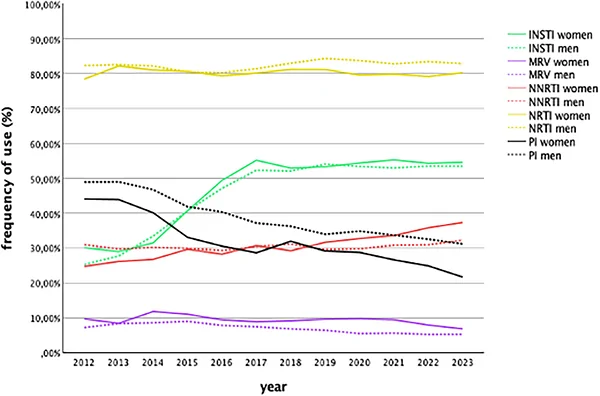
P180 | Trends in ART in people with HIV aged >50 (2012–2023).

### P181 The Burden of Copies: A Retrospective Study of Very Low‐Level Viraemia in People Living With HIV

Carolina Inacio Santos ^1^, Ana Cláudia Miranda^1^, Margarida Veloso^2^, Marta Ribeiro^2^, Sandra Mendes Fernandes^2^, Ana Isabel Duarte^2^, Isabel Diogo^2^, Inês Costa^2^, Fátima Gonçalves^2^, Joaquim Cabanas^2^, João Vaz Alves^1^, Perpétua Gomes^2,3^

^1^Hospital de Egas Moniz, Unidade Local de Saúde de Lisboa Ocidental, Infectious Diseases and Tropical Medicine Department, Lisbon, Portugal; ^2^Hospital de Egas Moniz, Unidade Local de Saúde de Lisboa Ocidental, Molecular Biology Laboratory, Lisbon, Portugal; ^3^Egas Moniz Center for Interdisciplinary Research (CiiEM), Egas Moniz School of Health & Science, Lisbon, Portugal

**Background**: To characterize the clinical and virological profiles of people living with HIV (PLWH) with very low‐level viraemia (VLLV) followed at a Portuguese hospital between 2019 and 2024.

**Methods**: PLWH with HIV‐1 viral loads between 20 and 200 copies/mL were screened. Inclusion required ≥6 months of antiretroviral therapy (ART) and two consecutive measurements > 20 copies/mL, not preceded or followed by values > 1000 copies/mL or two measures between 200 and 1000. Demographic, clinical and therapeutic data were collected.

**Results**: Among 1347 PLWH with detectable viraemia in the target range, 321 met inclusion criteria. Half were Portuguese and approximately one‐third were from African countries. At the time of VLLV detection, the mean age was 49 years old and median infection length was 11 years. Baseline data were frequently incomplete, often due to prior diagnoses made externally or before the implementation of electronic medical records. CD4+ T‐cell counts at baseline were available in 230 patients, of which a quarter had counts above 500 cells/µL and only 9% were under 50 cells/µL. Viral load at diagnosis was known in 172 patients, from which 133 (77%) had viral loads higher than 100,000 copies/mL. Most patients presented undetectable viral loads over time (72%). At the time of VLLV, 70% were on integrase inhibitors, predominantly dolutegravir and 82% of patients reported never missing a dose. Nonetheless, this conclusion is biased since it relies on patient honesty which in turn depends on various variables. Most ART modifications after VLLV were unrelated to detectable viraemia. Cardiovascular comorbidities were present in 50% and various intercurrent infections were recorded during VLLV episodes. However, exact tracking of concurrent clinical situations is biased since it highly depends on written records.

**Conclusions**: VLLV seems to appear during the fifth and sixth decade of life, infections longer than 10 years, suggesting a potential association with disease chronicity, ageing and comorbidities. High initial viral loads may present a risk factor for future VLLV episodes despite suppressive ART. The clinical implications of VLLV remain uncertain.

## Treatment Strategies: Rapid ART Initiation

### P182 Nurse‐Directed Same‐Day Initiation of cART in Persons With a New HIV Diagnosis

Jessica S. van der Mannen, Maaike Kok, Gijs Geerders, Narda van der Meche, Saskia M.E. Vrouenraets, Ineke W.E.M. Schouten, Marieke De Regt, Daoud Ait Moha, Kees Brinkman, on behalf of HIV team OLVG

Department of Internal Medicine, OLVG, Amsterdam, Netherlands

**Background**: Early initiation of combination antiretroviral treatment (cART) has been shown to decrease morbidity and mortality, while also significantly decreasing onwards HIV transmission. Several studies demonstrated that same‐day initiation confers several benefits and might accelerate viral control. In 2019, the OLVG Hospital Amsterdam implemented a standardized clinical HIV care pathway, whereby a specialized HIV nurse offered every newly referred person with HIV (PWH) immediate start of cART at the first clinical visit. In this study, we explore the feasibility of this pathway and its impact on time to viral suppression < 200 copies/mL.

**Methods**: We conducted a retrospective cohort analysis of all newly diagnosed PWH entering care between 2016 and 2024. Baseline characteristics were collected, along with dates of diagnosis, first visit, cART initiation and first viral load <200 copies/mL. We used Cox‐regression and non‐parametric statistical tests to analyse changes before and after pathway implementation. Subsequent analyses focussed on time from diagnosis to intake, long‐term results such as retention in care and adherence and adverse events.

**Results**: A total of 447 individuals were included in the analysis, 89% were men with a median age of 39.7 years at diagnosis. The pre‐implementation group (entering care between 2016 and 2018) included 243 individuals and the post‐implementation group (2020–2024) included 204 individuals. The proportion of PWH initiating cART at the first visit increased from 19% to 65%. In days, the median cART start‐up time after the first visit decreased from 9 (interquartile range 2–20) to 0 (0–2) days (*p* < 0.001). Median time from first visit to viral suppression decreased from 57 (55–63) to 46 (43–50) days (*p* = 0.002). Furthermore, median time from diagnosis to the first visit decreased from 7 (4–11) to 5 (2–9) days (*p* < 0.001). There were no differences between both groups in the overall high numbers of retention in care and adherence (*p* = 0.336). No extra unexpected adverse events were reported in the post‐implementation group.

**Conclusions**: A nurse‐directed implementation of a care pathway favouring rapid cART initiation in a high‐resource clinical setting is highly feasible without compromising on the patient's well‐being. Task‐shifting by nurses led to a faster linkage to care and cART initiation, accelerating viral control.

### P183 Comparison of Dual and Triple Integrase Inhibitor‐Based Regimens Towards the Undetectable=Untransmittable Target

Melike Kaya Kaptan^1^, Alper Gündüz ^1^, Okan Derin^2^, Gözde Kuran^2^, Ece Demirkırkan^1^, Yusuf Yiğit^1^, Esma Kaya Beyendi^1^, Berna Demirok^1^, Özlem Gül^2^, Gizem Çal^2^, Nuran Karabulut^3^, Meliha Meriç Koç^1^, Dilek Yıldız Sevgi^2^, Özlem Altuntaş Aydın^1^

^1^Başakşehir Çam and Sakura City Hospital, Department of Infectious Diseases and Clinical Microbiology, İstanbul, Türkiye; ^2^Şişli Hamidiye Etfal Training and Research Hospital, Department of Infectious Diseases and Clinical Microbiology, İstanbul, Türkiye; ^3^Başakşehir Çam and Sakura City Hospital, Department of Virology, İstanbul, Türkiye

**Background**: The concept of undetectable = untransmittable (U = U) diminishes the stigma associated with HIV and promotes consistent adherence to antiretroviral therapy (ART). This study aimed to compare the rates and speed of viral load suppression of dual and triple integrase‐based regimens in light of U = U criteria.

**Methods**: This retrospective study included adults aged ≥18 years newly diagnosed with HIV between 1 July 2020 and 1 March 2025 and followed for 6 months after ART initiation at two training and research hospitals in Istanbul. Eligible patients had no prior hepatitis B virus exposure, baseline HIV RNA <500,000 copies/mL and no concomitant medication or supplement use. Patients receiving first‐line INSTI+1 NRTI or INSTI+2 NRTIs were included. Data were obtained from hospital records and propensity score matching was used to reduce confounding. HIV RNA levels were assessed using the U = U threshold of < 200 copies/mL. Categorical variables were analysed using chi‐square or Fisher's exact tests and repeated measures using the Wilcoxon signed‐rank test.

**Results**: A total of 309 patients were included after 1:2 matching: 103 in the INSTI+1 NRTI group and 206 in the INSTI+2 NRTIs group. HIV RNA < 200 copies/mL was achieved at month 1 in 86.4% and 79.1% of patients, respectively (OR 1.710; *p* = 0.161) and at month 3 in 98.1% and 97.6% (OR 0.97; *p* = 0.571). By month 6, all patients had HIV RNA < 200 copies/mL. Analysis of the variations in baseline and follow‐up viral loads showed no significant difference between the INSTI+1 NRTI and INSTI+2 NRTI groups and both groups were determined to be effective in terms of viral suppression.

**Conclusions**: No significant difference was observed between dual and triple INSTI‐based ART regimens in time to achieve viral suppression below the transmission‐risk threshold. Both strategies were effective, suggesting that in individuals with baseline HIV RNA <500,000 copies/mL, early ART initiation may be more critical than regimen selection for achieving suppression sufficient to eliminate transmission risk.

### P184 Real‐World Treatment Pattern Changes and Time to Virological Suppression Among People Living With HIV in China

Hanxi Zhang ^1^, Hongxin Zhao^1^, Lijun Sun^2^, Jianhua Yu^3^, Fujie Zhang^1^

^1^Beijing Ditan Hospital Capital Medical University, Beijing, China; ^2^Beijing ouan Hospital Capital Medical University, Beijing, China; ^3^Xixi Hospital of Hangzhou, Zhejiang Chinese Medical University, Hangzhou, China

**Background**: The expansion of rapid antiretroviral therapy (ART) initiation and regimen transitions has transformed HIV care, but real‐world evidence on whether these changes have shortened the time to first viral suppression and attenuated the negative impact of treatment delay remains limited in developing countries.

**Methods**: This was a retrospective cohort study of 31,353 people living with HIV who initiated ART between 2003 and 2025. We analysed ART regimen transitions from baseline to current, estimated time to first viral suppression (HIV RNA <50 copies/mL) using Kaplan–Meier and Cox regression and tested the interaction between diagnosis‐to‐ART interval and ART initiation year, with stratification by early (2003–2014) and late (2015–2025) eras.

**Results**: The study population was predominantly male and about 65.0% were aged < 30 years. 16.9% had baseline CD4< 200 cells/µL and 55.7% had baseline viral load > 100,000 copies/mL. NNRTI (non‑nucleoside reverse transcriptase inhibitor)‐based regimens dominated initially (79.8%), as for current ART regimens, 43.1% of them switched to INSTI (integrase strand transfer inhibitor)‐based regimens, raising the proportion of INSTI‐based regimens to 51.5%. Rapid initiation (≤7 days) and initiation within 30 days increased between eras (Figure 1). Median suppression time was shorter for INSTI‐based (126 days) than NNRTI‐based (176 days) and PI‐based (175 days) regimens (*p* < 0.001). Similar findings were observed in the CD4< 200 subgroup (116 days for INSTI). INSTI‐based regimens were associated with faster suppression. Notably, a significant interaction was observed between treatment delay and year of ART initiation (*p* < 0.001). Stratified analysis revealed that in the early era, treatment delay had no significant impact on suppression (aHRs 0.892–1.130, *p* > 0.15); however, in the late era, delayed initiation was associated with significantly slower suppression compared with timely initiation (aHRs 0.660–0.840, *p* < 0.001), indicating that despite overall improvements in suppression times, delayed initiators remained at a relative disadvantage (Table 1).

**Conclusions**: The expansion of rapid ART initiation and the shift to INSTI‐based regimens have substantially shortened the time to viral suppression in the overall population. However, delayed initiation remains a significant risk factor for slower viral suppression.

**FIGURE 1:**
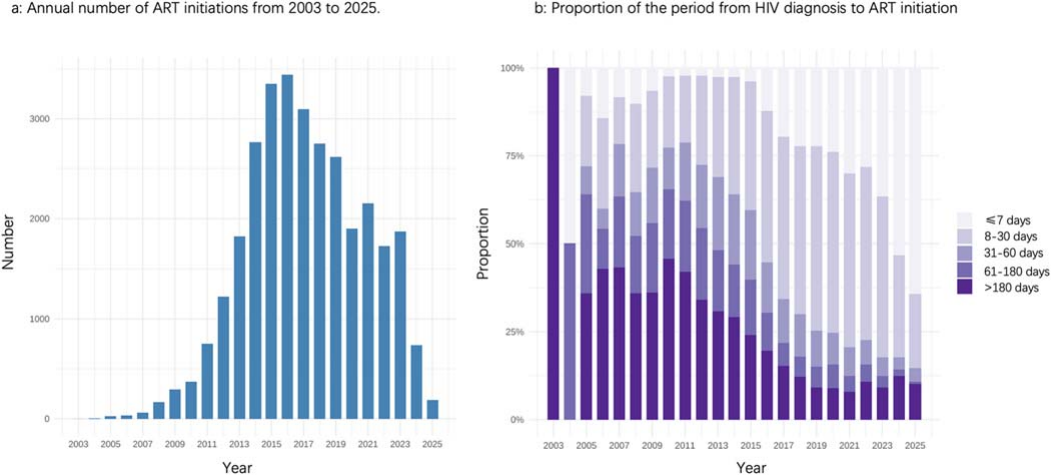
P184 | Annual number of ART initiations and proportion of the interval from HIV diagnosis to ART initiation (period) from 2003 to 2025.

**TABLE 1:**
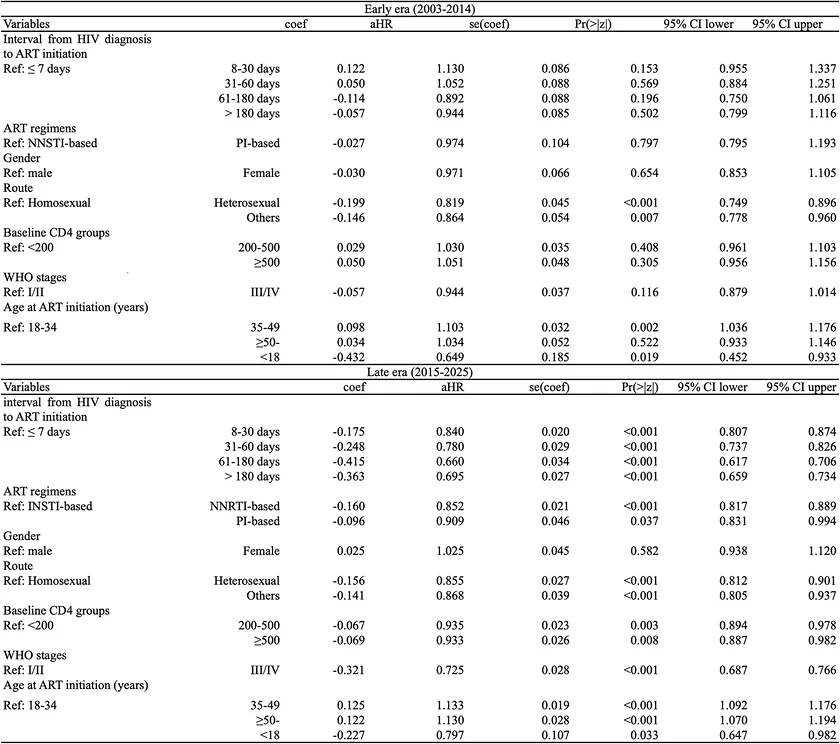
P184 | Multivariate Cox regression model on different eras.

### P185 Effectiveness of DTG/3TC Rapid Initiation in Treatment‐Naïve People Living With HIV in Kuwait: A Real‐World Cohort Study

Osama Ali Ahmad Albaksami ^1^, Essam Mohamed Ali Elhabbak^1^, Ahlam Mohamed Hafez Mohamed Abdalla^2^, Roy Thampy Cherian^1^, Hassan Fathy Sayed Abdelaal^1^, Ibrahim Ashour Hamed Hassan^1^, Evelina Zimovetz^3^, Averyan Vasylyev^3^, Hani Wali^3^

^1^Infectious Diseases Division, Al Jahra Hospital, Al Jahra, Kuwait; ^2^Paediatric Specialist, Al Jahra Hospital, Al Jahra, Kuwait; ^3^GSK Life Sciences FZE, Dubai, UAE

**Background**: Evidence supporting rapid initiation of dolutegravir/lamivudine (DTG/3TC) in test‐and‐treat settings remains limited, particularly in real‐world cohorts. This study described the clinical outcomes, demographics and clinical characteristics of treatment‐naïve people living with HIV who were initiated on a fixed‐dose combination (FDC) of DTG/3TC as part of a test‐and‐treat strategy in Kuwait.

**Methods**: This retrospective chart review evaluated adults with a confirmed diagnosis of HIV‐1 at Kuwait's centralized national HIV care centre (January 2021–September 2024). Eligible persons were antiretroviral therapy (ART)‐naïve and initiated on FDC DTG/3TC on the same day or within 14 days of diagnosis, before any or all of the laboratory results were available. The primary outcome was the proportion of participants achieving HIV‐1 RNA <50 copies/mL at week 52 (FDA Snapshot; missing data/discontinuations = failure). A sensitivity analysis was conducted using an alternative virologic suppression threshold of < 200 copies/mL at week 52. Secondary outcomes included virologic failure (≥200 copies/mL), change from baseline in CD4+ count, treatment persistence and change from baseline in renal parameters.

**Results**: 116 participants were evaluable at week 52 (median age 33.6 years; 90.5% male). At baseline, median CD4+ count was 384 cells/mm^3^ and median viral load (VL) was 46,575 copies/mL. Median time from HIV diagnosis to initiation of DTG/3TC was 7 days (Table 1). At week 52, 78.5% (91/116; 95% CI: 70.1–85.0%) had a VL <50 copies/mL, rising to 92.2% (107/116; 95% CI: 85.8–96.4%) at a VL < 200 copies/mL threshold (Figure 1). Virologic failure was recorded in 4.3% (5/116; 95% CI: 1.4–9.8%), with no treatment‐emergent resistance mutations. Immunologic recovery resulted in a median increase in CD4+ counts of +276 cells/mm^3^ from baseline. By week 52, treatment discontinuation was only 2.6% (3/116) due to non‐virologic reasons (weight gain, pregnancy or patient's decision). Kaplan–Meier analysis showed a 97.4% probability of remaining on treatment at week 52. Renal changes were modest and consistent with known effects; few participants (3.4%) experienced clinically significant declines in eGFR.

**Conclusions**: DTG/3TC demonstrated effectiveness, persistence and tolerability in a real‐world test‐and‐treat setting. These findings support its use for rapid ART initiation, although interpretation is limited by the retrospective single‐centre design.

**FIGURE 1:**
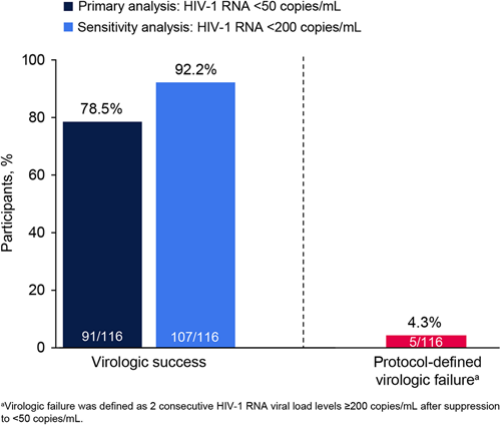
P185 | Virologic outcomes at week 52 (*N* = 116).

**TABLE 1:**
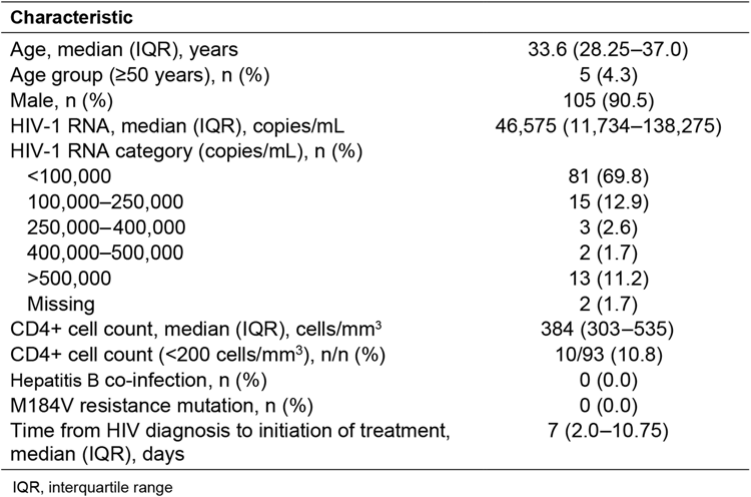
P185 | Demographic and baseline characteristics (*N* = 116).

### P186 Delayed Rapid Antiretroviral Therapy Initiation and Its Sociodemographic and Clinical Correlates: A Secondary Analysis of the ASAP Study in Montreal, Canada

Moustafa Laymouna^1,2^, Kim Engler^2^, David Lessard^2^, Serge Vicente^2,3^, Sean Yaphe^4^, Joseph Cox^4,5^, Nadine Kronfli^2,4^, Jean‐Pierre Routy^4^, Joel Ishak^2^, Alexandra Hamel^4^, Marina Klein^4^, Alexandra De Pokomandy^1,4^, Cecilia T. Costiniuk^4^, Kathy Nodzynski ^1,2^, Bertrand Lebouché^1,2^

^1^Department of Family Medicine, Faculty of Medicine and Health Sciences, McGill University, Montreal, Canada; ^2^Research Institute of the McGill University Health Centre, Montreal, Canada; ^3^Département des Enseignements Généraux, École de Technologie Supérieure, Montreal, Canada; ^4^Department of Medicine, Chronic Viral Illness Service, Division of Infectious Diseases, McGill University Health Centre, Montreal, Canada; ^5^Department of Epidemiology, Biostatistics and Occupational Health, Faculty of Medicine & Health Sciences, McGill University, Montreal, Canada

**Background**: Rapid antiretroviral therapy (ART) programmes aim to shorten the time from HIV care entry to treatment initiation, but initiation timing varies in practice. To help understand why, we examined associations between ART timing and socioeconomic factors, care processes, baseline clinical profiles and HIV‐related outcomes.

**Methods**: We analysed data of ART‐naïve adults living with HIV enrolled in the Montreal Antiretroviral Speed Access Programme (ASAP), which offered no‐cost, onsite bictegravir/emtricitabine/tenofovir alafenamide (B/F/TAF) for 96 weeks. ART timing from first clinic contact was categorized as same‐day, 1–7 days, 8–14 days and >14 days. Sociodemographics included age, gender, education, employment, income, housing, language, children, migration status and health coverage/insurance. The care‐process variable was seeing a physician the day of first clinic contact (yes/no). Baseline clinical variables were binary indicators of CD4s (<200 cells/mm^3^) and viral load (VL) (≥100,000 copies/mL). HIV‐related outcomes were binary VL undetectability (<50 copies/mL) by last available follow‐up and days from ART initiation to first undetectability. Associations with ART timing were assessed using Kruskal–Wallis tests for continuous/count variables and Fisher exact tests for categorical/binary variables.

**Results**: Among 72 participants, 22 (30.6%) initiated ART the same day, 18 (25.0%) within 1–7 days, 9 (12.5%) within 8–14 days and 23 (31.9%) after 14 days. Timing differed by employment (*p* = 0.002), education (*p* = 0.02) and number of children (*p* = 0.04). Participants initiating after 14 days were more often not working (19/22, 86.4%), less often university educated (3/22, 13.6%) and had more children (median 1.5 [Q1–Q3: 0–3]). Other sociodemographics showed no significant association with timing. Seeing a physician on the day of first clinic contact differed by timing (*p* = 0.019): 50.0%, 27.8%, 22.2% and 8.7% in same‐day, 1–7‐day, 8–14‐day and >14‐day groups, respectively, but baseline clinical profiles did not: CD4< 200 cells/mm^3^, 20/70 (28.6%), *p* = 0.57; VL ≥100,000 copies/mL, 26/71 (36.6%), *p* = 0.67. VL undetectability by last follow‐up was almost universal (70/72, 97.2%; *p* = 1.00) and time to first undetectable VL did not differ by timing (median 30.5 days [Q1–Q3: 28.0–71.5]; *p* = 0.74).

**Conclusions**: Later ART initiation was associated with sociodemographic and care process factors. Their further investigation may help optimize ART timing.

### P187 SWIFT: Start With Immediate First‐Line Therapy—Time to Viral Suppression, Baseline Immunovirologic Status and Retention After Rapid ART Initiation at a Toronto Community Sexual Health Clinic

Kevin Woodward ^1^, Tim Guimond^2^, Hommood Alrowais^3^, Victor Monroy^4^, Lucas Gergyek^5^

^1^Executive & Medical Director, HQ Health Hub, Toronto, Canada; ^2^Mental Health Director, HQ Health Hub, Toronto, Canada; ^3^Data Scientist & Privacy Officer, HQ Health Hub, Toronto, Canada; ^4^Research Analyst & Software Developer, Toronto, Canada; ^5^Research Coordinator, HQ Health Hub, Toronto, Canada

**Background**: ART should be initiated as soon as possible after HIV diagnosis, but a gap often separates testing, diagnosis and treatment [1]. Published rapid ART programmes in high‐income settings initiate treatment at a dedicated care visit following diagnosis notification; none has examined initiation within 24–48 h of the testing visit itself, enabled by same‐visit laboratory results. HQ (Toronto, ON) operates an on‐site laboratory enabling same‐visit results and immediate ART initiation with B/F/TAF.

**Methods**: We conducted a retrospective EMR chart review of all individuals newly diagnosed with HIV between July 2022 and December 2025. Time to virologic suppression (HIV‐1 RNA <50 copies/mL), from date of first test, was estimated by Kaplan–Meier. Retention was clinical activity at 6 and 12 months; confirmed transfers to external HIV care counted as retained.

**Results**: 200 individuals were newly diagnosed (median age 31 [IQR 26–36]; ∼93% GBMSM; 86% cisgender; 77% born outside Canada; 60% non‐citizens; 65% without drug coverage; 20% prior methamphetamine use). 192/200 (96%) initiated ART; median time from test to initiation was 1 day (IQR 1–2): 70% within 24 h, 76% within 48 h, 97% within 1 week. Median baseline CD4 was 380 cells/µL (IQR 270–560; 12% < 200, 42% < 350). 198/200 (99%) had detectable viraemia at presentation. Median time to suppression was 38 days (95% CI: 34–53); cumulative suppression 65% at 12 weeks, 89% at 24 weeks, 95% at 48 weeks. Engagement in care was 95% (174/184) at 6 months and 92% (144/157) at 12 months; 10 (5%) and 13 (8%) were lost to follow‐up.

**Conclusions**: On‐site laboratory capacity enabled ART initiation within 24 h of testing in 70% and within 1 week in 97%, compressing the window between testing and viral suppression to a median of 38 days. Initiation was offered to all individuals, with 96% starting ART, 92% engaged in care at 12 months and 95% virologically suppressed by 48 weeks. These findings support on‐site laboratory capacity as a high‐yield investment for test‐to‐treatment ART initiation in high‐risk populations.

**Reference**

1. Koenig SP, Dorvil N, Dévieux JG, Hedt‐Gauthier BL, Riviere C, Faustin M, Lavoile K, Perodin C, Apollon A, Duverger L, McNairy ML, Hennessey KA, Souroutzidis A, Cremieux PY, Severe P, Pape JW. Same‐day HIV testing with initiation of antiretroviral therapy versus standard care for persons living with HIV: a randomized unblinded trial. PLoS Med. 2017 Jul 25;14(7):e1002357. doi: 10.1371/journal.pmed.1002357. PMID: 28742880; PMCID: PMC5526526.

### P188 Rapid HIV Care Linkage in a No‐Cost Testing Setting

Heather Alejandro, Michael Greathouse

Prisma Community Care, Phoenix, AZ, US

**Background**: Over the past decade, Maricopa County has experienced a rise in new HIV diagnoses. Being in the heart of Maricopa County, Prisma Community Care (PCC) has intensified its commitment to providing linkage to care for patients who are newly diagnosed through rapid testing. PCC has implemented a streamlined approach to linkage to care focusing on removing barriers and putting an emphasis on integration.

**Methods**: During a no‐cost clinic visit, prevention specialists offer a 20‐min rapid HIV test to all clients. If a client tests reactive for HIV, immediate connection to a navigator facilitates expedited care, with linkage completed within 5 days of the result. Navigators facilitate appointments for both medical and social support services. Navigators confirm attendance of appointments directly with the care facility and conduct check‐ins with clients until they are connected to a case manager or feel confident.

**Results**: January–December 2025, PCC conducted 2906 rapid HIV tests during no‐cost testing visits. 13 clients received a new HIV diagnosis at time of visit. 11 were linked to medical care and social support services by navigators, while 2 declined navigator assistance and managed linkage independently. Of the 11 linked, 100% attended their first appointment and initiated antiretroviral therapy (ART); confirmed by the medical facility. Among them, 8 were linked internally and 3 were linked externally. Of the 8 clients linked internally, 7 achieved viral suppression, while 1 moved out of state before suppression could be confirmed. The 2 clients who handled linkage independently both reported attending a medical appointment and initiating ART; this was self‐reported (Figure 1).

**Conclusions**: Ensure staff have defined roles and are aware of what those roles are. Build connections with community partners and refer clients externally. HIV care should not be a competition but a combined community effort to end the HIV epidemic. Follow through on commitments made to clients; check in with them to ensure they are feeling confident with their care. Don't just look at how to treat HIV but look at how to treat the client as a whole and focus on linking clients to social support services that will increase their likelihood of achieving viral suppression.

**FIGURE 1:**
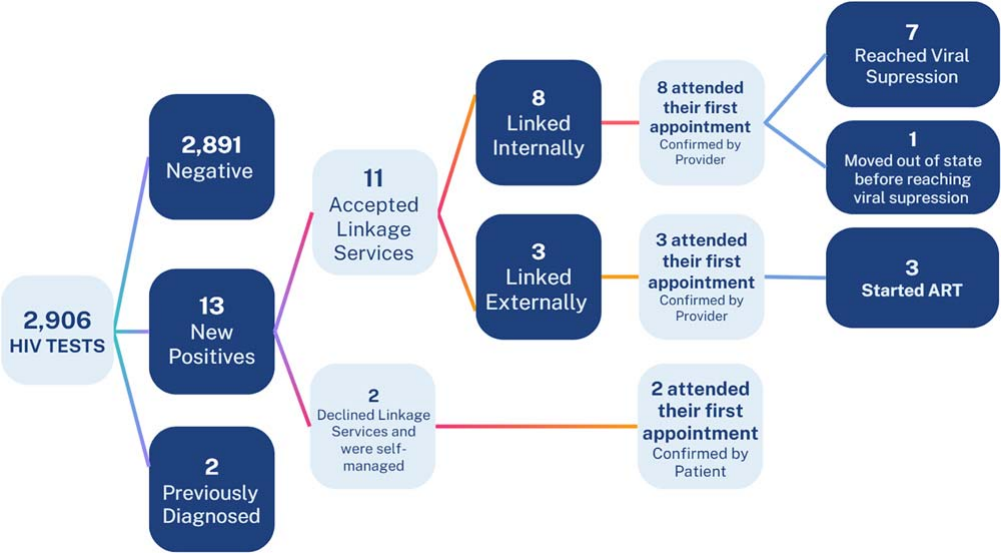
P188 | Linkage map.

### P189 Same‐Day B/F/TAF Re‐Initiation After NNRTI‐Based ART Discontinuation in China: Baseline Resistance and 24‐Week Effectiveness

Yan Zhao ^1^, Yuan Guan^1^, Hongxin Zhao^2^, Linghua Li^3^, Jun Liu^4^, Juan Jin^5^, Xia Li^6^, Yaokai Chen^7^, Yun He^8^, Jiahua Qiao^1^

^1^National Center for AIDS/STD Control and Prevention, Chinese Center for Disease Control and Prevention, Beijing, China; ^2^Clinical Center for HIV/AIDS, Beijing Ditan Hospital, Capital Medical University, Beijing, China; ^3^Infectious Disease Center, Guangzhou Eighth People's Hospital, Guangzhou Medical University, Guangzhou, Guangdong, China; ^4^Department of Infectious Diseases, The Third People's Hospital of Kunming, Kunming, Yunnan, China; ^5^Department of Infectious Diseases, Xi'an Eighth Hospital, Xi'an, Shaanxi, China; ^6^Yunnan Infectious Diseases Hospital, Kunming, Yunnan, China; ^7^Department of Infectious Diseases, Chongqing Public Health Medical Center, Chongqing, China; ^8^National Clinical Research Center for Infectious Diseases, The Third People's Hospital of Shenzhen and The Second Affiliated Hospital of Southern University of Science and Technology, Shenzhen, Guangdong, China

**Background**: Re‐initiating antiretroviral therapy (ART) after discontinuation of a non‐nucleoside reverse transcriptase inhibitor (NNRTI)‐based regimen may be complicated by previous drug resistance and the need to use alternative ART regimens. Prospective real‐world data on bictegravir/emtricitabine/tenofovir alafenamide (B/F/TAF) in this setting are limited. We evaluated resistance on the day of re‐initiation and virological outcomes after B/F/TAF re‐initiation.

**Methods**: This ongoing, prospective, single‐arm, multicentre study (Gilead Sciences, CO‐US‐380‐7381) plans to enrol 250 participants at seven sites in China who discontinued NNRTI‐based ART at least 90 days and re‐initiated treatment with B/F/TAF on the same day of returning to care. This interim analysis included 175 participants enrolled from 29 May 2025 to 31 May 2026. Baseline characteristics, reasons for ART discontinuation, genotypic resistance and Week 24 HIV‐1 RNA were assessed.

**Results**: Among 175 participants who re‐initiate ART on the same day of returning to care, median age was 42 (IQR 34–54) years, 80.57% were male and 51.43% acquired HIV through heterosexual transmission. The most common prior regimens were EFV+TDF+3TC (79.43%, 139/175). Median time from ART initiation to discontinuation was 35 (IQR 6–77) months; median time from ART discontinuation to re‐initiation was 17 (IQR 5–37) months; work‐related migration (36.57%, 64/175) and adverse effects (32.00%, 56/175) were the leading reasons for discontinuation. Median CD4 cell count and HIV‐1 RNA on the day of re‐initiation were 251.00 (IQR 135.00–430.50) cells/µL and 4.41 (IQR 3.77–5.18) log_10_ copies/mL, respectively. At enrolment, baseline drug resistance was detected in 26.01% (45/173) of participants, predominantly driven by NNRTI‐associated resistance 23.12%; NRTI‐ and PI‐associated resistance occurred in 2.31% and 1.19%, respectively. K103N, E138A and V179D were the most frequently mutations. At week 24, 81 of 175 participants had completed 6 months of follow‐up and had available HIV‐1 RNA results. 82.72% of them (67/81) achieved HIV‐1 RNA <50 copies/mL.

**Conclusions**: Previous drug resistance were detected in 26% participants who experienced discontinuation of first‐line NNRTI‐based regimen. Same‐day re‐initiation with B/F/TAF was feasible and was associated with favourable week 24 virologic outcomes among participants who completed follow‐up.

### P190 Indirect Comparison of the Efficacy of Bictegravir/Emtricitabine/Tenofovir Alafenamide Versus Dolutegravir/Lamivudine for First‐Line Therapy in a Rapid‐Initiation Setting: The BICNOW and DOLAVI Clinical Trial

Carmen Hidalgo‐Tenorio, Sergio Sequera, Rosario Javier Martinez, Mar Rivero, Coral Garcia Vallecillos, on behalf of BIC‐NOW Group

Infectious Diseases Service, Granada, Spain

**Background**: Second‐generation integrase strand transfer inhibitors are the backbone of current first‐line antiretroviral therapy (ART). However, triple therapy with bictegravir/emtricitabine/tenofovir alafenamide (BIC/FTC/TAF) and two‐drug therapy with dolutegravir/lamivudine (DTG/3TC) have not been compared in rapid‐initiation, “test‐and‐treat” clinical trial settings. We aimed to compare both strategies in treatment‐naïve people with HIV initiating ART in two Spanish studies.

**Methods**: We performed an indirect comparison of two studies evaluating first‐line ART initiation: BICNOW‐clinical trial (BIC/FTC/TAF, *n* = 208) and DOLAVI‐real‐life study (DTG/3TC, *n* = 84). Baseline characteristics and virological, immunological, lipid and anthropometric outcomes at weeks 4, 24 and 48 were compared using χ^2^, Student's *t*‐test or Mann–Whitney U test, as appropriate. All analyses were two‐sided, with statistical significance set at *p* < 0.05.

**Results**: Overall, 292 participants were included. Age was similar between the BIC/FTC/TAF and DTG/3TC groups (mean 35.9 ± 10.9 vs. 38.3 ± 12.0 years; *p* = 0.09), as was sex at birth (male: 87.5% vs. 91.7%; *p* = 0.31). Participants receiving BIC/FTC/TAF had more advanced disease at baseline, with higher proportions of HIV RNA > 100,000 copies/mL (43.0% vs. 21.0%; *p* < 0.001) and > 500,000 copies/mL (13.5% vs. 1.3%; *p* = 0.002), lower CD4 cell counts (median 315 [IQR 205–494] vs. 513 [359–672] cells/µL; *p* < 0.001), more frequent CD4 counts < 200 cells/µL (21.5% vs. 9.6%; *p* = 0.018), lower CD4/CD8 ratios (median 0.35 [0.20–0.58] vs. 0.54 [0.37–0.75]; *p*<0.001) and more AIDS‐defining conditions (22.7 vs. 11.9%; *p* = 0.035). At week 48, virological failure was uncommon and comparable between groups (1.1% vs. 0%; *p* = 1.00), as were discontinuation due to toxicity (1.4% vs. 1.2%; *p* = 0.87) and loss to follow‐up (11.1% vs. 7.1%; *p* = 0.31). In the ITT‐observed analysis, virological suppression < 50 copies/mL was achieved in 99.4% versus 100% of participants (*p* = 0.52). No significant differences were observed in total cholesterol/HDL ratio (3.9 ± 1.1 vs. 4.1 ± 1.2; *p* = 0.421), body mass index (25.2 [22.9–28.6] vs. 24.4 [22.3–27.9] kg/m^2^; *p* = 0.141), waist circumference (88 [80–97] vs. 87 [77–94.5] cm; *p* = 0.376) or CD4/CD8 ratio (0.7 [0.5–1.19] vs. 0.8 [0.6–1.1]; *p* = 0.095) (Table 1).

**Conclusions**: Despite more advanced baseline disease, BIC/FTC/TAF achieved virological outcomes comparable to DTG/3TC through week 48, with similarly low rates of virological failure, toxicity‐related discontinuation and loss to follow‐up. Lipid, anthropometric and immune recovery parameters were also comparable.

**TABLE 1:**
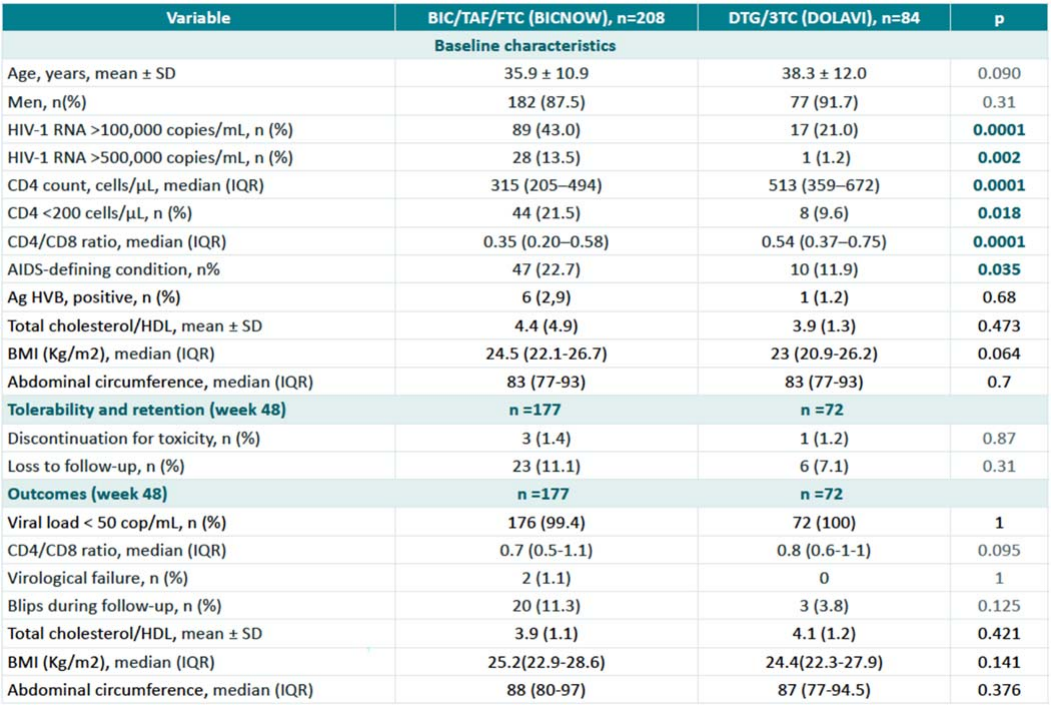
P190 | Baseline characteristics and outcomes by first‐line regimen.

### P191 Rapid ART Initiation and Early Retention Among Newly Diagnosed People Living With HIV: A Real‐World Cohort in Taiwan

Hsin‐Yen Ku ^1^, Na‐Lee Sun^1^, Shu‐Ying Chang^1^, Mei‐Hui Lin^1^, Shan‐wen Chi^1^, Yu‐Wen Lin^1^, Teng‐Chun Wang^1^, Chuan Pi^1^, Shu‐Yuan Li^1^, Jui‐Hsuan Yang^1^, Cheng‐Pin Chen^1^, Chien‐Yu Cheng^1,2^, Shu‐Hsing Cheng^1,3^

^1^Comprehensive AIDS Care Center and Department of Infectious Diseases, Taoyuan General Hospital, Taoyuan City, Taiwan; ^2^Institute of Public Health, National Yang Ming Chiao Tung University, Taipei, Taiwan; ^3^School of Public Health, College of Public Health, Taipei Medical University, New Taipei, Taiwan

**Background**: Rapid antiretroviral therapy (ART) initiation optimizes clinical outcomes [1], yet real‐world implementation faces systemic barriers. This study evaluates rapid ART efficiency and compares early virologic outcomes between same‐day and non‐same‐day ART initiation at a regional teaching hospital in Taiwan.

**Methods**: This retrospective cohort study included newly diagnosed individuals living with HIV linked to care between January 2025 and January 2026 (*N* = 71). Patients were stratified into same‐day ART (initiated on diagnosis day) and non‐same‐day ART groups. Early virologic suppression was defined as VL < 50 copies/mL within 6 months of ART initiation. Late presentation was defined as baseline CD4< 200 cells/mm^3^. Chi‐square and Mann–Whitney U tests were used for comparisons.

**Results**: Among 71 participants, the median age was 32 years (97.2% male; 83.1% MSM). Overall, 36.6% were late presenters. Rapid ART was highly efficient: 54.9% (*n* = 39) achieved same‐day initiation, while 45.1% (*n* = 32) were non‐same‐day. Notably, men who have sex with men (MSM) were significantly more likely to receive same‐day ART than non‐MSM individuals (92.3% vs. 65.6%; *p* = 0.013). The same‐day group tended to have higher baseline CD4 counts than the non‐same‐day group (median: 333 vs. 212.5 cells/mm^3^, *p* = 0.124) [2]. The same‐day ART group demonstrated a higher early virologic suppression rate (64.1% vs. 50.0%; *p* = 0.352) (Table 1). Across the cohort, early regimen switching occurred in 12.7% (*n* = 9) at a median of 21 days post‐ART. Case management logs revealed these switches were equally distributed among clinical indicators (high viral load, *n* = 3), physical adverse effects (gastrointestinal intolerance, rash, *n* = 3) and practical lifestyle concerns (pill size, daytime work impact, *n* = 3). Following individualized modifications—including one successful switch to long‐acting injectable cabotegravir/rilpivirine—77.8% (7/9) achieved viral suppression. Additionally, foreign migrant workers (11.3%, *n* = 8) faced a 50% late presentation rate; while 25% (*n* = 2) were repatriated, the remainder (75%) were successfully retained in care through national subsidies.

**Conclusions**: Same‐day ART is clinically feasible, particularly among the MSM population who demonstrated higher immediate linkage. Although early virologic suppression rates showed a favourable trend for same‐day initiation (64.1% vs. 50.0%), the difference was not statistically significant, indicating that larger‐scale studies are warranted to definitively confirm the virologic benefits of same‐day ART [3]. Nevertheless, proactive, individualized case management remains indispensable to monitor early switch dynamics and optimize retention.

**References**

1. Gandhi RT, Bedimo R, Hoy JF, et al. Antiretroviral drugs for treatment and prevention of HIV infection in adults: 2024 Recommendations of the International Antiviral Society‐USA Panel. JAMA. 2024;332(13):1090‐1110.

2. European AIDS Clinical Society. EACS Guidelines Version 12.0. Available from: https://www.eacsociety.org/guidelines/ (Accessed 23/06/2026).

3. Ford N, Vitoria M, Doherty M. Rapid initiation of antiretroviral therapy: global implementation and evidence. Curr Opin HIV AIDS. 2023;18(1):1‐7.

**TABLE 1:**
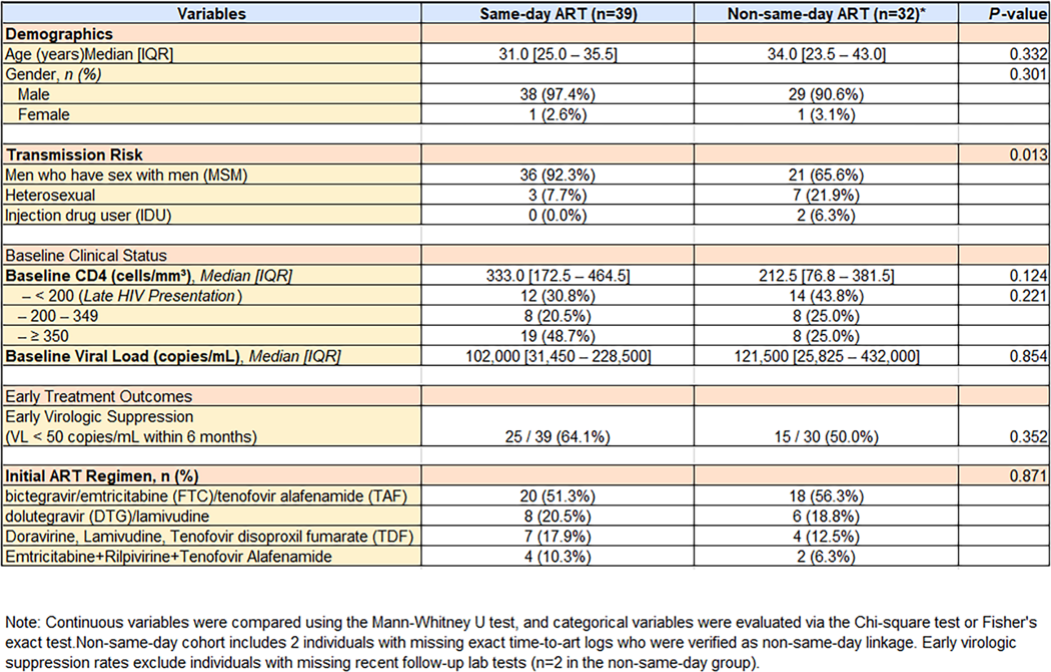
P191 | Comparison of baseline characteristics and early virologic outcomes between same‐day and non‐same‐day ART stratifications (*N* = 71).

### P192 One Day, One Week: Decoding the Optimal ART Start Timing in People With HIV in the Real‐World Setting

Giulia Micheli ^1^, Alessandro Caioli^2^, Rozenn Esvan^1^, Alessandro Giacinta^1^, Virginia Barchi^1^, Damiano Farinacci^1^, Rebecca Jo Steiner^1^, Neva Braccialarghe^1^, Arianna Forniti^1^, Eugenio Nelson Cavallari^1^, Isabella Abbate^3^, Lavinia Fabeni^3^, Gabriella Rozera^3^, Enrico Girardi^4^, Fabrizio Maggi^3^, Andrea Antinori^5^, Valentina Mazzotta^1^

^1^Counseling, Test and HIV Prophylaxis and STI Unit, Regional AIDS Reference Center, National Institute for Infectious Diseases Lazzaro Spallanzani IRCCS, Rome, Italy; ^2^Department of Experimental Medicine, Sapienza University of Rome, Rome, Italy; ^3^Laboratory of Virology, National Institute for Infectious Diseases Lazzaro Spallanzani IRCCS, Rome, Italy; ^4^Scientific Direction, National Institute for Infectious Diseases, Lazzaro Spallanzani IRCCS, Rome, Italy; ^5^Health Direction, National Institute for Infectious Diseases Lazzaro Spallanzani IRCCS, Rome, Italy

**Background**: Guidelines recommend starting antiretroviral therapy (ART) within 7 days after HIV diagnosis, ideally the same day, based mainly on trials from low‐ and middle‐income settings showing improved virological and retention outcomes. However, observational cohort results remain inconsistent and may be affected by immortal time bias. We investigated two different virological thresholds as outcomes by ART initiation timing.

**Methods**: Newly diagnosed outpatient people with HIV (PWH) starting ART at a single centre were included (January 2024–May 2025). Baseline was defined as the date of HIV diagnosis. Participants were classified according to ART initiation timing into same‐day (day 0), rapid (days 1–7) and non‐rapid start (> 7 days). Outcomes were time to first documented HIV‐RNA <50 cp/mL (virological suppression) and < 200 cp/mL (transmission threshold) within 12 months. Categorical and continuous variables were assessed by Fisher's exact test and Wilcoxon rank‐sum as appropriate. Cox proportional hazards models, adjusted for age, sex at birth, baseline HIV‐RNA and CD4 cell count, were used to assess outcomes.

**Results**: We included 136 PWH (23 same‐day, 77 rapid and 36 non‐rapid). Baseline features are detailed in Table 1. ART initiation within 7 days (same‐day+rapid) after HIV diagnosis was associated with faster time to HIV‐RNA < 200 compared with non‐rapid start (aHR 1.72, 95% CI: 1.06–2.78; *p* = 0.027), while higher baseline HIV‐1 RNA was independently associated with slower HIV‐RNA < 200 achievement (aHR per log_10_ increase 0.78, 0.62–0.99; *p* = 0.042). After stratification, same‐day was associated with a shorter time to HIV‐RNA <50 (2.07, 1.06–4.04; *p* = 0.033) compared with non‐rapid start. Both same‐day and rapid were associated with a shorter time to HIV‐RNA < 200 compared with non‐rapid start (Figure 1), with higher baseline HIV‐RNA independently associated with slower HIV‐RNA < 200 achievement (aHR per log_10_ increase 0.78, 0.61–0.98; *p* = 0.0343). No ART modifications occurred among participants starting ART before laboratory results were available.

**Conclusions**: ART initiation within 7 days after HIV diagnosis was associated with faster achievement of the < 200 cp/mL transmission threshold. Stratified analysis suggested that this benefit was largely driven by same‐day start, which was also associated with faster virological suppression to <50 cp/mL. Starting ART before laboratory results were available appeared safe, with no treatment modifications.

**FIGURE 1:**
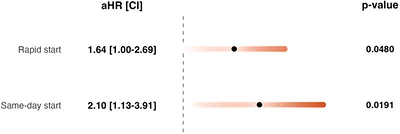
P192 | Adjusted hazard ratios from a Cox model for time to HIV‐RNA below 200 copies/mL within 12 months. Same‐day and rapid start were compared with non‐rapid start; the model was adjusted for age, sex at birth, baseline HIV‐RNA and CD4 cell count.

**TABLE 1:**
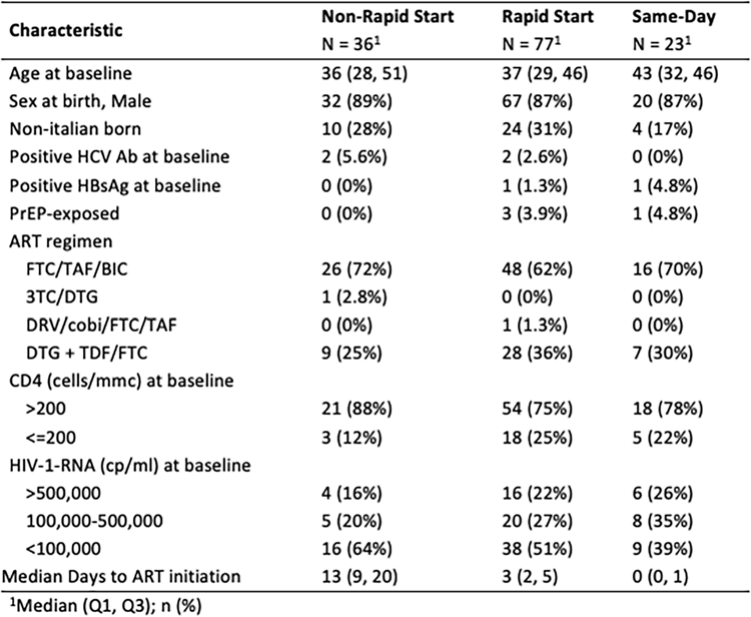
P192 | Baseline characteristics of the cohort by treatment timing group.

## Treatment Strategies: Adherence

### P193 Comparable Virologic Outcomes Across Adherence Levels With DTG/3TC and BIC/FTC/TAF in Adults Initiating ART: Week 48 Results From VOGUE

Karine Lacombe ^1,2^, Melchor Jaume^3,4^, Gustavo Lopardo^5^, Kehui Wang^6^, Ruolan Wang^7^, Adelaide Jewell^8^, Emilie Elliot^9^, Rebecca Borsi^7^, Julie Priest^7^, Michelle Kisare^8^, Piotr Budnik^8^, Jean van Wyk^8^

^1^Sorbonne Université, Inserm, Institut Pierre Louis d'Épidémiologie et de Santé Publique, Paris, France; ^2^Assistance Publique—Hôpitaux de Paris, Hôpital Saint‐Antoine, Paris, France; ^3^Fundació Institut d'Investigació Sanitària Illes Balears, Palma de Mallorca, Spain; ^4^Hospital Universitari Son Espases, Palma de Mallorca, Spain; ^5^Fundacion Centro de Estudios Infectologicos, Buenos Aires, Argentina; ^6^GSK, Collegeville, PA, US; ^7^ViiV Healthcare, Durham, NC, US; ^8^ViiV Healthcare, London, UK; ^9^ViiV Healthcare, Madrid, Spain

**Background**: Direct comparative evidence evaluating how adherence variability impacts virologic outcomes with contemporary first‐line, integrase strand transfer inhibitor‐based regimens remains limited. We evaluated the impact of adherence on week 48 virologic outcomes in VOGUE, a randomized, head‐to‐head study of dolutegravir/lamivudine (DTG/3TC) and bictegravir/emtricitabine/tenofovir alafenamide (BIC/FTC/TAF).

**Methods**: VOGUE (NCT05979311) is an ongoing phase 3b, open‐label, non‐inferiority study comparing DTG/3TC versus BIC/FTC/TAF as first‐line antiretroviral therapy (ART). Adherence was estimated based on the number of missed doses using pill count data from baseline (day 1) to week 48 and categorized as ≥90% or < 90%. The proportion of participants with HIV‐1 RNA <50 c/mL (Snapshot algorithm) by adherence category at week 48 was assessed. Treatment satisfaction was evaluated using the HIV Treatment Status Questionnaire, status version (HIVTSQs) from week 4 to week 48.

**Results**: Among 509 randomized participants (DTG/3TC, *n* = 254; BIC/FTC/TAF, *n* = 255), overall adherence was high and similar between treatment groups, with 98% of participants in both groups achieving ≥90% adherence across all visits (DTG/3TC, 248/254; BIC/FTC/TAF, 249/255). Six (2%) participants in each treatment group had < 90% adherence; of these, 4 participants receiving DTG/3TC and 2 receiving BIC/FTC/TAF had < 85% adherence. At week 48, virologic suppression rates were high across adherence categories and similar between DTG/3TC and BIC/FTC/TAF (≥90% adherence: 89% vs. 92%; < 90% adherence: 83% vs. 83%). Overall, 14 participants (DTG/3TC, *n* = 7; BIC/FTC/TAF, *n* = 7) met confirmed virologic withdrawal (CVW) criteria, with no treatment‐emergent resistance observed; of these, 1 participant in each treatment group had < 90% adherence. Treatment satisfaction was high at week 4 in both treatment groups (mean [SD] HIVTSQs total score: DTG/3TC, 53.2 [6.8]; BIC/FTC/TAF, 53.1 [7.5]) and remained stable through week 48.

**Conclusions**: In adults initiating ART, adherence and week 48 virologic outcomes were comparable between DTG/3TC and BIC/FTC/TAF. High virologic suppression rates were achieved across adherence categories, alongside high treatment satisfaction. Among the few participants experiencing suboptimal adherence, incidence of CVW was low, with no treatment‐emergent resistance observed. These findings suggest the 2‐drug regimen DTG/3TC demonstrates comparable clinical robustness to adherence variability as the 3‐drug regimen BIC/FTC/TAF.

### P194 Superior Long‐Term Real‐World Adherence and Persistence With Long‐Acting Cabotegravir+Rilpivirine (CAB+RPV LA) Compared to Oral Antiretroviral Therapy (ART) Among People With HIV in the US: The OPTIMIZE Study

Andrew P. Brogan^1^, Maral DerSarkissian^2^, Catherine Nguyen^2^, Megan Pinaire^2^, Emily Reichert^2^, Qingyi Cai^2^, Mark Lewandowski^1^, Shana Walko^1^, Ian Jacob ^1^, Megan Dominguez^1^, Gregory Huhn ^1^

^1^ViiV Healthcare, Durham, NC, US; ^2^Analysis Group, Inc., Los Angeles, CA, US

**Background**: Maintaining high levels of treatment adherence and persistence is essential to sustaining virologic control at levels sufficient for treatment as prevention, minimizing the risk of resistance and advancing progress towards ending the HIV epidemic. OPTIMIZE evaluated real‐world adherence and persistence to CAB+RPV LA versus oral ART regimens.

**Methods**: OPTIMIZE is a retrospective US cohort study using Komodo Research administrative claims data from 1 January 2019 to 31 July 2025. People with HIV (PWH) aged ≥12 years on guideline‐recommended first‐line oral ART were categorized as initiating CAB+RPV LA or remaining on oral ART. The index date was the first CAB+RPV LA injection on or after 1 January 2021 or a corresponding imputed date for the oral ART cohort. PWH were required to have ≥24 months of follow‐up unless deceased. Propensity score weighting was used to balance baseline characteristics. Adherence (proportion of days covered [PDC]) was assessed: (i) over the observation period and (ii) to discontinuation (or end of observation period). Persistence was time from index to discontinuation, censoring at end of observation. Doubly robust logistic and Cox proportional hazards regression estimated adjusted odds ratios (aOR) for adherence and hazard ratios (aHR) for persistence, with 95% confidence intervals (CI).

**Results**: Among the 514,809 identified, 2364 initiating CAB+RPV LA (median age 47 years, 22% female) and 41,347 remaining on oral ART (median age 55 years, 22% female) met eligibility criteria. After weighting, baseline characteristics were balanced (weighted oral cohort: median age 47 years, 23% female) and median follow‐up time was 32 months in both cohorts. In the weighted analysis, a greater proportion of people in the CAB+RPV LA cohort achieved PDC ≥0.9 than the oral ART cohort over both adherence periods (observation period: 60% vs. 39%; to discontinuation: 92% vs. 79%, both *p* < 0.001; Table 1). At 24 months, 72% of the CAB+RPV LA cohort remained persistent versus 57% of the oral ART cohort. CAB+RPV LA was associated with a statistically significant 43% lower rate of discontinuation versus oral ART (aHR [95% CI] = 0.57 [0.52, 0.63]; Figure 1).

**Conclusions**: CAB+RPV LA demonstrated superior long‐term adherence and persistence compared to oral ART in real‐world analyses in the US.

**FIGURE 1:**
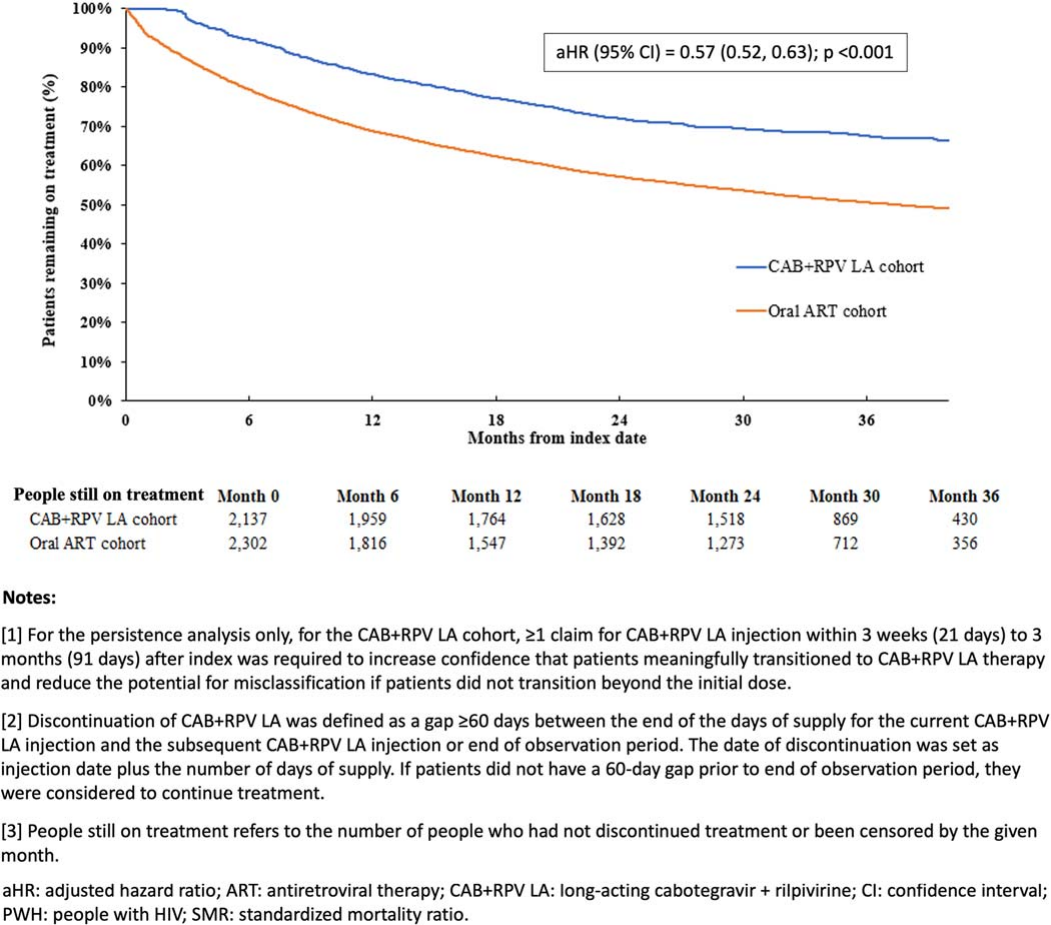
P194 | Persistence among PWH receiving CAB+RPV LA or oral ART (SMR‐weighted sample).

**TABLE 1:**
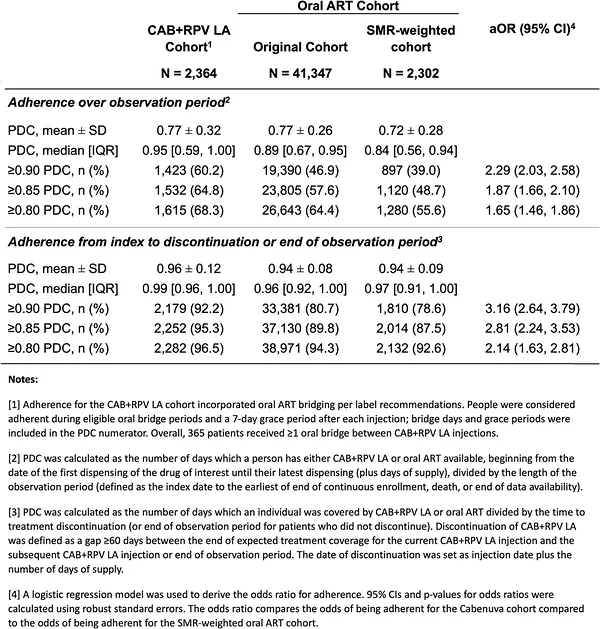
P194 | Treatment adherence among PWH in the CAB+RPV LA cohort and oral ART cohort.

### P195 Defects in the HIV 5′‐Leader May Help to Differentiate Active Replication From Non‐Suppressible Low‐Level Viraemia

Frank Wiesmann, Patrick Braun, Sarah Ritter, Jona‐Lukas Bittner, Daniel Beer, Heribert Knechten

PZB Aachen, Aachen, Germany

**Background**: Genetic aberrations affecting splicing recognition sites and packaging signal domains in HIV‐1 have been found to occur more frequently in plasma samples with low‐level viraemia below 1000 copies/mL. These defects are consistent with the virus's inability to form intact viral particles.

**Methods**: 86 patients were divided into two groups based on their HIV‐1 viral load results (measured using the Abbott Alinity m platform). Group A included 64 samples from patients with viral loads ≥1000 copies/mL, while Group B comprised 22 samples with viral loads < 1000 copies/mL. Sequencing of samples from both groups was performed using the Illumina iSeq 100 NGS platform (Nextera XT), focusing on the major splicing domain (MSD) and the packaging signal domain (PSI) located between the HIV‐1 5′ LTR and the gag region. Deletions and mutations were identified, documented and compared between the two groups based on their frequencies and the progression of corresponding viral load measurements.

**Results**: While deletions and mutations at the MSD site were observed in 8 of the 22 low‐viraemic samples from group B (36.3%), these specific aberrations were not present at all in group A. However, the first nucleotide of the MSD domain showed a T>C switch in 4 of 64 samples (6.2%) of group A, without any noticeable negative effect on plasma viraemia. These findings suggest that viruses from patients in group B carrying these specific defects were unable to produce higher viral titres. The PSI domain was also affected in group B; however, some mutations were not exclusive to this group and were also found in some isolates from group A.

**Conclusions**: Patients with defects in the MSD did not show viral loads much higher than 1000 copies/mL in plasma, suggesting the release of HIV fragments from cells in these cases and a potential inability of these viruses to actively replicate. These findings could help identify patients who would benefit from treatment intensification and those with non‐suppressible viral loads who would not.

### P196 ART Forgiveness: Redefining PDC Benchmarks Against Transient Viraemia and Confirmed Virological Failure in an INSTI Era Cohort

Beatrice Bragato ^1^, Francesco Vladimiro Segala^1^, Daniele Mengato^2^, Lolita Sasset^1^, Eleonora Vania^1^, Davide Leoni^1^, Samuele Gardin^1^, Anna Ferrari^1^, Giacomo Berti^3^, Annamaria Cattelan^4^

^1^Infectious and Tropical Diseases Unit, Padua University Hospital, Padua, Italy; ^2^Hospital Pharmacy Unit, Azienda Ospedale‐Università Padova, Padua, Italy; ^3^Unit of Biostatistics, Epidemiology and Public Health, Department of Cardiac, Thoracic, Vascular Sciences and Public Health, University of Padova, Padua, Italy; ^4^Department of Molecular Medicine, University of Padua, Padua, Italy

**Background**: A proportion of days covered (PDC) ≥90% is the conventional benchmark for adequate antiretroviral therapy (ART) adherence. In contemporary cohorts, the adherence threshold required may differ depending on whether single transient viraemia or confirmed virological failure is considered. We characterized the relationship between PDC and multiple virological endopoints across the full adherence spectrum and identified its determinants.

**Methods**: Retrospective cohort of people with HIV (PWH) on ART at Padua University Hospital (January 2024–June 2025) with ≥180 days of follow‐up and an available PDC. PDC was derived from pharmacy refills (0–100 scale). Outcomes were detectable viraemia (any ≥50 cp/mL), overt viraemia (any ≥200 cp/mL), persistent low‐level viraemia (pLLV; ≥2 consecutive 50–199) and confirmed virological failure (CVF; ≥2 consecutive ≥200). ART forgiveness (probability of virological success across PDC values) was modelled by logistic regression with natural cubic splines (df = 3); for each outcome, we estimated the minimum PDC at which ≥95% virological success was assured at the upper 95% CI: bound. Independent predictors of overt viraemia and CVF were assessed by Firth‐penalized logistic regression with multiple imputation (MICE, m = 20; Rubin's rules).

**Results**: 1412 patients were enrolled (Table 1). Median PDC was 90.4% (IQR 82.6–94.8). There were 75 overt viraemia and 19 CVF events. The minimum PDC ensuring ≥95% success was markedly lower when confirmed virological outcomes were considered: 74% for CVF versus 90% for single‐measurement overt viraemia (Figure 1). Each 10% PDC increment independently reduced the odds of overt viraemia (aOR 0.67, 95% CI: 0.58–0.78; *p* < 0.001) and CVF (aOR 0.68, 0.54–0.87; *p* = 0.002). Africa and Latin America region of origin were strong independent risk factors for both outcomes, while the number of comorbidities predicted overt viraemia (aOR 1.31 per condition, 1.13–1.51; *p* < 0.001).

**Conclusions**: In our cohort, the adherence threshold required to avoid confirmed virological failure was lower (PDC ≈ 74%) than the conventional 90% benchmark used to avoid any detectable viraemia. Nevertheless, higher PDC conferred protection across all virological endpoints. PWH of non‐European origin and those with a high comorbidity burden had increased risk of adverse virological outcome and should be prioritized for adherence support. These findings suggest that adherence benchmarks may need to be outcome‐specific and contextualized to modern ART regimens.

**FIGURE 1:**
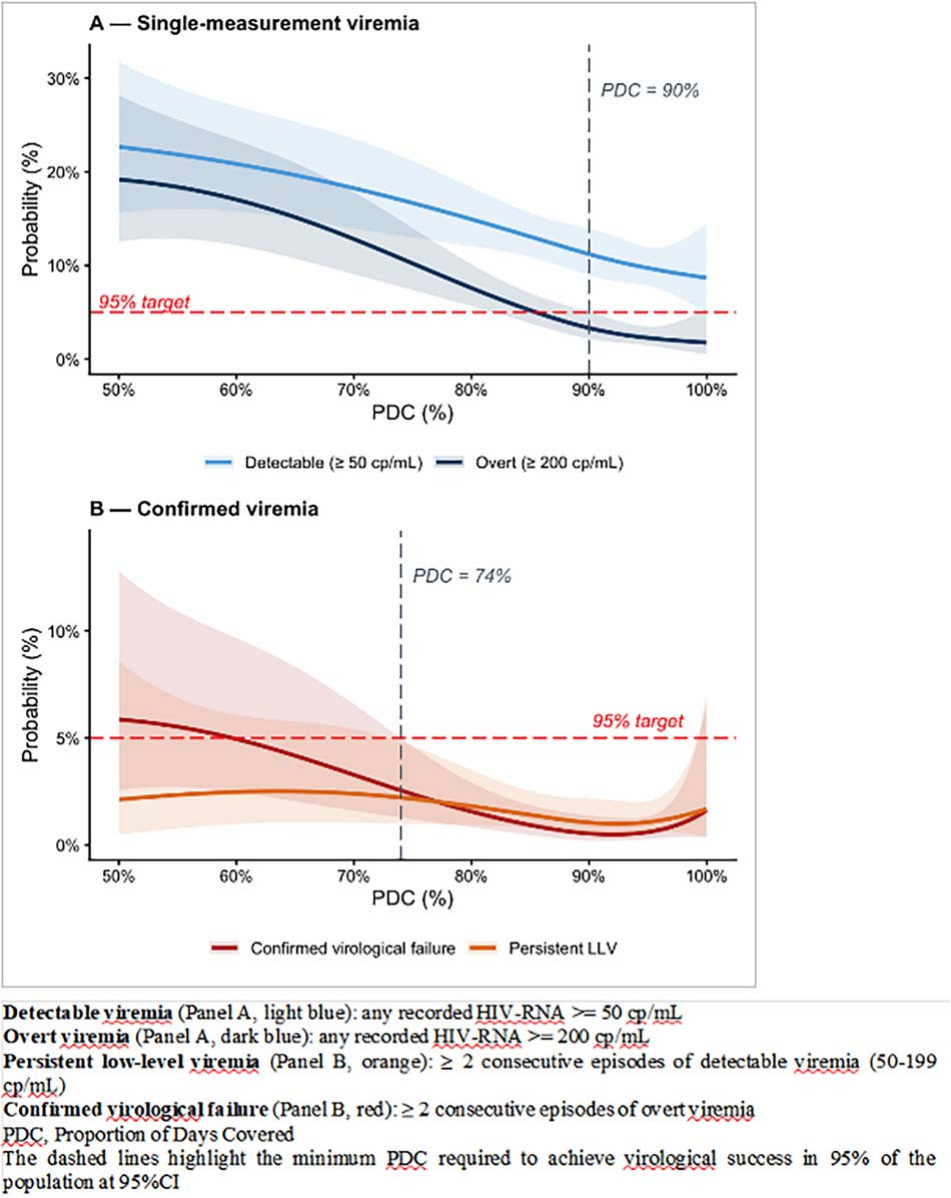
P196 | ART forgiveness across the adherence spectrum.

**TABLE 1:**
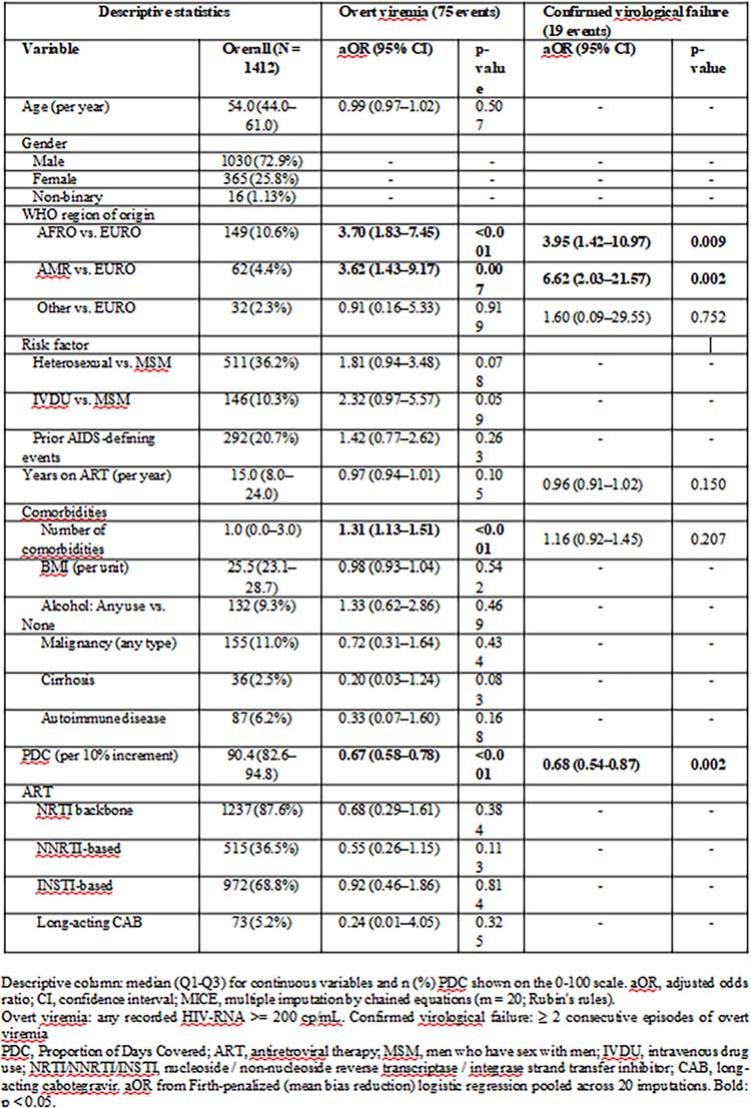
P196 | Demographic characteristics and descriptive statistics (multivariable Firth‐penalized logistic regression for overt viraemia and parsimonious Firth‐penalized model for confirmed virological failure).

### P197 Use of an Adapted Pre‐Consultation Adherence Screener to Identify Behavioural Barriers in HIV Treatment and Prevention Services Across the UK and Ireland

Nicola Galbraith ^1^, Jordan McLaren^2^, Sarah Edwards^1^, Kathryn Carroll^1^, Omoyemi Daramola^1^, John Weinman^3^

^1^HIV Standards Support Team, Gilead Sciences Ltd, London, UK; ^2^Opinium, London, UK; ^3^School of Cancer and Pharmaceutical Sciences, Kings College London, London, UK

**Background**: Making Medicines Work for You [1] is an established medicines adherence screener that has been adapted in collaboration with King's College London for use in HIV treatment and prevention. The adapted screener is designed as a pre‐consultation screener, supporting clinicians to identify adherence‐related behaviours and concerns that may warrant further discussion but may not be routinely elicited in clinical practice. This analysis presents preliminary findings from the ongoing evaluation.

**Methods**: Responses were analysed from individuals who completed the adapted HIV adherence screener within treatment and prevention services across the UK and Ireland and consented to anonymized, aggregated data use. The screener assessed self‐reported adherence ability, medication‐taking frequency, understanding of U = U and selected adherence‐related behaviours and concerns.

**Results**: A total of 118 responses were included. Self‐reported adherence was high: 86.4% rated their ability to take medicines as good or better, with 62.7% reporting excellent adherence. Additionally, 77.1% reported taking medication all of the time and 92.4% most or all of the time. However, the screener identified behaviours that may warrant further clinical exploration. Over half of respondents (51.7%) selected at least one adherence‐related statement, including adapting their medication use (27.9%) and forgetting doses (21.2%), while 10.2% reported concerns about their medication. Understanding of U = U was high overall (94.1%), with lower levels of adherence and understanding observed among prevention service users; however, this analysis was not intended to formally compare groups.

**Conclusions**: Despite high levels of self‐reported adherence, the pre‐consultation screener identified behavioural signals that may not otherwise be routinely elicited in clinical encounters. These findings suggest that the screener may support more focused and personalized adherence discussions by highlighting areas for further exploration across both treatment and prevention settings. Full evaluation of acceptability, feasibility and clinical utility is ongoing.

**Reference**

1. Weinman, John, et al. “Pilot testing of a brief pre‐consultation screener for improving the identification and discussion of medication adherence in routine consultations.” Patient Preference and Adherence (2019): 1895‐1898.

### P198 Abstract Withdrawn

### P199 Decreased Mood and Non‐Medical Reasons as Main Causes for ART Interruption—Results From a Survey Among People With HIV in a Single Centre Study in Poland

Dagny Krankowska^1,2^, Ewa Siwak^1,3^, Tomasz Mikuła^1,2^, Agnieszka Lembas^1,2^, Mariusz Sapuła^1,2^, Aneta Cybula ^1,2^, Wiktoria Gajek‐Flanczewska^4^, Sebastian Flanczewski^4^, Alicja Wiercińska‐Drapało^1,2^

^1^Department of Infectious and Tropical Diseases and Hepatology, Medical University of Warsaw, Warsaw, Poland; ^2^Hospital for Infectious Diseases, Warsaw, Poland; ^3^HIV Out‐Patient Clinic, Hospital for Infectious Diseases, Warsaw, Poland; ^4^Students’ Scientific Society of the Department of Infectious and Tropical Diseases and Hepatology, Warsaw, Poland

**Background**: Adherence to antiretroviral therapy (ART) is essential for achieving the WHO 95‐95‐95 targets. Studies indicate that 2%–15% of people living with HIV in Europe interrupt ART [1−3]. This study aimed to identify reasons for ART interruption among people living with HIV in a single centre in Poland and explore factors motivating re‐engagement in care.

**Methods**: Between 2024 and 2026, an anonymous survey was conducted among adult patients living with HIV who had interrupted ART for at least 4 weeks on one or more occasions after HIV diagnosis and subsequently relinked to care. Participants were recruited either during hospitalization for complications related to treatment interruption or during routine outpatient visits when they reported prior ART discontinuation. The questionnaire included 29 questions, some with multiple‐choice responses. The study was approved by the University's Bioethics Committee.

**Results**: Answers of *n* = 70 patients were analysed. Female‐to‐male ratio was 15:55 and *n* = 43/70 (61.4%) identified as heterosexual. Of those 67 of 70 who responded to the question regarding the number of ART interruptions, *n* = 35/67 (52.2%) interrupted ART once and *n* = 32/67 (47.8%) interrupted ART between 2 and 5 times. Duration of interruption ranged from 1 to 60 months. Of those 69 who answered the appropriate question, the general health condition of *n* = 33/69 (47.8%) worsened after ART interruption requiring hospital care, with wasting syndrome being the most common AIDS‐defining disease diagnosed. Respondents gave multiple reasons for ART interruption, of which the main ones were (multiple answers allowed): decreased mood *n* = 26/70 (37.1%), lack of time for visits *n* = 17/70 (24.3%) and loss of job *n* = 16/70 (22.9%) (Table 1). The most common motivations to return to HIV care were (multiple answers allowed): worsening of general condition *n* = 40/70 (62.9%), families and friends support *n* = 26/70 (37.1%) and getting an insurance *n* = 11/70 (15.7%).

**Conclusions**: Identifying people at risk of ART interruption is needed, including screening for decreased mood. Reasons for ART interruption often go beyond medical, thus multidisciplinary support with psychological and social workers support are needed for people with HIV to help them maintain adherence and prevent them from developing AIDS‐defining diseases.

**References**

1. Marcus U, Hickson F, Weatherburn P, et al.; EMIS Network. Antiretroviral therapy and reasons for not taking it among men having sex with men (MSM): results from the European MSM Internet Survey (EMIS). PLoS One. 2015;10(3):e0121047. doi:10.1371/journal.pone.0121047.

2. De Vito A, Ricci E, Menzaghi B, et al. Causes of HIV treatment interruption during the last 20 years: a multi‐cohort real‐life study. Viruses. 2023;15(3):720. doi:10.3390/v15030720.

3. Bansi‐Matharu L, Rodriguez Loria G, Cole SR, et al.; INSIGHT START Study Group. Risk factors for antiretroviral therapy (ART) discontinuation in a large multinational trial of early ART initiators. AIDS. 2019;33(8):1385‐90. doi:10.1097/QAD.0000000000002210.

**TABLE 1:**
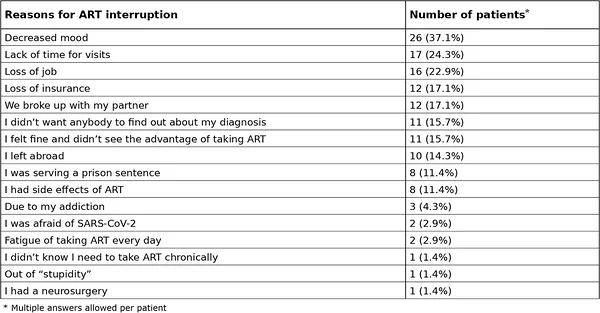
P199 | Reasons for ART interruption according to respondents.

### P200 Anxiety, Depression and Adherence to Antiretroviral Therapy in People Living With HIV

Marco Antonio Lopez Hernandez^1^, Ericka Denise Bustos Angel ^2^

^1^CLISIDA, Hospitales Generales Regionales, IMSS, Mexico City, Mexico; ^2^Infectology, Hospital General de Mexico, Mexico City, Mexico

**Background**: Anxiety and depression are among the most prevalent mental health disorders in people living with HIV (PLWH). Generalized anxiety disorder is often associated with socioeconomic disadvantage, comorbid physical conditions and unhealthy lifestyle behaviours such as physical inactivity. Depressive symptoms are consistently linked to poor adherence to antiretroviral therapy (ART), reduced quality of life and worse HIV‐related outcomes, including decreased life expectancy. This study aimed to examine the association between anxiety and depression symptoms and ART adherence among PLWH [1].

**Methods**: We conducted a cross‐sectional survey of 551 PLWH aged 18 years or older receiving care at the HIV Outpatient Clinic of General Regional Hospital No.1 in Mexico City between March and July 2025. ART adherence was assessed using the Simplified Medication Adherence Questionnaire (SMAQ), while anxiety and depression symptoms were evaluated using the Hospital Anxiety and Depression Scale (HADS). Associations between emotional symptoms and ART adherence were analysed using chi‐squared or Fisher's exact tests. Adjusted odds ratios (aOR) and 95% confidence intervals (CI) were calculated. A *p*‐value < 0.05 was considered statistically significant.

**Results**: Of the 551 participants, 199 (36.12%) screened positive for anxiety and/or depression: 113 (20.51%) for anxiety alone, 13 (2.36%) for depression alone and 73 (13.25%) for both. Among patients with emotional symptoms, 45.3% were adherent to ART, compared to 66.5% adherence in those without symptoms (χ^2^ = 20.3, *p* < 0.0001). The presence of anxiety and/or depression was associated with significantly increased odds of non‐adherence (OR 2.40, 95% CI: 1.61–3.56) (Figure 1).

**Conclusions**: Anxiety and/or depression symptoms were significantly associated with lower adherence to ART among PLWH. Individuals with emotional symptoms had a 2.4‐fold increased odds of non‐adherence compared to those without symptoms. These findings underscore the need to integrate routine mental health screening and psychosocial support into HIV care to enhance treatment adherence and improve clinical outcomes.

**Reference**

1. Mendez NA, Mayo D, Safren SA. Interventions addressing depression and HIV‐related outcomes in people with HIV. Curr HIV/AIDS Rep. 2021;18(4):377‐390. doi: 10.1007/s11904‐021‐00559‐w.

**FIGURE 1:**
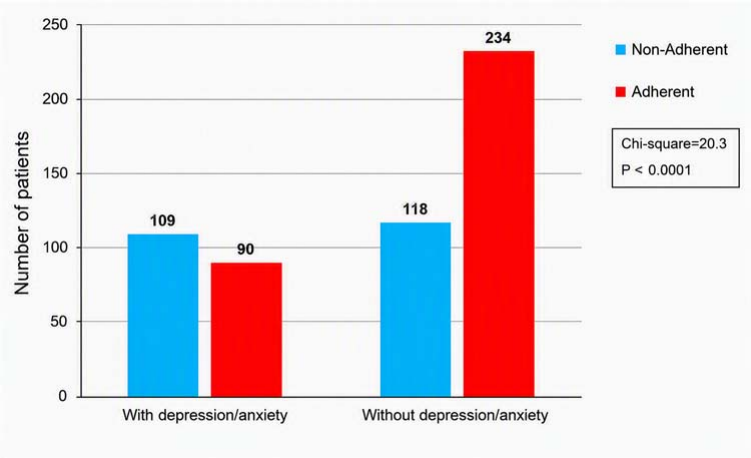
P200 | Adherence to antiretroviral therapy by depression/anxiety status.

### P201 Patterns of Care Interruption and Re‐Engagement in HIV Care Across the COVID‐19 Pandemic Eras

Kyoko Yoshida, Ryoko Sekiya, Seowoong Jung, Masaru Tanaka, Taiichiro Kobayashi, Kazuaki Fukushima, Keishiro Yajima, Akifumi Imamura

Department of Infectious Diseases, Tokyo Metropolitan Komagome Hospital, Bunkyo, Tokyo, Japan

**Background**: Retention in HIV care is essential for improving clinical outcomes among people living with HIV (PWH) and preventing HIV transmission. Although previous studies suggested that HIV care remained relatively resilient during the COVID‐19 pandemic, little is known about interruption patterns and clinical outcomes across the pre‐pandemic, pandemic and post‐pandemic eras. We evaluated care interruption, re‐engagement and clinical outcomes between 2016 and 2024 at an HIV referral centre.

**Methods**: We included PWH who received outpatient care between 1st January 2016 and 31st December 2024, regardless of antiretroviral therapy (ART) receipt. We defined care interruption as failure to attend a scheduled outpatient appointment for more than 60 days after the planned visit date, categorized into pre‐pandemic (2016–2019), pandemic (2020–2021) and post‐pandemic (2022–2024) eras according to the calendar year of interruption. Annual care interruption proportions, episode characteristics, re‐engagement and clinical outcomes were compared across eras.

**Results**: We included 1816 PWH. During the study period, 344 care interruption episodes occurred among 184 individuals, including 154, 76 and 114 episodes in the pre‐pandemic, pandemic and post‐pandemic eras, respectively (Table 1). The annual proportions of patients experiencing care interruptions remained stable across the pre‐pandemic, pandemic and post‐pandemic eras. Among 184 individuals with care interruptions, 75 (40.7%) experienced more than one interruption episode. Approximately one in eight interruption episodes occurred before ART initiation across all eras. Re‐engagement in care was observed in 83.1%, 77.6% and 77.2% of care interruption episodes in the pre‐pandemic, pandemic and post‐pandemic eras, respectively, with similar cumulative probability across eras. At re‐engagement, detectable HIV RNA (> 200 copies/mL) was observed in 55 (44.0%) of the pre‐pandemic group, 28 (49.1%) of the pandemic group and 38 (43.7%) of the post‐pandemic group. Overall, 139 (50.5%) of 275 re‐engagement episodes were followed by repeated care interruption.

**Conclusions**: Our findings suggest that HIV care remained resilient across the pandemic eras, while repeated interruption episodes may reflect enduring individual‐level barriers rather than pandemic‐related healthcare disruption. These findings suggest that interventions should focus not only on preventing care interruption but also on supporting sustained retention after re‐engagement.

**TABLE 1:**
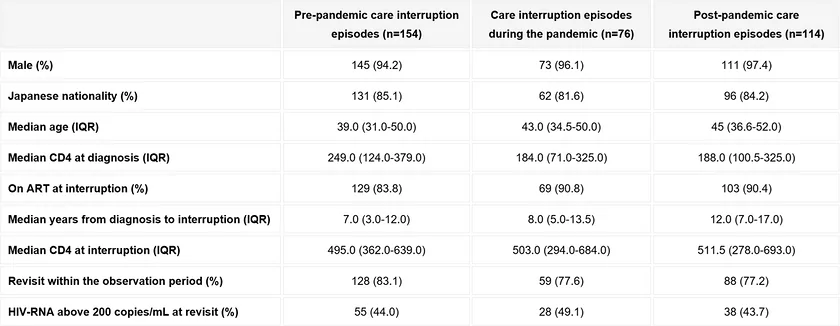
P201 | Characteristics of care interruption episodes.

### P202 Changes in Pattern of Adherence to Oral Antiretrovirals by Pharmacy Refill in a Large Cohort of People With HIV Over 11 Years

Alessandra A. M. Pagani^1^, Ludovica Mazzoleni^1^, Francesca Facciolo^1^, Michela Franzin^1^, Roberto Teocchi^2^, Laura Comi^3^, Federica Borghi^3^, Fulvio Crippa^3^, Diego Ripamonti ^3^

^1^Hospital Pharmacy Unit, ASST Papa GIOVANNI XXIII, Bergamo, Italy; ^2^Information Systems and Information & Communication Technology (ICT) Unit, ASST Papa GIOVANNI XXIII, Bergamo, Italy; ^3^Infectious Diseases Unit, ASST Papa GIOVANNI XXIII, Bergamo, Italy

**Background**: A high level of treatment adherence is required to persistently suppress HIV replication, although its pattern may be dynamic over time in single individuals and not all subjects are eligible for single tablet regimens (STRs), due to the current or historical drug‐resistance. We aim to assess the pattern of ART adherence by refill pharmacy over 11 years in a large cohort of people with HIV (PWH) on different oral regimens.

**Methods**: Data were retrieved from the electronic database of a single hospital pharmacy in northern Italy, providing up to 3‐month oral medication supply. PWH (≥18 years) receiving ART since 1 January 2015−31 December 2025 were included. Annual adherence rate was calculated by the number of daily doses dispensed from the pharmacy over 365 days per each year. Adherence category per year were grouped as ≤70, 71–80, 81–90 and ≥91% of the annual prescribed dose.

**Results**: A total of 4641 PWH had at least 1 access to the hospital pharmacy, over the study period. A total of 1331 (28.6%) were female, median (IQR) age was 47 (38–53) years, 907 (19.5%) were born outside Italy, while HIV risk factor was heterosexual, MSM and IVDUs for 48%, 24% and 25%, respectively. Table 1 shows the mean adherence rates over 11 years which were < 70, 71–80, 81–90 and > 91% in 8%, 2.6%, 5.1% and 84.3%, respectively. A total of 90% had adherence rate persistently ≥91% over the entire study period. Mean adherence rate in 2020, during the COVID outbreak, was similar to the other time periods. Over 11 years, the proportion of PWH on STRs changed from 32% to 76% on 2 pills/daily from 36% to 21% on ≥3 pills/daily from 32% to 3%.

**Conclusions**: A high proportion of PWH showed a high level of adherence by pharmacy refill without substantial change over the last 11 years, despite the proportion of people on STRs increased up to 76%.

**TABLE 1:**
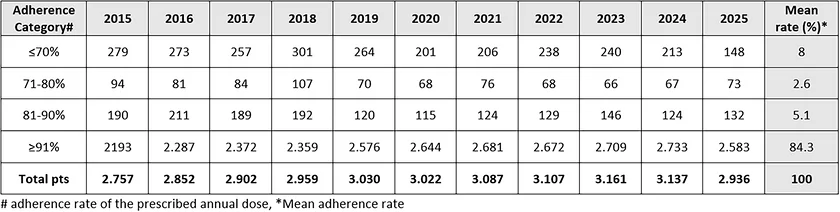
P202 | Number of patients by adherence categories and calendar year.

### P203 Community HERO Treatment Survey: Experiences of People With HIV in Taiwan

Yun‐Chung Lu^1^, Kris Mar^2^, Marcel Dams^3^, Nomfundo Eland^4^, Maureen Owino^5^, Carlos Saucedo^6^, Josep Mallolas^7,8^, Larkin Callaghan^9^, Michael Bogart^9^, Neia Prata Menezes^9^, MengJu Tsai^2^, Connie Kim ^9^

^1^We As One Association, Taipei, Taiwan; ^2^Gilead Sciences, Inc., Taipei, Taiwan; ^3^Aidshilfe NRW e.V., Cologne, Germany; ^4^Emthonjeni Counselling & Training, Cape Town, South Africa; ^5^York University, Toronto, Canada; ^6^Agenda LGBT A.C., Mexico City, Mexico; ^7^Infectious Disease Service, HIV Unit, Hospital Clínic Barcelona‐Instituto de Investigaciones Biomédicas August Pi I Sunyer, IDIBAPS, University of Barcelona, Barcelona, Spain; ^8^Centro de Investigación Biomédica en Red de Enfermedades Infecciosas, CIBERINFEC, Instituto de Salud Carlos III, Madrid, Spain; ^9^Gilead Sciences, Inc., Foster City, CA, US

**Background**: Understanding treatment experiences and priorities of people with HIV (PWH) is critical to inform person‐centred care. This Taiwan subanalysis provides insights into treatment experiences, including key motivators and factors influencing treatment initiation, maintenance and switching.

**Methods**: An ongoing cross‐sectional, 45‐min online survey was co‐developed with investigators and community advocates, conducted across 11 countries and translated into local languages. Eligible participants were ≥18 years with self‐reported HIV. The survey assessed experiences across the HIV care cascade. Descriptive analyses were conducted.

**Results**: A total of 208 participants from Taiwan were included; median age was 37 years, 95% were male at birth and 81% identified as men who have sex with men. Most participants reported currently receiving ART (96%), with a median of 8 years since ART initiation. Overall, 86% were on a single‐tablet regimen and 84% had switched treatment at least once. Rapid ART initiation was considered important by most participants (94%), primarily driven by perceived benefits in reducing transmission risk (75%), achieving rapid viral suppression (73%) and preventing disease progression (69%). At treatment initiation, key prioritized treatment features included convenience (once‐daily dosing) (47%), rapid and durable viral suppression (34%), tolerability (30%) and a high barrier to resistance (27%). For staying on treatment long‐term, the main prioritized treatment features were maintaining viral suppression (50%), dosing frequency (34%), reduced clinic visits (34%), tolerability (26%) and resistance barrier (25%). Prioritized treatment features of treatment switching were aligned, including viral suppression (44%), tolerability (32%), reduced clinic visits (31%) and resistance barrier (31%). Common reasons for prior switching included physician recommendation (30%), side effects (27%) and regimen simplification (27%). Adherence was supported by treatment features including achieving viral suppression (47%), long‐term tolerability (37%), dosing frequency (37%) and reduced clinic visits (37%). External support, including healthcare provider counselling, financial assistance and social support systems, was also mentioned as helpful for treatment adherence.

**Conclusions**: This Taiwan subanalysis demonstrates that viral suppression, tolerability and convenience are central to treatment decision‐making across initiation, maintenance and switching. Strong healthcare provider engagement and support systems further enhance adherence. These findings reinforce the importance of a person‐centred approach to optimize long‐term HIV outcomes.

### P204 Retaining Adolescents With HIV in Care Through Targeted Youth Camps in Kisumu, Kenya

Annabel Kokeyo

Sunburst Kenya, Kisumu, Kenya

**Background**: In Kenya, adolescents and young people account for a significant proportion of new HIV infections and HIV remains a leading cause of morbidity and mortality within this population. Psychosocial interventions are increasingly recognized as important in supporting treatment adherence and retention in care. Camp Sunburst‐Kenya was developed to provide social and psychological support through recreational and educational activities aimed at strengthening life skills, reducing stigma and improving retention in HIV care. This evaluation examined the effectiveness of Camp Sunburst‐Kenya in improving retention in care among adolescents living with HIV in Kisumu, Kenya.

**Methods**: HIV‐positive adolescents aged 10−19 years from Family AIDS Care and Education Services‐supported facilities participated in a 5‐day residential camp intervention involving peer engagement, art therapy, narrative writing, HIV education games, role plays and experience‐sharing sessions. Camp staff received training in leadership, stigma reduction and adolescent support. Two camp sessions were conducted in August 2013 and August 2014. Non‐camper controls were randomly selected and matched 2:1 to campers based on age, gender and antiretroviral therapy (ART) regimen. Demographic and clinical data were abstracted from medical records. Participants active in care 6 months after the camp period were classified as retained in care. A mixed‐effect generalized likelihood model was used to compare retention outcomes.

**Results**: A total of 71 campers and 142 non‐campers were analysed. The median age was 13.8 years (IQR 10.7−16.2) and 51% were female. Camp participants were more likely to be retained in care compared to non‐campers (aOR 4.03, 95% CI: 1.46–11.14, *p* < 0.01). Increasing age was associated with slightly lower retention (aOR 0.88, 95% CI: 0.78–1.00, *p* = 0.06). Adolescents on first‐line ART were more likely to remain retained in care than those not on ART (aOR 7.15, 95% CI: 3.06–16.7, *p* < 0.01).

**Conclusions**: Targeted residential youth camps may improve retention in care among adolescents living with HIV by addressing psychosocial challenges, stigma and peer support needs. Structured psychosocial interventions may strengthen engagement in HIV care and support treatment continuity.

### P205 Enhancing Adherence, Emotional Well‐Being and Psychological Resilience: Exploring Expressive Art Therapy for Children and Adolescents Affected by HIV

Annabel Kokeyo

Sunburst Kenya, Kisumu, Kenya

**Background**: Children and adolescents living with HIV often experience challenges related to medication adherence, emotional well‐being and psychological resilience. Traditional psychosocial interventions may not fully address these needs. Expressive art therapy offers a creative and supportive approach that encourages emotional expression, communication and coping. This study examined the impact of an expressive art therapy intervention on adherence, emotional regulation and psychosocial well‐being among children and adolescents affected by HIV.

**Methods**: A 12‐week expressive art therapy programme was conducted between October and December 2023 at Kisumu County Referral Hospital and Lumumba Health Centre. The intervention involved 100 children and adolescents aged 8−18 years, including 50 males and 50 females, participating in structured support group sessions facilitated by trained therapists and counsellors. Activities included tie‐dye, mask‐making, friendship bracelet creation, letter writing and performing arts. Sessions addressed themes related to emotional regulation, HIV education, medication adherence, disclosure, sexual health and grief. Pre‐ and post‐intervention assessments evaluated medication adherence, emotional distress, coping skills, self‐esteem and social connectedness.

**Results**: Participants demonstrated improved psychosocial and adherence outcomes following the intervention. Medication adherence improved substantially, while emotional distress scores decreased by 30%. Coping skills improved by 25% and most participants reported increased self‐esteem and emotional well‐being. Approximately 90% reported improved social connectedness and a stronger sense of peer support. Improvements were observed across both genders, with adolescents aged 13−18 years showing slightly greater gains compared to younger participants.

**Conclusions**: Expressive art therapy may serve as a valuable psychosocial support intervention for children and adolescents affected by HIV. The findings suggest potential benefits in improving adherence, emotional well‐being, coping capacity and peer connectedness within paediatric HIV care settings. Further research is needed to assess long‐term outcomes and the feasibility of integrating expressive art therapy into broader HIV care programmes.

### P206 High Acceptability and Adherence in a Pilot Randomized Controlled Trial of an Antiretroviral Therapy Real‐Time Electronic Adherence Monitoring and Response Intervention Among African American Men Who Have Sex With Men With HIV

Mark Dworkin ^1^, Sierra Upton^1^, Katherine Bezaitis^1^, Autumn Smith^1^, Corina Wagner^1^, Elle Martell^1^, Michael Reh^1^, Kara Herrera^1^, Li Liu^1^, Jessica Haberer^2,3^

^1^School of Public Health, University of Illinois Chicago, Chicago, IL, US; ^2^Center for Global Health, Massachusetts General Hospital, Boston, MA, US; ^3^Harvard Medical School, Boston, MA, US

**Background**: We developed the Antiretroviral Therapy Electronic Adherence Monitoring (A‐TEAM) intervention for African American men who have sex with men (AAMSM) with HIV—a behavioural intervention that employs a tiered support approach where electronic adherence monitoring (EAM) alerts: (i) the patient within hours of device non‐opening of planned medication‐taking time; (ii) a patient‐selected social support person when the device is not opened for 2 consecutive days; and (iii) a case manager when the device is not opened for 7 consecutive days or after 2 and 7 days of non‐opening, if no social support person is available. We performed a 6‐month pilot randomized controlled trial (RCT). We determined acceptability and observed if adherence differed between the study arms. We hypothesized that participants in the intervention arm would generally find the A‐TEAM approach acceptable.

**Methods**: AAMSM ages 18 years and older with non‐optimal ART adherence were recruited from 7 US states and randomized 2:1 to the intervention or usual care. ART adherence was measured with EAM for 6 months in all. In this descriptive analysis of the patients who completed 6 months follow‐up, we report intervention acceptability on a scale 1−10 (with increasing helpfulness) and Likert response for agreement with the approach. Overall adherence reflects the total number of days the device was opened out of the 180 days of follow‐up.

**Results**: Forty of 51 randomized AAMSM completed the study to date (20 intervention, 20 control). The median score for intervention acceptability was 10 and the median response for agreement with the intervention approach was “completely agree.” The median adherence was higher in the intervention arm (76%) than control arm (64%). Among intervention participants with complete data, 10 had a social support person and case manager and 8 had only a case manager who responded to missed alerts (median adherence 86% vs. 75%, respectively).

**Conclusions**: These pilot trial results suggest that a real‐time EAM and response intervention involving social support persons and case managers is acceptable to AAMSM with HIV and might have a beneficial effect on adherence. This study supports continued research with a larger RCT powered to establish evidence of efficacy.

### P207 When HIV Becomes Chronic but Never Ordinary: Experiences of Living With HIV and Disengagement From Care

Tea Nynne Sanders ^1^, Susanne Dam Nielsen^1,2^, Susanne Kriegel Pedersen^1^, Selina Kikkenborg Berg^2,3^, Ole Kirk^1,2^, Malene Missel^2,4^

^1^Department of Infectious Diseases, Copenhagen University Hospital Rigshospitalet, Copenhagen, Denmark; ^2^Faculty of Medical and Health Sciences, Institute of Public Health, University of Copenhagen, Copenhagen, Denmark; ^3^The Heart Center, Copenhagen University Hospital Rigshospitalet, Copenhagen, Denmark; ^4^Cardiothoracic Surgery, Copenhagen University Hospital Rigshospitalet, Copenhagen, Denmark

**Background**: HIV is now considered a manageable chronic condition due to advances in antiretroviral therapy (ART). However, HIV differs from other chronic conditions because of its history as a life‐threatening illness and the persistent challenges related to stigma and psychosocial factors. These dimensions may influence engagement in care but are not yet fully understood. The aim of this study was to explore how people living with HIV experience HIV as a chronic condition and how the historical and existential dimensions of HIV may influence engagement in care.

**Methods**: Inspired by Paul Ricoeur's phenomenological‐hermeneutic theory, this qualitative study included 14 in‐depth interviews with people living with HIV in Denmark who were currently or had recently experienced periods of disengagement from care. Participants were recruited through purposeful sampling. Interviews were conducted between March and May 2025, either face‐to‐face or by telephone (mean duration: 29 min) and analysed using Ricoeur's interpretive approach. Participants had a mean age of 49.5 years, 57% identified as men and 43% were born outside Denmark.

**Results**: The findings indicate that HIV is experienced as an existential life condition shaped by its historical context. Although ART has generally transformed HIV into a biomedically controllable illness, participants described HIV as a persistent existential presence shaped by memories, narratives, stigma and the ongoing demands of lifelong treatment. These experiences shaped participants’ relationship with HIV including periods of illness‐related weariness and disengagement from care.

**Conclusions**: Despite biomedical advances, some people living with HIV experience HIV as more than a chronic manageable condition. Historical memories, stigma and the demands of lifelong treatment remain intertwined with everyday life and may shape engagement in care. The findings highlight the importance of healthcare approaches that address not only clinical management but also the existential dimensions of living with HIV.

## Clinical Management Considerations: Women

### P208 PrEP Engagement Among Underserved Cisgender Women in the UK: Results From the PrEP‐Position Mixed‐Methods Study

Vian Rajabzadeh^1^, Sophie Strachan^1,2^, Melanie Smuk^1^, Nishat Halim^1^, Caro Camargo^1,2^, Chloé Pasin^1^, Megan Devonald^1^, Anqa Awan^2^, Taranjit Chana^3^, Kelsea Delatango^4^, Vimbai Mandaza^1^, Pamela Menzies‐Banton^3^, Zoch Nwaosu^3^, Juddy Otti^2,5^, Kyle Ring^1^, PrEP‐Position Steering Group Member^3^, Angelina Namiba^6^, Vanessa Apea^1^, Sara Paparini ^1^, Chloe Orkin^1^

^1^Queen Mary, University of London, London, UK; ^2^Sophia Forum, London, UK; ^3^PrEP‐Posiiton Steering Group, London, UK; ^4^Bare Radicals, London, UK; ^5^Africa Advocacy Foundation, London, UK; ^6^4M Net Mentor Mothers CIC, London, UK

**Background**: Oral PrEP uptake among cisgender women in the UK remains low at 3.5%. Injectable cabotegravir demonstrated superior efficacy to oral PrEP in cisgender women and is now NHS‐approved, making rollout an urgent priority. Data on PrEP engagement among underserved cisgender women in the UK, including those experiencing intimate partner violence (IPV), sex work and income insufficiency, remain limited.

**Methods**: In partnership with community organization Sophia Forum, we conducted a mixed‐methods co‐produced study (August 2025 to February 2026) with underserved groups. We report survey data analysed using descriptive statistics.

**Results**: Of 264 participants (Table 1), 132 were cisgender women, 84 cisgender men and 47 transgender and nonbinary (TNB). Cisgender women had the lowest PrEP awareness: nearly half had never heard of PrEP (46.2%), compared with 32.1% of cisgender men and 25.5% of TNB participants. Among those aware, cisgender women were least likely to be currently using PrEP (36.6%), with over half having never used it (50.7%). Structural vulnerability was concentrated in this group: over a third reported IPV in the past 12 months (36.5%), of whom fewer than half were currently using PrEP (43.5%); one in five had engaged in sex work (22.9%); and one in three reported insufficient income to meet basic needs (32.8%). Interest in injectable PrEP was high among cisgender women (81.2%) (Figure 1) and across all groups (88.2% among TNB participants; 94.6% among cisgender men). Among cisgender women, interest extended across other long‐acting and novel modalities: implants (42.3%), monthly pill (36.6%) and skin patch (35.2%) (Figure 1). In terms of preferred PrEP delivery settings, cisgender women most frequently selected GP surgeries and sexual health clinics (67.9% each), followed by pharmacy (44.6%) and community settings (36%).

**Conclusions**: Cisgender women—including those experiencing IPV, sex work and income insufficiency—showed the lowest PrEP awareness and uptake across all groups. Interest in injectable and other long‐acting modalities was high, indicating the barrier is access and reach rather than motivation. Delivery through the settings cisgender women most frequently identified, including GP surgeries and sexual health clinics, should be prioritized alongside outreach for those least likely to present to clinical services.

**FIGURE 1:**
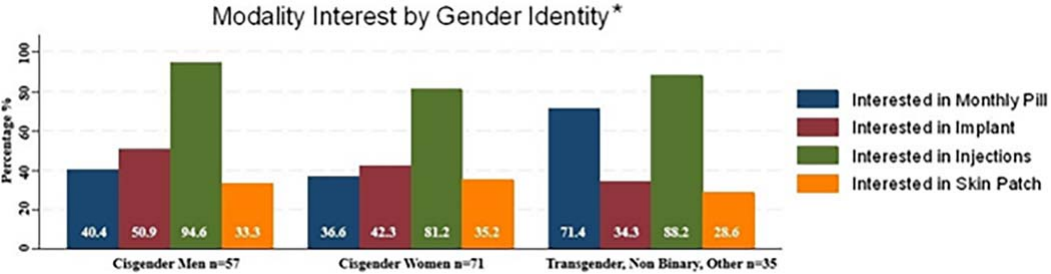
P208 | PrEP modality of interest by gender identity.

**TABLE 1:**
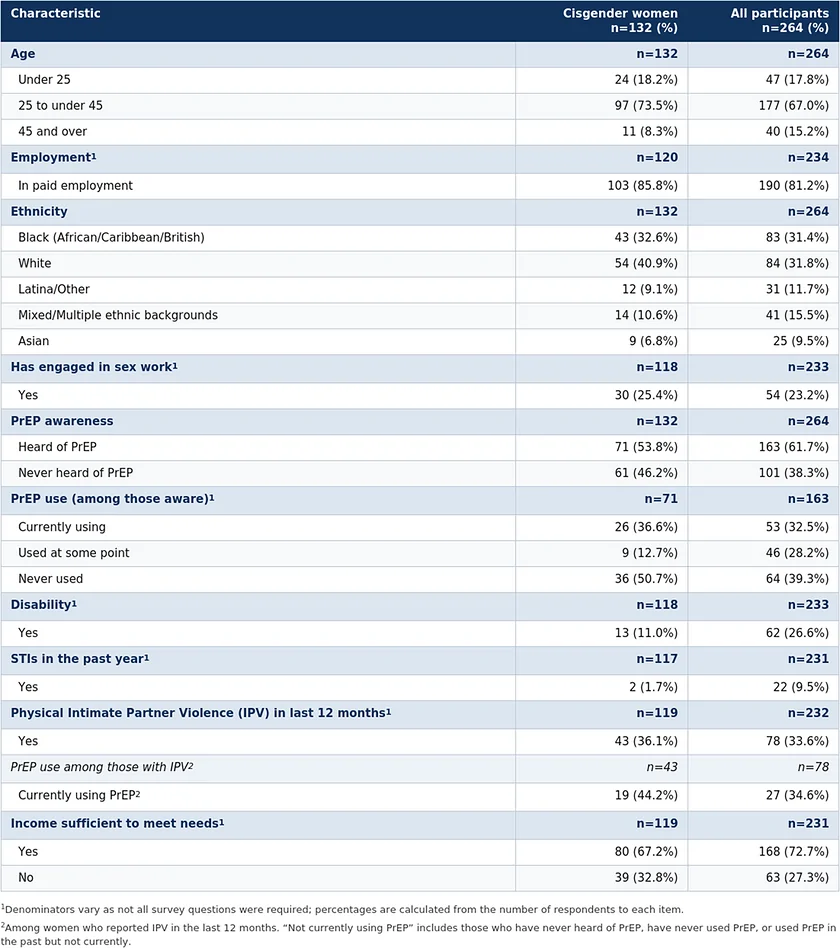
P208 | Characteristics of overall and cisgender women in the PrEP‐Position survey (*n* = 264).

### P209 Longitudinal Immune and Inflammatory Adaptations During Pregnancy in Women With HIV

Sandra Chaudron ^1,2^, Marius Zeeb^1,2^, Chloé Pasin^3^, Sara Schütz^1^, Mailin Waldecker^4^, Julia Notter^5^, Anna Hachfeld^6^, Paolo Paioni^7^, Annalisa Marinosci^8^, Matthias Cavassini^9^, Roger Kouyos^1,2^, Ladina Vonzun^10,11^, Irene A. Abela^1,2^, on behalf of Swiss HIV Cohort Study

^1^Department of Infectious Diseases and Hospital Epidemiology, University Hospital Zurich, University of Zurich, Zurich, Zurich, Switzerland; ^2^Institute of Medical Virology, University of Zurich, Zurich, Switzerland. 3 Department of Obstetrics, Inselspital, Bern University Hospital, University of Bern, Bern, Switzerland, Zurich, Switzerland; ^3^Blizard Institute, Queen Mary University of London, London, UK; ^4^Department of Obstetrics, Inselspital, Bern University Hospital, University of Bern, Bern, Switzerland; ^5^Division of Infectious Diseases and Hospital Epidemiology, University Hospital Basel, University of Basel, Basel, Switzerland; ^6^Department of Infectious Diseases, Inselspital, Bern University Hospital, University of Bern, Bern, Switzerland; ^7^Division of Infectious Diseases and Hospital Epidemiology, University Children's Hospital Zurich, Zurich, Switzerland; ^8^Service des Maladies Infectieuses, Hôpitaux Universitaires de Genève, Genève, Switzerland; ^9^Division of Infectious Diseases, Lausanne University Hospital and University of Lausanne, Lausanne, Switzerland; ^10^Department of Obstetrics, University Hospital Zurich, Zurich, Switzerland; ^11^Faculty of Medicine, University of Zurich, Zurich, Switzerland

**Background**: Pregnancy is associated with immunological adaptations that promote maternal–foetal tolerance while maintaining protection against infection. However, immune adaptations during pregnancy in women with HIV (WWH) receiving antiretroviral therapy (ART) remain incompletely characterized. We aimed to investigate longitudinal changes in HIV‐related immune markers and inflammatory protein profiles during pregnancy.

**Methods**: We analysed longitudinal clinical and proteomic data from pregnant WWH enrolled in the Swiss HIV Cohort Study (SHCS) between 1994 and 2024. The pregnancies were assessed at four time points: baseline (pre‐pregnancy), T1 (1–12 weeks), T2 (13–27 weeks), T3 (28–40 weeks). Immune outcomes included CD4, CD8, CD4/CD8 ratio and HIV RNA. Mixed‐effects models were used to assess changes across pregnancy, accounting for repeated pregnancies. Proteomic analyses using the Olink inflammation panel compared protein expression between paired T1/T3 samples from 194 pregnancies of virologically suppressed participants.

**Results**: 891 WWH contributed 1176 pregnancies, with 92.4% (1086) on pre‐conception ART (pART). Age and ethnicity differed across pregnancies without and with pART (16.7% vs. 12.2% aged < 25 years; 48.9% vs. 36.2% white ethnicity). CD4 counts declined transiently from baseline to T2, from 528 cells/µL (IQR 374–713) to 443 cells/µL (IQR 309–607), with partial recovery by T3 (473 cells/µL; IQR 333–641). CD8 counts declined initially then stabilized, resulting in a decreased CD4/CD8 ratio from baseline to T2 (−0.05, 95% CI: −0.08 to −0.02) due to greater CD4 decline. HIV RNA levels increased modestly in early pregnancy followed by a marked decline later in gestation, with a −0.46 log10 RNA copies/mL (CI −0.54, −0.37) decrease from baseline to T3. These immunological changes were accompanied by differential expression of proteins involved in immune regulation, tissue remodelling and placental vascular adaptation, including PD‐L1, LIF‐R, CCL28, VEGFA and TWEAK (Figure 1).

**Conclusions**: Pregnancy in women with HIV receiving ART is associated with dynamic changes in cellular immunity and inflammatory protein expression. The observed proteomic profile supports coordinated immune regulation, tissue remodelling and placental adaptation, providing a framework for understanding immune changes during pregnancy in women with HIV.

**FIGURE 1:**
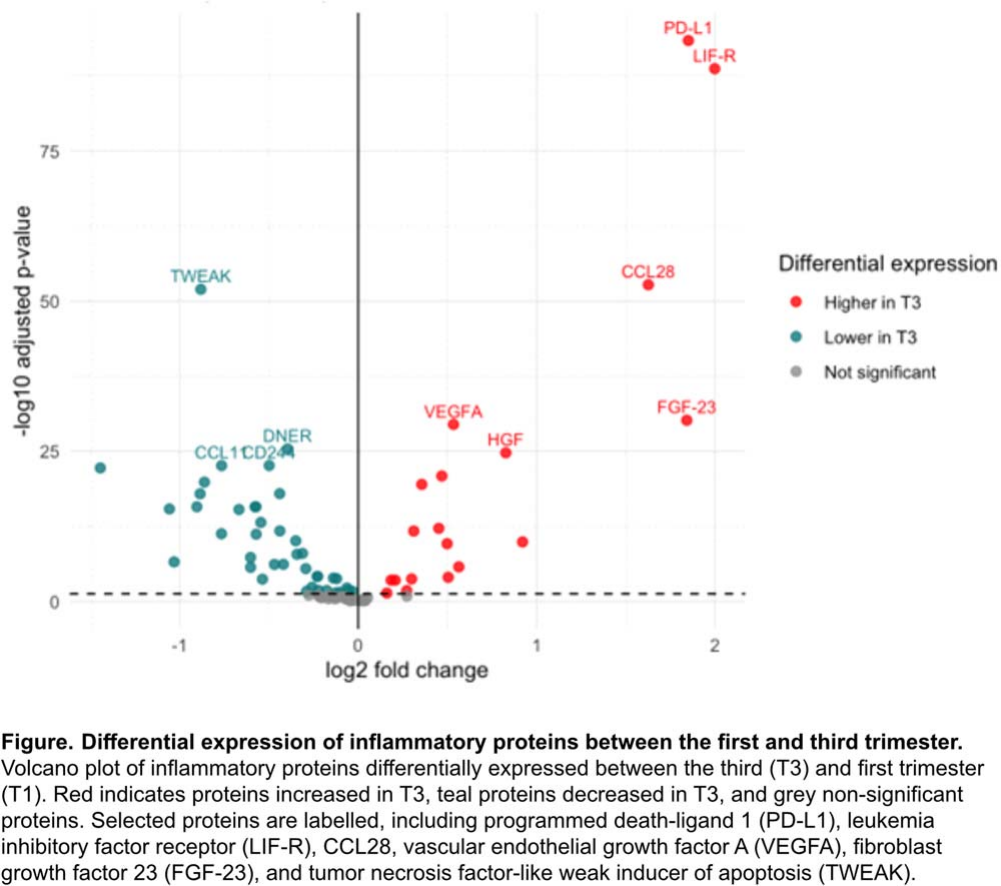
P209 |

### P210 Gender‐Specific Determinants of Frailty Among People With HIV Aged ≥50 Years (Over50 Cohort): Evidence for a Multidimensional Vulnerability Phenotype in Women With HIV

Patricia Echeverría ^1,2^, Jordi Puig^1,2^, Ana Martínez^3^, Itziar Arrieta^4^, Isabel Arnau^4^, Lucía Bailón^1,2^, Carla Estany^3^, Begoña Lemos^1^, Anna Bonjoch^1,2^, Robert Güerri^4,5^, Eugènia Negredo^1,6^

^1^Infectious Diseases Service, Hospital Universitari Germans Trias i Pujol, Fundació Lluita contra les Infeccions, Badalona, Spain; ^2^Universitat Autònoma de Barcelona, Barcelona, Spain; ^3^Fundació Lluita contra les Infeccions, Badalona, Spain; ^4^Hospital del Mar, Barcelona, Spain; ^5^CIBERINFEC, Madrid, Spain; ^6^Faculty of Medicine, University of Vic‐Central University of Catalonia (UVic‐UCC), Vic, Spain

**Background**: Gender differences in ageing among people with HIV (PWH) remain poorly characterized. Women with HIV (WWH) may experience more complex ageing trajectories due to the interplay of biological, clinical and psychosocial factors.

**Methods**: We performed a cross‐sectional analysis of the Over50 cohort, including PWH aged ≥50 years receiving care at two tertiary hospitals in Spain. Participants underwent a comprehensive geriatric assessment covering demographic, clinical, functional, neurocognitive, psychological and social domains. We aimed to characterize gender‐related differences among PWH and to identify gender‐specific frailty patterns by assessing independent associations between frailty and multidimensional determinants through stratified multivariable analyses. Frailty was defined according to the Fried criteria.

**Results**: Among 588 participants, 139 (23.6%) were cisgender WWH. Despite being slightly younger and immunologically more preserved, WWH showed a higher burden of age‐related conditions, including musculoskeletal disease (47% vs. 28%), frailty (17% vs. 9%), depressive symptoms (45% vs. 30%), sleep disturbances (10% vs. 5%) and cognitive complaints (23% vs. 11%), whereas cardiovascular and renal disease were more prevalent in MWH (48% vs. 35% and 22% vs. 15%, respectively). In multivariable analyses, frailty in WWH was associated with musculoskeletal disease (OR 3.85, 95% CI: 1.72–8.61, *p* < 0.01), cognitive impairment (OR 3.21, 95% CI: 1.45–7.10, *p* < 0.01), depressive symptoms (OR 2.67, 95% CI: 1.28–5.54, *p* < 0.01) and malnutrition (OR 2.14, 95% CI: 1.02–4.51, *p* = 0.04); while in MWH, frailty was also independently associated with musculoskeletal disease (OR 2.21, 95% CI: 1.34–3.66, *p* < 0.01), cognitive impairment (OR 2.54, 95% CI: 1.48–4.35, *p* < 0.01) and malnutrition (OR 2.36, 95% CI: 1.33–4.18, *p* < 0.01), but also with older age (OR 2.94, 95% CI: 1.85–4.68, *p* < 0.001), whereas depressive symptoms and sleep disorders were not independently associated.

**Conclusions**: WWH ageing carries a disproportionate burden of frailty, musculoskeletal comorbidity and psychosocial vulnerability despite younger age and better immune status. Frailty shows clear gender‐specific patterns, with a multidimensional phenotype in WWH driven by functional, neurocognitive and psychosocial factors, whereas in MWH it remains more closely linked to ageing. These findings support the need for gender‐responsive, multidisciplinary care integrating geriatric, mental and musculoskeletal health to optimize ageing outcomes in PWH.

### P211 High Burden of Dyslipidaemia and Hypertension Despite Virological Control in Women Living With HIV Over 50 Years

Joana Marinho ^1^, Filipe Medeiros^1^, Hugo Graça^2^, Luísa Lopes^2^, Cristina Valente^2^

^1^Infectious Diseases Department Coimbra Health, Coimbra, Portugal; ^2^Infectious, Coimbra, Portugal

**Background**: Perimenopausal and postmenopausal women living with HIV (WLWH) experience an increased burden of cardiovascular disease due to the combined factors—menopause, long‐term antiretroviral therapy (ART) exposure, dyslipidaemia, hypertension, diabetes, osteoporosis [1, 3]. These cardiometabolic factors significantly contribute to morbidity within this population [2]. This study aimed to characterize the cardiometabolic profile of women living with HIV aged over 50 years who were followed at a tertiary hospital in 2025.

**Methods**: A cross‐sectional, observational study was conducted including WLWH aged over 50 years receiving follow‐up care at a tertiary hospital in 2025. The data were collected from clinical records including demographic characteristics, ART regimens, virological status and cardiometabolic comorbidities.

**Results**: A total of 473 women were included. The most frequent age group was 50–59 years (45.24%), followed by 60–69 years (34.89%) and ≥70 years (19.66%). Most individuals were Portuguese (76.96%), while 16.07% were African and 4.23% were from Latin America. The most commonly used ART regimen was dolutegravir/lamidivudine (48.41%), followed by bictegravir/emtricitabine/tenofovir (18.39%). Overall, 96.83% (*n* = 458) of women were virologically suppressed and only seven patients (1.48%) had a viral load > 200 copies/mL. The most common cardiometabolic comorbidities identified were: dyslipidaemia was present in 53.59%, arterial hypertension in 42.49%, osteoporosis in 14.89%, obesity in 12.89% and diabetes mellitus in 11.63%. Body mass index data were incomplete, with no recorded value in 60.67% of cases.

**Conclusions**: Despite excellent virological control, WLWH demonstrated a substantial burden of cardiometabolic comorbidities, particularly dyslipidaemia and hypertension. These findings highlight the importance of a multidisciplinary women's health team—including gynaecology, internal medicine, endocrinology, cardiology and rheumatology—to incorporate cardiovascular, metabolic and bone health assessments for ageing WLWH [1, 2, 4, 5].

**References**

1. Mehta JM, Manson JE. The menopausal transition period and cardiovascular risk. Nature Reviews Cardiology. 2024;21:203–211.

2. Hsue PY, Waters DD. HIV infection and coronary heart disease: Mechanisms and management. Nature Reviews Cardiology. 2019;16:745–759.

3. Kentoffio K, Temu TM, Shakil SS, Zanni MV. Cardiovascular disease risk in women living with HIV. Current Opinion in HIV and AIDS. 2022;17(5):270–278.

4. Graham EE et al. Collection of menopause data in studies of women living with HIV: A systematic literature review. HIV Medicine. 2024.

5. Jamshaid M et al. Bone loss and fractures in post‐menopausal women living with HIV: A narrative review. Pathogens. 2024.

### P212 Late Antenatal Booking Among Women Living With HIV in England: Findings From National Surveillance Data 2020–2024

Helen Peters ^1^, Laura Byrne^2^, Rebecca Sconza^1^, Kate Francis^1^, Yvonne Gilleece^3^, Claire Thorne^1^

^1^Integrated Screening Outcomes Surveillance Service, UCL Great Ormond Street Institute of Child Health, London, UK; ^2^St George's University Hospitals NHS Foundation Trust, London, UK; ^3^Brighton & Sussex Medical School, University Hospitals Sussex NHS Foundation Trust, Brighton, UK

**Background**: Timely engagement with antenatal care (ANC) is essential for optimizing maternal health and pregnancy outcomes, particularly in women living with HIV, including approximately 10% undiagnosed at conception. Early ANC booking facilitates prompt screening, initiation/optimization of antiretroviral therapy (ART) and appropriate clinical management throughout pregnancy.

**Methods**: The Integrated Screening Outcomes Surveillance Service, commissioned by the NHS Infectious Diseases in Pregnancy Screening Programme, conducts population‐level surveillance of pregnancies in women diagnosed with HIV in England. We analysed all pregnancies resulting in livebirth/stillbirth in women booked 1 April 2020–31 March 2024, reported by 30 September 2025. “Late” ANC booking was defined as > 10 weeks gestation (NICE guidelines). Logistic regression was used to identify factors associated with late booking among women diagnosed pre‐pregnancy.

**Results**: Among 1993 pregnancies in 1815 women, 42.9% (855/1993), 46.9% (934/1993) and 10.2% (204/1993) were booked at ≤10, 11–19 and ≥20 weeks, respectively, 12 unbooked. Late booking was more common among women undiagnosed at conception than those diagnosed pre‐conception (70.6% [192/272] vs. 55% [946/1721], respectively, *p* < 0.001). Late booking was more frequent among non‐UK born women who arrived in the UK ≤12 months pre‐pregnancy. Among women diagnosed pre‐pregnancy, 78.5% (153/195) of those recently arrived booked late, versus 41.1% in UK‐born women (137/333) and 55.1% of non‐UK born women arrived > 12 months pre‐pregnancy (654/1186), *p* < 0.001 (Figure 1). Corresponding proportions among women diagnosed during pregnancy were 83.1% (98/118), 69.0% (80/116) and 33.3% (12/36), respectively, *p* < 0.001. In adjusted analysis, maternal age > 35 years (vs. 25–29) (OR 1.5, 95% 1.12, 2.11), multiparity (OR 1.4, 95% CI: 1.11, 1.86), ART at conception (OR 0.60, 95% CI: 0.37, 0.96), Black African ethnicity (vs. White British) (OR 2.28, 95% CI: 1.30, 4.01), recent arrival (<12 months) versus UK‐born (OR 3.20, 95% CI: 1.78, 5.77) and adverse social issues (OR 1.46, 95% CI: 1.11, 1.84) and region of booking were significantly associated with late booking.

**Conclusions**: Late ANC‐booking is more frequent among women living with HIV than the general population, including women already aware of their diagnosis and higher among those undiagnosed at conception. The strong association with recent migration highlights the need for coordinated clinical and social support to promote earlier engagement with antenatal services.

**FIGURE 1:**
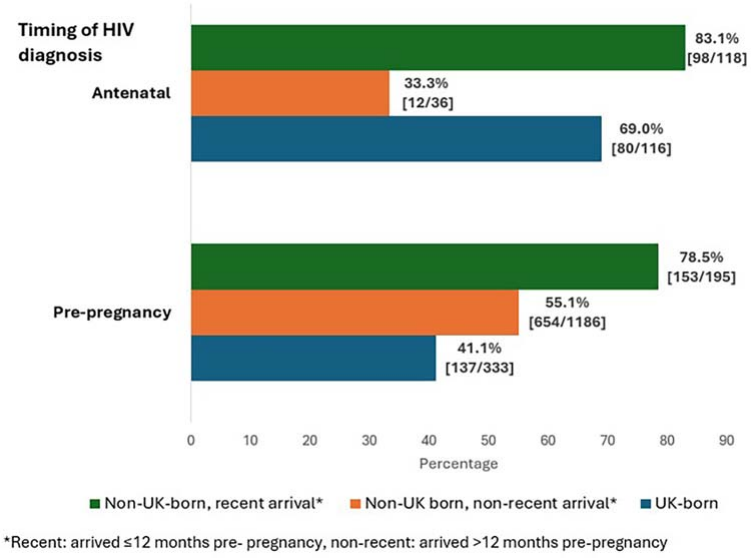
P212 | Proportion with late ANC booking by timing of UK arrival, stratified by timing of HIV diagnosis.

### P213 Treatment Persistence and Reasons for Discontinuation of Long‐Acting Cabotegravir/Rilpivirine Among Women Living With HIV: A Real‐World Multicentre Cohort Study

Layla Pagnucco ^1^, Maria Vittoria Cossu^2^, Maddalena Matone^2^, Annalisa Bergna^2^, Aurora Pozzi^1^, Agostino Riva^2^, Roberto Gulminetti^1^

^1^Infectious Diseases Unit, Medical Department, Fondazione IRCCS Policlinico San Matteo University of Pavia, Pavia, Italy; ^2^Department of Infectious Diseases Luigi Sacco Hospital, Regional Center for Infectious Diseases (CEREMI), Milan, Milano, Italy

**Background**: Long‐acting injectable cabotegravir plus rilpivirine (CAB/RPV LA) offers an alternative to daily oral antiretroviral therapy and has demonstrated high efficacy in maintaining virological suppression. However, women living with HIV (WLWH) have been underrepresented in pivotal clinical trials and real‐world data on treatment persistence and reasons for discontinuation remain limited. We aimed to characterize the frequency and reasons for CAB/RPV LA discontinuation in a real‐world cohort of WLWH in Italy.

**Methods**: We conducted a retrospective multicentre cohort study including adult WLWH who switched from a stable oral antiretroviral regimen to CAB/RPV LA between October 2022 and May 2026. Eligible participants had maintained HIV‐1 RNA <50 copies/mL for ≥6 months before switching. The primary outcome was treatment discontinuation and its underlying causes. Treatment discontinuation was defined as permanent interruption of CAB/RPV LA for clinical or patient‐related reasons; transfers of care were censored and excluded from discontinuation analyses. Secondary outcomes included maintenance of virological suppression and virological failure.

**Results**: Among 609 individuals initiating CAB/RPV LA, 127 (20.9%) were women. Median age was 54 years and eight (6.3%) had obesity (BMI ≥30 kg/m^2^). During follow‐up, 23 women (18.1%) permanently discontinued CAB/RPV. Confirmed virological failure occurred in three participants, all of whom achieved viral re‐suppression after switching to bictegravir/tenofovir alafenamide/emtricitabine (BIC/TAF/FTC). Treatment discontinuation was mainly driven by tolerability issues, including injection‐site pain (4/23), intolerance (3/23), fever (3/23) and injection‐site nodules (2/23). Other reasons included pregnancy planning (2/23), HBsAg positivity (2/23), logistical challenges (1/23) and physician decision (1/23). Apart from the three cases of virological failure, all remaining participants maintained virological suppression through week 96, demonstrating high treatment persistence. Notably, two women became pregnant while receiving CAB/RPV LA and elected to continue treatment; both maintained virological suppression throughout pregnancy and at delivery, with no adverse maternal outcomes reported.

**Conclusions**: In this real‐world multicentre cohort, CAB/RPV LA demonstrated high treatment persistence and durable virological effectiveness among WLWH. Most discontinuations were attributable to tolerability rather than virological failure, while pregnancy and other clinical events rarely required treatment interruption. These findings provide important sex‐specific real‐world evidence supporting the effectiveness and persistence of long‐acting CAB/RPV LA in women.

### P214 Pregnancy Outcomes Following Dolutegravir/Lamivudine Two‐Drug Therapy in Women Living With HIV: A Real‐World Case Series

Layla Pagnucco ^1^, Marianna Roccio^2^, Valentina Zuccaro^1,3^, Laura Maiocchi^1^, Raffaele Bruno^1,3^, Grazia Bossi^4^, Alessandro Borghesi^5^, Roberto Gulminetti^1^

^1^Infectious Diseases Unit, Medical Department, Fondazione IRCCS Policlinico San Matteo University of Pavia, Pavia, Italy; ^2^Fondazione IRCCS Policlinico San Matteo, Pavia, Department of Obstetrics and Gynecology, Pavia, PV, Italy; ^3^Department of Medicine, Surgical, Diagnostic and Pediatric Science, University of Pavia, Pavia, Pavia, Italy; ^4^Fondazione IRCCS Policlinico San Matteo, Pavia, Pediatrics Unit, Pavia, Italy; ^5^Fondazione IRCCS Policlinico San Matteo, Pavia, Neonatal Intensive Care Unit, Pavia, Italy

**Background**: Data on the use of the two‐drug regimen dolutegravir/lamivudine (DTG/3TC) during pregnancy remain limited. We evaluated the virological efficacy and maternal and neonatal safety of DTG/3TC in pregnant women receiving care at a HIV centre.

**Methods**: We conducted a retrospective case‐series analysis of pregnant women receiving DTG/3TC between October 2017 and May 2026. The primary efficacy endpoint was HIV‐1 RNA < 20 copies/mL at delivery. The primary safety endpoint was the occurrence of treatment‐related adverse events in mothers and infants through the postpartum visit. Collected data included maternal age, gestational age at delivery, timing of antiretroviral therapy (ART) initiation, mode of delivery, CD4 cell count, HIV‐1RNA during the first and second trimesters and at delivery and infant HIV status.

**Results**: Twenty‐one pregnant women receiving DTG/3TC were included, comprising both ART‐naïve women (*n* = 8) and women already receiving ART before conception. DTG/3TC was initiated or continued because of absent or positive HLA‐B*57:01 results, intolerance to abacavir or tenofovir, or patient preference. HIV‐1 RNA was monitored monthly according to routine clinical practice. At delivery, 20/21 (95.2%) women achieved virological suppression (<20 copies/mL). The only detectable viral load at delivery was 53 copies/mL in an ART‐naïve woman. One participant experienced a transient viral blip during the third trimester, likely related to concomitant administration of ART with iron/vitamin supplements; viral suppression was restored before delivery. Median CD4 cell count was 633 cells/µL during the first trimester and 627 cells/µL at delivery. Eleven women delivered vaginally and 10 by caesarean section, including eight elective procedures (breech presentation, previous caesarean section, detectable viraemia, high‐risk pregnancy or maternal request) and two emergency caesarean deliveries. All infants received neonatal antiretroviral prophylaxis, tested HIV‐negative during follow‐up and no congenital malformations were observed. One woman diagnosed with HIV infection at 32 weeks’ gestation initiated DTG/3TC while awaiting HLA‐B*57:01 testing, achieved viral suppression within 15 days and remained on this regimen until delivery.

**Conclusions**: In this case series, DTG/3TC was associated with high rates of maternal virological suppression, no cases of vertical HIV transmission and favourable maternal and neonatal safety outcomes. DTG/3TC may represent a treatment option during pregnancy when standard integrase inhibitor‐based three‐drug regimens are not suitable. Larger prospective studies are warranted to confirm these findings.

### P215 Abstract Withdrawn

### P216 Physical and Psychosocial Vulnerability Among Women Ageing With HIV

Andronikos Spyrou, Emmanouela Kalogianni, Michalis Ploumidis, Georgios Adamis, Myrto Astriti

Infectious Diseases Department, G. Gennimatas General Hospital of Athens, Athens, Greece

**Background**: Women ageing with HIV remain underrepresented in studies evaluating patient‐reported outcomes and psychosocial wellbeing. We aimed to assess sex differences in physical quality of life, psychosocial burden and social support among people living with HIV (PLWH) followed at a tertiary HIV clinic in Greece.

**Methods**: We conducted a cross‐sectional study among PLWH followed between November 2024 and February 2025. Demographic, clinical and psychosocial data were collected using structured questionnaires. Anxiety symptoms were assessed using the Generalized Anxiety Disorder‐7 (GAD‐7), social support using the Sarason Social Support Questionnaire (SSQ) and quality of life using the SF‐12 questionnaire. Women were compared with men regarding demographic characteristics, multimorbidity burden, psychosocial outcomes and quality‐of‐life measures.

**Results**: A total of 480 PLWH participated in the study, including 78 women (16.3%) and 402 men (83.7%). Women were older than men (54.4 vs. 51.1 years, *p* = 0.018), with a higher mean number of comorbidities (1.42 vs. 1.14, *p* = 0.052). HIV duration was similar between sexes (14.7 vs. 14.3 years), while alcohol use was lower among women (AUDIT‐C: 1.14 vs. 1.76, *p* = 0.006). Mean GAD‐7 score was 6.4 among women versus 5.9 among men. Moderate‐to‐severe anxiety symptoms were common among women and 23.1% had a previously diagnosed stress/anxiety disorder under treatment compared with 16.8% of men. Women demonstrated significantly worse physical quality of life compared with men (SF‐12 physical QoL: 58.3 vs. 75.5, *p* = 0.020), whereas mental QoL scores were comparable between sexes. Regarding social support, women reported a similar number of available supports compared with men (2.46 vs. 2.56), but lower satisfaction with support networks (4.40 vs. 4.81, *p* = 0.047). Among women, lower physical QoL scores were associated with greater multimorbidity burden, with women reporting SF‐12 physical QoL < 60 having a mean of 1.75 comorbidities versus 1.30 among those with better physical QoL scores.

**Conclusions**: Women living with HIV demonstrated a pattern of increased physical and psychosocial vulnerability characterized by impaired physical quality of life, greater multimorbidity burden and lower satisfaction with social support despite comparable HIV duration. These findings highlight the importance of sex‐sensitive multidisciplinary HIV care integrating functional assessment and psychosocial support beyond virological monitoring alone.

### P217 Community HERO Treatment Survey: Understanding Drivers of Treatment Satisfaction and Adherence Among Cis Women With HIV

Alissar Moussallem^1^, Xavier Guillaume^1^, Marcel Dams^2^, Nomfundo Eland^3^, Maureen Owino^4^, Carlos Saucedo^5^, Yun‐Chung Lu^6^, Josep Mallolas^7,8^, Amina Omri^1^, Larkin Callaghan^9^, Michael Bogart^9^, Neia Prata Menezes^9^, Connie Kim ^9^

^1^Oracle Life Sciences, Paris, France; ^2^Aidshilfe NRW e.V., Cologne, Germany; ^3^Emthonjeni Counselling & Training, Cape Town, South Africa; ^4^York University, Toronto, ON, Canada; ^5^Agenda LGBT A.C, Mexico City, Mexico; ^6^We As One Association, Taipei City, Taiwan; ^7^HIV Unit, Infectious Diseases Service, Hospital Clínic, Barcelona, Spain; ^8^Instituto de Investigaciones Biomédicas August Pi i Sunyer (IDIBAPS); Faculty of Medicine, University of Barcelona, Barcelona, Spain; ^9^Gilead Sciences, Inc., Foster City, CA, US

**Background**: Cis women remain underrepresented in HIV studies, limiting understanding of gender‐specific treatment experiences. Understanding how regimen characteristics and patient‐reported outcomes interact is critical to optimizing adherence among cis women with HIV.

**Methods**: The Community HERO Treatment Survey is an ongoing, cross‐sectional, online questionnaire conducted among adult persons living with HIV (PWH). The survey assessed history of antiretroviral therapy (ART), treatment satisfaction using the HIV Treatment Satisfaction Questionnaire status version (HIVTSQs), adherence based on self‐report and associated barriers and facilitators. Analyses focused on cis women, with comparisons to cis men to contextualize differences, using descriptive statistics.

**Results**: A total of 2532 PWH participated, of whom 561 were cis women. Demographic and clinical characteristics are shown in Table 1. Of those on treatment with available data, 72.5% (363/501) were on a single tablet regimen (STR), with B/F/TAF being the most common regimen (17.6%, 88/501). The median HIVTSQs score among all cis women on ART was 52.0 [IQR 45.0–58.0]. HIVTSQs scores were significantly higher among women on STRs compared to multiple tablet regimens (MTR) (*p* < 0.001) (median: 53.0 [47.0–59.0] vs. 47.0 [36.5–52.0]), with the highest scores observed among those on B/F/TAF (54.5 [47.5–59.2]). Adherence challenges were examined by gender and regimen type. Overall, 18.2% (91/501) of cis women reported difficulty with ART adherence versus 12.3% (211/1715) of cis men. Women receiving STRs reported significantly less adherence challenges than those on MTRs: 15.3% (55/363) versus 27.3% (27/99), *p* = 0.005. The most frequently reported barriers among cis women with adherence challenges were mental health reasons and financial costs, while cis men most frequently reported financial costs and limited social support (Figure 1). Notably, social support was more often identified as a facilitator among cis women than cis men: 56.0% (51/91) versus 48.8% (103/211), *p* = 0.015.

**Conclusions**: In this sample of cis women with HIV, STR use was associated with higher treatment satisfaction and fewer reported adherence challenges. Despite the cross‐sectional design, findings highlight the need for approaches that combine regimen simplicity and targeted support to optimize ART adherence and long‐term care in cis women.

**FIGURE 1:**
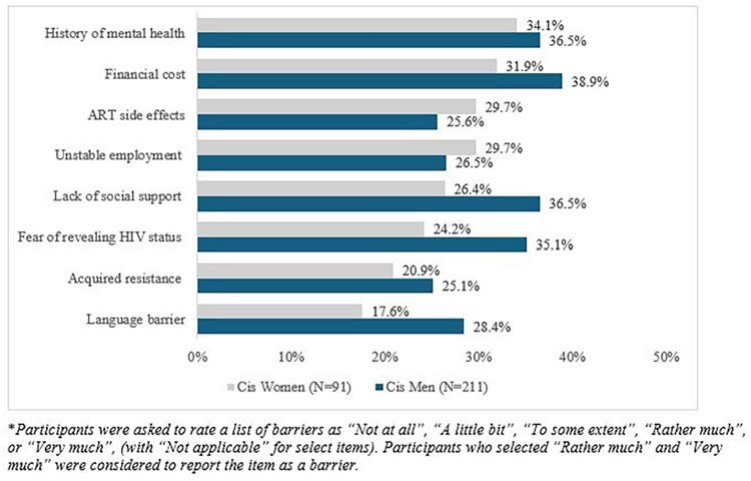
P217 | Most reported barriers* to ART adherence among cis women and cis men with adherence challenges.

**TABLE 1:**
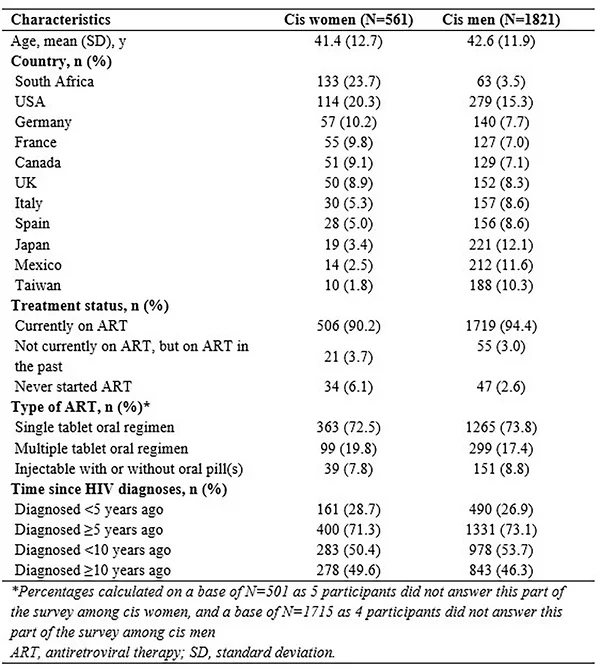
P217 | Participant demographic and clinical characteristics.

### P218 Menopause‐Associated Metabolic Changes in Women Living With HIV Switching to Bictegravir/Emtricitabine/Tenofovir Alafenamide: A Real‐World Analysis

Vito Speciale ^1^, Andrea Gasperin^2^, Giulia Mancarella^2^, Daniela Di Trento^2^, Lorenzo Ansaldo^2^, Margherita De Masi^2^, Lorenzo Mazzanti^1^, Livia Bresciani^1^, Salvatore Iaquinandi^1^, Serena Vita^3^, Anna Carraro^4^, Valeria Filippi^4^, Caterina Pasquazzi^4^, Cosmo Del Borgo^2^, Miriam Lichtner^3,4^, Raffaella Marocco^2^

^1^Sapienza, University of Rome, Rome, Italy; ^2^Infectious Disease Department, Santa Maria Goretti Hospital, Latina, Italy; ^3^Department of NESMOS, Sapienza, University of Rome, Rome, Italy; ^4^Infectious Disease Department, Sant'Andrea Hospital, Rome, Italy

**Background**: Women living with HIV (WLWH) represent an increasingly ageing population, with menopause potentially contributing to metabolic and cardiovascular risk. Data on menopausal WLWH receiving integrase inhibitor‐based regimens remain limited. We evaluated viro‐immunological and metabolic outcomes in WLWH switching to bictegravir/emtricitabine/tenofovir alafenamide (B/F/TAF) by menopausal status.

**Methods**: We performed a retrospective dual‐centre observational study including WLWH switching to B/F/TAF in routine clinical practice. Demographic, viro‐immunological and metabolic parameters were collected at switch, 12 and 24 months. Patients were stratified according to menopausal status. Continuous variables were analysed using non‐parametric tests and categorical variables using chi‐square or Fisher's exact test.

**Results**: Thirty‐seven WLWH were included: 15/37 (40.5%) menopausal and 22/37 (59.5%) non‐menopausal. Baseline viro‐immunological characteristics were comparable between groups. Median CD4 count was 679 versus 657 cells/mm^3^ (*p* = 0.536), while CD4/CD8 ratio was 0.9 versus 0.8 (*p* = 0.769) in menopausal and non‐menopausal women, respectively. Viral suppression rates were similar at baseline (80.0% vs. 86.4%; *p* = 0.670) and exceeded 90% in both groups during follow‐up. At baseline, menopausal women showed a less favourable metabolic profile, with higher triglyceride levels than non‐menopausal women (110 vs. 89 mg/dL; *p* = 0.086) (Table 1). At 12 months, this difference became significant (102 vs. 74.5 mg/dL; *p* = 0.0046). In the overall cohort, B/F/TAF maintained viro‐immunological efficacy, with improvement in CD4/CD8 ratio over time (0.9 at switch vs. 1.0 at 24 months; *p* = 0.001). Significant increases in weight and BMI were observed overall (64 kg at switch vs. 67 kg at 24 months, *p* = 0.003; BMI 24.5 vs. 25.8 kg/m^2^, *p* = 0.003). Weight gain appeared mainly driven by menopausal women. In this subgroup, median weight increased from 64 kg at switch to 70 kg at 24 months (*p* = 0.003), while BMI increased from 24.6 to 26.5 kg/m^2^ (*p* = 0.003). Conversely, non‐menopausal women showed non‐significant changes in weight and BMI (Figure 1).

**Conclusions**: Among WLWH switching to B/F/TAF, menopausal status was associated with increased metabolic burden despite preserved viro‐immunological efficacy. Menopausal women showed higher triglyceride levels and significant increases in weight and BMI, consistent with emerging evidence linking menopause and INSTI exposure to greater metabolic vulnerability [1–4]. Tailored cardio‐metabolic monitoring may, therefore, be warranted in ageing WLWH receiving INSTI‐based therapy.

**References**

1. Abelman R, et al. Switch to an integrase inhibitor leads to greater weight gain during and after menopause. Presented at CROI 2024.

2. Eckard AR, McComsey GA. Weight gain and integrase inhibitors. Curr Opin Infect Dis. 2020;33(1):10–19.

3. Markakis K, et al. Weight gain in adults receiving antiretroviral therapy: Mechanisms and clinical implications. Life (Basel). 2024;14:1367.

4. HIV i‐Base. Integrase inhibitors, TAF and weight gain: Review of current evidence. HIV Treatment Bulletin. 2023.

**FIGURE 1:**
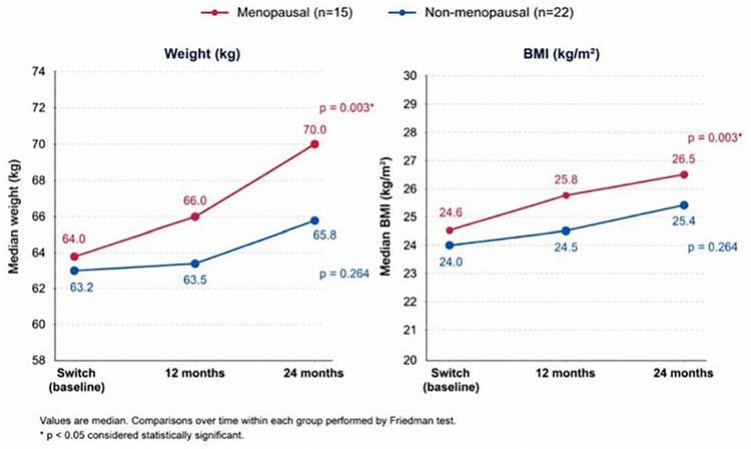
P218 | Longitudinal changes in weight and BMI according to menopausal status in women living with HIV switching to B/F/TAF.

**TABLE 1:**
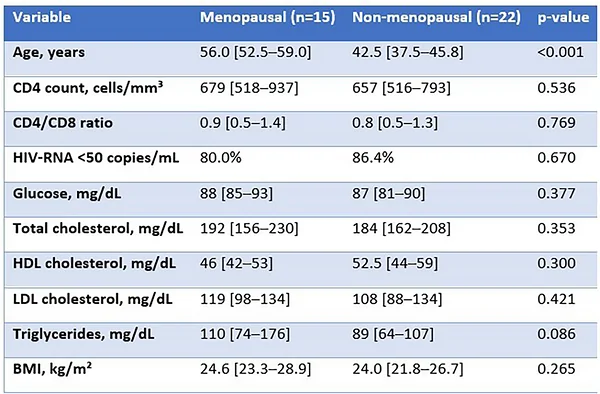
P218 | Baseline demographic, viro‐immunological and metabolic characteristics according to menopausal status.

### P219 Women Living With HIV and Menopause Status: Real‐World Evaluation of ART Efficacy, Metabolic Profiles and Comorbidities

Giuseppe Nunnari ^1^, Serena Spampinato^1,2^, Marta Scifo^1^, Dalida Bivona^1^ Andrea Marino^1^, Bruno Santi Cacopardo^1^, Alessandro Franzò^1^, Roberto Bruno^1^, Nunziatina Villari^1^, Benedetto Maurizio Celesia^1^, Paolo Fagone^2^, Grazia Scuderi^1^

^1^Unit of Infectious Diseases, ARNAS “Garibaldi Nesima” Hospital, Department of Clinical and Experimental Medicine, University of Catania, Catania, Italy; ^2^Department of Biomedical and Biotechnological Sciences, University of Catania, Catania, Italy

**Background**: Women living with HIV (WLWH) on antiretroviral therapy (ART) are ageing [1]. The combined impact of chronic viral infection, prolonged therapeutic exposure and physiological oestrogen decline is critical. Menopause may occur earlier and present with greater severity in WLWH; both HIV and menopause are independent negative predictors of bone mineral density [2, 3]. This study evaluated the multisystemic impact of menopausal status on metabolic, cardiovascular and bone health in virologically suppressed WLWH.

**Methods**: This retrospective, observational study evaluated 90 WLWH on ART, recruited at the Garibaldi Nesima Hospital in Catania. Participants were stratified by menopausal status (WHO criteria). Clinical and laboratory parameters were analysed using the Mann–Whitney U and Kolmogorov–Smirnov tests. Multivariable analysis adjusted for relevant variables. Spearman's correlation explored relationships among continuous variables. The ethic committee approved the study (SHICohort‐protocol code 34/17 of 22 March 2017). All participants provided written informed consent.

**Results**: The cohort comprised 90 WLH, 65 (72.2%) were postmenopausal. Postmenopausal women had a significantly higher overall comorbidity burden (80.0% vs. 40.0%, *p* < 0.001), including hypertension (56.9% vs. 12.0%, *p* < 0.001), dyslipidaemia (73.8% vs. 20.0%, *p* < 0.001) and osteoporosis (38.5% vs. 4.0%, *p* = 0.0012) (Figure 1). No significant differences emerged in CD4+ or CD8+ counts, CD4/CD8 ratio or viral load, indicating preserved immunovirological status across menopausal strata. Univariate analysis revealed significantly higher fasting glucose (89.6 vs. 73.5 mg/dL; *p* < 0.001), aspartate aminotransferase (28.0 vs. 21.4 U/L; *p* < 0.001), alanine aminotransferase (26.9 vs. 15.2 U/L; *p* < 0.001) and haemoglobin (13.6 vs. 12.3 g/dL; *p* < 0.01) in post‐menopausal women. After multivariable adjustment, menopausal status remained independently associated with elevated transaminases and haemoglobin levels. Platelet count was positively associated with CD4+ T cells (ρ = 0.278, *p* = 0.011) and negatively associated with GPT (ρ = −0.236, *p* = 0.032), further highlighting interactions between immune activation and liver function. Triglycerides were inversely correlated with CD4+ T cells (ρ = −0.251, *p* = 0.022) (Table 1).

**Conclusions**: Menopause independently amplifies cardiometabolic, hepatic and bone vulnerability in WLWH, whereas immune status and viral control remain largely determined by historical HIV factors rather than hormonal status. These findings highlight the importance of integrated care models addressing metabolic monitoring, cardiovascular prevention and bone health, to enable WLWH to age with comparable health and quality of life to the general population.

**References**

1. M. Smit et al., «Future challenges for clinical care of an ageing population infected with HIV: A modelling study», *Lancet Infect. Dis*., vol. 15, fasc. 7, pp. 810–818, lug. 2015, doi: 10.1016/S1473‐3099(15)00056‐0.

2. N. Andany et al., «Prevalence and correlates of early‐onset menopause among women living with HIV in Canada», *Menopause*, vol. 27, fasc. 1, pp. 66–75, gen. 2020, doi: 10.1097/GME.0000000000001423.

3. A. Sharma et al., «Human immunodeficiency virus (HIV) and menopause are independently associated with lower bone mineral density: Results from the Women's Interagency HIV study», *Clin. Infect. Dis*., vol. 75, fasc. 1, pp. 65–72, ago. 2022, doi: 10.1093/cid/ciab874.

**FIGURE 1:**
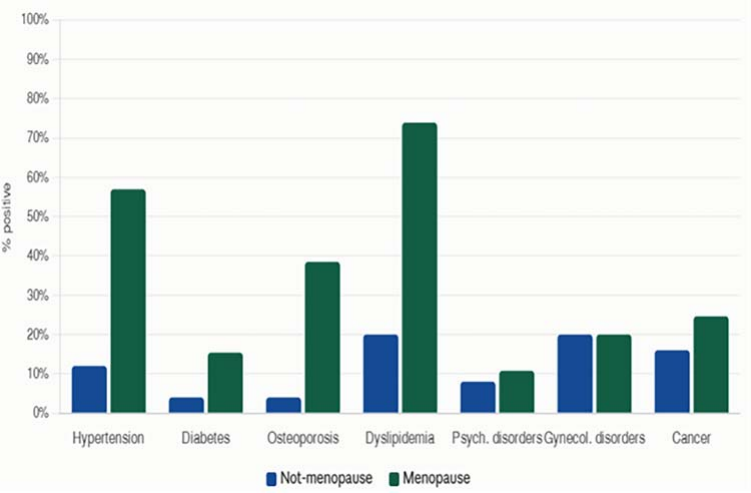
P219 | Percentage of subjects with comorbidities.

**TABLE 1:**
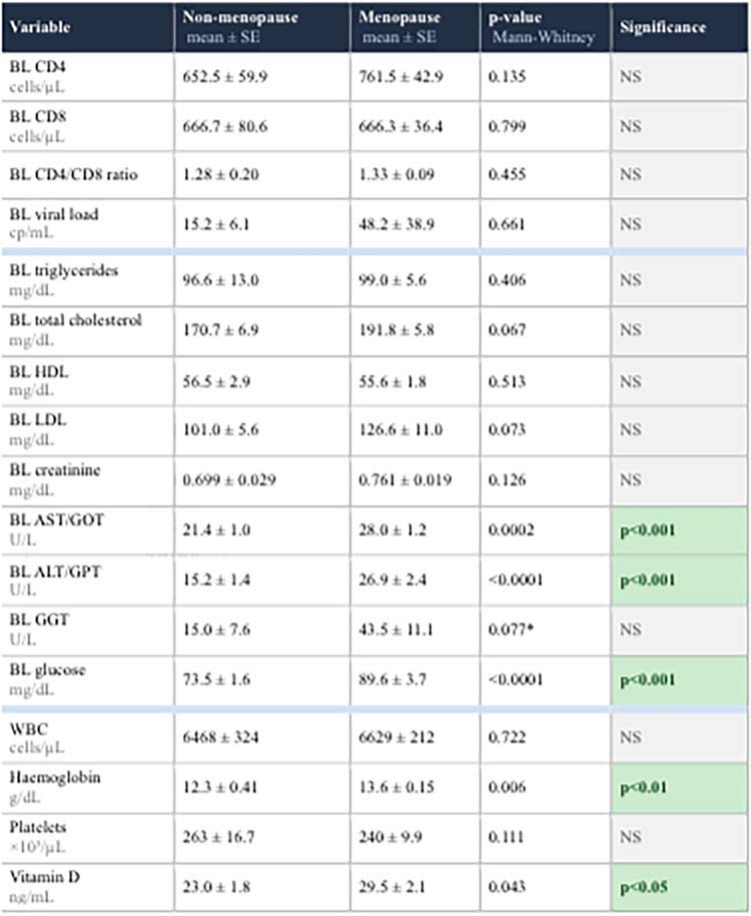
P219 | Current laboratory parameters in HIV‐positive women according to menopausal status. Values are expressed as mean ± standard error (SE). Statistical comparisons were performed using the independent‐samples Mann−Whitney U test. Significance threshold: α = 0.05. NS = not significant.

### P220 Kidney Function in Women Aged ≥50 Years Living With HIV: A Cross‐Sectional Analysis of a Portuguese Cohort

Hugo Graça, Filipe Medeiros, Luísa Lopes, Joana Marinho, Margarida Prata, Cristina Valente

Infectious Diseases, Local Health Unit of Coimbra, Coimbra, Portugal

**Background**: As people living with HIV age, kidney disease has emerged as an increasingly important comorbidity. Women remain underrepresented in studies evaluating renal outcomes in HIV, particularly older women who may be at increased risk due to age‐related decline in kidney function, comorbidities and cumulative antiretroviral exposure. We aimed to describe kidney function in women aged ≥50 years living with HIV.

**Methods**: We conducted a cross‐sectional study including women aged ≥50 years receiving HIV care at a Portuguese tertiary care centre. Demographic and clinical data were collected from electronic medical records. Kidney function was assessed using estimated glomerular filtration rate (eGFR) calculated with the CKD‐EPI equation and classified according to KDIGO GFR categories (G1–G5). Descriptive statistics were used to characterize the study population.

**Results**: A total of 473 women were included. Mean age was 62 ± 9 years, with a median age of 60 years (IQR 55–68). Participants were predominantly Portuguese (77.0%, *n* = 364), while women of Angolan (9.5%, *n* = 45), Brazilian (4.2%, *n* = 20) and Mozambican (3.8%, *n* = 18) origin represented the largest non‐Portuguese groups. Antiretroviral therapy was predominantly based on integrase inhibitors, with lamivudine/dolutegravir (49.5%) and bictegravir/emtricitabine/tenofovir alafenamide (18.6%) representing the most commonly prescribed regimens. Virological suppression (HIV RNA <50 copies/mL) was observed in 96.8% of participants (*n* = 458). Regarding kidney function, preserved renal function was observed in most participants, with 340 women (71.9%) classified as G1 and 78 (16.5%) as G2. Reduced kidney function (eGFR < 60 mL/min/1.73 m^2^) was observed in 55 women (11.6%), including 27 (5.7%) classified as G3a, 21 (4.4%) as G3b, 4 (0.8%) as G4 and 3 (0.6%) as G5. In this group, the median duration of antiretroviral therapy was 15.7 years (IQR 11.4–22.6), the median number of antiretroviral regimens was 3 (range, 1–7) and 25 women (45.5%) had been exposed to at least one regimen including tenofovir alafenamide.

**Conclusions**: Reduced kidney function was observed in 11.6% of women aged ≥50 years living with HIV. These findings highlight the importance of routine renal assessment in ageing women living with HIV and support further research into factors associated with kidney function decline and strategies to prevent progression of renal impairment in this population.

### P221 Long‐Term Effectiveness and Safety of Bictegravir/Tenofovir Alafenamide/Emtricitabine in Women Living With HIV in Argentina

Cinthia Lamaizón, Diego Cecchini, Gaston Copertari, Martín Brizuela, Brenda Bacelar, Macarena Roel, Edgardo Bottaro, Isabel Cassetti

Helios Salud, Buenos Aires, Argentina

**Background**: Women living with HIV (WLWH) have been historically underrepresented in both clinical and real‐world studies, which has limited the availability of specific evidence and generated knowledge gaps regarding their response to antiretroviral therapy (ART). In this context, evaluating the long‐term effectiveness, safety and metabolic impact of bictegravir/emtricitabine/tenofovir alafenamide (B/F/TAF) in WLWH is of particular relevance.

**Methods**: Sub‐analysis of the BICTARG study, a retrospective study conducted in Argentina between 2019 and 2024, including WLWH who switched to B/F/TAF. Over 4 years, we assessed virological outcome (virological suppression defined as HIV RNA <50 copies/mL), adverse events and metabolic parameters (total cholesterol, HDL, LDL, triglycerides and fasting glucose).

**Results**: Among 818 WLWH who switched to B/F/TAF, the median (IQR) age was 47 (41–53) years. At baseline, 78% had virologic suppression and median (IQR) CD4+ T‐cell was 597 (393–840) cell/mm^3^. Half had at least one comorbidity; the most frequent were dyslipidaemia (44.5%), osteopenia‐osteoporosis (30.1%), obesity (26.6%) and hypertension (24.7%). The main reasons for switching were simplification (57%), prevention of toxicity (24%) and current toxicity (17%). Previous regimens included: 39% boosted protease inhibitors; 32% non‐nucleoside reverse transcriptase inhibitors; and 23% integrase inhibitors. The median (IQR) prior to switching to B/F/TAF for total cholesterol, HDL, LDL and triglycerides (mg/dL) was 193 (162–224), 53 (44–64), 112 (91–137) and 117 (85–166), respectively, and at 48 months was 206 (163–235), 55 (45–66), 119 (83–146) and 123 (99–187). Median (IQR) fasting glucose at baseline was 90 (84–97) mg/dL and 90 (89–94) mg/dL at 48 months. No statistically significant changes were observed in the metabolic parameters evaluated. Virological suppression rates at 12, 24, 36 and 48 months were 96%, 96%, 99% and 100%, respectively. No virological failures occurred and adverse events were reported in < 1%.

**Conclusions**: This sub‐analysis represents the first Argentine cohort exclusively composed of WLWH receiving B/F/TAF. In this population, B/F/TAF demonstrated durable virological suppression, a favourable safety profile and a neutral metabolic impact over 4 years of follow‐up.

### P222 Age‐Related Differences in Virological Suppression Among Women Living With HIV Receiving Antiretroviral Therapy: A Real‐World Brazilian Cohort

Pietra Stanicki, Karen Ingrid Tasca, Alexandre Naime Barbosa

UNESP – Faculdade de Medicina de Botucatu, Departamento de Infectologia, Botucatu, SP, Brazil

**Background**: Women living with HIV experience distinct biological, social and structural contexts across the life course that may influence antiretroviral therapy (ART) outcomes. Younger women are often disproportionately affected by socioeconomic vulnerability, caregiving responsibilities and adherence challenges, whereas older women may benefit from longer treatment exposure, greater clinical stability and consistent engagement in care. Evidence on age‐related differences in virological suppression among women in real‐world settings, particularly in middle‐income countries, remains limited.

**Methods**: This observational cohort study included women aged ≥18 years receiving ART between January 2020 and July 2022 at a Brazilian HIV reference centre. Participants were stratified into two groups: 18–49 years and ≥50 years. Virological suppression was defined as HIV‐1 viral load < 50 copies/mL. ART regimens were categorized as simplified or standard triple‐drug therapy. Demographic, clinical and immunological variables were analysed. Associations were assessed using chi‐square tests and multivariable logistic regression models were constructed with virological suppression as the primary outcome.

**Results**: Among 362 eligible women, 334 were included in the final analysis; loss to follow‐up and transfer of care were the main reasons for exclusion. Virological suppression was significantly higher among women aged ≥50 years than among those aged 18–49 years (91.5% vs. 78.2%; *p* = 0.001). Treatment strategies differed by age group, with standard triple‐drug regimens prescribed more frequently among younger women (88% vs. 32%; *p* < 0.001). In adjusted analyses, use of simplified ART regimens was independently associated with virological suppression (*p* = 0.011), while age showed a borderline independent association (*p* = 0.056).

**Conclusions**: Older women living with HIV achieved significantly higher rates of virological suppression than younger women in this real‐world cohort. Use of simplified ART regimens was independently associated with virological suppression, whereas age showed only a borderline independent association after adjustment. These findings suggest that differences in treatment strategy alone do not fully explain age‐related differences in virological outcomes and support continued investigation of factors influencing HIV treatment success among younger women.

## Clinical Management Considerations: Late Presenters

### P223 Late Presentation and Advanced HIV Disease in Latin America After the COVID‐19 Pandemic: A Multi‐Country Analysis

María Greco ^1^, Pedro Zitko^2^, Carlos Beltrán^3^, Isabel Cassetti^4^, Ricardo Alcantar Morales^5^, Ana Arauz^6^, Lilian Astocondor^7^, Jesús Dawaher^8^, Idaura González^9^, Cinthia Lamaizón^4^, Eric Delgado^10^, Mónica Mantilla^11^, Alejandro Afani^12^, Ernesto Martínez Buitrago^13^, Rodolfo Pinzón Meza^14^, Javier Ricart^15^, Ana Vega Calderón^16^, David Forero^17^, Jenny Dreyfus^18^, Javier Mota^19^, Leonardo Arévalo^11^, on behalf of Latin American HIV Workshop Study Group

^1^Infectious Diseases, Hospital Español, La Plata, Argentina; ^2^Unit for Healthcare Research, Hospital Barros Luco Trudeau, Santiago, Chile; ^3^Infectious Diseases, Hospital Barros Luco Trudeau, Santiago, Chile; ^4^Infectious Diseases, Helios Salud, Buenos Aires, Argentina; ^5^Dirección Médica, Clinica Condesa, Ciudad de Mexico, Mexico; ^6^Infectious Diseases, Hospital Santo Tomás, Panamá, Panama; ^7^Infectious Diseases, Hospital Carrión, Callao, Peru; ^8^Infectious Diseases, Hospital Pablo A. Suárez, Quito, Ecuador; ^9^Infectious Diseases, Hospital de Antofagasta, Antofagasta, Chile; ^10^Programa VIH, VIVIR IPS, Bogotá, Colombia; ^11^Programa VIH, Virrey Solis IPS, Bogotá, Colombia; ^12^Centro VIH, Hospital Clínico Universidad de Chile, Santiago, Chile; ^13^Universidad del Valle, Cali, Colombia; ^14^Unidad de Atención Integral de VIH, Hospital Roosevelt, Guatemala, Guatemala; ^15^Hospital Francisco Muñiz, Buenos Aires, Argentina; ^16^Infectious Diseases, Hospital Calderón Guardia, San José, Costa Rica; ^17^Hospital Universitario de Caracas, Caracas, Venezuela; ^18^Centro Sanitario Santo Domingo, Santo Domingo, Dominican Republic; ^19^Hospital Militar Dr. C. Arvelo, Caracas, Venezuela

**Background**: Late presentation (LP) to HIV care (CD4+ count < 350 cells/µL) and advanced HIV disease (AD) (CD4+ count < 200 cells/µL or an AIDS‐defining event) remain public health challenges. In a previous analysis of 43,911 people entering HIV care at 101 Latin American centres (2013–2017), our group reported significant declines in AD (40.5% to 31.9%) and LP (63.7% to 54.1%) [1]. The COVID‐19 pandemic disrupted HIV testing and linkage to care, raising concerns about increased LP and AD. Evidence on subsequent trends remains limited. We assessed temporal trends in LP and AD during 2021–2024.

**Methods**: We conducted a multicentre, multicountry time‐series study using cross‐sectionally collected statistical data from 101 HIV centres across 14 countries belonging to the Latin American HIV Workshop Study Group. Data on people entering HIV care during 2021–2024 were stratified by age, sex and CD4+ count. A three‐level negative binomial random‐effects regression model was implemented. Uncertainty was incorporated through Monte Carlo simulation under distributional assumptions.

**Results**: We analysed data from 32,537 people entering HIV care at 84 centres in 13 Latin American countries. LP declined slightly, but not significantly, from 59.4% (95% CI: 53.8–66.6) in 2021 to 54.5% (95% CI: 46.2–58.4) in 2024. A similar pattern was observed for AD (34.9% [95% CI: 23.5–40.7] to 32.7% [95% CI: 25.7–40.3]). No significant sex differences were observed for LP or AD. Both outcomes increased with age, from 48.2% (95% CI: 47.4–49.0) among those aged 15–29 to 63.4% (95% CI: 61.7–64.7) among those aged ≥69 for LP and from 24.6% (95% CI: 24.0–25.3) to 40.4% (95% CI: 38.6–42.2) for AD in 2024 (Figure 1). Prevalence varied markedly across countries, ranging from 19.2% (95% CI: 18.7–19.7) in Argentina to 47.2% (95% CI: 45.6–48.8) in Guatemala for AD and from 37.2% (95% CI: 36.6–38.0) to 70.5% (95% CI: 69.4–71.6) for LP.

**Conclusions**: During 2021–2024, LP and AD declined only slightly and non‐significantly in Latin America. Both showed a clear increase with age and varied substantially across countries. HIV testing and linkage‐to‐care strategies should be tailored to populations at higher risk of LP while addressing regional disparities in access to diagnosis.

**Reference**

1. Beltrán C, Zitko P. Latin American HIV Workshop Study Group. Annual Epidemiological Report 2018. Available at: https://www.vila‐la.com/biblioteca. Accessed 26/06/2026

**FIGURE 1:**
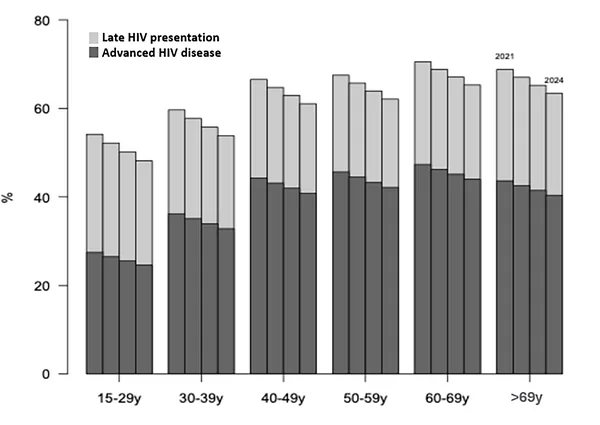
P223 | Late HIV presentation and advance HIV disease by age group, 2021–2024.

### P224 Clinical Complexity, Opportunistic Infections and Determinants of In‐Hospital Mortality in Young Adults Living With Perinatally Acquired HIV in a Reference Public Centre in Argentina

Javier Ricart, Ana Martina De Prada, Dana Mijalovsky, Laura Macías Moreno, Cinthia Giomi, Nicolas Nahoum, Matías Gastón Pérez, Matías Enzenhofer, Luis De Carolis, Natalia Soledad Vittar, Graciana Cárdenas, Gustavo Bruni, Sofía Bauer, María Florencia Villafañe Fioti, Emilse Vázquez, Rubén Solari

Hospital de Infecciosas “Francisco J. Muñiz”, Buenos Aires, Argentina

**Background**: Adults living with perinatally acquired HIV (PaHIV) represent a distinct population with lifelong viral and antiretroviral therapy (ART) exposure. As they transition into young adulthood, challenges in care continuity often lead to hospitalizations for severe complications, yet prognostic markers remain under‐characterized [1, 2]. This study aimed to evaluate the clinical profile of young adults living with PaHIV and identify independent predictors of in‐hospital mortality.

**Methods**: A retrospective study was conducted at a public reference hospital in Argentina (January 2024–January 2026). Admissions of individuals aged ≥18 years living with PaHIV were included. Continuous variables were expressed as medians with interquartile ranges (IQR). Clinical markers were compared between survivors and non‐survivors using the Mann–Whitney U test. A multivariate logistic regression model assessed the predictive value of clinical complexity (number of concurrent diagnoses) and immunological status on mortality.

**Results**: A total of 103 hospitalizations among 52 individuals were analysed (Table 1). Median age was 26.0 years (IQR 24.0–28.0), 67% were cis‐gender men, 31% cis‐gender women and 2% trans‐gender women. At admission, patients presented with advanced HIV disease, showing a median CD4 count of 33.0 cells/µL (IQR 14.0–87.0) and a median hospital stay of 12.0 days (IQR 8.0–23.0). In‐hospital mortality was 19.23% (*n* = 10). Non‐survivors exhibited profound immunological depletion with a median CD4 count of 10.0 cells/µL (IQR 2.0–32.0), significantly lower than survivors (55.0 cells/µL, IQR 28.0–136.0; *p* = 0.0168) (Figure 1). Multivariate analysis revealed that clinical complexity was a potent independent predictor of mortality; each additional diagnosis at admission significantly increased the odds of a fatal outcome (OR: 3.81; 95% CI: 1.25–11.6; *p* = 0.013). Reasons for hospitalization were disseminated tuberculosis (*n* = 13; 25.0%), community‐acquired pneumonia (*n* = 11; 21.2%), acute diarrhoea (*n* = 7; 13.5%), candidiasis (*n* = 3; 5.8%), cryptococcosis (*n* = 3; 5.8%) and disseminated non‐tuberculous mycobacteria (NTM) (*n* = 3; 5.8%). NTM infections emerged as a critical marker of mortality risk (*p* = 0.0054).

**Conclusions**: In young adults living with PaHIV, clinical complexity at admission is a more robust predictor of in‐hospital mortality than isolated immunological markers. Multimorbidity and severe immunosuppression dictates immediate prognosis. These results highlight the urgent need for integrated, multidisciplinary care models that prioritize early screening for opportunistic conditions and support treatment continuity to prevent the progression of advanced HIV disease.

**References**

1. Haw NJL, Lesko CR, Ng DK, Lam JO, Gebo K, Sterling TR, Rabkin CS, Li J, Buchacz K, Agwu A, Althoff K; North American AIDS Cohort Collaboration on Research and Design (NA‐ACCORD). Incidence of AIDS‐defining conditions among adults with perinatally acquired HIV after transition to adult HIV care in the United States and Canada, 2000−2022. Clin Infect Dis. 2025 Dec 24;81(5):962–969. doi: 10.1093/cid/ciaf300. PMID: 40469005; PMCID: PMC12375960.

2. Berzosa Sánchez A, Jiménez De Ory S, Frick MA, Menasalvas Ruiz AI, Couceiro JA, Mellado MJ, Bisbal O, Albendin Iglesias H, Montero M, Roca C, Samperiz G, Cervero M, Miralles C, Fortuny Guash C, Carrasco I, Navarro ML, Ramos Amador JT; Corispe‐Faro Cohort Working Group, Spain. Mortality in perinatally HIV‐infected adolescents after transition to adult care in Spain. Pediatr Infect Dis J. 2021 Apr 1;40(4):347–350. doi: 10.1097/INF.0000000000003009. PMID: 33264211.

**FIGURE 1:**
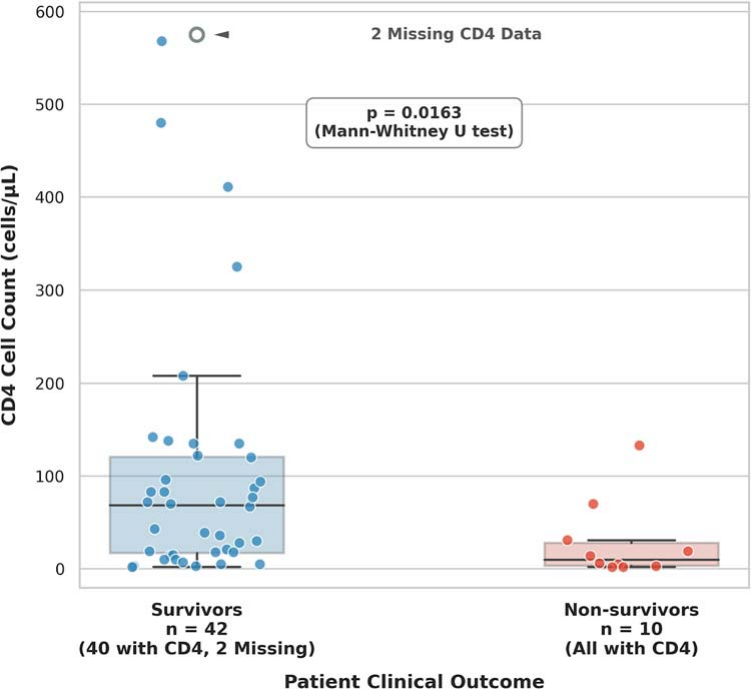
P224 | Distribution of CD4 counts by clinical outcome.

**TABLE 1:**
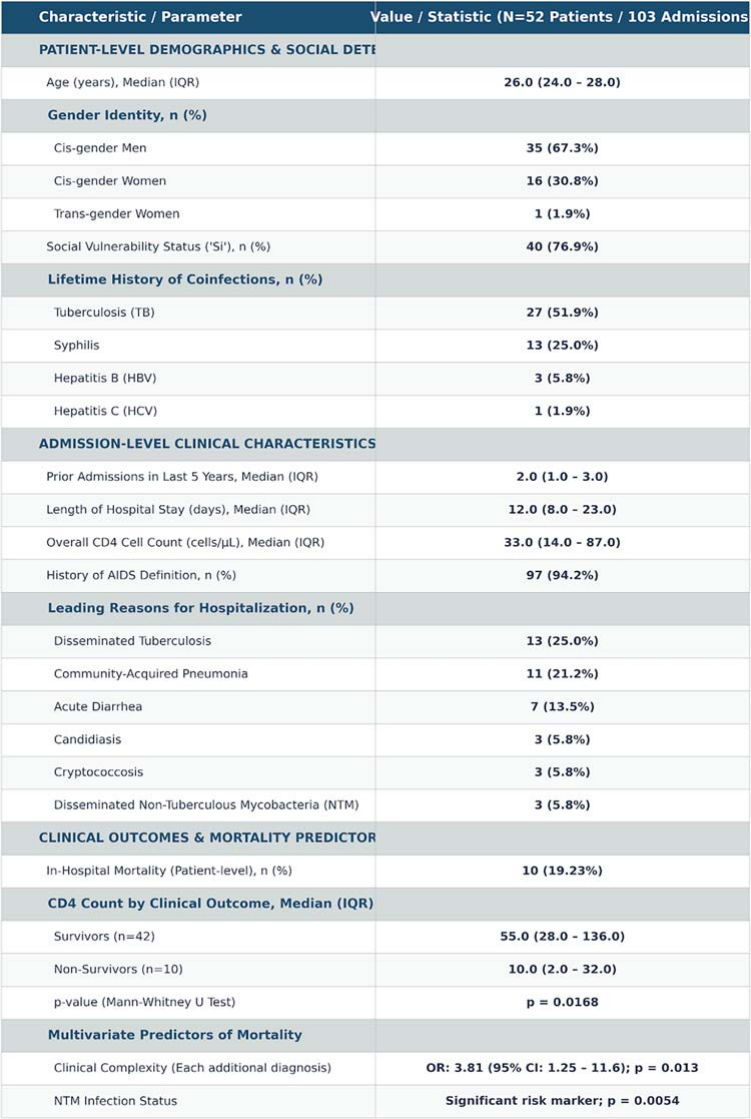
P224 | Clinical characteristics, social determinants and outcomes of young adults with PaHIV.

### P225 Risk of Not Achieving Virological Suppression by Week 48 in People With HIV Starting First‐Line Therapy: Differences by Calendar Period (2016–2025) and Contributing Factors

Manuela Colafigli ^1^, Giuseppe Lapadula^2^, Franco Maggiolo^3^, Samuel Lazzarin^4^, Lucia Taramasso^5^, Giulia Micheli^6^, Simona Di Giambenedetto^7^, Francesca Ceccherini‐Silberstein^8^, Antonella Castagna^9^, Sergio Lo Caputo^10^, Valentina Mazzotta^6^ Andrea Antinori^6^, Cristina Mussini^11^, Alessandro Cozzi‐Lepri^12^, on behalf of ICONA Cohort Study Group

^1^SC Malattie Infettive Ospedale Regionale U Parini, Aosta, Italy; ^2^Department of Infectious Diseases, IRCCS San Gerardo dei Tintori, University of Milano‐Bicocca, Monza, Italy; ^3^Infectious Diseases’ Specialist Freelance, Fabro, Italy; ^4^Department of Infectious Diseases, Luigi Sacco University Hospital, ASST Fatebenefratelli Sacco, Milano, Italy; ^5^Infectious Disease Clinic, IRCCS Ospedale Policlinico San Martino, Genova, Italy; ^6^Clinical and Research Infectious Diseases Department, National Institute for Infectious Diseases Lazzaro Spallanzani‐IRCCS, Roma, Italy; ^7^UOC Malattie Infettive, Fondazione Policlinico Universitario Agostino Gemelli IRCCS, Dipartimento di Sicurezza e Bioetica, Università Cattolica del Sacro Cuore, Roma, Italy; ^8^Department of Experimental Medicine, University of Rome ‘Tor Vergata’, Roma, Italy; ^9^Department of Infectious Diseases, IRCCS San Raffaele Scientific Institute, Vita‐Salute San Raffaele University, Milano, Italy; ^10^Clinic of Infectious Diseases, Department of Clinical and Surgical Sciences, University of Foggia, Foggia, Italy; ^11^Infectious Diseases Clinic, University of Modena and Reggio Emilia, Modena, Italy; ^12^Centre for Clinical Research, Epidemiology, Modelling and Evaluation (CREME), Institute for Global Health, UCL, London, UK

**Background**: Despite advances in combination antiretroviral therapy (cART), some people with HIV (PWH) never achieve virological suppression (VS ≤50 copies/mL) by week 48 (wk48 VNS). It remains unclear whether current virological non suppression (VNS) rates differ from those in the past and how many patients fail to suppress under routine care.

**Methods**: We conducted an observational study including cART‐naïve PWH aged ≥18 years from the ICONA Foundation Study cohort who initiated first‐line cART after 2016 and had ≥48 weeks of complete viral load (VL) follow‐up. The incidence of wk48 VNS was estimated overall and stratified by calendar year categories (CYCs): 2017–2019, 2020–2022 and 2023–2025. Advanced HIV disease (AHD) was defined as CD4 ≤200 cells/mm^3^ or AIDS diagnosis at cART initiation (baseline). Baseline differences across CYCs were assessed using chi‐square and Kruskal–Wallis tests. We used Kaplan–Meier methods to estimate the probability of achieving VS after wk48. A counterfactual mediation analysis explored mechanisms linking CYCs and VNS risk.

**Results**: The study included 2641 PWH (1159 2017–2019, 820 2020–2022, 662 2023–2025). Overall, 17% were female, 50% MSM, with a median age of 41 years (IQR 32–51); 26% had baseline VL > 500,000 copies/mL, 38% had AHD and 85% initiated INSTI‐based regimens. More recent CYCs was associated with higher frequency of AHD and higher baseline VL (Table 1). VNS incidence slightly increased over time from 6.0% (2017–2019) and 6.3% (2020–2022) to 8.1% (2023–2025) for an unadjusted odds ratio of 1.37 (95% CI: 0.95–1.98, *p* = 0.10) for 2023–2025 versus 2017–2019. Most of the association was mediated by baseline VL (which explained 58% of effect), while AHD contributed modestly (13%) and regimen type had minimal impact (Figure 1). Among wk48VNS, 66% (95% CI: 58–73%) reached suppression within the following year, yielding an overall 2‐year VS rate > 98%.

**Conclusions**: Among PWH who start cART and reach wk48 follow‐up, the proportion with VNS has remained stable in recent years, with < 7% never achieving VS by wk48 and < 2% by wk96. The slight increase observed in wk48 VNS risk appears to be driven primarily by higher baseline VL, rather than by late presentation or treatment regimens used.

**FIGURE 1:**
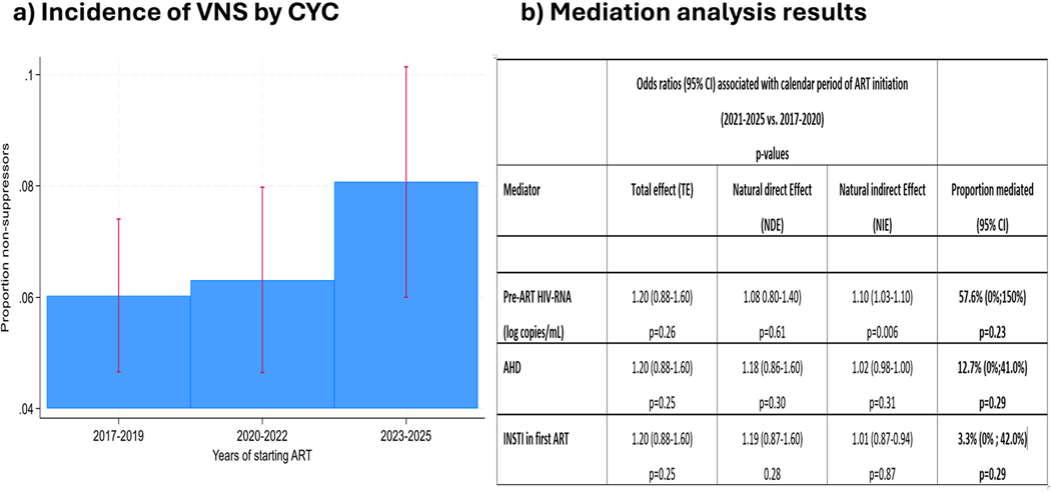
P225 | Main outcomes: (a) Crude incidence of virological non‐suppression (VNS) by calendar year categories (CYC); (b) results of the mediation analysis.

**TABLE 1:**
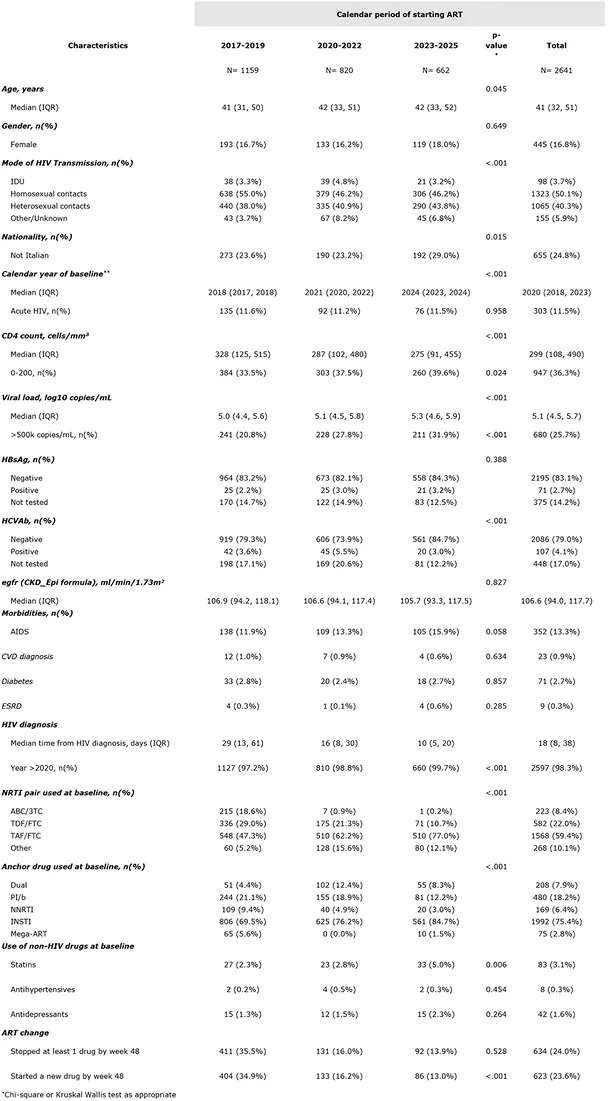
P225 | Patients’ characteristics, whole study population (right) and by calendar year categories (left).

### P226 Determinants of Suboptimal Immune Recovery Despite Viral Suppression Among People Living With HIV: The ACTHIV‐IST Cohort of Advanced Presenters

Ahsen Oncul ^1^, Okan Derin^1,2^, Sibel Yıldız Kaya^3^, Alper Gündüz^4^, İnci Yılmaz Nakir^5^, Bilgul Mete^3^, Özlem Altuntaş Aydın^4^, Hindirin Takak^5^, Ahmet Doğukan Bayrak^1^, Meliha Meriç Koç^4^, Esra Zerdali^5^, Dilek Yildiz Sevgi^1^, Fehmi Tabak^3^

^1^Department of Infectious Diseases and Clinical Microbiology, Health Sciences University Sisli Hamidiye Etfal Training and Research Hospital, Istanbul, Türkiye; ^2^Epidemiology PhD Program, Graduate School of Health Sciences, Istanbul Medipol University, Istanbul, Türkiye; ^3^Faculty of Medicine, Department of Infectious Diseases and Clinical Microbiology, Istanbul University Cerrahpaşa, Istanbul, Türkiye; ^4^Department of Infectious Diseases and Clinical Microbiology, Basaksehir Cam Sakura City Hospital, Istanbul, Türkiye; ^5^Department of Infectious Diseases and Clinical Microbiology, Health Sciences University İstanbul Haseki Training and Research Hospital, Istanbul, Türkiye

**Background**: Although antiretroviral therapy (ART) successfully suppresses the virus in most people living with HIV (PLWH), a substantial proportion fail to achieve adequate immune recovery. We investigated the frequency and determinants of suboptimal immune recovery (SOR) among advanced presenters in a multicentre cohort.

**Methods**: We conducted a retrospective cohort study across four ACTHIV‐IST treatment centres in Istanbul, Türkiye. Adults initiating ART between 2016 and 2024 with a baseline CD4 count <200 cells/µL, available CD4 measurements at baseline, 6 months and 24 months and HIV‐RNA ≤200 copies/mL at 24 months were included. The primary outcome was SOR, defined as a CD4 count ≤350 cells/µL at 24 months despite virological suppression. Categorical variables and continuous variables were compared using appropriate tests, based on the normality of data distribution. A *p*‐value of < 0.05 was considered statistically significant. Univariable and multivariable logistic regression was used to identify independent predictors. Model discrimination and calibration were assessed using the area under the receiver operating characteristic curve (AUC), Brier score and Hosmer–Lemeshow test. All analyses were performed using R.

**Results**: Among 350 eligible participants, median age at ART initiation was 40 years and 91% were male. SOR occurred in 124 individuals, 35.4% (95% CI: 30.4–40.7). Older age at ART initiation independently increased the odds of suboptimal recovery (adjusted odds ratio [aOR] 1.04 per year; 95% CI: 1.01–1.08; *p* = 0.025, Table 1). Higher baseline CD4 counts were strongly protective compared with baseline CD4 0–50 cells/µL (100–200 cells/µL: aOR 0.21, 95% CI: 0.08–0.53; *p* = 0.001, Figure 1). Greater early immune recovery, measured as the 6‐month CD4 increase, was independently associated with a lower risk of SOR (*p* < 0.001). Baseline HIV‐RNA level, comorbidities, coinfections and ART regimen were not significant predictors (Table 1). The prediction model demonstrated good discrimination (AUC = 0.83).

**Conclusions**: More than one‐third of virologically suppressed advanced presenters failed to achieve a CD4 count > 350 cells/µL after 24 months of ART. Older age, severe baseline immunosuppression and poor early CD4 recovery were the major determinants of suboptimal immune restoration. These findings highlight the importance of earlier HIV diagnosis and prompt ART initiation and suggest that early CD4 recovery may help identify individuals requiring closer clinical monitoring.

**FIGURE 1:**
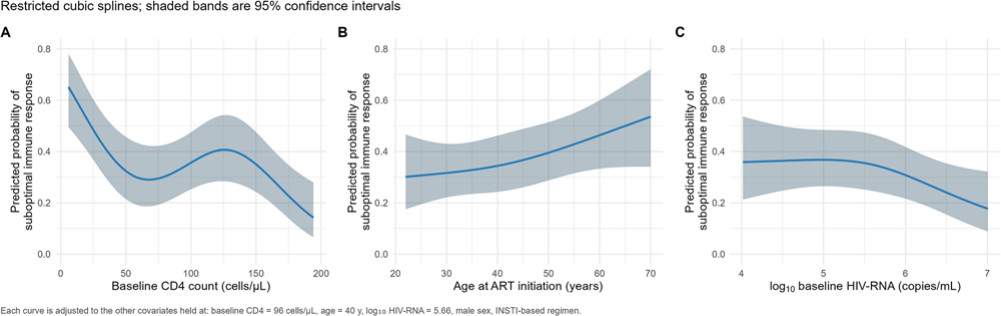
P226 | Adjusted nonlinear associations with suboptimal immune response.

**TABLE 1:**
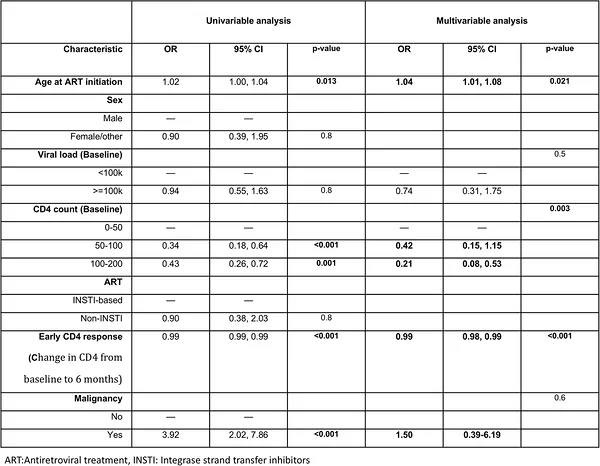
P226 | Factors associated with suboptimal immune recovery (SOR).

### P227 Routine Lumbar Puncture for Asymptomatic Cryptococcal Antigenemia in People Living With HIV: Should We Be More Selective?

Daniel Toscano, Inês Reis Luís, Patrícia Pacheco, Rita Patrocínio Jesus

Infectious diseases, Hospital Prof. Dr. Fernando Fonseca, Lisbon, Portugal

**Background**: International guidelines recommend routine screening of serum cryptococcal antigen (sCrAg) in PLWHIV with CD4<100 cells/µL [1] and some recommend additional screening in those with CD4 100–200 cells/µL [2, 3]. In sCrAg‐positive individuals, even in the absence of neurological symptoms, cerebrospinal fluid (CSF) evaluation is recommended whenever resources allow, to exclude central nervous system (CNS) cryptococcal infection and identify those who should be given pre‐emptive treatment. We aimed to determine the proportion of CNS cryptococcal infections among PLWHIV without neurological symptoms or signs (namely headache and focal neurological deficits) undergoing lumbar puncture (LP) following a positive sCrAg result.

**Methods**: We conducted a retrospective observational cohort including all asymptomatic adult PLWHIV with a positive sCrAg screening who underwent LP as part of routine evaluation in our institution between 2016 and 2025. Demographic, immunological and microbiological data were collected. We evaluated CSF cryptococcal antigen (CrAg), microbiological cultures, polymerase chain reaction (PCR) and cytochemistry. We assessed the proportion of asymptomatic CNS cryptococcal infections (defined by a positive CrAg, microbiology or PCR) and the use of pre‐emptive therapy.

**Results**: Forty‐one PLWHIV were included. Mean age was 47 ± 12 years, 61% were male and 46% originated from sub‐Saharan Africa. Median CD4 cell count was 48 (interquartile range 22–106) cells/µL, with 71% having CD4 < 100 cells/µL. Serum CrAg titres were generally low, with 85% presenting titres ≤1/40 (Table 1). No CSF findings compatible with CNS cryptococcal infection were identified. Pre‐emptive fluconazole was initiated in 18 (44%) patients. Among the remaining untreated patients, fluconazole was withheld due to drug toxicity concerns, since they were receiving therapy for other opportunistic infections.

**Conclusions**: In this cohort of PLWHIV with asymptomatic CrAg antigenemia with low CrAg titres, no cases of CNS cryptococcal infections were found. Considering LP is an invasive procedure associated with patient discomfort, resource use and potential complications, the findings suggest that routine LP in patients with low sCrAg titres may not be warranted in all patients, even in the context of well‐resourced settings.

**References**

1. EACS Guidelines version 13.0, October 2025.

2. World Health Organization. Guidelines for diagnosing, preventing and managing cryptococcal disease among adults, adolescents and children living with HIV. Geneva: World Health Organization; 2022. Licence: CC BY‐NC‐SA 3.0 IGO.

3. Panel on Guidelines for the Prevention and Treatment of Opportunistic Infections in Adults and Adolescents With HIV. Guidelines for the prevention and treatment of opportunistic infections in adults and adolescents with HIV. National Institutes of Health, HIV Medicine Association and Infectious Diseases Society of America. Available at NIH Clinical Info. Accessed October 29, 2024.

**TABLE 1:**
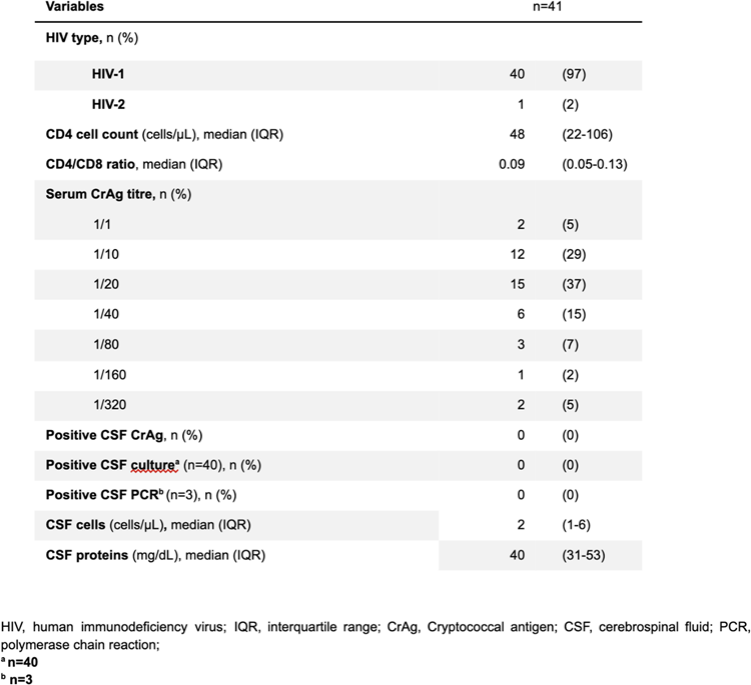
P227 | Immunological and microbiological data.

### P228 New HIV Diagnoses at a Portuguese Tertiary Care Centre: Does Migrant Status Impact Late Presentation and 1‐Year Outcomes?

Ana Jorge Martins, Rúben Carvalho, Joana Marinho, Tiago Neves, Luísa Cristina, Filipe Medeiros, Cristina Valente

Infectious Diseases Department, Coimbra, Portugal

**Background**: Given Portugal's high prevalence of late HIV diagnosis [1] and a migrant population that more than doubled between 2021 and 2025 [2], foreign residents may face barriers to early diagnosis. This study evaluates sociodemographic and clinical factors associated with late presentation (LP) and assesses potential health disparities between migrant and native‐born HIV patients at diagnosis and after 1 year of follow‐up.

**Methods**: We conducted a retrospective longitudinal observational study of individuals newly diagnosed with HIV attending their first outpatient consultation (2023–2025) at a Portuguese tertiary hospital. Demographic, epidemiological, clinical, laboratory and treatment‐related data were collected from medical records at diagnosis and at 1 year of follow‐up. LP was defined as TCD4+ count ≤350 cells/mm^3^ or an AIDS‐defining illness. Univariate comparisons utilized Pearson's chi‐square and independent‐samples *t*‐tests via SPSS (*p* < 0.05).

**Results**: Among 239 patients (71.5% male; mean age 44.2 years; 41.4% migrants), heterosexual contact was the main transmission route (66.9%). Mean baseline CD4 count was 312 cells/mm^3^ and 20.1% presented in CDC stage C3. Overall, 61.1% (*n* = 146) were late presenters. Age greater than 50 years was significantly associated with LP compared to younger patients (78.2% vs. 52.8%; *p* <0.001). LP trended higher in heterosexuals (*p* = 0.066), with no significant differences by sex (*p* = 0.874) or migrant status (*p* = 0.691). Hospitalization at diagnosis was required for 26.4%, with no significant difference in mean stay between migrants and non‐migrants (38.8 vs. 30.4 days; *p* = 0.244). At 1 year of follow‐up, retention in care at this centre was significantly higher for native‐born individuals (97.6% vs. 88.2%; *p* = 0.006). Among retained patients, 94.7% had HIV‐RNA < 200 copies/mL, 76.7% achieved viral suppression (<50 copies/mL) and mean TCD4+ increased by 229.8 cells/mm^3^.

**Conclusions**: Advanced age (greater than 50 years) was the primary predictor of LP, highlighting the need for targeted screening in older populations. Despite high overall late diagnosis rates, the absence of significant disparities in clinical presentation and hospitalization length between migrants and native‐born patients suggests equitable initial healthcare access, yielding comparable immunological and virological outcomes at 1 year of follow‐up.

**References**

1. Portugal. Ministério da Saúde. Direção‐Geral da Saúde/Instituto Nacional deSaúde Doutor Ricardo Jorge. Infeção por VIH em Portugal – 2025. Lisboa: DGS/INSA; 2025.

2. Instituto Nacional de Estatística. Estimativas de população residente. Available at: https://www.ine.pt/xportal/xmain?xpid=INE&xpgid=ine_destaques&DESTAQUESdest_boui=770295679&DESTAQUESmodo=2. Accessed 26/06/2026.

### P229 Dual Drug Regimens Simplification for Late Presenters: Real‐Life Data From 2016 to 2025 at Legnano Hospital, Italy

Maria Paola Bissanti^1^, Dario Bernacchia ^2^, Gabriele Pagani^2^, Maurizio Mena^2^, Michela Rigoni^1^, Marianna Ticozzelli^1^, Andrea Mulekela Tshishimbi^1^, Stefano Rusconi^1,2^

^1^University of Milan, Milan, Italy; ^2^Infectious Disease Unit, ASST Ovest MIlanese, Legnano, Italy

**Background**: HIV late diagnosis represents a main challenge in HIV management in Italy. Late diagnosis means more morbidity, mortality, AIDS‐related events. Dual drug regimens (2DR) and dual injectable regimens are the future of antiretroviral therapy (ART) in both naïve/experienced people with HIV (PWH) and represent the last achievement in ART management. Nowadays, ART simplification is even more tailored on PWH characteristics and personal approaches.

**Methods**: New HIV diagnosis from January 2016 and December 2025 at Legnano Hospital were collected. Late presenters (LPs, < 350 lymphocytes CD4+/mmc at baseline) and no‐late presenters (NO‐LPs) features were compared through a descriptive analysis: demographics, therapies, follow up and virological status. A Cox regression analysis was used to assess the timing of simplification on the two main 2DR available in Italy, dolutegravir/lamivudine (DTG/3TC) and cabotegravir/rilpivirine (CAB+RPV).

**Results**: Currently, 1194 PWH are actively followed up at Legnano Hospital. From 2016 to 2025, 2129 HIV tests were made and only 134 were confirmed as new diagnosis. At baseline, LPs (69.4%) had a median CD4+ count of 90 cells/mmc and median viral load of 237,000 copies/mL. Age at diagnosis was higher in LPs group (47 vs. 35 years, *p* < 0.001) with a prevalence of male sex (70%). Our analysis showed a delay in ART introduction in LPs versus NO‐LPs (7 vs. 11.5 days, *p* < 0.001). At the last follow up, there was no difference in ART efficacy in terms of viral load (both groups undetectable copies/mL, *p* = 0.524), but lower CD4+ levels in LPs (485 vs. 915 cells/mmc, *p* < 0.001) (Table 1). Finally, the Cox regression analysis underlined a significant delay (*p* < 0.023) in ART simplification in LPs to DTG/3TC or CAB+RPV (Figure 1).

**Conclusions**: The optimal timing for ART simplification in LPs is yet to be standardized [1, 2]. DTG/3TC and CAB+RPV represent some of the best ART simplifications to mitigate toxicities and pill burden, while improving quality of life [3, 4]. However, real‐world evidence highlights concerns regarding early switch strategies by physicians caring for PWH.

**References**

1. Rossetti B, Baldin G, Sterrantino G. Efficacy and safety of dolutegravir‐based regimens in advanced HIV‐infected naïve patients: Results from a multicenter cohort study. Antiviral Res. 2019;169:104552.

2. Mondi A, Cozzi‐Lepri A, Tavelli A. Persistent poor clinical outcomes of people living with HIV presenting with AIDS and late HIV diagnosis ‐ Results from the ICONA cohort in Italy, 2009–2022. Int J Infect Dis. 2024;142:106995.

3. Fabbiani M, Masini M, Rossetti B. Efficacy and durability of dolutegravir‐ or darunavir‐based regimens in ART‐naïve AIDS‐ or late‐presenting HIV‐infected patients. Viruses. 2023;15(5):1123.

4. Micheli G, Mondi A, Roen A. Did the COVID‐19 pandemic shift the landscape of late HIV diagnosis? Int J Infect Dis. 2026;165:108437.

**FIGURE 1:**
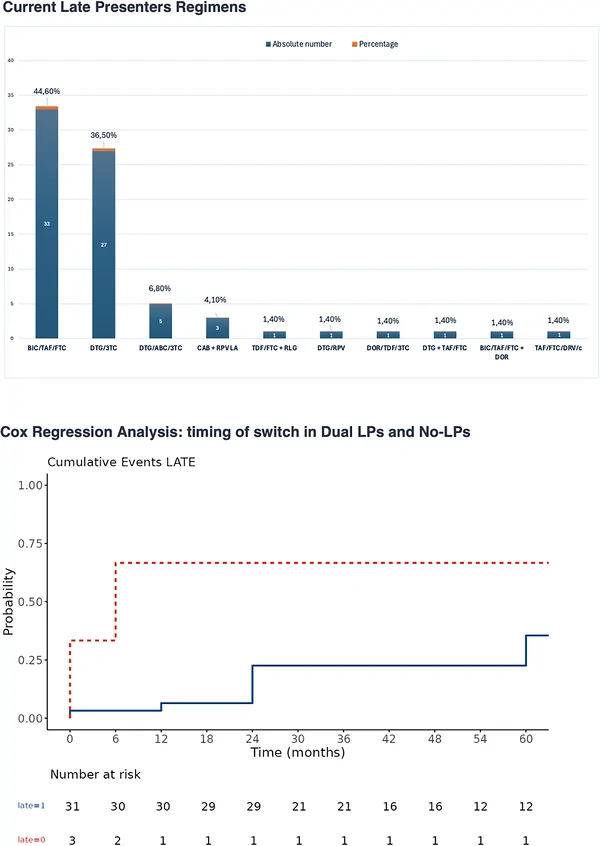
P229 | Current regimens of late presenters and Cox regression analysis.

**TABLE 1:**
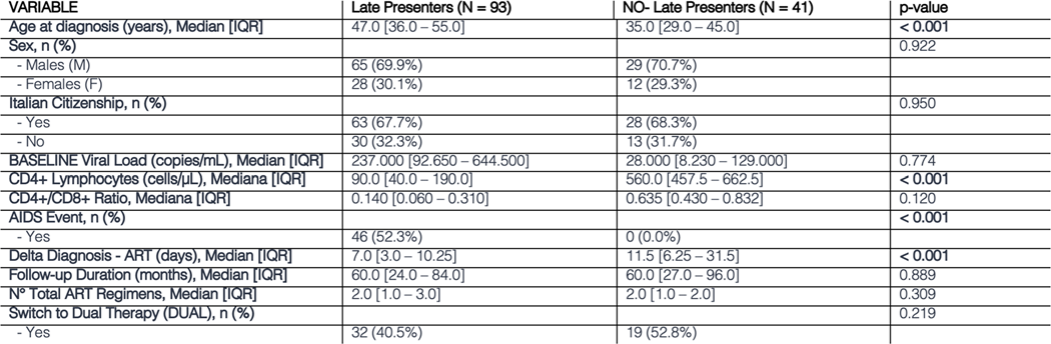
P229 | Demographics and clinical characteristics of the population.

### P230 Advanced HIV Disease Among Newly Diagnosed People Living With HIV in 39 Health Facilities in Enugu State, Nigeria, 2023–2025

Anuli Oyeka ^1^, Peter Chiagorom^1^, Ita Egharevba^1^, Elizabeth Marcus^1^, Ogechi Ezeobi^1^, Henrietta Ozobodo^1^, Chukwudi Okwor^1^, Fabian Bassey^1^, Ifeoma Tony‐Monye^1^, Eugene Onu^1^, Victor Enejoh^1^, Mabwe Kubwa^1^, Lawrencia Agur^1^, Dorcas Magbadelo^1^, Young Oluokun^1^, Amana Effiong^1^, John Oko^1^, Abaniwonnda Sulaiman^2^, Chukwudi Onwuchekwa^2^, Olufemi Oke^2^, Flora Nwagagbo^2^, Dennis Onotu^2^

^1^Catholic Caritas Foundation of Nigeria (CCFN), Abuja, Nigeria; ^2^Division of Global HIV and TB, US Centers for Disease Control and Prevention, Abuja, Nigeria

**Background**: Advanced HIV disease (AHD) is defined by the World Health Organization (WHO) as CD4 count < 200 cells/mm^3^ or WHO clinical stage 3 or 4 in adults and adolescents living with HIV, and all HIV‐positive children under 5 years of age. A substantial proportion of newly diagnosed people living with HIV continue to present with AHD at the time of treatment initiation, with increased morbidity and mortality, highlighting the need for early identification and management. This review assessed the prevalence and demographic correlates of AHD among newly diagnosed people living with HIV (PLWH) in Enugu State.

**Methods**: We used the Nigeria National Data Repository and reviewed data on newly identified PLHIV between October 2023 and September 2025 from 39 HIV treatment facilities that provide an AHD package of care in Enugu State. We disaggregated data by sex, age, CD4 test done, WHO stage and other diagnostic criteria for AHD among this cohort of PLHIV. We also examined laboratory investigations, including tuberculosis (TB) diagnosis using TB Lam, serum cryptococcal antigen screening (CrAg) and outcomes. Descriptive statistics were used to summarize demographics; chi‐squared tests were used to assess associations between demographic variables and AHD.

**Results**: Among 4720 newly diagnosed PLWH (1779 [38%] males and 2941 [62%] females), the mean age was 34 years (SD, 11.8). Of these, 4385 (93%) had a CD4 test done, 956 (22%) had CD4 < 200 cells/mm^3^ and 1076 (25%) had AHD. AHD prevalence was higher among males (25%) than females (21%). Among AHD cases, 276 (26%) had TB, of whom 258 (93%) initiated TB treatment. Furthermore, 14 (1.5%) were CrAg‐positive, (57%) underwent CSF CrAg testing and all 14 received fluconazole treatment (Figure 1). Male sex (*p*<0.05) and age ≥20 years (*p* < 0.001) were significantly associated with AHD.

**Conclusions**: One in four (25%) newly diagnosed PLWH in Enugu State presented with AHD, highlighting a substantial burden and potential care‐cascade gaps. Follow‐up at 3, 6 and 12 months after ART initiation is needed to assess outcomes and guide clinical and programmatic responses.

**FIGURE 1:**
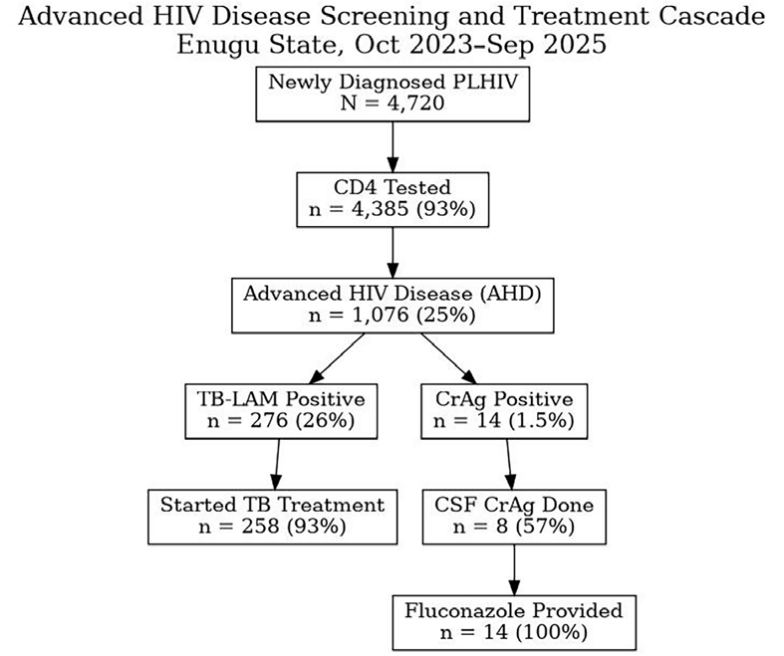
P230 | AHD cascade flow.

### P231 Clinical and Epidemiological Profile of People Newly Diagnosed With HIV in a Portuguese Infectious Diseases Department (2019–2025)

Mariana Azevedo, Estela Sousa, Natália Buruian, Carmela Piñeiro, Rosário Serrão, Lurdes Santos

Department of Infectious Diseases, São João University Hospital Centre, Porto, Portugal

**Background**: Rapid linkage to care and a comprehensive understanding of patient profiles are essential to curb the human immunodeficiency virus (HIV) epidemic and guide localized public health strategies. This study characterizes people newly diagnosed with HIV managed at our department over a 7‐year period.

**Methods**: We conducted a retrospective study of adults newly diagnosed with HIV between January 2019 and December 2025 at a Portuguese infectious diseases department. Demographic data, clinical presentation and care timelines were reviewed. Baseline CD4+ T‐cell counts and HIV RNA viral loads (VLs) were stratified according to EACS guidelines.

**Results**: A total of 527 patients were analysed. Mean age at diagnosis was 40 years (median 37; range 18–89); 78.56% (*n* = 414) were male and 5 were transgender women. Origin was primarily Portugal (64.71%, *n* = 341) and Brazil (20.11%, *n* = 106). Transmission routes included men who have sex with men (MSM) (47.44%, *n* = 250), heterosexual contact (39.85%, *n* = 210) and injecting drug use (3.99%, *n* = 21). Only 9 patients reported prior pre‐exposure prophylaxis (PrEP) use. At diagnosis, 69.45% (*n* = 366) were asymptomatic, while 12.14% (*n* = 64) had advanced disease. Notably, 53.89% were late presenters (CD4 <350 cells/mm^3^), including 31.69% with CD4 <200 cells/mm^3^. Baseline VL exceeded 100,000 copies/mL in 55.03% and 500,000 copies/mL in 23.34%. HIV‐1 predominated (98.29%, *n* = 518).

Median time from reactive test to first consultation was 14 days (mean 64; range 0–4603). Main referral sources were primary healthcare (27.32%), emergency departments and community organizations (14.23% each). Concurrent sexually transmitted infections (STIs) were frequent (*n* = 161 episodes): syphilis (68.9%), chlamydia (26.7%) and gonorrhoea (21.1%); 17.4% had ≥2 STIs. Active hepatitis B and C were identified in 11 and 14 people, respectively. AIDS‐defining events occurred in 56 patients (10.63%). Delays in antiretroviral therapy (ART) initiation (*n* = 98) were due to missed appointments, loss to follow‐up or declined ART initiation (21.4%), undocumented reasons (19.4%), delayed referral (12.2%), suspected/active tuberculosis (11.2%) or opportunistic infections (10.2%). Overall, 18.02% (*n* = 95) were lost to follow‐up or died.

**Conclusions**: Despite robust linkage to care and a short median interval to the first consultation, the high prevalence of late presenters and concurrent STIs remains alarming, underscoring a critical need to scale up targeted screening and early preventative strategies.

## Clinical Management Considerations: Transgender People

### P232 Integrating HIV Prevention, Same‐Day Antiretroviral Therapy Initiation, Primary Care and Gender‐Affirming Services to Support Engagement in Care Among Transgender People

Darya Fridman

The LGBTQ+ Center of Southern Nevada, Las Vegas, NV, US

**Background**: Transgender people experience substantial barriers to HIV prevention, diagnosis and sustained engagement in care. Integrating HIV prevention and treatment with gender‐affirming healthcare, primary care and multidisciplinary wrap‐around services may improve access and retention. We evaluated an integrated LGBTQ+‐centred model of care supporting engagement across the HIV prevention and treatment continuum.

**Methods**: A retrospective review of electronic medical records was conducted for transgender people receiving care in 2025. Demographic characteristics, clinic utilization, HIV testing, pre‐exposure prophylaxis (PrEP), HIV care, gender‐affirming hormone therapy (HRT), immediate antiretroviral therapy (ART) initiation and wrap‐around service utilization were evaluated.

**Results**: A total of 821 transgender people received care, including 768 (93.5%) receiving HRT. The cohort was predominantly young, with 456 (56%) aged ≤29 years. Age distribution was: 114 (14%) aged ≤19 years, 342 (42%) aged 20–29 years, 218 (27%) aged 30–39 years, 93 (11%) aged 40–49 years, 37 (5%) aged 50–59 years, 16 (2%) aged 60–69 years and 1 (<1%) aged ≥70 years. 701 individuals attended primary care, 219 attended sexually transmitted infection (STI) services and 99 attended both, accounting for 3540 clinical encounters (Table 1). Among those receiving HRT, 41 were people living with HIV, including 36 receiving integrated HIV and HRT care at the clinic. HIV testing was performed for 252 individuals, identifying 10 positive HIV test results, of which 7 represented new diagnoses. All newly diagnosed individuals initiated same‐day ART, with a median time from diagnosis to first HIV provider visit of 0 days. Overall, 16 transgender people initiated HIV care during the study period, including seven new diagnoses, five re‐engaging in care and four transferring care. Among HIV‐negative individuals, 63 initiated pre‐exposure prophylaxis (PrEP) (Figure 1). All participants completed a comprehensive social assessment and were offered individualized wrap‐around services including behavioural health referrals, transportation assistance, insurance navigation, legal support, food support, gender‐affirming clothing resources and peer support.

**Conclusions**: An integrated LGBTQ+‐centred model combining HIV prevention, same‐day ART initiation, PrEP, HIV care, primary care and gender‐affirming healthcare supported rapid linkage, re‐engagement and expansion of HIV prevention and treatment services. Integration of gender‐affirming and HIV services within community settings may reduce barriers and strengthen engagement across the HIV care continuum.

**FIGURE 1:**
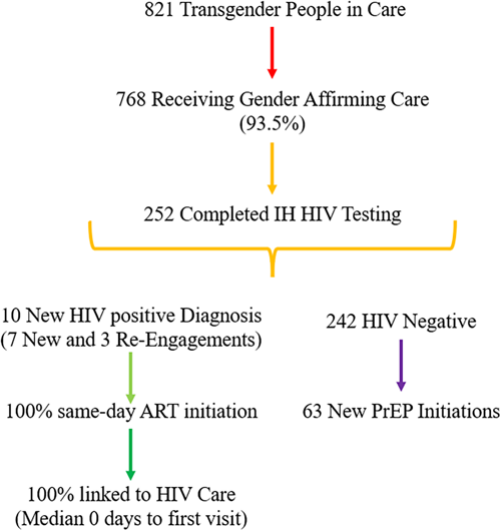
P232 | Transgender patient pathways into care: 2025 clinic data.

**TABLE 1:**
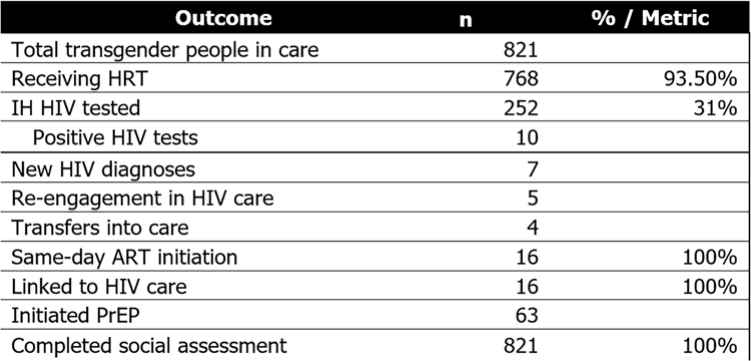
P232 | HIV prevention, treatment and gender‐affirming service utilization among transgender people.

### P233 Impact of Advanced HIV Disease on Mortality Among Transgender Women: A 13‐Year Cohort Study in Buenos Aires, Argentina

Mauro Bertolini, José Ángel Barletta, María José Rolón

Infectious Diseases Department, Hospital de Agudos Dr. Juan A. Fernández, Buenos Aires, Argentina

**Background**: Transgender women (TGW) in Argentina face a 20% HIV prevalence (vs. 0.4% in the general population). While late diagnosis rates in TGW are lower than in other groups, HIV‐related events remain a leading cause of mortality. We aimed to describe all‐cause mortality and clinical outcomes in a cohort of TGW with HIV linked to care (2011–2024) in Buenos Aires.

**Methods**: Retrospective cohort of adult TGW living with HIV. Primary exposure was advanced HIV disease (AHD) at enrolment (WHO definition). Baseline characteristics were compared using chi‐squared, Fisher's exact and Mann–Whitney U tests. Vital status was verified via national registries. Mortality was analysed using Poisson, Kaplan–Meier and multivariable Cox models (adjusting for age/splines, HCV, sex work and era of linkage). Multiple imputation by chained equations (MICE) addressed missing data. Statistical significance was set at *p* < 0.05.

**Results**: Among 238 TGW (median age 31; IQR 26–35; Table 1), 42% had AHD. The AHD group had higher prevalence of HCV (8% vs. 0.7%; *p* = 0.004), while active sex work was more frequent in those without AHD (72.5% vs. 55%; *p* = 0.042). Time to linkage was similar. Median follow‐up was 5.3 years (IQR 2.9–7.3). During follow‐up, 32 deaths occurred (69% AIDS‐related), corresponding to a rate of 25.3 (95% CI: 17.3–35.8)/1000PY. Mortality was significantly higher in the AHD group compared to those without (56.9 [37.2–83.4] vs. 7.4 [2.7–16.2]/1000PY; *p* < 0.001; Figure 1). Median age at death was 33 years (IQR 29–35.5), with no significant difference between groups. In the multivariable Cox model, AHD remained the strongest independent predictor of mortality (aHR 6.90; 95% CI: 2.70–17.65; *p* < 0.001).

**Conclusions**: Predominant AIDS‐related deaths at a median age of 33 underscore care cascade failures. The seven‐fold mortality risk with AHD highlights late clinical presentation as a fatal barrier, regardless of linkage time, likely reflecting care fragmentation and re‐entry gaps. Future comparative research is warranted to further address these structural inequities. Public health interventions must shift towards community‐led strategies that ensure earlier diagnosis and long‐term retention to prevent progression to advanced disease. Comprehensive differentiated care models are essential to eliminate preventable deaths and ensure survival equity for TGW.

**FIGURE 1:**
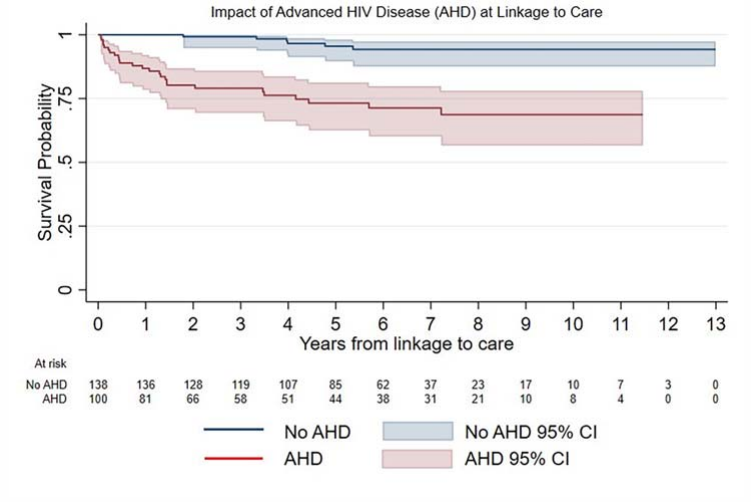
P233 | Survival probability in transgender women living with HIV (*N* = 238).

**TABLE 1:**
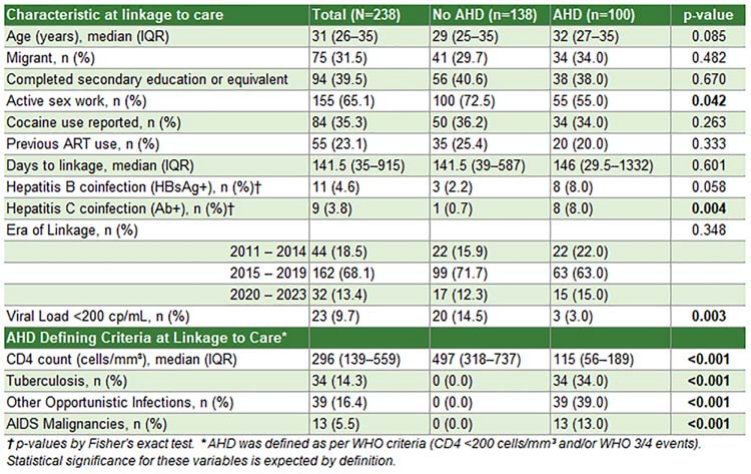
P233 | Baseline characteristics at linkage to care (*N* = 238).

### P234 Cabotegravir Rilpivirine LA Treatment for Transgender Women Living With HIV

Valentina Isernia ^1^, Marc Digumber^1^, Elisabet Costa I Otal^1^, Florence Michard^1^, Gerard Castanedo^1^, Antoine Bachelard^1^, Charlotte Charpentier^2,3^, Gilles Peytavin^3,4^, Jade Ghosn^1,3^

^1^Infectious Diseases Department, Bichat Hospital, Paris, France; ^2^Department of Virology, Bichat Hospital, Paris, France; ^3^Université Paris Cité, INSERM UMR 1137 IAME, Paris, France; ^4^Department of Pharmacology, Bichat Hospital, Paris, France

**Background**: Transgender women (TW) living with HIV present specific challenges in antiretroviral therapy (ART) management. Among TW originating from South America, the practice of gluteal silicone injections is highly prevalent. This may complicate the administration of gluteal intramuscular long‐acting injectable antiretroviral (LAI‐ART) therapies such as cabotegravir/rilpivirine (CAB/RPV). Limited data are available on LAI‐ART in this population [1, 2].

**Methods**: We conducted a retrospective observational study describing the epidemiological, clinical and pharmacological characteristics of TW who initiated CAB/RPV between September 2022 and January 2026 at the Department of Infectious Diseases, Bichat Hospital, in Paris. The drug levels of both medications were assessed during the oral phase in those who received oral lead‐in (OLI) and at weeks 4 and 8 after the initiation of injections.

**Results**: Between September 2022 and January 2026, 25 transgender women (TW) initiated CAB/RPV. Median age was 42 years (IQR 30–65) and 76% originated from Latin America. Fourteen (56%) were receiving gender‐affirming hormone therapy, while 10/25 (40%) had gluteal silicone fillers or prostheses. Baseline characteristics are summarized in Table 1. All TW had suppressed HIV viral loads at the time of switching to CAB/RPV. Among TW with gluteal silicone fillers or prostheses (10/25), 7/10 TW received CAB/RPV injections in the deltoid muscle, reporting injection‐site pain in two cases. One TW received CAB/RPV injections in the vastus lateralis muscle and did not report any adverse events. Among the 15/25 participants without gluteal fillers or prostheses, 12/15 received standard gluteal injections: one reported injection‐site pain and one reported a psychiatric adverse event after injection. For all sites, no case of injection‐site pain required treatment discontinuation. A total of five TW were still in OLI at the time of analysis. Pharmacological data were available at the end of OLI (10/25 TW) at W4 (22/25 TW) and W8 (23/25 TW) after CAB/RPV initiation. Drug concentrations remained within the target ranges for all injections sites (Table 2). HIV plasma viral load remained suppressed for all TW during the study period.

**Conclusions**: This real‐world study shows maintained virological suppression and sustained CAB/RPV exposure in TW living with HIV, supporting LAI‐ART feasibility using alternative intramuscular injection sites.

**References**

1. Cossu MV, D'Avolio A, Gervasoni C, Giacomelli A, Cattaneo D, Moschese D. Switching to deltoid intramuscular injections maintains therapeutic trough concentrations of rilpivirine and cabotegravir in people with HIV. Antimicrob Agents Chemother. 2024;68(5):e00175‐24.

2. Pannecoucke C, Valin N, Peytavin G, Lambert S, Morand‐Joubert L, Fofana D, et al. Safety and efficacy of deltoid intramuscular long‐acting cabotegravir and rilpivirine: A case series. Antiviral Ther. 2026;31(3):1–7.

**TABLE 1:**
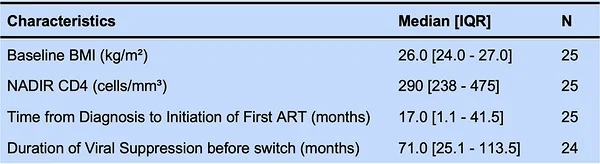
P234 | Baseline characteristics at initiation of injectable therapy.

**TABLE 2:**
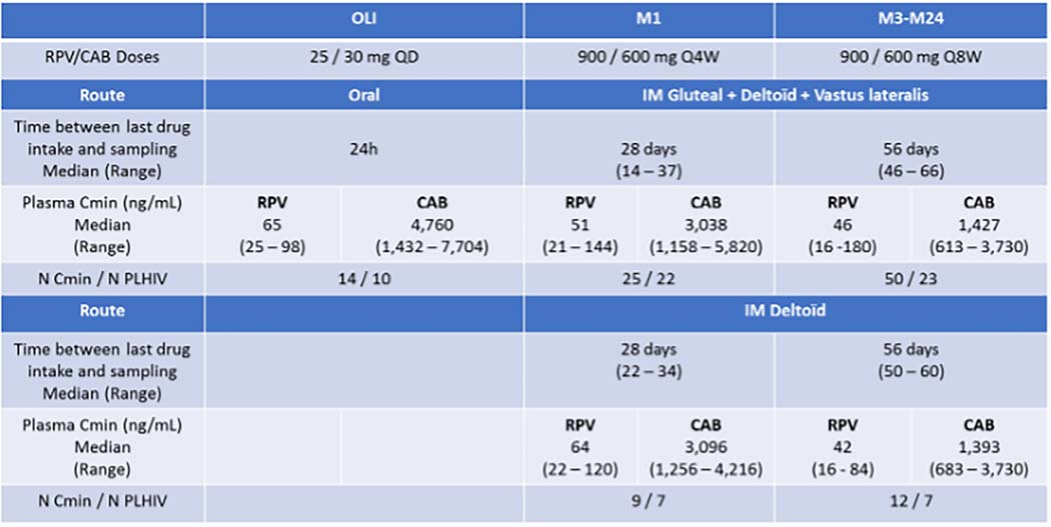
P234 | Drug concentrations of cabotegravir and rilpivirine during oral lead‐in and after intramuscular injections (gluteal, deltoid and vastus lateralis).

### P235 Impact of Antiretroviral Therapy on Virologic Failure and Loss to Follow‐Up Among Transgender Women and Travestis in São Paulo, Brazil: Data From the TransBordar Study

Maria Felipe Faustino de Medeiros, Victória Spínola Duarte de Oliveira, Camila Albuquerque de Moraes, Cristiane Pereira, Camilla Ferreira Santos, Cinthia Dellaqua, Giovanna Haimowski, Josiane Vitor de Souza Oliveira, Dyemison Pinheiro da Silva, Ricardo Augusto De Braga Castro, Priscilla Faria Pereira, Natalia Mercedes Cabral Amdi, Luciene Arrifano da Silva, Mylva Fonsi, Roberta Schiavon Nogueira, José Valdez Ramalho Madruga, Vinicius De Paula dos Anjos, Ana Caroline Coutinho Iglessias, on behalf of TransBordar Study Group

Casa da Pesquisa – Centro de Referência e Treinamento DST AIDS São Paulo, Unidade de Pesquisa, São Paulo, Brazil

**Background**: Antiretroviral therapy (ART) regimens significantly impact HIV care continuum, particularly regarding virologic failure (VF) and loss to follow‐up (LFU). Despite structural transphobia and high social vulnerability, we aim to evaluate how specific antiretroviral (ARV) use may influence these outcomes.

**Methods**: This retrospective cohort study included transgender women receiving longitudinal HIV care between 2019 and 2024 at a specialized referral centre in São Paulo. Data were abstracted from medical records and from the Brazilian national logistic and laboratory monitoring systems and subsequently entered into the REDCap platform. The statistical analysis was performed on Python v3.13.7 (statsmodels), performing logistic regression to calculate odds ratios (OR), assessing the influence of antiretroviral therapy on virologic failure and risks.

**Results**: The cohort included 299 eligible participants. The distribution of current ART is presented in Table 1. Overall, 12% (*n* = 36) of participants had current virologic failure (CVF), 32% (*n* = 97) had a history of previous virologic failure (PVF) and 44% (*n* = 131) experienced at least one LFU. The protease inhibitors class (PI) were associated with a higher risk of LFU (OR 2.06; 95% CI: 1.22–3.46; *p* < 0.05) and PVF (OR 4.97; 95% CI: 2.89–8.55; *p* < 0.05). Non‐nucleoside reverse transcriptase inhibitors (NNRTI) class use was also significantly related to PVF (OR 2.49; 95% CI: 1.51–4.11; *p* < 0.05). No statistically significant association was observed between ARVs from other classes. As shown in Figure 1, atazanavir (ATV) showed the strongest association with LFU, followed by ritonavir (RTV) and darunavir (DRV). The class of nucleoside/nucleotide reverse transcriptase inhibitors (NRTI), zidovudine (AZT) and estavudine (d4T) were associated with PVF, possibly related to side effects of this ARVs, as lipodystrophy and medullary toxicity and their use as monotherapy previous to combined antiretroviral therapy (cART). In the NNRTI class, efavirenz (EFZ) showed the strongest association with PVF. In the CVF analysis, no class or ARV reached statistical significance.

**Conclusions**: Specific ARVs were associated with PVF among transfeminine people in our cohort. The PVF rates are higher than CVF, possibly due to more tolerable ARVs options and to integrase inhibitor‐based regimens.

**FIGURE 1:**
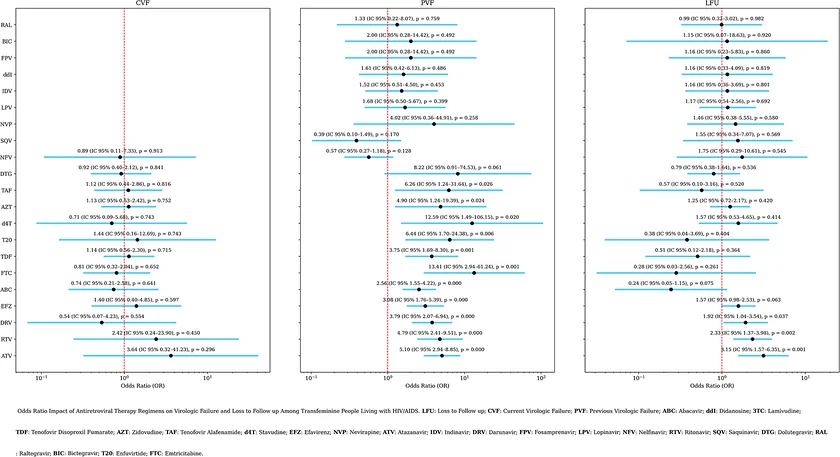
P235 | Odds ratio impact of antiretroviral therapy regimens on virologic failure and loss to follow‐up among transfeminine people living with HIV/AIDS.

**TABLE 1:**
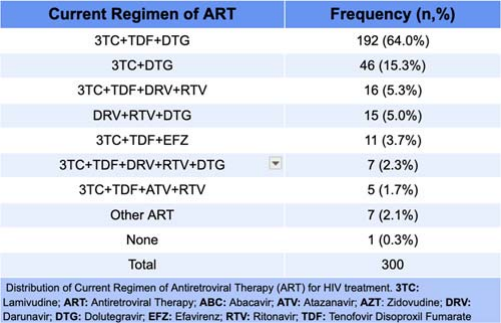
P235 | Distribution of current regimen of antiretroviral therapy (ART) for HIV treatment among transfeminine people living with HIV/AIDS in an STI reference service.

### P236 Sociodemographic Profile and Risk Factors for Loss to Follow‐Up and Virological Failure Among Transfeminine People Living With HIV: TransBordar—A Retrospective Study in São Paulo, Brazil

Maria Felipe Faustino de Medeiros, Victória Spínola Duarte de Oliveira, Camila Albuquerque de Moraes, Cristiane Pereira, Camilla Ferreira Santos, Cinthia Dellaqua, Giovanna Haimowski, Josiane Vitor de Souza Oliveira, Dyemison Pinheiro da Silva, Priscilla Faria Pereira, Natalia Mercedes Cabral Amdi, Luciene Arrifano da Silva, Lucas De Souza Loureiro Abbud Santos, Ricardo Augusto De Braga Castro, Roberta Schiavon Nogueira, José Valdez Ramalho Madruga, Vinicius De Paula dos Anjos, Ana Caroline Coutinho Iglessias, on behalf of TransBordar Study Group

Casa da Pesquisa – Centro de Referência e Treinamento DST AIDS São Paulo, Unidade de Pesquisa, São Paulo, Brazil

**Background**: Even with antiretroviral therapy (ART), HIV/AIDS treatment continues to be a challenge in scenarios where inequalities of health access occur. Among transgender community, we have not only high prevalence of HIV compared with cisgender people (36.7% vs. 0.5%), but high rates of loss to follow‐up (LFU), explained by structural transphobia intersecting with other social determinants of health. This study aims to investigate factors associated with LFU and virological failure (VF) in transfeminine people living with HIV/AIDS in a Brazilian specialist STI service.

**Methods**: This retrospective cohort study included transgender women receiving longitudinal HIV care between 2019 and 2024 at a specialized referral centre in São Paulo. Data were abstracted from medical records and from the Brazilian national logistic and laboratory monitoring systems and subsequently entered into the REDCap platform. The statistical analysis was performed on Python v3.13.7 (statsmodels), performing logistic regression to calculate odds ratios (OR). The evaluated outcomes were LFU and VF was divided into current virological failure (CVF) and previous virological failure (PVF).

**Results**: In the study population (*N* = 300), we had 250 transgender women, 40 travestis and 10 other transfeminine identities. The mean age was 41.2 years. Race/ethnicity was self‐reported as mixed race (pardo) in 128 participants (42.7%). The majority did not complete high school 152 (50.7%) and 62 (20.7%) did not complete primary education. The average time living with HIV was 12.7 years (0.15–40.5). Due to outcomes, 36 participants (12.0%) had CVF, 97 (32.3%) had a history of PVF and 131 (43.7%) had LFU. We illustrate the association of sociodemographic factors with LFU and VF in Table 1. Age between 20 and 32 years old was associated with LFU, PVF and CVF. Black race was associated with a higher risk of PVF. Having 10–11 years of education was a protective factor across all three outcomes.

**Conclusions**: We conclude that social‐demographic factors such as age, race/ethnicity and educational levels were associated with negative outcomes in transgender women and travesti individuals, emphasizing the role of addressing retention efforts to these subgroups that belong to an already vulnerable population.

**TABLE 1:**
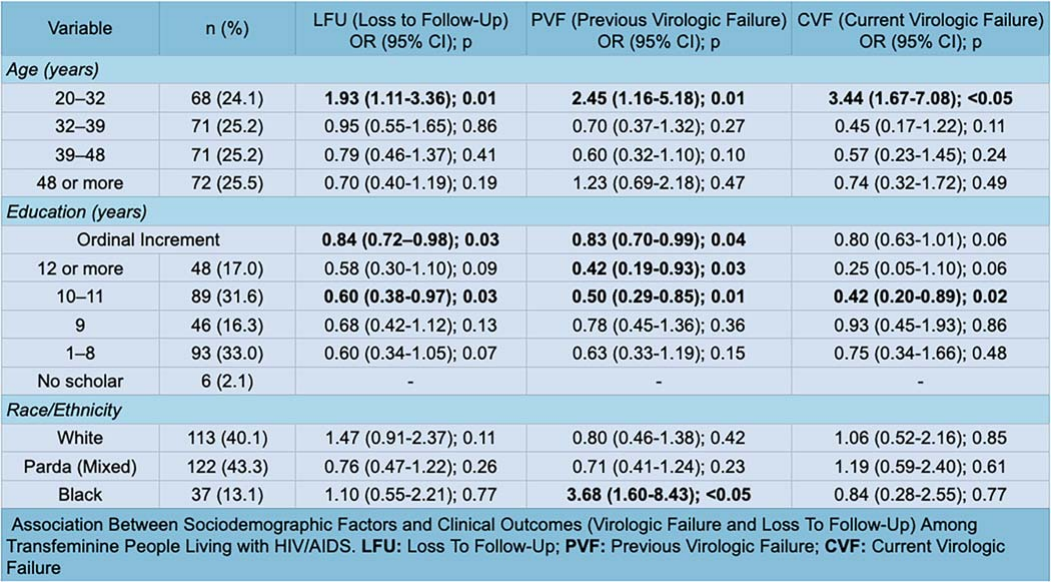
P236 | Odds ratio of sociodemographic factors and primary outcomes (virologic failure and loss to follow‐up).

### P237 PrEP Modality and Delivery Setting Preferences for Transgender and Nonbinary People in the UK: The PrEP‐Position Mixed‐Methods Study

Vian Rajabzadeh^1^, Sophie Strachan^1,2^, Melanie Smuk^1^, Nishat Halim^1^, Caro Camargo^1,2^, Chloé Pasin^1^, Megan Devonald^1^, Anqa Awan^2^, Taranjit Chana^3^, Kelsea Delatango^4^, Vimbai Mandaza^1^, Pamela Menzies‐Banton^3^, Zoch Nwaosu^3^, Juddy Otti^2,5^, Kyle Ring^1^, PrEP‐Position Steering Group Member^3^, Angelina Namiba^6^, Vanessa Apea^1^, Sara Paparini ^1^, Chloe Orkin^1^

^1^Queen Mary, University of London, London, UK; ^2^Sophia Forum, London, UK; ^3^The PrEP Position Steering Group, London, UK; ^4^Bare Radicals, London, UK; ^5^Africa Advocacy Foundation, London, UK; ^6^4M Net Mentor Mothers CIC, London, UK

**Background**: Transgender and nonbinary (TNB) people face disproportionate sexual health inequalities and layered structural barriers to PrEP access yet remain underrepresented in PrEP research and delivery planning. Long‐acting injectable (LAI) cabotegravir for PrEP is now NHS‐approved, but evidence on modality preferences and delivery setting barriers among TNB communities is scarce.

**Methods**: In partnership with community organization Sophia Forum, we conducted a convergent mixed‐methods, co‐produced survey and qualitative interview study (August 2025 to February 2026) with underserved groups. Qualitative interviews were analysed using an intersectional PrEP Need Contingency framework; survey data using descriptive statistics.

**Results**: Of 264 survey participants, 47 (17.8%) identified as TNB (Table 1): transgender men (*n* = 17, 36.2%), nonbinary/gender nonconforming (*n* = 23, 48.9%) and trans women (*n* = 8, 17.0%). PrEP awareness was 74.5%; among those aware (*n* = 35), interest in injectable PrEP was high (85.7%). Sexual health clinics were most selected as an acceptable delivery setting for LAI (66.7%); GP/family doctor and pharmacy by 53.3% each; community settings by 20.0%. Of 38 interviewees, 12 identified as TNB (3 transgender women, 3 non‐binary or genderfluid, 6 transmasculine/transgender men; ethnicities: white, mixed and South Asian). Qualitative interviews revealed participants framed injectable PrEP as a direct resolution to barriers to oral PrEP, rather than a preference. Across subgroups, injectable PrEP was valued for resolving trans healthcare bottlenecks, polypharmacy burden, aversive oral medication histories and for enabling co‐delivery with gender‐affirming hormone care. Participants described disclosure risk in non‐specialist settings and a strong preference for providers knowledgeable about trans healthcare and respectful of gender identity. GP settings generated ambivalence: over half selected GP (53.3%), yet precarious trans healthcare relationships, including misgendering and incorrect record‐keeping, made GP contact a source of harm for some. On‐demand oral PrEP was infrequently mentioned yet was discussed as potentially best suited to those with lower‐frequency or unpredictable sexual activity.

**Conclusions**: Injectable PrEP interest is very high among TNB people, addressing adherence burden, disclosure risk and alignment with existing injection care. Specialist setting preference reflects a rational response to generalist NHS trans healthcare failings. Priorities include pathway reform enabling injectable PrEP co‐delivery within gender‐affirming care, investment in trans‐affirming services and targeted on‐demand dosing communication.

**TABLE 1:**
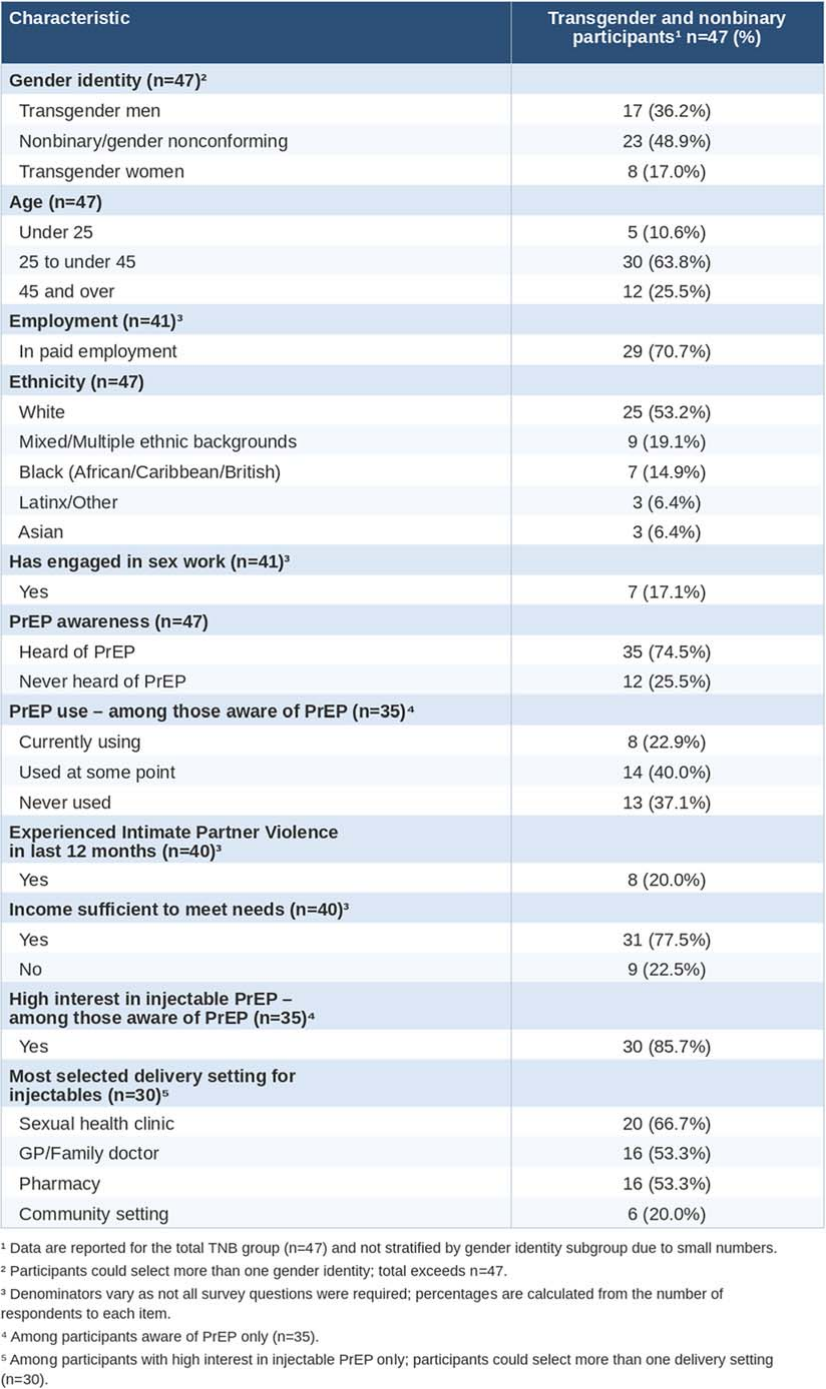
P237 | Baseline characteristics of transgender and nonbinary participants in the PrEP‐Position survey (*n* = 47).

## Clinical Management Considerations: Other

### P238 Efficacy and Safety of Bictegravir/Emtricitabine/Tenofovir Alafenamide (B/F/TAF) Tablet for Oral Suspension (TOS) in Children and Infants Aged ≥1 Month, Weighing 3 to <25 kg: Week 48 Outcomes

Natella Rakhmanina ^1^, Eva Natukunda^2^, Peter Elyanu^3^, Jeanne Coetzee^4^, Samantha Fry^5^, Elizabeth Hellström^6^, Umesh Lalloo^7^, Afaaf Liberty^8^, Kathleen McGann^9^, Carina A. Rodriguez^10^, Athira Sudhakaran^11^, Kate Anteyi^11^, Vinicius A. Vieira^11^, Kathryn Kersey^11^, Flavia Matovu Kiweewa^12^

^1^Division of Infectious Diseases, Children's National Hospital, Department of Pediatrics, The George Washington University School of Medicine and Health Sciences, Washington, DC, US; ^2^Department of Pediatrics, Joint Clinical Research Centre, Kampala, Uganda; ^3^Baylor College of Medicine Children's Foundation–Uganda, Kampala, Uganda; ^4^Wits RHI, University of the Witwatersrand, Johannesburg, South Africa; ^5^Department of Paediatrics and Child Health, FAMCRU, Stellenbosch University, Cape Town, South Africa; ^6^Be Part Yoluntu Centre, Paarl, South Africa; ^7^Enhancing Care Foundation, Durban University of Technology, Durban, South Africa; ^8^Perinatal HIV Research Unit, Chris Hani Baragwanath Hospital, Johannesburg, South Africa; ^9^Department of Pediatrics, Duke University Medical Center, Durham, NC, US; ^10^Department of Pediatrics, Division of Infectious Diseases, Morsani College of Medicine, University of South Florida, Tampa, FL, US; ^11^Gilead Sciences, Inc., Foster City, CA, US; ^12^Makerere University – Johns Hopkins University Research Collaboration, Kampala, Uganda

**Background**: B/F/TAF is an antiretroviral therapy (ART) approved in the USA for children weighing ≥14 kg. It is well tolerated, with a high barrier to resistance. A tablet for oral suspension (TOS) for those with difficulty swallowing tablets is being evaluated in a phase 2/3 trial of B/F/TAF in children with HIV‐1 (NCT02881320). We present week (W) 48 efficacy, safety and acceptability/palatability outcomes in participants enrolled in Cohort 4 (aged ≥1 month, weighing 3– < 25 kg).

**Methods**: Participants in Group (G) 1–4 (≥2 years, 14–< 25 kg; ≥1 month, 10– < 14 kg; ≥1 month, 6– < 10 kg; and ≥1 month, 3– < 6 kg, respectively) received B/F/TAF TOS 30/120/15 mg once daily, 7.5/30/3.75 mg twice daily (BID), 3.75/15/1.88 mg BID and 1.88/7.5/0.94 mg BID, respectively. At screening, participants in G1 had virologic suppression (VS; HIV‐1 RNA <50 copies/mL) on ART; those in G2–4 were treatment‐naïve or on ART. Efficacy, safety and acceptability/palatability were assessed.

**Results**: Of 53 participants (*n* = 8, *n* = 14, *n* = 16 and *n* = 15 in G1–4, respectively); 58% were female and 94% were black. At baseline, in G1–4, respectively, median (range) age was 4.0 (2.0–7.0) years, 2.0 (1.7–4.0) years, 8 (2–19) months and 4 (2–16) months; median (Q1, Q3) weight was 15.9 (14.6, 17.0), 11.3 (11.0, 13.0), 8.0 (6.8, 8.8) and 5.4 (5.3, 5.8) kg. At baseline, 8/8 (100%), 6/14 (43%), 9/16 (56%) and 1/15 (7%) participants in G1–4, respectively, had VS and the overall median (Q1, Q3) CD4% was 33.8% (29.3, 38.1). At W48, 7/8 (88%), 13/14 (93%), 12/16 (75%) and 9/15 (60%) participants in G1–4, respectively, had VS (US FDA Snapshot algorithm) and the overall CD4% was 35.3% (30.6, 41.4). Through W48, 15% of participants experienced drug‐related adverse events (8/53; all Grade 1). One adverse event (pulmonary tuberculosis; G1) resulted in study drug discontinuation and there was one death (pneumonia; G4); neither was considered study drug related. Most caregivers reported that B/F/TAF TOS was easy to prepare, with perceived neutral/favourable acceptability and palatability.

**Conclusions**: B/F/TAF demonstrated favourable efficacy, safety and acceptability/palatability through W48, supporting its use in this population, for whom safe and effective single‐tablet regimens are limited.

### P239 Virological Outcomes of Bictegravir/Emtricitabine/Tenofovir Alafenamide (B/F/TAF) Through Week 48 in Children and Infants Aged ≥1 Month and Weighing ≥3 to <25 kg

Natella Rakhmanina ^1^, Eva Natukunda^2^, Jeanne Coetzee^3^, Peter Elyanu^4^, Samantha Fry^5^, Elizabeth Hellström^6^, Umesh Lalloo^7^, Afaaf Liberty^8^, Carina A. Rodriguez^9^, Kristen Andreatta^10^, Jing Nui^10^, Athira Sudhakaran^10^, Vinicius A. Vieira^10^, Flavia Matovu Kiweewa^11^

^1^Children's National Hospital, The George Washington University, Washington, DC, US; ^2^Joint Clinical Research Centre, Kampala, Uganda; ^3^Wits RHI, University of the Witwatersrand, Johannesburg, South Africa; ^4^Baylor College of Medicine Children's Foundation–Uganda, Kampala, Uganda; ^5^Department of Paediatrics and Child Health, FAMCRU, Stellenbosch University, Cape Town, South Africa; ^6^Be Part Yoluntu Centre, Paarl, South Africa; ^7^Enhancing Care Foundation, Durban University of Technology, Durban, South Africa; ^8^Perinatal HIV Research Unit, University of Witwatersrand, Chris Hani Baragwanath Hospital, Johannesburg, South Africa; ^9^Morsani College of Medicine, University of South Florida, Tampa, FL, US; ^10^Gilead Sciences, Inc., Foster City, CA, US; ^11^Makerere University – Johns Hopkins University Research Collaboration, Kampala, Uganda

**Background**: B/F/TAF is an antiretroviral therapy (ART) approved for adults and children with HIV‐1 weighing ≥14 kg; however, treatment remains challenging in paediatric populations, particularly in infants where consistent administration may be affected by caregiver burden, disclosure considerations and tolerability/acceptability issues. A B/F/TAF tablet for oral suspension (TOS) is being evaluated in children with HIV‐1.

**Methods**: Participants in groups 1–4 (≥2 years, 14– < 25 kg; ≥1 month, 10– < 14 kg; ≥1 month, 6– < 10 kg; and ≥1 month, 3– < 6 kg, respectively) of Cohort 4 in this phase 2/3 trial (NCT02881320) received B/F/TAF TOS 30/120/15 mg once daily, 7.5/30/3.75 mg twice daily (BID), 3.75/15/1.88 mg BID and 1.88/7.5/0.94 mg BID, respectively. At screening, participants in group 1 had HIV‐1 RNA <50 copies/mL on ART; those in groups 2–4 were treatment‐naïve or on ART. Virological suppression and CD4 parameters were assessed through week (W) 48. HIV‐1 genotypic and phenotypic analyses were performed in participants who had HIV‐1 RNA ≥50 copies/mL at W48 and ≥200 copies/mL at confirmed virologic failure or last visit. In groups 2–4, intensive pharmacokinetic analyses were conducted at W4 and sparse pharmacokinetics throughout the study.

**Results**: Overall, 53 participants were enrolled; 58% were female, 94% were Black, median (range) age was 15 (2–84) months. Baseline and W48 efficacy outcomes are reported in Table 1. At W48, most participants had HIV‐1 RNA <50 copies/mL (US FDA snapshot and missing = excluded) and < 400 copies/mL (missing = excluded). CD4 count and % remained stable within the physiological variation for this age group. B/F/TAF TOS was generally well tolerated through W48, with a safety profile consistent with that previously reported and no new safety signals. Five participants met criteria for resistance analyses; two (both Group 4) developed treatment‐emergent resistance through W48. Both participants had suboptimal adherence reported by investigators and confirmed via drug levels.

**Conclusions**: B/F/TAF demonstrated favourable efficacy through W48, supporting its use in this population for whom treatment options are limited.

**TABLE 1:**
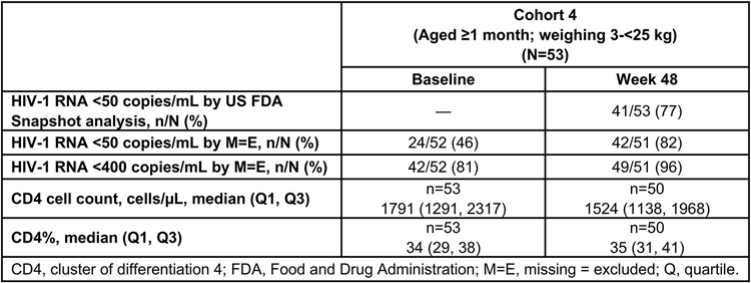
P239 | Efficacy outcomes at baseline and week 48.

### P240 Prescription Medication Use, Comorbidity Burden and Drug−Drug Interaction Potential Among People With HIV in a Large US Claims Database (2024)

Andrew Weber ^1^, Linda Grasfeder^1^, Harini Vadlamudi^2^, George Mu^3^, Palak Phansalkar^4^, Aimee Metzner^5^, Justin Hay^6^, Mahmoud Afifi^6^, Joseph Polli^7^

^1^Clinical Pharmacology, ViiV Healthcare, Durham, NC, US; ^2^Real World Data, Measurement and Analytics, GSK, Bengaluru, India; ^3^Real World Data, Measurement and Analytics, GSK, Collegeville, PA, US; ^4^Discovery DMPK, ViiV Healthcare, Branford, CT, US; ^5^US Health Outcomes, ViiV Healthcare, Tampa, FL, US; ^6^Certara, Radnor, PA, US; ^7^DMPK, ViiV Healthcare, Durham, NC, US

**Background**: People with HIV are an increasingly ageing population with associated comorbidities and comedications that influence treatment selection, drug–drug interaction (DDI) risk and tolerability. To aid in designing new HIV medicines, including long‐acting regimens and support healthcare professional use of these therapies, an analysis of current prescription usage and comorbidity burden in a US‐based healthcare claims database was performed.

**Methods**: A cross‐sectional analysis of Optum Clinformatics de‐identified claims was conducted where people with HIV were identified via HIV diagnosis codes or by use of an FDA‐approved HIV‐1 treatment regimen, along with continuous pharmacy benefits enrolment during the 2024 study period. Population demographics, prevalence of the top 100 National Drug Code (NDC) medications, Elixhauser comorbidities, along with selected comedications and comorbidities of special interest were summarized. The top 100 medications were cross referenced with the Certara Drug Interaction Database (DIDB) to further understand the mechanisms of potential interactions via individual enzymes and transporters routinely considered under regulatory (ICH M12) guidance.

**Results**: A population of 39,871 people with HIV was identified, which was predominantly male (77%) and aged ≥50 years (71%). The most frequently recorded races were Caucasian (37%) and Black (30%), with 60% non‐Hispanic. Comorbidity burden included hypertension (50%), diabetes (37%), depression (22%), obesity (19%), renal failure (18%), chronic pulmonary disease (17%) and liver disease (16%). Medication usage was common with >50% prevalence of anti‐infectives, central nervous system agents, immunologic agents and/or metabolic agents. The highest prescribed individual drug types included antiretroviral therapies, vaccines and cardiometabolic agents (Figure 1). Cytochrome P450 (CYP) enzymes were the most common route of potential interaction (object or precipitant), with CYP3A accounting for the largest overall share. Interactions via transporters were the second most common, with uridine 5′‐diphospho‐glucuronosyltransferase (UGT) enzymes having the lowest share of potential interactions.

**Conclusions**: In this large US claims analysis, the identified population in 2024 were predominantly ≥50 years of age and demonstrated high prevalence of cardiometabolic and other chronic conditions, as well as high rates of comedication usage alongside antiretroviral regimens. These findings highlight the importance of considering DDIs and comorbidity profiles into HIV regimen development and clinical practice.

**FIGURE 1:**
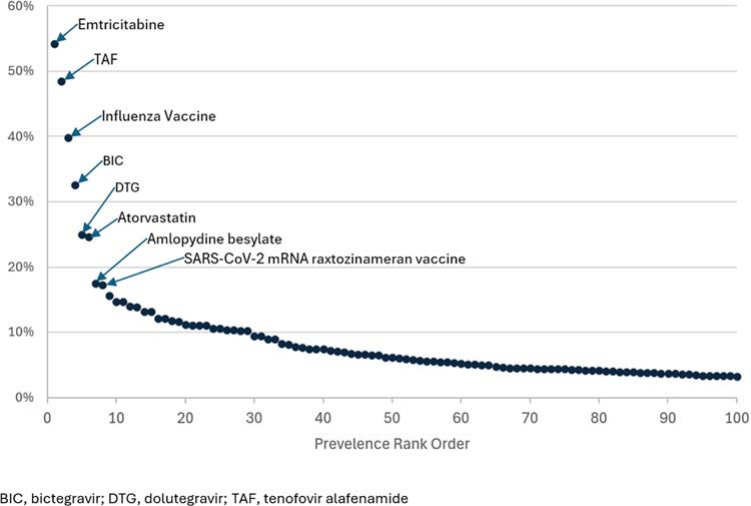
P240 | Prevalence of the top 100 NDC prescription comedications in people with HIV in the Optum Clinformatics database.

### P241 Characteristics of Children and Young People Newly Presenting to Paediatric HIV Services in England: 2023–2025

Alexandra David ^1^, Alasdair Bamford^2,3^, Kate Francis^1^, Charlie Gorman^1^, Anna Kafkalia^4^, Claire Thorne^1^, Helen Peters^1^

^1^Children's HIV and AIDS Reporting System, UCL Great Ormond Street Institute of Child Health, London, UK; ^2^Great Ormond Street Hospital for Children NHS Foundation Trust, London, UK; ^3^Infection, Immunity and Inflammation Department, UCL Great Ormond Street Institute of Child Health, London, UK; ^4^NHS England Specialised Commissioning, London, UK

**Background**: The number of children and young people (CYP) newly presenting to HIV services in England is increasing.

**Methods**: NHS England commissions the Children's HIV and AIDS Reporting System to collect data on CYP seen for HIV paediatric care in England until transition to adult services. We describe timing and circumstances surrounding HIV diagnosis, immune characteristics and treatment for those newly presenting in 2023–2025. Late HIV diagnosis was defined by age thresholds: ≥5 years: CD4 count ≤350 cells/mm^3^; 1–5 years: CD4 < 500 cells/mm^3^; < 1 year: CD4 < 750 cells/mm^3^; or presence of an AIDS‐defining condition [1].

**Results**: Of 74 CYP (median age 13 years, IQR 9–15), 52 (70.3%) were born and diagnosed abroad, 18 (24.3%) were born abroad and diagnosed in England and 4 (5.4%) were born and diagnosed in England. Among all CYP, 59% (40/68 with available data) were seen by specialist services ≤14 days from referral; for those seen > 14 days, the median was 26 days (IQR 18–37). Among 52 CYP diagnosed abroad, HIV was identified following symptomatic presentation (24/52), screening after mother/other family member diagnosed (13/52) and maternal diagnosis in pregnancy (11/52) (Figure 1). All 52 received ART abroad prior to arrival: 18 (34.6%) were dolutegravir‐based regimens, 24 (46.1%) bictegravir/emtricitabine/tenofovir alafenamide, 2 (3.9%) protease inhibitor‐based regimens and 8 (15.4%) other ART. 4/52 (7.7%) presented with advanced immunodeficiency (mean CD4 266.5 cells/mm^3^). Among 18 CYP born abroad and diagnosed after their arrival, HIV diagnosis followed symptomatic presentation (10/18), screening after mother/other family member was diagnosed (7/18) and screening when moving to England (1/18) (Figure 1). These 18 CYP were diagnosed within a median of 1.6 years (IQR 1.3–3.2) after their arrival, at a median of 13 years old (IQR 11–15). All 4 England‐born CYP were diagnosed following maternal diagnosis. Among all CYP diagnosed in England, 12 (55.5%) had a late HIV diagnosis; median time to ART initiation was within 5 days (IQR 0–10) of HIV diagnosis.

**Conclusions**: Just over half of CYP diagnosed in England presented late with advanced immunosuppression. Screening CYP following maternal/family diagnoses remains important. Ongoing surveillance is crucial in identifying missed opportunities and barriers to care and supporting services to ensure early recognition and treatment initiation.

**Reference**

1. Curto C, Tritzali M, Bamford A, Bailey AC, Lyall EGH, Mackie NE, Tickner, et al. Late diagnosis of paediatric HIV infection in high‐income countries: Lessons from the HIV Perinatal Virtual Clinic. HIV Medicine, 2026;27(3), 477–484.

**FIGURE 1:**
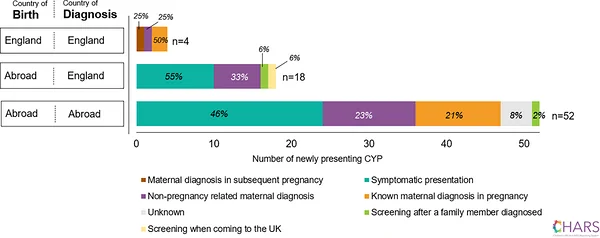
P241 | Circumstances at HIV diagnosis among newly presenting children and young people in England (2023–2025).

### P242 Age‐Related Decline in Virological Suppression Among Adolescents Living With HIV: Increasing Rates of Viral Load ≥1000 copies/mL From Early to Late Adolescence

Serhii Riabokon, Diana Shevchenko, Olena Samsonova, Larysa Hetman

Department of HIV management and counteraction of Public Health Center of the MOH of Ukraine, Kyiv, Ukraine

**Background**: Adolescents living with HIV represent one of the most vulnerable populations for maintaining long‐term antiretroviral therapy success. Biological, psychological and social changes during adolescence may negatively affect treatment adherence, leading to virological failure. Understanding age‐specific patterns of viral suppression is essential for developing targeted interventions.

**Methods**: We conducted a retrospective longitudinal analysis of 3239 adolescents living with HIV receiving ART. The study included 20,228 HIV viral load measurements obtained during routine follow‐up, allowing assessment of age‐specific trends in virological outcomes across adolescence. Virological outcomes were evaluated across consecutive age groups. The primary outcome was the proportion of viral load measurements ≥1000 copies/mL, consistent with the WHO definition of virological failure. Age‐specific trends in virological suppression were assessed from early to late adolescence.

**Results**: A progressive increase in the proportion of viral load measurements ≥1000 copies/mL was observed with increasing age (Figure 1). The lowest prevalence of virological failure was found among younger adolescents aged 13–14 years, followed by a steady increase through ages 15–20 years, reaching the highest level at 20 years. Consequently, the overall virological effectiveness of ART declined throughout adolescence, indicating a gradual loss of viral suppression during the transition to adulthood. The observed trend remained consistent across age groups.

**Conclusions**: Virological effectiveness of ART declines progressively during adolescence, with a marked increase in viral load ≥1000 copies/mL beginning at approximately 14 years of age and continuing through 20 years. These findings identify early adolescence as a critical period for intervention and support the implementation of enhanced adherence strategies, more intensive viral load monitoring and adolescent‐friendly HIV services before virological failure becomes established. Early targeted interventions may improve long‐term treatment outcomes and facilitate a successful transition to adult HIV care.

**FIGURE 1:**
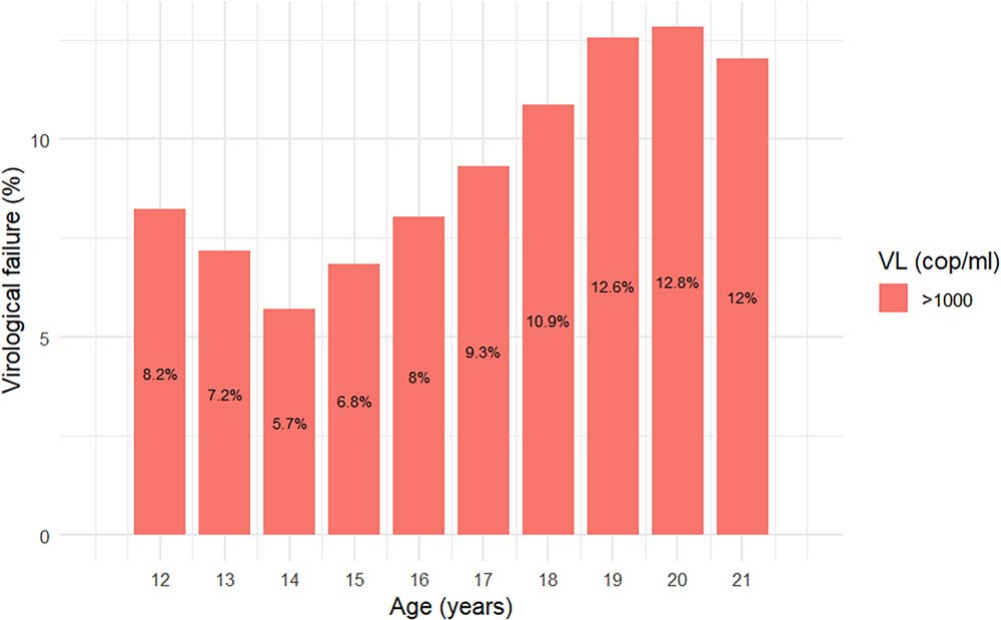
P242 | Virological ineffectiveness among adolescents on ART.

### P243 A Study on Depression and Stress Behaviours in People Living With HIV After 3 Years of Harm Reduction Intervention for Chemsex

Na‐Lee Sun, Chien‐Yu Cheng, Shu‐Hsing Cheng

Comprehensive AIDS Care Center, Taoyuan, Taiwan

**Background**: Chemsex, defined as the use of illicit substances to enhance sexual experiences among men who have sex with men (MSM), has been increasingly associated with adverse psychological outcomes among people living with HIV (PLWH), including substance‐related mood disorders, depression and elevated stress levels. In response to these challenges, Taoyuan General Hospital established a structured harm reduction group programme incorporating multi‐modal psychosocial interventions to support PLWH with chemsex experience. This study aimed to evaluate changes in depressive symptoms, perceived stress and related behavioural outcomes among PLWH with chemsex experience following participation in a multi‐modal harm reduction group intervention from 2023 to 2025.

**Methods**: A retrospective intervention study was conducted between 2023 and 2025, enrolling 40 PLWH with prior chemsex experience. Participants engaged in a 1‐year harm reduction programme consisting of weekly group sessions (2 h per session, 52 weeks per year). Intervention components included drug harm reduction education, emotional exploration, psychodrama, dance/movement therapy and ceramic art therapy, with voluntary participation across activities. Pre‐ and post‐intervention assessments were conducted using validated instruments, including the Brief Symptom Rating Scale (BSRS‐5), the Beck Depression Inventory–II (BDI‐II, Chinese version) and the Perceived Stress Scale (PSS). Changes across the 3‐year period were analysed using the Wilcoxon signed‐rank test for paired comparisons and the Friedman test for overall longitudinal analysis.

**Results**: All participants were men who have sex with men (mean age: 38 ± 8 years), averaging 8.1 years of substance use. Following the harm reduction intervention, psychological outcomes significantly improved over 3 years (*p* < 0.01). Mean BSRS‐5 scores decreased from 8.95 ± 6.40 (2023) to 5.95 ± 3.30 (2024) and 2.75 ± 1.25 (2025) (Table 1). Similarly, BDI‐II scores declined from 18.8 ± 12.75 to 13.0 ± 7.4 and 6.0 ± 3.2, while PSS scores dropped from 31.25±11.50 in 2023 to 24.1 ± 8.83 in 2024 and 15.35 ± 6.36 in 2025 (*p* < 0.01) (Table 2).

**Conclusions**: A harm reduction group intervention significantly reduced depression and stress among PLWH practicing chemsex. Three years of psychosocial support enhanced recovery, improved quality of life and lessened harms, proving integrated approaches promote long‐term well‐being.

**TABLE 1:**
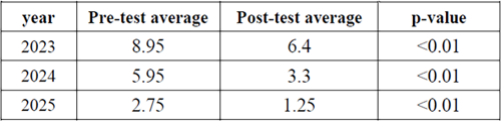
P243 | Comparison of BSRS‐5 Mood Thermometers.

**TABLE 2:**
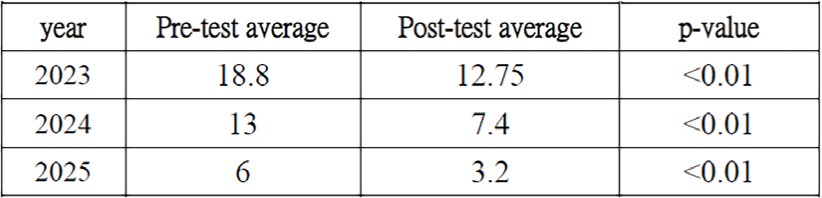
P243 | Comparison of the Beck Depression Scale.

### P244 Who Suppresses Faster? Exploring Time to Viral Suppression Among School Going Children and Adolescents Living With HIV in Kampala, Uganda: A Survival Analysis

Gertrude Namale, Patrick Kazooba, Emmanuel Sendaula, Francis Wasswa, Hilda Achayo, Joseph Lwembawo, Josephine Kaleebi

Reach Out Mbuya Community Health Initiative, Kampala, Uganda

**Background**: Timely viral load suppression (VLS) is essential for improving outcomes among children and adolescents living with HIV (CALHIV) and achieving the UNAIDS 95‐95‐95 targets [1]. However, CALHIV continue to experience lower VLS rates than adults, particularly in sub‐Saharan Africa [2, 3]. Evidence on time to suppression and its predictors in routine programme settings in Uganda remains limited. This study assessed time to VLS and associated predictors among school‐going CALHIV in Kampala, Uganda.

**Methods**: We conducted a retrospective cohort analysis of routinely collected programmatic data from October 2023 to September 2025 among school‐going CALHIV aged 10–19 years who had been on ART for at least 6 months in seven chronic ART clinics implementing the school health programme in Kampala. Socio‐demographic and clinical data were abstracted from medical records. The primary outcome was time‐to‐first documented VLS (HIV RNA < 200 copies/mL), measured in months from ART initiation. Kaplan–Meier methods estimated time to VLS and Cox proportional hazards models identified predictors.

**Results**: Of the 178 school‐going CALHIV in care, the mean age was 14 years (SD ± 3.3) and 57.3% were female. The median time to VLS after ART initiation was 1.4 years (IQR 0.53–4.8), with an estimated suppression rate of 29.6 per 100 person‐years (95% CI: 24.4–36.0). The hazard of VLS increased with age among male children compared with females (aHR 1.13, 95% CI: 1.02–1.25) (Figure 1). CALHIV whose caregivers were living with HIV (aHR 0.11, 95% CI: 0.02–0.53) or had unknown HIV status (aHR 0.11, 95% CI: 0.02–0.62) had significantly lower hazards of VLS compared with those with HIV‐negative caregivers. Children with signs of TB or suspected TB had reduced hazards of VLS (aHR 0.52, 95% CI: 0.29–0.96) compared with TB‐negative children. Enrolment in a differentiated service delivery model did not significantly affect time to VLS (aHR 1.46, 95% CI: 0.59–3.59).

**Conclusions**: VLS among school‐going CALHIV in Kampala was achieved slowly, with a median time exceeding 1 year after ART initiation. Age, sex, caregiver HIV status and TB co‐morbidity significantly influenced VLS, underscoring the importance of targeted caregiver support and integrated TB–HIV interventions to improve outcomes in routine care settings.

**References**

1. UNAIDS. UNAIDS Global AIDS Update 2024: The Urgency of Now: AIDS at a Crossroads: Stylus Publishing, LLC; 2024.

2. Slogrove AL, Sohn AH. The global epidemiology of adolescents living with HIV: Time for more granular data to improve adolescent health outcomes. Current Opinion in HIV and AIDS. 2018;13(3):170–8.

3. WHO. Consolidated guidelines on HIV prevention, testing, treatment, service delivery and monitoring: Recommendations for a public health approach. World Health Organization; 2021.

**FIGURE 1:**
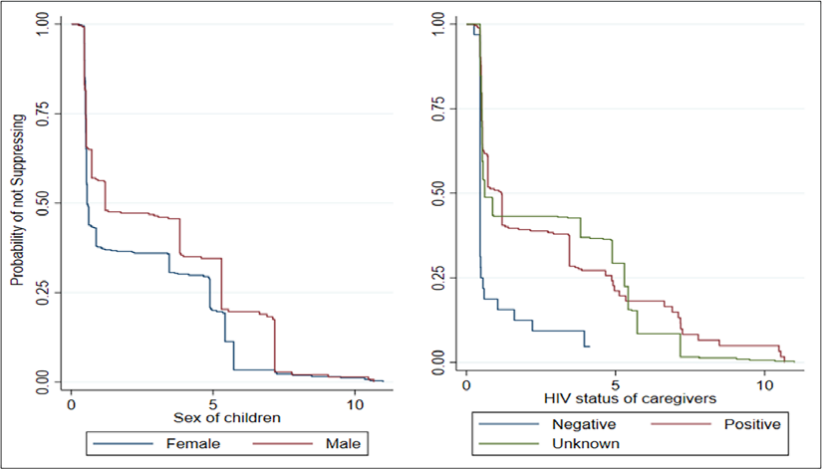
P244 | Kaplan–Meier curves showing time to viral suppression by sex and caregiver HIV status among children and adolescents living with HIV in Kampala, Uganda (October 2023 to September 2025).

### P245 Abstract Withdrawn

### P246 Cabotegravir and Rilpivirine Injectable Therapy With Concomitant Tuberculosis Preventative Treatment (TPT) Consisting of Rifapentine/Isoniazid as 1HP

Jinell White^1,2^, Brandon Christensen ^1,3^, Morgan Lahl^1,2^, M. John Gill^1,3^

^1^Southern Alberta Clinic, Calgary, AB, Canada; ^2^Alberta Health Services, Calgary, AB, Canada; ^3^Cumming School of Medicine, Department of Medicine | Infectious Diseases, Calgary, AB, Canada

**Background**: We assessed the pharmacokinetic impact of concomitant 1HP on LAI cabotegravir/rilpivirine (CAB/RPV) using therapeutic drug monitoring (TDM). Our case patient was indicated for TPT (tuberculosis preventative treatment) and given additional antiretroviral treatment (ART) to mitigate the drug−drug interactions between rifapentine and cabotegravir, rifapentine and rilpivirine and maintain viral suppression.

**Methods**: A 36‐year‐old male, BMI 31.79 kg/m^2^, on LAI CAB/RPV with rifapentine‐based therapy. 1HP is rifapentine 600 mg, isoniazid 300 mg and pyridoxine 25 mg daily for 30 days. Oral ART given, dolutegravir 50 mg twice daily, tenofovir disoproxil/emtricitabine 300/200 mg once daily, overlapping 30 days with 1 HP treatment following additional 2 weeks washout period, during CAB/RPV LAI. TDM process—British Columbia Centre for Excellence in HIV/AIDS laboratory using a valid LC‐MS/MS test (liquid chromatography‐tandem mass spectrometry) procedure [1]. Serum concentrations for dolutegravir, cabotegravir and rilpivirine, complete blood count. Alanine aminotransferase, total bilirubin, creatinine, collected at day 0, 14, 28 and 42. HIV RNA at baseline, day 28 and 56.

**Results**: Viral loads remained suppressed throughout 1HP treatment (day 28) and post 1HP treatment (day 56), prior to next CAB/RPV injection. Creatinine, bilirubin total, alanine aminotransferase (ALT) all remain within normal limits. No tolerability issues reported with oral ART and 1HP medications. Mild elevation in eosinophils was observed. Induction by rifapentine affected all three medications. On days 14 and 28, CAB level dropped to 1140 ng/mL and day 28, CAB level dropped further to 922 ng/mL, below the 1120 ng/mL target [2]. RPV levels were undetectable. Washout day 42, CAB level rebounded to 1367 ng/mL (baseline CAB was 2689 ng/mL) and RPV drug level was 52 ng/mL above the 32 ng/mL target [2] (baseline RPV 189 ng/mL). Dolutegravir levels returned to normal range after the 14‐day 1HP washout period. However, the induction effects on CAB/RPV continued after washout compared to baseline concentrations, demonstrating the impact on the absolute amount of medication administered (Figure 1).

**Conclusions**: Screening for latent TB is recommended for PWH, notably from countries endemic for Mycobacterium tuberculosis (Mtb), before initiating LAI cabotegravir and rilpivirine. Curing Mtb within the context of LAI for HIV is fraught with risk of inadequate drug levels leading to treatment failure and viral activity.

**References**

1. Watson BE, Atkinson K, Auyeung K, Baynes KA, Lepik KJ, Toy J, et al. Untimed efavirenz drug levels after switching from brand to generic formulations: A short communication. Ther Drug Monit. 2021;43(5):701–5. doi:10.1097/FTD.0000000000000876.

2. French HIV Resistance Society, ANRS‐MIE. HIV‐1 genotypic drug resistance interpretation algorithms [Internet]. Version 35. Paris: Agence Nationale de Recherches sur le SIDA et les Hépatites Virales; 2024 [cited 2026 Jun 25]. Available from: https://hivfrenchresistance.org

**FIGURE 1:**
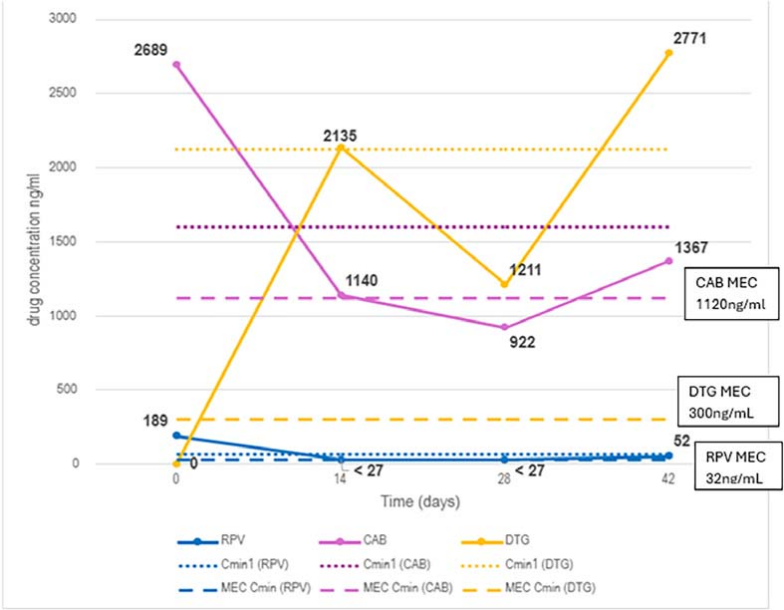
P246 | PK comparison of drug concentrations affected by rifapentine.

## Cure/Post‐Treatment Control

### P247 Context‐Dependent Innate Immune Reprogramming After BCG Vaccination in Treated HIV‐1 Infection

Cedric Dolle^1^, Terence Tutumlu^1^, Lena Bartl^1^, Baptiste Depouilly^2^, Doris Russenberger^1^, Marius Zeeb^1^, Katharina Kusejko^1^, Emily West^1^, Dominique Braun^1^, Magdalena Schwarzmüller^1^, Baptiste Elie^1^, Alexandra Trkola^1^, Huldrych F. Günthard^1^, Johannes Nemeth ^1^

^1^Department of Infectious Diseases and Hospital Epidemiology, University Hospital Zurich, University of Zurich, Zurich, Switzerland; ^2^Department of Mathematics, ETH Zurich, Zurich, Switzerland

**Background**: Persistent immune activation and altered innate immune function remain common in people with HIV (PWH) despite viral suppression on antiretroviral therapy (ART). Bacillus Calmette–Guérin (BCG) vaccination can induce heterologous immune effects through long‐term reprogramming of innate immunity. In this pre‐existing immune context, BCG vaccination might be expected to further amplify innate activation, but how these processes interact is unknown.

**Methods**: We performed longitudinal systems‐level immune profiling within the randomized, placebo‐controlled BELIEVE trial of BCG vaccination in ART‐treated PWH. Peripheral blood was analysed before vaccination and at 3 and 6 months using high‐dimensional flow cytometry, functional immune assays, single‐cell multiomic profiling, plasma proteomics and longitudinal clinical data.

**Results**: BCG vaccination was associated with a coordinated reorganization of innate immune activity. The most prominent changes involved the NK/interferon axis, with reduced unstimulated IFN‐γ production and lower direct cytotoxicity against several target cell lines. In contrast, cytokine responses remained inducible after stimulation and antibody‐dependent cellular cytotoxicity was preserved, with a potential transient increase at 3 months. Multiomic and proteomic analyses supported this functional pattern, showing reduced interferon‐ and activation‐associated programmes without major shifts in NK cell subset composition. Circulating myeloid cell numbers increased, whereas HLA‐DR expression decreased and heterologous TNF‐α responses remained unchanged, indicating a response distinct from canonical circulating myeloid trained immunity. Adaptive and humoral immune parameters remained largely stable and longer‐term follow‐up revealed no evidence of adverse virological or clinical outcomes.

**Conclusions**: In ART‐treated PWH, BCG vaccination selectively reprogrammed innate immune function towards lower tonic interferon‐linked and direct cytotoxic activity while preserving stimulus‐induced and Fc‐dependent effector responses. These findings reveal a context‐dependent reorganization of innate immune set‐points and provide a rationale for testing BCG‐modulated Fc‐dependent activity in HIV‐specific systems.

### P248 Abstract Withdrawn

### P249 Gut Microbiota Signatures Associated With Incomplete CD4 Recovery in Antiretroviral Therapy‐Treated People Living With HIV

Aycan Gundogdu^1^, Nermin Safarova^2^, Buket Banu Ozkan^3^, Özkan Ufuk Nalbantoglu^4^, Aysegul Ulu Kilic ^2^

^1^Immunology, Istanbul, Türkiye; ^2^Infectious Diseases, Kayseri, Türkiye; ^3^Genome and Stem Cell Center, Kayseri, Türkiye; ^4^Computer engineering, İzmir, Türkiye

**Background**: Incomplete CD4 T‐cell recovery despite sustained virological suppression remains a major clinical challenge in people living with HIV (PLWH) receiving antiretroviral therapy (ART). Although gut microbiota dysbiosis has been linked to persistent immune activation and impaired immune reconstitution, community‐wide microbial differences associated with poor CD4 recovery have been inconsistent, indicating the need for targeted compositional analyses. To identify gut microbiota features associated with low versus high CD4 recovery in ART‐treated PLWH with sustained viral suppression.

**Methods**: We analysed stool microbiota profiles from 40 ART‐treated PLWH stratified into low (*n* = 20) and high (*n* = 20) CD4 recovery groups based on longitudinal CD4 trajectories over 12 months. Microbial profiling was performed using 16S rRNA gene amplicon sequencing. Alpha diversity indices (Shannon, Simpson, observed richness) and beta diversity metrics (Bray−Curtis and Aitchison/CLR‐Euclidean distances) were calculated, with group differences assessed using permutation‐based multivariate analysis of variance (PERMANOVA). Differential abundance analyses were conducted on centred log‐ratio‐transformed relative abundances using covariate‐adjusted linear models with false discovery rate correction. Exploratory microbiota‐only classification analyses were performed using cross‐validated machine learning approaches.

**Results**: Alpha diversity and overall community structure showed substantial overlap between groups, with no significant differences in Shannon diversity, richness or beta diversity by PERMANOVA. Despite weak community‐wide separation, differential abundance analyses identified a focused set of taxa enriched in the low CD4 recovery group, most notably *Leptotrichia* and *Bacteroides* after false discovery rate adjustment (Figure 1 and Table 1). Several additional oral‐associated genera, including *Neisseria*, *Rothia*, *Actinomyces* and *Eikenella*, showed nominal enrichment. Targeted compositional analyses demonstrated a lower *Prevotella*‐to‐*Bacteroides* balance and a higher composite oralization score in the low CD4 recovery group, indicating a shift towards oral‐associated microbial signatures (Figure 2). Exploratory microbiota‐only classification models showed moderate discriminatory performance for low versus high CD4 recovery (mean ROC‐AUC ∼0.8).

**Conclusions**: Incomplete CD4 recovery during suppressive ART was associated with targeted gut microbiota signatures rather than broad community‐wide dysbiosis. Enrichment of oral‐associated taxa and a shift towards a more Bacteroides‐weighted community may reflect mucosal barrier dysfunction and persistent immune activation. These findings suggest that specific microbial features, rather than global diversity measures, contribute to immunologic heterogeneity among virologically suppressed PLWH and warrant further longitudinal and mechanistic investigation.

**FIGURE 1:**
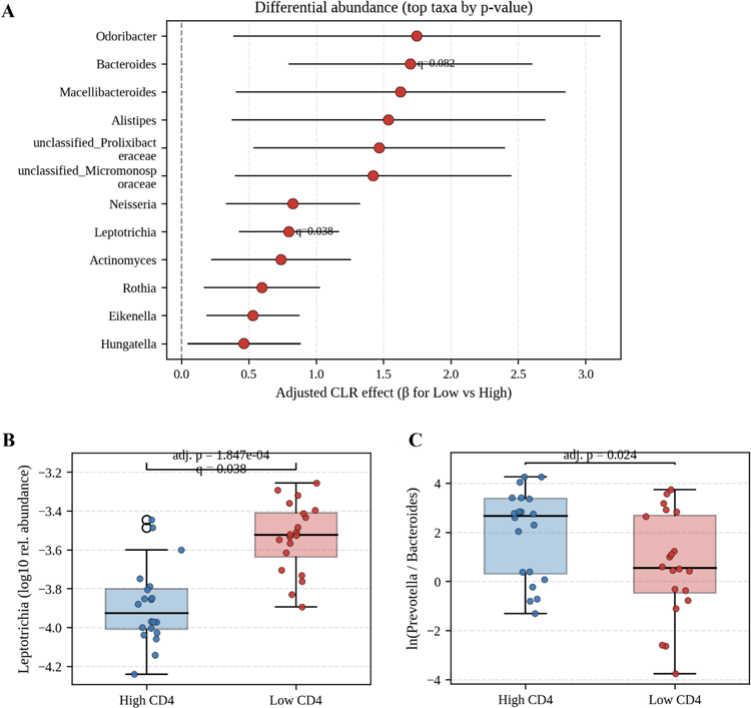
P249 | Key microbiome compositional signatures associated with the study groups (A). Differential abundance effect sizes (adjusted CLR model) for the most strongly associated taxa: relative abundance of *Leptotrichia* (B); ln (*Prevotella*/*Bacteroides*) balance (C).

**TABLE 1:**
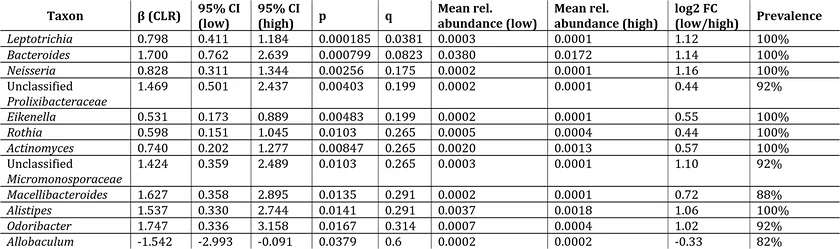
P249 | Top taxa associated with low CD4 recovery (CLR‐OLS; prespecified covariate‐adjusted model). β is the adjusted CLR effect for low versus high CD4 recovery. Mean relative abundances are shown after closure within the genus‐like layer. *q*‐values are Benjamini−Hochberg FDR across the 206 tested taxa.

### P250 Towards NGS‐Based Characterization of the HIV‐1 5′ Leader Region in Non‐Suppressible Viraemia

Marta Illescas‐López ^1,2^, Paloma Muñoz‐Báez^1,2^, Ana Fuentes^1,2^, Adolfo De Salazar^1,2^, Federico García^1,2^

^1^Hospital Universitario Clínico San Cecilio, Granada, Spain; ^2^Instituto de Investigación Biosanitaria Ibs.Granada, Granada, Spain

**Background**: The HIV‐1 5′ leader region is highly conserved and regulates essential viral life cycle steps. It contains key functional elements, including the major splice donor site (MSD). Mutations or deletions within this region have been associated with persistent non‐suppressible viraemia (NSV) as they enable transcription of defective proviruses and release of RNA‐containing but non‐infectious viral particles. Sequencing this region may help identify these mutations and clarify NSV origin, avoiding unnecessary treatment intensification. This study aimed to optimize an NGS protocol for analysing the region and detecting mutations potentially associated with NSV.

**Methods**: Seven plasma samples from viraemic patients with viral loads ranging from 10 million to 100 copies/mL were analysed to optimize amplification and sequencing protocol. RNA was extracted using the Maelstrom 4810 system (TanBead). Samples below 500 copies/mL were ultracentrifuged to concentrate viral genetic material. The HIV‐1 5′ leader region was amplified using specific primers (Nouhin et al. 2023) and the Q5 Hot Start High‐Fidelity PCR kit. Amplicons were sequenced on an Illumina NextSeq 1000 platform. FASTQ files were aligned and assembled against a Los Alamos database (https://www.hiv.lanl.gov) HIV‐1 reference sequence using CLC Genomics Workbench. Consensus sequences were analysed using BioEdit and MEGA to identify mutations.

**Results**: The 5′ leader region was successfully sequenced in six plasma samples from viraemic patients, the lowest successfully sequenced viral load was 100 copies/mL. Preliminary analysis revealed multiple nucleotide changes within the 5′ leader region, including A758T (*n* = 2), A760T (*n* = 2) and A757G (*n* = 1). Small deletions were detected at positions 756 (*n* = 4) and 760 (*n* = 4), together with small insertion at positions 734 (*n* = 1) and 759 (*n* = 2). However, none of the sequences showed the previously described deletions d22 (positions 727–748) or d21 (positions 740–760), nor the nucleotide substitutions T745A/C, which potentially generate defective proviruses. This is consistent with the use of samples from patients with active viraemia for protocol optimization.

**Conclusions**: An NGS‐based strategy for analysing the HIV‐1 5′ leader region was successfully optimized, demonstrating feasibility in low‐level viraemia plasma samples and enabling identification of mutations potentially associated with NSV. This approach may help clarify the origin of persistent viraemia under antiretroviral therapy.

### P251 Identification of the Molecular Target and Mechanism of Action of EK‐16A, a Natural HIV‐1 Latency‐Reversing Agent Derived From *Euphorbia kansui*

Jingna Xun ^1^, Xinyi Yang^2^, Yinzhong Shen^1^, Huanzhang Zhu^3^

^1^Shanghai Public Health Clinical Center, Shanghai, China; ^2^Yiwu Research Institute of Fudan University, YiWu, China; ^3^Fudan University, Shanghai, China

**Background**: HIV‐1 reservoirs represent a major obstacle to a functional cure. The “Shock and Kill” strategy has emerged as a promising approach for HIV‐1 eradication by reactivating latent virus and enabling clearance of infected cells. We previously identified EK‐16A as a potent and low‐toxicity natural latency‐reversing agent. This study aimed to identify its direct molecular target and elucidate the underlying mechanism of HIV‐1 latency reversal.

**Methods**: Thermal proteome profiling (TPP) was employed to screen potential direct targets of EK‐16A. The CRISPR/Cas9 lentivirus system was utilized to construct cell models with knocked‐out candidate target genes and functional verification was conducted in combination with the EK‐16A reactivation experiment. Furthermore, surface plasmon resonance (SPR) technology was used to verify the direct binding of EK‐16A to target proteins. Subsequently, the molecular mechanism of EK‐16A activating latent HIV‐1 was systematically analysed through methods such as Western blot, chromatin immunoprecipitation (ChIP) and luciferase reporter gene assay.

**Results**: A total of 25 differentially expressed proteins with significantly altered stability were identified. Through gene editing to knock out candidate genes followed by EK‐16A reactivation experiments, RNF168 and AKIRIN2 proteins were successfully identified as potential direct targets of EK‐16A. SPR experiments directly verified the direct interaction between RNF168, AKIRIN2 and EK‐16A. Subsequent Western blot and chromatin immunoprecipitation results further indicated that the two proteins mediate the HIV‐1 latent activation effect of EK‐16A by regulating the NF‐κB pathway, preliminarily revealing the potential mechanism of action of EK‐16A.

**Conclusions**: This study successfully identified RNF168 and AKIRIN2 as two direct key targets of EK‐16A through TPP screening and gene editing techniques. It preliminarily demonstrated that EK‐16A regulates the NF‐κB signalling pathway and promotes HIV‐1 latency activation through RNF168 and AKIRIN2.

### P252 From “What Might Have Been” to “What Could Be”: Counterfactuals in HIV Cure Research

Mark Janse ^1^, Kai Jonas^2^, Casper Rokx^1^

^1^Medical Microbiology and Infectious Disease, Erasmus MC, Rotterdam, Netherlands; ^2^Faculty of Psychology and Neuroscience Work and Social Psychology, Maastricht University, Maastricht, Netherlands

**Background**: Counterfactuals are part of the functional mental landscape simulating alternate realities (“what if, …”) and have been previously established as a coping mechanism also in PLWH [1]. Highly understudied, HIV cure clinical trials can constitute a context in which counterfactuals play a functional role for engagement decisions. We assessed counterfactual thinking before and after cure trial participation and examined whether it predicted intentions to participate in future trials.

**Methods**: Participants of the ORBIT randomized clinical trial (NCT06240520) were invited to complete the CTNES questionnaire [2] before and after trial participation. A higher CTNES score correlates with more counterfactual thoughts being present and can be investigated for four directional content specific subscales. Intention to participate in future trials, alongside demographic and psychological factors, was recorded through structured history taking. Main endpoints were the CTNES scores and future trial participation intention.

**Results**: A total of 45/49 (92%) participants filled in the pre‐treatment questionnaire and 40 (82%) participants filled in the post‐treatment questionnaire. Internal reliability of the CTNES scales was excellent (Cronbach's α = 0.90). Distribution of the CTNES scores remained stable throughout trial participation, both in the total score (*p* = 0.19) and relevant subscales (*p* = 0.18–0.72). Individual score evolution is shown in Figure 1. Multivariate analysis identified factors associated with counterfactuals. For pre‐treatment, CTNES score time spent living with HIV shows a negative association (β = –0.58, *p* < 0.01) and both trauma (β = 4.04, *p* = 0.10) and anxiety (β = 5.34, *p* < 0.01) showing a positive association. All participants indicated an interest in participating in follow‐up HIV cure clinical trials. As all ORBIT participants indicated an interest in participating in follow‐up trials, we could not reliably determine if counterfactuals increase or decrease the interest in participating in future HIV cure studies.

**Conclusions**: Previously lived traumatic experiences and anxiety increase the likelihood of having counterfactual thought patterns. Conversely, the longer someone lives with HIV, the lower the likelihood becomes of having counterfactual thought patterns. Willingness to enrol in future trials was universal and the resulting ceiling effect precluded testing its association with counterfactual thinking. Follow‐up studies with more sensitive measures are needed.

**References**

1. Epstude K, Jonas KJ. Regret and counterfactual thinking in the face of inevitability: The case of HIV‐positive men. Soc Psychol Personal Sci. 2015;6(2):157–63.

2. Rye MS, Cahoon MB, Ali RS, Daftary T. Development and validation of the counterfactual thinking for negative events scale. J Pers Assess. 2008;90(3):261–9.

**FIGURE 1:**
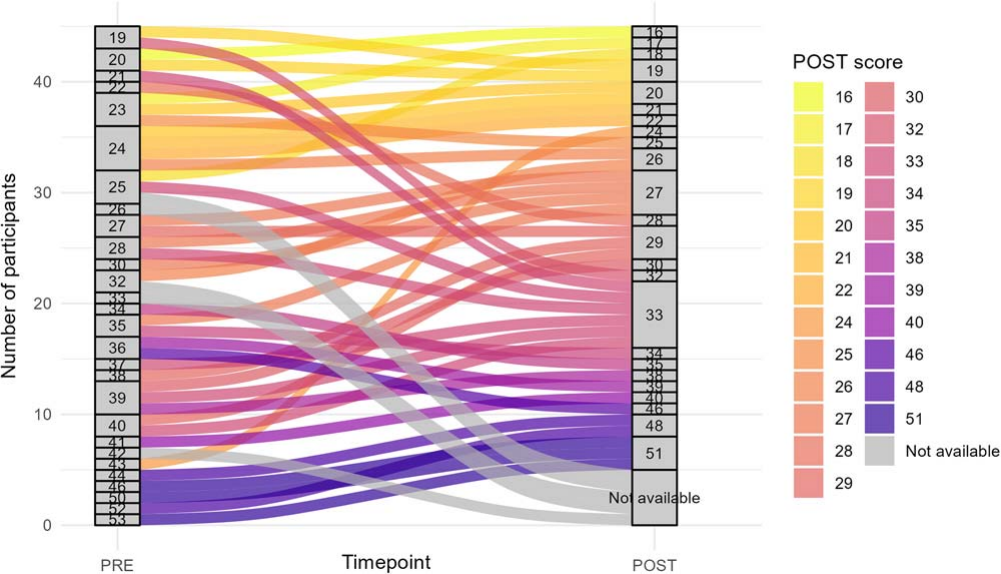
P252 | Change in total CTNES scores before and after participation in the ORBIT trial (*n* = 45 PRE treatment, *n* = 40 POST treatment, *n* = 5 not available).

### P253 Dynamics of Intact and Total HIV‐1 DNA‐Reservoirs in Relation to Plasma Viral Load Levels

David Mwangi, Malik Prentice, Hai‐Tam Lam, Robert Ehret, Martin Obermeier

Medical Center for Infectious Diseases, Berlin, Germany

**Background**: The intact proviral DNA assay (IPDA) is a high‐precision multiplex digital PCR (dPCR) method capable of discriminating between intact, hypermutated and 3′‐defective HIV‐1 proviruses. By enabling granular differentiation of the reservoir, IPDA offers potential for routine monitoring in people living with HIV and may support data‐driven therapeutic decisions. This study aimed to characterize reservoir dynamics by quantifying intact proviral loads and total HIV‐1 DNA across four viraemic strata ranging from suppression to high viral loads.

**Methods**: CD4+ T cells were isolated by immunomagnetic enrichment, followed by genomic DNA extraction. Quantification was performed by dPCR on the QIAcuityDx System (Qiagen), using a Triplex‐IPDA adapted from van Snippenberg et al. [1]. The assay targeted psi and env regions to identify intact proviruses, while the LTR region (RU5) quantified total HIV‐1 DNA, including intact and defective forms. Genomic integrity and cell equivalents were assessed using an RPP30 housekeeping gene assay targeting two cellular sequences equidistant to the HIV‐1 amplicons to monitor DNA shearing. Participants were stratified by plasma viral load (VL): G1 (<50 copies/mL), G2 (50–200 copies/mL), G3 (200–1000 copies/mL; target *N* = 50 due to limited availability) and G4 (> 1000 copies/mL), with 100 participants planned for the other groups.

**Results**: Testing was completed for 309 participants (G1: 87, G2: 93, G3: 40, G4: 96). Total HIV‐1 DNA differed significantly between viral load strata, with increasing proviral copies per 10^6 CD4+ T cells across ascending viraemic groups. Intact proviral DNA showed a comparable trend, with a significant increase in the viraemic group compared with the < 50 copies/mL group. These differences were observed despite substantial interindividual variability in both total and intact proviral DNA levels.

**Conclusions**: Quantification of total and intact proviral DNA provides new insight into HIV‐1 reservoir characteristics and dynamics. Significant differences between viraemic strata suggest potential utility as a diagnostic tool to identify individuals at risk of therapeutic failure. The observed variability highlights the need to integrate clinical data in future analyses. Since different IPDA variants are currently in use, an interlaboratory comparison within the Viromarkers consortium is planned to assess assay transferability, comparability and harmonization.

**Reference**

1. van Snippenberg W, Gleerup D, Rutsaert S, Vandekerckhove L, De Spiegelaere W, Trypsteen W. Triplex digital PCR assays for the quantification of intact proviral HIV‐1 DNA. Methods. 2022;201:41–48.

### P254 CD4/CD8 Ratio Recovery Versus Persistence of Inversion in Virologically Suppressed People With HIV: A 20‑Year Single‑Centre Cohort

Daniel Elbirt ^1,2^, Ramon Cohen^1^, Laliv Kadar^1^, Ilan Asher^1,2^, Shira Rosenberg‐Bezalel^1^, Keren Mahlab‐Guri^1,2^, Shay Nemet^1,2^

^1^Neve Or AIDS Center, Kaplan Medical Center, Rehovot, Israel; ^2^Faculty of Medicine, Hebrew University of Jerusalem, Jerusalem, Israel

**Background**: The CD4/CD8 ratio is an established marker of immune reconstitution in people with HIV (PWH), but many individuals fail to normalize this ratio despite long‑term viral suppression. Determinants of ratio normalization in routine clinical care remain incompletely defined.

**Methods**: This retrospective cohort included 301 adult PWH followed at a single HIV centre (Kaplan Medical Center, Israel) between 2004 and 2024, receiving combination ART with sustained viral suppression (HIV RNA < 30 copies/mL for ≥24 months). Longitudinal CD4, CD8 and CD4/CD8 ratios were abstracted from electronic records. Patients were classified according to final CD4/CD8 ratio: normalized (> 1) or persistently inverted (<1). Demographic, clinical and immunologic variables were compared using Mann–Whitney U and chi‑square tests (two‑sided *p* < 0.05).

**Results**: Median ART exposure was approximately 19 years; 168 patients (55.8%) were male and 133 (44.2%) female. Overall, 160 patients (53.2%) achieved CD4/CD8 ratio normalization, while 141 (46.8%) remained inverted. Female sex was associated with higher normalization rates than male sex (61.7% vs. 46.4%; *p* = 0.009). Patients who normalized were younger at HIV diagnosis (median 31 vs. 36 years; *p* = 0.002). Baseline CD4 count emerged as a critical determinant: among those starting ART with CD4 >350 cells/µL, 63.8% normalized the ratio compared with 47.4% of those with CD4 ≤350 cells/µL (*p* = 0.007). Final CD4 counts were substantially higher in the normalized versus inverted group (median 735.5 vs. 420 cells/µL; *p* < 0.001). There were no significant between‑group differences in AIDS history, comorbidities, baseline viral load or ART duration.

**Conclusions**: In this long‑term, virologically suppressed cohort, CD4/CD8 ratio normalization was achieved in just over half of patients and was strongly associated with female sex, younger age at diagnosis and baseline CD4 > 350 cells/µL. The 350‑cell threshold appears to mark an inflection point for immune recovery potential, supporting policies that prioritize early HIV diagnosis and immediate ART initiation (“test and treat”) to optimize long‑term immune reconstitution (Figure 1).

**FIGURE 1:**
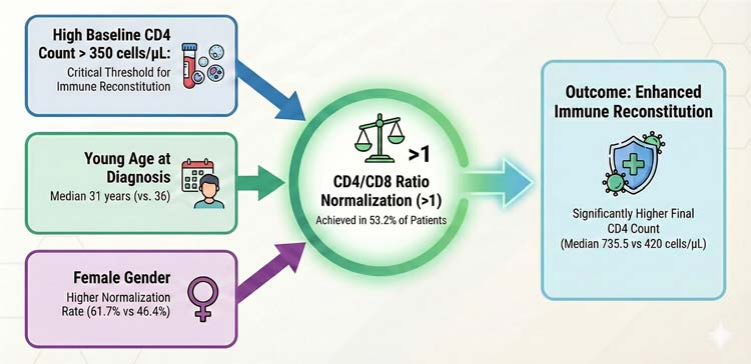
P254 | Factors promoting CD4/CD8 ratio normalization.

## Opportunistic Infections and AIDS‐Defining Cancers

### P255 Prevalent Cytomegalovirus (CMV) Disease Among ART‐Naïve People With HIV: Temporal Trends and Persistent Mortality in the ICONA Cohort, 1997–2024

Annalisa Mondi^1^, Nicolò Lentini^2^, Serena Vita^3^, Andrea Giacomelli^4,5^, Roberta Gagliardini^1^, Nicoletta Bobbio^6^, Giordano Madeddu^7^, Annalisa Saracino^8^, Elisabetta Teti^9^, Alessandra Rodanò^10^, Giulia C. Marchetti^11^, Cristina Mussini^12^, Antonella D'Arminio Monforte^10^, Andrea Antinori^1^, Miriam Lichtner ^3^, on behalf of ICONA Cohort Study Group

^1^Clinical and Research Infectious Diseases Department, National Institute for Infectious Diseases, Lazzaro Spallanzani IRCCS, Rome, Italy; ^2^Department of Life Sciences and Public Health, Section of Hygiene, Università Cattolica del Sacro Cuore, Rome, Italy; ^3^Department of Neurosciences, Mental Health and Sensory Organs (NESMOS) Sapienza University of Rome, Infectious Disease Unit, Saint Andrea Hospital, Rome, Italy; ^4^Department of Infectious Diseases, ASST Fatebenefratelli Sacco University Hospital, Milan, Italy; ^5^Department of Biomedical and Clinical Sciences, University of Milan, Milan, Italy; ^6^Department of Infectious Diseases, Galliera Hospital, Genoa, Italy; ^7^Unit of Infectious Disease, Department of Medicine, Surgery and Pharmacy, University of Sassari, Sassari, Italy; ^8^Clinic of Infectious Diseases, Department of Precision and Regenerative Medicine and Ionian Area, Polyclinic of Bari, University Hospital Polyclinic, University of Bari, Bari, Italy; ^9^Department of Systems Medicine, University of Rome Tor Vergata, Italy; Infectious Diseases Clinic, Policlinic of Tor Vergata, Rome, Italy; ^10^ICONA Foundation, Milan, Italy; ^11^Clinic of Infectious Diseases, ASST Santi Paolo e Carlo, Department of Health Sciences, University of Milan, Milan, Italy; ^12^Department of Infectious Diseases, Azienda Ospedaliero‐Universitaria di Modena, Policlinico di Modena, University of Modena and Reggio Emilia, Modena, Italy

**Background**: CMV remains a major challenge among people with HIV (PWH), yet data on CMV evolution across the antiretroviral therapy (ART) era remain limited. We investigated temporal trends, predictors and mortality associated with prevalent CMV disease (pCMV) in the ICONA Cohort over 30 years.

**Methods**: We included all ART‐naïve PWH enrolled in ICONA between 1997 and 2024. pCMV was defined as a first CMV manifestation diagnosed within 2 months of enrolment and before ART initiation. CMV manifestations were classified as end‐organ disease (EOD) or systemic infection (as reported by the treating physician and/or treated with anti‐CMV therapy). Temporal trends in prevalence were evaluated across three calendar periods (1997–2004, 2005–2014, 2015–2024) in the overall cohort and among PWH with very advanced HIV disease (vAHD; CD4 < 100 cells/mm^3^ and/or AIDS‐defining event). Predictors of pCMV among PWH with vAHD were assessed using multivariable logistic regression. The impact of pCMV on all‐cause and cause‐specific mortality was evaluated using Cox proportional hazards and competing‐risk models. Interaction tests assessed whether calendar year modified the association between pCMV and mortality.

**Results**: Among 22,039 PWH enrolled, 351 with pCMV were included (22% female, median age 45 years [IQR 38–53]): 166 had systemic infection and 185 EOD, including 13 with multiple‐organ involvement, accounting for 199 EOD localizations (49% retinitis, 25% gastrointestinal disease, 21% pneumonia and 5% encephalitis). CMV disease prevalence increased over time, from 0.83% (95% CI: 0.62%–1.10%) to 2.23% overall (95% CI: 1.95%–2.56%) and from 4.56% (95% CI: 3.42%–6.03%) to 9.13% (95% CI: 8.00%–10.40%) among PWH with vAHD, with similar trends for both EOD and systemic infection (Figure 1a,b). Among ART‐naïve PWH with vAHD, later calendar periods, higher HIV‐RNA and lower CD4 counts independently predicted a higher risk of pCMV (Figure 1c). pCMV was independently associated with increased all‐cause and AIDS‐related mortality in the overall cohort, whereas among PWH with vAHD, the association persisted only for AIDS‐related mortality. Interaction analyses showed no evidence that mortality risk differed across calendar years for either all‐cause or cause‐specific mortality (Table 1).

**Conclusions**: The burden of pCMV increased over time among ART‐naïve PWH, particularly those with vAHD at diagnosis, while its adverse impact on AIDS‐related mortality persisted despite advances in HIV care.

**FIGURE 1:**
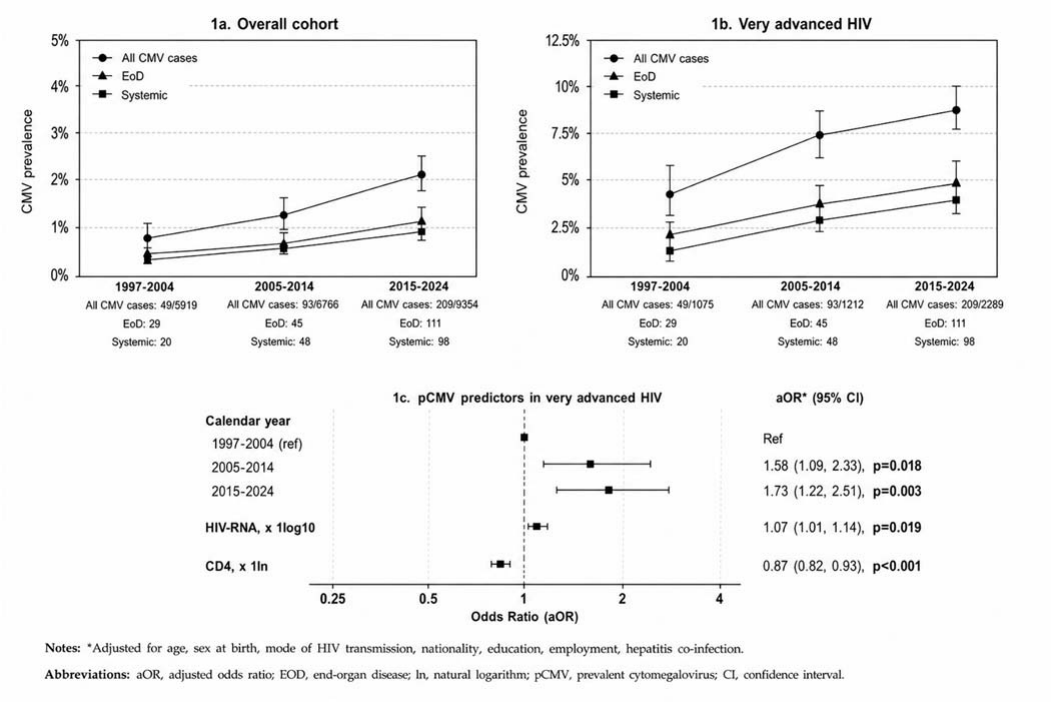
P255 | Temporal trends in CMV prevalence among ART‐naïve PWH overall (a) and among those with very advanced HIV disease (b), and predictors of prevalent CMV in ART‐naïve PWH with very advanced HIV disease (c).

**TABLE 1:**
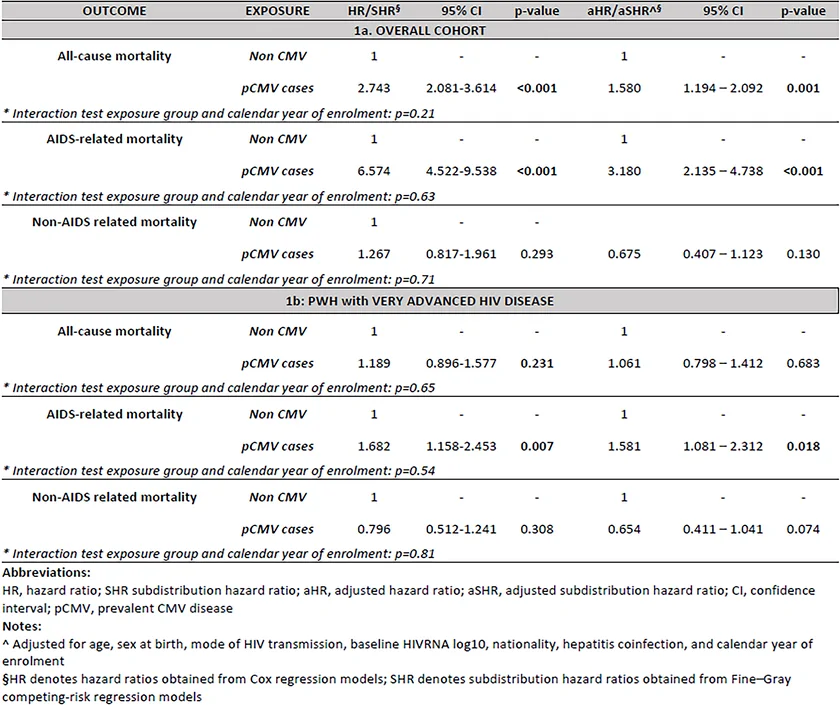
P255 | Association of prevalent CMV disease with all‐cause and cause‐specific mortality among ART‐naïve PWH overall cohort (a) and among those with very advanced HIV disease (b).

### P256 Abstract Withdrawn

### P257 Clinical and Immunological Characteristics of People Living With HIV Who Developed Immune Reconstitution Inflammatory Syndrome

Ana Salvador Rodríguez, Marianela Ciudad Sañudo

Department of Infectious Diseases, La Princesa University Hospital, Madrid, Spain

**Background**: Antiretroviral therapy (ART) has significantly improved outcomes for people living with HIV (PLWH). However, it may trigger immune reconstitution inflammatory syndrome (IRIS), particularly in individuals with advanced immunosuppression. IRIS represents a major clinical challenge due to its heterogeneous presentation and the wide range of pathogens involved. This study aimed to describe the demographic, clinical and immunological characteristics of a cohort of PLWH who developed IRIS, in order to improve risk stratification and clinical management.

**Methods**: We conducted a retrospective, observational, descriptive study including 633 ART‐naïve PLWH from the CoRIS Cohort followed at the Hospital Universitario La Princesa (2004–2023). Demographic, clinical and immunological variables were collected, including baseline and follow‐up CD4 cell counts and viral load. Associations with IRIS were assessed using chi‐square and Mann–Whitney U tests. Odds ratios (OR) with 95% confidence intervals (CI) were estimated; *p* < 0.05 was considered statistically significant.

**Results**: IRIS incidence was 5.1% (*n* = 32) and was significantly higher among individuals initiating ART between 2004 and 2014 compared with those initiating ART between 2015 and 2023 (7.6% vs. 2.9%; OR 2.7, 95% CI: 1.29–5.95; *p* = 0.007). A higher IRIS incidence was observed in women than in men (10% vs. 4.4%; OR 2.39, 95% CI: 0.99–5.75), although this difference did not reach statistical significance (*p* = 0.08). Individuals with IRIS had lower baseline CD4 cell counts (63.5 cells/µL; *p* < 0.0001) and higher viral load (117,500 vs. 78,164 copies/mL; *p* = 0.026) at ART initiation. A lower proportion of IRIS was observed among individuals receiving integrase inhibitor‐based regimens combined with nucleoside reverse transcriptase inhibitors (NRTIs) (OR 0.47, 95% CI: 0.22–0.98; *p* = 0.045) (Table 1). The most frequent opportunistic infections associated with IRIS were human herpesvirus 8‐associated Kaposi sarcoma, cytomegalovirus, tuberculosis and *Pneumocystis jirovecii*.

**Conclusions**: IRIS was more frequent in individuals with advanced immunosuppression. ART regimen selection may influence IRIS occurrence, with integrase inhibitor‐based therapies associated with a reduced incidence of IRIS compared with protease‐inhibitor‐based regimens. A higher frequency was observed in earlier periods characterized by more severe baseline immunosuppression. Early recognition remains essential for optimal clinical management.

**TABLE 1:**
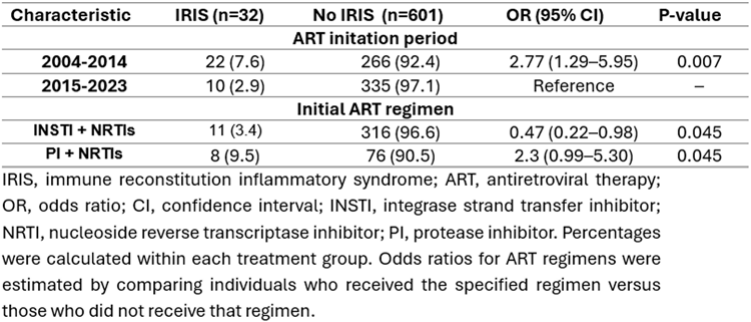
P257 | Immune reconstitution inflammatory syndrome according to ART initiation period and initial ART regimen.

### P258 Burden of Advanced HIV Disease and 1‐Year Outcomes Among People Re‐Engaging in HIV Care at a Public Hospital in Argentina

Ana Rodriguez, Victoria Pinto, Valentina Nuri Balbi Rocca, Eugenia Pia Sobrado, Lucia Nannini, Mariana Ariadna Paolini, Francisco Martin Ferreira Monteiro, Julian Vega, Veronica Azul Benseñor, Alicia Sisto, María José Rolón, Martín Jaume

Infectious Diseases Division, Hospital Fernández, Buenos Aires, Argentina

**Background**: Despite widespread availability of antiretroviral therapy (ART), disengagement from HIV care remains a major public health challenge. People living with HIV (PLWH) who return to care after prolonged periods of disengagement constitute a population at high risk of morbidity and mortality, for whom local data remain scarce. We aimed to describe the burden of disease and 1‐year outcomes among PLWH re‐engaging in care at a referral public hospital in Buenos Aires.

**Methods**: We conducted a retrospective cohort study including PLWH ≥16 years who re‐engaged in HIV care between March 2023 and December 2024 at Hospital Fernandez in Buenos Aires, Argentina. Re‐engagement was defined as returning to HIV medical care after interruption of ART for ≥6 months. Demographic, immunological and clinical characteristics at re‐entry were collected. WHO stage 3–4 events, hospitalizations and mortality during the first year of follow‐up were assessed using medical records and national HIV surveillance data. Descriptive and bivariable analyses were performed using Jamovi.

**Results**: 286 PLWH were included. At the time of re‐engagement, 55.9% (160) had advanced disease, with a median CD4 count of 184 cells/µL (IQR 72.5–356). Baseline demographic and immunological characteristics are summarized in Table 1. WHO stage 3–4 events were identified in 32.5% (93/286) of participants. Among affected individuals, 64.5% experienced one event, 23.7% two events and 11.8% three or more events. The distribution of these events is shown in Figure 1. During follow‐up, 24 additional WHO stage 3–4 events were diagnosed and 26.0% (74/286) of participants required hospitalization, of which 82.4% were HIV‐related. Overall mortality was 5.2% (15/286), while HIV‐attributable mortality was 3.1% (9/286). Mortality was associated with WHO stage 3–4 events (9.9% vs. 2.2%; OR 4.77, 95% CI: 1.4–15.9; *p* = 0.012), CD4 < 200 cells/µL (10.4% vs. 0%; *p* = 0.006) and older age (median 49 vs. 38 years; *p* = 0.002).

**Conclusions**: In our cohort, PLWH re‐engaging in care had a substantial burden of advanced HIV disease, opportunistic infections and hospitalization, with mortality disproportionately affecting those with severe immunosuppression. These findings underscore the significant clinical consequences of prolonged disengagement from care and highlight the need for proactive re‐engagement interventions, as well as the systematic implementation of advanced HIV disease care packages for individuals returning to care.

**FIGURE 1:**
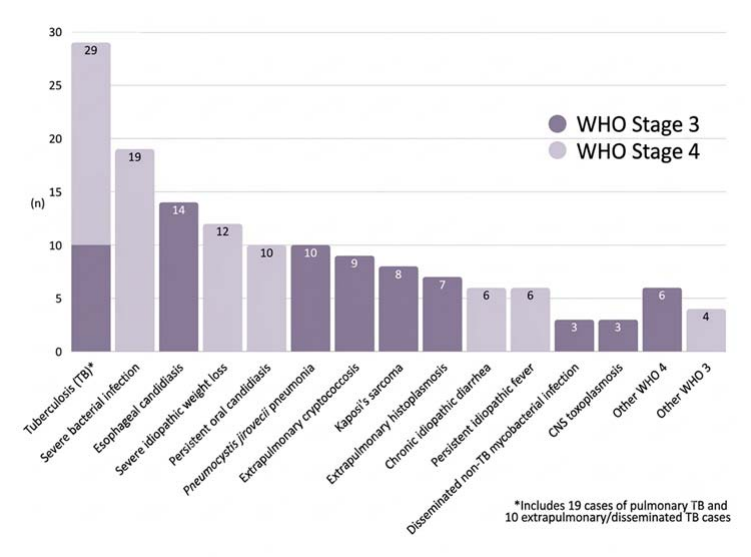
P258 | WHO stage 3–4 events.

**TABLE 1:**
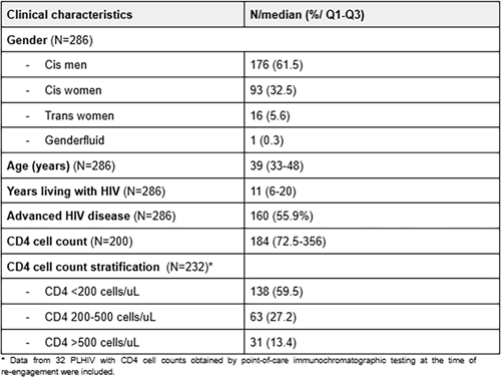
P258 | Baseline characteristics of the cohort.

### P259 Abstract Withdrawn

### P260 Lessons From the 90s: Sight Threatening CMV Retinitis as Part of an Immune Reconstitution Inflammatory Syndrome (IRIS) in a Patient Who Is Allergic to Valganciclovir

Emily Cullis^1^, Delia‐Mercedes Bianco^1^, Kala Gopalakrishnan^2^, Lorraine Cullen^3^, Kate Adams^1^, Mariya Molai ^1^

^1^Department of Infection, Hull University Teaching Hospitals NHS Trust, Kingston upon Hull, UK; ^2^Department of Ophthalmology, Hull University Teaching Hospitals NHS Trust, Kingston upon Hull, UK; ^3^Pharmacy Department, Hull University Teaching Hospitals NHS Trust, Kingston upon Hull, UK

**Background**: Cytomegalovirus (CMV) retinitis was once a common sight threatening opportunistic infection in advanced HIV. Fortunately, it has become increasingly rare over the last two decades due to earlier HIV diagnosis and widespread use of early antiretrovirals (ARVs). As such many HIV doctors may never have seen or treated a patient with CMV retinitis.

**Case Description**: We present a case of rapid progression from asymptomatic CMV viraemia to sight‐threatening retinitis as part of an IRIS in a 40‐year‐old man diagnosed with advanced HIV (CD4 count 9 cells/µL, HIV viral load 1,395,491 copies/mL). He was treated for *Pneumocystis jirovecii* pneumonia (PJP) and started on ARVs. His CMV PCR was 20,359 IU/mL but as he was asymptomatic with no signs of end organ damage, no CMV active antiviral was started. He was reviewed in clinic shortly after discharge and 2 weeks after starting ARVs and complained of blurred vision and floaters in his right eye. His vision was counting fingers only in the right eye and 6/9 in the left. He was urgently reviewed by ophthalmology and florid CMV retinitis was confirmed in the right eye with early changes in the left eye as well. He was commenced on oral valganciclovir.

**Outcomes**: Unfortunately, 10 days after starting the valganciclovir he developed a drug reaction. Initially the prophylactic co‐trimoxazole was stopped, but when this failed to settle the rash, the valganciclovir was also stopped with rapid improvement. After a short pause to let things fully resolve, he was commenced on IV cidofovir every 2 weeks and has now been maintained on this for 2 months. He also received 3 doses of intravitreal foscarnet into the right eye to bridge between stopping the valganciclovir and starting the cidofovir. His vision has improved in the right eye to 6/24 and remains 6/9 in the left eye.

**Conclusions**: This case highlights the risk of rapid progression from asymptomatic CMV viraemia to sight‐threatening retinitis as part of an IRIS in advanced HIV. Pre‐emptive ophthalmic assessment in cases with significant viraemia should be considered, even in the absence of symptoms. It also highlights the role of biweekly IV cidofovir as maintenance therapy when valganciclovir cannot be tolerated.

### P261 Neglected Killers, Shared Solutions: Talaromycosis in Advanced HIV Disease and Invasive Fungal Infections in Diabetic Patients—A Case Series and the Case for Integrated Fungal Diagnostic and Treatment Access in Resource‐Limited Indonesia

Yusuf Aulia Rahman ^1,2^, Lina Herliyana^3^, Otta Nur Kirana^4^, Muhammad Labib M. Y. Bima^5^, Lukman Pura^6^

^1^Infectious Diseases Division, Internal Medicine Department, Abdul Moeloek General Hospital, Bandar Lampung, Indonesia; ^2^Infectious Diseases Division, Internal Medicine Department, Universitas Lampung School of Medicine, Bandar Lampung, Indonesia; ^3^Clinical Microbiology Department, Abdul Moeloek General Hospital, Bandar Lampung, Indonesia; ^4^HIV/STIs Program, Lampung Provincial Health Office, Bandar Lampung, Indonesia; ^5^Universitas Lampung School of Medicine, Bandar Lampung, Indonesia; ^6^Indonesian Society of Internal Medicine Specialists, Bandar Lampung, Indonesia

**Background**: Invasive fungal infections (IFIs) account for an estimated 20–25% of AIDS‐related mortality globally yet remain inaccessible in most resource‐limited settings, a preventable gap towards zero AIDS‐related deaths by 2030. In Indonesia—a high HIV‐burden, neglected tropical disease (NTD)‐endemic country—no national guideline or procurement framework for IFI management in advanced HIV disease (AHD) exists. We propose that integrating IFI diagnostic and treatment access across HIV and NTD platforms can overcome access barriers, formalize informal cross‐programme drug utilization and generate combined demand to reduce antifungal costs.

**Methods**: We present five IFI cases from a resource‐limited district general hospital in Lampung, Indonesia. Three occurred in PLWH with AHD: talaromycosis with disseminated cutaneous, pulmonary and sinonasal presentations. Two occurred in diabetic non‐PLWH patients: sino‐orbital mucormycosis and lymphogenic fusariosis. Symptom onset to diagnosis, treatment access and 30‐day outcomes were documented.

**Results**: All three talaromycosis cases were diagnosed by smear morphology and fungal culture, with a uniform 56‐day diagnostic delay attributable to limited IFI suspicion at primary care, empirical treatment for alternative diagnoses and culture turnaround—reflecting failures of awareness, point‐of‐care access and laboratory infrastructure. No *Talaromyces marneffei* lateral flow assay was available nationally. All three had no recent travel to known higher‐endemicity regions, suggesting underrecognized local endemicity in Sumatra. All PLWH patients received amphotericin B induction followed by itraconazole consolidation and survived to 30 days with reversible toxicity. Both non‐PLWH cases had a 28‐day diagnostic delay. Amphotericin B, available solely through HIV programme procurement, was the only accessible treatment as no formal antifungal pathway existed outside the HIV programme. Both died on day 15 despite treatment.

**Conclusions**: Cascading diagnostic delays, absent point‐of‐care diagnostics and antifungal access informally concentrated within HIV programme infrastructure illustrate systemic fragility of IFI management in Indonesia. Three talaromycosis cases without travel history suggest underrecognized *T. marneffei* endemicity in Sumatra warranting surveillance. Integrating IFI access across HIV and NTD platforms would address all delay components—expanding awareness through joint training, rapid diagnosis through NTD‐model POCT and cost reduction through combined procurement. We call for national dialogue on integrated IFI guidelines and prospective surveillance towards Indonesia's 2030 zero AIDS‐related death target.

### P262 The Impact of R‐CHOP‐Based Immunochemotherapy on HIV Tropism, Quasi‐Species and Viral Reservoir in Patients With AIDS‐Related Lymphoma

Biao Zhu, Ye Xiong, Xiaorong Peng

The Department of Infectious Diseases, State Key Laboratory for Diagnosis and Treatment of Infectious Diseases, National Clinical Research Center for Infectious Diseases, Collaborative Innovation Center for Diagnosis and Treatment of Infectious Diseases, The First Affiliated Hospital, School of Medicine, Zhejiang University, Hangzhou, China

**Background**: R‐CHOP‐based immunochemotherapy is highly effective for AIDS‐related lymphoma (ARL), but its specific effects on HIV virological tropism, quasi‐species dynamics and the viral reservoir remain unclear. Current functional cure strategies, including bone marrow transplantation and gene editing, predominantly target the CCR5 receptor. However, viral tropism distribution in patients with both haematological malignancy and HIV infection is unknown, which is critical for guiding personalized functional cure approaches.

**Methods**: This prospective study enrolled 39 treatment‐naïve ARL patients between January 2014 and December 2024. The cohort had a mean age of 48±14.04 years, 51.28% diagnosed with diffuse large B‐cell lymphoma, 25.64% with Burkitt lymphoma and 92.31% presenting with stage III/IV disease. We performed HIV tropism analysis via env V3 region sequencing and comprehensively evaluated changes in the viral reservoir, quasi‐species diversity, phylogenetic relationships and evolutionary dynamics before and after immunochemotherapy.

**Results**: Sequence analysis revealed 74.36% of patients harboured R5‐tropic viruses and 25.64% carried X4‐tropic viruses. Longitudinal analysis of 12 paired baseline and end‐of‐treatment samples showed stable tropism in 11 patients, with only one switching from R5 to X4 (Table 1). Combined immunochemotherapy and cART significantly reduced total HIV DNA (*p* = 0.022) and cell‐associated HIV RNA (*p* = 0.0019) (Figure 1). Phylogenetic analysis demonstrated complex viral evolution, with sequences from different time points intermingling or forming distinct clusters and baseline quasi‐species disappearing while new variants emerged post‐treatment.

**Conclusions**: Combined therapy effectively reduces the HIV reservoir, but induces genetic variation and quasi‐species dynamics. The presence of CXCR4‐tropic viruses in a quarter of ARL patients may compromise CCR5‐targeted functional cure strategies. These findings highlight the need to optimize anti‐HIV treatment regimens and develop personalized functional cure approaches for this vulnerable population.

**FIGURE 1:**
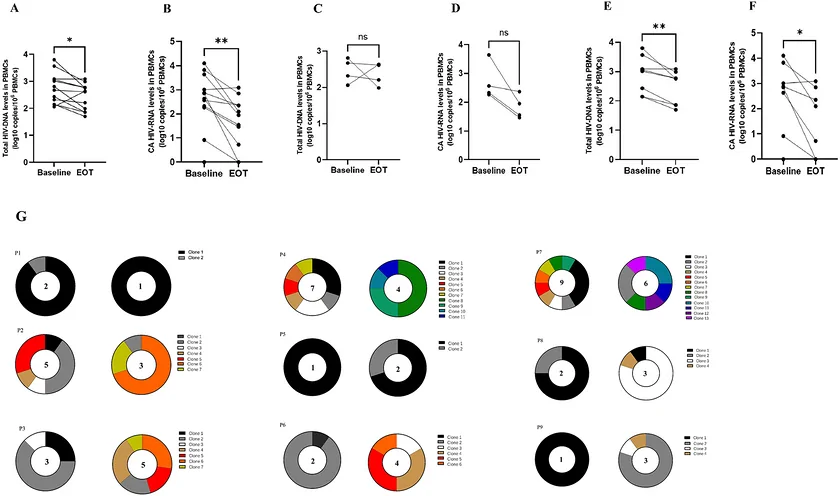
P262 | Change of HIV tropism, quasi‐species and viral reservoir in patients with AIDS‐related lymphoma. Panels A and B show the overall changes in total HIV DNA and CA‐HIV RNA levels across all 12 patients during the treatment period. To explore the potential influence of prior cART exposure, we stratified the patients into two subgroups. Panels C and D present the viral reservoir changes in 4 patients who had received cART for more than 6 months before ARL diagnosis, while panels E and F show the corresponding changes in 8 patients who had received cART for less than 6 months prior to diagnosis. Statistical significance is indicated by asterisks in the figure, where * represents *p* < 0.05 and ** represents *p* < 0.01. Panel G shows the composition and distribution of viral quasispecies at baseline and EOT in 9 patients, with each colour representing a distinct quasispecies. It is important to note that within the same patient, the same colour at baseline and EOT represents the identical quasispecies, but identical colours across different patients do not indicate the same quasispecies. For each patient, the left panel displays the baseline quasispecies composition, and the right panel shows the composition at EOT. The number in the circle of picture represents the detectable quantity of clones.

**TABLE 1:**
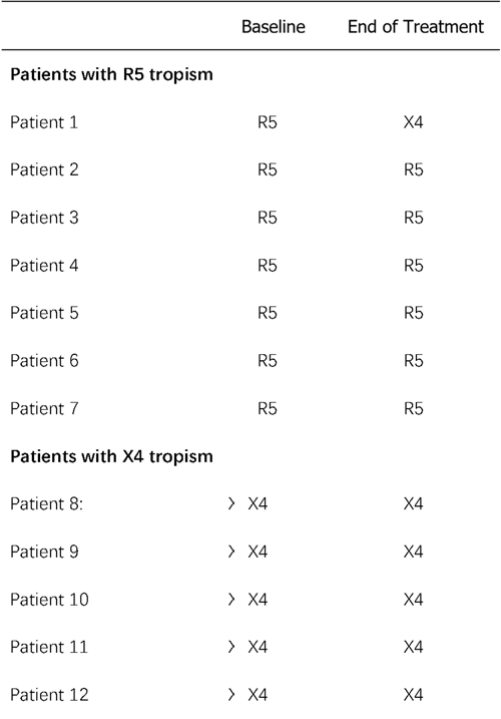
P262 | Viral tropism at baseline and end of treatment.

## Clinical Pharmacology

### P263 Transient Immediate Systemic Side Effects After Long‐Acting Cabotegravir/Rilpivirine Injections: A 5‐Year Cohort Study

Laurent Hocqueloux ^1^, Daniela Pires Roteia^1^, Sandra Pallay^1^, Aurélie Theillay^1^, Guillaume Beraud^1^, Julie Bois^2^, Thierry Prazuck^1^

^1^Infectious Diseases, CHU d'Orléans, Orléans, France; ^2^Biology Lab, CHU d'Orléans, Orléans, France

**Background**: Long‐acting cabotegravir/rilpivirine (CAB/RPV‐LA) is generally well tolerated, but immediate systemic reactions occurring during or shortly after injection are poorly characterized. We aimed to describe the incidence, clinical pattern, consequences and pharmacokinetic mechanism of transient immediate systemic side effects (TISSE) after CAB/RPV‐LA injections in people with HIV.

**Methods**: We conducted a prospective, single‐centre, real‐world cohort study at the Infectious Diseases Department, CHU d'Orléans, France. Adults receiving at least one intramuscular CAB/RPV‐LA injection session between 2021 and 2026 were included. Injection sessions administered on site were performed by dedicated nurses, with real‐time documentation of unexpected immediate events in the electronic medical record. Cabotegravir and rilpivirine plasma concentrations were measured 5–10 min after selected TISSE episodes and compared with post‐injection controls and routine trough concentrations.

**Results**: Among 308 participants, 5464 CAB/RPV‐LA injection sessions were recorded, including 4644 administered and monitored on site. Overall, 24 participants experienced 28 TISSE episodes, corresponding to an incidence of 0.60% per on‐site injection session (approximately 1 per 166 sessions; 95% CI, 1 per 115–250). TISSE occurred during injection or shortly afterwards, after a median of 2 min (IQR, 1–3) and resolved within a median of 10 min (IQR, 6–10). The most frequent manifestations were intense lumbar pain (18 episodes), dyspnoea or chest oppression (16), red eyes (13), headache (11) and transient blood pressure elevation (9). No episode required hospitalization. Treatment persistence was not affected (2/24 discontinuations among participants with TISSE vs. 38/284 without TISSE; *p* = 0.75). In all 17 episodes with immediate pharmacokinetic sampling, rilpivirine and/or cabotegravir concentrations were markedly elevated: rilpivirine alone in 13/17, cabotegravir alone in 3/17 and both drugs in 1/17.

**Conclusions**: TISSE is an uncommon but clinically striking, rapidly reversible and benign immediate reaction after CAB/RPV‐LA injection. Markedly elevated immediate plasma concentrations support accidental systemic passage of rilpivirine and/or cabotegravir. Recognizing this syndrome may prevent unnecessary emergency investigations and inappropriate discontinuation of long‐acting antiretroviral therapy.

### P264 Body Weight Changes, Rilpivirine and Cabotegravir Trough Concentrations in People Living With HIV Receiving Long‐Acting Injectable Antiretroviral Therapy in Southern Taiwan

Hung‐Chin Tsai ^1^, Pei‐Yun Chou^1^, Hui‐Min Chang^2^

^1^Division of Infectious Diseases, Kaohsiung Veterans General Hospital, Kaohsiung, Taiwan; ^2^Department of Pharmacy, Pingtung Veterans General Hospital, Pingtung, Taiwan

**Background**: Data regarding the pharmacokinetic variability of long‐acting injectable (LAI) rilpivirine (RPV) and cabotegravir (CAB) remain limited in Asian populations. This study evaluated the inter‐individual variability of RPV and CAB plasma trough concentrations and assessed the influence of demographic factors and body weight changes in people living with HIV (PLWH) receiving LAI therapy in southern Taiwan.

**Methods**: This prospective observational study enrolled PLWH treated with LAI RPV and CAB for up to 76 weeks, with serial plasma trough concentrations assessed before each LAI administration. Drug concentrations were quantified using LC‐MS/MS. Associations between drug levels, clinical variables and body weight changes were analysed.

**Results**: Twenty‐five PLWH were included (median 40 years old, IQR 34–47). There was 159 CAB and 158 RPV concentrations available for analysis. RPV and CAB showed moderate inter‐individual variability in trough concentrations, with 27–30% and 15–50% of patients below the designed Q1 < 1387 ng/mL for CAB and Q1 < 47.68 ng/mL for RPV in the first 28 weeks, respectively (Figure 1). All of the 25 PLWH maintained the plasma viral load < 20 copies/mL. The median (IQR) body weight immediately before switching was 74.9 (67.4–79.8) kg. There was no significant change in body weight during treatment. There was also no association between the drug concentrations, body weight changes and demographic factors.

**Conclusions**: Inter‐individual variability in RPV and CAB plasma trough concentrations is not uncommon among Asian PLWH receiving LAI therapy; however, this variability did not impact virological control. Drug concentrations were not associated with body weight changes or demographic factors.

**FIGURE 1:**
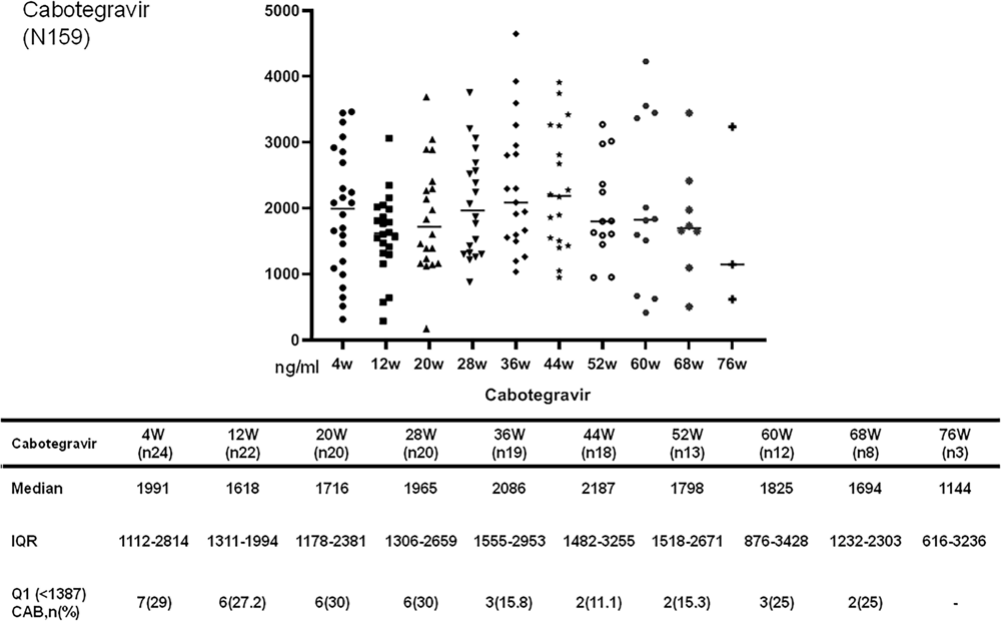
P264 | Serial cabotegravir trough concentration among 25 PLWH receiving LAI.

### P265 Phase 1 Study to Investigate the Absorption, Metabolism and Excretion of Ulonivirine, a Non‐Nucleoside Reverse Transcriptase Inhibitor, in Adults Without HIV

Gillian Gillespie ^1^, Michelle M. Pham^1^, Makeda Robinson^1^, Marissa Vavrek^1^, Yuexia Liang^1^, Yiran Huang^1^, Kimberly Thomas^1^, Yang Liu^1^, Jon Meiman^2^, Jesse C. Nussbaum^1^

^1^Merck & Co., Inc., Rahway, NJ, US; ^2^Fortrea, Madison, WI, US

**Background**: Ulonivirine (ULO; also known as MK‐8507) is an investigational non‐nucleoside reverse transcriptase inhibitor (NNRTI) in development as a once‐weekly treatment for HIV‐1, in combination with islatravir. A phase 1 study (MK‐8507‐006; NCT07334886) evaluated the absorption, metabolism and excretion of ULO.

**Methods**: This was a nonrandomized, single‐site, open‐label 1‐period study that enrolled 8 adults assigned male sex at birth without HIV. Fasted participants received a single 200 mg (∼200‐µCi) oral dose of [^14^C] ULO. Total radioactivity and metabolite profiles were assessed in plasma, urine and faeces and ULO concentrations were measured in plasma. Safety was assessed throughout by evaluation of adverse events (AEs), physical examination, vital signs, electrocardiogram and clinical laboratory tests. Participants were domiciled at the study site until discharge criteria were met (including recovery of ≥90% of administered radioactivity); time to discharge could range from 16 to 35 days.

**Results**: Eight participants were enrolled; all completed the study and were included in the analyses. Total radioactive recovery through 27 days was 93.3%; 38.0% (cumulative) of radioactive dose was excreted in urine and 55.2% in faeces. In plasma, ULO accounted for approximately 70% of total radioactivity in area under the concentration‐time curve from 0 to last measurement (AUC_0‑last_) and ∼90% of total radioactivity at maximum concentration (C_max_) (Table 1). The median time to ULO C_max_ in plasma was 4.00 h, with a geometric mean half‐life of 75.5 h. ULO was cleared primarily through metabolism by glutathione S‐transferases, with a minimal role for uridine diphosphate glucuronosyltransferase glucuronidation (<3%) and cytochrome P450 metabolism (<6%). Approximately 8% of the administered dose of ULO was excreted unchanged in the urine and ∼36% of the dose was excreted as unchanged ULO in faeces. Four participants (50.0%) reported AEs (all mild); AEs were considered treatment‐related by the investigator in 2 participants (both diarrhoea, at ∼2 h post‐dose, lasting 1–2 min).

**Conclusions**: ULO was the predominant drug‐related material representing ∼90% of the total plasma radioactivity at C_max_. The major clearance pathway of ULO was metabolism via glutathione S‐transferases, with direct urinary excretion playing a minor role. Unchanged ULO recovered in faeces (∼36% of administrated dose) was consistent with moderate absorption.

**TABLE 1:**
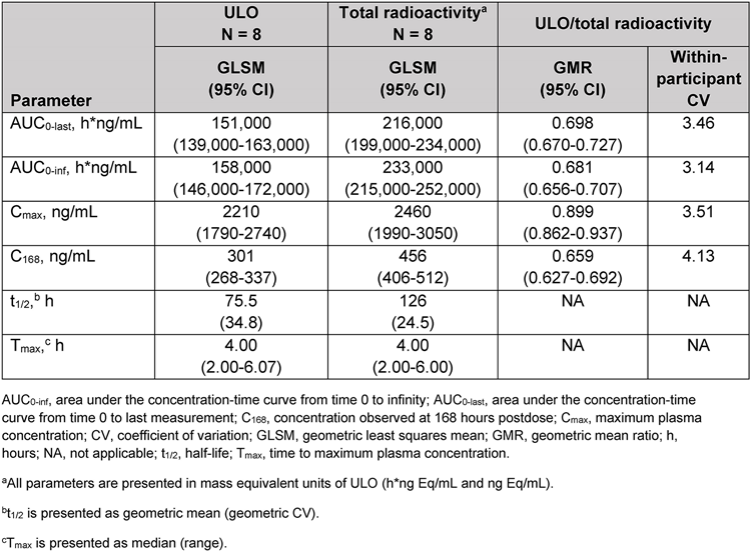
P265 | Plasma pharmacokinetics of ULO and total radioactivity.

### P266 Population Pharmacokinetic Modelling of Plasma and Intracellular Bictegravir in People Living With HIV

Emmanuel Niyigena^1,2^, Julien De Greef ^3,4^, Nadtha Panin^4^, Jean Cyr Yombi^3^, Erwin Dreesen^2^, Leïla Belkhir^3,4^, Vincent Haufroid^4,5^, Laure Elens^1^

^1^Faculty of Pharmacy and Biomedical Sciences, UCLouvain, Brussels, Belgium; ^2^Department of Pharmaceutical and Pharmacological Sciences, KU Leuven, Leuven, Belgium; ^3^Service de Médecine interne et Maladies Infectieuses, HIV/AIDS reference center, Cliniques universitaires Saint‐Luc, Brussels, Belgium; ^4^Louvain centre for Toxicology and Applied Pharmacology (LTAP), Institut de Recherche Expérimentale et Clinique, UCLouvain, Brussels, Belgium; ^5^Department of Clinical Chemistry, Cliniques Universitaires Saint‐Luc, Brussels, Belgium

**Background**: Bictegravir is a second‐generation human immunodeficiency virus (HIV) integrase strand‐transfer inhibitor (INSTI) widely used in first‐line antiretroviral therapy, yet interindividual variability in exposure and response persists [1–3]. Its plasma pharmacokinetics have been described [4], but intracellular exposure in peripheral blood mononuclear cells (PBMCs), where bictegravir inhibits HIV integrase, remains poorly characterized. This study aimed to develop a bictegravir population pharmacokinetic (popPK) model linking plasma and PBMC concentrations to quantify intracellular exposure variability and support future exposure–response analyses.

**Methods**: A secondary pharmacometric analysis was conducted in bictegravir‐treated adults living with HIV using prospectively collected plasma and PBMC concentration data. Bictegravir pharmacokinetics were characterized using nonlinear mixed‐effects modelling, linking plasma concentrations to intracellular PBMC exposure. Demographic and pharmacogenetic covariates were explored as sources of interindividual variability. Model‐based simulations were used to explore how variability in the plasma‐to‐intracellular relationship may influence intracellular exposure.

**Results**: Fifty‐one participants, 39% female, contributed 138 plasma and 42 PBMC bictegravir concentrations. Median (interquartile range) age and body weight were 44 years (36–52) and 81 kg (69–92) (Table 1). Plasma concentrations were described by a one‐compartment model with first‐order absorption and linear elimination. Typical apparent clearance and volume of distribution were 425 mL/h and 10,600 mL, with absorption rate fixed at 2.60 h^−1^. PBMC concentrations were best linked to plasma using an effect‐compartment model, yielding a steady‐state intracellular‐to‐plasma concentration ratio of 0.038 associated with high interindividual variability (89%). No demographic or pharmacogenetic covariate was retained in the final model. After adjustment to steady‐state trough values, no plasma concentration was below the suggested trough threshold of 760 ng/mL [4], while 7% of PBMC concentrations fell below the corresponding model‐derived intracellular threshold of 29 ng/mL (Figure 1). Monte Carlo simulations showed that the proportion of intracellular concentrations below 29 ng/mL decreased with increasing plasma trough concentrations, from 46% at 760 ng/mL to less than 5% above 3000 ng/mL.

**Conclusions**: This study provides the first model‐based link between bictegravir plasma and PBMC concentrations using real‐life TDM data, showing high variability in the plasma‐to‐intracellular relationship. No demographic or pharmacogenetic predictors of exposure variability were identified. This framework supports future intracellular exposure–response analyses.

**References**

1. De Clercq E, Zhang Z, Huang J, Zhang M, Li G. Biktarvy for the treatment of HIV infection: Progress and prospects. Biochemical Pharmacology. 2023;217:115862.

2. Chen GJ, Sun HY, Chen LY, Hsieh SM, Sheng WH, Liu WD, et al. Low‐level viremia and virologic failure among people living with HIV who received maintenance therapy with co‐formulated bictegravir, emtricitabine and tenofovir alafenamide versus dolutegravir‐based regimens. Int J Antimicrob Agents. 2022:106631.

3. Nasreddine R, Florence E, Yombi JC, Henrard S, Darcis G, Van Praet J, et al. Efficacy, durability and tolerability of bictegravir/emtricitabine/tenofovir alafenamide for the treatment of HIV in a real‐world setting in Belgium. HIV Med. 2023;24(8):914–24.

4. Ekobena P, Briki M, Dao K, Marzolini C andre P, Buclin T, et al. Population pharmacokinetics of bictegravir in real‐world people with HIV. J Antimicrob Chemother. 2025;80(10):2782–9.

**FIGURE 1:**
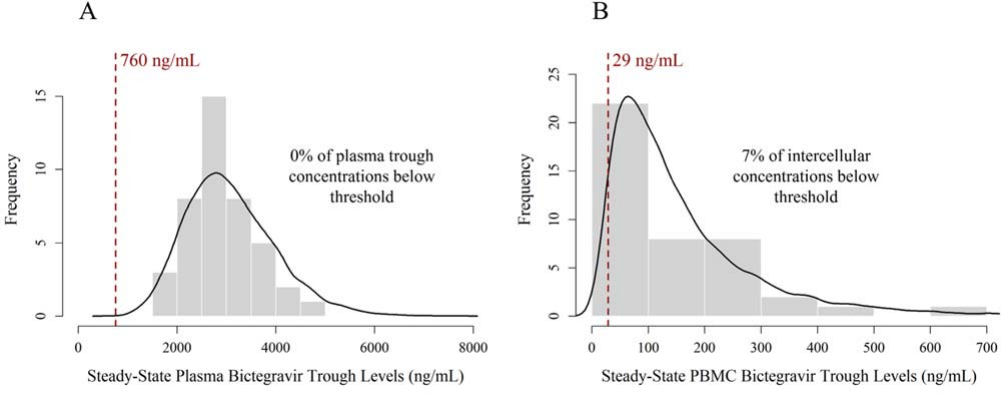
P266 | Observed bictegravir concentrations adjusted to steady‐state trough values in plasma and PBMCs. Histograms show observed bictegravir concentrations in plasma (A) and peripheral blood mononuclear cells (PBMCs) (B) adjusted to their corresponding model‐derived steady‐state trough concentrations using individual empirical Bayes estimates, thereby accounting for the irregular sampling design. Black curves represent kernel density estimates. Red dashed vertical lines indicate the plasma trough threshold of 760 ng/mL and the corresponding PBMC threshold of 29 ng/mL derived using the final model. In the original dataset, 0% of plasma trough concentrations were below the plasma threshold, whereas 7% of PBMC trough concentrations were below the intracellular threshold.

**TABLE 1:**
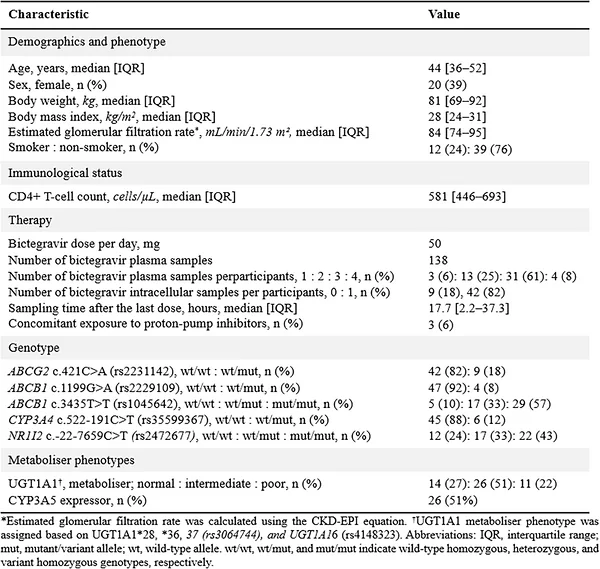
P266 | Baseline characteristics of the study population (*N* = 51).

### P267 Characterization of the Clinical Drug–Drug Interaction Profile of VH4524184 (VH‐184), a Third‐Generation Integrase Strand Transfer Inhibitor

Eileen Castronova^1^, Jon Collins ^2^, Fiona Halliday^3^, Alex Ballesteros‐Perez^4^, Riya Moodley^5^, Luise Rogg^2^, Jorge Villacian^3^, Hyunmoon Back^1^

^1^GSK, Collegeville, PA, US; ^2^ViiV Healthcare, Durham, NC, US; ^3^GSK, London, UK; ^4^Certara, Radnor, PA, US; ^5^ViiV Healthcare, London, UK

**Background**: VH4524184 (VH‐184) is a third‐generation integrase strand transfer inhibitor (INSTI) with an enhanced resistance profile, potent antiviral activity and potential for twice‐yearly dosing. We assessed the drug–drug interaction profile of VH‐184 using cytochrome P450 3A4 (CYP3A4) inhibitors/inducers and the transporter probe substrates metformin (organic cation transporter 1 [OCT1]/multidrug and toxin extrusion protein 1/2‐kidney [MATE1/2K]) and digoxin (P‐glycoprotein [P‐gp]).

**Methods**: We conducted a phase 1, open‐label study in adults without HIV (NCT07066722). To assess VH‐184 as an object, a single dose of oral VH‐184 100 mg was administered before and after steady‐state dosing of once‐daily itraconazole 200 mg, once‐daily rifabutin 300 mg, or 3‐times–daily phenytoin 100 mg. To assess VH‐184 as a precipitant, single doses of metformin 500 mg and digoxin 0.25 mg were administered alone and with steady‐state oral VH‐184 200 mg. Plasma concentration–time data were analysed using non‐compartmental methods. Geometric mean ratios (90% CIs) of maximum plasma concentration (C_max_), area under the concentration–time curve (AUC) from time 0 to end of dosing interval (AUC_0‐t_) and AUC from time 0 to infinity (AUC_0‐inf_; CYP3A4 only) were calculated for VH‐184 with versus without CYP3A4 modulators and for transporter substrates with versus without VH‐184. The primary endpoint was C_max_ and AUC_0‐t_ for VH‐184, metformin and digoxin; safety was a secondary endpoint.

**Results**: We enrolled 61 participants (male sex, 90% [55/61]; median age, 33 years). VH‐184 exposure was increased by the strong CYP3A4 inhibitor itraconazole and decreased by the moderate and strong CYP3A4 inducers rifabutin and phenytoin, respectively (see Table 1 and Figure 1). VH‐184 did not affect metformin or digoxin exposure. There were no VH‐184‐related grade ≥3 adverse events (AEs), serious AEs, or deaths. Thirty‐one participants (51%) discontinued treatment, mostly due to AEs from CYP3A4 modulators.

**Conclusions**: VH‐184 exposure was affected by CYP3A4 inhibitors and inducers, consistent with some other INSTIs. VH‐184 had no impact on OCT1/MATE1/2K or P‐gp substrates, demonstrating a favourable precipitant profile, potentially broadening its compatibility with co‐administered medications. VH‐184 was generally well tolerated with a favourable safety profile. The clinical impact of CYP3A4 inhibitors/inducers on VH‐184 exposure will be determined after VH‐184 dose selection.

**FIGURE 1:**
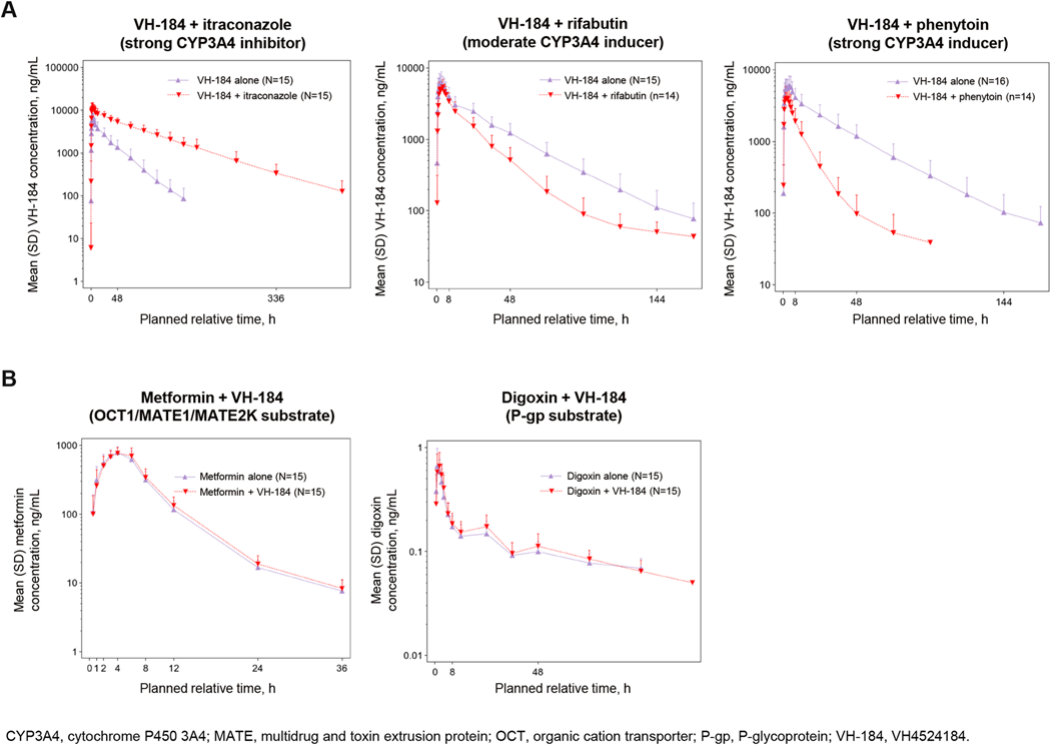
P267 | (A) Concentrations of VH‐184 as an object with and without CYP3A4 inhibitor or inducers. (B) Concentrations of metformin and digoxin with and without VH‐184 as a precipitant.

**TABLE 1:**
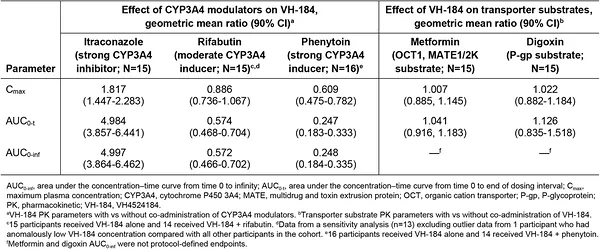
P267 | PK parameters: effect of CYP3A4 modulators on VH‐184 and effect of VH‐184 on transporter substrates metformin and digoxin.

### P268 Phase 1 Study to Evaluate the Effect of a Single Supratherapeutic Dose of MK‐8527 on QTc Interval Prolongation in Healthy Adults

Russ P. Carstens ^1^, Yash Kapoor^1^, Arinjita Bhattacharyya^1^, Bhavna Kantesaria^1^, Graigory C. Garrett^1^, Peter Addo^1^, Ryan Vargo^1^, Michael Hassman^2^, Jesse C. Nussbaum^1^

^1^Merck & Co., Inc., Rahway, NJ, US; ^2^CenExcel Clinical Research, Marlton, NJ, US

**Background**: MK‐8527 is a nucleoside reverse transcriptase translocation inhibitor (NRTTI) in clinical development as pre‐exposure prophylaxis (PrEP) for the prevention of HIV‐1. MK‐8527 11 mg oral once‐monthly is currently being evaluated in phase 3 studies. MK‐8527 had no effect on Fridericia's corrected QT interval (QTcF) or other electrocardiogram (ECG) intervals in previous preclinical and phase 1 studies. This phase 1 study (MK‐8527‐014, NCT07063238) evaluated the effect of a single supratherapeutic dose of MK‐8527 on QTc intervals in healthy adults.

**Methods**: This was a randomized, double‐blind, active‐controlled and placebo‐controlled, 3‐way crossover study. Fasted participants were randomized to 1 of 6 treatment sequences and received either blinded MK‐8527 (66 mg) or matching placebo or open‐label moxifloxacin (400 mg, active control) in each of 3 periods with a ≥28‐day washout between the dose in each period. Holter recordings were obtained from ≥60 min before dosing to 24 h after dosing. Blood was collected for pharmacokinetic analysis through 168 h after dosing. A linear mixed‐effects model was used to estimate mean difference in QTcF change from baseline between MK‐8527 or moxifloxacin and placebo (ΔΔQTcF). The dependent variable was QTcF and the independent variables were treatment, sequence, period, time and treatment‐by‐time interaction as fixed effects; baseline QTcF was a covariate. Mean (90% CI) ΔΔQTcF ≥10 ms was considered clinically significant. Safety was assessed throughout the study by physical examination, ECG, vital signs and laboratory tests.

**Results**: A total of 42 participants were enrolled, 7 in each treatment sequence and 38 (90.5%) participants completed the study. Participants were mostly male (57.1%) with a mean age of 42 years. The mean (90% CI) ΔΔQTcF after MK‐8527 administration was < 10 ms at all time points (Figure 1). At maximum concentration of plasma MK‐8527, the mean (90% CI) ΔΔQTcF was −0.14 (−2.07, 1.78) ms; at maximum concentration of moxifloxacin, mean (90% CI) ΔΔQTcF was 10.55 (8.96, 12.14) ms. Six participants (14.3%; 2 participants in each treatment group) reported adverse events (AEs). No AEs were serious and no participants discontinued due to AEs.

**Conclusions**: A single supratherapeutic dose of MK‐8527 (66 mg) did not prolong the QTc interval in adults without HIV‐1.

**FIGURE 1:**
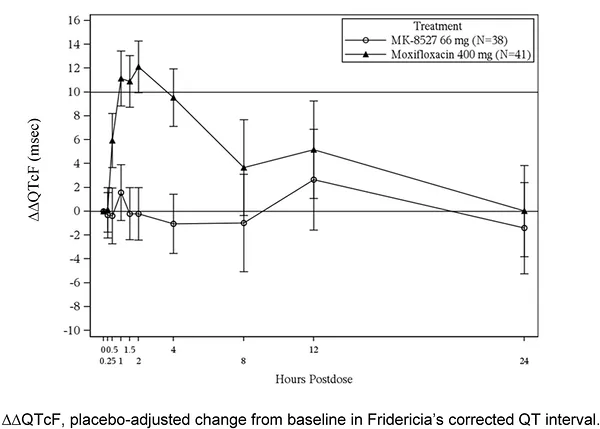
P268 | Time course of mean (90% CI) ∆∆QTcF following a single oral dose of MK‐8527 or moxifloxacin in healthy participants.

### P269 Abstract Withdrawn

### P270 Subtherapeutic Cabotegravir and/or Rilpivirine Plasma Concentrations After Long‐Acting Intramuscular Injections: Experience From a Nationwide TDM Service

David Burger ^1^, Anne Kamphuis^1^, Tom Jacobs^1^, Piter Oosterhof^2^, Lindsey Te Brake^1^, Ralf Stemkens^1^, Godelief Janssen‐van den Hurk^1^, Nynke Jager^1^, Roger Bruggemann^1^, Rob Aarnoutse^1^

^1^Pharmacy, Pharmacology & Toxicology, Radboudemc, Nijmegen, Netherlands; ^2^Pharmacy, OLVG, Amsterdam, Netherlands

**Background**: Low plasma concentrations of cabotegravir (CAB) and/or rilpivirine (RPV) after long‐acting (LA) intramuscular injections every 8 weeks have been shown to be associated with an increased risk of virological failure in some studies. Less is known how often patients have subtherapeutic levels of both agents or that one drug is more at risk than the other. In addition, no data are available in the literature what the effect is of shortening the dose interval of LA CAB and RPV to 6 weeks.

**Methods**: Drug concentration data were extracted from the laboratory information system for TDM samples received between June 2023 and June 2026. Samples were included in this analysis when a trough sample was drawn (i.e. 8 weeks since last injection), unless the application form indicated otherwise. Subtherapeutic plasma concentrations were defined as < 0.66 mg/L for CAB and < 0.032 mg/L for RPV. As this is an analysis based on TDM data only, no information on patient characteristics or clinical follow up is available to us.

**Results**: We analysed 218 samples from a total of 167 different patients. Subtherapeutic concentrations were found for CAB in 12 patients (7.2%), for RPV in 15 patients (9.0%) and both CAB and RPV were subtherapeutic in 7 patients (4.2%). A follow‐up TDM sample was available for 13 patients with an initial subtherapeutic drug concentration. In 6 patients, the dose interval was maintained at every 8 weeks and 2 of them (33%) had a subtherapeutic drug concentration again. In 7 patients, the dose interval was shortened to every 6 weeks and none of them (0%) had a subtherapeutic concentration in the second sample.

**Conclusions**: Our experience with TDM of CAB and RPV shows that patients can present with either low CAB or RPV concentrations, with a minority having low plasma concentrations of both agents. Shortening the dose interval to every 6 weeks demonstrated to be more effective than maintaining the 8‐week dose interval. Our data also show that patients may have incidental low drug concentrations, suggesting administration technique of individual injections in these patients is more likely explaining subtherapeutic concentrations than patient‐ or drug‐specific risk factors.

### P271 Clinical Consequences of the “Pharmacokinetic Tail” After Discontinuation of Long‐Acting Cabotegravir/Rilpivirine: Three Illustrative Cases

David Burger ^1^, Marieke De Regt^2^, Gijs Geerders^2^, Thijs van de Laar^2^, Jacobien Hoogerwerf^3^, Martine De Haan^3^, Piter Oosterhof^4^

^1^Pharmacy, Pharmacology & Toxicology, Radboudumc, Nijmegen, Netherlands; ^2^Internal Medicine, OLVG, Amsterdam, Netherlands; ^3^General Internal Medicine, Radboudumc, Nijmegen, Netherlands; ^4^Deot of Pharmacy, OLVG, Amsterdam, Netherlands

**Background**: Long‐acting (LA) intramuscular injections of cabotegravir (CAB) and rilpivirine (RPV) q8weeks is an effective alternative to daily oral antiretroviral therapy (ART). Unlike oral ART, discontinuation of LA CAB/RPV is followed by a “pharmacokinetic tail” with persistent low drug levels. The clinical implications remain largely unknown.

**Methods**: We describe three patients in whom therapeutic drug monitoring (TDM) was performed after discontinuation of LA CAB/RPV. Plasma concentrations of CAB/RPV were measured by LC‐MS/MS. Target concentrations used are > 0.66 mg/L for CAB and > 0.032 mg/L for RPV.

**Results**: Patient #1 was successfully treated with LA CAB/RPV for 6 months but developed a psychosis and was admitted elsewhere; no follow‐up injections were administered for the next 6 months. After recovery, the patent visited our clinic and HIV‐RNA was still <50 c/mL. TDM was performed and showed CAB: 0.14 mg/L and RPV: 0.022 mg/L. Patient #2 had achieved HIV‐RNA <50 c/mL on LA CAB/RPV but, due to challenging social circumstances, did not receive maintenance injections for 12 months and was lost to follow‐up. Upon return to care, HIV‐RNA remained < 50 c/mL. TDM was performed 12 months after the last injection and showed CAB: 0.02 mg/L and RPV: 0.039 mg/L. Patient #3 discontinued LA CAB/RPV after 24 months because of a pregnancy wish and switched to oral ART (darunavir/cobicistat/emtricitabine/tenofovir alafenamide fumarate). 27 months later, she presented with HIV‐RNA 30,000 c/mL and an undetectable darunavir plasma level. Genotypic resistance tests revealed mutations against NNRTI (V90I, K101E, Y181C) and InSTI (M50I, T97A, T125A); no mutations against NRTI or PI were present. TDM was performed and showed CAB: 0.01 mg/L and RPV: 0.022 mg/L.

**Conclusions**: These cases demonstrate that both CAB and RPV may remain detectable in plasma for substantially longer than generally anticipated after discontinuation. Persistent drug exposure was associated with contrasting clinical outcomes, including maintained virological suppression 6–12 months after the last injection (case #1 and #2) or development of resistance after 27 months (case #3). Awareness of this prolonged “pharmacokinetic tail” is important when counselling patients and when planning treatment discontinuation, monitoring and follow‐up on LA CAB/RPV.

### P272 Changes in Body Weight During Tenofovir Prodrug Exposure in Mice: Implications for Interpreting Weight Gain After Switching From TDF to TAF

Kensuke Abe ^1^, Emiko Sato^2^, Taku Obara^3^, Satomi Kamio^4^, Toshihiro Ito^5^, Nobuyuki Takahashi^2^

^1^Department of Pharmacy, National Hospital Organization Sendai Medical Center, Sendai, Japan; ^2^Division of Clinical Pharmacology and Therapeutics, Graduate School of Pharmaceutical Sciences, Tohoku University, Sendai, Japan; ^3^Department of Pharmaceutical Sciences, Tohoku University Hospital, Sendai, Japan; ^4^Department of Pharmacy, National Hospital Organization Takasaki General Medical Center, Takasaki, Japan; ^5^Department of Infectious Diseases, National Hospital Organization Sendai Medical Center, Sendai, Japan

**Background**: Switching from tenofovir disoproxil fumarate (TDF) to tenofovir alafenamide fumarate (TAF) has been associated with weight gain, but it remains unclear whether this reflects a weight‐promoting effect of TAF or removal of a weight‐suppressive effect of TDF. We examined the relationship between exposure to TDF or TAF and changes in body weight in mice.

**Methods**: Male C57BL/6J mice received drug‐containing chow. In Experiment 1, human‐equivalent doses of TDF (60 or 120 mg/kg/day) were administered for 4 weeks. In Experiment 2, high‐dose TDF (600 or 1200 mg/kg/day) and TAF (50 or 100 mg/kg/day) were administered for 2 weeks. In Experiment 3, right‐nephrectomized mice received TDF 600 mg/kg/day with or without allopurinol for 7 weeks. Body weight was expressed as median (interquartile range) change from baseline and compared with controls using the Wilcoxon rank‐sum test. Food intake was also recorded.

**Results**: In Experiment 1, TDF at human‐equivalent doses did not suppress body weight gain. In Experiment 2, body weight gain in the TAF 50 and 100 mg/kg/day groups did not differ from controls, whereas TDF 600 mg/kg/day attenuated body weight gain and TDF 1200 mg/kg/day caused significant weight loss at weeks 1 and 2. In Experiment 3, body weight gain was significantly suppressed versus controls from week 5 in the TDF 600 mg/kg/day group and from week 1 in the TDF 600 mg/kg/day plus allopurinol group (Figure 1). Although the most strongly suppressed groups also showed reduced food intake, the TDF 600 mg/kg/day group showed later body weight suppression despite food intake being comparable to controls, suggesting that reduced food intake alone did not fully explain the observed effect.

**Conclusions**: In mice, TAF did not clearly increase body weight, whereas TDF suppressed body weight in a dose‐dependent manner and more strongly in right‐nephrectomized mice. These findings suggest that weight gain after switching from TDF to TAF in humans may partly reflect removal of a weight‐suppressive effect of TDF rather than a direct weight‐promoting effect of TAF alone. Body weight monitoring after treatment switch may, therefore, need to take renal status into account.

**FIGURE 1:**
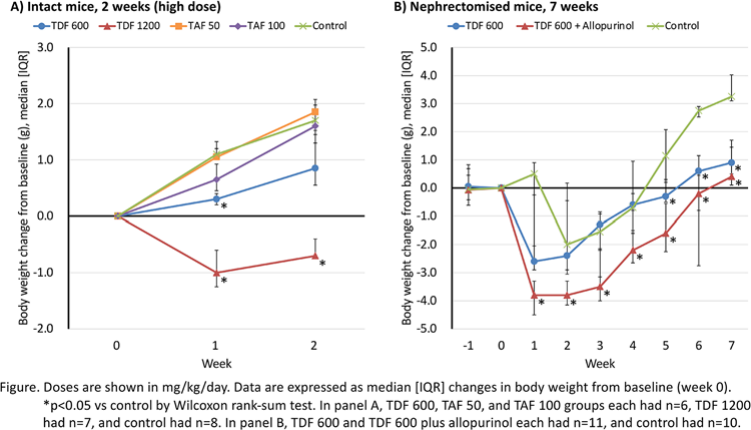
P272 | Body weight changes during high‐dose tenofovir prodrug exposure and in right‐nephrectomized mice.

### P273 Association of *UGT1A1* and *CYP3A5* Genetic Polymorphisms on Plasma Concentrations of Cabotegravir and Rilpivirine Following Long‐Acting Injectable Administration in Japanese HIV‐1‐Infected Patients

Hiroki Yagura ^1,2^, Takao Nakauchi^2^, Ayumi Hisari^1^, Ayano Fujiwara^1^, Keiji Konishi^3^, Kazuyuki Hirota^3^, Takashi Ueji^3^, Yasuharu Nishida^3^, Minoru Kawai^2^, Tomoko Uehira^3^, Takuma Shirasaka^3^, Jun Komano^4^, Dai Watanabe^1,3^

^1^Department of Advanced Medicine for HIV Infection, Institute for Clinical Research, NHO Osaka National Hospital, Osaka, Japan; ^2^Department of Pharmacy, NHO Osaka National Hospital, Osaka, Japan; ^3^AIDS Medical Center, NHO Osaka National Hospital, Osaka, Japan; ^4^Department of Microbiology and Infection Control, Faculty and Graduate School of Pharmaceutical Sciences, Osaka Medical and Pharmaceutical University, Osaka, Japan

**Background**: The long‐acting injectable formulations cabotegravir (CAB) and rilpivirine (RPV) are metabolized by UDP‐glucuronosyltransferase (UGT)1A1 and cytochrome P450(CYP) 3A5, respectively. We investigated the relationships between *UGT1A1* and *CYP3A5* genetic polymorphisms and plasma trough concentrations of CAB and RPV.

**Methods**: This study included 40 Japanese patients infected with HIV‐1 who received CAB and RPV at NHO Osaka National Hospital. Associations between these genetic variants and plasma concentrations of CAB and RPV were analysed. Furthermore, we compared the prevalence of UGT1A1*6, *28 and CYP3A5*3 genetic polymorphisms across different genotypes. We also evaluated the proportion of patients whose trough concentrations consecutively fell below the clinical trial first‐quartile (Q1) thresholds (1120 ng/mL for CAB and 32 ng/mL for RPV) at multiple time points.

**Results**: The allele frequencies of UGT1A1*6, *28 and CYP3A5*3 were 14%, 13% and 79%, respectively. Regarding *UGT1A1*, the study identified 1 case each of homozygous *6 and the *6/*28 compound heterozygote, alongside 9 cases each of the *6 and *28 heterozygotes. For CYP3A5*3, the homozygote and heterozygote were observed in 24 and 15 cases, respectively. Among cases in which the Q1 value fell below the threshold on multiple occasions, 10 cases (50%) in the CAB group carried the wild‐type allele for both UGT1A1*6 and *28, whereas 5 cases (28%) were heterozygous for *6 or *28. Regarding RPV, 3 cases (20%) were heterozygous for CYP3A5*3 and 1 case (4%) was homozygous.

**Conclusions**: The proportion of cases with trough concentrations falling below the Q1 thresholds on multiple occasions was relatively high among patients carrying the wild‐type UGT1A1*6 and *28 alleles for CAB and among those carrying the CYP3A5*3 heterozygous genotype for RPV. It has been reported that low CAB and RPV concentrations may be associated with low‐level viraemia. Future studies with larger sample sizes are needed to elucidate whether these genetic variants are key determinants of trough concentrations for each agent.

### P274 Treatment‐Emergent Bictegravir Resistance Following Nasogastric Administration of Bictegravir/Emtricitabine/Tenofovir Alafenamide in a Patient With Advanced AIDS and Cerebral Toxoplasmosis

David Kleinman^1^, Stephen Shafran^2^, Pam Nickel^3^, Conar O'Neil ^2^

^1^Lakeridge Healthcare Corporation, Oshawa, ON, Canada; ^2^Division of Infectious Diseases, University of Alberta, Edmonton, AB, Canada; ^3^Alberta Health Services, Edmonton, AB, Canada

**Background**: Bictegravir/emtricitabine/tenofovir alafenamide (B/F/TAF) is a recommended first‐line antiretroviral regimen with a high genetic barrier to resistance and exceptionally low rates of virological failure. Data supporting administration of dissolved B/F/TAF via enteral feeding tubes remain limited [1]. We describe a rare case of treatment‐emergent resistance during nasogastric administration of B/F/TAF in a treatment‐naïve individual with advanced AIDS and cerebral toxoplasmosis.

**Case Description**: A 49‐year‐old woman with newly diagnosed HIV presented with progressive headaches and multiple ring‐enhancing cerebral lesions. She was diagnosed with cerebral toxoplasmosis. Baseline HIV‐1 RNA was 1,194,690 copies/mL and CD4 count was 18 cells/µL (11%). Baseline genotyping demonstrated HIV‐1 subtype G without clinically significant resistance‐associated mutations. Seven days after initiation of pyrimethamine, sulfadiazine and leucovorin, B/F/TAF was started. Owing to persistent impaired consciousness, tablets were dissolved in water and administered via a nasogastric tube during enteral feeding.

**Outcomes**: Despite radiographic improvement in cerebral lesions and an increase in CD4 count to 125 cells/µL, HIV‐1 RNA increased to 2,262,510 copies/mL after 42 days of therapy. Repeat genotyping demonstrated emergence of the integrase mutation R263K and reverse transcriptase mutation M184V/I. Therapeutic drug monitoring confirmed low bictegravir concentration of 1823 ng/mL (reference C_min_ 2610 ng/mL). Antiretroviral therapy was changed to darunavir/cobicistat, tenofovir disoproxil fumarate/emtricitabine and doravirine administered via gastrostomy tube. HIV‐1 RNA subsequently declined to 61 copies/mL. The patient later died from neurologic complications of advanced disease.

**Conclusions**: Virological failure with B/F/TAF is exceedingly uncommon. This case demonstrates treatment‐emergent R263K and M184V/I mutations following administration of dissolved B/F/TAF via nasogastric tube in a treatment‐naïve individual with advanced AIDS, cerebral toxoplasmosis, severe immunosuppression and high baseline viraemia. Clinicians should exercise caution when administering B/F/TAF through enteral feeding tubes and consider alternative regimens with established pharmacokinetic data. This case supports further investigation into bictegravir exposure and resistance emergence during enteral administration.

**Reference**

1. Hocqueloux L, Lefeuvre S, Bois J, Brucato S, Alix A, Valentin C, Peyro‐Saint‐Paul L, Got L, Fournel F, Dargere S, Prazuck T, Fournier A, Gregoire N, McNicholl I, Parienti JJ. Bioavailability of dissolved and crushed single tablets of bictegravir, emtricitabine, tenofovir alafenamide in healthy adults: The SOLUBIC randomized crossover study. J Antimicrob Chemother. 2022;78(1):161–168.

## Community‐Based Treatment and Prevention Initiatives, Including Primary Care Screening

### P275 Creating the Conditions for Shared Decision‐Making: Perspectives of People Living With HIV and HIV Support Organizations Across 29 Countries

Ama Appiah ^1^, Brent Allan^2^, Garry Brough^3^, Erika Castellanos^4^, Daniela Rojas Castro^5^, W. David Hardy^6^, Kota Iwahashi^7^, Sindy Mbudwini^8^, Marta McBritton^9^, Mary Ndungu^10^, Annette Piecha^11^, Bruce Richman^12^, Mercy Shibemba^13^, Dainielle Fox^14^, Shaun Mellors^1^, Vilma Vega^14^, Nneka Nwokolo^15^, Emily Carr^1^, Rickesh Patel^16^

^1^ViiV Healthcare, London, UK; ^2^National Association of People Living with HIV Australia, Newtown, Australia; ^3^Transformation Partners in Health and Care, London, UK; ^4^Global Action for Trans Equality (GATE), Mijdrecht, Netherlands; ^5^Fundacion Huesped, Buenos Aires, Argentina; ^6^USC Rand Schrader (HIV) Clinic, Keck School of Medicine of USC, Los Angeles, CA, US; ^7^Akta, Tokyo, Japan; ^8^Nelson Mandela University, Port Elizabeth, South Africa; ^9^Barong, São Paulo, Brazil; ^10^Women's Health in Women's Hands Community Centre, Toronto, Canada; ^11^Kompetenznetz HIV/AIDS e.V, Frankfurt, Germany; ^12^Independent Consultant, New York City, NY, US; ^13^Independent Consultant, Manchester, UK; ^14^ViiV Healthcare, Durham, NC, US; ^15^St Stephen's Centre, Chelsea and Westminster NHS Foundation Trust, London, UK; ^16^GSK, London, UK

**Background**: Shared decision‐making improves HIV treatment experiences and outcomes, but depends on provider practices, patient comfort and support systems. We examined associations between involvement in HIV treatment decisions and treatment‐related outcomes among people living with HIV, alongside HIV patient organization's capacity to support treatment communication.

**Methods**: We analysed interim Positive Perspectives Wave 3 data from 2705 people living with HIV and 357 HIV patient‐organization personnel (PAGs) across 29 countries. Treatment decision‐making involvement was assessed by agreement that their provider asked for their opinion before prescribing their current HIV medication. HIV PAGs services provided by their organizations and the perceived comfort of people living with HIV in discussing treatment‐related concerns with their HIV provider were reported. Descriptive analyses and multivariable regression models were performed.

**Results**: Overall, 64.5% (*n* = 1745) of people living with HIV reported involvement in treatment decisions. Compared with those who reported no involvement, they were more likely to report treatment satisfaction (89.9% vs. 54.9%, *p* < 0.001), to discuss alternative treatment options (43.6% vs. 26.2%, *p* < 0.001) and the importance of adherence (86.5% vs. 78.1%, *p* < 0.001). After adjustment, involvement in treatment decisions remained associated with discussing treatment alternatives (aOR 2.13, 95% CI: 1.74–2.61), treatment satisfaction (aOR 6.98, 95% CI: 5.64–8.64), willingness to recommend treatment (aOR 4.12, 95% CI: 3.43–4.96), viral suppression (aOR 1.28, 95% CI: 1.06–1.54) and better quality of life. HIV PAGS reported challenges for the average service user and their providers with only 42% (*n* = 153) comfortable discussing adherence challenges, 39.4% (*n* = 141) work‐related impacts of HIV, 35.8% (*n* = 128) mental health concerns, 35.2% (*n* = 126) stigma experiences or daily medication as a reminder of HIV (35.3%, *n* = 126). HIV PAGs reported providing services, including linkage to care (95.1%, *n* = 340), emotional support and adherence counselling (90.8%, *n* = 324), treatment‐plan discussions (86.7%, *n* = 310), access to educational resources (85.3%, *n* = 305) and peer connections (80.7%, *n* = 288).

**Conclusions**: Patient involvement in HIV treatment decisions was independently associated with higher satisfaction, better adherence‐related outcomes, viral suppression and quality of life. However, many people living with HIV remain uncomfortable discussing key treatment and psychosocial concerns with providers. Meaningful shared decision‐making requires provider engagement and stronger community support.

### P276 Beyond the Primary Provider: Peer Support, U = U Communication and HIV Treatment Outcomes in Global Care Settings

Ama Appiah ^1^, Brent Allan^2^, Garry Brough^3^, Erika Castellanos^4^, Daniela Rojas Castro^5^, W. David Hardy^6^, Kota Iwahashi^7^, Sindy Mbudwini^8^, Marta McBritton^9^, Mary Ndungu^10^, Annette Piecha^11^, Bruce Richman^12^, Mercy Shibemba^13^, Dainielle Fox^14^, Shaun Mellors^1^, Vilma Vega^14^, Nneka Nwokolo^15^, Emily Carr^1^, Rickesh Patel^16^

^1^ViiV Healthcare, London, UK; ^2^National Association of People Living with HIV Australia, Newtown, Australia; ^3^Transformation Partners in Health and Care, London, UK; ^4^Global Action for Trans Equality (GATE), Mijdrecht, Netherlands; ^5^Fundacion Huesped, Buenos Aires, Argentina; ^6^USC Rand Schrader (HIV) Clinic, Keck School of Medicine of USC, Los Angeles, CA, US; ^7^Akta, Tokyo, Japan; ^8^Nelson Mandela University, Port Elizabeth, South Africa; ^9^Barong, São Paulo, Brazil; ^10^Women's Health in Women's Hands Community Centre, Toronto, Canada; ^11^Kompetenznetz HIV/AIDS e.V., Frankfurt, Germany; ^12^Independent Consultant, New York, NY, US; ^13^Independent Consultant, Manchester, UK; ^14^ViiV Healthcare, Durham, NC, US; ^15^St Stephen's Centre, Chelsea and Westminster NHS Foundation Trust, London, UK; ^16^GSK, London, UK

**Background**: Effective HIV care incorporates peer educators and community organizations/HIV networks. Yet, how peer support impacts HIV knowledge and adherence‐related outcomes remains incompletely understood. We examined associations between peer‐support in HIV services and HIV‐related outcomes across multiple countries.

**Methods**: Interim data from Positive Perspectives Wave 3 included surveys from 2705 people with HIV and 357 personnel from HIV organizations across 29 countries. Personnel reported roles, confidence discussing U = U and adherence and availability of peer‑support. A peer‑support index combined 10 activities, peer mentoring, support groups, volunteer programmes, advocacy, workshops, collaborative projects, walk‑in services, telephone outreach, referral systems and scheduled peer‑linked services, categorized into low, medium and high integration. Among people with HIV receiving U = U information from at least one healthcare‑associated source, we assessed whether additional exposure to peer/community U = U sources (peer educators, social workers, HIV organizations and other people with HIV) was associated with ability to explain U = U and self‑stigma. Multivariable logistic regression was used to estimate adjusted odds ratios (aORs) and 95% confidence intervals (CI).

**Results**: Among HIV‑organization personnel, 18.8% had formal peer‑support roles and 29.7% participated in peer‑led stigma training. Confidence was high for explaining U = U (83.5%) and supporting adherence (79.6%), but lower for assessing adherence (61.9%) and discussing adherence challenges, sexual health, or HIV‑status sharing. Common peer‑support services included linkage to peer mentors (56.3%), support groups (45.9%) and formal peer‑to‑peer programmes (42.6%). Median peer‑support index score was 3 (range 0–10). Greater peer‑support integration strongly correlated with higher estimated adherence. Each additional peer‑support activity increased odds of optimal adherence by 28% (aOR 1.28) and the highest tertile had nearly fourfold higher odds of reporting high adherence (aOR 3.83). Compared with individuals receiving U = U information only from healthcare sources, those additionally exposed to one peer/community source had 37% higher odds of being able to explain U = U, while those exposed to three additional sources had more than sevenfold higher odds of reporting low self‑stigma.

**Conclusions**: Stronger peer‑support integration within HIV services was associated with improved adherence and broader U = U dissemination. Peer‑mediated communication was linked to greater U = U understanding and reduced self‑stigma, underscoring the value of peer‑support systems in HIV care.

### P277 Living With HIV Across the Life Course: Gender and Age Differences in Stigma, Sexual Health Communication and Care Needs in 29 Countries

Ama Appiah ^1^, Brent Allan^2^, Garry Brough^3^, Erika Castellanos^4^, Daniela Rojas Castro^5^, W. David Hardy^6^, Kota Iwahashi^7^, Sindy Mbudwini^8^, Marta McBritton^9^, Mary Ndungu^10^, Annette Piecha^11^, Bruce Richman^12^, Mercy Shibemba^13^, Dainielle Fox^14^, Shaun Mellors^1^, Vilma Vega^15^, Nneka Nwokolo^16^, Emily Carr^1^, Rickesh Patel^17^

^1^Patient Affairs‐ViiV Healthcare, London, UK; ^2^National Association of People Living with HIV Australia, Newtown, Australia; ^3^Transformation Partners in Health and Care, London, UK; ^4^Global Action for Trans Equality (GATE), Mijdrecht, Utrecht, Netherlands; ^5^Fundacion Huesped, Buenos Aires, Argentina; ^6^USC Rand Schrader (HIV) Clinic, Keck School of Medicine of USC, Los Angeles, CA, US; ^7^Akta, Tokyo, Japan; ^8^Nelson Mandela University, Port Elizabeth, South Africa; ^9^Barong, São Paulo, Brazil; ^10^Women's Health in Women's Hands Community Centre, Toronto, Canada; ^11^Kompetenznetz HIV/AIDS, Frankfurt, Germany; ^12^Independent Consultant, New York, NY, US; ^13^Independent Consultant, Manchester, UK; ^14^Global Medical Affairs, ViiV Healthcare, Durham, NC, US; ^15^US Patient Affairs, ViiV Healthcare, Durham, NC, US; ^16^Stephen's Centre, Chelsea and Westminster NHS Foundation Trust, London, UK; ^17^GSK, London, UK

**Background**: Challenges faced by people living with HIV vary by gender and life stage. Assessing these challenges can help to identify distinct needs and opportunities for individualized care.

**Methods**: We analysed interim data from the Positive Perspectives Wave 3 survey of people living with HIV across 29 countries. Participants were categorized into four age‐sex groups: women < 50 years, women ≥50 years, men < 50 years and men ≥50 years. Gender categories included both cisgender and transgender participants. Outcomes were examined across five domains: (i) diagnosis and stigma, (ii) patient−provider relationships, (iii) sexual health and U = U communication, (iv) hormonal therapy experiences among women and (v) psychosocial wellbeing and quality of life. Descriptive comparisons were conducted across groups.

**Results**: Among 2705 people living with HIV, 20.6% (*n* = 553) were women < 50 years, 18.8% (*n* = 505) were women ≥50 years, 34.9% (*n* = 937) were men < 50 years and 25.7% (*n* = 689) were men ≥50 years. Women < 50 years were more likely to report difficulty accepting their HIV diagnosis (25.7% vs. 18.4% among women ≥50 years, 20.5% among men < 50 years and 14.6% among men ≥50 years; *p* < 0.001) and more likely to report feelings of shame related to HIV (34.4% vs. 27.0%, 33.0% and 25.4%, respectively; *p* < 0.001). Women < 50 years were also less likely to share their HIV status or discuss HIV‐related concerns with others and more commonly reported difficulty discussing sexual health with healthcare providers compared to men. Women < 50 years were most comfortable discussing U = U with healthcare providers (71.0%). Women receiving hormonal therapy reported substantial unmet information needs: around half changed or missed antiretroviral therapy due to interaction concerns, fewer than two‐thirds received relevant information and over half wanted more. Psychosocial wellbeing also varied across age and gender groups. Loneliness was highest among women < 50 years (42.0%) and lowest among men < 50 years (31.8%; *p* = 0.002).

**Conclusions**: Challenges relating to stigma, sharing HIV status and communication with provider was most common in women < 50 years. Women receiving hormonal therapy reported additional unmet needs regarding counselling on interactions with antiretroviral therapy. These findings underscore the importance of moving beyond a one‐size‐fits‐all approach to HIV care and the need for more individualized, life‐stage‐ and gender‐specific care.

### P278 Reach and HIV Outcomes Among Women Who Use Drugs in Abuja, Nigeria: Evidence From a Gender‐Responsive Community Harm Reduction Programme, 2024–2026

Ifeanyi Ngwoke ^1,2^, Oluwafisayo Alao‐Amiola^1^, Boluwatife Owoyemi^1^, Adeolu Adebiyi^3^

^1^YouthRISE Nigeria, Abuja, Nigeria; ^2^Royal Dick(s) School of Veterinary Studies, University of Edinburgh, Scotland, UK; ^3^Global Health Advocacy Incubator, WA, US

**Background**: People who use drugs (PWUD) in Nigeria face a disproportionate HIV burden, with prevalence among people who inject drugs estimated at 10.9% [1], nearly nine times the national general population rate of 1.3% [2]. Structural barriers, including criminalization, stigma and gender‐based violence (GBV) compound this burden, particularly for women. YouthRISE Nigeria's gender‐responsive harm reduction programme delivers integrated HIV prevention, sexual and reproductive health and rights (SRHR), mental wellbeing and economic empowerment services to women who use drugs and their children across three drop‐in centres (DICs) in three LGAs in Abuja (AMAC, Bwari and Gwagwalada). We describe programme reach, HIV service uptake and treatment linkage outcomes from January 2024 to June 2026.

**Methods**: Community mapping was conducted in 2024 and 2025 across three LGAs in Abuja to identify and characterize drug‐use hotspots and to inform site selection. Field teams engaged with community gatekeepers, law enforcement and PWUD through structured surveys in SurveyCTO, qualitative interviews and focus group discussions. Over 30 hotspots were identified as intervention sites. Peer educators with lived experience of drug use subsequently delivered HIV prevention, harm reduction and SRHR education at established sites. Routine programme data were extracted from an online‐centralized reporting system and analysed descriptively across all three reporting DICs for the reporting period.

**Results**: Between January 2024 and June 2026, the programme directly reached 30,617 individuals. A total of 11,268 received HIV prevention services, of whom 81.7% were female. HIV testing was accessed by 2078 individuals (65.1% female), with 22 newly diagnosed individuals enrolled into HIV care, 77.3% of whom were women. An additional 288 individuals on antiretroviral therapy (ART) received adherence counselling and support. A total of 354 HIV‐negative PWUD initiated pre‐exposure prophylaxis (PrEP) for the first time. Safe space services were accessed by 10,642 individuals and 1388 received mental wellbeing services. Over 4419 additional service contacts were recorded across SRHR, hepatitis, tuberculosis and drug counselling services and 41,295 commodities, including condoms, lubricants and needle‐syringe products, were distributed.

**Conclusions**: This programme demonstrates that a community‐rooted, peer‐led, gender‐responsive harm reduction model can achieve substantial reach and HIV service uptake among the most marginalized populations in an urban Nigerian setting.

**References**

1. Adebajo S, Eluwa G, Ahonsi B, et al. HIV epidemic among key populations in Nigeria: Results of the integrated biological and behavioural surveillance survey (IBBSS), 2020–2021. *Sex Transm Infect*. 2025;101(1):10–17. doi:10.1136/sti‐2023‐056116

2. Federal Ministry of Health Nigeria. Nigeria HIV/AIDS Indicator and Impact Survey (NAIIS) 2018: Summary Sheet. Abuja: Federal Ministry of Health; 2019.

### P279 Feasibility of Delivering Long‐Acting Cabotegravir as PrEP Beyond Sexual Health Services—A Mixed Methods Study

Rosalie Hayes^1^, Chloe Orkin^2^, Melanie Smuk^2^, Nishat Halim^2^, Sara Paparini ^1^

^1^Wolfson Institute, Queen Mary University of London, London, UK; ^2^Blizard Institute, Queen Mary University of London, London, UK

**Background**: Long‐acting cabotegravir (CAB‐LA) PrEP is approved in England, Scotland and Wales. CAB‐LA offers benefits over oral PrEP, but barriers to accessing sexual health services (SHS) remain. The “Beyond the Clinic” study addresses evidence gaps about the implementation of non‐SHS based CAB‐LA provision to improve equitable PrEP access.

**Methods**: “Beyond the Clinic” was a multi‐phase prospective feasibility study (March 2025–2026). Qualitative interviews were conducted with sexual health clinicians, informing a cross‐sectional survey of SHS, with further interviews then conducted with non‐SHS service providers in settings identified within the survey as feasible for CAB‐LA provision. The study captured perspectives on overall feasibility of delivering CAB for PrEP outside of SHS, potential settings and provider types and implementation facilitators, challenges and uncertainties.

**Results**: 77% (89/116) of SHS in the UK participated in the survey. Under a third (28%, *n* = 89) agreed out‐of‐clinic CAB‐LA provision is feasible within the next 3−5 years. Figure 1 depicts the range of non‐SHS settings which were deemed feasible by SHS respondents for CAB‐LA provision, including GP practices (43%), drug and alcohol services (39%), prisons (33%) and voluntary sector organizations (26%). Other services were mainly seen as feasible for promoting PrEP awareness and making referrals. 13 sexual health clinicians and 30 non‐SHS service providers were interviewed. Interviewees worked in varied settings, including GP practices, community pharmacies and drug and alcohol support services. Overall, non‐SHS service provider participants were more positive about the feasibility of non‐SHS based CAB‐LA provision than their SHS counterparts. Key themes included challenges around prescribing and procuring CAB‐LA in non‐SHS settings; concerns around being adequately involved in and remunerated for, non‐SHS clinic service delivery; and perceived low levels of PrEP awareness and demand among service users considered by providers as in need of CAB as PrEP.

**Conclusions**: Non‐SHS based CAB‐LA provision is considered feasible in certain settings by both SHS and non‐SHS service providers. Key strategies for facilitating implementation must include (i) addressing legal and regulatory barriers to integrated service provision; (ii) involving non‐SHS service providers as equal partners to promote buy‐in; and (iii) generating demand for CAB‐LA and addressing inequitable uptake through education, awareness‐raising and removal of structural barriers.

**FIGURE 1:**
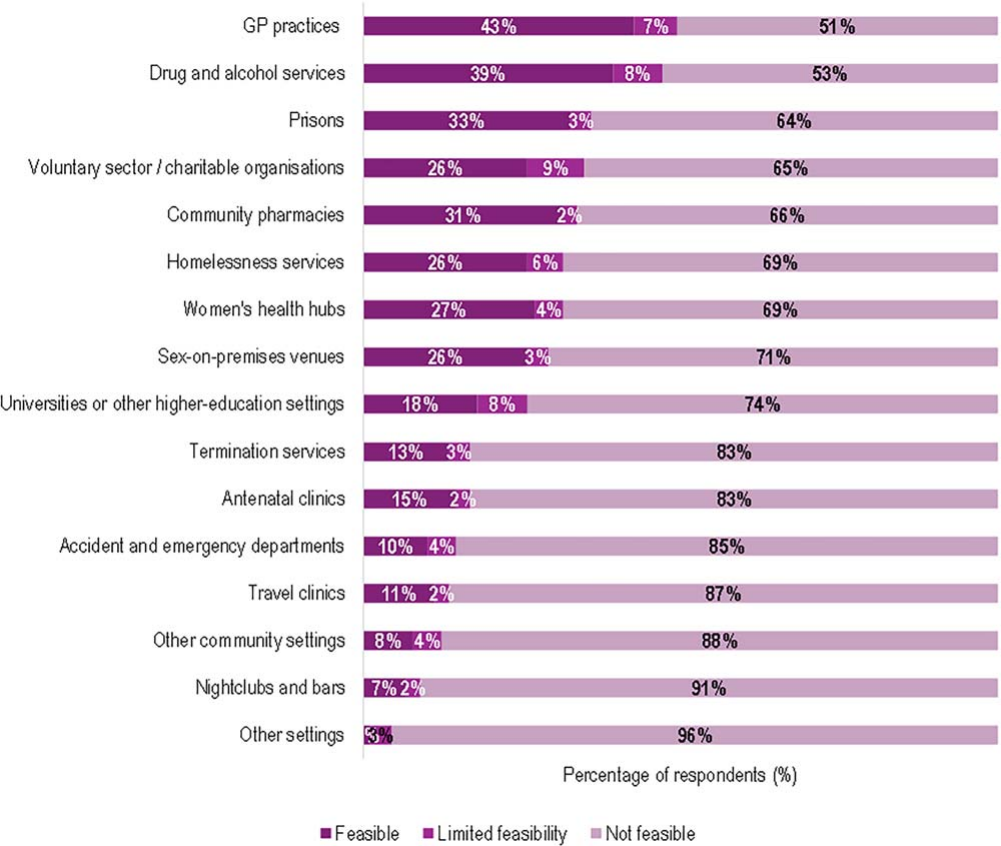
P279 | External settings perceived as feasible^*^ for delivery of CAB‐LA in the next 3–5 years by clinic survey respondents (*n* = 89). ^*^“Limited feasibility” captures responses where respondents considered these settings feasible ONLY for “Promoting CAB‐LA awareness” AND/OR “Identifying CAB‐LA candidates.” “Feasible” settings captures responses where respondents considered these settings as feasible for “Promoting CAB‐LA awareness” AND/OR “Identifying CAB‐LA candidates” AND “Conducting baseline testing,” “Prescribing CAB‐LA,” “Administering CAB‐LA,” “Conducting follow‐up testing,” AND/OR “CAB‐LA adherence support.”

### P280 Bridging the Gap Between HIV Vulnerability and Prevention: Risk Perception and PrEP Engagement Among Cisgender Women, Accessing a Community‐Based Centre in Italy

Liudmila Nekrasova ^1^, Lorenzo Cavallero^1^, Enrico Caruso^1^, Roberto Repossi^1^, Donatello Zagato^1^, Ivan Gotti^1^, Paolo Russo^1^, Pietro Vinti^1^, Davide Moschese^1,2^, Valentina Iannone^1,2^, Chiara Fusetti^1,2^, Roberto Rossotti^1,3^, Massimo Cernuschi^1,4^, Nicoletta Frattini^1^

^1^Milano Checkpoint ETS, Milan, Italy; ^2^ASST Fatebenefratelli‐Sacco, Milan, Italy; ^3^ASST Grande Ospedale Metropolitano Niguarda, Milan, Italy; ^4^San Raffaele Scientific Institute, Milan, Italy

**Background**: Despite accounting for 45% of all new HIV infections globally, cisgender women remain underrepresented in PrEP programmes. This study aimed to explore the relationship between HIV vulnerability, risk perception and PrEP engagement among cisgender women in a community‐based setting.

**Methods**: We conducted a cross‐sectional study among cisgender women accessing community‐based HIV/STI testing at “Milano Checkpoint ETS” between April and December 2025. Data were collected during peer‐led counselling using the COBATEST platform and an ad‐hoc survey. HIV vulnerability was assessed through a cumulative risk score (0–8) based on self‐reported STI diagnosis, ≥2 sexual partners, group sex, recreational drug use, irregular STI screenings, inconsistent condom use, sex work and sex with cisgender male partners in the previous 12 months. Participants were categorized as reporting 0–1, 2, or ≥3 risk indicators. PrEP awareness, interest and reasons for declining PrEP were assessed. Associations were evaluated using chi‐square tests and multivariable logistic regression.

**Results**: Among 250 cisgender women, 94.4% reported at least one guideline‐based (CDC, EACS, BHIVA/BASHH) indication for PrEP and 72.8% reported ≥3 concurrent indicators on the cumulative vulnerability score (Table 1). PrEP awareness (*p* = 0.572) and PrEP interest (*p* = 0.700) did not differ across vulnerability categories and no individual behavioural risk factor was independently associated with PrEP interest (*p* > 0.05). Among the 194 women not interested in PrEP, perceived adequate risk control was the most commonly reported reason for non‐interest (75.8%), followed by gendered or misinformation‐based beliefs (35.1%) and medical/pharmaceutical concerns (21.6%). Higher vulnerability was equally prevalent among women endorsing perceived protection and those who did not (72.5% vs. 71.1%; *p* = 0.857). Among women citing condom use as a reason for declining PrEP, 40.7% reported a condomless intercourses and/or inconsistent condom use; among those reporting “only one partner,” 62.8% reported ≥2 partners or non‐monogamous partnerships in the previous year.

**Conclusions**: Most interviewed women met guideline‐based indications for PrEP and reported multiple concurrent HIV‐related risk factors. However, greater vulnerability was not associated with increased PrEP interest. The discordance between perceived protection and reported behaviours suggests that inaccurate risk perception may be a key barrier to PrEP engagement among cisgender women and tailored communication is needed.

**TABLE 1:**
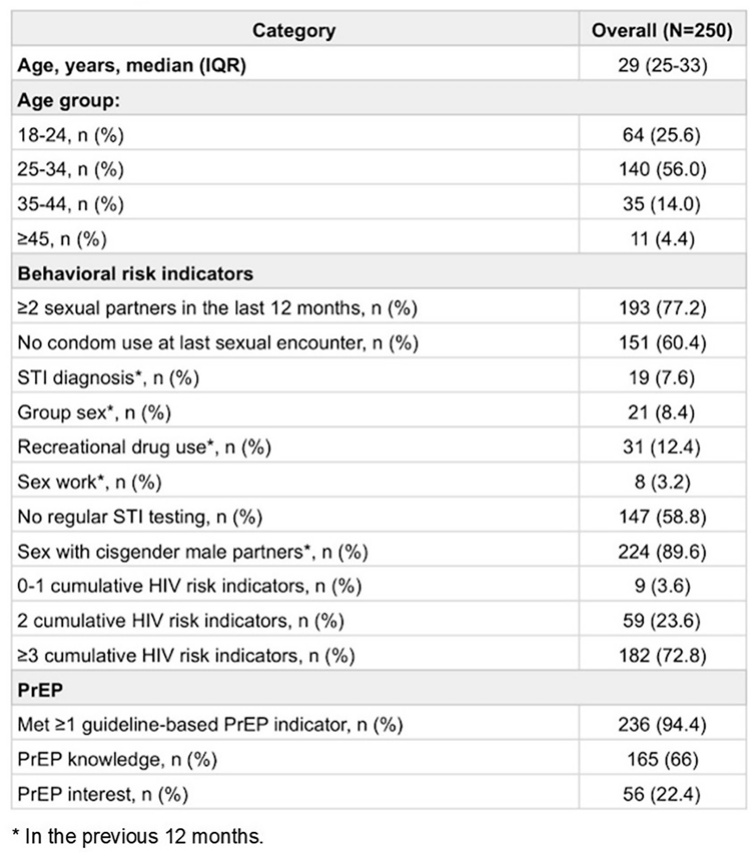
P280 | Characteristics of cisgender women accessing a community‐based HIV/STI testing service.

### P281 Viral Suppression and Retention in Care for Non‐Suppressing Children and Adolescents Living With HIV Enrolled in ICSDM in East Central Africa

Ibrahim Twinomujuni

Makerere University Kampala, Kampala, Kampala, Uganda

**Background**: The World Health Organization (WHO) recommended differentiated service delivery models (DSDMs) to provide client centred anti retral viral therapy (ART) services and relieve client load on overworked health systems. The integrated community service delivery model (ICSDM), one of the DSDMs recommended by the Ministry of Health of Uganda is hinged on a family‐centred and community‐led approach to achieve HIV viral suppression. We studied viral suppression and adherence to ART among CALHIV enrolled in ICSDM in rural public HIV clinics in East Central Uganda.

**Methods**: This was a retrospective cohort study between October 2023 and January 2026. Data abstraction for non‐suppressing CALHIV eligible for ICSDM was conducted between February and April 2026. Records of 243 non‐suppressing CALHIV between October 2023 and January 2026 were reviewed. Inclusion required HIV records of all non‐suppressing CALHIV (0–19 years) at least 3 months since enrolment by the time of the study, regardless of whether clients in the model. For analysis of enrolment into ICSDM a generalized linear model with a Poisson distribution, log link and robust standard errors was used to estimate risk ratios, retention in HIV care, viral load were assessed using binary logistic regression.

**Results**: A total of 243 CALHIV records of non‐suppressed viral load were reviewed, 79.8% (*n* = 194) were enrolled in ICSDM, of whom 90.7% (*n* = 176) were retained in care while on the ICSDM and 9.3% (*n* = 18) declined. Overall, 61.3% (*n* = 119) achieved viral load suppression while on the ICSDM and 74.8% (*n* = 89) suppressed within 6 months, 19.3% (*n* = 23) suppressed between 6–12 months and 5.8% (*n* = 7) after 12 months.

**Conclusions**: In rural public HIV clinics, non‐suppressing CALHIV enrolled on ICSDM achieved high retention in HIV care and overall viral suppression within 6 months of enrolling on ICSDM emerging as a potential alternative to facility‐based management of non‐suppressing CALHIV. However, CALHIV in the age category 10–14 and those lacking psychosocial support did not achieve viral suppression in spite enrolling on ICSDM. Future studies should explore alternative approaches to address viral suppression and retention among adolescent aged 10–14 years.

### P282 Emergency Department (ED) Bundled HIV, HBV and HCV Screening Using Residual Blood Samples in Italy: Challenges and Opportunities

Federico Cecilia ^1,2^, Martina Leone^1,2^, Sara Ciarpaglini^1,2^, Enrico Perugini^1,2^, Diletta Meloni^1^, Livia Benedetti^1^, Alfredo Giovannelli^3,4^, Martina Pelagalli^3,4^, Flaminia Tomassetti^3,4^, Drieda Zace^1^, Andrea Di Lorenzo^1,2^, Jacopo Maria Legramante^1,5^, Carla Paganelli^5^, Vincenzo Malagnino^1,2^, Sergio Bernardini^3,4^, Massimo Pieri^3,4^, Loredana Sarmati^1,2^, Anna Maria Geretti^1,2^, on behalf of PROGRESSO‐T Study Team

^1^Department of Medicine of Systems, University of Rome Tor Vergata, Rome, Italy; ^2^Department of Infectious Diseases, Tor Vergata University Hospital, Rome, Italy; ^3^Department of Experimental Medicine, University of Rome Tor Vergata, Rome, Italy; ^4^Department of Laboratory Medicine, Tor Vergata University Hospital, Rome, Italy; ^5^Emergency Department, Tor Vergata University Hospital, Rome, Italy

**Background**: In Italy, HBV prevalence has declined following implementation of universal vaccination in 1991, whereas late diagnosis of HIV and HCV infection remains a major public health challenge. A national HCV screening programme launched in 2021 targets the birth cohort 1969–1989. Written informed consent remains mandatory for HIV testing, but not for HCV or HBV testing. We evaluated the feasibility, uptake and diagnostic yield of opportunistic HIV, HCV and HBV screening at the Policlinico Tor Vergata ED in Rome.

**Methods**: Between December 2025 and April 2026, adult ED attendees were offered screening during predefined time slots by dedicated non‐ED staff. Written informed consent was obtained for HIV testing separately from consent to study participation. Residual blood samples from routine haematology testing were tested for anti‐HIV, anti‐HCV and HBsAg using rapid lateral flow assays.

**Results**: Among 1000 individuals offered screening (55% male; median age 55 years), 759 (76%) accepted and 241 (24%) declined (Figure 1). Uptake was highest during daytime, peaking in the afternoon (81.3%). After adjustment for sex and time slot, increasing age independently predicted lower uptake (aOR per decade older 0.87; 95% CI: 0.80–0.95; *p* = 0.002). Common reported reasons for declining testing included having recently tested (26%) and refusing to sign the HIV consent form (20%). Residual samples were retrieved for 701/759 (92%) participants. Among tested individuals, 12 (1.7%; 95% CI: 0.9–3.0) had detectable anti‐HCV, including three new diagnoses and two previously lost‐to‐follow‐up cases. Three participants (0.4%) had detectable anti‐HIV, including one new diagnosis. Two participants (0.3%) had detectable HBsAg, both newly diagnosed. None of the newly identified HCV cases fell within the target birth cohort (1969–1989). Six of eight newly diagnosed or previously lost‐to‐follow‐up individuals were successfully linked to care, including one with HCV and hepatocellular carcinoma.

**Conclusions**: ED‐based bundled HIV/HCV/HBV screening using residual blood samples was feasible and achieved high uptake, but was operationally burdensome. Newly identified HCV cases fell outside the currently targeted screening birth cohort, highlighting limitations of age‐restricted screening strategies and supporting the value of opportunistic ED‐based testing approaches. Simplification of HIV testing consent procedures is essential to support scalable integrated screening strategies in Italy.

**FIGURE 1:**
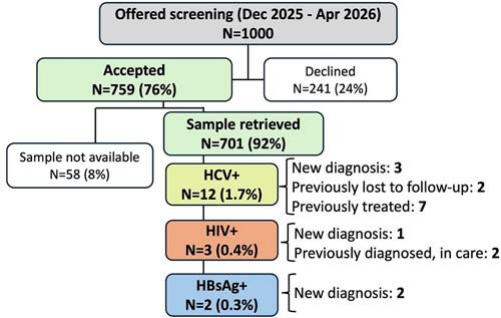
P282 | Screening cascade.

### P283 Peer‐Led Territorial Outreach Is Associated With Higher Screening Uptake Among Young Candidates for Long‐Acting PrEP Research in São Paulo, Brazil

Vinicius Silva ^1^, Lucas Ramos^1^, Camila Moraes^1^, Aline Cano^2^, Giovanna Haimowski^1^, Camilla Ferreira Santos^1^, Fabíola Rocha^1^, Pisci Bruja^1^, Dyemison Silva^1^, José Madruga^1^, Roberta Nogueira^1^

^1^Centro de Referência e Treinamento IST/AIDS, São Paulo, Brazil; ^2^Centro de Referência e Treinamento IST/AIDS, São Paulo, Brazil

**Background**: Long‐acting antiretroviral‐based HIV prevention can expand prevention choice for populations underserved by conventional services. However, digital recruitment may generate interest without ensuring progression to screening, particularly for young people facing structural barriers to research participation. We evaluated whether peer‐led territorial outreach was associated with screening uptake and youth participation in a long‐acting pre‐exposure prophylaxis (PrEP) study in São Paulo, Brazil.

**Methods**: We conducted a cross‐sectional analysis of recruitment and screening data from a long‐acting PrEP study at a research centre in São Paulo. Eligible candidates were aged ≥18 years and reported recent likelihood of HIV acquisition, including condomless receptive anal sex, a recent sexually transmitted infection and/or stimulant drug use during sex. Recruitment occurred between February and March 2026 through digital/media strategies, including social media, dating applications and LGBTQIA+ media and peer‐led territorial outreach in LGBTQIA+ social spaces and peripheral areas. Screening uptake was compared between strategies. Among screened candidates, Pearson's chi‐squared test assessed the association between recruitment strategy and age group, defined as ≤24 versus > 24 years.

**Results**: Digital/media strategies generated 766 online registrations, of whom 47 (6.1%) attended screening. Peer‐led territorial outreach approached 149 people, of whom 50 (33.6%) attended screening. Screening uptake was higher through territorial outreach than through digital/media recruitment (odds ratio [OR] 7.73, 95% confidence interval [CI] 4.93–12.12; *p* < 0.001). Among screened candidates, 16/47 (34.0%) from digital/media recruitment and 31/50 (62.0%) from territorial outreach were aged ≤24 years. Territorial outreach was associated with higher odds of screening a young candidate (OR 3.16, 95% CI: 1.38–7.25; Pearson's chi‐squared *p* = 0.006).

**Conclusions**: Peer‐led territorial outreach was associated with substantially higher progression to screening and reached a higher proportion of young candidates in long‐acting PrEP research. Digital visibility remained important but was insufficient on its own to ensure study access. Community‐led, trust‐based recruitment can connect prevention research with the territories where young people socialize, seek information and make prevention decisions. Peer educators should be recognized as strategic partners in person‐centred HIV prevention implementation and research.

### P284 A Case‐Manager‐Lead Telephone Call‐Back Strategy Substantially Increases HIV Testing Coverage Among Patients Diagnosed With Sexually Transmitted Infections: A 4‐Year Single‐Centre Analysis

Chia‐Jui Yang ^1,2^, Chung‐Yu Shih^3^, Yin‐Hsuan Chiang^3^, Mao‐Song Tsai^1^

^1^Department of Internal Medicine, Far Eastern Memorial Hospital, New Taipei City, Taiwan; ^2^School of Medicine, National Yang Ming Chiao Tung University, Taipei, Taiwan; ^3^Infection Control Center, Far Eastern Memorial Hospital, New Taipei City, Taiwan

**Background**: Achieving the UNAIDS 95‐95‐95 targets requires expanding HIV screening to detect undiagnosed infection; Taiwan's 2025 performance was 93‐96‐95. Patients with sexually transmitted infections (STIs) are at elevated HIV risk and a high‐yield testing population. We implemented a case‐manager (CM)‐led telephone call‐back intervention and evaluated its impact on testing coverage, departmental performance and positivity.

**Methods**: From July 2023, CMs used the hospital infection‐control system to identify notifiable STI cases (syphilis, gonorrhoea, mpox, acute viral hepatitis A/B/C, bacillary and amoebic dysentery) and cross‐checked HIV testing status. When testing was incomplete, the CM telephoned the attending physician to prompt testing, or the patient directly to schedule screening. We analysed all eligible cases (July 2021–June 2025), excluding known HIV‐positive patients; the denominator was all eligible notified cases and the numerator those completing a test. Screening rate, its decomposition by testing route, departmental “true” rate (screened ÷ all eligible cases; target 100%) and positivity were compared before (July 2021–June 2023) versus after (July 2023–June 2025) the intervention.

**Results**: Among 1072 eligible patients, the screening rate rose from 54.5% (283/519) to 79.6% (440/553)—an increase of +25.0 percentage points (χ^2^ = 75.3, *p* < 0.0001) with a significant rising monthly trend (Cochran–Armitage *z* = 6.97, *p* < 0.0001; Figure 1). On‐visit testing was essentially unchanged (≈47%); the entire gain was attributable to call‐back, which contributed +32.4 percentage points in the post‐period (Figure 2). Improvement occurred across departments: Urology, the largest contributor, rose 49%→82% (*n* = 472) and Emergency Medicine 42%→70%, both driven predominantly by call‐back, while Infection improved 83%→94%; none reached the 100% target. Call‐back roughly doubled the number screened (266→457) while positivity remained stable (7.1% [19/266] vs. 6.1% [28/457]; *z* = −0.53, *p* = 0.59), identifying more confirmed cases (28 vs. 19) without diluting yield.

**Conclusions**: A CM‐led telephone call‐back strategy produced a large, statistically significant and sustained increase in HIV testing coverage among STI patients, accounting for essentially the entire observed improvement. Because positivity was unchanged, the additional tests captured proportionally as many true infections, supporting call‐back as an efficient, scalable route to earlier diagnosis, linkage to care and treatment‐as‐prevention. Residual departmental gaps mark clear targets for further optimization.

**FIGURE 1:**
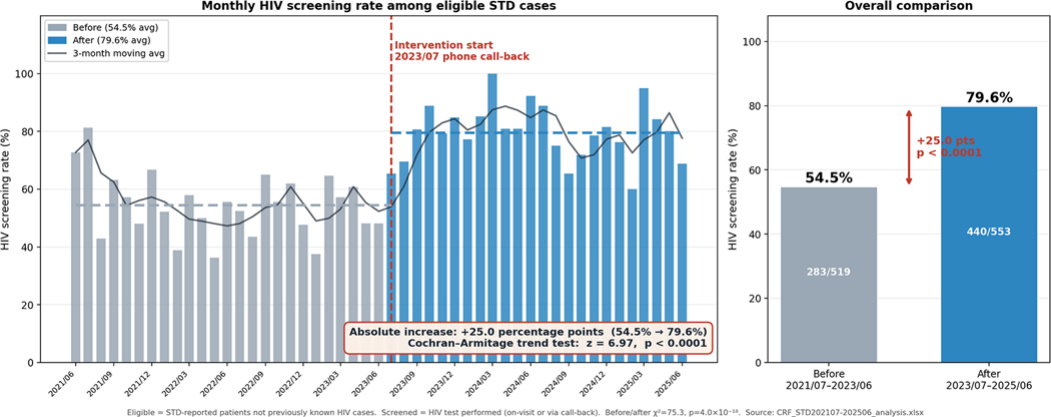
P284 | Monthly HIV screening rate before versus after the call‐back intervention (overall +25.0 pp; *p* < 0.0001).

**FIGURE 2:**
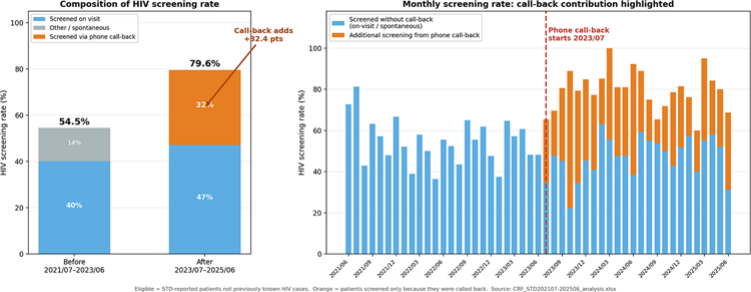
P284 | Decomposition of the screening rate: call‐back accounts for +32.4 pp of post‐intervention screening.

### P285 Bundled, Community‐Embedded HIV Pre‐Exposure Prophylaxis Pathways for Underserved Groups: A Multi‐Site Quality Improvement Initiative

Jennifer Whetham^1^, Luke Byrne^2^, Emma Rutland^3^, Elisabete Miranda^4^, Marc Tweed^5^, Emma Lock^1^, Clare Lewandowski‐Fowler‐Porter^1^, Kamran Khan^1,2^, Vikki Pearce^6^, Martyn Wood ^7^

^1^University Hospitals Sussex NHS Foundation Trust, Brighton, UK; ^2^NHS University Hospitals of Liverpool Group, Liverpool, UK; ^3^University Hospitals Sussex NHS Foundation Trust, West Sussex, UK; ^4^BHA for Equality, Liverpool, UK; ^5^Terrence Higgins Trust, Brighton, UK; ^6^Elton John AIDS Foundation, London, UK; ^7^Axess Sexual Health Medicine, Liverpool, UK

**Background**: Despite national progress in HIV prevention, access to pre‐exposure prophylaxis (PrEP) remains inequitable across underserved populations including women, people seeking asylum, refugees, migrants, ethnic minorities (especially Black people), sex workers, trans and non‐binary people and people experiencing homelessness. This initiative, funded by the Elton John AIDS Foundation, tested a multi‐component, community‐embedded model for PrEP delivery across three regions in England, aligned with the UK HIV Action Plan 2025–2030.

**Methods**: Using a place‐based approach, interventions included: (i) a period pack initiative bundling menstrual and sexual health products with PrEP conversations for women; (ii) outreach with on‐site PrEP prescribing and signposting within (a) women's health hubs (WHH) and (b) asylum, refugee and migrant (ARM) primary care settings; (iii) co‐design with community organizations; and (iv) clinic‐led digital PrEP pathways. Data were extracted from electronic and outreach records; insights were synthesized from co‐design workshops.

**Results**: During the first 6 months, the period pack initiative delivered 30 training sessions reaching 74 staff in community organizations, distributing 2000 packs: PrEP was offered to 125 women and started in 12. At a WHH, 40 women were screened over 6 months: PrEP was discussed with 34 women (85%) and PrEP started with three on‐site. In the ARM setting, documented PrEP discussions average around 49% of all conversation (296 out of 628 total conversations) across 9 months. Co‐design with 84 individuals from 10 community organizations identified stigma, digital exclusion and perceptions of PrEP as not for “us” as key barriers, prioritizing trusted messengers and multi‐route access. Clinic‐led digital pathways supported release of capacity in clinics offering an alternative route to access PrEP/PrEP follow‐up. Of those issued PrEP via a direct‐to‐consumer digital pathway in the last 6 months, 58% (37 out of 64 patients) were clinic to digital channel shift.

**Conclusions**: A bundled model combining outreach, on‐site prescribing, co‐design and digital self‐service can expand reach into underserved populations while releasing clinical capacity via channel shift to digital. On‐site provision remains essential for converting outreach contact into commencement and sustained use, complementing rather than replacing digital pathways. Evaluation, including an economic evaluation, is ongoing due in December 2026.

### P286 A Preliminary Evaluation of the Reimbursement Application Process for Long‐Acting Injectable Antiretroviral Therapy Among Healthcare Professionals in Taiwan

Na‐Lee Sun, Shu‐Hsing Cheng, Chien‐Yu Cheng

Comprehensive AIDS Care Center, Taoyuan, Taiwan

**Background**: Since April 2024, long‐acting injectable antiretroviral therapy (LA‐ART) has been included in Taiwan's National Health Insurance (NHI) reimbursement programme. With increasing real‐world experience, both clinical benefits and administrative challenges have emerged. This study aimed to assess healthcare professionals’ perceptions of the clinical value of LA‐ART for people living with HIV (PLWH), particularly regarding medication adherence, quality of life and disease control and to identify challenges within the current reimbursement application process along with practical recommendations for optimization.

**Methods**: A cross‐sectional study was conducted using an anonymous online questionnaire targeting healthcare professionals directly involved in HIV care and LA‐ART–related services. The survey included three components: respondent characteristics and experience with LA‐ART reimbursement applications; perceived clinical benefits of LA‐ART assessed using a 5‐point Likert scale across domains such as medication adherence, quality of life and concerns about HIV disclosure; and perceived challenges and suggested improvements related to the reimbursement application process. Open‐ended questions were included to capture qualitative insights. Quantitative data were analysed using descriptive statistics, while qualitative responses were examined through content analysis, including coding and thematic synthesis, to capture in‐depth professional perspectives.

**Results**: A total of 43 valid responses were analysed (67.4% case managers, 25.6% infectious disease physicians; 58.1% from regional hospitals).

Clinical value: There was an overwhelming consensus on the benefits of LA ART, with 93.0% agreeing it reduces daily pill burden and 90.7% noting improved patient treatment adherence.

Administrative barriers: Despite high clinical buy in, 74.5% of respondents characterized the current reimbursement process as “strict” or “complex” (Table 1).

Operational pain points: The primary hurdles identified were extensive documentation requirements (46.5%), prolonged review timelines by the CDC (44.2%) and difficulties in assessing patient eligibility (41.9%) (Table 2).

Systemic friction: Notably, 30.2% of respondents reported rejections for patients who seemingly met eligibility criteria, suggesting a lack of transparency in the review mechanism.

**Conclusions**: While the clinical transition to LA ART is highly endorsed by HCPs in Taiwan, administrative friction serves as a significant deterrent. To maximize the public health impact of this innovation, strategic focus must shift towards process optimization, specifically shortening review cycles and digitizing ap plication workflows. Streamlining these “back office” operations is essential to ensure that clinical breakthroughs translate efficiently into patient outcomes.

**Keywords**: long‐acting injectable antiretroviral therapy, medication adherence, people living with HIV, reimbursement process.

**TABLE 1:**
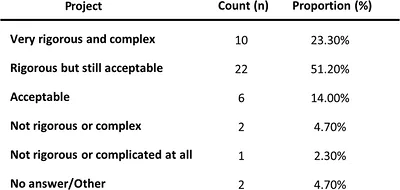
P286 | Challenges and improvements to the application process. Is the application process rigorous and complicated? (*N* = 43).

**TABLE 2:**
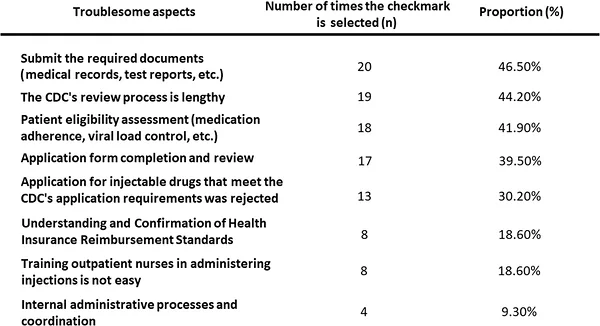
P286 | Challenges and improvements to the application process. What is the most troublesome or time‐consuming part? (*N* = 43).

### P287 Enhancing Community‐Centred Treatment, Prevention and Early Screening Strategies

Ronald Ondari, Sarah Nyaboke

Rescue Hope International, Nairobi, Kenya

**Background**: Despite significant advances in antiretroviral therapy (ART), persistent gaps in early HIV diagnosis, treatment initiation and long‐term viral suppression continue to challenge epidemic control in sub‐Saharan Africa (SSA). Community‐level barriers including stigma, delayed healthcare access, limited primary care screening and poor retention in care contribute to increased morbidity and ongoing transmission. This study evaluated the impact of integrated community‐centred prevention, treatment and early screening interventions on HIV clinical outcomes and care engagement among people living with HIV (PLWH).

**Methods**: A prospective multicentre cohort study was conducted from January 2024 to February 2026 across six community primary healthcare facilities in SSA. Adults aged ≥18 years with confirmed HIV infection or identified as high‐risk during community screening campaigns were enrolled. Interventions included decentralized HIV screening, rapid linkage‐to‐care pathways, adherence support, peer‐led education and optimized dolutegravir‐based ART regimens (DTG/3TC). Primary endpoints were viral suppression (VL < 50 copies/mL) at 48 weeks and uptake of HIV screening services. Secondary outcomes included retention in care, CD4 cell recovery, time‐to‐suppression assessed using Kaplan–Meier analysis and adverse events graded according to DAIDS criteria. Intention‐to‐treat (ITT) analysis was performed.

**Results**: Among 1248 participants enrolled, 91.6% initiated ART within 14 days of diagnosis. Community‐based screening increased HIV testing uptake by 38% and reduced late‐stage presentation by 27%. Viral suppression at 48 weeks was achieved in 92.4% of participants receiving DTG/3TC‐based therapy compared with 84.7% on standard regimens (HR 0.65, 95% CI: 0.48–0.88; *p* < 0.001). Mean CD4 recovery improved significantly (ΔCD4 +154 cells/µL). Retention in care reached 89.2%, while severe adverse drug events remained low (3.1%). Kaplan–Meier analysis demonstrated faster time‐to‐viral suppression in the intervention cohort.

**Conclusions**: Integrated community‐centred treatment, prevention and primary care screening strategies were associated with measurable improvements across key HIV outcomes. Participants receiving the intervention demonstrated higher viral suppression rates at 48 weeks (92.4% vs. 84.7%), increased retention in care (89.2%) and greater immune recovery (ΔCD4 +154 cells/µL) compared with standard care approaches. Community‐based screening also increased HIV testing uptake by 38% and reduced late‐stage presentation by 27%, highlighting the positive impact of decentralized and community‐driven HIV service delivery in resource‐limited settings.

### P288 Implementation of Integrated HIV and Viral Hepatitis Screening in a Federally Qualified Health Centre: A Retrospective Evaluation

Lamiya Hussain

CPACS Cosmo Health Center, Norcross, GA, US

**Background**: Despite recommendations for routine HIV and viral hepatitis screening, implementation within primary care serving medically underserved populations remains inconsistent. Federally qualified health centres (FQHCs) provide an opportunity to integrate comprehensive infectious disease screening into routine clinical care and expand equitable access to early diagnosis and linkage to care. This study evaluated the implementation and outcomes of an integrated HIV, hepatitis B virus (HBV) and hepatitis C virus (HCV) screening programme at an FQHC serving a diverse patient population in metropolitan Atlanta, Georgia.

**Methods**: A retrospective evaluation of de‐identified electronic health record laboratory data collected between December 2022 and May 2026 was conducted. Monthly laboratory reports were standardized into a longitudinal dataset comprising HIV fourth‐generation screening, hepatitis B surface antigen (HBsAg) and HCV antibody test results. Patient demographics, screening volume, same‐day co‐screening and abnormal screening outcomes were analysed using descriptive statistics. Abnormal screening results included preliminary reactive HIV screens, positive HBsAg results and reactive HCV antibody tests requiring confirmatory testing and linkage to care.

**Results**: Across 41 analysable monthly reports, 9495 screening tests were performed among 2819 unique patients, representing 3502 screening encounters. The study population was racially and ethnically diverse, with 55.0% identifying as Hispanic or Latino. Screening included 2776 HIV tests, 3396 HBsAg tests and 3323 HCV antibody tests. Abnormal screening results were identified in 11 HIV tests (0.4%), 53 HBsAg tests (1.6%) and 22 HCV antibody tests (0.7%). Integrated same‐day HIV, HBV and HCV screening occurred during 76.0% of screening encounters and 95.8% of encounters that included HIV testing, demonstrating successful integration of comprehensive infectious disease screening into routine primary care workflows. Screening volume increased throughout the study period as the programme became embedded within routine clinical practice (Figure 1 and Table 1).

**Conclusions**: Integrated HIV and viral hepatitis screening was successfully implemented within routine primary care at an FQHC serving medically underserved communities. High rates of same‐day co‐screening demonstrate that comprehensive infectious disease screening can be incorporated into routine clinical workflows while expanding equitable access to early diagnosis and linkage to care. This model may inform similar community‐based primary care programmes seeking to strengthen integrated HIV and viral hepatitis screening and improve linkage to care among underserved populations.

**FIGURE 1:**
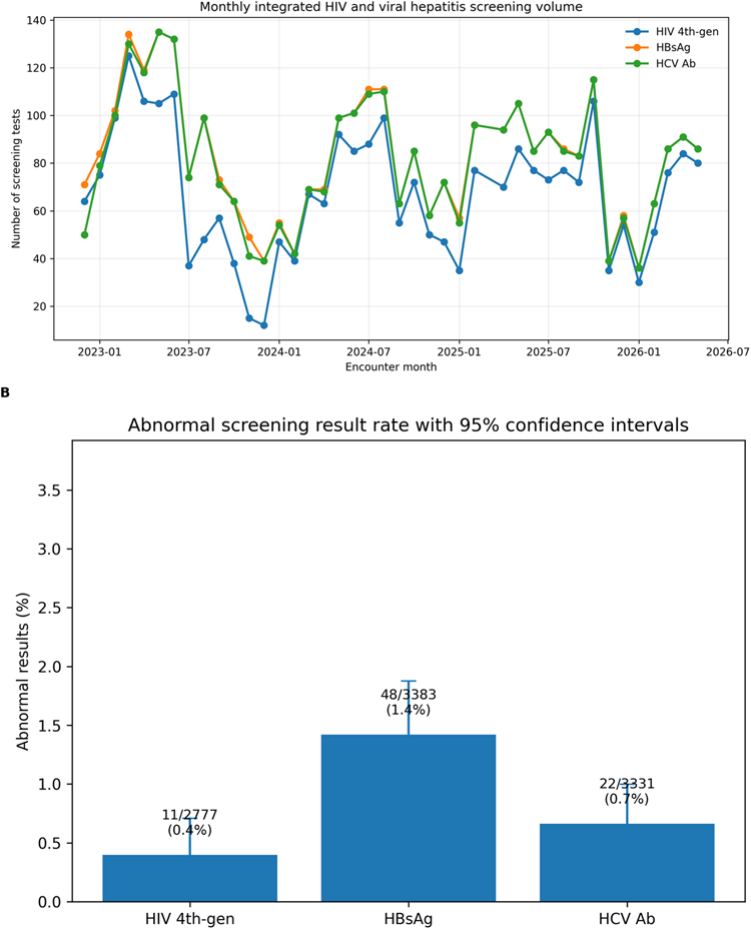
P288 | Integrated HIV and viral hepatitis screening implementation outcomes.

**TABLE 1:**
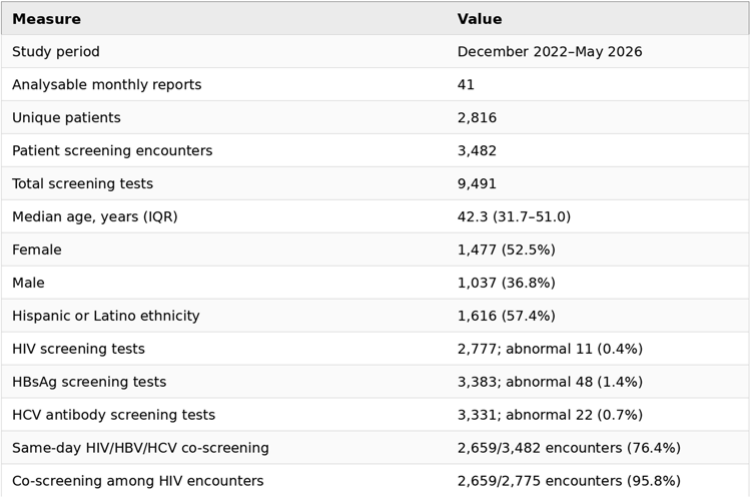
P288 | Summary of integrated HIV and viral hepatitis screening outcomes.

### P289 Abstract Withdrawn

### P290 Have I Taken Enough Preventive Measures? A Cross‐Sectional Study Related to HIV Testing Behaviour and Associated Factors Among Men Who Have Sex With Men in the Yogyakarta Region, Indonesia

Dwi Kartika Rukmi, Dewi Nur Amannah, Novita Nirmalasari, Afi Lutfiyati

Nursing Department of Universitas Jenderal Achmad Yani Yogyakarta, Sleman, Indonesia

**Background**: HIV new cases in Indonesia rose from 16,410 in 2023 to 35,415 in 2024, with men, including men who have sex with men (MSM), accounting for 71%. Regular HIV testing is crucial for determining HIV status and preventing transmission. This study aims to evaluate HIV prevention behaviours among MSM and their relationship with regular HIV testing.

**Methods**: A cross‐sectional survey was conducted among 175 MSM aged 18 years or older, selected via convenience sampling in five districts of Yogyakarta, Indonesia, from June to December 2024. The participants independently completed online questionnaires covering demographics, HIV knowledge (Table 1) and HIV prevention behaviours. The data were analysed using descriptive statistics, chi‐square tests and binary logistic regression.

**Results**: MSMs have adopted various preventive measures to avoid HIV acquisition, including consistent condom use during sex (35.4%) and participating in HIV education (97.7%) with 61.7% demonstrating a good level of HIV knowledge. Additionally, 94.3% have undergone HIV testing and 40.6% do so regularly. Among the 88% (154/175) MSMs who were aware of PrEP, 39,6% (61/154) reported having used it, with 70,5% (43/61) using it regularly. After the chi‐square test, Binary logistic regression between ever using PrEP (*p* < 0.001), routinely using PrEP (*p* = 0.007) and consistent condom use (*p* = 0.119) revealed that ever using PrEP (*p* = 0.02; AOR: 2.85; 95% CI: 1.18–6.86) is the most associated factor with HIV testing behaviour among MSM in Yogyakarta (Table 2).

**Conclusions**: The findings suggest that condoms and PrEP are the most common prevention strategies among MSM, though their use is not always consistent. The health authorities, health workers and HIV activists need to increase the dissemination of information about PrEP as a method of HIV transmission prevention, which is still uneven in Indonesia. The requirement for HIV testing before starting PrEP and the need for periodic HIV testing during PrEP use can increase HIV testing as well as the use of PrEP among MSM in Yogyakarta, Indonesia.

**TABLE 1:**
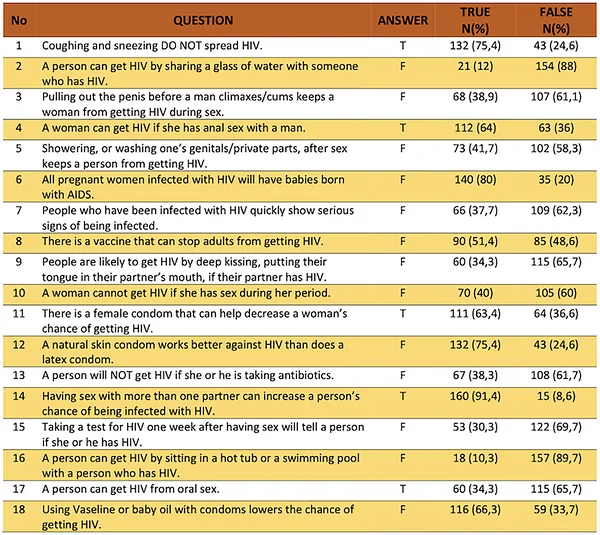
P290 | HIV knowledge among MSM.

**TABLE 2:**
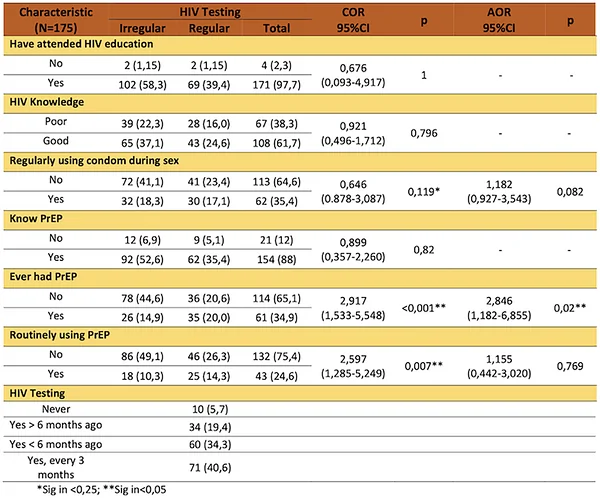
P290 | HIV testing and associated factors among MSM.

### P291 Group by Time Analyses of Aligning Sexual Agreement Behaviours With Using HIV Prevention and Care Strategies: Pooled Intervention Exposure Results From a Digital HIV Neutral Intervention Randomized Controlled Trial With Adult, Cisgender Male Couples

Jason Mitchell ^1^, Christofer Rodriguez^1^, Amber Sophus^2^, Zoran Bursac^1^

^1^Florida International University, Stempel College of Public Health and Social Work, Miami, FL, US; ^2^University of Texas – San Antonio, School of Public Health, San Antonio, TX, US

**Background**: LuvHub is a couples‐based, digital HIV neutral intervention that was evaluated via a nation‐wide, digital randomized controlled trial (RCT). LuvHub teaches couples critical skills to manage and align their HIV/STI vulnerabilities with their sexual pleasure and relationship needs.

**Methods**: 389 eligible and consented adult, cisgender sexual minority male couples throughout the US were enrolled in a 16‐month, digital RCT to assess the efficacy of LuvHub over time. Post baseline, couples were randomized to either an immediate intervention condition (G1) for 16 months or to an 8‐month waitlist control condition (G2). G2 was given access to use LuvHub from 8‐ to 16‐month. Assessments occurred every 4 months and were completed individually. Outcomes analysed included whether individuals’ perceptions about their own, as well as partner's sexual agreement and permitted behaviours had aligned with their respective use of HIV prevention and care strategies. Both items were captured using a Likert‐type response range of 1 = not very well to 5 = perfectly. Analyses compared outcomes over the 8‐month pooled time period when both G1 (Baseline–8‐month) and G2 (8‐ to 16‐month) had access to LuvHub. Generalized linear mixed models (GLMMs) were conducted to test for group, time and group by time interaction effects.

**Results**: The GLMMs did not detect a significant group effect or a significant group by time interaction effect for either outcome over the 8‐month pooled time period. Significant time effects were detected for both outcomes. Individuals’ perceptions about their sexual agreement and permitted behaviours aligning with their own use of HIV prevention and care strategies significantly increased over the 8‐month time period of having access to LuvHub (b = 0.24, SE 0.04, *p* < 0.001). Individual's perceptions about their partners’ sexual agreement and permitted behaviours aligning with their partners’ use of HIV prevention and care strategies also significantly increased over the same 8‐month pooled time period of having access to LuvHub (b = 0.27, SE 0.04, *p* < 0.001).

**Conclusions**: After having access to LuvHub for 8 months, couples’ alignment of their sexual agreement and permitted behaviours with their use of HIV prevention and care strategies improved over time. Improvements in these outcomes occurred in both trial arms (i.e. G1 and G2).

### P292 Abstract Withdrawn

### P293 A Compact Platform for Decentralized HIV Viral Load and Serological Testing Using Small‐Volume Blood Samples

Tim Cummins^1^, Ivan O'Connell^2^, Anita Schuler^2^, David McGuire^1^, Moira McCarthy^1^, Colin King^1^, Paul Free^1^, Xi Cao^1^, Lorcan Walsh^1^, John O'Driscoll^1^, Kaylyn Oshaben^1^, Margaret Aherne^1^, John Walshe^1^, Tara Dalton^1,3^, Federico García^4,5^, Ana Fuentes^4,5^, Daniel H. Appella^6^, Brian O'Farrell ^1^

^1^Altratech Limited, Cork, Ireland; ^2^Microelectronics Circuits Centre Ireland (MCCI), Tyndall National Institute, University College Cork, Cork, Ireland; ^3^Stokes Institute, University of Limerick, Limerick, Ireland; ^4^Servicio de Microbiología, Hospital Universitario Clinico San Cecilio, Granada, Spain; ^5^Ciber de Enfermedades Infecciosas, CIBERINFEC, Madrid, Spain; ^6^Synthetic Bioactive Molecules Section, Laboratory of Bioorganic Chemistry, National Institute of Diabetes and Digestive and Kidney Diseases, National Institutes of Health, Bethesda, MD, USA

**Background**: Decentralized HIV testing requires robust, low‐cost platforms that operate beyond central laboratories. A key gap is the ability to support both viral load and serological testing from small, minimally processed samples. We present a compact biosensing platform designed for molecular and serological detection at or near the point of care.

**Methods**: The platform is built on a standard CMOS chip using capacitive sensing of peptide nucleic acid (PNA)‐functionalized beads. Sequence‐specific capture is achieved via PNA probes on both beads and sensor surfaces. Assays were developed for HIV‐1 RNA detection following amplification and separately for HIV‐1/2 antibody detection using an adapted immunoassay workflow. Performance was evaluated in plasma, fingerstick‐scale whole blood with a simple filtration step and a pilot set of clinical samples.

**Results**: Robust detection of HIV‐1 RNA was achieved in plasma across a broad concentration range, with sensitivity down to 50 copies/mL. Performance was maintained under reduced amplification conditions and low sample input volumes. A streamlined workflow enabled RNA detection from ∼100 µL whole blood, supporting use with minimally processed fingerstick samples. In a pilot pre‐clinical evaluation, HIV RNA was detected in all HIV‐positive clinical samples tested (*n* = 6), with no signal observed in negative controls. Separately, proof‐of‐concept detection of HIV‐1/2 antibodies was demonstrated in patient plasma, indicating that the platform can be extended to serological assays.

**Conclusions**: This work demonstrates a compact CMOS‐based platform for decentralized HIV viral load testing with consistent analytical performance from small‐volume, minimally processed samples. Early proof‐of‐concept results also support extension to antibody detection. Together, these findings highlight the potential of a single platform to support both molecular and serological testing in near‐patient and community settings, with ongoing work focused on further clinical validation and assay integration.

### P294 Abstract Withdrawn

### P295 Abstract Withdrawn

### P296 “Arrested Because We Help”: Psychological Burden, Demotivation and Impact of Arrests on Men Who Have Sex With Men Community Counsellors in Dakar, Senegal—A Mixed‐Methods Study After the February 2026 Media Crisis

Élisabeth Kangou Diouf, Abdou Khoudia Diop

DLSI, Dakar, Senegal

**Background**: Community counsellors (peer educators) are key actors in the HIV care continuum for men who have sex with men (MSM) in Senegal. In February 2026, a national media crisis intensified stigma and arrests of people perceived as homosexual, directly affecting counsellors, patients and healthcare providers. We explored the impact of these arrests on counsellors’ mental health, motivation and HIV testing trends in Dakar [1, 2].

**Methods**: A mixed‐methods study was conducted in June 2026 across multiple HIV care facilities in Dakar. Semi‐structured interviews were carried out with MSM reference physicians and MSM community counsellors using a CAP (Knowledge, Attitudes, Practices) guide. HIV testing data (HTS_TST) were extracted from national databases for Q1 2022–2026, aggregated at Dakar region level. Thematic analysis was used for verbatim.

**Results**: Interviews revealed severe psychological burden among counsellors: anxiety, depression, paranoia, fear of arrest and desire to leave the country. Physicians reported numerous arrests of MSM patients and a flight of key populations to neighbouring countries. Counsellors described a significant drop in motivation, contractual non‐recognition and complete suspension of index testing due to fear of threats. HIV testing among MSM in Dakar showed a marked decline between the period before the crisis and Q1 2026.

**Conclusions**: Repeated arrests of MSM counsellors, patients and physicians create a climate of fear that affects frontline providers’ mental health and reduces testing access in Dakar. Urgent measures are needed: official professional cards, recognized employment contracts, law enforcement training on medical confidentiality and intersectoral advocacy.

**References**

1. Scheibe A, et al. Criminalisation of consensual same‐sex sexual practices and HIV vulnerability in sub‐Saharan Africa. J Int AIDS Soc. 2014;17(1):18858.

2. UNAIDS. Global AIDS Update 2025. Geneva: UNAIDS; 2025.

### P297 Clinical Outreach for Atlanta's Unhoused—An Opportunity to Increase HIV Detection and Linkage Through Collaborative Screening Strategies

Victoria Brown ^1^, Julia Blackwell^1^, Ian Fennell^1^, Breanna Lathrop^2^, Jennifer Mathew^2^, Michael Gibson^2^

^1^Health Programs, Good Samaritan Health Center, Atlanta, GA, US; ^2^Medical, Atlanta, GA, US

**Background**: Good Samaritan Health Center (Atlanta) is a community health centre that prioritizes the underserved, including predominantly low‐income, uninsured patients. In addition to its traditional population, Good Samaritan offers targeted health programming to provide clinical support, including HIV testing, to unhoused individuals. Unhoused populations are particularly vulnerable to the spread of sexually transmitted infections due to the lack of available preventative and clinical resources. Through a dedicated weekly homeless clinic and partnership with Atlanta Misson's Restoration House (satellite clinic), Good Samaritan has established targeted testing strategies to increase HIV screening and facilitate linkage to care among unhoused patients.

**Methods**: A retrospective study was conducted October 2025 to April 2026 of funding Y2 of Gilead Science's FOCUS grant using HIV testing data from Good Samaritan's EMR system. Data were stratified to explore differences in its traditional patient population and its unhoused patients. Testing and linkage outcomes were compared.

**Results**: 1276 HIV tests were conducted within the entire Good Samaritan health system, with 9 individuals having been identified as HIV positive. Of these 9 individuals, 7 were part of specialized unhoused programming (seropositivity_housed_ 0.16% vs. seropositivity_unhoused_ 0.56%). Among the 9 individuals with positive HIV test results, 4 were successfully linked to Ryan White patient care homes, while outcomes for the remaining 5 are pending or reflect loss to follow‐up.

**Conclusions**: Developing community partnerships to leverage clinical touchpoints for unhoused, vulnerable populations is a useful strategy for targeted, routine STI screening. Adding STI/STD testing to existing clinical pre‐screening eligibility to shelters may be a beneficial screening strategy. Additional strategies are needed to strengthen linkage outcomes among transient populations.

### P298 Abstract Withdrawn

### P299 Integrating HIV Care, Primary Care and Wrap‐Around Services to Support Engagement in Care in an LGBTQ+‐Centred Community Health Setting

Darya Fridman

The LGBTQ+ Center of Southern Nevada, Las Vegas, NV, US

**Background**: People living with HIV (PLWH) may experience barriers to engagement in care, including housing instability, behavioural health needs, substance use, transportation challenges and difficulties accessing insurance and support services. The LGBTQ+ Center of Southern Nevada developed an integrated model combining HIV care, primary care, peer navigation, Ryan White programme support, behavioural health services and community‐based wrap‐around services to strengthen engagement across the HIV care continuum.

**Methods**: Programme data from 2025 were analysed across two coordinated clinical sites: the Arlene Cooper Community Center STI Clinic and the Gavin J. Goorjian Community Health Center Primary Care Clinic. Descriptive analyses assessed HIV care engagement, transitions from STI services to primary care and utilization of selected support services among PLWH receiving care. All patients were assigned an HIV Peer Navigator and contacted by the Ryan White manager. Providers conducted social needs assessments and facilitated access to behavioural health services, housing support, transportation assistance, food services, harm‐reduction programmes and other community resources.

**Results**: A total of 461 PLWH received care during 2025. Of these, 204 were newly engaged in care at one of the clinics. The STI clinic referred 148 PLWH to the primary care clinic to support continuity of care. All 204 newly engaged patients attended an initial clinical appointment, completed laboratory testing and received prescriptions. Of these, 169 patients (82.8%) continued in ongoing care with more than one clinic visit during the reporting period (Figure 1). All patients received outreach from the Ryan White manager. Forty individuals received assistance with Ryan White enrolment or re‐enrolment, 42 experienced housing instability, 24 reported injecting drug use and were connected to harm‐reduction services and 8 were connected with behavioural health services.

**Conclusions**: An integrated HIV care model combining primary care, peer navigation, Ryan White programme support, behavioural health services and wrap‐around services facilitated linkage and ongoing engagement in care among PLWH. Coordinated service delivery across clinical and community‐based programmes may help address barriers to care and strengthen continuity across the HIV care continuum.

**FIGURE 1:**
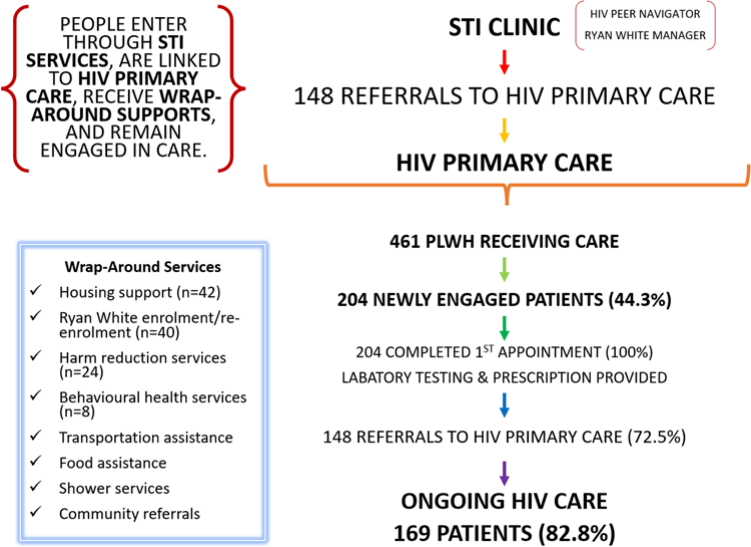
P299 | Integrated HIV care and wrap‐around services in an LGBTQ+‐centred community health setting, 2025.

## Public Health Strategies and Options

### P300 The Paradox of High Coverage: Persistent HCV Reinfection in Care‐Engaged Men Who Have Sex With Men Networks

Daniele Calzavara ^1,2^, Alessandro Soria^3^, Roberto Rossotti^4^, Nicoletta Policeck^5^

^1^conigli bianchi APS, Roma, Italy; ^2^EATG, Brussels, Belgium; ^3^Dipartimento di Malattie InfettiveIRCCS SAN GERARDO, Monza, Italy; ^4^Dipartimento di Diagnostica e Sanità Pubblica, Università di Verona, Verona, Italy; ^5^INDIPENDENCE ACTIVIST, Venezia, Italy

**Background**: Since direct‐acting antiviral (DAA) regimens entered European practice in 2014, hepatitis C virus (HCV) control in men who have sex with men (MSM) is often framed as a treatment‐led elimination story [1, 2]. Yet a paradox remains: primary incidence can decline while reinfection persists in MSM linked to HIV care, PrEP and treatment [3–6]. We contrasted national/multicentric cohorts with metropolitan hubs to examine the gap between biomedical coverage and network control [7].

**Methods**: We conducted a structured literature review of post‐2010 European studies identified from PubMed and Europe PMC exports and checked in full text. Studies came from Germany, Switzerland, France, Italy, the Netherlands and Spain. We extracted paired infection and reinfection signals among MSM living with HIV, MSM using PrEP and MSM with prior HCV exposure, classifying observations within national/multicentric and metropolitan‐hub frameworks. A separate phylogeny table captured clustering, overlap and cross‐city or cross‐country reseeding.

**Results**: Across national/multicentric cohorts, primary HCV incidence fell to low levels after DAA scale‐up, exemplified by 0.37/100 PY in the Dutch national PrEP programme [8]. Reinfection nevertheless remained substantial in care‐engaged MSM with prior HCV exposure, reaching 15.5/100 PY in the German recent‐HCV cohort, with multiple reinfections in 31.3% of cases [9]. Urban hubs showed a sharper signal: second and subsequent reinfections reached 38.3/100 PY in Amsterdam after primary HCV clearance [10] and Madrid data showed reinfection exceeding incident HCV, with the highest rate in PrEP users [11] (Table 1). Phylogenetic studies showed MSM‐dominant clustering, overlap between MSM living with HIV and MSM without HIV or using PrEP and repeated reseeding between cities [6, 12–18].

**Conclusions**: The central paradox is not that HCV persists in populations excluded from care, but that it persists in people visible to services. This suggests a residual urban MSM reservoir in which HCV cure reduces viraemia without preventing rapid re‐exposure, recurrent reinfection and onward transmission [19–21]. Annual HCV burden may increasingly reflect recurrent rather than first infections, helping explain why MSM networks remain off track for 2030 HCV elimination targets despite biomedical coverage. HCV elimination strategies should pair DAA access with tailored, network‐level interventions beyond the clinic, improving peer‐led experiences, to reduce re‐exposure in connected MSM urban hubs.

**References**

1. Braun et al. 2021. A Treatment‐as‐Prevention Trial to eliminate hepatitis C among men who have sex with men living with human immunodeficiency virus (HIV) in the Swiss HIV Cohort Study. Clinical Infectious Diseases. DOI: 10.1093/cid/ciaa1124.

2. Kusejko et al. 2022. Sustained effect on hepatitis C elimination among men who have sex with men in the Swiss HIV Cohort Study: A systematic re‐screening for hepatitis C RNA two years following a nation‐wide elimination program. Clinical Infectious Diseases. DOI: 10.1093/cid/ciac273.

3. Pradat et al. 2018. Incidence of new hepatitis C virus infection is still increasing in French MSM living with HIV. AIDS. DOI: 10.1097/QAD.0000000000001789.

4. Ingiliz et al. 2020. Reinfection with the hepatitis C virus in men who have sex with men after successful treatment with direct‐acting antivirals in Germany: Current incidence rates, compared with rates during the interferon era. Clinical Infectious Diseases. DOI: 10.1093/cid/ciz949.

5. Smit et al. 2021. Netherlands ATHENA MSM with HIV and previous HCV. Lancet HIV. DOI: 10.1016/S2352‐3018(20)30301‐5.

6. Popping et al. 2022. Persistent transmission of HCV among men who have sex with men despite widespread screening and treatment with direct‐acting antivirals. Viruses. DOI: 10.3390/v14091953.

7. Lozano Rincon et al. 2019. Hepatitis C: New diagnosis and seroconversions in a Madrid sexually transmitted diseases clinic. Revista Espanola de Quimioterapia. PMID: 31558009.

8. Hage et al. 2024. Hepatitis C virus infection is uncommon at baseline and during follow‐up among individuals using PrEP in the Dutch national PrEP programme between 2019 and 2022. Sexually Transmitted Infections. DOI: 10.1136/sextrans‐2024‐056169.

9. Ingiliz et al. 2024. Recently acquired HCV infection in men who have sex with men in Germany in the direct‐acting antivirals era and during the COVID‐19 pandemic. HIV Medicine. DOI: 10.1111/hiv.13651.

10. Newsum et al. 2021. Amsterdam/MOSAIC HCV reinfection cohort. Clinical Infectious Diseases. DOI: 10.1093/cid/ciaa645.

11. Ryan et al. 2025. Madrid MSM with HIV and PrEP users, 2022–2024. Open Forum Infectious Diseases. DOI: 10.1093/ofid/ofaf678.

12. Hoornenborg et al. 2017. MSM starting preexposure prophylaxis are at risk of hepatitis C virus infection. AIDS. DOI: 10.1097/QAD.0000000000001522.

13. Hage et al. 2025. Incidence of hepatitis C virus infection and associated determinants among men who have sex with men without HIV in Amsterdam, the Netherlands, between 2012 and 2021. European Journal of Gastroenterology & Hepatology. DOI: 10.1097/MEG.0000000000003008.

14. Vogel et al. 2010. German HCV‐4d network among HIV‐positive MSM with acute HCV. DOI: 10.1111/j.1478‐3231.2010.02305.x.

15. Caro‐Perez et al. 2017. Barcelona acute HCV outbreak among HIV‐positive MSM. Journal of Clinical Virology. DOI: 10.1016/j.jcv.2017.05.008.

16. Visseaux et al. 2020. Paris–Netherlands HCV genotype 4d link. Clinical Microbiology and Infection. DOI: 10.1016/j.cmi.2020.01.034.

17. Kouyos et al. 2014. Clustering of HCV coinfections on HIV phylogeny indicates domestic and sexual transmission of HCV. International Journal of Epidemiology. DOI: 10.1093/ije/dyt276.

18. Koopsen et al. 2023. Hepatitis C virus transmission between eight high‐income countries among men who have sex with men: A whole‐genome analysis. Lancet Microbe. DOI: 10.1016/S2666‐5247(23)00108‐8.

19. Vanhommerig et al. 2015. Dutch MSM risk‐factor study. Open Forum Infectious Diseases. DOI: 10.1093/ofid/ofv115.

20. Barcelona HIV cohort study. 2021. Infectious diseases and therapy. DOI: 10.1007/s40121‐021‐00448‐0.

21. German NoCo reinfection abstract. CROI 2021. Source record: R00117.

22. d'Arminio Monforte et al. 2023. Is HCV elimination among persons living with HIV feasible? Data from the NoCo study in the setting of the ICONA cohort. Liver International. DOI: 10.1111/liv.15700.

**TABLE 1:**
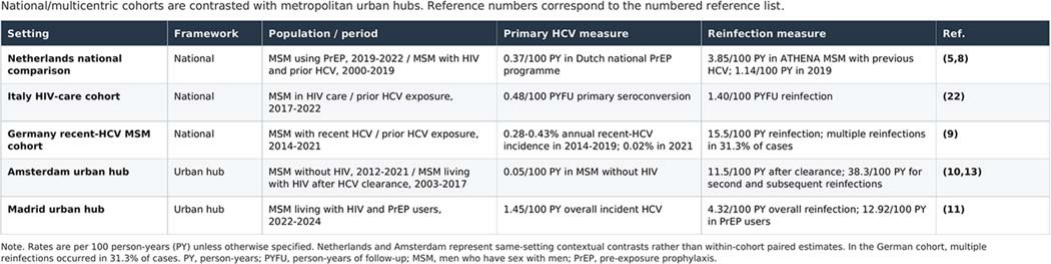
P300 | Core epidemiologic anchors: primary HCV incidence and reinfection in care‐engaged MSM cohorts.

### P301 Long‐Term Effectiveness of the Quadrivalent HPV Vaccine Against Anal Dysplastic Lesions in HIV‐MSM From a Phase 4 RCT: A Propensity Score‐Matched Post‐RCT Cohort Follow‐Up Study

Carmen Hidalgo‐Tenorio ^1^, Saioa Igartua^2^, Mohamed Omar^3^, SamPedro Antonio^4^, Javier Lopez Hidalgo^5^, Jessica Ramirez Taboada^2^

^1^Infectious Diseases Service, Granada, Spain; ^2^Internal Medicine Service, Granada, Spain; ^3^Infectious Diseases Unit, Jaén, Spain; ^4^Microbiology Service, Granada, Spain; ^5^Pathology Service, Granada, Spain

**Background**: HIV‐positive MSM face a 30–100‐fold higher anal cancer incidence than the general population. The quadrivalent HPV vaccine (qHPVv; Gardasil) demonstrated immunogenicity and safety in our double‐blind phase 4 RCT (ISRCTN14732216 [http://www.isrctn.com/ISRCTN14732216]). Long‐term effectiveness beyond a decade had not previously been reported.

**Methods**: Post‐RCT observational cohort. All RCT participants without baseline HSIL were included (2012–2014) (*n* = 129; 66 vaccinated, 63 non‐vaccinated). The efficacy population comprised 88 participants with more than 48 months of follow‐up (45 vaccinated/43 non‐vaccinated; median 133 months, ∼11 years). During the 4‐year RCT, all participants underwent annual anal HPV PCR, cytology and high‐resolution anoscopy (HRA) with biopsy. Thereafter, annual cytology and HPV PCR were performed; HRA was triggered by abnormal cytology or positive HPV PCR, while those with normal results continued with annual cytology and PCR the following year. Propensity score matching (PSM) was used to assess baseline comparability between arms and to test whether completing longer follow‐up was independent of vaccination. Primary endpoint: HSIL incidence per 100,000 person‐years (PY). Secondary: new LSIL (AIN1), anogenital condyloma and HPV6/11/16/18 acquisition.

**Results**: Both arms were well balanced: age 39.5 versus 40.8 years (*p* = 0.591), AIDS‐defining events 42.4% versus 36.4% (*p* = 0.614), CD4 724 versus 777 cells/µL (*p* = 0.438), undetectable viral load 90.9% versus 90.9% (*p* = 1.000), AIN1 at baseline biopsy 54.5% versus 42.4% (*p* = 0.325), HR‐HPV prevalence 75.8% versus 66.7% (*p* = 0.415). Follow‐up completion was independent of vaccination status. In the efficacy population, HSIL incidence was 677 versus 233 per 100,000 PY (vaccinated vs. non‐vaccinated; RR 2.91, 95% CI: 0.30–27.93; *p* = 0.625). Condyloma: 2481 versus 3031/100,000 PY (*p* = 0.686). New AIN1: 1804 versus 1399/100,000 PY (*p* = 0.791). No anal cancers occurred in either arm. New HPV6/11/16/18 acquisition was similar in both arms (*p* = 1; *p* = 0.242; *p* = 0.242; *p* = 1, receptivity). No statistically significant differences were found for any endpoint.

**Conclusions**: Both arms were fully comparable at baseline and there was no differential attrition, which supports the robustness of these results. Despite 11 years of structured follow‐up, the qHPVv did not confer significant long‐term protection against anal lesions. Structured HRA‐based anal cancer surveillance must be maintained in all HIV‐positive MSM regardless of vaccination status.

### P302 The Role of Digital Tools in Facilitating PrEP Uptake: A Systematic Review and Meta‐Analysis

Victoria Tittle ^1,2^, Alejandro De Gea Grela^3^, Andrea Perez Navarro^4^, Sarah Fidler^1^, Andrew Hill^5^, Charlotte‐Eve Short^1,6^, Fiona Burns^7^, Marta Boffito^1,6^

^1^Imperial College London, London, UK; ^2^56 Dean Street, Chelsea and Westminster NHS Trust, London, UK; ^3^Servicio de Medicina Interna, Hospital Universitario la Paz, Madrid, Spain; ^4^Imperial College Healthcare, London, UK; ^5^University of Liverpool, Liverpool, UK; ^6^Chelsea and Westminster NHS Trust, London, UK; ^7^University College London, London, UK

**Background**: Pre‐exposure prophylaxis (PrEP) initiation and continuation rates have been reported as low as 35% [1]. Prevention and digital interventions are part of the 2025 HIV action plan, but there are limited data on if and how, digital tools support PrEP initiations and continuations [2]. This is a systematic review and meta‐analysis to assess whether digital tools facilitate PrEP uptake and persistence.

**Methods**: A systematic search of Medline, Embase, Scopus, Global health, PsycInfo, Cochrane Library and the Clinical Trials Register of randomized controlled trials since 2012. Eligible trials included participants aged 13 years old and older, who were not living with HIV and would benefit from PrEP, comparing digital tool intervention studies with standard of care. Outcome measure was initiation PrEP and/or PrEP continuation. Random‐effects models explored odds ratios (OR) for initiating PrEP and continuing PrEP. Heterogeneity was explored with meta‐regression (duration of follow‐up: months) and subgroup analysis (digital modality: mobile applications, text messages (SMS), other; study country; populations). Risk‐of bias and GRADE assessments reviewed quality of evidence.

**Results**: Table 1 presents the outcome of study selection with 24 studies meeting criteria, comprising of 10,336 participants (5422 in intervention and 4914 in control group at baseline). Eleven studies (1 study had two intervention arms: 12 effect estimates) reported PrEP initiation, 17 studies reported PrEP continuation (4 studies reported both). Pooled OR for PrEP initiation with digital tool intervention was 1.49 (95% confidence intervals, CI, 1.07–2.08) with *I* ^2^ = 61.2% (*p* = 0.006) and for PrEP continuation was 1.74 (95% CI: 1.31–2.37) with *I* ^2^ = 69.3% (*p* ≤ 0.001) (Figure 1). The type of digital modality may explain some of the heterogeneity in PrEP continuations from subgroup analyses (*I* ^2^ = 69.2%, *p* = 0.03), but subgroup analyses for duration of study follow‐up, study country and populations did not explain heterogeneity in either PrEP initiations or PrEP continuations. GRADE assessment report low quality of studies for both PrEP initiation and PrEP continuation.

**Conclusions**: Digital tools may support PrEP initiation and continuation: people using digital tools were 74% more likely to continue PrEP than those receiving standard of care. However, high heterogeneity requires these results to be interpreted with caution.

**References**

1. UKHSA. HIV testing, PrEP, new HIV diagnoses and care outcomes for people accessing HIV services: 2025 report. https://www.gov.uk/government/statistics/hiv‐annual‐data/hiv‐testing‐prep‐new‐hiv‐diagnoses‐and‐care‐outcomes‐for‐people‐accessing‐hiv‐services‐2025‐report (accessed 19/06/2026).

2. Department of Health & Social Care. HIV Action Plan for England, 2025 to 2030. (2025) https://www.gov.uk/government/publications/hiv‐action‐plan‐for‐england‐2025‐to‐2030/hiv‐action‐plan‐for‐england‐2025‐to‐2030#ministerial‐foreword (accessed 19/06/2026).[Fig jia270211-fig-0339]

**FIGURE 1:**
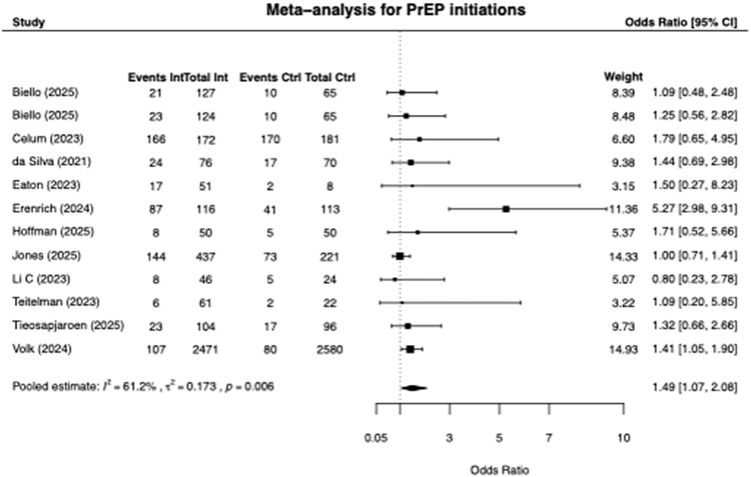
P302 | Meta‐analyses for PrEP initiations and PrEP continuations.

**FIGURE 2 jia270211-fig-0339:**
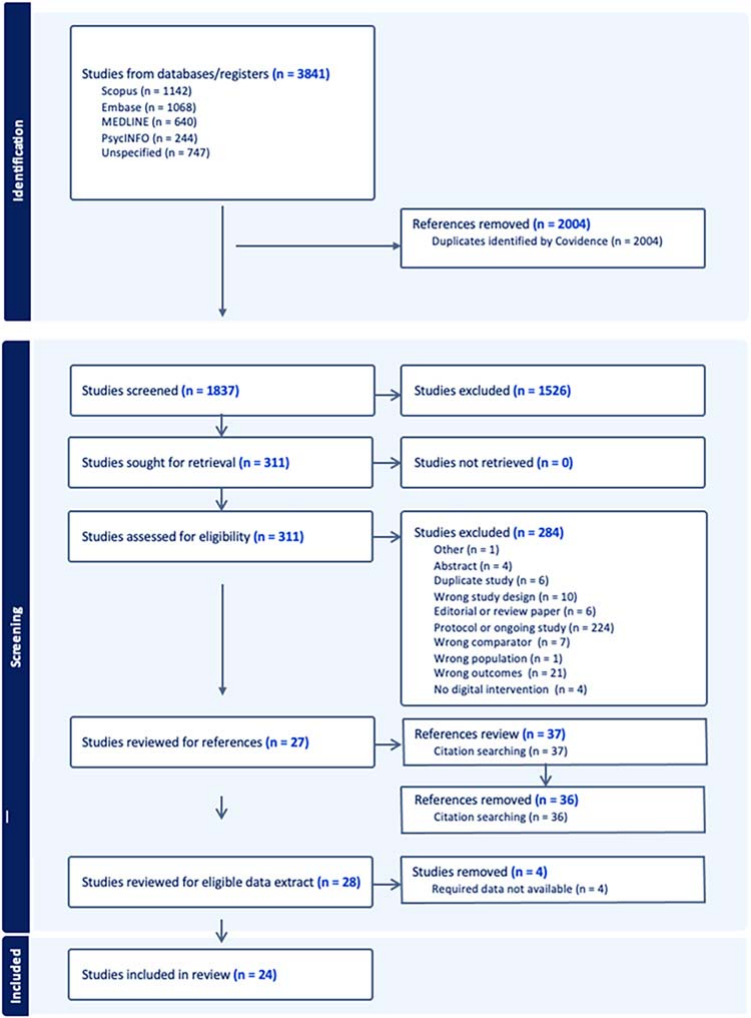
P302 | Outcome of study selection.

### P303 The Impact of Migrant‐Inclusive Policy in Botswana's Universal HIV Treatment Programme: A Retrospective Analysis of the National HIV Treatment Programme Data (2016–2022)

Patrick T. Mokgethi ^1,2^, Ontlametse T. Choga^1^, Wonderful T. Choga^1^, Dorcas Maruapula^1^, Natasha Moraka‐Mankge^1^, Graceful Mulenga^1^, Kesaobaka Molebatsi^1^, Catherine Koofhethile^1,3^, Sununguko W. Mpoloka^2^, Tafireyi Marukutira^1,4^, Sikhulile Moyo^1,3^, Simani Gaseitsiwe^1,3^

^1^Botswana Harvard Health Partnership, Gaborone, Botswana; ^2^Department of Biological Sciences, University of Botswana, Gaborone, Botswana; ^3^Department of Immunology and Infectious Diseases, Harvard T.H. Chan School of Public Health, Boston, MA, US; ^4^Burnet Institute, Melbourne, Australia

**Background**: Migrants were historically excluded from free antiretroviral therapy (ART) in Botswana. In December 2019, Botswana extended free HIV testing and treatment to migrants. This study intends to assess the impact of including migrants in the national HIV treatment programme by determining ART coverage, baseline CD4 cell counts at enrolment and virologic outcomes before and after the policy.

**Methods**: Using a retrospective longitudinal observational dataset of all people living with HIV (PLWH) enrolled in the national treatment programme from 2016 to 2022, with citizen status documented and aged 18+ years, the dataset was classified into two categories of individuals enrolled on ART using enrolment year: pre‐2019 (citizen‐only policy) and post‐2019 (migrant‐inclusive policy). The baseline CD4 cell count at ART initiation was compared between pre‐ and post‐2019 eras using a modified Poisson and linear regression, adjusted for age, sex and facility, with a difference‐in‐differences (DiD) model to provide a controlled comparison. The virologic outcomes and mortality were evaluated using modified Poisson and Cox models, adjusted for age, sex and baseline CD4 cell counts.

**Results**: Among 87,063 PLWH, 40,165 had baseline CD4 cell count data, while 50,043 individuals had VL data. Migrant ART initiation rates increase fivefold from 1.3–4.4% pre‐2019 to 19–20% post‐2019. The study revealed that including migrants in the treatment programme led to a median increase of 54.2 cells/mm^3^ (CI 37.7−70.6; *p*‐value < 0.001) in CD4 cell counts. The proportion of migrants presenting with advanced HIV disease dropped from 22.9% pre‐2019 to 17.7% in post‐2019 (adjusted RR 0.76, 95% CI: 0.63–0.90; *p* = 0.002), with 24% lower risk of advanced disease at ART initiation. VL measurement capture among migrants increased from 27.7% to 58.5% at 6 months and 40.5% to 82.6% at 36 months (all adjusted RRs 1.86–2.25; *p* < 0.001) after the policy shift. Virologic suppression (<400 copies/mL) remained high (≥94%) with no significant post‐2019 change. Mortality was low and unchanged post‐2019.

**Conclusions**: Extending Botswana's ART programme to migrants reduced advanced HIV disease at initiation and significantly improved viral load monitoring and sustained viral suppression. Removing citizenship‐based barriers reduces health inequities and supports epidemic control towards the UNAIDS 95‐95‐95 targets.

### P304 Timing of HIV Acquisition Among Migrants in the Netherlands

Ard van Sighem ^1,2^, Eline Op de Coul^3^, Daniela Bezemer^1,2^, Marc van der Valk^1,4^, on behalf of ATHENA Observational HIV Cohort

^1^Stichting HIV Monitoring, Amsterdam, Netherlands; ^2^Amsterdam institute for Immunology & Infectious diseases, Amsterdam, Netherlands; ^3^National Institute for Public Health and the Environment, Bilthoven, Netherlands; ^4^Amsterdam UMC, location University of Amsterdam, Department of Infectious Diseases, Amsterdam, Netherlands

**Background**: Migrant populations are disproportionately affected by HIV. Potential hardships experienced during migration and settling may be associated with increased risk of HIV acquisition. Here we investigate time of HIV acquisition relative to time of arrival in the Netherlands.

**Methods**: Data on all foreign‐born individuals (migrants) who arrived and were diagnosed with HIV‐1 in the Netherlands in 2000–2024 were retrieved from the ATHENA cohort, excluding migrants with confirmed pre‐migration diagnosis. For each individual, a probability distribution of HIV acquisition dates was estimated based on sex, region of origin, date of last negative HIV test, age, CD4‐count and concurrent AIDS event at diagnosis using a CD4‐cell depletion method in the ECDC HIV Platform tool. Fifty HIV acquisition dates were randomly drawn per individual, creating 50 datasets that were analysed separately. Outcomes were pooled using Rubin's rules. Probabilities of acquiring HIV within 5 years pre‐arrival (assuming migration may take several years) or within 1‐year post‐arrival (settling) were analysed with multivariable logistic models.

**Results**: The 50 datasets included 4212 migrants (range 4163–4256) on average. Most migrants originated from sub‐Saharan Africa (1487, 35.3%), South America (648, 15.4%), Central Europe (442, 10.5%), Asia (440, 10.4%) and Western Europe (438, 10.4%). Proportions with HIV acquisition before arrival were highest in women, 67.8% (95% confidence interval [CI], 64.6–69.6) and other men, 57.7% (54.0–61.7) (Figure 1 and Table 1). HIV acquisition post‐arrival was most common among men who have sex with men (MSM), 14.7% (12.8–16.9) within 1 year, 42.0% (39.4–44.7) within 5 years and transgender individuals, 17.3% (11.2–26.8) within 1 year, 40.6% (31.8–51.8) within 5 years. In multivariable models, other men (adjusted odds ratio 1.47, 95% CI: 1.12–1.92) and women (1.79, 1.36–2.36) had higher odds of HIV acquisition within 5 years before arrival compared to MSM. Associations were inverse and less pronounced for HIV acquisition within 1‐year post‐arrival. Age and origin were not independently associated.

**Conclusions**: A high proportion of migrants, in particular women and men other than MSM, acquire their HIV infection before arrival in the Netherlands, suggesting HIV testing upon arrival could be warranted. MSM and transgender individuals are more likely to acquire HIV post‐arrival and, may, in addition to testing, benefit from pre‐exposure prophylaxis.

**FIGURE 1:**
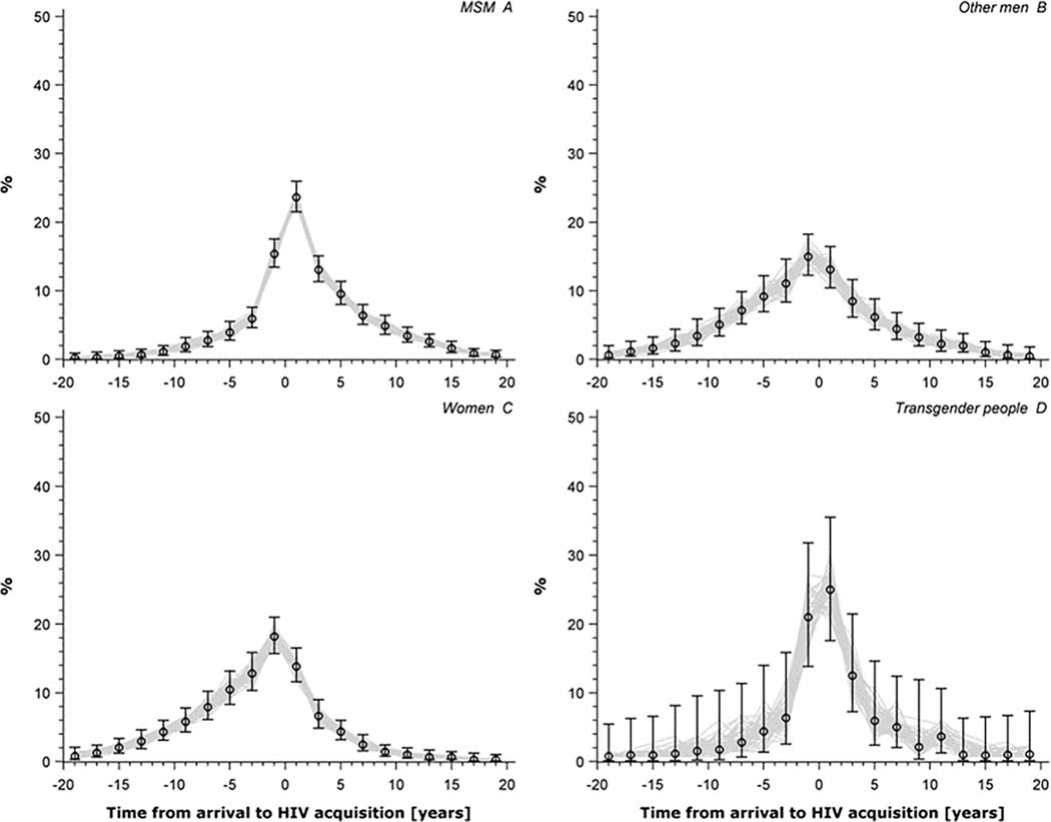
P304 | Proportion of individuals who acquired HIV between 20 years before and 20 years after arrival in the Netherlands, (A) men who have sex with men (MSM), (B) other men, (C) women, (D) transgender people. Time between arrival and HIV acquisition was stratified in 2‐year intervals; light grey lines represent the 50 datasets; circles represent pooled estimates of the proportion with uncertainty bounds.

**TABLE 1:**
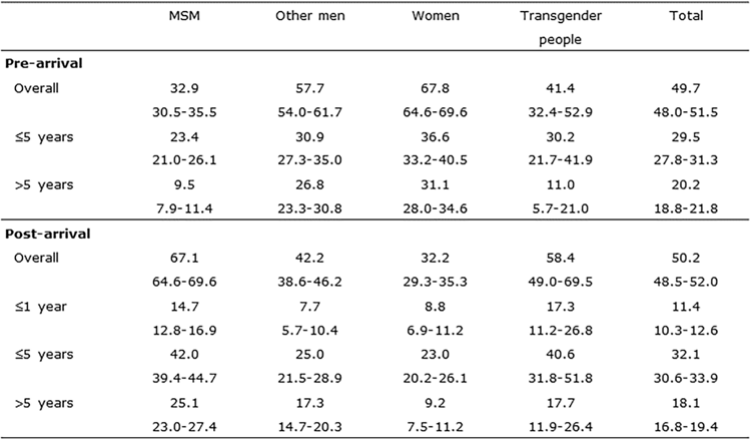
P304 | Proportion (with 95% confidence intervals) of individuals who acquired HIV before or after arrival in the Netherlands, stratified by group, across the 50 datasets.

### P305 Different Viral Load Criteria to Trigger Resistance Testing—Implications for HIV Programmes in Southern Africa

Niklaus D. Labhardt ^1,2^, Anna Klicpera^1,2^, Maren Röhl^1,2^, Blaise Lukau^3^, Lorena Urda^1,2^, Giuliana Sanchez Samaniego^1,2^, Moleboheng Mokebe^3^, Lipontso Motaboli^3^, Mosa M. Monyake^4^, Tapiwa Tarumbiswa^4^, Irene Ayakaka^3^, Jochen Ehmer^5^, Roger Kouyos^6,7^, Gioia Wick^6,7^, Tom Loosli^6,7^, Richard Lessells^8,9^, Jennifer Giandhari^9^, Maria Selebalo^4^, Nadine Tschumi^1,2^, Jennifer A. Brown^1,6^

^1^Division of Clinical Epidemiology, Department of Clinical Research, University Hospital Basel, Basel, Switzerland; ^2^Department of Clinical Research, University of Basel, Basel, Switzerland; ^3^SolidarMed, Partnerships for Health, Maseru, Lesotho; ^4^Ministry of Health Lesotho, Maseru, Lesotho; ^5^SolidarMed, Partnerships for Health, Lucerne, Switzerland; ^6^Department of Infectious Diseases and Hospital Epidemiology, University Hospital Zurich, Zurich, Switzerland; ^7^Institute of Medical Virology, University of Zurich, Zurich, Switzerland; ^8^Centre for the AIDS Programme of Research in South Africa (CAPRISA), University of KwaZulu‐Natal, Durban, South Africa; ^9^KwaZulu‐Natal Research Innovation and Sequencing Platform, University of KwaZulu‐Natal, Durban, South Africa

**Background**: Viral load (VL) thresholds defining virological failure (VF) determine eligibility for resistance testing. The World Health Organization (WHO) currently defines VF as two consecutive VLs ≥1000 copies/mL [1], but lowering this to ≥200 copies/mL [2] is under consideration. We assessed how different thresholds affect cumulative 5‐year incidence of VF and detection of dolutegravir resistance in Lesotho.

**Methods**: The Viral Load Cohort of North‐East Lesotho (VICONEL) is an open routine‐care cohort covering three districts [3]. We included people with HIV of any age who started dolutegravir‐based antiretroviral therapy before 2024 and had ≥2 subsequent VL measurements. Participants were censored at their last VL measurement. We used Kaplan−Meier estimation with left‐truncation to adjust for late cohort entry and estimated cumulative 5‐year incidence of VF, defined as: (i) two consecutive VLs ≥1000 copies/mL (WHO criteria), (ii) two consecutive VLs ≥200 copies/mL, or (iii) one VL ≥500 copies/mL after at least one prior VL ≥50 copies/mL (not necessarily consecutive). We tested bio‐banked samples for dolutegravir resistance, defined as at least low‐level resistance according to Stanford Database V10.2 with ≥5% variant frequency. The prevalence of detected dolutegravir resistance and the number of required resistance tests were calculated for each VF definition.

**Results**: Among 23,437 participants (64% female, median age 45.6 years [IQR 20.7], median ART duration 9.0 years [IQR 6.8]), cumulative 5‐year incidence of VF using definition (i), (ii) and (iii) was 1.29% (95% CI: 1.12–1.46%), 2.14% (95% CI: 1.93–2.36%) and 3.58% (95% CI: 3.28–3.89%), respectively (Figure 1). Dolutegravir resistance prevalence was similar across VF definitions; however, of 24 cases identified using definition (iii), 10 (42%) or 3 (13%) would be missed using definitions (i) or (ii), respectively (Table 1). Extrapolating to 100,000 people over 5 years, lowering the WHO threshold to 200 copies/mL would increase the number of individuals requiring resistance testing from 1131 to 1924 and anticipated detected cases with dolutegravir resistance from 114 to 210.

**Conclusions**: Lowering the WHO threshold from 1000 to 200 copies/mL would increase required resistance testing by 70% and detection of dolutegravir‐resistant HIV by 84%, with important implications for resource allocation in HIV programmes in southern Africa.

**References**

1. Consolidated Guidelines on HIV Prevention, Testing, Treatment, Service Delivery and Monitoring: Recommendations for a Public Health Approach. World Health Organization; 2021. Accessed August 30, 2021. https://www.who.int/publications/i/item/9789240031593

2. EACS: Virological Failure. Accessed June 25, 2026. https://eacs.sanfordguide.com/en/eacs‐hiv/art/eacs‐virological‐failure

3. Brown JA, Motaboli L, Lerotholi M, et al. Cohort profile: the Viral load Cohort North‐East Lesotho (VICONEL) from 2016 to 2023 ‐ cohort description, test volumes, predictors of viraemia and the road ahead. BMJ Open. 2025;15(2):e085404. doi:10.1136/bmjopen‐2024‐085404

**FIGURE 1:**
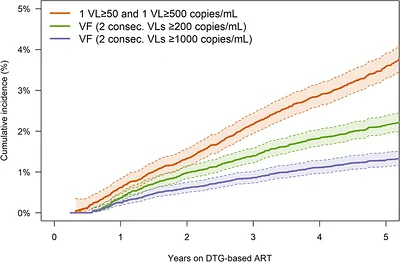
P305 | Incidence of virologic failure among the 23,437 eligible VICONEL participants using different viral load thresholds.

**TABLE 1:**
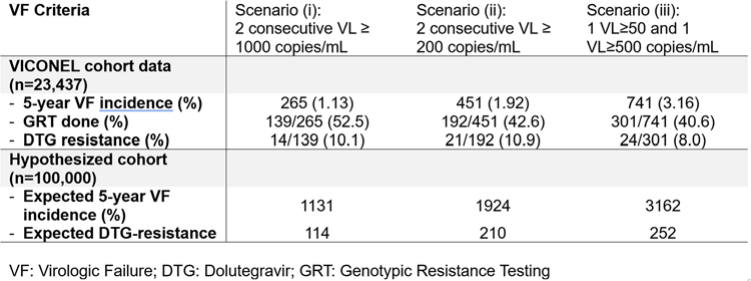
P305 | Eligibility for HIV resistance testing and prevalence of dolutegravir resistance over 5 years in VICONEL and in a hypothetical cohort of 100,000 participants over 5 years.

### P306 Capturing Mortality Among People Living With HIV Across Europe: A Comparison of Routine Surveillance Data With an International Cohort Study

Bastian Neesgaard ^1^, Joanne Reekie^1^, Teymur Noori^2^, Erika Duffell^2^, Disa Hansson^2^, Anemarie Rinder Stengaard^1^, Dorthe Raben^1^, Lene Ryom Nielsen^1,3^, Juliana Reyes‐Urueña^2^, on behalf of RESPOND and the EU/EEA HIV Networks

^1^CHIP, Centre of Excellence for Health, Immunity and Infections, Copenhagen, Denmark; ^2^European Centre for Disease Prevention and Control (ECDC), Stockholm, Sweden; ^3^Department of Infectious Diseases 144, Hvidovre University Hospital, Copenhagen, Denmark

**Background**: Accurate monitoring of mortality among people living with HIV is essential for informing public health actions and assessing progress towards the global targets. We compared the reporting of HIV‐related deaths to the European Surveillance system (TESSy) and vital statistics data (Eurostat) to an international HIV cohort with validated cause of death reporting (RESPOND), to understand the strengths and limitations of EU/EEA‐level surveillance.

**Methods**: We compared standardized mortality rates (SMRs) among people living with HIV in the EU/EEA area between 2018 and 2022, using data from RESPOND, TESSy and Eurostat. Annual SMRs were calculated among adults (≥20 years) per 1000 person‐years of follow‐up, based on the number of deaths. Cause of death in RESPOND was determined via CoDe methodology [1] and for TESSy from country reports/prior AIDS notifications. Eurostat rates were based on HIV‐specific ICD‐10 codes (B20–B24). Results were standardized to a reference population derived from the mean number of people living with diagnosed HIV in reporting countries [2].

**Results**: During the study period, there were 813 deaths among the 20,449 individuals in RESPOND (SMR 8.94, 95% CI: 7.80–10.07), 10,032 deaths among 444,687 individuals with an HIV notification reported to TESSy (SMR 6.95, 95% CI: 6.77–7.14) and 8480 deaths reported to Eurostat (SMR 2.82, 95% CI: 2.76–2.88). SMR for all‐cause mortality were similar between RESPOND and TESSy (Figure 1). 48% of the deaths reported to TESSy were classed as AIDS‐related compared to 18% in RESPOND (Table 1). Subsequently, the rate of AIDS‐related mortality was significantly higher for TESSy (SMR 3.37, 95% CI: 3.24–3.50 vs. SMR 1.48, 95% CI: 0.98–1.97). The SMR for Eurostat was consistently lower than for all‐cause mortality in RESPOND but higher than AIDS‐related mortality.

**Conclusions**: Mortality estimates derived from different European data sources vary considerably. TESSy may overestimate AIDS‐related mortality and underestimate all‐cause mortality due to misclassification and incomplete reporting, while Eurostat estimates were more conservative. Cohort data offer centrally validated data but may suffer from selection bias. Strengthening linkages between HIV surveillance systems and national mortality registries, alongside harmonized approaches to cause‐of‐death reporting, could improve mortality data and enhance the value of EU/EEA‐level monitoring of HIV‐related mortality.

**References**

1. Kowalska JD, Friis‐Møller N, Kirk O, et al. The coding causes of death in HIV (code) project initial results and evaluation of methodology. *Epidemiology* 2011; 22. DOI:10.1097/EDE.0b013e31821b5332.

2. European Centre for Disease Prevention and Control/WHO Regional Office for Europe. HIV/AIDS surveillance in Europe 2025 – 2024 data. Stockholm: ECDC; 2025.

**FIGURE 1:**
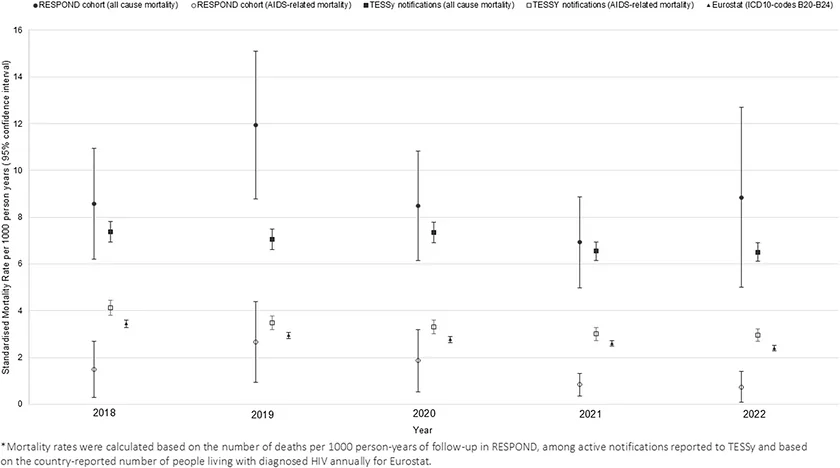
P306 | Standardized rate* of mortality in the RESPOND cohort, among TESSy notifications and based on HIV specific ICD‐10 codes reported to Eurostat, 2018–2022.

**TABLE 1:**
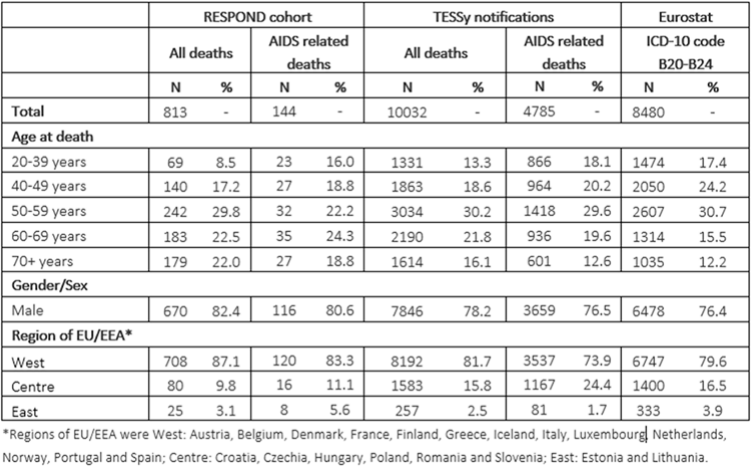
P306 | Demographic characteristics at the time of death among the RESPOND cohort, TESSy HIV notifications and Eurostat between 2018 and 2022 in the 21 EU/EEA countries reporting to all three sources.

### P307 HIV Care Cascade During the First Year Among People Re‐Engaging in Care

Ana Rodriguez, Victoria Pinto, Eugenia Pia Sobrado, Mariana Ariadna Paolini, Valentina Nuri Balbi Rocca, Julian Vega, Lucia Nannini, Francisco Martin Ferreira Monteiro, Veronica Azul Benseñor, Alicia Sisto, María José Rolón, Martín Jaume

Infectious Diseases Division, Hospital Fernández, Buenos Aires, Argentina

**Background**: Antiretroviral therapy (ART) discontinuation and loss to follow‐up represent key barriers to achieving the UNAIDS 95‐95‐95 targets. Gaps in the continuum of care among people living with HIV (PLWH) negatively impact both individual and population‐level outcomes. We aimed to assess the 1‐year HIV care cascade and describe ART re‐initiation among PLWH returning to HIV care after a period of disengagement.

**Methods**: We conducted a retrospective cohort study including PLWH aged ≥16 years who re‐engaged in HIV care between March 2023 and December 2024 at Hospital Fernandez in Buenos Aires, Argentina. Re‐engagement was defined as returning to HIV medical care after interruption of ART for ≥6 months. The 12‐month care cascade was analysed using definitions adapted from the PAHO Monitoring Framework (2014), excluding transferred and deceased individuals from the denominator. Data were collected from clinical records and national HIV surveillance data and analysed using Jamovi.

**Results**: A total of 286 PLWH were included, with a median age of 39 years (IQR 33–48); 176 (61.5%) were cisgender men, 93 (32.5%) cisgender women and 16 (5.6%) transgender women. Overall, 55.9% (160) had advanced HIV disease, with a median CD4 cell count of 184 cells/µL (IQR 72.5–356). Following re‐engagement, 93.4% (267) initiated ART, with a median time to initiation of 3 days (IQR 0–10); 67.8% initiated ART within the first 7 days. Comparing ART regimens before and after re‐engagement, a significant increase in INSTI‐based regimens was observed (56.9% vs. 85.9%; *p* < 0.001), with a reduction in NNRTIs (15.6% vs. 2.3%; *p* < 0.001) and PIs (38.9% vs. 26.0%; *p* < 0.001) (Figure 1A). For the care cascade analysis, 27 transferred and 15 deceased PLWH were excluded (*n* = 244). Retention was 67.2% (164/244); among retained individuals, 93.3% (153/164) were receiving ART; and among these, 41.8% (64/153) had an undetectable viral load (Figure 1B).

**Conclusions**: Re‐engagement in care facilitated rapid ART re‐initiation, with most PLWH receiving INSTI‐based regimens. However, the care cascade revealed substantial gaps, including one‐third loss in retention and low viral suppression rates. These findings identify PLWH re‐engaging in care as a priority population requiring differentiated retention strategies to contribute to achieving the 95‐95‐95 targets.

**FIGURE 1:**
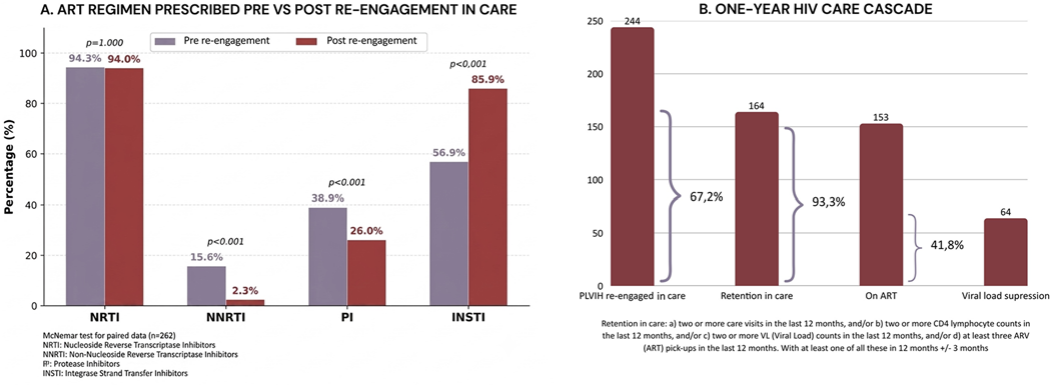
P307 | Continuum of care.

### P308 Evaluating Outcomes of Indeterminate Results in Emergency Department Opt‐Out HIV Testing

Anika Jayanth^1^, Lauren Pereira‐Greene^1^, Mark Longman^2^, Alex Comer^2^, Rachel Thompson^2^, Patrycja Puszkiewicz^2^, Patrick French^2^, Stuart Flanagan^2^, Nadia Ahmed ^2^

^1^UCL Medical School, University College London, London, UK; ^2^Central and North West London NHS Foundation Trust, London, UK

**Background**: Opt‐out HIV testing was introduced in NHS‐funded emergency departments (EDs) in April 2022 [1]. ED opt‐out HIV testing has increased case finding but also generates a proportion of indeterminate results. These results require follow‐up by healthcare professionals including result counselling, risk assessment and repeat testing advice. We evaluated outcomes, follow‐up completion and resource implications associated with indeterminate HIV results from the ED opt‐out testing pathway at a tertiary London hospital and associated HIV outpatient service.

**Methods**: A retrospective evaluation was conducted using electronic patient records for all indeterminate results received through our ED opt‐out testing pathway between February 2022 and May 2026. Data collected included demographics, documented HIV risk factors, repeat testing recommendations and follow‐up outcomes. Patients were stratified into high‐, moderate‐, low‐ or unknown‐risk groups according to documented HIV risk factors. Descriptive statistics were used to summarize testing patterns and clinical outcomes. Incomplete and duplicate entries were excluded.

**Results**: A total of 329 patients with indeterminate HIV testing results were identified, including 171 females (52%) and 158 males (48%). Of those who disclosed their sexual orientation, 155 identified as heterosexual (155/169, 91.7%) and 14 as homosexual (14/169, 8.2%). Most patients were classified as low risk (286/329, 86.9%), followed by moderate risk (25/329, 7.6%), unknown risk (12/329, 3.6%) and high risk (6/329, 1.8%). Repeat testing was advised in 49/329 cases (14.9%), not advised in 265/329 (80.5%) and undocumented in 15/329 (4.6%). Repeat testing recommendations correlated strongly with risk stratification: all high‐risk patients (6/6, 100%) and most moderate‐risk patients (23/25, 92.0%) were advised to repeat testing, compared with 13/286 low‐risk patients (4.5%). No confirmed HIV diagnoses were identified.

**Conclusions**: Most indeterminate HIV results occurred in low‐risk patients and did not result in confirmed HIV diagnoses. Although repeat testing was appropriately targeted towards higher‐risk individuals, indeterminate results generated substantial clinical and administrative workload with low diagnostic yield. Follow‐up also carries significant emotional burden, as patients are often unaware HIV testing has been performed and require unexpected counselling and reassurance. Further work should focus on optimizing follow‐up pathways while preserving the benefits of ED opt‐out testing in enabling early diagnosis, reducing health inequalities and supporting the national goal of ending new HIV transmissions by 2030 [2].

**References**

1. NHS England (2024). *Blood‐borne viruses opt‐out testing in emergency departments in areas of high and extremely high HIV prevalence: Good practice guidance*. [online] England.nhs.uk. Available at: https://www.england.nhs.uk/long‐read/blood‐borne‐viruses‐opt‐out‐testing‐in‐emergency‐departments/. [Accessed 26 May 2026].

2. GOV.UK (2025). *HIV Action Plan for England, 2025 to 2030*. [online] Available at: https://www.gov.uk/government/publications/hiv‐action‐plan‐for‐england‐2025‐to‐2030 [Accessed 26 May 2026].

### P309 To What Extent Does Pol‑Based HIV‑1 Subtyping Underestimate Regional CRF and Recombinant‑Complex Diversity? A Quantification of Bias Using COMET

Alexandria Williams ^1^, Virginie Mortier^1^, Sonia Menon^2^

^1^Ghent University, Ghent, Belgium; ^2^Epitech Research, Brussels, Belgium

**Background**: It is well known that routine HIV‑1 surveillance relies heavily on pol sequences for subtype identification, which are easier and cheaper to generate than nearly full‑length genomes (NFLGs). As HIV circulating recombinant forms (CRFs) and more complex recombinants become increasingly common in some regions, particularly those with high HIV burden, it is important to quantify how much underestimation occurs. Clinically and epidemiologically, under‑recognition of recombinant lineages may distort interpretation of regional subtype distributions, drug‑resistance patterns and transmission dynamics, particularly in high‑burden settings.

**Methods**: We assembled a dataset of 11,969 NFLG sequences from the LANL database (2010–2024) and drew a random sample of up to 400 sequences per UNAIDS region (*n* = 2117), to maintain computational feasibility. We then created a matching subset of position‑based clipped pol sequences and ran both datasets through the COMET subtyping tool. Three flags were created to categorize the sequences: (i) named CRF versus pure subtype; (ii) recombinant‑complex; and (iii) missing subtype. For each region we estimated the proportion of CRFs and recombinant‑complex sequences from full‑genome versus pol data and calculated simple “underestimation factors” (full/pol).

**Results**: Across UNAIDS regions, COMET pol systematically underestimated recombinant‑complex sequences by a factor (AW1) of 3.5 to 4, compared with NFLG. In most regions, the proportion of sequences classified as recombinant‑complex increased from roughly 6–13% with pol to around 24–51% with NFLG. In contrast, named CRF characterization was more variable and region‐dependent: in the Asia‐Pacific and Western and Central Africa, pol‐based COMET slightly overestimated CRFs.

**Conclusions**: Pol‑only HIV‑1 subtyping substantially underestimates recombinant‑complex diversity across multiple UNAIDS regions and can mischaracterize named CRFs in settings where recombinant lineages dominate. These biases may obscure important shifts in the circulating viral population and affect clinical and programme decisions based on subtype patterns and resistance surveillance. Our findings support targeted use of extended‑genome sequencing within surveillance systems, particularly in settings with a high HIV burden and highlight the need to interpret pol‑based subtype and resistance data with caution when informing guidelines and public health strategies.

### P310 Beyond Guidelines: A Thematic Synthesis of Facilitators and Barriers for Embedding Routine HIV Testing Into European Hospitals

Klaske Vliegenthart‐Jongbloed^1*^, Isabella Pepe Razzolini ^1*^, Ciska Hoving^2^, Winston van Druten^3^, Sarah Stutterheim^2^, Casper Rokx^1^

^1^Department of Internal Medicine, Infectious Diseases Section & Department of Medical Microbiology and Infectious Diseases, Erasmus University Medical Center, Rotterdam, Netherlands; ^2^Department of Health Promotion & Care and Public Health Research Institute (CAPHRI), Maastricht University, Maastricht, Netherlands; ^3^Master's Programme in Biomedical Sciences, Vrije Universiteit Amsterdam, Amsterdam, Netherlands

^*^Contributed equally to this work as co‐first authors.

**Background**: Despite guidelines recommending hospital‐based HIV testing, more than half of people diagnosed with HIV in the WHO European Region are diagnosed late [1]. Evidence on contextual determinants of HIV testing remains fragmented across countries, testing strategies and care settings. This systematic review and thematic synthesis aimed to identify recurring determinants influencing hospital‐based HIV testing across Europe.

**Methods**: MEDLINE, Embase, Web of Science, CINAHL and ProQuest were searched (2005–2025) for studies reporting contextual barriers or facilitators to hospital‐based HIV testing in the WHO European Region (PROSPERO CRD420251006898). Findings were coded using the Consolidated Framework for Implementation Research (CFIR) [2] and their direction as barrier or facilitator was recorded, followed by thematic synthesis to generate higher‐order analytical themes.

**Results**: Twenty‐five studies were included. Line‐by‐line coding yielded 467 excerpts mapped to CFIR constructs and synthesized into 13 themes across socioecological levels (Table 1). While all CFIR domains were represented, “Individuals” and “Inner Setting” predominated over “Outer setting,” “Innovation” and “Process.” At the societal level, contact with HIV could normalize HIV testing, while HIV exceptionalism, public health messaging and assumptions about HIV epidemiology could reinforce perceptions of HIV as relevant primarily to certain populations. At the organizational level, capacity constraints were barriers to testing. How testing was organized, including workflow integration and consent and follow‐up procedures, influenced implementation both positively and negatively. The same applied to compatibility with the care setting and institutional policies. Staff engagement and ongoing reinforcement strategies were facilitators. At the interpersonal level, providers’ anticipation of negative patient reactions was a barrier. At the individual level, provider knowledge, skills and confidence could hinder testing. Decisions to offer HIV tests were influenced by clinical appraisal, guidelines and perceived patient benefit. Although patients usually accepted HIV testing, decline was usually the result of low perceived risk, fear of diagnosis, or prioritization of the presenting complaint.

**Conclusions**: This first Europe‐wide synthesis shows that hospital‐based HIV testing depends on how testing is organized, clinicians’ decision‐making and their knowledge, skills and confidence. HIV exceptionalism and capacity constraints remain important barriers. Staff engagement and ongoing reinforcement were facilitators. The identified themes can provide a roadmap for improving HIV testing across European hospitals.

**References**

1. European Centre for Disease Prevention and Control/WHO Regional Office for Europe. HIV/AIDS surveillance in Europe 2024–2025 data. Stockholm: ECDC; 2025.

2. Damschroder LJ, Reardon CM, Widerquist MAO, Lowery J. The updated Consolidated Framework for Implementation Research based on user feedback. Implement Sci. 2022;17(1):75.

**TABLE 1:**
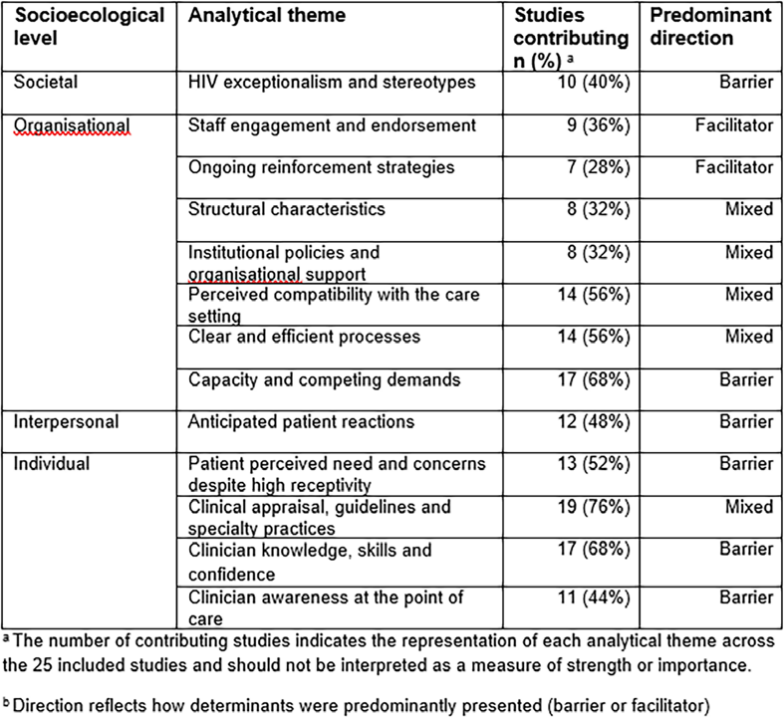
P310 | Analytical themes and direction of determinants influencing hospital‐based HIV testing across socioecological levels.

### P311 Implementation of HIV Screening and Direct Connection to Care in an Emergency Department in Very Large US Hospital

Jeffrey Kuhlman

AdventHealth Clinical, Orlando, FL, US

**Background**: America's public health system has been chronically underfunded, with ripples across US healthcare. Public health interventions are being introduced in Emergency Departments, as they have unique ability to reach underserved populations. Screening all patients for blood‐borne pathogens, HIV, HCV, HBV and syphilis and connecting them directly to curative care is being done at some hospitals.

**Methods**: AdventHealth Orlando is a quaternary referral medical centre with 150,000 inpatient admissions annually and > 100,000 adult ED visits at their Orlando campus. In March 2025, they implemented the CDC recommendations for HIV “opt out” screening by performing HIV test on all adult ED patients who are having their blood drawn. This was a paradigm shift for many doctors and nurses with many barriers and obstacles (real and perceived) addressed. FOCUS BBV public health programme provided guidance.

**Results**: In the first year of the programme, 103,712 patients presented to the adult ED, 79,267 had blood work. HIV test was performed on 37,210 with 176 positive cases, of which 65 new HIV cases were discovered. 109 were prior known cases with many of them fallen out of care (Figure 1). All were promptly connected to the county health department and their funded, staffed, well run HIV clinic (Table 1).

**Conclusions**: Individuals living with HIV, whether they are unaware or they have fallen out of the system, are being discovered and promptly connected to care, improving their health and helping to prevent spread to others [1]. The overwhelming successful proof‐of‐concept programme at one hospital ED will lead to this health system implementing in their 80 emergency departments across nine states in the US Additional “screen and connect” programme for HCV, HBP and syphilis are now in the works, as well as immunizing individuals for Hep A/B and identifying substance use disorder patients with MAT and coordinated care. Public Health in the ED! [2]

**References**

1. Trust for America's Health. The Impact of Chronic Underfunding on America's Public Health System. August 2024.

2. Fassas E, Fischer K, Schenkel S, Gatz J, Gingold D. Public health interventions in the emergency department: A framework for evaluation. Western Journal of Emergency Medicine. Volume 25, No. 3, May 2024.[Fig jia270211-fig-0349]

**FIGURE 1:**
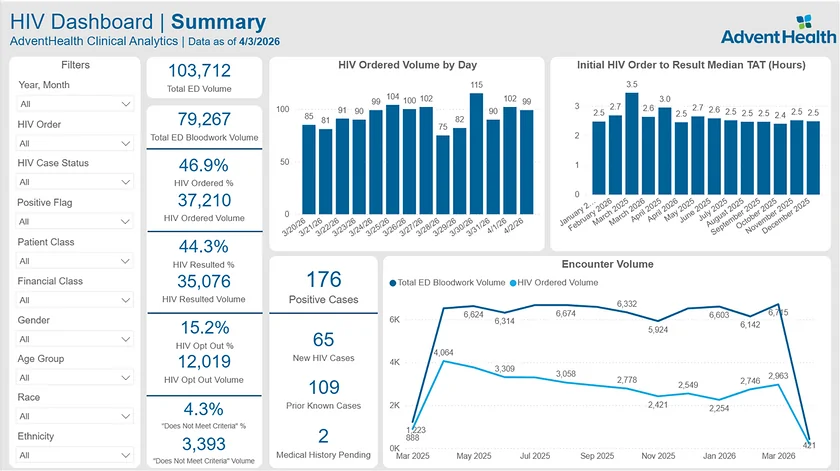
P311 | HIV dashboard of first year.

**FIGURE 2 jia270211-fig-0349:**
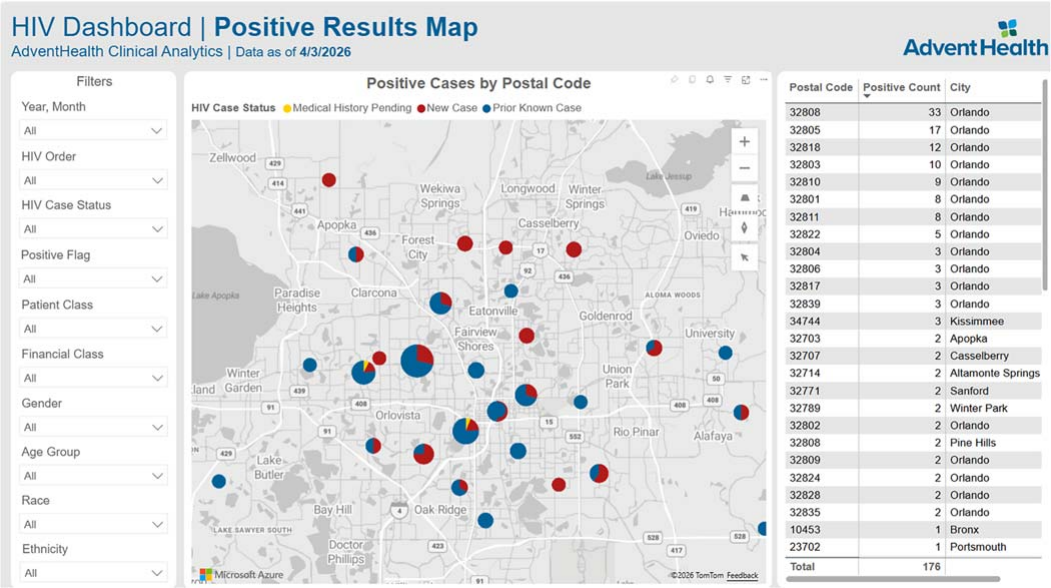
P311 | HIV cases by geographic mapping.

### P312 Smoking and HIV in the UK: Prevalence, Patterns and Associated Factors in the Positive Voices 2022 Cross‐Sectional Study

Tamsin McKinnon ^1^, Fiona Lampe^2^, Fumiyo Nakagawa^2^, Janey Sewell^2^, Marc Lipman^1,3^, James Brown^1^, Bernard Kelly^4^, Shalini Andrews^5^, Alex Sparrowhawk^6^, Carole Kelly^7^, Meaghan Kall^7^, Valerie Delpech^7^, Andrew Phillips^2^, Colette Smith^2^, Alison Rodger^1,2^, on behalf of PV2022 Study Group

^1^Royal Free London NHS Foundation Trust, London, UK; ^2^Institute for Global Health, University College London, London, UK; ^3^Respiratory Medicine, University College London, London, UK; ^4^The Courtyard Clinic, St George's Hospital, London, UK; ^5^Buryfields Sexual Health Clinic, Surrey Sexual Health Service, Guilford, UK; ^6^George House Trust, Manchester, UK; ^7^UK Health Security Agency, London, UK

**Background**: People with HIV have higher smoking prevalence and worse smoking‐related outcomes than the general population. We assessed current smoking among people with HIV, the association with sociodemographic, health and lifestyle factors and engagement with smoking cessation services. We compared this to historical smoking behaviours.

**Methods**: Positive Voices 2022 is a cross‐sectional questionnaire study of people accessing HIV care in the UK (April 2022–March 2023). Sociodemographic and health‐related factors associated with current and previous smoking, compared to never smoking, were assessed with multinomial logistic regression, adjusted for age, demographic group and HIV diagnosis year. Smoking cessation services engagement in the past year was assessed. Vaping data was not collected. Smoking prevalence was compared with two earlier UK cross‐sectional studies of people with HIV: ASTRA 2011–2012 and Positive Voices 2017.

**Results**: Of 4111 participants (mean age 53 [IQR 44–60]; 74.8% men), 17.4% (714/4111, 95% CI: 16.2–18.6%) currently smoked, 32.4 % (1330/4111) previously smoked and 50.3% (2067/4111) had never smoked. Prevalence of current smoking was 20.0% (616/3073, 95% CI: 18.6–21.5%) in men and 9.0% (88/979, 95% CI: 7.2–10.8%) in women. Current smoking was significantly associated with younger age, white ethnicity, being gay, bisexual or other MSM, lower socioeconomic status (non‐employment/financial hardship), earlier HIV diagnosis date, slower walking pace, poor self‐rated health, anxiety (GAD‐7 ≥10) and depression (PHQ‐9 ≥10) symptoms, harmful alcohol use (AUDIT‐C) and recent recreational drug use. It was also significantly associated with having a diagnosis of COPD, heart attack and/or stroke and asthma. Similar though weaker associations were seen in previous smokers. Regarding smoking cessation services for current smokers, 54.1% (374/691) reported they did not need, 34.0% (235/691) reported needing but not accessing and 11.9% (82/691) reported having accessed. Smoking prevalence fell sequentially across the surveys, from 31.5% in 2011 and 19.7% in 2017.

**Conclusions**: Tobacco smoking remains a key modifiable risk factor among people with HIV in the UK, associated with worse overall health, specific physical and mental health conditions and substance use. Though smoking prevalence has reduced over the past decade, current smoking prevalence remains high and uptake of cessation support low, suggesting a role for integrated smoking cessation services within HIV care.

### P313 From Recommendation to Routine: What It Takes to Integrate HIV Testing Into European Hospital Care

Klaske Vliegenthart‐Jongbloed ^1^, Divya Purwaha^2^, Winston van Druten^3^, Casper Rokx^1^, on behalf of #aware.hiv Europe Study Group

^1^Department of Internal Medicine, Infectious Diseases Section & Department of Medical Microbiology and Infectious Diseases, Erasmus University Medical Center, Rotterdam, Netherlands; ^2^Medical Student, Erasmus MC University Medical Center, Rotterdam, Netherlands; ^3^Master's Programme in Biomedical Sciences, Vrije Universiteit Amsterdam, Amsterdam, Netherlands

**Background**: Despite recommendations for indicator condition‐guided HIV testing, testing opportunities continue to be missed in European hospitals [1]. #aware.hiv Europe aims to improve indicator condition‐guided HIV testing through audit and feedback, teaching, linkage to prevention and stigma reduction. Understanding pre‐implementation barriers and facilitators may help tailor current and future HIV testing initiatives.

**Methods**: We invited all implementation leaders across all participating #aware.hiv Europe hospitals in a semi‐structured interview conducted in English (NCT06900829). Two researchers carried out the interviews using a common interview guide. Transcripts were coded using the Consolidated Framework for Implementation Research (CFIR), excluding the Process domain as implementation has not yet started. Determinants were classified as barriers or facilitators, followed by the development of descriptive and higher‐order analytical themes.

**Results**: Twenty‐eight of 34 invited implementation leaders from ten countries participated. Interviews yielded 836 coded excerpts, broadly balanced across domains and between barriers and facilitators (Figure 1). Thematic synthesis generated 11 analytical themes.

Initiating HIV testing initiatives requires evidence of cost‐effectiveness, locally feasible strategies and hospital management support. Engaged staff help drive initiatives forward, but competing clinical responsibilities and staff shortages constrain implementation. Integration into routine workflows and automation can support sustainability. Teaching increases awareness, while feedback shows performance and missed testing opportunities. Networks linking HIV expertise with clinical and community services create additional testing opportunities and strengthen care pathways. National action plans and funding support HIV testing but remain vulnerable to changing political priorities and societal attitudes. Specialty‐specific guidance and supportive regulations facilitate testing, particularly as clinicians may overestimate counselling and consent requirements. Clinicians are more likely to test when HIV is familiar and the clinical indication is clear; however, stereotypes regarding who is at risk continue to influence decision‐making. HIV testing can become routine when staff look beyond their own specialty, viewing it as part of their responsibility and remain open to change.

**Conclusions**: Embedding routine HIV testing in hospitals requires feasible implementation and a supportive organizational context. Testing should be integrated into clinical workflows and automated where possible, supported by engaged staff with sufficient capacity, management commitment, collaboration across departments and recognition of HIV testing as a shared responsibility across specialties.

**Reference**

1. Bogers SJ, Hulstein SH, Schim van der Loeff MF, de Bree GJ, Reiss P, van Bergen J, et al. Current evidence on the adoption of indicator condition guided testing for HIV in western countries: A systematic review and meta‐analysis. EClinicalMedicine. 2021;35:100877.

**FIGURE 1:**
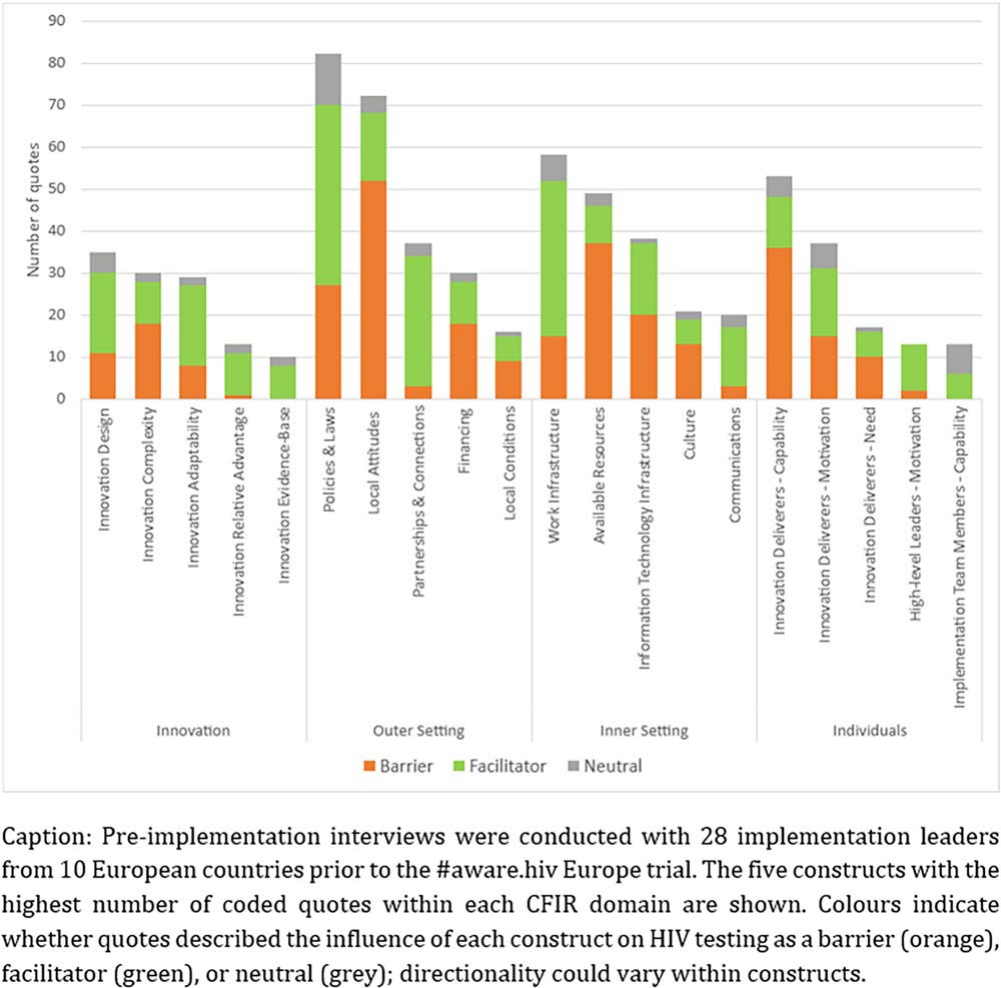
P313 | Five most frequently identified CFIR constructs per domain influencing hospital‐based HIV testing.

### P314 Projected Public Health Impact of Removing Financial Barriers to PrEP in High‐Incidence Populations

Turgay Ayer^1,2^, JeanPierre Coaquira Castro^3^, Emir Gursel^1^, Alice Hsiao^3^, Woodie Zachry^3^, Patrick Sullivan^4^

^1^Value Analytics Labs, Boston, MA, US; ^2^Georgia Institute of Technology, Atlanta, GA, US; ^3^Gilead Sciences, Inc., Foster City, CA, US; ^4^Emory University, Atlanta, GA, US

**Background**: HIV‐1 incidence disproportionately affects men who have sex with men (MSM), transgender women (TGW) and cisgender women (CGW) with pre‐exposure prophylaxis (PrEP) indications, with Black and Hispanic populations at higher risk in the US. Although guidelines recommend PrEP without cost sharing under coverage policies, continued out‐of‐pocket (OOP) costs remain a barrier to access, limiting uptake and prevention efforts in the US. Between 2021 and 2023, 12.5% of PrEP prescriptions required OOP payments. These costs have been associated with higher rates of PrEP abandonment and increased HIV‐1 acquisition. We evaluated the clinical benefits of removing OOP costs to improve access to oral and long‐acting injectable (bimonthly and twice‐yearly) PrEP in high‐incidence US populations.

**Methods**: We developed a lifetime‐horizon HIV‐1 microsimulation model to assess the impact of removing OOP costs in high‐incidence US populations. Two scenarios were evaluated in each population: current OOP cost barriers (comparator) and complete removal of OOP costs (intervention). Model inputs—including HIV‐1 incidence rates, PrEP use behaviour (adherence and persistence), OOP cost distribution and OOP‐cost‐dependent PrEP abandonment probabilities (with higher OOP costs increasing abandonment probability)—were informed by published and real‐world data. Oral PrEP was modelled as the predominant PrEP modality. The primary outcome was the number of HIV cases averted over 5 years.

**Results**: Removing OOP costs reduced HIV‐1 incidence, averting 40.4 HIV‐1 cases per 10,000 individuals over 5 years across combined populations weighted by population size. Among MSM and TGW populations, 32.1 and 29.6 cases per 10,000 individuals were averted, respectively. Public health benefits were greater in populations with higher baseline incidence, including Black MSM and CGW with PrEP indications—117.9 and 108.5 cases averted per 10,000 individuals, respectively (Table 1).

**Conclusions**: Removing financial barriers to oral and long‐acting injectable PrEP access is likely to improve uptake and reduce HIV‐1 incidence in overburdened populations. These findings highlight the public health importance of eliminating access barriers to PrEP access as part of HIV prevention strategies. Conversely, the presence of access barriers can limit PrEP uptake and hinder progress towards public health goals, thereby increasing the burden on healthcare systems.

**TABLE 1:**
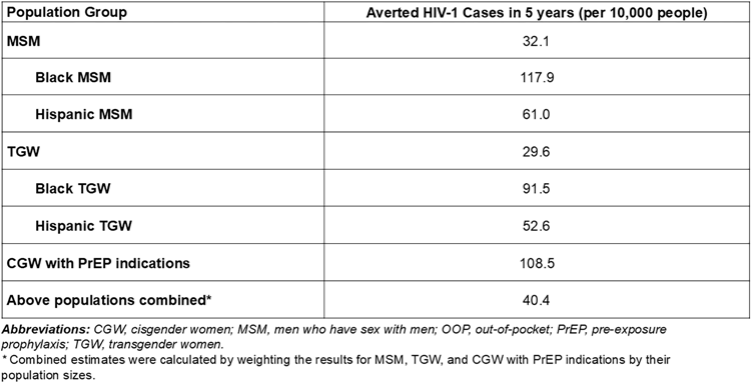
P314 | Averted HIV‐1 cases after elimination of PrEP OOP cost, for MSM, TGW and CGW with PrEP indications and across all of these populations, US.

### P315 Age and Syndemic Disparities in Clinician‐Initiated HIV Screening in the Emergency Department and Projected Impact of an Automated Opt‐Out Bundle

Omvikneswer Muralitharan

Community Hospital of San Bernardino, CommonSpirit Health, San Bernardino, CA, US. Gilead FOCUS, Gilead Sciences, Foster City, CA, US

**Background**: Early identification of HIV remains essential to reduce transmission. Emergency departments (EDs) offer a critical opportunity to diagnose and connect individuals to treatment. We examined clinician‐initiated HIV testing in a Southern California ED, alongside syphilis and hepatitis C (HCV) testing as syndemic markers.

**Methods**: Clinician‐initiated test ordering and reactives were analysed among individuals aged ≥13 years attending the ED from April 2025 to March 2026. A binomial generalized mixed model evaluated testing likelihood by age, test‐type and age by test‐type interaction, alongside marginal contrasts at age 20, 40 and 60. A clustered generalized estimating equation assessed a three‐point syndemic burden score, defined by recent STI reactivity, substance use presentation or mental health admission. Finally, we projected annual testing and positive results under an opt‐out, three‐test automated bundle assuming baseline of ∼1600 tests/month and a 95% acceptance rate.

**Results**: We analysed 13,703 tests clustered within 5869 unique individuals (ICC 0.20). Median age was 37 years for HIV (IQR 27–50) and 40 years for both HCV (IQR 29–56) and syphilis (IQR 29–56). HCV testing odds increased with age (OR 1.014; 95% CI: 1.007–1.020). Conversely, testing odds declined with age for HIV (OR = 0.972; 95% CI: 0.965–0.980) and syphilis (OR 0.988; 95% CI: 0.978–0.998). The relative odds of HIV versus HCV testing decreased across ages 20, 40 and 60 years (OR 0.021, 0.012 and 0.007, respectively). Syphilis testing odds exceeded HCV at age 20 (OR 2.16; 95% CI: 1.58–2.96) but converged by age 60 (OR 1.35; 95% CI: 1.00–1.81). One syndemic marker significantly reduced testing odds (aOR 0.25, *p* < 0.001), with a similar reduction for two markers (aOR 0.11, *p* < 0.001); post‐hoc differences were non‐significant (*p* = 0.081). The automated bundle of 19,200 tests would increase newly identified reactive test results for HIV (+21, +150%), HCV (+445, +180%) and syphilis (+331, +181%).

**Conclusions**: Clinician‐led ordering produced systematic age‐related and syndemic screening gaps hindering HIV screening initiatives. Scaling an automated, opt‐out three‐test bundle (HIV, HCV, syphilis) through EHR‐integrated workflows at the health‐system and national levels would capture overlapping and distinct groups, advancing equitable infectious‐disease detection.

### P316 Phylogenetic Investigation of HIV‐1 Integrase L74I in Greece and Poland: Evidence for Subtype‐Dependent Onward Transmission

Evangelia Georgia Kostaki ^1*^, Karol Serwin^2*^, Eleni Papachristou^1^, Kaja Mielczak^2^, Alexandros Vassilakis^1^, Chrysoula Rokka^1^, Panagiotis Iliopoulos^1^, Pagona Lagiou^1^, Gkikas Magiorkinis^1†^, Miłosz Parczewski^2†^, Dimitrios Paraskevis^1†^

^1^Department of Preventive Medicine, Epidemiology and Medical Statistics, Medical School, National and Kapodistrian University of Athens, Athens, Greece; ^2^Department of Infectious, Tropical Diseases and Immune Deficiency, Pomeranian Medical University, Szczecin, Poland

**Background**: The presence of HIV‐1 subtype A6 with integrase strand transfer inhibitor (INSTI)‐associated mutation L74I has been associated with an increased risk of virological failure in individuals receiving long‐acting injectable cabotegravir/rilpivirine (CAB/RPV). We aimed to estimate the prevalence of L74I and to investigate the dispersal patterns of HIV‐1 strains carrying L74I in Greece and Poland across divergent subtypes.

**Methods**: We analysed 3046 integrase gene sequences from antiretroviral therapy (ART)‐naïve (*n* = 2274) and ART‐experienced (*n* = 772) people living with HIV (PLWH). Sequences were sampled in Greece (*n* = 1066, 2021–2024) and Poland (*n* = 1980, 1997–2024). HIV‐1 subtyping was performed using COMET and REGA/Genome Detective and confirmed by phylogenetic analysis including references of all known HIV‐1 subtypes and more than 150 CRFs. INSTI resistance mutations were identified through the Stanford HIV Drug Resistance Database. Dispersal patterns of HIV‐1 strains carrying L74I were investigated by maximum likelihood phylogenetic analysis (FastTree) performed on the study sequences along with 5072 globally sampled reference sequences.

**Results**: The Greek HIV‐1 epidemic was more genetically diverse, with A1 being the most prevalent HIV‐1 subtype (22.5%), followed by B (17.1%), CRF02_AG (11.0%), CRF14_BG (10.4%) and A6 (6.9%). In Poland, subtype B predominated (53.6%), followed by A6 (30.6%) with little contribution of other variants. Among ART‐naïve PLWH, L74I prevalence across subtypes was similar between the two countries: A6 (90.0%), A1 (26.9%), CRF14_BG (10.7%), CRF02_AG (8.4%), B (4.1%). Phylogenetic analyses revealed onward transmission of L74I within phylogenetic clusters for A6 (Greece: 70.0%, Poland: 75.8%), A1 (Greece: 88.3%) and CRF14_BG (Greece: 75.0%). For A6, 11 clusters were identified in Greece (cluster size: 2–10) and 89 in Poland (cluster size: 2–32). In Greece, 6 clusters of viruses carrying L74I were detected for A1 (cluster size: 2–33) and one for CRF14_BG (cluster size: 9). Lower levels of L74I transmission within clusters were observed for B (Greece: 40.0%, Poland: 54.3%) and CRF02_AG (Greece: 25.0%).

**Conclusions**: L74I prevalence varied greatly across HIV‐1 subtypes, ranging from 4.1% to 26.9% outside A6. Subtypes that spread locally also exhibited more extensive clustering of L74I, consistent with onward transmission. Our findings support L74I surveillance and baseline integrase resistance testing, particularly in regions with high L74I prevalence.

^*^Contributed equally to this work as co‐first authors.

^†^Contributed equally to this work as co‐last authors.

### P317 Healthcare Worker HIV Stigma and Willingness to Provide Care for Key Populations in Taiwan: Findings From Integrated WHO and HPASS Measures

Su‐Han Hsu^1^, Hsin‐Hao Lai ^2^, Shan‐Lin Liu^2^, Chien Chun Wang^2^, Tsen‐Fang Yen^3^, Chih Chieh Tsai^4^

^1^ Pharmacy, Taipei City Hospital, Taipei, Taiwan; ^2^Infectious Diseases, Taipei City Hospital, Taipei, Taiwan; ^3^Department of Nursing, Taipei City Hospital, Taipei, Taiwan; ^4^Developing and Planning Department, Taipei City Hospital, Taipei, Taiwan

**Background**: Healthcare worker stigma remains a significant barrier to equitable HIV care. While attitudinal stigma has been associated with discriminatory behaviours, its influence on willingness to provide care for key populations remains insufficiently understood. This study examined the relationship between HIV‐related stigma and willingness to provide care among healthcare workers in Taiwan using integrated measures from the WHO Health Facility Staff Stigma Questionnaire [1] and the Healthcare Provider HIV/AIDS Stigma Scale (HPASS) [2].

**Methods**: A cross‐sectional survey was conducted among 619 healthcare workers from multiple hospital settings in Taiwan. Participants completed the Taiwanese version of the WHO Health Facility Staff Stigma Questionnaire and the Healthcare Provider HIV/AIDS Stigma Scale (HPASS). Hierarchical regression analyses were performed to identify predictors of unwillingness to provide care for key populations after adjustment for demographic characteristics, HIV‐related training and exposure to people living with HIV (PLWH) (Figure 1). Differences in willingness to provide care across key populations were examined using repeated‐measures ANOVA.

**Results**: Prejudice emerged as the strongest predictor of unwillingness to provide care for key populations (β = 0.39, *p* < 0.001), followed by discrimination (β = 0.24, *p* < 0.001). The final regression model explained 40.1% of the variance in willingness to provide care (*R* ^2^ = 0.401). HIV‐related training and greater exposure to PLWH were associated with lower stigma scores. Significant differences were observed across key populations (Greenhouse–Geisser corrected *F* [1.49, 920.18] = 143.34, *p* < 0.001, partial η^2^ = 0.188), Table 1. Healthcare workers reported significantly greater reluctance to provide care for people who inject drugs (PWID) compared with men who have sex with men (MSM) and sex workers (mean scores: 2.38, 1.89 and 1.90, respectively; both pairwise comparisons *p* < 0.001). No significant difference was observed between MSM and sex workers.

**Conclusions**: Attitudinal stigma, particularly prejudice, is a major determinant of unwillingness to provide care for key populations among healthcare workers. Despite increasing acceptance of MSM and sex workers, substantial stigma towards PWID persists. Interventions targeting prejudice reduction, in addition to HIV education and training, may be critical for improving equitable access to HIV‐related healthcare services.

**References**

1. Anne C. Wagner, Trevor A. Hart, Kelly E. McShane, Shari Margolese & Todd A. Girard. Health care provider attitudes and beliefs about people living with HIV: Initial validation of the Health Care Provider HIV/AIDS Stigma Scale (HPASS). AIDS and Behavior. Soc, 2014;18: p. 2397–2408.

2. Nyblade, L., et al. A brief, standardized tool for measuring HIV‐related stigma among health facility staff: Results of field testing in China, Dominica, Egypt, Kenya, Puerto Rico and St. Christopher & Nevis. J Int AIDS Soc, 2013;16(3 Suppl 2): p. 18718.

**FIGURE 1:**
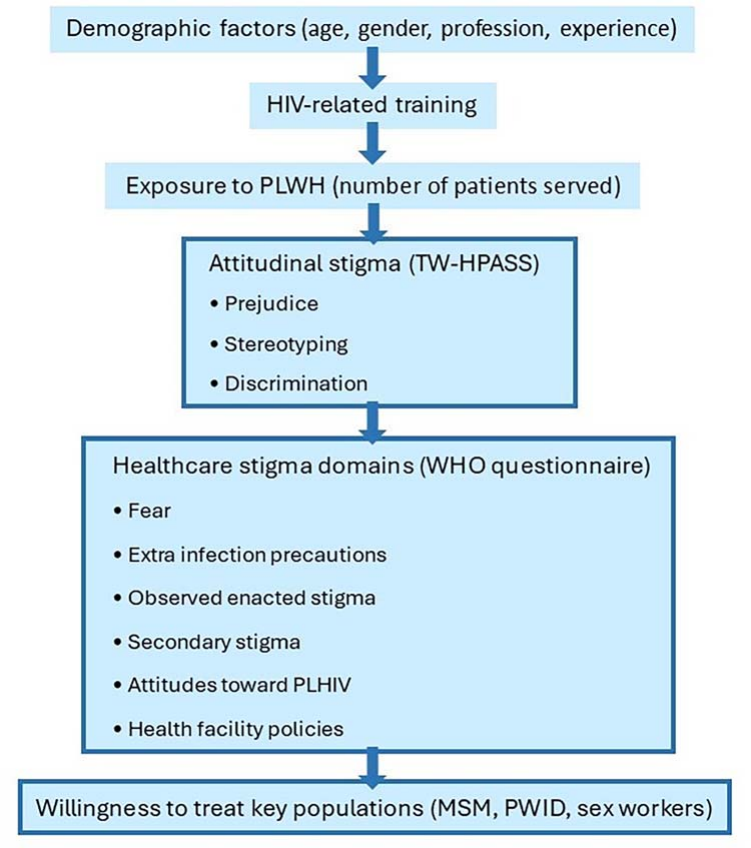
P317 | Conceptual framework of the relationship between attitudinal stigma and healthcare stigma towards people living with HIV.

**TABLE 1:**
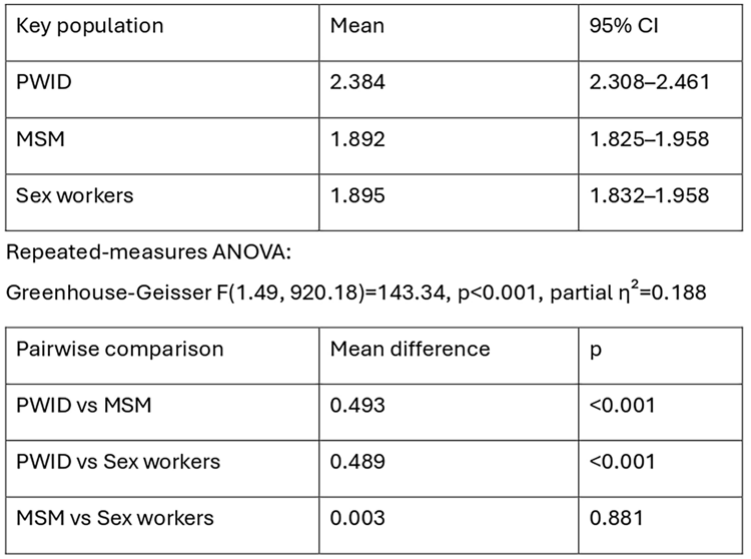
P317 | Comparison of willingness to provide care across key populations.

### P318 Strategies for Recruiting and Retaining Pregnant and Breastfeeding Women Living With HIV in Pharmacokinetic Studies in Uganda

Ritah Nakijoba ^1^, Aida N. Kawuma^1^, Simon Peter Asiimwe^1^, Christine Turyahabwe^1^, Jacqueline Kyeyune^1^, Johnson Magoola^1^, Francis W. Ojara^1,2^, Catriona Waitt^1,3^

^1^Infectious Diseases Institute, Makerere University College of Health Sciences, Kampala, Uganda; ^2^Department of Pharmacology and Therapeutics, Gulu University, Gulu, Uganda; ^3^Department of Pharmacology and Therapeutics, University of Liverpool, Liverpool, UK

**Background**: Pregnant and breastfeeding women living with HIV are often underrepresented in clinical and pharmacokinetic (PK) research due to ethical concerns, perceived risks to mothers and infants and operational challenges in recruitment and retention. This exclusion has contributed to persistent evidence gaps regarding the safety, efficacy and optimal dosing of antiretroviral and other medicines used during pregnancy and lactation. This paper describes strategies used to recruit and retain pregnant and breastfeeding women in PK studies in Uganda, with a focus on HIV‐related research and highlights key opportunities, barriers and lessons learned.

**Methods**: Experiences from five PK studies conducted in Uganda, including studies involving women living with HIV and those receiving antiretroviral therapy, were reviewed to identify effective recruitment and retention strategies. These included community engagements, stakeholder involvement, collaboration with healthcare providers, safety monitoring systems and participant follow‐up approaches.

**Results**: Several strategies were found to enhance recruitment and retention among pregnant and breastfeeding women living with HIV in PK studies. Community engagement activities, including workshops, outreach initiatives, stakeholder meetings and Community Advisory Board consultations, improved awareness of research and strengthened trust within communities. The involvement of peer mothers, including women living with HIV, as co‐investigators and community ambassadors improved communication between researchers and participants, enhancing study acceptance and engagement. Collaboration with HIV care providers and dedicated recruitment sites facilitated the identification and enrolment of eligible participants. In addition, structured safety monitoring and flexible communication approaches, including telephone follow‐up, social networks and community linkages, supported retention. Despite these successes, barriers persisted, including transport challenges, competing household responsibilities, scheduling difficulties and staff turnover at recruitment sites, which affected recruitment and follow‐up. However, flexible participant‐centred approaches and sustained stakeholder engagement helped mitigate these challenges.

**Conclusions**: Community engagement, stakeholder involvement and participant‐centred approaches are essential for improving recruitment and retention of pregnant and breastfeeding women living with HIV in pharmacokinetic research. Strengthening these strategies is critical for generating robust evidence to optimize antiretroviral use during pregnancy and lactation and to support safer, more equitable HIV care in high‐burden settings.

### P319 TURHIV‐DR: A Nationwide Multicentre Study of Transmitted HIV‐1 Drug Resistance Among Treatment‐Naïve People Living With HIV in Türkiye

Ilkay Akbulut ^1^, Tülin Demir^2^, Zeynep Sedef Varol^3^, Taliha Karakök^4^, Ayşe Deniz Gökengin^5^, Sabri Atalay^1^, Emel Eroğul^1^, Esra Duru Oz^1^, Arzu Varlı^1^, Ilker Odemis^1^, Ekrem Sağtaş^6^, Mehmet Balcı^7^, Yeşim Taşova^8^, Nefise Oztoprak Cuvalci^9^, Meliha Çağla Sönmezer^10^, Adalet Altunsoy^11^, Tuna Demirdal^12^, İlkay Karaoğlan^13^, Elif Doyuk Kartal^14^, Cigdem Ataman Hatipoglu^15^, Dilara İnan^16^, Sua Sumer^17^, Ozgur Gunal^18^, Aydın Deveci^19^, Bircan Kayaaslan^11,20^, Deniz Ozer^21^, Gonul Cicek Senturk^22^, Aliye Bastug^11,23^, Mehmet Cabalak^24^, Aysin Kılınç Toker^25^, Selcuk Kaya^26^, Nevin Ince^27^, Haluk Erdogan^28^, Zehra Karacaer^29^, Hüsnü Pullukçu^5^, Mehmet Serhat Birengel^30^, Yesim Yildiz^31^, Veysel Akca^32^, Tuba Damar Çakırca^33^, Meltem Arzu Yetkin^34^, Funda Memisoglu^35^, Ayse Albayrak^36^, Gamze Kalin Unuvar^37^, Gulden Ersoz^38^, Habibe Tulin Elmaslar Mert^39^, Fehmi Tabak^40^, Selma Ates^41^, Ahmet Çağkan İnkaya^10^, on behalf of TURHIV‐DR Study Group

^1^Department of Infectious Diseases and Clinical Microbiology, Health Sciences University Tepecik Training and Research Hospital, Izmir, Türkiye; ^2^Department of Medical Microbiology, Ahi Evran University Training and Research Hospital, Kirsehir, Türkiye; ^3^Department of Public Health, Izmir Democracy University Faculty of Medicine, Izmir, Türkiye; ^4^Department of Infectious Diseases and Clinical Microbiology, Fatsa State Hospital, Ordu, Türkiye; ^5^Department of Infectious Diseases and Clinical Microbiology, Faculty of Medicine, Ege University, Izmir, Türkiye; ^6^Microbiology Reference Laboratory and Department of Biological Products, General Directorate of Public Health, Ministry of Health, Ankara, Türkiye; ^7^Department of Communicable Diseases and Early Warning, General Directorate of Public Health, Ministry of Health, Ankara, Türkiye; ^8^Department of Infectious Diseases and Clinical Microbiology, Faculty of Medicine, Cukurova University, Adana, Türkiye; ^9^Department of Infectious Diseases and Clinical Microbiology, Antalya Training and Research Hospital, Antalya, Türkiye; ^10^Department of Infectious Diseases and Clinical Microbiology, Faculty of Medicine, Hacettepe University, Ankara, Türkiye; ^11^Department of Infectious Diseases and Clinical Microbiology, Bilkent City Hospital, Ankara, Türkiye; ^12^Department of Infectious Diseases and Clinical Microbiology, Katip Celebi University, Ataturk Training and Research Hospital, Izmir, Türkiye; ^13^Department of Infectious Diseases and Clinical Microbiology, Faculty of Medicine, Gaziantep University, Gaziantep, Türkiye; ^14^Department of Infectious Diseases and Clinical Microbiology, Faculty of Medicine, Eskisehir Osmangazi University, Eskisehir, Türkiye; ^15^Department of Infectious Diseases and Clinical Microbiology, University of Health Sciences, Ankara Training and Research Hospital, Ankara, Türkiye; ^16^Department of Infectious Diseases and Clinical Microbiology, Faculty of Medicine, Akdeniz University, Antalya, Türkiye; ^17^Department of Infectious Diseases and Clinical Microbiology, Faculty of Medicine, Selcuk University, Konya, Türkiye; ^18^Department of Infectious Diseases and Clinical Microbiology, Samsun Training and Research Hospital, Samsun University Faculty of Medicine, Samsun, Türkiye; ^19^Department of Infectious Diseases and Clinical Microbiology, Faculty of Medicine, Ondokuz Mayis University, Samsun, Türkiye; ^20^Department of Infectious Diseases and Clinical Microbiology, Atatürk Training and Research Hospital, Ankara, Türkiye; ^21^Department of Infectious Diseases and Clinical Microbiology, Manisa Celal Bayar University, Hafsa Sultan Hospital, Manisa, Türkiye; ^22^Department of Infectious Diseases and Clinical Microbiology, Etlik City Hospital, Ankara, Türkiye; ^23^Department of Infectious Diseases and Clinical Microbiology, University of Health Sciences, Ankara Numune Training and Research Hospital, Ankara, Türkiye; ^24^Department of Infectious Diseases and Clinical Microbiology, Faculty of Medicine, Mustafa Kemal University, Hatay, Türkiye; ^25^Department of Infectious Diseases and Clinical Microbiology, University of Health Sciences, Kayseri City Hospital, Kayseri, Türkiye; ^26^Department of Infectious Diseases and Clinical Microbiology, Faculty of Medicine, Onsekiz Mart University, Canakkale, Türkiye; ^27^Department of Infectious Diseases and Clinical Microbiology, Faculty of Medicine, Duzce University, Duzce, Türkiye; ^28^Department of Infectious Diseases and Clinical Microbiology, Alanya Training and Research Hospital, Alanya Aladdin Keykubat University, Antalya, Türkiye; ^29^Department of Infectious Diseases and Clinical Microbiology, University of Health Sciences, Gulhane Training and Research Hospital, Ankara, Türkiye; ^30^Department of Infectious Diseases and Clinical Microbiology, Faculty of Medicine, Ankara University, Ankara, Türkiye; ^31^Department of Infectious Diseases and Clinical Microbiology, Faculty of Medicine, Gazi University, Ankara, Türkiye; ^32^Department of Infectious Diseases and Clinical Microbiology, Sandıklı State Hospital, Afyonkarahisar, Türkiye; ^33^Department of Infectious Diseases and Clinical Microbiology, Faculty of Medicine, Harran University, Sanliurfa, Türkiye; ^34^Department of Infectious Diseases and Clinical Microbiology, Faculty of Medicine, Giresun University, Giresun, Türkiye; ^35^Department of Infectious Diseases and Clinical Microbiology, Faculty of Medicine, Inonu University, Malatya, Türkiye; ^36^Department of Infectious Diseases and Clinical Microbiology, Faculty of Medicine, Ataturk University, Erzurum, Türkiye; ^37^Department of Infectious Diseases and Clinical Microbiology, Faculty of Medicine, Erciyes University, Kayseri, Türkiye; ^38^Department of Infectious Diseases and Clinical Microbiology, Faculty of Medicine, Mersin University, Mersin, Türkiye; ^39^Department of Infectious Diseases and Clinical Microbiology, Faculty of Medicine, Trakya University, Edirne, Türkiye; ^40^Department of Infectious Diseases and Clinical Microbiology, Cerrahpaşa Faculty of Medicine, Istanbul University, Istanbul, Türkiye; ^41^Department of Infectious Diseases and Clinical Microbiology, Faculty of Medicine, Kahramanmaras Sutcu Imam University, Kahramanmaras, Türkiye

**Background**: HIV‐1 molecular epidemiology is shaped by multiple interconnected drivers, including human mobility across the Europe–Middle East–Central Asia–Africa interface driven by migration, displacement and regional instability and ongoing local transmission. At Europe's eastern gateway, Türkiye is well placed to track subtype diversity and transmitted drug resistance (TDR). We characterized nationwide trends in HIV‐1 subtypes, recombinant patterns and TDR among treatment‐naïve (TN) people living with HIV.

**Methods**: In this retrospective, multicentre study (TURHIV‐DR), we analysed baseline genotypic resistance test results of TN adults (≥18 years) with confirmed HIV‐1 infection, studied at the National HIV/AIDS Confirmation and Viral Hepatitis Reference Laboratory between August 2016 and December 2024. All participants had protease and reverse transcriptase sequences, and a subset additionally had integrase sequences. Resistance was interpreted with Stanford HIVdb v10.1; TDR was defined as a penalty score ≥10 for at least one drug in the relevant class (NRTI, NNRTI, PI or INSTI). Temporal trends across 2016–2019, 2020–2021 and 2022–2024 were assessed by multivariable logistic regression adjusted for age, sex and subtype.

**Results**: Among 8002 TN PLWH (median age 36 years; 89.6% male), subtype B predominated (50.5%), followed by A6 (14.3%), B+CRF02_AG (9.7%), B+F (5.8%), F (3.8%), G (3.8%) and CRF02_AG (3.1%); A6 was the leading non‐B subtype, reflecting regional mobility. TDR prevalence was 2.8% for NRTIs (223/7972), 12.7% for NNRTIs (1009/7972), 1.0% for PIs (82/7921) and 1.6% for INSTIs (29/1835) (Table 1). Between 2016 and 2024, while the prevalence of PI TDR dropped from 2.5% to 0.5%, NNRTI TDR rose from 8.5% to 13.8%. Subtype B declined over time, while A6, F and G increased (Figure 1).

**Conclusions**: TDR to INSTIs, PIs and NRTIs remained low. In contrast, NNRTI resistance was comparatively higher and rising and the epidemic became increasingly non‐B and recombinant. At Europe's eastern gateway, Türkiye can serve as a sentinel for subtype‐ and mobility‐linked shifts in HIV‐1 resistance. These trends underscore the need for sustained, subtype‐aware surveillance and for extending integrase genotyping beyond the current subset.

**FIGURE 1:**
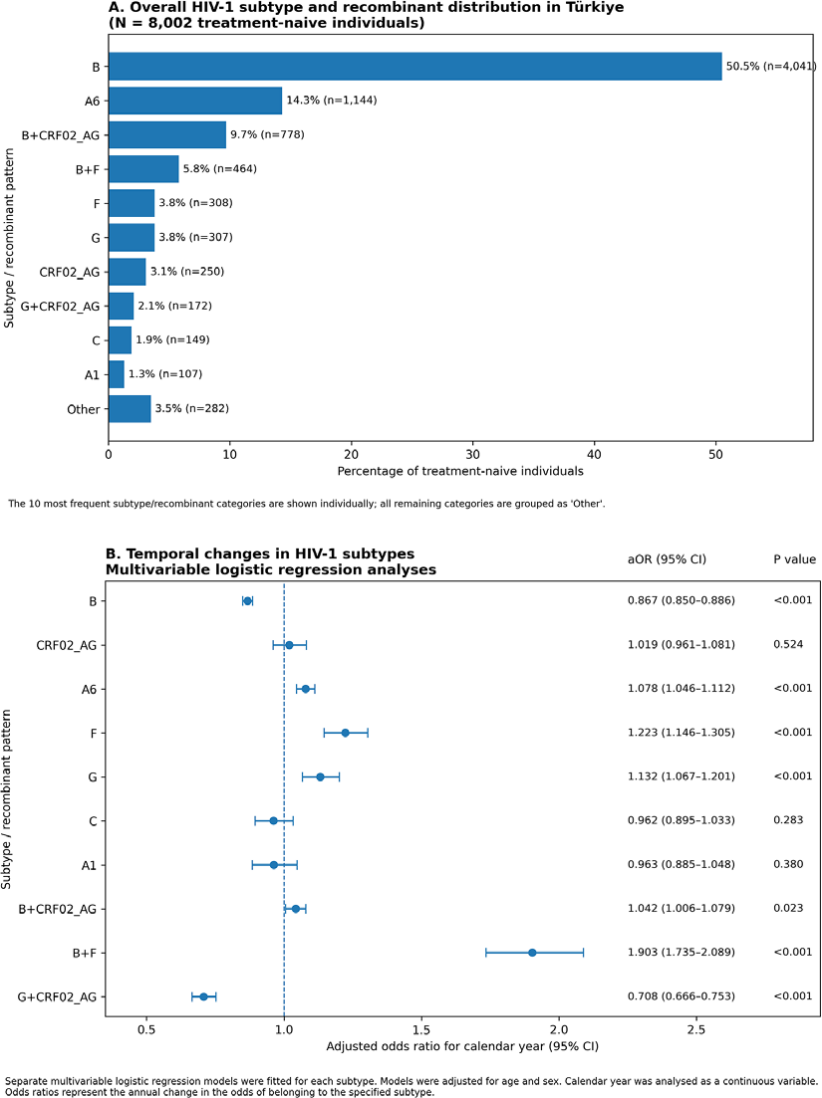
P319 | HIV‐1 subtype diversity and temporal subtype shifts among treatment‐naïve individuals in Türkiye.

**TABLE 1:**
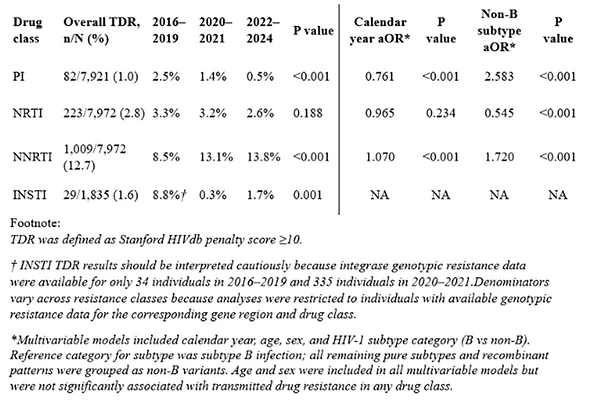
P319 | Class‐specific transmitted drug resistance, temporal trends and subtype‐associated risk.

### P320 Real‐Time Automation and Detection of HIV‐1 Transmission Clusters: Pilot Validation of the SIEGA‐MicrobeTrace System

Paloma Muñoz‐Báez ^1^, Andrea Aguado^2^, Marta Illescas‐López^1^, Antonio Ramirez‐Hurtado^2^, Alberto Vazquez‐Blanquiño^1^, Gema Roldan^2^, Ana Alberola^1^, Maria Lara^2^, Lucia Perez^1^, Javier Perez‐Florido^2^, Adolfo De Salazar^1^, Carlos Sanchez^2^, Casimiro Soriguer^2^, Federico García^1^

^1^Hospital Universitario Clínico San Cecilio, Granada, Spain; ^2^Fundación Progreso y Salud, Sevilla, Spain

**Background**: The integration of automated genomic epidemiology is a paradigm shift in HIV‐1 control. Currently, real‐time transmission cluster detection is not usually performed, limiting public health responsiveness. To bridge this gap, a novel bioinformatic tool has been developed within the SIEGA platform, piloting the MicrobeTrace software. This represents the first tool in Spain designed to enable real‐time molecular surveillance, automating the detection of active networks to guide immediate interventions. The project aims to validate the analytical capacity and epidemiological utility of this new biotool through automated detection, molecular characterization and real‐time monitoring of an active HIV‐1 transmission cluster in Andalusia.

**Methods**: To test the tool's robustness, HIV‐1 reverse transcriptase and protease sequences from a massive database of 7220 samples (collected continuously since 2003 at our institution) were analysed in collaboration with the Progress and Health Foundation's Computational Medicine Platform. The tool's pipeline automatically calculates genetic distances using the Tamura‐Nei (TN93) nucleotide substitution model. A strict genetic distance threshold of < = 1.5% was established to validate real‐time molecular link detection.

**Results**: The biotool's performance enabled the identification of multiple molecular clusters of varying timelines, including two historical networks (39 and 29 patients) and two recent networks (3 and 7 individuals). Since this study focuses on real‐time identification for immediate public health interventions, our analysis centred on the 7‐patient cluster. This represents the most recent, active transmission network to curb further viral expansion. Specifically, the tool successfully identified this automated, tightly connected molecular cluster in Granada (diagnosed October 2022–October 2025). The system alerted to a notable network expansion in 2025 (5 new cases) with significant sociodemographic diversification (age range: 18–74; median: 49 years). Furthermore, the automated network centrality analysis accurately identified the central hub of the outbreak: a patient diagnosed in June 2025 with high global genetic proximity and late presentation criteria (CD4 < 350), proving the software's ability to pinpoint critical drivers of viral dispersion.

**Conclusions**: The pilot implementation of SIEGA constitutes a technological milestone, demonstrating that this new biotool overcomes classic epidemiology limitations by offering Spain's first real‐time molecular surveillance. System validation shows that automated, early network identification provides a crucial window for public health, allowing immediate, geographically targeted screening and prevention strategies.

### P321 Natural Prevalence of HIV‐1 Capsid Inhibitor Resistance‐Associated Mutations in Southern Taiwan: A Retrospective Study (2022–2025)

Hung‐Chin Tsai, I‐Tzu Chen, Ya‐Wei Weng, Yu‐Ting Tseng, Jui‐Kuang Chen, Kuan‐Sheng Wu

Division of Infectious Diseases, Kaohsiung Veterans General Hospital, Kaohsiung, Taiwan

**Background**: Routine HIV‐1 genotypic drug resistance testing typically excludes the capsid region, leading to limited surveillance data. Lenacapavir (LEN) is a first‐in‐class HIV‐1 capsid inhibitor with potent long‐acting properties. This study aimed to investigate the natural occurrence of LEN resistance‐associated mutations (RAMs) among both antiretroviral therapy (ART)‐naïve and ART‐experienced people living with HIV‐1 (PLWH) between 2022 and 2025 in southern Taiwan.

**Methods**: We conducted a retrospective study using Sanger sequencing [1, 2] to identify mutations at positions associated with capsid inhibitors prior to the clinical introduction of LEN in Taiwan. RAMs were defined according to the 2025 International Antiviral Society‐USA (IAS‐USA) update. The HIVdb programme for Capsid was utilized for drug resistance analysis.

**Results**: A total of 113 capsid sequences from 109 individuals were analysed. Subtype B was consisted of 86% (94/109) of PLWH, followed by CRF01_AE 8% (9/109), CRF07_BC 4% (4/109) and subtype C 2% (2/109). RAMs associated with capsid inhibitor resistance were rare, identified in 1.7% (1/60) of ART‐naïve individuals and 0% (0/49) of ART‐experienced individuals. Notably, a Q67H mutation was detected in one ART‐naïve individual. Additionally, a T107A polymorphic mutation was observed transiently in an ART‐experienced individual across five different longitudinal sequencing time points (Figure 1).

**Conclusions**: Our findings demonstrate a low baseline prevalence of capsid inhibitor RAMs among LEN‐naïve PLWH in southern Taiwan. These results provide critical baseline surveillance data and support the potential clinical utility of LEN in this region.

**References**

1. Link JO, Rhee MS, Tse WC, et al. Clinical targeting of HIV capsid protein with a long‐acting small molecule. Nature. 2020;584(7822):614–618

2. Nka AD, Bouba Y, Teto G, et al. Evaluation of HIV‐1 capsid genetic variability and lenacapavir (GS‐6207) drug resistance‐associated mutations according to viral clades among drug‐naive individuals. J Antimicrob Chemother. 2022;78(1):272–275.[Fig jia270211-fig-0356]

**TABLE 1 jia270211-fig-0356:**
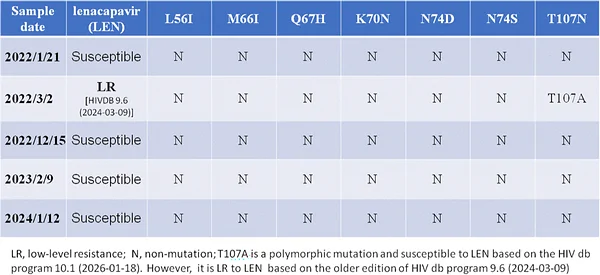
P321 | T107A polymorphic mutation was observed transiently in an ART‐experienced individual across five different longitudinal sequencing time points.

### P322 The RIDING Cohort: Access to Care and 1‐Year Outcomes Among Migrant People With HIV in Madrid

Juan Carlos Donaire^1^, Noemí Cabello‐Clotet^2^, Samuel Manzano^3^, Maria Jesus Vivancos^4^, Rafael Mican^5^, Otilia Bisbal^6^, Violeta Samperiz^7^, Aws Al‐Hayani^8^, Sara De la Fuente^9^, Laura Sindín‐Martín^10^, Cristina Hernández Gutiérrez^11^, Oriol Martin‐Segarra^12^, Alejandra Gimeno^13^, Juan Quintero^14^, Fernando Duran^1^, Nieves Sanz^2^, Pablo Ryan^3^, Belen Milla‐Olaya^4^, María del Mar Arcos Rueda^5^, Belen Sanchez‐Lopez^6^, Lucio Jesus García Fraile Fraile^7^, Alfonso Cabello^8^, Alberto Diaz de Santiago^9^, Irene Cabello‐Rojano^10^, Maria Novella^11^, Marta De Miguel Montero^14^, Olaya Huergo^1^, Claudia Dorta^2^, Miriam Estebánez^14^, Jose Maria Bellon^15^, Teresa Aldamiz‐Echevarria ^1^, on behalf of RIDING Study Group

^1^Department of Infectious Diseases, Gregorio Marañon Hospital, Madrid, Spain; ^2^Hospital Clinico San Carlos, Madrid, Spain; ^3^Hospital Infanta Leonor, Madrid, Spain; ^4^Hospital Ramon y Cajal, Madrid, Spain; ^5^Hospital La Paz, Madrid, Spain; ^6^Hospital Doce de Octubre, Madrid, Spain; ^7^Hospital de La Princesa, Madrid, Spain; ^8^Fundacion Jimenez Diaz, Madrid, Spain; ^9^Hospital Puerta de Hierro, Madrid, Spain; ^10^Hospital Infanta Sofia, Madrid, Spain; ^11^Hospital Principe de Asturias, Madrid, Spain; ^12^Fundacion de Alcorcon, Madrid, Spain; ^13^Hospital de Torrejón, Madrid, Spain; ^14^Fundacion SEIMC‐Gesida, Madrid, Spain; ^15^Fundacion de Investigacion Gregorio Marañon, Madrid, Spain

**Background**: In recent years, Spain has experienced an increasing influx of migrants with HIV (mPWH), with Madrid as the main point of entry. This study aimed to describe the epidemiological characteristics, healthcare access patterns and clinical outcomes of mPWH newly linked to HIV care in Madrid.

**Methods**: We conducted a retrospective, multicentre observational study across 13 hospitals in Madrid. Adult mPWH who initiated follow‐up in HIV units between 1 January 2023 and 30 June 2024 were included. Previously diagnosed and treated individuals were eligible if living in Spain for less than 1 year, while treatment‐naïve individuals were included regardless of time since arrival. Baseline and 12‐month follow‐up data were collected.

**Results**: A total of 1360 mPWH were included: 934 (68.7%) previously treated and 426 (31.3%) treatment‐naïve, of whom 74 (17.4%) presented with acute HIV infection. The cohort was predominantly young (median age 33.1 years), male (85.4%), Latin American (88%) and men who have sex with men (78.6%). Main baseline characteristics, treatment patterns and 12‐month outcomes are shown in Table 1. Despite 44.4% lacking healthcare coverage and 30.5% of pre‐treated individuals reporting ART interruption before entry, retention in care reached 84.6% and virological suppression 91.2% at 12 months. Loss to follow‐up was independently associated with acute HIV infection at presentation (adjusted OR 1.81, 95% CI: 1.21–2.69; *p* = 0.004). Virological failure was independently associated with transgender identity (adjusted OR 3.89, 95% CI: 1.60–9.46; *p* = 0.003).

**Conclusions**: Despite substantial barriers to healthcare access at presentation—including lack of coverage in nearly half of patients and prior ART interruption in almost one‐third of pre‐treated individuals—linkage to specialized HIV care in Madrid achieved high retention and virological suppression rates at 12 months. These findings support strengthening rapid‐access and retention pathways for migrant PWH, with particular attention to groups facing structural vulnerabilities, including transgender women.

**TABLE 1:**
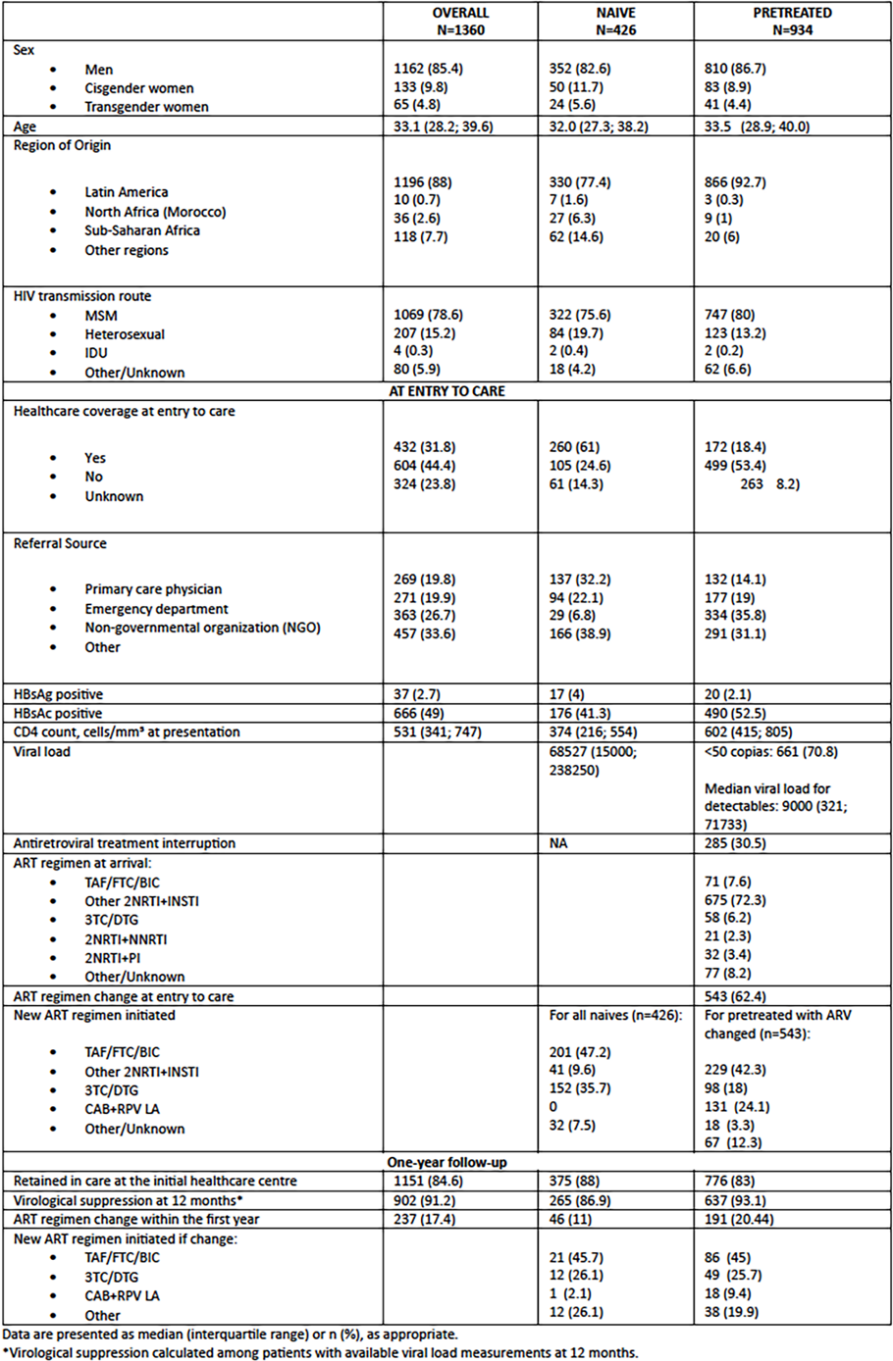
P322 | Characteristics and clinical outcomes of migrant people living with HIV entering HIV care in Madrid.

### P323 Abstract Withdrawn

### P324 Diagnostic Pathways and Missed Opportunities for HIV Testing Among Heterosexual Individuals Newly Diagnosed With HIV

Claudia Nevado Pavón^1^, Xabier Kortajarena Urkola^1^, Maialen Lekuona Sanz^1^, Miguel Ángel Goegana Sánchez ^1^, Harkaitz Azkune Galparsoro^1,2^, Xabier Camino Ortiz de Barrón^1^, Ignacio Álvarez Rodríguez^1^, Maialen Ibarguren Pinilla^1^, Francisco de Asís Carmona de la Torre^1^, Maria Jesús Bustinduy Odriozola^1^, Ana Bayona Carlos^1^, Leire Gil Alonso^1^, Leire Balerdi Sarasola^1^, Ander Goyache Moreno^1^, Jose Antonio Iribarren Loyarte^1,2^

^1^Biogipuzkoa HRI, Infectious Diseases Area, Infectious Diseases and Global Health Research Group, Osakidetza, Donostia University Hospital, Infectious Diseases Service, San Sebastian, Spain; ^2^Medicine Department, EHU, San Sebastian, Spain

**Background**: Early HIV diagnosis remains essential for improving clinical outcomes and reducing transmission. Understanding the epidemiological profile and sexual behaviour of individuals newly diagnosed with HIV may help identify prevention gaps and missed opportunities for earlier diagnosis. This study describes the characteristics of heterosexual (HTX) people newly diagnosed with HIV in an Infectious Diseases Service in the Basque Country between 2021 and 2025.

**Methods**: We conducted a prospective, descriptive and observational study including all individuals with a new HIV diagnosis attending the Infectious Diseases Service in the Basque Country between 2021 and 2025. A structured questionnaire collected sociodemographic information, sexual behaviour and previous HIV/STI testing. Clinical records were reviewed to identify prior healthcare contacts that could represent missed opportunities for earlier HIV diagnosis. For this abstract, only results for HTX are presented.

**Results**: A total of 50 HTX (of 143 total) were included. The median age was 37 years and 58% were men. Most diagnoses occurred outside specialized services; mainly in primary care or other non–HIV/STD‐related specialties, indicating that HTX are predominantly diagnosed in general clinical settings rather than through structured screening pathways. Regarding the reason for test request, clinical symptoms present 48% of cases, while patient initiative represented 32% (Table 1). In addition, 76% of individuals had at least one medical encounter in the 3 years before diagnosis for conditions in which HIV testing is recommended (ECDC/EACS Module on HIV Testing (2025)) [1], revealing multiple missed opportunities for earlier detection (Figure 1). Moreover, 60% of HTX presented CD4 < 350 and 44% advanced disease (CD4 < 200), a markedly higher proportion of advanced immunosuppression than the observed among men who have sex with men in the same cohort (28%) (*p* = 0.0002).

**Conclusions**: In our study, heterosexual individuals were predominantly diagnosed with HIV late, often outside specialized services and mainly after symptoms or self‑initiated testing. Nearly 80% had prior healthcare encounters without HIV screening, representing clear and repeated missed opportunities for earlier diagnosis. The high proportion of advanced disease at presentation highlights the urgent need to reinforce opportunistic testing in primary care and other non‑specialized settings.

**Reference**

1. European Centre for Disease Prevention and Control. (Año). European standards of HIV prevention and care: Module on HIV testing. ECDC.

**FIGURE 1:**
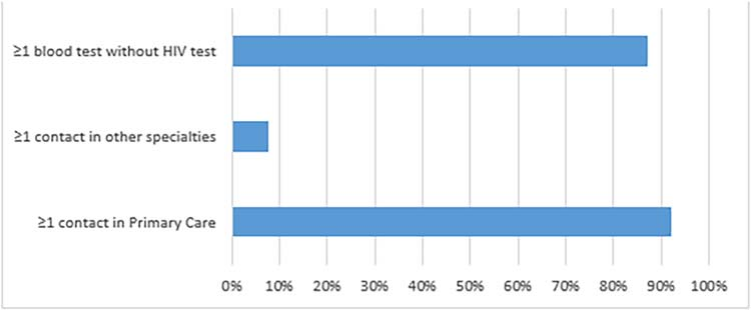
P324 | Missed opportunities for HIV testing in the 3 years prior to diagnosis. 92.3% of patients (36/39) had at least one contact in primary care, while the remaining 7.7% (3/39) were seen only in other specialties. Additionally, 87.2% of all patients (34/39) had at least one blood test performed without an HIV test.

**TABLE 1:**
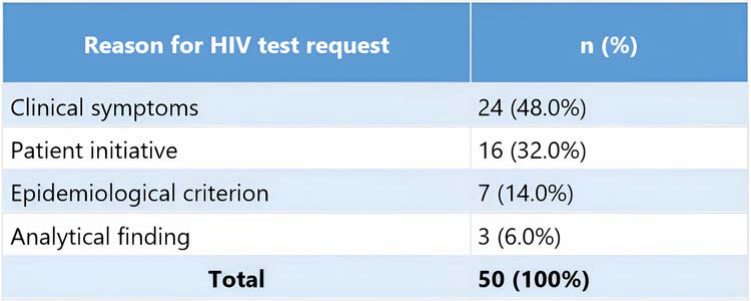
P324 | Diagnostic setting. Half of the diagnoses were triggered by clinical symptoms (24/50; 48%) and one third by patient initiative (16/50; 32%), highlighting the limited proactive testing within the healthcare system.

### P325 Recombinant Zoster Vaccination Uptake and Determinants in People With HIV in the Swiss HIV Cohort Study

Emma Tinguely^1^, Bernard Surial^1^, Alain Amstutz^2^, Marcel Stöckle^2^, Luigia Elzi^3^, Patrick Schmid^4^, Oksana Fiammingo^5^, Alexandra Calmy^5^, Matthias Cavassini^6^, David Haerry^7^, Irene A. Abela^8^, Marius Zeeb ^1,9^, on behalf of Swiss HIV Cohort Study

^1^Department of Infectious Diseases, Bern University Hospital, University of Bern, Bern, Switzerland; ^2^Division of Clinical Epidemiology, Department of Clinical Research, University Hospital Basel, Basel, Switzerland; ^3^Malattie Infettive, Ospedale Regionale di Bellinzona e Valli, Bellinzona, Switzerland; ^4^HOCH Health Ostschweiz, Division of Infectious Diseases, Infection Prevention and Travel Medicine, Department General Internal Medicine, St Gallen, Switzerland; ^5^HIV AIDS Unit & Division of Infectious Diseases, Geneva University Hospitals, University of Geneva, Geneva, Switzerland; ^6^Division of Infectious Diseases, University Hospital of Lausanne, University of Lausanne, Lausanne, Switzerland; ^7^Positive Council, Zurich, Switzerland; ^8^Division of Infectious Diseases and Hospital Epidemiology, University Hospital Zurich, University of Zurich, Zurich, Switzerland; ^9^Division of Infectious Diseases and Hospital Epidemiology, University Hospital Zurich, University of Zurich, Zurich, Switzerland

**Background**: People with HIV (PWH) are at increased risk for herpes zoster and related complications. Since 2022, the recombinant zoster vaccine (RZV) has been recommended in Switzerland for PWH aged ≥50 years or for those with severe immunosuppression, compared with ≥65 years in the general population. We assessed RZV vaccination uptake and associated factors in the Swiss HIV Cohort Study (SHCS).

**Methods**: We analysed data from the SHCS, a nationwide prospective observational cohort of PWH in Switzerland. Individuals under follow‐up between January 2022 and April 2026 were included. Vaccination coverage was estimated overall and across demographic subgroups. Time to first RZV vaccination was analysed using cumulative incidence methods. Factors associated with time to first RZV dose were evaluated using uni‐ and multivariable Cox regression models, adjusted for age, sex, ethnicity, HIV transmission group, cohort centre and educational level.

**Results**: Among 10,902 PWH included in the analysis, 3434 (31.5%) received at least one RZV dose, of whom 2930 (85.3%) received two doses. RZV coverage varied across demographic groups and cohort centres (Table 1). The cumulative incidence of first RZV vaccination increased from 11.3% at the end of 2022 to 21.3% in 2023, 28.4% in 2024 and reached 34.3% at the end of follow‐up (Figure 1A). In multivariable analyses, older age was strongly associated with a higher RZV uptake (hazard ratio [HR] per 10‐year age increase 1.85, 95% confidence interval [CI] 1.80–1.91). RZV uptake was lower in female PWH compared to male (HR 0.84, 95% CI: 0.76–0.93). Men who have sex with men showed increased RZV uptake than those who acquired HIV through heterosexual contacts (HR 1.12, 95% CI: 1.03–1.23) (Figure 1B).

**Conclusions**: Four years after the licensing of RZV vaccine in Switzerland, 31.5% of PWH received at least one RZV dose. However, reimbursement of RZV for PWH aged 50–64 years is currently restricted to those with severe immunosuppression, which may have limited vaccine uptake. Differences across patient groups and centres suggest opportunities to improve and harmonize implementation of vaccination recommendations in HIV preventive care.

**FIGURE 1:**
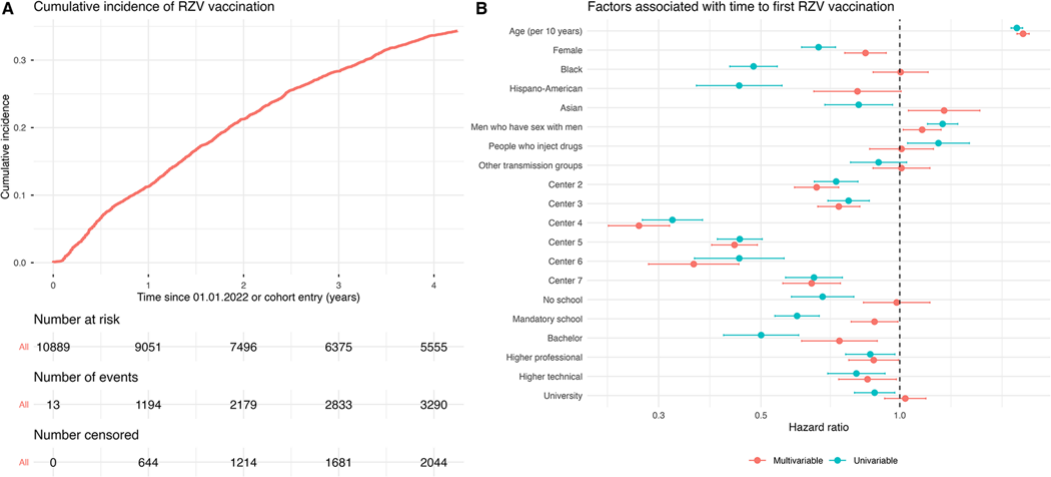
P325 | Cumulative incidence of first RZV vaccination and factors associated with time to first RZV vaccination among people with HIV in the Swiss HIV Cohort Study. (A) Cumulative incidence of first RZV vaccination. (B) Factors associated with time to first RZV vaccination. Forest plot showing hazard ratios (HRs) and 95% confidence intervals from uni‐ and multivariable Cox regression models. Reference categories were male sex, white ethnicity, heterosexual contacts, centre 1 and apprenticeship education.

**TABLE 1:**
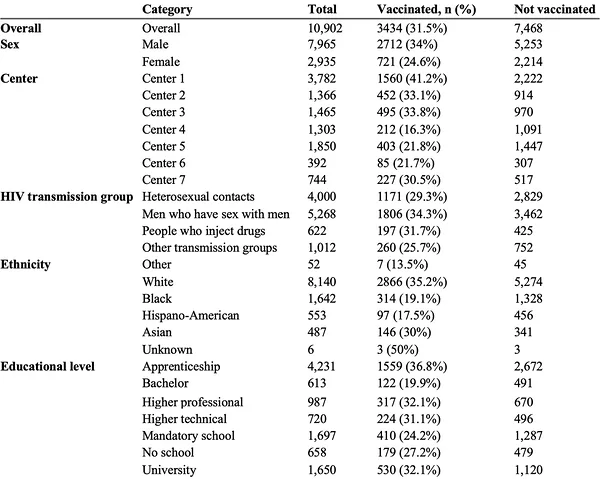
P325 | RZV vaccination coverage by subgroup.

### P326 The Impact of Perceptions: Exploring Patient Opt‐Out Despite Pre‐Test Counselling and Stigma Reduction Efforts in a Standardized Emergency Department STI Screening Programme

Makayla Sinness, Andrew Salter, Jessica Chavez

Emergency Department, CommonSpirit Health, Westminster, CO, US

**Background**: Routine, non‐targeted screening for HIV, hepatitis C (HCV) and syphilis is an important public health strategy for identifying undiagnosed infections and facilitating timely linkage‐to‐care. This emergency department (ED) based screening programme (Denver, Colorado, USA) incorporates pre‐test counselling designed to normalize testing, reduce stigma and support informed decision‐making. A dedicated patient counsellor delivers a concise educational statement outlining the purpose of routine screening, modes of transmission and immediate linkage‐to‐care for abnormal findings. Testing is integrated using existing venepuncture lines to eliminate additional discomfort and requires explicit verbal consent. Understanding why patients decline STI testing despite these interventions is essential to improving programme effectiveness.

**Methods**: This analysis examined patient‐reported reasons for declining STI testing using information documented during pre‐test counselling from 17 June 2025 to 17 June 2026. While not required to provide a reason, many patients voluntarily shared factors influencing their decision to opt‐out. These disclosures provide qualitative insights into patient perspectives and were documented by screening staff. Responses were categorized using thematic analysis and frequencies were calculated to identify common barriers to participation.

**Results**: Recurring themes were identified through qualitative analysis, as illustrated in Figure 1. The most frequently observed opt‐out reason was disinterest (39.79%). Many patients reported recent testing and perceived additional testing as unnecessary (17.92%). Perceived low risk also emerged prominently, often related to a belief of low exposure risk (14.38%). Other common themes included privacy concerns, particularly when family or partners were present during the counselling process (13.13%). Concerns regarding financial responsibility were also reported (6.67%) and a smaller portion declined due to their original ED chief complaint (2.92%). Additionally, 5.21% opted‐out for other miscellaneous reasons.

**Conclusions**: Despite structured pre‐test counselling and efforts to normalize routine STI testing, several barriers continue to influence patient participation. Common reasons included disinterest, prior testing, perceived low risk, privacy concerns, financial considerations and the demands of their original ED chief complaint. These findings suggest patient decision‐making extends beyond awareness and convenience, encompassing social dynamics, perceived vulnerability and healthcare system factors. Understanding these patient‐centred reasons for declining is critical to optimizing routine ED screening programmes and improving public health outcomes.

**FIGURE 1:**
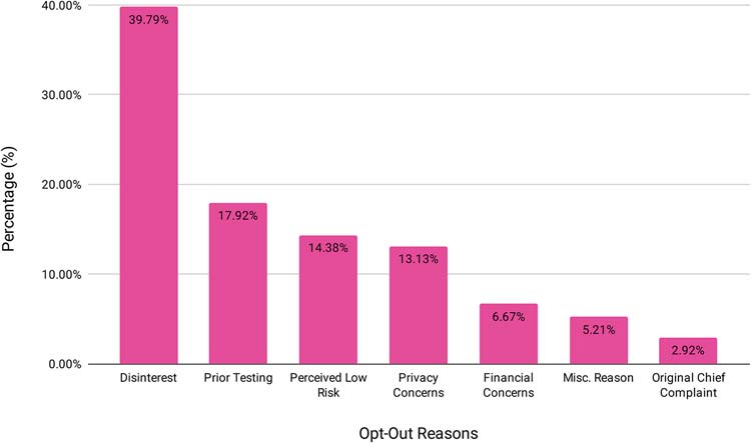
P326 | Patient opt‐out reasons.

### P327 Social Vulnerability Is Associated With Persistent Detectable Viraemia in a Brazilian Real‐World HIV Cohort

Pietra Stanicki ^1^, Karen Ingrid Tasca^2^, Alexandre Naime Barbosa^1^

^1^UNESP – Faculdade de Medicina de Botucatu, Departamento de Infectologia, Botucatu, SP, Brazil; ^2^Faculdade de Medicina de Botucatu, Departamento de Infectologia, Botucatu, SP, Brazil

**Background**: Sustained viral suppression is central to individual health outcomes and population‐level HIV prevention. Despite the high effectiveness of contemporary antiretroviral therapy (ART), a subset of people living with HIV continues to experience detectable viraemia, including persistent low‐level viraemia (PLLV) and early virological failure (EVF). Understanding the demographic and social determinants of persistent detectable viraemia may help identify structural gaps in HIV care.

**Methods**: This observational cohort included 1050 people living with HIV receiving ART at a Brazilian reference centre between the first semester of 2023 and the second semester of 2024. Eligibility required at least four consecutive semi‐annual HIV viral load measurements and a documented ART regimen. PLLV was defined as two or more consecutive viral load measurements between 50–199 copies/mL and EVF as two or more consecutive measurements between 200–499 copies/mL. Demographic and social variables included age, sex, race/skin colour, educational attainment, occupational status, substance use and hepatitis B or C diagnosis. Associations were evaluated using chi‐square tests.

**Results**: Among the 1050 participants, 57 (5.43%) met the definition of persistent detectable viraemia, comprising 25 (2.38%) cases of PLLV and 32 (3.05%) cases of EVF. Older age (≥50 years) was significantly associated with detectable baseline viraemia (*p* < 0.001). Detectable viraemia was also associated with race/skin colour (*p* < 0.001), lower educational attainment (*p* = 0.001), unfavourable occupational status (*p* < 0.001), substance use (*p* < 0.001) and hepatitis B or C diagnosis (*p* = 0.03). No significant associations were observed with sex (*p* = 0.35) or ART regimen.

**Conclusions**: Persistent detectable viraemia was associated primarily with markers of social vulnerability rather than ART regimen characteristics. Older age, lower educational attainment, unfavourable occupational status, substance use and hepatitis B or C coinfection were all associated with adverse virological outcomes. These findings highlight the continued influence of social determinants on HIV treatment outcomes despite universal access to ART in Brazil and support targeted interventions to improve retention in care and sustained viral suppression among vulnerable populations.

### P328 Abstract Withdrawn

### P329 Abstract Withdrawn

### P330 Abstract Withdrawn

### P331 Tale of Three Pandemics—Disparities in HIV Risk Profile, Access to Care and Treatment Success, Comparing a Rural‐Mining Community, a Semi‐Urban Area and an Urban Area in Cameroon

Patrick Herve Mfondoum

Center, Yaounde, Cameroon

**Background**: Improving trends in national indicators mask substantial disparities across geographies and vulnerable populations. Resource‐limited and geographically isolated communities face specific challenges driving low access to care and poor outcomes. Study objective: to compare HIV risk profile, treatment access and outcomes, across communities along the rural‐urban continuum.

**Methods**: This was a 3‐year retrospective cross‐sectional multicentre study of the Cameroon National HIV Treatment programme, 08‐2023 through 08‐2025. Study sites: rural‐mining community (Betare‐Oya), semi‐urban community (Akonolinga), urban community (Bertoua). Inclusion criteria: HIV‐positive and treated at study sites during the study period. Medical charts data were collected as part of programme evaluation. Sociodemographic characteristics, treatment interruption rates (i.e. missing medication for ≥2 months), viral load testing rates, viral suppression status and pregnancy/breastfeeding status for female participants were analysed. Median (interquartile range [IQR]) for continuous variables and percentages for categorical variables were described and narratively compared across sites.

**Results**: 10,786 people were included: 1729 in Betare‐Oya, 2273 in Akonolinga and 6784 in Bertoua. Median age was 28.7; 68.22% were females. Median programme participation was 41 months (IQR 23.0–59.5). Treatment interruption rates were highest in rural‐mining communities (3.74 per 100 person‐months), 3x‐ and 8x‐higher than semi‐urban (1.16/100 person‐months) and urban (0.44/100 person‐months) community rates, respectively. Viral load testing rates were lowest in rural‐mining community (45.9%), about half those of semi‐urban (88%) and urban (79.7%) communities. Among those tested, viral load was comparable between rural‐mining and semi‐urban communities and slightly lower than urban communities (87.0%, 86.8% and 92.6%, respectively), demonstrating narrowing outcomes disparity gaps among patients consistently engaged in care, irrespective of rural‐urban setting. Risk profiles differed along the rural‐urban continuum. The prevalence of pregnancy or breastfeeding among HIV‐treated adolescent females in Betare‐Oya (27%) was five‐fold higher than Akonolinga (5.3%) or Bertoua (4.9%), indicating high vulnerability to sexual transmission among adolescent females in low‐income rural‐mining communities.

**Conclusions**: This tale of three pandemics highlights a vulnerability gradient, with progressively worsening indicators along the rural‐urban continuum. Consistent access to care narrowed the gap while vulnerabilities among adolescent females in rural‐mining communities exacerbate it. National public health strategies should design targeted approaches to increase access to care and decrease sexual transmission among adolescent females in low‐income rural‐mining communities.[Fig jia270211-fig-0365]

**FIGURE 1 jia270211-fig-0365:**
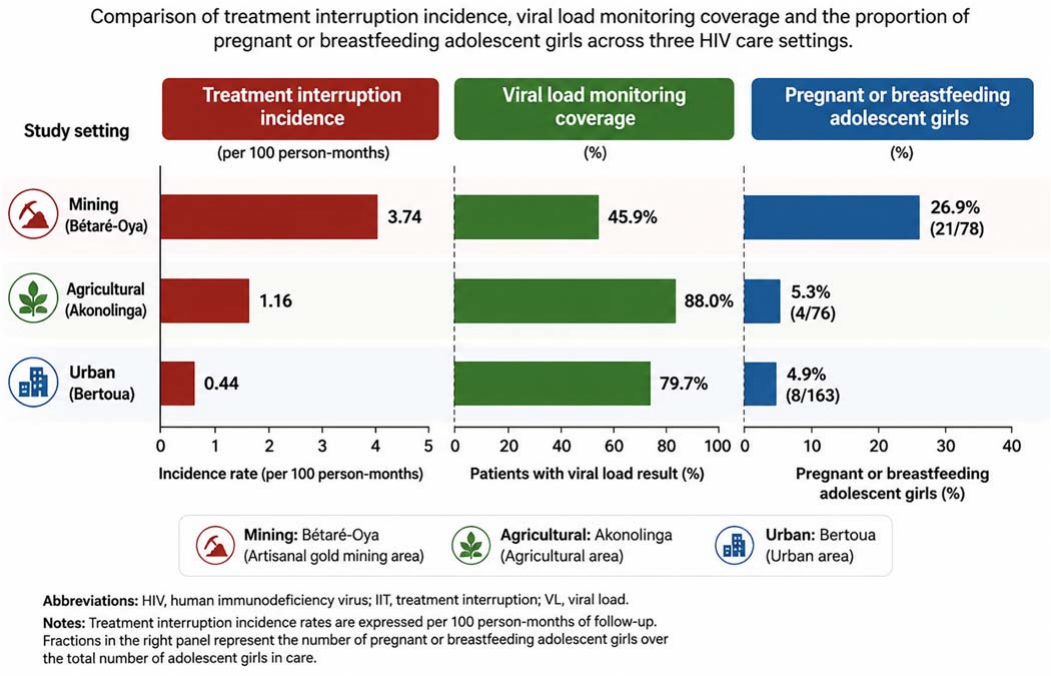
P331 | Triple burden of HIV care across mining, agricultural and urban settings in Cameroon.

### P332 Prevalence of BBV and Syphilis in Those Presenting to Cardiff and Vale Health Inclusion Service (CAVHIS)

Leah Sell ^1^, Rhys Oakley^2^, Bazga Ali^1,2^

^1^Public Health Wales, Cardiff, UK; ^2^Cardiff & Vale Healthboard, Cardiff, UK

**Background**: In February 2026, the UK Health Security Agency (UKHSA) published an evaluation of blood‑borne virus (BBV) opt‑out testing across 34 emergency departments (EDs) in England over a 33‑month period. The programme demonstrated high acceptability, with 70% uptake among eligible attendees and effectively identified new infections, with positivity highest for hepatitis B virus (HBV) (0.42%), followed by hepatitis C virus (HCV) (0.08%) and HIV (0.05%). These findings support recommendations from organizations such as the British HIV Association (BHIVA) and contribute to progress towards the World Health Organization (WHO) target of eliminating HIV and viral hepatitis as public health threats by 2030. However, evidence remains limited regarding opt‑out testing in other high‑risk healthcare populations and no publicly available data exist from Welsh healthcare settings.

**Methods**: The Cardiff and Vale Health Inclusion Service (CAVHIS) provides care for populations underserved by conventional healthcare models, including individuals seeking asylum, people experiencing homelessness, sex workers and people who use illicit drugs. This retrospective analysis includes all patients within this defined cohort who underwent testing for syphilis, HIV, hepatitis B and hepatitis C between 22 September 2021 and 17 September 2025. Data was extracted using the Public Health Wales laboratory integrated management system.

**Results**: A total of 4344 individuals received syphilis testing, 4437 underwent HIV testing, 4437 received hepatitis C antibody testing and 4925 were tested for hepatitis B. Positivity rates were 0.74% for syphilis, 0.47% for HIV, 1.28% for hepatitis C, 1.86% for current hepatitis B infection and 8.74% for evidence of past hepatitis B infection.

**Conclusions**: These findings will inform a review of individuals accessing healthcare through CAVHIS who declined BBV and syphilis testing. The service aims to implement an opt‑out testing model for this cohort and publish subsequent outcomes to support other UK health inclusion services. The overarching goal is to reduce morbidity and mortality associated with infectious hepatitis, syphilis and HIV, prevent onward transmission through early identification and vaccination and contribute to micro‑elimination of viral hepatitis in alignment with WHO elimination targets.

**References**

1. UKHSA, Public health evaluation of BBV opt‐out testing in EDs in England, 33‐month final report 2025 ‐ GOV.UK, Available at URL https://www.gov.uk/government/publications/bloodborne‐viruses‐opt‐out‐testing‐in‐emergency‐departments/public‐health‐evaluation‐of‐bbv‐opt‐out‐testing‐in‐eds‐in‐england‐33‐month‐final‐report‐2025, Accessed 01/05/2026.

2. WHO, Global health sector strategies on respectively, HIV, viral hepatitis & sexually transmitted infections for the period 2022−2030, Available at URL chrome‐extension://efaidnbmnnnibpcajpcglclefindmkaj/https://iris.who.int/server/api/core/bitstreams/6b18ac34‐f56a‐466e‐9660‐b5653cb52ef0/content, Accessed 01/05/2026.

### P333 Integrating a Gender Perspective Into HIV Care: A Qualitative Study in a Specialized Hospital in Buenos Aires, Argentina

Romina Mauas ^1^, María Fernanda Rombini^2^, Isabel Cassetti^3^, Mario Martin Pecheny^4,5^

^1^HIV Outpatient Clinic Unit, Hospital de Infecciosas “F. J. Muñiz”, Buenos Aires, Argentina; ^2^Hospital General de Agudos J.M. Ramos Mejía, Buenos Aires, Argentina; ^3^Sanatorio de la Trinidad, San Isidro, Buenos Aires, Argentina; ^4^Universidad de Buenos Aires, Buenos Aires, Argentina; ^5^Consejo Nacional de Investigaciones Científicas, CONICET, Buenos Aires, Argentina

**Background**: Despite advances in the HIV response in Argentina, the implementation of a comprehensive, people‐centred model of care continues to face barriers that limit equitable access to health services. Gaps persist between the normative framework, social representations and institutional practices perpetuating stigma and inequalities experienced by people living with HIV (PLWH). A gender and intersectional approach provides a framework to examine how institutional and social dynamics shape HIV care beyond formal policies. The aim of this study was to analyse how a gender perspective is integrated into HIV care at the “Francisco J. Muñiz” Infectious Diseases Hospital in Buenos Aires, Argentina.

**Methods**: A qualitative exploratory and descriptive study was conducted based on 22 semi‐structured interviews with healthcare professionals working at the hospital. Fieldwork was carried out between May and September 2025. Participants included 12 infectious disease specialists and 10 professionals from other specialties involved in the care of PLWH, aged 32–64 years. Interviews were audio‐recorded, transcribed and thematically analysed using ATLAS.ti. The study was approved by the institutional Research Ethics Committee. Written informed consent was obtained and confidentiality was ensured.

**Results**: The analysis revealed a gradual and heterogeneous incorporation of the gender perspective in HIV care. Three main patterns emerged: (i) greater development of gender‐sensitive approaches within infectious diseases care compared with other specialties; (ii) increasing professional awareness of gender inequalities and stigma experienced by women living with HIV and gender‐diverse people; and (iii) limited translation of this awareness into transformative care practices at the institutional level. Although many participants recognized the relevance of gender and social vulnerability in shaping health outcomes, the implementation of gender‐sensitive approaches largely depended on individual initiative rather than institutional policies, limiting their systematic incorporation into clinical practice.

**Conclusions**: Integrating a gender perspective into HIV care requires stronger gender mainstreaming and the development of institutional policies that reduce programmatic vulnerability, understood as structural and organizational limitations of the health system and promote more equitable, comprehensive and rights‐based care.

### P334 Factors Associated With HIV‐1 Infection Recency Among Newly HIV Diagnosed Individuals in the Era of “Treat All” in Botswana

Goitseone Lemogang ^1^, Natasha Moraka‐Mankge^1,2^, Charity Ralegoreng^3^, Richard Makwakwa^4^, Marea N. Pema^1^, Patrick T. Mokgethi^1,5^, Ontlametse T. Choga^1,2^, Dorcas Maruapula^1^, Salang T. Moutswi^1^, Laone Rabatoko^1^, Queen Leteemane^1^, Vanessa Strachan^1^, Phenyo Sabone^1^, Terence Mohammed^1^, Sikhulile Moyo^1,6^, Simani Gaseitsiwe^1,6^

^1^Research Department, Botswana Harvard Health Partnership, Gaborone, Botswana; ^2^Faculty of Health Sciences, School of Allied Health Professionals, University of Botswana, Gaborone, Botswana; ^3^Serology Department, Botswana Harvard Health Partnership, Gaborone, Botswana; ^4^Viral Load Department, Botswana Harvard Health Partnership, Gaborone, Botswana; ^5^Department of Biological Sciences, University of Botswana, Gaborone, Botswana; ^6^Department of Immunology and Infectious Diseases, Harvard University, T.H. Chan School of Public Health, Boston, MA, US

**Background**: To establish useful factors for HIV intervention strategies in greater Gaborone, Botswana, we here aimed to investigate HIV infection recency and its associated factors among newly diagnosed individuals near time of seroconversion.

**Methods**: This analysis used plasma samples from a prospective longitudinal cohort study of individuals recently diagnosed with HIV within the Greater Gaborone area (2023–2025), the Tekodiso study. Recent infection classification was established using Limiting Antigen Avidity (LAg‐Avidity) assay, stratified by HIV viral load (VL) > 1000 copies/mL. Where LAg‐normalized optical density (ODn) ≤1.5 represents a recency window of within 130 days post‐seroconversion. HIV VL in plasma was quantified by Abbott m2000sp/Abbott m2000rt. Univariate logistic regression was used to identify factors associated with recent HIV infection.

**Results**: A total of 410 participants were included in this analysis. Median age at enrolment was 33 years (Q1, Q3: 27, 40) and majority 262 (63.9%) were female. The median log10 HIV viral load (HIV VL) was 4.5 copies/mL (Q1, Q3: 3.6, 5.2). The median CD4 count was 349 cells/mL (Q1, Q3: 206, 531). A total of 34/381 (8.9%; 95% CI: 6.3–12.2) individuals were classified as having a recent infection by LAg‐based RITA. We observed a lower likelihood of recent HIV infection with having secondary or higher education (OR 0.8; 95% CI: 0.3–2.3). Females were 2.7 times more likely to be classified as having recent HIV infection than males. HIV recent infection was also associated with non‐Motswana nationality (OR 1.5; 95% CI: 0.7–3.4) and a trend for high viral load (OR 1.3; 95% CI: 0.9–1.8). Recent infection was not associated with baseline CD4 count or employment status.

**Conclusions**: We report moderate levels of HIV infection recency among newly HIV diagnosed individuals in Greater Gaborone, Botswana. Recency of infection was associated with being female and having non‐Motswana citizenship status. Our results contribute to national efforts to establish targeted interventions for prevention and monitoring of HIV transmission in Botswana.

### P335 Developing an AI‐Powered Chatbot Integrated Into an Anonymous STI Partner Notification App: Insights From a Survey Among PrEP Users

Felix Straga, Maria del Carmen Navarro Sáez, Marta Navarro Vilasaró, Sonia Calzado Isbert

Infectious Diseases, Sabadell, Barcelona, Spain

**Background**: Despite regular engagement in specialized pre‐exposure prophylaxis (PrEP) care, barriers to discussing sexual health may persist. Embarrassment, fear of judgement and unmet informational needs may hinder access to reliable sexual health information and support. An artificial intelligence (AI)‐powered chatbot integrated into an anonymous sexually transmitted infection (STI) partner notification app could provide confidential support. We explored PrEP users’ perceptions, informational needs and preferences to inform its development.

**Methods**: An anonymous cross‐sectional survey was conducted among attendees of the specialized PrEP clinic at Hospital Universitari Parc Taulí (Sabadell, Spain) between May and June 2026. Participants completed a self‐administered questionnaire accessed via QR code during routine visits. The survey assessed comfort discussing sexual health with healthcare professionals, perceived barriers, informational needs, preferred chatbot functionalities and trust in AI‐generated information. Descriptive analyses were performed. Overall, 107 participants completed the survey.

**Results**: Participants had a mean age of 39 years; 92.5% identified as men who have sex with men and 83.2% were active PrEP users. Participants reported greater comfort discussing sexual health with PrEP specialists than with general practitioners (91.3% vs. 63.8% reporting feeling fairly or very comfortable, respectively). Nevertheless, fear of being judged (36.9%) and embarrassment (24.3%) remained common barriers. Informational needs were substantial, particularly regarding STIs and testing (90.1%), PrEP/PEP (61.4%), sexuality‐related mental wellbeing (39.6%), safer sexual practices (35.6%) and partner notification (30.7%) (Table 1). The chatbot was perceived as most useful following sexual risk exposure (86.7%) or when experiencing symptoms suggestive of an STI before seeking medical attention (83.1%). Screening reminders were considered a desirable functionality by 83.1% of respondents, while 69.1% preferred referral to a healthcare professional if the chatbot was unable to answer a query.

**Conclusions**: Among PrEP users, specialized services appear to facilitate discussions on sexual health; however, communication barriers and unmet informational needs persist beyond dedicated HIV prevention settings. An AI‐powered chatbot integrated into an anonymous STI partner notification app may complement clinician‐delivered care by providing confidential information, supporting timely testing and facilitating access to healthcare services.

**TABLE 1:**
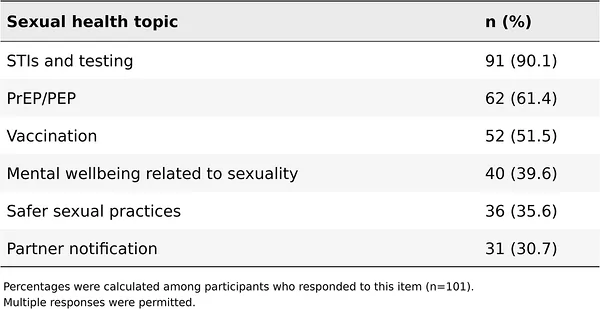
P335 | Sexual health topics participants would like to discuss with an AI‐powered chatbot.

### P336 A 12‐Fold Expansion of Subtype F and the Rise of E138A: Molecular Transformation of the HIV‐1 Epidemic in Southern Türkiye, 2016–2024

Aslıhan Candevir^1^, Tülin Demir^2^, Ferit Kuşcu ^1^, Süheyla Kömür^1^, Ayşe Seza İnal^1^, Damla Ertürk^1^, Özgür Ogün Sayın^1^, Ömer Faruk Yardımcı^1^, Behice Kurtaran^1^, Yeşim Taşova^1^

^1^Infectious Diseases, Adana, Türkiye; ^2^Microbiology, Kırşehir, Türkiye

**Background**: The molecular epidemiology of HIV‐1 in Türkiye is undergoing rapid diversification, yet contemporary data integrating transmitted drug resistance (TDR), subtype evolution and virologic outcomes remain limited [1, 2]. We aimed to characterize resistance patterns, subtype dynamics and their clinical implications in a large treatment‐naïve cohort from southern Türkiye.

**Methods**: In this retrospective cohort study, 813 antiretroviral‐naïve individuals diagnosed with HIV‐1 between 2016 and 2024 at the Çukurova University HIV referral centre were included. Resistance mutations were interpreted using the Stanford HIV Drug Resistance Database (HIVdb v9.8) and subtypes were assigned using the Rega HIV‐1 Subtyping Tool. Temporal trends in subtype distribution, resistance profiles and virologic outcomes were evaluated. Virologic suppression was assessed at 6 months and 1 year. Statistical analyses were performed using Jamovi.

**Results**: Overall TDR prevalence was 18.0% (146/813), driven predominantly by NNRTI‐associated resistance (14.3%) (Table 1). The most striking finding was the dominance of the E138A mutation, detected in 10.0% of all treatment‐naïve individuals, making it the single most frequent resistance‐associated mutation in the cohort. A total of 25 HIV‐1 subtypes and recombinant forms were identified. While subtype B remained predominant (44.4%), a marked molecular shift was observed over time, characterized by a twelve‐fold increase in F‐clade viruses, rising from 2.6% in 2016 to 31.3% in 2024 (Figure 1). E138G demonstrated a strong subtype‐specific association, with 88.9% of cases occurring in subtype F viruses (*p* < 0.001), suggesting a clade‐related polymorphic background. S68G clustered within F‐related lineages and was present in all B+F recombinant strains. Clinically, NNRTI resistance was significantly associated with failure to achieve virologic suppression at 6 months (*p* = 0.002). Patients with virologic non‐suppression had substantially higher baseline viral loads and lower CD4+ T‐cell counts at both 6 months and 1 year (*p* < 0.001). Major transmitted INSTI resistance remained uncommon (1.0%).

**Conclusions**: This study reveals a substantial molecular transformation of the HIV epidemic in Southern Türkiye, characterized by rapid expansion of F‐related viral lineages and dominance of E138A. The observed relationship between baseline NNRTI resistance and early virologic outcomes highlights the continuing clinical relevance of resistance surveillance despite widespread use of INSTI‐based regimens. These findings support expanded baseline genotypic testing and underscore the need for nationwide molecular surveillance incorporating phylogenetic and next‐generation sequencing approaches.

**References**

1. Ödemiş İ, Akbulut İ, Duru Öz E, Harput AC, Özcan BY, Demir T, et al. Transmitted HIV‐1 drug resistance and subtype distribution among treatment‐naïve individuals at a tertiary referral center in the Aegean Region of Türkiye: An eight‐year cohort study. Infection. 2026.

2. Khodadad, N., Hashempour, A., Nazar, M.M.K. et al. Evaluating HIV drug resistance in the middle East and North Africa and its associated factors: A systematic review. *Virol J* 22, 112 (2025).

**FIGURE 1:**
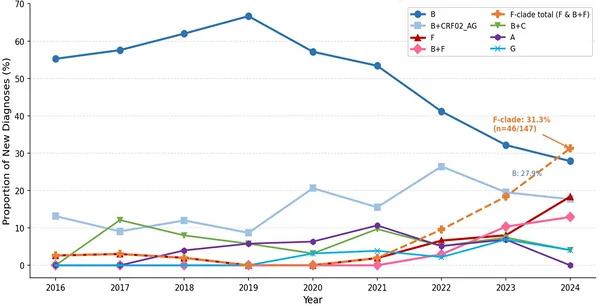
P336 | Temporal trends in HIV‐1 subtype distribution (2016–2024).

**TABLE 1:**
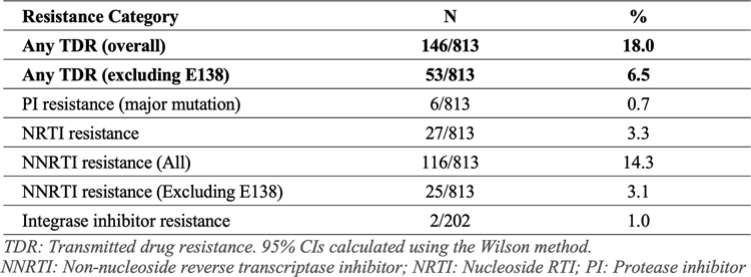
P336 | Transmitted HIV drug resistance (TDR) rates by drug class.

### P337 Burden of Socio‐Structural Vulnerability and Self‐Perceived Health in People Living With HIV Receiving Clinical Care in Mexico

Hector Rivera, Jessica Mejia, Yanink Caro Vega, Fernanda Ramos, Ever Corral, Alvaro López Iñiguez, Paola Alarcon, Geovanna Coello, Brenda Crabtree Ramirez

Infectious Diseases Department, Instituto Nacional de Ciencias Medicas y Nutricion Salvador Zubiran, Mexico City, Mexico

**Background**: In people living with HIV (PLWH), clinical success is commonly assessed through viral suppression, functional status and retention in care [1]. However, these indicators may not reflect perceived health [2, 3]. We evaluated whether greater cumulative socio‐structural vulnerability was associated with poorer self‐perceived health among PLWH receiving care at a referral clinic in Mexico City.

**Methods**: We conducted a cross‐sectional analytical study of PLWH attending outpatient care during 2024–2025 who were invited to complete socio‐structural and health questionnaires. Self‐perceived health was assessed with the EQ‐VAS, ranging from 0 to 100, with higher scores indicating better perceived health. Cumulative socio‐structural vulnerability was defined as the proportion of affected domains across seven pre‐specified domains: low income, unemployment, low educational attainment, lack of social security, residential mobility, longer distance to HIV care and reported discrimination. When one or two domains were missing, burden was calculated among available domains; at least five evaluable domains were required. Vulnerability was categorized as low (<33%), intermediate (33– < 67%) or high (≥67%) for descriptive analyses and analysed as a continuous variable in linear regression adjusted for age, sex and years since HIV diagnosis.

**Results**: EQ‐VAS was available for 395 participants. Median age was 47.4 years (IQR 37.8–55.7) and 323 (81.6%) identified as men. 83.5% were virologically suppressed, 82.1% had a Karnofsky score of 10 and median CD4 count was 610 cells/µL. Vulnerability burden was low in 113 participants (26.4%), intermediate in 206 (48.1%) and high in 83 (19.4%). High vulnerability was more frequent in women than men (39.0% vs. 18.0%). Mean EQ‐VAS decreased across vulnerability groups: 83.7, 83.0 and 73.8 in low, intermediate and high vulnerability, respectively. Any EQ‐5D problem increased with vulnerability burden, while CD4 count, viral suppression and Karnofsky score did not show clear differences across groups (Table 1). In the adjusted model, each 25% increase in socio‐structural vulnerability was associated with a 4.0‐point lower EQ‐VAS score (β = −3.96; 95% CI: −6.18 to −1.73; *p* = 0.0005; *n* = 370).

**Conclusions**: In PLWH with high rates of viral suppression and preserved functional status, greater socio‐structural burden was associated with worse self‐perceived health, supporting the incorporation of patient‐reported outcomes and socio‐structural factors into the assessment of PLWH to identify needs not reflected by traditional clinical markers.

**References**

1. Cowden M, Clavijo A, Bitar Y, Diaz M, Merced X, Meza K, Iyengar P, Avery A. Prevalence of social determinants of health risk factors among and their impact on viral suppression, consistent visits and no‐show rates among persons with HIV who identify as Hispanic. AIDS Behav. 2025;29(5):1598–1607.

2. Shokoohi M, Bauer GR, Kaida A, Lacombe‐Duncan A, Kazemi M, Gagnier B, de Pokomandy A, Loutfy M; CHIWOS Research Team. Social determinants of health and self‐rated health status: A comparison between women with HIV and women without HIV from the general population in Canada. PLoS One. 2019 Mar 21;14(3):e0213901.

3. Cholli PA, Buchacz KM, Harris NS, Crim SM, Yuan X, Tie Y, Koenig LJ, Beer L. Self‐rated health and HIV outcomes among adults with diagnosed HIV ‐ Medical Monitoring Project, United States. AIDS. 2024;38(8):1237–1247.

**TABLE 1:**
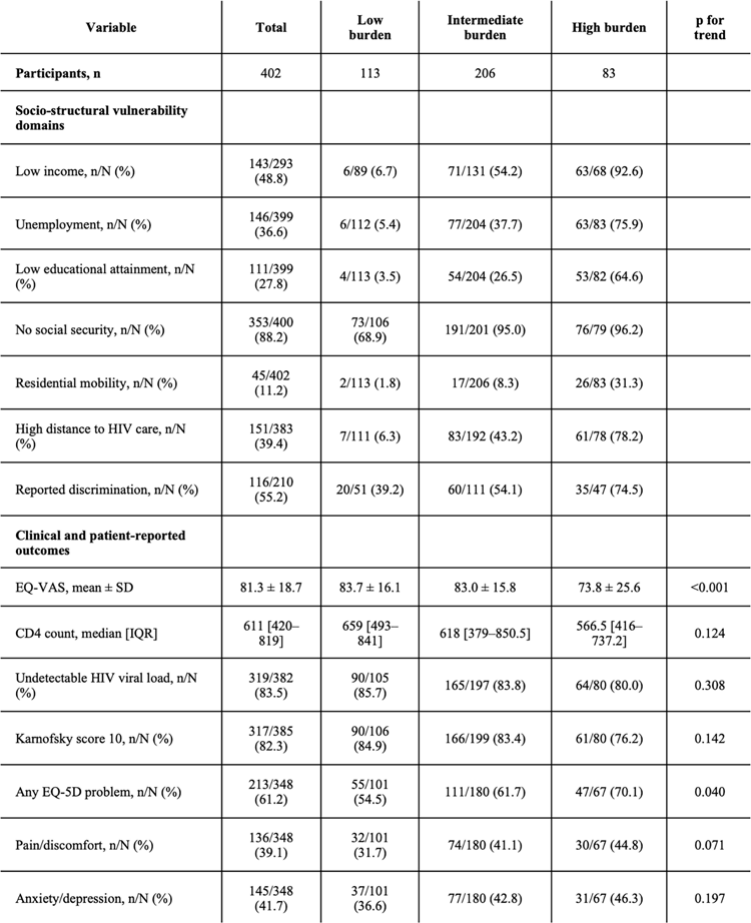
P337 | Socio‐structural vulnerability domains and clinical and patient‐reported outcomes by vulnerability burden category. The total column includes participants with calculable vulnerability burden. Denominators vary according to data availability for each variable.

### P338 Implementation of an Infectious Diseases Clinic for Sexual Diversity Within the Public Health System (PHS) of Buenos Aires, Argentina

Iael Altclas, Maria Teresa Rodriguez Brieschke, Pablo Scapellato

Infectious Diseases Department, Hospital Santojanni, Buenos Aires, Argentina

**Background**: The LGBTTTIQ+ community faces systemic health inequities driven by stigma and structural barriers to HIV/STI care [1–3]. In Argentina, despite combined prevention (CP) expansion [4], an implementation gap persists: while 120,000 individuals are PrEP‐eligible, public coverage reached only 3000 by early 2023 [5]. To decentralize care, a specialized infectious diseases clinic for sexual diversity was established at Hospital Santojanni. Aligned with human rights frameworks [6–8], this initiative created a rights‐based, gender‐sensitive space to improve access to PrEP/PEP and integrated HIV follow‐up, targeting historical barriers that distance this community from public healthcare [9, 10].

**Methods**: An implementation research study (November 2023–November 2025) evaluated the clinic's integration into the PHS. Four outcomes were analysed: acceptability/adoption/appropriateness/feasibility. Primary data were collected through structured surveys administered to users before and after their first consultation. Clinical data were retrieved from electronic medical records. Recruitment was performed via social media and internal hospital referrals. Analysis focused on resource utilization, service uptake and outreach to excluded populations

**Results**: The clinic enrolled 101 individuals (450 consultations; average 4.4/person). Primary motives for consultation were PrEP (61%), HIV follow‐up (22%) and STIs (14%) (Figure 1). Among those with available data (*n* = 69), 36% reported ≥4 sexual partners in the last month, confirming a high‐risk profile.

Acceptability: Surveys (*n* = 59) showed high satisfaction: 98% valued the exclusive space, 95% reported respect for gender identity and 87% rated care quality as excellent.

Adoption: It became an institutional reference for CP, showing longitudinal follow‐up, steady user growth (Table 1) and inter‐service coordination.

Appropriateness: The device adapted to diverse care trajectories, transitioning 2 of 3 of emergency PEP users to PrEP and progressively diversified to include trans and non‐binary identities.

Feasibility: It proved viable utilizing existing physical space, limited human resources and social media to bypass traditional appointment barriers. Sustainability remains conditioned on formal institutional integration post‐funding.

**Conclusions**: The specialized, rights‐based clinic demonstrated high acceptability and reduced structural barriers for highly vulnerable populations. While feasible within existing public hospital structures, long‐term sustainability remains a challenge. This model provides evidence for public policies aimed at decentralizing CP and guaranteeing health equity for the LGBTTTIQ+ community.

**References**

1. Directorate of HIV, STIs, Viral Hepatitis and Tuberculosis. Comprehensive health care for trans people: Recommendations for health teams [Atención integral de la salud de las personas trans]. Buenos Aires: National Ministry of Health; 2018.

2. Directorate of Sexual and Reproductive Health. Comprehensive health care for trans, transvestite and non‐binary people: A guide for health teams. Buenos Aires: National Ministry of Health; 2020.

3. Pan American Health Organization. For the Health of Trans Persons: Elements for the development of comprehensive care for trans persons and their communities in Latin America and the Caribbean. Washington, D.C.: PAHO; 2013.

4. Directorate of HIV, STIs, Viral Hepatitis and Tuberculosis. Combined HIV and STI Prevention: Implementation Guide. Version 1.0. Buenos Aires: National Ministry of Health; 2021.

5. Directorate of HIV, STIs, Viral Hepatitis and Tuberculosis. 2025 HIV and STI Bulletin in Argentina [Boletín sobre el VIH y las ITS en la Argentina]. Buenos Aires: National Ministry of Health; 2025.

6. Argentina. Gender Identity Law No. 26.743. Official Gazette of the Argentine Republic, July 2, 2012.

7. The Yogyakarta Principles: Principles on the application of international human rights law in relation to sexual orientation and gender identity. 2007.

8. Argentina. Same‐Sex Marriage Law No. 26.618 [Ley de Matrimonio Igualitario]. Official Gazette of the Argentine Republic, July 22, 2010.

9. Directorate of HIV, STIs, Viral Hepatitis and Tuberculosis. Friendly Clinics: A first step in the care of people of sexual diversity. Buenos Aires: National Ministry of Health; 2018.

10. Fajardo Flores C, et al. Implementation research: Characteristics and opportunities for practice. Rev Med Hondur. 2019; 87(2).

**FIGURE 1:**
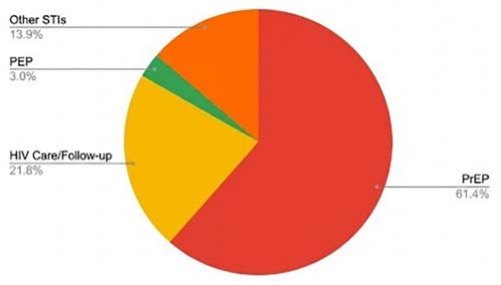
P338 | Primary reasons for consultation.

**TABLE 1:**
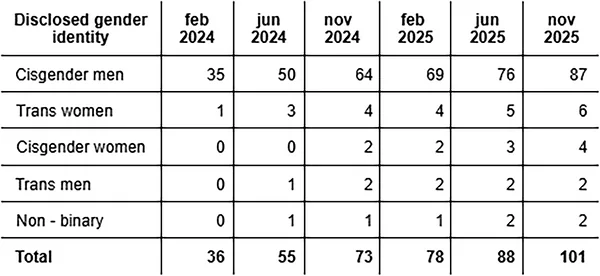
P338 | Steady user growth.

### P339 Client‐Centred Digital Approaches for Ensuring Continuity of HIV Care and Support Programmes in Ukraine

Nataliia Zapolska, Diana Shevchenko, Larysa Hetman, Liudmyla Legkostup

Department of HIV Management and Response, Public Health Center of the Ministry of Health of Ukraine, Kyiv, Ukraine

**Background**: Achieving the 95‐95‐95 targets requires effective approaches to linking people living with HIV (PLWH) to care and supporting treatment adherence. The national HIV care and support (C&S) programme, implemented by non‐governmental organizations, is an important component of Ukraine's HIV response and contributes to achieving national strategic goals in HIV prevention and treatment. The programme is implemented throughout Ukraine, except for temporarily occupied territories and aims to link clients to care, retain them in treatment and support long‐term adherence to antiretroviral therapy. Digital solutions were introduced to improve service accessibility, client‐centredness and programme management.

**Methods**: A digital solution was introduced in the fourth quarter of 2023 through the Information and Analytical System “Management of Services in the Field of Combating Socially Dangerous Diseases” (IAS MSSDD) and the Public Health mobile application to improve the quality and client‐centredness of the national HIV C&S programme. The system enabled electronic documentation, monitoring and service delivery in both face‐to‐face and remote settings. Synchronization with the Information System “Service Management in the Field of Socially Significant Diseases” (IS SSD), together with telephone and SMS verification, was implemented to enhance data reliability and quality assurance.

**Results**: In 2024, HIV C&S programmes reached 22,678 clients, increasing to 34,496 clients in 2025, representing a 52% increase compared with the previous year. Digital tools improved service accessibility, expanded programme coverage and reduced the administrative burden on staff. The platform is used across all regions of Ukraine except temporarily occupied territories. Synchronization with IS SSD ensured confirmed HIV status for all programme beneficiaries and helped prevent duplication of services. A multi‐level verification system ensured the reliability of programme data and supported effective service quality control.

**Conclusions**: Client‐centred digital approaches improved accessibility of the national HIV C&S programme and ensured continuity of services. The combination of mobile service delivery, electronic documentation and multi‐level verification strengthened programme management, supported service quality and enhanced the reliability of programme data. These approaches proved particularly valuable in the context of war, migration and high population mobility.

### P340 HIV Infection, Confirmed Recent Infection and Sentinel Events Among Newly Diagnosed HIV Cases in Enugu State: Insights From Case‐Based Surveillance

Anuli Oyeka ^1^, Oluwaseun Garuba^1^, Yakubu Yusuf^1^, Patience Muogbo^1^, Bidi Tordue^1^, Chukwudi Okwor^1^, Fabian Bassey^1^, Ifeoma Tony‐Monye^1^, Victor Enejoh^1^, Mabwe Kubwa^1^, Eugene Onu^1^, Lawrencia Agur^1^, Young Oluokun^1^, Amana Effiong^1^, John Oko^1^, Olumide Okunoye^2^, Olufemi Oke^2^, Flora Nwagagbo^2^, Jerry Gwamna^2^

^1^Catholic Caritas Foundation of Nigeria (CCFN), Abuja, Nigeria; ^2^Division of Global HIV and TB, United States Centers for Disease Control and Prevention, Abuja, Nigeria

**Background**: HIV case‐based surveillance (HIV‐CBS) tracks individuals from diagnosis through treatment and outcomes, supporting epidemic monitoring and programmatic decisions. Understanding demographics, recency of infection and early clinical outcomes among newly diagnosed individuals can strengthen case finding, prevention, and linkage to and retention in care. This review analysed the distribution of newly diagnosed HIV cases, estimated the proportion of recent infections and assessed key sentinel clinical events in Enugu State, Nigeria.

**Methods**: A retrospective desk review of HIV‐CBS data was conducted across 39 systematically selected health facilities in Enugu State, using records from the National Data Repository and Electronic Medical Records collected between October 2023 and September 2024. Descriptive analyses summarized demographic characteristics, recency testing outcomes and sentinel events along the HIV care continuum. Associations between recent HIV infection and selected socio‐demographic variables were assessed using chi‐square tests, with statistical analyses done using SPSS.

**Results**: Among 2541 newly diagnosed HIV cases, 2532 (99.6%) initiated antiretroviral therapy; 60.8% were female and 39.2% male, and the mean age was 35.2 ± 11.8 years. Of 2135 people eligible for recency testing, 2066 (96.8%) were tested, with 33 (1.6%) recent infections identified. The highest proportion of recent infections was recorded in Enugu North (9; 27.3%), followed by Enugu East (7; 21.2%), and Nsukka (6; 18.2%), with the remaining cases (11; 33.3%) distributed across other local government areas (Figure 1). Recent infections were significantly associated with marital status (single and married), occupation (unemployed and students) and educational level (secondary and tertiary education) (*p* < 0.001), but not with sex or age. Sentinel events showed that 506 (20%) Advanced HIV disease cases were identified, with 426 (84%) being CD4 < 200 cells/mm^3^. Of 1351 people living with HIV eligible for a viral load test, 876 (65%) had results, with a suppression rate of 95%. Overall attrition was 17.4% (441), primarily due to treatment interruption (81.4%; 359), with 9.8% (43) mortality (Figure 2).

**Conclusions**: HIV‐CBS in Enugu State highlights persistent geographic and socio‐demographic disparities in recent infection and a substantial burden of AHD at diagnosis. These findings demonstrate the value of HIV‐CBS as a public health strategy for identifying transmission hotspots, monitoring programme performance, and guiding targeted prevention and care interventions to improve HIV outcomes.

**FIGURE 1:**
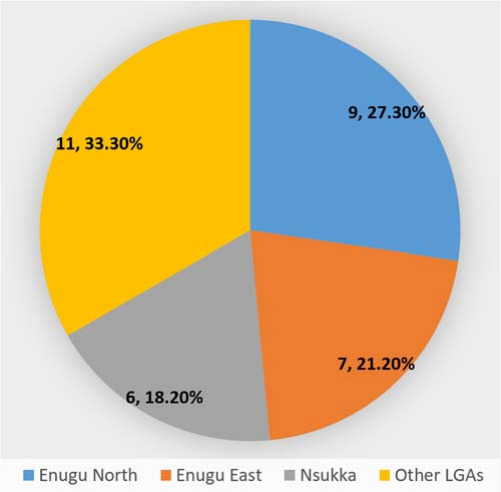
P340 | Distribution of confirmed recent HIV infections by Local Government Areas (LGAs) in Enugu State.

**FIGURE 2:**
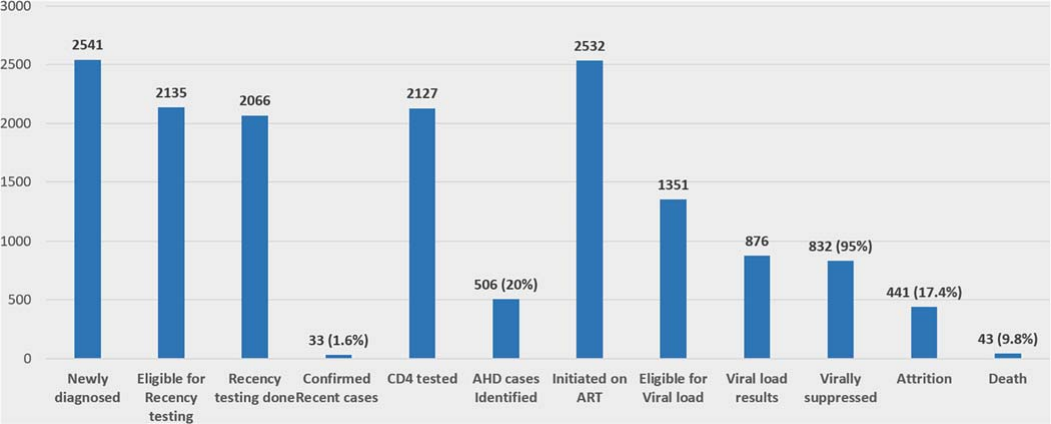
P340 | Cascade of sentinel events among newly diagnosed HIV cases in Enugu State.

### P341 HIV‐1 Subtypes Dynamic and Transmission Drug Resistance Among Patient Followed in Tel Aviv Area Between Years 2001–2025

Dan Turner ^1,2^, Rotem Marom^1^, Katia Levytskyi^3^, Steven Rapaport^1^, Eugene Katchman^1,2^, Svetlana Ahsanov^1^, Natasha Matus^1^, Shirley Girshengorn^1^, Luba Tau^1,2^

^1^CRUSAID Kobler AIDS Center, Tel Aviv Sourasky University Medical Center (Ichilov), Tel Aviv, Israel; ^2^Gray Faculty of Medical and Health Sciences, Tel Aviv University, Tel Aviv, Israel; ^3^Microbiology Laboratory, Tel Aviv Sourasky University Medical Center (Ichilov), Tel Aviv, Israel

**Background**: The demographic characteristics of patients in the Tel Aviv area likely differ from those in other regions of Israel. This retrospective study aimed to characterize the current distribution of HIV‐1 subtypes in Tel Aviv and to assess the prevalence and patterns of transmitted drug resistance (TDR).

**Methods**: HIV‐1 subtypes and transmitted drug resistance (TDR) mutations in the protease (PR) and reverse transcriptase (RT) regions were identified using the Stanford University HIV Drug Resistance Database. TDR mutations were defined according to Surveillance of Transmitted HIV‐1 Drug‐Resistance [1]. The integrase region was excluded due to limited sequence availability prior to 2017. Phylogenetic analysis was conducted to identify genetic clustering and infer transmission networks.

**Results**: A total of 1584 HIV‐1 sequences from treatment‐naïve patients (2000–2025) were analysed. Subtype B was predominant (*n* = 834), followed by A6 (*n* = 370), A3 (*n* = 60) and A1 (*n* = 47). Sub‐subtype A3 emerged after 2010 and expanded markedly after 2020. Subtype B infections were predominantly observed among men who have sex with men (MSM; 87%), with fewer cases among heterosexual individuals (7.5%). Sub‐subtypes A1 (66.6%) and A3 (73.3%) were also primarily detected among MSM. Most A6 infections occurred in individuals from Eastern Europe and Central Asia (EEU/CA) (75%), with transmission mainly via heterosexual contact (37%) and intravenous drug use (IDU; 37%). Phylogenetic analysis identified three A6 clusters: one associated with IDU and two with MSM networks. Clusters in other subtypes were also predominantly observed among MSM. Subtype C was mainly found among individuals from East Africa (55.7%), with 18% of cases occurring in Israeli‐born individuals, predominantly MSM. TDR prevalence remained stable (10–12%) and was mainly associated with NNRTI mutations. Most TDR cases occurred in subtype A1 among MSM and were frequently clustered, followed by subtypes B and C.

**Conclusions**: The distribution of HIV‐1 subtypes in Tel Aviv reflects dynamic transmission patterns. Transmission clusters are prominent among MSM and IDU networks. Identification of such clusters has important implications for targeted epidemiological interventions. The recent expansion of subtype A3 among MSM highlights ongoing epidemiological shifts. Continuous monitoring of subtype distribution and TDR is also important for therapeutic decision‐making, as certain subtypes—such as A6—may influence the effectiveness long‐acting cabotegravir/rilpivirine regimens.

**Reference**

1. Bennett DE, Camacho RJ, Otelea D, Kuritzkes DR, Fleury H, Kiuchi M, et al. Drug resistance mutations for surveillance of transmitted HIV‐1 drug‐resistance: 2009 update. PLoS One. 2009;4(3):e4724.

## Cost and Cost‐Effectiveness

### P342 Return on Investment of Eliminating Insurance Barriers to PrEP in High‐Incidence Populations

Emir Gursel^1^, JeanPierre Coaquira Castro^2^, Turgay Ayer^1,3^, Alice Hsiao^2^, Woodie Zachry ^1^, Mert Edali^4^, Patrick Sullivan^5^

^1^Value Analytics Labs, Boston, MA, US; ^2^Gilead Sciences, Inc., Foster City, CA, US; ^3^Georgia Institute of Technology, Atlanta, GA, US; ^4^Yildiz Technical University, Istanbul, Türkiye; ^5^Emory University, Emory University, GA, US

**Background**: HIV‐1 continues to disproportionately affect US populations, with higher incidence among Black and Hispanic men who have sex with men, transgender women and cisgender women with pre‐exposure prophylaxis (PrEP) indications. Insurance barriers, including prior authorization and coverage restrictions, limit PrEP access and contribute to PrEP abandonment in underserved populations. Recent findings demonstrate that individuals unable to initiate PrEP due to insurance rejection face approximately twice the risk of HIV‐1 acquisition. Limiting PrEP initiation may increase downstream HIV treatment costs and productivity losses. We evaluated the clinical and economic impact of removing insurance barriers to oral and long‐acting injectable (bimonthly and twice‐yearly) PrEP.

**Methods**: A lifetime‐horizon HIV‐1 microsimulation model projected the financial impact of removing insurance barriers among high‐incidence US populations. Current insurance barriers (comparator) were compared with complete removal of these barriers (intervention). Return on investment (ROI) was defined as lifetime HIV‐related treatment costs averted per additional dollar spent on PrEP; ROI > 1 indicated net savings. ROI was evaluated from payer, healthcare ecosystem and societal perspectives. Model inputs included HIV‐1 incidence and progression, PrEP utilization patterns, treatment and PrEP costs and the separate probabilities of PrEP initiation following insurance approval and initial rejection, based on published and real‐world dispensation data.

**Results**: Removing insurance barriers was projected to increase PrEP uptake, prevent new HIV‐1 cases and generate net savings through reduced downstream HIV‐related costs, despite higher PrEP spending. In the aggregate population, the intervention averted an estimated 28.8 HIV‐1 cases per 10,000 individuals over 5 years. ROI of removing insurance barriers was estimated to be 2.9, 2.9 and 3.3 from payer, healthcare ecosystem and societal perspectives, respectively. Payer, healthcare ecosystem and societal perspectives yielded an ROI range of 2.2–4.0 across all populations (Table 1).

**Conclusions**: Removing PrEP insurance barriers for high‐incidence populations was projected to avert new HIV‐1 cases and reduce downstream treatment costs, resulting in net economic savings. These findings support policies that improve access to oral and long‐acting injectable PrEP options. Expanding PrEP access to populations disproportionately affected by HIV may support public health goals while bringing fiscal and productivity benefits for payers, the healthcare ecosystem and society.

**TABLE 1:**
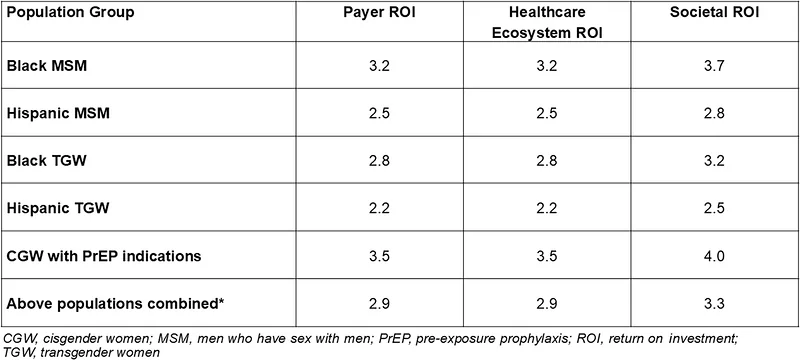
P342 | ROI outcomes and averted HIV‐1 cases after elimination of PrEP insurance barriers from the payer's, healthcare ecosystem and societal perspectives, for Black and Hispanic MSM and TGW, CGW with PrEP indications and weighted across all of these populations. ^*^Combined estimates were calculated by weighting the results for the above population groups by their population sizes to generate aggregate population ROI outcomes.

### P343 Projected Health Benefits and Economic Value of Combined Opt‐Out Testing for HIV/HBV/HCV in Urban Emergency Departments in Germany, Greece and Poland

Magdalena Rosińska^1,2^, Robert Flisiak^3^, Andrzej Fal^4^, Justyna Kowalska^5,6^, Hartmut Stocker^7^, Mina Psichogiou^8^, Charalampos Gogos^9^, George Papatheodoridis^10^, Nikolaos Mangafas^11^, Laurel Bates ^12^, Xinlu Pan^12^, Andrea Polokaova^12^, Erik Robinson^12^, Hairuo He^12^, James Jarrett^13^, Murad Ruf^14^, Jürgen Rockstroh^15^

^1^Department of Infectious Disease Epidemiology and Surveillance, National Institute of Public Health PZH‐National Research Institute, Warsaw, Poland; ^2^Digital Medicine Centre, Maria Skłodowska‐Curie National Research Institute of Oncology, Warsaw, Poland, Warsaw, Poland; ^3^Department of Infectious Diseases and Hepatology, Medical University of Białystok, Białystok, Poland; ^4^Collegium Medicum, Warsaw Faculty of Medicine, Cardinal Stefan Wyszynski University, Warsaw, Poland; ^5^Infectious Diseases Observational Research Unit, Medical University of Warsaw, Warsaw, Poland; ^6^Hospital for Infectious Diseases, Hospital for Infectious Diseases, Warsaw, Poland; ^7^Department of Infectious Diseases, St Joseph Krankenhaus, Berlin, Germany; ^8^First Department of Medicine, Laiko General Hospital, Medical School, National and Kapodistrian University of Athens, Athens, Greece; ^9^Department of Medicine and Infectious Diseases Unit, University of Patras School of Medicine, Patras, Greece; ^10^First Academic Department of Gastroenterology and Liver Transplantation Unit, Laiko General Hospital, Medical School, National and Kapodistrian University of Athens, Athens, Greece; ^11^Third Department of Internal Medicine and Infectious Diseases Unit, Red Cross General Hospital, Athens, Greece; ^12^Aquarius Population Health, London, UK; ^13^Global Health Economics and Outcomes Research, Gilead Sciences, London, UK; ^14^Medical Department Public Health, Gilead Sciences Europe, London, UK; ^15^Department of Internal Medicine I, University Hospital Bonn, Bonn, Germany

**Background**: Germany, Greece and Poland have not yet met the first 95% target set by UNAIDS under Sustainable Development Goal (SDG) 3.3 [1]. SDG 3.3 also calls for action to combat viral hepatitis. Emergency department (ED) blood‐borne virus (BBV) opt‐out testing (OOT) has been shown to detect previously unidentified infections, many of which are among people who do not access traditional testing services [2]. Currently, there is no systematic ED BBV testing in these countries. Testing consent guidance varies by country and infection, with generally stricter rules for HIV than viral hepatitis [3]. However, these rules do not prohibit routine testing; all three countries employ universal HIV screening to prevent transmission in antenatal care [4]. Based on published clinical models, we evaluated the potential health and economic value of published best‐practice ED BBV OOT in high‐prevalence EDs across these three countries.

**Methods**: We adapted a hybrid cost‐effectiveness model to compare ED BBV OOT with current ED testing practice. A decision tree simulated testing, diagnosis and linkage/re‐linkage to care among eligible ED attendees; disease‐specific Markov models projected lifetime costs and outcomes for HIV, HBV and HCV diagnoses. Key outcomes included new diagnoses, linkage/re‐linkage to care, quality‐adjusted life years (QALYs), healthcare costs and incremental cost‐effectiveness ratios (ICERs) for combined BBV testing. We used country‐specific literature inputs where available, supplemented by expert opinion.

**Results**: ED BBV OOT was projected to increase new BBV diagnoses, linkage/re‐linkage to care, QALYs and healthcare costs within each country over a lifetime horizon (Table 1). Cost‐effectiveness varied by virus and country. Combined BBV testing was cost‐effective in all three countries (Figure 1 and Table 1) and was more cost‐effective than HIV‐only testing.

**Conclusions**: Best‐practice ED BBV OOT in high‐prevalence areas in Germany, Greece and Poland would complement case‐finding strategies for patients not reached by current testing. Integrated testing for BBVs allowed for greater cost‐effectiveness than HIV‐only testing. Our results likely underestimate impacts as we did not model transmissions averted. Together with ED BBV OOT implementation evidence from England [2], these findings support further informed national dialogue and local pilot evaluations for population‐level case‐finding strategies.

**References**

1. Continuum of HIV care: Monitoring the implementation of the Dublin Declaration on partnership to fight HIV/AIDS in Europe and Central Asia: 2025 progress report (2024 data). Available at: https://www.ecdc.europa.eu/en/publications‐data/continuum‐hiv‐care‐monitoring‐implementation‐dublin‐declaration‐partnership‐1. Accessed 03/06/2026.

2. UK Health Security Agency. Public health evaluation of BBV opt‐out testing in EDs in England, 33‐month final report 2025. Available at: https://www.gov.uk/government/publications/bloodborne‐viruses‐opt‐out‐testing‐in‐emergency‐departments/public‐health‐evaluation‐of‐bbv‐opt‐out‐testing‐in‐eds‐in‐england‐33‐month‐final‐report‐2025. Accessed 12/03/2026.

3. Stengaard AR, Sullivan AK, Simões D, Diderichsen J, Raben D, Rockstroh JK, et al. An overview of consent requirements for HIV and viral hepatitis B and C testing in Europe. HIV Med. 2026. doi:10.1111/hiv.70248.

4. European Centre for Disease Prevention and Control. Antenatal screening for HIV, hepatitis B, syphilis and rubella susceptibility in the EU/EEA. Stockholm: ECDC; 2016. Available at: https://www.ecdc.europa.eu/sites/default/files/media/en/publications/Publications/antenatal‐screening‐HIV‐hepatitis‐B‐syphilis‐rubella‐EU.pdf. Accessed 24/06/2026.

5. Gandjour A. Comment on: “10 Years of AMNOG: What is the Willingness‐to‐Pay for Pharmaceuticals in Germany?” Appl Health Econ Health Policy. 2024;22(1):125–6. doi:10.1007/s40258‐023‐00852‐2.

6. Athanasakis K, Agorastos G, Kyriopoulos I. Estimating a cost‐effectiveness threshold for healthcare decision‐making in the Greek NHS. Health Policy Technol. 2024;13(3):100882. doi:10.1016/j.hlpt.2024.100882.

7. Tesar T, Obsitnik B, Kaló Z, Kristensen FB. How changes in reimbursement practices influence the financial sustainability of medicine policy: Lessons learned from Slovakia. Front Pharmacol. 2019;10:664. doi:10.3389/fphar.2019.00664.

**FIGURE 1:**
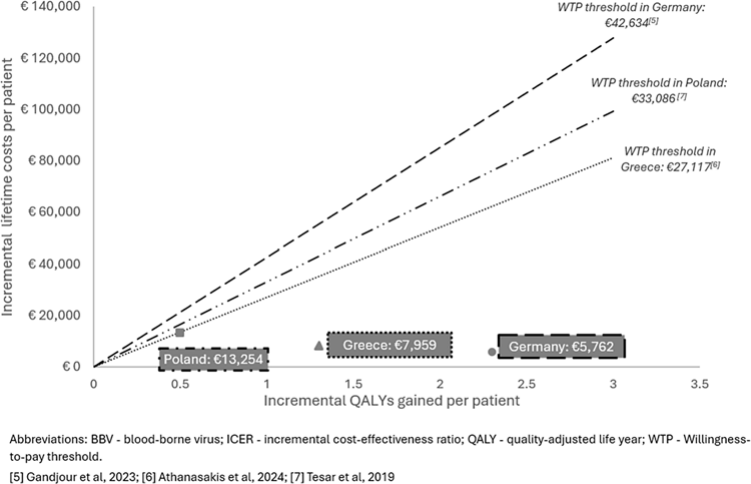
P343 | ICER (costs‐per‐QALY) per patient for combined BBV testing across lifetime by country. ^*^Poland costs and ICERs were converted from PLN to EUR using the exchange rate €1 = PLN 4.23.

**TABLE 1:**
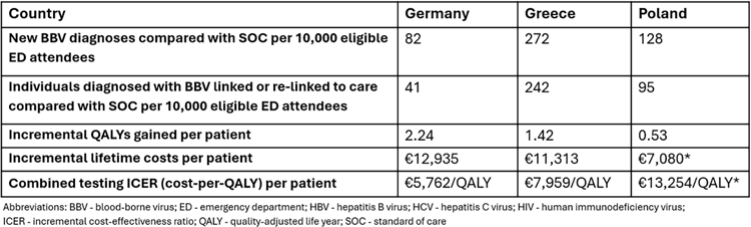
P343 | New BBV diagnoses, linkage/re‐linkage to care, costs, QALYs and ICERs by country. ^*^Poland costs and ICERs were converted from PLN to EUR using the exchange rate €1 = PLN 4.23.

### P344 Economic Impact of Extended Monitoring Intervals in People With HIV Receiving Bictegravir/Emtricitabine/Tenofovir Alafenamide: An Italian Real‐World Analysis (EXTEND‐BIC Study)

Diego Ripamonti ^1^, Roberta Gagliardini^2^, Valentina Perrone^3^, Stefania Mazzoni^3^, Luca Degli Esposti^3^, Laura Albini^4^, Gabriele Forcina^4^, Giovanni Di Perri^5^

^1^Ospedale Papa Giovanni XXIII, Bergamo, Italy; ^2^INMI Lazzaro Spallanzani, Rome, Italy; ^3^CliCon S.r.l. Società Benefit Health, Economics & Outcomes Research, Bologna, Italy; ^4^Gilead Sciences Italy, Milan, Italy; ^5^Università degli Studi di Torino, Turin, Italy

**Background**: Bictegravir/emtricitabine/tenofovir alafenamide (B‐F‐TAF) is recommended for people with HIV (PWH), with high forgiveness and genetic‐barrier. Optimized monitoring may affect healthcare resource utilization (HCRU) and costs. This analysis assessed the economic impact of extended monitoring intervals among B‐F‐TAF‐treated PWH in clinical practice.

**Methods**: EXTEND‐BIC is a retrospective analysis based on Italian administrative/laboratory databases covering ∼6.7 million individuals. Adult PWH initiating B‐F‐TAF (July 2019–December 2025), with ≥12 months of follow‐up and ≥2 monitoring‐tests after treatment initiation, were included. The first monitoring‐test after B/F/TAF initiation was the index date. PWH with co‐infections (HCV, HBV, CMV, mpox), cancers, or pregnancy in the previous 12 months were excluded. Monitoring interval, defined as the time between the first and second tests, was classified as standard (<9 months) or extended (9–12 months); PWH with intervals > 12 months were excluded. Baseline characteristics were balanced using propensity score matching (PSM). Among PWH alive during follow‐up, HCRU and direct costs were evaluated. A generalized linear model (GLM) adjusted for age, sex, Charlson Comorbidity Index, previous antiretroviral therapies and index year compared costs between groups.

**Results**: 3985 B‐F‐TAF‐treated PWH were identified; 1683 met inclusion criteria. Of these, 1445 (85.9%) received standard and 119 (7.1%) extended monitoring. After PSM, 117 balanced PWH per group were included, with all standardized mean differences < 0.2 (Table 1). Extended monitoring was associated with lower outpatient services use (10.2 vs. 12.3; *p* = 0.002), including fewer monitoring tests (2.1 vs. 2.9; *p* < 0.001) and other diagnostic‐tests (4.6 vs. 5.8; *p* = 0.002) versus standard monitoring. Mean direct healthcare costs, excluding drug expenditures, were 27% lower with extended versus standard monitoring (€1544.1 vs. €2104.3 per patient‐year; *p* < 0.001), without impact on B‐F‐TAF switch or discontinuation (80% were persistent). In GLM analyses, extended monitoring was associated with a mean cost reduction of €563.7 (95% CI: −1048.6; −78.8; *p* = 0.023) (Figure 1).

**Conclusions**: In this observational analysis, extended monitoring intervals among B‐F‐TAF‐treated PWH was associated with reduced outpatient service use and lower direct costs. These findings support extended monitoring in appropriately selected B‐F‐TAF‐treated PWH. The ongoing nationwide estimation of B‐F‐TAF recipients who might be monitored at decreased frequency will provide a preliminary assessment of potential overall cost reduction benefits.

**FIGURE 1:**
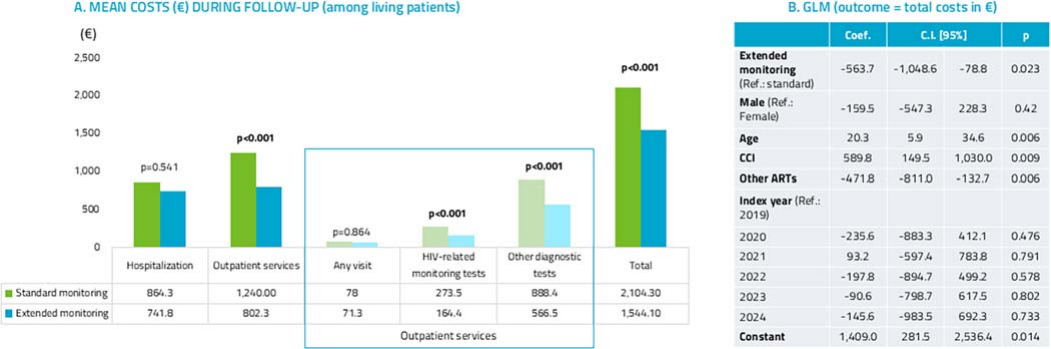
P344 | (A) Mean direct healthcare costs during follow‐up; (B) GLM analysis of total direct healthcare costs.

**TABLE 1:**
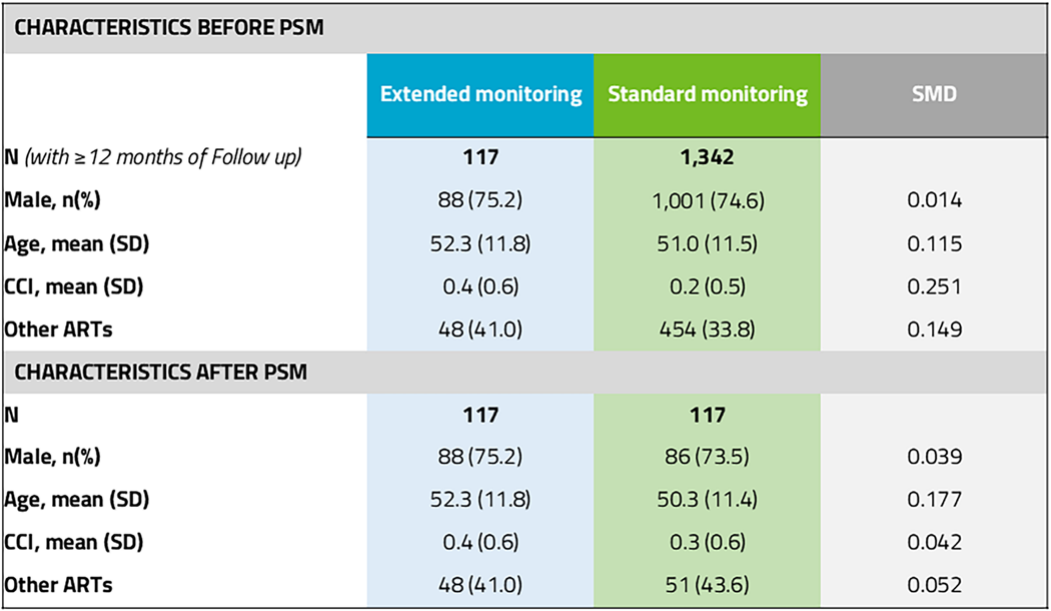
P344 | Baseline characteristics of the study cohorts (extended and standard monitoring) before and after PSM.

### P345 An Annual HIV Review Pathway: Delivering Safe, Sustainable and High‐Value Care

Lily Edwards, Rachel Kirby, Callum Neill, Justine Mellor, Vincent Lee

The Northern Contraception, Sexual Health & HIV Service, Manchester University NHS Foundation Trust, Manchester, UK

**Background**: Most people living with HIV taking antiretroviral therapy (ART) achieve sustained virological suppression and require less intensive monitoring. An annual review pathway was developed to reduce the number of appointments, travel to clinic and increase patient choice and support more sustainable healthcare delivery.

Yearly face to face appointments were offered to patients who met predefined with an interim virtual review at 6 months incorporating a patient health questionnaire.

**Methods**: Retrospective case note review to identify patients meeting inclusion criteria for transition to the new pathway between March 2023 and March 2026. Demographics, clinical outcomes and healthcare resource utilization were evaluated.

**Results**: 381 patients were identified. The majority were male (80.6%, *n* = 307). The median age was 47 years (range 23–78) and median duration of HIV diagnosis was 16 years (range 3–42). After 12 months, 99% (*n* = 378) remained undetectable viral load. 2 had a break in treatment and resuppressed at follow up, 1 had viral blip and remained undetectable. 10% (*n* = 38) had their ART changed. One patient died from a cerebrovascular accident. No new resistance was detected. Our typical face to face follow up appointment is 30 min compared to 10 min for virtual review. This means we have saved 127 h of clinician time in this new pathway. Elimination of laboratory testing at virtual results generated estimated cost savings of £16,626.84.

Potential travel‐related carbon savings were calculated using two scenarios. Assuming all patients travelled by car, an estimated 2637 kg carbon dioxide equivalent (CO_2_e) was saved. Using a modal transport distribution based on national averages, estimated savings were 2374 kg CO_2_e.

**Conclusions**: Transition to less frequent in person contact has potential significant cost and environmental benefits in addition to improving clinician workload. Less frequent in‐person contact offered significant benefits in clinician workload, laboratory costs and carbon emissions, but also provided appropriate patients with greater choice and flexibility in the management of their HIV care.

### P346 Expanding HIV Pre‐Exposure Prophylaxis Could Reduce New HIV Infections by 69% and Generate Long‐Term Cost Savings in Italy

Chiara Bini^1^, Valentina Mazzotta ^2^, Silvia Nozza^3,4^, Martina Paoletti^1^, Virginia Scansetti^1^, Paolo Sciattella^1^

^1^Economic Evaluation and HTA (EEHTA) – CEIS, Faculty of Economics, University of Rome “Tor Vergata”, Rome, Italy; ^2^Clinical Infectious Diseases Department, National Institute for Infectious Diseases Lazzaro Spallanzani IRCCS, Rome, Italy; ^3^Infectious Diseases Unit, IRCCS San Raffaele Scientific Institute, Milan, Italy; ^4^Vita‐Salute San Raffaele University, Milan, Italy

**Background**: HIV infection remains a significant public health issue in Italy, generating a substantial clinical and economic burden despite high treatment coverage. Population ageing and increasing comorbidities further amplify this impact. Pre‐exposure prophylaxis (PrEP) is an effective primary prevention strategy. This study estimates the economic burden of HIV in Italy and assesses the clinical and economic impact of PrEP in a subgroup of population at high risk of HIV transmission (men who have sex with men [MSM]).

**Methods**: A prevalence‐based cost of illness (COI) model was developed, including both direct healthcare costs and indirect costs related to productivity losses due to the disease, to estimate the overall economic burden of HIV in Italy. In addition, a cost‐consequence analysis (CCA) using a Markov model simulated a closed cohort of 1000 MSM with and without PrEP over a 20‐year horizon. In the scenario including PrEP, the hypothetical cohort was assumed to reflect current patterns of use in Italian clinical practice, with 85% of individuals receiving oral PrEP with tenofovir disoproxil fumarate (TDF) and emtricitabine (FTC) and the remaining 15% receiving long‐acting injectable PrEP with lenacapavir. Epidemiological, clinical and cost inputs were derived from literature, institutional data sources and expert opinion. Efficacy data for oral and injectable PrEP were sourced from the ANRS PREVENIR cohort and the PURPOSE‐2 trial, respectively.

**Results**: The annual cost of HIV management in Italy was estimated at approximately €994 million, corresponding to €6626 per person, with pharmaceutical costs representing the largest component (65%), followed by indirect costs (19%). In a simulated cohort of 1000 MSM, PrEP reduced cumulative HIV infections over 20 years by about 69% compared with no PrEP (Figure 1). This decrease translated into cumulative savings of approximately €32.8 million in direct costs related to newly diagnosed HIV cases.

**Conclusions**: HIV infection imposes a considerable financial burden on the healthcare system. Expanding the use of PrEP among high‐risk individuals could substantially reduce the number of new infections, thereby alleviating future costs associated with long‐term clinical management and antiretroviral treatment and improving the overall sustainability of healthcare resources.

**FIGURE 1:**
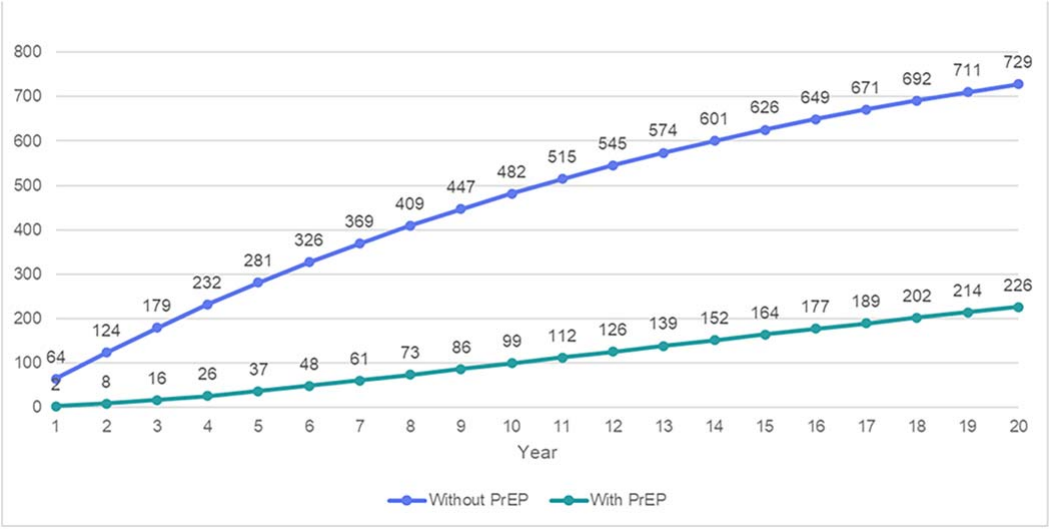
P346 | Cumulative HIV infection over 20 years time horizon.

### P347 Closing the Gap to Zero HIV Transmission: Modelling the Impact of Long‐Acting Injectables (LAI) in the UK

Melanie Schroeder ^1^, Johan Klint^2^, Jonathan Tosh^1^, Rob Blissett^2^, Sarah‐Jane Anderson^3^, Gavin Jones^2^, Jeremy Fraysse^4^, Julie Priest^4^

^1^ViiV Healthcare, London, UK; ^2^Maple Health Group, LLC, New York, NY, US; ^3^GSK, London, UK; ^4^ViiV Healthcare, Durham, NC, US

**Background**: England has achieved the UNAIDS 95‐95‐95 targets and has committed to ending HIV transmission (<532 new diagnoses annually) by 2030. LAI pre‐exposure prophylaxis (PrEP) and antiretroviral treatment (ART) offer improved adherence, persistence and effectiveness compared with daily oral (DO) ART. This study estimated the epidemiological and economic impact of scaling up combined LAI PrEP and LAI treatment in the UK.

**Methods**: A published compartmental dynamic HIV transmission model capturing disease progression was calibrated to UK Health Security Agency surveillance data. The model stratified the population (≥15 years) by acquisition risk and included people with HIV across the full care continuum with model outputs aligned to observed epidemiological endpoints including prevalence and new diagnoses. Two scenarios were compared: (1) baseline continuation of current care, with oral PrEP uptake increasing 1% annually; and (2) 20% scale‐up with LAI PrEP and switching 50% of low‐adherence oral PrEP users to LAI PrEP, as well as switching 100% of viraemic individuals to LAI ART by 2028 and switching 20% of suppressed individuals to LAI ART within 1 year. Outcomes included HIV acquisitions, diagnoses and return on investment (ROI).

**Results**: In scenario 1, HIV incidence remains above elimination targets, with lifetime costs of new acquisitions projected to exceed £27 billion. Scenario 2 demonstrated substantial epidemiological impact: 25,235 cumulative HIV acquisitions were averted over the 2025–2040 analysis period versus baseline. Reductions in new diagnoses and stabilization of prevalence were observed, and elimination targets were met in year 2037 (Figure 1). From an economic perspective, the intervention generated a positive ROI, increasing over time. Cumulative averted lifetime costs exceeded intervention costs from 2030 onwards (Table 1).

**Conclusions**: Current prevention and treatment strategies are insufficient to meet zero‐transmission targets, with HIV acquisitions projected to remain above 2000 annually under current practice. Combined scale‐up of LAI PrEP and LAI ART substantially reduced HIV transmission, with elimination targets projected to be achieved by 2037. These targets could be reached sooner with concurrent expansion in HIV testing, increasing diagnosis rates and accelerating entry into the care continuum. Overall, integrated LAI strategies represent a critical component of achieving HIV elimination targets while reducing long‐term system burden.

P353: Figure 1. Projected number of new HIV diagnoses annually with scale‐up of LAI PrEP and LAI ART.

Due to less frequent dosing and directly observed therapy, viraemic individuals switching from DO ART to LAI ART were modelled to experience virologic suppression upon switch.

**TABLE 1:**
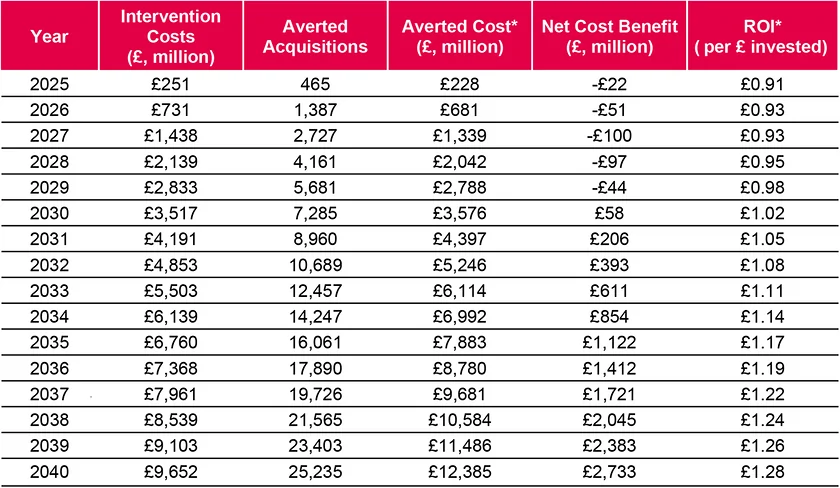
P347 | Return on investment for scale‐up of LAI PrEP and LAI ART. ^*^Estimated lifetime HIV management cost £659,620. ROI was calculated as lifetime cost savings for averted HIV acquisitions relative to additional prevention costs incurred with LAI PrEP scale‐up and incremental treatment costs with LAI ART.

### P348 Historical Pricing Dynamics of Generic Antiretroviral Agents: Implications for the Future Introduction of TLD in High‐Income Countries

Piter Oosterhof ^1^, Kees Brinkman^2^, David Burger^3^

^1^Department of Clinical Pharmacy, OLVG, Amsterdam, Netherlands; ^2^Department of Internal Medicine, OLVG, Amsterdam, Netherlands; ^3^Department of Pharmacy, Pharmacology and Toxicology, Research Institute for Medical Innovation, Radboudumc, Nijmegen, Netherlands

**Background**: Tenofovir disoproxil/lamivudine/dolutegravir (TLD) is the World Health Organization (WHO)‐recommended first‐line regimen and the most widely used antiretroviral combination globally. With the anticipated introduction of generic dolutegravir in high‐income countries, TLD is expected to become an increasingly relevant treatment option. However, the financial impact of generic TLD is likely to depend on more than the availability of generic dolutegravir alone. We evaluated historical pricing dynamics of generic antiretroviral agents to identify factors likely to influence the future economic impact of generic TLD.

**Methods**: A retrospective market analysis was performed using Dutch antiretroviral pricing data between 2017 and 2023. Monthly prices of branded and generic antiretroviral agents, timing of generic market entry and the number of generic suppliers were evaluated to characterize historical pricing dynamics following generic introduction.

**Results**: Between 2017 and 2023, substantial variation was observed in the timing of generic market entry, price reductions and market competition across antiretroviral agents (Table 1). The magnitude and rate of price reductions differed considerably between drug classes despite generic availability. Likewise, the number of generic suppliers varied substantially between compounds, reflecting heterogeneous market competition. These findings demonstrate that generic availability alone does not determine the ultimate price evolution of antiretroviral agents.

**Conclusions**: Introduction will depend on country‐specific market dynamics rather than the availability of generic dolutegravir alone. National pricing policies, procurement strategies, reimbursement systems and market competition are likely to determine the magnitude of potential savings. Anticipating these factors may help policymakers and guideline committees maximize the clinical and economic value of generic TLD following its introduction into high‐income countries.

**TABLE 1:**
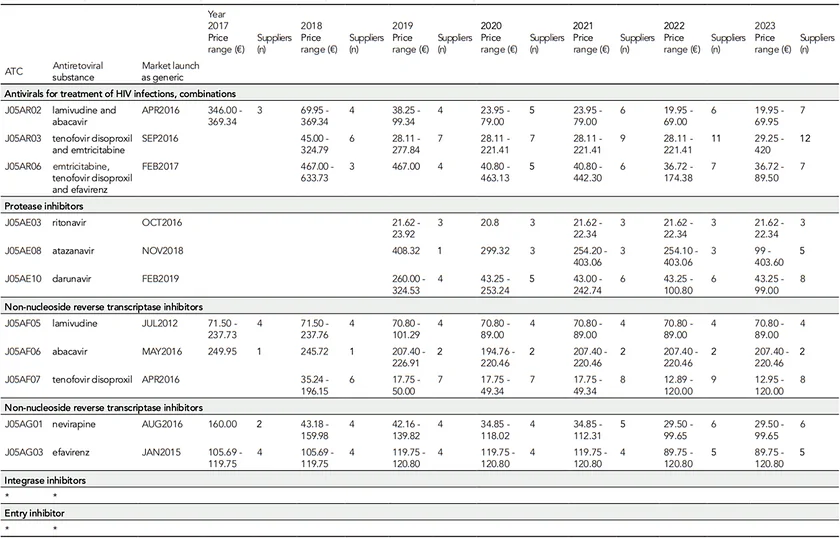
P348 | Historical monthly price ranges and number of generic suppliers for antiretroviral agents in the Netherlands, 2017–2023.

### P349 Translating Evidence Into Practice: A Comparative Analysis of International HIV Treatment Guidelines on Medication Cost‐Driven ART Optimization

Piter Oosterhof ^1^, Kees Brinkman^2^, David Burger^3^

^1^Department of Clinical Pharmacy, OLVG, Amsterdam, Netherlands; ^2^Department of Internal Medicine, OLVG, Amsterdam, Netherlands; ^3^Department of Pharmacy, Pharmacology and Toxicology, Research Institute for Medical Innovation, Radboudumc, Nijmegen, Netherlands

**Background**: Antiretroviral therapy (ART) accounts for more than 70% of direct HIV healthcare costs in many high‐income countries, with most costs attributable to treatment‐experienced people with HIV who receive lifelong therapy. Several studies have shown that ART optimization strategies, including generic substitution, regimen simplification and structured treatment switches, reduce medication costs without compromising virological suppression, treatment effectiveness or patient satisfaction. Whether this evidence has been translated into international HIV treatment guidelines remains unknown. Therefore, we evaluated the extent to which contemporary guidelines incorporate cost‐conscious treatment optimization into recommendations for treatment‐experienced people with HIV.

**Methods**: A comparative policy analysis of five contemporary international HIV treatment guidelines (EACS, DHHS, IAS–USA, BHIVA and NVHB) was conducted. Recommendations regarding ART optimization in virologically suppressed treatment‐experienced people with HIV were assessed for (i) recognition of medication costs as an indication for switching and (ii) provision of practical recommendations, prioritization strategies, or structured treatment algorithms to facilitate cost‐conscious ART optimization.

**Results**: All five guidelines recommend ART optimization to improve tolerability, reduce toxicity, simplify treatment and manage drug–drug interactions while maintaining virological suppression. Four of the five guidelines (EACS, DHHS, IAS–USA and BHIVA) acknowledged medication costs as a potential reason for switching ART. However, none of these studies provided practical recommendations, prioritization strategies, or structured algorithms to identify patients eligible for cost‐conscious optimization or to support clinicians in implementing these strategies.

**Conclusions**: Although international HIV treatment guidelines recognize medication costs as a potential reason for ART switching, none translate the available evidence into practical guidance for clinical decision‐making. As treatment‐experienced people with HIV account for the majority of ART expenditure, this represents a missed opportunity to improve the sustainability of HIV care without compromising treatment quality. Incorporating evidence‐based treatment optimization strategies into future guideline updates could help clinicians systematically identify opportunities for safe, effective and lower‐cost ART optimization.

### P350 Abstract Withdrawn

### P351 Cost‐Effectiveness of a Low‐Intensity Primary Care Educational Intervention to Reduce Late HIV Diagnosis in Madrid, Spain

Alejandro García‐Ruiz de Morales^1,2^, Javier Martinez Sanz^1,2^, Rosas Marta^1,2^, Maria Jesus Vivancos^1,2^, Beatriz Romero‐Hernández^3^, Beatriz Mateos^4^, Ana Moreno Zamora^1,2^, Santiago Moreno^1,2^, Maria Jesus Perez Elias ^1,2^, on behalf of Atenea Study Group

^1^Infectious Diseases, Hospital Ramon y Cajal, Madrid, Spain; ^2^CIBERINFEC, Madrid, Spain; ^3^Microbiology, Hospital Ramon y Cajal, Madrid, Spain; ^4^Gastroenterology Hospital Ramon y Cajal, Madrid, Spain

**Background**: Late HIV diagnosis remains common in Europe and increases morbidity, mortality and healthcare costs. In 2017, a 2‐h in‐person educational intervention was delivered to primary care physicians across 20 centres in Madrid, Spain, to improve HIV testing practice [1]. We evaluated its cost‐effectiveness from the health system perspective.

**Methods**: We performed a retrospective before–after cost‐effectiveness analysis comparing 2012–2016 with 2017–2022. HIV testing and diagnosis data were obtained from hospital microbiology records and stage at diagnosis from data of the CoRIS cohort. Late diagnosis was defined as CD4 < 350 cells/mm^3^ or an AIDS‐defining event at diagnosis; advanced HIV disease was defined as CD4 < 200 cells/mm^3^ or an AIDS‐defining event. Advanced presentations were treated as a subset of late diagnoses to avoid double counting. Costs were expressed in 2023 euros and included programme delivery and incremental HIV testing. Healthcare savings reflected avoided 5‐year HIV care costs from preventing late and advanced diagnoses, using estimates from published economic evaluations weighted towards European data. Outcomes included cost savings per diagnosis prevented, quality‐adjusted life years (QALYs) gained, incremental cost‐effectiveness ratio (ICER) and benefit‐to‐cost index (BCI). Uncertainty was assessed using one‐way sensitivity analysis and a 1000‐draw probabilistic sensitivity analysis (PSA).

**Results**: Mean annual HIV testing increased from 9187 to 12,191 tests. The proportion of late diagnoses decreased from 58.9% to 48.7%. Applying pre‐intervention rates to post‐intervention diagnoses suggested that 20 late diagnoses were prevented, including 12 advanced presentations. Programme and incremental testing costs totalled €150,197. Estimated healthcare savings were €482,280, generating net savings of €332,083 and a BCI of 3.21. The intervention gained 62.6 QALYs and was dominant, with an ICER of –€5305/QALY (Figure 1). In PSA, all simulations were cost‐saving.

**Conclusions**: A brief, low‐cost primary‐care educational intervention was associated with reduced late and advanced HIV diagnosis and was cost‐saving. These findings support integrating similar training into routine primary‐care HIV testing strategies.

**Reference**

1. García‐Ruiz de Morales AG, Vivancos MJ, Romero‐Hernández B, Rial‐Crestelo D, Arnaiz de Las Revillas F, Sanchez‐Villegas J, Montero Alonso M, López Sánchez MV, Alemán Valls MR, Moreno S, Martínez‐Sanz J, Pérez‐Elías MJ; CoRIS. Low‐effort HIV training in Spanish primary care: Long‐term impact on testing and diagnosis. Enferm Infecc Microbiol Clin (Engl Ed). 2025;43(10):645–651. doi: 10.1016/j.eimce.2025.06.015. PMID: 41338820.

**FIGURE 1:**
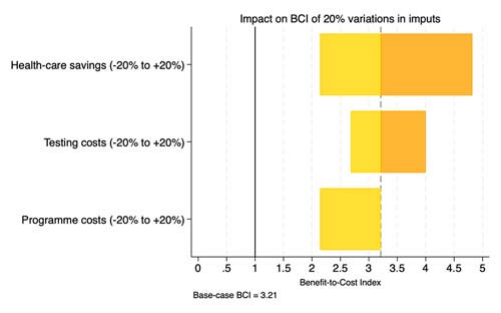
P351 | Tornado plot showing the Benefit‐to‐Cost index in scenarios of ± 20% variations of healthcare savings, testing costs and programme costs.

### P352 Abstract Withdrawn

## Models of Care: Evaluation of ARV Delivery and Coverage

### P353 European Healthcare Providers’ PrEP Practices and Barriers to Equitable Access for Migrant Populations: Insights From an EACS‐Network Survey

Haoyi Wang ^1,2^, Anne Flaherty‐Gupta^3^, Hala Majdoub^3^, Juan Soto^1^, Gonçalo Lobo^4^, Joëlle Verluyten^4^, Kai Jonas^1^, Hanne Zimmermann^1^, Denis Onyango^3^

^1^Department of Work and Social Psychology, Maastricht University, Maastricht, Netherlands; ^2^Viroscience Department, Rotterdam, Netherlands; ^3^Africa Advocacy Foundation, London, UK; ^4^European AIDS Clinical Society, Brussels, Belgium

**Background**: Migrants, including undocumented individuals and asylum seekers, face persistent structural barriers to accessing PrEP in Europe. Healthcare providers (HCPs) are central to the overall PrEP implementation, yet evidence on their prescribing practices and perceived barriers to PrEP provision among migrant populations remains limited. We examined PrEP prescribing patterns among European HCPs and identified barriers and facilitators to equitable PrEP access for migrants.

**Methods**: We conducted a cross‐sectional survey of HCPs involved in HIV prevention and care across Europe using purposive, multi‐channel recruitment. Recruitment began at the HIV Glasgow conference 2024, followed by dissemination through the EACS membership newsletter (February 2025). Targeted dissemination was conducted through clinical networks in Poland, Portugal and Spain. Survey items assessed HIV care engagement, PrEP prescribing practices, reimbursement experiences and perceived barriers and facilitators to PrEP provision for migrant populations.

**Results**: Among 107 participating HCPs, engagement in HIV prevention was high: 98.1% provided HIV care, 95.3% prevention services and 95.3% discussed PrEP with individuals at risk. Among those with prescribing privileges (*n* = 83), 90.4% had prescribed PrEP. Prescribing rates declined by legal status: 81.9% had prescribed to documented migrants versus 69.9% for refugees, asylum seekers and undocumented migrants. Reimbursement challenges mirrored this pattern, reported by 65.1% for documented migrants versus 47.0% for undocumented migrants and refugees. Medical doctors reported higher prescribing and reimbursement rates than other prescribing HCPs (Figure 1a). All HCPs identified migrants being perceived as “hard‐to‐reach” as a barrier. Other barriers included lack of proactive PrEP requests from migrants (20.5%), affordability concerns (10.8%) and anticipated follow‐up challenges (10.8%). Universally endorsed facilitators included streamlined care pathways for uninsured and low‐income migrants and institutional recognition for providers serving diverse populations. Partnerships with community‐based organizations, multilingual materials, translation services and enhanced provider education were also frequently endorsed (Figure 1b).

**Conclusions**: Despite high levels of HIV prevention engagement among this experienced clinical sample, substantial inequality in PrEP access persists across migrant groups, particularly for undocumented migrants. Structural barriers remain evident even among providers with established prevention expertise. Policies that streamline access pathways, strengthen reimbursement mechanisms and support community‐based delivery models are needed to advance equitable PrEP access for migrant communities.

**FIGURE 1:**
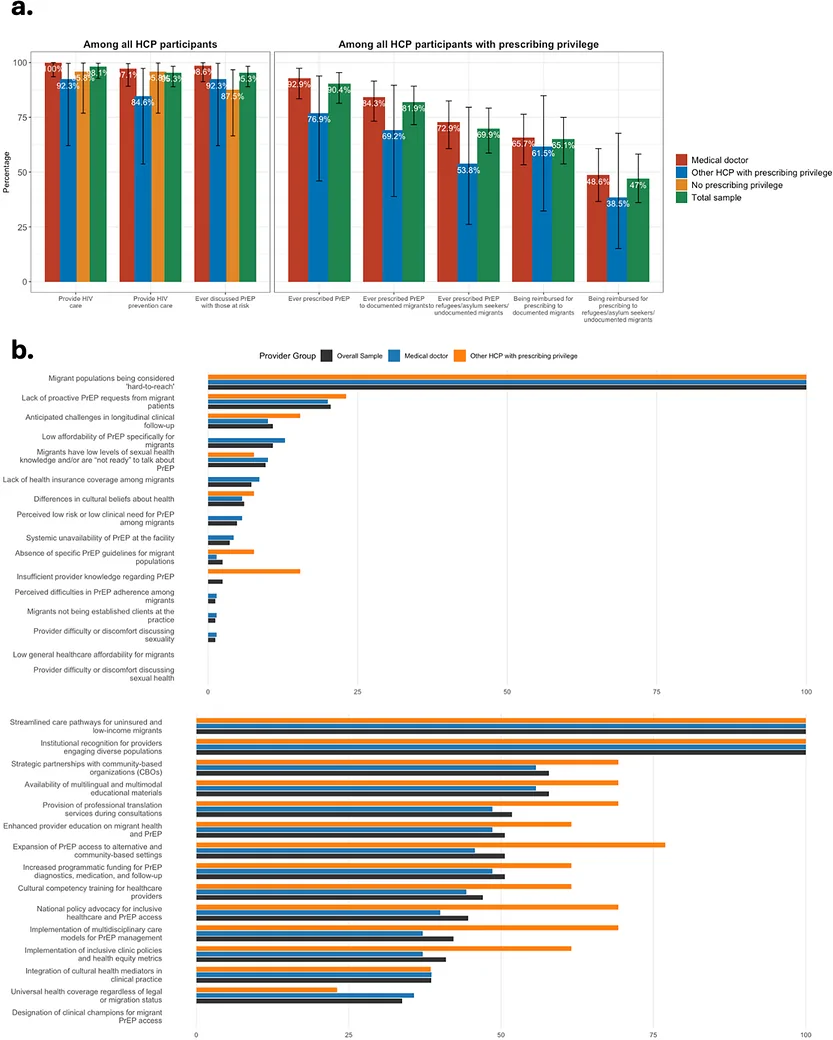
P353 | (a) Healthcare provider HIV care and PrEP prescribing patterns, among medical doctors, other healthcare providers with and without prescribing privilege and (b) perceived barriers and facilitators for healthcare providers with prescribing privilege on PrEP prescription and delivery to migrant populations.

### P354 Trajectories of Sustained Virological Suppression Over Time Are Not Determined by Socio‐Economic Factors in the Netherlands

Kyra Mendes de Leon ^1,2^, Ferdinand Wit^3,4^, Marc van der Valk^1,4^, Vita Jongen^1,4^

^1^Amsterdam UMC, University of Amsterdam, Infectious Diseases, Amsterdam, Netherlands; ^2^Amsterdam UMC, University of Amsterdam, Medical Psychology, Amsterdam, Netherlands; ^3^Department of Internal Medicine, Amsterdam UMC, University of Amsterdam, Amsterdam, Netherlands; ^4^HIV Monitoring Foundation, Amsterdam, Netherlands

**Background**: Advances in antiretroviral therapy (ART) have enabled sustained virological suppression in the majority of people with HIV. This has created opportunities for differentiated care, enabling care intensity to be tailored to individual needs and preferences, while reducing demands on healthcare. However, individuals with prolonged virological suppression who may benefit from less intensive, differentiated care remain poorly characterized. We aimed to identify individuals eligible for less intensive follow‐up within differentiated HIV care.

**Methods**: We combined data from the ATHENA cohort, an ongoing nationwide HIV cohort, with registry data from Statistics Netherlands. Group‐based trajectory modelling was used to identify trajectories of viral load (VL) suppression (HIV‐1 RNA < 200 copies/mL) over 10 years following ART initiation from 2002 onwards and their clinical and socio‐economic determinants (including income). Incidence rates of unsuppressed VL were calculated per 100 person‐years (PY), allowing for multiple incidence events. Incidence rate ratios (IRRs) comparing trajectories were estimated using Poisson regression.

**Results**: Among 11,290 people with HIV included in the analysis, three trajectories were identified: delayed virologic suppression (with ∼60% suppressed after one year) (29%, 2983/11,290; trajectory 1), rapid virologic suppression (60%; trajectory 2) and prolonged detectable viral load (detectable for ∼96 months) (11%; trajectory 3) (Figure 1). Individuals in the rapid suppression trajectory were more likely to have initiated ART from 2015 onwards (OR 3.87, 95% CI: 3.35–4.46) and less likely to have received an AIDS diagnosis (OR 0.66, 95% CI: 0.58–0.76), switched ART due to VF or adverse events (OR 0.69, 95% CI: 0.62–0.78), or had treatment interruptions (OR 0.13, 95% CI: 0.12–0.15). Compared with the delayed suppression trajectory, VF rates were lower in the rapid suppression (IRR 0.11, 95% CI: 0.10–0.11; IR 2.67/100 PY) and higher in the prolonged detectable trajectory (IRR 2.54, 95% CI: 2.47–2.60; IR 63.12/100 PY).

**Conclusions**: We identified three distinct trajectories, with nearly two‐thirds of the cohort achieving rapid virological suppression sustained over 10 years. Membership of this trajectory was primarily associated with clinical characteristics. While preferences of people with HIV remain central to differentiated care, these findings suggest that eligibility may be primarily informed by clinical rather than socioeconomic characteristics.

**FIGURE 1:**
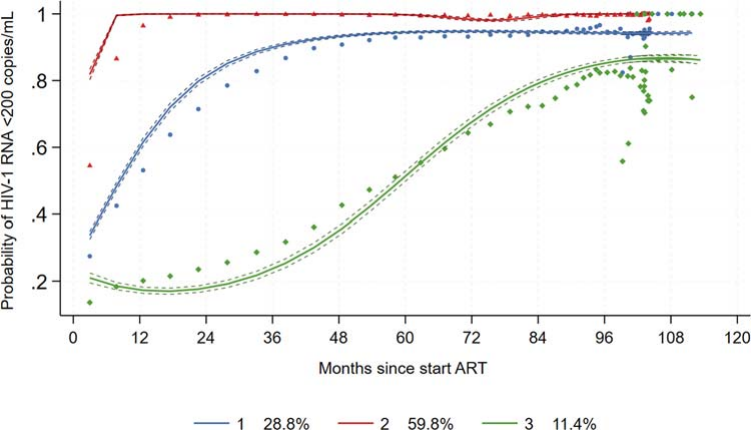
P354 | Trajectories of virological suppression over 10 years.

### P355 Towards Annual Monitoring in HIV Care: A Rapid Review of Outcomes and Implementation Considerations

Breda Patterson ^1^, Nicola Galbraith^1^, Laura Waters^1^, Liz Walder^2^, Phillipa Pristerà^2^

^1^Gilead Sciences Ltd, London, UK; ^2^PhiZ & Co, London, UK

**Background**: Monitoring intervals for people with HIV vary across settings, with guidelines typically recommending 6‐monthly clinical review and viral load testing for individuals with sustained viral suppression. However, evidence from real‐world practice, COVID‐19 service adaptations and risk‐stratified care models suggests that reduced‐frequency or annual monitoring may be feasible for selected individuals. This rapid review synthesized the most directly relevant evidence on extended monitoring beyond 6 months and explored factors associated with successful implementation.

**Methods**: A targeted rapid review was conducted to identify real‐world evidence published between 2019 and 2026 around “extended monitoring” or “reduced‐frequency monitoring” (> 6 months) for people with HIV. Searches were undertaken in PubMed, Google Scholar, national and regional HIV conference proceedings and relevant guidelines from Europe (primarily UK, Spain and Italy). Evidence from the United States and Africa was excluded. Data were extracted on monitoring interval, population characteristics, outcomes and implementation considerations.

**Results**: Twelve directly relevant items were identified and reviewed (Table 1). Extended monitoring typically involved 9–12‑monthly review, sometimes supported by a mid‑point remote or telephone check. Across settings and study types, viral suppression, adherence and quality of life were generally maintained; a small minority described increased risk of treatment failure or loss to care. Service outcomes demonstrated increased capacity through fewer routine appointments, enabling specialist teams to focus on people with greater clinical need. Remote and telephone follow‐up became routine in many settings, while structured risk‐stratified pathways, particularly in Spain, supported more personalized and efficient care. Financial outcomes included reduced laboratory utilization and lower overall service costs. People‑centred outcomes showed that many individuals valued the convenience and were generally in support of extended monitoring. However, a meaningful minority preferred more frequent contact for reassurance, reinforcing the need for choice. Emerging guidelines increasingly support flexible, risk‑stratified intervals, including annual monitoring for some.

**Conclusions**: The available evidence suggests that extended monitoring can be a feasible and acceptable option for selected people with sustained viral suppression, with potential benefits for services and their users. Successful implementation depends on clear stability criteria, robust risk stratification, effective communication, mid‑point safety checks and rapid re‑engagement pathways.

**TABLE 1:**
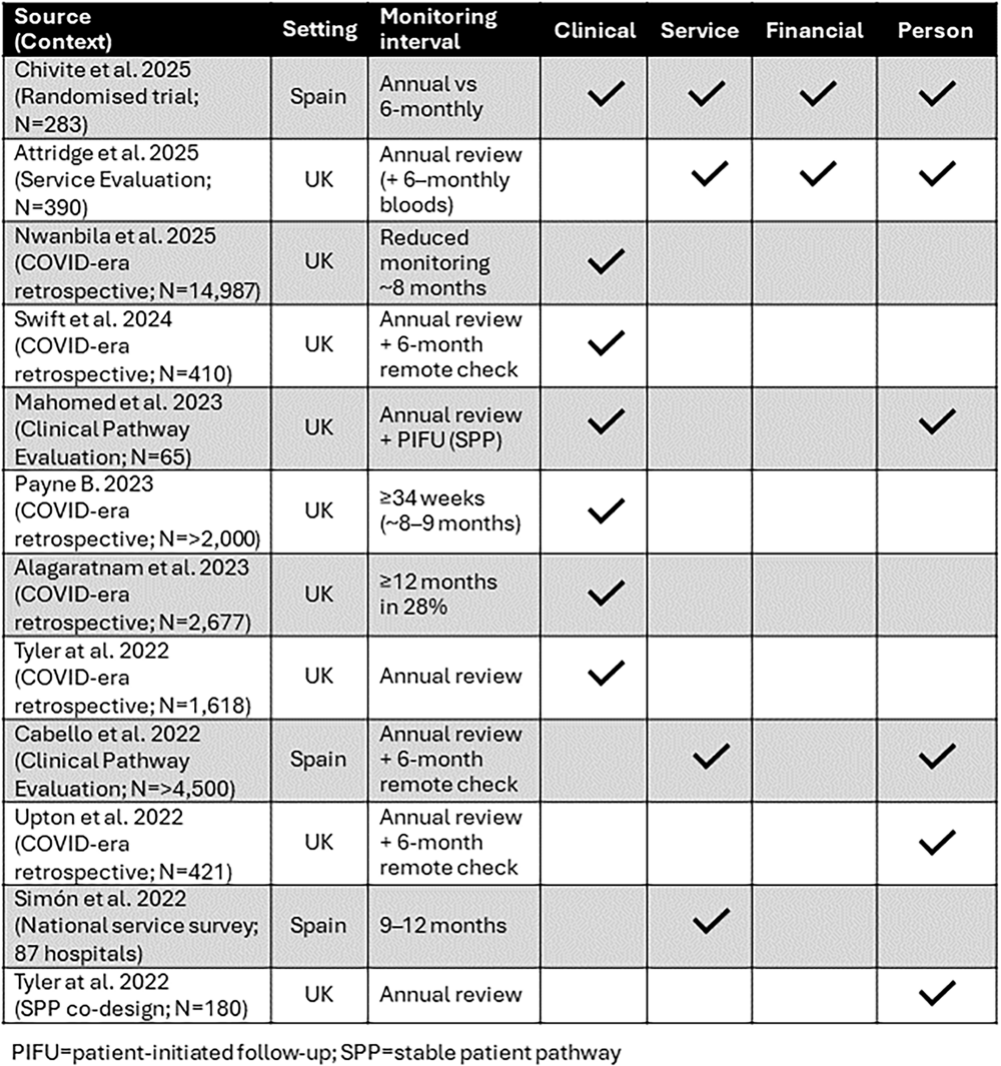
P355 | Evidence from Europe evaluating extended (≥9–12 month) monitoring intervals.

### P356 Clinician‐Reported Reasons for Switching Antiretroviral Medications in the Publicly Funded HIV Drug Treatment Programme in British Columbia, Canada: A Real‐World 16‐Year Retrospective Analysis (2009–2024)

Rosie B. Murphy^1^, Sandra Chang ^1,2^, Cassidy Tam^1^, Raquel M. Espinoza^1^, Jason Trigg^1^, Paul Sereda^1^, Wendy Zhang^1^, Julio S.G. Montaner^1,3^, Junine K. Toy^1,2^

^1^British Columbia Centre for Excellence in HIV/AIDS, Vancouver, Canada; ^2^St Pauls Hospital, Vancouver, Canada; ^3^The University of British Columbia, Vancouver, Canada

**Background**: In British Columbia (BC), Canada, the centralized BC Centre for Excellence Drug Treatment Program (DTP) provides publicly funded HIV antiretroviral treatment (ART) at no cost, to persons living with HIV. The DTP evaluates clinician requests for ART, to ensure safety and efficacy, in conjunction with clinical factors (e.g. resistance and adverse drug reaction [ADR] history). The Pharmacovigilance Initiative, established in June 2008, analyses clinician reported adverse drug‐related events as a component of participant safety monitoring. We evaluated evolving patterns in clinician‐reported reasons for ART switches, to provide a longitudinal, descriptive overview from 2009 to 2024, linking temporal prescribing patterns to market availability of newer agents.

**Methods**: This retrospective study included clients aged 18 years and older from the DTP, with a minimum of 2 ART dispensations between 1 January 2009 and 31 December 2024. A switch was defined as any addition, removal or change of drugs, in a dispensed ART regimen and follow‐up was censored at the last ART dispense on or before 31 December 2024. The 16 years of data were grouped into 3 time periods (2009–2013, 2014–2018 and 2019–2024), informed by prescribing practices and drug availability in BC. Where multiple reasons were provided, a hierarchy was developed to prioritize most clinically relevant reasons for switching. Data from the Pharmacovigilance Initiative was extracted to distinguish switches due to ADRs from switches to prevent potential ADRs.

**Results**: A total of 11,648 clients were included (median age at baseline 44 [Q1–Q3, 35–51] years; 17% assigned female sex at birth). Median follow‐up time was 9.1 (Q1–Q3, 4.0–14.0) years. During the study period, 8657 (74%) clients had ≥1 ART switch and a total of 20,187 switches were observed. Trends over time showed a decrease in the proportion of switches due to ADRs (50.4% to 31.3%) and effectiveness concerns (7.0% to 5.0%). Conversely, switches increased over time for simplification (9.9% to 32.3%) and prevention of potential ADRs (2.4% to 9.1%) (Figure 1).

**Conclusions**: In this retrospective analysis from 2009 to 2024 of a publicly funded cohort, reasons for switching ART have changed longitudinally, with availability of newer drug products providing more tolerable, effective and simpler options.

**FIGURE 1:**
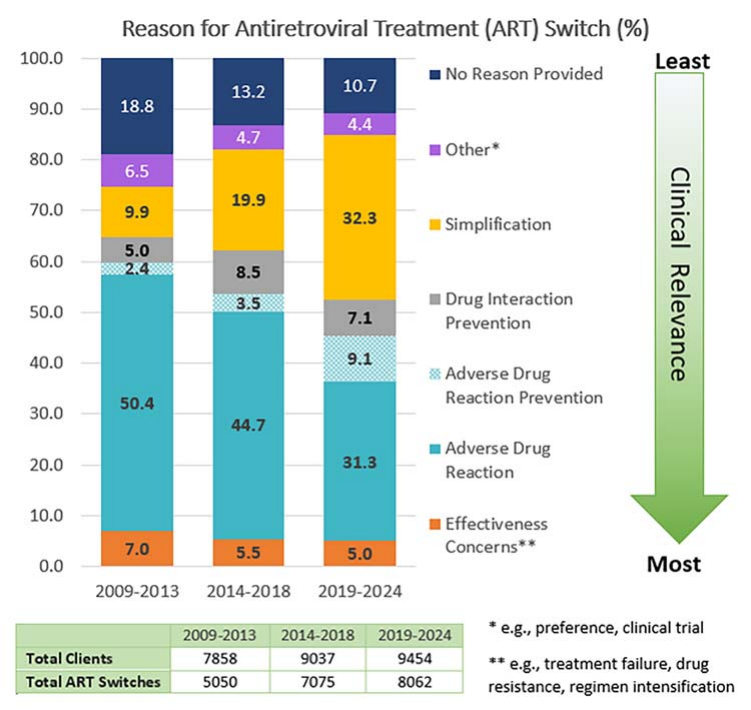
P356 | Reason for antiretroviral treatment (ART) switch (%).

### P357 Sex Work Identifies Structural Vulnerability and Lower Retention Among Migrants Living With HIV in Barcelona, 2010–2023

Elizabeth Zamora‐Clemente^1,2^, Lorena De la Mora^1,2^, Leire Berrocal^1^, Berta Torres^1,2^, Ana González‐Cordón^1,2^, María Martínez‐Rebollar^1,2^, Montserrat Laguno^1,2^, Juan Ambrosioni^1,2^, Iván Chivite^1^, Elisa De Lazzari^1,2^, Alberto Foncillas^1,2^, Júlia Calvo^1,2^, Abiu Sempere^1,2^, Raquel Aguiló^1^, Esteban Martínez^1,2^, José M. Miró^1,2^, José Luis Blanco^1,2^, Josep Mallolas^1,2^, Alexy Inciarte ^1,2^

^1^HIV Unit, Hospital Clínic de Barcelona‐IDIBAPS, Barcelona, Spain; ^2^Universitat de Barcelona, Barcelona, Spain

**Background**: Sex workers are disproportionately affected by HIV, facing discrimination and structural, social and cultural challenges. Evidence on the intersection of sex work (SW), migration and continuity of HIV care is limited. We characterized migrants living with HIV reporting SW and compared them with other migrants.

**Methods**: We conducted a retrospective study of people born outside Spain with first HIV‐care visit at Hospital Clínic de Barcelona between 2010 and 2023. SW was proactively assessed at entry. Multivariable logistic regression identified independent correlates; retention was estimated by Kaplan–Meier methods. Loss to follow‐up was defined as >6 months without an HIV care visit or CD4/HIV RNA measurement; participants with <6 months of potential follow‐up were excluded and deaths/transfers censored.

**Results**: Among 3572 migrants, 386 (10.8%) reported SW. 47.7% were ART‐naïve (vs. 39.3%; *p* = 0.002). SW were predominantly identified as transgender women (63.7% vs. 4.4%; *p* < 0.001) and referred from NGOs (56.3% vs. 20.3%; *p* < 0.001). They also had lower education, with only 12.6% reporting university studies (vs. 52.6%; *p* < 0.001) (Figure 1A). Among ART‐naïve, SW experienced lower rates of late diagnosis (42.4% vs. 50.4%, *p* = 0.043). ART‐pretreated SW reported lower virological suppression rates (58.9% vs. 77.7%, *p* < 0.001). In 3067 complete cases, reported SW was independently associated with transgender identity (aOR 21.83, 95% CI: 15.69–30.38), Latin American origin (4.02, 2.16–7.51), length in Spain (1–3 years: 2.24, 1.47–3.40; <1 year: 1.85, 1.23–2.80), syphilis seropositivity (2.12, 1.56–2.87) and ART‐naïve status (1.48, 1.04–2.11); older age (aOR/year 0.95, 0.93–0.97) and university education (0.26, 0.18–0.38) were inversely associated (Table 1). In unadjusted retention analyses among 3383 eligible participants, 5‐year retention was 46.1% (95% CI: 40.4–51.5) in SW versus 68.6% (66.8–70.3) in non‐SW (log‐rank *p* < 0.001; Figure 1B).

**Conclusions**: SW displayed lower late diagnosis rates among ART‐naïve participants, but poorer retention outcomes. These findings suggest persistent vulnerabilities and structural barriers for engagement and long‐term retention among SW. Proactive assessment at entry may identify people who could benefit from differentiated, low‐barrier retention strategies. Because retention was compared without multivariable adjustment, SW should be interpreted as a pragmatic vulnerability marker rather than an independent causal disengagement predictor.

**FIGURE 1:**
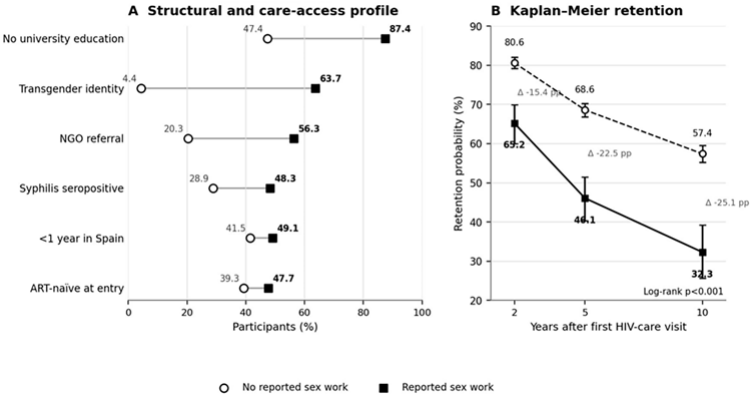
P357 | Panel A shows available‐case percentages; denominators varied by indicator (reported sex work, *n* = 304–386; no reported sex work, *n* = 2627–3186); all comparisons *p* ≤ 0.05. Panel B shows unadjusted Kaplan–Meier retention estimates with 95% CIs (*n* = 356 and *n* = 3027, respectively); log‐rank *p* < 0.001. Deaths and transfers were censored.

**TABLE 1:**
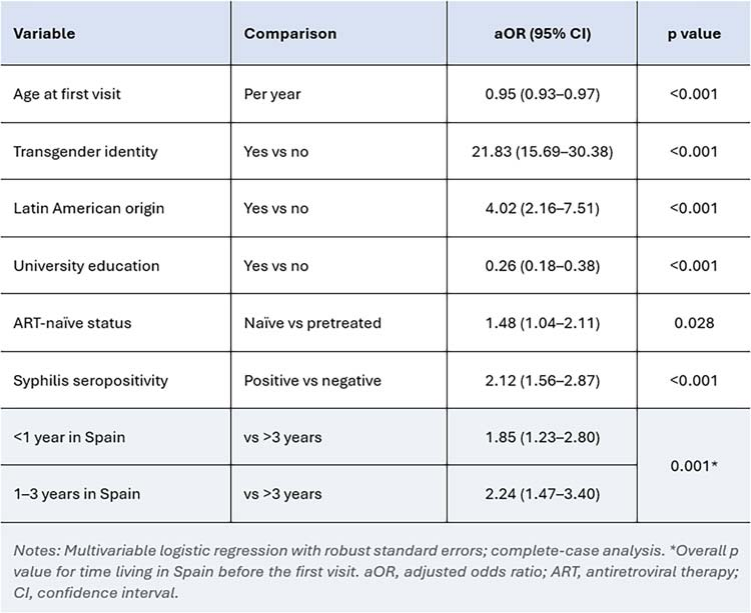
P357 | Factors independently associated with reported sex work (*n* = 3067).

### P358 Venezuelan Migrants Living With HIV in Barcelona, Spain: Addressing Knowledge Gap in Europe, a Historical Cohort

Alexy Inciarte ^1,2^, Elizabeth Zamora‐Clemente^1,2^, Leire Berrocal^1^, Ana González‐Cordón^1,2^, Lorena De la Mora^1,2^, María Martínez‐Rebollar^1,2^, Montserrat Laguno^1,2^, Juan Ambrosioni^1,2^, Iván Chivite^1^, Elisa De Lazzari^1,2^, Alberto Foncillas^1,2^, Júlia Calvo^1,2^, Abiu Sempere^1,2^, Esteban Martínez^1,2^, José M. Miró^1,2^, Jose Luis Blanco^1,2^, Berta Torres^1,2^, Josep Mallolas^1,2^

^1^HIV Unit, Infectious Diseases Department, Hospital Clínic de Barcelona‐IDIBAPS, Barcelona, Spain; ^2^Universitat de Barcelona, Barcelona, Spain

**Background**: Venezuelan humanitarian crisis has led to one of the largest cross‐border displaced population. Deterioration of Venezuela's healthcare infrastructure and infectious diseases control is associated to its resurgence and limited and shortages of diagnostics and treatment, including HIV. However, research among Venezuelan people living with HIV (PLWH) is limited and scarce. The objective was to describe sociodemographic characteristics, migration and immunovirological outcomes of Venezuelan migrants and to compare to other Latin American (LA) PLWH.

**Methods**: We conducted a retrospective observational cohort study including LA migrant PLWH attending their first consultation at the HIV Unit of Hospital Clínic de Barcelona between 2000 and 2023. Venezuelan PLWH were compared with other LA migrant PLWH regarding demographics, HIV diagnosis timing, CD4 cell count, viral load and late HIV diagnosis (LHD).

**Results**: A total of 493 Venezuelan PLWH were included, representing 16% of LA migrant PLWH in the cohort. Venezuelan PLWH were predominantly men (97%; *n* = 478). Most first consultations occurred after 2014 (81%; *n* = 399), peaking in 2018. Half were diagnosed with HIV after arrival in Spain similarly to other LA migrants (50%; *n* = 224 vs. 53%; *n* = 1233, *p* = 0.250) and 73% (*n* = 328/451) had lived in Spain for less than 3 years (Figure 1A). They also had higher unemployment rates (38%; *n* = 171 vs. 27%; *n* = 654; *p* < 0.001), but higher proportion of completed university studies (51%; *n* = 225 vs. 42%; *n* = 976; *p* < 0.001) (Table 1).

Half of Venezuelan PLWH were diagnosed with HIV between 2015 and 2023 (*n* = 248), whereas 40% (*n* = 1067) of other LA migrants were diagnosed in the same period. Among ART‐naïve (42%, *n* = 204) individuals, LHD increased over time from 39% in 2000–2008 to 52% in 2015–2023, while baseline CD4 counts progressively declined (Figure 1B). Immunological outcomes were similar between Venezuelan and other LA migrants, including LHD (49%; *n* = 99 vs. 51%; *n* = 624) and advanced HIV disease (24%; *n* = 48 vs. 25%; *n* = 302). However, ART‐experienced Venezuelan PLWH showed lower virological suppression rates (66%; *n* = 186 vs. 72%; *n* = 1002; *p* = 0.053).

**Conclusions**: Venezuelan PLWH in Spain represent a rapidly growing migrant population. Increasing LHD and lower virological suppression among ART‐experienced individuals suggest continuity‐of‐care disruptions prior to migration.

**FIGURE 1:**
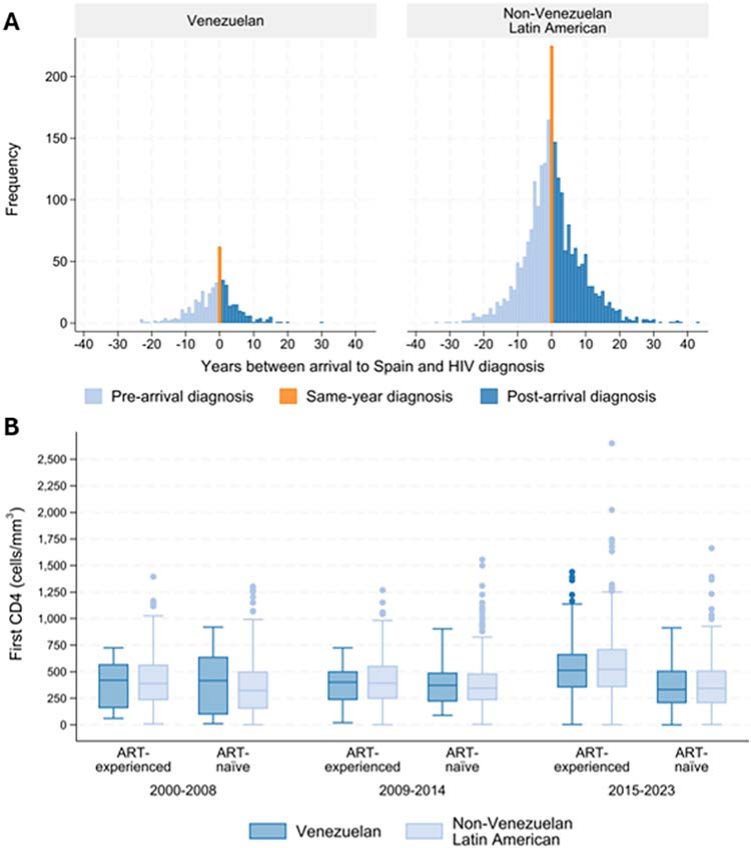
P358 | (A) Distribution of HIV diagnosis depending on the arrival to Spain (Venezuelan vs. Other Latin American migrants); (B) baseline CD4 cell count among newly enrolled Venezuelan and other Latin American migrant PLWH by time periods.

**TABLE 1:**
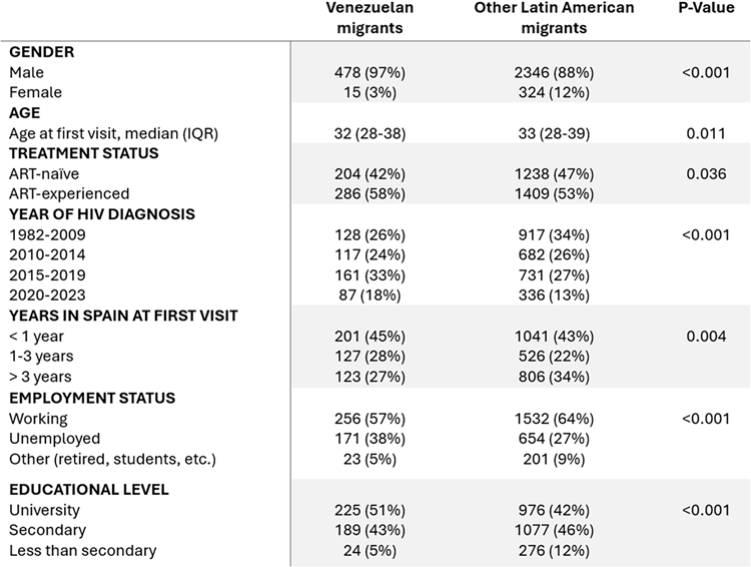
P358 | Baseline sociodemographics among newly enrolled Venezuelan and other Latin American migrant PLWH.

### P359 Abstract Withdrawn

### P360 BRIDGE Study: Evaluating Prior Antiretroviral Therapy in Foreign‐Born People Living With HIV Entering Care in Spain

Verónica Gil‐Girona^1^, Francisco Jover‐Díaz ^1,2^, Jorge Peris‐Garcia^1,2^, Elisabet Delgado‐Sánchez^2^, Andrea Riaño‐Pérez^2^, Maria Ángeles Bernabeu‐Martínez^2^

^1^Miguel Hernandez University, Alicante, Spain; ^2^Hospital Clinico Universitario San Juan, Alicante, Spain

**Background**: Migration continues to shape the HIV epidemic in Europe, yet disparities in antiretroviral therapy (ART) access persist globally. Many foreign‐born people living with HIV (PLWH) arrive in high‐income countries on regimens that differ from local guideline standards. This study assessed the adequacy of prior ART among foreign‐born PLWH entering care in a Spanish hospital.

**Methods**: A retrospective, observational study was conducted using clinical records of foreign‐born adults with confirmed HIV infection who initiated follow‐up at the Infectious Diseases Unit of Sant Joan d'Alacant Hospital after receiving ART abroad. Sociodemographic, clinical and therapeutic variables were collected. ART regimens were classified according to GeSIDA guidelines. Descriptive statistics and chi‐square or Fisher's exact tests were applied.

**Results**: A total of 130 patients were included; median age was 28 years and 71.5% originated from Latin America and the Caribbean. Most had undetectable viral load (74.6%) and preserved immune status (CD4 ≥500/µL in 70.8%). Prior ART consisted mainly of triple therapy (89.2%), predominantly integrase inhibitor‐based (39.2%) or NNRTI‐based (37.7%). Only 12.3% of regimens met preferred or alternative criteria, while 77.7% were classified as not recommended. Only 3.1% represented regimens with known low efficacy. After entering care, 66.9% of patients underwent ART modification, mainly for optimization (57.7%), see Table 1. Pill burden significantly decreased (Wilcoxon Z = –3.91; *p* < 0.001).

**Conclusions**: Most foreign‐born PLWH arrived on ART regimens not aligned with national guidelines, reflecting global inequities in ART availability and the coexistence of historical and modern regimens. Despite this, virological control was generally preserved. Transition to guideline‐recommended therapy frequently resulted in optimization and reduced pill burden. These findings highlight the need for harmonized international ART access and structured evaluation of prior regimens in migrant populations.

**TABLE 1:**
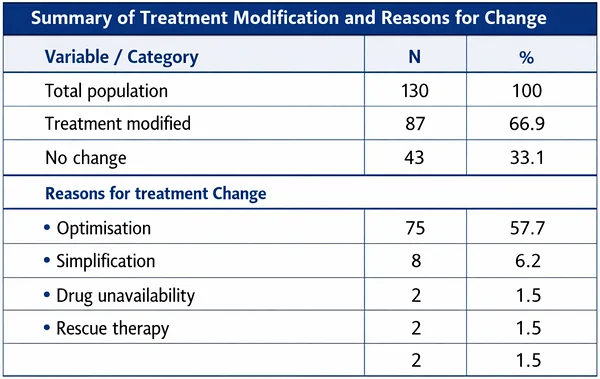
P360 | Overview of ART changes and optimization motives in migrant HIV population.

### P361 Abstract Withdrawn

### P362 Using a Large Language Model to Classify Reasons for Multi‐Tablet Regimen Use in People Living With HIV: A Pilot Study Within the Swiss HIV Cohort Study

Lena Bartl ^1^, Marius Zeeb^1,2^, Cedric Dolle^1^, Krystyna Vulchanova^1^, Johannes Nemeth^1^

^1^Department of Infectious Diseases, University Hospital Zürich, Zürich, Switzerland; ^2^Instiute of Medical Virology, University of Zürich, Zürich, Switzerland

**Background**: Single‐tablet regimens (STR) are the preferred treatment for people living with HIV due to improved adherence, tolerability and simplified dosing. Despite STR availability in Switzerland, a subset of participants in the Swiss HIV Cohort Study (SHCS) at the Zürich centre remain on multi‐tablet regimens (MTR). We aimed to develop and evaluate a large language model (LLM)‐based classification system to identify the underlying reasons for continued MTR use from routine electronic patient records, to optimize clinical workflow and reduce administrative burden on physicians.

**Methods**: The study population consisted of all SHCS participants under care at the Zürich centre currently receiving MTR for HIV treatment. A classification system comprising seven categories was developed, including resistance, virological failure, side effects, long‐acting therapy and patient preference. A gold standard dataset of 381 patient records was compiled by two independent physicians who each reviewed the full patient history and assigned a category, after which assignments were compared, and the most aligned classification was retained. A structured prompt encoding the classification rules was developed and applied to the USZ‐hosted internal LLM, which processed patient records and returned a category output. Accuracy was assessed against the gold standard in a pilot dataset.

**Results**: Using single‐shot prompting, the LLM correctly identified 328 out of 381 cases in the pilot phase, corresponding to an accuracy of 86.1% (Figure 1). Discrepancies were reviewed to inform prompt refinement before further application.

**Conclusions**: When provided with a structured and explicit prompt, the LLM hosted within the secure USZ network demonstrated high classification accuracy regarding MTR use among SHCS participants. These findings suggest that LLM‐based processing of clinical records holds promise for streamlining documentation tasks and reducing administrative workload, allowing physicians to redirect time towards direct patient care.

**FIGURE 1:**
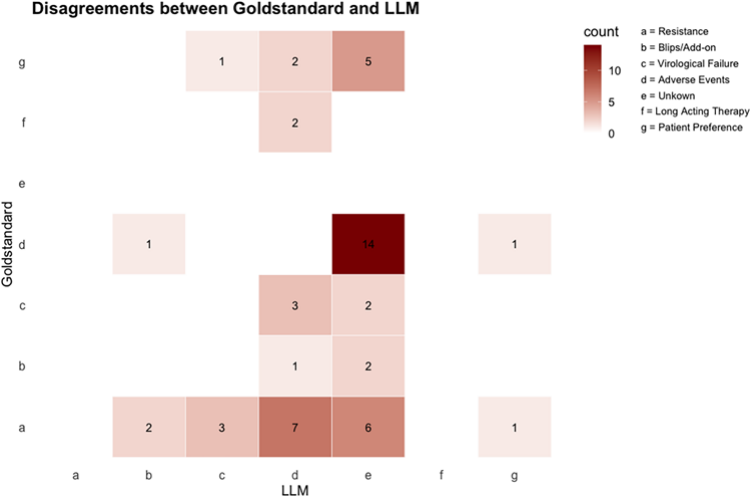
P362 | LLM categorization mismatch heatmap.

### P363 Effectiveness and Implementation Outcomes of a Co‐Designed, Person‐Centred Care Model for People Living With HIV in Delta State, Nigeria: A Hybrid Stepped‐Wedge Cluster‐Randomized Trial

Bonaventure Ukoaka ^1,2^, Awajinor Ismael^1^, Nerrisa Okwuokei^1^, Morayo Happy Akinyomi^1^, Oghenekparobor Elijah^1^, Chinazom Ifedigbo^1^, Gerald Oraegbu^1^, Etieno Mbaba^1^, Saviour Wilson^1^, Henry Udenenwu^1^, Grace Jimbo^1^, Okezie Onyedinachi^1^, Andy Eyo^1^

^1^Excellence Community Education Welfare Scheme (ECEWS), Asaba, Delta, Nigeria; ^2^First On‐Call Initiative, Port Harcourt, Rivers, Nigeria

**Background**: Suboptimal client–provider experiences perpetuate HIV treatment interruptions, discourage re‐engagement and undermine long‐term outcomes. The role of person‐centred care (PCC) in improving client–provider interactions and clinic communication remains underexplored in sub‐Saharan Africa.

**Methods**: We conducted a hybrid type 2 effectiveness‐implementation, stepped‐wedge, cluster‐randomized trial with an embedded qualitative process evaluation, assessing a co‐designed PCC model (PCCm) across two large ART sites in Delta State, Nigeria. The PCCm comprised healthcare worker training, client health promotion and routine clinic feedback. Primary outcomes were viral suppression (<200 copies/mL) and ART retention (proxy continuity of treatment, CoT) at 6 months. PCC fidelity and client satisfaction were assessed via pre‐ and post‐intervention surveys; focus group discussions and in‐depth interviews explored barriers and preferences to care and were analysed thematically. Multivariable logistic regression evaluated viral suppression and retention, adjusting for age, sex and facility.

**Results**: Among 2425 clients receiving care during the intervention (73.5% female; mean age: 46.6 years), PCC scores were high at both sites (mean 55.5 ± 3.3; *F* = 52.73, *p* < 0.001). Post‐intervention satisfaction was high overall, with facility being the only significant predictor of lower scores (Site 2: B = –0.307, 95% CI: −0.527 to −0.088). Median clinic visit duration was 1h:48m (IQR 1h:10m–3h:08m). While 95.8% found their appointment date convenient, only 27.8% were offered a choice of appointment time. ARV refill coverage improved at site 1 (92% to 94%) but declined at site 2 (97% to 90%) between phases. Proxy CoT remained high at both sites (96.8% and 94.0%, respectively). Viral suppression improved at site 1 (93.9% to 95.6%) and rose markedly at site 2 in phase 2 (95.8% to 99.1%) (Figure 1). Qualitative findings identified provider empathy, shared decision‐making, communication and stigma‐free services as key PCC touchpoints. Decentralized service integration was a major barrier, driven by prior negative experiences with siloed care, privacy concerns and unreceptive healthcare workers. Proposed solutions included digital health records, strengthened peer support and enforcement of anti‐stigma legislation.

**Conclusions**: The PCCm improved clinic experience and care engagement, with corresponding gains in retention and viral suppression at both sites. A PCC‐focused service delivery approach shows promise in strengthening HIV treatment outcomes and warrants further evaluation for scale‐up.

**FIGURE 1:**
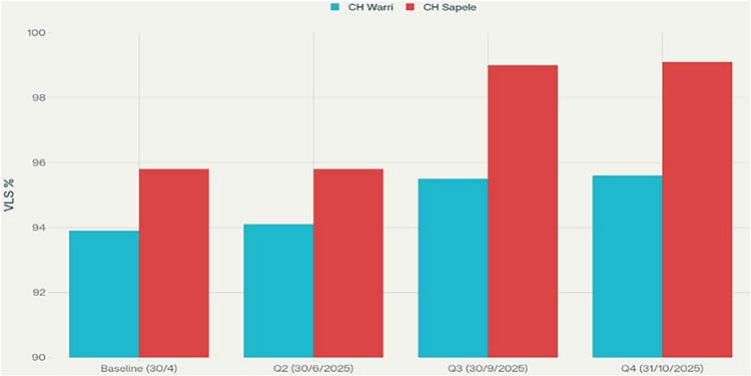
P363 | Viral load suppression (VLS) trend across both sites.

### P364 Patterns of HIV Care Transfer Among Migrant Populations in Two HIV Clinics in Hertfordshire: Identifying Trends in Antiretroviral Therapy, Treatment Interruptions and Virological Suppression

Sorina Bolache ^1^, Marta Boffito^2^, Rasha Dabis^2^, Sarah Edwards^2^, Nadia Naous^1^

^1^Pharmacy, Chelsea and Westminster NHS Foundation Trust, London, UK; ^2^HIV, Chelsea and Westminster NHS Foundation Trust, London, UK

**Background**: Transfer of HIV care following migration accounted for just over 2500 people living with HIV in England in 2024 [1]. HIV services must ensure safe and effective transfer of care (TOC) pathways [2]. We evaluated trends in TOC among people diagnosed with HIV before continuing care in England, subsequently attending services in Hertfordshire.

**Methods**: We performed a retrospective audit of case notes for people attending our services who were diagnosed with HIV before continuing care in England between January 2023 and December 2025. Data were extracted from routine clinical records and handled in accordance with UK GDPR and Data Protection Act 2018. Prison service TOCs were excluded. Demographic, clinical and prescribing data were extracted from electronic patient records. Variables included demographics, country/region of origin, year of transfer, availability of TOC documentation, viral load and antiretroviral therapy (ART) regimen at transfer and subsequent regimen changes. ART regimens were categorized by backbone and third agent class. We analysed prescribing patterns, virological suppression and interruptions to care.

**Results**: Overall, 223 individuals were identified, with 16 excluded from this review, of whom 122(59%) were female. In total, 100 (53%) originated from African countries and 85 (41%) from other UK centres (Figure 1). TOC documentation availability and regimen at the point of transfer are shown in Table 1. Twenty‐five individuals (12%) experienced treatment interruptions, of whom 20 (9%) had detectable viral loads. Following re‐initiation of therapy, all achieved viral suppression within 6 months, with 100% restarted on a second‐generation integrase inhibitor (INSTI). Of note, 80% of those with treatment interruptions had no TOC documentation.

**Conclusions**: A high incidence of missing transfer information was identified, potentially affecting continuity of care and treatment choice post‐transfer. This highlights the importance of robust transfer processes, accompanied by prompt clinical review, access to multidisciplinary team support and virtual clinic review to maintain continuity of care and ensure appropriate ongoing management.

**References**

1. UK Health Security Agency. HIV testing, PrEP, new HIV diagnoses and care outcomes for people accessing HIV services: 2025 report. Available at: https://www.gov.uk/government/statistics/hiv‐annual‐data/hiv‐testing‐prep‐new‐hiv‐diagnoses‐and‐care‐outcomes‐for‐people‐accessing‐hiv‐services‐2025‐report. Accessed 19/06/2026.

2. British HIV Association (BHIVA). BHIVA Standards of Care for People Living with HIV 2018. Available at: https://bhiva.org/standards‐of‐care‐2018/. Accessed 02/06/2026.

**FIGURE 1:**
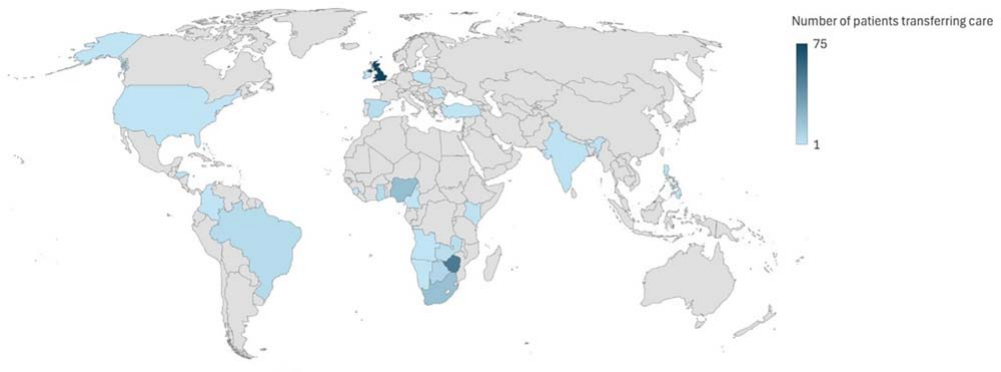
P364 | Global origins of transfer of care to Hertfordshire HIV services between January 2023 and December 2025.

**TABLE 1:**
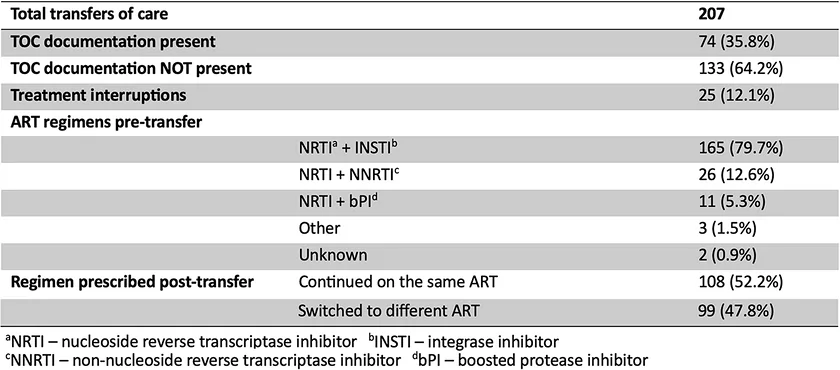
P364 | Summary or transfer of care data: TOC documentation, treatment interruptions and breakdown of ART regimens pre‐transfer.

### P365 An Evaluation of the HIV In‐Reach Service at a Multi‐Hospital Urban Centre: A New Model for HIV Inpatient Care

Oliver Hug, Tamara Elliott, Lauren Nicholls, Lucy Garvey, Nicola Mackie, Linda Greene, Demiana Sidhom, Rory Grier‐Gavin, Borja Mora‐Peris

Imperial College Healthcare NHS Trust, London, UK

**Background**: In 2020 the HIV inpatient service at Imperial College Healthcare NHS Trust (ICHNT) was merged with Chelsea and Westminster Hospital (CWH) and HIV‐related inpatient admissions were relocated there. However, people with HIV (PWH) still present to ICHNT and require HIV specialist input, thus a HIV in‐reach service was introduced. A multi‐disciplinary team remotely reviews PWH admitted to anywhere within ICHNT. An evaluation of the in‐reach service was performed including assessment of the volume, flow and input from the HIV in‐reach team.

**Methods**: Data was collected from a prospectively completed in‐reach spreadsheet updated at daily multidisciplinary meetings alongside a review of electronic patient records for all PWH admitted at ICHNT between August 2023 and August 2024. This included type of input and need for transfer to the HIV inpatient service at CWH.

**Results**: The HIV in‐reach service reviewed 281 PWH during this period. 32/281 (11%) were accepted for transfer to CWH. 201/281 (72%) had an undetectable viral load on antiretroviral therapy (ART) and 137/281 (49%) had an admission CD4 count of > 200 cells/mL. This contrasted with those requiring transfer to CWH with 13/32 (41%) undetectable on ART and 14/32 (44%) with a CD4 count of > 200 cells/mL. The most common reason for admission was infection at 30% for the overall cohort and 69% of those requiring transfer. Most frequent advice from the in‐reach team was regarding blood tests and other investigations, but also concerned drug−drug interactions and management of HIV specific complications including opportunistic infections in 25% of admissions. Of note, ART switch was recommended in 35 (12%) cases overall and in 3 (9%) of the transfers (see Table 1).

**Conclusions**: As the population living with HIV ages with disproportionate rates of multimorbidity, the number of PWH admitted to hospitals with non‐HIV related issues will increase. However, significant HIV specialist input is required regardless of the reason for admission. This in‐reach model offers timely transfer to a dedicated HIV inpatient service when required, while providing equitable access to specialist HIV advice, review and active monitoring for all inpatients living with HIV, demonstrating a sustainable approach to future HIV inpatient care.

**TABLE 1:**
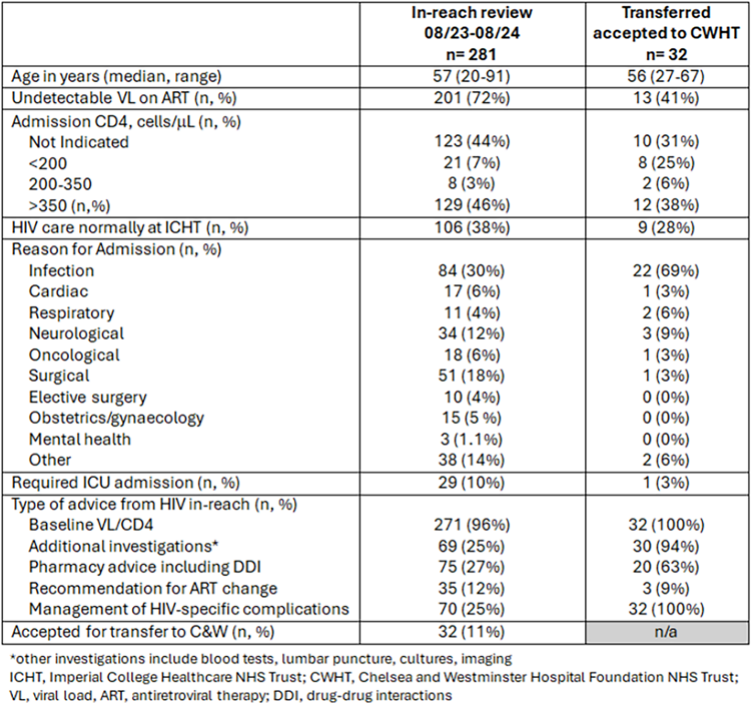
P365 | Details of inpatient admissions for all in‐reach patients from August 2023 to August 2024 and those requiring transfer to an HIV inpatient service.

### P366 Capacity Modelling in HIV Care: Workforce Implications of a Nurse‐Led Long‐Acting Injectable ART Service

Amy Walsh, Aoife G Cotter, Christine Kelly, Padraig McGettrick, Eavan Muldoon, James Woo, Mairead O'Connor, Tara McGinty

Infectious Diseases, Mater Misericordiae University Hospital, Dublin, Ireland

**Background**: Long‐acting injectable (LAI) antiretroviral therapy (ART) is an alternative to daily oral ART for people with HIV (PWH). While clinical efficacy is established, workforce implications remain poorly characterized. Following implementation of a structured nurse‐led LAI pathway within a large Dublin HIV service (> 1800 PWH), delivered by a multidisciplinary team including a single clinical nurse specialist (CNS), we evaluated nursing workload, service capacity, treatment continuity and virological outcomes. The LAI pathway was introduced within existing resources, requiring capacity modelling to determine the maximum number of patients that could be safely supported.

**Methods**: Nursing workload associated with LAI and stable oral ART was prospectively measured and standardized into 5‐min workload units, comprising direct and indirect nursing activities. LAI workload was compared with stable oral ART over an equivalent 8‐week period. Treatment continuity (oral bridging) and virological outcomes were also recorded. Data are presented as *n* (%), median (interquartile range) unless otherwise stated.

**Results**: 20 patients, 50% male, median age 41 (32–43) years, contributed 104 LAI clinic appointments between April 2025 and April 2026. All were clinically stable and virologically suppressed before switching to LAI therapy. Each 8‐week follow‐up cycle required 8 workload units (40 min), comprising 4 direct and 4 indirect units. Appointment duration ranged from 30 to 50 min, with 40 min representing the typical workload used for capacity modelling. Stable oral ART required 2.5 workload units, representing a threefold increase in nursing workload for LAI care. Within a service of > 1800 PWH supported by a single HIV CNS, capacity was limited to 30 LAI patients. At full capacity, LAI delivery required 240 workload units per 8‐week cycle (20 nursing hours), equating to approximately 8% of one CNS full‐time position for 1.7% of the clinic population. Equivalent management on oral ART required 75 workload units (2.5% of CNS capacity). Since service initiation in January 2024, no virological failures and one planned oral bridging episode have occurred, demonstrating maintained treatment continuity within the nurse‐led model.

**Conclusions**: Nurse‐led LAI ART delivery was safe but required substantially greater CNS resource than oral ART. These findings highlight workforce constraints within existing service models and emphasize the need for workforce planning and investment to support sustainable expansion of LAI programmes.

### P367 PERDÍ‐VIH Study: Model of Care Evaluation and Risk Factors for Loss to Follow‑Up in People Living With HIV

Andrés Fernández‐Montealegre^1^, Francisco Jover‐Díaz ^1,2^, Maria Ángeles Bernabeu‐Martínez^3^, Elisabet Delgado‐Sánchez^2^, Andrea Riaño‐Pérez^2^, Jorge Peris‐Garcia^1,2^

^1^Universidad Miguel Hernandez, Alicante, Spain; ^2^Unidad de Enfermedades Infecciosas, Hospital Clínico Universitario San Juan de Alicante, Alicante, Spain; ^3^Servicio de Farmacia. Hospital Clínico Universitario San Juan de Alicante, Alicante, Spain

**Background**: Loss to follow‑up among people living with HIV represents a clinical and public health challenge, associated with an increased risk of transmission and higher mortality. Understanding the characteristics of patients who are lost to follow‑up and the clinical consequences of this phenomenon is essential to improving continuity of care.

**Methods**: A retrospective observational study was conducted in the Infectious Diseases Unit of the Hospital Clínico Universitario de San Juan (Alicante). A total of 209 patients diagnosed with HIV since 2016 were reviewed and sociodemographic, clinical and virological variables were recorded. Loss to follow‑up was defined as the absence of clinical visits for ≥12 months. Losses were analysed using bivariate analyses, a multivariable logistic regression model and Kaplan–Meier curves to estimate time to loss. The potential influence of the COVID‑19 pandemic was also assessed.

**Results**: Overall, 14.8% of patients (*n* = 50) were lost to follow‑up. Those lost were younger (30.5 vs. 34 years; *p* = 0.016), had a higher proportion of non‑Spanish nationalities (29.8% vs. 14.1%; *p* = 0.010) and were less likely to have a stable partner (12.3% vs. 29.2%; *p* = 0.008). Detectable viral load at the last visit was the factor most strongly associated with loss (52.4% vs. 16.5%; *p* < 0.001). In the multivariate model, non‑Spanish nationality (OR 2.84; 95% CI: 1.21–6.62; *p* = 0.016) and detectable viral load (OR 5.9; 95% CI: 2.4–12.5; *p* < 0.001) remained independently associated with a higher risk of loss. The probability of remaining in care at 1, 3 and 5 years was 86%, 74.5% and 70.2%, respectively. Kaplan–Meier curves showing that patients with an undetectable viral load before loss to follow‑up have higher retention over time than those with a detectable viral load (Figure 1).

**Conclusions**: The rate of loss to follow‑up in our cohort is comparable to that reported in similar settings. Detectable viral load at the last visit, younger age and non‑Spanish origin were associated with a higher likelihood of loss to follow‑up. A temporal analysis showed a greater proportion of losses occurring early in the clinical follow‑up period. Unlike other cohorts, a negative impact on patient retention during the post‑COVID period.

**FIGURE 1:**
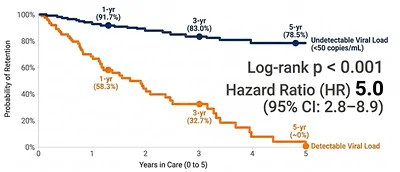
P367 | Kaplan–Meier curves of time to loss to follow‑up stratified by viral load at the last visit (undetectable vs. detectable).

### P368 Clinical Dataset Findings From the Evaluation of the EXPAND NEL North East London‐Wide Digital Oral PrEP Pilot

Laura Leigh Vetters ^1^, Melanie Smuk^1^, Ollie Lloyd‐Houldey^1^, Nishat Halim^1^, Sara Paparini^1^, Vanessa Apea^1,2^, Chloe Orkin^1,2^

^1^SHARE Collaborative, Centre for Immunobiology, Blizard Institute, Queen Mary University of London, London, UK; ^2^Barts Health NHS Trust, London, UK

**Background**: North East London has one of the highest HIV incidence rates in England, suggesting that current delivery models are failing to reach those in need. To address this, the ExPAND NEL pilot aims to improve access to PrEP by offering a digital PrEP pathway to users attending NHS sexual health clinics in East London.

**Methods**: Electronic clinical records from people prescribed PrEP at 5 East London clinics were stratified into pilot pre‐ and post‐launch cohorts using the first digital record date for the digital PrEP service (pre 2/5/2023–27/2/2025, post plus digital service 28/2/2025–5/12/2025). Data for clinic and digital PrEP users were aggregated to a single person level capturing cumulative individual prescribing and visit information. Descriptive statistics were used to compare demographics and total months of PrEP dispensed across the pilot pre‐launch, post‐launch and digital cohorts, with clear denominators reported to account for missing data. Pie charts describe the distribution of PrEP prescriptions by pathway.

**Results**: 4458 PrEP users accessed PrEP through the clinic before pilot launch and 2736 after (Table 1). The majority identified as men both pre‐launch (91.7%) and post‐launch (92.3%), compared to 94.4% of digital service users (DSUs) (*n* = 2517). The proportion of women was low across both pathways (5.5% and 5.2% vs. 3.2%). Most users identified as homosexual across all pathways (83.70% and 84.7% vs. 79.5%). Users were predominantly White, with a slightly higher proportion among DSUs (70.4%) than clinic users (62.9% and 60.6%). Uptake among Black Heritage Heterosexual Men, Black Heritage Heterosexual Women and Ethnic Minority Heterosexuals without Black Heritage was low and broadly similar across modalities (Table 1). Persistence, determined by users who obtained a second prescription, was much higher among clinic users (92.4%) compared to DSUs (68.9%) (Figure 1). However, among those who continued, a higher proportion of DSUs remained on PrEP for more than 6 months (26% vs. 3%).

**Conclusions**: Despite digital PrEP expansion, key underserved populations are still not accessing North East London services. Lower prescription persistence rates among DSUs suggest additional challenges to sustained engagement. Targeted interventions and novel modalities are needed to improve equitable uptake among underserved populations.

**TABLE 1:**
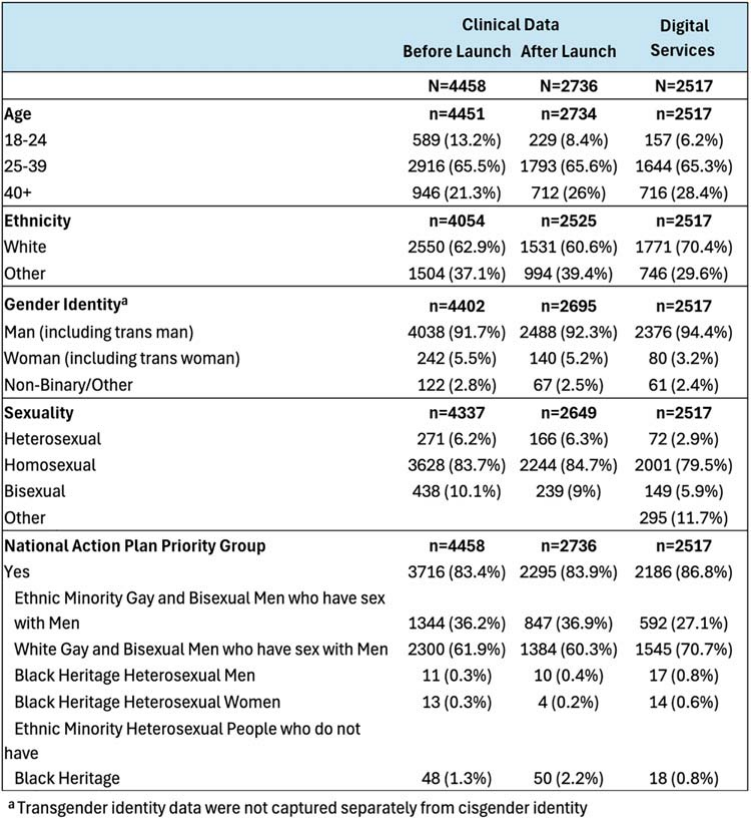
P368 | Characteristics of PrEP users accessing clinical and digital services (reported based on the first recorded entry).

**FIGURE 1:**
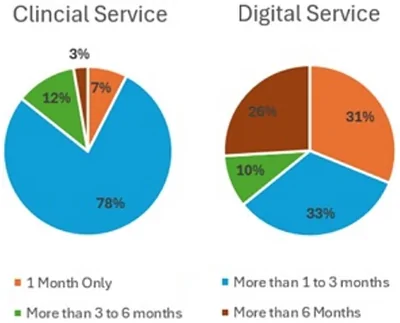
P368 | Duration of PrEP tablet prescriptions in clinical and digital service models (prescription duration reflects post‐launch data only).

### P369 Survey Findings From the Evaluation of the EXPAND NEL North East London‐Wide Digital Oral PrEP Pilot

Laura Leigh Vetters ^1^, Melanie Smuk^1^, Ollie Lloyd‐Houldey^1^, Nishat Halim^1^, Sara Paparini^1^, Vanessa Apea^1,2^, Chloe Orkin^1,2^

^1^SHARE Collaborative, Centre for Immunobiology, Blizard Institute, Queen Mary University of London, London, UK; ^2^Barts Health NHS Trust, London, UK

**Background**: North East London has one of the highest HIV incidence rates in England, suggesting that current delivery models are failing to reach those in need. To address this, the ExPAND NEL pilot aims to improve access to PrEP by offering a digital PrEP pathway to PrEP users attending NHS sexual health clinics in East London.

**Methods**: Comparative analyses were conducted using clinical and digital service survey data. Matched survey items were summarized descriptively, with denominators reported to quantify missingness. Two prespecified outcomes were assessed inferentially: (i) likelihood of initiating PrEP in the absence of the selected delivery mode and (ii) perceived inclusivity and cultural sensitivity of the delivery mode. Pearson's chi‐squared and Fisher's exact tests were applied. Family‐wise error rate was controlled at 5% using Bonferroni correction with statistical significance defined as *p* < 0.025.

**Results**: PrEP users were predominantly white, gay, cisgender men (Table 1). However, this was more pronounced among digital service users (DSUs) compared to clinic users, where more PrEP users identified as White (77.7% and 68.9%), gay (72.8% and 63.0%) and cisgender men (92.2% and 80.9%). Cisgender women and heterosexual participants were underrepresented among both pathways (2.1% and 4.1%). There was a significant difference in response to PrEP initiation if their delivery service was unavailable (*p* < 0.001). Users reported they would not have initiated PrEP without their chosen delivery pathway (DSU: 15.6% and clinic: 38.3%). Meanwhile, DSUs were more likely to report that they would have initiated PrEP even if online provision had not been available (DSU 70.8% vs. clinic 34.0%). Both DSU and clinic users considered the service to be inclusive and culturally sensitive with no statistical differences between the delivery methods (*p* = 0.096).

**Conclusions**: Digital service users remained predominantly white, cisgender men who have sex with men. However, the pilot is an important step forward towards more equitable PrEP access because both pathways reached users who indicated that would not have initiated PrEP without their chosen delivery pathway. The findings emphasize the need for differentiated delivery pathways to equitably reach underserved populations.

**TABLE 1:**
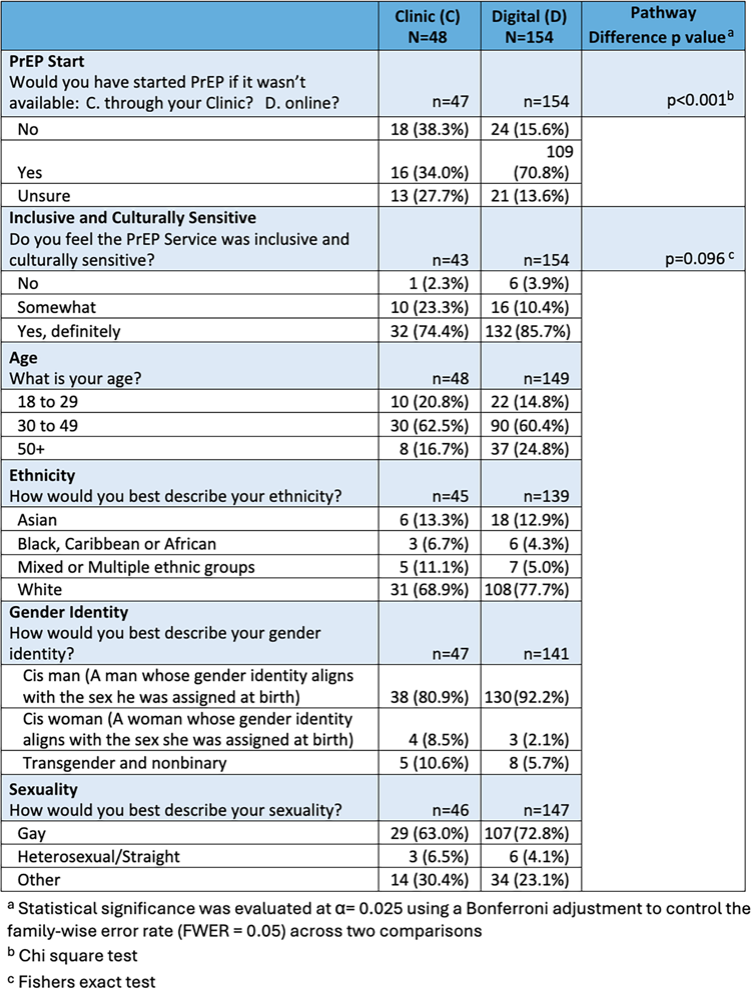
P369 | Survey results table for digital and clinic users.

### P370 “U = U for All”: The Personal and Public Health Impact of Long‐Acting Injectable Antiretrovirals in a Systemically Marginalized Population of People Living With HIV at the Kirketon Road Centre

Bruce Taylor^1^, Vendula Belackova^1,2^, Ian Anderson^1^, Matilda Eyre^1^, Lucy Fromel^1^, Sam King^1^, Eugene Priscott^1^, Phillip Read^1,3^, Yen Li ^3^

^1^Kirketon Road Centre, South Eastern Local Health District, Sydney, Australia; ^2^Social Policy Research Centre, University of New South Wales, Sydney, Australia; ^3^Sexual Health and Blood Borne Virus Services, South Eastern Local Health District, Sydney, Australia

**Background**: KRC provides care to people experiencing significant disadvantage. People living with HIV (PLWH) in this setting often face challenges adhering to daily oral antiretroviral therapy (ART), resulting in detectable viral load (VL), risking progression of HIV‐related illness and increased public health risk. Long‐acting, injectable antiretrovirals (LAI‐ARVs) can be suitable for individuals with adherence difficulties.

**Methods**: A retrospective review was conducted of all PLWH who initiated long‐acting cabotegravir plus rilpivirine at KRC between December 2022 and December 2025 (*n* = 15). Demographics, behavioural risks, adherence, CD4 counts, viral load and engagement with care were examined for 6 and 12 months before and after LAI‐ARV initiation.

**Results**: The mean client age was 40. 67% were cis‑male (all men who have sex with men), 40% identified as Aboriginal and 13% were ineligible for Medicare. In the 12 months prior to starting LAI‐ARV, 87% reported injecting drugs, 33% had no fixed address and 33% were on antipsychotics; 80% had poor adherence to oral ART and 60% had at least one episode of detectable VL (> 200 copies/mL). At 6 and 12 months, 12 and 8 participants, respectively, remained on LAI‐ARV. At analysis in December 2025, 12 (80%) of the 15 participants remained on LAI‐ARV. At 12 months, three had been overdue by > 1 week. People with VL > 200 before switch were 4.9 times less likely to have detectable VL post switch (OR 4.91, 95% CI: 1.63–14.81, *p* = 0.005). Only 13% (*n* = 2) had a VL > 200 in the 12 months post‐initiation while on LAI‐ARV. CD4 count increased by 120 cells/mm^3^ (β = 120.3; 95% CI: 46.6–193.9; *p* = 0.002). There was no significant change in clients’ attendance to the service.

**Conclusions**: LAI‐ARVs were effective and feasible in a highly marginalized urban population. LAI‐ARVs reduced VL and increased CD4 count among people with complex needs, offering a positive personal and population‐level impact.

## Co‐Morbidities and Complications of Disease and/or Treatment: Ageing and Frailty

### P371 Immune‐Inflammatory Profile With High Inflammation, Innate Immune Activation and Immunosenescence Is Associated With Higher Mortality in People With HIV

Moises Alberto Suarez‐Zdunek ^1^, Ayleen Mufudza^2^, Andreas Dehlbæk Knudsen^1^, Sisse R. Ostrowski^3,4^, Marius Trøseid^5,6^, Thor Ueland^6,7^, Thomas L. Benfield^4,8^, Patrick W.G. Mallon^2^, Susanne Dam Nielsen^1,4^

^1^Viroimmunology Research Unit, Department of Infectious Diseases, Copenhagen University Hospital – Rigshospitalet, Copenhagen, Denmark; ^2^Centre for Experimental Pathogen Host Research, School of Medicine, University College Dublin, Dublin, Ireland; ^3^Department of Clinical Immunology, Copenhagen University Hospital – Rigshospitalet, Copenhagen, Denmark; ^4^Department of Clinical Medicine, Faculty of Health and Medical Sciences, University of Copenhagen, Copenhagen, Denmark; ^5^Research Institute of Internal Medicine, Oslo University Hospital Rikshospitalet, Oslo, Norway; ^6^Section of Clinical Immunology and Infectious Diseases, Oslo University Hospital Rikshospitalet, Oslo, Norway; ^7^Institute of Clinical Medicine, University of Oslo, Oslo, Norway; ^8^Department of Infectious Diseases, Copenhagen University Hospital – Amager and Hvidovre, Hvidovre, Denmark

**Background**: Living with HIV is associated with inflammation‐associated comorbidities, and persistent inflammation may also impact long‐term survival. Yet, integrative immune‐inflammatory profiling remains to be assessed in relation to incident outcomes. We aimed to assess if immune‐inflammatory profiles are associated with overall and cause‐specific mortality in people with HIV.

**Methods**: In the Copenhagen Comorbidity in HIV Infection (COCOMO) study, we measured 25 biomarkers of systemic inflammation, innate activation, endothelial dysfunction and T‐cell phenotypes on people with HIV with undetectable HIV RNA. Using principal component analysis followed by unsupervised hierarchical clustering, we grouped participants by immune‐inflammatory profiles. Participants were followed and the underlying causes of death were adjudicated from national patient records. Using cause‐specific Cox proportional hazards models, we assessed associations of immune‐inflammatory profiles with overall, cancer‐related, non‐HIV‐related and HIV‐related mortality.

**Results**: We included 651 people with HIV (Table 1). Identified immune‐inflammatory profiles included one characterized by high systemic inflammation, innate activation and immunosenescence (high inflammation cluster, 30.6% of participants), one by low immunosenescence (low senescence cluster, 29.8% of participants) and one by low systemic inflammation (reference cluster, 39.6% of participants). During follow‐up (median 10.7 years, total 6733 person‐years), 52 participants died, of whom 26 (50%) had cancer as the underlying cause of death, and 12 deaths (23%) were HIV‐related. The high inflammation cluster was associated with higher overall (HR 4.15 [95% CI: 2.02–8.49], *p* < 0.001) and cause‐specific cancer‐related (HR 4.48 [95% CI: 1.64–12.2], *p* = 0.003) and non‐HIV related mortality (HR 4.22 [95% CI: 1.68–10.7], *p* = 0.002) compared to the reference cluster. These associations remained significant after adjustment for age, sex, smoking status and current CD4+ T cell counts for both overall (aHR 2.82 [95% CI: 1.36–5.83], *p* = 0.005), cancer‐related (aHR 2.81 [95% CI: 1.01–7.83], *p* = 0.049) and non‐HIV‐related mortality (aHR 2.58 [95% CI: 1.01–6.62], *p* = 0.049). While the direction of associations was similar for HIV‐related mortality, the results did not reach statistical significance.

**Conclusions**: People with HIV who exhibited an inflammatory profile characterized by high systemic inflammation, innate activation and immunosenescence had higher overall, cancer‐related and non‐HIV‐related mortality, suggesting that integrative immune‐inflammatory profiling may have potential for clinical risk stratification. Identifying people with this immune‐inflammatory profile is warranted for targeted interventions.

**TABLE 1:**
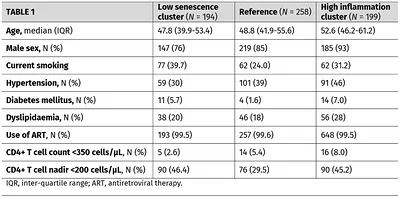
P371 | Participant characteristics (*N* = 651).

### P372 Antiretroviral Therapy Use in Ageing People With HIV: RESPOND Cohort Study

Olof Elvstam ^1,2^, Jaime Vera^3,4^, Ferdinand Wit^5^, Marc van der Valk^5,6^, Ole Kirk^7,8^, Lene Ryom Nielsen^7,8^, on behalf of RESPOND Study Group

^1^Department of Translational Medicine, Lund University, Malmö, Sweden; ^2^Department of Infectious Diseases, Växjö Hospital, Växjö, Sweden; ^3^Department of Global Health and Infection, Brighton and Sussex Medical School, Brighton, UK; ^4^University Hospital Sussex NHS Foundation Trust, Worthing, UK; ^5^AIDS Therapy Evaluation in the Netherlands (ATHENA) Cohort, HIV Monitoring Foundation, Amsterdam, Netherlands; ^6^Department of Infectious Diseases and Amsterdam Institute for Infection and Immunity, Amsterdam University Medical Centers, University of Amsterdam, Amsterdam, Netherlands; ^7^Centre of Excellence for Health, Immunity and Infections, Copenhagen University Hospital ‐ Rigshospitalet, Copenhagen, Denmark; ^8^Department of Clinical Medicine, Faculty of Health and Medical Sciences, University of Copenhagen, Copenhagen, Denmark

**Background**: While most people with HIV now survive into old age, data on antiretroviral therapy (ART) use across older age strata remain limited. We characterized the RESPOND cohort by age and studied distributions of characteristics, ART use and reasons for treatment discontinuation.

**Methods**: Participants were followed from the earliest of 01‐01‐2012 or enrolment until death, loss to follow‐up, or administrative censoring (31‐12‐2024). To assess whether underlying factors explained age‐group differences, we constructed two separate multivariable logistic regression models for tenofovir disoproxil fumarate (TDF) and two‐drug regimen use (any type) at end of follow‐up, adjusting for chronic kidney disease, hepatitis B, previous fracture, comorbidity index (numerically) and time since HIV diagnosis. We also assessed how ART use changed over time. Reason for discontinuation of last regimen (toxicity vs. other) was analysed adjusting for time since HIV diagnosis, prior virologic failure, sex/gender and comorbidity index.

**Results**: Of 44,369 persons included, 57% were ≥50, 26% ≥60 and 8% ≥70 years at end of follow‐up. Comorbidity and polypharmacy rates increased with age (Table 1). Dolutegravir and bictegravir were the most used anchor drugs across ages. TDF was more frequently used than tenofovir alafenamide before age 50, whereas the reverse was true for older individuals (Figure 1). This difference remained after adjustment (adjusted odds ratio for TDF ≥70 years vs. 18–25 years: 0.47 [95% confidence interval 0.40–0.55]). After adjustment, two‐drug regimens were more often used in older people (≥70 years: 2.25 [1.84–2.74]). Use of abacavir and boosters, respectively, decreased from 2012 to 2024, without clear differences between age strata and were used in 14% and 17% of people aged ≥70 years at end of follow‐up. Toxicity was a more common reason for last regimen discontinuation in both older men and women, also after adjustment (≥70 years: 1.78 [1.42–2.23]).

**Conclusions**: This study, including high numbers of individuals ≥60 and ≥70 years, provides a detailed overview of characteristics and ART use across ages. Use of TDF decreased and of two‐drug regimens increased with higher age. To support healthy ageing, ART optimization, including reduction of abacavir and booster use, deserves continued attention in older people with HIV.

**FIGURE 1:**
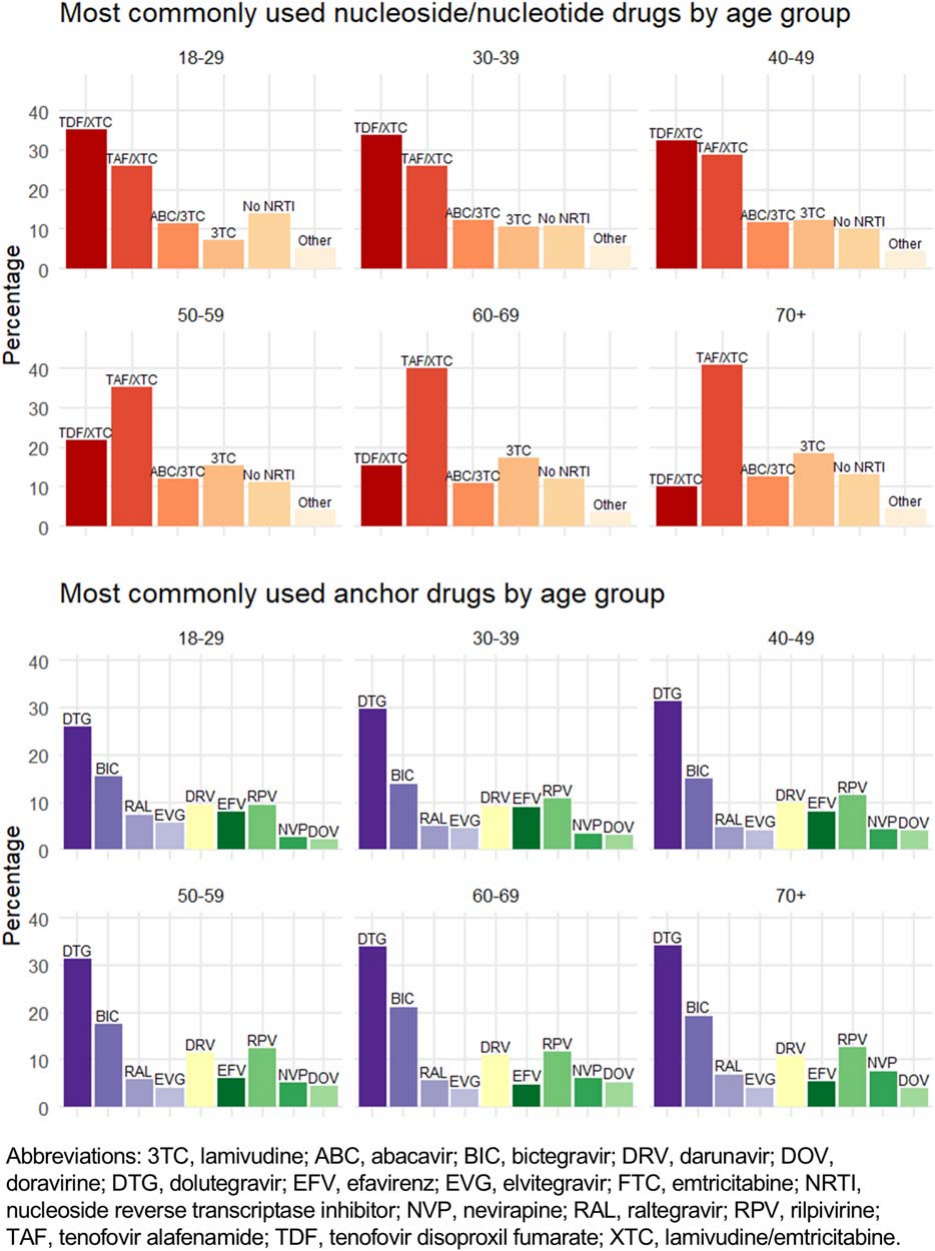
P372 | Antiretroviral use at end of follow‐up across age categories.

**TABLE 1:**
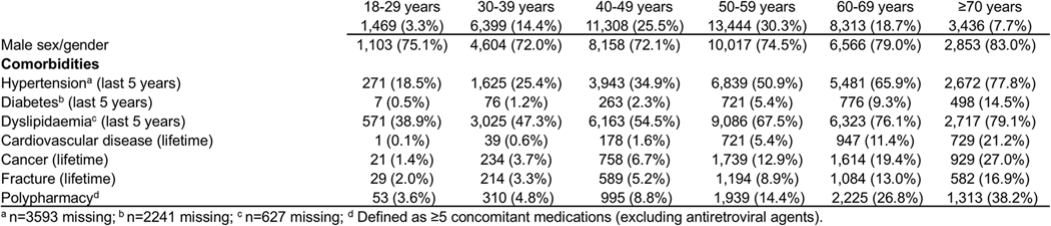
P372 | Characteristics of people with HIV across age categories summarized at end of follow‐up.

### P373 Beyond Viral Suppression: Loneliness and Financial Hardship Independently Predict High Unmet‐Needs Burden in Older People Living With HIV—A National Community‐Based Survey in Italy

Giovanni Guaraldi ^1^, Aurelio Castro^2^, Jovana Milić^1^, Michela Belli^1^, Giusi Giupponi^3^, Patrizia Perrone^3^, Sabrina Penon^4^, Salvio Cecere^5^, Chiara Solico^6^, Stefano Patrucco^7^, Aleksandra Moznic^8^, Luca Butini^9^, Paolo Meli^10^, Leonardo Del Negro^11^, Maria Stagnitta^12^, Daniela Masci^13^, Hermine Gbedo^14^, Filippo von Shlosser^15^, Mario Cascio^16^, Nicoletta Policek^8^, Alessandra Bianchi^17^, Colette Smith^18^

^1^University of Modena and Reggio Emilia, Modena, Italy; ^2^University of Milan Bicocca, Milano, Italy; ^3^Lila Onlus, Como, Italy; ^4^Lila Onlus, Milano, Italy; ^5^PLUS, Bologna, Italy; ^6^ASA Milano, Milano, Italy; ^7^Arcobaleno AIDS, Torino, Italy; ^8^NPS Italia, Milano, Italy; ^9^ANLAIS, Ancona, Italy; ^10^CICA, Bergamo, Italy; ^11^PLUS, Roma, Italy; ^12^CNCA, Roma, Italy; ^13^Fondazione Villa Marini, Roma, Italy; ^14^CDCP, Bologna, Italy; ^15^Nadir, Roma, Italy; ^16^NPS Italia, Palermo, Israel; ^17^ASA, Milano, Italy; ^18^UCL Institute for Global Health, London, UK

**Background**: More than half of people living with HIV (PLWH) in high‐income settings are now aged ≥50 years. Despite near‐universal virological suppression, healthy ageing in this population remains constrained by multimorbidity, loneliness, financial insecurity and healthcare fragmentation. Evidence characterizing the multidimensional unmet needs of older PLWH and their determinants remains limited, particularly in Southern European settings.

**Methods**: We conducted a national community‐based cross‐sectional survey of PLWH aged ≥50 years in Italy (February 2026), co‐produced with 12 HIV community organizations. The questionnaire assessed 34 services across five healthy ageing domains: psychological wellbeing, relational wellbeing, social vulnerability, healthy ageing and prevention, and healthcare navigation. Unmet need was defined as inability to access a needed service, lack of service awareness, or failure to receive support despite perceived need. High unmet‐needs burden was defined as ≥4 unmet needs, representing the highest quartile. Independent predictors were identified using multivariable logistic regression.

**Results**: Of 478 participants (mean age 60.7 years; 67.6% men; 88.1% with undetectable viral load), 91.8% reported need in ≥1 healthcare navigation service, of whom 50.6% had ≥1 unmet need. Unmet need prevalence among those with reported need were highest in healthy ageing and prevention (68.5%), relational wellbeing (60.3%) and psychological wellbeing (45.9%). The leading individual services unmet needs were stress management (24.7%), sexual health support (23.6%), nutritional counselling for osteosarcopenia (23.0%), psychotherapy (20.7%) and support for loneliness and isolation (20.5%). Overall, 35.2% (*n* = 168) had ≥4 unmet needs. In multivariable analysis, loneliness score > 2 (adjusted odds ratio [aOR] 3.33, 95% confidence interval [CI] 2.12–5.22; *p* < 0.0001), financial hardship (aOR 2.04; 95% CI: 1.22, 3.57; *p* = 0.038) and self‐rated health (aOR 1.14 per 10‐units lower; 95% CI: 1.01, 1.28; *p* = 0.036) were independently associated with high unmet‐needs burden. Age and HIV duration did not predict high burden (Figure 1).

**Conclusions**: Multidimensional unmet needs affecting psychological, relational and healthy ageing domains are highly prevalent among older PLWH in Italy. These findings support reorientation of HIV care towards integrated, person‐centred models addressing social determinants and functional wellbeing alongside virological monitoring.

Filled circles indicate *p* < 0.05; open circles indicate non‐significant associations. The reference line is placed at aOR = 1. Scale is logarithmic. Multivariable logistic regression adjusted for all variables shown. Source: “HIV and AGEING – la tua voce conta” community survey, Italy, 2026.

**FIGURE 1:**
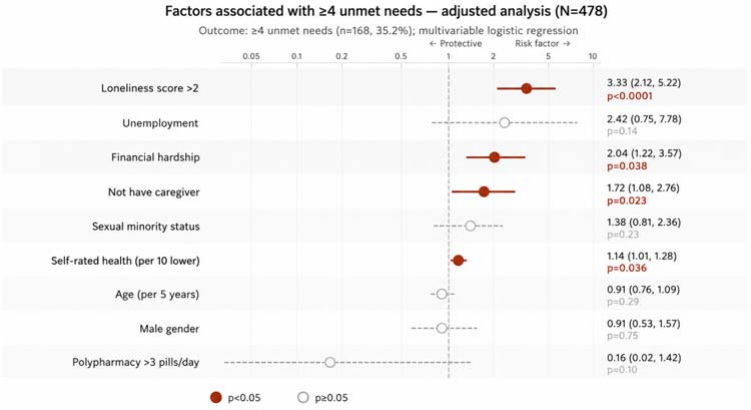
P373 | Adjusted odds ratios (aOR) and 95% confidence intervals for factors independently associated with high unmet‐needs burden (≥4 unmet needs) among people living with HIV aged ≥50 years in Italy (*N* = 478; outcome *N* = 168, 35.2%).

### P374 Age Is Independently Associated With Incident Low‐Level Viraemia During Suppressive Antiretroviral Therapy: Data From the ICONA Cohort

Anna Maria Geretti ^1,2^, Roberta Gagliardini^3^, Davide Checchi^4^, Camilla Muccini^5^, Alessandra Vergori^3^, Valentina Mazzotta^3^, Giuseppe Vittorio De Socio^6,7^, Alessandro Tavelli^8^, Giulia C. Marchetti^9^, Loredana Sarmati^2^, Antonella D'Arminio Monforte^9^, Cristina Mussini^10^, Andrea Antinori^3^, Alessandro Cozzi‐Lepri^11^, on behalf of ICONA Cohort Study Group

^1^Royal Free London NHS Trust, London, UK; ^2^University of Rome Tor Vergata, Rome, Italy; ^3^National Institute for Infectious Diseases Lazzaro Spallanzani‐IRCCS, Rome, Italy; ^4^Policlinico Tor Vergata, Rome, Italy; ^5^San Raffaele Scientific Institute‐IRCCS, Milan, Italy; ^6^University of Perugia, Perugia, Italy; ^7^Santa Maria della Misericordia Hospital, Perugia, Italy; ^8^Fondazione ICONA, Milan, Italy; ^9^University of Milan, Milan, Italy; ^10^University of Modena & Reggio Emilia, Modena, Italy; ^11^University College London, London, UK

**Background**: New‐onset low‐level viraemia (LLV) during suppressive ART may reflect viral replication or release from long‐lived reservoirs of replication‐competent or defective virus. Age‐related changes in immune function could influence the occurrence of LLV, but data on this relationship are limited. We examined the association between age and incident LLV in the Italian ICONA cohort.

**Methods**: We analysed 13,432 adults in ICONA who achieved confirmed virological suppression (HIV RNA ≤50 copies/mL) on ART. The primary outcome was incident LLV, defined as two consecutive HIV RNA measurements between 51 and 199 copies/mL. Kaplan−Meier methods were used to estimate cumulative incidence of LLV. Cox proportional hazards models were used to evaluate the association between age and incident LLV. To account for imbalances within age groups (Table 1), models adjusted for sex at birth, country of birth, calendar year and either duration of ART exposure or time since HIV diagnosis. Interaction between age and nadir CD4 category was formally tested.

**Results**: Among 13,432 participants, 489 (3.6%) developed LLV during follow‐up, with higher incidence across all age strata above 30 years (Figure 1). Compared with individuals aged 18–30 years, adjusted hazard ratios (aHRs) for LLV were 2.07 (95% CI: 1.39–3.08) for ages 31–40 years, 2.42 (1.67–3.50) for ages 41–45 years, 2.51 (1.69–3.73) for ages 46–55 years and 1.74 (1.19–2.53) for ages 56 years. Alternative adjustment for time since HIV diagnosis yielded similar results. Among participants with LLV, 39/489 (8.0%) subsequently experienced virological failure (confirmed HIV RNA ≥200 copies/mL); age associations remained similar after excluding these individuals from the analysis. A significant interaction was observed between age and nadir CD4 category (*p* = 0.003); the magnitude of the effect of age on LLV risk was larger among participants with nadir CD4 > 200 cells/mm^3^.

**Conclusions**: During suppressive ART, older age showed an association with incident LLV, with higher risk across all age groups above 30 years. The effect was not explained by other analysed factors. Effect measure modification by nadir CD4 category suggests that severe historical immune damage may attenuate age‐related differences in LLV risk, whereas these differences are more apparent among individuals with higher nadir CD4 counts.

**TABLE 1:**
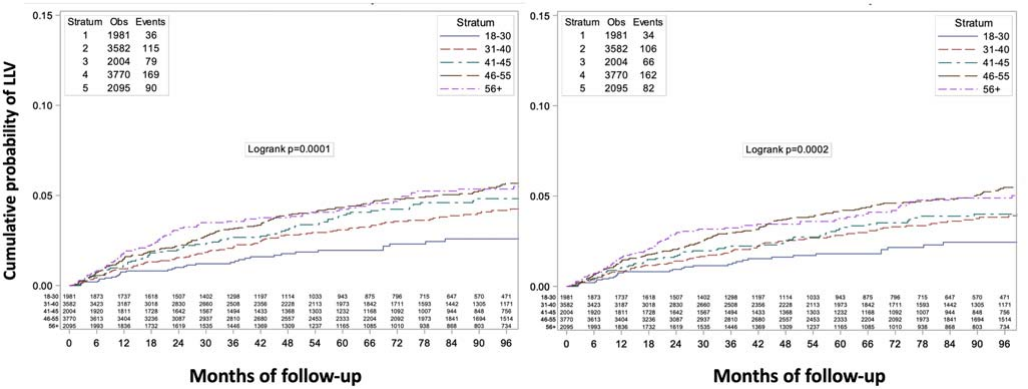
P374 | Cumulative incidence of confirmed low‐level viraemia according to age group. Left: primary analysis. Right: sensitivity analysis excluding LLV events followed by virological failure.

**FIGURE 1:**
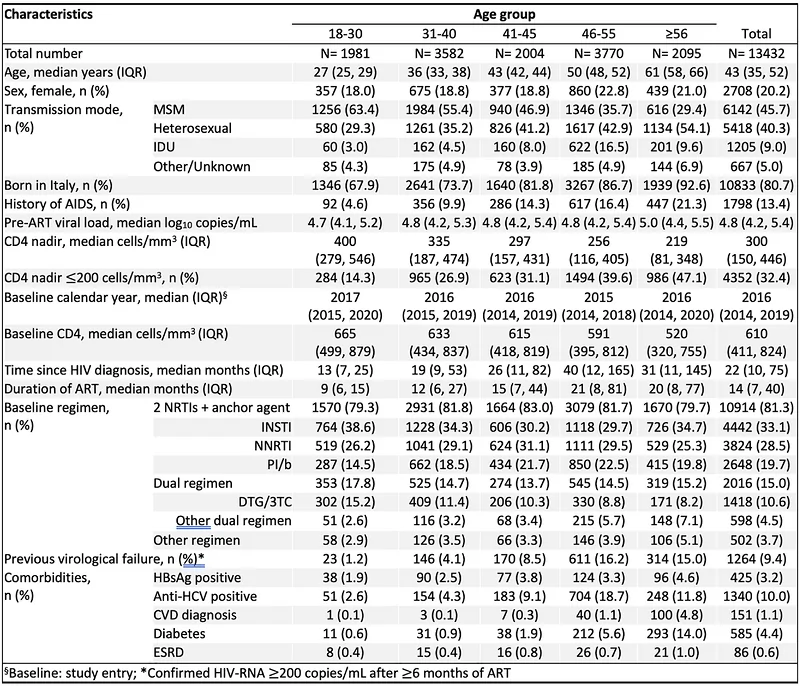
P374 | Characteristics of the study population.

### P375 The Impact of Semaglutide on Weight Loss and Inflammation in People With HIV: An Observational Prospective Study in Greek Population

Vasileios Petrakis, Periklis Panagopoulos, Petros Rafailidis, Maria Panopoulou, Dimitrios Papazoglou

Democritus University of Thrace, Alexandroupolis, Greece

**Background**: The existing literature on the effects of semaglutide in people with HIV (PWH) and diabetes mellitus remains limited. In this study, the effects of semaglutide on weight control and inflammation in PWH were evaluated. This is a prospective observational study that included PWH with type 2 diabetes mellitus monitored at the HIV Unit of the University General Hospital of Alexandroupolis and a matched control group of non‐HIV individuals.

**Methods**: Demographic, anthropometric and clinical characteristics, including HIV‐related data and comorbidities, were reported. All participants received semaglutide with a gradual dose increase to 1 mg once weekly. Body mass index (BMI), glycosylated haemoglobin (HbA1c) and inflammatory markers (IL‐6, TNF, hsCRP, sCD14, CD4/CD8 ratio) were recorded at baseline and at 6, 12, 18 and 24 months. Fifty participants (PWH: *n* = 25; non‐HIV: *n* = 25) were included.

**Results**: At baseline, the mean BMI was 35.2 ± 8.0 kg/m^2^ for PWH and 36.1 ± 6.0 kg/m^2^ for non‐HIV controls. Semaglutide treatment resulted in significant and sustained weight loss in both groups (*p* < 0.001). At 24 months, the median weight loss was −14.6 kg in the PWH group and −18.8 kg in the non‐HIV group for those with a baseline BMI > 35 kg/m^2^. Glycaemic control also improved significantly, with mean HbA1c decreasing from 7.7% ± 1.23 to 5.2% ± 1.02 in PWH (*p* < 0.001), and from 7.9% ± 1.16 to 5.6% ± 1.21 in non‐HIV controls (*p* < 0.001). Significant reductions were observed in hsCRP and sCD14 levels in both cohorts. A unique finding was the significant increase in the CD4/CD8 ratio in the PWH group, from a mean baseline of 0.54 ± 0.12 to 0.83 ± 0.14 at 24 months (*p* < 0.001), a change not seen in the non‐HIV controls.

**Conclusions**: Semaglutide appears to be an effective and safe option for weight reduction and inflammation control in PWH. Further studies with a larger number of patients are necessary to substantiate these findings.

### P376 Association of Anti‐Müllerian Hormone With Oxidative Stress, Microbial Translocation and Monocyte Activation Following cART Initiation in Women With HIV

Valeria Bono^1^, Valentina Sala^1^, Mariacristina Poliseno^2^, Alessandro Tavelli^3^, Hanieh Sadeghi^3^, Eugenia Quiros‐Roldan^4^, Matteo Augello^1^, Giuseppe Vittorio De Socio^5^, Carmen Santoro^2^, Claudia Maestripieri^6^, Cristina Mussini^7^, Miriam Lichtner^8^, Camilla Tincati^1^, Annalisa Saracino^2^, Giulia C. Marchetti ^1^, on behalf of ICONA Cohort Study Group

^1^Clinic of Infectious Diseases and Tropical Medicine, San Paolo Hospital, ASST Santi Paolo e Carlo, Department of Health Sciences, University of Milan, Milan, Italy; ^2^Clinic of Infectious Diseases, Department of Precision and Regenerative Medicine and Jonian Area (DiMePreJ), A.O.U.C. Policlinic di Bari, Bari, Italy; ^3^ICONA Foundation, Milan, Italy; ^4^Department of Clinical and Experimental Sciences, Unit of Infectious and Tropical Diseases, University of Brescia and ASST Spedali Civili di Brescia, Brescia, Italy; ^5^Unit of Infectious Diseases, Santa Maria Hospital, University of Perugia, Perugia, Italy; ^6^Unity of Microbiology and Biobank, INMI, Rome, Italy; ^7^Department of Infectious Diseases, Azienda Ospedaliero‐Universitaria di Modena, Policlinico di Modena, University of Modena and Reggio Emilia, Modena, Italy; ^8^Clinical Infectious Diseases, Sant'Andrea Hospital, Sapienza University of Rome, Rome, Italy

**Background**: Anti‐Müllerian hormone (AMH), a marker of ovarian reserve, has emerged as a candidate indicator of biological ageing. Its relationship with oxidative stress, microbial translocation and monocyte activation in women with HIV (WWH) remains unclear. We investigated the associations between AMH and these pathways before and after virologic suppression following combination antiretroviral therapy (cART).

**Methods**: We conducted a longitudinal study including 103WWH (35–55 years) evaluated before cART initiation (T0) and 6–18 months after virologic suppression (T1). Plasma AMH, lipopolysaccharide‐binding protein (LBP), soluble CD14 (sCD14) and reactive oxygen species modulator 1 (ROMO1) were measured by ELISA. AMH was analysed as both a continuous and categorical variable (≤0.2 vs. > 0.2 ng/mL). Multivariable linear models fitted to assess associations of AMH and biomarkers. Adjusted logistic models to evaluate the association between baseline AMH strata and CD4 count. Longitudinal changes were evaluated using Wilcoxon signed‐rank tests.

**Results**: At baseline, 40 women (38.8%) had AMH ≤0.2 ng/mL and 63 (61.2%) had AMH > 0.2 ng/mL (Table 1). No differences in LBP, sCD14 or ROMO1 were observed between AMH strata at either timepoint. Lower baseline AMH was associated with higher ROMO1 at both T0 (β = −0.111 ng/mL, 95% CI: −0.206, −0.016; *p* = 0.023) and T1 (β = −0.132 ng/mL, 95% CI: −0.248, −0.016; *p* = 0.026) after adjustment for age, baseline CD4 cell count and HIV RNA. After further adjustment for BMI, smoking status and HIV acquisition risk, the association remained significant at T0 (β = −0.103 ng/mL, *p* = 0.041) and persisted as a trend at T1 (β = −0.114 ng/mL, *p* = 0.062). Overall, cART reduced LBP (5.1 vs. 4.8 µg/mL, *p* = 0.01, Figure 1A) and sCD14 (1.2 vs. 1.1 ng/mL, *p* = 0.02, Figure 1B), with no differences by AMH status. After adjustment for age, T0 HIV‐RNA and HIV acquisition risk, baseline CD4 < 200 cells/µL was associated with low ovarian reserve (vs. CD4 > 500 aOR 7.44, 95% CI: 1.04–52.86; *p* = 0.045).

**Conclusions**: Lower AMH was independently associated with increased oxidative stress before cART initiation and after virologic suppression, suggesting a link between reduced ovarian reserve and systemic redox imbalance in WWH. cART reduced microbial translocation and monocyte activation, while severe baseline immunodeficiency was independently associated with low ovarian reserve. These findings support further investigation of oxidative stress and biological ageing in WWH using AMH as a biomarker.

**FIGURE 1:**
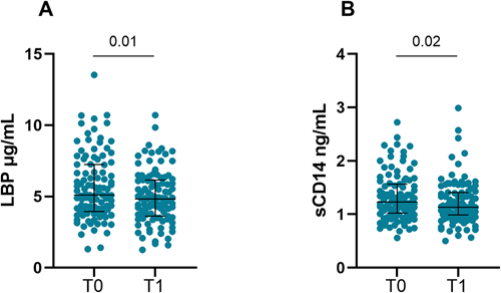
P376 | Lipopolysaccharide‐binding protein (LBP; A) and soluble CD14 (sCD14; B) before the start of combination antiretroviral therapy (cART) (T0); and 6–18 months after virologic suppression in women with HIV.

**TABLE 1:**
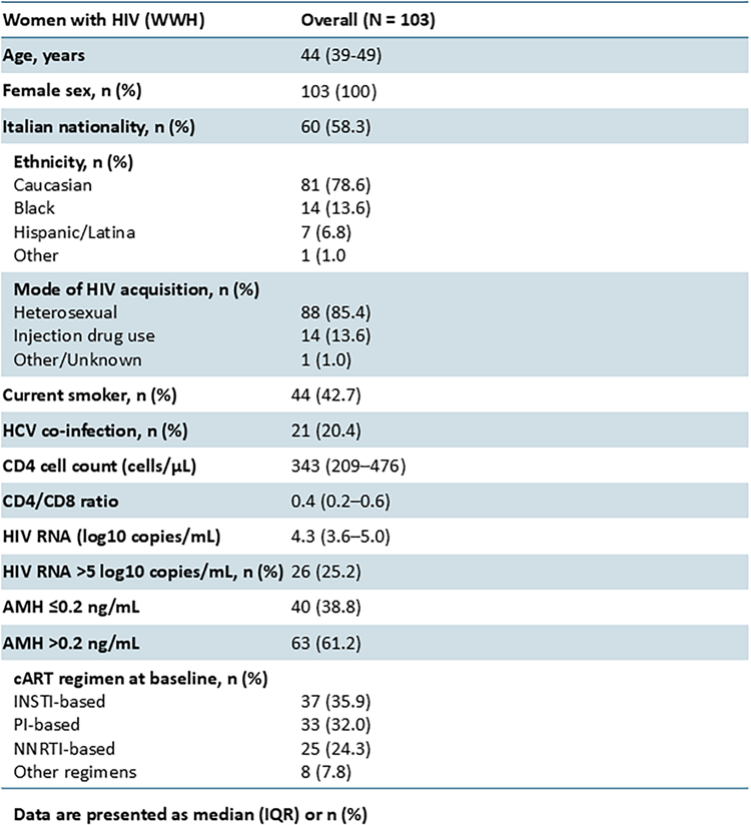
P376 | Baseline demographic and clinical characteristics of the study population.

### P377 Polypharmacy, Anticholinergic Burden (ACB) and Potential Drug−Drug Interactions (PDDIs) in Older People Living With HIV (PLWH) Who Attended an HIV Outpatient Clinic, Including a Specialist Joint HIV and Geriatric Medicine Service (Platinum Clinic)

Arlene Heekin ^1^, Miriam Moriarty^1^, Gail Melanophy^2^, David Robinson^3^, Emma Devitt^1^

^1^Department of Genitourinary Medicine and Infectious Diseases, St James's Hospital, Dublin, Ireland; ^2^Pharmacy Department, St James's Hospital, Dublin, Ireland; ^3^Department of Geriatric Medicine, St James's Hospital, Dublin, Ireland

**Background**: With advancement in HIV care, PLWH are living to normal life expectancy resulting in an increased prevalence of co‐morbidities and concomitant medication use. Consequently, medication‐related risks are becoming more frequent as the population ages. This study aimed to evaluate polypharmacy, ACB and PDDIs among PLWH ≥50 years.

**Methods**: This prospective cohort study was conducted over a 2‐month period and included PLWH aged ≥50 years who attended HIV and Platinum outpatient clinics. A specialist pharmacist assessed polypharmacy, ACB and PDDIs with antiretroviral therapy (ART). PDDIs were assessed using the Liverpool HIV Drug Interactions Checker. Interactions were classified as weak (yellow), moderate (amber) and red (severe). Polypharmacy was defined as use of ≥5 non‐ART medications and hyperpolypharmacy was defined as ≥10 non‐ART medications.

**Results**: Data was gathered from a total of 207 individuals. The median age was 57 years. Polypharmacy was reported in 41.5%, with hyperpolypharmacy in 11.1%, see Table 1. In relation to opportunistic infections and medication burden, ten patients (4.8%) received *Pneumocystis jirovecii* Pneumonia prophylaxis. Eighty‐three patients (40%) experienced at least one PDDI, resulting in 132 identified interactions. Most interactions were classified as moderate (80%, *n* = 106/132). The most frequently implicated co‐medications were mineral supplements, drugs used in diabetes management and vitamins, see Figure 1. Among ART classes, integrase strand transfer inhibitors (INSTIs) were most commonly associated with moderate‐severe PDDIs (65.4%, *n* = 70/107), followed by boosted protease inhibitors (PIs) (15.8%, *n* = 17/107). ACB with a score of ≥3 was recorded in 14.5% of the study population.

**Conclusions**: These findings demonstrate polypharmacy and PDDIs are frequent among PLWH ≥50 years. This may contribute to adverse clinical outcomes. With increased polypharmacy, assessing ACB is also important as it can contribute to frailty, cognitive impairment and falls. Antiretroviral stewardship programmes (ARVSPs) provide an effective strategy to enhance medication safety and optimize therapeutic management. Pharmacists play a pivotal role in ARVSPs through medication reconciliation and identification of PDDIs. Pharmacist‐led medication reviews may effectively identify, prevent and mitigate medication related risks in this ageing HIV population.

**FIGURE 1:**
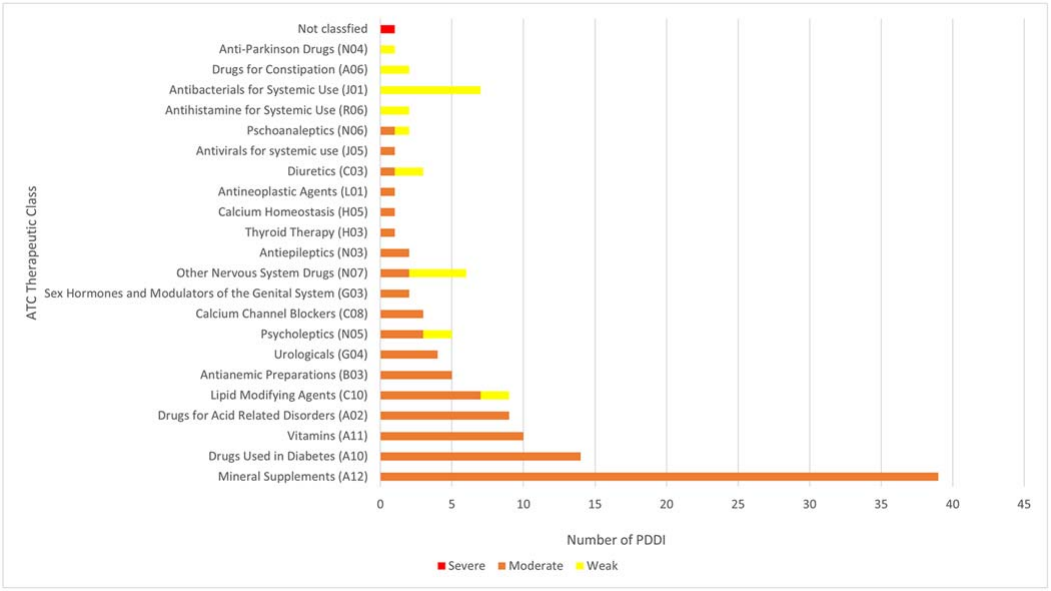
P377 | Non‐ARVs associated with severe, moderate and weak potential drug−drug interactions.

**TABLE 1:**
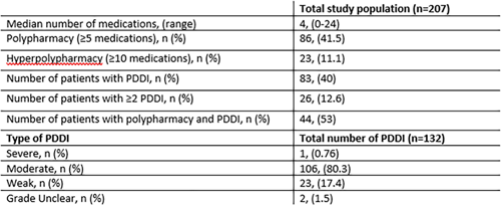
P377 | Polypharmacy and frequency of PDDI.

### P378 Sarcopenia, Frailty and Biological Age in People Living With HIV

Jose L. Casado, Laura Luna, Isabel Izuzquiza, Ana Moreno Zamora, Cristina Fernandez‐Chica, Alejandro Vallejo

Ramon y Cajal Hospital, Madrid, Spain

**Background**: Low muscle mass has been observed in people living with HIV (PLWH). The aim of this study was to determine the incidence of sarcopenia using EWGSOP2 (European Working Group on Sarcopenia in Older People 2) operational definition, its association with frailty and to establish a chronological age associated with sarcopenia and functional limitations.

**Methods**: Prospective cohort study. Appendicular skeletal muscle was estimated using dual X‐ray absorptiometry (DXA). SARC‐F questionnaire (strength, assistance with walking, rise from a chair, climb stairs, falls) was used as muscle function measure, whereas Frailty scale was determined in each individual case. Spearman correlation coefficient was calculated and ROC curves were used to determine chronological threshold age value associated with sarcopenia/limitation.

**Results**: A total of 286 PLWH (19% female) were included. Median age was 52 years, median duration of HIV was 19 years (IQR 13–30), and median nadir CD4+ count was 230 cells/ µL (IQR 113–369). Based on DXA, the prevalence of reduced muscle quantity (below EWGSOP2 cutoff) was 38% in men and 36% in women. Appendicular skeletal muscle correlated significantly with age (rho −0.256; *p* < 0.01), nadir CD4+ count (rho 0.187; *p* = 0.002) and duration of HIV (rho −0.249; *p* < 0.01). Muscle mass showed strong agreement with SARC‐F and frailty scores (*p* < 0.01). However, according to the EWGSOP2 definition, only 3% met criteria for sarcopenia, associated with reduced functionality. Low skeletal muscle determination without evaluation of SARC‐F or frailty scale showed limited ability to define a biological age cut‐off (AUC 0.57). In contrast, combining low muscle mass, SARC‐F and frailty improved discrimination, identifying ≥60 years as a meaningful threshold for functional limitation in this heavily treated population (AUC 0.76; *p* = 0.003; sensitivity 68%, specificity 75%).

**Conclusions**: Although reduced muscle quantity is frequent in PLWH, sarcopenia, when functional measures are included, remains relatively infrequent. The combination of DXA‐derived appendicular skeletal muscle mass with functional assessments and frailty scale may help identifying a biological age associated with clinically relevant functional limitations in this population.

### P379 Abstract Withdrawn

### P380 Never Too Old to Recover: A Longitudinal Analysis of People With HIV Aged ≥75 Years in the ICONA Cohort

Camilla Muccini ^1^, Andrea De Vito^2^, Alessandro Tavelli^3,4^, Andrea Santoro^5^, Giovanni Festa^6^, Eugenia Quiros‐Roldan^7^, Cecilia Costa^8^, Andrea Calcagno^9^, Giulia C. Marchetti^5^, Antonella Cingolani^10^, Enrico Girardi^11^, Antonella D'Arminio Monforte^3^, Giovanni Guaraldi^12^, Giordano Madeddu^2^, on behalf of ICONA Cohort Study Group

^1^Department of Infectious Diseases, ASST Grande Ospedale Metropolitano Niguarda, Milan, Italy; ^2^Unit of Infectious Disease, Department of Medicine, Surgery and Pharmacy, University of Sassari, Sassari, Italy; ^3^ICONA Foundation, Milan, Italy; ^4^National PhD Programme in One Health approaches to infectious diseases and life science research, Department of Public Health, Experimental and Forensic Medicine, University of Pavia, Pavia, Italy; ^5^Clinic of Infectious Diseases, ASST Santi Paolo e Carlo, Department of Health Sciences, University of Milan, Milan, Italy; ^6^Vita‐Salute San Raffaele University, Milan, Italy; ^7^Department of Clinical and Experimental Sciences, Unit of Infectious and Tropical Diseases, University of Brescia and ASST Spedali Civili di Brescia, Brescia, Italy; ^8^Infectious Diseases Department, SOC 1, USLCENTROFIRENZE, Santa Maria Annunziata Hospital, Florence, Italy; ^9^Department of Translational Medicine, University of Eastern Piedmont, Novara, Novara, Italy; ^10^UOC Malattie Infettive, Fondazione Policlinico Universitario Agostino Gemelli IRCCS, Dipartimento di Sicurezza e Bioetica, Università Cattolica del Sacro Cuore, Rome, Italy; ^11^Scientific Direction, National Institute for Infectious Diseases, Lazzaro Spallanzani IRCCS, Rome, Italy; ^12^Department of Infectious Diseases, Azienda Ospedaliero‐Universitaria di Modena, Policlinico di Modena, University of Modena and Reggio Emilia, Modena, Italy

**Background**: People with HIV (PWH) aged ≥75 years represent a growing but poorly characterized population. The study aimed to describe virological outcomes and immunological trajectories in PWH ≥75 years.

**Methods**: Longitudinal study conducted within Italian ICONA cohort. Index date was defined as the date PWH turned 75 or date of enrolment from ART‐naïve if already ≥75. ART‐experienced and ART‐naïve PWH were analysed separately. Study endpoints: (i) virological failure (VF) among ART‐experienced virologically suppressed PWH; (ii) virological suppression (VS) among ART‐experienced and ART‐naïve; (iii) longitudinal changes in CD4 count and CD4/CD8 ratio among ART‐experienced and ART‐naïve. Immunological trajectories were estimated using linear mixed‐effects models with random intercepts and slopes. Time‐to‐event outcomes were analysed using cumulative incidence functions (CIFs), accounting for death as a competing event.

**Results**: We included 424 PWH (334 ART‐experienced and 90 ART‐naïve, Table 1). The proportion of PWH aged ≥75 years in follow‐up in the ICONA cohort increased from 0.08% in 1997–2000 to 2.9% in 2021–2025 (*p*‐for‐trend < 0.001). Median follow‐up after index date was 3.5 and 3.0 years for ART‐experienced and ART‐naïve PWH, respectively. Among ART‐experienced PWH, 93.7% had HIV‐RNA <50 copies/mL at index date and 94.6% at last visit (*p* = 0.690); 17 VF occurred (5.4%) with a CIF at 5‐year of 5.4% (95% CI: 2.5–8.2, Figure 1A). Despite advanced age, CD4 continued to rise significantly (+7.1 cells/mcL/year, *p* = 0.008) post‐index, with recovery rate progressively attenuated with increased ART duration pre‐index (*p* < 0.001). Nevertheless, CD4 gains persisted in those who started ART ≤11 years earlier (Figure 1B); CD4/CD8 also improved progressively (+0.02/year, *p* < 0.001) after index, for up to 15 years after ART initiation, although the rate of increase declined over‐time (*p* = 0.040) (Figure 1C). Among ART‐naïve PWH, 94.9% achieved VS after a median time of 4.6 months; the CIF of VS by 12 months was 90.3% (95% CI: 83.6–97.1, Figure 1D). CD4 and CD4/CD8 gains in ART‐naïve individuals reached +164 (95% CI: 132–196) cells/mcL and +0.25 (95% CI: 0.13–0.38) at 12 months (*p* < 0.001), respectively, and were sustained throughout follow‐up (Figure 1E,F).

**Conclusions**: PWH aged ≥75 years represent a growing population and demonstrated favourable virological outcomes under ART, regardless of treatment experience. ART‐experienced PWH showed sustained immune recovery persisting for years after ART initiation.

**FIGURE 1:**
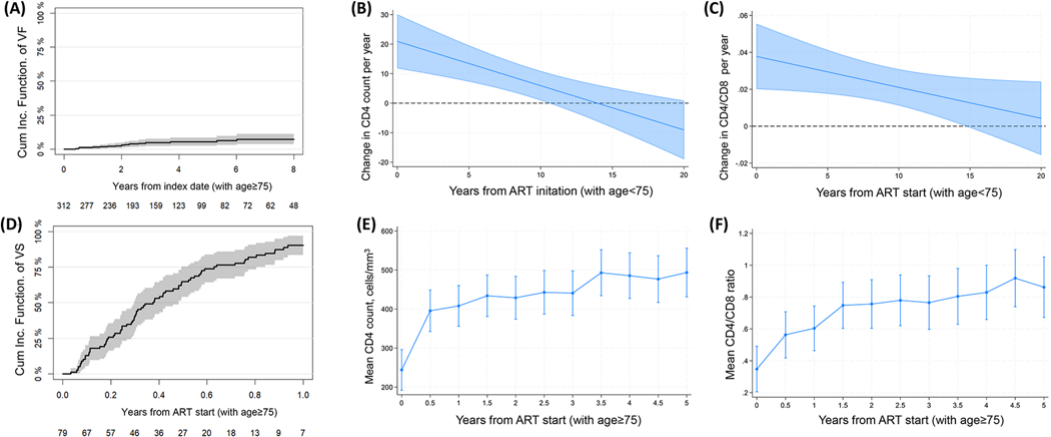
P380 | (A) Cumulative incidence of virological failure (2 HIV‐RNA greater than 50 copies/mL or single greater than 1000 followed by ART‐change) among ART‐experienced PWH ≥75 years with HIV‐RNA greater than 50 copies/mL at index. (B, C) Estimated annual change in CD4 cell count and CD4/CD8 ratio according to time since ART initiation among ART‐experienced PWH ≥75 years. (D) Cumulative incidence of achieving viral suppression (HIV‐RNA greater than 50 copies/mL) among ART‐naïve PWH ≥75 years after ART initiation. (E, F) Mean CD4 cell count and CD4/CD8 ratio trajectories following ART initiation among ART‐naïve PWH ≥75 years.

**TABLE 1:**
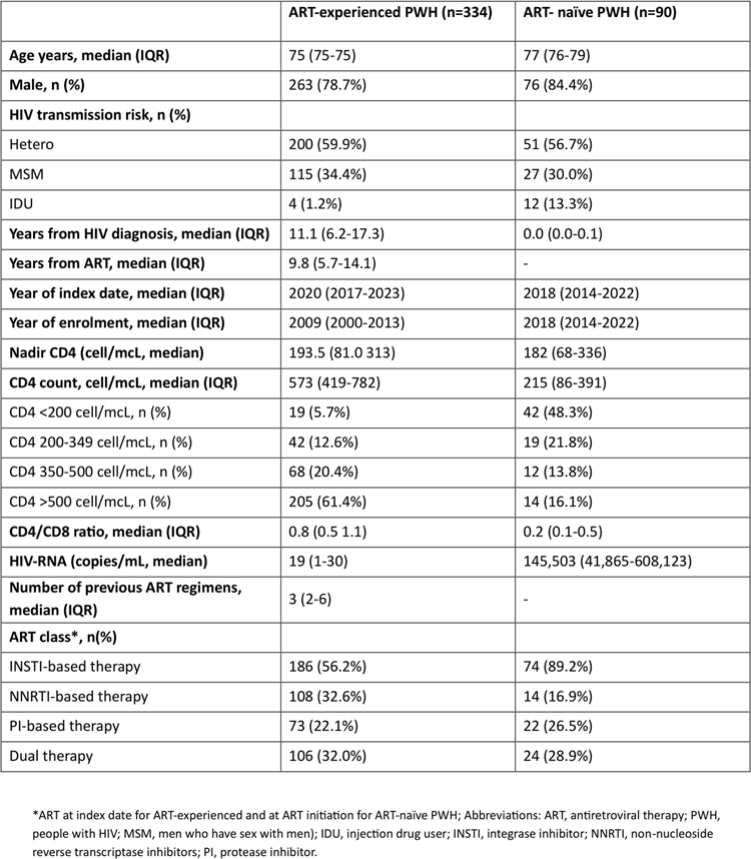
P380 | Participants’ characteristics at index date.

### P381 Outcomes and Comorbidity Burden Among People Diagnosed With HIV at Age ≥60 Years: The German ClinSurv Cohort

Smaranda Gliga ^1^, Uwe Koppe^2^, Christian Kollan^2^, Andrea Sailer^2^, Guido Schäfer^3^, Matthias Müller^4^, Peter Buggisch^5^, Dirk Schürmann^6^, Stefan Esser^7^, Alexander Killer^1^, Tom Luedde^1^, Barbara Gusenheimer‐Bartmeyer^2^, Björn‐Erik Ole Jensen^1^, on behalf of ClinSurv Study Group

^1^Department of Gastroenterology, Hepatology and Infectious Diseases, University Hospital Düsseldorf, Medical Faculty, Heinrich‐Heine‐University Düsseldorf, Dusseldorf, Germany; ^2^Department of Infectious Disease Epidemiology, Robert Koch Institute, Berlin, Germany; ^3^ICH Hamburg‐Stendal, Hamburg, Germany; ^4^Division of Infectious Diseases, Department of Medicine II, Faculty of Medicine, Medical Center, University of Freiburg, Freiburg, Germany; ^5^ifi‐Institut für interdisziplinäre Medizin, Hamburg, Germany; ^6^Charité, Universitätsmedizin Berlin, Berlin, Germany; ^7^Department of Dermatology and Venereology, University Hospital Essen, University Duisburg‐Essen, Essen, Germany

**Background**: New HIV diagnoses among older adults are rising. Whether people diagnosed at age ≥60 years have poorer treatment outcomes remains unclear. We compared HIV outcomes and comorbidity burden by age at diagnosis.

**Methods**: We used data from the German ClinSurv HIV cohort from 2010 to 2025 to compare adults diagnosed with HIV at age ≥60 years with those diagnosed at age 40–59 years. Time from antiretroviral therapy (ART) initiation to virological suppression (HIV‐1 RNA <50 copies/mL) and CD4 recovery to ≥500 cells/µL (among those with baseline CD4 < 500), and time from suppression to virological failure (a single HIV‐1 RNA measurement > 200 copies/mL), were analysed using Cox models adjusted for calendar period, region of origin, transmission group and sex. Comorbidity burden at diagnosis was compared descriptively.

**Results**: Of 1334 adults, 170 (12.7%) were diagnosed with HIV at age ≥60 years. Baseline CD4 counts (median 217 vs. 245 cells/µL) and HIV‐1 RNA levels (median 5.07 vs. 4.88 log_10_ copies/mL) were broadly similar between age groups, whereas cardiovascular disease, diabetes, hypertension and chronic obstructive pulmonary disease were substantially more common among those diagnosed at age ≥60 years (Table 1). The analysis of virological suppression included 1251 participants with an eligible ART initiation date and at least one follow‐up HIV‐1 RNA measurement (≥60 years, *n* = 158; 40–59 years, *n* = 1093). After adjustment, time to virological suppression did not differ between age groups (adjusted hazard ratio [aHR] 1.09, 95% confidence interval [CI] 0.90–1.31); the Kaplan–Meier median time to suppression was 175 versus 189 days, and the cumulative probability of suppression by 12 months was 73% versus 67% (Figure 1). CD4 recovery was also similar (aHR 0.94, 95% CI: 0.73–1.21). Virological failure occurred numerically less frequently among participants diagnosed at age ≥60 years, with 15 versus 163 events and incidence rates of 1.89 versus 2.90 per 100 person‐years (aHR 0.66, 95% CI: 0.39–1.12).

**Conclusions**: Adults diagnosed with HIV at age ≥60 years achieved virological and immunological responses comparable to those diagnosed at age 40–59 years, despite a substantially higher comorbidity burden at diagnosis. Cardiovascular and metabolic assessment should be embedded in HIV care from diagnosis in older adults.

**FIGURE 1:**
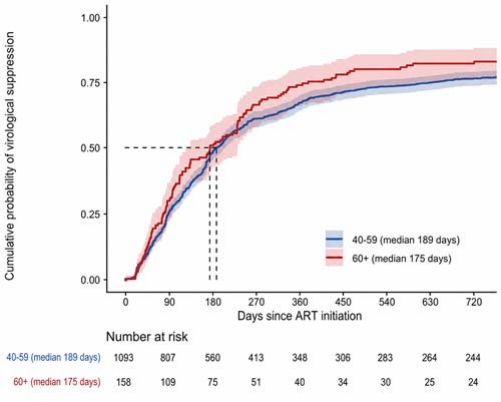
P381 | Time to virological suppression by age at first HIV diagnosis. Kaplan–Meier curves showing time from ART initiation to first virological suppression, defined as HIV‐1 RNA <50 copies/mL, in adults first diagnosed with HIV at age ≥60 years compared with those diagnosed at age 40–59 years. The analysis is restricted to the 1251 participants with an eligible ART initiation date and at least one follow‐up viral‐load measurement (≥60 years, *n* = 158; 40–59 years, *n* = 1093); 83 of the 1334 cohort members lacked these data and could not contribute to this time‐to‐event analysis. Shaded areas indicate 95% confidence intervals; the row below the plot shows the number at risk in each group at each time point.

**TABLE 1:**
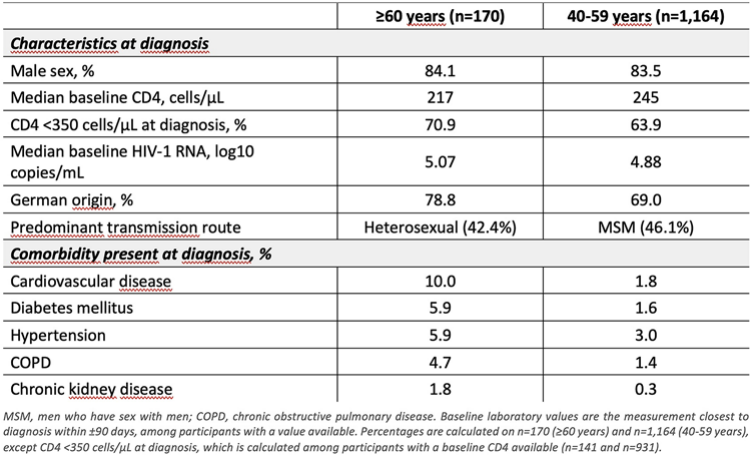
P381 | Baseline characteristics and comorbidity burden at diagnosis by age at first HIV diagnosis.

### P382 Elderly People Living With HIV on Successful Modern Treatment Have Increased Levels of Immunosenescence, With Men Who Have Sex With Men Displaying the Highest Level

Matteo Vassallo ^1^, Jacques Durant^2^, Sami Addou^3^, Michel Ticchioni^3^, Roxane Fabre^4^, David Chirio^2^, Sara Ferrando^2^, Alissa Naqvi^2^, Eric Cua^2^, Leslie Ameil^2^, Maeva Godemert^5^, Edouard Tuaillon^6^, Amandine Pisoni^6^, Anaelle Bisch^6^, Jacqueline Capeau^7^, Christian Pradier^4^, Michel Carles^2^

^1^Internal Medicine/Infectious Diseases, Cannes, France; ^2^Infectious Diseases, Nice, France; ^3^Immunology, Nice, France; ^4^Public Health, Nice, France; ^5^Clinical Research, Nice, France; ^6^Pathogenesis and Control of Chronic and Emerging Infections, Montpellier, France; ^7^Research Saint Antoine, Paris, France

**Background**: Increased immunosenescence, characterized by T‐ and NK‐cell dysfunctions, is suspected among people living with HIV (PLWH). The aim of this study was to explore markers of immunosenescence in elderly PWH controlled by modern integrase strand transfer inhibitors (INSTI)‐based antiretroviral treatment (ART) and their associated risk factors.

**Methods**: We performed a cross‐sectional study including successfully treated PWH, followed in Nice, France, compared to healthy uninfected subjects, matched for age. Senescence markers were assessed on CD4+ and CD8+ T‐cells (KLRG1+CD57+) and NK cells (KLRG1+ and KLRG1+CD56+^bright^), along with markers of cellular activation on T‐cells (CD4+DR+38+ and CD8+DR+38+). We correlated immunosenescence and activation markers with background measurements and comorbid conditions.

**Results**: From 2023 to 2024, 93 PLWH under INSTI‐based ART were included (mean age 63 years, 78% male, time since HIV infection 27 years, CD4 nadir < 200 cc/mm^3^ 46%) and 10 HIV‐ controls. Flow cytometric analysis showed that PLWH had higher total, senescent and activated CD8+ T‐cells than uninfected subjects. Among PLWH, those older than 60 years had higher senescent CD8+ cells, while senescent NK cells increased from the age of 50. After adjustments, men who have sex with men (MSM) had higher senescent CD4+ (coefficient = 1.01, *p* = 0.006) and senescent CD8+ T‐cells (coeff = 0.32, *p* = 0.04), the latter being also associated with age (coeff = 0.03, *p* = 0.002), in contrast with senescent CD4+ cells (coeff = 0.02, *p* = 0.403). NK senescence was associated with both MSM status (coeff = 0.39, *p* = 0.044) and age (coeff = 0.03, *p* = 0.001).

**Conclusions**: We found increased markers of immunosenescence among elderly PWH receiving modern INSTI‐based ART, mainly among MSM, requiring further prospective studies.

### P383 Expectations Regarding Ageing as a Potential Entry Point to Identify Depressive Symptoms in Long‐Term HIV Care: A Japanese Multi‐Centre Study With HIV‐Negative Controls

Hideta Nakamura ^1^, Kazutaka Yamaniha^1^, Nanae Ikemiyagi^1^, Shuhei Ideguchi^1^, Shin Yamashiro^2^, Hitoshi Tamaki^2^, Masashi Narita^3^, Michinori Shirano^4^, Sei Samukawa^5^, Tomoyuki Endo^6^, Junji Imamura^7^, Mayumi Imahashi^8^, Rumi Minami^9^, Kazuko Yamamoto^1^

^1^First Department of Internal Medicine, Division of Infectious, Respiratory, and Digestive Medicine, University of the Ryukyus Graduate School of Medicine, Ginowan, Japan; ^2^Department of Internal Medicine, Naha Yui Hospital, Naha, Japan; ^3^Division of Infectious Diseases, Department of Internal Medicine, Okinawa Prefectural Nanbu Medical Center and Children's Medical Center, Haebaru, Japan; ^4^Department of Infectious Diseases, Osaka City General Hospital, Osaka, Japan; ^5^Department of Hematology and Clinical Immunology, Yokohama City University School of Medicine, Yokohama, Japan; ^6^Department of Infection Control and Prevention, Hokkaido University Hospital, Sapporo, Japan; ^7^Department of Infectious Diseases, NHO Sendai Medical Center, Sendai, Japan; ^8^Department of Infectious Diseases and Immunology, Clinical Research Center, NHO Nagoya Medical Center, Nagoya, Japan; ^9^Department of Internal Medicine, Clinical Research Institute, NHO Kyushu Medical Center, Hakata, Japan

**Background**: As people with HIV age on long‐term antiretroviral therapy (ART), expectations regarding ageing may capture psychosocial vulnerability beyond age and virological control. Asian data with HIV‐negative comparators are scarce. We examined associations of HIV status and depressive symptoms with expectations regarding ageing, and whether discussing ageing expectations could offer a clinical entry point to identify depressive symptoms in long‐term HIV care.

**Methods**: We analysed cross‐sectional data from 325 people with HIV aged ≥40 years recruited at eight HIV care centres spanning Hokkaido to Okinawa, and 160 hospital‐based HIV‐negative controls aged ≥40 years from Okinawa. Expectations regarding ageing were measured with the 12‐item Japanese Expectations Regarding Ageing survey (ERA‐12‐J; total and physical, mental and cognitive domain scores). Depressive and anxiety symptoms were measured with the Patient Health Questionnaire‐9 (PHQ‐9) and Generalized Anxiety Disorder‐7 (GAD‐7). Multivariable linear regression compared ERA‐12‐J scores between groups adjusting for age, sex and body mass index (BMI; Model 1), then additionally for PHQ‐9 and GAD‐7 (Model 2). Associations between PHQ‐9 score and ERA‐12‐J domains were modelled adjusting for HIV status, age, sex, BMI and GAD‐7.

**Results**: People with HIV were younger than controls (median 54.0 vs. 64.0 years), predominantly men (95.6% vs. 69.2%), more often living alone (48.9% vs. 26.0%) and more often current smokers (30.9% vs. 11.0%). Among people with HIV, median ART duration was 12 years, median CD4 count 545 cells/µL and 96.8% had HIV RNA <50 copies/mL. Unadjusted ERA‐12‐J scores were similar between groups. Under Model 1, people with HIV had modestly lower ERA‐12‐J total scores (β = −4.49, 95% CI: −8.96 to −0.01, *p* = 0.049; men‐only β = −5.96, *p* = 0.017); this association attenuated under Model 2. Higher PHQ‐9 score was independently associated with lower ERA‐12‐J total score (β = −1.77 per point, 95% CI: −2.36 to −1.17, *p* < 0.001) and with lower physical, mental and cognitive domain scores (Figure 1).

**Conclusions**: In this predominantly male, virologically suppressed Japanese cohort of people with HIV, lower expectations regarding ageing were most consistently associated with depressive symptom burden. Discussing ageing expectations may offer an acceptable entry point for identifying depressive symptoms and unmet psychosocial needs in routine long‐term HIV care.

**FIGURE 1:**
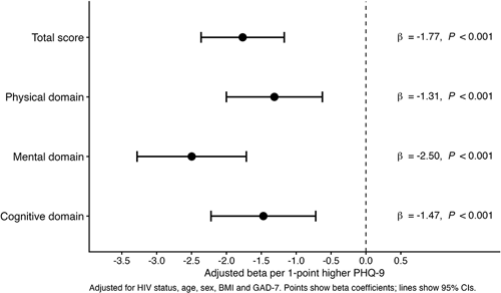
P383 | Adjusted association between PHQ‐9 and ERA‐12‐J scores.

### P384 Ageing With HIV: Longitudinal Changes in Social Support Among People Aged ≥50 in the ELEA‐Brasil Cohort

Camilla Ferreira Santos ^1^, Roberta Schiavon Nogueira^1^, Lucas Rocker Ramos^1^, Giovanna Haimowski^1^, Marilia Bordignon Antonio^2^, Leticia Sanchez Labadia Hypolito^3^, Márlon Juliano Romero Alibert^4^, Beatriz Grinsztejn^5^, Sandra Wagner Cardoso^5^, Thiago S. Torres^5^, Carlos Brites^6^, Estela Luz^6^, Marcio H. Pimenta^6^, Paridhi Ranadive^7^, Jessica Castilho^8^, on behalf of ELEA‐Brasil Cohort

^1^Centro de Referência e Treinamento DST/AIDS, São Paulo, Brazil; ^2^Departamento de Moléstias Infecciosas e Parasitárias do Hospital das Clínicas da Faculdade de Medicina de São Paulo, São Paulo, Brazil; ^3^Centro de Pesquisas Clínicas do Hospital das Clínicas da Faculdade de Medicina de São Paulo, São Paulo, Brazil; ^4^Laboratorio de Investigação Medica em Envelhecimento ‐ LIM‐66, Servico de Geriatria, Hospital das Clinicas HCFMUSP, Faculdade de Medicina, Universidade de Sao Paulo, São Paulo, Brazil; ^5^Instituto Nacional de Infectologia Evandro Chagas, Fundação Oswaldo Cruz (INI‐Fiocruz), Rio de Janeiro, Brazil; ^6^Hospital Universitário Prof. Edgard Santos‐UFBA‐Ebserh, Fundação Bahiana de Infectologia, Salvador, Brazil; ^7^Department of Biostatistics, Vanderbilt University Medical Center, Nashville, TN, USA; ^8^Division of Infectious Diseases, Vanderbilt University Medical Center, Nashville, TN, USA

**Background**: As people living with HIV (PLWH) age, understanding changes in social and emotional support and their impact on clinical outcomes is essential. While social support is a key determinant of health, its relationship with falls remains poorly understood [1]. We evaluated longitudinal changes in perceived support and its association with falls among PLWH in Brazil.

**Methods**: PLWH aged ≥50 on antiretroviral treatment were enrolled in ELEA‐Brasil in São Paulo, Rio de Janeiro and Salvador and underwent geriatric assessments. Social support was measured using the Social Provisions Scale (SPS) [2] at baseline, 12 and 24 months. SPS scores were categorized by quartiles and characteristics associated with stable and dynamic scores over time were compared using descriptive analysis. Logistic regression with GEE evaluated the association between SPS and recent falls, adjusting for sociodemographics, depression (PHQ‐9) [3], cognition (MoCA) [4] and HIV‐related stigma [5].

**Results**: Among 702 participants, median baseline SPS score was 75 (IQR 68–84). Average SPS scores increased from baseline to 12 months (OR 2.76; 95% CI: 1.38–4.14) and from 12 to 24 months (OR 1.91; 95% CI: 0.59–3.23). Support varied over time across individuals (Figure 1) and only 260/696 (37%) had stable high, medium/medium‐high, or low SPS quartiles across all time points. Lower support was reported among females, those with less education, higher HIV‐related stigma, more comorbidities and depressive symptoms, and by city. SPS increase was inversely associated with odds of falls as an outcome in unadjusted analysis (per 10‐point increase OR = 0.79; 95% CI: 0.70–0.88), but was mitigated and no longer significant in adjusted model (Figure 2). In the adjusted model, odds of falls were associated with city, HIV‐related stigma, depression and cognition.

**Conclusions**: Social support improved over time among PLWH but remains uneven and was not associated with falls after adjustment for structural, mental health and cognitive factors. These findings underscore the need for integrated interventions addressing stigma, depression and cognitive decline to support healthy ageing with HIV.

**References**

1. Guaraldi G, Palella FJ Jr. Clinical implications of aging with HIV infection: Perspectives and the future medical care agenda. AIDS. 2017;31(Suppl 2):S129–S135.

2. Caron J. A validation of the Social Provisions Scale: The SPS‐10 items. Sante Ment Que. 2013;38(1):297–318.

3. Kroenke K, Spitzer RL, Williams JB. The PHQ‐9: Validity of a brief depression severity measure. J Gen Intern Med. 2001;16(9):606–613.

4. Nasreddine ZS, Phillips NA, Bédirian V, Charbonneau S, Whitehead V, Collin I, et al. The Montreal Cognitive Assessment, MoCA: A brief screening tool for mild cognitive impairment. J Am Geriatr Soc. 2005;53(4):695–699.

5. Berger BE, Ferrans CE, Lashley FR. Measuring stigma in people with HIV: Psychometric assessment of the HIV Stigma Scale. Res Nurs Health. 2001;24(6):518–529.

**FIGURE 1:**
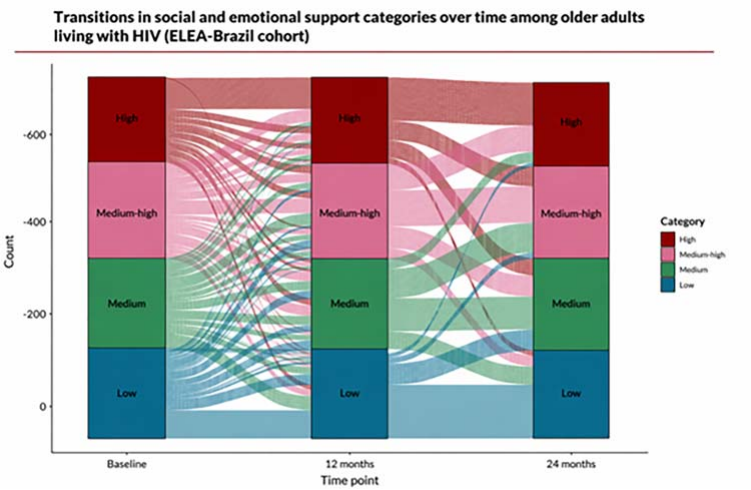
P384 | Sankey diagram showing transitions in social and emotional support categories (SPS quartiles) at baseline, 12 and 24 months among older people living with HIV.

**FIGURE 2:**
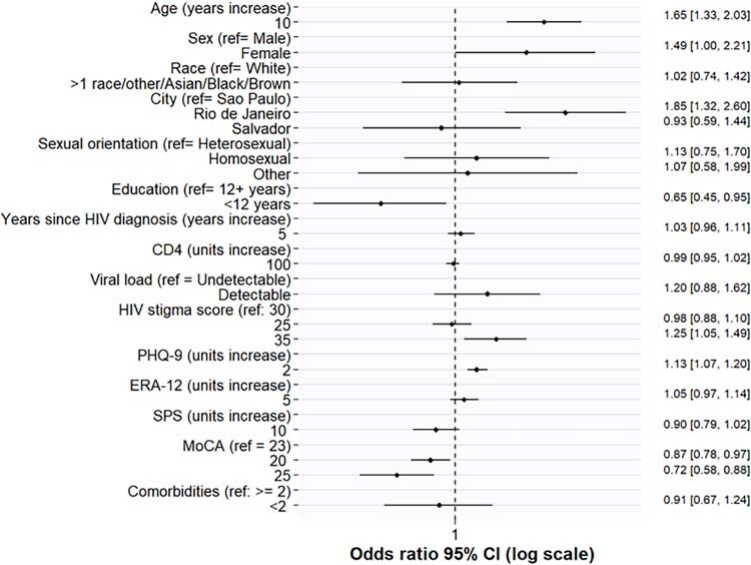
P384 | Forest plot showing factors associated with falls in the 12 months preceding the 24 months visit among older people living with HIV.

### P385 HIV‐Induced Microglial Senescence: A Potential Driver of Neuroinflammation and HAND

Pratiksha Pansambal ^1^, Vitthal Barvkar^2^, Swarali Kurle^1^

^1^ICMR ‐ National Institute of Translational Virology and AIDS Research (Formerly NARI), Pune, Maharashtra, India; ^2^Department of Botany, Savitribai Phule Pune University, Pune, Maharashtra, India

**Background**: People living with HIV experience higher rates of accelerated ageing and HIV‐associated neurocognitive disorders (HAND) [1]. Persistent neuroinflammation is thought to contribute to these complications, but the underlying cellular mechanisms remain incompletely understood [2]. Cellular senescence is a stress‐induced state characterized by irreversible cell‐cycle arrest and secretion of pro‐inflammatory factors [3]. As the resident immune cells of the brain, microglia may play a key role in linking HIV infection to chronic neuroinflammation [4]. We investigated whether HIV induces senescence‐associated changes in human microglia and explored the underlying molecular mechanisms.

**Methods**: Human microglial HMC3 cells and monocyte‐derived microglia (MDMi) were exposed to neurotropic HIV‐1 strains YU2 and JR‐CSF. Doxorubicin was used as a positive control for senescence. Senescence was assessed by quantifying SA‐β‐gal‐positive cells, and reduction in SIRT3 enzymatic activity. Intracellular reactive oxygen species (ROS) and senescence‐associated secretory phenotype (SASP) factors such as CCL2 (MCP‐1), IFN‐γ, IL‐12, TGF‐β, IL‐17A were quantified by flow cytometry. Whole‐transcriptome sequencing was performed to analyse the molecular pathways associated with HIV‐induced senescence.

**Results**: HIV exposure increased the proportion of SA‐β‐gal‐positive cells, reduced SIRT3 enzymatic activity, lead to ROS production, and induced a robust inflammatory SASP in HMC3 cells and these findings were confirmed on MDMi. Integrated functional and transcriptomic analyses demonstrated extensive HIV‐induced reprogramming of microglia, linking oxidative stress and senescence with activation of inflammatory and DNA damage‐response pathways (Figure 1). Transcriptomic profiling identified 2052‐upregulated and 54‐downregulated genes. Senescence‐associated genes including CDKN1A, BTG2, BBC3, TP53I3, MDM2, IGFBP3, RRM2B, PIDD1 and SESN1, together with inflammatory and antiviral response genes including CXCL10 and ISG15, were significantly upregulated. Pathway enrichment demonstrated activation of the p53 signalling pathway, a central regulator of DNA damage responses and cellular senescence, together with cellular senescence, DNA damage response, interferon signalling and Toll‐like receptor signalling pathways. Protein interaction network analysis identified interconnected senescence, interferon and inflammasome‐associated modules, while transcription factor enrichment highlighted TP53 as the dominant upstream regulator.

**Conclusions**: Our study findings demonstrated HIV induced senescence in human microglial cells indicating its possible association with HAND. These findings provide new mechanistic insights into how HIV may contribute to persistent neuroinflammation and the development of HAND.

**References**

1. Cañizares S, Cherner M, Ellis RJ. HIV and aging: Effects on the central nervous system. Semin Neurol. 2014 34(1):27–34. doi: 10.1055/s‐0034‐1372340. Epub 2014 Apr 8. PMID: 24715486; PMCID: PMC4296735.

2. García‐Domínguez M. Neuroinflammation: Mechanisms, dual roles, and therapeutic strategies in neurological disorders. Curr Issues Mol Biol. 2025;47(6):417. doi: 10.3390/cimb47060417. PMID: 40699816; PMCID: PMC12191620.

3.Saito, Y., Yamamoto, S. & Chikenji, T.S. Role of cellular senescence in inflammation and regeneration. Inflamm Regener 44, 28 (2024). https://doi.org/10.1186/s41232‐024‐00342‐5

4.Bai R, Song C, Lv S, Chang L, Hua W, Weng W, Wu H, Dai L. Role of microglia in HIV‐1 infection. AIDS Res Ther. 2023 16;20(1):16. doi: 10.1186/s12981‐023‐00511‐5. PMID: 36927791; PMCID: PMC10018946.

**FIGURE 1:**
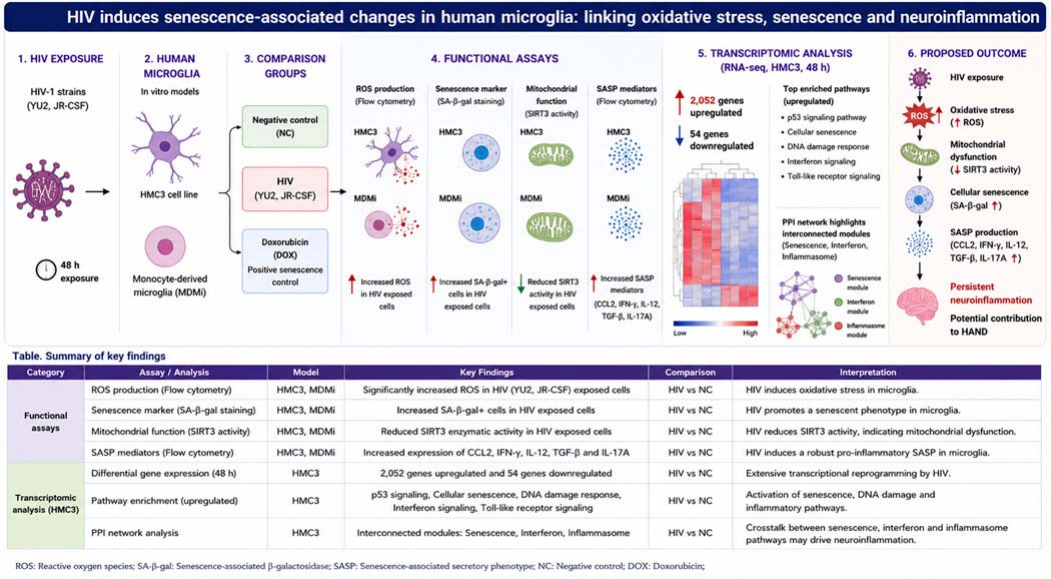
P385 | Experimental workflow and key findings demonstrating HIV‐induced microglial senescence.

### P386 Ageing With HIV: A Cross‐Sectional Analysis of Comorbidities Among Older People Living With HIV in Saudi Arabia

Roaa Alosaimi, Batool Ali, Alaa Malki, Ibrahim Alshehri

First Health Cluster, East Jeddah Hospital, Jeddah, Saudi Arabia

**Background**: People living with HIV (PLWH) are experiencing longer life expectancy and an increasing burden of age‐related comorbidities. In Saudi Arabia, diabetes affects approximately 23% of adults [1], while hypertension affects an estimated 26–32% of the adult population [2]. However, the burden of chronic comorbidities among older PLWH remains poorly characterized. This study evaluated the prevalence and pattern of comorbidities among PLWH aged ≥50 years receiving care at East Jeddah Hospital, which hosts one of the largest HIV care centres in Saudi Arabia.

**Methods**: Demographic and clinical data, including smoking status and comorbid conditions, were collected using a predefined case report form. Participants were categorized into 10‐year age groups. The prevalence of comorbidities was estimated using the exact binomial method with 95% confidence intervals (CI).

**Results**: Data from 495 PLWH aged ≥50 years were analysed. Most participants were aged 50–59 years (55.0%), followed by 60–69 years (35.3%), 70–79 years (8.7%) and ≥80 years (1.0%). Smoking exposure was common: 26.4% were current smokers, 11.6% former smokers and 56.1% reported passive smoking exposure.

Diabetes mellitus was the most prevalent comorbidity, affecting 43.2% of participants (95% CI: 38.8–47.5%), nearly twice the prevalence reported in the general Saudi adult population [2]. Hypertension was present in 37.3% (95% CI: 33.1–41.5%), exceeding national estimates [1]. Cardiovascular comorbidities included myocardial infarction (5.0%), stroke (5.2%) and congestive heart failure (3.5%). Lipid profiles indicated increased cardiometabolic risk, with mean low‐density lipoprotein cholesterol of 128.1 ± 44.2 mg/dL, triglycerides of 182.0 ± 119.8 mg/dL and total cholesterol of 199.4 ± 55.2 mg/dL.

**Conclusions**: Older PLWH in Saudi Arabia carry a substantial burden of cardiometabolic comorbidities, particularly diabetes mellitus and hypertension. Given that East Jeddah Hospital hosts one of the largest HIV care centres in Saudi Arabia, these findings provide important insight into the health needs of the ageing HIV population in the Kingdom. They support the integration of HIV and chronic disease management, with routine cardiovascular risk assessment and preventive care as essential components of ageing HIV care. Multicentre studies are needed to further define the burden of comorbidities among older PLWH in Saudi Arabia.

**References**

1. International Diabetes Federation. Saudi Arabia country profile. Available at: https://diabetesatlas.org. Accessed 13/06/2026.

2. AlRahimi JS, AlHabib KF, AlFaleh HF, et al. National Heart Center/Saudi Heart Association 2025 Guidelines for Cardiovascular Diseases Prevention and Risk Assessment. Saudi Med J. 2026;47:120–177.

### P387 Polypharmacy, Pill Burden, Drug−Drug Interactions, Quality of Medication History Documentation and Rates of HIV Pharmacy Review in the Person ≥75 Years Living With HIV: A Retrospective Audit at a Specialist HIV Service the Albion Centre, Sexual Health and Blood Borne Virus, South Eastern Sydney Local Health District, Sydney, Australia

Yen Li Lim ^1,2^, Natasha McLellan^1^, Melissa Kelly^1^, Arthur Wong^3,4^, Bruce Hamish Bowden^1^

^1^The Albion Centre, Sexual Health and Blood Borne Virus, South Eastern Sydney Local Health District, Sydney, Australia; ^2^Sydney University, Sydney, Australia; ^3^Sydney Sexual Health, Sexual health and Blood Borne Virus, South Eastern Sydney Local Health District, Sydney, Australia; ^4^The Kirby Institute, Sydney, Australia

**Background**: Polypharmacy, typically ≥5 routine medications, is linked to drug–drug and drug–disease interactions, reduced adherence, functional decline and medication errors. In HIV care, polypharmacy definitions vary across studies, limiting comparability [1, 2]. As people living with HIV (PLWH) age, multimorbidity and treatment complexity increase the risk of clinically significant drug–drug interactions (DDIs) and high pill burden. This audit will assess polypharmacy prevalence, pill burden, rates of DDIs and best possible medication history (BPMH) documentation, and will compare these findings with clinician medication records to inform strategies that optimize medication management for older PLWH.

**Methods**: A retrospective audit of the electronic medical records between 1 June 2025 and 31 May 2026 at The Albion Centre was conducted by two medical officers and one pharmacist. Inclusion criteria: clients of the Albion Centre attending for medical specialist HIV care within this period, aged ≥75 years on 31 May 2026 and have antiretrovirals (ARVs) dispensed from on‐site HIV pharmacy. Data collected was de‐identified.

**Results**: 43 records were audited (Table 1). 97.7% had an ARV regimen containing integrase inhibitors (INSTIs). 86% had ≥90% adherence by ARV pick up rate. Polypharmacy was identified in 76.7% and DDIs in 30.2%. Discrepancies between pharmacy and medical documentation were identified for number of prescribed medications (74.4%) and pill burden (81.4%). Figure 1 effectively highlights the extent of these discrepancies.

**Conclusions**: This cohort of long‑term survivors remains highly engaged with the Albion Centre and demonstrates excellent ARV adherence. Regular BPMH has enabled early identification and mitigation of DDIs over many years, contributing to the lower DDI rates observed in this audit, highlighting the benefits of specialist HIV pharmacists. However, gaps in documentation of non‑ARV medications and supplements may lead to missed DDIs and other polypharmacy‑related adverse outcomes. INSTI‑based ARV regimens predominate, underscoring the need for identification of cation‑containing supplements, which can lower integrase inhibitor levels and compromise viral suppression, increasing the risk of resistance. Extending this audit to include PLWH aged ≥65, together with a comprehensive qualitative review of all DDIs, will generate valuable insights to inform targeted, evidence‑based strategies that strengthen active medication management and promote a person‑centred approach for the ageing population living with HIV.

**References**

1. Sukumaran L, Winston A, Marzolini C, et al. Polypharmacy in HIV: Rethinking what counts and why it matters. HIV Med. 2026;27(2):186‐199. Available at: doi:10.1111/hiv.70129. Accessed on 26/6/26

2. Danjuma, M.I., Khan, S., Wahbeh, F., Naseralallah, L.M., Jumbo, U.E., & Elzouki, A. (2022). What is polypharmacy in people living with HIV/AIDS? A systematic review. AIDS Research and Therapy, 19(1), 37. Available at: https://doi.org/10.1186/s12981‐022‐00461‐4 Accessed on 26/6/26

**FIGURE 1:**
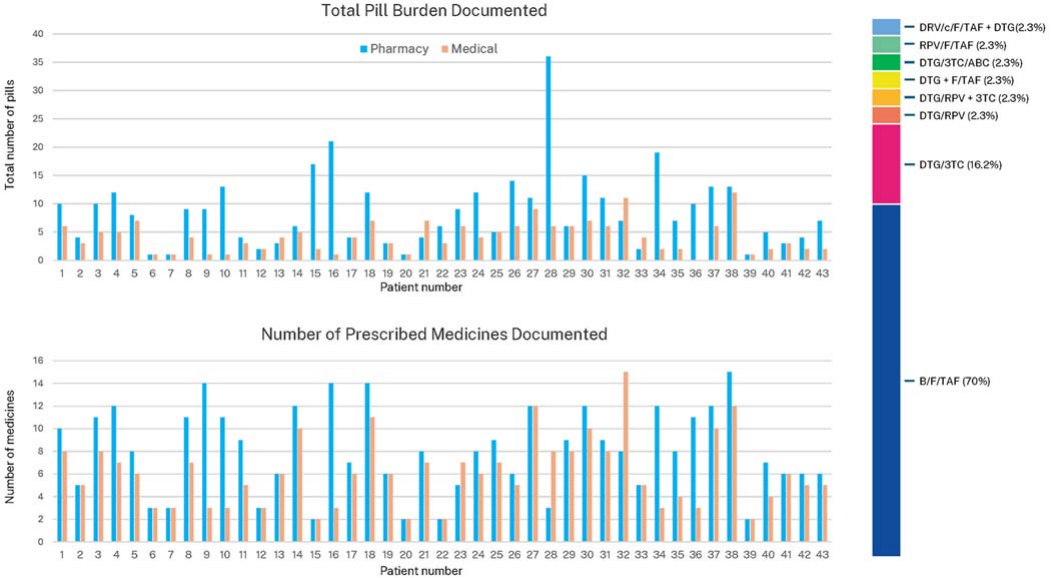
P387 | Comparison of pharmacist‐ and medical‐documented medication histories for both total pill burden and number of prescribed medications. The right panel displays the percentage distribution of antiretroviral regimens among patients.

**TABLE 1:**
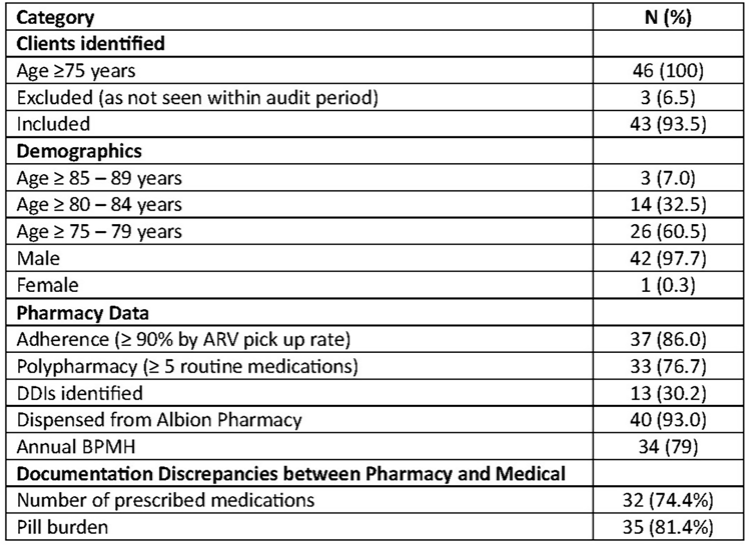
P387 | Demographics of identified clients age ≥75 years, adherence, polypharmacy, BPMH review rates and documentation discrepancies between pharmacy and medical.

### P388 Abstract Withdrawn

## Co‐Morbidities and Complications of Disease and/or Treatment: CV/Metabolic Including Weight Gain

### P389 The Impact of Viraemia on Diabetes in the Modern INSTI Era: A Time‐Updated Analysis

Giovanni Festa ^1^, Dario Diana^1,2^, Nicolò Capra^3^, Riccardo Lolatto^3^, Silvia Nozza^1,3^, Angelo Roberto Raccagni^1,3^, Hamid Hasson^3^, Golnaz Torkjazi^1^, Flavia Passini^1^, Antonella Castagna^1,3^, Vincenzo Spagnuolo^1,3^, Tommaso Clemente^3^

^1^Vita‐Salute San Raffaele University, Milan, Italy; ^2^Infectious Diseases Unit, University of Naples Federico II, Naples, Italy; ^3^Infectious Diseases, IRCCS San Raffaele Scientific Institute, Milan, Italy

**Background**: Metabolic dysfunction emerged as a major contributor to long‐term morbidity in people with HIV. Although persistent inflammation may play a key role in diabetes incidence, the metabolic impact of viraemia remains uncertain. We aimed to investigate the association between viraemia and type‐2 diabetes mellitus among people receiving antiretroviral therapy (ART) during integrase inhibitor (INSTI) era, focusing on persistent low‐level viraemia (pLLV).

**Methods**: Cohort study including people with HIV initiating ART between 2008 and 2024 and with ≥3 viral load (VL) measurements after ≥1 year on ART. Individuals with pre‐existing diabetes were excluded. Participants were classified according to virological status as virological suppression (VS, < 50 copies/mL), pLLV (≥2 consecutive VLs 50–199 copies/mL) or virological failure (VF, ≥2 consecutive VLs ≥200 or ≥1 VL ≥1000 copies/mL). VL was modelled as a time‐updated exposure; participants were reclassified over time according to their highest virological category. The primary endpoint was incident diabetes, defined by fasting glucose ≥126 mg/dL on two occasions, random glucose ≥200 mg/dL or glycated haemoglobin ≥48 mmol/mol. Follow‐up accrued from the first VL after ≥1 year on ART (baseline) until incident diabetes or last visit. Poisson regression modelled incidence rate (IR). Cox models estimated the associations between virological status and incident diabetes.

**Results**: Overall, 2330 people were included. According to the highest virological category reached, 1771 (76.0%) remained virologically suppressed, 261 (11.2%) experienced ≥1 episodes of pLLV and 298 (12.8%) VF (Table 1). During a median follow‐up of 8.9 years (interquartile range [IQR], 5.5–12.2; 20,390 person‐years), 136 participants developed diabetes (IR 0.67/100 person‐years). Diabetes occurred in 100/1771 (5.7%) individuals with VS, 13/261 (5.0%) with pLLV and 23/298 (7.7%) with VF. Compared with VS, pLLV was not associated with incident diabetes (unadjusted hazard ratio [uHR], 1.11, 95% confidence interval 0.62–2.00), whereas VF conferred a significantly increased risk (uHR 1.72, 95% CI: 1.07–2.77). In multivariable model, VF but not pLLV independently predicted diabetes alongside traditional risk factors (Figure 1).

**Conclusions**: In our modern cohort, VF but not pLLV was associated with risk of diabetes. These findings suggest that metabolic impact of HIV replication may be threshold‐dependent, with clinically relevant consequences emerging only at higher levels of viraemia.

**FIGURE 1:**
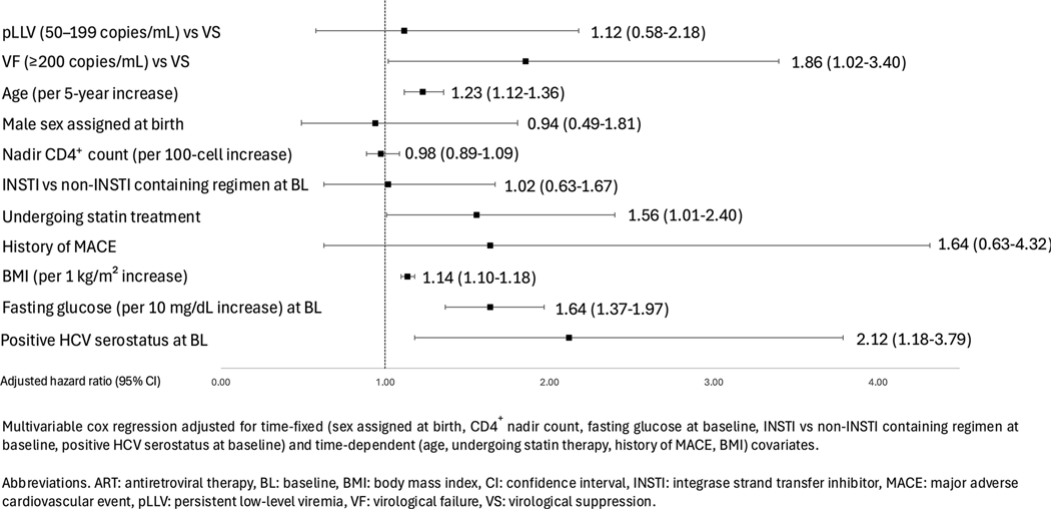
P389 | Multivariable predictors of incident diabetes mellitus.

**TABLE 1:**
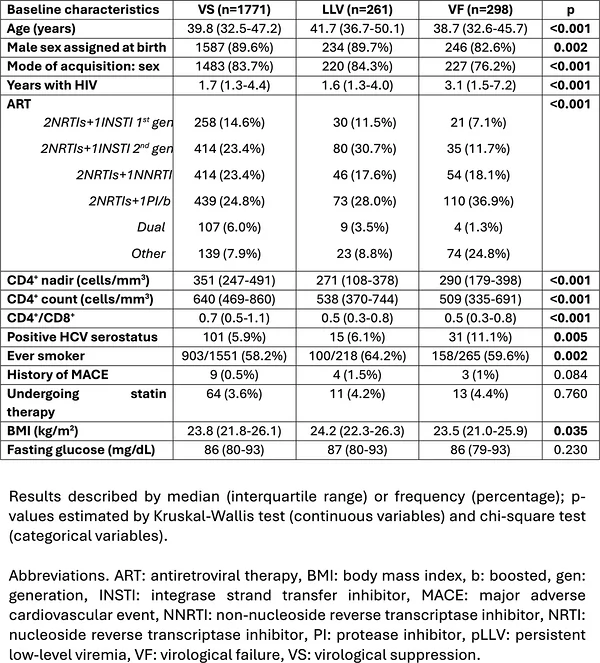
P389 | Baseline characteristics of people with HIV included in the analysis stratified according to their highest virological category.

### P390 Abstract Withdrawn

### P391 Barriers and Facilitators to Statin Prescribing in Hospital‐Based HIV Care: Insights From a Dutch Clinician Survey

Rosa Elias ^1,2^, Kim Romijnders^3^, Casper Rokx^2^, Judith Branger^4^, Aafke Cents‐Bosma^4^, Fanny Lauw^5^, Annelies Verbon^1^, Berend van Welzen^1^

^1^Department of Infectious Diseases, University Medical Centre Utrecht, Utrecht, Netherlands; ^2^Department of Internal Medicine, and Department of Medical Microbiology and Infectious Diseases, Erasmus University Medical Centre, Rotterdam, Netherlands; ^3^Julius Centre for Health Sciences and Primary Care, University Medical Centre Utrecht, Utrecht University, Utrecht, Netherlands; ^4^Department of Internal Medicine, Flevo Hospital, Almere, Netherlands; ^5^Department of Internal Medicine, Alrijne Hospital, Leiderdorp, Netherlands

**Background**: People with HIV (PWH) face a twofold risk of atherosclerotic cardiovascular diseases (ASCVD) compared to people without HIV, yet despite evidence from the REPRIEVE trial and the EACS‐guidelines emphasizing the importance of statin therapy in PWH, Dutch surveillance data show that only 28–44% of eligible PWH were receiving statins in 2024. The aim of our study is to identify and quantify the barriers and facilitators of statin prescribing among HIV treating clinicians in the Netherlands.

**Methods**: A cross‐sectional survey was conducted among HIV‐treating clinicians in hospital care in the Netherlands between January and March 2026. This survey comprised 34 statements across 14 theoretical domains based on the Theoretical Domain Framework, representing the main areas of behaviour and implementation research. These statements were answered on a 5‐point Likert scale and transformed to a 1–100 scale from strong barrier to strong facilitator. Self‐reported rates of successful statin discussion, initiation, and discontinuation and reasons for failure were also collected.

**Results**: A total of 72 prescribers completed the survey (response rate 47.5%). Self‐reported rates for discussing statin therapy were 80% and successfully initiating statins was 68%. Patient decision was the primary reason for non‐initiation. The discontinuation rate was 10%, estimated by the respondents. The most prominent barriers were absence of feedback on prescribing behaviour (51%), anticipated discontinuation of statin (45%) and forgetting to address statin therapy during consultation (41%) (Figure 1). Infectious disease specialists reported significantly higher capability scores and less concern about discontinuation and decision‐making process compared to nurse practitioners and physician assistants.

**Conclusions**: Suboptimal prescribing of statins in the Dutch HIV‐care appears to arise from behavioural and structural barriers rather than knowledge deficits. The discrepancy between anticipated discontinuation and the self‐reported estimated discontinuation rates are noteworthy and may reflect a sense of therapeutic futility. This study signals the clinician's assumptions regarding statin prescribing which can be encouraged to improve preventive cardiovascular care in this population.

**FIGURE 1:**
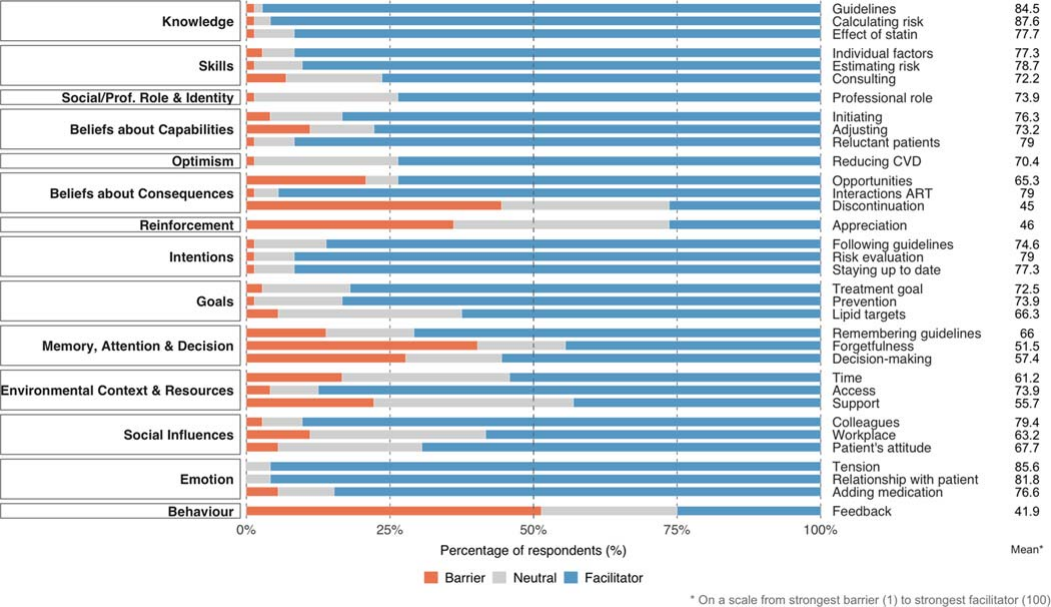
P391 | Perceived barriers and facilitators per theoretical domain and individual statements of the questionnaire.

### P392 No Impact of Metformin on Body Fat Compartments, Including Visceral Fat, as Measured by Whole Body Magnetic Resonance Imaging in the MAVMET Trial

James Burns^1,2^, Augustus Rottenburg^3^, Abulrahman Mohamed^3^, Stuart Taylor^3^, Naomi Sakai^3^, Chloe Dennis^3^, Yolanda Collaco‐Moraes^4^, Frank Post^5,6^, Julie Fox^7^, Margaret Johnson^8^, Lucy Garvey^9^, Chloe Orkin^10,11^, David Dunn^4^, Sarah Pett ^2,4^, Manil Chouhan^3^, on behalf of MAVMET Study Group

^1^Centre for Clinical Research in Sexual Health and HIV, Institute for Global Health, University College London (UCL), London, UK; ^2^Mortimer Market Centre, Central and Northwest London NHS Foundation Trust, London, UK; ^3^UCL Centre for Medical Imaging, UCL, London, UK; ^4^UCL Innovative CTU, Institute for Clinical Trials and Methodology, UCL, London, UK; ^5^King's College Hospital NHS Foundation Trust, London, UK; ^6^King's College London, London, UK; ^7^Guy's and St Thomas’ NHS Foundation Trust, London, UK; ^8^Royal Free London NHS Foundation Trust, London, UK; ^9^Imperial College Healthcare NHS Trust, London, UK; ^10^Royal London Hospital, Barts Health NHS Trust, London, UK; ^11^Queen Mary University of London, London, UK

**Background**: Fat redistribution and weight changes are becoming increasingly problematic in people with HIV (PLWH). Antiretroviral (ART) side effects and societal trends towards being overweight or obese are contributing factors. Contrary to our hypothesis, in the MAVMET trial, neither maraviroc, metformin, or the combination significantly reduced liver fat as measured by magnetic resonance proton density fat fraction (MRPDFF) compared to ART alone in non‐diabetic, suppressed PLWH [1]. Metformin has been associated with reductions in visceral fat [2] which we sought to evaluate using this high‐resolution imaging modality.

**Methods**: In this sub‐study, we analysed changes in whole body, visceral, subcutaneous and paraspinal muscle fat; visceral to subcutaneous fat ratio; and skeletal muscle volume by whole body MRPDFF. We compared participants with week 0 and 48 scans who received metformin versus no metformin. Measurements were recorded by MRPDFF‐experienced radiologists; analysis of covariance on a log scale was used.

**Results**: 80 participants were included (*n* = 2/82 missing scans); Table 1 includes the sample sizes for each measurement. Median age was 52 (IQR 47–57) years, mean (SD) weight and body mass index were 90 kg (SD 14.5) and 29 kg/m^2^ (SD 4.3), respectively. Higher baseline visceral fat correlated with increased liver fat fraction, age and weight (coefficients 0.41, 0.30 and 0.43, respectively, all *p* < 0.01). Older baseline age also correlated with less skeletal muscle volume (−0.39, *p* < 0.01). For the metformin versus no metformin groups, we observed no significant difference between the two scans in any of the radiological measurements (Table 1). Visceral fat had less variation compared to subcutaneous fat (Figure 1a,b); changes in skeletal muscle volume and paraspinal muscle fat were more heterogenous (Figure 1d,e). We observed no correlation between changes in subcutaneous fat and skeletal muscle volume. A sensitivity analysis with maraviroc also yielded no significant findings.

**Conclusions**: In this MAVMET sub‐study, metformin did not have any impact on visceral or subcutaneous fat. Additionally, there was no impact on secondary muscle parameters or weight. These findings support the need to focus investigation on the potential beneficial role of other classes of drugs, including the GLP‐1 receptor agonists, for reducing visceral fat in PLWH.

**References**

1. McCabe L, Burns JE, Latifoltojar A, Post FA, Fox J, Pool E, et al; the MAVMET Study group. MAVMET trial: Maraviroc and/or metformin for metabolic dysfunction associated fatty liver disease in adults with suppressed HIV. AIDS. 2024;38(10):1513–1522. doi: 10.1097/QAD.0000000000003947. Accessed 22‐Jun‐2026.

2. Hadigan C, Corcoran C, Basgoz N, Davis B, Sax P, Grinspoon S. Metformin in the treatment of HIV lipodystrophy syndrome: A randomized controlled trial. JAMA. 2000;284(4):472–7. doi: 10.1001/jama.284.4.472. PMID: 10904511. Accessed 22‐Jun‐2026.

**FIGURE 1a‐f:**
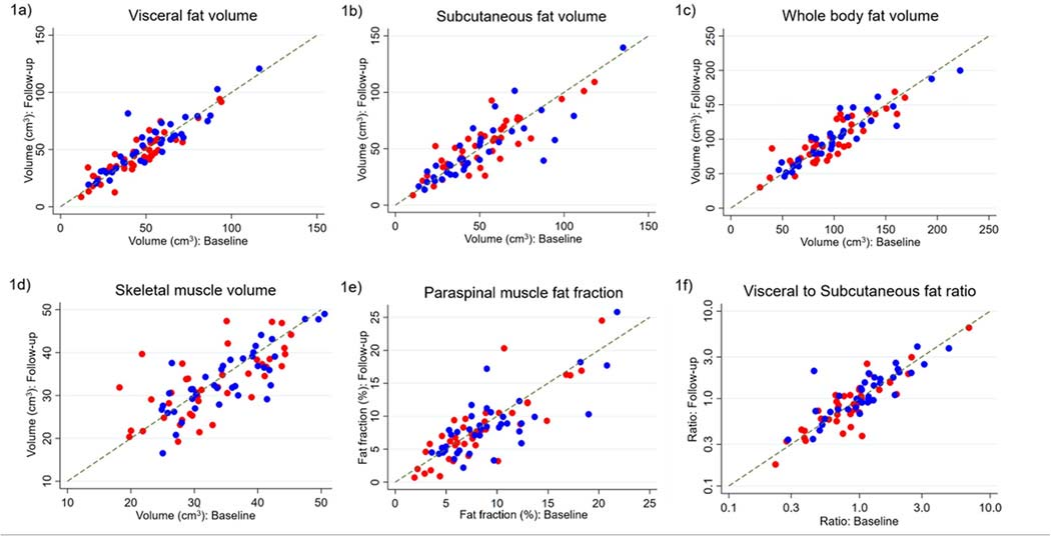
P392 | Scatterplots showing the change between week 0 and week 48 scans for the MRPDFF measurements (blue: metformin; red: no metformin).

**TABLE 1:**
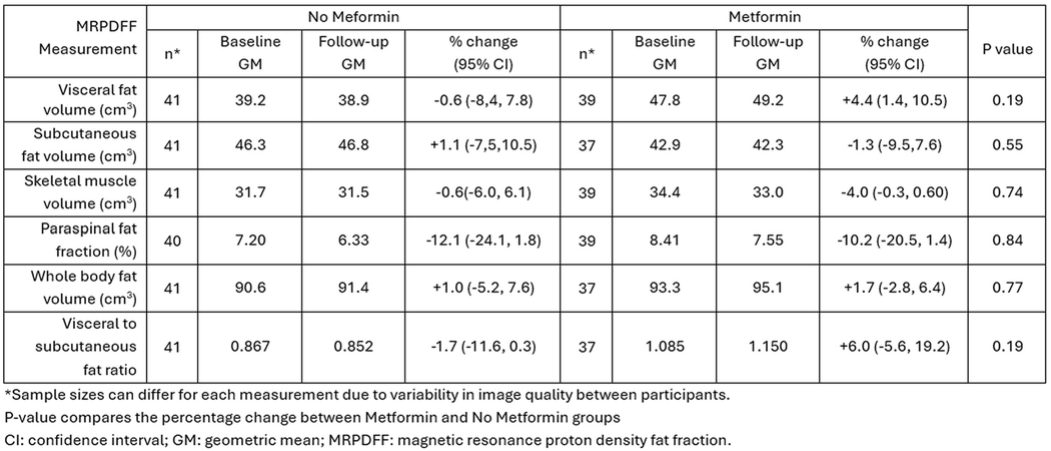
P392 | Analysis of covariance of the change in MRPDFF measurements between week 0 and week 48.

### P393 Cardiovascular Risk Assessment by Imaging in People With HIV: Added Value Compared With Clinical Risk Scores and Impact on Preventive Management

Martin Chaix ^1^, Remy Duong^2^, Dominique Batisse^1^, Marina Karmochkine^1^, Juliette Pavie^1^, Anne Rachline^1^, Laurence Slama^1^, Jean‐Paul Viard^1^, Romain Palich^1^, Clémentine De la Porte des Vaux^1^

^1^Immuno‐infectiology department, Hôtel Dieu Hospital, Paris, France; ^2^Diagnostic and Treatment Center, Hôtel Dieu Hospital, Paris, France

**Background**: People with HIV (PWH) are at increased risk of cardiovascular disease compared with the general population [1, 2]. Current guidelines recommend SCORE2‐based algorithms to estimate cardiovascular risk (CVR) and guide preventive strategies [3]; however, their performance in PWH remains uncertain. We evaluated the added value of multimodal imaging‐based CVR assessment beyond guideline‐recommended clinical risk scores, and its therapeutic implications.

**Methods**: We conducted a prospective observational study including adult PWH admitted in day‐hospital at Hôtel‐Dieu Hospital (Paris, France). Eligible participants had no prior cardiovascular event and underwent at least one vascular imaging examination (coronary CT angiography [CCTA], coronary artery calcium [CAC] score and/or carotid ultrasound) between November 2025 and April 2026. CVR was assed according to (i) imaging findings (based on the most severe vascular lesion identified across the 3 imaging modalities and categorized as low‐to‐moderate, high or very high risk), and (ii) SCORE2 calculation. Concordance between the two approaches was assessed using Cohen's kappa coefficient and Gwet's AC2 statistic.

**Results**: A total of 208 participants were included (median age: 60 years; 69% male; median duration of HIV infection: 24 years), of whom 38% were already receiving statin therapy, 12% had diabetes and 24% were active smokers. Concordance between imaging‐based CVR classification and SCORE2 was poor (κ = 0.14). Imaging‐based CVR evaluation classified 30% of participants at very high CVR, compared with 10% using SCORE2 (Figure 1). This discordance persisted across all cardiovascular risk factors subgroups. The concordance between CVR assessed by CCTA alone and by the combined multimodal imaging approach (3 modalities) was moderate (κ = 0.60; Gwet's AC2 0.66); however, CCTA alone underestimated CVR in 30% of participants relative to multimodal imaging. Imaging findings led to lipid‐lowering therapy initiation/dose increase in 107 (51%) and to CVR based ART modification in 26 (12.5%) participants. Relying on SCORE‐2 alone, as suggested in guidelines, would have resulted in undertreatment with lipid‐lowering therapy in 19% of participants and overtreatment in 4%.

**Conclusions**: SCORE‐2 may underestimate subclinical atherosclerotic burden in PWH. Multimodal vascular imaging may refine CVR stratification in ageing PWH. A multidisciplinary day‐hospital approach appears valuable.

**References**

1. Triant VA, Lee H, Hadigan C, Grinspoon SK. Increased acute myocardial infarction rates and cardiovascular risk factors among patients with human immunodeficiency virus disease. J Clin Endocrinol Metab. 2007;92(7):2506‑12.

2. Shah ASV, Stelzle D, Lee KK, Beck EJ, Alam S, Clifford S, et al. Global burden of atherosclerotic cardiovascular disease in people living with HIV: Systematic review and meta‐analysis. Circulation. 2018;138(11):1100‑12.

3. CNS, ANRS MIE. Screening and management of comorbidities in adults living with HIV. Expert group recommendations. Paris: National AIDS and Viral Hepatitis Council; September 2024. Available from: https://www.anrs.fr/wp‐content/uploads/2024/11/vih‐comorbidites‐recommandations‐rapport‐dexperts–20241104‐light.pdf.

**FIGURE 1:**
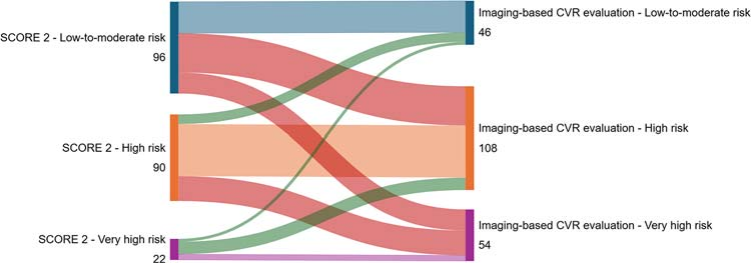
P393 | Reclassification of CVR with vascular imaging (carotid ultrasound, CAC score, CCTA).

### P394 Metabolic Outcomes After Switch to Long‐Acting CAB+RPV Versus DOR/3TC/TDF in Virologically Suppressed People With HIV: A Real‐World Cohort Study

Arturo Ciccullo ^1^, Gianmaria Baldin^2^, Adriana Cervo^3^, Andrea Giacomelli^4^, Sasha Trevisan^5^, Andrea Carbone^2^, Andrea De Vito^6^, Maria Vittoria Cossu^4^, Massimiliano Fabbiani^7^, Emilio Altavilla^8^, Francesco Bassani^9^, Filippo Lagi^5^, Alberto Borghetti^10^, Chiara Papalini^11^, Giordano Madeddu^6^, Spinello Antinori^4^, Andrea Gori^4^, Cristina Mussini^3^, Simona Di Giambenedetto^2^

^1^Infectious Diseases Unit, L'Aquila, Italy; ^2^Fondazione Policlinico Universitario A. Gemelli IRCCS, Rome, Italy; ^3^Infectious Diseases Clinic, University Hospital of Modena, Modena, Italy; ^4^Department of Infectious Diseases, ASST Fatebenefratelli Sacco, Luigi Sacco University Hospital, Milan, Italy; ^5^Department of Experimental and Clinical Medicine, University of Florence, Firenze, Italy; ^6^Unit of Infectious Diseases, Department of Medicine, Surgery and Pharmacy, Sassari, Italy; ^7^Department of Medical Sciences, Infectious and Tropical Diseases Unit, University Hospital of Siena, Siena, Italy; ^8^Department of Public Health and Infectious Diseases, Sapienza University of Rome, Rome, Italy; ^9^Infectious Diseases Unit, Ospedale Civile di Legnano ASST Ovest Milanese, Legnano, Italy; ^10^Infectious Diseases Unit, Department of Clinical and Experimental Medicine, Azienda Ospedaliera Universitaria Pisana, Pisa, Italy; ^11^Department of Medicine, Clinic of Infectious Diseases, “Santa Maria della Misericordia” Hospital, University of Perugia, Perugia, Italy

**Background**: Metabolic alterations, including dyslipidaemia, weight gain and insulin resistance, remain important considerations when selecting maintenance antiretroviral regimens in virologically suppressed people with HIV (PWH), particularly with the increasing use of long‐acting and novel oral switch strategies.

**Methods**: We conducted a multicentre observational study including virologically suppressed PWH switching to either DOR/3TC/TDF or long‐acting CAB+RPV (LAI). Metabolic outcomes included insulin resistance (METS‐IR), BMI and lipid profile parameters (total cholesterol, LDL cholesterol, HDL cholesterol, triglycerides and total cholesterol/HDL ratio). Changes were assessed at weeks 48 and 144. Longitudinal analyses and linear regression models were used to evaluate predictors of metabolic changes and the association between treatment group and outcomes.

**Results**: Overall, 857 PWH were included: 438 in the DOR group and 419 in the LAI group. Baseline characteristics are summarized in Table 1. METS‐IR significantly decreased in the DOR group at weeks 48 (median change −0.5, *p* < 0.001) and week 144 (−1.0, *p* < 0.001) while no changes were observed in the LAI group. The reduction was more pronounced in PWH with a higher baseline METS‐IR (*p* = 0.035) and longer time from HIV diagnosis (*p* = 0.047), and less pronounced in those with longer cumulative ARV exposure (*p* = 0.020). BMI increased significantly in the LAI group at week 48 (*p* = 0.033), and CAB+RPV exposure was independently associated with a greater BMI increase (*t* = 2.7, *p* = 0.007). Lipid parameters improved in the DOR group, with significant reductions in total cholesterol, LDL cholesterol and triglycerides at weeks 48 and 144 (all *p* < 0.001). Higher baseline lipid levels were independently associated with greater reductions in total cholesterol (*t* = −8.0, *p* = 0.001), triglycerides (*t* = −21.4, *p* < 0.001) and LDL cholesterol (*t* = −6.2, *p* < 0.001). Total cholesterol/HDL ratio also decreased significantly (−0.33 at week 144, *p* < 0.001). In the LAI group, a modest improvement in the ratio was observed at week 144 (−0.24, *p* = 0.009). After 144 weeks, CAB+RPV exposure was associated with less pronounced improvements in total cholesterol (*t* = 3.0, *p* = 0.003), LDL cholesterol (*t* = 2.2, *p* = 0.028) and triglycerides (*t* = 3.1, *p* = 0.002).

**Conclusions**: In this real‐world cohort of virologically suppressed PWH, DOR/3TC/TDF was associated with more favourable metabolic outcomes, including reductions in insulin resistance and greater improvements in lipid profile compared with CAB+RPV.

**TABLE 1:**
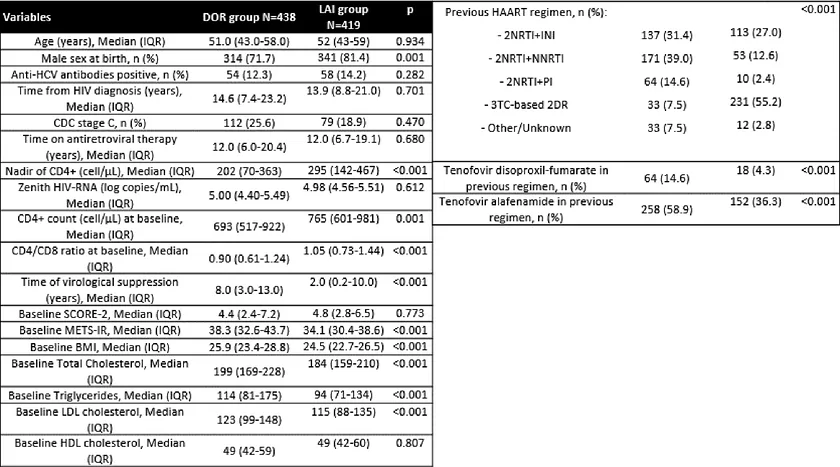
P394 | Population characteristics at baseline.

### P395 Incident MASLD During the First Year After HIV Diagnosis: Longitudinal Changes in Liver Metabolic Health From the Prospective MASHIV‐TR Cohort

Ayşe Özlem Mete^1^, Çiğdem Mermutluoğlu^2^, Yeşim Taşova^3^, İlkay Karaoğlan^1^, Hüsnü Pullukçu^4^, Meliha Çağla Sönmezer^5^, Dilara İnan^6^, Funda Şimşek^7^, Sıla Akhan^8^, Tuba Damar Çakırca^9^, Nursel Çalık Başaran^10^, Aslıhan Candevir^3^, Yeliz Çiçek^7^, Damla Ertürk^3^, Ahmet Çağkan İnkaya^5^, İsmail Yıldız^11^, Behice Kurtaran^3^, Müge Toygar Deniz^8^, Yaşar Bayındır^5^, Mustafa Kemal Çelen ^2^

^1^Infectious diseases, Gaziantep, Türkiye; ^2^Infectious diseases, Diyarbakir, Türkiye; ^3^Infectious diseases, Adana, Türkiye; ^4^Infectious diseases, Izmir, Türkiye; ^5^Infectious diseases, Ankara, Türkiye; ^6^Infectious diseases, Antalya, Türkiye; ^7^Infectious diseases, Istanbul, Türkiye; ^8^Infectious diseases, Kocaeli, Türkiye; ^9^Infectious diseases, Şanlıurfa, Türkiye; ^10^Internal Medicine, Ankara, Türkiye; ^11^Biostatistics, Diyarbakir, Türkiye

**Background**: Metabolic dysfunction‐associated steatotic liver disease (MASLD) is increasingly recognized as an important comorbidity in people living with HIV (PLWH). We prospectively evaluated changes in liver metabolic health during the first year after HIV diagnosis and examined factors associated with incident MASLD.

**Methods**: In this prospective multicentre cohort from Türkiye, 411 participants were enrolled at baseline, including 250 newly diagnosed PLWH and 161 HIV‐negative controls. Controlled attenuation parameter (CAP) was assessed by transient elastography (FibroScan) at baseline, and participants with available follow‐up data were reassessed at month 12. MASLD was defined as CAP ≥248 dB/m in the presence of at least one cardiometabolic risk factor. Propensity score overlap weighting was used to address baseline imbalance between PLWH and controls (Figure 1).

**Results**: After overlap weighting, baseline covariates were well balanced between PLWH and controls. In adjusted between‐group models, PLWH had higher month‐12 CAP than controls (difference, 11.66 dB/m; 95% CI, 5.01–18.31; *p* < 0.001) and higher odds of incident MASLD (OR, 7.30; 95% CI, 2.93–18.19; *p* < 0.001) (Table 1). Incident MASLD occurred in 56 of 170 baseline MASLD‐negative PLWH with follow‐up data (32.9%) and in 9 of 111 baseline MASLD‐negative controls with follow‐up data (8.1%). Overlap‐weighted analyses were consistent with the primary adjusted models for both month‐12 CAP (difference, 10.95 dB/m; 95% CI, 2.72–19.18; *p* = 0.009) and incident MASLD (OR, 5.52; 95% CI, 1.74–17.48; *p* = 0.004). Baseline antiretroviral therapy regimen was associated with month‐12 CAP and incident MASLD. Incident MASLD was more frequent in participants receiving B/F/TAF (33/69, 47.8%) than in those receiving LAM+DTG (14/56, 25.0%) or TDF/FTC+DTG (9/65, 13.8%).

**Conclusions**: In this prospective multicentre cohort, newly diagnosed PLWH had greater progression of hepatic steatosis and higher odds of incident MASLD during the first year after HIV diagnosis than HIV‐negative controls. Incident MASLD developed in approximately one‐third of baseline MASLD‐negative PLWH with follow‐up data. These findings support early metabolic risk assessment and FibroScan‐based liver monitoring after HIV diagnosis and ART initiation. Associations with specific ART regimens should be interpreted cautiously and require confirmation in studies designed to evaluate treatment‐specific effects.

**FIGURE 1:**
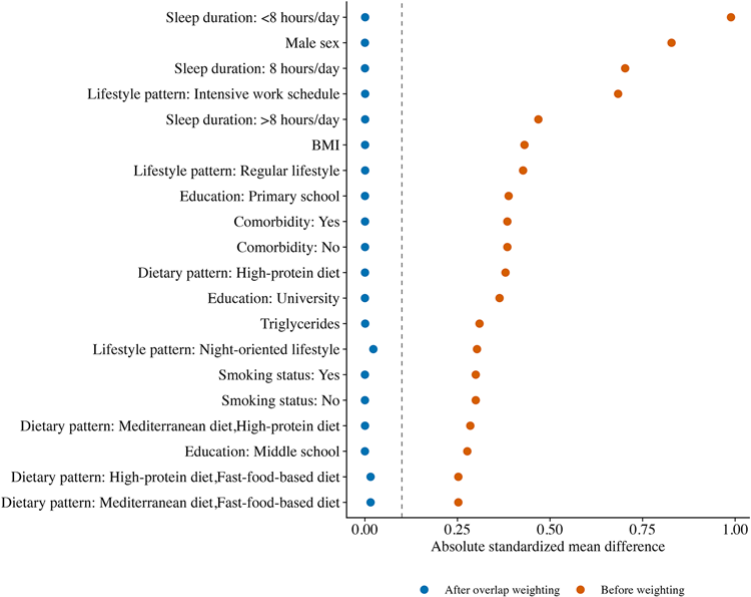
P395 | Covariate balance before and after propensity score overlap weighting.

**TABLE 1:**
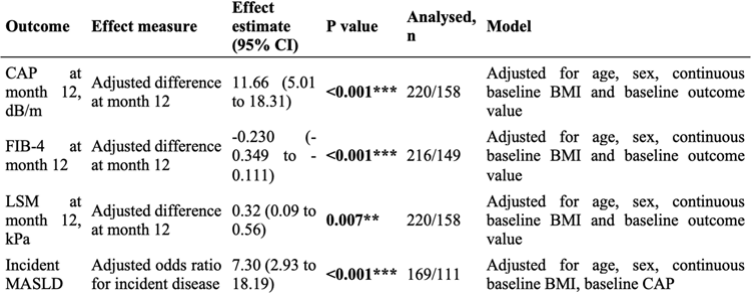
P395 | Adjusted between‐group differences in 1‐year liver outcomes.

### P396 Reframing Obesity in HIV Care: Agreement Between BMI and the Lancet Commission Definition

Jovana Milić^1,2^, Enrico Roma^3^, Licia Gozzi^2^, Marianna Menozzi^2^, Lorenzo Benassi^1,2^, Severino Ambrosio^1,2^, Cristina Mussini^1,2^, Giovanni Guaraldi ^1,2^

^1^Department of Surgical, Medical, Dental and Morphological Sciences, University of Modena and Reggio Emilia, Italy, Modena, Italy; ^2^Modena HIV Metabolic Clinic, University of Modena and Reggio Emilia, Modena, Italy, Modena, Italy; ^3^University of Bologna, Bologna, Italy

**Background**: The 2025 Lancet Commission redefined obesity as a clinical diagnosis based on adiposity‐related organ dysfunction rather than BMI. We assessed the prevalence of clinical obesity in people with HIV (PWH), its evolution over two decades, and the agreement between BMI‐based and Lancet Commission classifications.

**Methods**: This was a longitudinal observational study including PWH attending Modena HIV Metabolic Clinic, Italy, with ≥2 anthropometric and laboratory assessments (2005–2025). The BMI‐based definition required BMI ≥30 kg/m^2^. For the Lancet definition, excess adiposity was defined as visceral adipose tissue ≥150 cm^2^ or ≥2 anthropometric criteria (waist‐to‐height ratio ≥0.50 and waist circumference ≥102 cm in men/≥88 cm in women); individuals were classified as metabolically healthy (no adiposity criteria), preclinical obesity (adiposity without organ damage), or clinical obesity (adiposity plus ≥1 obesity‐related condition: diabetes, hypertension, cardiovascular disease, chronic kidney disease, COPD or liver disease). Concordance was assessed using overall agreement and weighted kappa.

**Results**: 3571 PWH contributed to 29,711 observations. At baseline, 689 (19.3%) had clinical obesity, 399 (11.2%) preclinical obesity and 2483 (69.5%) were metabolically healthy. Characteristics of the study population at baseline is shown in Table 1. The proportion of clinical obesity captured by a BMI ≥30 kg/m^2^ was low and stable in the earlier periods but rose in the most recent years. Among PWH with clinical obesity, only 20.6% had a BMI ≥30 in 2005–2014 and 20.2% in 2015–2019, increasing to 31.5% in 2020–2025. The majority of clinical obesity occurred in PWH who would not be classified as obese by BMI: 78.9% had a BMI < 30 at the random visit, and this remained the case for 68.5% even in 2020–2025. Agreement between the BMI‐based and Lancet Commission definitions was moderate (overall agreement 0.63; weighted kappa 0.47) and was lower in men than in women (Figure 1). Disagreement was driven by BMI underestimating metabolic severity, from 23.7% in 2005–2014 to 31.2% in 2020–2025.

**Conclusions**: BMI demonstrates only moderate agreement with clinical obesity and consistently underestimates its prevalence, failing to identify approximately 60–80% of cases. Adoption of the Lancet Commission framework may improve identification of clinical obesity and better inform metabolic care in PWH.

**FIGURE 1:**
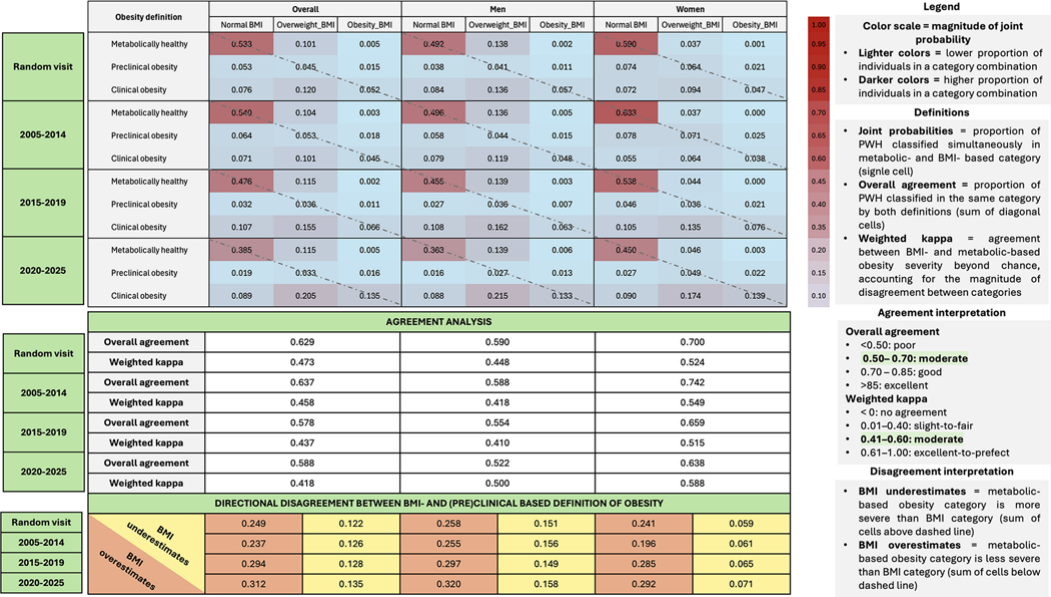
P396 | Agreement and directional disagreement between BMI‐based and metabolic‐based obesity classifications overall, by sex and across study periods.

**TABLE 1:**
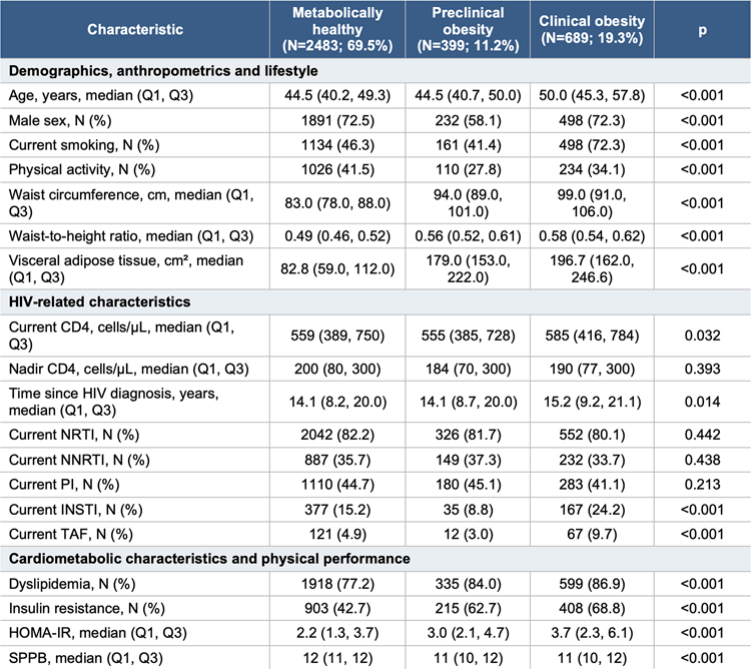
P396 | Baseline characteristics of the study population by obesity state according to the Lancet Commission definition. Data are median (Q1, Q3) for continuous variables and *N* (%) for categorical variables. Abbreviations: CD4, cluster of differentiation 4; HOMA‐IR, homeostatic model assessment of insulin resistance; INSTI, integrase strand transfer inhibitor; NNRTI, non‐nucleoside reverse transcriptase inhibitor; NRTI, nucleoside reverse transcriptase inhibitor; PI, protease inhibitor; SPPB, Short Physical Performance Battery; TAF, tenofovir alafenamide.

### P397 Abstract Withdrawn

### P398 Implementation of a Multi‐Component mHealth Programme as Differentiated Service Delivery Model for People With HIV and Cardiometabolic Comorbidities

Grace C.Y. Lui ^1,2^, Rebecca KK Yuen^1^, Heather W.K. To^3^, Bonnie C.K. Wong^3^, Tracy H.Y. Ho^1^, Fion W.L. Luk^1^, Shui‐Shan Lee^2^, Candy N.S. Wong^2^

^1^Department of Medicine and Therapeutics, The Chinese University of Hong Kong, Hong Kong; ^2^S.H. Ho Research Centre for Infectious Diseases, The Chinese University of Hong Kong, Hong Kong; ^3^Integrated Treatment Centre, Department of Health, Hong Kong

**Background**: People with HIV (PWH) have earlier onset of cardio‐metabolic comorbidities. Integrated care for HIV and comorbidities is challenging due to resource constraints.

**Methods**: This is an ongoing implementation study to evaluate a Differentiated Service Delivery programme involving mHeatlh for PWH with comorbidities (Figure 1). Adult PWH with ≥1 cardiometabolic morbidities followed at an HIV clinic with suboptimal control were invited to participate in an online survey to indicate their willingness to participate in the programme (Figure 1). Participant acceptability of the programme, adherence to different components, retention rate and satisfaction were assessed. Service and client outcomes will be examined after completion of all participants’ 12‐month follow‐up.

**Results**: Among the 300 participants who participated in the survey, 130 (43.3%) were willing to participate in the programme. Acceptability of the programme was more likely in participants who were ≤50 years, male, received tertiary education, worked full‐time and perceived benefits of each programme component, and less likely in those with lower level of digital literacy (Table 1). Among the 130 participants who indicated “willing to participate” (age 53.9 ± 11.8 years, 92.1% male), 127 (97.7%) attended the Comorbidities Clinic for first assessment, 126 (96.9%) completed all complication screening procedures, 62 (47.7%) completed 12 months of follow‐up, 57 (43.8%) with ongoing follow‐up and 8 (6.2%) had withdrawn from the programme. Among the 212 scheduled teleconsultations, 201 (94.8%) proceeded as planned, while 11 (5.2%) were conducted as in‐person consultation at Comorbidities Clinic. Among the 182 scheduled teleconsultations in which co‐medications were prescribed, 77 (42.3%) chose to be collected at Comorbidities Clinic, 59 (32.4%) delivered to home, 24 (13.2%) at HIV clinic and 20 (11.0%) delivered to community pharmacy. Co‐medications were collected according to the chosen route in 177 (97.3%) episodes. Adherence rates for self‐monitoring blood pressure, glucose and body weight were 85.5%, 76.7% and 73.2%, respectively. The number of new diagnoses made were 53 per 100 person‐years follow‐up (mostly MASLD, albuminuria, diabetes or pre‐diabetes and hypertension). Changes in antiretroviral regimen were recommended for 5 participants due to renal or cardiovascular risk. 84.2% (48/57) of participants were satisfied with the programme at completion of follow‐up.

**Conclusions**: A multicomponent mHealth programme for management of cardiometabolic comorbidities in PWH was feasible and acceptable.

**FIGURE 1:**
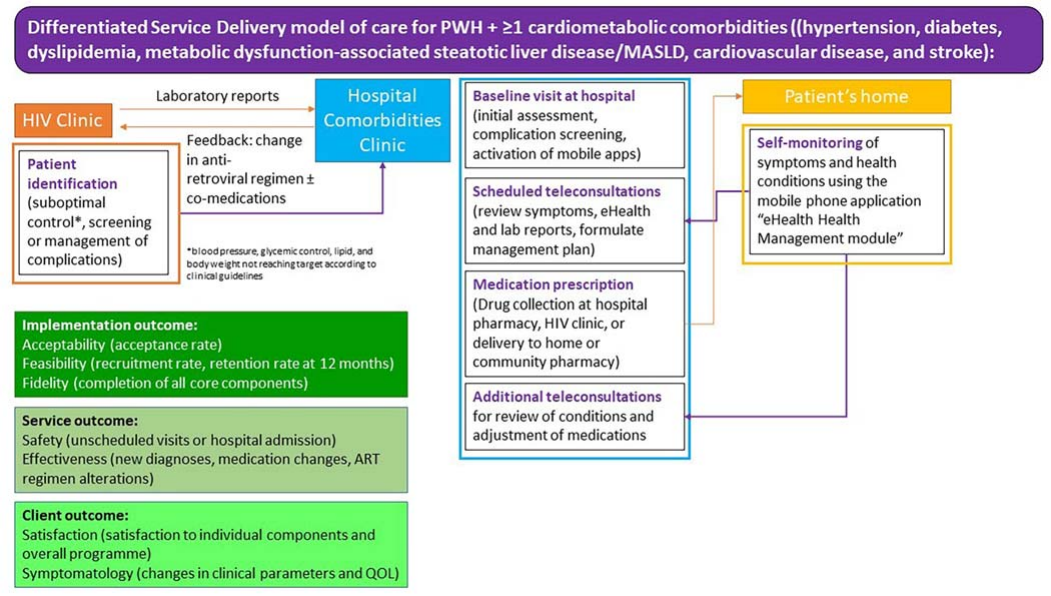
P398 | Components and outcome measures of a differentiated service delivery model involving mHealth for PWH and cardiometabolic comorbidities.

**TABLE 1:**
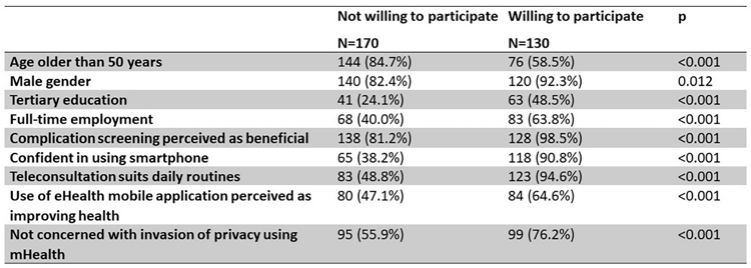
P398 | Characteristics and preferences of participants who were willing and reluctant to participate in the programme.

### P399 Sex‐Specific Patterns of Cardiovascular‐Kidney‐Metabolic Syndrome Progression in People With HIV

Jovana Milić^1,2^, Laura E. Biondi^3^, Lorenzo Salmoni ^4^, Alessandro Pecchi^3^, Matteo Menozzi^3^, Marianna Menozzi^5^, Sonia Generoso^1^, Gianluca Cuomo^5^, Giuseppe Mancini^5^, Cristina Mussini^1,2^, Giovanni Guaraldi^1,2^

^1^Department of Surgical, Medical, Dental and Morphological Sciences, University of Modena and Reggio Emilia, Modena, Italy; ^2^Modena HIV Metabolic Clinic, University of Modena and Reggio Emilia, Modena, Italy; ^3^University of Modena and Reggio Emilia, Modena, Italy; ^4^Department of Mental and Physical Health and Preventive Medicine, University of Campania “Luigi Vanvitelli”, Naples, Italy; ^5^Clinic for Infectious Diseases, University Hospital of Modena, Modena, Italy

**Background**: The cardiovascular‐kidney‐metabolic (CKM) framework links cardiometabolic risk, kidney disease and metabolic health in a patient‐centred model. People with HIV (PWH) have a higher prevalence of CKM syndrome than the general population, yet sex differences in CKM trajectories remain poorly understood. The aim of this study was to assess sex‐related differences in CKM stage distribution and transition between its stages among PWH.

**Methods**: We conducted a longitudinal observational study including PWH attending the Modena HIV Metabolic Clinic, Italy (2005 and 2026). CKM stages were defined as follows: stage 0, no metabolic risk factors; stage 1, early metabolic or anthropometric abnormalities; stage 2, metabolic or cardiovascular risk factors with established organ damage (ectopic fat, CKD, diabetes or hypertension); stage 3, additional subclinical/high‐risk cardiovascular disease, significant liver fibrosis or very high CV risk; stage 4, overt CVD events, end‐stage CKD, CAC score > 400 or solid organ transplantation. A multivariable Cox proportional hazards model was used to estimate the association between sex and progression to CKM stage ≥3.

**Results**: Among 5474 people with HIV, 1785 (32.6%) were women, median age was 51 years (IQR 44–59), time since HIV diagnosis, 20 (IQR 13–28) years, current CD4 count 652 cells/µL (IQR 464–881). HIV RNA viral load was undetectable in 96.3% of PWH. Women showed a higher proportion of stage 0 and 1 CKM compared with men (2.6% vs. 1.5% and 37.4% vs. 23.3%, respectively), whereas men showed a higher prevalence of stage 3 and 4 (33.6% vs. 22.0% and 12.9% vs. 6.4%, *p* < 0.001). Across calendar periods men had a consistently high burden of advanced CKM stages; however, women showed a marked increase over time (Figure 1). Female sex was independently associated with a lower risk of progression to CKM stage ≥3 (Table 1).

**Conclusions**: Men showed a higher overall burden of advanced CKM syndrome and a greater risk of progression to CKM stage ≥3. However, the temporal increase in advanced CKM stages was proportionally greater among women, suggesting a progressive narrowing of the sex gap. These findings support the need for sex‐tailored CKM prevention strategies in PWH.

**FIGURE 1:**
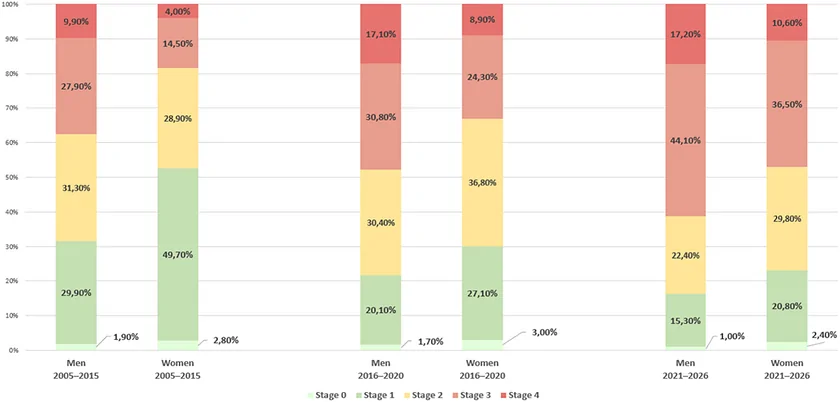
P399 | Temporal distribution of CKM stages according to sex.

**TABLE 1:**
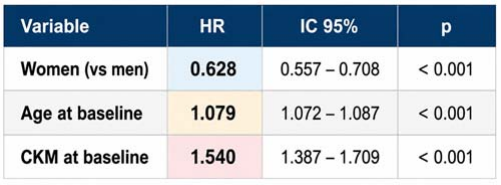
P399 | Factors associated with progression to advanced CKM stage.

### P400 Longitudinal Changes and Risk Factors of Steatotic Liver Disease in People With HIV in Switzerland

Eloïse Magnenat ^1^, Nadia Meyer^1^, Annalisa Berzigotti^2,3^, Isabella C. Schoepf^3,4^, David Haerry^5^, Huldrych F. Günthard^6,7^, Philip E. Tarr^4,8^, Andri Rauch^1^, Bernard Surial^1^, Gilles Wandeler^1,9^, Anders Boyd^1^, Carlotta Mondoka^1,9^, on behalf of Swiss HIV Cohort Study

^1^Department of Infectious Diseases, Inselspital, Bern University Hospital, University of Bern, Bern, Switzerland; ^2^Department for Visceral Surgery and Medicine, Inselspital, Bern University Hospital, University of Bern, Bern, Switzerland; ^3^Hepatology, Department of BioMedical Research, University of Bern, Bern, Switzerland; ^4^University Center for Internal Medicine, Kantonsspital Baselland, University of Basel, Bruderholz, Switzerland; ^5^Positive Council Switzerland, Bern, Switzerland; ^6^Department of Infectious Diseases and Hospital Epidemiology, University Hospital Zurich, University of Zurich, Zurich, Switzerland; ^7^Institute of Medical Virology, University of Zurich, Zurich, Switzerland; ^8^Department of Infectious Diseases, Kantonsspital Baselland, University of Basel, Bruderholz, Switzerland; ^9^Institute of Social and Preventive Medicine, University of Bern, Bern, Switzerland

**Background**: Steatotic liver disease (SLD) affects up to 50% of people with HIV (PWH). However, longitudinal data on the progression and regression of steatosis in SLD, and their determinants, remain scarce.

**Methods**: Between November 2019 and December 2025, participants enrolled in the Swiss HIV Cohort Study (SHCS) at Bern University Hospital were consecutively recruited and underwent serial vibration‐controlled transient elastography (VCTE) with controlled attenuation parameter (CAP). Severe steatosis was defined as a CAP ≥280 dB/m. Using data from participants with ≥2 VCTE assessments performed ≥12 months apart, transitions from no or mild steatosis (S0–S2) to severe steatosis (S3) (i.e. progression) and from severe steatosis to no or mild steatosis (i.e. regression), along with their determinants, were estimated with a time‐homogeneous, continuous‐time Markov model. The transition intensities (TI; i.e. model‐estimated rates of transition between states, equivalent to incidence rates) and their hazard ratios (HRs) were estimated using maximum likelihood methods.

**Results**: Among 576 participants enrolled, 454 (78.8%) participants had ≥2 VCTE assessments and were included. Among these, 121 (26.6%) were female, median age was 52 years (interquartile range 43–59), 229 (50.4%) were overweight, and 148 (32.6%) had severe steatosis at baseline. Over a median follow‐up of 3.0 years (IQR 2.0–4.7; 1453 person‐years), participants underwent a median of three VCTE assessments (IQR 2–4). The evolution of steatosis grades over time is depicted in Figure 1. The TI for progression from no or mild steatosis to severe steatosis was 0.13/person‐year (95% CI, 0.11–0.17) and was independently associated with dyslipidaemia (HR 2.71; 95% CI: 1.36–5.41). The TI for regression from severe steatosis to no or mild steatosis was 0.34/person‐year (95% CI, 0.28–0.43). Higher regression rates were associated with Black ethnicity (HR 3.74; 95% CI: 1.41–9.93), whereas lower regression rates were associated with being overweight (HR 0.47; 95% CI: 0.27–0.81).

**Conclusions**: In PWH, the presence of SLD changed over time, with regression occurring more frequently than progression when comparing model‐estimated transition intensities. Dyslipidaemia was associated with progression, whereas being overweight was associated with lack of regression, highlighting the importance of metabolic risk factor management in PWH.

**FIGURE 1:**
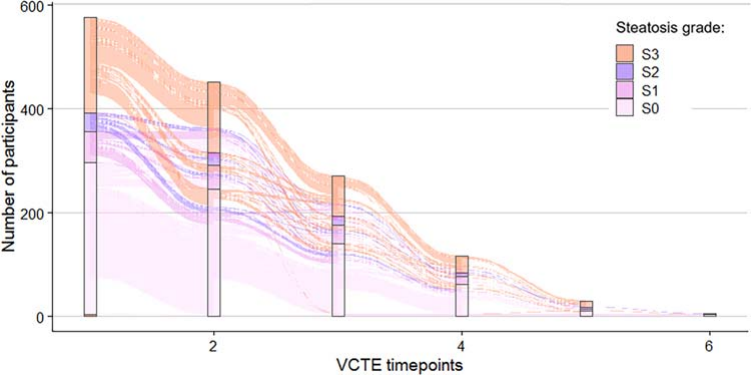
P400 | Sankey graph showing changes of steatotic liver disease (SLD) over vibration controlled transient elastography (VTCE) assessments. Bars represent the number of participants with severe (S3) steatosis compared to those with no (S0) steatosis, mild (S1) or moderate (S2) steatosis at the first VTCE and each follow‐up assessment. Flows represent transitions between steatosis grades over time and are coloured by baseline steatosis grade. Participants with only a single VCTE assessment were excluded from the longitudinal transition analysis.

### P401 Evaluation of Weight at Week 48 in a Phase 3 Study in Adults Initiating Treatment for HIV‐1 With Doravirine/Islatravir (100/0.25 mg) Versus Bictegravir/Emtricitabine/Tenofovir Alafenamide

Jürgen Rockstroh ^1^, Gary Sinclair^2^, Brenda Crabtree Ramirez^3^, Sheetal Kassim^4^, Jean‐Michel Molina^5^, Anchalee Avihingsanon^6^, Dorothy McCoy^7^, Yayun Xu^7^, Stephanie Klopfer^7^, Michelle C. Fox^7^, Mary Pisculli^7^

^1^Department of Medicine I, University Hospital Bonn, Bonn, Germany; ^2^Clinical Research, Prism Health North Texas, Dallas, TX, USA; ^3^Departamento de Infectología, Instituto Nacional de Ciencias Médicas y Nutrición, Mexico City, Mexico; ^4^Department of Medicine, Desmond Tutu HIV Centre, University of Cape Town, Cape Town, South Africa; ^5^Département des Maladies Infectieuses, University of Paris Cité, Paris, France; ^6^Infectious Diseases Research, HIVNAT, Thai Red Cross AIDS and Infectious Diseases Research Centre and Center of Excellence in Tuberculosis, Bangkok, Thailand; ^7^MRL, Merck & Co., Inc., Rahway, NJ, USA

**Background**: Antiretroviral therapy is associated with weight gain. In a phase 3 study in adults initiating treatment with doravirine/islatravir (DOR/ISL) or bictegravir/emtricitabine/ tenofovir alafenamide (BIC/FTC/TAF), the mean increase in weight at week (W) 48 was similar between treatment groups [1]. Here, we evaluated changes in weight by baseline viral load (VL), CD4+ T‐cell count (CD4) and body mass index (BMI).

**Methods**: Treatment‐naïve adults with HIV‐1 were randomized 1:1 to daily oral DOR/ISL (100/0.25 mg) or BIC/FTC/TAF (50/200/25 mg) in MK‐8591A‐053 (NCT05705349). This post‐hoc analysis assessed mean change in weight from baseline by baseline VL (HIV‐1 RNA ≤100,000/> 100,000 copies/mL) and CD4 (≥200/< 200 cells/mm^3^), and BMI and the percent of participants with a ≥5% and ≥10% increase in weight by VL and CD4 across and within treatment groups at W48.

**Results**: 536 participants were treated: DOR/ISL *n* = 269, BIC/FTC/TAF *n* = 267. At baseline, mean age, weight and BMI were 34 years, 75 kg and 26 kg/m^2^; 75% of participants were male at birth, 86% with HIV‐1 diagnosed < 3 months, 37% had a VL > 100,000 and 17% had a CD4 < 200 cells/mm^3^. Less weight gain was observed with DOR/ISL versus BIC/FTC/TAF in participants with baseline VL ≤100,000 and CD4 ≥200 cells/mm^3^ (difference −1.4 kg, 95% CI: −2.6, −0.1) (Figure 1). With DOR/ISL, participants with a VL > 100,000 and CD4 < 200 cells/mm^3^ gained 8.8 kg versus 1.8 kg for those with VL ≤100,000 and CD4 ≥200 cells/mm^3^; with BIC/FTC/TAF, weight gain was 6.7 versus 3.2 kg. No difference between groups was identified for each baseline BMI category. In both groups, participants with a higher VL and lower CD4 within BMI categories generally had greater mean weight gain. More participants had a ≥5% and ≥10% increase in weight with a VL > 100,000 and CD4 < 200 cells/mm^3^ versus those with a VL ≤100,000 and CD4 ≥200 cells/mm^3^ in both groups.

**Conclusions**: The greatest weight gain in both treatment groups was in participants with advanced HIV (VL > 100,000 and CD4 < 200 cells/mm^3^), likely associated with a return‐to‐health. In participants with less advanced disease (VL ≤100,000 and CD4 ≥200 cells/mm^3^), DOR/ISL may be associated with less weight gain versus BIC/FTC/TAF.

**Reference**

1. Rockstroh JK, Kassim S, Paredes R, et al. Fixed‐dose daily doravirine (100 mg) with islatravir (0·25 mg) versus bictegravir, emtricitabine, and tenofovir alafenamide for initial HIV‐1 therapy: 48‐week results of a phase 3, randomised, controlled, double‐blind, non‐inferiority trial. *Lancet HIV*. Published online February 25, 2026. doi:10.1016/S2352‐3018(26)00033‐0.

**FIGURE 1:**
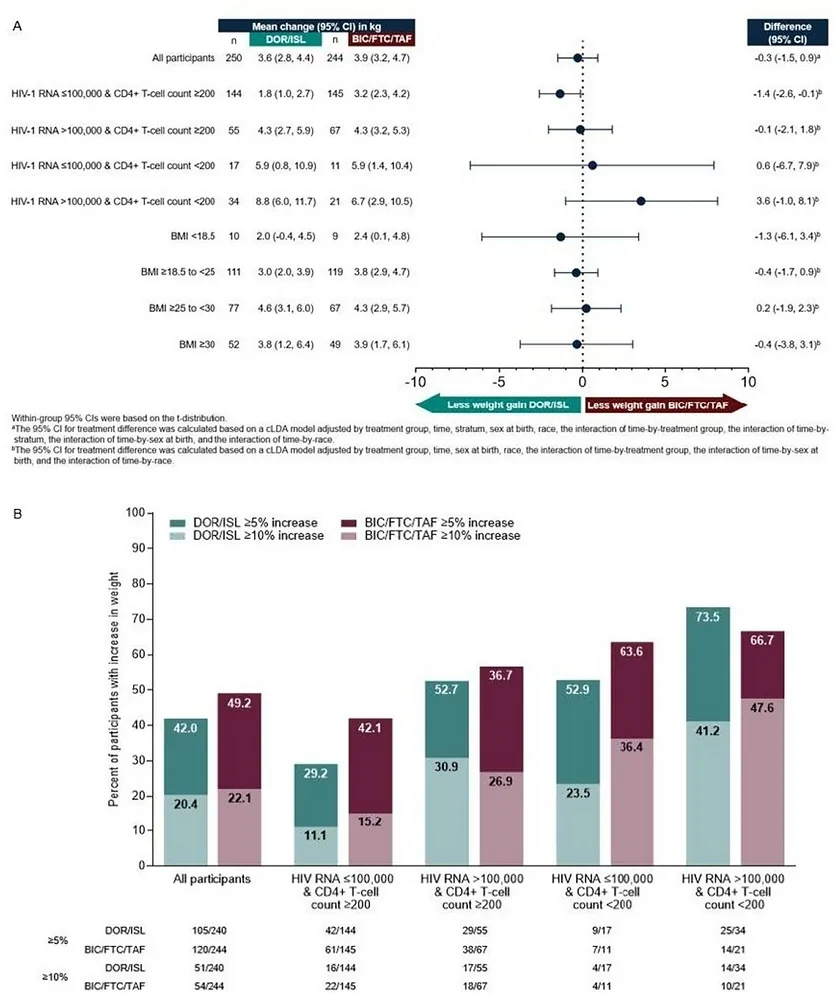
P401 | Mean change in weight from baseline by treatment group, HIV‐1 RNA and CD4+ T‐cell count, and baseline BMI (A) and participants with ≥5% and ≥10% increase in weight by treatment group and HIV‐1 RNA and CD4+ T‐cell count (B) at week 48.

### P402 Glucagon‐Like Peptide‐1 Receptor Agonists and Musculoskeletal Outcomes: A Longitudinal Study in People Living With HIV

Marianna Menozzi ^1^, Jovana Milić^2^, Alessandro Pecchi^3^, Gianluca Cuomo^1^, Giuseppe Mancini^1^, Uberto Calvi^2^, Filippo Calandra Buonaura^2^, Cristina Mussini^1,2^, Giovanni Guaraldi^2^

^1^Clinic of Infectious Disease, University Hospital of Modena, Modena, Italy; ^2^Clinic of Infectious Disease, University of Modena and Reggio Emilia, Modena, Italy; ^3^University of Modena and Reggio Emilia, Modena, Italy

**Background**: Glucagon‐like peptide‐1 receptor agonists (GLP1‐RAs) are increasingly prescribed in people with HIV (PWH) to treat clinical obesity. However, the obtained weight loss has raised concerns regarding potential adverse effects on skeletal muscle and bone health, particularly in PWH, who are at increased risk of sarcopenia and osteoporosis. We evaluated longitudinal changes in body composition, muscle function and bone mineral density (BMD) following GLP1‐RAs and specifically subcutaneous semaglutide treatment.

**Methods**: A longitudinal observational study including PWH aged ≥18 who attended at least two times the Modena HIV Metabolic Clinic (Italy) between 2023 and 2026. GLP1‐RAs users were propensity score matched (1:3) with unexposed controls according to age, sex and baseline cardiometabolic (CKM) status. Two matched cohorts were analysed: all GLP1‐RAs (semaglutide, dulaglutide, tirzepatide or liraglutide) users and a semaglutide‐only subgroup. Outcomes included DXA‐derived body composition, appendicular skeletal muscle index (ASMI), muscle function (grip strength, gait speed, Short Physical Performance Battery [SPPB], chair‐stand performance) and BMD at lumbar spine, femoral neck, and total body.

**Results**: Thirty‐seven PWH on GLP‐1 RAs (18 on subcutaneous semaglutide) were matched 1:3 to non‐users; treated participants were predominantly male, with long‐standing HIV and lower nadir CD4 (Table 1). Total and appendicular lean mass and ASMI were preserved in both groups, the only significant lean loss being confined to the trunk of semaglutide users (Δ −0.97 kg), a less specific muscle compartment (Figure 1A,B). Grip strength was maintained in the semaglutide subgroup while controls declined, and gait speed improved; in the pooled class, grip fell comparably in both arms, consistent with an age‐related effect (Figure 1C,D). BMD was stable at all sites in both groups (Figure 1E,F). CTX rose during treatment but did not differ from non‐users, with no change in other turnover markers.

**Conclusions**: In this propensity score‐matched cohort of PWH, GLP‐1 RA therapy, particularly subcutaneous semaglutide, preserved muscle mass, physical function and bone health. Trunk lean mass loss was not accompanied by any functional or densitometric decline. The discordance between reduced trunk lean mass and preserved muscle function may suggests that DXA‐derived changes not reflect true loss of skeletal muscle mass and function.

**FIGURE 1:**
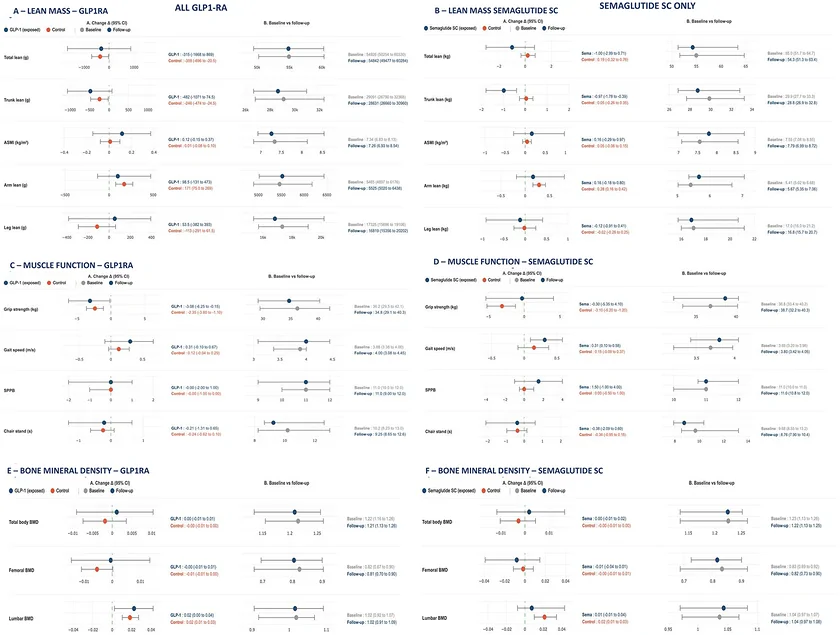
P402 | Changes in lean mass, muscle function and bone mineral density across GLP1‐RA treatment group (exposed vs. controls) and period (baseline vs. follow‐up).

**TABLE 1:**
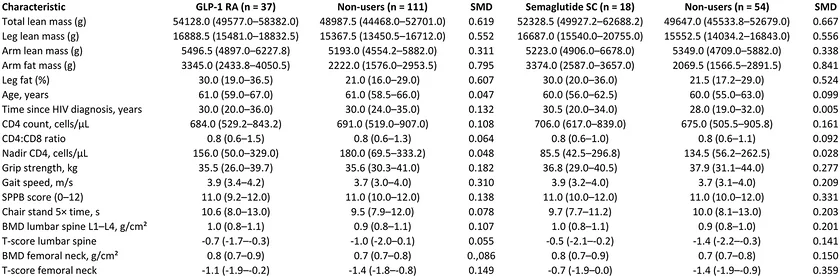
P402 | Baseline characteristics: GLP‐1 RA and semaglutide SC users versus matched non‐users.

### P403 Characterization of Metabolic Liver Disease in People Living With HIV Using Liver Elastography: A Prevalence Study

Claudia Maya Salazar ^1^, Bianca P. Aguilar Rodea^2^, Jezer Lezama Mora^3^, Luis Eduardo Cázerez Villareal^4^, Eva Juárez Hernández^4^, Iván López Méndez^4^

^1^Internal Medicine Unit, Medica Sur Clinic & Foundation, Mexico City, Mexico; ^2^Infectology Unit, Hospital General Manuel Gea González, Mexico City, Mexico; ^3^Clínica Condesa, Mexico City, Mexico; ^4^Translational Research Unit, Medica Sur Clinic & Foundation, Mexico City, Mexico

**Background**: Metabolic dysfunction‐associated steatotic liver disease (MASLD) is now the most prevalent chronic liver disease worldwide. Reported prevalence among people living with HIV (PLWH) ranges from 13% to over 55%, depending on diagnostic modality. We aimed to estimate the prevalence of hepatic steatosis and fibrosis in adults with HIV monoinfection and to identify associated factors.

**Methods**: We conducted a prospective cross‐sectional study of adults with HIV monoinfection in Mexico City. Liver steatosis and fibrosis were evaluated by transient elastography (TE). Additionally, non‐invasive scores were calculated: for liver steatosis the Hepatic Steatosis Index (HSI), triglyceride‐glucose index (TyG) and TyG‐waist circumference (TyGWC); for liver fibrosis the FIB4 and APRI scores. The diagnostic accuracy of each was evaluated with TE as the reference.

**Results**: 213 patients were included; 85.9% (*n* = 183) were male, with a median age of 46 (38–55) years and a median body mass index (BMI) of 26 (23–29.2) kg/m^2^; 87.8% (*n* = 187) of patients had an undetectable viral load. Most of the patients (59.6%, *n* = 127) were on antiretroviral regimens containing integrase plus non‐nucleoside reverse transcriptase inhibitors. MASLD prevalence was 26.8% (*n* = 57), while the prevalence of clinically significant fibrosis (> 8.1 kPa) was 0.9% (*n* = 2) (Figure 1). In the main multivariable model, BMI was an independent predictor of MASLD (OR 1.3 per unit; 95% CI: 1.1–1.4; *p* < 0.001). Antiretroviral regimens did not show an independent association. After adjusting for antiretroviral treatment duration, waist circumference (WC) also emerged as an independent predictor (OR 5.2; 95% CI: 2.5–10.5; *p* < 0.001). In the analysis of the diagnostic accuracy of non‐invasive scores, HSI and TyGWC had sensitivities of 0.78 and 0.82, with equivalent AUCs (ΔAUC −0.008; *p* = 0.809), and both were superior to TyG (*p* = 0.003 and *p* < 0.001).

**Conclusions**: In PLWH, the prevalence of steatosis is similar to that reported for the general population. A per‐unit increase in BMI and WC above their respective thresholds is an independent predictor of MASLD. HSI or TyGWC are non‐invasive scores that offer good diagnostic performance for evaluating liver steatosis and are accessible alternatives when TE is unavailable.

**FIGURE 1:**
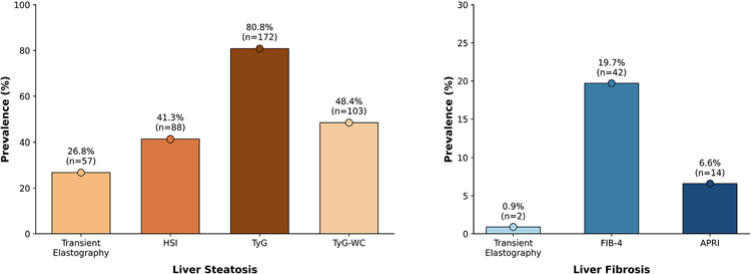
P403 | Prevalence of liver steatosis and liver fibrosis by non‐invasive methods in PLWH.

### P404 Incidence of Metabolic Syndrome in People With HIV Who Start Dolutegravir Based‐Regimen Compared With Bictegravir Based‐Regimen After 96 Weeks (MICTLAN Study)

Mara Rodriguez Evaristo ^1^, José Antonio Mata Marín^1^, Ana Luz Cano Díaz^1^, Emily Itzel Pecero García^1^, Gloria Elizabeth Salinas Velázquez^2^, Alberto Chaparro Sánchez^1^, Ericka Nelly Pompa Mera^3^, Betzahida Meneses Cisneros^4^, Jesús Enrique Gaytán Martínez^1^

^1^Infectious Diseases Department, Hospital de Infectología “La Raza” National Medical Center, Instituto Mexicano del Seguro Social, Mexico City, Mexico; ^2^Radiology Department, Hospital de Infectología “La Raza” National Medical Center, Instituto Mexicano del Seguro Social, Mexico City, Mexico; ^3^Research Unit, Hospital de Infectología “La Raza” National Medical Center, Instituto Mexicano del Seguro Social, Mexico City, Mexico; ^4^Electrocardiography Department, Hospital de Infectología “La Raza” National Medical Center, Instituto Mexicano del Seguro Social, Mexico City, Mexico

**Background**: Evidence suggests that people living with HIV (PWH) initiating integrase strand transfer inhibitor (INSTI) regimens are more likely to experience weight gain and metabolic syndrome (MetS) compared to other antiretroviral regimens. This study aimed to evaluate the incidence of MetS at 96 weeks, using the ATP III criteria, in PWH starting antiretroviral therapy (ART) with dolutegravir (DTG)‐based versus bictegravir (BIC)‐based regimens.

**Methods**: A randomized, clinical trial open‐label was conducted in men living with HIV who initiated ART, randomized to either BIC/FTC/TAF or DTG/3TC/ABC. Weight, blood pressure and waist circumference were measured during routine clinical visits, with bioelectrical impedance analysis performed at baseline, 48 and 96 weeks. Laboratory tests were conducted concurrently. MICTLAN Study trial: Clinical Trials.gov NCT06629480.

**Results**: 311 PWH were enrolled. At the time of analysis, 232 participants completed 96 weeks of follow‐up. The median age was 24 years (IQR 20–31), with a baseline viral load of 21,603 copies/mL (IQR 7287–89,294). The overall incidence of MetS was 19 cases (6.1%): 8 (5.2%) in the BIC/FTC/TAF group and 11 (6.9%) in the DTG/3TC/ABC group (*p* = 0.690). Median weight gain was 2.9 kg (IQR 1.9–3.9) for BIC/FTC/TAF and 2.3 kg (IQR 1.3–3.7) for DTG/3TC/ABC (*p* = 0.645). Hypertriglyceridemia occurred in 17 cases (11.1%) in the BIC/FTC/TAF group and 14 (8.8%) in the DTG/3TC/ABC group (*p* = 0.813). During the 2 years, 21% of individuals in the BIC/TAF/FTC group experienced a > 5% weight gain, compared to 17% in the DTG/ABC/3TC group (*p* = 0.1). Additionally, 13% of the BIC/TAF/FTC group had a > 10% weight gain, versus 10% in the DTG/ABC/3TC group (*p* = 0.72) (Table 1).

**Conclusions**: This Mexican trial demonstrates that the incidence of MetS was high but comparable between groups. Second‐generation INSTI‐based regimens were effective and well‐tolerated in this population.

**TABLE 1:**
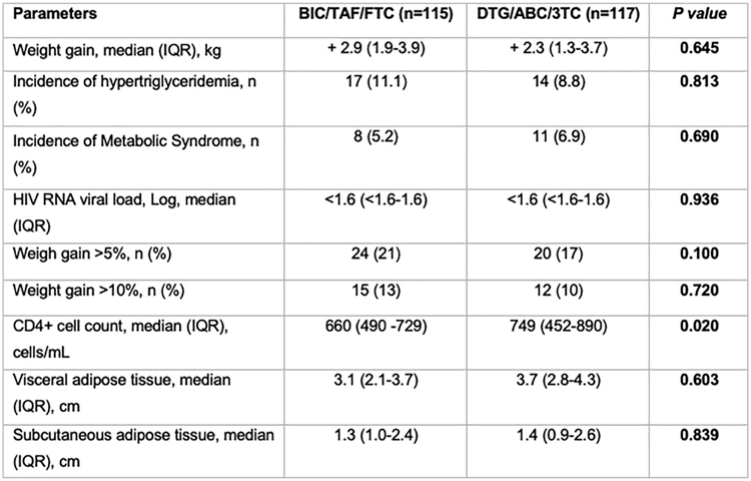
P404 | Metabolic characteristics of the population at 48 weeks.

### P405 Long‐Term Progression of Hepatic Steatosis and Fibrosis in People With HIV: A 5‐Year Longitudinal Cohort Study

Wei Xu, Wei Song, Li Liu, Renfang Zhang, Jun Chen, Yinzhong Shen

Department of Infection and Immunity, Shanghai, China

**Background**: Chronic liver disease has become an important comorbidity in people with HIV (PWH) in the antiretroviral therapy era. We investigated the incidence and predictors of hepatic steatosis and fibrosis progression over 5 years using serial transient elastography.

**Methods**: PWH who underwent baseline transient elastography between December 2019 and April 2020 were enrolled in a retrospectively constructed cohort with prospective follow‐up until February 2026. Steatosis progression was defined as incident steatosis (controlled attenuation parameter [CAP] ≥248 dB/m) or progression to severe steatosis (CAP ≥292 dB/m). Fibrosis progression was defined as incident significant fibrosis (liver stiffness measurement [LSM] ≥7.1 kPa) or progression to cirrhosis (LSM ≥12.5 kPa). Independent predictors were identified using multivariable Cox regression.

**Results**: Among 315 PWH (Figure 1), median follow‐up was 67.3 months. Hepatic steatosis progressed in 83 patients (30.4%; 55.7/1000 person‐years), and fibrosis progressed in 47 (16.9%; 30.8/1000 person‐years). Baseline MASLD independently predicted fibrosis progression (adjusted hazard ratio [aHR] 3.83, 95% confidence interval [CI] 1.49–9.87; *p* = 0.005), but not steatosis progression. Weight change rate independently predicted steatosis progression (aHR 1.01, 95% CI: 1.00–1.02; *p* = 0.01) and fibrosis progression (aHR 1.02, 95% CI: 1.01–1.03; *p* < 0.001). INSTI use was independently associated with de novo MASLD (aHR 1.88, 95% CI: 1.05–3.36; *p* = 0.03).

**Conclusions**: In PWH, MASLD drives fibrosis progression, while weight change is a key modifiable predictor of both steatosis and fibrosis. INSTI use may increase the risk of new‐onset MASLD.

**FIGURE 1:**
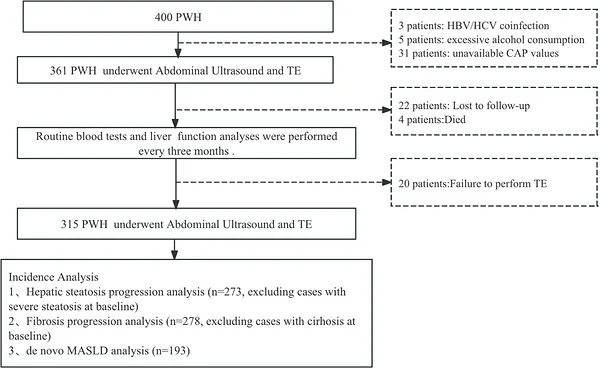
P405 | Flowchart displaying the selection of study participants.

### P406 From Primary to Secondary Cardiovascular Prevention Among People Living With Multidrug‐Resistant HIV: Missed Opportunities in the Statin Cascade of Care in the PRESTIGIO Registry

Tommaso Clemente ^1^, Dario Diana^2,3^, Riccardo Lolatto^1^, Giovanni Cenderello^4^, Giulia C. Marchetti^5^, Simona Di Giambenedetto^6,7^, Golnaz Torkjazi^2^, Daniela Messeri^8^, Giordano Madeddu^9^, Vincenzo Malagnino^10,11^, Giovanni Festa^2^, Stefano Rusconi^12,13^, Laura Comi^14^, Vincenzo Spagnuolo^1,2^, on behalf of PRESTIGIO Study Group

^1^Infectious Diseases, IRCCS San Raffaele Scientific Institute, Milan, Italy; ^2^Vita‐Salute San Raffaele University, Milan, Italy; ^3^Infectious Diseases Unit, University of Naples “Federico II”, Naples, Italy; ^4^Infectious Diseases Department of Graduated Medical Care, Sanremo Hospital, Sanremo, Italy; ^5^Infectious Diseases Unit, ASST Santi Paolo e Carlo, University of Milano, Milan, Italy; ^6^Infectious Diseases Unit, Fondazione Policlinico Universitario Agostino Gemelli IRCCS, Rome, Italy; ^7^Catholic University of the Sacred Heart, Rome, Italy; ^8^Infectious Diseases Unit, San Jacopo Hospital, Azienda USL Toscana Centro, Pistoia, Italy; ^9^Unit of Infectious Diseases, Department of Medicine, Surgery and Pharmacy, Sassari, Italy; ^10^Infectious Diseases, Department of Systems Medicine, Tor Vergata University, Rome, Italy; ^11^Infectious Disease Clinic, Policlinico Tor Vergata, Rome, Italy; ^12^Infectious Diseases Unit, Ospedale Civile di Legnano ASST Ovest Milanese, Legnano, Italy; ^13^Department of Biomedical and Clinical Sciences, Università Degli Studi di Milano, Milan, Italy; ^14^UOC Infectious Diseases, ASST Papa Giovanni XXII, Bergamo, Italy

**Background**: Among people living with HIV, multidrug resistance is associated with a > 2‐fold increase in cardiovascular risk [1]. Therefore, beyond virological suppression, optimal lipid management is crucial in this fragile population. The aim of this study was to evaluate the statin cascade of care in people living with 4‐class drug‐resistant (4DR) HIV.

**Methods**: Cross‐sectional study including ≥40‐year‐old individuals with HIV resistant to nucleoside reverse transcriptase inhibitors (NRTIs), non‐nucleoside reverse transcriptase inhibitors (NNRTIs), protease inhibitors (PIs) and integrase strand transfer inhibitors (INSTIs) from the PRESTIGIO Registry [2] and with ≥1 available low‐density lipoprotein‐cholesterol (LDL‐C) measurement from 01 January 2024 onwards. The statin cascade of care included statin use and achievement of predefined LDL‐C targets: < 70 mg/dL in individuals without prior major adverse cardiovascular events (MACEs; primary prevention) and < 55 mg/dL in those with a history of MACEs (secondary prevention). Evaluation was performed on the date of the last available LDL‐C.

**Results**: Among 295 people living with 4DR HIV from the PRESTIGIO Registry, 200 were ≥40‐year‐old and had ≥1 visit from 2024 onwards. Of these, 189 (94.5%) had ≥1 LDL‐C measurement and were included in the analysis. Median age was 61.9 (interquartile range 58.5–65.2) years, 137 (72.5%) male at birth, ART duration 29.3 (27.7–31.9) years, and 159 (84.1%) virologically suppressed < 50 copies/mL (Table 1). Statin therapy was reported in 96/189 (50.8%) individuals: 75.0% as monotherapy, 25.0% with ezetimibe. Overall, only 22/189 (11.6%) achieved LDL‐C targets (Figure 1). On primary prevention 72/162 (44.4%) were receiving statins, but only 12/72 (16.4%) achieved LDL‐C target. Among the 90 individuals not receiving statins, 8 (8.9%) maintained LDL‐C < 70 mg/dL (*p* = 0.155). On secondary prevention, 24/27 (88.9%) were receiving statins, yet only 2/24 (8.3%) achieved LDL‐C target. None of the 3 individuals not receiving statins achieved LDL‐C < 55 mg/dL.

**Conclusions**: In this nationwide cohort of people living with 4DR HIV, substantial gaps were observed in both statin use and LDL‐C target achievement. The low proportion of individuals reaching guideline‐recommended LDL‐C goals despite lipid‐lowering therapy, even among those with prior cardiovascular events, highlights cardiovascular prevention as a major unmet need beyond virological suppression in the population with multidrug‐resistant HIV.

**References**

1. Clemente T, Diotallevi S, Minisci D, et al. Two‐fold increased risk of cardiovascular events in people with MDR HIV: A matched cohort analysis with data from the PRESTIGIO registry. J Antimicrob Chemother. 2025;80(3):731–737.

2. Clemente T, Galli L, Lolatto R, et al. Cohort profile: PRESTIGIO, an Italian prospective registry‐based cohort of people with HIV‐1 resistant to reverse transcriptase, protease and integrase inhibitors. BMJ Open. 2024;14(2):e080606.

**FIGURE 1:**
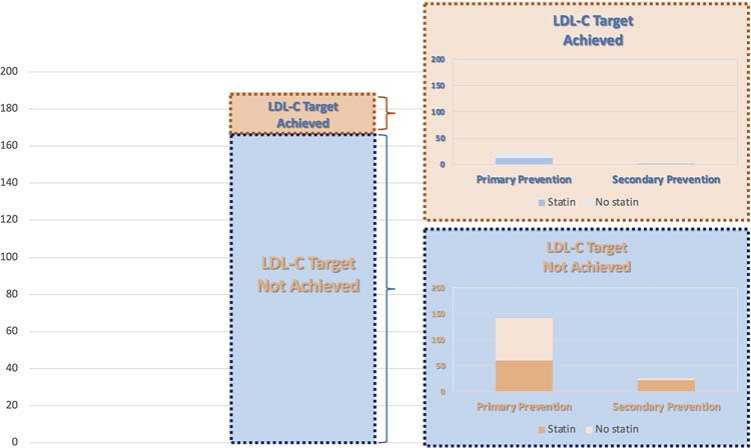
P406 | Statin cascade of care in people living with 4‐class drug‐resistant HIV included in the analysis.

**TABLE 1:**
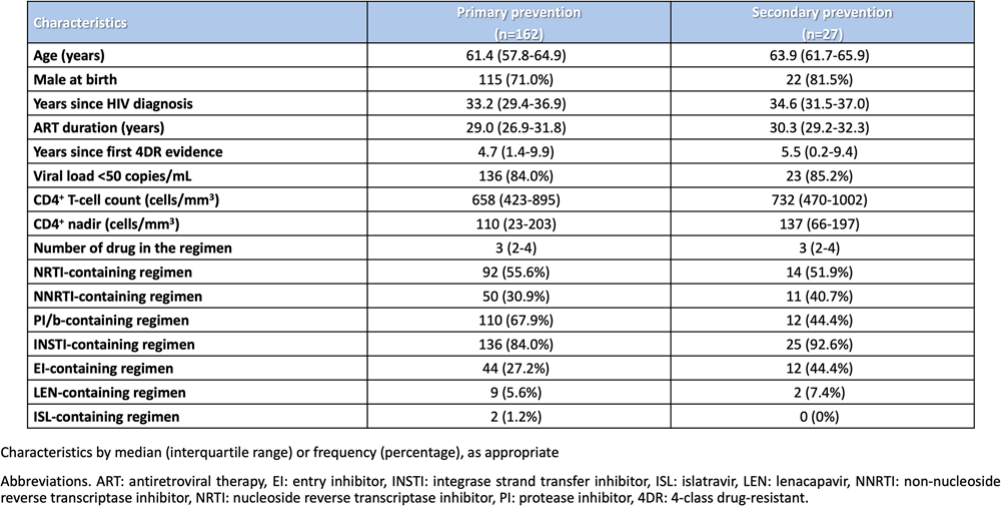
P406 | Characteristics of people living with 4‐class drug‐resistant HIV included in the analysis.

### P407 Is Menopause Really a Cardiovascular Turning Point in Women With HIV?

Laura E. Biondi ^1^, Filippo Calandra Buonaura^2^, Marianna Menozzi^3^, Cristina Mussini^2,3^, Giovanni Guaraldi^2,3^, Jovana Milić^2,3^

^1^School of Medicine, University of Modena and Reggio Emilia, Italy, Modena, Italy; ^2^Department of Surgical, Medical, Dental and Morphological Sciences, University of Modena and Reggio Emilia, Italy, Modena, Italy; ^3^Modena HIV Metabolic Clinic, University of Modena and Reggio Emilia, Modena, Italy, Modena, Italy

**Background**: Menopause is recognized as a period of accelerated cardiovascular (CV) risk in the general population, driven by oestrogen withdrawal and adverse metabolic changes. Women living with HIV (WLWH) experience menopause approximately 3 years earlier than women without HIV and enter midlife with persistent immune activation, chronic inflammation, antiretroviral‐related metabolic alterations and an increased CV disease burden. We investigated the impact of menopause on CV risk factors, preventive interventions and cardiometabolic‐kidney‐metabolic (CKM) syndrome in WLWH.

**Methods**: We conducted a longitudinal observational study of ART‐experienced people with HIV followed at the Modena HIV Metabolic Clinic (2005–2026). Women with at least one observation either in pre‐ and post‐menopause were included. Menopause was defined as self‐reported amenorrhea ≥12 months or age ≥49 years when menopausal status was unavailable). CV factors, preventive measures and CKM stage (0–4) were compared between groups.

**Results**: Among 1785 WLWH (229 present in both periods, 868 in pre‐menopause only and 688 in post‐menopause only), post‐menopausal women showed a markedly greater cardiometabolic burden, with CKM stage 3–4 increasing from 11.8% to 44.6% (*p* < 0.001). Post‐menopausal women had lower rates of current smoking (35.3% vs. 50.0%) and higher use of statins (34.1% vs. 6.9%). Despite this more proactive preventive profile, LDL‐C target achievement remained similarly suboptimal in both groups (24.1% vs. 25.6%) (Table 1). The number of CV risk factors (Figure 1A) and CV preventive measures (Figure 1B) differed according to menopause status (*p* < 0.001).

**Conclusions**: Although menopause was associated with an increase in overt cardiometabolic burden, our findings suggest that cardiovascular vulnerability in WLWH is established before the menopausal transition rather than initiated by it. Menopause may, therefore, act less as the biological onset of cardiovascular risk than as a care‐delivery turning point, when pre‐existing risk is more readily recognized and preventive care more consistently implemented, reflecting both greater clinical attention at midlife and broader improvements in HIV management over time. Yet a persistent gap in lipid control remains, supporting earlier, sustained cardiovascular prevention throughout routine HIV care in women rather than care concentrated around menopause.

**FIGURE 1:**
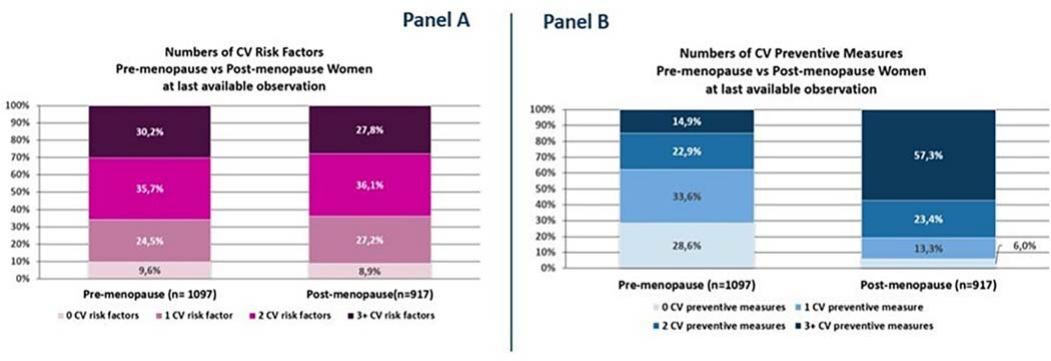
P407 | Number of CV risk factors (A) and CV preventive measures (B) in pre‐ versus post‐menopausal women.

**TABLE 1:**
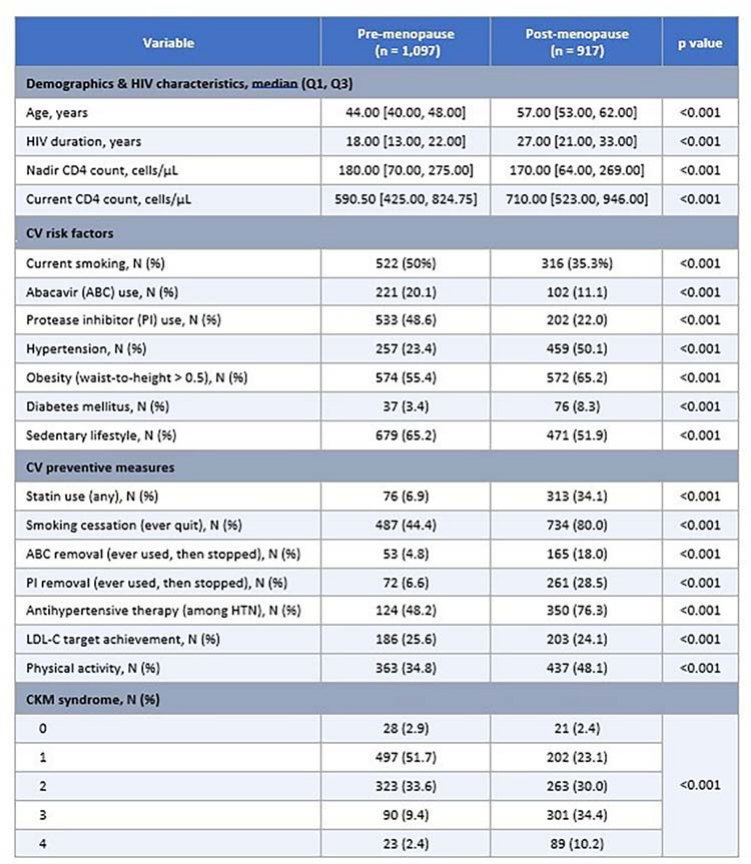
P407 | CV risk and prevention: pre‐menopause versus post‐menopause women.

### P408 Lower Incidence of TyG‐Defined Insulin Resistance After Boosted Protease Inhibitor‐to‐Dolutegravir Switch in People With HIV at High Cardiovascular Risk: NEAT‐022

Abiu Sempere ^1^, Lambert Assoumou^2^, Ana González‐Cordón^1^, Laura Waters^3^, Stefano Rusconi^4^, Pere Domingo^5^, Mark Gompels^6^, Stéphane De Wit^7^, François Raffi^8^, Christoph Stephan^9^, Mar Masía^10^, Jürgen Rockstroh^11^, Christine Katlama^12^, Georg M. Behrens^13^, Graeme Moyle^14^, Margaret Johnson^15^, Julie Fox^16^, Hans‐Jürgen Stellbrink^17^, Giovanni Guaraldi^18^, Eric Florence^19^, Stefan Esser^20^, José M. Gatell^21^, Anton Pozniak^22^, Esteban Martínez^1^, on behalf of NEAT‐022 Study Group

^1^Hospital Clínic‐IDIBAPS, University of Barcelona, Barcelona, Spain; ^2^Département d'Epidémiologie, Paris, France; ^3^Global HIV Medical Affairs, London, UK; ^4^Ospedale Luigi Sacco, Milano, Italy; ^5^Hospital Sant Pau, Barcelona, Spain; ^6^North Bristol NHS Trust, Bristol, Spain; ^7^Centre Hospitalier Universitaire Saint Pierre, Brussels, Belgium; ^8^Nantes Université, Nantes, France; ^9^Universitätsmedizin, Med.Klinik 2/Infektiologie, Frankfurt, Germany; ^10^Hospital General Universitario de Elche, Elche, Spain; ^11^Universitätsklinikum, Bonn, Germany; ^12^Hôpital Universitaire Pitié Salpêtrière, Paris, France; ^13^Medizinische Hochschule Hannover, Hannover, Germany; ^14^Chelsea and Westminster Hospital, London, UK; ^15^Royal Free London NHS Foundation Trust, London, UK; ^16^Guy's and St Thomas’ NHS Foundation Trust, London, UK; ^17^Infektionsmedizinisches Centrum Hamburg, Hamburg, Germany; ^18^Università degli Studi di Modena e Reggio Emilia, Modena, Italy; ^19^Universitair Ziekenhuis Antwerpen, Edegem, Belgium; ^20^Universitätsklinikum, Universität Duisburg‐Essen, Essen, Germany; ^21^Universitat de Barcelona, Barcelona, Spain; ^22^Chelsea and Westminster Hospital, London, London, UK

**Background**: Insulin resistance is common in ageing people with HIV (PWH) and contributes to diabetes and cardiovascular disease, yet it is rarely measured in antiretroviral switch trials. The triglyceride–glucose (TyG) index, calculated from fasting triglyceride and glucose values, offers a pragmatic way to detect early metabolic change using routine laboratory data. We assessed whether switching from boosted protease inhibitors (bPIs) to dolutegravir (DTG) reduced TyG‐defined insulin resistance in PWH at high cardiovascular risk.

**Methods**: NEAT‐022 was a 96‐week, open‐label, randomized trial conducted across 32 European sites in virologically suppressed adults receiving two nucleoside reverse transcriptase inhibitors plus a bPI. Participants were randomized to immediate switch to DTG (DTG‐I) or to continue bPI‐based therapy for 48 weeks before switching to DTG (DTG‐D), while maintaining the nucleoside backbone. TyG‐defined insulin resistance was defined as TyG index ≥8.7. Baseline prevalence was assessed in the modified intention‐to‐treat population (*n* = 412). Incidence was analysed among participants without baseline TyG‐defined insulin resistance or diabetes and with follow‐up TyG data (*n* = 178). Incidence rates, Kaplan–Meier estimates and multivariable Poisson models were used.

**Results**: Of 412 participants in the modified intention‐to‐treat population, 223 (54.1%) had TyG‐defined insulin resistance at baseline, highlighting a substantial metabolic burden despite virological suppression. In participants without baseline TyG‐defined insulin resistance or diabetes, incidence, time‐to‐event and adjusted analyses consistently favoured immediate switch to DTG over continued bPI therapy during the randomized 0–48‐week phase (Table 1). The full‐population trajectory followed bPI withdrawal: TyG‐defined insulin resistance decreased after immediate switch, remained high during continued bPI therapy, and fell after delayed switch to DTG (Figure 1). The pattern was mainly triglyceride‐driven, while fasting glucose remained stable. TyG correlated with HOMA‐IR, and weight changes were modest and not consistently related to TyG changes. Findings were supported by sensitivity analysis using a higher TyG threshold.

**Conclusions**: In high‐risk, virologically suppressed PWH, switching from bPIs to DTG reduced 48‐week incidence of TyG‐defined insulin resistance. TyG may provide a simple, routinely available marker of early metabolic improvement after bPI withdrawal.

**FIGURE 1:**
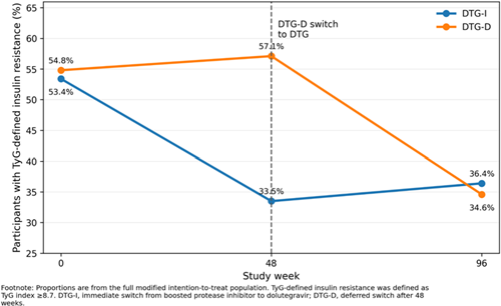
P408 | Proportion of participants with TyG‐defined insulin resistance over time.

**TABLE 1:**
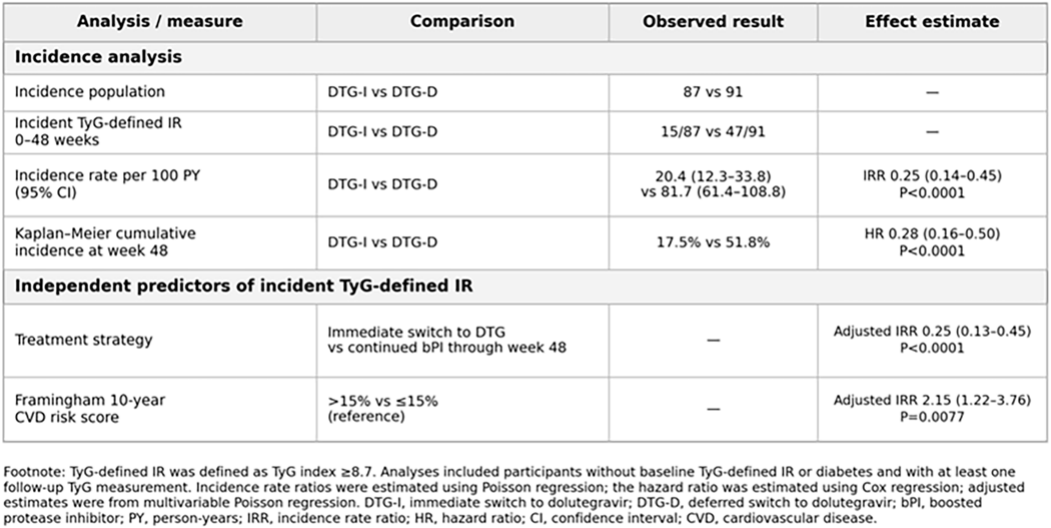
P408 | Randomized 0–48‐week analysis of TyG‐defined insulin resistance.

### P409 Statin Cascade of Care in People Living With HIV Reveals Missed Opportunities for Cardiovascular Prevention: A Retrospective Cohort Study

Christos Arkadianos, Konstantinos Protopapas, Charalampos D. Moschopoulos, Christos Filis, Kimon Koussoulakos‐Vassilounis, Elias Ernst Lykos, Anna Maria Loukaidou, Dimitra Kavatha, Antonios Papadopoulos

4th Department of Internal Medicine, National and Kapodistrian University of Athens, ATTIKON Hospital, Athens, Greece

**Background**: People living with HIV (PLWH) experience an increased burden of cardiovascular disease (CVD) driven by traditional risk factors, chronic inflammation and persistent immune activation. Following the REPRIEVE trial [1], EACS recommendations expanded indications for statin therapy for primary prevention [2]. We evaluated implementation of these recommendations using a statin cascade‐of‐care framework and identified gaps from statin eligibility to treatment initiation, persistence and LDL‐cholesterol target attainment.

**Methods**: A retrospective cohort study was conducted among adult PLWH > 40 years old, receiving care at a tertiary HIV clinic between 2023 and 2025. Cardiovascular risk was estimated using SCORE2, and statin eligibility was determined according to EACS recommendations. Individuals receiving statins before 2023 were excluded. The statin cascade of care comprised five sequential stages: (i) identification of statin eligibility, (ii) documented physician recommendation, (iii) treatment initiation, (iv) treatment continuation and (v) achievement of LDL‐cholesterol targets (<70 mg/dL). Multivariable logistic regression identified factors associated with receiving a physician recommendation for statin initiation.

**Results**: Among 885 PLWH over 40 years receiving care during the study period, 642 were not receiving statin therapy before 2023 and were included in the primary prevention analysis. Retrospective application of the current EACS recommendations identified 453/642 (70.6%) individuals eligible for statin therapy. However, only 162/453 (35.8%) had a documented recommendation for statin initiation, representing the greatest loss within the cascade. Among those recommended treatment, 152 (93.8%) initiated statin therapy and 102 (67.1%) remained on treatment during follow‐up. Only 8 patients (7.8% of those continuing treatment) achieved LDL‐cholesterol levels < 70 mg/dL (Figure 1). Higher baseline LDL‐cholesterol concentrations (OR 3.17, 95% CI: 2.20–4.57; *p* < 0.001) and established cardiovascular disease (OR 28.21, 95% CI: 2.60–305.84; *p* = 0.006) were independently associated with receiving a recommendation for statin initiation. The final model demonstrated good discrimination (AUC 0.807).

**Conclusions**: Application of a statin cascade‐of‐care framework revealed substantial missed opportunities for cardiovascular prevention among PLWH. The principal implementation gap occurred at recognizing and documenting statin eligibility, whereas treatment uptake following physician recommendation was high. Although eligibility was assessed retrospectively, these findings highlight the need for systematic cardiovascular risk assessment, improved implementation of contemporary recommendations, optimization of lipid‐lowering strategies and enhanced long‐term follow‐up to maximize the benefits of statin‐based primary prevention in the post‐REPRIEVE era.

**References**

1. Grinspoon SK, Fitch KV, Overton ET, Fichtenbaum CJ, Zanni MV, Aberg JA, et al. Pitavastatin to prevent cardiovascular disease in HIV infection. N Engl J Med. 2023;389(10):687–699.

2. Ambrosioni J, Levi LI, Alagaratnam J, Sempere A, Mastrangelo A, Paioni P, et al. Major revision version 13.0 of the European AIDS Clinical Society guidelines 2025. HIV Med. 2026;27(1):18–32.

**FIGURE 1:**
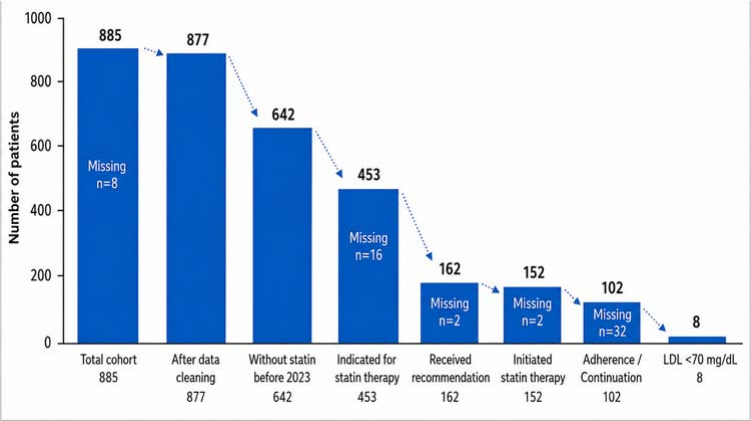
P409 | Cascade of care for statin therapy according to the EACS HIV 2025 guidelines (2023–2025).

### P410 Improved Statin Prescribing in People Living With HIV But Persistent Gaps in Highest‐Risk Patients: A Multi‐Site Service Evaluation

Bethany Harrison, Courtney Murray, Paul Hine

University Hospitals Liverpool Group, Liverpool, UK

**Background**: People living with HIV have increased cardiovascular disease (CVD) risk. BHIVA recommends statin therapy for all aged >40 years, prioritizing those at highest risk. Following a 2022 audit, we introduced a multi‐component service improvement programme including clinician education, a patient information leaflet and pharmacy‐supported pre‐clinic review. We assessed whether these interventions improved CVD prevention activity.

**Methods**: We reviewed electronic patient records for people living with HIV aged >40 years receiving care across Cheshire and Merseyside. We collected demographic, clinical and CVD risk data and analysed them using Excel. We manually reviewed a random subset of 30 records to assess free‐text documentation of statin discussion and lifestyle counselling (including smoking cessation). We compared findings with BHIVA auditable standards and results from the 2022 audit.

**Results**: We analysed 2106 patient records. Overweight or obesity was recorded in 69% (*n* = 1467), and 19% were current smokers; smoking status was documented in 97% (*n* = 2034). Statin prescribing increased from 27% (*n* = 364) in 2022 to 36% (*n* = 755). QRISK calculation improved from 76% to 81%. Statin use increased with age (17% in 40–49 vs. 67% in 70–79). When stratified by risk in line with BHIVA guidance, statin prescribing increased across groups: 22% (232/1063) in those with QRISK < 5% (discussion advised), 40% (188/466) in those with QRISK 5–10% (statin recommended) and 58% (335/577) in those with QRISK > 10% (statin strongly recommended). In a subset of 30 records, 77% had documented statin discussion in the previous 3 years and 50% had lifestyle counselling documented in the previous 2 years.

**Conclusions**: Our service improvement programme increased overall statin prescribing and improved CVD risk assessment. However, a substantial proportion of those at highest risk remained untreated, with 42% of people with QRISK > 10% not receiving statins. These findings emphasize the need to maintain a risk‐stratified approach alongside “statins for all” strategies, ensuring those at highest risk are prioritized. Continued focus on targeted prescribing and lifestyle interventions, alongside evaluation of patient‐level and prescribing barriers, may maximize CVD prevention in people living with HIV.

### P411 Abstract Withdrawn

### P412 Atherogenic Index of Plasma (AIP) as a Stable Pre‐Event Marker of Major Adverse Cardiovascular Events in People With HIV: A Time‐Anchored Matched Case−Control Study From the ICONA Cohort

Nicola Squillace ^1^, Simone Lanini^2^, Alessandro Tavelli^3^, Marco Cotrufo^2^, Roberta Gagliardini^4^, Matteo Augello^5^, Carlo Torti^6^, Jacopo Vecchiet^7^, Anna Beltrami^8^, Antonella Castagna^9^, Antonio Di Biagio^10^, Giordano Madeddu^11^, Alessandra Bandera^12^, Giovanni Guaraldi^13^, Sergio Lo Caputo^14^, Andrea Antinori^15^, on behalf of ICONA Study Group

^1^Infectious Diseases Clinic, Fondazione IRCCS, San Gerardo dei Tintori, Monza, Italy; ^2^Infectious Diseases Clinic, Department of Medicine (DAME), University of Udine, Udine, Italy; ^3^ICONA Foundation, Milano, Italy; ^4^Clinical Infectious Diseases Department, National Institute for Infectious Diseases Lazzaro Spallanzani IRCCS, Roma, Italy; ^5^Clinic of Infectious Diseases and Tropical Medicine, San Paolo Hospital, ASST Santi Paolo e Carlo, Department of Health Sciences, University of Milan, Milano, Italy; ^6^UOC Malattie Infettive, Fondazione Policlinico Universitario Agostino Gemelli IRCCS, Dipartimento di Sicurezza e Bioetica, Università Cattolica del Sacro Cuore, Roma, Italy; ^7^Clinic of Infectious Diseases, Department of Medicine and Science of Aging, University “G. d'Annunzio”, Chieti, Italy; ^8^Unit of Infectious Diseases, Santa Maria Annunziata Hospital, Firenze, Italy; ^9^Department of Infectious Diseases, IRCCS San Raffaele Scientific Institute, Vita‐Salute San Raffaele University Department of Infectious Diseases, IRCCS San Raffaele Scientific Institute, Vita‐Salute San Raffaele University, Milano, Italy; ^10^.Infectious Diseases Clinic, IRCCS Azienda Ospedaliera Metropolitana, Department of Health Science (DISSAL), University of Genova, Genova, Italy; ^11^Unit of Infectious Disease, Department of Medicine, Surgery and Pharmacy, University of Sassari, Sassari, Italy; ^12^Clinic of Infectious Diseases, Fondazione IRCCS Ca' Granda Ospedale Maggiore Policlinico, Department of Pathophysiology and Transplantation, University of Milan, Milano, Italy; ^13^Department of Infectious Diseases, Azienda Ospedaliero‐Universitaria di Modena, Policlinico di Modena, University of Modena and Reggio Emilia, Modena, Italy; ^14^Clinic of Infectious Diseases, Department of Clinical and Surgical Sciences, University of Foggia, Foggia, Italy; ^15^HIV/AIDS Department, National Institute of Infectious Diseases, L. Spallanzani, IRCCS, Roma, Italy

**Background**: People with HIV (PWH) remain at increased cardiovascular risk, including major adverse cardiovascular events (MACE). AIP, a triglyceride/HDL‐C composite, is poorly studied in PWH. We assessed whether AIP was associated with MACE and more informative than HDL‐C or triglycerides alone.

**Methods**: We conducted a time‐anchored nested matched case−control study within ICONA, including virologically suppressed PWH. For each MACE case (myocardial infarction, stroke or coronary revascularization), five controls were selected from the cohort, matched by sex and age ±5 years, and in follow‐up at the same index date. Lipid markers were measured yearly over the 5 pre‐event years. We used two samples: an extended sample with all available observations, and a restricted sample with complete covariate data (no missing data). For study flow chart, see Figure 1A. Conditional logistic regression models were used to investigate the association between MACE and AIP, HDL‐C and TG in each year pre‐index. Unadjusted models fitted in both samples, adjusted analyses only on the restricted/common dataset. Marker trajectories were examined using linear mixed models.

**Results**: Among 22,202 PWH, 168 MACE cases were identified. The extended dataset included 4146 observations (3455 control‐ and 691 case‐observations); 3817 were retained in the restricted/common sample (Figure 1A). Mean age was 53.9 years and 88.6% were male. Case‐observations more often involved current smoking (61.3% vs. 44.8%, *p* < 0.001), lower nadir CD4 (208 vs. 229 cells/µL, *p* = 0.004), longer HIV duration, longer PI exposure, shorter NNRTI exposure, and with lower HDL‐C, higher triglycerides and higher AIP (all *p* < 0.001). In adjusted models, AIP was associated with MACE in each pre‐event year, with ORs per 0.1‐unit increase from 1.08 (−5 years) to 1.15 (−1 year), all significant, without marker‐by‐year heterogeneity (*p* = 0.424). HDL‐C was significant only at years −4 and −5, while triglycerides showed a weaker time‐dependent pattern (Table 1). Mixed‐model trends confirmed persistently higher AIP and lower HDL‐C in case‐observations (Figure 1B); triglyceride differences were less stable. AIP showed the highest, although modest, adjusted discrimination (AUC 0.652 vs. 0.640 for HDL‐C and 0.639 for triglycerides).

**Conclusions**: In virologically suppressed PWH, AIP was consistently associated with MACE throughout the 5‐year pre‐event window and appeared more stable than HDL‐C or triglycerides alone, supporting its use for risk profiling.

**FIGURE 1:**
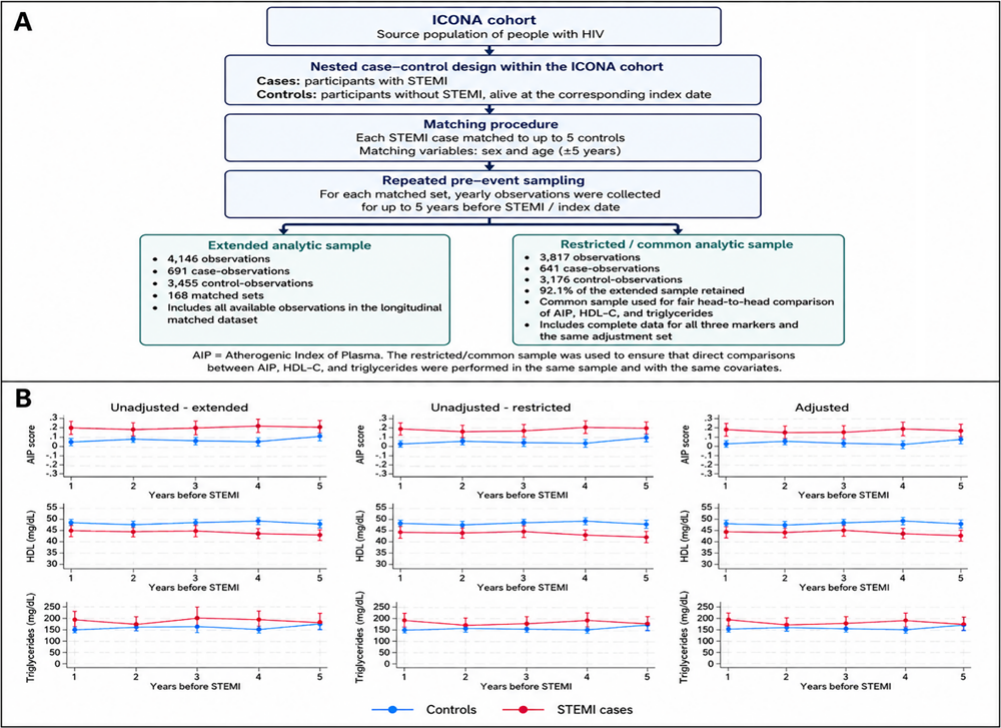
P412 | (A) Study flow chart; (B) markers variation across time and adjustments.

**TABLE 1:**
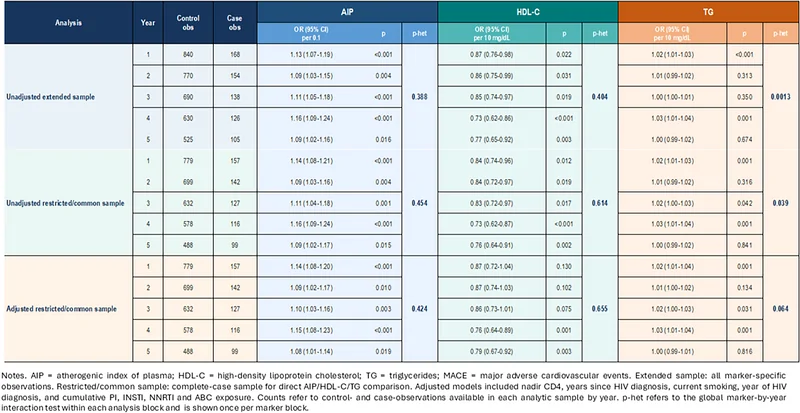
P412 | AIP, HDL‐C and triglycerides as markers of MACE risk: risk analysis by pre‐event year by conditional logistic models. Odds ratios are reported by year before MACE; marker columns are shown horizontally. Counts are observation counts for each analytic sample.

### P413 Switching From TAF‐Based Regimens to Long‐Acting Therapy Versus FTC/TAF/BIC: Differential Effects on Weight and Lipid Profile in People With HIV

Nicola Squillace ^1^, Elena Delfina Ricci^2^, Lucia Taramasso^3^, Teresa Bini^4^, Barbara Menzaghi^5^, Giancarlo Orofino^6^, Giordano Madeddu^7^, Sergio Ferrara^8^, Giuseppe Vittorio De Socio^9^, Giovanni Cenderello^10^, Emanuele Pontali^11^, Stefania Piconi^12^, Giovanni Francesco Pellicano^13^, Antonio Di Biagio^3,14^, Paolo Bonfanti^1,15^, on behalf of CISAI/SCOLTA Study Group

^1^Infectious Diseases Clinic Fondazione IRCCS, San Gerardo dei Tintori, Monza, Italy; ^2^Fondazione A.S.I.A. Onlus, Buccinasco (MI), Italy; ^3^Infectious Diseases Clinic, IRCCS Azienda Ospedaliera Metropolitana, Genova, Italy; ^4^ASST Santi Paolo e Carlo, Unit of Infectious Diseases, Milano, Italy; ^5^Unit of Infectious Diseases, ASST della Valle Olona, Busto Arsizio (VA), Italy; ^6^Division I of Infectious and Tropical Diseases, ASL Città di Torino, Torino, Italy; ^7^Unit of Infectious Diseases, Department of Medicine, Surgery and Pharmacy, University of Sassari, Sassari, Italy; ^8^Unit of Infectious Diseases, Department of Clinical and Experimental Medicine, University of Foggia, Foggia, Italy; ^9^Infectious Diseases Clinic, Department of Medicine and Surgery, University of Perugia, Santa Maria Hospital, Perugia, Italy; ^10^Infectious Diseases Department, Sanremo Hospital, Sanremo, Italy; ^11^Department of Infectious Diseases, Galliera Hospital, Genova, Italy; ^12^Unit of Infectious Diseases, A. Manzoni Hospital, Lecco, Italy; ^13^Infectious Diseases, G. Martino Hospital ‐University of Messina, Messina, Italy; ^14^Department of Health's Sciences (DISSAL), University of Genoa, Genova, Italy; ^15^University of Milano‐Bicocca, Monza, Italy

**Background**: We aimed to compare the effect of switching to long‐acting (LA) therapy versus emtricitabine/tenofovir alafenamide/bictegravir (BIC) in people with HIV (PWH) already receiving TAF, focusing on changes in weight and lipid profile.

**Methods**: Consecutive PWH enrolled in the multicentre observational SCOLTA cohort, with HIV‐RNA <50 copies/mL, who switched from a stable FTC/TAF‐containing regimen to either BIC or LA therapy were included. Changes from baseline (T0) to week 24 (T1) and week 48 (T2) were described as means and standard errors and analysed using analysis of variance and general linear models.

**Results**: Overall, 690 PWH were enrolled: 299 switched to LA therapy and 391 to BIC (see Table 1 for description of the sample). At T1, the following variables differed significantly between the LA and BIC groups: weight (mean difference −0.3 ± 0.2 vs. 0.3 ± 0.2 kg; *p* = 0.04), alanine aminotransferase (ALT) (−1.0 ± 0.6 vs. 2.2 ± 0.8 IU/L; *p* = 0.002), total cholesterol (TC) (4.6 ± 1.6 vs. −4.0 ± 1.7 mg/dL; *p* = 0.0003) and LDL cholesterol (LDL) (3.6 ± 1.5 vs. −2.1 ± 1.6 mg/dL; *p* = 0.007). At T2 univariate analyses confirmed the differences for ALT, TC and LDL. In the multivariate model, we included the variables significantly different at baseline between groups. Mean weight (LA 0.1 ± 1.1 kg, FTC/TAF/BIC 0.8 ± 1.0 kg, *p* = 0.049) and mean TC (LA −5.2 ± 8.3 mg/dL, BIC −11.3 ± 8.0 mg/dL, *p* = 0.042) at T1, and HDL‐cholesterol (HDL) (LA 0.7 ± 4.9 mg/dL, BIC −3.4 ± 4.8 mg/dL, *p* = 0.006) at T2 were confirmed different between LA and BIC. Among 252 PWH switching only from an INSTI‐containing regimen, mean weight (LA −1.1 ± 0.9 kg, BIC 0.0 ± 0.9 kg, *p* = 0.024) and ALT (LA −0.7 ± 3.6 IU/L, BIC 3.9 ± 3.4 IU/L, *p* = 0.005) at T1 and TC (LA 11.1 ± 10.3 mg/dL, BIC −3.3 ± 10.1 mg/dL, *p* = 0.001) at T2 showed significant differences.

**Conclusions**: In PWH already receiving TAF, switching to LA therapy was associated with significantly less weight gain at 24 weeks and a smaller decrease in HDL at 48 weeks compared with switching to BIC.

**TABLE 1:**
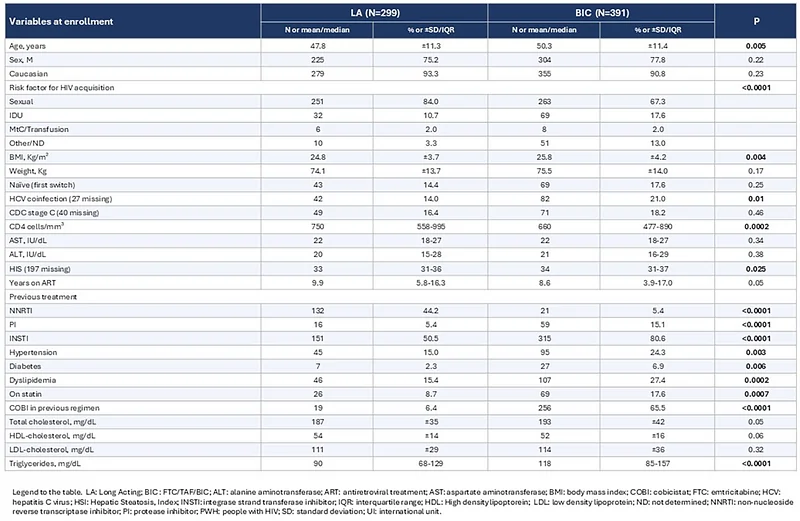
P413 | Baseline characteristics.

### P414 Sex Disparities in Cardiovascular Risk Factor Burden and Preventive Care Among People With HIV Across Three Antiretroviral Therapy Eras

Jovana Milić ^1,2^, Laura E. Biondi^3^, Michela Belli^1,2^, Elisabetta Delmonte^1,2^, Irene Avanzini^1,2^, Cristina Mussini^1,2^, Giovanni Guaraldi^1,2^

^1^Department of Surgical, Medical, Dental and Morphological Sciences, University of Modena and Reggio Emilia, Italy, Modena, Italy; ^2^Modena HIV Metabolic Clinic, University of Modena and Reggio Emilia, Modena, Italy, Modena, Italy; ^3^School of Medicine, University of Modena and Reggio Emilia, Modena, Italy

**Background**: Although contemporary HIV care increasingly emphasizes cardiovascular (CV) prevention, little is known about temporal sex differences in CV risk burden and implementation of preventive measures. We evaluated sex‐specific trends in CV risk factors and CV preventive care across three study periods.

**Methods**: We conducted a longitudinal observational study of people with HIV (PWH) attending the Modena HIV Metabolic Clinic, Italy, between January 2005 and May 2026. Participants with at least one clinic visit were included and categorized into three predefined periods: 2005–2015, 2016–2020 and 2021–2026. CV risk factors included smoking, abacavir use, protease inhibitor (PI) use, hypertension, diabetes, obesity and physical inactivity. CV preventive measures included statin use, smoking cessation, discontinuation of abacavir and PI, antihypertensive therapy, physical activity, and LDL‐cholesterol target achievement. Sex‐specific differences were across study periods. Multivariable Cox proportional hazards models evaluated the association between sex and adoption of preventive measures.

**Results**: A total of 5474 PWH were included, of whom 1785 (32.6%) were women. Table 1 summarizes sex‐specific CV risk factors and preventive measures across study periods. Women were less likely to receive statins despite comparable or higher rates of LDL‐C target achievement. The proportion of participants with ≥3 CV risk factors declined from 35.0% to 21.0% in women and from 41.9% to 27.3% in men (*p* < 0.001) (Figure 1A). The proportion of participants receiving ≥3 preventive measures increased from 23.4% to 63.0% in women and from 31.7% to 70.4% in men (*p* < 0.001) (Figure 1B). Among participants at high CV risk, physical activity was less frequent in women than men (39.7% vs. 50.0%, *p* < 0.001). Female sex remained independently associated with a lower likelihood of receiving CV preventive measures after adjustment for age and SCORE2 (HR 0.62, 95% CI: 0.43–0.89; *p* = 0.009) and after adjustment for HIV duration, nadir CD4 count, and HIV RNA suppression (HR 0.60, 95% CI: 0.41–0.90; *p* = 0.012).

**Conclusions**: Although CV risk factor burden declined and uptake of preventive interventions improved across successive ART eras, significant sex disparities persisted. Women were consistently less likely than men to receive CV preventive measures, including among those at highest CV risk. Targeted strategies are needed to ensure equitable implementation of cardiovascular prevention in women with HIV.

**FIGURE 1:**
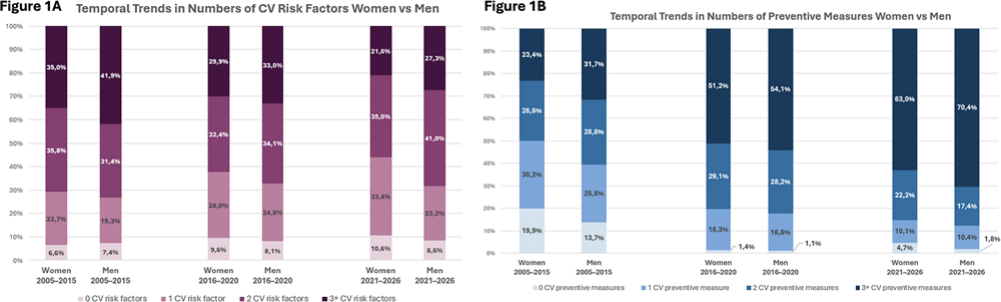
P414 | Prevalence of CV risk factors and CV preventive measures according to sex across three time periods.

**TABLE 1:**
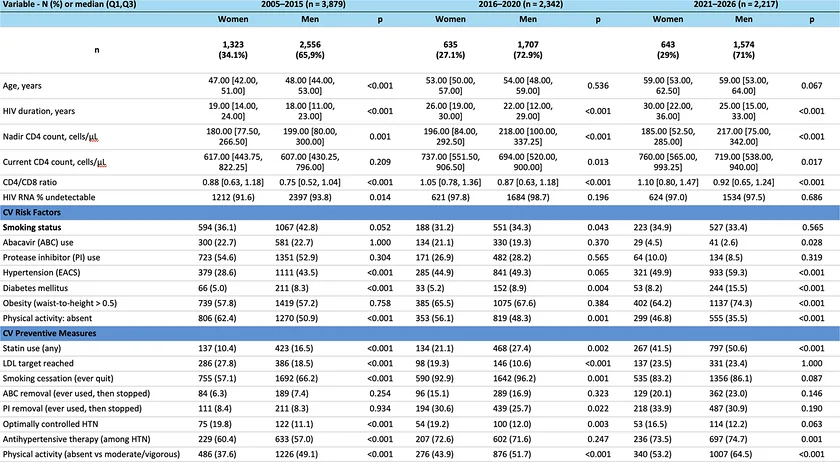
P414 | Sex differences in CV risk and prevention across time periods. Last available observation per patient within each period, stratified by sex.

### P415 Applying the 2025 AHA/ACC Definition Triples the Incidence of Hypertension in People With HIV Compared With EACS

Jovana Milić ^1,2^, Alessandra Carobbio^3^, Anna Katrine Haslund Roed^4^, Eugenio Carriero^1^, Lorenzo Salmoni^5^, Filippo Buffagni^1,2^, Cristina Mussini^1,2^, Susanne Dam Nielsen^4^, Giovanni Guaraldi^1,2^, Esteban Martínez^6^

^1^Department of Surgical, Medical, Dental and Morphological Sciences, University of Modena and Reggio Emilia, Italy, Modena, Italy; ^2^Modena HIV Metabolic Clinic, Modena, Italy; ^3^Department of Medical and Surgical Sciences for Children and Adults, Modena, Italy; ^4^Viro‐Immunology Research Unit, Department of Infectious Diseases, Copenhagen University Hospital, Rigshospitalet, Copenhagen, Denmark; ^5^Department of Mental and Physical Health and Preventive Medicine, University of Campania “Luigi Vanvitelli”, Naples, Italy; ^6^HIV Unit, Hospital Clínic de Barcelona‐IDIBAPS, Barcelona, Italy

**Background**: Hypertension is one of the most common non‐communicable diseases among people with HIV (PWH) and an important contributor to cardiovascular morbidity. The objective was to estimate and compare the incidence of elevated blood pressure (EBP) and hypertension among PWH using two definitions: (i) the European AIDS Clinical Society (EACS) guideline definition (v13.0, October 2025) and (ii) the 2025 American Heart Association/American College of Cardiology (AHA/ACC) guidelines definition, and to identify determinants of incident hypertension according to each definition.1, 1

**Methods**: This was a longitudinal observational study (2005–2026) including PWH attending Modena HIV Metabolic Clinic, Italy including all PWH without antihypertensive treatment or hypertension or EBP at baseline. Incident EBP and hypertension were classified by EACS (hypertension ≥140/90; EBP 130–139/85–89 mmHg) and 2025 AHA/ACC (≥130/80; 120–129/< 80 mmHg). Incidence rates per 100 person‐years and cumulative incidence were estimated. Determinants of incident hypertension were identified by backward stepwise Cox regression, including cardiometabolic variables, statin use and exposure to integrase inhibitors (INSTI) and tenofovir alafenamide (TAF).

**Results**: We analysed 1638 PWH, 1029 men (62.8%). At baseline, median was age 42 years (IQR 38–47), HIV duration 13 years (7–19), baseline CD4 563 cells/µL, HIV RNA undetectable in 81.7%, 11.3% were receiving INSTI, 5.6% TAF and 3.8% statins. INSTI, TAF and polypharmacy were more frequent among those without incident hypertension according to both definitions. Incident hypertension was 6.91 per 100 person‐years using the EACS definition and 19.66 per 100 person‐years using the 2025 AHA/ACC definition, corresponding to a 2.8‐fold higher incidence rate. EBP rates were similar across definitions. In final Cox models, male sex, older age, waist circumference, HOMA‐IR and total cholesterol were associated with incident hypertension under both definitions. INSTI, TAF and statin use were not retained in either model.

**Conclusions**: Applying the 2025 AHA/ACC definition identified a nearly threefold higher incidence of hypertension in PWH compared with EACS. This reclassification may be clinically relevant, as it identifies a larger group of PWH who may benefit from closer cardiovascular risk assessment, lifestyle intervention and BP follow‐up. Whether lower BP thresholds should be adopted in HIV care requires studies linking these definitions with cardiovascular outcomes.

**FIGURE 1:**
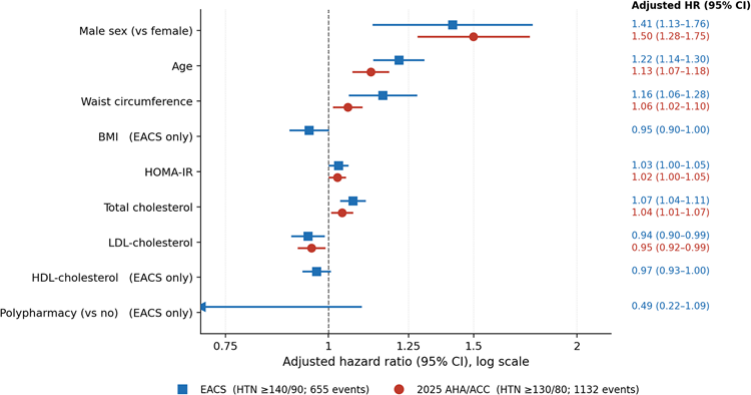
P415 | Independent determinants of incident hypertension in people with HIV final backward‐stepwise Cox models (retention *p* < 0.10). ← indicates the hazard ratio and/or 95% CI: extend beyond the axis (polypharmacy HR 0.49, 95% CI: 0.22–1.09). Hazard ratios per increment: age, 5 years; waist circumference, 5 cm; total and LDL cholesterol, 10 mg/dL; HDL cholesterol, 5 mg/dL; BMI and HOMA‐IR, 1 unit. INSTI and TAF exposure and statin use were entered as candidate covariates but were not associated with incident hypertension (not retained).

**TABLE 1:**
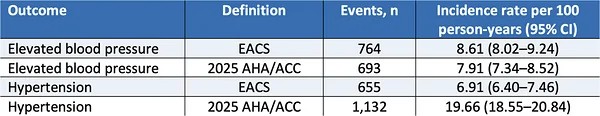
P415 | Incidence of elevated blood pressure and hypertension in 1638 people with HIV, according to the EACS and 2025 AHA/ACC definitions. EACS, European AIDS Clinical Society; AHA/ACC, American Heart Association/American College of Cardiology; CI, confidence interval. Elevated blood pressure: EACS 130–139/85–89 mmHg, AHA/ACC 120–129/< 80 mmHg. Hypertension: EACS ≥140/90 mmHg, AHA/ACC ≥130/80 mmHg.

### P416 Metabolic Impact of Switching to Doravirine‐Based Regimens in Treatment‐Experienced People Living With HIV: 48‐Week Results From the DOLART Study in Argentina

Diego Cecchini ^1^, Edgardo Bottaro^1^, Gastón Copertari^1^, Martín Brizuela^1^, Carla Serrano^2^, María Magdalena Puchulu^2^, Juan Manuel Nuñez^3^, Juan Gonzalo Tomás^3^, Macarena Roel^1^, Isabel Cassetti^1^

^1^HELIOS SALUD SA., Buenos Aires, Argentina; ^2^Centro de Enfermedades Infecciosas y Parasitarias (CEIP), Tucumán, Argentina; ^3^Clínica Mayo, Unidad Médica Cruz Blanca (UMCB), Tucumán, Argentina

**Background**: Doravirine‑based antiretroviral therapy (ART) has shown favourable lipid effects in clinical trials and European real‑world cohorts, but metabolic data from Latin America are lacking. The DOLART study provides the first Latin American real‑world evaluation of metabolic changes after switching to doravirine‑based ART in treatment‑experienced people living with HIV (PLWH).

**Methods**: DOLART is a multicentre, retrospective cohort in Argentina including adults with HIV who initiated doravirine‑based ART in routine care. We evaluated treatment‑experienced PLWH with available baseline and on‑treatment metabolic data. Fasting glucose, total cholesterol, HDL‑cholesterol, LDL‑cholesterol, triglycerides and estimated glomerular filtration rate (eGFR) were assessed at baseline, week 24 and week 48.

**Results**: Of 567 treatment‐experienced PLWH, 71% switched from EFV‑based regimens and 29% from non‑EFV regimens (nevirapine, ritonavir‐booster darunavir and second generation INSTIs). Overall, there were significant reductions in total cholesterol, LDL‑cholesterol and triglycerides between baseline and both week 24 and week 48, while fasting glucose remained stable and eGFR showed a modest decline with small absolute changes (Table 1). HDL‑cholesterol also decreased significantly over time. Metabolic and renal trajectories over 48 weeks were similar in participants switching from EFV‑based and non‑EFV‑based regimens.

**Conclusions**: In this first Latin American real‑world cohort of treatment‑experienced PLWH, switching to doravirine‑based regimens was associated with sustained improvements in total cholesterol, LDL‑cholesterol and triglycerides over 48 weeks, along with modest reductions in HDL‑cholesterol, and stable fasting glucose. Observed declines in eGFR were statistically significant but not clinically meaningful. These findings support doravirine‑based ART as a metabolically favourable switch option in routine clinical practice in Argentina.

**TABLE 1:**
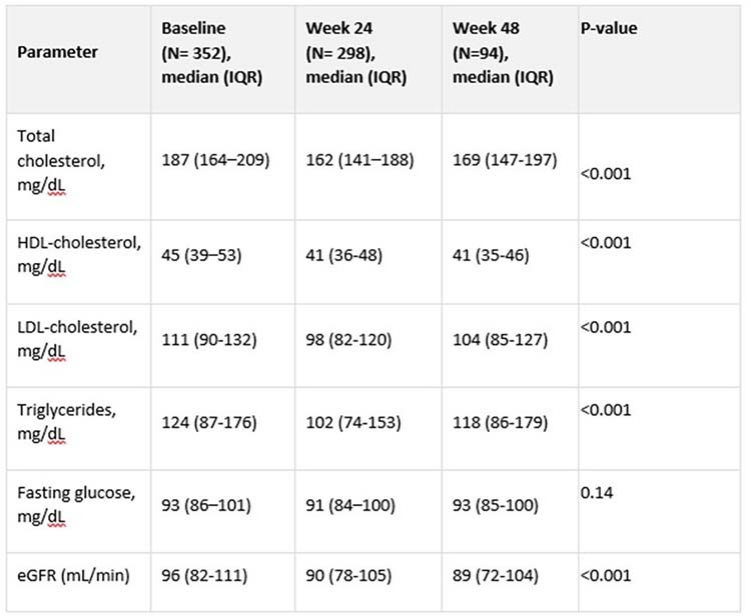
P416 | Total cholesterol, HDL‐cholesterol, LDL‐cholesterol, triglycerides, fasting glucose and eGFR at baseline, week 24 and week 48 in treatment‐experienced participants who switched to doravirine‐based regimens in the DOLART study.

### P417 Incretin‐Based Therapies in People With HIV: Weight, Glycaemic and Virological Outcomes in a Real‐World Cohort

Ling‐Ya Chen ^1^, Yi‐Ting Chen^1^, Hsin‐Yun Sun^2,3^, Hsi‐Yen Chang^1^, Yu‐Zhen Luo^1^, Pei‐Ying Wu^1^, Chien‐Ching Hung^4,5^

^1^Center for Infection Control, National Taiwan University Hospital, Taipei, Taiwan; ^2^Department of Internal Medicine, National Taiwan University Hospital, Taipei, Taiwan; ^3^National Taiwan University College of Medicine, Taipei, Taiwan; ^4^Department of Internal Medicine, National Taiwan University Hospital Yunlin Branch, Yunlin, Taiwan; ^5^Department of Tropical Medicine and Parasitology, National Taiwan University College of Medicine, Taipei, Taiwan

**Background**: Incretin‐based therapies, including glucagon‐like peptide‐1 receptor agonists (GLP‐1 RAs) and dual glucose‐dependent insulinotropic polypeptide (GIP)/GLP‐1 receptor agonists (dual GIP/GLP‐1 RAs), are increasingly used for weight management and glycaemic control. Because these agents may affect concomitant oral drug absorption, real‐world data on metabolic outcomes, HIV viral suppression and immunological follow‐up during use among people with HIV are needed.

**Methods**: At a Northern Taiwan tertiary HIV clinic, we conducted a single‐centre retrospective cohort of people with HIV receiving incretin‐based therapy from May 2021 to January 2026, with outcomes ascertained through May 2026. Available data were summarized at baseline and weeks 12, 24, 48, 72 and 96; later windows were exploratory. Outcomes included body weight, glycated haemoglobin (HbA1c), HIV RNA, CD4 measures, discontinuation and antiretroviral therapy (ART) continuity.

**Results**: Seventy‐eight people with HIV were included: 52 received tirzepatide, 19 semaglutide and 7 other incretin‐based agents (Table 1). Median body weight percentage changes were −6.11% (IQR −9.75, −3.85) at week 12 (*n* = 60) and −5.47% (IQR −10.37, −2.37) at week 24 (*n* = 39). Overall HbA1c changes were −0.30 percentage points (IQR −0.40, −0.20) at week 12 (*n* = 41), −0.30 (IQR −0.85, 0.00) at week 24 (*n* = 26) and −0.50 (IQR −0.95, 0.00) at week 48 (*n* = 14) (Figure 1). Viral suppression < 50 copies/mL was observed in 94.9% (74/78) at baseline, 95.8% (23/24) at week 24, 94.7% (18/19) at week 48 and 77.8% (7/9) at week 96. The two week 96 cases with HIV RNA > 200 copies/mL occurred after ART discontinuation and subsequently re‐suppressed without detected resistance. Baseline median CD4 cell count was 692.50 cells/µL (IQR 571.75–901.00) and CD4/CD8 ratio was 0.80 (IQR 0.63–1.17). Discontinuation was recorded in 21 participants, most commonly after achieving the desired weight‐loss effect (13/21); median time to discontinuation was 22.3 weeks.

**Conclusions**: In this real‐world HIV clinic cohort, incretin‐based therapy was associated with early body weight reduction and modest HbA1c reductions. HIV viral suppression was generally maintained among participants with available follow‐up. Short‐term discontinuation and non‐linear medication‐use patterns were common, supporting integrated metabolic monitoring and ART continuity assessment.

**FIGURE 1:**
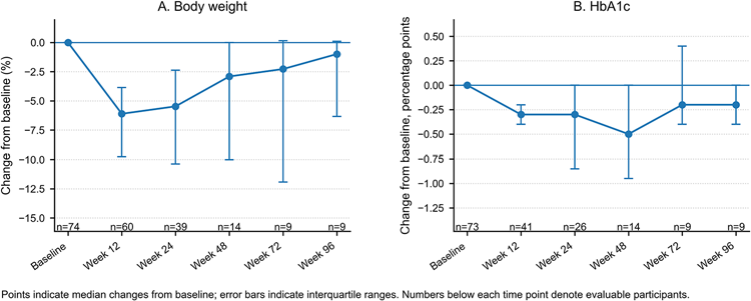
P417 | Changes in body weight and HbA1c during incretin‐based therapy among people with HIV.

**TABLE 1:**
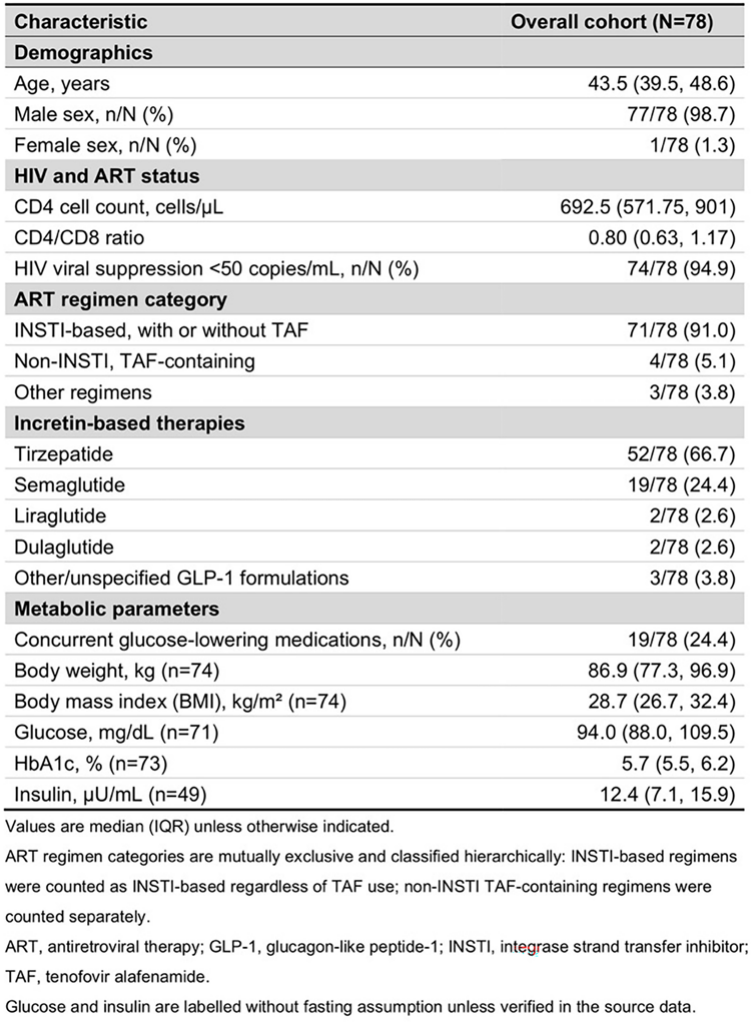
P417 | Baseline characteristics of the overall cohort.

### P418 *PNPLA3* rs738409 Polymorphism Is Associated With Non‐Alcoholic Fatty Liver Disease in Treatment‐Naïve HIV‐Infected Individuals, Predominantly in Lean Patients

Yanyan Li, Hongxia Wei, Qin Wang, Chen Chen, Rentian Cai, Lianfeng Lu, Shuo Yang, Jiale Chi, Chunmiao Zou, Ping Luo

The Second Hospital of Nanjing, Nanjing, China

**Background**: Non‐alcoholic fatty liver disease (NAFLD) is a common comorbidity among people living with HIV (PLWH) with adverse hepatic outcomes. While metabolic factors are established contributors, the roles of host genetic susceptibility, particularly the *PNPLA3* rs738409 polymorphism, and immune status remain unclear in treatment‐naïve individuals. This study evaluated the association of *PNPLA3* rs738409 with NAFLD across body mass index (BMI) and CD4+ T‐cell strata.

**Methods**: This cross‐sectional study included 708 treatment‐naïve PLWH. NAFLD was diagnosed via abdominal ultrasonography. Participants were stratified by Chinese BMI criteria (<24 and ≥24 kg/m^2^) and CD4+ T‐cell counts (<200 and ≥200 cells/mm^3^). Multivariable logistic regression and chi‐square tests identified factors associated with NAFLD and compared prevalence across strata.

**Results**: The overall NAFLD prevalence was 36.2% (256/708). In multivariable analysis, the *PNPLA3* rs738409 CG/GG genotype was independently associated with NAFLD (OR 1.561, 95% CI: 1.053–2.314), along with BMI, alanine aminotransferase, triglyceride‐glucose index and albumin. In BMI‐stratified analysis, NAFLD prevalence was significantly higher in CG/GG versus CC carriers among lean individuals (26.7% vs. 16.1%, *p* = 0.006), where the CG/GG genotype remained a significant risk factor (OR 2.016, 95% CI: 1.209–3.361). Conversely, no significant difference in prevalence (66.1% vs. 68.7%, *p* = 0.686) or genetic association (OR 0.967, *p* = 0.923) was observed in overweight/obese individuals. Exploratory analysis showed higher NAFLD prevalence in participants with CD4+ ≥200 versus < 200 cells/mm^3^ (38.7% vs. 30.1%, *p* = 0.031), though CD4+ count was not an independent predictor in the multivariable model (Figure 1).

**Conclusions**: The *PNPLA3* rs738409 polymorphism is independently associated with NAFLD in treatment‐naïve PLWH, with a stronger genetic effect observed in lean participants. NAFLD risk profiles differ across BMI strata, suggesting distinct pathogenic mechanisms. Although CD4+ count correlates with NAFLD prevalence, its independent role requires further investigation. These findings highlight the importance of considering both genetic and metabolic factors for NAFLD risk stratification in PLWH.

**FIGURE 1:**
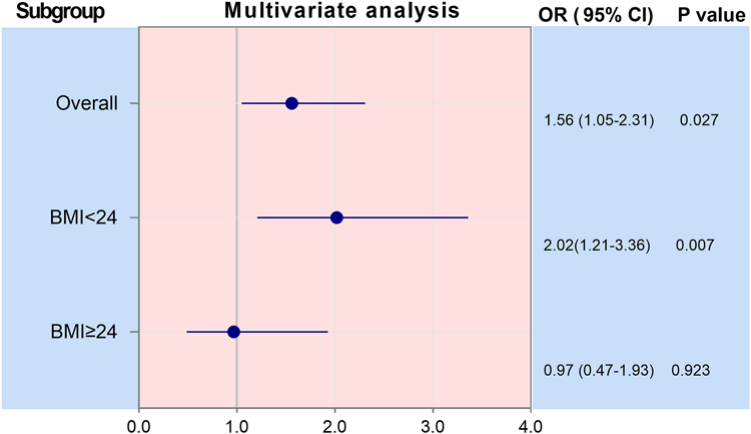
P418 | Association between PNPLA3 rs738409 polymorphism and NAFLD according to BMI strata.

### P419 Moderate/Severe Steatotic Liver Disease (SLD) and At‐Risk MASH in People With and Without HIV

Alejandro Arenas‐Pinto ^1,2^, Maeve Barlow^1,2^, Andrew Copas^3^, Sarah Pett^1,2^, Agnieszka Kemper^2^, Florence Bascombe^1,2^, Kat Johnson^1,2^, Sadiyah Mukhtar^1,2^, Indrajit Ghosh^2^, Stuart Flanagan^2^, Richard Gilson^1,2^

^1^UCL Centre for Clinical Research in Infection and Sexual Health, London, UK; ^2^The Mortimer Market Centre, London, UK; ^3^UCL Institute for Global Health, London, UK

**Background**: Steatotic liver disease (SLD) is a leading cause of liver morbidity in the general population and among people with HIV. Risk factors for SLD appear more common in people living with HIV (PLWH); however, comparative studies assessing the prevalence and determinants of SLD and at‑risk metabolic‐associated steatohepatitis (MASH) in individuals with and without HIV remain limited.

**Methods**: We conducted a cross‐sectional comparison of the prevalence and factors associated with moderate/severe SLD and at‐risk MASH in adults referred for investigation using transient elastography (TE) with controlled attenuation parameter (CAP). Referral criteria included chronic viral hepatitis, other suspected chronic liver disease, abnormal LFT, BMI > 28, SLD was defined as CAP > 268 dB/m [1] and at‐risk MASH as FibroScan‐AST (FAST) score > 0.67 [2]. Logistic regression models were used to identify factors associated with these outcomes including HIV status as covariate.

**Results**: A total of 1605 adults (1060 with HIV) underwent TE. Compared with participants without HIV, PLWH were older (median: 54 [95% CI: 47–61] vs. 41 [35–51] years), more often white (63.9 and 30.6%) and had higher BMI (26.8 [24.1–30.1] and 25.1 [22.2–28.4] kg/m^2^); alcohol consumption above recommended limits was higher (9.5% vs. 4.7%).The prevalence of SLD in PLWH was 37.0% versus 19.0% in those without it (*p* < 0.001), and PLWH had higher CAP score (244 [interquartile range {IQR} 207–289] vs. 218 [186; 257] dB/m; *p* < 0.001), higher liver stiffness score (4.9 [IQR 4.1; 5.9] vs. 4.7 [IQR 3.9; 5.6] kPa, *p* = 0.020) and FAST score (0.21 [IQR 0.12; 0.32] vs. 0.13 [0.08; 0.21]; *p* < 0.001). After adjusting for covariates, HIV status was independently associated with SLD (odds ratio [OR] 1.39; [95% CI: 1.00; 1.91]; *p* = 0.047) (Table 1) and at‐risk MASH (OR 2.97; [1.0; 8.83]; *p* = 0.05). In a sensitivity analysis limited to participants older than 45 years (to improve comparability between groups), HIV continued to be associated with SLD (OR 1.55, [1.05; 2.29], *p* = 0.028) after adjustment for sex, ethnicity and BMI.

**Conclusions**: Compared with those not living with HIV, PLWH had a higher prevalence of moderate/severe SLD and at‐risk MASH. HIV status remained associated with SLD and MASH after adjustment for relevant covariates suggesting HIV confers additional risk beyond known risk factors such as increased BMI or age.

**References**

1. Karlas T, Petroff D, Sasso M, et al. Individual patient data meta‐analysis of controlled attenuation parameter (CAP) technology for assessing steatosis. *J Hepatol* 2017; 66:1022–1030.

2. Newsome PN, Sasso M, Deeks JJ, et al. FibroScan‐AST (FAST) score for the non‐invasive identification of patients with non‐alcoholic steatohepatitis with significant activity and fibrosis: A prospective derivation and global validation study. *Lancet Gastroenterol Hepatol* 2020;5(4):362–373.

**TABLE 1:**
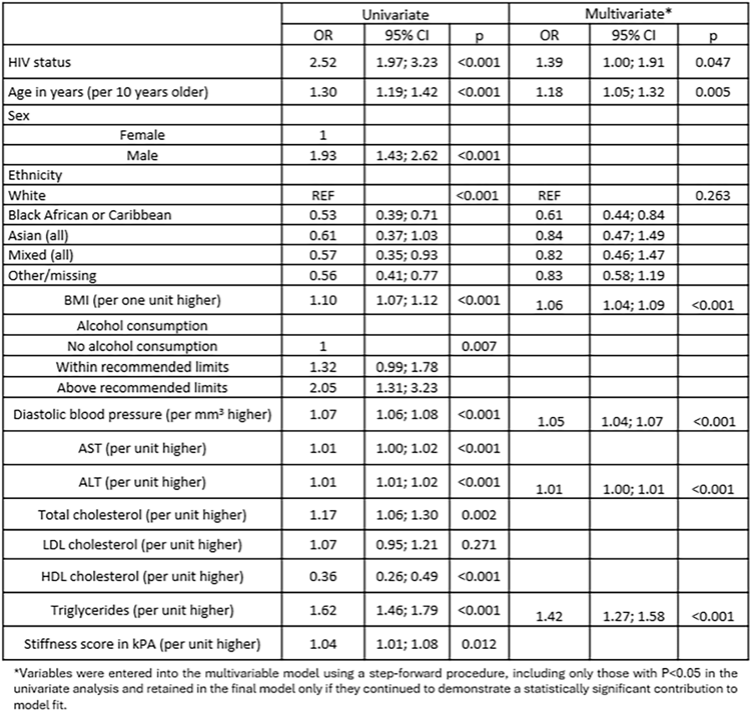
P419 | Summary of logistic regression models for factors associated with moderate/severe SLD.

### P420 Diabetes Incidence Trends and Statin Use Among People Living With HIV, 2016–2025

Golnaz Torkjazi^1,2^, Tommaso Clemente^1^, Nicolò Capra^1^, Riccardo Lolatto^1^, Emanuela Messina^1^, Diana Canetti^1^, Giovanni Festa^1,2^, Dario Diana^1^, Gabriele Loi^1,2^, Silvia Nozza^1,2^, Antonella Castagna^1,2^, Vincenzo Spagnuolo ^1,2^

^1^Unit of Infectious Diseases, San Raffaele Scientific Institute, Milan, Italy; ^2^School of Medicine, Vita‐Salute San Raffaele University, Milan, Italy

**Background**: Diabetes is a major comorbidity in ageing people living with HIV (PLWH), in whom statins are a cornerstone of cardiovascular prevention. We aimed to evaluate real‐world temporal trends in diabetes incidence and its association with statin use from 2016 to 2025.

**Methods**: Real‐world cohort study including PLWH followed at San Raffaele Scientific Institute, Milan, Italy, between 2016 and 2025. Annual diabetes incidence was expressed as new cases per 1000 people at risk, and statin use as annual prevalence. Incident diabetes was diagnosed based on HbA1c ≥48 mmol/mol (6.5%), fasting glucose ≥126 mg/dL on two occasions and random glucose ≥200 mg/dL. Descriptive characteristics were summarized as median (interquartile range) or number (%) and compared using Wilcoxon rank‐sum, chi‐square or Fisher's exact tests. Associations with incident diabetes were assessed using GEE logistic regression models accounting for repeated observations. Temporal trends in diabetes incidence were assessed before and after adjustment for age, sex and statin use.

**Results**: Overall, 6143 PLWH were included, of whom 501 developed diabetes (8.2%). Individuals with diabetes were older, had longer HIV duration, lower CD4+ counts, higher rates of HCV positive serology, and were more frequently receiving statins than those without diabetes (Table 1). Diabetes incidence was 11.2/1000 persons at risk, ranging from 7.7 in 2017 to 14.5 in 2024. Statin use increased from 18.4% in 2016 to 42.5% in 2025 (Figure 1). In univariable GEE analysis, calendar year was associated with increasing diabetes incidence over time (OR/year 1.04, 95% CI: 1.01–1.07; *p* = 0.019), but this association was lost after adjustment for age, sex and statin use (aOR 0.99, 95% CI: 0.96–1.02; *p* = 0.607). In the multivariable model, older age (OR/year 1.05, 95% CI: 1.04–1.06; *p* < 0.001) and statin use (OR 1.51, 95% CI: 1.25–1.81; *p* < 0.001) remained independently associated with incident diabetes, whereas female sex‐assigned‐at‐birth showed a borderline inverse association (OR 0.81, 95% CI: 0.64–1.02; *p* = 0.070).

**Conclusions**: In this real‐world PLWH cohort, the crude increase in diabetes incidence over time was attenuated after adjustment. Although the clinical benefits of statins remain unquestionable in individuals with elevated cardiovascular risk, their association with incident diabetes supports metabolic monitoring alongside indicated therapy.

**FIGURE 1:**
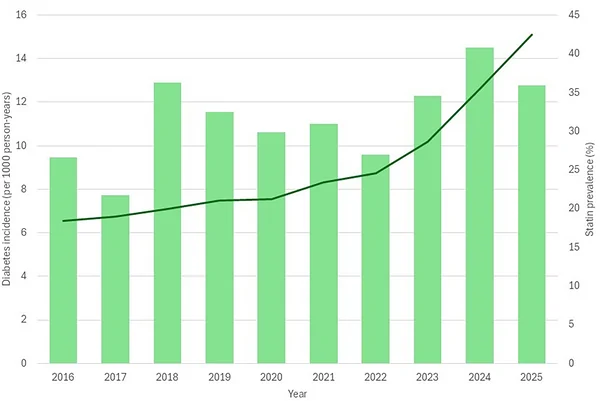
P420 | Annual diabetes incidence and statin use among PLWH, 2016–2025. Diabetes incidence is shown as bars and reported as new cases per 1000 person‐years. Statin use is shown as a line and reported as annual prevalence.

**TABLE 1:**
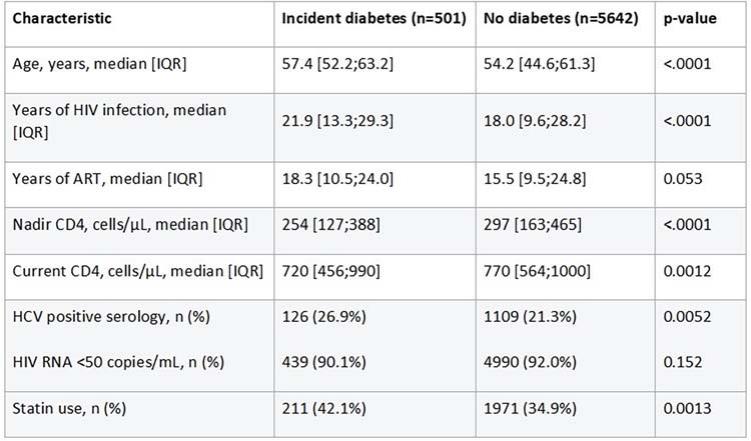
P420 | Characteristics of subjects with and without diabetes.

### P421 Acceptability of Body Composition Assessment Methods in People Living With HIV and/or Chronic Hepatitis B: Findings From the BEHIVe Study

Florence Bascombe ^1,2^, Catherine Johnson^1,2^, Jennifer Curley^1,2^, Ebunoluwa Taiwo^1,2^, Christos Karathanasis^1,2^, Laura Hennelly^1,2^, Deirdre Sally^1,2^, Jonathan Wells^3^, Alun Hughes^4^, Stuart Flanagan^1,2^, Oliver Stirrup^1,2^, Sarah Pett^1,2^, Richard Gilson^1,2^, Alejandro Arenas‐Pinto^1,2^

^1^Centre for Clinical Research in Infection and Sexual Health, Institute for Global Health, University College London, London, UK; ^2^The Mortimer Market Centre, Central and North West London NHS Foundation Trust, London, UK; ^3^Department of Population, Policy and Practice, Institute of Child Health, University College London, London, UK; ^4^Research Department of Population Science & Experimental Medicine, Institute of Cardiovascular Science, University College London, London, UK

**Background**: Weight gain and altered fat distribution are important concerns in HIV care, with implications for metabolic health. Because metabolic risk may be driven by factors such as visceral adiposity, fat distribution and lean mass, body composition measurements may better reflect metabolic health than body size measures alone. However, clinical decisions are often based on body mass index (BMI) which inadequately characterizes body composition. Bioelectrical impedance analysis (BIA), three‐dimensional body scanning (3D BS) and silhouette‐based tools such as the Stunkard Figure Rating Scale (SFRS) may improve assessment but need to be acceptable for use in clinical practice.

**Methods**: BEHIVe is a cross‐sectional study evaluating the acceptability of BIA, 3D BS and the 9‐item SFRS in adults with HIV and/or chronic hepatitis B attending an HIV service. Acceptability was assessed using adapted questionnaires informed by the Theoretical Framework of Acceptability (TFA) [1]. Composite acceptability scores (range 1–5) were derived across six domains, with negatively worded items reverse coded. Results are presented as medians (IQR). Differences between methods were assessed using Wilcoxon signed‐rank tests. Free‐text comments were reviewed descriptively.

**Results**: We recruited 101 participants, median age 53 years (IQR 44–61), median BMI 27.3 kg/m^2^ (24.1–29.6), and 84% were male at birth. Most were living with HIV alone (94%), 3% chronic HBV alone, 3% HIV/HBV co‐infection. Acceptability was high across all methods (Figure 1 and Table 1). Median composite acceptability scores were highest for BIA (4.67; 4.33–5.00) and 3D BS (4.75; 4.50–5.00), and lower for SFRS (4.17; 3.67–4.50). There was no difference between BIA and 3D BS (*p* = 0.50), both were more acceptable than SFRS (both *p* < 0.001). Despite a shorter completion time (median 1 vs. 10.5 and 12 min), free‐text comments suggested lower SFRS acceptability reflected concerns around body representation, inclusivity and emotional sensitivity.

**Conclusions**: Body composition assessment methods were highly acceptable among people with HIV, and small groups with chronic HBV/HIV. Despite requiring more time, objective body composition methods (BIA and 3D BS) were preferred over silhouette‐based assessment. This suggests these methods would be feasible for implementation in routine HIV care as well as research.

**Reference**

1. Sekhon M, Cartwright M, Francis JJ. Development of a theory‐informed questionnaire to assess the acceptability of healthcare interventions. *BMC Health Serv Res*. 2022;22:279.

**FIGURE 1:**
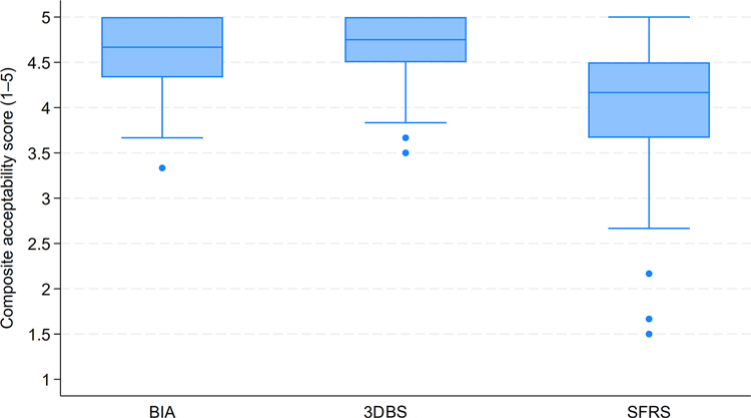
P421 | Distribution of participant‐level mean composite acceptability scores for bioelectrical impedance analysis, three‐dimensional body scanning and Stunkard Figure Rating Scale among people with HIV and/or chronic hepatitis B. Higher scores indicate greater acceptability.

**TABLE 1:**
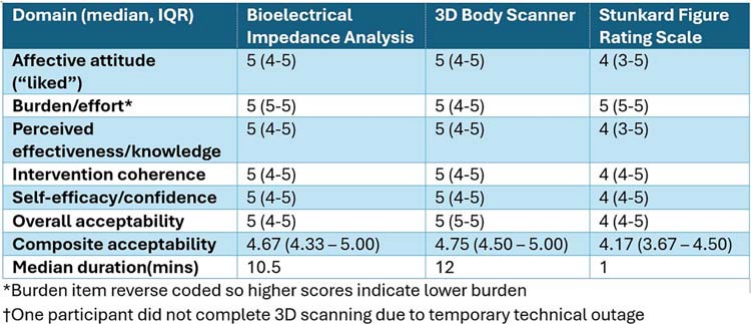
P421 | Acceptability of Theoretical Framework of Acceptability (TFA) construct and assessment duration for bioelectrical impedance analysis, three‐dimensional body scanning and Stunkard Figure Rating Scale.

### P422 After REPRIEVE: Are We Treating the Right Patients?

Beatrice Barda^1^, Nadine Clea Natter^1^, Lorenzo Magenta^2^, Alessandra Bruno^2^, Bruno Bertola^1^, Enos Bernasconi^1^, Marco Bongiovanni ^1,3^

^1^Division of Infectious Diseases, Ente Ospedaliero Cantonale, Lugano, Switzerland; ^2^Epatocentro, Ticino, Lugano, Switzerland; ^3^Department of Biomedical Sciences, University of Southern Switzerland, Lugano, Switzerland

**Background**: People with HIV (PWH) remain at increased risk of atherosclerotic cardiovascular disease (ASCVD) despite effective antiretroviral therapy. The REPRIEVE trial demonstrated that statin therapy reduces major cardiovascular events even among PWH at low‐to‐moderate risk, suggesting that a broader population may benefit from primary prevention. However, identifying candidates for lipid‐lowering therapy remains challenging, as most prediction tools were developed in HIV‐uninfected populations and may not fully capture cardiovascular risk in PWH.

**Methods**: We included in this study all consecutive PWH followed at HIV Clinic of Ente Ospedaliero Cantonale, Ticino, Southern Switzerland, between January 2025 and April 2026, who fulfilled key REPRIEVE eligibility criteria, including age 40–75 years, LDL‐C < 4.4 mmol/L, low‐to‐moderate (<7.5%) 10‐year ASCVD risk according to the Pooled Cohort Equations (PCE), and absence of known cardiovascular disease. Baseline statin use was assessed to evaluate the implementation of cardiovascular prevention strategies. Cardiovascular risk was further evaluated using alternative risk scores, including D:A:D, PREVENT‐ASCVD, AGLA and SCORE2.

**Results**: Among 297 PLWH included in the overall cohort, 89 (30.0%) fulfilled key REPRIEVE eligibility criteria; 48 (53.9%) were men, mean age was 55.3 years, mean BMI was 25.3 kg/m^2^, and all participants were virologically suppressed. Mean PCE 10‐year ASCVD risk at baseline was 5.7%. Twenty‐three participants (25.8%) were receiving statin therapy at baseline. After 12 months of follow‐up, mean PCE 10‐year ASCVD risk remained stable at 5.7% and only 3 additional participants started statin (26/89, 29.2%). Thus, more than two‐thirds of potentially eligible individuals remained untreated despite 1 year of follow‐up. Cardiovascular risk classification varied markedly according to the risk score applied. While all participants met low‐to‐moderate risk criteria by PCE, 81/89 (91.0%) were classified at moderate‐to‐high risk by D:A:D, compared with only 27/89 (30.3%) by SCORE2, 5/89 (5.6%) by AGLA and 2/89 (2.2%) by PREVENT‐ASCVD.

**Conclusions**: In this real‐world cohort, nearly three‐quarters of individuals fulfilling key REPRIEVE eligibility criteria were not receiving statin therapy, and treatment uptake remained minimal over 12 months of follow‐up. The marked discordance across cardiovascular risk scores highlights the challenges of translating REPRIEVE findings into European clinical practice and supports the development and validation of HIV‐specific cardiovascular risk assessment tools.

### P423 Discordance Among Cardiovascular Risk Prediction Tools in People Living With HIV: A Contemporary Cohort Study

Beatrice Barda^1^, Enos Bernasconi^1^, Bruno Bertola^1^, Lorenzo Magenta^2^, Alessandra Bruno^2^, Marco Bongiovanni ^1,3^

^1^Division of Infectious Diseases, Ente Ospedaliero Cantonale, Lugano, Switzerland; ^2^Epatocentro, Ticino, Lugano, Switzerland; ^3^Department of Biomedical Sciences, University of Southern Switzerland, Lugano, Switzerland

**Background**: People living with HIV (PLWH) have an increased risk of atherosclerotic cardiovascular disease (ASCVD) compared with the general population. However, the optimal approach for ASCVD risk prediction in PLWH remains uncertain. The aim of this study was to compare ASCVD risk stratification across different prediction tools and assess their agreement in contemporary PLWH.

**Methods**: We included 299 PLWH aged 30–70 years, without a known history of cardiovascular diseases. Evaluation of ASCVD risk factors was performed during routine follow‐up visits. ASCVD risk was calculated using Pooled Cohort Equations (PCE), SCORE2, D:A:D, PREVENT and AGLA. Participants were categorized into low, moderate or high 10‐year ASCVD risk. Concordance of continuous risk estimates was assessed with Lin's concordance correlation coefficient and Bland–Altman analysis; categorical agreement was evaluated using chi‐square tests.

**Results**: Median age was 58 years; 70.6% were male and all were virally suppressed. Only one participant was classified at high risk across all scores. The proportion of PWH classified at high 10‐year ASCVD risk varied markedly depending on the score used: 152 with PCE (51%), 24 with SCORE2 (8%), 227 with D:A:D (76.2%), 33 with PREVENT (11.1%) and 15 with AGLA (5%) (*p* < 0.001); concordance across low and moderate risk categories was limited. Lin's concordance correlation coefficients demonstrated low agreement between most pairs of scores (CCC range: 0.16–0.51); Bland–Altman analyses showed poor concordance with wide limits of agreement in most cases and revealed systematic biases, with some tools consistently providing higher or lower risk estimates compared with others (Figure 1). Among PLWH aged < 50 years, discordance across risk scores remained substantial, with only 6 (8.7%), 3 (4.3%) and 14 (20.3%) individuals classified as high risk by PCE, SCORE2 and D:A:D, respectively, whereas no participants were classified as high risk using PREVENT or AGLA (*p* < 0.001).

**Conclusions**: ASCVD risk prediction in PLWH is highly heterogeneous, with poor agreement between HIV‐specific and general population tools. This heterogeneity may lead to substantially different preventive strategies, particularly among younger individuals. These findings support the development and validation of HIV‐specific cardiovascular risk models to improve cardiovascular prevention in contemporary PLWH.

**FIGURE 1:**
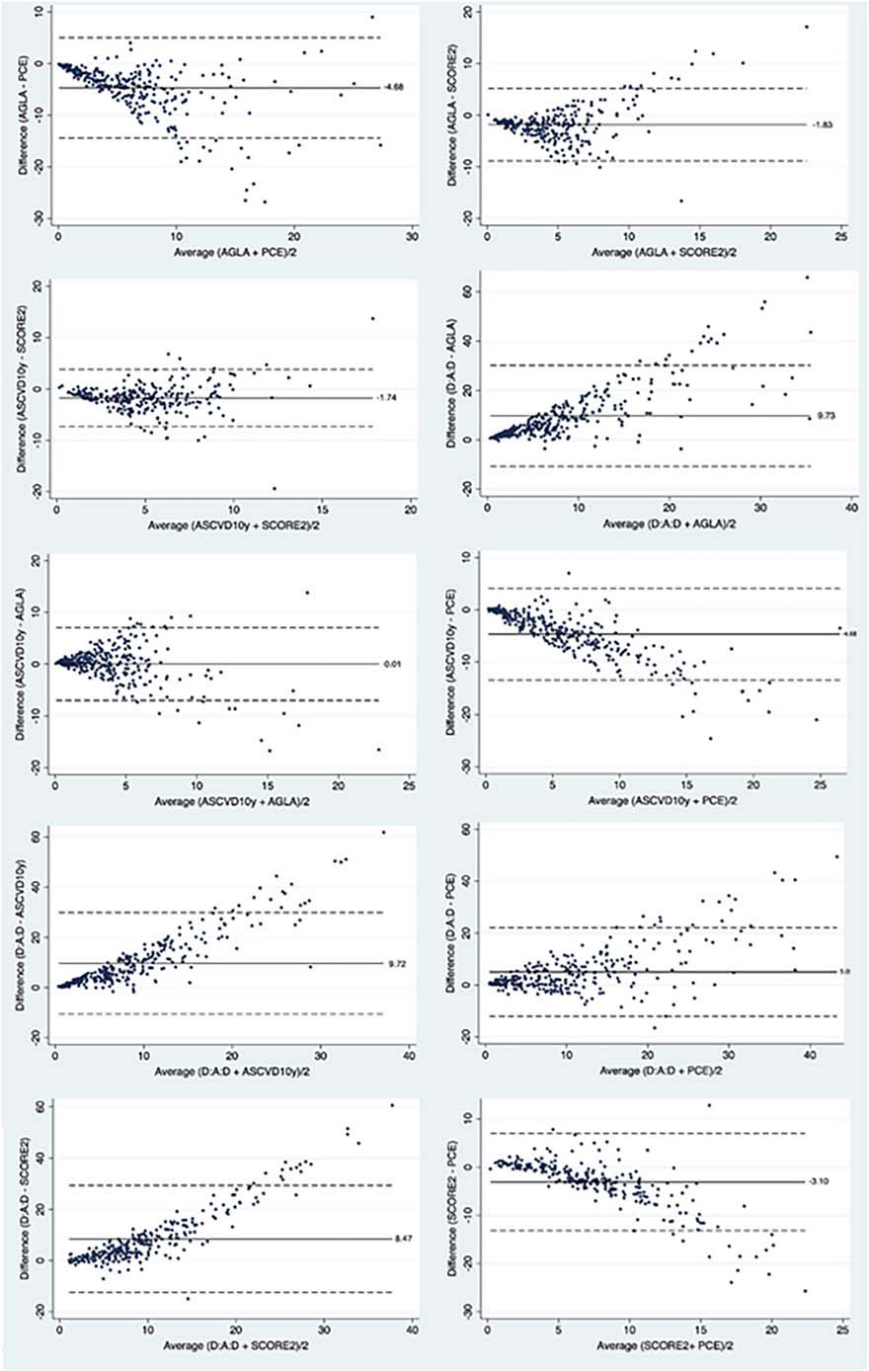
P423 | Bland–Altman plots comparing pairwise agreement between 10‐year cardiovascular risk prediction models.

### P424 Performance of the SCORE2 Cardiovascular Risk Model in People Living With HIV Diagnosed Prior to the Modern ART Era

Lieven Legius ^1^, Rosa Elias^1,2^, Casper Rokx^2^, Annelies Verbon^1^, Berend van Welzen^1^

^1^Department of Infectious Diseases, University Medical Centre Utrecht, Utrecht, Netherlands; ^2^Department of Internal Medicine, and Department of Medical Microbiology and Infectious Diseases, Erasmus University Medical Centre, Rotterdam, Netherlands

**Background**: People living with HIV (PLWH) bear a substantially higher risk of atherosclerotic cardiovascular disease (ASCVD) than the general population. HIV clinicians widely rely on prediction models to guide preventive strategies, and the EACS guidelines recommend the SCORE2 model to estimate the 10‐year risk of a first ASCVD event. However, its performance may differ across subgroups because many PLWH were exposed to earlier HIV treatment strategies, including more toxic antiretroviral therapy (ART) regimens and delayed ART initiation until advanced immunodeficiency, both of which may have lasting effects on cardiovascular health. We, therefore, compared the performance of SCORE2 between PLWH exposed to these earlier treatment strategies and those who initiated less toxic ART at higher CD4 cell counts in later calendar periods.

**Methods**: We conducted a single‐centre retrospective cohort study of all PLWH aged 40–90 years, without prior ASCVD or diabetes, in care in 2016. At baseline, SCORE2 was applied to estimate the risk of a first ASCVD event (i.e. non‐fatal myocardial infarction, non‐fatal stroke or cardiovascular death), with subsequent 10‐year follow‐up until the first event or until competing non‐cardiovascular death. Participants were stratified into either HIV diagnosis in early ART era (before 2006) or late ART era (after 2006). SCORE2 performance was assessed by calibration using competing‐risk adjusted expected‐to‐observed (E/O) ratios and calibration plots, and by discrimination using time‐dependent C‐statistic.

**Results**: Of 893 eligible patients, 459 were in the early‐ART group and 434 in the late‐ART group (Table 1). Overall, SCORE2 underestimated cardiovascular risk, with an E/O ratio of 0.65 (95% CI: 0.52–0.85) (Figure 1). This underestimation was driven by the early‐ART group (E/O ratio 0.48, 95% CI: 0.37–0.66), while the late‐ART group showed adequate calibration (E/O ratio 1.09, 95% CI: 0.74–2.04). The C‐statistic was 0.68 (95% CI: 0.55–0.80) in the early‐ART group and 0.67 (95% CI: 0.52–0.84) in the late‐ART group, indicating moderate discrimination in both groups.

**Conclusions**: SCORE2 showed underestimation of cardiovascular risk, particularly in the early‐ART group, where twice as many events occurred as predicted. These findings suggest that current prediction models are insufficient for PLWH diagnosed before the modern ART era, warranting intensified prophylactic interventions in this at‐risk group.

**FIGURE 1:**
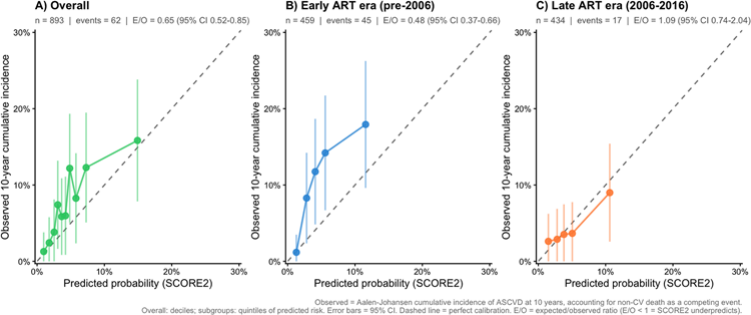
P424 | SCORE2 calibration (competing‐risk adjusted), stratified by timing of HIV diagnosis. Determinants of SCORE2 include age, sex, smoking status, systolic blood pressure, non‐high density lipoprotein cholesterol.

**TABLE 1:**
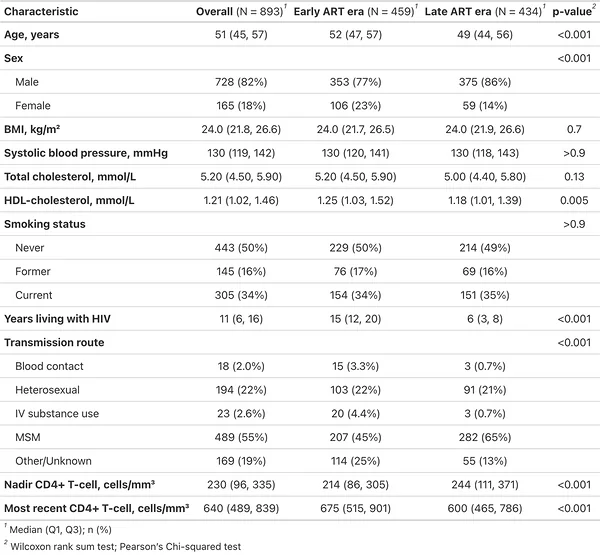
P424 | Baseline characteristics at study entry for the overall population and stratified by ART era, including demographics, cardiovascular parameters and HIV‐specific parameters.

### P425 Efficacy, Metabolic Safety and Patient Satisfaction of DTG/3TC Versus BIC/TAF/FTC in Treatment‐Naïve Men Living With HIV: 24‐Week Results From the Randomized TEOTL Trial

Paola Edith Padilla Noguera^1^, Marco Antonio Cruz León^1^, José Antonio Mata Marín^2^, Ana Luz Cano Díaz ^2^, Gloria Elizabeth Salinas Velázquez^3^, Emily Itzel Pecero García^2^, Alan De León Aguilar^4^, Alberto Chaparro Sánchez^2^, Ericka Nelly Pompa Mera^2^, Jesús Enrique Gaytán Martínez^2^

^1^Medicine School, Universidad Nacional Autónoma de México, Mexico City, Mexico; ^2^Infectious Diseases Department, CMN La Raza, Mexico City, Mexico; ^3^Department of Radiology, CMN La Raza, Mexico City, Mexico; ^4^Internal Medicine department, Hospital Magdalena de las Salinas, Mexico City, Mexico

**Background**: Emerging evidence links integrase strand transfer inhibitors (INSTIs) to weight gain and metabolic disturbances, raising concerns about long‐term cardiovascular risk. In Mexico, data regarding treatment‐naïve populations remain scarce. The aim of this study is to compare DTG/3TC versus BIC/TAF/FTC regarding their clinical efficacy, metabolic changes, treatment satisfaction and ART‐related distress.

**Methods**: This open‐label, randomized clinical trial was conducted at the Hospital de Infectología, “La Raza” National Medical Center in Mexico City, and included ART‐naïve men living with HIV (MLH). After recording baseline anthropometric and metabolic parameters, outcomes were evaluated at week 24. These included virologic suppression (HIV‐1 RNA <50 copies/mL), CD4+ cell count, weight, body mass index (BMI), fasting glucose, lipid profile, metabolic syndrome incidence and patient‐reported outcomes via the HIV Treatment Satisfaction Questionnaire (HIVTSQ) and HIV Symptoms Distress Module (HIV‐SDM).

**Results**: This preliminary analysis included 166 treatment‐naïve MLH: 83 randomized to DTG/3TC and 83 to BIC/TAF/FTC. Median age was 25 years (IQR 22–31), baseline median weight 63.8 kg (IQR 57.2–70.1) and mean BMI 22.1 kg/m^2^. Baseline clinical markers were comparable: median HIV‐1 RNA was 33,469 versus 48,923 copies/mL (*p* = 0.690) and median CD4+ count 278 versus 283 cells/mm^3^ (*p* = 0.908). At 24 weeks, virologic suppression (<50 copies/mL) rates were 90.3% for DTG/3TC and 95.1% for BIC/TAF/FTC (*p* = 0.370). Median CD4+ counts increased to 599 cells/mm^3^ (IQR 435–873) and 594 cells/mm^3^ (IQR 476–806), respectively (*p* = 0.872). No significant differences were observed regarding metabolic changes: 24‐week median weight was 68.0 kg (IQR 60.8–73.6) versus 65.6 kg (IQR 59.6–73.1; *p* = 0.378), with a mean weight gain of +2.2 versus +2.9 kg (*p* = 0.303). Mean BMI was 23.3 versus 22.9 kg/m^2^ (*p* = 0.397), mean body fat 19.0% versus 19.2% (*p* = 0.823) and muscle mass 51.0 versus 50.2 kg (*p* = 0.115). Metabolic syndrome developed in only one participant (1.3%, DTG/3TC group; *p* = 0.494). Median HIVTSQ: 59.0 (IQR 56.2–60) versus 58.0 (IQR 55–60); *p* = 0.124; median HIV‐SDM: 6.0 versus 5.0; *p* = 0.306, in DTG/3TC and BIC/TAF/FTC, did not differ between regimens (Figure 1 and Table 1).

**Conclusions**: Both regimens demonstrated high efficacy and similar metabolic safety at 24 weeks. Treatment satisfaction and symptom‐related distress did not differ significantly between groups.

**FIGURE 1:**
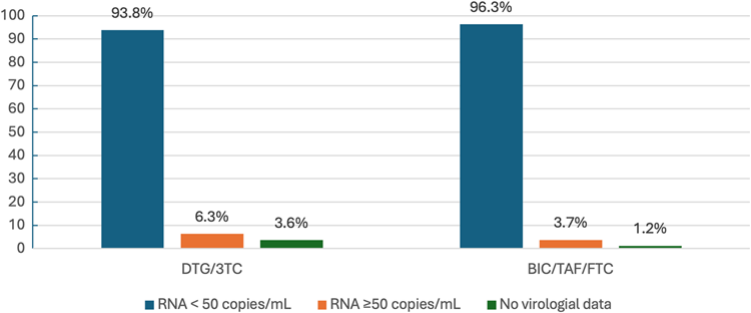
P425 | Efficacy of DTG/3TC versus BIC/TAF/FTC on PLH naïve to ART at 24 weeks.

**TABLE 1:**
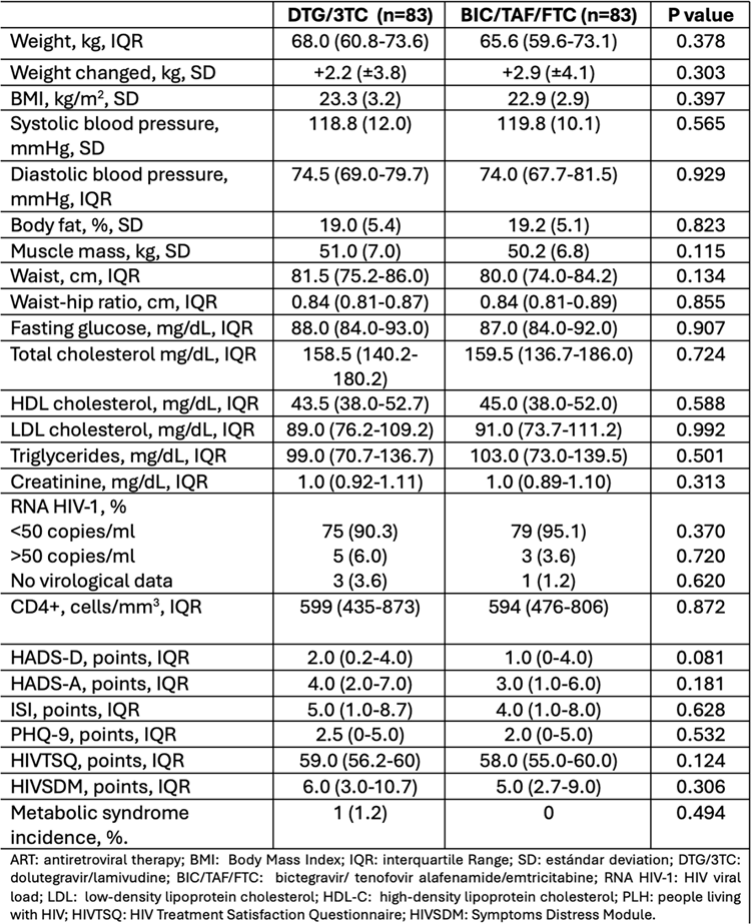
P425 | Clinical and biochemical characteristics in PLH at 24 weeks after randomization with BIC/TAF/FTC or DTG/3TC.

### P426 One Patient, Two Visits, Five Answers: Longitudinal Reclassification of Cardiovascular Risk in People With HIV

Beatrice Barda ^1^, Bruno Bertola^1^, Enos Bernasconi^1^, Lorenzo Magenta^2^, Marco Bongiovanni^1^

^1^EOC Infectious Diseases, Lugano, Switzerland; ^2^Epatocentro Ticino, Lugano, Switzerland

**Background**: Cardiovascular risk assessment is routinely recommended for people with HIV (PWH), yet substantial disagreement exists among available prediction tools. Little is known about how cardiovascular risk classification changes over time in contemporary PWH receiving effective antiretroviral therapy. We, therefore, evaluated the longitudinal stability of risk categorization across commonly used prediction models over 12 months.

**Methods**: We included 222 PWH without known cardiovascular disease who had complete cardiovascular risk assessment at baseline and after 12 months of follow‐up. Ten‐year ASCVD risk was calculated using D:A:D, PREVENT–ASCVD, AGLA, SCORE2 and Pooled Cohort Equations (PCE). Participants were categorized as low, moderate or high cardiovascular risk according to each score. Changes in risk category between baseline and follow‐up were evaluated overall and stratified by age (<50 vs. ≥50 years). SCORE2 was applied only to participants aged 40–69 years according to the validated age range for this tool.

**Results**: Participants were predominantly male and all were virologically suppressed. Overall, cardiovascular risk classification remained relatively stable across all prediction tools over 12 months; however, the proportion of participants reclassified varied substantially according to the score used. Among PWH aged < 50 years, reclassification rates ranged from 0% with PREVENT‐ASCVD to 27.3% with SCORE2, while intermediate rates were observed with D:A:D (5.9%), AGLA (9.8%) and PCE (17.6%). In participants aged ≥50 years, reclassification ranged from 12.9% with D:A:D to 25.4% with PREVENT‐ASCVD, compared with 15.8% for AGLA, 22.2% for SCORE2 and 19.9% for PCE (Table 1). The direction of reclassification also differed between tools. D:A:D and PCE tended to classify a greater proportion of participants as high risk at follow‐up, whereas PREVENT‐ASCVD and AGLA generally shifted participants towards lower‐risk categories. SCORE2 showed bidirectional movement between categories despite relatively stable overall distributions.

**Conclusions**: In virologically suppressed PWH, cardiovascular risk classification remained largely stable over 1 year, although reclassification rates and directions of change varied substantially across prediction tools. The observed differences between risk scores exceeded the changes occurring over time, suggesting that score selection may have a greater impact on cardiovascular risk categorization than short‐term longitudinal changes in risk factors. These findings support the development and validation of HIV‐specific cardiovascular risk assessment tools.

**TABLE 1:**
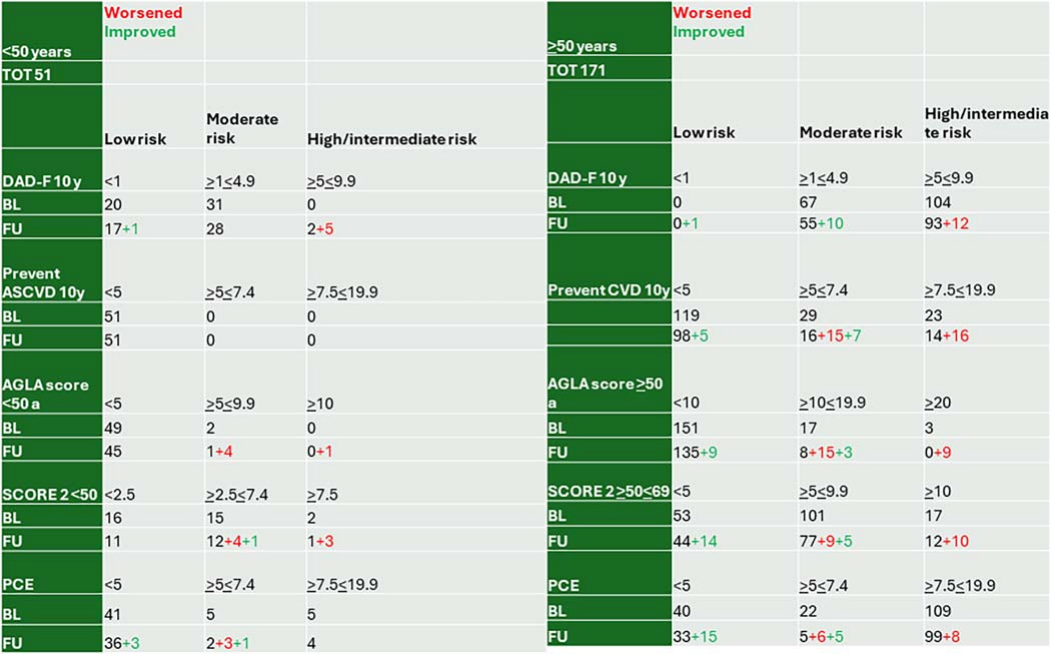
P426 | Evolution of CVD risk scores from baseline (BL) to follow‐up (FU) in the two groups (<50 and >50 years).

### P427 The Cost of Looking Too Young: Missed Opportunities for Cardiovascular Prevention in People With HIV

Beatrice Barda ^1^, Enos Bernasconi^1^, Bruno Bertola^1^, Lorenzo Magenta^2^, Alessandra Bruno^2^, Marco Bongiovanni^1,3^

^1^Division of Infectious Diseases, Ente Ospedaliero Cantonale, Lugano, Switzerland; ^2^Epatocentro, Ticino, Lugano, Switzerland; ^3^Department of Biomedical Sciences, University of Southern Switzerland, Lugano, Switzerland

**Background**: People with HIV (PWH) remain at increased risk of atherosclerotic cardiovascular disease (ASCVD) despite effective antiretroviral therapy. Cardiovascular risk stratification is essential to guide preventive interventions; however, most commonly used risk scores were developed in HIV‐uninfected populations and their applicability to PWH remains uncertain.

**Methods**: To assess statin use among PWH meeting score‐based criteria for lipid‐lowering therapy according to SCORE2, AGLA and PCE, and to evaluate changes in treatment over 12 months follow‐up.

**Results**: 212 participants with complete 12‐month‐follow‐up data (mean age 55.3 years, 70.3% male, all virally suppressed) were included. Among 161 participants >50 years, 108 (67.1%), 121 (75.2%) and 19 (11.8%) were classified at moderate‐to‐high risk by SCORE2, PCE and AGLA, respectively. Among these, 51 (47.2%), 57 (47.1%) and 11 (57.9%) participants classified at moderate‐to‐high risk by SCORE2, PCE and AGLA, respectively, were receiving statin therapy. Statin underutilization was markedly more severe among 51 participants < 50 years. Participants classified at moderate‐to‐high risk were 17 (33.3%), 10 (19.6%) and 2 (4%) using SCORE2, PCE and AGLA; among these, 2/17 (11.8%), 1/10 (10.0%) and 2/2 (100%) were receiving statins, corresponding to approximately four‐fold lower statin use than in older participants. Overall, statin use among at‐risk PWH < 50 years remained around 10–15%, compared with approximately 50% among older participants (*p* < 0.001).

During 12‐month follow‐up, statin initiation remained minimal among younger PWH, with only one additional participant initiating therapy. At 12 months, 22, 12 and 6 participants < 50 years were classified at moderate‐to‐high risk according to SCORE2, PCE and AGLA, respectively. Among participants aged >50 years, 7 and 8 individuals meeting SCORE2 and PCE criteria, respectively, initiated statin therapy. Overall, at follow‐up, 3/22 (13.6%) participants < 50 years on SCORE2, 2/12 (16.7%) on PCE and 1/6 (16.7%) on AGLA were receiving statins, compared to 58/103 (56.3%) on SCORE2, 59/119 (49.6%) on PCE and 13/25 (52%) on AGLA among participants > 50 years (Figure 1).

**Conclusions**: Statins remain underutilized among PWH, particularly in younger individuals, despite meeting criteria supporting their use. These findings highlight missed opportunities for cardiovascular prevention in younger PWH and support earlier and more proactive implementation of guideline‐based lipid‐lowering strategies.

**FIGURE 1:**
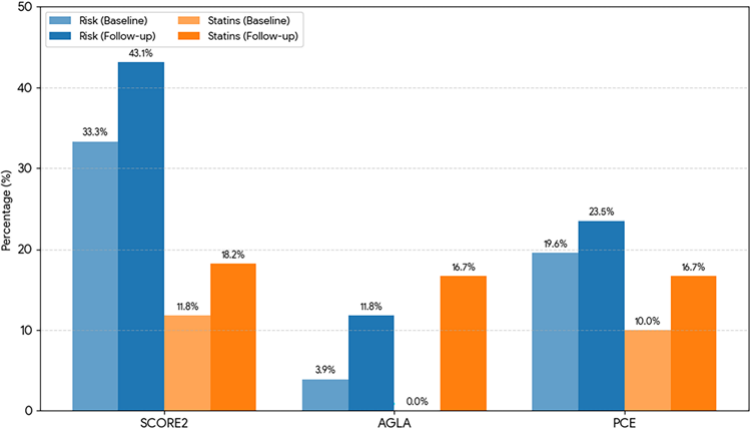
P427 | Cardiovascular risk classification and statin use in people with HIV < 50 years old.

### P428 Factors Associated With Liver Enzyme Elevation in People Living With HIV: Metabolic, Viral and Endocrine Co‐Factors Outweigh Antiretroviral Exposure in the Integrase Inhibitor Era

Natalia Laufer ^1,2^, Yasmin El Kozah^3^, Jorge Gentile^4^, Emilia Cohen^5^, Marcelo Gañete^6^, Gabriela Poblete^3^, Graciela Guaragna^7,8^, Florencia Ferrero^3,9^, Raquel Zhumi^10^, Fabricio Morales^3,11^, Marina Grand^12^

^1^Infectious Diseases Unit, Hospital Juan A Fernández, Buenos Aires, Argentina; ^2^CONICET‐Universidad de Buenos Aires, Instituto de Investigaciones Biomédicas en Retrovirus y SIDA (INBIRS), Buenos Aires, Argentina; ^3^Centro Médico Accord (CEMAC), Buenos Aires, Argentina; ^4^Hospital Municipal “Ramón Santamarina”, Tandil, Argentina; ^5^Hospital Interzonal General de Agudos “Eva Perón” (ex Castex), San Martin, Argentina; ^6^Hospital Higa Evita Lanús, Infectious Diseases Department, Lanu, Argentina; ^7^Unidad de Infectología Hospital Teodoro Alvarez, Buenos Aires, Argentina; ^8^Sanatorio Nuestra Señora del Pilar, Ciudadela, Argentina; ^9^Sanatorio Trinidad Mitre, Buenos Aires, Argentina; ^10^Sanatorio San Justo, San Justo, Argentina; ^11^Servicio de Infectología Sanatorio Anchorena, San Martin, Argentina; ^12^Hospital Municipal “Dr. Héctor M. Cura”, Olavarria, Argentina

**Background**: HIV infection and old antiretroviral therapy (ART) contribute to hepatic inflammation in people living with HIV (PLWH) [1], new data in the integrase strand transfer inhibitor (INSTI) era is scarce. Factors associated with abnormal liver tests beyond viral hepatitis, and whether these differ by sex, remain poorly characterized. We aimed to identify factors independently associated with elevated aspartate aminotransferase (AST) and alanine aminotransferase (ALT), including non‐viral and sex‐specific co‐factors, in a multicentre study of PLWH under ART.

**Methods**: Cross‐sectional multicentre study of PLWH attending scheduled visits during 2025.at 9 HIV centres in Buenos Aires City and Province, Argentina. Inclusion required current ART use (≥6 months) and available liver chemistry data. AST/ALT elevation was defined using sex‐specific upper limits of normal (> 35U/L males, > 25U/L females) per ACG guidelines [2]. Associations were assessed by chi‐square/Fisher's exact test and Mann–Whitney U test, stratified by sex. Independent associations were assessed by multivariable logistic regression adjusted for sex, age, body mass index (BMI), years on ART, ART class, viral hepatitis serology, prior hepatotoxic ARV exposure and herbal supplement use.

**Results**: Among 679 PLWH (64.7% male, median age 48 years, 77.6% on INSTI), AST was elevated in 18.6% and ALT in 25.0%, with AST elevation significantly more common in women (24.6% vs. 15.3%, *p* = 0.004). Univariate associations with AST elevation differed by sex: in men, HBsAg positivity (14.1% vs. 3.7%, *p* = 0.003) and herbal/sports supplement use (37.3% vs. 17.0%, *p* = 0.002) were significant, whereas in women, elevated TSH (> 4 mIU/L) was associated (29.6% vs. 7.4%, *p* = 0.006). In multivariable analysis (*n* = 333), BMI and HBsAg+ were independently associated with elevated AST and ALT (Table 1). In the subgroup with TSH tested (*n* = 125), elevated TSH remained independently associated with AST elevation after adjustment (OR 5.02, 95% CI: 1.17–21.52, *p* = 0.030).

**Conclusions**: BMI, HBV co‐infection and elevated TSH emerged as independent correlates of transaminase elevation, with distinct sex‐specific patterns: viral and supplement‐related factors predominated in men, whereas elevated TSH predominated in women. ART class and prior exposure to older ARVs were not independently associated. Because information on drug, alcohol, and herbal supplement use was poorly recorded and may have been preferentially collected in patients with abnormal enzymes, bias cannot be excluded. These findings support sex‐specific screening in individuals with elevated transaminases.

**References**

1.Strauss KE, Phoswa WN, Hanser S, Mokgalaboni K. HIV infection and antiretroviral therapy impair liver function in people living with HIV: Systematic review and meta‐analysis. Pharmaceuticals (Basel). 2025;18(7):955.

2. Kwo PY, Cohen SM, Lim JK. ACG Clinical Guideline: Evaluation of Abnormal Liver Chemistries. Am J Gastroenterol. 2017;112(1):18–35.

**TABLE 1:**
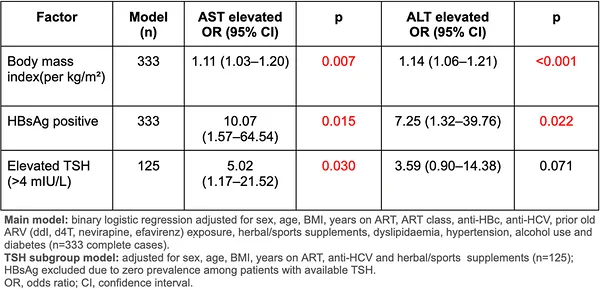
P428 | Factors independently associated with elevated AST and ALT.

### P429 Efficacy, Safety, Metabolic Impact and BMI Evolution After Switching to B/F/TAF in Treatment‐Experienced People With HIV With Obesity

Cinthia Lamaizón, Diego Cecchini, Macarena Roel, Gaston Copertari, Martín Brizuela, Brenda Bacelar, Edgardo Bottaro, Isabel Cassetti

Helios Salud, Buenos Aires, Argentina

**Background**: Obesity prevalence among people with HIV (PWH) on ART has risen globally, often exceeding general population rates and disproportionately affecting women. Assessing weight, BMI categories and metabolic parameters after switching to bictegravir/emtricitabine/tenofovir alafenamide (B/F/TAF) may help inform HIV treatment guidelines in this population.

**Methods**: Subanalysis of the BICTARG cohort, a large Argentine real‐world study of 2632 treatment‐experienced PWH who switched to B/F/TAF, focusing on individuals with obesity (BMI ≥30 kg/m^2^). Follow‐up through 24 months assessed virologic suppression (viral load < 50 c/mL), CD4 T‐cell count, BMI, glucose, lipids and adverse events (AEs). Analyses were stratified by sex and obesity categories.

**Results**: Among 290 participants with obesity (11% of the overall cohort), 189 (65%) were men. Median age was 49 years (IQR, 41–56), and all were Latin American/Hispanic. At baseline, 84% had an undetectable viral load and median CD4+ T‐cell count was 644 cells/mm^3^ (IQR, 453–853). Reasons for switching were simplification (47%), toxicity prevention (38%), current toxicity (13%) and virologic failure (2%). Dyslipidaemia was present in 56%. Median baseline weight was 93.5 kg (IQR, 86.3–103); 68% had mild obesity, 22% had moderate obesity and 10% had severe obesity. Baseline fasting glucose and lipid parameters showed no significant changes at 24 months. At 24 months, both virologic suppression and median CD4+ T‐cell count increased significantly, reaching 98% and 718 cells/mm^3^ (IQR, 531–923), respectively. Treatment‐related AEs occurred in 1.1% at 6 months and in none thereafter. In women, BMI categories shifted towards lower weight categories, with mild obesity decreasing from 57% to 35% and 22% classified as overweight at 24 months; moderate and severe obesity changed minimally (Figure 1a). In men, BMI categories shifted towards higher obesity classes, with mild obesity decreasing from 74% to 50% (*p* = 0.004) and moderate obesity increasing from 19% to 32% (*p* = 0.032) (Figure 1b).

**Conclusions**: In treatment‐experienced PWH and obesity who switched to B/F/TAF, virologic suppression and metabolic parameters remained stable and treatment was well tolerated over 24 months, with sex‐specific changes in BMI categories.

**FIGURE 1:**
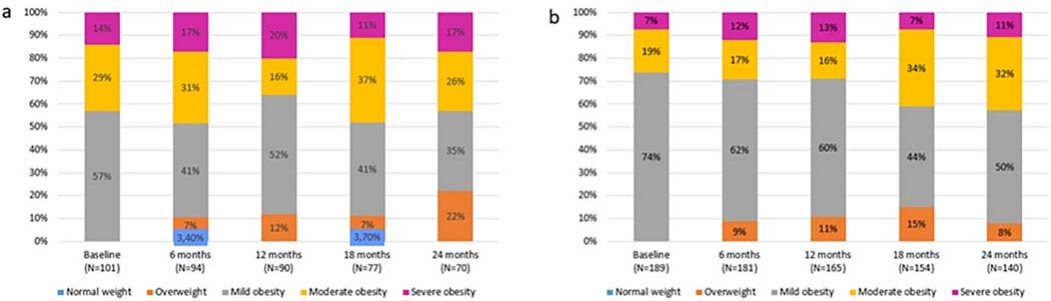
P429 | Distribution of BMI categories at baseline and throughout 24 months of follow‐up among women (a) and men (b) receiving B/F/TAF.

### P430 Accessibility of Glucagon‐Like‐Peptide‐1 Receptor Agonists (GLP‐1 RAs) in Patients With Type 2 Diabetes and HIV Infection

Alan Neiman, Ashley Wright, Brenda Martinez, Gerald Pierone Jr

The Pierone Research Institute, Whole Family Health Center, Vero Beach, FL, USA

**Background**: People living with HIV (PLWH) and type 2 diabetes mellitus (T2DM) are candidates for treatment with glucagon‐like peptide‐1 receptor agonists (GLP‐1 RAs) due to high burdens of obesity, cardiovascular disease, chronic kidney disease and metabolic dysfunction‐associated steatohepatitis (MASH). Despite therapeutic potential, access to and persistence with GLP‐1 RA therapy may be limited by financial barriers, insurance restrictions, medication intolerance and other treatment‐related challenges. This study evaluated GLP‐1 RA utilization and barriers to treatment initiation and persistence among PLWH and T2DM.

**Methods**: This retrospective descriptive observational study was conducted at Whole Family Health Center, a non‐profit community health centre in Vero Beach and Fort Pierce, Florida. Adults (≥18 years) with HIV infection, T2DM and haemoglobin A1C > 6.5% as of July 2025 were included. Demographic and clinical data, medication histories, prescription fill records, and documented reasons for non‐initiation or discontinuation were abstracted from electronic medical records.

**Results**: A total of 147 patients met inclusion criteria (mean age 62.4 years; 56% male). Fifty‐four patients (36.7%) were actively taking therapy. 57 (38.8%) had never been prescribed a GLP‐1 RA. 8 (5.4%) patients were prescribed medication but never initiated therapy. 28 (19%) patients had previously taken GLP‐1 RAs but discontinued treatment (Table 1). Among the 54 patients on GLP‐1 RAs, 26 of them had switched from another GLP‐1 RA. Of the 36 patients who stopped or never started treatment, 20 (55.6%) were not on medication because of insurance exclusion or coverage limitations. Another 11 (30.6%) stopped because of intolerance/adverse effects. All other reasons were less than 10%. Among the 54 patients on treatment, 29 (53.7%) were prescribed semaglutide, 14 (25.9%) tirzepatide, 7 (13%) dulaglutide and 4 (7.4%) oral semaglutide.

**Conclusions**: Although more than half of PLWH with T2DM were prescribed a GLP‐1 RA, only 54 of 147 patients were actively receiving therapy. Financial and insurance‐related barriers were the primary obstacles to initiation and persistence, while medication intolerance was less common. Improving insurance coverage and reducing cost burden promises to expand access and improve outcomes in this high‐risk population.

**TABLE 1:**
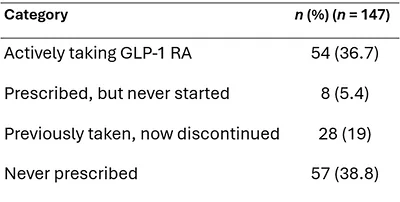
P430 | GLP‐1 RA utilization and barriers among PLWH with T2DM.

### P431 Incidence of Hypertension and Associated Factors in People With HIV Initiating Antiretroviral Therapy With Guideline‐Recommended Regimens Since 2009

Andrea De Vito ^1^, Marta Rava^2,4^, Sergio Padilla^3,4^, Julia Barrado Cuchillo^5^, Marianela Ciudad Sañudo^6^, Maria Saumoy^7^, Cristina Hernández Gutiérrez^8^, Marta Montero‐Alonso^9^, José Ignacio Bernardino^10^, on behalf of CoRIS Cohort

^1^Unit of Infectious Diseases, Sassari University Hospital, Sassari, Italy; ^2^CoRIS Coordination Unit, National Center for Epidemiology, Madrid, Spain; ^3^Infectious Diseases Unit, Hospital General Universitario de Elche, Alicante, Spain; ^4^CIBERINFEC, Carlos III Health Institute, Madrid, Spain; ^5^Facultativo Especialista de Área en Medicina Interna, Hospital Clínico San Carlos, Madrid, Spain; ^6^Sección Enfermedades Infecciosas, Hospital Universitario de La Princesa, Madrid, Spain; ^7^Unidad de VIH y ITS, Servicio de Enfermedades Infecciosas. Hospital Universitari de Bellvitge, Barcelona, Spain; ^8^Servicio de Medicina Interna, Hospital Universitario Príncipe de Asturias Alcalá de Henares, Madrid, Spain; ^9^Unidad de Enfermedades Infecciosas, Servicio de Medicina Interna, Hospital Universitario y Politécnico la Fe, Valencia, Spain; ^10^HIV Unit, Department of Internal Medicine, Hospital Universitario La Paz, IdiPAZ, Madrid, Spain

**Background**: Hypertension is increasingly common in people with HIV (PWH), but the contribution of antiretroviral therapy (ART) remains debated. We assessed whether initial ART regimen was associated with incident hypertension in the CoRIS cohort.

**Methods**: We conducted an observational cohort study including PWH who initiated guideline‐recommended ART from 2009 onwards. Incident hypertension was defined as a new clinical diagnosis, initiation of antihypertensive treatment, or two consecutive blood pressure ≥140/90 mmHg. Participants with previously diagnosed hypertension were excluded. Baseline was defined as ART initiation. In intention‐to‐treat analysis, participants were followed from ART start until incident hypertension, death, loss to follow‐up or administrative censoring (30 November 2024). Death was treated as a competing event. We obtained cumulative incidence and estimated unadjusted and adjusted sub‐distribution hazard ratios (SHR) using Fine–Gray competing risk models. The primary exposure was initial ART class (NNRTI, PI or INSTI). Additional analyses evaluated tenofovir backbone and sub‐analysis on PWH starting second‐generation INSTI regimens.

**Results**: Overall, 3398 PWH were included; median age was 35.8 years (IQR 29.1–43.6), 86.9% were male, and the most frequent initial ART regimen class was INSTI‐based (65.0%), followed by NNRTI‐based (20.3%) and PI‐based regimens (14.7%). During follow‐up (median 84.4 months, IQR 45.3–121.9), 763 participants (22.5%) developed hypertension and 63 (1.9%) died before hypertension onset. The overall incidence rate of hypertension was 3.09 per 100 person‐years (95% CI: 2.88–3.32). The cumulative incidence of hypertension increased progressively from 5.24% at 12 months to 18.12% at 60 months and 25.04% at 120 months. At 120 months, cumulative incidence was 25.19% for those starting treatment with NNRTI‐, 26.77% for PI‐ and 23.71% for INSTI‐based regimens (Figure 1A). In competing‐risk regression, ART class was not associated with incident hypertension in unadjusted or adjusted analyses (Table 1). Tenofovir backbone was not associated with hypertension risk in univariable analysis (TAF SHR 1.00, 95% CI: 0.82–1.22; TDF 1.22, 95% CI: 0.95–1.33). Among people receiving second‐generation INSTI‐based regimens, DTG/3TC showed lower cumulative incidence at 60 months than ABC/3TC/DTG, BIC/FTC/TAF and DTG/FTC/TXF (Figure 1B), but this association was not statistically significant after adjustment (SHR 0.80, 95% CI: 0.54–1.21).

**Conclusions**: Initial ART class was not independently associated with incident hypertension. The lower observed cumulative incidence among people receiving DTG/3TC deserves further investigation.

**FIGURE 1:**
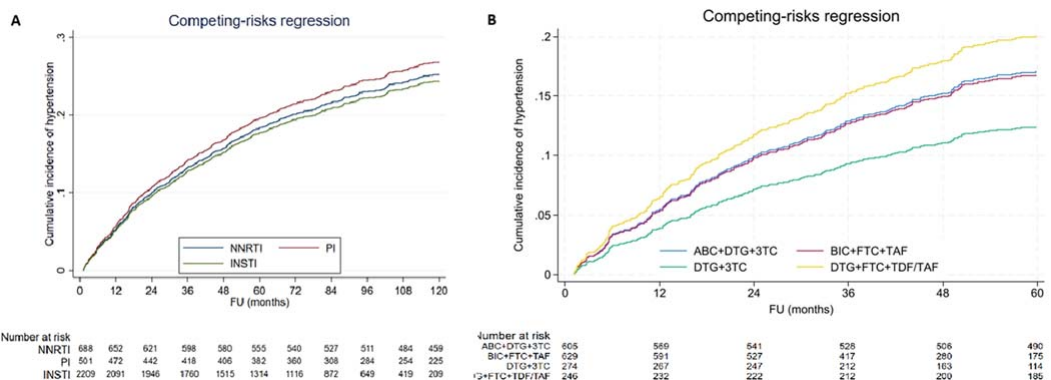
P431 | (A) Cumulative incidence of hypertension by initial ART regimen class; (B) cumulative incidence of hypertension among people starting second‐generation INSTIs.

**TABLE 1:**
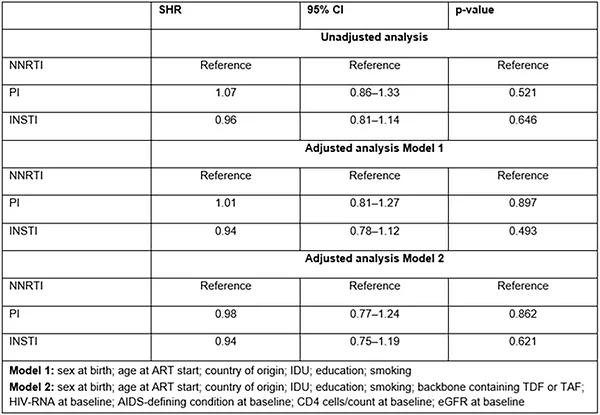
P431 | Competing‐risk regression for incident hypertension according to initial ART regimen class.

### P432 Peripheral and Non‐Compressible Arterial Diseases in HIV Patients on Dolutegravir Versus Atazanavir‐Based Regimens: Findings in a Multi‐Centre Nigerian Study

Roland Ijimdiya Stephen ^1^, Jimmy Reyes^2^, Li Ting Tan^3^

^1^Public Health, Modibbo Adama University Teaching Hospital, Yola, Nigeria; ^2^Department of Nursing, Samuel Merritt University, Oakland, CA, USA; ^3^Department of General Surgery, Johns Hopkins Hospital, Baltimore, MD, USA

**Background**: People living with HIV (PLWH) have an elevated risk of cardiovascular disease, including peripheral arterial disease (PAD), driven by chronic inflammation, immune activation and antiretroviral therapy–related metabolic effects. Integrase strand transfer inhibitors, particularly dolutegravir (DTG), have been linked to weight gain and metabolic disturbances, yet their vascular implications in sub‐Saharan Africa remain poorly characterized. This study compared the burden of PAD, non‐compressible arterial disease (NCAD) and cardiometabolic profiles among PLWH receiving DTG‐based therapy versus atazanavir/ritonavir‐based protease inhibitor therapy (ATV/r).

**Methods**: We conducted a multicentre comparative cross‐sectional study among 370 adults with HIV attending two tertiary hospitals in northeastern Nigeria between February and October 2024. Participants had received either dolutegravir‐based (*n* = 292) or atazanavir/ritonavir‐based (*n* = 78) antiretroviral therapy for ≥6 months. Sociodemographic, clinical, anthropometric, laboratory and vascular data were collected. Peripheral arterial disease (PAD) and non‐compressible arterial disease (NCAD) were assessed using the ankle–brachial index (ABI). Multivariable logistic regression was used to evaluate the association between antiretroviral regimen and PAD/NCAD after adjusting for relevant covariates

**Results**: Among 370 participants (DTG: *n* = 292; ATV/r: *n* = 78; mean age 46.5 ± 11.4 years; 74.3% female), PAD prevalence was 2.4%, with no significant difference between regimens. NCAD was common, affecting 31.6% of participants. After adjustment, antiretroviral regimen was not associated with PAD (adjusted odds ratio [aOR] 0.60; 95% CI: 0.07–5.00) or NCAD (aOR 1.00; 95% CI: 0.50–1.99). Central obesity was significantly more prevalent among participants receiving DTG‐based therapy, whereas absolute weight again per individual was greater among those on ATV/r. NCAD showed a strong association with history of stroke. Female gender confers protection against NCAD. Nomogram analyses further identified cumulative antiretroviral therapy exposure and cardiometabolic comorbidities as the principal drivers of predicted PAD and NCAD risk.

**Conclusions**: In this virologically suppressed cohort, PAD prevalence was low and did not differ by antiretroviral regimen. However, the high burden of NCAD and central adiposity highlights emerging cardiometabolic concerns in the integrase inhibitor era. Routine ABI screening and comprehensive cardiometabolic monitoring should be integrated into HIV care to improve early detection of vascular disease in sub‐Saharan Africa.

**Keywords**: peripheral arterial disease, subclinical atherosclerosis, people with HIV, dolutegravir, protease inhibitors, cardiovascular risk, antiretroviral therapy

### P433 Modifying Cardiovascular‐Kidney‐Metabolic Syndrome Trajectory in HIV: Early Evidence for GLP‐1 Receptor Agonists

Alessandro Pecchi^1^, Marianna Menozzi^2^, Michela Belli^2^, Cristina Mussini^2,3^, Giovanni Guaraldi^2,3^, Jovana Milić ^2,3^

^1^School of Medicine, University of Modena and Reggio Emilia, Modena, Italy, Modena, Italy; ^2^Modena HIV Metabolic Clinic, University of Modena and Reggio Emilia, Modena, Italy, Modena, Italy; ^3^Department of Surgical, Medical, Dental and Morphological Sciences, University of Modena and Reggio Emilia, Italy, Modena, Italy

**Background**: People with HIV (PWH) face a growing burden of cardiovascular‐kidney‐metabolic syndrome (CKM) [1]. Glucagon‐like peptide‐1 receptor agonists (GLP‐1 RAs) have emerged as a promising option to address CKM. The objective of this study was to assess changes in body weight and fat, longitudinal CKM trajectory through stage transitions and to evaluate improvements within stages with a purpose‐built continuous CKM Z‐score.

**Methods**: This longitudinal observational study enrolled PWH attending the Modena HIV Metabolic Clinic, Italy, in period 2023–2026 with ≥2 evaluations. The exposed group received at least one GLP‐1 RA prescription. Baseline was the closest visit within 12 months of treatment initiation (9 months before to 3 months after) and follow up last available visit. GLP‐1 RA and semaglutide users and non‐users were propensity score matched 1:3 by age, sex and baseline CKM stage separately. CKM Z‐score was developed as a continuous score including: eGFR, waist circumference, waist‐to‐height ratio, BMI, VAT, liver steatosis, glucose, HOMA‐IR, HbA1c and triglycerides. Higher scores reflected better overall CKM health. Treatment effects over time were assessed using linear mixed‐effects models.

**Results**: 27 GLP‐1 RA users were compared with 111 non‐users, and 14 SC semaglutide users with 54 non‐users; median follow‐up 13 months, median age ∼60 years across groups. Semaglutide users greater reductions in BMI, waist circumference, visceral and hepatic fat (Table 1 and Figure 1, Panels A, B). They also gained significantly more on the CKM Z‐score than controls (Δ +2.90 vs. +0.80; Figure 1, Panel D), whereas the change was comparable in the overall GLP‐1 cohort (Panel C). CKM stage transitions trended towards slowed progression without reaching significance (Table 1). In the mixed‐effects models, both showed a significant CKM Z‐score increase over time (GLP‐1: 1.51, *p* < 0.001; semaglutide: 0.92, *p* = 0.045), but the time×treatment interaction was significant only for semaglutide SC (β = 2.14, *p* = 0.032; GLP‐1: −0.73, *p* = 0.411), indicating a treatment‐driven gain only with semaglutide.

**Conclusions**: GLP‐1 RA therapy, most consistently SC semaglutide, was associated with a more favourable CKM trajectory than non‐users, with slowed stage progression and within‐stage improvement on the continuous CKM Z‐score, alongside reductions in adiposity, visceral and hepatic fat. This single‐centre observational proof‐of‐concept is hypothesis generating to test whether GLP‐1 RAs can modify CKM trajectory in PLWH.

**Reference**

1. Milic J. Prevalence and Long‐Term Transitions of Cardiovascular‐Kidney‐Metabolic Syndrome Stages in PWH, abstract no. 734, CROI conference, 22–25 February 2026, Denver, CO, USA, https://www.croiconference.org/abstract/1851‐2026/

**FIGURE 1:**
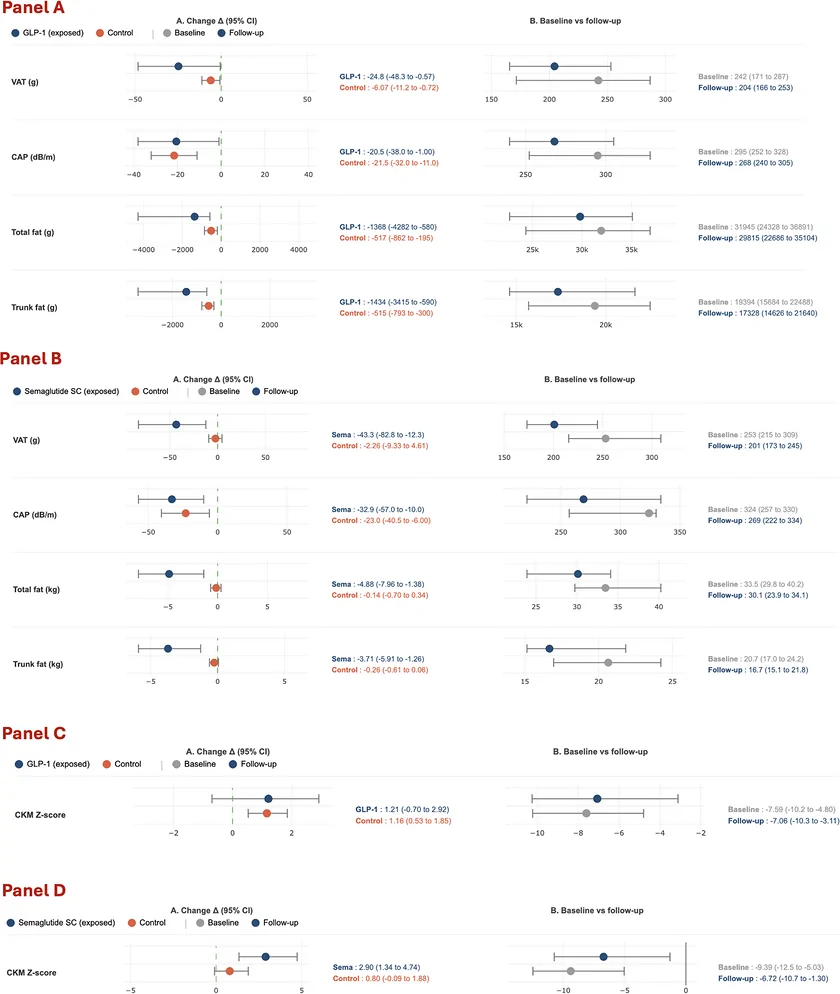
P433 | Changes in fat‐mass parameters (VAT, CAP, total and trunk fat) and the CKM Z‐score across the two cohorts (GLP‐1 RA users vs. non‐users and in semaglutide SC users vs. non‐users). Panel A: fat mass, GLP‐1 RA cohort; Panel B: fat mass, semaglutide SC cohort; Panel C: CKM Z‐score, GLP‐1 RA cohort; Panel D: CKM Z‐score, semaglutide SC cohort. CAP = controlled attenuation parameter; CKM = cardiovascular‐kidney‐metabolic; VAT = visceral adipose tissue.

**TABLE 1:**
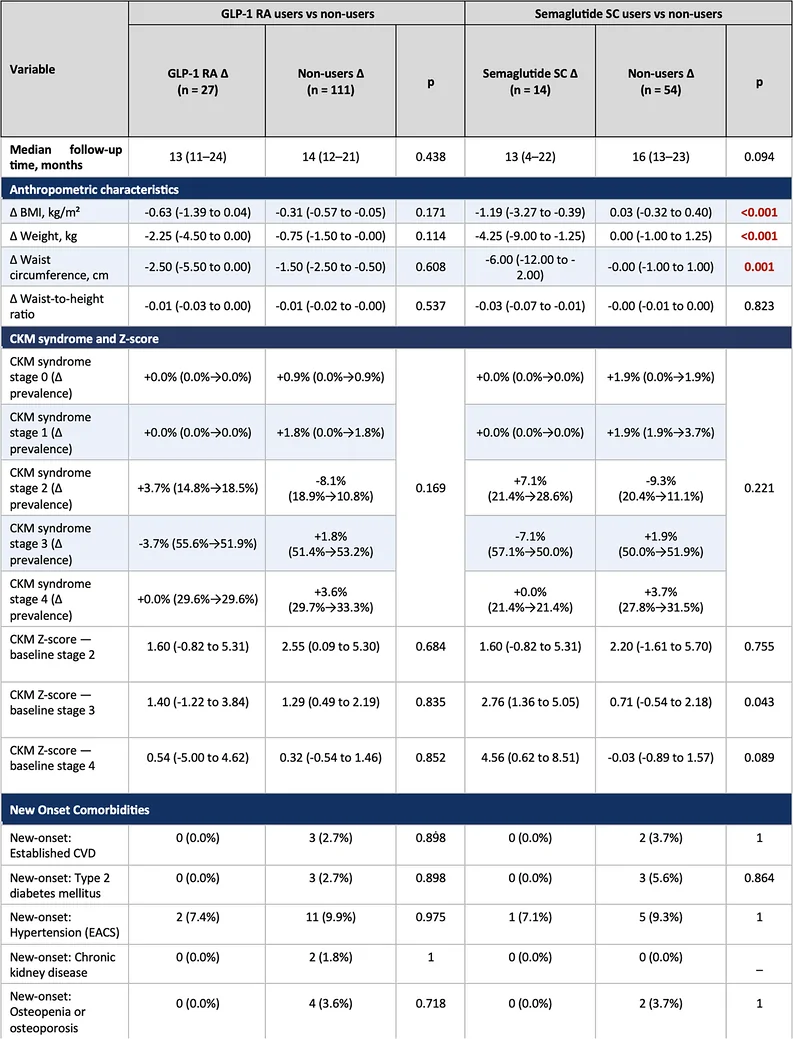
P433 | Changes from baseline to follow‐up in anthropometric measures, CKM syndrome stage distribution, stage‐stratified CKM Z‐score and new‐onset comorbidities, in GLP‐1 RA users versus non‐users and semaglutide SC users versus non‐users. Continuous variables are shown as median change (IQR) and categorical variables as *n* (%); *p* values refer to the between‐group comparison of the change. CKM = cardiovascular‐kidney‐metabolic; CVD = cardiovascular disease; EACS = European AIDS Clinical Society.

### P434 Efficacy and Metabolic Outcomes of DOR/TDF/3TC Versus BIC/TAF/FTC in Overweight, Treatment‐Naïve People Living With HIV: 24‐Week Results From the COATL Randomized Trial

Ana Luz Cano Díaz ^1^, Mitzi Daniela Ramírez Ramírez^2^, Paola Edith Padilla Noguera^2^, José Antonio Mata Marín^1^, Gloria Elizabeth Salinas Velázquez^3^, Emily Itzel Pecero García^1^, Alberto Chaparro Sánchez^1^, Ericka Nelly Pompa Mera^1^, Jesús Enrique Gaytán Martínez^1^

^1^Infectious Diseases Department, CMN La Raza, Mexico City, Mexico; ^2^Medical School, Universidad Nacional Autónoma de México, Mexico City, Mexico; ^3^Department of Radiology, CMN La Raza, Mexico City, Mexico

**Background**: To determine the efficacy and metabolic changes in people living with HIV (PLWH) and overweight with no prior experience to antiretroviral treatment (ART) randomized to DOR/TDF/3TC or BIC/TAF/FTC at 24 weeks of follow up.

**Methods**: Open‐label, randomized clinical trial was conducted at the Hospital de Infectología, “La Raza” National Medical Center in Mexico City, and included ART‐naïve people living with HIV (PLWH) with body mass index (BMI) ≥25 kg/m^2^ at the expense of body fat (> 20% in men and > 30% in women). Virologic, immunologic, anthropometric and metabolic parameters were evaluated at 24 weeks.

**Results**: This preliminary analysis included 60 PLWH, 57 (95%) men and 3 women (5%), median age 30.5 years (IQR 26.0‒36.7), baseline mean weight 85.6 kg (±12.1), and median BMI 28.3 kg/m^2^, body fat and muscle mass mean were 28.1% (±4.6) and 58.0 kg (±8.2), respectively. Participants were randomly assigned, 28 to DOR/TDF/3TC and 32 to BIC/TAF/FTC; after 24 weeks, mean body weight was 85.4 kg (±14.6) in DOR and 87.6 kg (±17.1) in BIC, *p* = 0.591; median BMI was 27.9 kg/m^2^ (IQR 26.1‒30.4) in DOR and 29.2 kg/m^2^ (IQR 28.0‒32.0) in BIC, *p* = 0.112; mean body fat and muscle mass were 27.9% (±5.4) and 57.8 kg (±7.5) in DOR, compared to BIC with 28.7% (±5.0) and 60.4 kg (±8.7), with no significant differences (*p* = 0.549 and *p* = 0.237, respectively). When the weight change was analysed at 24 weeks, a significant increase was observed in the BIC group, with a median gain of +2.8 kg (IQR +0.70–+5.1) compared to DOR with median +0.20 kg (−1.3‒+2.9), *p* = 0.016. Regarding the primary outcome, RNA HIV‐1 < 50 copies/mL was achieved in 27/28 (96.4%) in DOR and 31/32 (96.9%) in BIC, *p* = 1.000; just 1 participant in the BIC group had viral load ≥50 copies/mL. Mean creatinine was greater in BIC with 1.02 mg/dL (±0.12) compared to 0.88 mg/dL (±0.11) in DOR, *p* < 0.001 (Figure 1 and Table 1).

**Conclusions**: In the COATL trial, both regimens achieved high virologic efficacy at 24 weeks. While final absolute weights were similar, the BIC/TAF/FTC group experienced a significantly higher median weight gain compared to DOR/TDF/3TC among overweight or obese PLWH.

**FIGURE 1:**
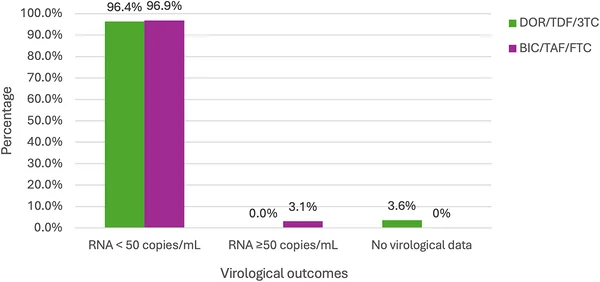
P434 | Efficacy of DOR/TDF/3TC versus BIC/TAF/FTC in overweight/obese PLWH without prior ART experience.

**TABLE 1:**
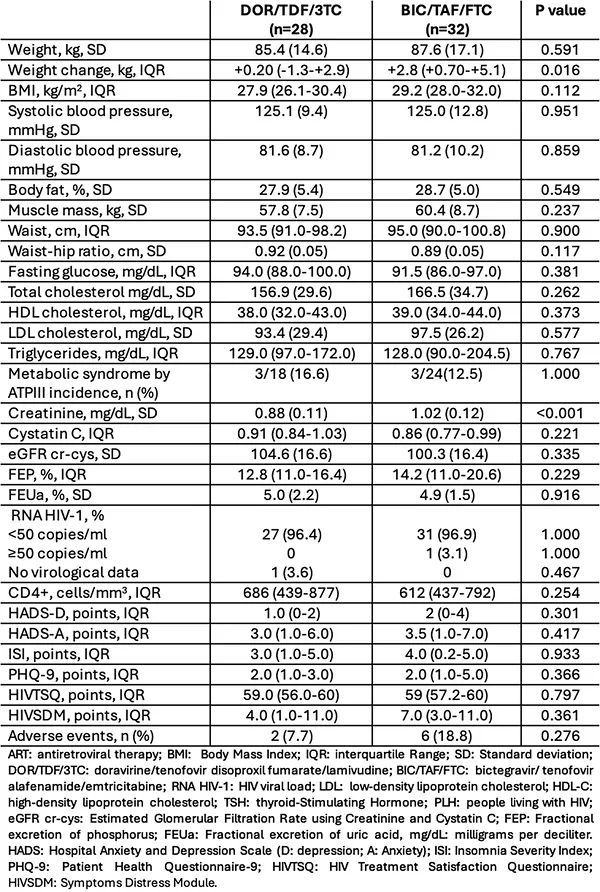
P434 | Clinical and biochemical characteristics in PLWH and overweight at 24 weeks after start of ART: DOR/TDF/3TC versus BIC/TAF/FTC.

### P435 Efficacy, Safety and Tolerability of Switching to Dolutegravir/Lamivudine Versus Continuing INSTI‐Based Triple Therapy in Virologically Suppressed People Living With HIV: 24‐Week Results From the Randomized TLACAELEL Trial

Diego Armando Arteaga Badillo^1^, José Antonio Mata Marín^1^, Ana Luz Cano Díaz ^1^, Gloria Elizabeth Salinas Velázquez^2^, Emily Itzel Pecero García^3^, Jorge Fabian Pimentel González^4^, Alberto Chaparro Sánchez^1^, Ericka Nelly Pompa Mera^1^, Sergio Santillán Arroyo^1^, Jesús Enrique Gaytán Martínez^1^

^1^Infectious Diseases Department, CMN La Raza, Mexico City, Mexico; ^2^Department of Radiology, CMN La Raza, Mexico City, Mexico; ^3^Infectious Diseases Department, Mexico City, Mexico; ^4^HIV Clinic, HGZ 27, Mexico City, Mexico

**Background**: Long‐term triple‐drug regimens cause cumulative toxicities. Simplification to dual therapy dolutegravir/lamivudine (DTG/3TC) aims to optimize safety while preserving efficacy and genetic barrier. This study evaluated the efficacy, safety and tolerability of switching to DTG/3TC versus continuing INSTI‐based triple therapy (TT) in virologically suppressed people living with HIV (PWH) in Mexico City.

**Methods**: This phase 4, open‐label, randomized, single‐centre clinical trial (NCT07138144) was conducted at the Hospital de Infectología, “La Raza” National Medical Center in Mexico City and included ART‐naïve men living with HIV (MLH) virologically suppressed (<50 copies/mL) for ≥48 weeks without prior failure and with eGFR ≥60 mL/min. Patients were randomized 2:1 to switch to dual therapy (DT) or maintain baseline TT. Longitudinal 24‐week outcomes evaluated anthropometric parameters, body composition (muscle, body fat, body water), metabolic, renal and virological markers.

**Results**: Preliminary analysis included 71 PWH (46 in DT and 25 in TT). Baseline median age was 29 years (IQR 27–36), median weight 66.3 kg (IQR 59.3–74.0), and mean CD4+ count 741 (SD ±268) cells/mm^3^. Active smoking was reported in 20% of TT versus 39.1% of DT participants (*p* = 0.122). At 24 weeks, virological suppression was maintained in 24 participants (96%) in TT group and 41 participants (89.1%) in DT group, *p* = 0.414; 1 (2.1%) in DT patient exhibiting ≥50 copies/mL. Adverse events were reported grade I by DAIDS in 1 (4%) participant in TT and 5 (10.9%) in DT, *p* = 0.414; grade II was reported in just 1 participant (2.2%) in DT. No participant experienced grade III or IV events. Both cohorts experienced increases in weight (+1.92 kg TT, *p* = 0.003; +1.95 kg DT, *p* < 0.001); BMI (+0.6 kg/m^2^ TT, *p* = 0.016; +0.57 kg/m^2^; *p* = 0.006); and body fat (+1.8% TT, *p* = 0.011; +1.0% DT, *p* = 0.007). The DT group experienced a significant intra‐group CD4+ increase (+57 cells/mm^3^; *p* = 0.034). At week 24, mean total cholesterol was higher in DTG/3TC group than in INSTI group (177 ± 36 mg/dL vs. 160 ± 23 mg/dL; *p* = 0.047) (Figure 1 and Table 1).

**Conclusions**: Switching to DTG/3TC maintains virological suppression with no differences in virological efficacy and favourable safety profile at 24 weeks, supporting its use as a simplification strategy that reduces cumulative drug exposure while maintaining clinical stability.

**FIGURE 1:**
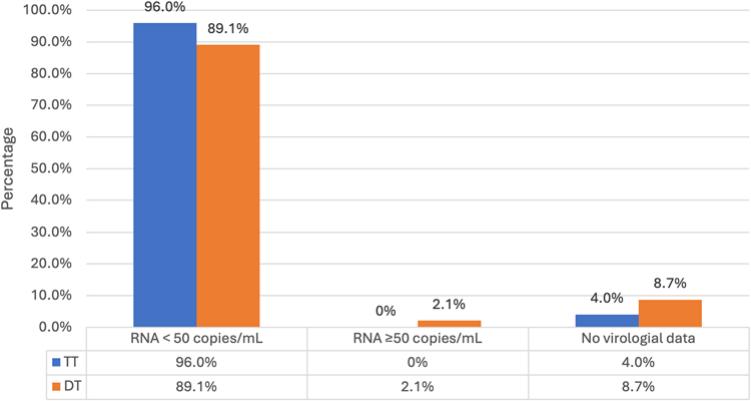
P435 | Efficacy DT compared to TT at 24 weeks.

**TABLE 1:**
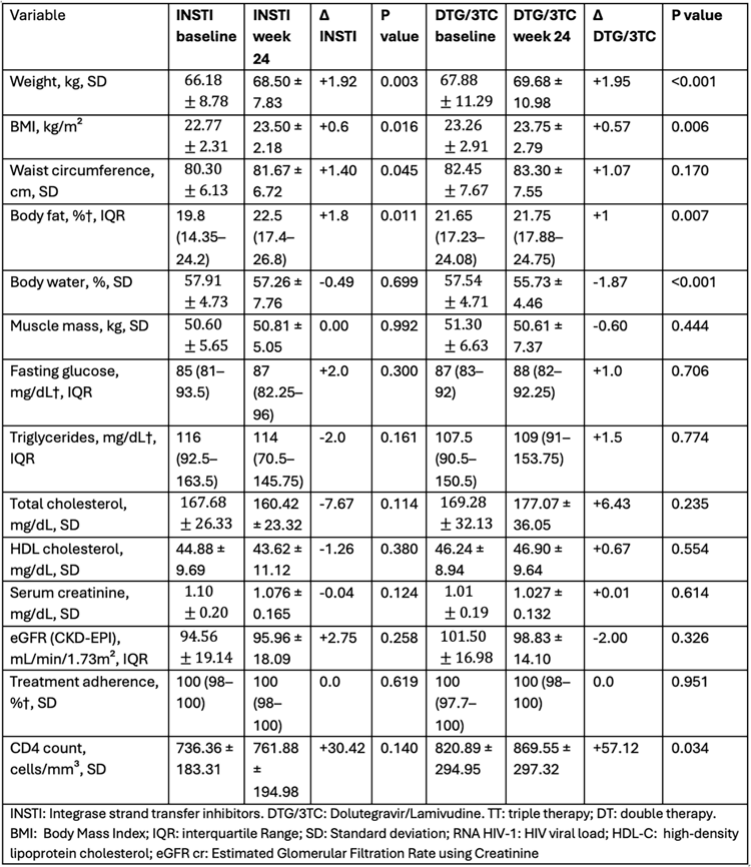
P435 | Intragroup differences INSTI versus DTG/3TC from baseline to 24 weeks.

### P436 Abstract Withdrawn

### P437 Abstract Withdrawn

### P438 The Influence of the T Allele and CT+TT Genotypes of rs2057482 on the Presence of Hypertension in People Living With HIV

Nemanja Đorđević^1,2^, Jovan Ranin^3,4^, Ivana Gmizić^3,4^, Marko Marković^3,4^, Ivan Rajković^3,4^, Jovana Ranin^3,4^, Biljana Ljujić^5^, Sofija Sekulić Marković^1,2^, Ivana Lešnjak^1,2^, Biljana Popovska Jovičić ^1,2^

^1^Department of Infectious Diseases, Faculty of Medical Sciences, University of Kragujevac, Kragujevac, Serbia; ^2^Clinic for Infectious Diseases, University Clinical Centre Kragujevac, Kragujevac, Serbia; ^3^Department of Infectious Diseases, Faculty of Medicine, University of Belgrade, Belgrade, Serbia; ^4^Clinic for Infectious and Tropical Diseases, University Clinical Centre of Serbia, Belgrade, Serbia; ^5^Department of Genetics, Faculty of Medical Sciences, University of Kragujevac, Kragujevac, Serbia, Kragujevac, Serbia

**Background**: Metabolic syndrome (MS) is common among people living with HIV (PLWH), with a recent global meta‐analysis reporting a combined prevalence of approximately 25.3%. Given the central role of HIF‐1α in regulating cellular adaptation to hypoxia, metabolic switching towards glycolysis, and the integration of glucose and lipid metabolic pathways, we analysed the association of the HIF‐1α rs2057482 polymorphism with metabolic syndrome and hypertension in PLWH undergoing antiretroviral therapy (ART).

**Methods**: This multicentre, retrospective, cross‐sectional, case−control study included 121 PLWH. Participants were classified as MS cases or controls according to the NCEP ATP III criteria. Genotyping of the HIF‐1α rs2057482 polymorphism was performed using real‐time PCR with a TaqMan assay. Clinical and metabolic parameters were analysed. Multivariable logistic regression was used to identify independent factors associated with MS.

**Results**: MS was identified in 47 participants (38.8%), while 74 (61.2%) served as controls. The minor T allele frequency of HIF‐1α rs2057482 was 20.7%. The frequency of the variant T allele was similar between the study groups, with no significant difference in risk compared to the reference C allele (OR 1.063; 95% CI: 0.563–2.008; *p* = 0.851). No statistically significant association was found between the alleles and genotype groups of the HIF‐1α rs2057482 gene polymorphism and the presence of metabolic syndrome in PLWH treated with ART. Carriers of the variant allele (CT+TT) of the HIF‐1α rs2057482 polymorphism had a significantly higher odds of having arterial hypertension compared to carriers of the reference CC genotype in the control group of participants living with HIV infection (OR 8.467; 95% CI: 1.600–44.700; *p* = 0.007), whereas such an association was not found in the group of participants with MS (OR 0.416; 95% CI: 0.120–1.445; *p* = 0.227) (Figure 1).

**Conclusions**: Our results indicate the importance of examining the association of the HIF‐1α genotype, specifically its polymorphism rs2057482, with the development of hypertension as a risk factor for cardiovascular comorbidities in PLWH.

**FIGURE 1:**
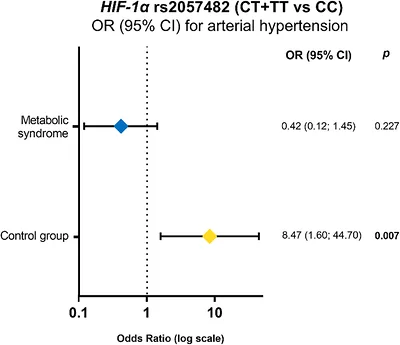
P438 | Graphical comparative presentation of the association of HIF‐1α rs2057482 polymorphism genotypes with arterial hypertension among study groups of PLWH. The results are presented as odds ratios (OR) on a logarithmic scale, represented by diamonds of different colours relative to the study group. The black horizontal lines represent the 95% confidence interval values. The black vertical dashed line indicates OR = 1 (line with no effect).

### P439 Dynamic Changes in Lipidome and Cardiovascular Risk Factors After a Stable Switch to B/F/TAF in People With HIV

Biao Zhu, Zhikai Wan

The Department of Infectious Diseases, State Key Laboratory for Diagnosis and Treatment of Infectious Diseases, National Clinical Research Center for Infectious Diseases, Collaborative Innovation Center for Diagnosis and Treatment of Infectious Diseases, The First Affiliated Hospital, School of Medicine, Zhejiang University, Hangzhou, China

**Background**: HIV‐infected patients undergoing long‐term antiretroviral therapy (ART) frequently develop lipid metabolism disturbances, which significantly increase the risk of cardiovascular disease (CVD). E/C/F/TAF and B/F/TAF are widely used first‐line ART regimens, yet the dynamic changes in lipid profiles and their correlations with cardiovascular risk factors after conversion from E/C/F/TAF to B/F/TAF remain to be fully elucidated.

**Methods**: Fifty HIV‐infected patients with stable E/C/F/TAF treatment were enrolled, and 44 completed at least 6 months of follow‐up, including 22 switched to B/F/TAF and 22 continuing E/C/F/TAF. Untargeted lipidomics was applied to identify plasma lipid molecules. Time‐series analysis and correlation analysis were used to assess lipid dynamics and associations with Framingham general cardiovascular risk score (FRS) and clinical metabolic parameters.

**Results**: A total of 1093 lipid molecules were identified. Time‐series analysis showed that 526 lipids in 7 clusters changed significantly in the E/C/F/TAF group, mainly characterized by increased triacylglycerol (TAG) and lysophosphatidylcholine (LPC) and decreased phosphatidylcholine (PC) and sphingomyelin (SM). In the B/F/TAF group, 553 lipids in 4 clusters altered markedly, dominated by elevated PC and SM and reduced diacylglycerol (DG) and TAG. Forty‐two significantly altered lipids were closely related to clinical biochemical parameters. Elevated DG in the E/C/F/TAF group was positively correlated with fasting blood glucose, TAG and very low‐density lipoprotein cholesterol (VLDL‐C), and negatively correlated with high‐density lipoprotein cholesterol (HDL‐C). In contrast, increased ceramide (Cer), LPC, SM and sphingosine (SO) in the B/F/TAF group were negatively associated with fasting blood glucose, TAG, VLDL‐C and low‐density lipoprotein cholesterol (LDL‐C), and positively correlated with total bilirubin. After adjusting for age, gender, BMI, total cholesterol (TC) and HDL‐C, DG (16:0/18:2), DG (18:3/18:2), DG (20:5/18:2) and LPC (22:6) remained independently positively correlated with FRS.

**Conclusions**: Switching from E/C/F/TAF to B/F/TAF favourably remodels plasma lipid metabolism and reduces pro‐atherogenic lipid levels. Specific DG and LPC lipids can serve as independent biomarkers for evaluating cardiovascular risk in HIV patients during ART regimen adjustment.

### P440 Impact of Antiretroviral Class Switching on Cardiometabolic Risk: Data From the HIV‐HEART‐Aging Cohort

Christoph Zacher^1^, Laven Mavarani^2^, Anja Stempel^3^, Anja Potthoff^4^, Martin Hower^5^, Stefanie Sammet^3^, Felix Maischack^3^, Börge Schmidt^2^, Stefan Esser ^3^, on behalf of HIV‐HEART Aging Study Group

^1^Department of Infectious Diseases, West German Center of Infectious Diseases University Hospital Essen, University Duisburg‐Essen, Essen, Germany; ^2^Institute for Medical Informatics, Biometry and Epidemiology (IMIBE), University Hospital Essen, University Duisburg‐Essen, Essen, Germany; ^3^Department of Dermatology and Venereology, HIV Outpatient Clinic, University Hospital Essen, University Duisburg‐Essen, Essen, Germany; ^4^Interdisciplinary Immunological Outpatient Clinic, Center for Sexual Health and Medicine, Department of Dermatology, Venereology and Allergology, Ruhr University Bochum, Bochum, Germany; ^5^Department of Pneumology, Infectious Diseases and Internal Medicine, Klinikum Dortmund, Hospital University Witten/Herdecke, Dortmund, Germany

**Background**: Ageing people with HIV (PWH) have an increased cardiometabolic risk (CMR), which may be influenced by antiretroviral therapy (ART). The impact of switching ART drug classes on CMR in real‐world settings remains unclear.

**Methods**: The HIV‐HEART‐Aging cohort is a prospective study of cardiovascular and metabolic risk factors in PWH in the Ruhr area since 2004. Participants attending both the 15‐year (FU15; 2020–2022) and 17.5‐year (FU17) follow‐ups were included if they had been on a stable ART drug class for ≥6 months prior to FU15. CMR in PWH with stable ART drug class within 2.5 years were compared to PWH who switched drug class during this interval (Figure 1 and Table 1).

**Results**: A total of 1177 participants from FU15 were included between 2020 and 2022; 999 (84.9%) remained on the same ART class and 178 (15.1%) switched within the observation period. At FU15, switchers had a lower mean BMI compared with the stable group (25.8 ± 5 vs. 26.7 ± 4.8 kg/m^2^), with a lower prevalence of overweight/obesity (51.1% vs. 59%). These differences remained stable over 2.5 years. Cardiometabolic markers differed modestly between groups. Hyperlipidaemia prevalence was similar at FU15, but decreased among switchers and increased in the stable group at FU17 (54.5% vs. 58.8%). Among switchers, there was a marked shift in ART composition, with reductions in PI‐based (30.9% to 7.3%) and NNRTI‐based regimens (50.6% to 30.9%), and an increase in INSTI‐based regimens (25.3% to 87.1%) and a decrease in the use of NRTI backbones (88.2% to 81.5%).

**Conclusions**: In the HIV‐HEART‐Aging cohort, switching ART drug class did not show an adverse CMR profile. Changes in cardiometabolic markers were generally modest, suggesting that ART class switching to contemporary regimens—predominantly INSTI‐based—has limited impact on weight and metabolic outcomes in this cohort. Targeted therapies and lifestyle changes are more crucial for improving cardiometabolic risk than switching ART.

**FIGURE 1:**
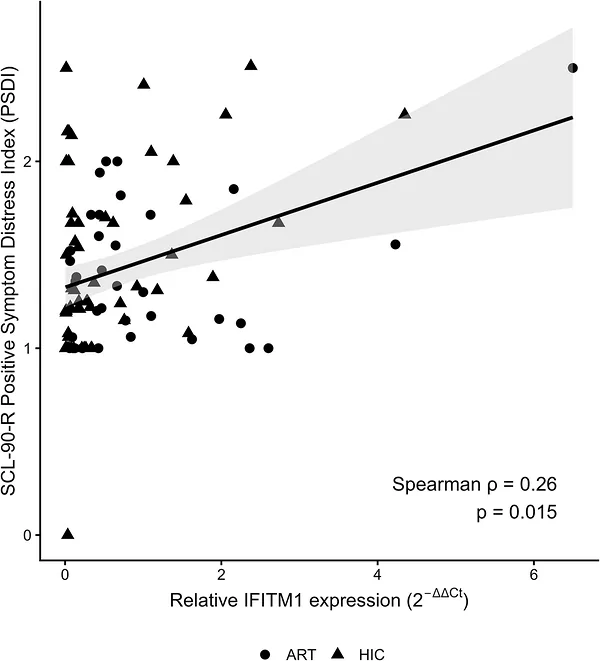
P440 | Schematic overview of the study design, showing the 1177 participants from the HIV‐Heart‐Aging cohort included in the analysis, divided into stable ART class and switched ART class group.

**TABLE 1:**
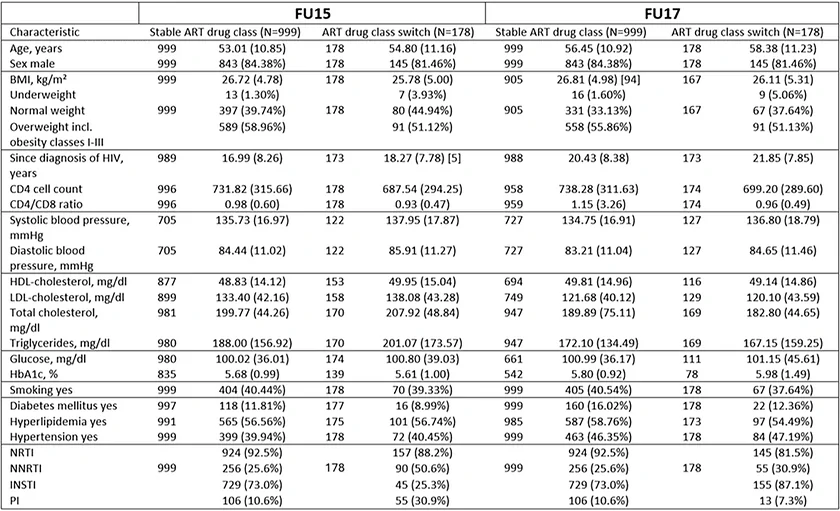
P440 | Characteristics of PWH from the HIV‐Heart‐Aging cohort at FU15 and FU17, grouped by stable ART class regimes and switched ART class regimes over 2.5 years. Continuous variables are shown as mean (standard deviation), while categoric variables are shown as frequency (percent). INSTI, integrase strand transfer inhibitors; NNRTI, non‐nucleoside reverse transcriptase inhibitors; NRTI, nucleoside/nucleotide reverse transcriptase inhibitors; PI, protease inhibitors.

### P441 Abstract Withdrawn

## Co‐Morbidities and Complications of Disease and/or Treatment: Malignancies (Non‐AIDS Defining)

### P442 More Than Doubled Risk of Virus‐Related Non‐AIDS‐Defining Cancers in People Living With Multidrug‐Resistant HIV: A Matched Multicohort Analysis With Data From the PRESTIGIO Registry

Tommaso Clemente ^1^, Nicolò Capra^1^, Letizia Oreni^2^, Riccardo Lolatto^1^, Giacomo Pozza^2^, Simona Di Giambenedetto^3,4^, Chiara Fornabaio^5^, Elisa Fronti^6^, Daniela Distefano^7^, Maddalena Matone^2^, Filippo Lagi^8^, Adriana Cervo^9^, Vincenzo Spagnuolo^1,7^, Andrea Giacomelli^2,10^, on behalf of PRESTIGIO Study Group

^1^Infectious Diseases, IRCCS San Raffaele Scientific Institute, Milan, Italy; ^2^Department of infectious diseases, ASST Fatebenefratelli Sacco, Milan, Italy; ^3^Infectious Diseases Unit, Fondazione Policlinico Universitario Agostino Gemelli IRCCS, Rome, Italy; ^4^Catholic University of the Sacred Heart, Rome, Italy; ^5^Infectious Diseases Unit, ASST Cremona, Cremona, Italy; ^6^Unit of Infectious Diseases, AO‐Universitaria, Parma, Italy; ^7^Vita‐Salute San Raffaele University, Milan, Italy; ^8^Department of Experimental and Clinical Medicine, University of Florence, Florence, Italy; ^9^Department of Infectious Diseases, Azienda Ospedaliero‐Universitaria di Modena, Policlinico di Modena, Modena, Italy; ^10^Department of Biomedical and Clinical Sciences, Università degli Studi di Milano, Milan, Italy

**Background**: HIV multidrug resistance is associated with a high disease burden [1], but data on cancer risk are limited [2]. We aimed to explore the risk of non‐AIDS‐defining cancers (NADCs) in people with 4‐class drug‐resistant (4DR) HIV.

**Methods**: Matched cohort study including adults on antiretroviral therapy (ART) without prior NADCs, classified as 4DR (PRESTIGIO registry) [3] and non‐4DR (ASST FbF Sacco and IRCCS San Raffaele). Groups were matched by age (±5 years), sex‐at‐birth and ART duration (±5 years) with a 1:3 ratio. Baseline was the date of first 4DR evidence for exposed participants and the nearest visit (±6 months) for unexposed participants. Follow‐up accrued until first NADC, last visit or death. The primary endpoint was the first NADC (excluding non‐melanoma skin cancers); the secondary was the first virus‐related NADC (Hodgkin lymphoma, anal, liver, vulvar, penile cancer and Merkel cell carcinoma). Cumulative incidence functions accounted for competing risks. Poisson regression modelled incidence rates (IRs), 95% confidence intervals (95% CIs) and incidence rate ratios (IRRs). Multivariable Fine–Gray models adjusted for smoking, HCV serostatus and CD4^+^ nadir. Analyses were repeated using multiple imputation to handle missing data.

**Results**: Overall, 236 4DR and 708 non‐4DR people included (Table 1). During a median follow‐up of 7.9 (interquartile range 3.9–11.2) years (7275 person‐years [PY]), 28/236 (11.9%) 4DR and 64/708 (9.0%) non‐4DR individuals developed a NADC (4DR: IR 1.42 [95% CI: 0.94–2.06]/100 PY; non‐4DR: IR 1.21 [95% CI: 0.93–1.54]/100 PY; IRR 1.18 [95% CI: 0.76–1.84], *p* = 0.466). No association was observed between 4DR status and overall NADC risk (adjusted sub‐distribution hazard ratio [sHR_adj_] 1.15 [0.68–1.95], *p* = 0.593; Figure 1). Restricting to virus‐related NADCs, 16/236 (6.8%) 4DR and 21/708 (3.0%) non‐4DR people developed the event (4DR: IR 0.81 [95% CI: 0.46–1.32]/100 PY; non‐4DR: IR 0.40 [95% CI: 0.24–0.60]/100 PY; IRR 2.05 [1.07–3.94] *p* = 0.027). Individuals with 4DR HIV had a more than doubled risk of virus‐related NADCs (sHR_adj_ 2.60 [1.20–5.63], *p* = 0.015; Figure 1). The distribution of first virus‐related NADC differed significantly by 4DR status (*p* = 0.036): anal cancer predominated among 4DR people, hepatocellular carcinoma among non‐4DR. Results were consistent after multiple imputation.

**Conclusions**: Among NADCs, 4DR status was associated with a more than doubled risk of virus‐related malignancies, particularly anal cancer. These findings support enhanced prevention and targeted screening strategies in this population.

**References**

1. Galli L, Parisi MR, Poli A, et al. Burden of disease in PWH harboring a multidrug‐resistant virus: Data from the PRESTIGIO Registry. Open Forum Infect Dis. 2020;7(11):ofaa456.

2. Clemente T, Pontillo D, Malagnino V, et al. Cancer in people with multidrug‐resistant HIV. AIDS. 2024;38(12):1801–1806.

3. Clemente T, Galli L, Lolatto R, et al. Cohort profile: PRESTIGIO, an Italian prospective registry‐based cohort of people with HIV‐1 resistant to reverse transcriptase, protease and integrase inhibitors. BMJ Open. 2024;14(2):e080606.

**FIGURE 1:**
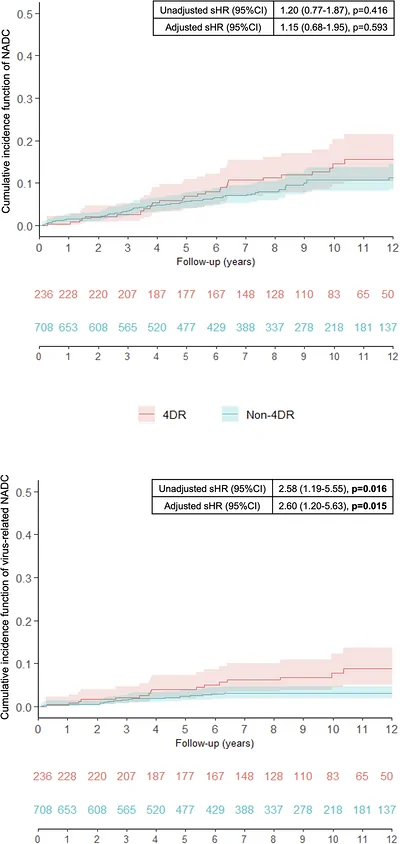
P442 | Cumulative incidence function and risk of non‐AIDS‐defining cancer.

**TABLE 1:**
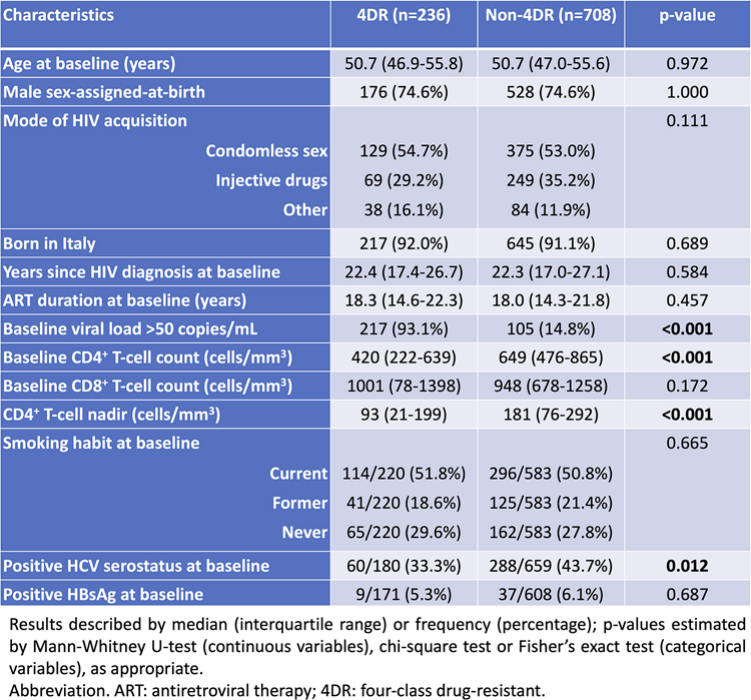
P442 | Characteristics of people with HIV included in the analysis.

### P443 Limited Impact of Late HPV Vaccination on Anal Cytological Outcomes in an Ageing Cohort of People Living With HIV

Davide Leoni ^1^, Elena Agostini^1^, Angelica Buffa^1^, Lolita Sasset^1^, Samuele Gardin^1^, Francesco Vladimiro Segala^1^, Eleonora Vania^1^, Anna Ferrari^1^, Mazzitelli Maria^2^, Cesare Ruffolo^3^, Gaya Spolverato^3^, Annamaria Cattelan^1^

^1^Infectious Diseases Unit, Padua University Hospital, Padua, Italy; ^2^Infectious Diseases Unit, IRCCS Sacro Cuore, Rome, Italy; ^3^General Surgery 3, Padua University Hospital, Padua, Italy

**Background**: Human papillomavirus (HPV) infection remains highly prevalent in people living with HIV (PLWH), driving the burden of anal dysplasia and cancer. While HPV vaccination is a proven preventive strategy, its clinical effectiveness, when administered to older adults beyond guideline‐recommended ages, remains uncertain. This study evaluates the impact of late HPV immunization within an ageing HIV cohort.

**Methods**: We retrospectively analysed adult PLWH undergoing anal HPV‐DNA screening and Pap testing at the Padua Infectious Diseases Unit between 2022 and 2025. Multivariate logistic regression models were used to identify factors associated with high‐risk HPV (HR‐HPV) carriage and anal cytological abnormalities.

**Results**: Overall (Figure 1), 272 PLWH were included (median age 52 years; 75.4% male; 61.8% MSM). Most participants were virologically suppressed (98.8%), with a median CD4+ count of 675 cells/mm^3^. HPV vaccination had been received by 107 individuals (40.5%) at a median age of 47 years. Anal HPV‐DNA positivity was 91.9%; 58.8% carried at least one HR‐HPV genotype and 15.1% carried HPV 16/18. Among 195 satisfactory anal Pap tests, 35.4% revealed cytological abnormalities (29.7% LSIL, 3.6% ASCUS, 2.1% HSIL). In multivariate analysis (Table 1), HPV vaccination was significantly associated with a reduction in HR‐HPV carriage (OR 0.57, 95% CI: 0.38–0.99, *p* = 0.04). Borderline associations suggested reduced HR‐HPV carriage in heterosexual men (OR 0.51, *p* = 0.09) and increased risk in individuals with prior AIDS‐defining illness (OR 1.85, *p* = 0.08). Cytological abnormalities were strongly predicted by current HR‐HPV positivity (multivariate OR 2.56, 95% CI: 1.27–5.15, *p* = 0.008) while Vaccination status showed no association with cytological outcomes (*p* = 0.54).

**Conclusions**: In this ageing HIV cohort, late HPV vaccination was associated with a reduced burden of HR‑HPV infection but did not translate into improved anal cytological outcomes. These findings highlight the partial and genotype‑level impact of late immunization and underscore its limited ability to modify established disease pathways in older adults. While vaccination may still offer virological benefit, it cannot replace systematic secondary screening, which remains essential for detecting and managing pre‑neoplastic anal lesions in PLWH.

**FIGURE 1:**
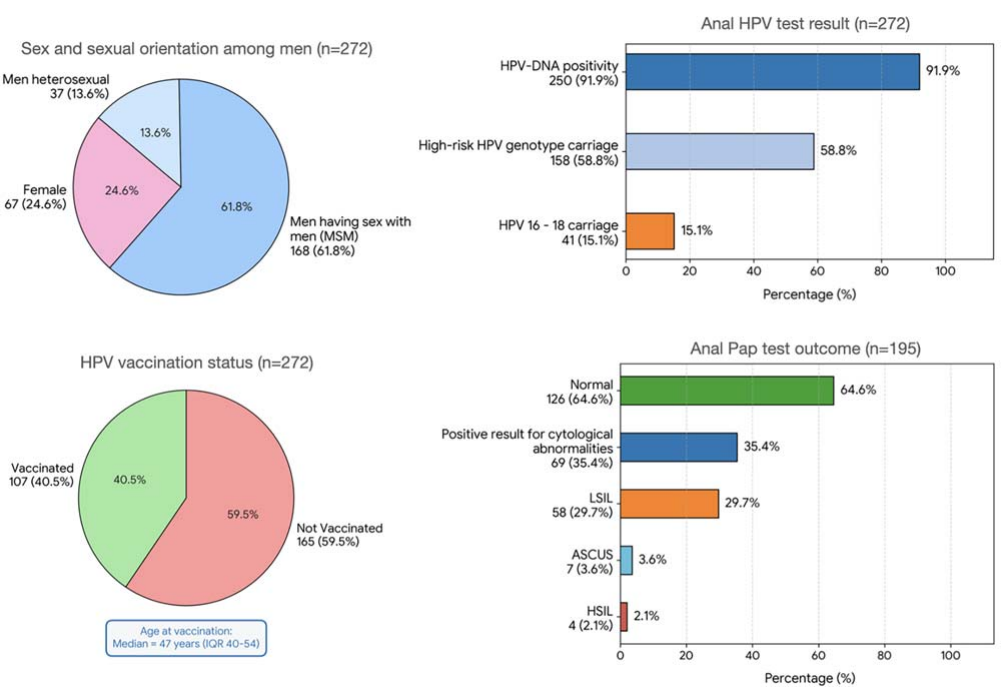
P443 | Study cohort characteristics.

**TABLE 1:**
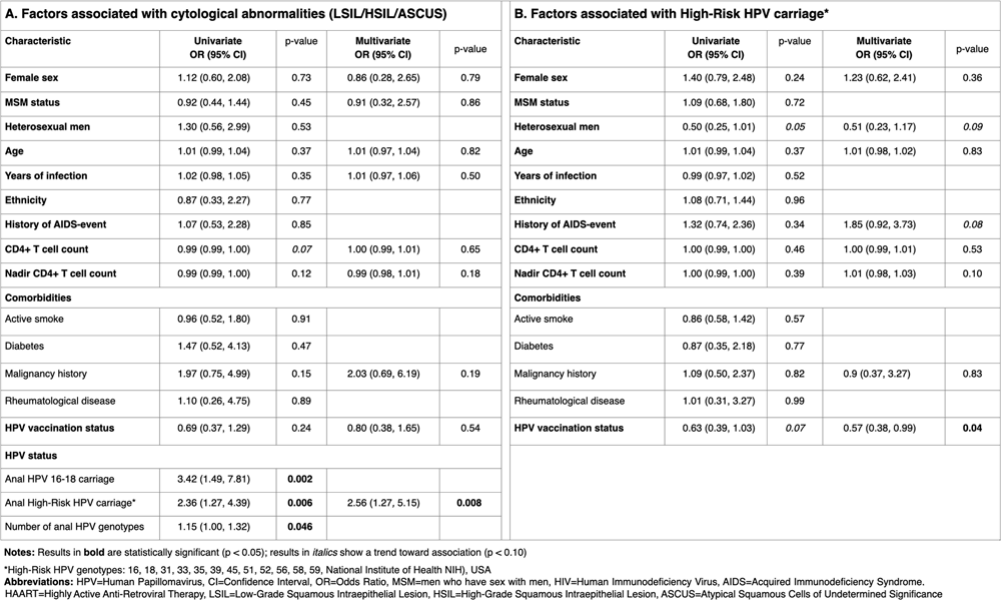
P443 | Multivariate analysis outcomes.

### P444 Two‐Thirds Mortality Within 5 Years of Lung Cancer Diagnosis Among People With HIV of an Italian Cohort

Gabriele Loi^1^, Dario Diana^2^, Nicolò Capra^3^, Riccardo Lolatto^3^, Vincenzo Spagnuolo^1,3^, Angelo Roberto Raccagni^1,3^, Tommaso Clemente^3^, Elena Bruzzesi^3^, Antonella Castagna^1,3^, Silvia Nozza ^1,3^

^1^Vita Salute San Raffaele University, Milan, Italy; ^2^University of Naples “Federico II”, Naples, Italy; ^3^Infectious Diseases Unit, IRCCS San Raffaele Scientific Institute, Milan, Italy

**Background**: Lung cancer is a major non‐virus‐related malignancy and an important cause of mortality among people with HIV (PWH). Although poor short‐term outcomes have been reported, contemporary data on long‐term survival following lung cancer diagnosis in PWH remain limited. We aimed to evaluate 1‐ and 5‐year overall mortality after lung cancer diagnosis in an Italian HIV cohort.

**Methods**: We retrospectively identified PWH followed at IRCCS San Raffaele Hospital, Milan, who were diagnosed with lung cancer between 2008 and 2025. Demographic, immunovirological, cancer and survival data were evaluated. Continuous variables are reported as median [interquartile range] and categorical variables as *n*/*N* (%). Clinical stage was assigned based on the latest National Comprehensive Cancer Network (NCCN) guidelines. Kaplan–Meier curves estimated overall survival and the corresponding 1‐ and 5‐year mortality, with 95% confidence intervals (CIs).

**Results**: Out of 7456 PWH in follow‐up, 51 had lung cancer between 2008 and 2025; at cancer diagnosis median age was 58.4 years [52.7–63.7] and 37/51 (72.6%) were male. Median time since HIV diagnosis was 26.2 years [16.9–30.6]; CD4 count at cancer diagnosis was 640 [374–814] cells/mm^3^, while HIV RNA was < 50 copies/mL in 46/51 (90.2%). Of 47 participants with smoking data, 37 (78.7%) were current smokers and 8 (17.0%) were former smokers. Among the 44 participants with available histology, non‐small‐cell lung cancer (NSCLC) accounted for 88.6%, adenocarcinoma being the most common subtype (61.1% of cases). At diagnosis, metastatic disease was present in 32/46 participants (69.6%), corresponding to stage IV cancer (Table 1). Among participants with available treatment data (*n* = 33), surgery was performed in 10 (30.3%), chemotherapy was administered in 19 (57.5%), immunotherapy in 14 (42.4%) and radiotherapy in 15 (45.4%). During a median follow‐up of 0.89 years [0.18–3.69], 32 deaths occurred. Estimated mortality was 44.0% (95% CI: 31.2–59.3) at 1 year and 66.5% (95% CI: 51.7–80.6) at 5 years (Figure 1).

**Conclusions**: Lung cancer in our cohort of PWH was predominantly diagnosed at an advanced stage and was associated with two‐thirds mortality within 5 years after diagnosis. These findings support efforts to promote timely cancer screening strategies, as well as coordinated HIV and oncologic care.

**FIGURE 1:**
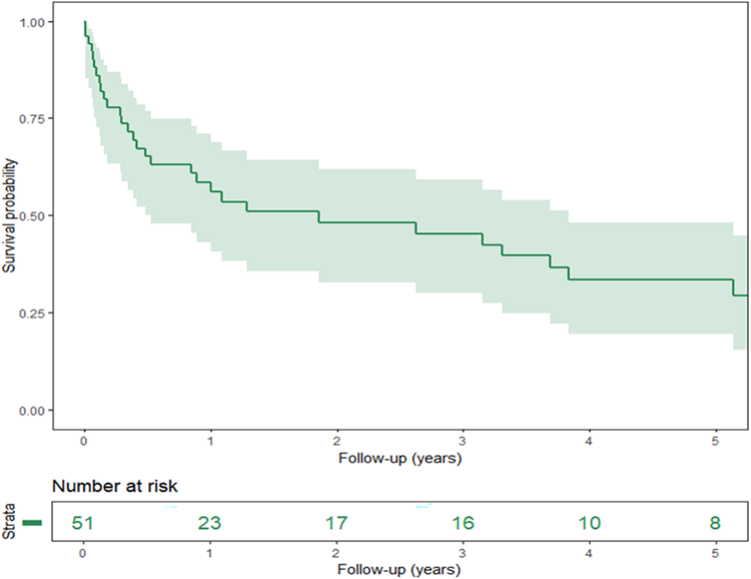
P444 | Kaplan–Meier estimates of overall survival from the date of lung cancer diagnosis in 51 people with HIV. Shaded areas represent 95% confidence intervals. Overall survival was 56.0% (95% CI: 40.7–68.8) at 1 year and 33.5% (95% CI: 19.4–48.3) at 5 years.

**TABLE 1:**
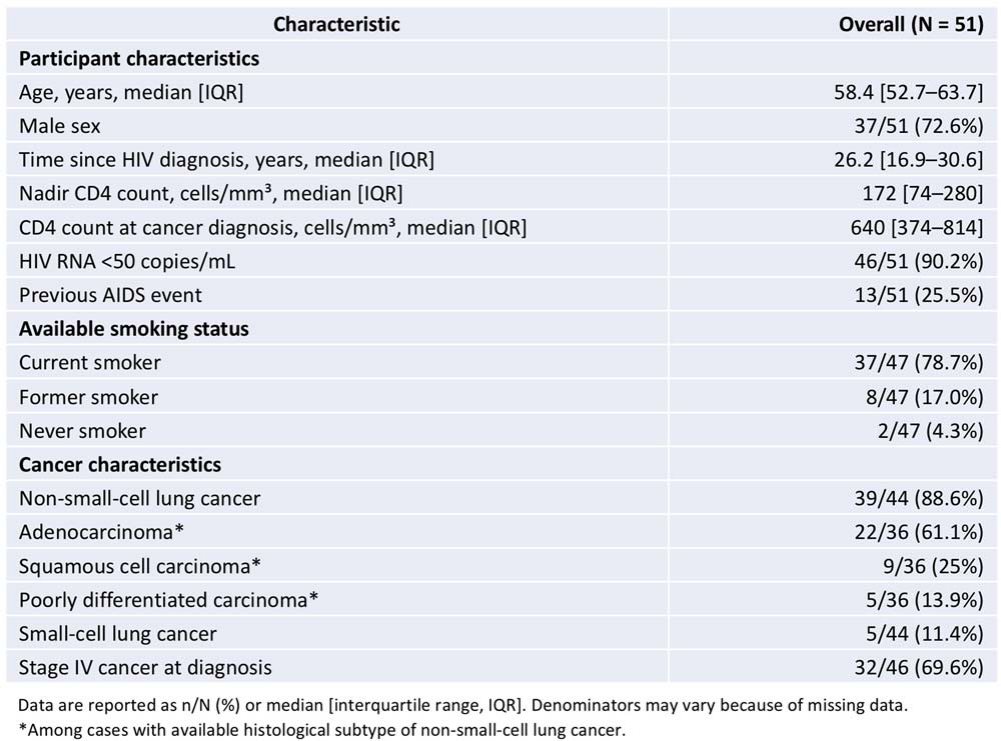
P444 | Characteristics of people with HIV diagnosed with lung cancer.

### P445 Burden of Lung Cancer Among People With HIV in Italy Compared to General Population

Pierluca Piselli ^1^, Claudia Cimaglia^2^, Alessandro Tavelli^3^, Valentina Mazzotta^4^, Arturo Montalbano^5^, Alberto Zuppiroli^6^, Adriana Cervo^7^, Loredana Sarmati^8^, Giuseppe Lapadula^9^, Maria Vittoria Cossu^10^, Antonella Castagna^11^, Diego Serraino^12^, Andrea Giacomelli^10^, Antonella Cingolani^13^, on behalf of ICONA Foundation Study Group

^1^Epidemiology, National Institute for Infectious Diseases L. Spallanzani, IRCCS, Rome, Italy; ^2^Epidemio logy, National Institute for Infectious Diseases L. Spallanzani, IRCCS, Rome, Italy; ^3^ICONA Foundation, Milan, Italy; ^4^Clinical Department, National Institute for Infectious Diseases L. Spallanzani, IRCCS, Rome, Italy; ^5^Infectious Diseases, Amedeo di Savoia Hospital, Turin, Italy; ^6^Medical and Surgical Sciences, Alma Mater University, Bologna, Italy; ^7^Infectious Diseases, University of Modena and Reggio Emilia, Modena, Italy; ^8^Infectious Diseases, University of Tor Vergata, Rome, Italy; ^9^Infectious Diseases, San Gerardo Hospital, IRCCS, Milan, Italy; ^10^Infectious Diseases, Sacco Hospital, Milan, Italy; ^11^Infectious Diseases, San Raffaele Hospital IRCCS, Milan, Italy; ^12^Epidemiology, Reference Oncologic Center, Aviano, Italy; ^13^Medical and Surgical Sciences, Fondazione Policlinico A. Gemelli IRCCS, Rome, Italy

**Background**: Lung cancer is a major non‐virus‐related malignancy and cause of cancer death among people with HIV (PWH). Despite effective antiretroviral therapy (ART), shared risk exposures and HIV‐associated vulnerabilities may affect outcomes. Contemporary estimates are needed to inform prevention and early diagnosis. We compared lung cancer incidence and mortality in PWH with those expected in Italy.

**Methods**: Two endpoint‐specific analyses were conducted within the ICONA cohort. Incidence was assessed among PWH diagnosed with HIV between 1997 and 2023, to improve precision for infrequent events. Mortality was assessed among participants enrolled between 2006 and 2023 and followed through 31 December 2024, focusing on contemporary fatal outcomes and aligning follow‐up with calendar‐period‐specific national mortality rates. Events were classified using ICD‐10. Standardized incidence ratios (SIRs) and mortality ratios (SMRs), with 95% CI, compared observed with expected events using Italian population rates stratified by sex, age, area and calendar period.

**Results**: The incidence analysis included 17,585 PWH contributing 150,364 person‐years. Overall, 70 incident trachea/bronchus/lung cancers were observed after HIV diagnosis (55 in males, 15 in females). Incidence was modestly higher than expected overall (SIR 1.18; 95% CI: 0.92–1.49), with significant excess among females (SIR 2.32; 95% CI: 1.30–3.83) but not males. Risk appeared anticipated in PWH aged 40–49 years, approximating that of the general population aged 50–59 years, while incidence overlapped at older ages. Higher incidence was observed in 2009–2015, without a temporal trend (Figure 1A,B). In the mortality analysis, 15,061 PWH contributed 102,258 person‐years. Among 53 PWH with lung cancer diagnosis, including 47 incident cases, 31 deaths occurred (25 in males, 6 in females). Mortality from trachea, bronchus and lung cancer was markedly elevated overall (SMR 3.50; 95% CI: 2.38–4.97) and in both sexes, particularly females (SMR 12.67 vs. 2.98 in males). Mortality was increased across age groups, with a slight decrease over time (Figure 1C,D).

**Conclusions**: Within ICONA, PWH in Italy showed a modest overall excess of lung cancer incidence, driven by females, and an anticipated risk. The disproportionate mortality burden supports intensified smoking cessation, consideration of earlier screening, and integrated HIV–oncology pathways to improve early detection and outcomes.

**FIGURE 1:**
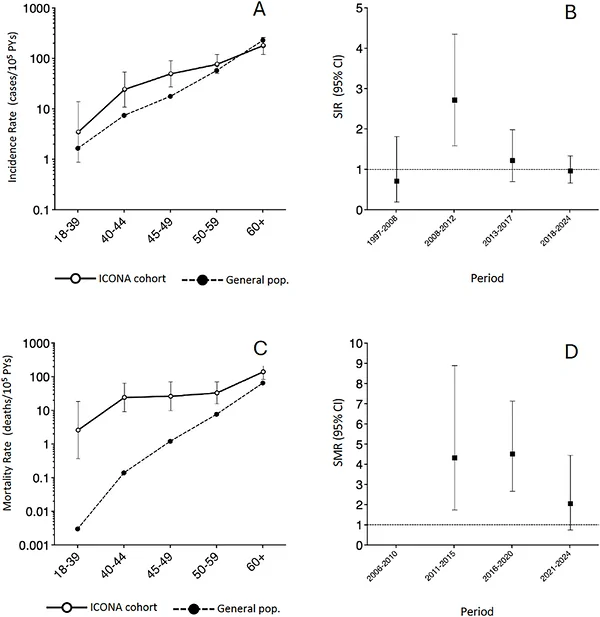
P445 | Comparison of age‐specific trachea, bronchus and lung cancer incidence rates in the ICONA cohort versus the general population (A). Standardized incidence ratio (SIR) for trachea, bronchus and lung cancer by period (B). Comparison of age‐specific trachea, bronchus and lung cancer mortality rates in the ICONA cohort versus the general population (C). Standardized mortality ratio (SMR) for trachea, bronchus and lung cancer by period (D).

### P446 Migration and HPV Mucosal Infection in People Living With HIV: A Prospective Spanish Cohort Study

Carmen Hidalgo‐Tenorio ^1^, Cristina Rodriguez‐Fernandez^1^, Saioa Igartua^2^, Mohamed Omar^3^, Javier Lopez Hidalgo^4^, Javier Rodriguez^5^, Mar Rivero^1^, Coral Garcia Vallecillos^1^, Inmaculada Calle^3^

^1^Infectious Diseases Service, Granada, Spain; ^2^Internal Medicine Service, Granada, Spain; ^3^Infectious Diseases Unit, Jaen, Spain; ^4^Pathology Service, Granada, Spain; ^5^Microbiology Service, Granada, Spain

**Background**: Migration has been consistently associated with increased vulnerability to sexually transmitted infections, yet its specific role as a risk factor for HPV infection and related mucosal dysplasia in people living with HIV (PLWH) remains unexplored. We, therefore, assessed the prevalence and 12‐month incidence of hr‐HPV infection and associated dysplasia across all three mucosal sites in migrant versus non‐migrant PLWH attending a specialized HIV clinic in Spain.

**Methods**: Prospective longitudinal study (12‐month follow‐up) consecutively enrolling PLWH attending a specialized HIV outpatient clinic in Spain. Anal and female genital cytology with HPV PCR/genotyping were performed at baseline and month 12. Patients with oropharyngeal HPV positivity or symptoms were referred to otorhinolaryngology; those with abnormal cytology or HPV at genital/anal sites were referred to gynaecology or underwent high‐resolution anoscopy, respectively.

**Results**: 276 PLWH were enrolled; 33 (11.9%) were migrants, 15 (45.5%) MSM and 18 (54.5%) women (*p* = 0.0001), from South America (60.6%), sub‐Saharan Africa (15.2%), Eastern Europe (15.2%) and Central Europe (9.1%). Migrants differed significantly from Spanish‐born participants in several baseline characteristics: higher HPV vaccine coverage (54.5% vs. 30.9%; *p* < 0.007), lower educational level (illiterate 15.2% vs. 3.3%; *p* = 0.05), heterosexual HIV acquisition route (43.8% vs. 12%; *p* < 0.001), higher rate of prior AIDS‐defining events (42.4% vs. 21.8%; *p* = 0.01), lower nadir CD4 count (239.8 vs. 348.8 cells/µL; *p* = 0.014) and lower current CD4 count (601.1 vs. 741.8 cells/µL; *p* = 0.013). No baseline differences were found in oropharyngeal HPV prevalence (12.1% vs. 11.1%; *p* = 0.774), female genital HPV infection (55.6% vs. 42.5%; *p* = 0.356), CIN1 (5.9% vs. 5.1%; *p* = 1.0), anal HPV infection (83.9% vs. 79.2%; *p* = 0.546), AIN1/LSIL (31% vs. 29.8%; *p* = 1.0) or AIN2–3/HSIL (3.2% vs. 1.3%; *p* = 0.387). No oropharyngeal dysplasia was detected in either group at baseline. At 12 months, migrants showed significantly higher anal hr‐HPV clearance (34.5% vs. 14.5%; *p* = 0.014) and lower new anal hr‐HPV acquisition (10.3% vs. 27.6%; *p* = 0.045), with no differences in oropharyngeal or female genital hr‐HPV dynamics. No new high‐grade dysplastic lesions emerged in either group at any mucosal site during follow‐up.

**Conclusions**: Migrant PLWH show a more favourable anal hr‐HPV dynamic at 12 months despite more advanced baseline immunosuppression. That this did not translate into fewer high‐grade lesions. Extended prospective follow‐up is needed to establish whether these findings carry meaningful protection against anal HSIL and anal cancer

### P447 Oral HPV Prevalence and Genotype Concordance With Anal HPV in HIV‐Positive MSM

Yoshiaki Kanno ^1^, Michiko Koga^1,2^, Aiko Okazaki^1,2^, Aya Ishizaka^3,4^, Eisuke Adachi^1^, Hiroshi Yotsuyanagi^1,5^

^1^Department of Infectious Diseases and Applied Immunology, IMSUT Hospital, Institute of Medical Science, University of Tokyo, Tokyo, Japan; ^2^University of Tokyo Pandemic Preparedness, Infection and Advanced Research Center, Tokyo, Japan; ^3^Department of Infectious Diseases and Applied Immunology, IMSUT Hospital, Institute of Medical Science, the University of Tokyo, Tokyo, Japan; ^4^AIDS Research Center, Japan Institute for Health Security, Tokyo, Japan; ^5^Japan Institute for Health Security, Tokyo, Japan

**Background**: Human papillomavirus (HPV)‐related oropharyngeal cancer is increasing [1]; however, the prevalence of oral HPV and the genotype concordance between oral and anal sites among people with HIV (PWH) remain incompletely characterized.

**Methods**: We conducted an observational study among PWH who have sex with men (MSM). Oral rinse/gargle and anal swab specimens were collected, and HPV genotyping was performed using PCR‐rSSO assay. The prevalence of oral and HPV infection and the distribution of HPV genotypes were evaluated. Concordance between oral and anal HPV infections were assessed for any HPV and for high‐risk HPV (HR‐HPV). HIV‐related clinical profiles were retrospectively collected from medical records.

**Results**: Among 48 PWH‐MSM enrolled in 2025 at IMSUT clinic, oral HPV infection had 27% (13/48) participants. Anal HPV was detected in 71% (29/41). The most frequently detected oral genotypes were HPV82: 4/48, HPV62: 2/48 and HPV66: 2/48. The most frequently detected anal genotypes were HPV52: 8/41, HPV6: 7/41 and HPV11: 6/41 (Figure 1A). In oral rinse/gargle specimens, HR‐HPV was detected significantly less frequently than low‐risk HPV (4.2% vs. 25.0%, *p* = 0.0063) (Figure 1A). The same HPV genotype was detected in both oral and anal specimens in 7.3% (3/41) of participants. No participant had concordant HR‐HPV genotypes. The median age was 57 years, and the median CD4‐positive T‐cell count was 579 cells/µL. Forty‐seven participants (97.9%) had HIV RNA levels below 200 copies/mL. No significant associations were observed between oral HPV positivity and age, current and nadir CD4 count, TPAb positivity or HBcAb positivity (Table 1).

**Conclusions**: Oral HPV detection was less frequent than anal HPV detection among HIV‐MSM. Concordance of HPV genotypes between oral rinse and anal swab specimens was low and HR genotypes were not concordant, suggesting that HPV infection may occur independently at these anatomical sites. These findings may indicate site‐specific differences in tissue tropism, viral persistence or assay sensitivity between oral and anal specimens.

**Reference**

1. E. A. Van Dyne, S. J. Henley, M. Saraiya, C. C. Thomas, L. E. Markowitz, and V. B. Benard, Trends in human papillomavirus‐associated cancers—United States, 1999–2015, MMWR. Morbidity and Mortality Weekly Report 67, no. 33. 2018, 918–924.

**FIGURE 1:**
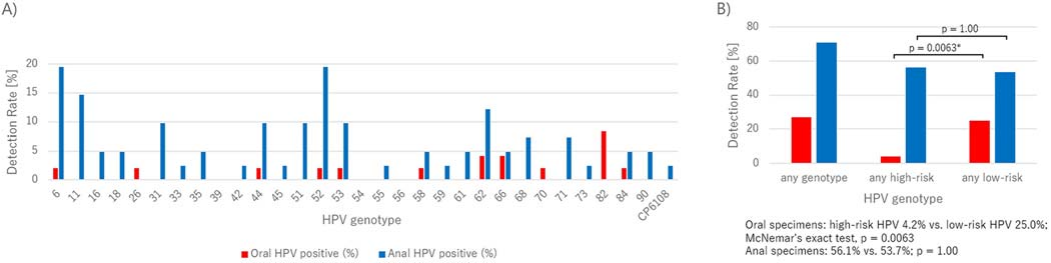
P447 | (A) HPV genotype distribution in oral and anal specimens; (B) difference of HPV detection rates between high‐risk and low‐risk HPV.

**TABLE 1:**
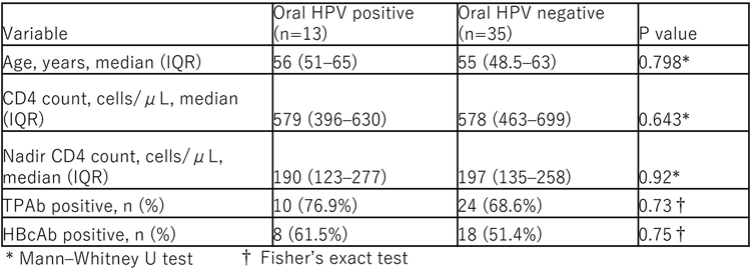
P447 | Comparison of characteristics according to oral HPV status.

## Co‐Morbidities and Complications of Disease and/or Treatment: Neurological

### P448 Greater Neurocognitive Decline in Older Adults With Well‐Controlled HIV Infection Compared With Matched Controls: The ANRS 0093s Ageing HAND Cohort Study

Alain Makinson ^1^, Laure‐Anne Gutierrez^2^, Christian Geny^3^, Eric Thouvenot^4^, Valerie Andriantsoanirina^5^, Manon Ollivier^6^, Matteo Vassallo^7^, Adrien Lemaignen^8^, Charles Cazanave^9^, Fabrice Bonnet^10^, Marion Bourgey^11^, Laurence Meyer^12^, Olivia Zaegel^13^, Thibault Mura^14^

^1^Infectious Diseases, University of Montpellier, Montpellier, France; ^2^Institute for Neurosciences of Montpellier, Inserm, Montpellier, Montpellier, France; ^3^Geriatric Department, University Hospital Montpellier, Montpellier, France; ^4^Neurology Department, University of Montpellier, Nimes, France; ^5^Université Paris Sarclay, CESP Inserm U101, Paris, France; ^6^ANRS/MIE, Paris, France; ^7^Internal Medicine & infectiology, Cannes Hospital, Cannes, France; ^8^Infectious Diseases Department, University of Tours, Tours, France; ^9^Infectious Diseases Department, University of Bordeaux, Bordeaux, France; ^10^Internal Medicine, University Hospital Bordeaux, Bordeaux, France; ^11^Infectious Diseases, University Hospital Montpellier, Montpellier, France; ^12^University Paris Sarclay, CESP Inserm U101, Paris, France; ^13^CISIH, Hôpital Sainte Margeurite, Marseille, France; ^14^University of Montpellier, Institute for Neurosciences of Montpellier (INM), Inserm, Montpellier, France, Department of Biostatistics, Clinical Epidemiology, Public Health, and Innovation in Methodology, Nimes, France

**Background**: We determined whether global neurocognitive performance declines more rapidly in older people with HIV (PWH) with controlled HIV infection than in matched individuals from the French general population cohort CONSTANCES.

**Methods**: The cohort is a follow‐up study of PWH previously enrolled in a multicentre neurocognitive evaluation. Participants aged 55–70 years with sustained virological suppression and CD4 cell counts of at least 200 cells/µL were matched to five HIV‐negative controls from CONSTANCES by age, sex and educational level; PWH were not included if confused, non‐fluent in French, had significant brain disease, psychiatric syndromes or sensorial loss. Neurocognitive performance and functional impact were assessed using validated and standardized tests covering multiple cognitive domains. The primary outcome was change in global neurocognitive performance, measured by a global Z score, and adjusted for smoking, cannabis use, physical activity, living alone, alcohol consumption, body mass index, kidney disease, hypertriglyceridemia, cardiovascular disease, hypertension, depression and the interaction of each covariate with time. Longitudinal trajectories were analysed using hierarchical linear mixed‐effects models.

**Results**: 117 PWH were matched to 578 controls, respectively, followed‐up for 6.8 years (IQR 5.6–7.7) and 5.0 years (3.9–7.0). Median age of PWH was 62.5 years (55.0–71.5), 20 (17%) were women, and 41 (35%) had university‐level education. Median current and nadir CD4 counts were 663 cells/µL (IQR 495–790) and 180 cells/µL (105–267), respectively. At baseline, global Z scores were lower in PWH than in controls (adjusted mean difference −0.117 SD, 95% CI: −0.211 to −0.024; *p* = 0.014). Estimated 5‐year change in global Z score was −0.171 SD in PWH and −0.097 SD in controls, with a greater decline among PWH (adjusted between‐group difference in change −0.074 SD, 95% CI: −0.135 to −0.012; *p* = 0.019) (Table 1). Among participants classified as cognitively normal at baseline, PWH were more likely than controls to develop Frascati‐defined impairment during follow‐up (adjusted relative risk 2.99, 95% CI: 1.79–4.43).

**Conclusions**: Older PWH with well‐controlled infection experienced a significantly greater decline in neurocognitive performance than non‐HIV matched controls. Mechanisms underlying these results need to be assessed.

**TABLE 1:**
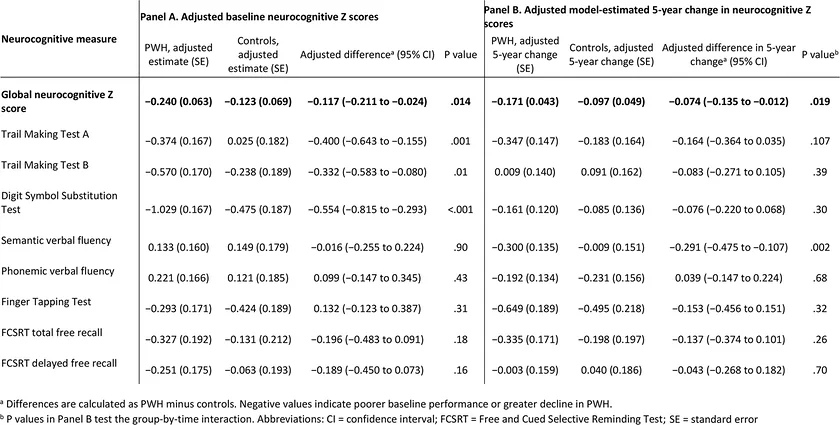
P448 | Adjusted baseline neurocognitive performance and 5‐year change in people with HIV and controls (higher Z scores indicate better neurocognitive performance).

### P449 Perceived Sleep Quality and Domain‐Specific Cognitive Performance in People With HIV: Unexpected Findings From a Neuropsychological Cohort Study

Giulia Del Duca ^1^, Ilaria Mastrorosa^1^, Mattia Trunfio^2^, Martina Maresca^1^, Anna Clelia Brita^1^, Roberta Gagliardini^1^, Sandrine Ottou^1^, Federico De Zottis^1^, Rita Bellagamba^1^, Giulia Micheli^1^, Alessandro Giacinta^1^, Valentina Mazzotta^1^, Carmela Pinnetti^1^

^1^National Institute for Infectious Diseases “Lazzaro Spallanzani” – IRCCS, Rome, Italy; ^2^Department of Clinical and Biological Sciences, University of Turin, Turin, Italy

**Background**: Sleep disorders have been linked to neuropsychiatric outcomes in people with HIV (PWH), although evidence regarding their impact on specific cognitive domains remains inconsistent, partly due to clinical confounders. We evaluated the association between perceived sleep quality and domain‐specific neurocognitive performance in a cohort of PWH.

**Methods**: Single‐centre retrospective study of PWH undergoing sleep assessment using the Pittsburgh Sleep Quality Index (PSQI) and neuropsychological assessment between 2016 and 2026. Poor sleep was defined as PSQI > 5. Cognitive performance was assessed through a standardized battery of 12 tests across five domains. Domain Z‐scores adjusted for demographic variables served as primary outcomes. Anxiety and depression were evaluated using the Beck Anxiety Inventory and Beck Depression Inventory‐II. Associations between sleep quality and cognitive outcomes were assessed through multivariable regression models adjusted for clinically relevant confounders.

**Results**: 304 participants (clinical characteristics shown in Table 1): 84.9% normal sleepers (NS), 15.1% poor sleepers (PS). The PSQI for the PS ranged from 6 to 15, with most < 8. Compared with NS, PS more frequently received Efavirenz (89.1% vs. 27.5%, *p* < 0.001), and less frequently integrase inhibitors (10.9% vs. 62.0%, *p* < 0.001), had lower rates of detectable viraemia (8.7% vs. 27.5%, *p* = 0.005) and higher CD4 counts (*p* = 0.008). At univariate, higher PSQI was associated with poorer cognitive flexibility but better processing speed, attention/working memory and memory. After adjustment for anxiety, depression, demographics and antiretroviral exposure, higher PSQI remained independently associated with better attention/working memory (adjusted β = 0.16, *p* = 0.034) (Figure 1). The PSQI‐cognitive flexibility association was largely explained by confounding from EFV use, with no evidence of effect modification or mediation. Similar findings were observed in the subgroup of virological suppression (*n* = 229). In secondary test‐level analyses, higher PSQI was associated with better Digit Span Backward (adjusted β = 0.23, *p* = 0.028), immediate recall on the Rey Auditory Verbal Learning Test (adjusted β = 2.059, *p* = 0.017) and Trail Making Test A (adjusted β=−3.60, *p* = 0.018).

**Conclusions**: Subjective poor sleep was uncommon and generally mild in this cohort. Unexpectedly, poorer perceived sleep quality was associated with better attention/working memory and selected measures of attention, memory, and processing speed. These findings may reflect a limited sensitivity of subjective sleep assessment, reduced awareness of sleep disturbances among cognitively impaired individuals, or compensatory mechanisms associated with mild sleep impairment.

**FIGURE 1:**
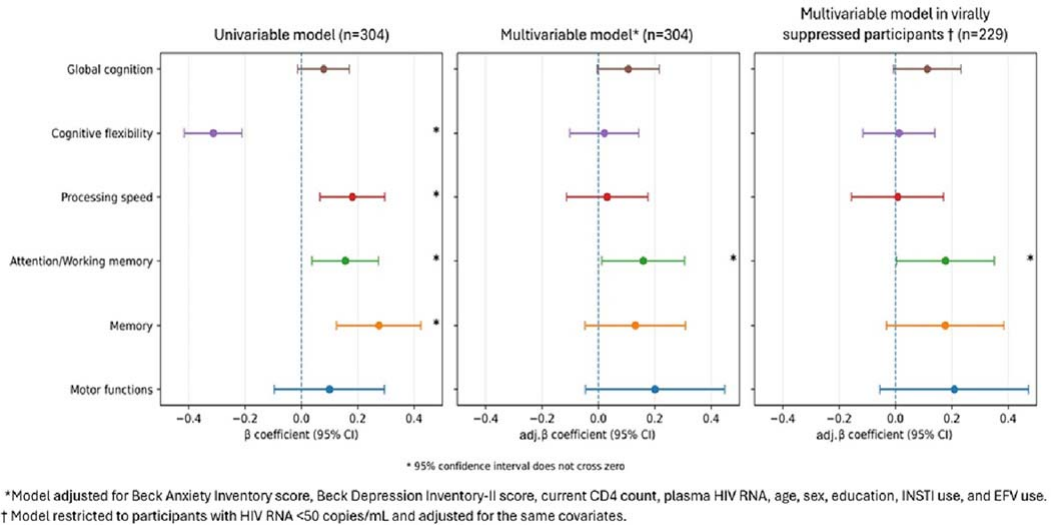
P449 | Forest plot for univariable and multivariable models of the relationship between PSQI score (predictor) and cognitive domain scores.

**TABLE 1:**
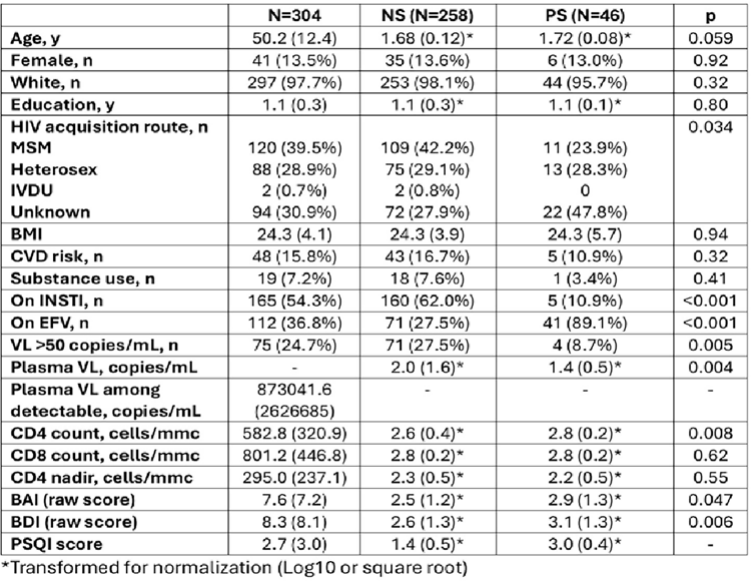
P449 | Study population and comparison between NS and PS.

### P450 Proteomic Profiling of CSF and Plasma in People With and Without HIV

Merle Henderson ^1,2^, Luxsena Sukumaran^3^, Caroline Sabin^3^, Owen Swann^4^, Amanda Heslegrave^4^, Henrik Zetterberg^4,5^, Alex Everitt^2^, John Tregoning^1^, Sarah Fidler^1,2^, Alan Winston^1,2^

^1^Department of Infectious Disease, Imperial College London, London, UK; ^2^St. Mary's Hospital, Imperial College Healthcare NHS Trust, London, UK; ^3^Institute for Global Health, University College London, London, UK; ^4^UK Research Dementia Institute, University College London, London, UK; ^5^Department of Psychiatry and Neurochemistry, Institute of Neuroscience and Physiology, Sahlgrenska Academy at the University of Gothenburg, Mölndal, Sweden

**Background**: Disorders of brain health remain prevalent in persons with HIV on antiretroviral therapy, with the underlying pathogenic mechanisms remaining elusive. Ultrasensitive proteomics platforms provide an opportunity to investigate potential mechanistic pathways.

**Methods**: Paired plasma and CSF samples were collected from persons undergoing cerebrospinal fluid (CSF) examination for diagnostic assessment (Imperial College Healthcare NHS Trust, 2022–2024). Samples were processed immediately and stored at −80°C prior to biomarker analysis (NULISA, University College London). Data were analysed using principal component analysis to reduce biomarker dimensionality, followed by hierarchical clustering to identify biomarker patterns within plasma and CSF. Heatmaps were used to visualize and categorize clusters into proteomic activation patterns, and between cluster differences explored.

**Results**: Of 43 individuals (30 [70%] with, 13 [30%] without HIV) the median age was 51 (IQR 40, 59) years, 32 (74%) were male and 21 (49%) of White ethnicity. Most persons with HIV were virologically suppressed (77%) with a median CD4 count 650 (IQR 505, 720) cells/µL. Clinical indications for CSF examination included the investigation of neurological and ocular symptoms and signs (*n* = 18; e.g. disturbance of memory, balance, headaches, papilloedema), HIV viraemia (*n* = 8) and neurosyphilis (*n* = 17). Distinct clusters of proteomic activation patterns (PAPs) were identified (low, low‐medium, medium, medium‐high and high‐PAP), which differed between compartments (Figure 1). While cluster assignments varied across compartments, medium‐high‐ and high‐PAP cerebrospinal fluid clusters aligned with medium‐ or high‐PAP plasma clusters. In cerebrospinal fluid, differences were observed between clusters by the number of underlying comorbidities (e.g. 1 [IQR 0,1] in cluster 1, 4 [IQR 3,5] in cluster 5), prior AIDS (e.g. 0% in cluster 2, 67% in cluster 5) and clinical diagnosis (e.g. neurosyphilis [100% cluster 1]); all *p* ≤ 0.02.

**Conclusions**: We describe distinct clusters of PAP biomarker expression which differed between the plasma and CSF compartments. These findings highlighting the value of CSF analysis in the evaluation of CNS disease, which may provide additional information not provided by blood biomarkers alone.

**FIGURE 1:**
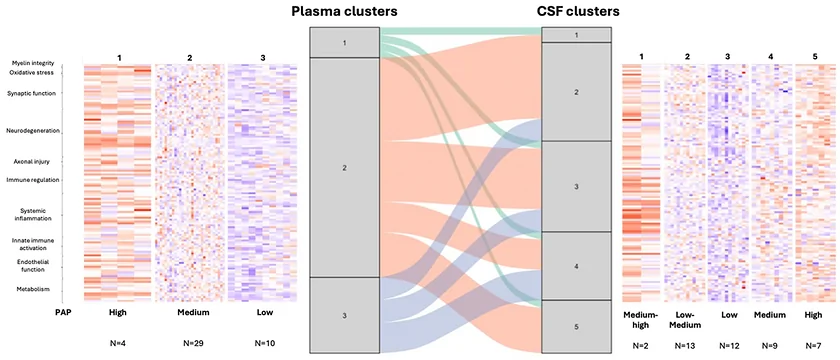
P450 | Cluster assignment between plasma and CSF compartments. Heatmaps of biomarker concentrations within the plasma (left) and CSF compartments (right). Upregulated biomarkers shown in red, downregulated in purple. Each column within a cluster represents a participant. Each row represents a different biomarker, which are subcategorized as shown on the left of the figure. Some individuals grouped into similar profiles across both compartments, but for others, the cluster assignments were different. Abbreviations: CSF, cerebrospinal fluid; PAP, proteomic activation pattern.

### P451 Domain‐Specific Cognitive Impairment in People With HIV on Suppressive ART: A Case−Control Study Using MoCA

Domenico Velluto^1^, Pasquale Troisi^1^, Valentina Fortunato^2^, Simona Madonia^2^, Mirella Onofrio^2^, Anna Maria Spera ^2^, Maria Teresa Pellecchia^1,3^, Pasquale Pagliano^1,2^

^1^University of Salerno, Salerno, Italy; ^2^Infectious Disease Unit, AOU San Giovanni di Dio e Ruggi d'Aragona, Salerno, Italy; ^3^Neurology Unit, AOU San Giovanni di Dio e Ruggi d'Aragona, Salerno, Italy

**Background**: HIV‐associated neurocognitive disorders (HAND) remain a relevant clinical problem, despite the use of antiretroviral therapy (ART), which has allowed a marked decline in severe HIV‐associated dementia. The Montreal Cognitive Assessment (MoCA) is widely used as a brief cognitive screening tool and may also support the stratification of cognitive vulnerability in people living with HIV.

The aim of the study was to compare global and domain‐specific MoCA performance between people with HIV (PWH) receiving ART and virologically suppressed and healthy controls (HCs) and to investigate whether cognitive performance differed according to the characteristics of virologic control.

**Methods**: This was an observational study including 93 PWH receiving ART and followed at the Infectious Disease Outpatient Clinic of the University Hospital “Ruggi” of Salerno, and 104 HC matched for age, sex, education and comorbidity. Patients with major potential confounders for neurocognitive assessment, including neurological, cerebrovascular, substance‐related and systemic conditions, were excluded. Cognitive performance was assessed using the MoCA Italian version. Global score and performance across the visuospatial, executive, attention and language domains were compared between PWH and HC. Data were analysed using the IBM SPSS Statistics, version 32.0, as appropriate, using non‐parametric tests.

**Results**: PWH showed lower global cognitive performance than HC (median MoCA score 22 vs. 25; *p* < 0.001). Even among virologically suppressed PWH, global MoCA performance remained significantly lower than in HC (median 22 vs. 25; *p* < 0.001), with poorer visuospatial (median 3 vs. 4), executive (2 vs. 3) and attentional (5 vs. 6) performance (all *p* ≤ 0.01). In clock‐drawing analysis, incorrect hand placement was associated with lower residual MoCA performance even after excluding the corresponding clock item from the total score (*p* = 0.019) (Table 1), suggesting possible involvement of fronto‐parietal impairment. No difference was found when patients were stratified according to evidence of duration of HIV infection, viral blips, T‐cell subsets dynamics.

**Conclusions**: Cognitive performance in PWH is characterized by global impairment and selective domain‐specific vulnerability. MoCA may be useful as initial cognitive screening tool, particularly when the total score is interpreted alongside domain‐ and item‐level error patterns.

**TABLE 1:**
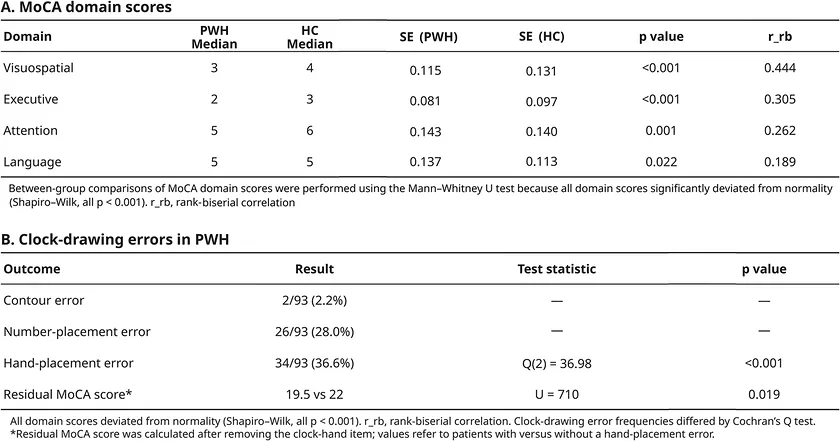
P451 | Domain‐specific cognitive performance and clock‐drawing error profile (people with HIV [PWH], *n* = 93; healthy controls [HCs], *n* = 104).

### P452 Neurosyphilis in the Era of Rising Syphilis: CSF Findings and Clinical Decision‐Making Concordance in a Real‐World Portuguese Cohort

Constança Azeredo, Lara Neto, Clara Bacelar, Sara Araújo, Maria João Gonçalves, Ricardo Correia de Abreu, Frederico Duarte

Infectious Diseases, Pedro Hispano Hospital, Matosinhos, Portugal

**Background**: Neurosyphilis (NS) is a neurological complication of *Treponema pallidum* infection that can occur at any stage of syphilis. Diagnosis remains challenging, as no single cerebrospinal fluid (CSF) test is both sensitive and specific. Rates of syphilis are rising globally, including among people living with HIV (PLWH). Concordance between CSF findings and clinical decision‐making was evaluated. We aimed to understand what factors favoured the diagnostic decision of NS in a real‐world cohort.

**Methods**: Single‐centre retrospective cohort study of adults with reactive syphilis serology referred for LP between 2014 and 2024. CSF results were classified as diagnostic (reactive CSF VDRL), supportive (protein > 45 mg/dL and/or pleocytosis and/or reactive CSF TPHA), or not supportive, following Smibert et al. [1]. Univariate odds ratios (OR) used Fisher's exact test; receiver operating characteristic (ROC) analysis with Youden‐optimal thresholds was applied to continuous markers.

**Results**: 126 individuals underwent LP (63.5% male, median age 56 years [IQR 45–68]); 20.6% were PLWH. NS was assumed in 15/126 (11.9%) and treatment initiated in 14/118 analysable individuals (11.9%). CSF VDRL was reactive in 5/126 (4.0%) and CSF TPHA in 15/126 (11.9%). All treated individuals met at least supportive CSF criteria; 45.2% of untreated individuals had supportive criteria. Neurological or ocular symptoms were the highest‐yield LP indication (NS rate 18.8% vs. 6.7–10.9% for other indications). Reactive CSF TPHA (OR 50.0, 95% CI: 11.5–216.8), pleocytosis (OR 14.3, 95% CI: 3.9–52.8) and serum VDRL ≥1:32 (OR 9.7, 95% CI: 2.9–31.8) were the strongest factors that favoured clinical decision of diagnosis of NS (all *p* < 0.001). HIV status was not associated with NS (OR 0.9, *p* = 1.000). ROC analysis identified serum VDRL titre (AUC 0.78, optimal cut ≥1:32) and CSF nucleated cells (AUC 0.77, optimal cut > 7/µL) as the best continuous discriminators. Key differences between groups are shown in the Table 1.

**Conclusions**: Therapeutic decisions were well aligned with CSF findings; no individual was treated without supportive criteria. CSF VDRL sensitivity was low (4.0%), underscoring reliance on multimodal diagnostics. Reactive CSF TPHA, serum VDRL ≥1:32 and CSF nucleated cells > 7/µL were the findings that most strongly favoured clinicians’ decision to diagnose NS and may inform data‐driven LP selection algorithms.

**Reference**

1. Smibert OC, Abbinga S, Spelman DW, Jenney AWJ. Neurosyphilis: Concordance between cerebrospinal fluid analysis and subsequent antibiotic strategy for patients undergoing evaluation of a diagnosis of neurosyphilis. Int J Infect Dis. 2019;82:73–76.

**TABLE 1:**
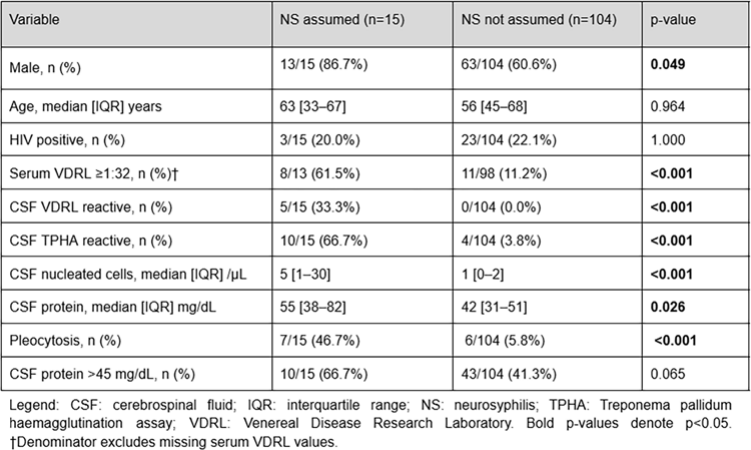
P452 | Comparison between assumed and not assumed neurosyphilis groups.

## Co‐Morbidities and Complications of Disease and/or Treatment: Mental Health Disorders

### P453 Sleep Disturbances in People Living With HIV on Stable Antiretroviral Therapy: A Cross‐Sectional Screening Analysis

Vito Speciale ^1^, Daniele Pucci^1^, Anna Carraro^1^, Andrea Gasperin^2^, Elker De Santis^2^, Alessandro Di Nardo^1^, Margherita De Masi^2^, Daniela Di Trento^2^, Giulia Mancarella^2^, Valeria Filippi^3^, Lorenzo Ansaldo^2^, Cosmo Del Borgo^2^, Serena Vita^4^, Miriam Lichtner^4^, Raffaella Marocco^2^

^1^Sapienza University of Rome, Rome, Italy; ^2^Infectious Diseases Unit, S.M Goretti Hospital, Sapienza University, Latina, Italy; ^3^UOC Infectious Disease Sant'Andrea Hospital, Rome, Italy; ^4^Department of NESMOS, Sapienza University of Rome, Rome, Italy

**Background**: Sleep disturbances are increasingly recognized as an important contributor to impaired quality of life, frailty and neurocognitive burden among people living with HIV (PLWH) [1–4]. Despite virological suppression and modern antiretroviral therapy (ART), insomnia remains highly prevalent, although real‐world outpatient data are still limited [1, 2].

**Methods**: Clinically stable PLWH attending routine outpatient follow‐up were invited to complete validated sleep questionnaires during clinical visits, including the Insomnia Severity Index (ISI) and Epworth Sleepiness Scale (ESS). ISI ≥8 was considered indicative of at least subclinical insomnia, while ISI ≥15 identified moderate‐to‐severe insomnia [5]. ESS > 10 defined excessive daytime sleepiness (EDS). Demographic, immunological and clinical variables were collected from routine clinical records. Non‐parametric tests and Spearman correlations were used for statistical analysis.

**Results**: A total of 146 PLWH were included; 68.5% were male, with median age 55 years (IQR 45–70) and median BMI 25.0 kg/m^2^ (23.1–27.1). Median CD4 count was 664 cells/mm^3^ (447–891), while median CD4/CD8 ratio was 0.9 (0.7–1.4). All patients were virologically suppressed. The most represented ART regimens were bictegravir/emtricitabine/tenofovir alafenamide, long‐acting cabotegravir/rilpivirine and dolutegravir/lamivudine (Table 1). Median ESS was 5 (3–6). EDS (ESS > 10) was observed in 8.3% of patients, while severe EDS (ESS ≥16) was present in 2.8%. Median ISI was 5 (2–11). Overall, 37.7% of patients presented at least subclinical insomnia (ISI ≥8), while 15.1% showed moderate‐to‐severe insomnia (ISI ≥15) (Figure 1). Smoking exposure was associated with significantly higher ESS scores compared with non‐smokers (6 [4–7] vs. 4 [2–6]; *p* = 0.0063). Patients receiving sleep medications showed significantly higher ISI scores (11 [6.5–15.2] vs. 4 [2–10]; *p* = 0.0014). Previous HCV infection was associated with higher ISI values (9 [6–12] vs. 4 [2–11.8]; *p* = 0.0406). No significant associations emerged between sleep outcomes and specific ART regimens.

**Conclusions**: Insomnia represents a frequent and clinically relevant condition among virologically suppressed PLWH, whereas EDS appears less prevalent. Sleep impairment was associated mainly with smoking exposure, previous HCV infection and chronic clinical vulnerability rather than with specific ART regimens, supporting the potential role of persistent immune dysfunction and inflammaging in sleep disorders among ageing PLWH.

**References**

1. Lee S, Oh JW, Park KM, et al. The prevalence and moderating factors of sleep disturbances in people living with HIV: A systematic review and meta‐analysis. Sci Rep. 2024;14:14817.

2. Kunisaki KM, De Francesco D, Sabin CA, et al. Sleep disorders in human immunodeficiency virus: A substudy of the Pharmacokinetics and Clinical Observations in People Over Fifty (POPPY) Study. Open Forum Infect Dis. 2021;8(1).

3. Daubert E, French AL, Burgess HJ, et al. Association of poor sleep with depressive and anxiety symptoms by HIV disease status: Women's Interagency HIV Study. J Acquir Immune Defic Syndr. 2022;89(2):222–230.

4. Patil SP, Brown TT, Jacobson LP, et al. Sleep disordered breathing, fatigue, and sleepiness in HIV‐infected and ‐uninfected men. PLoS One. 2014;9(7).

5. Morin CM, Belleville G, Bélanger L, Ivers H. The Insomnia Severity Index: Psychometric indicators to detect insomnia cases and evaluate treatment response. Sleep. 2011;34(5):601–608.

**FIGURE 1:**
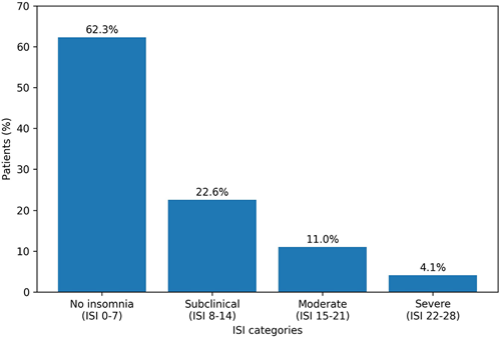
P453 | Distribution of insomnia severity according to Insomnia Severity Index (ISI) categories among people living with HIV.

**TABLE 1:**
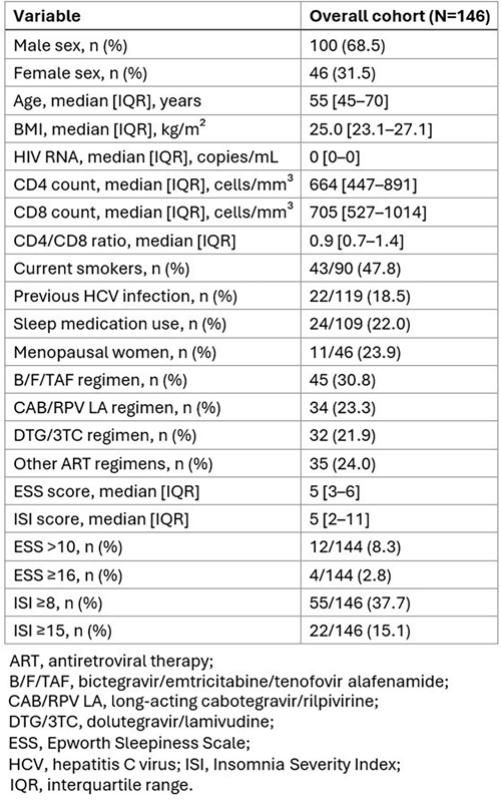
P453 | Baseline demographic and clinical characteristics of the study population.

### P454 Persistent Cognitive Depressive Symptoms and Their Link to Neurocognitive Status in People With HIV: A Longitudinal Study

Giulia Del Duca, Alice Lener, Gianluca Frate, Martina Maresca, Valentina Mazzotta, Roberta Gagliardini, Ilaria Mastrorosa, Carmela Pinnetti, Andrea Antinori

INMI L. Spallanzani, Rome, Italy

**Background**: As people with HIV (PWH) age on antiretroviral therapy (ART), distinguishing HIV‐related neurocognitive impairment from treatment effects and psychiatric comorbidity becomes increasingly difficult. Standard mood scales treat depression as a unitary construct, conflating somatic‐affective and cognitive components, a distinction relevant where neurocognitive dysfunction overlaps with depressive cognition. We examined whether disaggregating depression reveals distinct trajectories shaped by neurocognitive status, sex and ART regimen.

**Methods**: Longitudinal analysis of PWH who underwent neuropsychological assessment at baseline (T0) and at 1‐year follow‐up (T1) from January 2018 to March 2025. HIV‐associated neurocognitive disorder (HAND) was classified according to the Frascati criteria. Anxiety and depression were assessed using the Beck Anxiety Inventory (BAI) and Beck Depression Inventory‐II (BDI‐II), with the latter decomposed into cognitive and somatic‐affective subscales. Multivariable logistic regression identified independent predictors of clinically significant affective symptoms.

**Results**: Of 181 PWH assessed (median age 56 years, IQR 46–63; 85.6% male), 32% had normal cognition and 68% had a diagnosis of ANI or MND. The cognitive depressive subscale showed markedly greater persistence than the somatic‐affective subscale, with 16.6% remaining pathological at T1 versus 8.8%, indicating a neurocognitive‐affective overlap that global scores would miss (Figure 1). In multivariable models, neurocognitive impairment was strongly and selectively associated with anxiety but not with depression: ANI/MND (aOR 11.42, 95% CI: 5.38–24.24; *p* < 0.001) and unclassifiable HAND (aOR 11.51; *p* = 0.001) both markedly increased the odds of anxiety. Female sex independently increased the odds of both anxiety (aOR 4.33, 95% CI: 1.28–14.63; *p* = 0.019) and depression (aOR 4.13, 95% CI: 1.45–11.78; *p* = 0.008). Among ART regimens, legacy efavirenz exposure remained associated with higher odds of anxiety (aOR 19.22, 95% CI: 3.34–110.59; *p* = 0.001) and depression (aOR 5.70; *p* = 0.040) versus B/F/TAF, while DTG+3TC showed a non‐significant trend (aOR 2.23; *p* = 0.342, Table 1).

**Conclusions**: Disaggregating depression into cognitive and somatic‐affective components reveals a selectively persistent cognitive depressive burden in PWH that conventional mood scores obscure and that tracks with neurocognitive status rather than somatic distress. Together with the selective anxiety–HAND association and a disproportionate affective burden in women, these findings support symptom‐disaggregated, ART‐aware psychiatric monitoring as a standard component of long‐term HIV care.

**FIGURE 1:**
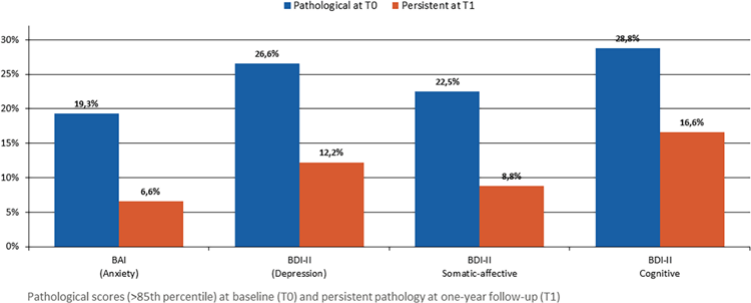
P454 | Longitudinal trajectories of depression and anxiety among 181 PLWH.

**TABLE 1:**
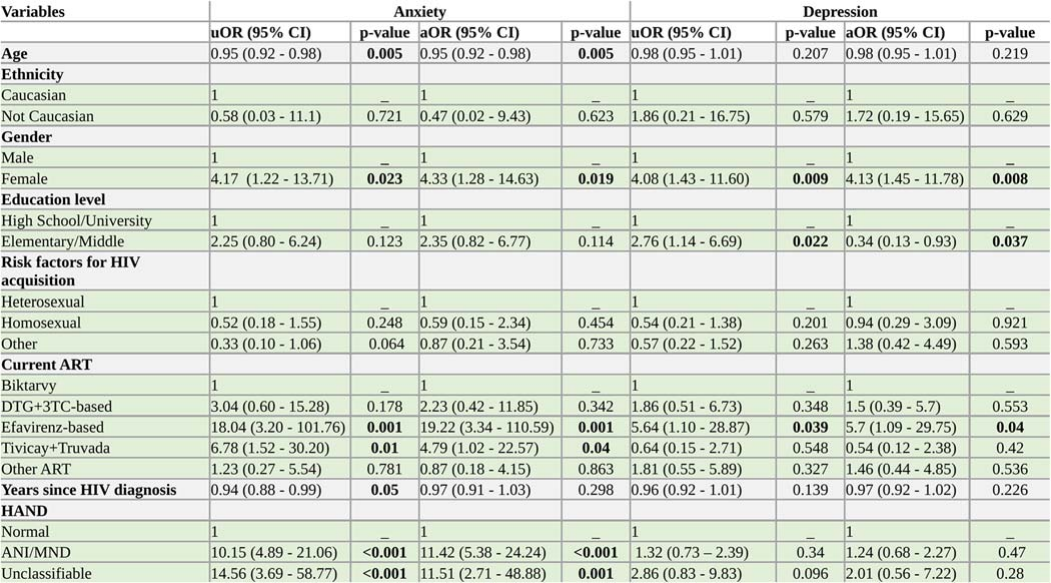
P454 | Multivariable logistic regression analysis.

### P455 Major Depressive Episodes and Psychiatric Symptoms Among HIV Controllers Compared With Antiretroviral‐Treated People Living With HIV

Marion Camard^1,2^, Samia Hendou^3^, Marie Bitu^2^, Christelliah Mouanga^2^, Faroudy Boufassa^3^, Salomé Laurans^2^, Jérôme Le Chenadec^3^, Léo Plaçais^1,2^, Véronique Avettand‐Fenoël^4,5^, Laurence Meyer^3,6^, Christine Bourgeois^2^, Emmanuelle Corruble^7,8^, Olivier Lambotte^1,2^, Romain Colle^7,8^, Nicolas Noel ^1,2^

^1^AP‐HP, Hôpital Bicêtre, Service de Médecine Interne et Immunologie Clinique, Le Kremlin‐Bicêtre, France; ^2^Université Paris‐Saclay, Inserm, CEA; Immune Diseases, Microbiology and Innovative Therapies (IDMIT/UMRS1184), Fontenay‐aux‐Roses & Le Kremlin‐Bicêtre, France; ^3^Université Paris‐Saclay, Inserm, Center for Research in Epidemiology and Population Health (CESP), INSERM U1018, Le Kremlin Bicêtre, France; ^4^CHU d'Orléans, Virologie, Orléans, France; ^5^Université d'Orléans, LI2RSO, Orléans, France; ^6^AP‐HP, Hôpital Bicêtre, Service de Santé Publique, Le Kremlin‐Bicêtre, France; ^7^AP‐HP, Hôpital Bicêtre, Service Hospitalo‐Universitaire de Psychiatrie de Bicêtre, Mood Center Paris Saclay, Le Kremlin‐Bicêtre, France; ^8^MOODS Team, Université Paris‐Saclay, Inserm, Center for Research in Epidemiology and Population Health (CESP), INSERM U1018, Le Kremlin‐Bicêtre, France

**Background**: Major depressive episodes are highly prevalent among people living with HIV (PWH), despite virological suppression [1]. Increasing evidence suggests that chronic inflammation and immune activation may contribute to psychiatric symptoms in PWH [2, 3]. HIV controllers (HICs), who maintain spontaneous viral control without antiretroviral therapy (ART), represent a unique model to explore the relationship between immune activation and clinical outcomes, including psychiatric symptoms. However, psychiatric outcomes in this population remain poorly characterized [4]. We aimed to compare psychiatric outcomes between HIC and ART‐treated PWH and to investigate their immunological correlates.

**Methods**: In this prospective observational study, HIC from the French ANRS CO21 CODEX cohort were compared with PWH receiving suppressive ART. Major depressive episodes were assessed using the Mini‐International Neuropsychiatric Interview (M.I.N.I.) and psychiatric symptoms using the Symptom Checklist‐90‐Revised. Immunological assessments included flow cytometry of myeloid and lymphoid cell subsets, inflammatory cytokines and expression of interferon‐stimulated genes (ISGs). Associations between psychiatric outcomes and clinical, virological, and immunological parameters were evaluated.

**Results**: Ninety‐eight participants were included (57 HIC and 41 ART‐treated PWH). The prevalence of lifetime major depressive episodes was similarly high in HIC and ART‐treated participants (35% vs. 41%) (Table 1). Psychiatric outcomes were associated with pre‐existing psychiatric history, alcohol use and socio‐demographic factors, but not with conventional immunovirological parameters, including HIV RNA, HIV DNA, CD4 T‐cell count, CD4/CD8 ratio or controller status. In contrast, several associations were observed with markers of innate immune activation and interferon signalling. Global symptom distress, assessed using the SCL‐90‐R Positive Symptom Distress Index (PSDI), was positively associated with expression of the interferon‐stimulated genes IFITM1 (Figure 1) and IFI44L, and these associations remained significant after adjustment for controller status. Higher IL‐15 levels and increased expression of the regulatory receptors VISTA and LILRB2 on non‐classical monocytes were associated with several SCL‐90‐R psychiatric symptom dimensions. No associations were observed with lymphoid parameters.

**Conclusions**: Despite spontaneous viral control, HIC did not exhibit more favourable psychiatric outcomes than ART‐treated PWH. Psychiatric symptoms were not associated with conventional HIV‐related clinical or immunovirological parameters but were linked to interferon‐stimulated gene expression and markers of innate immune regulation. These findings support a potential role of innate immune pathways in psychiatric symptom burden among PWH.

**References**

1. Gooden TE, Gardner M, Wang J, et al. The risk of mental illness in people living with HIV in the UK: A propensity score‐matched cohort study. Lancet HIV. 2022;9.

2. Mudra Rakshasa‐Loots A, Whalley HC, Vera JH, Cox SR. Neuroinflammation in HIV‐associated depression: Evidence and future perspectives. Mol Psychiatry. 2022;27:3619–3632.

3. Anderson AM, Bhondoekhan F, Curanovic D, et al. Higher soluble CD163 in blood is associated with significant depression symptoms in men with HIV. J Acquir Immune Defic Syndr. 2022;91:325–333.

4. Préau M, Mora M, Puppo C, et al. Does quality of life and sexual quality of life in HIV patients differ between non‐treated HIV controllers and treated patients in the French ANRS VESPA 2 national survey? AIDS Behav. 2019;23:132–139.

**FIGURE 1:**
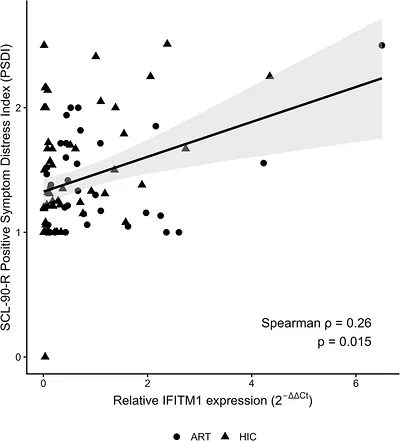
P455 | Association between IFITM1 relative expression and psychological distress severity assessed using the SCL‐90‐R Positive Symptom Distress Index (PSDI). Relative IFITM1 expression was quantified by RT‐qPCR using the 2^‐ΔΔCt method and plotted against SCL‐90‐R Positive Symptom Distress Index (PSDI) scores. Circles represent ART‐treated people living with HIV (ART) and triangles represent HIV controllers (HIC). The solid line represents the linear regression fitted across all participants, and the shaded area indicates the 95% confidence interval. A weak but significant positive association was observed between IFITM1 expression and PSDI score (Spearman's ρ = 0.26, *p* = 0.015). This association remained significant after adjustment for HIV control status (ART‐treated vs. HIC).

**TABLE 1:**
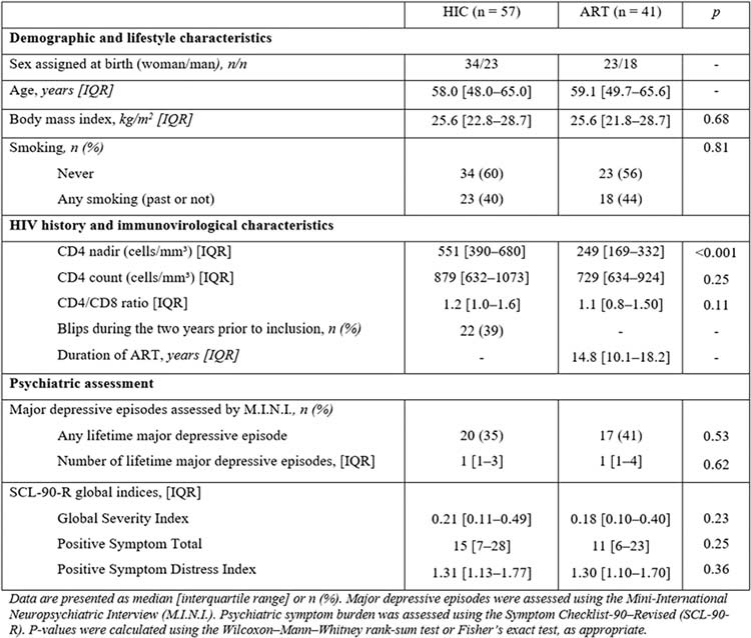
P455 | Demographic, immunovirological and psychiatric characteristics of HIV controllers (HIC) and ART‐treated people living with HIV.

### P456 Psychological Distress and Housing Instability as Warning Signs for Early Loss to Follow‐Up Among Latin American Asylum Seekers Living With HIV

Samuel Manzano, Jorge Valencia, Guillermo Cuevas, Victor De la Fuente, Juan Torres Macho, Pablo Ryan

Internal Medicine. Hospital Universitario Infanta Leonor, Madrid, Spain

**Background**: Retention in HIV care is essential to maintain virological suppression, but may be challenging among asylum seekers exposed to social instability and psychological distress. We aimed to evaluate whether baseline psychological and psychosocial vulnerability was associated with loss to follow‐up at 6 months among Latin American asylum seekers living with HIV in Madrid.

**Methods**: We performed an exploratory analysis within a prospective cohort of Latin American asylum seekers living with HIV attending a specialized HIV clinic. Loss to follow‐up at 6 months was analysed as a binary outcome. Baseline sociodemographic, clinical, behavioural and psychosocial variables were compared according to follow‐up status. Psychological assessment included PSS‐14, HADS‐A, HADS‐D, HSS‐30 and EARLY‐G. As shown in Table 1, bivariate analyses were used to identify variables associated with loss to follow‐up. To avoid overfitting and collinearity between scales, independent logistic regression models were built for each scale, adjusted for age, gender recoded as cisgender male versus other genders, homelessness and drug use.

**Results**: Among 603 patients with available 6‐month follow‐up data, 130 (21.6%) were lost to follow‐up. In bivariate analysis, homelessness was associated with loss to follow‐up (43.8% vs. 17.0%; *p* < 0.001), while age, gender, educational level, social support, healthcare access barriers, previous HIV treatment status, ART maintenance and detectable baseline viral load were not significantly associated. Drug use showed a non‐significant trend. In adjusted models, higher PSS‐14 scores were independently associated with loss to follow‐up (aOR 1.054 per point; 95% CI: 1.013–1.096; *p* = 0.009), as were HADS‐D (aOR 1.098; 95% CI: 1.014–1.189; *p* = 0.021) and EARLY‐G scores (aOR 1.170; 95% CI: 1.026–1.335; *p* = 0.020). HADS‐A showed a non‐significant trend (aOR 1.071; 95% CI: 0.995–1.153; *p* = 0.067), whereas HSS‐30 was not independently associated (aOR 1.017; 95% CI: 0.995–1.040; *p* = 0.128). Homelessness remained consistently associated across models.

**Conclusions**: Perceived stress, depressive symptoms, adverse childhood experiences and homelessness were independently associated with early loss to follow‐up. Future studies should explore whether risk stratification approaches based on psychological distress and housing instability can help identify patients at higher risk of disengagement from HIV care and inform targeted retention interventions.

**TABLE 1:**
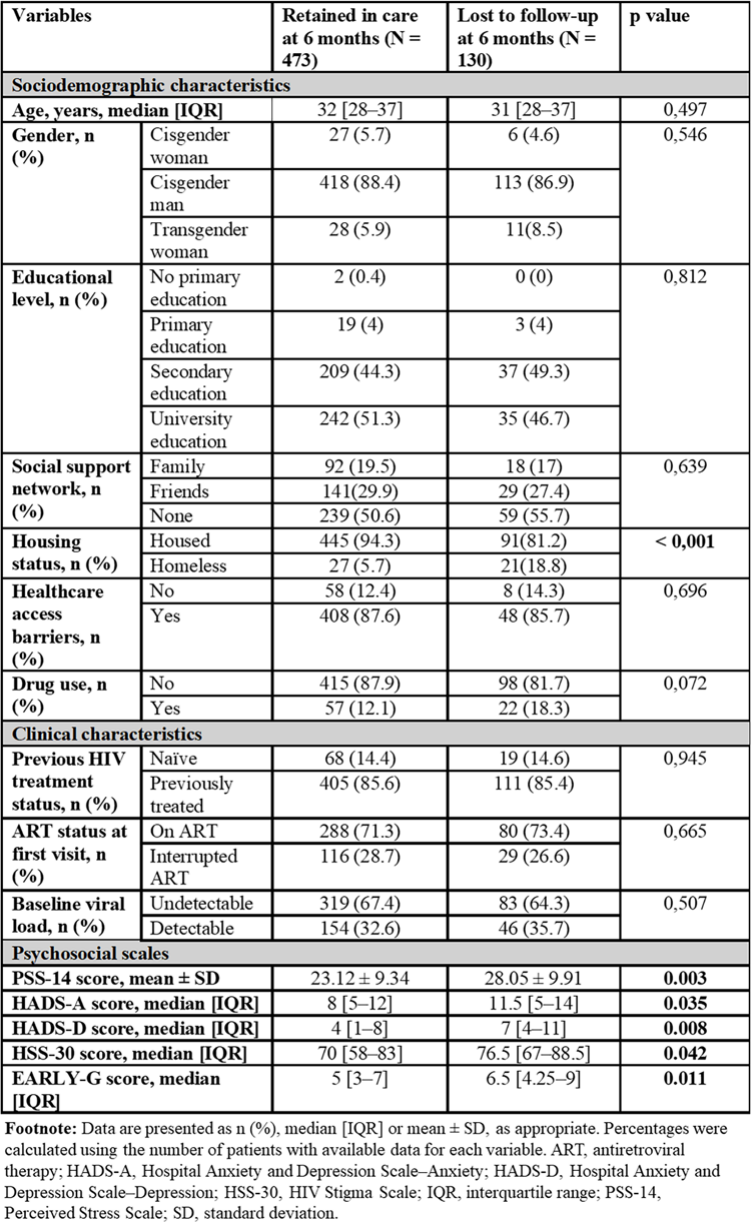
P456 | Baseline sociodemographic, clinical and psychosocial characteristics according to 6‐month follow‐up status.

### P457 Hidden Interface: HIV, Severe Mental Illness and Missed Opportunities for Care

Kevan Hilemichel^1^, Sylviane Defres^2^, Paul Hine ^2^

^1^University of Liverpool, Liverpool, UK; ^2^University Hospitals Liverpool Group, Liverpool, UK

**Background**: People living with HIV frequently experience comorbid mental illness, which can disrupt engagement with care, adherence and long‐term outcomes. Psychiatric admission represents a period of clinical vulnerability but may also provide an opportunity to support engagement with HIV care. Our aim was to evaluate HIV outcomes and engagement with HIV care in people living with HIV following psychiatric admission within a tertiary HIV service.

**Methods**: We conducted a retrospective service evaluation using electronic patient records and multidisciplinary team (MDT) documentation in a multisite HIV service in Cheshire and Merseyside. We screened 1968 HIV MDT entries to identify people living with HIV admitted to inpatient psychiatric services within the preceding 3 years. We analysed each admission episode separately. We collected data on demographics, indication for admission, length of stay, viral load before and after admission, and post‐discharge HIV follow‐up. We performed descriptive analysis using Microsoft Excel.

**Results**: We identified 18 patients accounting for 25 psychiatric admission episodes. Most patients were male (78%) and aged 30–49 years (72%); 67% lived in the most deprived IMD deciles. Compared with the wider HIV cohort, this group was younger and more socioeconomically deprived, with a greater proportion aged 30–49 and living in the most deprived IMD deciles. Psychotic disorders were the primary reason for admission in 80% (20/25). Admissions were often prolonged (median 23 days, range 6–104), representing a sustained opportunity for intervention. Viral suppression prior to admission was documented in 23/25 admissions, indicating high baseline engagement with care. Post‐admission viral load monitoring was inconsistent: suppression was documented in 15 admissions, while 6/25 had no recorded viral load following discharge. Post‐discharge HIV follow‐up was documented in 15/25 admissions. In the remaining admissions, there was no evidence of follow‐up (4), documented non‐attendance (1) or ongoing attempts at engagement (5).

**Conclusions**: People living with HIV admitted to psychiatric services were largely engaged and virologically suppressed prior to admission, but continuity of HIV care was inconsistent following discharge. Psychiatric admission represents a key interface and a sustained opportunity to improve engagement. Strengthening HIV service to mental health in‐reach, clarifying responsibility for follow‐up, and improving coordination between services may reduce missed opportunities for care and improve outcomes.

### P458 The Accuracy of a Multidimensional Screening Questionnaire for People With HIV: The MUSA Study

Andrea Calcagno ^1,2^, Elisa Sala^3^, Maria Cristina Poliseno^4^, Maria Mazzitelli^5^, Giordano Madeddu^6^, Benedetto Maurizio Celesia^7^, Giulia C. Marchetti^8^, Laura Petroli^9^, Maria Vittoria Cossu^10^, Paola Cinque^11^, Simona Di Giambenedetto^12^, Mauro Giacomini^13^, Rosaria Iardino^3^, Daniela Vai^14^, Luisa Brogonzoli^3^

^1^Department of Translational Medicine, University of “Piemonte Orientale”, Novara, Italy; ^2^SC Malattie Infettive, “AOU” Maggiore della Carità, Novara, Italy; ^3^Fondazione The Bridge, Milan, Italy; ^4^Clinic of Infectious Diseases, DiMePre‐J, Università of Bari, Bari, Italy; ^5^Infectious and Tropical Diseases Unit, Padua University Hospital, Padua, Italy; ^6^Unit of Immune Deficiency, Department of Medicine, Surgery and Pharmacy, University of Sassari, Sassari, Italy; ^7^Unit of Infectious Diseases, Garibaldi Hospital, Catania, Italy; ^8^Department of Health Sciences, Clinic of Infectious Diseases, University of Milan, ASST Santi Paolo e Carlo, Milan, Italy; ^9^Department of Medical Sciences, University of Turin, Turin, Italy; ^10^Department of Infectious Diseases, ASST Fatebenefratelli Sacco University Hospital, Milan, Italy; ^11^Unit of Infectious Diseases, San Raffaele Scientific Institute, Milan, Italy; ^12^Fondazione Policlinico Universitario Agostino Gemelli IRCCS, Rome, Italy; ^13^Department of Informatics, Bioengineering, Robotics and System Engineering (DIBRIS), University of Genoa, Genoa, Italy; ^14^Unit of Psychology, “Psicologia Aziendale”, ASL “Città di Torino”, Turin, Italy

**Background**: Recent advancements in antiretroviral therapies have resulted in substantial improvements in the life expectancy of people with HIV (PWH). However, lifelong medications, advancing age and biological ageing have been associated to a substantial burden of physical and psychological comorbidities. The early recognition of health needs ensures better personalized care and improved quality of life. In this spirit, a multidimensional screening questionnaire and an in‐depth flowchart that were developed to timely identify and address alterations. In order for the questionnaire to be employed as a tool in clinical practice, it was subjected to validation through the MUSA study.

**Methods**: The multidimensional questionnaire comprised 23 questions covering 12 domains (cognition, functional capacity, depressive and anxiety symptoms, sleep quality, quality of life, nutritional status, socioeconomic factors, stigma, comorbidities, polypharmacy, and alcohol and substance use). The MUSA study was designed as an observational, prospective, multicentre study and involved 8 clinical centres across Italy. The study's procedures entailed patients undertaking the multidimensional questionnaire (approximately 5 min) and subsequently confirmation tests for each of the 12 domains (approximately 1 h), in order to evaluate the questionnaire's performance.

**Results**: We enrolled 202 participants. Study participants were mostly male (63.4%) with a median age of 58 years and 27.2% older persons were enrolled. Dyslipidaemia and hypertension were the most prevalent comorbidities (35.1 and 30.7%, respectively); multimorbidity was observed in 20.1% of participants and polypharmacy in 16.9%. Immunovirological status was excellent with median CD4 above 700 cell/mm^3^ and virological control in the vast majority of study participants (97.5% and 5 with HIV RNA <200 copies/mL).

A significant direct correlation was observed between the total number of abnormal domains at screening and at confirmatory testing (*R* ^2^ = 0.364, *p* < 0.001): the screening identified participants with at least one altered domain at confirmation tests (sensitivity 96.3%, specificity 11.8%, accuracy 84.8%). Diagnostic accuracy varied considerably across different domains as shown in Table 1 and Figure 1.

**Conclusions**: Despite differential diagnostic accuracy, the multidimensional questionnaire has the potential to be used in clinical practice for capturing health needs of PWH beyond standard clinical visits: confirmatory tests and tailored interventions are needed for optimizing patients’ care.

**FIGURE 1:**
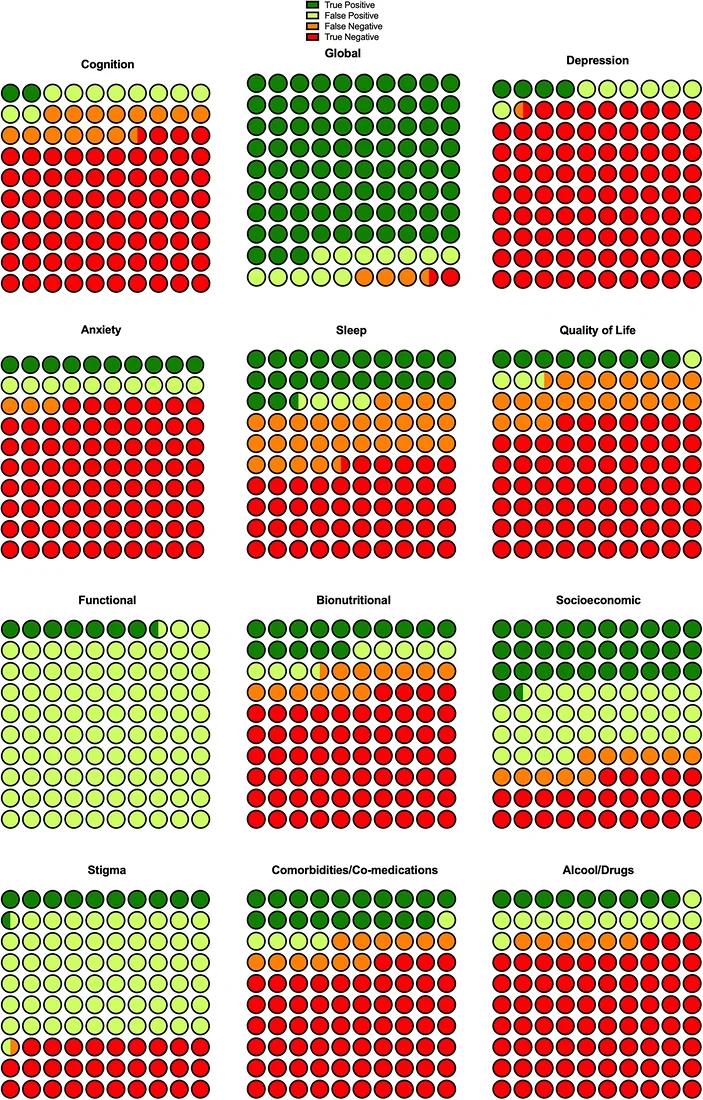
P458 | 10×10 dot plot depicting the accuracy of the screening test. Green and red circles depict the true positive and true negative results, respectively; light green and orange circles represent false positive and false negative results, respectively.

**TABLE 1:**
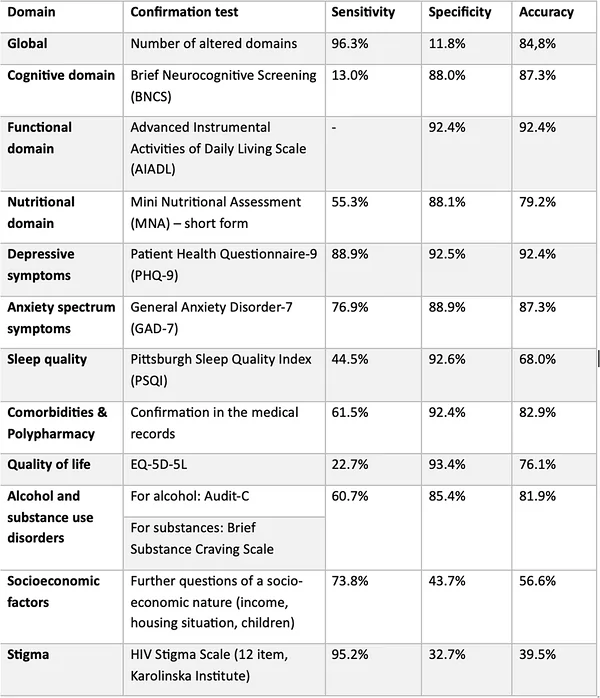
P458 | Confirmation tests, sensitivity, specificity and accuracy for each domain.

### P459 No Differential Impact on 10‐Year Cardiovascular Risk Despite Lipid Differences Between BIC/FTC/TAF and DTG/3TC: A Post‐Hoc Analysis of the MIND Trial (MIND‐CORE Study)

Sebastian Mendoza Ceacero, Diana Corona Mata, Claudia Ferreira‐Tata, Angela Camacho, Antonio Rivero‐Juarez, Antonio Rivero, Ignacio Pérez‐Valero, on behalf of MIND Study Group

Infectious Diseases, Hospital Universitario Reina Sofía, Cordoba, Spain

**Background**: People living with HIV (PLWH) with neuropsychiatric comorbidities carry a compounded cardiovascular risk (CVR). The MIND trial (GESIDA 11920), a phase 4, randomized, double‐blind, placebo‐controlled trial in PLWH with stable neuropsychiatric comorbidities, demonstrated that switching to bictegravir/emtricitabine/tenofovir alafenamide (BIC/FTC/TAF) produced significant improvements in lipid profile and atherogenic index compared to maintaining dolutegravir/lamivudine (DTG/3TC) at 48 weeks. The MIND‐CORE study is a pre‐specified post‐hoc analysis examining whether these lipid differences translated into a measurable reduction in estimated 10‐year CVR.

**Methods**: CVR was estimated using SCORE2 (ESC 2021, low‐risk region; validated for PLWH) at baseline (BL), week 24 (W24) and week 48 (W48) in the full MIND cohort (BIC/FTC/TAF *n* = 41; DTG/3TC *n* = 39). Participants with prior major cerebrovascular events (*n* = 3) were excluded. Age was incremented by 1 year for W48. Modified intention‐to‐treat (mITT; *n* = 77) and per‐protocol (PP; *n* = 63) analyses were performed. Between‐group comparisons used Mann–Whitney U or *t*‐tests; within‐group changes used Wilcoxon signed‐rank test. ANCOVA assessed treatment effect on W48 SCORE2 adjusted for baseline. REGICOR/COMVIH‐COR (Framingham recalibrated for Spanish PLWH) was used as sensitivity analysis.

**Results**: Median (IQR) age was 49 (41–55) years; 29% female; 22% current smokers. Despite significant differences in lipid profile favouring BIC/FTC/TAF, median SCORE2 did not differ between arms at BL (3.5% vs. 3.4%; *p* = 0.82), W24 or W48 (3.5% vs. 3.3%; *p* = 0.95) (Figure 1). Within‐group changes were small and non‐significant (median delta BL–W48: +0.20% BIC/FTC/TAF, +0.12% DTG/3TC). ANCOVA confirmed no treatment effect adjusting for baseline (coefficient +0.25, 95% CI: −0.26 to +0.76; *p* = 0.34). Risk category remained stable in 90% of BIC/FTC/TAF and 82% of DTG/3TC participants (*p* = 0.44). Sensitivity analysis with REGICOR/COMVIH‐COR was consistent.

**Conclusions**: In PLWH with stable neuropsychiatric comorbidities, the lipid improvements associated with switching to BIC/FTC/TAF did not translate into a statistically significant reduction in estimated 10‐year CVR compared to maintaining DTG/3TC at 48 weeks. The short follow‐up and low absolute baseline risk may have limited the detectability of a cardiovascular benefit. Longer‐term studies are needed to determine whether the metabolic advantages observed with BIC/FTC/TAF ultimately reduce hard cardiovascular endpoints in this population.

**FIGURE 1:**
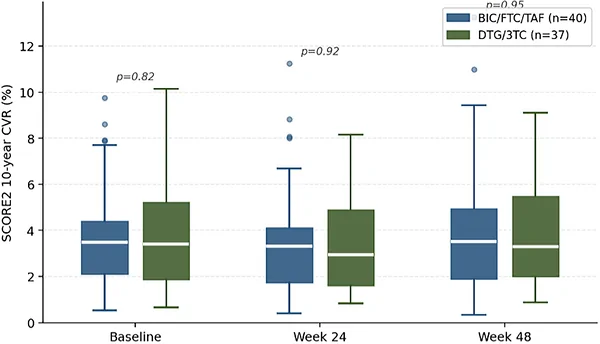
P459 | SCORE2‐estimated 10‐year CVR by treatment arm and time point (mITT, excluding prior cerebrovascular events); boxes: median and IQR; whiskers: 1.5×IQR; *p*‐values: between‐group comparisons (Mann–Whitney U).

### P460 Lost Before Discharge: Psychiatric Comorbidity and Substance Use Associated With Care Disengagement in Hospitalized Young Adults With Perinatally Acquired HIV in Argentina

Ana Martina De Prada, Javier Ricart, Laura Moreno Macias, Nicolas Nahoum, Cinthia Giomi, Matías Gastón Pérez, Dana Mijalovsky, Graciana Cárdenas, Matías Enzenhofer, Luis De Carolis, Natalia Soledad Vittar, Gustavo Bruni, María Florencia Villafañe Fioti, Emilse Vázquez, Sofía Bauer, Rubén Solari

Hospital de Infecciosas “Francisco J. Muñiz”, Buenos Aires, Argentina

**Background**: Adults with perinatally acquired HIV (PaHIV) represent a distinct and vulnerable population characterized by lifelong exposure to the virus, prolonged ART use and compounded psychosocial adversity. While clinical predictors of mortality in this group have been described, the social and behavioural determinants of care disengagement—including self‐discharge against medical advice (AMA) and failure to attend post‐discharge follow‐up—remain poorly characterized, particularly in Latin America.

**Methods**: Retrospective, cross‐sectional study of adults (≥18 years) with PaHIV hospitalized at a public reference centre in Buenos Aires, Argentina, between January 2024 and January 2026. We analysed 103 admissions corresponding to 52 unique healthcare clients. Analyses were conducted at the healthcare client level. A composite care disengagement outcome was defined as AMA discharge or failure to attend outpatient follow‐up within 6 months of discharge. Associations were assessed using Fisher's exact test.

**Results**: Median age was 26 years (IQR 24–29); 67% were cis‐gender men, 31% cis‐gender women and 2% trans‐gender women. Median CD4 count at admission was 33 cells/µL (IQR 14–87). Among 52 clients, 9 (17.3%) were discharged AMA at least once. Overall, 40% of surviving clients met the composite care disengagement outcome. Both substance use (OR 15.12; *p* = 0.003) and psychiatric comorbidity (OR 22.00; *p* = 0.003) were significantly associated with the composite care disengagement outcome. The presence of family or social support was strongly protective against AMA discharge specifically (*p* = 0.017), with no AMA discharges occurring among clients with family support (Table 1).

**Conclusions**: In hospitalized young adults with PaHIV, care disengagement was strongly associated with substance use and psychiatric comorbidity, while the presence of family or social support was protective. These findings add to the limited evidence on social determinants of care disengagement among adults with PaHIV in Latin America and support the integration of mental health and psychosocial interventions care services for this population.

**TABLE 1:**
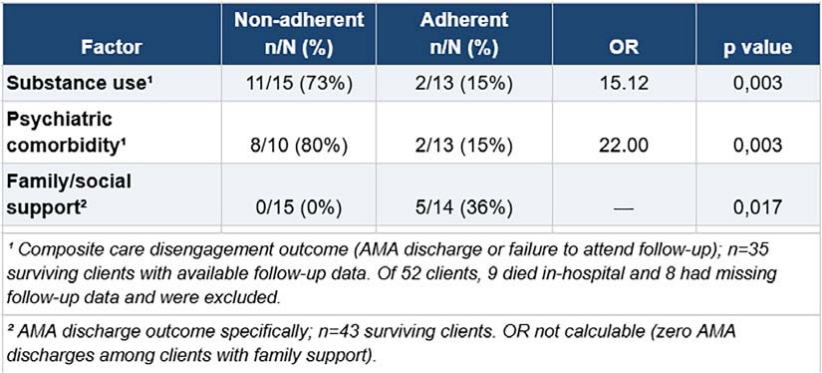
P460 | Factors associated with care disengagement outcomes among surviving clients.

## Co‐Morbidities and Complications of Disease and/or Treatment: Other

### P461 Immunomodulatory Therapies in People Living With HIV: Changing Patterns and Infectious Outcome From a Real‐World Cohort

Anne‐Claire Tournay^1^, Emmanuelle Bontemps ^1^, Agnès Meybeck^1^, Victoria Manda^2^, François Raffi^3^, Olivia Zaegel‐Faucher^4^, Manuel Etienne^5^, Solene Patrat‐Delon^6^, Jean‐Paul Viard^7^, Pierre Delobel^8^, Gwenael Le Moal^9^, Olivier Robineau^1^, Nathalie Viget^1^, on behalf of MAIVIH Group

^1^Infectious Disease Department, CH Dron, Tourcoing, France; ^2^Infectious Disease Department, Hôpital Saint‐Louis, Paris, France; ^3^Infectious Disease Department, CHU Nantes, Nantes, France; ^4^Infectious Disease Department, APHM, Marseille, France; ^5^Infectious Disease Department, CHU Rouen, Rouen, France; ^6^Infectious Disease Department, CHU Rennes, Rennes, France; ^7^Infectious Disease Department, Hôpital Hôtel Dieu, Paris, France; ^8^Infectious Disease Department, CHU Toulouse, Toulouse, France; ^9^Infectious Disease Department, CHU Poitiers, Poitiers, France

**Background**: Immunomodulatory drugs (IDs) are increasingly used to treat inflammatory and autoimmune diseases (IADs). However, data regarding their effectiveness and safety in people living with HIV (PLWH) remains scarce, particularly for newer biologic agents. We aimed to describe treatment patterns and clinical outcomes in PLWH with IADs.

**Methods**: We conducted a multicentre retrospective cohort study across 19 French referral centres. PLWH with IADs followed between June 2021 and December 2024 were included. Clinical, therapeutic and immunovirological data were retrospectively collected. HIV‐related immunovirological markers were assessed longitudinally from ID initiation until the last recorded visit. Immunovirological status, infectious events and disease control were assessed according to ID use.

**Results**: Among 200 PLWH with IADs (median age 57 years, male 59.5%), chronic inflammatory rheumatic diseases and inflammatory bowel diseases accounted for 40% and 20% of diagnoses, respectively. Overall, 103 (51.5%) PLWH received ID, mainly conventional agents (72%) and TNF inhibitors (37%). The use of non‐TNF biologic agents increased over time, rising from 4% of first‐line therapies to 17% of the last prescribed therapies (*p* < 0.01). All PLWH initiated ID while receiving antiretroviral treatment. HIV parameters remained stable throughout follow‐up, with median CD4 T cell counts increasing from 636 (IQR 434) cells/mm^3^ before ID initiation to 680 (IQR 422) cells/mm3 at the end of follow‐up (*p* = 0.07), while HIV viral load < 50 copies/mL was stable (93% and 92.5%, respectively). Complete or partial remission of IAD was achieved in 40% and 53% of patients, respectively. Infection incidence rates were 10.9, 12.0 and 42.1 per 100 patient‐years among patients receiving conventional immunomodulators, TNF inhibitors and other biologic agents, respectively. Compared with conventional immunomodulators, other biologic agents and TNF inhibitors were associated with a higher risk of infection (IRR 3.86, 95% CI: 2.10–7.10; *p* < 0.001; IRR 3.52, 95% CI: 1.82–6.79; *p* < 0.001, respectively).

**Conclusions**: In PLWH with IADs, immunomodulatory therapies achieved remission rates similar to the one observed in the general population, while maintaining HIV immune‐virological control. As the use of newer biologic agents expands, their potential association with increased infectious risk warrants particular attention and prospective confirmation.

### P462 Renal Impairment in Children and Young Adults Living With HIV in Europe and Thailand

Hannah Castro^1^, John O'Rourke^1^, Gabriela Toledo ^1^, Punnathorn Auaboonkanok^2^, Katja Doerholt^3^, Luminita Ene^4^, Tessa Goetghebuer^5^, Christian R. Kahlert^6^, Marisa Navarro^7^, Lars Naver^8,9^, Antoni Noguera‐Julian^10,11^, Magdalena Pluta^12^, Wipaporn Natalie Songtaweesin^13^, Vana Spoulou^14^, Alla Volokha^15^, Nadia Ahmed^16^, Giorgia Dalla Valle^17^, Luca Stona^17^, Cassidy Henegar^18^, Vani Vannappagari^18^, Ana Puga^18^, Charlotte Jackson^1^, Ali Judd^1,17^, Intira Jeannie Collins^1^, Siobhan Crichton^1^, on behalf of Epidemiology of Pregnancy and Paediatric Infections International Cohort Collaboration (EPPICC)

^1^UCL Innovative Clinical Trials Unit, Institute of Clinical Trials & Methodology, University College London, London, UK; ^2^Department of Pediatrics, Faculty of Medicine Khon Kaen University, Khon Kaen, Thailand; ^3^St George's University Hospitals, London, UK; ^4^“Dr. Victor Babes” Hospital for Infectious and Tropical Diseases, Bucharest, Romania; ^5^Hospital St Pierre, Université libre de Bruxelles, Brussels, Belgium; ^6^Children's Hospital of Eastern Switzerland, St. Gallen, Switzerland; ^7^Hospital General Universitario ‘Gregorio Marañón’, IISGM, UCM, CIBERINFEC, ISCIII, Madrid, Spain; ^8^Department of Pediatrics, Karolinska University Hospital, Stockholm, Sweden; ^9^Department of Clinical Science, Intervention and Technology, CLINTEC, Karolinska Institutet, Stockholm, Sweden; ^10^Malalties Infeccioses i Resposta Inflamatòria Sistèmica en Pediatria, Servei d´Infectologia, Institut de Recerca Sant Joan de Déu, and Departament de Cirurgia i Especialitats Medicoquirúrgiques, Facultat de Medicina i Ciències de la Salut, Universitat de Barcelona, Barcelona, Spain; ^11^Centro de Investigación Biomédica en Red de Epidemiología y Salud Pública, CIBERESP, Madrid, Spain; ^12^Department of Children's Infectious Diseases, Medical University of Warsaw, Warsaw, Poland; ^13^Department of Pediatrics, and School of Global Health, Faculty of Medicine, Chulalongkorn University, Chulalongkorn University, Thailand; ^14^First Dept of Paediatrics, “Agia Sofia” Children's Hospital, Athens, Greece; ^15^Department of Pediatrics, Infectious Diseases and Immunology, Shupyk National Healthcare University of Ukraine, Kyiv, Ukraine; ^16^Mortimer Market Centre, Central North West London NHS Trust, London, UK; ^17^Fondazione Penta ETS, Padova, Italy; ^18^Epidemiology and Real‐World Evidence, ViiV Healthcare, Durham, NC, USA

**Background**: The long‐term impact of HIV and antiretroviral therapy (ART) on renal health in children and young adults living with HIV (CYALHIV) is not fully understood.

**Methods**: We aimed to assess renal impairment (RI) in CYALHIV who had initiated ART and were in follow‐up aged < 18 years at any point between 01/01/2015 and 31/12/2024, across 13 cohorts in the Epidemiology of Pregnancy and Paediatric Infections International Cohort Collaboration (EPPICC). RI was defined as two consecutive grade ≥2 (DAIDS) serum creatinine or age‐specific estimated glomerular filtration rate measurements; resolution was two consecutive normal measurements. We estimated (i) cumulative prevalence of RI; (ii) duration of RI episodes; (iii) incidence of first RI from 01/01/2015, overall and by time‐updated characteristics; (iv) characteristics at onset of incident RI.

**Results**: Overall, 1618 CYALHIV were included; 797 (49%) male, 1350 (83%) had perinatally acquired HIV. Median (IQR) age at ART initiation was 3.1 (0.6, 9.5) and at censoring 17.8 (14.3, 19.6) years (239 [15%] aged >21 years). There were 725 RI episodes among 617 CYALHIV; overall RI prevalence (95% CI) was 38% (36%,41%). Of these, 382 (53%) episodes resolved after a median (IQR) of 1.8 (0.9, 3.0) years. Among 1490 CYALHIV included in the incidence analysis, median (IQR) follow‐up was 3.6 (1.9, 5.9) years. Overall RI incidence (95% CI) was 7.3 (6.6, 8.0) episodes per 100 person‐years. RI incidence generally declined from 2017 (Figure 1). Incidence was highest in Central/Eastern Europe (9.6 [8.0, 11.5]) and among those aged 12– < 18 years (12.1 [10.8, 13.4]). Across anchor drug classes, highest incidence was observed among those on integrase inhibitor (INSTI) based regimens; there was limited difference by nucleoside reverse transcriptase inhibitor (NRTI) backbone (Figure 1). At RI onset (*n* = 454), median (IQR] time from ART initiation was 9.0 (4.9, 13.4) years, 41% were on INSTI‐based regimens and 42% on a NRTI backbone of abacavir with lamivudine or emtricitabine (Table 1). Most (93%) had no/mild immunosuppression‐for‐age, although 54% had prior advanced/severe immunosuppression and 24% had a prior AIDS‐defining illness.

**Conclusions**: RI was common; over one‐third of CYALHIV experienced RI, with episodes persisting an average of 2 years. RI incidence was highest among adolescents and occurred largely in those with good immune health. High incidence on INSTIs may reflect inhibition of tubular secretion of creatinine rather than pathological RI.

**FIGURE 1:**
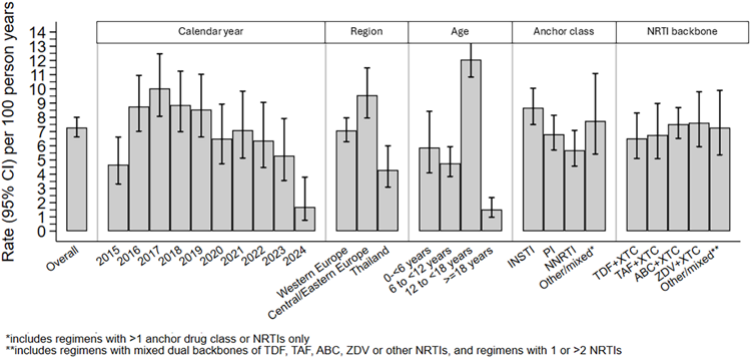
P462 | Incidence of first renal impairment (since 1 January 2015), overall and by time‐updated characteristics.

**TABLE 1:**
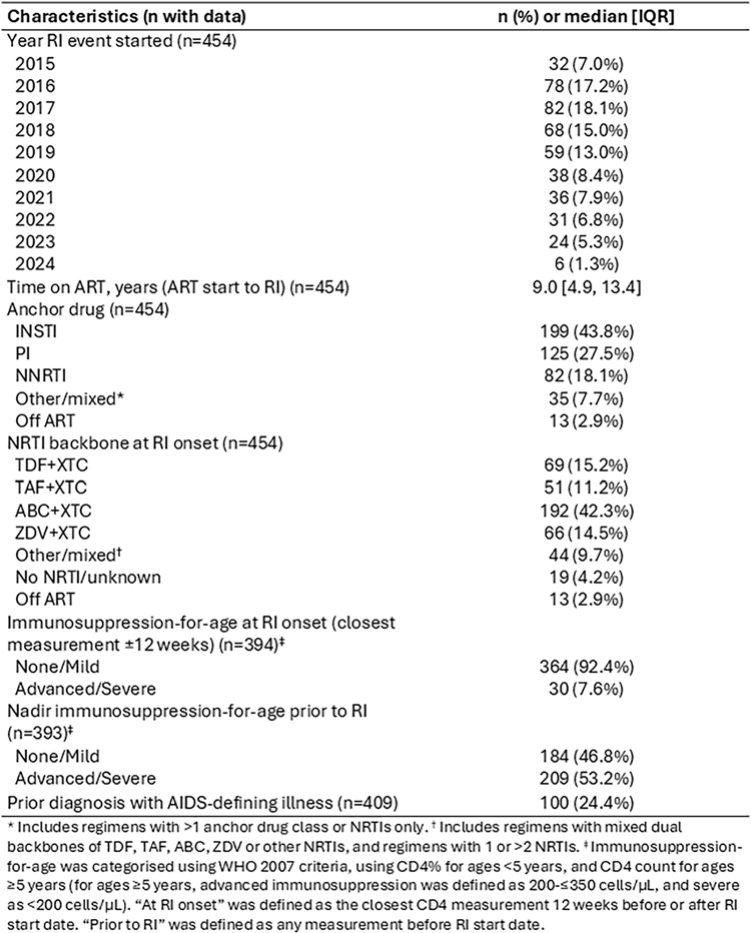
P462 | Characteristics of the 454 children and young adults living with HIV who experienced renal impairment (first event since 1 January 2015).

### P463 Efficacy and Safety of Darunavir/Cobicistat Plus Lamivudine Compared With Darunavir/Cobicistat Plus Tenofovir Alafenamide/Emtricitabine in Virologically Suppressed People Living With HIV: Less Is More? A Pilot Study (TLALOC‐2 Trial)

Sandra Patricia Ramírez Eguía, José Antonio Mata Marín, Ana Luz Cano Díaz, Cruzwaldo Campos Cano, Emily Itzel Pecero García, Ericka Nelly Pompa Mera, Alberto Chaparro Sánchez, Jesús Enrique Gaytán Martínez

Infectious Diseases Department, CMN La Raza, Mexico City, Mexico

**Background**: Dual antiretroviral therapy (2DR) strategies aim to maintain virological suppression while mitigating the long‐term cumulative toxicities and metabolic complications associated with triple‐drug regimens. This phase 4 pilot study evaluated the 24‐week efficacy, safety and patient‐reported outcomes of simplifying to darunavir/cobicistat plus lamivudine (DRV/c+3TC) compared with switching to darunavir/cobicistat plus tenofovir alafenamide/emtricitabine (DRV/c+TAF/FTC) in virologically suppressed men living with HIV‐1 in Mexico.

**Methods**: A single‐centre, prospective, open‐label, non‐randomized trial was conducted from March 2025 to March 2026. Adult males with viral suppression (HIV‐1 RNA <50 copies/mL) for ≥48 weeks on DRV/c+3TC or DRV/c+TDF/FTC (TLALOC‐1 trial) and an eGFR ≥60 mL/min/1.73 m^2^ were eligible. Primary endpoints were virological efficacy (FDA Snapshot algorithm) and safety at 24 weeks. Secondary outcomes included body composition (bioimpedance), renal biomarkers and mental health scores.

**Results**: Of 81 screened participants, 78 completed the 24‐week follow‐up: 36 continued DRV/c+3TC and 42 switched to DRV/c+TAF/FTC. At week 24, no confirmed virological failures occurred in either arm. By FDA Snapshot analysis, the proportion with HIV‐1 RNA ≥50 copies/mL was 5.6% (2/36) in the dual arm versus 0% (0/42) in the triple arm (*p* = 0.220). Mean CD4+ cells counts remained stable (728 ± 333 vs. 734 ± 207 cells/mm^3^; *p* = 0.948). At week 24, total cholesterol (185 ± 41 vs. 195 ± 32 mg/dL; *p* = 0.427), triglycerides 160 mg/dL (IQR 112–207) versus 165 mg/dL (IQR 101–208; *p* = 0.905), body fat (24.2% ± 9.2 vs. 24.0% ± 7.1; *p* = 0.360) and BMI (25.5 ± 4.7 vs. 27.2 ± 4.4 kg/m^2^; *p* = 0.082) remained statistically comparable between the dual and triple therapy arms, respectively. Drug‐related adverse events were infrequent and entirely grade 1 (5.7% [2/35] in dual therapy vs. 7.7% [3/39] in triple therapy; *p* = 0.504). No significant between‐group differences were observed in cystatin C‐based eGFR or fractional excretion of phosphorus and uric acid. Adherence and patient‐reported outcomes were comparable between arms (Figure 1 and Table 1).

**Conclusions**: Both DRV/c+3TC and DRV/c+TAF/FTC regimens demonstrated excellent virological efficacy, safety and high treatment satisfaction at 24 weeks. These findings support dual therapy with DRV/c+3TC as a safe, well‐tolerated maintenance alternative for regimen simplification in Latin American population.

**FIGURE 1:**
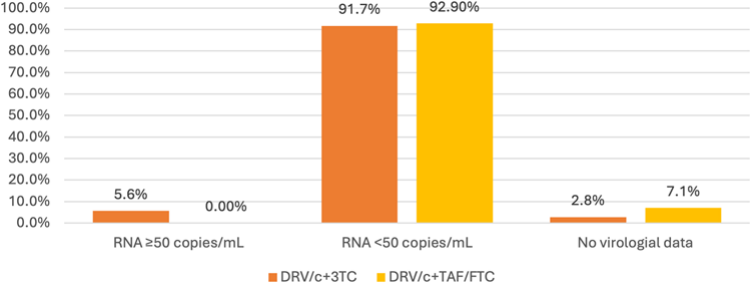
P463 | Efficacy of DRV/c+3RC compared to DRV/c+TAF/FTC at 24 weeks.

**TABLE 1:**
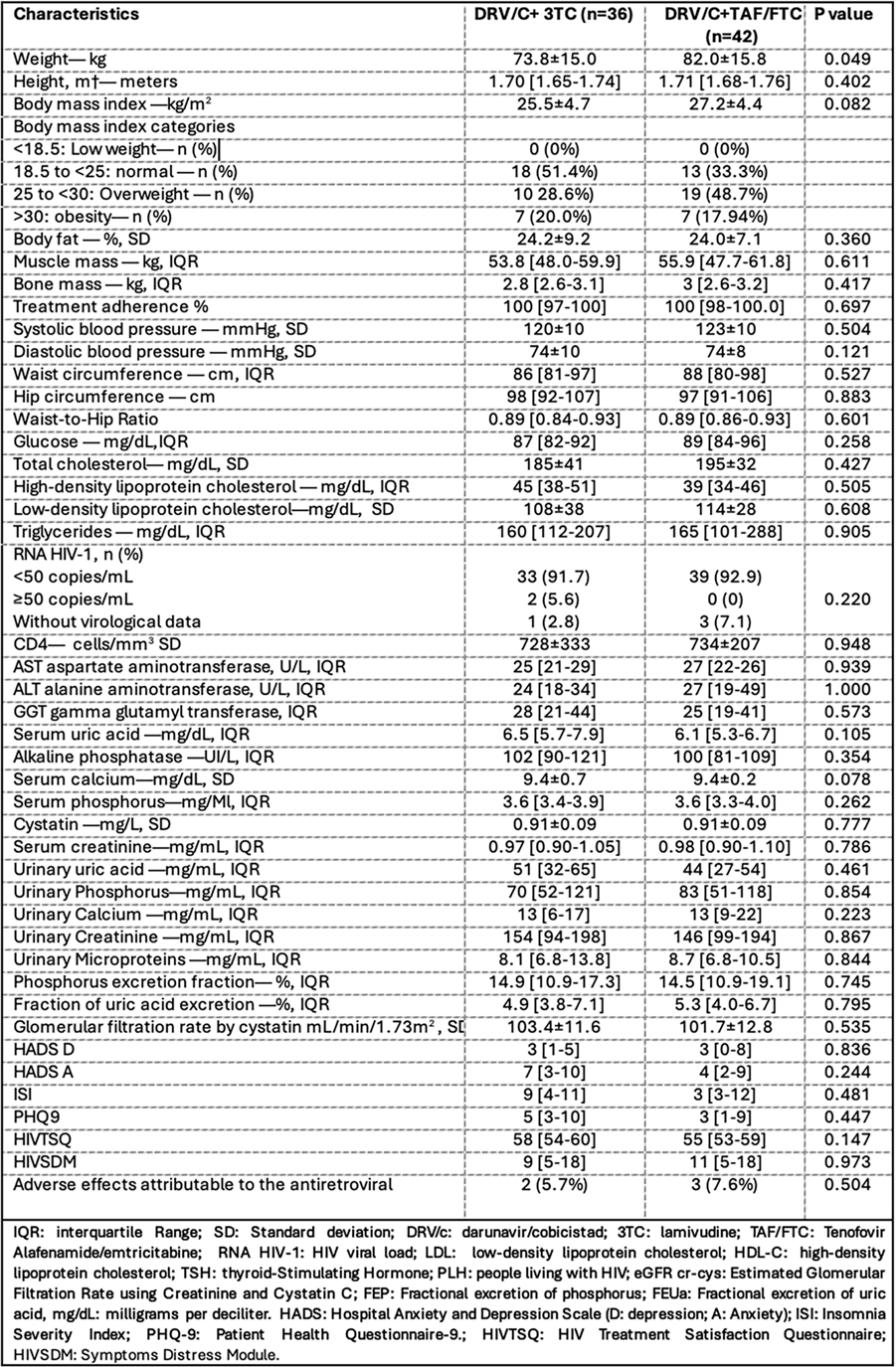
P463 | Clinical, metabolic, virologic and satisfaction results at 24 weeks of DRV/c+3TC compared DRV/c+TAF/FTC (*n* = 74).

### P464 Persistent Low‐Level Viraemia Is Not Associated With Increased Risk of Serious Clinical Outcomes in the Contemporary INSTI Era: A Time‐Updated Analysis

Giovanni Festa ^1^, Riccardo Patanè^1^, Nicolò Capra^2^, Riccardo Lolatto^2^, Gabriele Loi^1^, Golnaz Torkjazi^1^, Gaia Catalano^1^, Hamid Hasson^2^, Silvia Nozza^1,2^, Antonella Castagna^1,2^, Vincenzo Spagnuolo^1,2^, Tommaso Clemente^2^

^1^Vita‐Salute San Raffaele University, Milan, Italy; ^2^Infectious Diseases, IRCCS San Raffaele Scientific Institute, Milan, Italy

**Background**: The clinical significance of persistent low‐level viraemia (pLLV) remains uncertain in the contemporary integrase strand transfer inhibitor (INSTI) era. We aimed to investigate the association between pLLV and the risk of serious clinical events or death among people living with HIV receiving contemporary antiretroviral therapy (ART).

**Methods**: Cohort study on individuals initiating ART between 2008 and 2024 with ≥3 viral load (VL) measurements after ≥1 year of ART. Participants were classified as virological suppression (VS, < 50 copies/mL), pLLV (≥2 consecutive VLs 50–199 copies/mL), or virological failure (VF, ≥2 consecutive VLs ≥200 copies/mL or ≥1 VL ≥1000 copies/mL). VL was modelled as a time‐updated exposure; participants were reclassified during follow‐up with transitions allowed only towards higher virological strata. Follow‐up accrued from the first VL after 1 year of ART until the first serious clinical event, death or last visit. The primary endpoint was the first serious clinical event (major adverse cardiovascular events, non‐AIDS‐defining cancers or AIDS‐defining events) or death. Cox regression estimated associations between VL strata and outcomes. Cause‐specific Cox models assessed non‐AIDS‐defining and AIDS‐defining events and death separately.

**Results**: We included 2358 people: 1789 (75.8%) had stable VS, 263 (11.1%) experienced ≥1 episodes of pLLV and 306 (12.9%) VF (Figure 1). Participants were predominantly male‐at‐birth (88.8%) with median age of 40.0 years (interquartile range 33.0–47.4) and median CD4^+^ count of 612 cells/µL (437–828). Overall, 844 (35.8%) were receiving INSTI‐based triple therapy at baseline. During a median follow‐up of 8.8 years (5.4–12.1), 185 participants experienced a serious clinical event or died: 128/1789 (7.2%) on VS, 15/263 (5.7%) with pLLV and 42/306 (13.7%) with VF. Compared with VS, pLLV was not associated with an increased risk of the primary endpoint, whereas VF was associated with ≥2‐fold higher risk. Results were consistent for serious non‐AIDS‐defining outcomes. For AIDS‐defining events or death, risk was non‐significantly higher with pLLV and markedly higher with VF (Table 1).

**Conclusions**: In our contemporary cohort, pLLV was not associated with an increased risk of serious clinical outcomes, particularly non‐AIDS‐defining events. These findings suggest that pLLV may have limited prognostic significance in the INSTI era.

**FIGURE 1:**
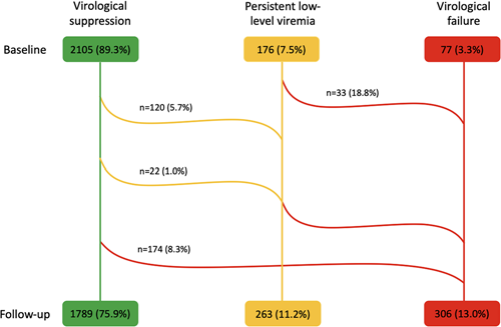
P464 | Baseline distribution and follow‐up reclassification of people with HIV by highest viral load stratum achieved.

**TABLE 1:**
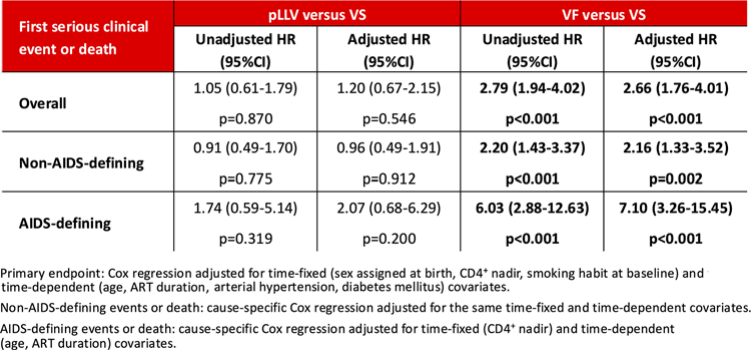
P464 | Univariable and multivariable Cox analyses of first serious clinical event or death by viral load strata.

### P465 Long‐Term Trends in Serious Non‐AIDS Events Following ART Initiation in the ICONA Cohort, 1997–2026

Adriana Cervo ^1^, Andrea Giacomelli^2^, Alessandra Bandera^3^, Rosaria Viglietti^4^, Federica Casari^1^, Sarah Dal Zoppo^5^, Silvia Nozza^6^, Andrea Gori^7^, Antonella Cingolani^8^, Enrico Girardi^9^, Antonio Di Biagio^10^, Antonella D'Arminio Monforte^11^, Giovanni Guaraldi^1^, Alessandro Cozzi‐Lepri^12^, on behalf of ICONA Cohort Study Group

^1^Department of Infectious Diseases, Azienda Ospedaliero‐Universitaria di Modena, Policlinico di Modena, University of Modena and Reggio Emilia, Modena, Italy; ^2^III Infectious Diseases Unit, ASST Fatebenefratelli‐Sacco, DIBIC Luigi Sacco, Università degli Studi di Milano, Milan, Italy; ^3^Clinic of Infectious Diseases, Fondazione IRCCS Ca' Granda Ospedale Maggiore Policlinico, Department of Pathophysiology and Transplantation, University of Milan, Milan, Italy; ^4^P.O. “D. Cotugno”, Azienda Ospedaliera dei Colli, Naples, Italy; ^5^Unit of Infectious Diseases – ASST Cremona, Cremona, Italy; ^6^Department of Infectious Diseases, IRCCS San Raffaele Scientific Institute, Vita‐Salute San Raffaele University, Milan, Italy; ^7^II Infectious Diseases Unit, ASST Fatebenefratelli Sacco, Department of Biomedical and Clinical Sciences L. Sacco, Centre for Multidisciplinary Research in Health Science (MACH), University of Milan, Milan, Italy; ^8^UOC Malattie Infettive, Fondazione Policlinico Universitario Agostino Gemelli IRCCS, Dipartimento di Sicurezza e Bioetica, Università Cattolica del Sacro Cuore, Rome, Italy; ^9^Scientific Direction, National Institute for Infectious Diseases, Lazzaro Spallanzani IRCCS, Rome, Italy; ^10^Infectious Disease Clinic, IRCCS Ospedale Policlinico San Martino, Department of Health Science (DISSAL), University of Genova, Genova, Italy; ^11^ICONA Foundation, Milan, Italy; ^12^Centre for Clinical Research, Epidemiology, Modelling and Evaluation (CREME), Institute for Global Health, UCL, London, UK

**Background**: Long‐term estimates of serious non‐AIDS event (SNAE) incidence after ART initiation by calendar period in people with HIV (PWH) remain limited.

**Methods**: We conducted a longitudinal analysis of PWH in the ICONA cohort who initiated ART between 1997 and 2026. Participants were grouped by calendar period of ART initiation (1997–2003, 2004–2010, 2011–2016, 2017–2026); characteristics were compared across periods. Outcomes included cardiovascular disease (CVD), non‐AIDS malignancies (NAM), severe renal disease (SRD) or death and SNAE (composite of CVD, NAM, SRD, end‐stage liver disease or death). Cumulative‐SNAE was defined as the accumulation of ≥2 SNAE outcomes over time (including death). Incidence was assessed using Kaplan–Meier methods and Cox proportional hazards models with calendar period as a fixed exposure, adjusting for demographic, HIV‐related, clinical and behavioural/socioeconomic factors. Effect modification by age and sex‐at‐birth was evaluated. Further analyses considering death as competing risk were performed.

**Results**: Among 20,387 participants (21% females; age 39 years [IQR 32–48]), PWH initiating ART after 2017 were older, had higher baseline HIV‐RNA and shorter time from HIV diagnosis to ART start. Overall, 7.8% (95% CI: 7.4–8.3) and 23.0% (95% CI: 21.6–24.4) of PWH developed SNAE by 7 and 20 years from ART start, respectively. The risk of CVD, NAM, SNAE and cumulative‐SNAE declined with more recent ART initiation, while the risk of SRD appeared to plateau after 2011. Compared with 1997–2003, PWH initiating ART after 2017 had the greatest risk reductions across outcomes, ranging from 54% (NAM/death) to 62% (CVD/death, Table 1). However, women who initiated ART more recently showed a less pronounced reduction in cumulative‐SNAE risk compared to men (*p*‐value for interaction 0.0007, Figure 1a,b). For SNAE, benefit was attenuated among those initiating ART aged 50+ (*p*‐value 0.03, Figure 1c,f). Results were similar in a competing risk analysis using morbidity alone as endpoint and after excluding participants initiating ART in 2025–2026.

**Conclusions**: SNAE incidence after ART has decreased substantially in more recent ART eras in a linear fashion. These findings support tailored preventive strategies, with particular attention to women and older PWH, ensuring that gains achieved across ART eras translate into equitable healthy ageing outcomes.

**FIGURE 1:**
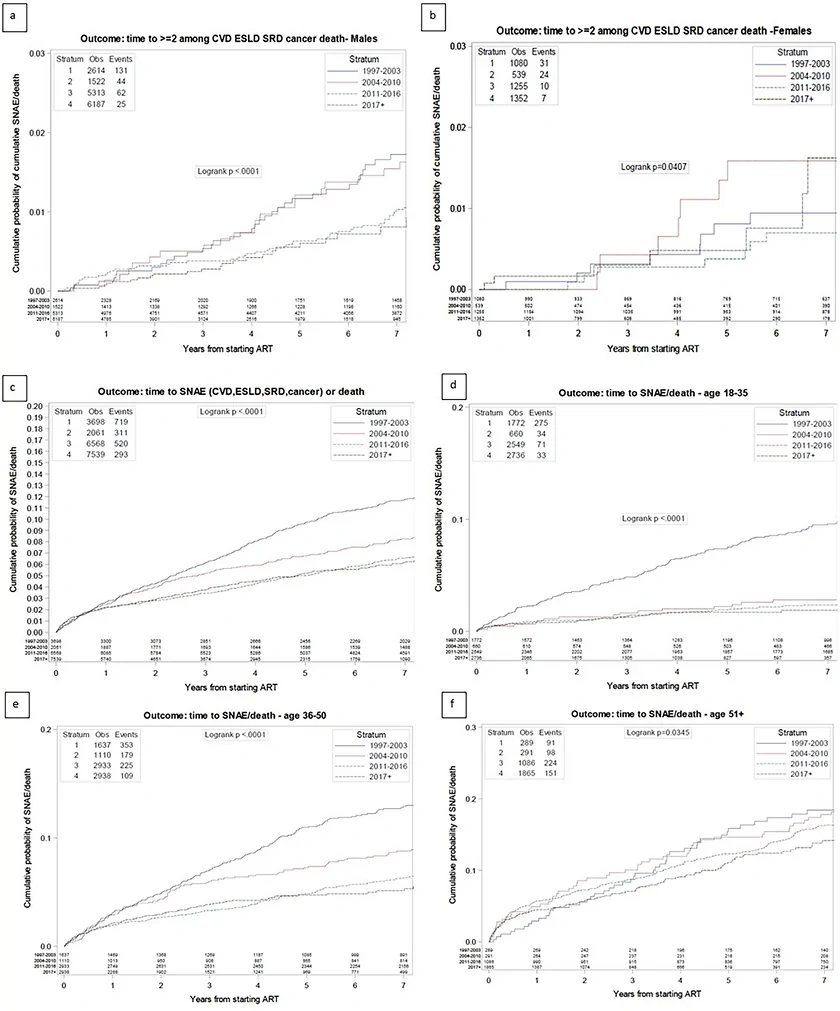
P465 | Time to ≥2 SNAE accumulation or death in males (a) and females (b) and the time to SNAE or death overall (c) and according to age (d−f).

**TABLE 1:**
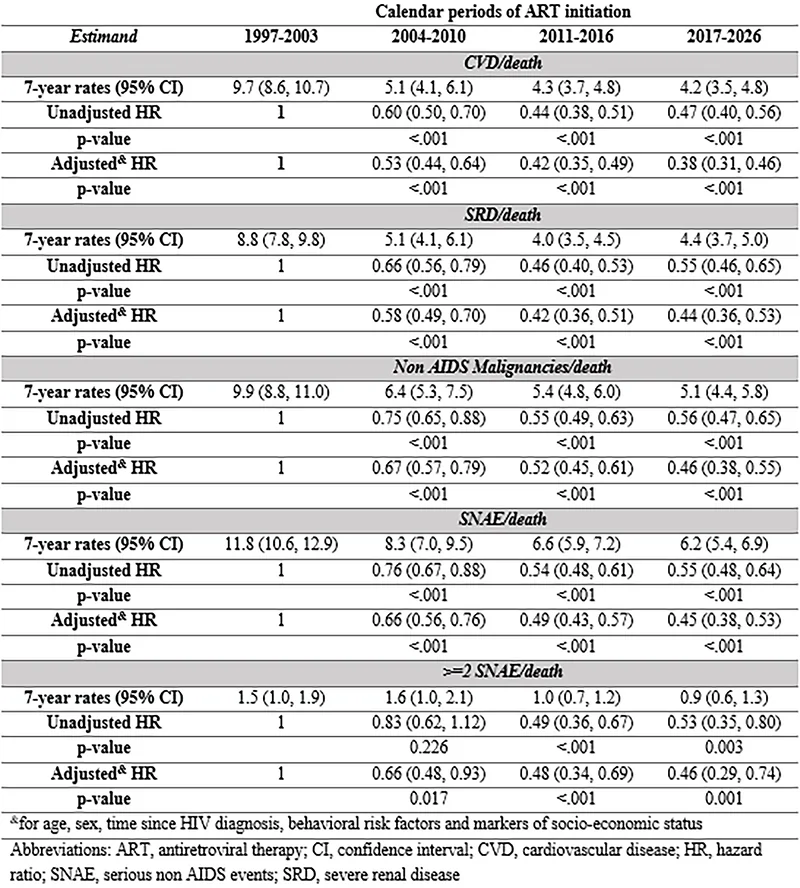
P465 | Incidence rates, unadjusted and adjusted hazard ratios of developing main non‐AIDS related comorbidities in people with HIV after ART initiation.

### P466 Comparative Performance of FRAX and FRAXPlus in Predicting Fracture Risk and Guiding Osteoporosis Management in People Living With HIV

Giulia Moi ^1,2^, Dela Wordi^1^, Andrea De Vito^2^, Maria Mazzitelli^3,4^, Mattia Trunfio^5^, Aparna Chaganti^6^, Hana Soetanto^6^, Alice Reeve^6^, Shreeyaa Mishra^6^, Jasmini Alagaratnam^1,6^, Marta Boffito^1,6^

^1^Chelsea and Westminster Hospital Foundation Trust, London, UK; ^2^Unit of Infectious Disease, Department of Medicine, Surgery and Pharmacy, University of Sassari, Sassari, Italy; ^3^Dipartimento di Sicurezza e Bioetica ‐ Sezione di Malattie Infettive, Università Cattolica del Sacro Cuore, Rome, Italy; ^4^Department of Medical and Surgical Sciences, Fondazione Policlinico Gemelli, Rome, Italy; ^5^Infectious Diseases, University of Turin, Turin, Italy; ^6^Imperial College London, London, UK

**Background**: In people with HIV (PWH), accurate bone health assessment is essential to optimize care. Fracture risk assessment (FRAX) may underestimate risk in individuals with lower spinal bone mineral density (BMD) compared to the hip but the new FRAXPlus addresses some of these limitations. We aimed to compare FRAX and FRAXPlus for risk stratification and treatment guidance in PWH.

**Methods**: PWH aged ≥40 years attending Chelsea and Westminster Hospital who underwent a dual‐energy X‐ray absorptiometry (DXA) between March 2009 and October 2025 were included in the analysis. Fracture risk was categorized according to National Osteoporosis Guideline Group (NOGG) using FRAX and FRAXPlus adjusted for hip and spine BMD, respectively. Factors associated with reclassification were assessed using logistic generalized estimating equations accounting for repeated assessments within individuals. Multivariable models included clinically relevant covariates associated with outcomes in univariable analyses, excluding variables directly involved in FRAX calculation to avoid collinearity.

**Results**: We included 1917 DXA assessments from 899 PWH, median age 54 years (IQR 52–59); 823 (90.7%) were male and 737 (81.3%) MSM. Median time since HIV diagnosis was 15.9 years (IQR 10.2–21.7) and 94.6% had HIV‐RNA <50 copies/mL. For major osteoporotic fracture risk, FRAXPlus reclassified 8/1888 (0.42%) assessments from low to high‐risk and 1/22 (4.55%) from high to very high risk. For hip fracture risk, 24/1664 (1.44%) low‐risk assessments moved to high‐risk and 13/150 (8.67%) high‐risk to very high‐risk. In multivariable analyses, upward reclassification was associated with age (OR 0.54, 95% CI: 0.38–0.76), BMI (OR 0.45, 95% CI: 0.28–0.73), vitamin D (OR 0.9, 95% CI: 0.81–0.99), smoking (OR 2.92, 95% CI: 1.48–5.75) and normal eGFR (OR 2.07, 95% CI: 1.03–4.16) (Table 1). The adjusted probability of any upward reclassification decreased progressively with age, from 8.3% at 45 years to 1.5% at 60 years. Age showed moderate ability to discriminate assessments undergoing upward reclassification (cluster‐bootstrap AUROC 0.717, 95% CI: 0.639–0.796). An age threshold of < 60 years identified 84.4% of upward reclassifications, although specificity was limited (40.9%) (Figure 1).

**Conclusions**: FRAXPlus modestly modified fracture risk categorization in PWH. Age was consistently associated with upward reclassification across models. FRAXPlus may improve risk stratification, versus using FRAX alone especially for individuals aged < 60 years, and support individualized fracture prevention strategies in PWH.

**FIGURE 1:**
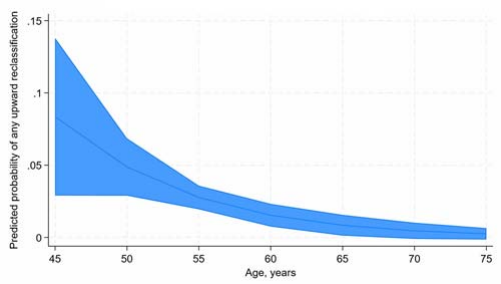
P466 | Any FRAXPlus upward reclassification by age.

**TABLE 1:**
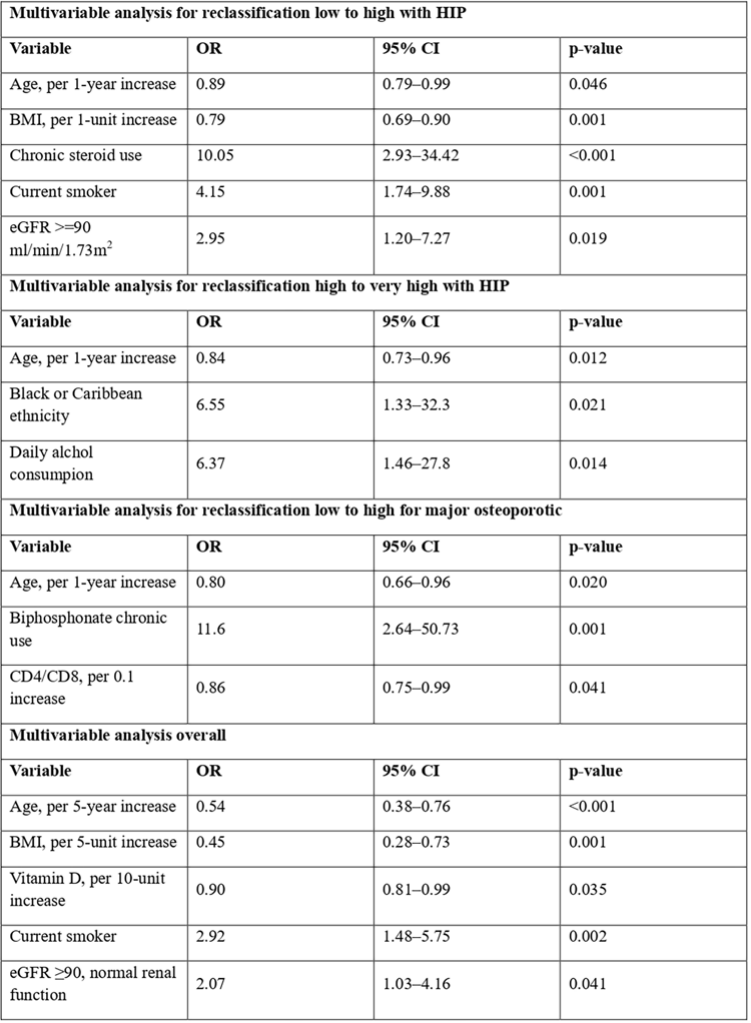
P466 | Multivariable analysis.

### P467 Obstructive Lung Disease and HIV: A High‐Risk Combination for Major Clinical Outcomes

Andreas Ronit ^1,2^, Magnus Glindvad Ahlström^3^, Josefine Amalie Loft^1^, Andreas Dehlbæk Knudsen^1^, Nicoline Arentoft^1^, Thomas L. Benfield^4^, Ken M. Kunisaki^5^, Børge Nordestgaard^6,7^, Shoaib Afzal^6,7^, Susanne Dam Nielsen^1^

^1^Viro‐immunology Research Unit, Department of Infectious Diseases 8632, University Hospital of Copenhagen ‐ Rigshospitalet, Copenhagen, Denmark; ^2^Department of Infectious Diseases 144, University Hospital of Copenhagen ‐ Amager Hvidovre Hospital, Hvidovre, Denmark; ^3^Department of Clinical Microbiology, Copenhagen University Hospital ‐ Rigshospitalet, Copenhagen, Denmark; ^4^Department of Infectious Diseases, Copenhagen University Hospital ‐ Amager and Hvidovre, Hvidovre, Denmark; ^5^Boise Veterans Affairs (VA) Medical Center, Boise, ID, USA; ^6^Department of Clinical Biochemistry, University Hospital of Copenhagen ‐ Herlev and Gentofte Hospital, Herlev, Denmark; ^7^The Copenhagen General Population Study, University Hospital Copenhagen ‐ Herlev and Gentofte Hospital, Herlev, Denmark

**Background**: Obstructive lung disease (OLD) is more prevalent among people with HIV (PWH) than in uninfected controls, but its prognostic significance and association with long‐term outcomes remain uncertain.

**Methods**: We analysed spirometry data from the Copenhagen Comorbidity in HIV Infection Study and the Copenhagen General Population Study to assess the risk of major clinical endpoints according to OLD, defined primarily as an FEV₁/FVC ratio < 0.7. Controls were matched to PWH based on age and sex. The primary composite outcome was all‐cause mortality and hospitalization, with additional analyses of respiratory‐specific mortality and hospitalizations, as well as major adverse cardiovascular events (MACEs) defined by cardiovascular death, myocardial infarction and stroke. Cox regression models were used to estimate adjusted hazard ratios (aHRs) while adjusting for potential confounders, including smoking.

**Results**: A total of 1022 PWH and 10,368 controls with spirometry were included, with a total of 2522 primary events over 72,100 person years. PWH with OLD had an aHR of 1.98 (95% CI: 1.61–2.43) for the primary composite outcome, while controls with OLD had an aHR of 1.13 (95% CI: 1.00–1.29), both compared to controls without OLD. Similar associations were observed for respiratory events and MACE. There was no evidence of interaction between HIV status and OLD for any of the outcomes (likelihood ratio test *p* > 0.45).

**Conclusions**: OLD is a risk factor for respiratory and non‐respiratory clinical events in PWH, underscoring its importance for long‐term risk assessment and management in this population.

### P468 Ten Years on: How Has Incorporation of Health‐Related Quality of Life (HRQoL) in HIV Research Changed Since the Proposal of the “4^th^ 90” Target?

Garry Brough ^1,2^, Fernanda Duarte‐Walker^3^, José M. Gatell^4^, Benjamin Young^5^, Charles Hicks^6,7^, Brent Allan^8,9^, Erika Castellanos^10^, Florence Riako Anam^11^, Richard Harding^12^, Nneka Nwokolo^13^, Ian Jacob^14^, Antonio Antunes^3^, Felipe Rogatto^15^, Ama Appiah^16^, Chinyere Okoli^3^

^1^Fast‐Track Cities London, Transformation Partners in Health & Care, London, UK; ^2^UK CAB, Positively UK, London, UK; ^3^Europe and International Medical Affairs, ViiV Healthcare, London, UK; ^4^Honorary Professor of Medicine, University of Barcelona Medical School, Barcelona, Spain; ^5^Independent Consultant, Guanajuato, Mexico; ^6^Medical Department, University of California, San Diego, CA, USA; ^7^Global Medical Affairs, ViiV Healthcare, Durham, NC, USA; ^8^International Department, National Association of People Living with HIV in Australia, Sydney, New South Wales, Australia; ^9^International Department, ASHM – Health, Sydney, New South Wales, Australia; ^10^Executive Director, Global Action for Trans Equality, New York, NY, USA; ^11^Co‐executive Director, GNP+, Kisumu, Kenya; ^12^Florence Nightingale Faculty of Nursing, Midwifery & Palliative Care, King's College London, London, UK; ^13^St Stephen's Centre, Chelsea and Westminster Hospital, London, UK; ^14^Global Health Outcomes, ViiV Healthcare, London, UK; ^15^Alliance Europe Medical Affairs, ViiV Healthcare, London, UK; ^16^Global Patient Engagement, ViiV Healthcare, London, UK

**Background**: Following UNAIDS’ adoption of the “90‐90‐90” targets, the “4^th^ 90” target (2016) aimed to extend outcomes of focus beyond viral suppression to include HRQoL [1]. When the UNAIDS targets were updated to “95‐95‐95,” the 4^th^ 90 was not incorporated, and translation of HRQoL in clinical practice remains unclear. We examined how integration of HRQoL and related concepts have evolved in literature and public discourse as an indicator of progress towards a broader definition of ending the HIV epidemic.

**Methods**: We conducted a pragmatic literature review of congress and journal article abstracts via PubMed, Embase and selected congresses between 01 June 2014 and 31 May 2016 (“Period 1,” before proposed 4^th^ 90) and 01 May 2024 and 30 April 2026 (“Period 2”). Entries were screened for the following inclusion criteria: study population of people with HIV; any interventional or observational study assessing HRQoL using qualitative or quantitative methods; and mentions HRQoL or constituent constructs (physical, mental, social, energy‐related, treatment‐related, general). To assess public discourse, we searched the r/hivaids subreddit via the Talkwalker platform for posts mentioning QoL/HRQoL, constituent constructs and/or HRQoL instruments from 01 January 2025 to 04 June 2026. International HIV guidelines were also investigated [2–5].

**Results**: Following screening, 707 abstracts were included in the literature analysis. Reporting of HRQoL‐related studies increased from Period 1 to Period 2 (Table 1), with most HRQoL‐related studies non‐industry supported (Period 1: *n* = 172/179 [96%]; Period 2: *n* = 489/528 [93%]). Use of validated HIV‐specific and generic tools also increased from Period 1 to Period 2, with the HIV Treatment Satisfaction Questionnaire, WHO QoL‐HIV Brief Questionnaire and HIV Stigma Scale being the most frequently utilized HIV‐specific tools in recent literature (Figure 1). The pre‐defined subreddit search identified 4303 posts in 2025/26 referring to HRQoL or associated terms. However, reference to HRQoL in current HIV clinical guidelines was limited.

**Conclusions**: HRQoL has become increasingly prominent in research and community discussions, demonstrating its importance in modern HIV care. However, as a subjective, multidimensional outcome, and with limited incorporation in guidelines, the definition and application of HRQoL is highly variable. It is essential that this growing research and public interest in HRQoL is translated into routine care for effective person‐centred care for people with HIV.

**References**

1. Lazarus J, Safreed‐Harmon K, Barton S, et al. Beyond viral suppression of HIV ‐ The new quality of life frontier. BMC Med. 2016;14(1):94.

2. US Department of Health & Human Services. Clinical Guidelines. Available at: https://clinicalinfo.hiv.gov/en/guidelines. Accessed 05/06/2026.

3. European AIDS Clinical Society. EACS Guidelines. Available at: https://eacs.sanfordguide.com/. Accessed 05/06/2026.

4. International Antiviral Society‐USA. Guidelines and Recommendations. Available at: https://www.iasusa.org/fellow‐student‐resources/guidelines/. Accessed 05/06/2026.

5. World Health Organization. Consolidated guidelines on HIV prevention, testing, treatment, service delivery and monitoring: Recommendations for a public health approach. Available at: https://www.who.int/publications/i/item/9789240031593. Accessed 11/06/2026.

**FIGURE 1:**
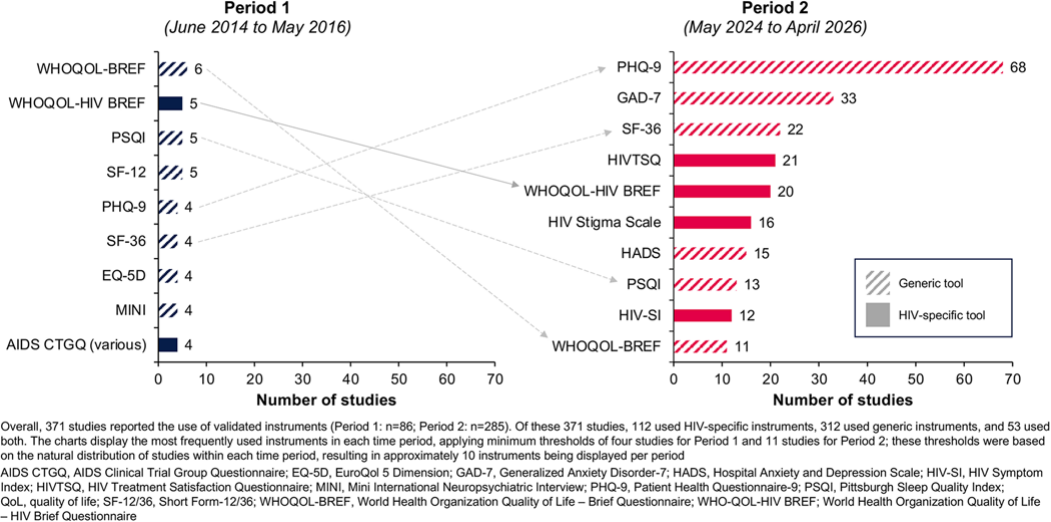
P468 | Most frequently used QoL instruments reported in literature in Period 1 (June 2014 to May 2016) versus Period 2 (May 2024 to April 2026).

**TABLE 1:**
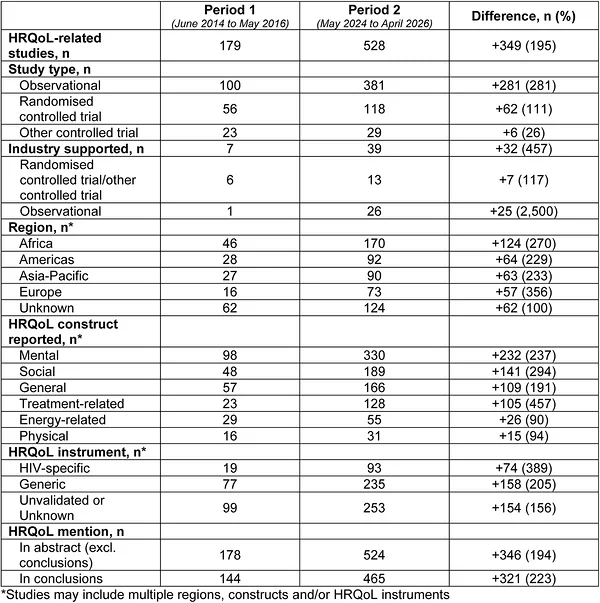
P468 | Analysis of HRQoL‐related studies reported in literature from Period 1 (June 2014 to May 2016) versus Period 2 (May 2024 to April 2026).

### P469 Abstract Withdrawn

### P470 Abstract Withdrawn

### P471 Causes of Death Among People Living With HIV in Lesotho: A Descriptive Analysis of Mortality Data From the VITAL and SaDAPT Studies

Fabian Raeber ^1,2^, Mokhebe Moleboheng^3^, Malebanye Lerotholi^1,2^, Lipontso Motaboli^3^, Blaise Lukau^3^, Palesa Mahlatsi^1,2^, Felix Gerber^1,2^, Nthuseng Bridgett Marake^4^, Tarumbiswa Tapiwa^4^, Irene Ayakaka^3^, Nadine Tschumi^1,2^, Niklaus D. Labhardt^1,2^

^1^Division of Clinical Epidemiology, Department of Clinical Research, University Hospital Basel, Basel, Switzerland; ^2^University of Basel, Basel, Switzerland; ^3^SolidarMed Partnerships for Health, Maseru, Lesotho; ^4^Ministry of Health Lesotho, Maseru, Lesotho

**Background**: Mortality patterns among people living with HIV have substantially changed in the era of antiretroviral therapy (ART) [1]. Newer data on causes of death among people living with HIV in Southern Africa remain, however, limited. We assessed mortality causes in two cohorts in Lesotho: one cohort receiving ART for at least 6 months and one cohort (re)initiating ART.

**Methods**: We categorized and descriptively analysed 142 deaths in the first group (VITAL trial) [2] and 29 deaths in the latter (SaDAPT study) [3]. The deaths were observed between 14 October 2020 and 26 November 2024. Mortality causes, certainty of categorization and immunodeficiency‐relatedness were assessed using an adapted version of the Coding Causes of Death in HIV protocol [4]. Immunodeficiency‐relatedness was determined based on the event descriptions, as well as CD4 counts and viral load measurements within 12 months preceding death.

**Results**: Among people living with HIV engaged in care, the median age at death was 52 years, and the median time from HIV diagnosis until death was 9.4 years, compared with 41 and 0.19 years for people living with HIV (re)intiating ART (Table 1). In the group engaged in care, 26.1% of deaths were classified as related or potentially related to immunodeficiency (Figure 1). For 71 deaths for which a cause could be determined, 43.6% were due to physical trauma, 29.6% due to AIDS‐defining conditions, 8.4% due to cardiovascular conditions, and 5.6% due to non‐AIDS‐defining infections (Table 1). In the group (re)initiating ART, 82.8% of deaths were classified as related or potentially related to immunodeficiency (Figure 1). Of the 23 deaths with an established cause, 91.3% were due to AIDS‐defining conditions (Table 1).

**Conclusions**: Among people living with HIV engaged in care in Lesotho, causes of death have shifted from AIDS‐defining conditions to non‐immunodeficiency‐related causes, with trauma accounting for nearly half of deaths that could be categorized. This underscores the need for integrated care addressing non‐communicable comorbidities and broader injury and violence prevention strategies. Among those (re)initiating ART, immunodeficiency‐related mortality remains dominant, emphasizing the critical need for enhanced care of advanced HIV disease.

**References**

1. Trickey A, McGinnis K, Gill MJ, Abgrall S, Berenguer J, Wyen C, et al. Longitudinal trends in causes of death among adults with HIV in Europe and North America on antiretroviral therapy from 1996 to 2020: A collaboration of cohort studies. Lancet HIV. 2024;11(3):e176–85. doi:10.1016/S2352‐3018(23)00272‐2 PubMed PMID: 38280393; PubMed Central PMCID: PMC11656032.

2. Tschumi N, Lerotholi M, Kopo M, Kao M, Motaboli L, Mokebe M, et al. Integrating clinical decision support and mobile health for differentiated HIV service delivery in Lesotho (VITAL): A cluster‐randomised non‐inferiority trial. eClinicalMedicine. 2026;94:103850. doi:10.1016/j.eclinm.2026.103850 PubMed PMID: 42005925; PubMed Central PMCID: PMC13084333.

3. Gerber F, Semphere R, Lukau B, Mahlatsi P, Mbale H, Sanchez‐Samaniego G, et al. Same‐day versus rapid ART initiation in people with HIV and symptoms of tuberculosis (SaDAPT): A randomised, non‐inferiority trial in Lesotho and Malawi. Lancet HIV. 2026;13(4):e225–34. doi:10.1016/S2352‐3018(25)00276‐0 PubMed PMID: 41692011.

4. Kowalska JD, Friis‐Møller N, Kirk O, Bannister W, Mocroft A, Sabin C, et al. The Coding Causes of Death in HIV (CoDe) Project: Initial results and evaluation of methodology. Epidemiology. 2011;22(4):516. doi:10.1097/EDE.0b013e31821b5332

**FIGURE 1:**
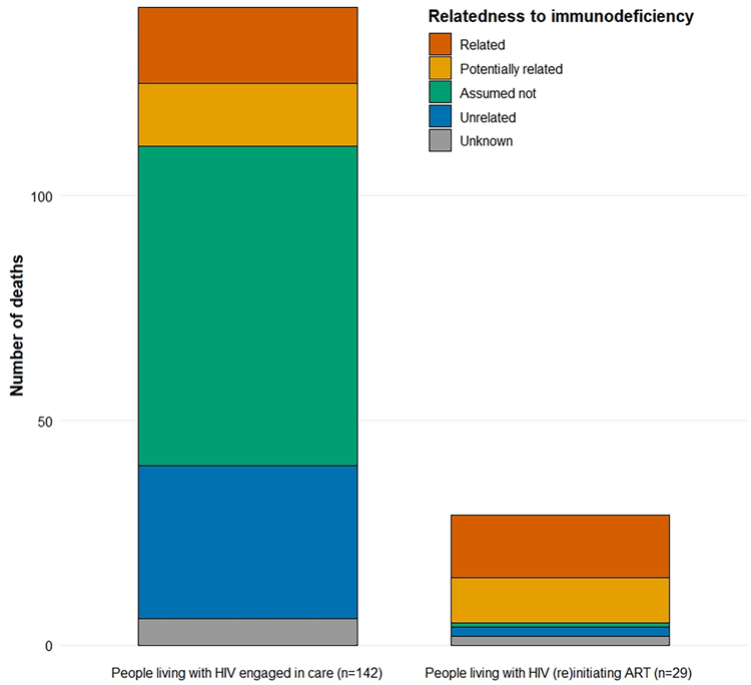
P471 | Immunodeficiency‐relatedness of deaths by study populations.

**TABLE 1:**
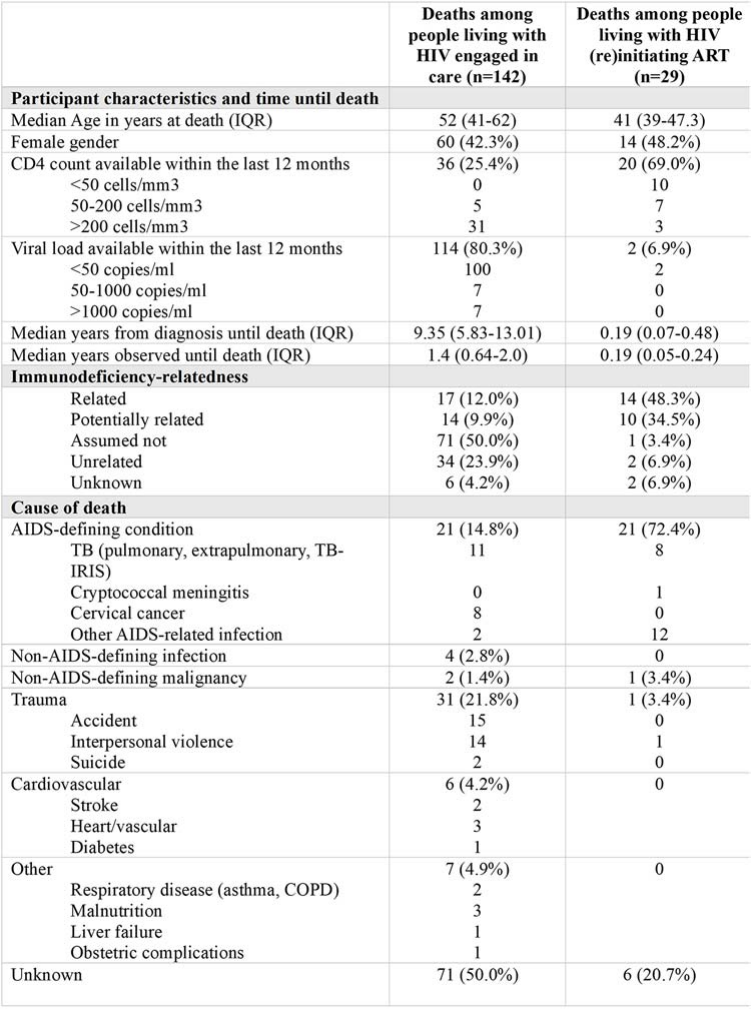
P471 | Participant characteristics, causes of death and relatedness to immunodeficiency by study population.

### P472 Exploring the Associations of Pre‐Existing Metabolic Comorbidities on Changes in Systemic Inflammation Indices in Newly Diagnosed People With HIV Initiating Antiretroviral Therapy

Didem Çelik ^1^, Dicle Zeynep Aksu^1^, Ayleen Mufudza^2^, Sabri Atalay^1^, Patrick W. G. Mallon^2^

^1^Department of Infectious Diseases and Clinical Microbiology, University of Health Sciences Tepecik Education and Research Hospital, Izmir, Türkiye; ^2^Infection Research Ireland, School of Medicine, University College Dublin, Dublin, Ireland

**Background**: Metabolic comorbidities occur commonly in people with HIV (PWH) and are linked to inflammation. We explored whether the presence of chronic metabolic comorbidities at antiretroviral therapy (ART) initiation impacted on changes in measures of systemic inflammation.

**Methods**: This observational, single‐centre, retrospective analysis examined adult PWH starting ART between January 2019 and May 2025 allocated to two groups; those with pre‐existing comorbidities (hypertension, diabetes mellitus, dyslipidaemia, cardiovascular disease, obesity [BMI > 30 kg/m^2^]) and those without. Exclusion criteria included malignancy, co‐infection and pregnancy. Four systemic inflammatory indices (systemic immune‐inflammation index [SIII], systemic inflammatory response index [SIRI], neutrophil‐lymphocyte ratio [NLR], platelet‐lymphocyte ratio [PLR]) were assessed at baseline, 6, 12 and 24 months after ART initiation. Baseline covariates were compared using non‐parametric analyses and changes over 24 months in the four inflammatory indices were compared between groups using linear mixed‐effects models, with data presented as geometric mean (95% CI).

**Results**: Of 341 participants (Table 1), the majority (87.4%) were male and Caucasian (99.1%). 130 (38.1%) participants reported ≥1 comorbidity. Those with comorbidities were older, less likely to attain higher education, had lower MSM transmission risk and were less likely to be on bictegravir. While baseline CD4 (385.2 vs. 339.7 mm^3^), CD4/CD8 ratio (0.43 vs. 0.36), and HIV RNA (113,000 vs. 91,650 cp/mL) were similar between groups (all *p* > 0.05), those with comorbidities had higher baseline total cholesterol (186 vs. 157 mg/dL) and LDL (114 vs. 96.5 mg/dL) (both *p* < 0.001). There were no between‐group baseline differences in the four inflammatory indices (*p* > 0.05). In those with comorbidities, SIII was significantly higher at month 12 and PLR at month 24. NLR and SIRI levels did not differ between groups over time (Figure 1). SIII geometric mean was lower in men (−22.1% [−32.3, −10.3], *p* < 0.001), higher in smokers (+13.9% [+3.5, +25.3], *p* = 0.008) and lower in those on BIC/TAF (−10.6% [−18.8, −1.6], *p* = 0.023), while PLR decreased over 24 months, reducing 10.2%, 14.8% and 17.3% at months 6, 12 and 24, respectively, and was lower in men (−23.7% [−31.5, −14.9], *p* < 0.001) and in those with higher CD4 counts (−2.1% [−3.6, −0.6] per 100 cells/mm^3^ increase, *p* = 0.005). There were no relationships between baseline comorbidities and any of the four inflammatory indices.

**Conclusions**: In PWH, initiating ART, sex, smoking status, duration of treatment and specific treatment regimens, but not presence of baseline metabolic comorbidities, were associated with change in inflammatory indices over 24 months.

**FIGURE 1:**
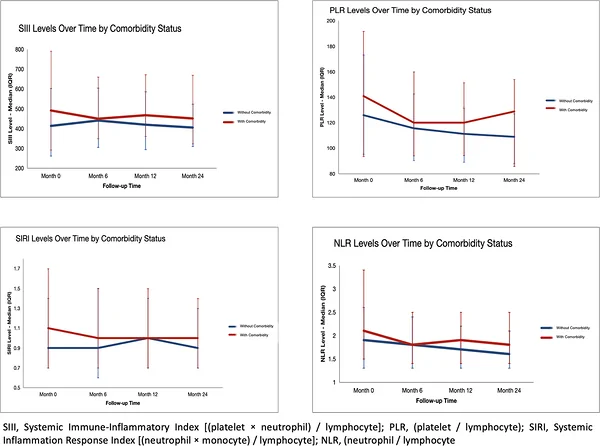
P472 | Longitudinal changes of systemic inflammatory indices (SIII, PLR, SIRI, NLR) in PWH undergoing antiretroviral therapy, according to the presence of comorbidities, over a 24‐month period.

**TABLE 1:**
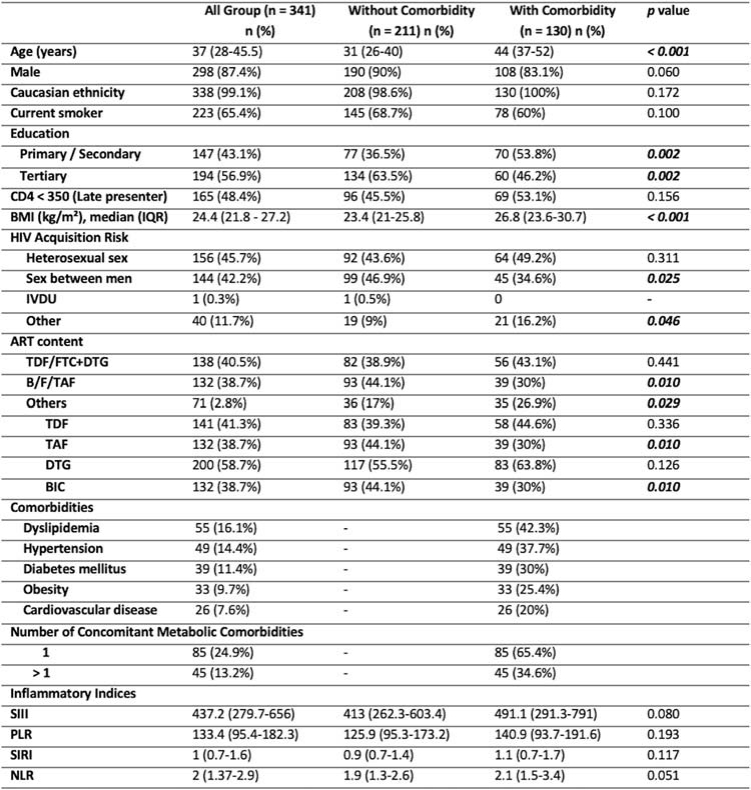
P472 | Distribution of demographic, clinical and comorbidity characteristics of patients included in the study according to groups. Data are presented as numbers and percentages (%) or median (interquartile range, IQR). Categorical and continuous variables were compared using Pearson's chi‐square and Mann–Whitney U tests, respectively. *p*‐values indicate comparisons between the group without comorbidities and the group with comorbidities and were described as statistically significant when they were < 0.05.

### P473 Bone Health in Women Living With HIV: Unravelling Distinct Pathways of Bone Deterioration in a Premenopausal Cohort

Roser Navarro‐Soler, María De Lagarde Sebastián, David Rial‐Crestelo, Juan Martín‐Torres, Otilia Bisbal, Adriana Pinto‐Martinez, Laura Bermejo Plaza, Mireia Santacreu‐Guerrero, Fede Pulido, Rafael Rubio García

HIV Unit. Hospital Universitario 12 de Octubre. Imas12, MADRID, Spain

**Background**: Premenopausal women with HIV carry underrecognized bone deficits, yet the contributions of HIV‐related and traditional risk factors to bone density and microarchitecture remain poorly characterized. We aimed to determine the prevalence of bone alterations and to identify factors independently associated with bone quantity and bone quality in this population.

**Methods**: We conducted a cross‐sectional study of 66 premenopausal women with HIV (aged 30–50) on antiretroviral therapy at Hospital Universitario 12 de Octubre, Madrid. Bone mineral density (BMD) and trabecular bone score (TBS) were assessed by dual‐energy X‐ray absorptiometry (DXA); bone metabolism markers, cumulative tenofovir disoproxil fumarate (TDF) exposure and FRAX scores were collected. Logistic regression modelled bone alteration (T‐score ≤−1.0, Z‐score ≤−2.0); linear regression with robust standard errors modelled TBS. Internal validation used bootstrap resampling (1000 replications).

**Results**: Bone alteration prevalence was 47.0% by T‐score ≤−1.0 (95% CI: 34.6–59.7) and 7.6% by Z‐score ≤−2.0, while degraded TBS was 32.0% (95% CI: 19.5–46.7). Factors predicting bone density differed from those predicting microarchitecture. In multivariable analysis, BMI (OR 0.74, *p* = 0.001) and PTH (OR 0.95, *p* = 0.023) were independently associated with bone alteration, while cumulative TDF showed a non‐significant trend (OR 1.012 per month, *p* = 0.102). For TBS, years since HIV diagnosis was the strongest predictor (β = −0.007 per year, *p* < 0.001), with vitamin D showing a protective association (*p* = 0.020). The logistic model showed good discrimination (apparent AUC 0.84; optimism‐corrected AUC 0.80 by bootstrap internal validation). FRAX discriminated osteoporosis (AUC 0.81), but not degraded TBS (AUC 0.52–0.54) (Figure 1 and Table 1).

**Conclusions**: Premenopausal women with HIV show a high prevalence of bone alterations affecting both density and microarchitecture, with distinct underlying determinants: BMI and PTH for bone quantity, and disease duration and vitamin D for bone quality. These dimensions have distinct determinants, and FRAX does not capture microarchitectural deterioration, supporting comprehensive bone assessment beyond standard DXA in this population.

**FIGURE 1:**
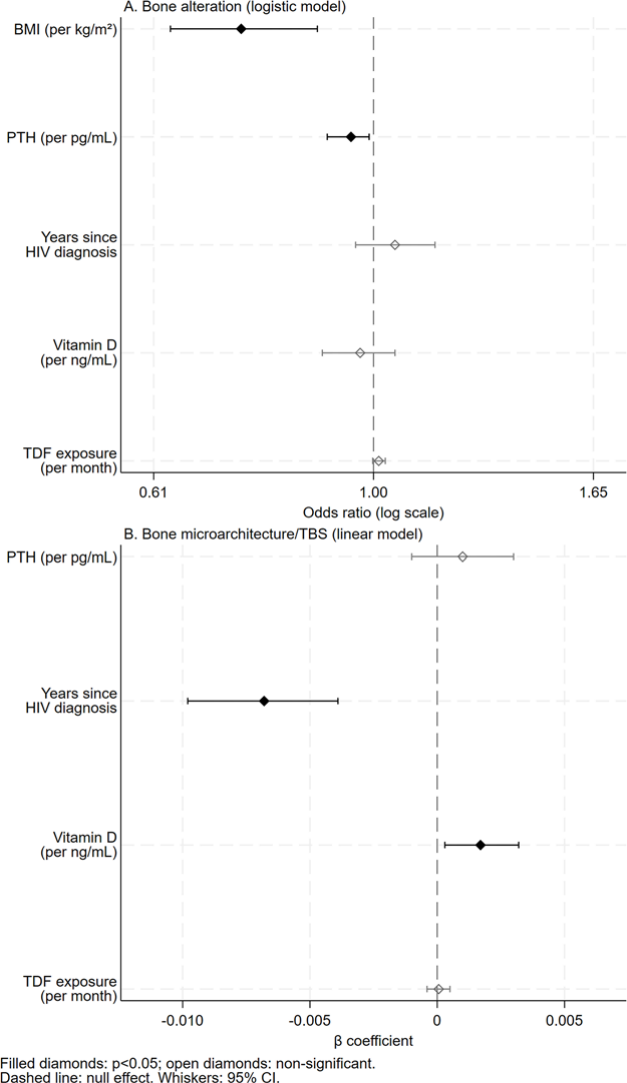
P473 | Multivariable model results for bone quantity and bone quality. Panel A shows odds ratios (OR) from the logistic regression model for bone alteration; Panel B shows β coefficients from the linear regression model for trabecular bone score (TBS). Filled diamonds indicate statistical significance (*p* < 0.05); open diamonds indicate non‐significant associations. Dashed line: null effect. Whiskers: 95% CI. BMI, body mass index; PTH, parathyroid hormone.

**TABLE 1:**
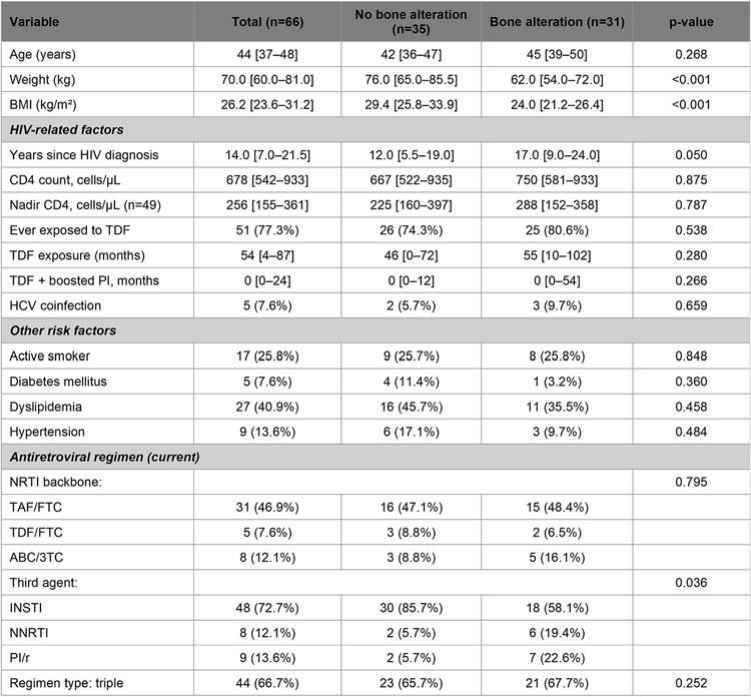
P473 | Characteristics of the study population stratified by bone alteration status. Data shown as median (IQR) or *n* (%). Bold *p*‐values: *p* < 0.05. ABC, abacavir; BMI, body mass index; HCV, hepatitis C virus; INSTI, integrase strand transfer inhibitor; NNRTI, non‐nucleoside reverse transcriptase inhibitor; PI/r, ritonavir‐boosted protease inhibitor; PTH, parathyroid hormone; TAF, tenofovir alafenamide; TDF, tenofovir disoproxil fumarate.

### P474 Prevalence of Osteoporosis Among Women Living With HIV Aged ≥50 Years: A Cross‐Sectional Study

Luísa Lopes, Joana Marinho, Hugo Graça, Filipe Medeiros, Gonçalo Silva, Cristina Valente

Infectious Diseases, Local Health Unit of Coimbra, Coimbra, Portugal

**Background**: People living with HIV (PLWH) are at increased risk of reduced bone mineral density (BMD), osteoporosis and fragility fractures due to the combined effects of chronic inflammation, ageing, menopause and long‐term antiretroviral therapy (ART). Identifying the burden of bone disease in ageing women living with HIV (WLWH) is essential to optimize screening and preventive strategies. This study aimed to determine the prevalence of osteoporosis among WLWH aged ≥50 years receiving care at a tertiary HIV clinic.

**Methods**: A cross‐sectional observational study was conducted including WLWH aged ≥50 years followed at a tertiary hospital during 2025. Data were collected from electronic medical records and included demographic characteristics, current and previous ART regimens, dual‐energy X‐ray absorptiometry (DXA) results, T‐Score and osteoporosis treatment.

**Results**: A total of 473 women were included. The most frequent age group was 50–59 years (45.24%), followed by 60–69 years (34.89%) and ≥70 years (19.66%). The most commonly prescribed ART regimen was lamivudine/dolutegravir (48.41%), followed by bictegravir/emtricitabine/tenofovir (18.39%). Only 142 women (30.0%) had undergone DXA assessment. Among these, osteoporosis was identified in 47.89% (*n* = 68) and osteopenia in 21.13% (*n* = 30). From those with osteoporosis, 44.11% (*n* = 30) had previous exposure to tenofovir disoproxil fumarate (TDF), whereas 52.94% (*n* = 36) had no documented prior TDF exposure. However, bone health evaluation appeared suboptimal, with 331 (69.98%) women lacking DXA records and 23 (4.86%) remaining on TDF‐containing regimens without documented densitometric assessment.

**Conclusions**: A high prevalence of osteoporosis was observed among WLWH aged ≥50 years who underwent DXA assessment. These findings highlight the need for improved implementation of bone health screening recommendations in ageing WLWH.

### P475 Immune Status and Tenofovir Exposure Influence Discordance Between Creatinine‐ and Cystatin C‐Based eGFR Equations in People Living With HIV: A Longitudinal Analysis

Josip Begovac, Matea Rebrina, Ivana Javorić, Šime Zekan, Renata Laškaj, Mirjana Stupnišek

University Hospital for Infectious Diseases, Zagreb, Croatia

**Background**: We evaluated agreement between the 2021 creatinine‐based estimated glomerular filtration rate (eGFRcr) and the combined creatinine–cystatin C equation (eGFRcr‐cys) [1] in people living with HIV (PLWH) [2], focusing on HIV‐specific predictors of formula discordance.

**Methods**: This longitudinal cohort study (18 August 2021–15 May 2024) included 182 PLWH (89.0% male) contributing 251 paired eGFR measurements. Agreement between equations was assessed using a modified Bland–Altman method. Predictors of formula discrepancy (bias; eGFRcr−eGFRcr‐cys) were evaluated using linear mixed‐effects models with age adjustment that accounted for repeated measurements and time‐varying participant characteristics. The proportions of eGFRcr‐cys estimates within ±10% and ±30% of corresponding eGFRcr values were calculated at baseline.

**Results**: At baseline, median age was 56.6 years (IQR 53.3–62.8), median HIV duration was 11.2 years (IQR 5.8–19.0), 38.5% had a history of clinical AIDS, median current CD4 count was 589.5 cells/µL (IQR 421.0–801.0) and 94.5% had HIV RNA <50 copies/mL. Corresponding baseline eGFRcr‐cys estimates were within ±10% and ±30% of eGFRcr in 64.8% and 98.4% of paired measurements, respectively. Longitudinal mixed‐effects analysis showed negligible systematic bias between equations (−0.5 mL/min/1.73 m^2^; 95% CI: −1.8 to 0.8). Modified Bland–Altman analysis demonstrated narrower limits of agreement among participants with eGFR < 60 mL/min/1.73 m^2^ (−10.2 to 13.2 mL/min/1.73 m^2^) than among those with eGFR ≥60 mL/min/1.73 m^2^ (−19.4 to 17.5 mL/min/1.73 m^2^) (Figure 1). Equivalence testing supported average agreement between equations, with the 90% CI for mean bias (−1.6 to 0.6 mL/min/1.73 m^2^) falling within predefined equivalence margins of ±5 mL/min/1.73 m^2^. In age‐adjusted LMMs, cardiometabolic factors were not associated with formula discordance. Lower current CD4 count, prior clinical AIDS and tenofovir exposure were associated with greater discordance between equations. A gradient in bias was observed across non‐tenofovir, TAF‐ and TDF‐containing regimens (*p* = 0.0188), whereas INSTI use had no effect (*p* = 0.8177) (Table 1).

**Conclusions**: Overall agreement between eGFRcr and eGFRcr‐cys was high in this cohort of PLWH. However, discordance varied according to immune status, history of AIDS and tenofovir exposure. Combined creatinine–cystatin C assessment may, therefore, be particularly informative in selected PLWH and should be evaluated against measured glomerular filtration rate.

**References**

1. Inker LA, Eneanya ND, Coresh J, et al. New creatinine‐ and cystatin C‐based equations to estimate GFR without race. N Engl J Med. 2021;385:1737–1749.

2. Mondesert E, Reynes J, Makinson A, et al. Cystatin C in addition to creatinine for better assessment of glomerular renal function decline in people with HIV receiving antiretroviral therapy. AIDS. 2023;37:447–454.

**FIGURE 1:**
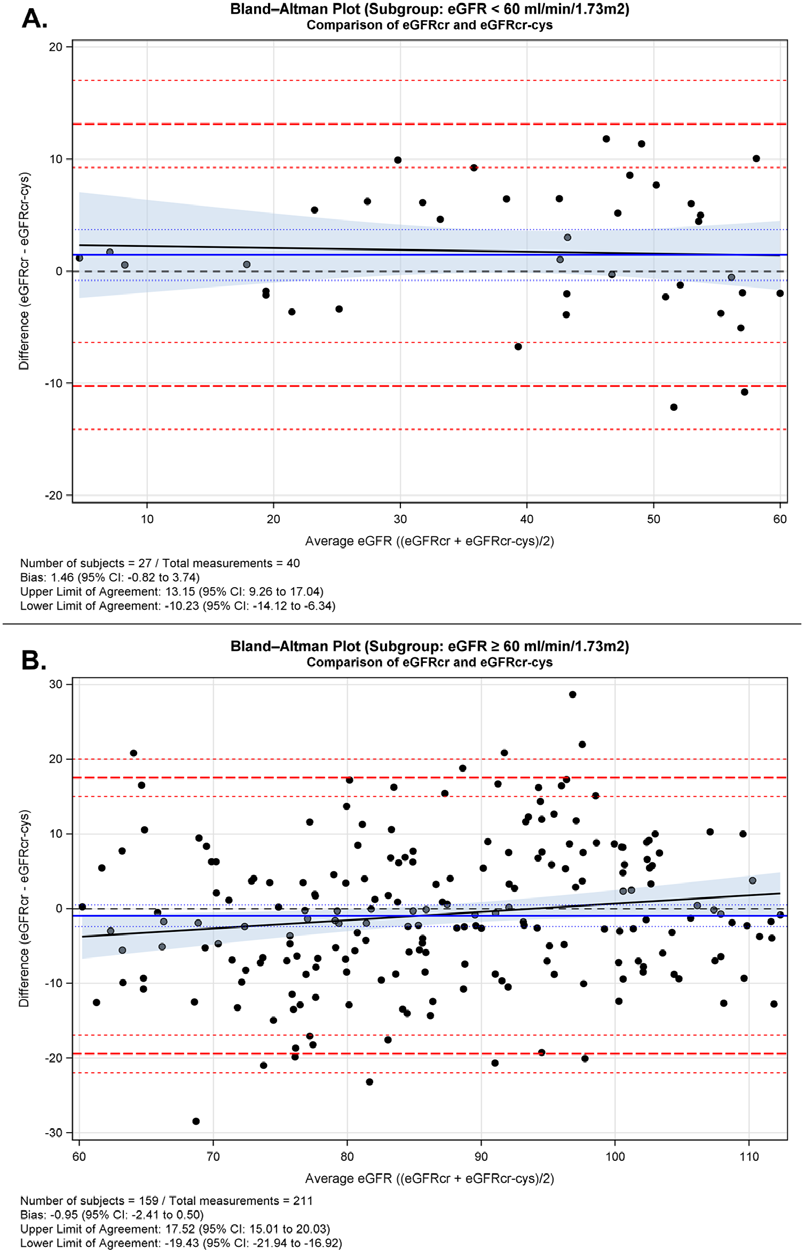
P475 | Modified Bland–Altman plots of agreement between eGFRcr and eGFRcr‐cys equations stratified by renal function. Panel A displays the subgroup with impaired renal function (eGFR < 60 mL/min/1.73 m^2^), and Panel B displays the subgroup with preserved filtration (eGFR ≥60 mL/min/1.73 m^2^). Solid blue lines represent the estimated systematic bias, and red dashed lines indicate the 95% limits of agreement, adjusted for repeated measures within the same individual. The black slanted line in Panel B depicts the regression trend of the differences.

**TABLE 1:**
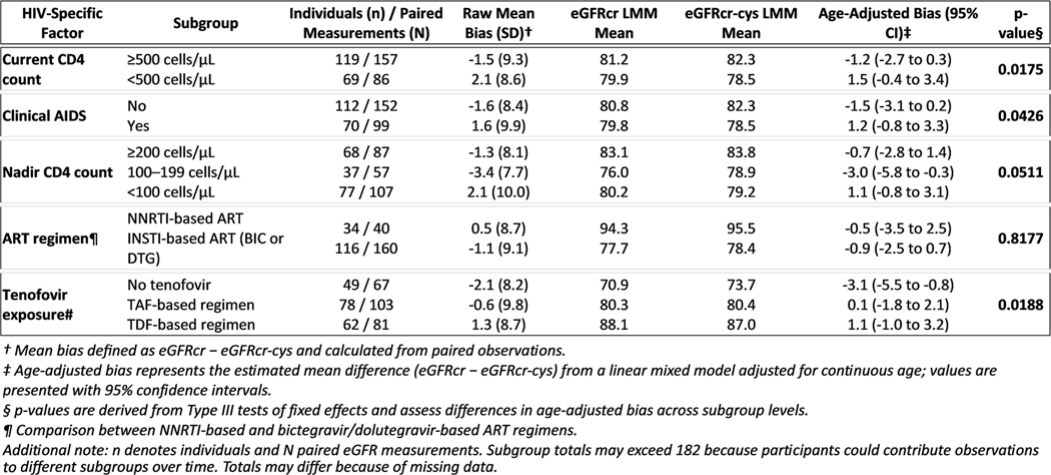
P475 | Agreement between eGFRcr and eGFRcr‐cys according to HIV‐specific clinical factors.

### P476 Similar Prevalence of Age‐Related Comorbidities in People Living With HIV and the General Population in Israel: A Multicentre Matched Cross‐Sectional Study

Daniel Elbirt^1,2^, Itzchak Levy ^3,4^, Liraz Olmer^5^, Daniel Zibli^3^, Anna Eisenberg^6^, Ran Numa^6^, Hila Elinav^2,7^

^1^Department of Allergy, Immunology and HIV, Kaplan Medical Centre, Rehovot, Israel; ^2^Faculty of Medicine, Hebrew University of Jerusalem, Jerusalem, Israel; ^3^Infectious Diseases Unit, Sheba Medical Center, Tel Hashomer, Israel; ^4^Gray School of Medicine, Tel‐Aviv University, Tel Aviv, Israel; ^5^Biostatistics & Biomathematics Unit, Data & Analytics Division, Sheba Medical Center, Tel Hashomer, Israel; ^6^MSD Israel, Hod Hasharon, Israel; ^7^Hadassah AIDS Center, Clinical Microbiology and Infectious Disease Department, Hadassah Hebrew University Medical Center, Jerusalem, Israel

**Background**: As life expectancy for people living with HIV (PLWH) in high‐income settings approaches that of HIV‐negative individuals, more PLWH reach older ages and develop age‐related chronic conditions. Israel's ethnic and lifestyle diversity may result in epidemiology differing from other high‐income countries. We aimed to describe prevalence of chronic disease comorbidities of interest among HIV‐positive patients, as compared to HIV‐negative patients in Israel.

**Methods**: In this retrospective cohort study, we analysed data from three Israeli AIDS centres (PLWH group) and the Maccabi Health Maintenance Organization database (controls). We included 1070 PLWH aged ≥18, diagnosed 2000–2019, with ≥1‐year follow‐up, and matched to one control by age (±1 year), sex and geographic identifier. Comorbidities (CVDs, hypertension, diabetes mellitus, CKD, lipid disorder, neuropsychiatric diseases, malignancies, bone disease) and medications patterns are reported descriptively. For each patient we calculated the total number of comorbidities and categorized multimorbidity as 0, 1, 2 or ≥3 conditions. Group differences were tested using chi‐square, Student's *t*, Kruskal–Wallis or Fisher's exact tests.

**Results**: Nearly one‐third of PLWH had at least one comorbidity (28.4%, Figure 1), with obesity (12.5%), depression (11.3%) and hypertension (9.2%) being the most common ones. Overall multimorbidity prevalence did not differ between PLWH and controls (*p* = 0.059, Figure 1). PLWH had statistically significant higher prevalence of bone disease than controls (3.0% vs. 1.7%; *p* = 0.0451). Dementia trended higher in PLWH than in controls (0.7% vs. 0.2%; *p* = 0.0949). The control group showed statistically significant higher prevalence than PLWH for hypertension, diabetes mellitus and obesity, (14.3% vs. 9.2% [*p* = 0.0002], 7.1% vs. 4.9% [*p* = 0.0287] and 18.3% vs. 12.5% [*p* = 0.0002], respectively). Chronic kidney disease trended higher in controls than in PLWH (4.2% vs. 2.7%; *p* = 0.0584). Age‑stratified analysis showed the largest between‑group difference in the 45–54‑year stratum (Figure 1), where controls had higher multimorbidity driven by greater hypertension, diabetes mellitus and obesity.

**Conclusions**: Nearly one‐third of PLWH had ≥1 comorbidity, most commonly obesity, depression and hypertension. Overall multimorbidity was similar to matched controls, but PLWH showed more bone disease and control group more cardiometabolic disease. These findings support routine bone health screening for PLWH and individualized cardiometabolic and mental health assessment across both groups.

**FIGURE 1:**
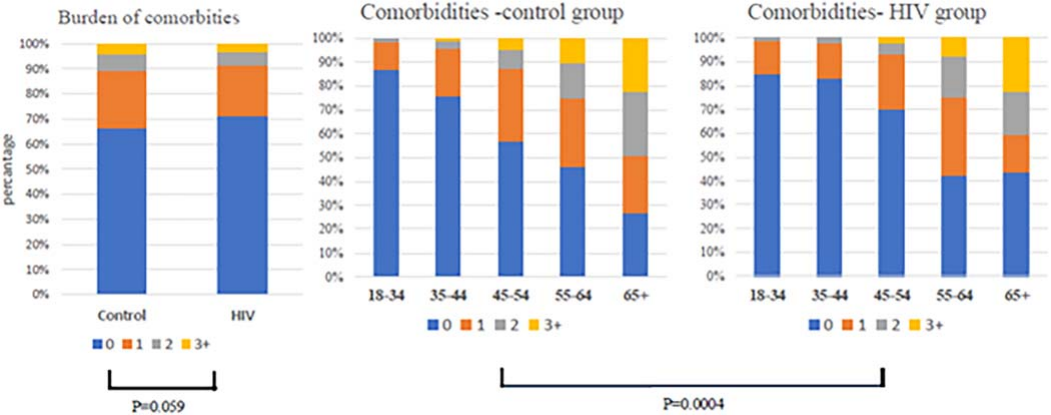
P476 | Comorbidities among PLWH and the control group.

### P477 Maintenance of HIV‐RNA Target Not Detected Status After Switching to BIC/FTC/TAF or DTG/3TC in Anti‐HBc‐Positive People With HIV

Arturo Ciccullo ^1^, Silvia Colaiuda^2^, Francesca Lombardi^3^, Andrea Carbone^3^, Pierluigi Francesco Salvo^3^, Giuseppe Cucinotta^2^, Riccardo Serraino^2^, Gianmaria Baldin^3^, Carlo Torti^2,3^, Simona Di Giambenedetto^2,3^

^1^Infectious Diseases Unit, San Salvatore Hospital, L'Aquila, Italy; ^2^Università Cattolica del Sacro Cuore, Roma, Italy; ^3^Fondazione Policlinico Universitario A. Gemelli IRCCS, Roma, Italy

**Background**: Previous HBV exposure may represent a clinically relevant condition when ART simplification is considered in people with HIV (PWH). Data comparing long‐term maintenance of HIV‐RNA target not detected (TND) status and hepatic surrogate marker evolution after switch to dual versus triple therapy remain limited. We evaluated longitudinal changes in TND prevalence and FIB‐4 score among treatment‐experienced PWH switched to BIC/FTC/TAF or DTG/3TC.

**Methods**: This retrospective longitudinal study included treatment‐experienced PWH switching to BIC/FTC/TAF or DTG/3TC with available follow‐up at 48, 144 and 240 weeks. Primary endpoints were longitudinal maintenance of HIV‐RNA TND and changes in FIB‐4 score. Prespecified subgroup analyses were performed according to HBV serological status. Time to TND loss among individuals with baseline TND was assessed using Kaplan−Meier survival analysis and compared between treatment groups using the log‐rank test. Continuous and categorical variables were compared using non‐parametric and chi‐square/Fisher tests, as appropriate.

**Results**: A total of 1151 PWH were included: 649 in the DTG group and 502 in the BIC group (baseline characteristics in Table 1). No significant longitudinal differences in TND prevalence were observed between treatment groups in the overall study population irrespective of HBV serological status. However, among anti‐HBc‐positive individuals with baseline TND, loss of TND during follow‐up occurred more frequently in those receiving DTG/3TC compared with BIC/FTC/TAF (37.5% vs. 20.9%; *p* = 0.014). Three‐year probability of maintaining TND was 93.0% with DTG/3TC and 98.6% with BIC/FTC/TAF (log‐rank *p* < 0.001).

Regarding hepatic surrogate markers, participants receiving DTG/3TC showed a significant increase in FIB‐4 at week 48 (*p* = 0.010) and 144 (*p* < 0.001), while no significant changes were observed with BIC/FTC/TAF. In anti‐HBc‐positive individuals receiving DTG/3TC, median FIB‐4 significantly increased from baseline to week 48 (*p* = 0.050) and 144 (*p* = 0.033), despite modest absolute variation (median ∆FIB‐4 approximately +0.1).

**Conclusions**: Among anti‐HBc‐positive PWH, DTG/3TC was associated with reduced long‐term maintenance of HIV‐RNA TND status compared with BIC/FTC/TAF. These findings suggest that previous HBV exposure may identify a clinically relevant subgroup when ART simplification strategies are considered.

**TABLE 1:**
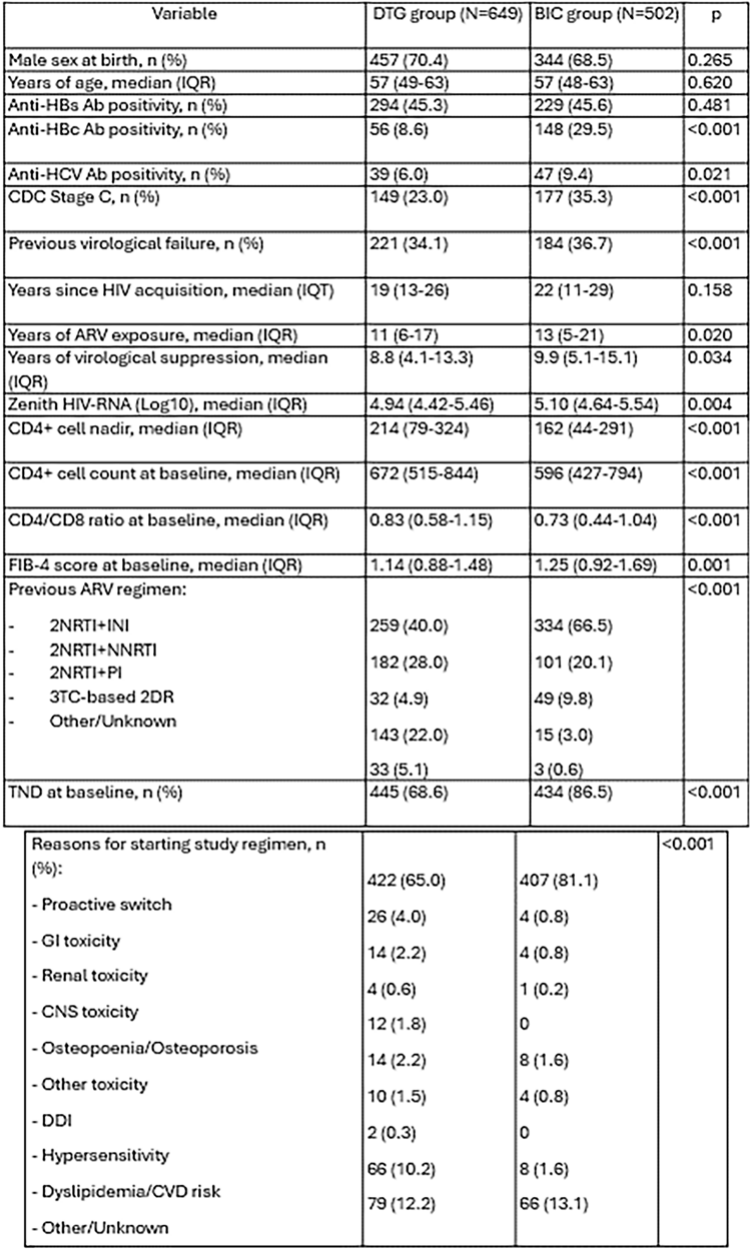
P477 | Population characteristics at baseline.

### P478 Streamlining Holistic Care for People Living With HIV: Implementation of a Digital Referral Tool to Improve Efficiency and Patient Outcomes

Katie Lamb ^1^, Dayan Vijeratnam^1^, Sarah Oliver^2^

^1^Chelsea and Westminster Hospital NHS Foundation Trust, London, UK; ^2^University Of Hertfordshire, Hertfordshire, UK

**Background**: People living with HIV (PLWH) increasingly present with complex needs beyond clinical management, including mental health concerns, social care requirements and experiences of stigma or domestic abuse. Addressing these psychosocial factors is essential for person‐centred care, as stability is directly linked to improved medication adherence and viral suppression. However, clinicians often find referring to external support agencies time‐consuming and difficult, frequently relying on “pockets of expertise” held by specific staff members rather than a unified system.

**Methods**: This quality improvement (QI) project utilized Lean methodology and the NHS Change Model to identify and eliminate waste in the referral process. A mixed‐methods baseline survey was conducted with 22 clinicians (doctors, nurses and allied healthcare professionals) across five HIV clinic sites to establish referral barriers. A pilot digital referral tool—a centralized directory of support agencies with direct links to agency web pages and referral forms—was implemented at a primary site. Effectiveness was evaluated through a second survey of the pilot group.

**Results**: The baseline survey revealed that 77% of referrals took at least 10 min, with 32% requiring 20–30 min or more. Furthermore, 54% of respondents found identifying the correct organization or pathway “hard” or “very hard.” Expertise was unevenly distributed, with many clinicians frequently consulting health advisors or nurses to facilitate referrals. Following the pilot, 100% of respondents reported that the tool had changed their process and significantly reduced the time required for referrals. All users rated the tool as easy to navigate.

**Conclusions**: The implementation of a unified digital directory resulted in a leaner, more productive process for holistic care. By centralizing information, the tool reduced clinician burden and potential delays in patient support. While successful as a pilot, this project highlights the potential for further digital expansion, such as a dedicated mobile app, to enhance patient autonomy and align with national digital healthcare strategies.

### P479 Differential Impact of Tenofovir Formulations on Bone Mineral Density: Long‐Term Real‐World Evidence From a Longitudinal HIV Cohort

Giuseppe Lapadula ^1,2^, Laura Antolini^1^, Francesca Iannuzzi^2^, Alice Ranzani^2^, Fiore Artelli^1^, Viola Cogliandro^2^, Nicola Squillace^2^, Alessandro Soria^2^, Maria Elena Locatelli^2^, Paola Columpsi^2^, Paolo Bonfanti^1,2^

^1^University of Milano Bicocca, Milan, Italy; ^2^IRCCS Fondazione San Gerardo dei Tintori, Monza, Italy

**Background**: Tenofovir disoproxil fumarate (TDF) is associated with reduced bone mineral density, whereas tenofovir alafenamide (TAF) showed a more favourable profile in randomized clinical trial and short‐term observational studies. Long‐term real‐world evidence remains limited.

**Methods**: We conducted a longitudinal study within the HIV‐HSG cohort including persons with HIV (PWH) with ≥2 dual‐energy X‐ray absorptiometry (DXA) scans. Follow‐up was partitioned into intervals defined by consecutive DXA measurements. Linear mixed‐effects models evaluated the association between T‐score measured at the end of each interval and the cumulative exposure to tenofovir‐free, TDF‐containing and TAF‐containing regimens, adjusting for T‐score at the beginning of the interval. Effect modification by clinical characteristics was assessed through interaction terms with exposure components. Random effects accounted for repeated measurements.

**Results**: 481 patients contributed 1684 DXA measurements, defining 1203 observation intervals. The median number of DXA scans per patient was 3 (IQR 2–4) covering a median follow‐up of 6.5 years (IQR 3.8–9.6). In mixed‐effects models adjusted for baseline value, femoral T‐score showed a significant decline during TDF exposure (per year: β = −0.025, 95% CI: −0.05 to −0.001, *p* = 0.043), whereas no significant change was observed during TAF (β = −0.01, 95% CI: −0.037 to 0.017, *p* = 0.456) or during periods without tenofovir (β = −0.018, 95% CI: −0.04 to 0.005, *p* = 0.121). Table 1 summarizes the interaction effects of each tested covariate with tenofovir exposure. The most relevant effect modifier was the interaction between age and sex: women > 50 years old showed a markedly steeper decline under TDF (additional β = −0.160, *p* < 0.001), while older men showed an attenuated effect (β = 0.045, *p* = 0.027). Underweight (i.e. body mass index < 18.5) was independently associated with worse T‐score trajectories (β = −0.139 to −0.209, *p* ≤ 0.001). No significant effects were found for smoking, alcohol, hepatitis C, diabetes or glomerular filtration. In contrast to the femoral site, no significant association was found between treatment and change in lumbar spine T‐score (*p* > 0.05).

**Conclusions**: Cumulative TDF exposure, but not TAF exposure, was independently associated with a progressive decline in femoral T‐score. This effect was amplified in women over 50 and in underweight individuals, who experienced the steepest bone loss under TDF. These findings support a preferential use of TAF‐based regimens in these individuals.

**TABLE 1:**
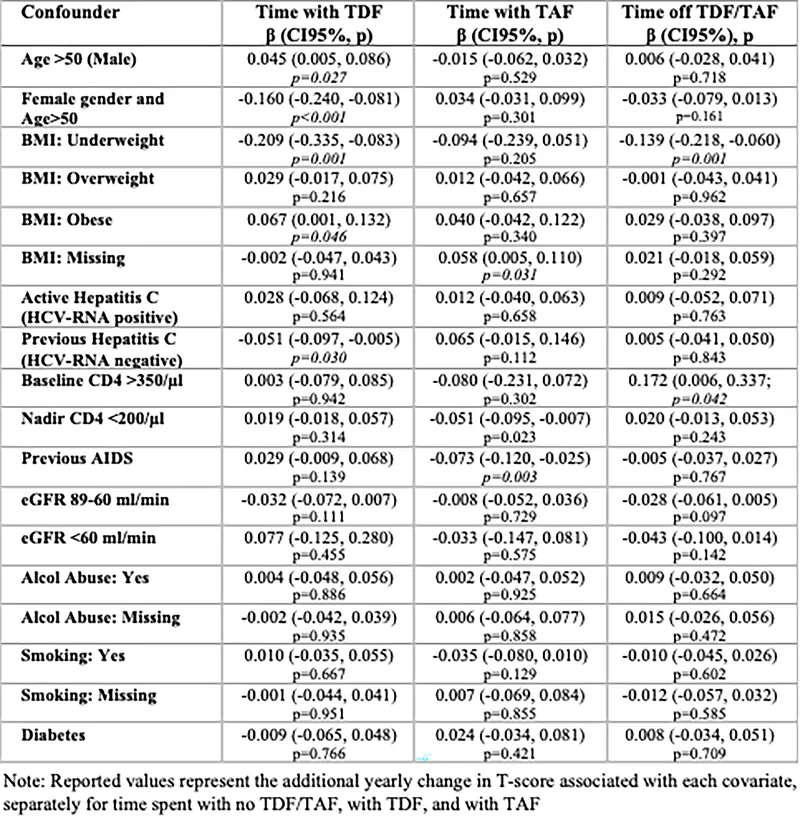
P479 | Interaction effects (β, 95% CI, *p*‐value) of each tested covariate with time (in years) spent with no tenofovir, under TDF and under TAF.

### P480 Comparing Hospital Morbidity Between People With HIV Enrolled and Unenrolled in the Swiss HIV Cohort Study

Marius Zeeb ^1,2^, Frederique Hovaguimian^3^, Matthias Joos^4^, Anders Boyd^2^, Bernard Surial^2^, Johannes Nemeth^1^, Irene A. Abela^1^, Silvio Brugger^1^, Huldrych F. Günthard^1^, Roger Kouyos^1^

^1^Department of Infectious Diseases and Hospital Epidemiology, University Hospital Zurich, University Zurich, Zurich, Switzerland; ^2^Department of Infectious Diseases, Inselspital Bern, Bern, Switzerland; ^3^Clinical Trials Center, University Hospital Zurich, Zurich, Switzerland; ^4^Direktion Digital & IT, University Hospital Zurich, Zurich, Switzerland

**Background**: People with HIV (PWH) have a high comorbidity burden despite anti‐retroviral therapy. As most estimates of morbidity burden are based on PWH enrolled in prospective cohort studies, little is known about unenrolled PWH. To assess morbidity differences, we used data from the Zurich HIV Hospital Cohort (ZHHC), containing electronic health record (EHR)‐data from persons hospitalized at the University Hospital Zurich, and data from the Swiss HIV Cohort Study (SHCS). We determined factors associated with SHCS enrolment and compared the morbidity burden (i.e. standardized morbidity ratios [SMR] and disability‐adjusted life years [DALY]) between enrolled and unenrolled PWH.

**Methods**: We determined factors associated with time to SHCS enrolment using a Fine−Gray model, accounting for death as a competing risk. We determined the association between time‐updated SHCS enrolment and all‐cause mortality using a proportional hazards Cox model. For both analyses, follow‐up started at the date of the first hospital visit with HIV. Lastly, we compared SMRs (by sex, age, calendar years: 2013–2022) and DALY between enrolled and unenrolled PWH using data from the global burden of disease (GBD) study and the Federal Statistical Office for mapping of ZHHC‐ICD10 codes to GBD causes, Swiss‐specific GBD‐cause prevalence and disability weights, and life expectancies.

**Results**: We included 3063 PWH enrolled and 2167 unenrolled in the SHCS (Table 1). Enrolment was associated with male sex (subdistribution‐hazard‐ratio [sHR] 1.4 95% confidence interval [CI] 1.2–1.5) and non‐European origin (sHR 0.61, 95% CI: 0.56–0.67). PWH unenrolled in the SHCS had a significantly higher mortality (HR 2.6, 95% CI: 2.2–3.2). SMR was similar in 2013 (enrolled 1.24, 95% CI: 1.15–1.33; unenrolled 1.32, 95% CI: 1.13–1.52), but dissimilar in 2022 (enrolled 1.06, 95% CI: 0.99–1.13; unenrolled 1.74, 95% CI: 1.66–1.83) (Figure 1). Total DALYs were 412 and 1157 per 100 PWH in enrolled and unenrolled, respectively. Assuming a counterfactual DALYs replacing unenrolled for enrolled PWH between 2013 and 2022, the avoided economic loss attributed to participation in the SHCS was 1,061,978,172 CHF based on gross‐domestic product data.

**Conclusions**: Unenrolment was associated with significantly increased morbidity and all‐cause mortality. Overall, this study gives unique insight into the health of PWH unenrolled in cohort studies, showing the benefits for PWH of being enrolled in the SHCS and, consequently, decreasing the burden on the public health‐care system.

**FIGURE 1:**
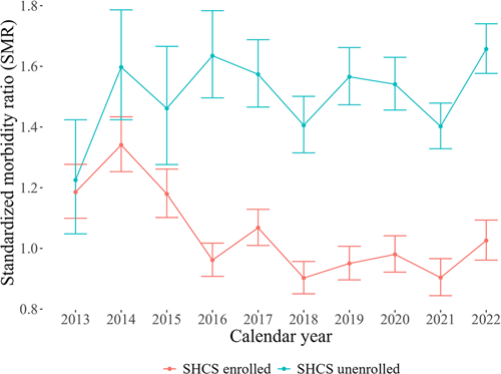
P480 | Standardized morbidity ratios.

**TABLE 1:**
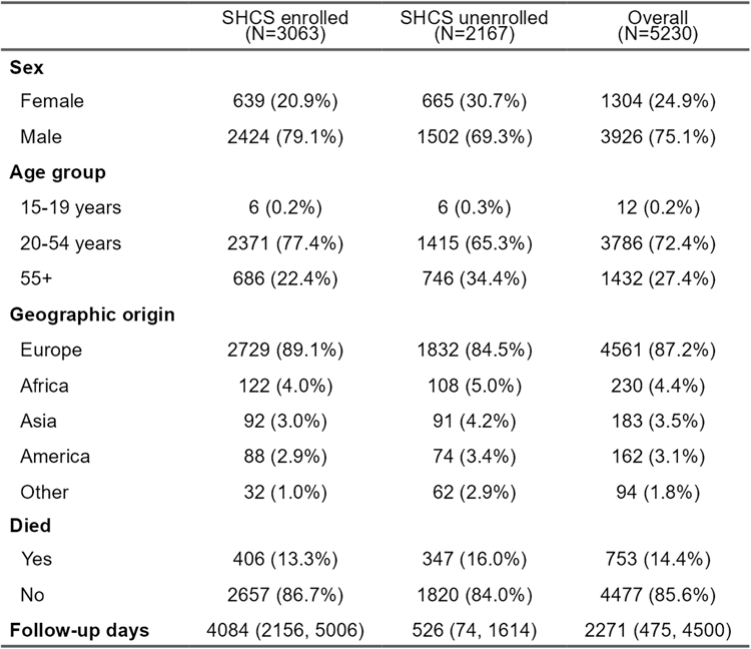
P480 | Study population characteristics.

### P481 Solid Organ Transplantation in People Living With HIV: Long‐Term Outcomes From a Portuguese Tertiary Centre

Rita De Almeida Teixeira ^1^, Márcia Daniela Silva^1^, Marcelo Pinto Alves^1^, João Matos^1^, Luís Marques Silva^2^, Sofia Ferreira^3^, Maria La Salete Martins^4^, Ana Cipriano^1^, Josefina Méndez^1^

^1^Serviço de Doenças Infeciosas da Unidade Local de Saúde Santo António, Porto, Portugal; ^2^Serviço de Microbiologia da Unidade Local de Saúde Santo António, Porto, Portugal; ^3^Unidade de Transplante Hepático da Unidade Local de Saúde Santo António, Porto, Portugal; ^4^Unidade Transplante Renal da Unidade Local de Saúde Santo António, Porto, Portugal

**Background**: Solid organ transplantation (SOT) is established for selected people living with HIV (PLWH) [1], but Portuguese data remain scarce [2–4]. Portuguese legislation and regulatory guidance exclude donors with HIV antibodies, restricting HIV‐positive donor transplantation [5, 6]. We describe long‐term outcomes after SOT in PLWH at a tertiary centre.

**Methods**: Single‐centre retrospective case series of PLWH who underwent kidney or liver transplantation at Unidade Local de Saúde Santo António, Portugal, from programme inception to June 2026. Patients were identified by cross‐referencing transplant databases, HIV laboratory records and the HIV outpatient registry. Data were obtained by chart review and analysed in SPSS (v30.0). ART–immunosuppression interactions were assessed using the University of Liverpool database [7].

**Results**: Seven PLWH underwent SOT, predominantly liver transplantation; baseline and transplant characteristics are summarized in Table 1. ART was modified around transplantation in 3/7 recipients (42.9%), mainly from boosted protease inhibitor‐ or efavirenz‐based regimens to unboosted integrase inhibitor‐based ART. Potential clinically relevant ART–immunosuppression interactions were identified in 4/7 (57.1%) with calcineurin inhibitors, supporting systematic pre‐transplant pharmacological review. One liver recipient underwent two further liver transplant procedures, with familial amyloid polyneuropathy and hepatic artery thrombosis recorded as retransplant indications. Post‐transplant rejection occurred in 4/7 recipients (57.1%), comprising five episodes due to recurrent rejection in one recipient; treatment included corticosteroid pulses, tacrolimus optimization and basiliximab in the recurrent case. Severe non‐CMV infections requiring hospitalization occurred in 2/7 recipients (28.6%): invasive listeriosis and emphysematous pyelitis. CMV viraemia occurred in 4/7 (57.1%), without CMV disease. No post‐transplant AIDS‐defining opportunistic infection was documented. At 12 months, HIV RNA was undetectable in 5/6 recipients (83.3%); one had viraemia while off ART and one had not yet reached 12‐month follow‐up. At last follow‐up, all recipients had undetectable HIV RNA. Six recipients were alive at analysis date (85.7%); one died 13.4 years after index transplantation from non‐infectious surgical complications of a third liver transplant.

**Conclusions**: SOT was feasible in carefully selected PLWH, with sustained HIV virological control despite frequent rejection and complex drug–drug interaction challenges.

**References**

1. Zarinsefat A, Gulati A, Shui A, Braun H, Rogers R, Hirose R, et al. Long‐term outcomes following kidney and liver transplant in recipients with HIV. JAMA Surg. 2022;157(3):240–247. doi:10.1001/jamasurg.2021.6798.

2. Querido S, Machado D, Silva C, Nolasco F, Nunes A, Sampaio S, et al. Renal transplantation in HIV‐infected patients: The first Portuguese review. Transplant Proc. 2015;47(4):946–949. doi:10.1016/j.transproceed.2015.03.029.

3. Ferreira Leal E, Dias A, Póvoas D, Leal M, Caeiro A, Pinheiro H, et al. Liver transplantation among HIV‐infected patients: Experience with 37 patients from a reference centre in Lisbon, Portugal. Available at: https://escmid.reg.key4events.com/AbstractList.aspx?ai=5437&aig=‐1&e=5&preview=1. Accessed 23/06/2026.

4. Agüero F, Rimola A, Stock P, Grossi P, Rockstroh JK, Agarwal K, et al. Liver retransplantation in patients with HIV‐1 infection: An international multicenter cohort study. Am J Transplant. 2016;16(2):679–687. doi:10.1111/ajt.13461.

5. Assembleia da República. Lei n.° 36/2013, de 12 de junho. Available at: https://diariodarepublica.pt/dr/detalhe/lei/36‐2013‐496738. Accessed 23/06/2026.

6. Instituto Português do Sangue e da Transplantação. Circular Normativa n.° 001/CN‐IPST, IP/2018: prevenção da transmissão de doenças infeciosas por dadores de órgãos. Available at: https://www.ipst.pt/files/IPST/INFORMACAO_DOCUMENTACAO/Circular_Normativa_001_2018_dador_orgaos.pdf. Accessed 23/06/2026.

7. University of Liverpool. HIV Drug Interactions: interaction checker. Available at: https://www.hiv‐druginteractions.org/checker. Accessed 23/06/2026.

**TABLE 1:**
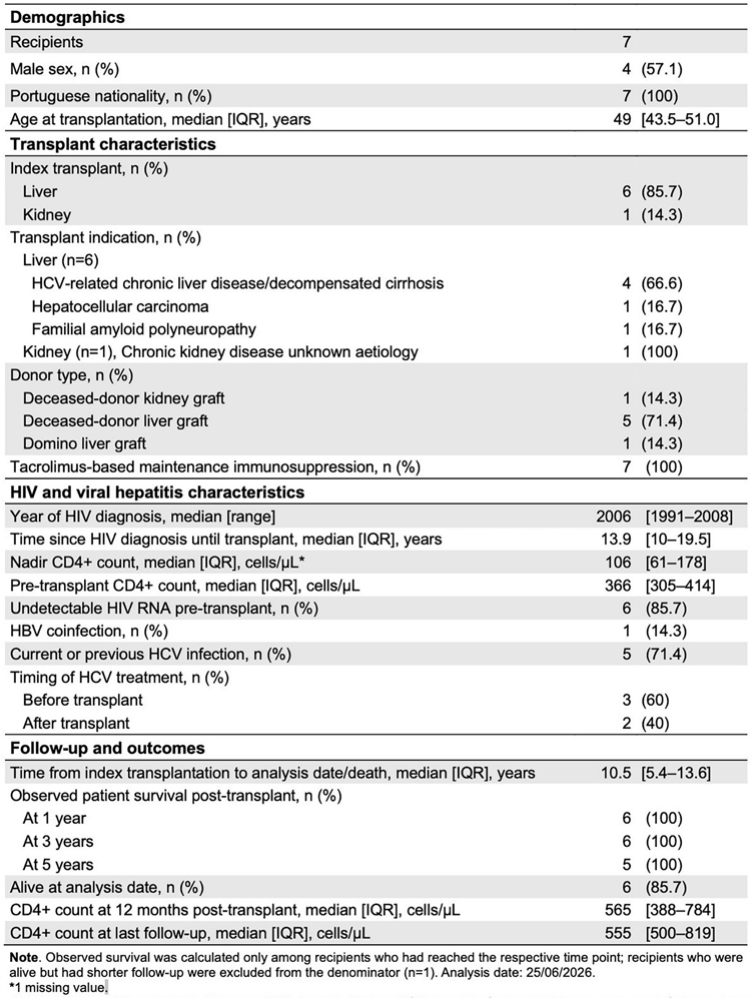
P481 | Baseline and transplant characteristics.

### P482 Layer‐by‐Layer Macular, Peripapillary Retinal Nerve Fibre Layer Thickness and Minimum Rim Width Changes in People Living With HIV With Undetectable Viral Load: An OCT‐Based Study

Müge Toygar Deniz ^1^, Büşra Yılmaz Tuğan^2^, Gözde Karaca^2^, Nurşen Yüksel^2^, Sıla Akhan^1^

^1^Department of Infectious Diseases and Clinical Microbiology, Kocaeli, Türkiye; ^2^Department of Ophthalmology, Kocaeli, Türkiye

**Background**: This study aimed to evaluate layer‐by‐layer macular thickness and optic nerve head (ONH) parameters including the peripapillary retinal nerve fibre layer (pRNFL) and Bruch's membrane opening minimum rim width (BMO‐MRW) in virologically suppressed people living with HIV (PLWH) and to compare them with healthy controls.

**Methods**: This prospective cross‐sectional study included 94 eyes from virologically suppressed PLWH and 80 eyes from age‐ and sex‐matched healthy controls. All participants underwent spectral‐domain optical coherence tomography (SD‐OCT). Layer‐by‐layer macular thickness, pRNFL, BMO‐MRW and sub‐foveal CT thickness parameters were analysed.

**Results**: The groups were comparable in age, sex, intraocular pressure, best corrected visual acuity, central corneal thickness and axial length (*p* > 0.05). Most layer‐by‐layer thickness parameters in macular region, as well as subfoveal CT, were significantly thinner in PLWH than in controls (*p* < 0.05, for all) (Table 1). Also, significant thinning observed in global, temporal and nasal inferior sectors of the pRNFL and all sectors of the BMO‐MRW (*p* < 0.05) in PLWH compared to the controls (Figure 1). Correlation analysis revealed that baseline HIV RNA levels were moderately negatively correlated with the thickness of the inner temporal NFL (*r* = −0.542, *p* < 0.001), inner nasal NFL (*r* = −0.516, *p* < 0.001), central NFL (*r* = −0.553, *p* < 0.001) and central inner retinal layer (IRL) (*r* = −0.503, *p* < 0.001).

**Conclusions**: These findings indicate that retinal and optic nerve head alterations may occur even in immunocompetent PLWH with an undetectable viral load. These changes may reflect persistent chronic inflammation or long‐term ART effects. BMO‐MRW thinning may serve as valuable indicator to reflect optic nerve integrity. OCT provides a valuable, non‐invasive tool for detecting early structural retinal and optic nerve changes in this population.

**FIGURE 1:**
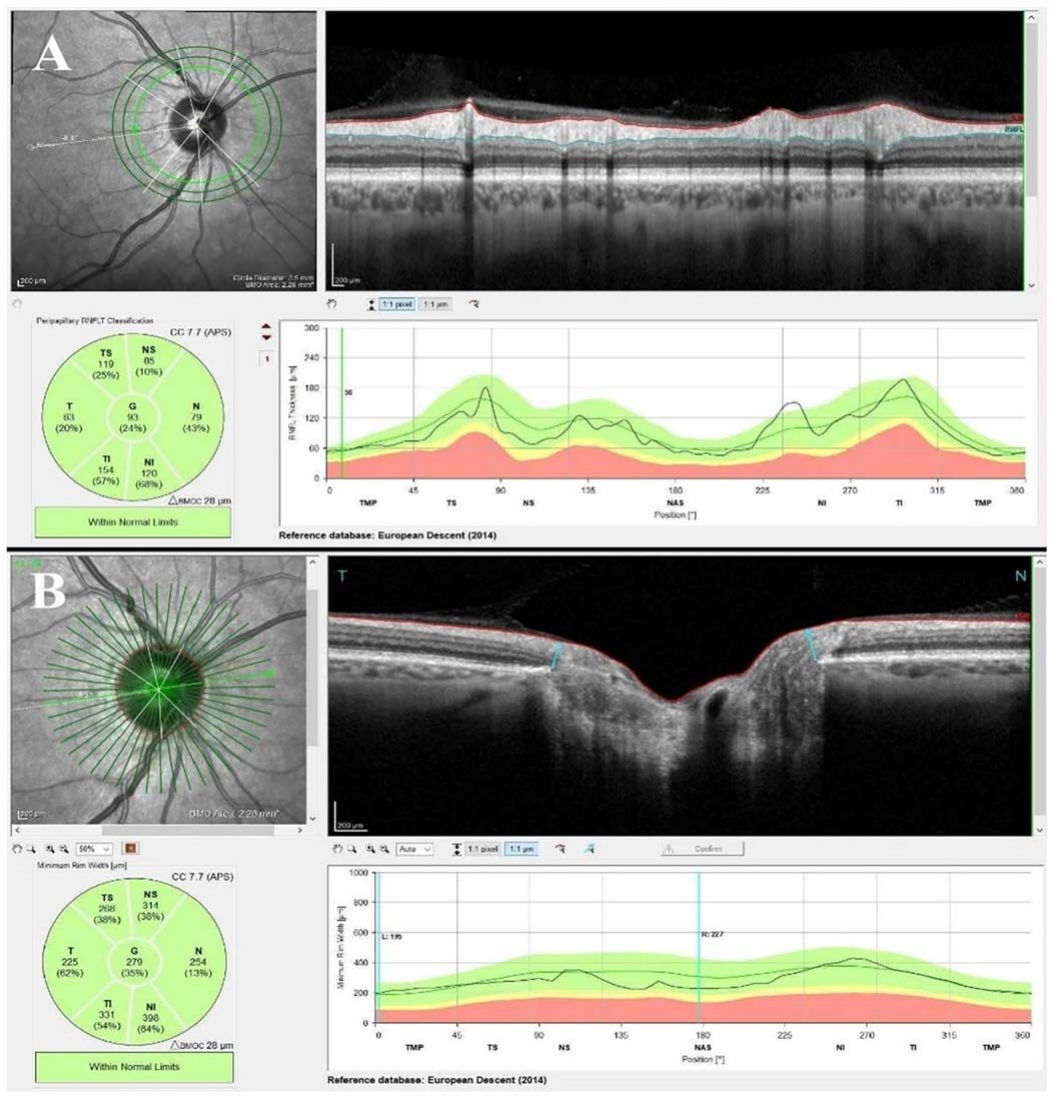
P482 | (A) Retinal nerve fibre layer (RNFL) thickness analysis in PLWH using a 3.5‐mm‐diameter peripapillary scan circle; (B) Bruch's membrane opening–minimum rim width (BMO‐MRW) analysis in PLWH for optic nerve head rim evaluation.

**TABLE 1:**
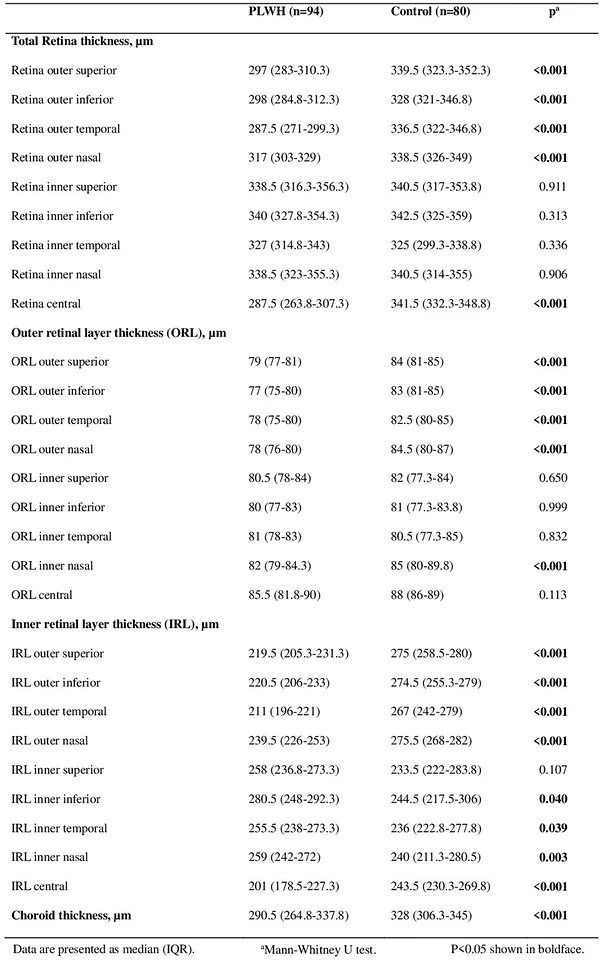
P482 | Comparison of thickness parameters in different sectors of whole retina, outer, inner retinal layers and choroidal thickness between the groups.

### P483 Occurrence of Bacterial Sexually Transmitted Infections in a Cork‐Based HIV Pre‐Exposure Prophylaxis (PrEP) Clinic

Ciarán Hurley ^1^, Gabriella Rizzo^1,2^

^1^School of Medicine, University College Cork, Cork, Ireland; ^2^Cork University Hospital, Cork, Ireland

**Background**: *Neisseria gonorrhoeae* (GC), *Chlamydia trachomatis* (CT) and early syphilis infection (EIS) remain consistently reported by gay, bisexual and men who have sex with men (gbMSM), particularly PrEP users. While national data demonstrates increasing rates of these bacterial STIs, limited regional data exist outside Dublin. This study aims to examine the occurrence and predictors of GC, CT and EIS among PrEP users attending a Cork‐based PrEP clinic. We wished to determine the occurrence of GC, CT and EIS, and to assess behavioural and personal factors associated with bacterial sexually transmitted infections (STIs) acquisition.

**Methods**: We conducted a retrospective chart review of the PrEP users attending the South Infirmary Victoria University Hospital (SIVUH) HIV PrEP clinic between February 2022 and February 2025. Eligible candidates (*N* = 453) had taken PrEP with at least one follow‐up appointment. We documented demographic, behavioural, and clinical variables and utilized chi‐squared testing, Cochran's *Q* test, McNemar tests, logistic regression and Spearman's rank correlation for statistical analysis.

**Results**: Among the 453 eligible patients (mean age 37.4 ± 10.39, 92.39% MSM), 37.3% were diagnosed with reported occurrence of a bacterial STI after initiation of PrEP. GC was the most common (22.52%), followed by CT (17.7) and EIS (6.4%) (*p* = 0.001). GC pharyngeal infection was the most commonly known site. STI acquisition was associated with having over five recent sexual partners (RR 1.46, 95% CI: 1.15–1.86, *p* = 0.002), and suboptimal condom use (OR 2.64, 95% CI: 1.44–4.82, *p* = 0.001). Age, dosing regimen and frequency of clinical attendance did not affect the occurrence of bacterial STIs.

**Conclusions**: Bacterial STIs are common in attendees of this Cork‐based clinic. Condom usage and increased sexual partners were the predominant predictors in the acquisition of bacterial STI. Our findings emphasize the importance of behavioural counselling, routine screening and focused regional sexual health facilities for those who use PrEP.

## People Living With HIV and Mpox Virus

### P484 MPXV‐Positive Samples in Berlin 2022−2026: Clade‐Specific PCR, NGS Sequencing and Phylogenetic Analyses

Merlin Lautner, Robert Ehret, Özlem Yildirim, Malik Prentice, Martin Obermeier

Medical Center for Infectious Diseases, Berlin, Germany

**Background**: Since the major 2022 outbreak of MPXV clade IIb, infections in Europe have declined after vaccination campaigns, but sporadic detections continue, including in vaccinated individuals. We investigated viral diversity, phylogenetic development and clade distribution among MPXV‐positive samples from Berlin.

**Methods**: We sequenced 43 MPXV‐positive samples from 2022 and 77 samples from 2023 to 2026. Whole‐genome amplification used the xGen Monkeypox Virus Amplicon Panel, a single‐tube, two‐step PCR workflow covering the MPXV genome. Libraries were prepared by PCR‐based barcoding or the QIAseq FX DNA Library Kit for Illumina MiSeq sequencing, and by rapid or native barcoding for Oxford Nanopore MinION sequencing. Clade assignment was additionally supported by clade‐specific real‐time PCR assays for MPXV clades I/II and subclades/lineages as described by Huo et al. [1] and Li et al. [2] Bioinformatic analysis used Illumina DRAGEN or an in‐house pipeline, followed by lineage and phylogenetic analysis with Nextclade.

**Results**: Comparison of the four sequencing workflows showed substantial differences in genome coverage, read distribution and frameshift artefacts. The IDT amplicon panel performed best on MiSeq, consistent with its short amplicon design, which was less suitable for Nanopore rapid barcoding. Nanopore native barcoding achieved results comparable to MiSeq. Lineage analysis revealed increasing diversification over time. In 2022, 97.2% of sequences clustered within lineage B, whereas more diverse lineages were observed from 2023 to 2026. Until Q3 2025, all patient samples belonged to clade IIb. In Q4 2025, the first clade Ib infection was detected. In Q1 2026, 80% of 42 positive samples belonged to clade Ib, whereas in Q2 2026, 62% of 29 positive samples again belonged to clade IIb. Several phylogenetic clusters were identified, suggesting repeated introductions of different MPXV variants into the Berlin MSM community rather than continuous evolution from a single local transmission chain.

**Conclusions**: MPXV clade IIb lineages diversified substantially after the 2022 outbreak, with lineages D, E and F emerging in addition to lineage B. Since December 2025, clade Ib infections have been increasingly detected. The observed pattern supports multiple independent introduction and transmission events. To date, no clear genomic evidence of vaccine escape has been identified.

**References**

1. Huo S, Chen Y, Lu R, et al. Development of two multiplex real‐time PCR assays for simultaneous detection and differentiation of monkeypox virus IIa, IIb, and I clades and the B.1 lineage. Biosaf Health. 2022;4(6):392–398.

2. Li Y, Zhao H, Wilkins K, Hughes C, Damon IK. Real‐time PCR assays for the specific detection of monkeypox virus West African and Congo Basin strain DNA. J Virol Methods. 2010;169(1):223–227.

### P485 Clinical, Epidemiological, Vaccination‐Related and Virological Features of Mpox Clade Ib and Contemporaneous Clade IIb Infections in Spain

Josep Riera^1^, Laura Fuertes^2^, Adrián Alfonso^3^, Adrià Mendoza^4^, Patricia Alvarez‐López^5^, Irene Fuertes^1^, Maria Carmen Ceballos^6^, Elena Sendagorta^3^, Angel Rivero^4^, Vicente Descalzo^5^, Alba Catala^1^, Alexy Inciarte ^1^, Ignacio Gadea^6^, Almudena Gutierrez‐Arroyo^7,8^, Susana Muñoz^4^, Maider Arando^5^, Esteban Martínez^9,10^, Jose L. Villanueva‐Cañas^11^, José Carlos Montañes^11^, Miguel Julian Martinez^12,13^, Sofia Perez‐del‐Pulgar^12^, Jose Luis Blanco^9,10^, on behalf of MPOX1bESP Study Team

^1^Department of Dermatology, Hospital Clinic, Barcelona, Spain; ^2^Department of Dermatology, Fundacion Jimenez‐Diaz, Madrid, Spain; ^3^Department of Dermatology, University Hospital La Paz, Madrid, Spain; ^4^Hospital Germans Trias I Pujol & PreP Point, Barcelona, Spain; ^5^Unitat de ITS/VIH Drassanes‐Vall d'Hebron, Servicio Infecciosas, Hospital Vall d'Hebron, Barcelona, Spain; ^6^Department of Dermatology, Fundacion Jimenez‐Diaz, Madrid, Spain; ^7^Department of Microbiology, University Hospital La Paz, Madrid, Spain; ^8^Grupo de investigación en Microbiología Molecular, Instituto de Investigación Sanitaria del Hospital Universitario La Paz, IdiPAZ, Madrid, Spain; ^9^Infectious Department, Hospital Clinic, Barcelona, Spain; ^10^Biomedical Research Networking Centre in Infectious Diseases, CIBERINFEC, Instituto de Salud Carlos III, Madrid, Spain; ^11^Hospital Clinic, Barcelona, Spain; ^12^Department of Microbiology, University Hospital Clinic, Barcelona, Spain; ^13^Biomedical Research Networking Centre in Liver and Digestive Diseases, CIBEREHD, Instituto de Salud Carlos III, Madrid, Spain

**Background**: Mpox virus clade Ib has recently emerged outside historically endemic regions, with locally acquired infections reported in Europe and concern about transmission within sexual networks. However, comparative data between clade Ib and contemporaneous clade IIb infections in European settings remain limited. We compared epidemiological, clinical, vaccination‐related and virological features of clade Ib and clade IIb mpox diagnosed in Spain.

**Methods**: We conducted a multicentre observational cohort study across five Spanish tertiary centres and specialized sexually transmitted infection units. Consecutive PCR‐confirmed mpox cases diagnosed between October 2025 and March 2026 were classified by viral clade and compared within the same epidemiological period. A historical Spanish clade IIb cohort from the 2022 outbreak was used for contextual comparison. Severe mpox was defined as hospitalization, death or clinically relevant mucosal or organ‐specific complications. Interclade recombination was assessed in available genomes.

**Results**: Forty‐eight cases were included: 20 clade Ib and 28 clade IIb infections (temporal distribution shown in Figure 1). Median age was 38 years (IQR 33–45), 45/48 patients (94%) were men, 19/48 (40%) were people living with HIV, and 24/48 (50%) had received at least one dose of modified vaccinia Ankara–Bavarian Nordic vaccine. Transmission occurred predominantly among gay, bisexual and other men who have sex with men. Epidemiological characteristics, vaccination status, anatomical distribution and mucosal involvement were broadly similar between clades (Table 1). Systemic symptoms were more frequent in clade Ib infection. Cutaneous involvement was universal, most often affecting genital and perianal areas. Severe mpox occurred in two cases, both with clade Ib infection; no intensive care admissions, deaths or organ‐specific complications were observed. Among clade Ib cases, unvaccinated individuals showed greater clinical burden, and all severe cases and hospital admissions occurred in unvaccinated patients. Compared with the 2022 clade IIb cohort, vaccination was more frequent in the contemporary cohort, and systemic symptoms were less common among vaccinated individuals. No evidence of interclade recombination was identified.

**Conclusions**: In this Spanish multicentre cohort, clade Ib and contemporaneous clade IIb mpox showed broadly similar clinical presentations. Severe outcomes occurred exclusively among unvaccinated clade Ib cases, supporting continued clinical and genomic surveillance, clade determination and targeted vaccination in populations at higher risk.

**FIGURE 1:**
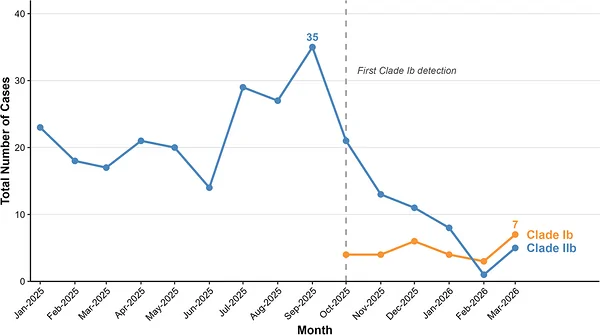
P485 | Temporal distribution of mpox cases across participating centres in Spain, January 2025 to March 2026.

**TABLE 1:**
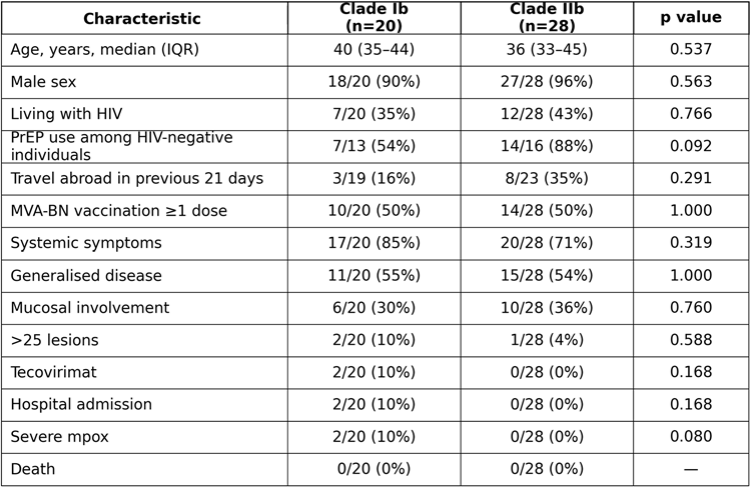
P485 | Key epidemiological and clinical characteristics of mpox cases stratified by viral clade. Data are presented as *n*/*N* (%) or median (IQR). *p*‐values compare clade Ib and clade IIb. Severe mpox was defined as hospital admission, death, or clinically relevant mucosal or organ‐specific complications. Generalized disease was defined as involvement of more than one anatomical region. Mucosal involvement included oropharyngeal or genito‐anorectal lesions. Percentages were calculated using available data.

### P486 Clinical and Virological Features of MPXV Clade Ib Versus Clade IIb Infection in the Italian MPOX‐ICONA Cohort

Valentina Mazzotta^1^, Rebecca Jo Steiner ^1^, Paola Stolfi^2^, Alessandro Tavelli^3,4^, Angelo Roberto Raccagni^5^, Maria Beatrice Valli^6^, Francesco Caruso^7^, Damiano Farinacci^1^, Flavia Passini^5^, Alessandro Mancon^8^, Neva Braccialarghe^1^, Nicola Clementi^9^, Nicholas Brian Bana^10^, Francesco Maria Fusco^11^, Lorenzo Biasoli^12^, Andrea Gori^7^, Antonella Castagna^5^, Fabrizio Maggi^6^, Andrea Antinori^13^, Licia Bordi^6^, Davide Moschese^7^, Silvia Nozza^5^

^1^Regional AIDS Reference Center, National Institute for Infectious Diseases, Lazzaro Spallanzani IRCCS, Rome, Italy; ^2^Istituto per le Applicazioni del Calcolo “Mauro Picone”, Consiglio Nazionale delle Ricerche, Rome, Italy; ^3^ICONA Foundation, Milan, Italy; ^4^National PhD Programme in One Health approaches to infectious diseases and life science research, Department of Public Health, Experimental and Forensic Medicine, University of Pavia, Pavia, Italy; ^5^Department of Infectious Diseases, IRCCS San Raffaele Scientific Institute, Vita‐Salute San Raffaele University, Milan, Italy; ^6^Laboratory of Virology, National Institute for Infectious Diseases, Lazzaro Spallanzani IRCCS, Rome, Italy; ^7^Department of infectious Diseases ‐ Luigi Sacco University Hospital, Milan, Italy; ^8^Laboratory of Microbiology and Bioemergencies ‐ Luigi Sacco University Hospital, Milan, Italy; ^9^Laboratory of Microbiology and Virology, IRCCS San Raffaele Hospital, Milan, Italy; ^10^Department of Infectious Diseases, ASST Grande Ospedale Metropolitano Niguarda, Milan, Italy; ^11^Infectious Diseases Unit, UOC Infezioni Sistemiche e dell'Immunodepresso, AORN Ospedali dei Colli, Naples, Italy; ^12^Clinic of Infectious Diseases, San Paolo Hospital, ASST Santi Paolo e Carlo, Milan, Italy; ^13^Health Direction, National Institute for Infectious Diseases Lazzaro Spallanzani IRCCS, Rome, Italy

**Background**: Following the global 2022 clade IIb mpox outbreak, the emergence of MPXV clade Ib has raised concerns regarding potential epidemiological, clinical and virological differences, but comparative data remain limited. We aimed to compare clinical and virological features of MPXV infection by clade.

**Methods**: MPOX‐ICONA is a prospective multicentre Italian cohort including laboratory‐confirmed mpox cases (2022–2026). Disease severity was defined as the presence of ≥1 of the following: involvement of two or more mucosal sites, ocular involvement, extended rash (≥10 lesions) or hospitalization. Demographic, epidemiological and clinical data were compared by clade using logistic regression. Time to healing was evaluated using Cox proportional hazard model. Ct‐values from samples with detectable MPXV‐PCR, used as a surrogate for viral load, were analysed using the Mann–Whitney U test and ANCOVA, adjusted for days from symptom onset.

**Results**: Among 689 participants (681 assigned male at birth [AMAB]; 636 GBMSM), 659 (95.6%) had clade IIb and 30 (4.4%) clade Ib infection. HIV status and CD4 count did not differ across groups. Compared with clade IIb, clade Ib infection was associated with lower odds of being AMAB, GBMSM, sexual transmission and mucosal involvement; conversely, higher odds were observed for extended rash, systemic symptoms, severe disease and any orthopoxvirus vaccination (Figure 1). Time to healing did not differ between clades (aHR 0.87, 95% CI: 0.60–1.26). HIV infection was independently associated with slower healing (0.54, 0.39–0.74; *p* < 0.001), while prior vaccination predicted faster recovery (1.44, 1.14–1.83; *p* = 0.002). Among PWH, higher CD4 count was associated with faster healing (per 100 CD4/µL increase: 1.06, 1.02–1.10). The lowest Ct value per patient across biological specimens collected at enrolment did not differ significantly by clade, after adjustment for time from onset (Table 1).

**Conclusions**: Clade Ib infection showed a distinct epidemiological and clinical profile, not exclusively linked to sexual transmission and with more extensive cutaneous involvement, but without evidence of higher viral load at presentation or a different healing rate compared with clade IIb. HIV infection and lower CD4 count were associated with slower recovery, whereas prior vaccination was associated with faster healing. These findings may help define the evolving spectrum of mpox.

**FIGURE 1:**
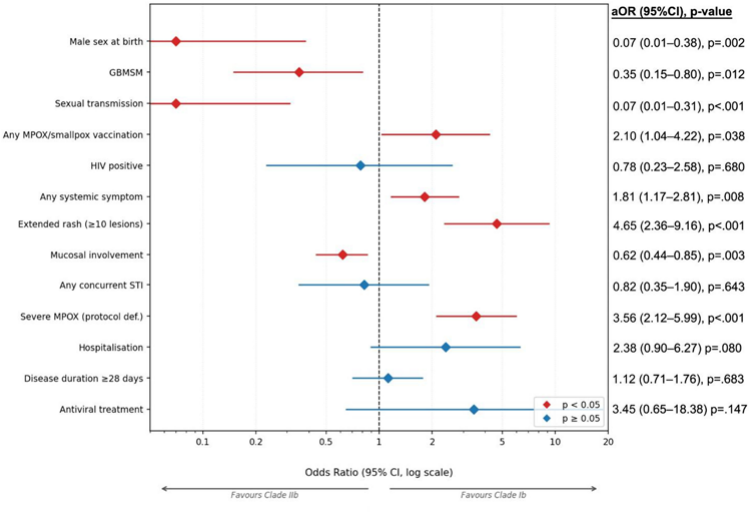
P486 | Adjusted odds ratios, clade Ib versus IIb (*N* = 689), adjusted for: age, HIV status, CD4 count, vaccination; SEs clustered by centre. Adjusted for: age at diagnosis, HIV status, CD4 count (continuous) and vaccination status. Standard errors clustered by clinical centre. Red diamonds: *p* < 0.05; blue: *p* ≥0.05.

**TABLE 1:**
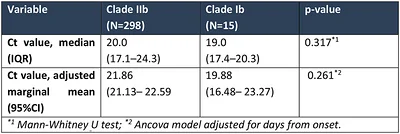
P486 | MPXV Ct values at enrolment by clade, using the lowest Ct value per patient across available biological specimens. Ct values were analysed among patients with detectable MPXV DNA and valid Ct data. When multiple biological specimens were collected at enrolment, the lowest Ct value per patient was selected. Adjusted marginal means were estimated using ANCOVA adjusted for time from symptom onset to specimen collection. A lower Ct value indicates higher viral DNA levels.

### P487 No Changes in HIV RNA Levels Following Mpox Vaccination Among People With HIV

Christian Hoffmann^1,2^, Jens Forthmann^1^, Carl Knud Schewe^1^, Michael Sabranski^1^, Axel Adam^1^, Stefan Hansen^1^, Stefan Fenske^1^, Katharina Cron^1^, Guido Schäfer ^1^

^1^ICH Study Center, Hamburg, Germany; ^2^II. Dept. of Medicine, UKSH, Campus Kiel, Germany

**Background**: Recent reports from small cohorts have suggested that people with HIV (PWH) might experience transient viraemia or confirmed virological failure (CVF) after mpox vaccination with modified vaccinia Ankara–Bavarian Nordic (MVA‐BN), prompting calls for intensified HIV‐RNA monitoring during vaccination.

**Methods**: In this single‐centre observational study, we included all PWH who received at least one MVA‐BN vaccination and had at least one HIV‐RNA measurement below 200 copies per mL in the year before vaccination during routine care. CVF was defined as HIV‐RNA greater than 1000 copies per mL or two consecutive values of at least 200 copies per mL. Among values below 200 copies per mL, we distinguished target not detected (TND), target detected with an HIV‐RNA below 40 copies per mL (TD) and low‐level viraemia (40–199 copies per mL); TD and low‐level viraemia were combined as detectable viraemia (DV). HIV‐RNA category distributions were compared across prespecified windows before and after dose 1 and dose 2, and associated factors were assessed in multivariable analysis.

**Results**: Between 2022 and 2025, 922 PWH (909 male and 13 transgender) received 1692 MVA‐BN doses. HIV‐RNA category distributions were similar across pre‐vaccination and post‐vaccination time windows, with no evidence of increased viraemia after vaccination (Figure 1). All seven CVF cases occurred in the context of treatment interruption or poor adherence rather than vaccination. After exclusion of individuals with CVF, peak HIV‐RNA before ART initiation of greater than 100,000 copies per mL (adjusted odds ratio [aOR] 2.85, 95% CI: 1.75–4.62; *p* < 0.001) and CD4 nadir below 200 cells per µL (2.11, 1.14–3.90; *p* = 0.017) were independently associated with DV. In contrast, continuous ART during and after primary HIV infection (0.22, 0.10–0.50; *p* < 0.001) and virological suppression for at least 10 years (0.53, 0.30–0.94; *p* = 0.03) were associated with lower odds of DV. PI‐based treatment versus INSTI showed a non‐significant trend (2.58, 0.97–6.86; *p* = 0.057).

**Conclusions**: In this large cohort, we found no evidence that MVA‐BN vaccination affected HIV‐RNA levels. Detectable low‐level viraemia was associated with pre‐existing virological, immunological and treatment‐related factors. These findings do not support intensified HIV‐RNA monitoring during mpox vaccination.

**FIGURE 1:**
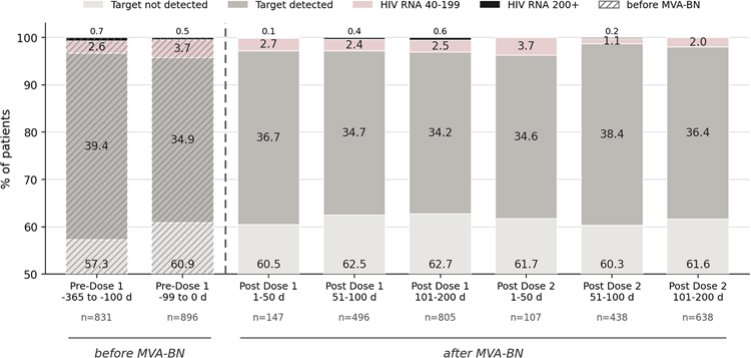
P487 | HIV RNA distribution across time windows before and after first and second MVA‐BN doses.

### P488 Emerging Mpox Outbreak in Verona, Italy: Incidence, Clinical Presentation and Determinants of Disease Severity

Andrea Tamanini^1^, Lorenza Lambertenghi^1^, Maddalena Cordioli^1^, Chiara Zanchi^2^, Arianna Bellati^1^, Anna Giulia Salvadori^1^, Federica Salfa^1^, Daria Pietroluongo^3^, Evelina Tacconelli^1^, Roberto Rossotti ^1^

^1^Infectious and Tropical Disease Department, Università degli Studi di Verona, Verona, Italy; ^2^Infectious and Tropical Disease Department, IRCCS Ospedale Sacro Cuore Don Calabria, Negrar di Valpolicella (Verona), Italy; ^3^Infectious and Tropical Disease Department, Università degli Studi di Napoli Federico II, Napoli, Italy

**Background**: Mpox represents an emerging clinical and diagnostic challenge. In Italy, the incidence was 0.16 cases per 100,000 inhabitants in 2025. Given the spread of clade Ib in the province of Verona in recent months, we aimed to evaluate the incidence and severity of the disease in our territory.

**Methods**: This retrospective analysis evaluated all mpox cases between December 2025 and June 2026 in the province of Verona, located in north‐eastern Italy. Demographic, behavioural and clinical features were analysed. Disease severity was estimated using the MPOX‐SSS score, and a Poisson regression model was applied to assess its determinants.

**Results**: During the study period, we registered 7 cases, resulting in an annualized rate of 1.5 cases per 100,000 inhabitants. All cases occurred in male subjects and were sexually transmitted. None of the patients had a concomitant sexually transmitted infection. Table 1 describes the main features of the study population. The median MPOX‐SSS value was 9. Notably, six of the seven patients reported scores greater than 8, the threshold defining a severe case. One patient with an AIDS‐defining illness died despite antiviral treatment with cART and tecovirimat. Using Poisson regression, we observed that white ethnicity and chemsex use were associated with lower disease severity. Conversely, immunosuppression, lack of vaccination and membership in the same cluster were associated with higher scores.

**Conclusions**: An ongoing mpox outbreak is affecting our Province, with an incidence exceeding the national average. The cases evaluated in our study were predominantly severe. Our findings indicate that immunocompetence and vaccination represent the most significant protective factors against severe disease.

**TABLE 1:**
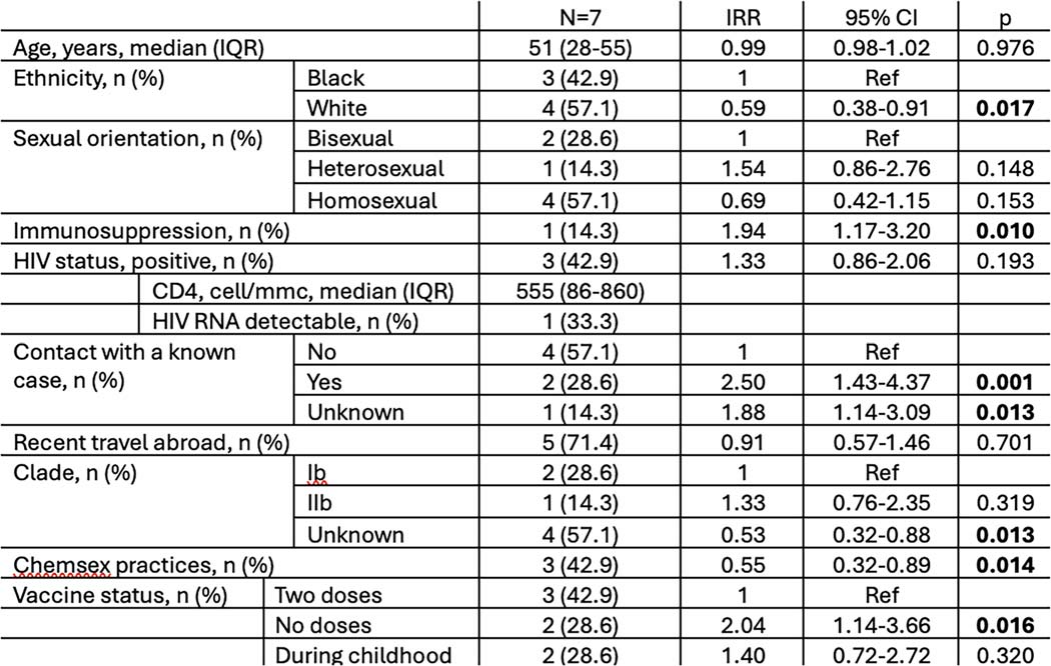
P488 | Demographic and clinical features of mpox cases.

## People Living With HIV and Sexually Transmitted Diseases

### P489 Tetracycline Resistance and Characteristics of *Neisseria gonorrhoeae* Infection in MSM With and Without Doxycycline Post‐Exposure Prophylaxis

Cristina Gómez‐Ayerbe ^1^, María López‐Jódar^1^, Salvador Martín‐Cortés^1^, Isabel A. Pérez Hernández^1^, Laura Mora^2^, María Blanco^1^, Rosario Palacios^1^, Jesús Santos^1^

^1^UEI, H. Virgen de la Victoria IBIMA, Malaga, Spain; ^2^Microbiology Department, H. Virgen de la Victoria IBIMA, Malaga, Spain

**Background**: *Neisseria gonorrhoeae* (NG) remains one of the most common sexually transmitted infections (STIs) among men who have sex with men (MSM). As doxycycline post‐exposure prophylaxis (DoxyPEP) is increasingly used for STI prevention, concerns regarding its potential impact on antimicrobial resistance have. We evaluated the clinical and microbiological characteristics of NG infections and factors associated with tetracycline resistance.

**Methods**: All diagnosed NG infections in MSM (on PrEP or PLWH) at risk of STIs undergoing regular STI screening from June 2022 to June 2026 were analysed. Cases without culture were excluded. Samples from pharyngeal, rectal and urethral/urine sites were collected by PCR and culture. Antimicrobial susceptibility testing was performed using E‐test methodology according to EUCAST recommendations. Clinical and epidemiological variables were analysed. Logistic regression was used to assess factors independently associated with tetracycline resistance. Statistical programme: jamovi 2.7.31.

**Results**: A total of 707 NG episodes were identified; 554 occurred in PrEP users and 153 in PLWH. Mean age was 37.8 years (SD 9.9). Most infections were asymptomatic (86.9%) and 14.0% were associated with a concomitant STI. Culture was positive in 190/707 episodes (26.6%). All isolates remained susceptible to ceftriaxone, while 126/190 (66.3%) were resistant to tetracycline. The proportion of tetracycline‐resistant isolates increased markedly over time, from 25.0% in 2022 to 96.2% in 2026. DoxyPEP use also increased during the study period (0%−35.8%). In univariable analysis, tetracycline resistance was more frequent among DoxyPEP users than in non‐users (80.0% vs. 60.7%; OR 2.6; *p* = 0.017). However, after stratification by calendar year, this association disappeared. In multivariable logistic regression adjusted for year of diagnosis, HIV status, clinical status, reinfection, anatomical site and DoxyPEP were not associated with tetracycline resistance (OR 0.55; *p* = 0.206). The year of diagnosis was the only variable independently associated with resistance.

**Conclusions**: A substantial increase in tetracycline‐resistant NG was observed between 2022 and 2026. Although DoxyPEP use appeared to be associated with resistance in crude analyses, this association was no longer observed after adjustment for temporal trends. Calendar year was the main determinant of resistance, suggesting that broader epidemiological changes rather than DoxyPEP use alone explain the observed increase.

### P490 Three Years of Real‐World DoxyPEP Use Among PrEP Users and People Living With HIV: Sustained Effectiveness, Safety and High Adherence

Salvador Martín‐Cortés^1^, Cristina Gómez‐Ayerbe ^2^, María López‐Jódar^2^, Andrea Prolo^2^, Marina Villalobos^2^, Andrés González‐Jiménez^3^, Jesús Santos^2^, Rosario Palacios^2^

^1^Unidad de Enfermedades Infecciosas, Hospital Universitario Virgen de la Victoria, IBIMA‐Plataforma Bionand, Universidad de Málaga, Malaga, Spain; ^2^Unidad de Enfermedades Infecciosas, Hospital Universitario Virgen de la Victoria, IBIMA‐Plataforma Bionand, Malaga, Spain; ^3^ECAI Bioinformática, IBIMA‐Plataforma Bionand, Malaga, Spain

**Background**: Randomized clinical trials have demonstrated high efficacy of DoxyPEP for preventing STIs among MSM. However, data on long‐term effectiveness, adherence and safety in clinical practice remain limited. We evaluated the real‐world impact of DoxyPEP in a high‐risk MSM cohort.

**Methods**: PrEP users and people with HIV (PWH) followed up at our clinic who reported risk for STIs were offered DoxyPEP between March 2023 and May 2026. DoxyPEP period extended from PrEP initiation to DoxyPEP initiation (PrEP users) and from entry into care or, when applicable, from the PrEP initiation date of the participant with the longest follow‐up (PWH). The post‐DoxyPEP period extended from DoxyPEP initiation until May/26, or discontinuation. Participants underwent screening for *Neisseria gonorrhoeae*, *Chlamydia trachomatis* and syphilis at baseline, scheduled visits and when symptomatic. Nasal and rectal swabs for multidrug‐resistant bacterial cultures were obtained baseline and after 12, 24 and 36 months. Incidence rates of first episodes of each STIs were compared between periods using Cox proportional hazards models. Statistical program: SPSS 21.0.

**Results**: Among 379 participants, 315 (83.1%) were PrEP users and 64 (16.9%) were PWH. Mean age was 39 ± 9.4 years. Median pre‐DoxyPEP and post‐DoxyPEP periods were 24 and 23 months, respectively. Incidence rates per 100 person‐years before and after DoxyPEP were 21.9 versus 2.3 for syphilis (*p* < 0.001), 35.6 versus 5.45 for chlamydia (*p* < 0.001) and 36.6 versus 27.3 for gonorrhoea (*p* = 0.08), Figure 1. DoxyPEP was discontinued in 56 participants (14.8%) mainly due to follow‐up loss, reduced STI risk, mild adverse events, transfer of care and medical indication. No clinically relevant adverse events were reported. Adequate adherence was documented in 84.6%, 88.4% and 88.3% of participants at 12, 24 and 36 months, respectively. Baseline multidrug‐resistant bacterial cultures were available for 330 participants, 44 (13.3%) positive results; at the end of follow‐up, from 48 available cultures, seven (14.6%) were positive.

**Conclusions**: Our data demonstrate the effectiveness, safety and durability of DoxyPEP in a real word setting, significantly reducing the incidence of syphilis and chlamydia over 3 years. High long‐term adherence and low discontinuation rates further support its implementation among individuals at ongoing risk of bacterial STIs. Continued surveillance of antimicrobial resistance and microbiome changes remains warranted.

**FIGURE 1:**
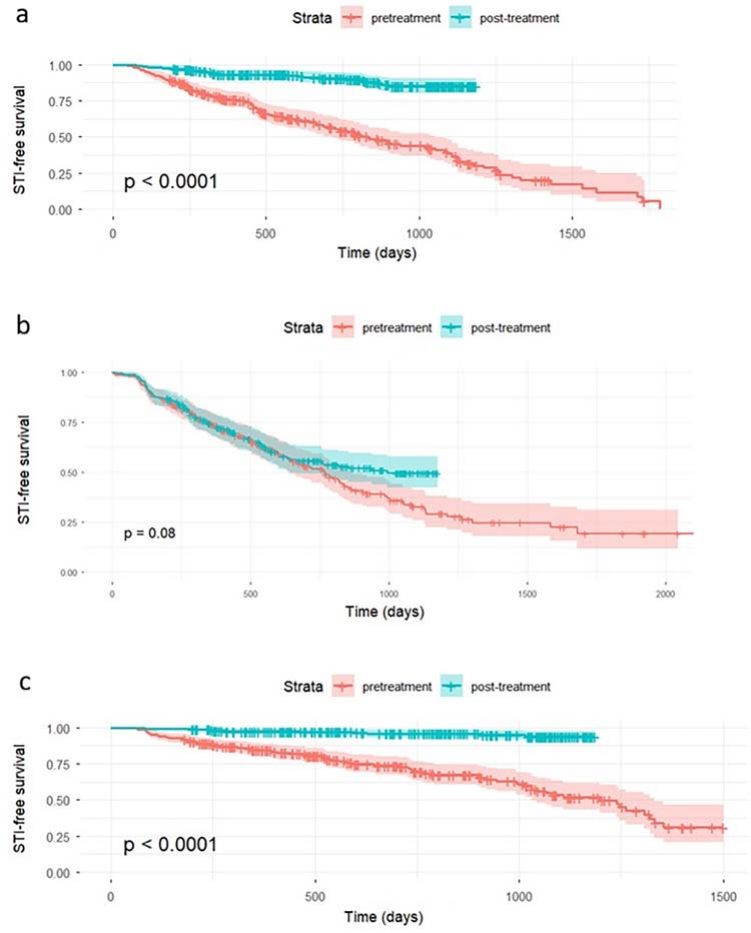
P490 | Kaplan–Meir survival curve for CT (a), NG (b) and syphilis (c).

### P491 Implementation of Routine HPV Screening in HIV Care: High Acceptability and Clinical Yield in a Real‐World Cohort of People With HIV

Layla Pagnucco ^1^, Semela Resuli^2^, Sofia Frattola^2^, Raffaele Bruno^1,2^, Roberto Gulminetti^1^

^1^Infectious Diseases Unit, Medical Department, Fondazione IRCCS Policlinico San Matteo University of Pavia, Pavia, Italy; ^2^Department of Medicine, Surgical, Diagnostic and Pediatric Science, University of Pavia, Pavia, Italy

**Background**: People with HIV (PWH) are at increased risk of persistent human papillomavirus (HPV) infection and HPV‐related malignancies. While cervical HPV screening is routinely recommended in women, HPV testing at other anatomical sites is not routinely incorporated into HIV care. We evaluated the feasibility of implementing routine HPV screening in an HIV outpatient clinic and assessed its clinical yield in a real‐world cohort.

**Methods**: We conducted a cross‐sectional implementation study among PWH attending routine outpatient visits between February and May 2026. Participants were offered physician‐collected oral swabs and self‐collected anal and vaginal swabs, according to sex and anatomical site. Feasibility was assessed through screening uptake and acceptability of self‐sampling. Clinical yield was evaluated by HPV genotype prevalence and distribution.

**Results**: Among 246 PWH offered screening, only eight declined participation, yielding an acceptance rate of 96.7%. Anal swabs showed the highest diagnostic yield, with 158 positive samples. High‐risk (HR) HPV genotypes accounted for 72.8% of infections, including HPV‐16 in 21 participants and HPV‐18 in four. Intermediate‐risk, low‐risk and unclassified genotypes represented 9.5%,14.6% and 3.2% of detections, respectively. Of 60 vaginal swabs, 33 were HPV‐positive; 69.7% contained HR‐genotypes, including HPV‐16 in five participants, while 3.0% and 27.3% were intermediate‐ and low‐risk types, respectively. Oral HPV infection was uncommon, with eight positive samples (HPV‐16, *n* = 3; HPV‐58, *n* = 2; HPV‐18, HPV‐56 and HPV‐120, *n* = 1 each). Self‐collection was well accepted, supporting the operational feasibility of integrating HPV screening into routine HIV care. Participants without prior HPV vaccination were offered vaccination. Individuals with HR‐HPV‐positive anal swabs were referred for proctological assessment including anoscopy; among 23 who underwent the procedure, three were diagnosed with high‐grade squamous intraepithelial lesions (HSIL) and treated surgically. Women with positive cervical/vaginal findings were referred for gynaecological evaluation, while referral pathways with otolaryngology specialists were established for oral HPV‐positive PWH.

**Conclusions**: Routine HPV‐screening in an HIV outpatient clinic was highly feasible, with excellent patient acceptance and successful integration into routine care. The high prevalence of HR‐HPV, particularly in anal and vaginal specimens, highlights the potential value of systematic screening to identify individuals at increased risk of HPV‐related disease and facilitate timely preventive interventions. While established management pathways exist for anal and cervical disease, standardized approaches for oral HPV infection remain to be defined.

### P492 When HIV Prevention Meets Sexually Transmitted Infections (STIs) Resurgence: The Paradox of Treatment as Prevention (TasP) and Pre‐Exposure Prophylaxis (PrEP)

Barbara Bellocchi ^1^, Alessandro Franzò^2^, Laura Todaro^3^, Laura Pappalardo^4^, Andrea Marino^1^, Mariagiovanna Coco^1^, Vittoria Moscatt^1^, Federica Cosentino^1^, Eugenia Pistarà^1^, Serena Spampinato^2^, Nunziatina Villari^1^, Bruno Santi Cacopardo^1^, Giuseppe Nunnari^1^, Benedetto Maurizio Celesia^1^

^1^Unit of Infectious Diseases, Department of Clinical and Experimental Medicine, ARNAS Garibaldi Hospital, University of Catania, Catania, Italy; ^2^Department of Clinical and Experimental Medicine, ARNAS Garibaldi Hospital, University of Catania, Catania, Italy; ^3^Unit of Infectious Diseases, Azienda Sanitaria dell'Alto Adige, Bolzano, Italy; ^4^Faculty of Medicine and Surgery Department of Experimental and Clinical Medicine, University of Catania, Catania, Italy

**Background**: Pre‐exposure prophylaxis (PrEP) has revolutionized HIV prevention but there are still concerns it would reduce condom use thereby increasing other sexually transmitted infections (STIs). This study assesses the impact of reimbursable PrEP programmes on the increased overall syphilis infections.

**Methods**: Data was collected from a single‐centre retrospective observational study, reviewing medical records from January 2014 and December 2025. Demographic and epidemiological variables, HIV serological status, clinical stage of the disease at the time of diagnosis, and any PrEP taken were recorded for each person. The outcomes were compared between the pre‐reimbursable PrEP and post‐reimbursable PrEP periods in Italy (May 2023).

**Results**: A total of 759 syphilis cases were analysed. In the pre‐reimbursable PrEP period, 47.5% of syphilis new diagnoses and treatments were people living with HIV (PLWH), with men who have sex with men (MSM) accounting for 52.3% of the cases. Late latent syphilis was the most frequent clinical presentation (18.9%) and the reinfection rate was 25.8%. In the post‐reimbursable PrEP period, the proportion of PLWH decreased to 39.2%, while MSM represented 72.4% of cases. Early latent syphilis became the predominant clinical presentation (19.3%), showing a significant increase compared to the pre‐reimbursable PrEP period (*p* < 0.001). The reinfection rate also rose significantly to 40.8% (*p* < 0.001) (Table 1).

**Conclusions**: PrEP introduction coincided with perceived reduced vulnerability to STIs, reflecting in rising syphilis incidence. The predominance of latent cases underscores the importance of systematic screening and early intervention. PrEP use may lower perceived risk thereby facilitating syphilis transmission. At the same time, PrEP programmes have expanded STIs screening, increasing the detection of asymptomatic latent infections and apparently contributing to the rise in incidence. Moreover, PLWH with sustained viral suppression may perceive a lower risk for other STIs under the U = U principle (undetectable = untransmittable), therefore, further influencing behaviours. Lastly, STIs act as a proxy for condomless sex and reveal a “hidden” population—neither PLWH nor on PrEP—who engage in similar high‐risk behaviours but remain outside structured prevention and testing pathways, empowering silent transmission. In conclusion, integrated strategies combining PrEP, STIs screening, targeted education and early treatment altogether are all major contributing factors in preventing further spread of STIs.

**TABLE 1:**
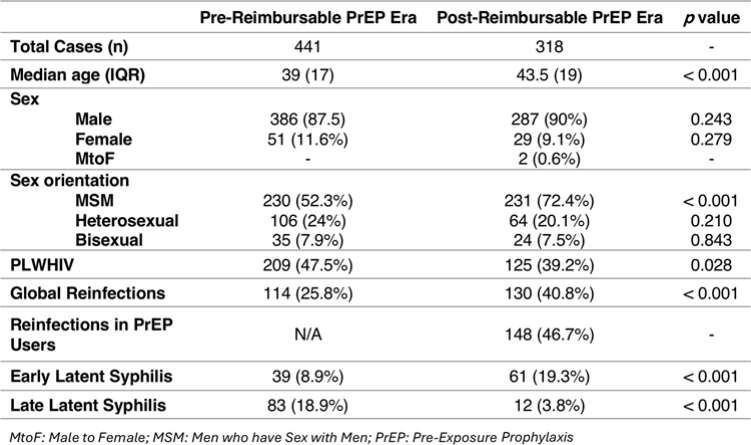
P492 | Epidemiological and clinical shift in syphilis diagnoses (2014–2025).

### P493 Syphilis Serological Dynamics After Penicillin Versus Doxycycline Treatment Among People Living With HIV in Milan, Italy

Angelo Roberto Raccagni ^1,2^, Domenico Morace^2^, Nicolò Capra^1^, Riccardo Lolatto^1^, Serena Rolla^3^, Diana Canetti^1^, Emanuela Messina^1^, Vincenzo Spagnuolo^1,2^, Tommaso Clemente^1,2^, Antonella Castagna^1,2^, Silvia Nozza^1,2^

^1^Infectious Diseases Unit; IRCCS San Raffaele Scientific Institute, Milan, Italy; ^2^Vita‐Salute San Raffaele University, Milan, Italy; ^3^Serology Unit; IRCCS San Raffaele Scientific Institute, Milan, Italy

**Background**: Serological follow‐up after syphilis treatment relies on rapid plasma regain (RPR) titres, but response kinetics in people living with HIV (PLWH) may vary by syphilis stage, immune status and treatment regimen. We evaluated time to serological resolution after penicillin or doxycycline treatment in a real‐world cohort.

**Methods**: Cohort study including PLWH with laboratory‐confirmed syphilis between January 2008 and March 2026 at IRCCS San Raffaele Scientific Institute, Milan, Italy. Each episode with ≥1 post‐treatment RPR measurement was included; multiple episodes per individual were analysed separately. Treatment was prescribed according to international guidelines; doxycycline was used in case of penicillin allergy, drug shortages or logistic issues. Serological resolution was defined as a ≥4‐fold decline in RPR titre from baseline. Cox proportional hazards models with a gamma frailty term accounted for repeated episodes and were adjusted for age, baseline RPR titre, nadir and baseline CD4^+^ count, and history of previous syphilis.

**Results**: Overall, 1827 syphilis episodes among 1025 PLWH were included: 437 primary, 336 secondary, 383 early latent and 671 late latent (Table 1). Penicillin was used in 1697/1827 (92.9%) episodes and doxycycline in 130/1827 (7.1%). Median follow‐up was 7.8 years (interquartile range [IQR] 4.2–12.0), without follow‐up or baseline RPR titres differences across treatment groups. Serological resolution occurred in 1744 episodes (95.5%): 1627/1697 (95.9%) after penicillin and 117/130 (90.0%) after doxycycline. Resolution within 6, 12 and 24 months was 53.2%, 84.1% and 92.8% with penicillin, versus 41.5%, 76.9% and 84.6% with doxycycline. In primary syphilis, penicillin was independently associated with faster resolution at 6 months (adjusted hazard ratio [aHR] 2.77, 95% confidence interval [CI] 1.38–5.56; *p* = 0.004), 12 months (aHR 1.99, 95% CI: 1.17–3.36; *p* = 0.011) and 24 months (aHR 2.11, 95% CI: 1.27–3.49; *p* = 0.004). No significant treatment‐related differences were observed in secondary, early or late latent syphilis (Figure 1).

**Conclusions**: Most syphilis episodes in people living with HIV achieved serological resolution regardless of treatment. Penicillin was associated with faster resolution in primary syphilis compared to doxycycline, due to possible higher treponemicidal activity, whereas outcomes were broadly comparable in other stages. Syphilis serological response should be interpreted according to disease stage and treatment context.

**FIGURE 1:**
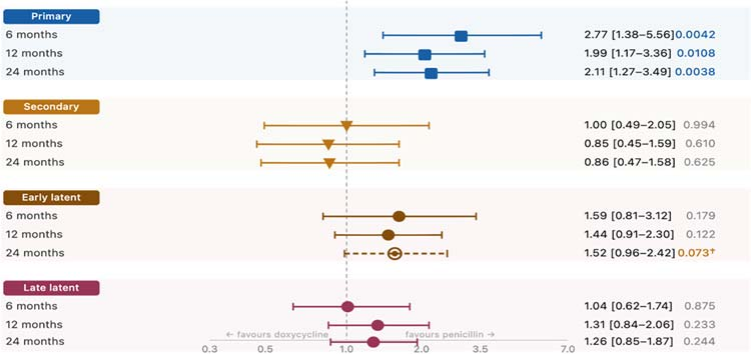
P493 | Adjusted hazard ratios for serological resolution of syphilis by disease stage at 6, 12 and 24 months (left favours doxycycline, right favours penicillin).

**TABLE 1:**
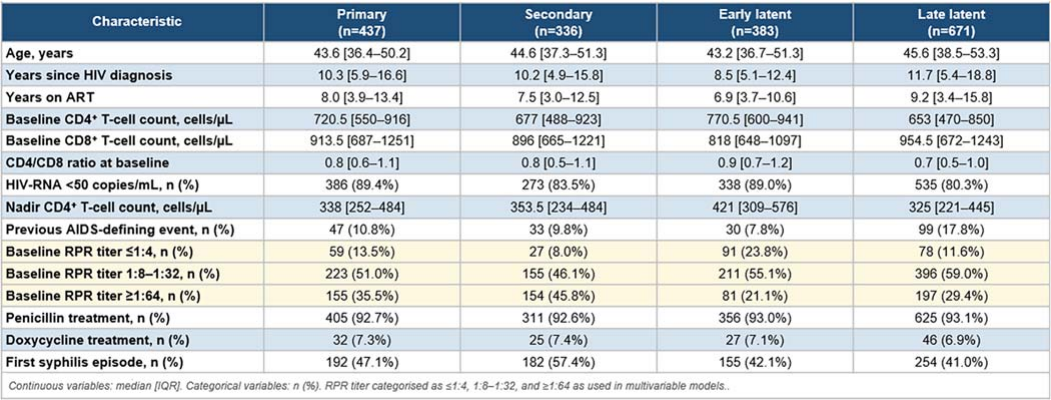
P493 | Baseline characteristics of syphilis episodes according to clinical stage.

### P494 Abstract Withdrawn

### P495 HPV Awareness Among People Living With HIV

Aysegul Inci Sezen^1^, Yusuf Emre Ozdemir^1^, Esra Ensaroglu^1^, Samiha Akkaya^1^, Deniz Borcak ^1^, Sevtap Senoglu^2^, Kadriye Kart Yasar^1^

^1^Department of Infectious Diseases and Clinical Microbiology, Bakirkoy Dr. Sadi Konuk Training and Research Hospital, University of Health Sciences, Istanbul, Türkiye; ^2^Department of Infectious Diseases and Clinical Microbiology, Medipol Bahcelievler Hospital, Istanbul, Türkiye

**Background**: HPV vaccination is recognized as an effective and reliable strategy for preventing HPV‐related cancers. Among people living with HIV (PLWH), HPV infections are more common, persist for longer periods and are associated with higher rates of HPV‐related malignancies than in the general population. Consequently, increasing awareness of HPV and promoting vaccination among PLWH are critically important. This study evaluated HPV vaccine awareness, knowledge levels and vaccine uptake among PLWH and identified factors associated with adequate knowledge and vaccination status.

**Methods**: This is a cross‐sectional, descriptive study. The study population is PLWH who were followed up at the Bakırköy Dr Sadi Konuk Training and Research Hospital outpatient clinic between 1 December 2025 and 1 February 2026.

**Results**: A total of 200 PLWH participated in the study. The mean age was 37.4 ± 8.8 years. In terms of gender, 87.5% (*n* = 175) identified as men, 11.0% (*n* = 22) as women and 1.5% (*n* = 3) as another gender identity. Regarding sexual orientation, 39.5% (*n* = 79) identified as heterosexual, 39.0% (*n* = 78) as men who have sex with men (MSM) and 21.5% (*n* = 43) as bisexual. Overall, 30% of participants demonstrated adequate knowledge of HPV vaccination. The only factor significantly associated with adequate knowledge was educational attainment (*p* < 0.005; OR 2.24) (Table 1). Among unvaccinated participants, 66% reported that they would accept vaccination if it were provided free of charge by the government, while 74% indicated that a healthcare professional's recommendation would be the key factor influencing their decision to be vaccinated.

**Conclusions**: This cross‐sectional study found a very low HPV vaccination rate (9.5%) among PLWH. The low uptake of HPV vaccination in this population, which is at increased risk of HPV‐related infections and malignancies, suggests the presence of substantial barriers to vaccine access and delivery in our country. Educational attainment was the only independent predictor of adequate knowledge regarding HPV vaccination. This finding suggests that awareness of HPV and its prevention is closely associated with education level and health literacy. Our study also demonstrated that cost plays a significant role in vaccination decision‐making. On the other hand, our findings underline the key role of healthcare professionals in promoting HPV vaccination. Improving HPV vaccination uptake among PLWH requires effective clinician counselling, accessible vaccination programmes and targeted educational strategies.

**TABLE 1:**
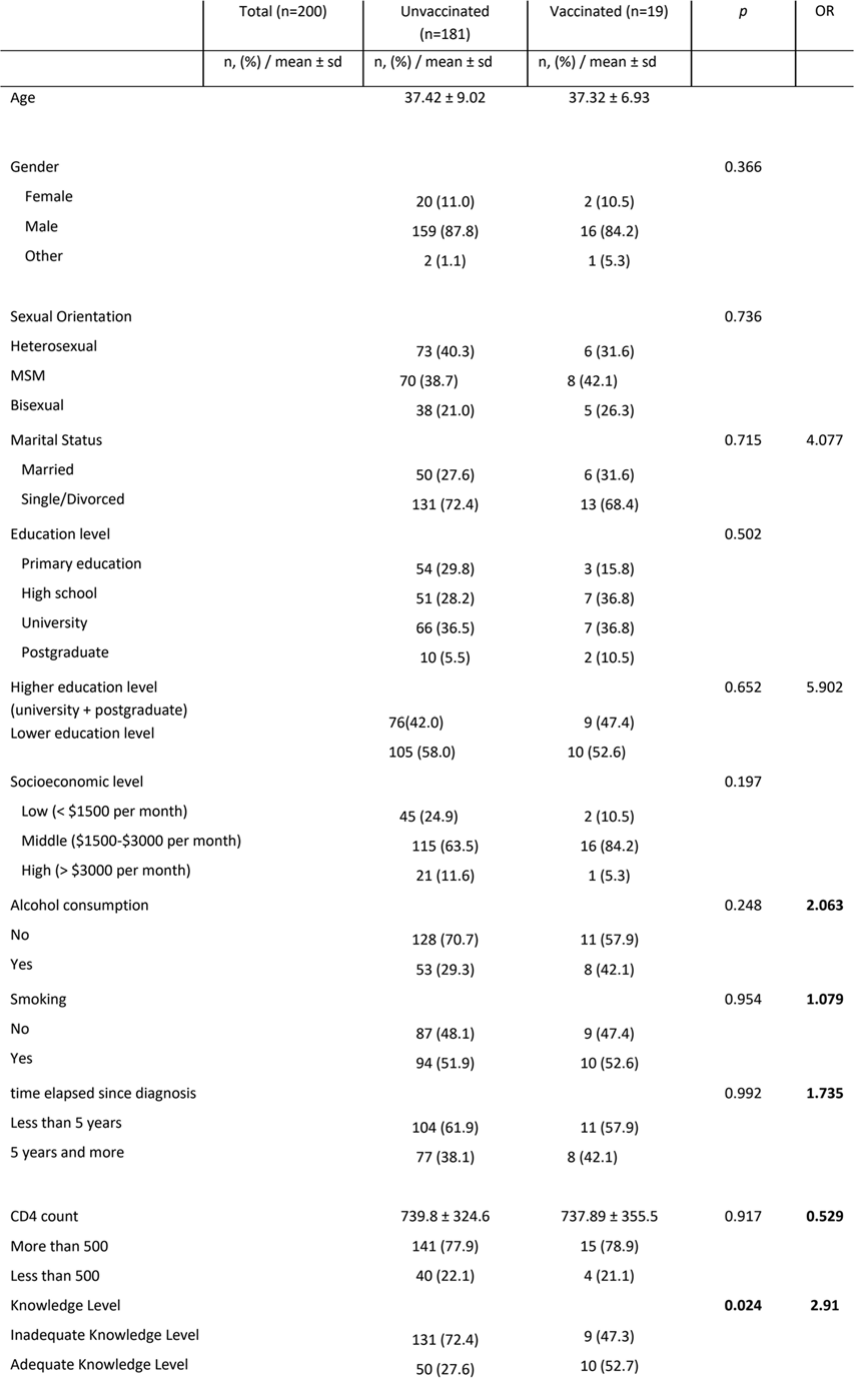
P495 | Comparison of HPV‐vaccinated and unvaccinated PLWH.

### P496 Abstract Withdrawn

## People Living With HIV and Tuberculosis

### P497 Prevalence of Tuberculosis Infection and Associated Risk Factors Among People With HIV and HIV‐Negative Controls in Lithuania

Sarunas Raudonis ^1^, Birute Nakceriene^2^, Rasa Girdziuniene^3^, Aidas Kausas^4^, Daiva Velyvyte^5,6^, Aldona Eimutiene^3^, Gertruda Arstikaitiene^4^, Kotryna Vagoraite^1,7^, Vilnele Lipnickiene^8^, Jelena Kopeykiniene^9^, Raimonda Matulionyte^1,7^, on behalf of WEEPI Collaboration Group

^1^Vilnius University, Faculty of Medicine, Vilnius, Lithuania; ^2^Department of Programs and State Tuberculosis Information System, Vilnius University Hospital Santaros Klinikos, Vilnius, Lithuania; ^3^Department of Infectious Diseases, University Hospital of Klaipeda, Klaipeda, Lithuania; ^4^Clinic of Conservative Medicine, Adult Infectious Diseases Unit, Republican Siauliai County Hospital, Siauliai, Lithuania; ^5^Department of Infectious Diseases, Lithuanian University of Health Sciences, Kaunas, Lithuania; ^6^Kaunas Hospital of the Lithuanian University of Health Sciences, Kaunas, Lithuania; ^7^Centre of Infectious Diseases, Vilnius University Hospital Santaros Klinikos, Vilnius, Lithuania; ^8^National Public Health Surveillance Laboratory, Vilnius, Lithuania; ^9^Department of Diagnostics, University Hospital of Klaipeda, Klaipeda, Lithuania

**Background**: Tuberculosis (TB) incidence in Lithuania declined from 54.6 to 23.1 per 100,000 population between 2014 and 2024 but remains above the EU/EEA average (8.4 per 100,000), and TB is the most common AIDS‐defining illness. People with HIV (PWH) are at increased risk of progression from tuberculosis infection (TBI) to active TB. We assessed TBI prevalence and associated risk factors in PWH and HIV‐negative controls and identified groups at increased risk.

**Methods**: A cross‐sectional study was conducted at four geographically distinct Lithuanian infectious diseases centres during WEEPI 1.1 (08/2018–05/2022) [1] and 1.2 (03/2023–02/2026). TBI was defined as a positive interferon‐gamma release assay (IGRA) and/or tuberculin skin test (TST). Participants completed a structured questionnaire. Risk factors were evaluated using univariable and multivariable logistic regression adjusted for age and sex, IGRA–TST agreement evaluated by Cohen's κ.

**Results**: The study included 743 PWH (median age 41, IQR 35–48; 69.7% men) and 1456 HIV‐negative controls (median age 43, IQR 36–51; 61.1% men). TBI prevalence was similar in PWH and controls (15.6% vs. 14.9%; OR 1.06, 95% CI: 0.83–1.35; *p* = 0.65). Univariable associations are shown in Table 1. In multivariable analysis of all participants, TBI was independently associated with injecting drug use (aOR 2.34), previous incarceration (aOR 1.83), non‐higher education (aOR 1.57) and HIV status (aOR 1.65, all *p* ≤ 0.003), with HIV becoming significant after adjustment. Among PWH, injecting drug use (aOR 2.33) and a CD4/CD8 ratio ≥0.5 at HIV diagnosis (aOR 2.01) were independently associated with TBI (Figure 1). Among controls, injecting drug use (aOR 2.53), previous incarceration (aOR 1.87), non‐higher education (aOR 1.57) and male sex (aOR 1.55) were independently associated with TBI (all *p* ≤ 0.025). IGRA–TST concordance was fair in PWH (κ = 0.26, 95% CI: 0.13–0.39) and controls (κ = 0.21, 95% CI: 0.07–0.35). IGRA detected more TBI than TST in PWH (15.3% vs. 6.3%; *p* < 0.001) but not in controls (9.6% vs. 9.3%; *p* = 1.000).

**Conclusions**: TBI prevalence in both groups exceeded the European estimate (12.2%) [2]. HIV status, injecting drug use, previous incarceration and low education were independent determinants of TBI, highlighting priority groups for targeted screening and preventive treatment.

**References**

1. Matulyte E, Kancauskiene Z, Kausas A, Urboniene J, Lipnickiene V, Kopeykiniene J, Gudaitis T, Raudonis S, Danila E, Costagliola D, Matulionyte R. Latent tuberculosis infection and associated risk factors among people living with HIV and HIV‐uninfected individuals in Lithuania. Pathogens. 2023;12(8):990.

2. Cohen A, Mathiasen VD, Schön T, Wejse C. The global prevalence of latent tuberculosis: A systematic review and meta‐analysis. Eur Respir J. 2019;54(3):1900655.

**FIGURE 1:**
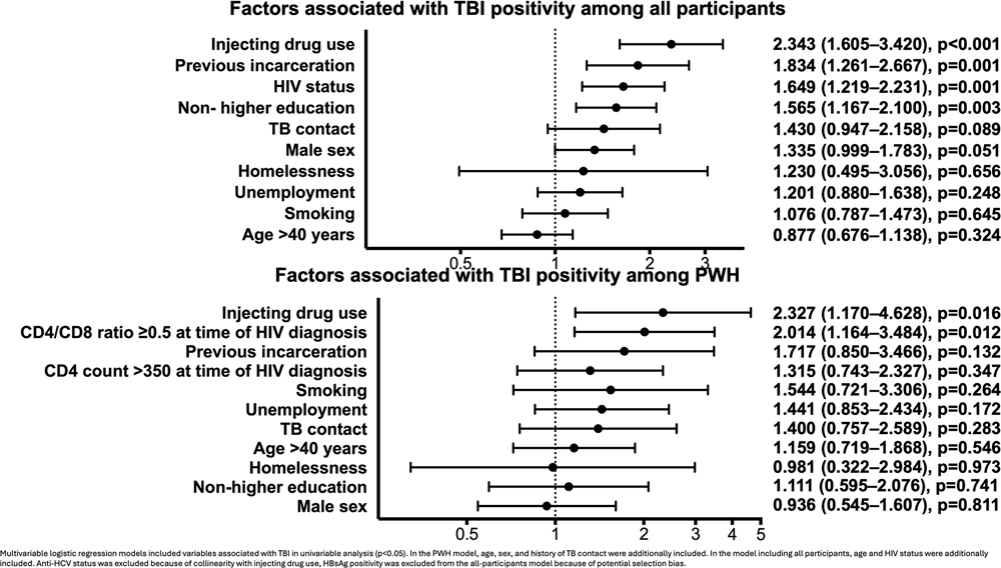
P497 | Multivariable analysis of factors associated with TBI among all participants and PWH.

**TABLE 1:**
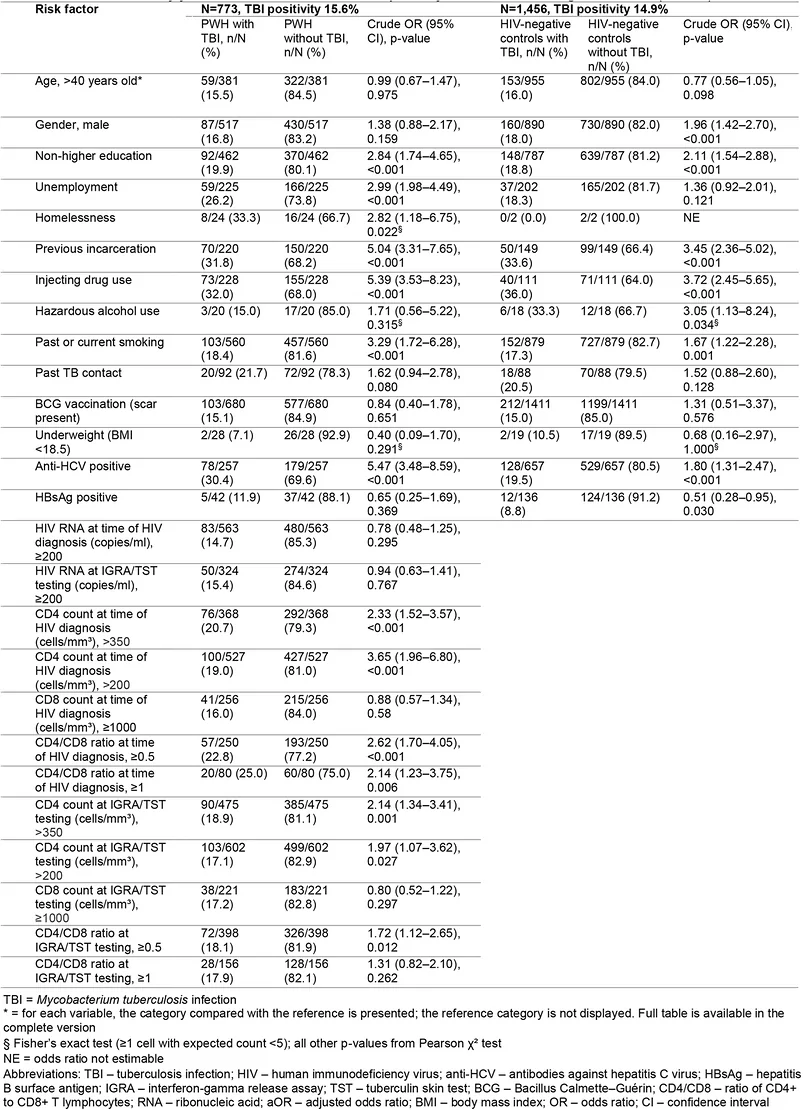
P497 | Univariable analysis of factors associated with TBI positivity in PWH and HIV‐negative controls (*n* = 2199).

### P498 Abstract Withdrawn

### P499 Abstract Withdrawn

### P500 High Burden of HIV and Sexually Transmitted Coinfections Among Men Admitted for Tuberculosis at a Referral Infectious Diseases Hospital in Buenos Aires: Seroprevalence and Diagnostic Yield of Admission‐Based Screening

Gabriela Poblete ^1^, Yasmin El Kozah^2^, Nahuel Alberdi^3^, Fernanda Sarralde^1^, Karin Semorile^1^, Guillermo María Onis^1^, Natalia Pacífico^1^

^1^Hospital de Infecciosas Francisco J. Muñiz, Buenos Aires, Argentina; ^2^Centro Médico Accord (CEMAC), Buenos Aires, Argentina; ^3^Independent Biostatistician, Buenos Aires, Argentina

**Background**: People with tuberculosis (TB) face a disproportionate burden of HIV, viral hepatitis and sexually transmitted infections (STIs) due to overlapping epidemiological and social vulnerabilities [1]. Hospitalization offers an opportunity for integrated screening among populations with limited access to primary healthcare [2]. We evaluated the seroprevalence of HIV, hepatitis B virus (HBV), hepatitis C virus (HCV), hepatitis A virus (HAV) and syphilis among men admitted to a TB ward and assessed the diagnostic yield of admission‐based screening.

**Methods**: Retrospective cross‐sectional study of adult men admitted to a TB ward at a public referral infectious diseases hospital in Buenos Aires, Argentina, between December 2025 and May 2026. Protocol included HBsAg and anti‐HBc testing, anti‐HBs was added when anti‐HBc was reactive. HIV testing was offered to individuals without a previous diagnosis. A social vulnerability index (SVI) included substance use, homelessness, incarceration, unemployment and harmful alcohol use. Syphilis was classified as active (anti‐Tp+/VDRL+) or latent (anti‐Tp+/VDRL−). Proportions are presented with 95% confidence intervals (CI).

**Results**: Fifty‐two men were included (median age 36 years [IQR 30–46]); 36.5% had an SVI ≥4. HIV prevalence was 61.5% (32/52, 95% CI: 47.0–74.7%). Among 26 individuals with a prior HIV diagnosis, only 8 (30.8%) were receiving ART; median CD4 count was 82 cells/mm^3^ (IQR 33–206), and 73.1% had CD4 < 200 cells/mm^3^. Six of 26 HIV‐naïve individuals were newly diagnosed (23.1%, 95% CI: 9.0–43.6%), all with advanced disease (median CD4 34 cells/mm^3^; median HIV RNA 5.85 log_10_ copies/mL). Treponemal seroprevalence was 17.3% (9/52), including 5 active and 4 latent infections; all occurred among people living with HIV. Anti‐HCV was reactive in 3/51 (5.9%), anti‐HBc in 7/51 (13.7%), including one HBsAg‐positive individual, and 7/41 (17.1%) were susceptible to HAV. Overall, 15/52 admissions (28.8%, 95% CI: 17.1–43.1%) yielded at least one new diagnosis (Table 1).

**Conclusions**: Men admitted with TB carry an extraordinary burden of HIV and STI co‐infections. Hospitalization for TB in high‐vulnerability settings represents a high‐yield opportunity for integrated screening. Integrated screening for HIV, viral hepatitis and syphilis among vulnerable populations can identify a significant proportion of individuals with active infections [3].

**References**

1. GBD Collaborators. Global burden of disease study [título completo del artículo]. Public Health. 2025;231:104–115.

2. World Health Organization. *Consolidated guidelines on tuberculosis. Module 6*. Geneva: World Health Organization; 2024.

3. Saleem K, et al. Integration of sexual health and HIV services: A systematic review. Lancet HIV. 2024;11(4):e230–e242.

**TABLE 1:**
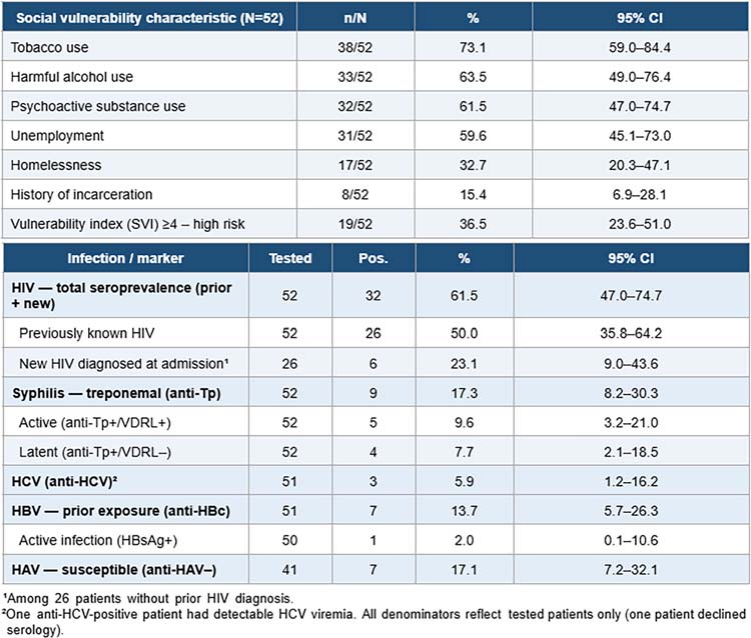
P500 | Social vulnerability profile and seroprevalence of HIV and co‐infections (*N* = 52).

### P501 Early Neutrophil‐to‐Lymphocyte Ratio Dynamics at Anti‐Tuberculosis Treatment Initiation as a Bedside Marker of Subsequent Clinical Complications in Hospitalized People With HIV/TB Co‐Infection

Electra Stravodimou, Elli Kouriannidi, Christos Mamarelis, Vasiliki Rapti, Kalomira Athanasiou, Vasileios Maravitsas, Eleni Kakalou, Vissaria Sakka, Garyphallia Poulakou

3rd University Department of Internal Medicine, National Kapodistrian University of Athens, Athens, Greece

**Background**: The early treatment period of HIV/tuberculosis (HIV/TB) coinfection is frequently complicated by inflammatory or treatment‐related events that require additional investigations, treatment modifications and prolonged hospitalization. Such events may occur after anti‐tuberculosis treatment but before antiretroviral therapy (ART), potentially postponing ART initiation. The neutrophil‐to‐lymphocyte ratio (NLR), an inexpensive marker derived from routine complete blood counts, is associated with advanced HIV disease and tuberculosis severity, but its role during this early phase is poorly characterized. We explored whether early NLR dynamics are associated with subsequent clinical complications in hospitalized people with HIV/TB coinfection.

**Methods**: We conducted a retrospective pilot cohort study including 10 consecutive hospitalized adults with HIV/TB coinfection at a tertiary referral centre. Demographics, CD4 T‐cell count, HIV viral load, tuberculosis extent, anti‐tuberculosis treatment and ART initiation dates, event dates and NLR values at these timepoints were recorded. Complications included unexplained fever, rash, drug hypersensitivity with hypereosinophilia, thrombocytopenia, lymphadenopathy and drug‐induced liver injury. Events preceding any treatment initiation were excluded. Continuous variables are presented as medians (IQR), categorical variables as numbers (%) and exploratory comparisons used exact Mann–Whitney U tests.

**Results**: Ten male patients were included. Median age was 33.5 years (31.3–39.0) and median CD4 count 131.5 cells/µL (18.0–250.2). Disseminated tuberculosis was present in 6/10 (60%) patients. Eight patients (80%) developed at least one complication. Patients with complications had higher NLR at anti‐tuberculosis treatment initiation than those remaining event‐free (10.01 [3.39–17.78] vs. 1.20 [1.15–1.25]; exact *p* = 0.044) (Figure 1). Median NLR at ART initiation was 2.63 (2.28–3.61), and median NLR across complications was 4.58 (2.41–6.24). Among patients who started ART after anti‐tuberculosis treatment (*n* = 9), the median interval was 24 days (14–62). Overall, 8/12 evaluable post‐treatment events (66.7%) occurred before or on the day of ART initiation, a median of 17 days (10–47) after anti‐tuberculosis treatment initiation.

**Conclusions**: Higher NLR values were observed among patients who subsequently developed clinical complications. The predominance of events before ART initiation suggests that the initial anti‐tuberculosis treatment period is a clinically vulnerable window that may delay ART initiation. NLR cannot determine event causality, but may support bedside risk stratification and closer monitoring. Larger prospective studies are warranted.[Fig jia270211-fig-0572]

**FIGURE 1:**
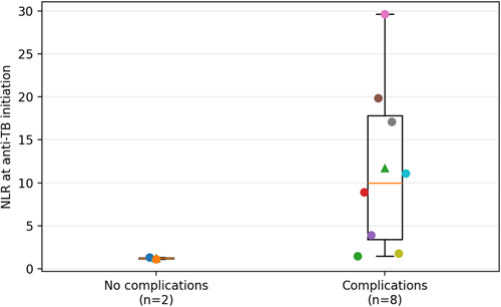
P501 | Baseline NLR at anti‐tuberculosis treatment initiation by subsequent clinical complication status.

**TABLE 1 jia270211-fig-0572:**
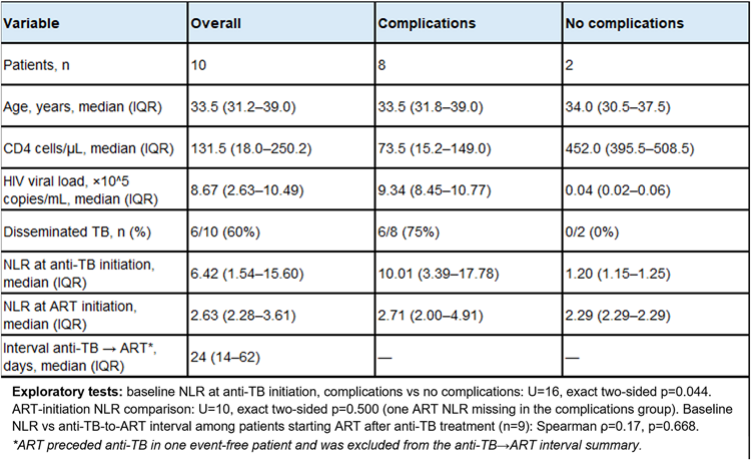
P501 | Patient demographics and statistical summary.

## People Living With HIV and Viral Hepatitis

### P502 Establishing International Expert Consensus on Hepatitis B Assessment, Monitoring and Management in the Context of HBV‐Inactive Two‐Drug HIV Therapy

Amy Paterson^1^, Kyle Ring^1^, Esteban Martínez^2,3^, Jürgen Rockstroh^4^, Alvaro López Iñiguez^5^, Chloe Orkin ^1^, on behalf of Hep B REACTIVATE 2DR Delphi Panel

^1^SHARE Collaborative, Centre for Immunobiology, Blizard Institute, Queen Mary University of London, London, UK; ^2^Hospital Clínic Barcelona‐Instituto de Investigaciones Biomédicas August Pi i Sunyer (IDIBAPS), Barcelona, Spain; ^3^University of Barcelona, Barcelona, Spain; ^4^Reial Acadèmia de Medicina de Catalunya (RAMC), Barcelona, Spain; ^5^Departamento de Infectología. Instituto Nacional de Ciencias Médicas y Nutrición, Mexico City, Mexico

**Background**: As the use of two‐drug and long‐acting HIV regimens expands, clinicians are increasingly starting and switching to regimens without hepatitis B virus (HBV) activity [1]. However, this raises practical questions that are not addressed in current guidance. This includes what screening and monitoring are required, when these should be performed, and how clinicians should respond if hepatitis B surface antigen or HBV DNA is detected [2, 3]. We sought international expert consensus on HBV screening, post‐switch monitoring and management for HBV‐inactive two‐drug antiretroviral therapy.

**Methods**: The Hep B REACTIVATE 2DR Study was an international mixed‐methods Delphi study conducted through iterative online questionnaires in February–March 2026. Delphi panellists were selected for clinical, research, guideline, advocacy or lived‐experience expertise in HIV and/or hepatitis B. Panellists rated statements on the topic using a scale of 1 (strongly disagree) to 9 (strongly agree). Open‐text comments informed statement revisions for the subsequent round. The stopping criteria for the Delphi were expert consensus (predefined as ≥75% experts scoring a statement 7–9) on all statements or a maximum of four rounds. Panellists were also asked to rank nine potential research priorities in order of importance.

**Results**: Panellists were drawn from 27 countries across Africa, the Americas, Asia, Europe and Oceania; 44/67 (65.6%) had more than 20 years’ experience, and 64/67 completed all study activities (95.5% retention). All screening, monitoring and management statements reached consensus in round 2. Final statements supported baseline hepatitis B serology and liver enzyme assessment close to switch; reserving HBV DNA testing, where available, to selective circumstances; risk‐stratified post‐switch monitoring; and prompt re‐establishment of HBV‐active therapy if confirmed reactivation (Figure 1). Isolated or very low‐level HBV DNA after switch remains an area where management is not fully settled. The highest‐ranked research priorities were determining the incidence and timing of HBV reactivation, identifying predictors for risk stratification and clarifying the clinical significance of low‐level HBV DNA viraemia.

**Conclusions**: These consensus statements offer clinicians, programmes and guideline developers a practical framework to standardize HBV risk assessment, monitor higher‐risk individuals and respond promptly to reactivation while implementing two‐drug antiretroviral therapy. The identified research priorities provide a guide for future work to support safe implementation of the HIV treatment pipeline.

**References**

1. Geretti AM, Boffito M, Bonora S, Braun P, Charpentier C, Garcia F, et al. Optimizing the use of cabotegravir plus rilpivirine long‐acting therapy in HIV care: Evidence, implementation, and unanswered questions. Open Forum Infect Dis. 2025;12(7):ofaf368. doi:10.1093/ofid/ofaf368.

2. Vasishta S, Dieterich DM, Mullen MM, Aberg JA. Brief report: Hepatitis B infection or reactivation after switch to 2‐drug antiretroviral therapy: A case series, literature review, and management discussion. J Acquir Immune Defic Syndr. 2023;94(2):160–164. doi:10.1097/QAI.0000000000003239.

3. Ring K, Orkin C, Parczewski M, Boffito M, Garcia F, Geretti AM. HBV reactivation post‐switch to HBV‐inactive ART: A scoping review. Conference on Retroviruses and Opportunistic Infections; 2025; San Francisco, CA. Abstract 771.

**FIGURE 1:**
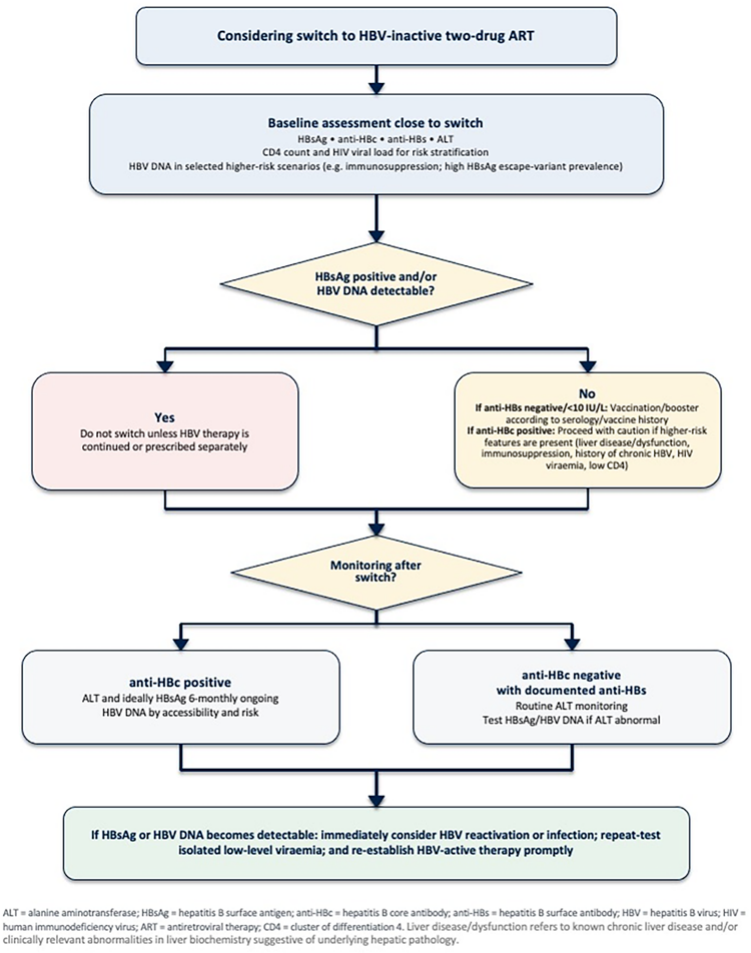
P502 | Decision tree: HBV assessment, monitoring and management.

### P503 International Expert Consensus on Hepatitis B Vaccination for People Switching to HBV‐Inactive Two‐Drug HIV Therapy

Paterson Amy^1^, Kyle Ring^1^, Esteban Martínez^2,3^, Jürgen Rockstroh^4^, Alvaro López Iñiguez^5^, Chloe Orkin ^1^, on behalf of Hep B REACTIVATE 2DR Delphi Panel

^1^SHARE Collaborative, Centre for Immunobiology, Blizard Institute, Queen Mary University of London, London, UK; ^2^2Hospital Clínic Barcelona‐Instituto de Investigaciones Biomédicas August Pi i Sunyer (IDIBAPS), Barcelona, Spain; ^3^University of Barcelona, Barcelona, Spain; ^4^Department of Medicine I, Bonn, Germany; ^5^Departamento de Infectología. Instituto Nacional de Ciencias Médicas y Nutrición, Salvador Zubirán, Mexico City, Mexico

**Background**: Antiretroviral therapy for HIV is increasingly moving towards drug‐sparing approaches using two‐drug regimens, none of which currently include tenofovir plus lamivudine or emtricitabine, the recommended backbone for HIV and hepatitis B coinfection. This means switching may expose people without documented immunity to hepatitis B virus (HBV) acquisition or reactivation. Guidance on vaccination before and after switching remains limited, particularly for people with isolated hepatitis B core antibody (anti‐HBc) or incomplete vaccine response. We aimed to establish international expert consensus on HBV vaccination and boosting in the context of HBV‐inactive two‐drug ART.

**Methods**: The Hep B REACTIVATE 2DR Study was an international mixed‐methods Delphi study conducted through online questionnaires in February–March 2026. Eligible panellists had relevant expertise in HIV and/or hepatitis B, including clinical, research, guideline development, advocacy or lived‐experience expertise. As part of the Delphi, panellists rated statements addressing HBV vaccination strategies before and after initiating HBV‐inactive ART using a 9‐point Likert scale. Expert agreement was calculated as the proportion of scores of 7–9, with unsure responses excluded from item‐specific denominators. Free‐text responses were reviewed inductively to inform revision of statements for the subsequent round. Consensus was predefined as at least 75% expert agreement. The predetermined stopping criteria for the Delphi were consensus on all statements or a maximum of four rounds.

**Results**: Sixty‐seven experts from 27 countries participated in the Delphi, with a retention rate of 95.5% across rounds. All eight final vaccination statements reached consensus in round 2, with expert agreement ranging from 55/63 (87.3%) to 62/64 (96.9%). The consensus statements detail vaccination or booster strategies across several serological scenarios, including triple‐negative serology, previous documented vaccine response with current loss of anti‐HBs, isolated anti‐HBc positivity, low or undetectable anti‐HBs and prior non‐response to a complete vaccine series (Figure 1).

**Conclusions**: The Delphi established consensus on vaccination and booster strategies for people being considered for, or already receiving, HBV‐inactive two‐drug ART. These strategies still need to be interpreted alongside HBV serology, individual risk and post‐switch monitoring where appropriate. Remaining evidence gaps include the utility and durability of vaccination responses in people with isolated anti‐HBc positivity, what anti‐HBs titre is sufficiently reassuring, and whether switching is appropriate in persistent vaccine non‐responders.

**FIGURE 1:**
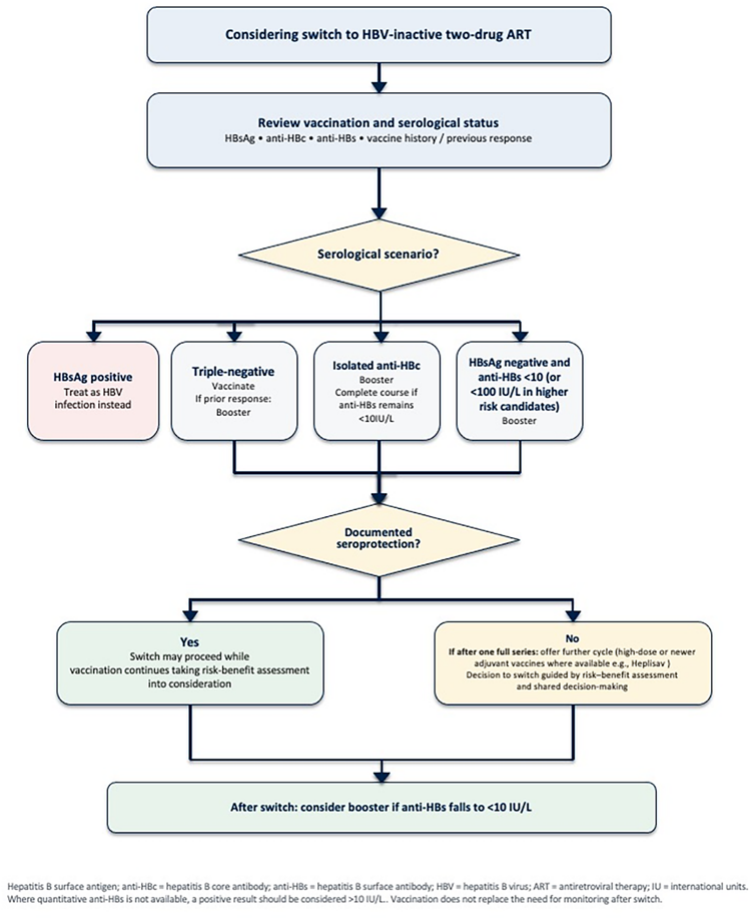
P503 | Decision tree: HBV vaccination and boosting.

### P504 Safety of Cabotegravir+Rilpivirine Long‐Acting Regimen Among People With HIV With Previous HBV Exposure: Last Update of the SCRIBE Study

Nicholas Brian Bana ^1^, Camilla Muccini^1^, Davide Paolo Bernasconi^2^, Andrea Carbone^3,4^, Simona Di Giambenedetto^3,4^, Carlo Torti^3,4^, Fiore Artelli^2,5^, Giuseppe Lapadula^2,5^, Paolo Bonfanti^2,5^, Paola Vitiello^6^, Clara Abeli^6^, Eugenia Quiros‐Roldan^7,8^, Giorgio Tiecco^7,8^, Laura Comi^9^, Diego Ripamonti^9^, Gaia Cologgi^10,11^, Antonio Di Biagio^10,11^, Massimo Puoti^1,2^, Roberto Rossotti^12^

^1^ASST Grande Ospedale Metropolitano Niguarda, Milan, Italy; ^2^School of Medicine and Surgery, University of Milano Bicocca, Monza, Italy; ^3^IRCCS Fondazione Policlinico Universitario A.Gemelli, Rome, Italy; ^4^Catholic University of the Sacred Heart, Rome, Italy; ^5^IRCCS Fondazione San Gerardo dei Tintori, Monza, Italy; ^6^ASST Valle Olona, Ospedale di Circolo, Busto Arsizio, Busto Arsizio (VA), Italy; ^7^ASST Spedali Civili di Brescia, Brescia, Italy; ^8^Department of Clinical and Experimental Sciences, Unit of Infectious and Tropical Diseases, University of Brescia, Brescia, Italy; ^9^ASST Papa Giovanni XXIII, Bergamo, Italy; ^10^IRCCS Azienda Ospedaliera Universitaria, Policlinico San Martino, Genoa, Italy; ^11^University of Genoa, Genoa, Italy; ^12^University of Verona, Verona, Italy

**Background**: Cabotegravir+rilpivirine long‐acting regimen (CARLA) does not contain anti‐HBV drugs and is not recommended among people with HIV (PWH) with active HBV coinfection. Guidelines allow its use for people with resolved HepB (HBcAb+, HBsAb+/−, HBsAg−) although caution is recommended since evidence on CARLA hepatic safety in this population remains limited.

**Methods**: Retrospective multicentric observational study including PWH who switched to CARLA. Primary aim was to assess liver safety among PWH with resolved HepB, analysing ALT and FIB4 trend over time. Furthermore, incidence rates for hepatitis flare (increase 3x of ALT normal values) and HBV reactivation (flare associated with detectable HBV DNA or HBsAg reappearance) were reported. FIB4 and ALT temporal trends were assessed with univariate and multivariate mixed linear model with a random intercept and slope adjusted, while factors associated with hepatitis flair were analysed with standard survival analyses.

**Results**: 721 PWH were included, with a median follow‐up time of 1.50 years. 185 (25.7%) had resolved HepB (Table 1). The multivariate mixed model did not find any significant impact of resolved HepB status on both ALT and FIB4 trend over time (Figure 1), in terms of changes per week (coef −0.01 IU/mL, 95% CI: −0.03 to 0.01, *p* = 0.393 and coef 0.0003, 95% CI: −0.0009 to 0.001, *p* = 0.674, respectively). Liver steatosis, previous HCV exposure and alcohol intake were not associated with either outcome. Hepatic flares incidence was higher among PWH with resolved HepB compared with HBV‐unexposed people: 22.0 (95% CI: 8.1–47.9) versus 9.1 (95% CI: 3.7–18.8) per 1000 person/years, but without reaching statistical significance (IRR 2.42, 95% CI: 0.72–8.07, *p* = 0.15). Only 2/42 (4.8%) flares happened in people with isolated HBcAb+. At a multivariate Cox's regression analysis, HBV exposure was slightly associated with higher risk of hepatic flares (aHR 3.45, 95% CI: 0.95–12.52, *p* = 0.06). No confirmed HBV reactivation was detected. One person with isolated HBcAb+ had a flare associated with HBsAb seroconversion and HBV DNA 17 IU/mL but no HBsAg reappearance, followed by spontaneous resolution.

**Conclusions**: CARLA showed a favourable hepatic safety profile in PWH with resolved HepB. Previous HBV exposure did not affect ALT or FIB‐4 trajectories, and no confirmed HBV reactivation was observed. Although hepatic flare incidence was higher among HBV‐exposed individuals, no significant association was demonstrated.

**FIGURE 1:**
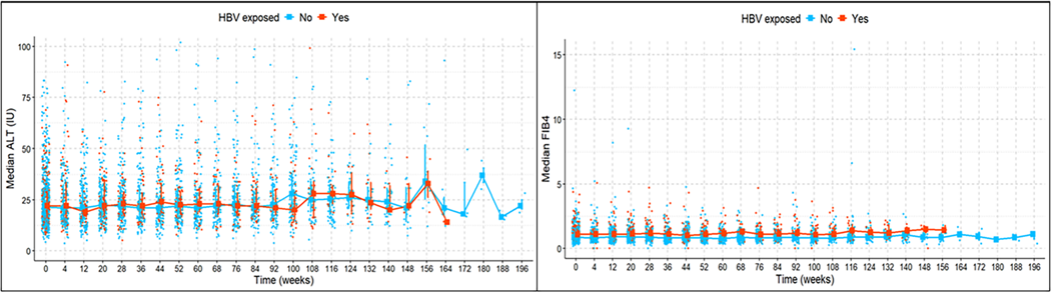
P504 | ALT and FIB4 trend over time among PWH with resolved HepB and HBV‐unexposed individuals.

**TABLE 1:**
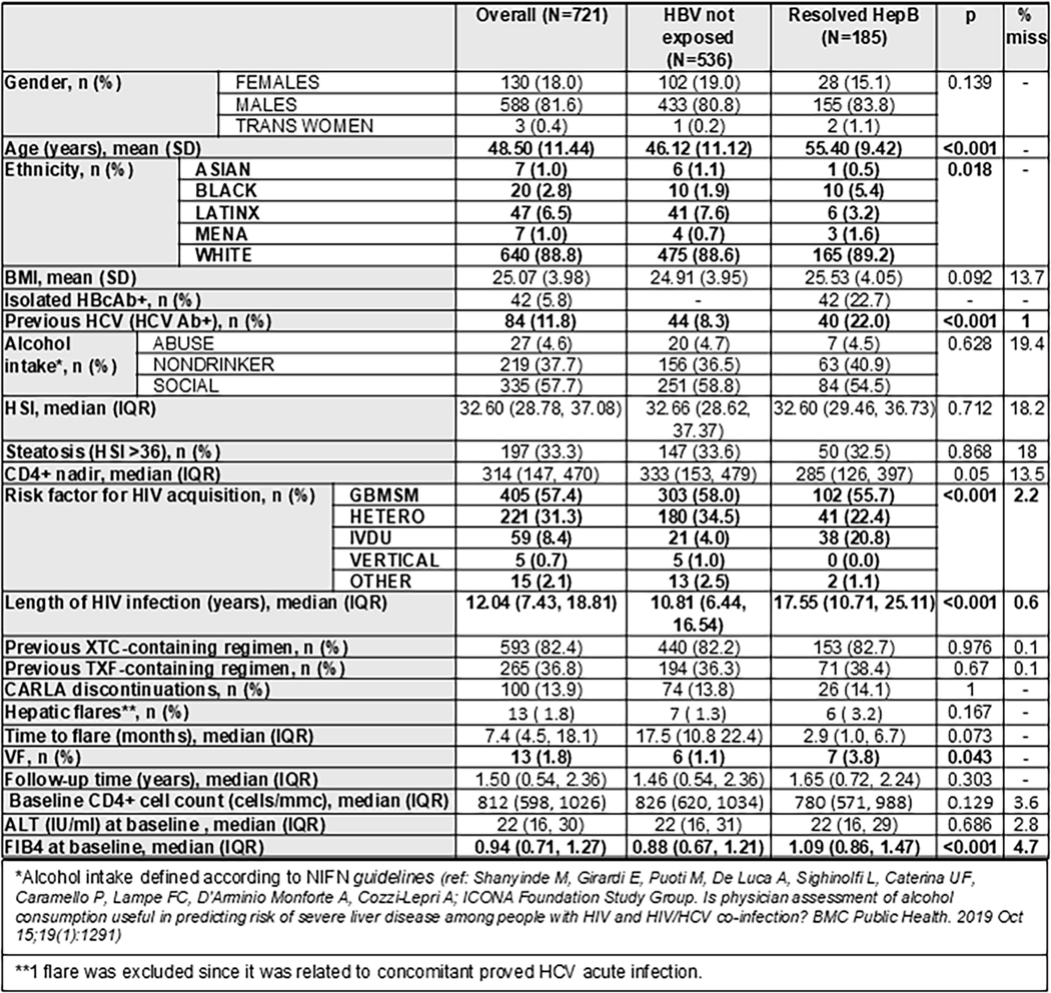
P504 | Mean features of study population.

### P505 Incomplete HBV Serological Documentation Could Mask Unrecognized Immunity Gaps in People Living With HIV Receiving Tenofovir‐Sparing Antiretroviral Therapy

Natalia Laufer ^1,2^, Yasmin El Kozah^3^, Jorge Gentile^4^, Emilia Cohen^5^, Marcelo Gañete^6^, Gabriela Poblete^3^, Graciela Guaragna^7,8^, Florencia Ferrero^3,9^, Raquel Zhumi^10^, Fabricio Morales^3,11^, Marina Grand^12^

^1^División Infectología, Hospital Juan A. Fernández, Buenos Aires, Argentina; ^2^CONICET‐Universidad de Buenos Aires, Instituto de Investigaciones Biomédicas en Retrovirus y SIDA (INBIRS), Buenos Aires, Argentina; ^3^Centro Médico Accord (CEMAC), Buenos Aires, Argentina; ^4^Hospital Municipal “Ramón Santamarina”, Tandil, Argentina; ^5^Hospital Interzonal General de Agudos “Eva Perón” (ex Castex), San Martín, Argentina; ^6^Hospital Higa Evita Lanús, Lanus, Argentina; ^7^Unidad de Infectología Hospital Teodoro Alvarez, Buenos Aires, Argentina; ^8^Sanatorio Nuestra Señora del Pilar, Ciudadela, Argentina; ^9^Sanatorio Trinidad Mitre, Buenos Aires, Argentina; ^10^Sanatorio San Justo, San Justo, Argentina; ^11^Servicio de Infectología Sanatorio Anchorena, San Martín, Argentina; ^12^Hospital Municipal “Dr. Héctor M. Cura”, Olavarria, Argentina

**Background**: In people living with HIV (PLWH), routine HBV serology extends beyond HBsAg: characterizing prior exposure patterns and anti‐HBs protective status is essential to guide vaccination and monitor reactivation risk in tenofovir‐sparing regimens whose use has increased [1]. We aimed to describe HBV serological patterns, quantify documentation gaps and identify patients lacking protective immunity on TXF‐sparing ART.

**Methods**: Cross‐sectional multicentre study of 679 PLWH on ART attending scheduled visits at 9 HIV centres in Buenos Aires, Argentina, during 2025. ART regimens were categorized as tenofovir alafenamide or disoproxil (TXF)‐containing or TXF‐sparing. HBV serological patterns were classified in five categories: chronic infection (HBsAg‐positive), resolved infection (anti‐HBc‐positive/anti‐HBs > 10 IU/L), isolated anti‐HBc, vaccine‐induced immunity (anti‐HBc‐negative/anti‐HBs > 10 IU/L) and no exposure/immunity (anti‐HBc‐negative/anti‐HBs ≤10 IU/L). Associations with CD4 count and CD4/CD8 ratio were assessed using chi‐square/Fisher's exact and Mann–Whitney U tests.

**Results**: Among 679 participants (64.7% cisgender men; median age 48 years), 396 (58.3%) received TXF‐containing and 281 (41.4%) TXF‐sparing regimens, 89.7% of the latter with 3TC. HBsAg was positive in 22(3.2%), all on TXF‐based ART. Complete HBV serological data allowed pattern classification in only 383/679 (56.4%), reflecting systematic gaps in HBV documentation. Among classifiable patients, the distribution was: 5.7% chronic infection, 13.6% resolved infection, 2.1% isolated anti‐HBc, 50.1% vaccine‐induced immunity and 28.5% no exposure/immunity. Overall, 117/383 (30.5%) lacked protective HBV immunity, of whom 46 (39.7%) were on TXF‐sparing regimens. Among anti‐HBc‐negative patients with anti‐HBs recorded, non‐protective anti‐HBs (≤10 IU/L) was associated with a higher proportion of CD4 < 200 cells/µL (10.5% vs. 1.1%, *p* < 0.001), lower CD4/CD8 ratio (median 0.64 vs. 0.90, *p* = 0.008; CD4/CD8 < 0.4 in 24.2% vs. 4.0%, *p* = 0.012) and lower CD4 count (median 611 vs. 690 cells/µL, *p* = 0.025) compared to vaccine‐induced immunity (Table 1).

**Conclusions**: Nearly one‐third of PLWH with classifiable HBV serology lacked protective immunity, almost 40% of whom were receiving TXF‐sparing regimens. The high proportion of unclassifiable patients (43.6%) underscores critical gaps in systematic HBV serological documentation in clinical practice. Strengthening routine HBV screening, ensuring anti‐HBs titres are recorded in all anti‐HBc‐negative patients, and prioritizing vaccination, particularly in those with TXF‐sparing regimens (including vaccination of individuals with isolated core for anamnestic response) are essential steps to prevent HBV acquisition and potential reactivation.

**Reference**

1. Denyer RV, Tate JP, Benator DA, Lim JK, Weintrob A. Hepatitis B reactivation in a US cohort of people with HIV and hepatitis B core antibody after switch to antiretroviral therapy without hepatitis B activity. Clin Infect Dis. 2026 Apr 30;82(4):e735–e742. doi: 10.1093/cid/ciag001. PMID: 41848012; PMCID: PMC13131950.

**TABLE 1:**
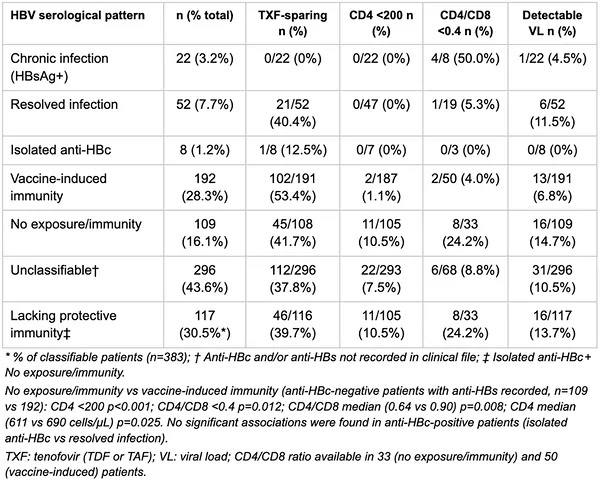
P505 | HBV serological patterns, ART regimen and immune status among 679 PLWH.

### P506 Cascade of Care for Chronic Hepatitis B Virus in People With HIV in Brazil and Mexico

Alvaro López Iñiguez ^1^, Yanink Caro Vega^1^, Juan G Sierra Madero^1^, Jessica Castilho^2^, Fernanda Maruri^2^, Sandra Wagner Cardoso^3^, Hugo Perazzo^3^, Esteväo Portela Nunes^3^, Valdilea Gonçalves Veloso^3^, Brenda Crabtree Ramirez^1^

^1^Infectious Diseases Department, Instituto Nacional de Ciencias Médicas y Nutrición Salvador Zubirán, Mexico City, Mexico; ^2^Vanderbilt University Medical Center, Division of Infectious Diseases, Nashville, TN, USA; ^3^Fundacäo Oswaldo Cruz, Instituto Nacional de Infectologia Evandro Chagas, Río de Janeiro, Brazil

**Background**: Hepatitis B virus infection (HBV) among people with HIV (PWH) is a major global issue. By 2025, 40 million individuals were projected to have HIV, while over 250 million have chronic HBV. The WHO aims to eradicate HBV by 2030, targeting 90% status awareness, 80% treatment coverage and 60% viral suppression. There is limited information on the HBV care cascade (CC) among PWH in Latin America. This study examined the CC from tertiary care centres in Brazil and Mexico, in CCASAnet.

**Methods**: We conducted a cross‐sectional analysis of HBV data available from the CCASAnet clinical sites in Brazil (January 2000 to November 2023) and Mexico (January 2000 to October 2024). We included people in care within the past year, and with HBV laboratory data available. We analysed these components of the CC: (i) total and percentage of PWH screened for HBV (surface antigen [HBsAg] and/or anti‐core antibodies [HBcAb] at any time point); (ii) number and percentage of individuals with chronic HBV (HBsAg+) or prior HBV exposure (HBcAb+) among those screened; (iii) number and percentage of individuals receiving HBV treatment; (iv) data on treatment response. We reported HBV‐immunity among those screened and from those with chronic HBV/prior exposure, number and percentage with reactive antigen E (HBeAg+).

**Results**: 12,776 PWH were included during the study period, but only 6531 (51%) had documented HBV information. Among those 6531, the majority were male (5051; 77%), with a median age of 34.4 years (IQR 21.1–35.9) and a median CD4 of 357 (IQR 154–580) at HBV screening. Of these 5896 (90%) had documented HBV screening, and among those 1546 (26%) had at least one reactive marker: 8% (125/1546) with HBsAg and 92% (1421/1546) with HBcAb. Among those 1546 PWH with chronic HBV/prior HBV exposure, 1424 (92%) received tenofovir‐containing ART. There was no information available about HBV viral load. Immunity information was available for 4985, of whom 2675 (53%) were immune (Figure 1). HBeAg testing was performed in 706 with 15% (105/706) reactive.

**Conclusions**: The cascade of care for HBV in this cohort's PWH identified areas for improvement, particularly in treatment response and immunity. The percentage of reactive HBsAg and HBcAb was higher than reported previously.

**FIGURE 1:**
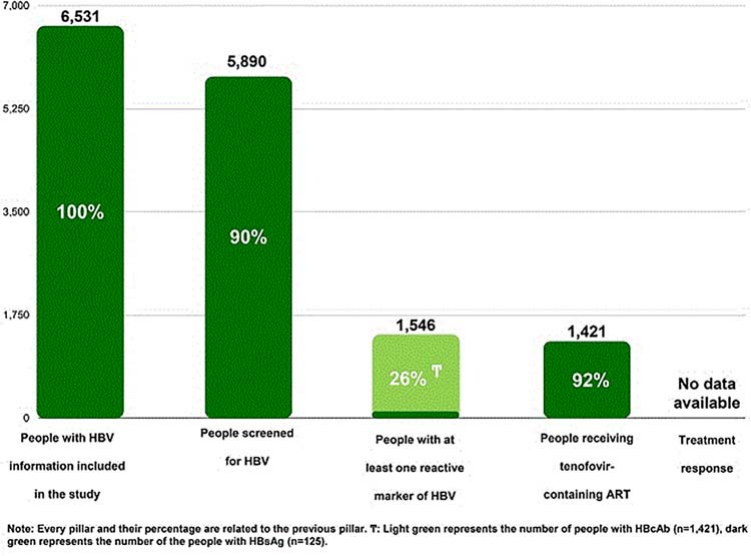
P506 | Cascade of care for chronic HBV in PWH in Brazil and Mexico.

### P507 Persistent HBV Viraemia Despite ART: The Critical Role of HIV Suppression in HIV/HBV Co‐Infected Individuals

Sara Nsibirwa ^1^, Katuku Aizire^2^, Ponsiano Ocama^3^, Gregory Kirk^2^, on behalf of H2U Consortium

^1^Infectious Diseases Institute, Makerere University College of Health Sciences, Kampala, Uganda; ^2^Department of Epidemiology, Johns Hopkins Bloomberg School of Public Health, Baltimore, MD, USA; ^3^Department of Internal Medicine, Makerere University College of Health Sciences, Kampala, Uganda

**Background**: Combined antiretroviral therapy (ART) effectively controls both HIV and HBV replication but the extent and predictors of dual viral control in co‐infected individuals in Africa remain unclear. We evaluated the relationship between ART duration and HIV/HBV viral suppression among co‐infected individuals, receiving HBV‐active ART in Uganda.

**Methods**: We analysed ART‐experienced individuals attending an urban HIV clinic. ART duration was categorized as short‐term (<2 years), medium‐term (2–6 years) or long‐term (≥6 years). HIV and HBV viral loads were classified as undetectable (<20 copies/mL), low‐level viraemia (20–999 copies/mL) or unsuppressed (≥1000 copies/mL). Multinomial logistic regression assessed the effect of ART duration on viral suppression while logistic regression identified predictors of persistent HBV viraemia (≥1000 copies/mL). Marginal analyses estimated adjusted probabilities of HBV persistence across ART duration and HIV suppression strata.

**Results**: Of 278 individuals, 53.2 % were male, median age was 45 (37–52) years and median ART duration was 5.9 years (IQR 2.8–10.5). Over 50% had a nadir CD4 count < 500 cells/µL. HIV and HBV viral suppression were achieved in 95.7% and 84.2%, respectively. Among long‐term ART users (≥6 years), 98.5% had undetectable HIV viral loads compared to 85% of short‐term ART users (<2 years). Similarly, 88.4% of long‐term ART users had undetectable HBV DNA, compared to 67% of short‐term ART users. Longer ART duration significantly reduced the odds of unsuppressed HIV (*p* = 0.002) and HBV (*p* = 0.002), but no association seen for low‐level viraemia. Unsuppressed HIV was the strongest predictor of persistent HBV viraemia (aOR 4.3, 95% CI: 1.1–16.3, *p* = 0.035), while long‐term ART showed a protective trend (aOR 0.48, 95% CI: 0.21–1.07, *p* = 0.073). Adjusted probability analysis showed that with HIV suppression, the likelihood of persistent HBV viraemia declined from 19.0% (95% CI: 11.6–26.4) among short‐term ART users to 10.2% (95% CI: 5.0–15.3) among long‐term users (Table 1).

**Conclusions**: ART duration is significantly associated with HIV and HBV viral suppression in co‐infected individuals. Delayed HBV suppression was evident in the early ART phase and persistent HBV viraemia observed in some long‐term ART users. Unsuppressed HIV strongly predicted ongoing HBV replication. This emphasizes the significance of adherence support, routine HBV monitoring and integrated HIV/HBV care to optimize outcomes.

**TABLE 1:**
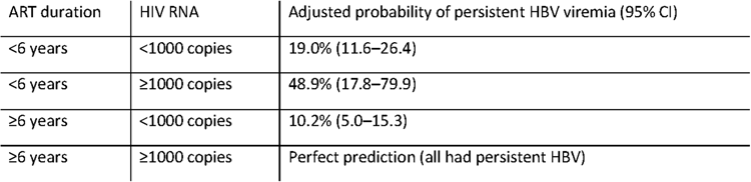
P507 | Probability of persistent HBV viraemia against HIV viral load suppression.

### P508 Hepatitis C Virus Coinfection and Reinfection After Achieving Sustained Virological Response Among People Living With HIV in Northern Vietnam: A Prospective Multicentre Cohort Study

Junko Tanuma ^1^, Giang Van Tran^2^, Truong Manh Nguyen^2^, Thach Ngoc Pham^3^, Masaya Sugiyama^4^, on behalf of SATREPS HIV Cohort

^1^Department of Infectious Diseases, School of Medicine, International University of Health and Welfare, Chiba, Japan; ^2^Department of Infectious Diseases, Hanoi Medical University, Hanoi, Vietnam; ^3^National Hospital of Tropical Diseases, Hanoi, Vietnam; ^4^Department of Viral Pathogenesis and Controls, National Institute of Global Health and Medicine, Japan Institute for Health Security, Ichikawa, Japan

**Background**: Viral hepatitis and HIV share transmission routes and integrated care is recommended. Vietnam carries a high hepatitis C virus (HCV) burden, yet data on HCV‐RNA positivity and reinfection after achieving sustained virological response (SVR) among people living with HIV (PLWH) are limited. We assessed HCV coinfection and reinfection rate after achieving SVR with direct‐acting antivirals (DAAs) in a prospective cohort in northern Vietnam.

**Methods**: Within the SATREPS HIV cohort, established in 2019 across 11 hospitals in northern Vietnam, PLWH receiving regular outpatient care were prospectively studied. At baseline, plasma collected between April and September 2023 underwent anti‐HCV antibody testing, and HCV‐RNA was measured in anti‐HCV‐positive individuals to identify active infections. Factors associated with anti‐HCV positivity were identified by multivariable analysis. At the next routine cohort visit, follow‐up samples were obtained from antibody‐positive and HCV‐RNA‐negative participants previously treated with DAAs or peginterferon plus ribavirin, to evaluate the rate of reinfection, defined as reappearance of HCV‐RNA.

**Results**: Among 2050 PLWH who provided consent, the median age was 44 years and 59% were men. At baseline, 764 participants (37.3%) were anti‐HCV antibody‐positive, including 22 with previously documented negative results, suggesting incident HCV infection. Anti‐HCV antibody positivity was highest among people who inject drugs (91%), and injection drug use was independently associated with HCV coinfection. Among the 764 anti‐HCV antibody‐positive participants, 211 (27.6%) had detectable HCV‐RNA, indicating active HCV infection; 25 of these had previously been treated for HCV. Among the 533 HCV‐RNA‐negative participants, 175 had previously received DAA and 46 had received peginterferon plus ribavirin. Follow‐up samples, collected a median of 19.7 months after baseline, were available for 158 participants, including 130 treated with DAAs and 28 treated with peginterferon plus ribavirin. Reinfection occurred in one participant previously treated with sofosbuvir plus daclatasvir, corresponding to a reinfection rate of 0.39 per 100 person‐years. (95% CI: 0.01–2.19).

**Conclusions**: HCV coinfection remains highly prevalent among PLWH in northern Vietnam, particularly among people who inject drugs, but is not limited to this group. Universal HCV screening should, therefore, be offered to all PLWH, rather than only to those reporting injecting drug use. Furthermore, although reinfection after SVR was uncommon, it can occur; therefore, periodic HCV‐RNA monitoring may be considered for high‐risk populations.

### P509 Abstract Withdrawn

### P510 Glecaprevir/Pibrentasvir Versus Sofosbuvir/Velpatasvir for Chronic Hepatitis C in Latin America, Including People Living With HIV: The Randomized ESGRIMA Trial

Said López García^1^, Ana Luz Cano Díaz ^1^, José Antonio Mata Marín^1^, Emily Itzel Pecero García^1^, Gloria Elizabeth Salinas Velázquez^2^, Alberto Chaparro Sánchez^1^, Ericka Nelly Pompa Mera^1^, Jesús Enrique Gaytán Martínez^1^

^1^Infectious Diseases Department, CMN La Raza, Mexico City, Mexico City, Mexico; ^2^Department of Radiology, CMN La Raza, Mexico City, Mexico City, Mexico

**Background**: Head‐to‐head data comparing pangenotypic hepatitis C virus (HCV) regimens in Latin America are limited, particularly among people living with HIV. We compared the efficacy and safety of glecaprevir/pibrentasvir (GLE/PIB) versus sofosbuvir/velpatasvir (SOF/VEL).

**Methods**: ESGRIMA was an open‐label, single‐centre, randomized 2:1 (2 GLE/PIB; 1 SOF/VEL) clinical trial conducted in Mexico City. Adults with chronic HCV infection, without decompensated cirrhosis or prior exposure to direct‐acting antivirals, were randomized to receive either GLE/PIB for 8 weeks or SOF/VEL for 12 weeks. People living with HIV were eligible if their HIV‐1 RNA was < 50 copies/mL. The primary endpoint was sustained virological response 12 weeks after treatment completion (SVR12). The modified intention‐to‐treat (mITT) analysis included participants with available SVR12 results, whereas the intention‐to‐treat (ITT) analysis classified missing results as non‐response. Secondary endpoints included end‐of‐treatment (EOT) response, self‐reported adherence, liver biochemistry, clinical events, and changes in HADS‐A, HADS‐D, PHQ‐9, and ISI scores.

**Results**: Forty‐six participants were randomized: 31 (67.4%) to GLE/PIB and 15 (32.6%) to SOF/VEL. The mean age was 44.6 ± 15.6 years; 39 (84.8%) were male, 20 (43.5%) were people living with HIV, and the median FIB‐4 score was 1.04 (IQR, 0.74–1.70). SVR12 data were unavailable for 6/46 (13.0%) participants: 3/31 (9.7%) in the GLE/PIB group and 3/15 (20.0%) in the SOF/VEL group. In the mITT analysis, SVR12 was 100.0% (40/40; 95% confidence interval [CI], 91.2–100.0%): 28/28 (100.0%) with GLE/PIB versus 12/12 (100.0%) with SOF/VEL. In the ITT analysis, SVR12 was 87.0% (40/46; 95% CI, 73.7–95.1%): 28/31 (90.3%) GLE/PIB versus 12/15 (80.0%; *p* = 0.375) in SOF/VEL. All 17/17 (100.0%) evaluated people living with HIV achieved SVR12. EOT HCV RNA was available for 43/46 (93.5%) participants: 40/43 (93.0%) were undetectable and 3/43 (7.0%) had low‐level viraemia; all three subsequently achieved SVR12. Median ALT decreased from 117.5 to 21.0 IU/L (*p* < 0.001). No grade 3–4 adverse events, treatment discontinuations, or deaths occurred.

**Conclusions**: Both regimens achieved complete observed SVR12, high adherence, significant improvements in liver biochemistry, and a favourable clinical and neuropsychiatric safety profile, including among PLH. Neither regimen demonstrated a significant efficacy or safety advantage.[Fig jia270211-fig-0581]

**TABLE 1 jia270211-fig-0581:**
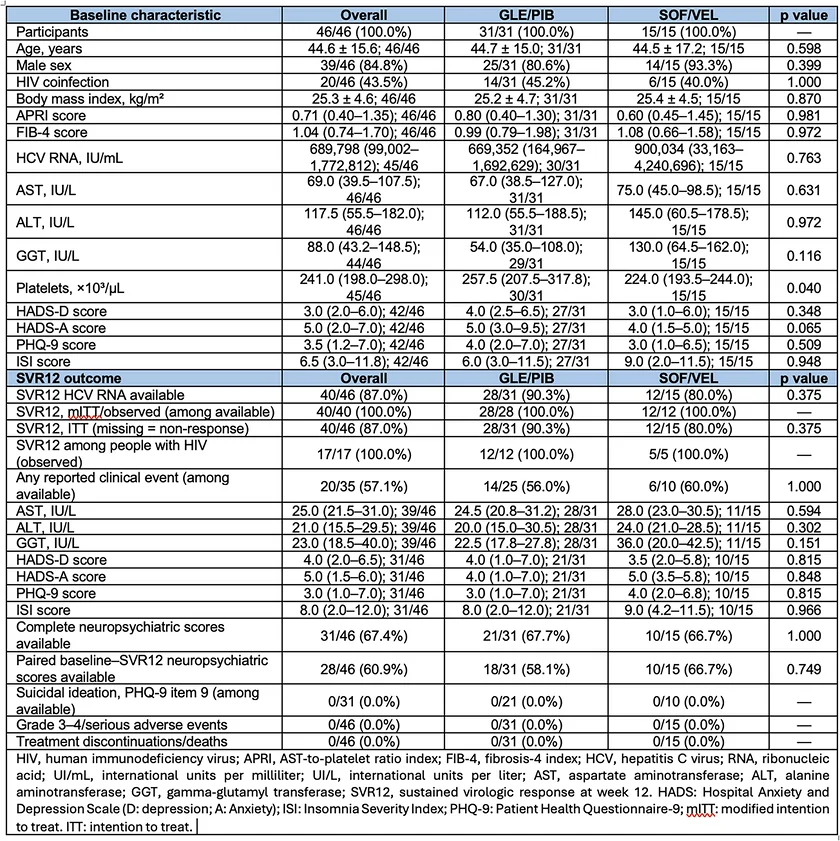
P510 | Baseline characteristics before randomization and at sustained viral response at 12 weeks of GLE/PIB versus SOF/VEL.

### P511 Liver Fibrosis and Steatosis Burden in Ageing People Living With HIV: A Real‐World FibroScan‐Based Assessment

Micaela Cueto‐Marquez, Inês Cachapa‐Vieira, Julia Lleixa‐Martin, Marc PedrosaAragon, Sonia Calzado Isbert, Marta Navarro óVilasar, Maria del Carmen Navarro Sáez

Infectious Diseases Department, Parc Taulí Hospital Universitari, Institut d'Investigació i Innovació Parc Taulí (I3PT‐CERCA), Sabadell, Spain

**Background**: Liver disease remains a major comorbidity in people living with HIV (PLWH), driven by hepatitis C virus (HCV) coinfection and metabolic dysfunction. Transient elastography (FibroScan) enables simultaneous non‐invasive assessment of hepatic fibrosis and steatosis, yet data from contemporary real‐world HIV outpatient cohorts in the era of universal HCV treatment remain scarce.

**Methods**: Cross‐sectional observational study at the HIV outpatient clinic of Hospital Universitari Parc Taulí (Sabadell, Spain), June 2025–January 2026. Consecutive PLWH aged ≥60 years attending routine visits were enrolled. Hepatic assessment included history of HCV and hepatitis B virus (HBV) infection, anticore antibody status and FibroScan where clinically indicated (liver stiffness in kPa; controlled attenuation parameter [CAP] in dB/m). Fibrosis was staged as F0/F1 (<7.1 kPa), F2 (7.1–9.5 kPa), F3 (9.6–12.5 kPa) or F4/cirrhosis (> 12.5 kPa). Steatosis was graded S0/S1 (<260 dB/m), S2 (260–290 dB/m) or S3 (> 290 dB/m). Descriptive statistics were performed. The study was conducted in accordance with local ethical standards.

**Results**: Fifty‐eight PLWH were included (median age 69 years). HCV coinfection history was present in 38%, chronic HBV in 7% and anticore positivity in 44% (25/57). FibroScan was performed in 34 participants (59%); liver stiffness was measurable in 27, with a median of 6.8 kPa (mean 9.1, SD 7.6). Fibrosis staging (*n* = 34) revealed F0/F1 in 47%, F2 in 18%, F3 in 18% and F4/cirrhosis in 18% (Table 1). Among participants undergoing FibroScan, advanced fibrosis (F3–F4) was present in 35% of cases. This rate rose to 45% in those with prior HCV exposure and reached 50% (8/16) within the anti‐HBc‐positive subgroup. CAP was available in 33 participants (mean 264.8 dB/m, SD 49.7): S0/S1 45%, S2 27%, S3 27%; significant steatosis (S2–S3) was present in 55%.

**Conclusions**: In this real‐world cohort of ageing PLWH, liver disease burden remained high despite effective HCV treatment: 35% of those assessed showed advanced fibrosis, rising to 45% among those with prior HCV exposure, and over half had significant steatosis. These findings support consideration of systematic hepatic assessment, including FibroScan where appropriate, in ageing PLWH, particularly those with prior HCV exposure.

**TABLE 1:**
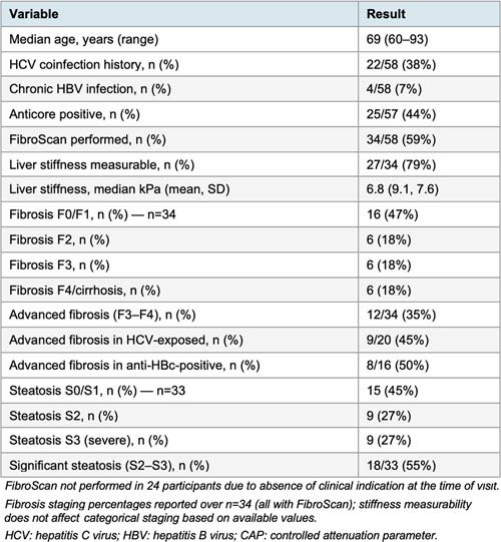
P511 | Hepatic profile of the cohort (*n* = 58).

### P512 Linkage to HCV and HIV Care Among People With Opioid Use Disorder in Methadone and Buprenorphine/Naloxone Clinics in Taiwan

Chien‐Yu Cheng ^1^, Hui‐Ting Hsieh^2^, Sheng‐Chang Wang^3^, Shu‐Hsing Cheng^1^

^1^Department of Infectious Disease, Taoyuan General Hospital, Ministry of Health and Welfare, Taoyuan City, Taiwan; ^2^Department of AIDS Care Center, Taoyuan General Hospital, Ministry of Health and Welfare, Taoyuan City, Taiwan; ^3^Center for Neuropsychiatric Research, National Health Research Institutes, Miaoli County, Taiwan

**Background**: Linkage to HIV and HCV care among people who use drugs remains a critical public health challenge, as stigma, legal risk and systemic barriers cause this marginalized population to fall through the cracks of traditional healthcare [1]. A community‐based outreach screening‐and‐referral programme for people with opioid use disorder (OUD) has been implemented across methadone and BN clinics in Taoyuan City since 2024.

**Methods**: This study enrolled 153 patients with opioid and/or methamphetamine dependence from methadone or buprenorphine/naloxone clinics. Rapid anti‐HIV and anti‐HCV testing was performed for those with unknown status, and those who tested positive were referred for diagnostic confirmation. For participants with established HIV infection, current treatment status was evaluated; individuals identified as antiretroviral therapy (ART) dropouts were subsequently recommended for care referral. Among participants with a known history of HCV infection, individuals without prior interferon or direct‐acting antiviral treatment were likewise recommended for referral. Demographics, drug use history, BN treatment status and spatial accessibility to referral hospitals were collected to assess factors associated with willingness to accept referral [2, 3].

**Results**: Among 140 participants with unknown HIV status, rapid testing identified no new HIV infections. All 13 participants with established HIV infection (100%) had HCV co‐infection. Of 7 patients on active ART, 3 were concurrently receiving HCV direct‐acting antiviral therapy, 3 accepted referral and 1 declined. Of 6 ART dropouts, 4 accepted linkage to HIV/HCV care and 2 declined ART resumption (Figure 1).

Within the BN‐treatment subgroup (*n* = 90), 46 patients were eligible for HCV care linkage (12 newly diagnosed, 34 with known untreated HCV); 27 (58.7%) accepted referral. Male sex (*p* = 0.016), buprenorphine dose < 4mg/day (*p* = 0.004) and geographic distance to referral hospital > 10 km (*p* = 0.015) were significantly associated with referral refusal (Table 1).

**Conclusions**: Methadone and BN clinics are critical settings for integrated HIV/HCV care. Strengthening addiction treatment quality, HCV‐related health education, and on‐site diagnostic and therapeutic pathways could reduce barriers to care linkage in this population.

**References**

1. Chung YE, Tseng CI, Wang SC, et al. Opioid agonist treatment programs and hepatitis C virus elimination in Taiwan: Ways of expanding the care cascade in screening and treatment. Int J Drug Policy. 2024;134:104658.

2. Ramers CB, Vawter N, Northrup A, et al. Simplified and integrated hepatitis C virus testing and treatment algorithm for unhoused people who inject drugs. Open Forum Infect Dis. 2025;12(6):ofaf302.

3. Ku YC, Chung HP, Hsu CY, et al. Recidivism of individuals who completed schedule I drugs deferred prosecution treatment: A population‐based follow‐up study from 2008 to 2020 in Taiwan. Int J Ment Health Addiction 2024;22, 3040–3059.

**FIGURE 1:**
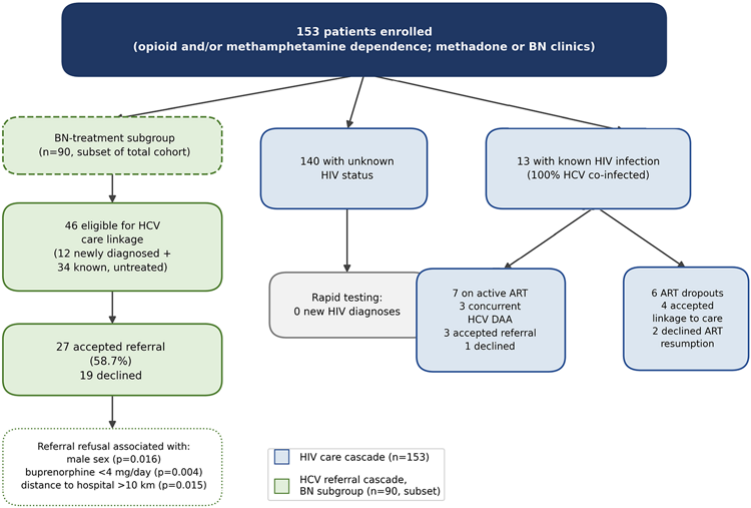
P512 | Patient flow through HIV and HCV testing, treatment and care linkage.

**TABLE 1:**
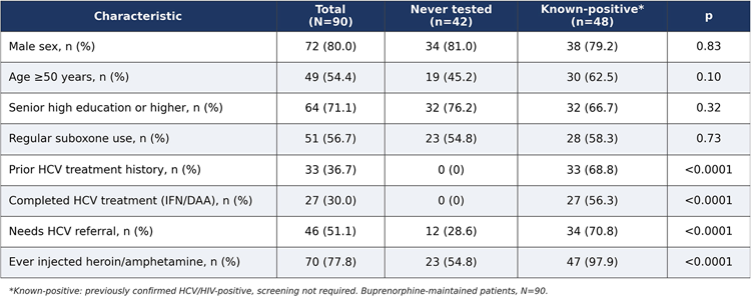
P512 | Baseline characteristics by prior HCV/HIV screening status.

## People Living With HIV and Other Infections

### P513 Increased Risk of Laboratory‐Confirmed and Severe Influenza in a Contemporary Cohort of Well‐Treated Persons With HIV Compared With Matched Population Controls

Josefine Amalie Loft^1^, Magnus Glindvad Ahlström^2^, Andreas Dehlbæk Knudsen^1^, Dina Leth Møller^1,2^, Andreas Ronit^1,3^, Thomas L. Benfield^3^, Børge Nordestgaard^4,5^, Shoaib Afzal^4,5^, Susanne Dam Nielsen ^1^

^1^Department of Infectious Diseases, Copenhagen University Hospital ‐ Rigshospitalet, Copenhagen, Denmark; ^2^Department of Clinical Microbiology, Copenhagen University Hospital ‐ Rigshospitalet, Copenhagen, Denmark; ^3^Department of Infectious Diseases, Copenhagen University Hospital ‐ Amager and Hvidovre, Hvidovre, Denmark; ^4^The Copenhagen General Population Study, Copenhagen University Hospital ‐ Herlev and Gentofte, Herlev, Denmark; ^5^Department of Clinical Biochemistry, Copenhagen University Hospital ‐ Herlev and Gentofte, Herlev, Denmark

**Background**: Influenza virus infection is a major cause of global morbidity and mortality. While influenza burden is increased among untreated persons with HIV (PWH) compared with the general population, it is unclear if this also applies to well‐treated PWH. We investigated the incidence of laboratory‐confirmed influenza and severe influenza, that is influenza‐associated hospitalization and death, in a contemporary cohort of virologically suppressed PWH and matched controls from the Danish general population and examined whether HIV status was associated with the risk of these outcomes.

**Methods**: We included 1005 virologically suppressed PWH from the Copenhagen Comorbidity in HIV Infection Study and 10,050 matched controls from the Copenhagen General Population Study. Data on influenza, hospitalizations death and vital status were obtained from national registries. Incidence rates (IRs) per 1000 person‐years (PY) and incidence rate ratios (IRRs) were calculated, and cumulative incidences were estimated accounting for competing risks. Associations between HIV status and outcomes were explored using Fine–Gray sub‐distribution hazards models.

**Results**: Among PWH, 99.3% were receiving antiretroviral therapy at inclusion and the mean CD4+ T cell count was 721.0 cells/µL. Follow‐up time was 6286 PY in PWH and 60,596 PY in matched controls for laboratory‐confirmed influenza, and 5890 PY and 56,015 PY, respectively, for severe influenza. The IRs per 1000 PY of laboratory‐confirmed influenza were 4.1 (95% CI: 2.7–6.1) in PWH and 1.8 (95% CI: 1.5–2.1) in controls (IRR 2.3, *p* < 0.001). For severe influenza, IRs were 1.9 (95% CI: 0.9–3.3) and 0.5 (95% CI: 0.4–0.7), respectively (IRR 3.6, *p* < 0.001). Cumulative incidences of both outcomes were higher in PWH (*p* < 0.001) (Figure 1) and HIV status was associated with increased risk of laboratory‐confirmed influenza (sub‐distribution hazard rate [sHR] 2.5, 95% CI: 1.6–3.8, *p* < 0.001) and severe influenza (sHR 3.9, 95% CI: 2.0–7.8, *p* < 0.001).

**Conclusions**: Even well‐treated PWH have higher risk of laboratory‐confirmed influenza and severe influenza compared with the general population, highlighting the importance of preventive measures, including optimal treatment of comorbidities and continued promotion of influenza vaccination.

**FIGURE 1:**
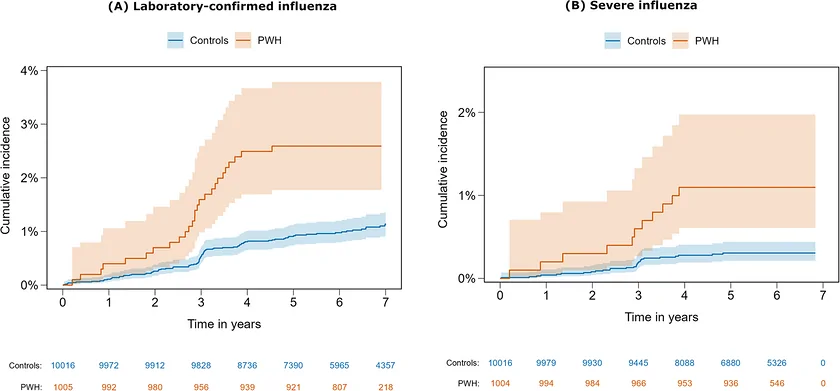
P513 | Cumulative incidence of laboratory‐confirmed influenza (A) and severe influenza (B) in PWH and matched controls.

### P514 Prevalence of Measles Immunity and Implementation of Vaccination in London Clinics

Suki Leung, Ruth Byrne, Nicolo Girometti, Lauren Bull, Michael Rayment

Chelsea and Westminster NHS Foundation Trust, London, UK

**Background**: People living with HIV, especially those with low CD4 counts, may be more likely to acquire and suffer complications from measles infection. Around 10% of adults with HIV lack measles immunity [1]. With the surge in transmissions in the UK recently due to suboptimal vaccine coverage and in light of the British HIV Association (BHIVA) statement [2], our Trust has adopted the recommendations of screening for measles IgG in people lacking documented seropositivity, regardless of prior vaccination or disease history and offering MMR vaccination to seronegative individuals.

**Methods**: Retrospective review of electronic patient records and biochemistry results for people with HIV with measles IgG results seen in HIV clinics at Chelsea and Westminster Hospital between August 2024 and April 2026.

**Results**: Of 6908 screened for measles IgG, 5917 (86%) were seropositive and 991 (14%) were seronegative. The majority screened were UK‐born White men, with mean age of 48 (IQR 39–56) and mean CD4 count of 601 (IQR 370–853) cells/mm^3^. HIV virological suppression remained high across all screened individuals, with an average years of diagnosis of 15 (IQR 8–20). The seronegative group was younger than the seropositive group (see Table 1). The proportions of country‐of‐birth were largely similar in both groups, with the largest percentage in the UK, followed by Europe and America in the seropositive cohort and vice versa in the seronegative cohort. Of 73 who have been vaccinated (7%), although there were 21 (29%) individuals in each UK‐, Europe‐ or America‐born groups, the uptake was highest in Asia‐born group (10%). Majority were male (70, 96%). Two‐thirds (67%) had two doses and a quarter (26%) had immunity after 1 dose. The mean interval from screening to receipt of the first vaccine dose was 138 days, while the mean time to completion of the full vaccination course was 203 days.

**Conclusions**: In our large, urban multi‐site cohort, measles seroprevalence remained high. Among those seronegative, vaccination uptake was low, with delays between screening and vaccine administration, likely reflecting routine clinic follow‐up intervals. This highlights the need for more targeted vaccination strategies, particularly for younger individuals, to reduce the risk of measles infection in people with HIV.

**References**

1. Molton J, Smith C, Chaytor S, et al. Seroprevalence of common vaccine‐preventable viral infections in HIV‐positive adults. J Infect. 2010;61(1):73–80. doi:10.1016/j.jinf.2010.04.004

2. Geretti AM, on behalf of the BHIVA immunisation in adults with HIV guidelines writing group. BHIVA position statement on measles in people living with HIV. London: British HIV Association; 2024. Available at: https://bhiva.org/wp‐content/uploads/2024/10/BHIVA‐position‐statement‐on‐measles‐in‐people‐living‐with‐HIV.pdf.pdf. Accessed 10/06/2026.

**TABLE 1:**
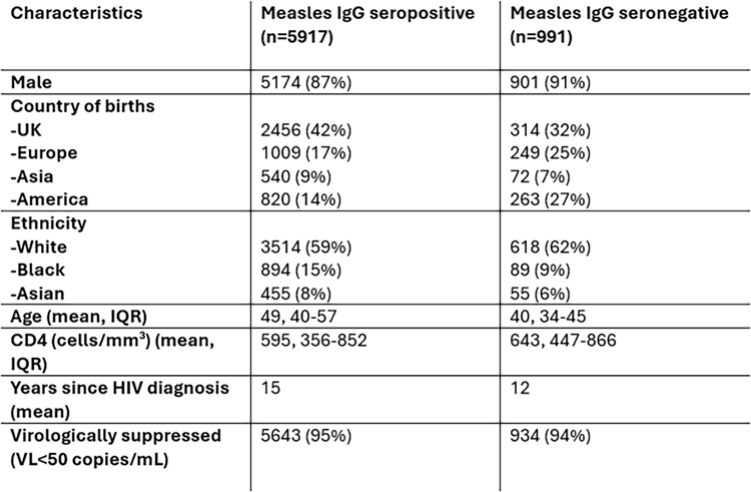
P514 | Table illustrating characteristics of people with HIV screened for measles immunity.

### P515 Archived HIV Drug Resistance Mutations Predict Virological Failure in Virologically Suppressed Individuals on First‐Line Antiretroviral Therapy in Botswana

Dorcas Maruapula ^1^, Ontlametse T. Choga^1^, Wonderful T. Choga^1^, Natasha Mankge^1^, Catherine Koofhethile^1,2^, Joseph Makhema^1^, Roger Shapiro^1,2^, Shahin Lockman^1,2^, Sikhulile Moyo^1,2^, Simani Gaseitsiwe^1,2^

^1^Botswana Harvard Health Partnership, Gaborone, Botswana; ^2^Department of Immunology and Infectious Diseases, Harvard T.H. Chan School of Public Health, Boston, MA, USA

**Background**: Antiretroviral therapy (ART) has greatly improved viral suppression in people living with HIV (PLWH). However, the information on the impact of archived proviral drug resistance mutations (DRMs) on treatment outcomes is limited. We investigated the role of archived HIV DRMs in predicting virological failure (VF) among individuals exposed to first‐line non‐nucleoside reverse transcriptase Inhibitors (NNRTIs) and first‐line dolutegravir (DTG)‐based ART in Botswana.

**Methods**: We analysed proviral HIV‐1 *pol* sequences from participants in the Botswana Combination Prevention Project (BCPP) (2013 and 2018) aged 16 years or older with baseline virological suppression (≤400 copies/mL). The included participants were on known first‐line NNRTI or DTG‐based regimens for at least 6 months, and without evidence of APOBEC‐driven hypermutation. All participants had at least two follow‐up viral load measurements. We identified major DRMs using the Stanford HIV Drug Resistance Database. The multivariable Cox proportional models were used to identify predictors of VF. Virological failure was defined as two or more consecutive viral loads of > 400 copies/mL.

**Results**: A total of 1317 PLWH with baseline virological suppression were included in our analysis. The median age was 43 years (Q1, Q3: 36, 50) and 73.4% were female. Most participants (97.2%) were on first‐line NNRTIs, and 2.8% were on first‐line DTG‐based ART. Archived DRMs rates to any drug class were 22.8% (301/1317). Virologic failure occurred in 3.3% (43/1317) of participants. When adjusting for sex, age, duration on ART and alcohol consumption, archived DRMs were significantly associated with virological failure (adjusted hazard ratio [aHR] 2.32, 95% confidence interval [CI] 1.09–4.95, *p* = 0.03) (Table 1).

**Conclusions**: Archived DRMs among virally suppressed individuals on first‐line ART predicted confirmed VF. These findings warrant larger studies on the impact of archived DRMs among virally suppressed individuals to understand the mechanisms driving future virological failure. Drug resistance is common among PLWH failing ART, dominated by NNRTI.

**TABLE 1:**
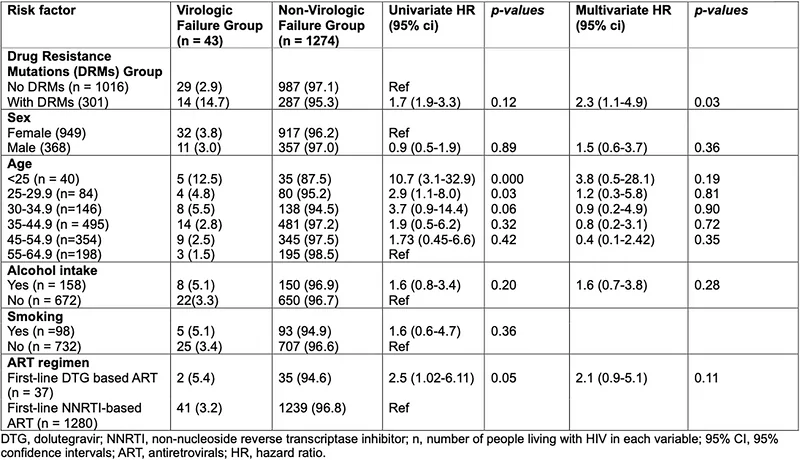
P515 | Factors associated with virologic failure (viral load > 400 copies/mL) among people living with HIV with previous viral suppression on ART in Botswana.

### P516 Abstract Withdrawn

### P517 Reactogenicity and Immunogenicity of the Recombinant HZ/su Vaccine in People Living With HIV

Valentina Sala^1^, Roberta Rovito^1^, Elena Zaninetta^1^, Claudia Acampora^1^, Marco Iannetta^2,3^, Livia Benedetti^2^, Matteo Augello^1^, Valeria Bono^1^, Camilla Tincati ^1^, Giulia C. Marchetti^1^

^1^Department of Health Sciences, Clinic of Infectious Diseases, University of Milan, ASST Santi Paolo e Carlo, Milan, Italy; ^2^Infectious Disease Clinic, Policlinic of Tor Vergata, Rome, Italy; ^3^Department of Systemic Medicine, Tor Vergata University, Rome, Italy

**Background**: People with HIV (PWH) have increased risk of herpes zoster (HZ) and related complications. Recombinant subunit vaccine (HZ/su) is recommended for immunocompromised, but data on safety and immunogenicity in PWH, especially with low CD4 T‐cell counts, remain limited. This study evaluated HZ/su reactogenicity and immunogenicity in PWH according to immune status.

**Methods**: Virally suppressed PWH receiving two HZ/su‐doses 2–6 months apart were consecutively enrolled. Peripheral whole blood was collected before first dose (T0) and 1 month post‐second (T1) for serum isolation. Anti‐glycoprotein E (gE)‐specific IgG antibodies were quantified with in‐house ELISA standardized against a commercial reference standard (25/B960 Varicella‐Zoster Serology Reagent, NIBSC), including sera from healthy HZ/su‐vaccinated as internal controls. Reactogenicity was assessed after completion of the cycle with standardized questionnaire recording local (pain, erythema, swelling, itching) and systemic (fever, fatigue, myalgia, headache, lymphadenopathy, skin rash) adverse events (AEs).

**Results**: 182 PWH were included: 157 with CD4 T‐cell counts ≥350 cells/mm^3^ (high), 25 with < 350 cells/mm^3^ (low) (Table 1). No statistically significant differences in the frequency of AEs were observed between groups (9/16, 56% and 51/115, 44%, low and high, *p* = 0.301), with injection‐site pain being the most frequently reported AE. Similarly, severity and duration of AE were comparable between groups. No vaccine‐related serious AEs were observed. Overall, gE‐specific IgG significantly increased at T1 as compared to T0 (Figure 1A). When stratifying participants according to CD4 T‐cell counts, no significant differences were observed at T1 between groups (Figure 1B). No significant correlation was found between gE‐specific IgG levels at T1 and CD4 T‐cell nadir in the overall cohort nor according CD4 T‐cell counts (overall: *r* = −0.1, *p* = 0.372; low: *r* = 0.3, *p* = 0.245; high: *r* = −0.1, *p* = 0.331). No significant differences in gE‐specific IgG levels were observed between subjects experiencing AEs (AE+) and those without (AE−) (73.5 IU/mL, 48.9–149.2 IQR vs. 96.4 IU/mL, 52.6–154.8 IQR, AEs+ and AEs−, *p* = 0.677).

**Conclusions**: One month after completion of vaccination schedule, Hz/su vaccine elicited a robust humoral response in PWH regardless of immune status and CD4 T‐cell nadir. The vaccine was well tolerated, and the magnitude of immune response was not associated with the frequency of AEs. These data confirm robust vaccine immunogenicity even in moderately immunocompromised individuals.

**FIGURE 1:**
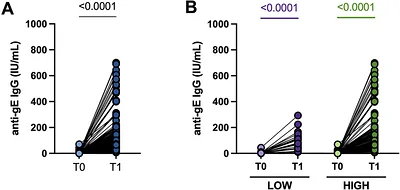
P517 | Humoral response in PWH: level of anti‐gE IgG antibodies in IU/mL as determined by ELISA in the whole cohort (A) and stratified according to CD4+ T‐cell count (B). Low: people with HIV with CD4+ T cell count below 350 cells/mm^3^. High: people with HIV with CD4+ T cell count above 350 cells/mm^3^. T0, before 1st dose; T1, 1 month post‐2nd dose. Longitudinal analyses (T1 compared to T0): Wilcoxon signed‐rank test; cross‐sectional analyses (at each time points): Mann−Whitney U test; *p* < 0.05 considered statistically significant.

**TABLE 1:**
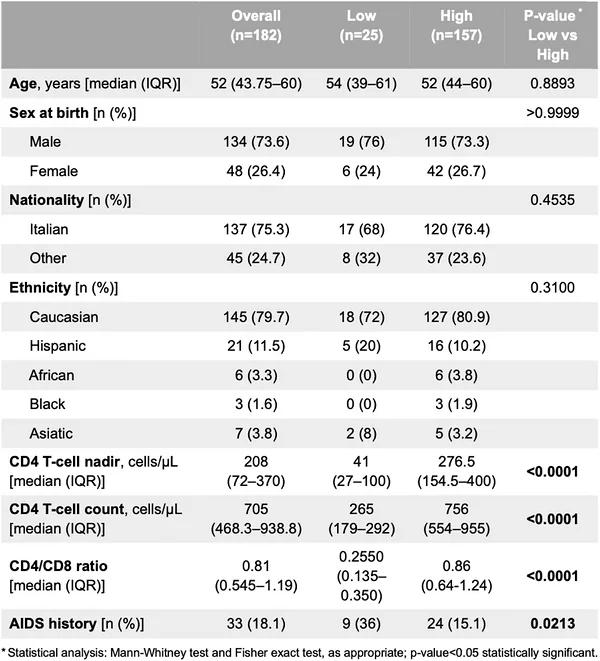
P517 | Demographic characteristics of the study population.

### P518 Abstract Withdrawn

### P519 The Unexpected Mimics of HIV Serology: Clinical Predictors of False‐Positive HIV Screening Results in a 10‐Year Retrospective Cohort Study

Yusuf Emre Ozdemir^1^, Enes Topcu^1^, Busra Nur Ozkan Ertay^1^, Kubra Nur Bali Yurdakul^1^, Samiha Akkaya^1^, Damla Koklu^2^, Zulal Zeynep Yurdakul^2^, Deniz Borcak ^1^, Aysegul Inci Sezen^1^, Sevtap Senoglu^3^, Habip Gedik^1^, Kadriye Kart Yasar^1^

^1^Department of Infectious Diseases and Clinical Microbiology, Bakirkoy Dr. Sadi Konuk Training and Research Hospital, University of Health Sciences, Istanbul, Türkiye; ^2^Department of Medical Microbiology, Bakirkoy Dr Sadi Konuk Training and Research Hospital, Istanbul, Türkiye; ^3^Department of Infectious Diseases and Clinical Microbiology, Medipol Bahçelievler University Hospital, Istanbul, Türkiye

**Background**: Despite the excellent performance of fourth‐generation HIV‐1/2 antigen/antibody assays, data defining the clinical predictors of false‐positive screening results remain limited. False‐reactive results can cause significant psychological distress for individuals awaiting confirmatory testing [1] and may also lead to unnecessary investigations as well as potential delays in treatment, particularly in critical patient populations such as those with rheumatologic diseases or malignancies. Such outcomes have been reported with fourth‑generation HIV antigen/antibody (Ag/Ab) assays in patients with various conditions including pregnancy [2], malignant neoplasms [3], vaccinations [4], autoimmune diseases [5] and infections [6]. This study aimed to determine the frequency of false‐positive fourth‐generation HIV‐1/2 antigen/antibody screening results and identify independent clinical predictors of false positivity.

**Methods**: We conducted a retrospective cohort study of adults who underwent fourth‐generation HIV‐1/2 Ag/Ab screening at a tertiary hospital between August 2015 and August 2025. Screening was performed using ELISA (DiaSorin) or ECLIA (Roche) platforms. Reactive samples (index/COI ≥1) underwent confirmatory testing using the Geenius HIV‐1/2 Supplemental Assay and/or HIV‐1 RNA testing. The patient selection process is presented in Figure 1. Demographic and clinical data were retrieved from electronic medical records.

**Results**: Of the patients with initially reactive screening results, 2054 met the study criteria and were included in the analysis (75.4% male; mean age, 39.8 ± 10.4 years). Confirmatory testing identified 1725 (84.0%) patients as true positive and 329 (16.0%) as false positive. The most common comorbidities were hypertension (11.5%, *n* = 237), diabetes mellitus (7.6%, *n* = 157), coronary artery disease (6.9%, *n* = 141), asthma/chronic obstructive pulmonary disease (COPD) (4.7%, *n* = 97) and solid malignancy (3.2%, *n* = 66). Additionally, 49 (2.4%) patients were pregnant. Multivariable logistic regression identified female sex (aOR 3.44, *p* < 0.001), age >65 years (aOR 6.32, *p* < 0.001), pregnancy (aOR 7.36, *p* < 0.001), hypertension (aOR 2.28, *p* < 0.001), solid malignancies (aOR 4.05, *p* < 0.001), rheumatic diseases (aOR 2.83, *p* = 0.022) and multiple sclerosis (aOR 9.69, *p* = 0.018) as independent predictors of false‐positive anti‐HIV screening results. In contrast, VDRL positivity was significantly associated with true HIV infection (*p* < 0.001) (Table 1).

**Conclusions**: Our findings demonstrate that false‐positive fourth‐generation anti‐HIV screening results are influenced by patient‐specific demographic and clinical characteristics rather than occurring randomly. Recognizing these characteristics may facilitate a more individualized interpretation of reactive screening results, reduce unnecessary psychological distress and inappropriate diagnostic interventions, and improve clinical decision‐making. Ultimately, these findings support a more context‐based interpretation of reactive anti‐HIV screening results rather than a uniform approach based solely on assay reactivity.

**References**

1. Bhattacharya R, Barton S, Catalan J. When good news is bad news: Psychological impact of false positive diagnosis of HIV. AIDS Care. 2008;20(5):560–564.

2. Hastie E, Stangl L, Deutsch K, Yin J, Hull A, Spector SA, et al. Case report: Erroneous human immunodeficiency virus‐1 diagnosis in a pregnant woman using Centers for Disease Control and Prevention diagnostic algorithm. Open Forum Infect Dis. 2022;9(8):ofac402.

3. Liu P, Jackson P, Shaw N, Heysell S. Spectrum of false positivity for the fourth generation human immunodeficiency virus diagnostic tests. AIDS Res Ther. 2016;13:1.

4. Ramadan HK, Salah AE, Mohamed ZR, Ayoub A, AboDief A, Badry SH. False‐positive reactivity of fourth‐generation HIV enzyme immunoassay caused by COVID‐19 and other infections. HIV Med. 2025;26(10):1485–1492.

5. Robinson MA, Nagurla SR, Noblitt TR, Almaghlouth NK, Al‐Rahamneh MM, Cashin LM. Falsely positive fourth generation ADVIA Centaur HIV antigen/antibody combo assay in the presence of autoimmune hepatitis type I. IDCases. 2020;21:e00886.

6. Stempel JM, Mora Carpio AL, Puga D, Perloff S. False positive fourth generation HIV test in a patient with severe malaria. Int J Infect Dis. 2019;83:86–87.

**FIGURE 1:**
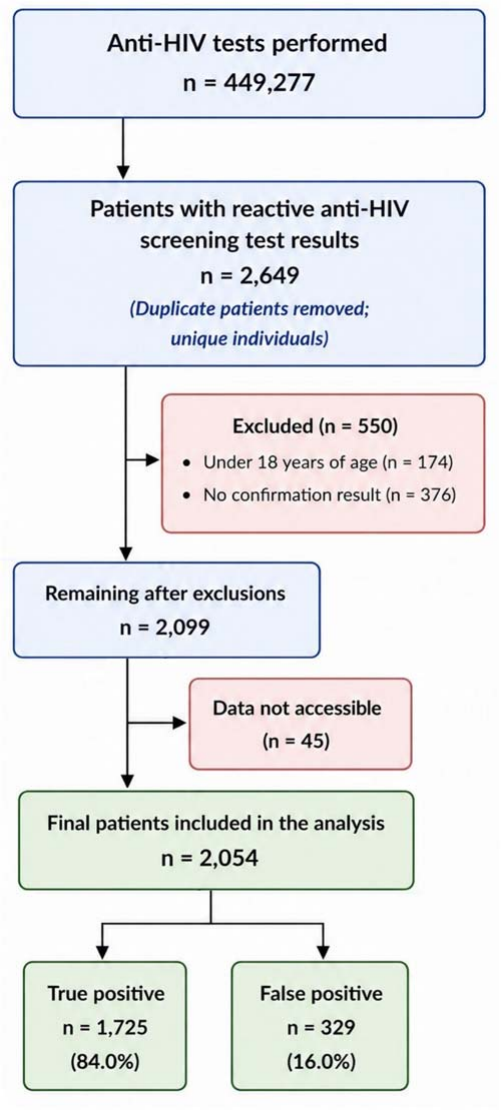
P519 | Flow diagram illustrating patient screening, exclusion criteria and final anti‐HIV positivity breakdown.

**TABLE 1:**
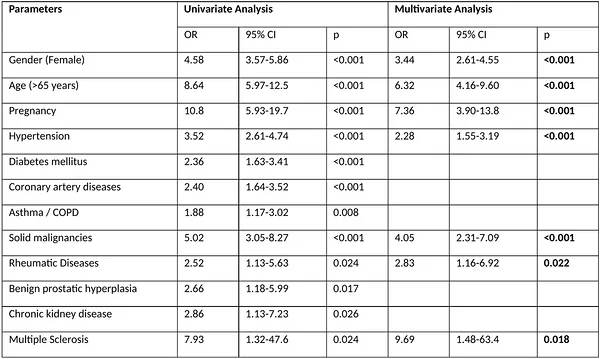
P519 | Univariate and multivariate logistic regression analyses of risk factors associated with false‐positive anti‐HIV test results.

### P520 Lived Experiences and Pathways to Cryptococcal Disease Care and Treatment: Qualitative Insights From People Living With HIV‐Associated Cryptococcal Antigenemia in a Clinical Trial

Tshiamo M. Mmotsa ^1^, Emily Prendergast^1^, Nellie Myburgh^2^, David S. Lawrence^1,3^, Nelesh P. Govender^1,4^

^1^Wits Mycology Division, Wits Health Consortium, Johannesburg, South Africa; ^2^Africa Health Research Institute, Mtubatuba, South Africa; ^3^Department of Clinical Research, London School of Hygiene and Tropical Health, London, UK; ^4^Institute for Infection and Immunity, City St. George's University, London, UK

**Background**: Advanced HIV disease (CD4 count < 200 cells/µL) remains highly prevalent in South Africa and Tanzania and is associated with an increased risk of life‐threatening cryptococcal disease. In both countries, national laboratory screening programmes reflexively test CD4 counts < 200 cells/µL for cryptococcal antigen. A positive test necessitates a lumbar puncture to rule out meningitis and, if negative, treatment with oral fluconazole. Given the limited previous qualitative methods research, we explored the lived experiences and pathways to care and treatment among adults with cryptococcal antigenemia recruited into a clinical trial in South Africa and Tanzania.

**Methods**: In‐depth interviews were conducted with consecutively recruited participants from the “Efficacy of flucytosine and fluconazole as early cryptococcal treatment (EFFECT) trial” (ISRCTN30579828) comparing standard fluconazole monotherapy with combination fluconazole and flucytosine therapy. Interviews were conducted in English, isiZulu, Sesotho and Swahili. Transcripts and field notes were analysed thematically using an integrated framework drawing on four established models: the Consolidated Framework for Implementation Research, Kleinman's explanatory model of illness and disease, the candidacy framework and the theoretical framework of acceptability.

**Results**: Between September 2025 and April 2026 35 participants were recruited. Median age 40 years, 21 females, 22 new diagnosis HIV, 24 in South Africa and 11 in Tanzania. Participants described pathways to care and treatment with cryptococcal antigenemia alongside those for comorbid conditions including HIV, malaria and tuberculosis (Figure 1). Although they initially delayed seeking care, persistent symptoms of immunosuppression such as cough, diarrhoea, weight loss, shingles, joint pain and/or skin rashes prompted them to seek medical attention. Participants reported feelings of fear and worry after their diagnosis, the desire to feel better and live, particularly among those with AHD‐related symptoms, positive interactions with trial staff, support from relatives and awareness that untreated cryptococcal antigenemia could progress to severe meningitis. Negative experiences with antifungal treatment included the high pill burden, complex dosing schedules and side effects such as nausea, vomiting, and skin hyperpigmentation.

**Conclusions**: Pathways to cryptococcal antigenemia care and treatment are complex and dependent on engagement with HIV services. This work highlights the need to strengthen the HIV cascade and provide thorough counselling to address barriers to care and/or treatment uptake.

**FIGURE 1:**
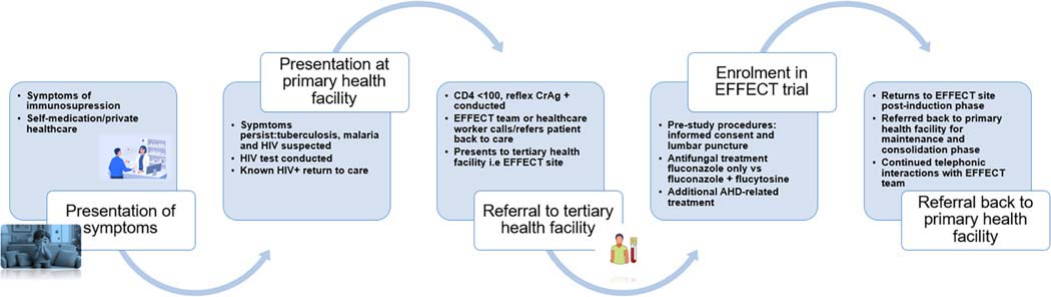
P520 | Described pathways to care and treatment for patients with HIV‐related cryptococcal antigenemia in the EFFECT trial.

### P521 Abstract Withdrawn

### P522 Acetate, a Metabolite of Gut Commensal *Blautia*, Specifically Reverses CD8^+^ T‐Cell Exhaustion in HIV/SARS‐CoV‐2 Co‐Infection

Xuan Yan, Xinyu Zhang, Yinzhong Shen

Department of Infection and Immunity, Shanghai Public Health Clinical Center, Fudan University, Shanghai, China

**Background**: HIV infection establishes a state of chronic T‐cell exhaustion. The impact of SARS‐CoV‐2 infection on this pre‐exhausted immune landscape remains poorly understood. Our preliminary data indicate that HIV/SARS‐CoV‐2 co‐infected patients exhibit exacerbated CD8+ T‐cell exhaustion compared to HIV/AIDS patient without COVID‐19. We observed a significant reduction in the gut commensal *Blautia* and its metabolite acetate, which strongly correlated with exhaustion severity. This study investigates whether the *Blautia*–acetate axis plays a causal and specific role in driving this aggravated phenotype.

**Methods**: We enrolled HIV/SARS‐CoV‐2 co‐infected patients (PC group) and HIV/AIDS individuals without SARS‐CoV‐2 infection (NC group). CD8^+^ T‐cell exhaustion was assessed by flow cytometry (PD‐1, TIM‐3). Gut microbiota composition was analysed by metagenomic sequencing, and plasma metabolites by targeted metabolomics. Ex vivo, CD8^+^ T‐cells isolated from both groups were stimulated with acetate (100–200 µM) in the presence of anti‐CD3/CD28. T‐cell proliferation (Ki‐67), activation (CD69, CD25), function (IFN‐γ, TNF‐α) and exhaustion markers were evaluated by flow cytometry.

**Results**: Co‐infected patients (PC group) showed significantly increased frequencies of exhausted PD‐1^+^CD8^+^ and TIM‐3^+^CD8^+^ T‐cells (Figure 1), along with elevated levels of exhaustion‐associated (IL‐10, TGF‐β) and pro‐inflammatory (sTNFRII, IL‐6) cytokines (all *p* < 0.05). Metagenomic sequencing revealed a significant reduction of the genus *Blautia* in co‐infected patients, which inversely correlated with disease severity (Figure 2). Targeted metabolomics confirmed a stepwise decrease in plasma acetate levels (NC group > mild co‐infected > severe/critical co‐infected). Acetate levels strongly correlated with *Blautia* abundance and PD‐1/TIM‐3 expression on CD8^+^ T‐cells. Ex vivo, acetate treatment (100–200 µM) had a minimal effect on CD8^+^ T‐cells from NC group. Strikingly, in co‐infected (PC group) patients, acetate significantly reversed the exhausted phenotype, as evidenced by reduced PD‐1/TIM‐3 expression, restored proliferative capacity (Ki‐67^+^), and enhanced effector function (increased IFN‐γ and TNF‐α production).

**Conclusions**: Unlike its marginal effect in HIV/AIDS patients without COVID‐19, acetate potently reverses the exhausted phenotype of CD8^+^ T‐cells from co‐infected patients, restoring their proliferative and effector functions. Our findings identify the *Blautia*–acetate axis as a novel, co‐infection‐specific driver of exacerbated T‐cell exhaustion in HIV/SARS‐CoV‐2 co‐infected patients and suggest a potential microbiota‐derived therapeutic target.

**FIGURE 1:**
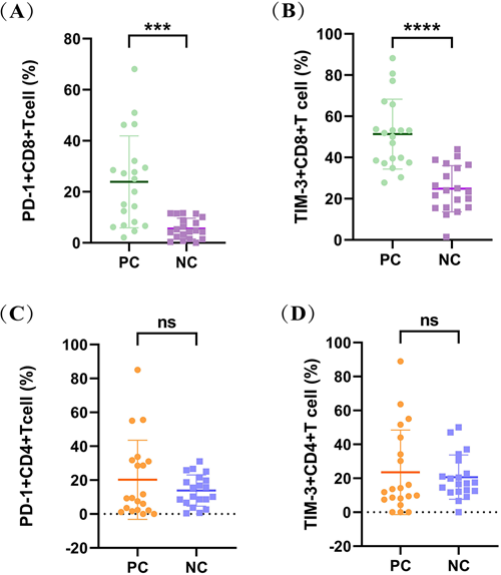
P522 | CD8^+^ and CD4^+^ T cell exhaustion profiles in HIV/SARS‐CoV‐2 co‐infected patients (PC group) and HIV/AIDS individuals without SARS‐CoV‐2 infection (NC group).

**FIGURE 2:**
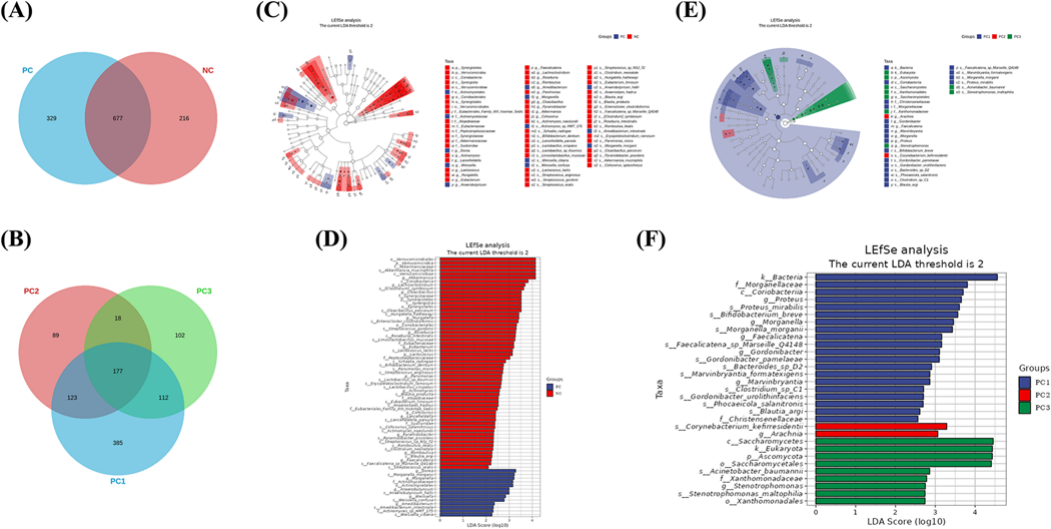
P522 | Gut microbial differences between PC and NC groups revealed by metagenomic sequencing and LEfSe analysis.

## Author Index

### A

Aarnoutse, R. P270

Abbate, I. P192

Abdalla, A. M. H. M. P185

Abdelaal, H. F. S. P185

Abe, K. P272

Abedin, F. P044

Abeguille, C. P101

Abela, I. A. O22, P209, P325, P480

Abeli, C. P081, P082, P504

Aberg, J. P158

Acampora, C. P517

Achayo, H. P244

Adachi, E. P447

Adam, A. P487

Adamis, G. P216

Adams, K. P260

Addo, P. P268

Addou, S. P382

Adebiyi, A. P278

Adriani, B. A. P124, P125, P126

Adrover, A. A. P087

Afani, A. P164, P223

Afifi, M. P240

Afzal, S. P467, P513

Agostini, E. P443

Aguado, A. P320

Aguilar Rodea, B. P. P403

Aguilera García, M. O38.2, P119

Aguiló, R. P357

Agur, L. P230, P340

Aherne, M. P293

Ahlström, M. G. P467, P513

Ahmad, S. P088

Ahmed, N. P308, P462

Aho, I. P145

Ahsanov, S. P341

Ait Moha, D. P182

Aizen, K. P108

Aizire, K. P507

Ajomale, A. O39.2, P020

Akbulut, I. P319

Akca, V. P128, P319

Akhan, S. P395, P482

Akinyomi, M. H. P363

Akkaya, S. P495, P519

Akpinar, M. S. P133

Aksak‐Wąs, B. O39.3, O44

Aksu, D. Z. P472

Al‐Hayani, A. P322

Aladdin, H. P145

Alagaratnam, J. P466

Alain, T. P101

Alao‐Amiola, O. P278

Alarcon, P. P337

Albaksami, O. A. A. P185

Albayrak, A. P319

Albendin Iglesias, H. P103, P104, P105

Albendín, H. P159

Alberdi, N. P500

Alberola, A. P320

Albini, L. P168, P174, P344

Albuquerque de Moraes, C. P235, P236

Alcantar Morales, R. P223

Alcaraz Garcia, A. P103, P105

Aldamiz‐Echevarria, T. P058, P322

Alejandro, H. P188

Aleman, S. P104

Alemayehu, M. P150

Alemán Valls, M. R. O35

Alemán, M. R. P092

Alfonso, A. P485

Alford, K. P121

Alghamdi, O. P141

Ali, B. P141, P386

Ali, B. P121, P332

Ali, S. P101

Allan, B. P275, P276, P277, P468

Allard, S. D. P136

Allavena, C. P073, P175

Almeida, M. P147

Alonso Navarro, R. P130, P178

Alosaimi, R. P141, P386

Alrowais, H. P034, P035, P187

Alshehri, I. P141, P386

Altavilla, E. P394

Altclas, I. P338

Altunsoy, A. P319

Altuntaş Aydın, Ö. P183, P226

Alvarez‐López, P. P485

Alves, H. P116

Alves, J. P147

Amannah, D. N. P290

Ambrosio, S. P396

Ambrosioni, J. P357, P358

Ameil, L. P382

Amoah, S. O39.2, P020

Amstutz, A. P325

Amy, P. P503

Anas, A. P089

Anderson, I. P370

Anderson, S.‐J. P019, P347

Andersson, E. P099

Andreatta, K. P012, P042, P048, P239

Andrews, S. P312

Andriantsoanirina, V. P448

Ankiersztejn‐Bartczak, M. O39.3

Ansaldo, L. P068, P123, P138, P218, P453

Anteyi, K. P238

Antinori, A. P009, P010, P011, P025, P032, P055, P058, P059, P152, P155, P170, P192, P225, P255, P374, P412, P454, P486

Antinori, S. P083, P084, P085, P394

Antolini, L. P479

Antonacci, M. P123, P138

Antonella, V. P125

Antonio, S. P. P301

Antunes, A. P468

Antunes, C. P147

Apea, V. P208, P237, P368, P369

Appella, D. H. P293

Appiah, A. P079, P275, P276, P277, P468

Aractingi, M. P005

Arando, M. P485

Arauz, A. P223

Araújo Abreu, M. P100, P146

Araújo, S. P452

Arcos Rueda, M. d. M. O35, O38.3, P106, P322

Arduino, J. M. P015, P023

Arenas García, V. O38.2

Arenas‐Pinto, A. P056, P057, P419, P421

Arentoft, N. P467

Arévalo, L. P223

Arkadianos, C. P409

Armenia, D. P153

Arnau, I. P210

Arora, P. P048, P167

Arribas, J. R. O38.3, P106

Arrieta, I. P210

Arrifano da Silva, L. P235, P236

Arstikaitiene, G. P497

Arteaga Badillo, D. A. P435

Artelli, F. P081, P479, P504

Asante‐Appiah, E. P060

Asher, I. P254

Asiimwe, S. P. P318

Asquier Khati, A. P175

Assoumou, L. P070, P175, P408

Astocondor, L. P223

Astriti, M. P216

Atalay, S. P319, P472

Ataman Hatipoglu, C. P319

Ates, S. P319

Athanasiou, K. P501

Atkins, K. E. P111

Auaboonkanok, P. P462

Augello, M. P135, P376, P412, P517

Augusto, I. P074, P118, P120

Aulia Rahman, Y. P261

Ausselet, N. P136

Avanzini, I. P414

Averitt, D. P015

Avettand‐Fenoël, V. P455

Avihingsanon, A. P042, P049, P401

Awan, A. P036, P208, P237

Ayakaka, I. O42, P305, P471

Ayer, T. P314, P342

Aymes, E. O34

Azeredo, C. P114, P452

Azevedo, M. P231

Azkune Galparsoro, H. P324

Álvarez Mendoza, J. P. P165

Álvarez Rodríguez, I. P324

### B

Bacelar, B. P221, P429

Bacelar, C. P452

Bachelard, A. P234

Back, H. P267

Badalucco, F. P066

Badhoniya, N. P158

Badia, L. P115

Badshah, C. P017

Baek, J. P102

Bagaglio, S. P171

Bailón, L. P210

Bainbridge, V. P041

Baker, D. P056

Balbi Rocca, V. N. P258, P307

Balcı, M. P319

Baldassari, L. P161

Balde, A. P175

Baldin, G. P083, P084, P085, P134, P394, P477

Balerdi Sarasola, L. P324

Bali Yurdakul, K. N. P519

Ballesteros‐Perez, A. P267

Ballivian, J. P053

Balogh, A. P151

Bamford, A. P002, P241

Bana, N. B. P009, P080, P081, P082, P155, P486, P504

Bandera, A. P412, P465

Barath, J. P026

Barbaro, F. P009

Barber, T. P091, P109

Barchi, V. P009, P010, P192

Barda, B. P422, P423, P426, P427

Barletta, J. Á. P233

Barlow, M. P419

Barnett, S. P166

Barrado Cuchillo, J. P431

Barragán Gallo, P. O38.2

Barrios Sanchez, P. O39.2, P020

Bartalesi, F. P124, P125, P126

Barthoulot, M. O34

Bartl, L. P247, P362

Bartoloni, A. P055, P124, P125, P126

Barvkar, V. P385

Bascombe, F. P419, P421

Bassani, F. P134, P394

Bassetti, M. P093, P142

Bassey, F. P230, P340

Bastug, A. P319

Bates, L. P343

Bather, J. P015

Batisse, D. O39.1, P393

Batista, C. P114, P129

Battagin, G. P115

Bauer, S. P224, P460

Baumela, A. P117

Bax, H. P089

Bayona Carlos, A. P324

Bayrak, A. D. P226

Bayındır, Y. P395

Beer, A. P067

Beer, D. P094, P195

Begovac, J. P037, P475

Begré, L. O34

Behrens, G. M. P049, P057, P408

Bekerman, E. P049

Bekker, L.‐G. O43, P017

Belackova, V. P370

Belkhir, L. P266

Bellagamba, R. P055, P170, P449

Bellati, A. P488

Belli, M. P373, P414, P433

Bellissimo, F. O39.2, P020

Bellocchi, B. P492

Bellon, J. M. P322

Beltrami, A. P412

Beltrán, C. P223

Benabadji, E. P022

Benachir, H. P073

Benalycherif, A. P175

Benassi, L. P396

Bendala Estrada, A. D. O35

Bendall, T. P079

Benedetti, L. P282, P517

Benfield, T. L. O37.3, P371, P467, P513

Benjamin, L. D. P019

Benn, P. P041

Benseñor, V. A. P258, P307

Benson, P. P058

Beraud, G. P263

Beretta, D. P003

Berg, S. K. P207

Bergna, A. P174, P213

Berhe, M. O31, P042, P076, P077

Bermejo Plaza, L. P473

Bernabeu‐Martínez, M. Á. P360, P367

Bernacchia, D. P229

Bernal Morell, E. O35, O38.2, P103, P104, P105

Bernal, E. P092, P119

Bernardini, S. P282

Bernardino, J. I. O38.3, P092, P106, P159, P431

Bernasconi, D. P. P504

Bernasconi, E. P422, P423, P426, P427

Berner, A. O44

Berrocal, L. P357, P358

Berti, G. P196

Bertocchi, V. P068

Bertola, B. P422, P423, P426, P427

Bertolini, M. P233

Berzigotti, A. P400

Bezaitis, K. P206

Bezemer, D. P304

Bezenchek, A. P153

Bhattacharyya, A. P268

Bianchi, A. P373

Bianco, C. P124, P125, P126

Bianco, D.‐M. P260

Biasoli, L. P486

Bickel, M. P151, P180

Biello, K. B. P023

Bierman, W. O37.2

Bima, M. L. M. Y. P261

Bini, C. P346

Bini, T. P155, P413

Biondi, L. E. P399, P407, P414

Birengel, M. S. P319

Birmingham, E. P039

Bisbal, O. P322, P473

Bisch, A. P382

Bissanti, M. P. P229

Bittner, J.‐L. P195

Bitu, M. P455

Bivona, D. P219

Blackwell, J. P297

Blanco, J. R. P119

Blanco, J. L. P357, P358, P485

Blanco, M. P489

Blazquez Garrido, R. P103, P105

Blissett, R. P347

Bobbio, N. P255

Bock, A. O37.3

Boffito, M. P050, P054, P091, P095, P302, P364, P466

Bogart, M. P203, P217

Bois, J. P263

Bolache, S. P364

Bonfanti, P. P055, P081, P082, P413, P479, P504

Bongiovanni, M. P422, P423, P426, P427

Bonjoch, A. P210

Bonnet, F. P448

Bono, V. P135, P376, P517

Bonora, S. P152, P171

Bontempo, G. P039, P041

Bontemps, E. P461

Borcak, D. P495, P519

Borch, J. P061

Bordi, L. P486

Bordignon Antonio, M. P384

Borges de Pinho, R. P028

Borghesi, A. P214

Borghetti, A. P083, P084, P134, P394

Borghi, F. P202

Borsi, R. P051, P193

Bossi, G. P214

Bottaro, E. P221, P416, P429

Bouee, S. P086

Boufassa, F. P455

Boukli, N. O39.1

Bourgeois, C. P455

Bourgey, M. P448

Bowden, B. H. P387

Boyd, A. O44, P400, P480

Braccialarghe, N. P192, P486

Bragato, B. P176, P196

Branger, J. P391

Braun, D. O22, O34, P247

Braun, P. P151, P195

Bravo Urbieta, J. P058, P103, P104, P105

Brazille, P. O39.1

Bresciani, L. P218

Bright, P. P063

Brinkman, K. O37.2, P006, P182, P348, P349

Brita, A. C. P449

Brites, C. P053, P384

Brizuela, M. P221, P416, P429

Brogan, A. P. P194

Brogonzoli, L. P458

Brough, G. P079, P275, P276, P277, P468

Brouwer, J. P046

Brown, J. P312

Brown, J. A. O42, P305

Brown, K. P016, P078

Brown, L. P004

Brown, V. P297

Bruggemann, R. P270

Brugger, S. P480

Bruja, P. P283

Brunel, S. O39.3, P091

Brunet, L. P013, P016, P078

Bruni, G. P224, P460

Bruno, A. P422, P423, P427

Bruno, C. J. P015

Bruno, R. P214, P491

Bruno, R. P219

Bruxvoort, K. J. P023

Bruzzesi, E. O36, P003, P066, P444

Bruzzone, B. P153

Buchbinder, S. P015

Budnik, P. P013, P051, P052, P053, P154, P158, P193

Bufali, M. V. P066

Buffa, A. P443

Buffagni, F. P107, P415

Buggisch, P. P381

Bui, M. P076, P077

Bukusi, E. A. P017

Bull, L. P514

Burastero, G. P080, P082

Burger, D. P270, P271, P348, P349

Burns, F. P109, P302

Burns, J. P392

Bursac, Z. P291

Buruian, N. P231

Busca, C. O38.3, P106

Bustinduy Odriozola, M. J. P324

Bustos Angel, E. D. P165, P200

Butini, L. P373

Buzón, L. O35, O38.2, P092

Byrne, L. P212

Byrne, L. P285

Byrne, R. P514

### C

Cabalak, M. P071, P319

Caballero Asensio, R. O35

Cabanas, J. P181

Cabello, A. P322

Cabello‐Clotet, N. O38.2, P087, P092, P322

Cabello‐Rojano, I. P322

Cachapa‐Vieira, I. P511

Cacopardo, B. S. P219, P492

Cahn, P. P052, P053, P058

Cai, L. P143

Cai, Q. P194

Cai, R. P418

Caioli, A. P192

Cairó, M. P092

Calame, A. O34

Calandra Buonaura, F. P107, P402, P407

Calandrino, L. P176

Calcagno, A. P054, P118, P380, P458

Caldas, C. P028, P162

Caldera, R. P011, P032, P170

Calderón Hernáiz, R. O38.2

Callaghan, L. P203, P217

Calle, I. P446

Callebaut, C. P012, P048

Calmy, A. O22, O34, P049, P057, P091, P173, P325

Caluwaerts, S. P005

Calvi, U. P107, P402

Calvo, J. P357, P358

Calza, L. P115

Calzado Isbert, S. O38.2, P130, P178, P335, P511

Calzavara, D. P300

Camacho, A. P117, P459

Camard, M. P455

Camargo, C. P036, P208, P237

Camino Ortiz de Barrón, X. P324

Campbell, T. B. P049

Campos Cano, C. P463

Candevir, A. P071, P336, P395

Canetti, D. P094, P420, P493

Cano Díaz, A. L. P404, P425, P434, P435, P463, P510

Cano, A. P283

Cao, X. P293

Capeau, J. P382

Capra, N. O36, P003, P066, P163, P171, P389, P420, P442, P444, P464, P493

Carbone, A. P081, P394, P477, P504

Carleo, M. A. P084

Carles, M. P382

Carlicchi, A. P073

Carmona de la Torre, F. d. A. P324

Carmona, I. P117

Caro Vega, Y. P337, P506

Carobbio, A. P415

Carpena Martínez, I. P103

Carpena, I. P105

Carr, A. P063

Carr, E. P275, P276, P277

Carraro, A. P068, P123, P138, P218, P453

Carriero, E. P107, P415

Carroll, K. P197

Carrozzo, G. P174

Carstens, R. P. P268

Carter, C. C. O21, P012

Caruso, E. P009, P025, P032, P280

Caruso, F. P010, P486

Carvalho, R. P228

Casabianca, A. P135

Casado, J. L. P378

Casal, M. C. P154

Casari, F. P465

Cascio, M. P373

Cassetti, I. P057, P221, P223, P333, P416, P429

Cassidy, T. P095

Castagna, A. O36, P003, P009, P011, P055, P066, P163, P171, P225, P389, P412, P420, P444, P445, P464, P486, P493

Castanedo, G. P234

Castellanos, E. P275, P276, P277, P468

Castilho, J. P384, P506

Castillo‐Mancilla, J. R. P041

Castillo Navarro, A. P103, P104, P105

Castro, A. P373

Castro, H. P462

Castronova, E. P039, P267

Castronuovo, P. P017

Catala, A. P485

Catalano, G. P003, P464

Cattelan, A. P011, P032, P055, P085, P115, P163, P176, P196, P443

Cavallari, E. N. P192

Cavallero, L. P280

Cavassini, M. O22, O34, P173, P209, P325

Cavinato, S. P176

Cazanave, C. P448

Cárdenas, G. P224, P460

Cázerez Villareal, L. E. P403

Ceballos, M. C. P485

Ceccherini‐Silberstein, F. P153, P225

Cecchini, D. P221, P416, P429

Cecere, S. P032, P373

Cecilia, F. P282

Celesia, B. M. P219, P458, P492

Celesia, M. P171

Celum, C. L. P017

Cenderello, G. P171, P406, P413

Cenoz Gomis, S. P118

Cents‐Bosma, A. P391

Cernuschi, M. P011, P025, P032, P280

Cervo, A. P083, P084, P085, P134, P171, P394, P442, P445, P465

Chaganti, A. P466

Chaix, M. P393

Chakrabarti, T. O33

Chana, T. P036, P208, P237

Chandan, K. P045

Chang, H.‐Y. P417

Chang, H.‐M. P264

Chang, J. P041

Chang, S. P356

Chang, S.‐Y. P191

Chaparro Sánchez, A. P404, P425, P434, P435, P463, P510

Charilaou, Y. O39.2, P020

Charpentier, C. P234

Chatterjee, A. P052, P058, P059, P076, P077

Chaudron, S. P209

Chavez, J. P326

Checchi, D. P374

Chen, A. P.‐L. P072

Chen, C. P418

Chen, C.‐P. P072, P191

Chen, I.‐T. P321

Chen, J.‐K. P321

Chen, J. P405

Chen, K. P049, P050, P056

Chen, L.‐Y. P417

Chen, S. P144

Chen, T.‐C. P072

Chen, W. P045

Chen, X. P045

Chen, Y. P189

Chen, Y.‐T. P417

Chen, Y. O21

Cheng, C.‐Y. P072, P191, P243, P286, P512

Cheng, S.‐H. P072, P094, P191, P243, P286, P512

Cheon, S. P140

Cherabie, J. P014

Cherian, R. T. P185

Chetchotisakd, P. P056, P057

Chi, J. P418

Chi, S.‐W. P191

Chiagorom, P. P230

Chiang, Y.‐H. P284

Chin, B. S. P148

Chirio, D. P382

Chivite, I. P357, P358

Choga, O. T. P303, P334, P515

Choga, W. T. P303, P515

Choi, J. Y. P102

Chopamba, D. P150

Chou, P.‐Y. P264

Chouhan, M. P392

Choy, C. Y. P095

Christensen, B. P246

Christinet, V. O22

Christoph, M. P127

Chu, J.‐C. P007, P021

Ciani, O. P066

Ciardi, M. R. P123

Ciarpaglini, S. P282

Cicconi, P. P121

Ciccullo, A. P083, P084, P085, P134, P394, P477

Cicek Senturk, G. P319

Cielniak, I. O44

Cimaglia, C. P445

Cimini, E. P010

Cingolani, A. P380, P445, P465

Cinque, P. P458

Cipriano, A. P481

Ciudad Sañudo, M. P257, P431

Clarke, A. O21

Clarke, E. O21, P027, P121

Clavero Olmos, M. O38.2

Clemente, T. O36, P003, P066, P163, P171, P389, P406, P420, P442, P444, P464, P493

Clementi, N. P486

Clutterbuck, D. P121

Coaquira Castro, J. P. P314, P342

Cochran, Q. P078

Coco, M. P492

Coello, G. P337

Coetzee, J. P238, P239

Cogliandro, V. P479

Cohen, E. P428, P505

Cohen, J. P127

Cohen, R. P254

Colafigli, M. P225

Colaiuda, S. P477

Collaco‐Moraes, Y. P392

Collado, D. P087

Collarino, R. P073

Colle, R. P455

Collins, B. P091

Collins, I. J. P462

Collins, J. P043, P267

Collins, S. P091

Cologgi, G. P082, P504

Colombo, V. P174

Colson, A. E. P049, P056, P057, P063

Columpsi, P. P479

Comer, A. P308

Comi, L. P055, P082, P161, P202, P406, P504

Conway, B. P096, P097, P098

Cooper, O. P046

Copas, A. P419

Copertari, G. P221, P416, P429

Cordel, H. O21

Cordes, C. P061

Cordioli, M. P488

Corona‐Mata, D. P117, P459

Corral, E. P337

Correia de Abreu, R. P114, P129, P156, P452

Corruble, E. P455

Corsi, P. P124, P125, P126

Cosentino, F. P492

Cosh, A. P332

Cossu, M. V. P055, P084, P174, P213, P394, P445, P458

Costa I Otal, E. P234

Costa, C. P100, P146

Costa, C. P124, P125, P126, P163, P380

Costa, I. P181

Costantini, A. P168

Costarelli, S. P124, P125, P126

Costiniuk, C. T. P186

Cotrufo, M. P412

Cotter, A. G. P366

Coutinho Iglessias, A. C. P235, P236

Coutureau, C. P173

Cox, J. P026, P186

Cox, S. P012

Cozzi‐Lepri, A. P055, P225, P374, P465

Córdova, E. P053

Crabtree Ramirez, B. P337, P401, P506

Crauwels, H. P039

Cresswell, F. P121

Cretella, S. P115

Crichton, S. P462

Crippa, F. P202

Cristina, L. P228

Crofoot, G. O31

Cron, K. P487

Crone, A. P014

Crusells Canales, M. J. O35, P092

Cruz León, M. A. P425

Cruz, G. P116

Cua, E. O21, P382

Cucinotta, G. P477

Cueto‐Marquez, M. P511

Cuevas, G. P456

Culakova, E. P127

Cullen, L. P260

Cullis, E. P260

Cummins, T. P293

Cuomo, G. P399, P402

Curley, J. P421

Curran, A. P159

Custers, D. P006

Cybula, A. P199

Çal, G. P183

Çalık Başaran, N. P395

Çelen, M. K. P071, P395

Çelik, D. P472

Çitil, B. E. P128

Çiçek, Y. P395

### D

D'Agostino, R. P046

D'Amico, R. P039

D'Antoni, M. L. P048, P090, P127

D'Arminio Monforte, A. P152, P155, P168, P255, P374, P380, P465

D'Avolio, A. P010, P161

D'Ettorre, G. P083, P084

Dabis, R. P364

Daeumer, M. P151

Dai, B. P047

Dakhia, S. P058, P059

Dal Zoppo, S. P465

Dalla Valle, G. P462

Dalmia, M. P050

Dalton, T. P293

Dam Nielsen, S. P415

Damar Çakırca, T. P071, P319, P395

Dams, M. O39.3, P091, P203, P217

Danchenko, N. P019

Daniela Silva, M. P481

Danise, A. P003

Daramola, O. P197

Darcis, G. P136

Das, M. O21

David, A. P241

Davidson, J. P108

Davis, A. P019

Dawaher, J. P223

De Almeida Mota, R. P162

De Almeida Teixeira, R. P100, P146, P481

De Braga Castro, R. A. P235, P236

De Carolis, L. P224, P460

De Castro, N. P070

De Gea Grela, A. O38.3, P092, P106, P302

De Gennaro, M. P124, P125, P126

De Greef, J. P266

De Haan, M. P271

De la Asunción Villaverde, M. J. O35, P092

De la Fuente, S. P322

De la Fuente, V. P456

De la Mora, L. P357, P358

De la Porte des Vaux, C. O39.1, P393

De Lagarde Sebastián, M. P049, P051, P092, P473

De Lazzari, E. P357, P358

De León Aguilar, A. P425

De los Rios, P. P058, P059, P074, P076, P077, P118, P120

De los Santos Gil, I. O35, P033, P092, P119

De Masi, M. P138

De Masi, M. P218, P453

De Mendonca Melo, M. P089

De Miguel‐Buckley, R. O38.3, P106

De Miguel Montero, M. P033, P322

De Montigny, L. P015

De Paula dos Anjos, V. P235, P236

De Pokomandy, A. P149, P186

De Prada, A. M. P224, P460

De Regt, M. P182, P271

De Salazar, A. P250, P320

De Santis, E. P453

De Scheerder, M.‐A. P136

De Socio, G. V. P374, P376, P413

De Souza Loureiro Abbud Santos, L. P236

De Vito, A. P083, P084, P153, P168, P380, P394, P431, P466

De Vries‐Sluijs, D. O37.2, P089

De Wit, S. P136, P408

De Zottis, F. P055, P170, P449

Defres, S. P457

Degen, O. P151

Degli Esposti, L. P344

Deig Comerma, E. P130, P178

DeJesus, E. O31, P040, P062

Del Borgo, C. P068, P123, P138, P218, P453

Del‐Campo‐Terrón, S. P087

Del Duca, G. P170, P449, P454

Del Negro, V. L. P009, P011, P025, P032, P373

Del Rio Perez, O. P130, P178

Delany‐Moretlwe, S. P017

Delatango, K. P036, P208, P237

Delforge, M. P005, P136

Delgado, E. P223

Delgado‐Sánchez, E. P360, P367

Dellaqua, C. P235, P236

Delmonte, E. P414

Delobel, P. P461

Delpech, V. P312

Deltoro, M. G. P095

Demeester, R. P136

Demir, T. P319, P336

Demirdal, T. P319

Demirkırkan, E. P183

Demirok, B. P183

Den Hollander, J. O37.2, P094

Dennis, C. P392

Depouilly, B. P247

Derin, O. P183, P226

DerSarkissian, M. P194

Descalzo, V. P485

Deschanvres, C. P175

Desclaux, A. P094

Deveci, A. P319

Devitt, E. P377

Devonald, M. O39.3, P036, P091, P208, P237

Dewsnap, C. P019

Di Biagio, A. P055, P093, P142, P163, P168, P171, P412, P413, P465, P504

Di Gennaro, E. P080

Di Giambenedetto, S. P081, P083, P084, P085, P134, P225, P394, P406, P442, P458, P477, P504

Di Lorenzo, A. P282

Di Nardo, A. P453

Di Perri, G. P344

Di Trento, D. P218, P453

Diamond, T. L. P049, P060

Diana, D. P389, P406, P420, P444

Diaz de Santiago, A. P322

Diemer, M. P070

Dietz, C. A. P063

Digumber, M. P175, P234

Diogo, I. P181

Diop, A. K. P296

Diouf, É. K. P296

Distefano, D. P442

Dixon, J. P121

Díez Romero, C. O38.2

Doblas, A. P117

Dobrzyńska, M. P024, P110

Doerholt, K. P462

Dolle, C. P247, P362

Dominelli, F. P123, P138

Domingo, P. P408

Dominguez, M. P194

Donaire, J. C. P322

Donini, V. P085, P124, P125

Donnell, D. P017

Donovan, C. P043, P158

Dorey, D. P039

Dorresteijn, J. O37.1

Dorta, C. P322

Dousa, S. P073

Dowiatt, M. P024, P110

Doyuk Kartal, E. P319

Dreesen, E. P266

Dretler, A. W. P063

Dreyfus, J. P223

Dręczewski, M. P024, P110

Duarte Rocha, S. P114

Duarte, A. I. P147, P181

Duarte, F. O39.3, P114, P129, P156, P452

Duarte‐Walker, F. P468

Dubois, V. P038

Duffell, E. P306

Dugal, A. P076, P077

Duncan, A. P157

Dunham, R. P045

Dunn, D. P392

Dunn, K. P056, P087, P166, P167

Duong, R. P393

Duracinsky, M. P101

Duran, F. P322

Durant, J. P382

Duru Oz, E. P319

Duvivier, C. O39.1, P101

Dvory‐Sobol, H. P049, P050, P056, P057

Dworkin, M. P206

Dyda, T. P024, P110

Đorđević, N. P438

### E

Echeverría, P. P049, P056, P057, P210

Edali, M. P342

Edwards, L. P345

Edwards, S. P197, P364

Effiong, A. P230, P340

Egger, M. P111

Egharevba, I. P230

Egido Murciano, M. V. O38.2

Egle, A. P067

Ehmer, J. P305

Ehret, R. P253, P484

Eimutiene, A. P497

Eisenberg, A. P476

Eithne McKeand, E. P036

El Khalili, O. P153

El Kozah, Y. P428, P500, P505

Eland, N. P203, P217

Elbirt, D. P254, P476

Elens, L. P266

Elhabbak, E. M. A. P185

Elias, R. O37.1, P391, P424

Elie, B. P247

Elijah, O. P363

Elinav, H. P476

Elliot, E. P051, P193

Elliott, T. P365

Elmaslar Mert, H. T. P319

Elvstam, O. P372

Elyanu, P. P238, P239

Elzi, L. O22, P173, P325

Endo, T. P094, P383

Ene, L. P462

Enejoh, V. P230, P340

Engler, K. P149, P186

Ensaroglu, E. P495

Enzenhofer, M. P224, P460

Erdogan, H. P319

Erevbenagie‐Johnbull, E. O39.2, P020

Erlandson, K. P118

Eroğul, E. P319

Ersoz, G. P319

Ertürk, D. P071, P336, P395

Escrich, C. O38.2

Escura Esteban, M. P130, P178

Espinoza, R. M. P356

Esposito, V. P085

Esser, S. P151, P381, P408, P440

Estany, C. P210

Estebánez, M. O38.2, P322

Estrada‐Pérez, V. P087

Esvan, R. P192

Etienne, M. P461

Evans, B. P015

Everitt, A. P450

Eyo, A. P363

Eyre, M. P370

Ezeobi, O. P230

### F

Fabbiani, M. P055, P083, P085, P124, P125, P134, P394

Fabeni, L. P170, P192

Fabre, R. P382

Facciolo, F. P202

Fagone, P. P219

Fal, A. P343

Falcão, M. P147

Fanjul, F. O35, P092

Faria Pereira, P. P235, P236

Farinacci, D. P009, P010, P192, P486

Fariñas Álvarez, M. C. O35, O38.2

Farnham, A. O22

Faro, N. P087

Fasani, G. P115

Faustino de Medeiros, M. F. P235, P236

Fehr, J. O22

Fennell, I. P297

Fenske, S. P487

Fernandez‐Chica, C. P378

Fernandez Lopez, M. A. P103, P104, P105

Fernández, A. O35, O38.2

Fernández‐Blest, M. P087

Fernández‐López, M. P087

Fernández‐Montealegre, A. P367

Ferrando, S. P382

Ferrara, S. P413

Ferrari, A. P176, P196, P443

Ferreira, S. P481

Ferreira Monteiro, F. M. P258, P307

Ferreira Santos, C. P235, P236, P283, P384

Ferreira‐Tata, C. P117, P459

Ferrer, P. P164

Ferrero, F. P428, P505

Ferrero Benéitez, Ó. L. O38.2

Festa, G. P163, P380, P389, P406, P420, P464

Fiammingo, O. O34, P325

Fiaschi, L. P161

Fidler, S. P302, P450

Figueroa, M. I. P053

Fihman, V. O39.1

Filippi, V. P218, P453

Filis, C. P409

Fito, L. P092

Flaherty‐Gupta, A. O39.2, P020, P353

Flanagan, S. P308, P419, P421

Flanczewski, S. P199

Fleming, S. P064

Flisiak, R. P343

Florence, E. P408

Foncillas, A. P357, P358

Fonsi, M. P235

Foote, L. P079

Forbici, F. P170

Forcina, G. P011, P032, P168, P170, P174, P344

Forero, D. P223

Fornabaio, C. P442

Forni, A. P080

Forniti, A. P192

Forthmann, J. P487

Fortin, J.‐F. P150

Fortunato, V. P451

Fox, D. P275, P276, P277

Fox, J. P049, P121, P392, P408

Fox, M. C. P060, P063 P401

Fraaij, P. P089

Francis, K. P001, P002, P212, P241

Franco, C. P028

Frange, P. O39.1

Franzin, M. P202

Franzò, A. P219, P492

Frate, G. P009, P011, P025, P032, P454

Frattini, N. P280

Frattola, S. P491

Fraysse, J. P347

Free, P. P293

French, P. P308

Fridman, D. P232, P299

Fromel, L. P370

Fronti, E. P442

Frost, K. P013

Fry, S. P238, P239

Fuchs, A. O37.3

Fuentes, A. P250, P293

Fuertes, I. P485

Fuertes, L. P485

Fujiwara, A. P273

Fukushima, K. P201

Furlan, F. P003

Fusco, F. M. P486

Fusco, G. P013, P016, P078, P166, P167

Fusco, J. P013, P016, P078, P166, P167

Fusetti, C. P009, P011, P280

Fusto, M. P170

### G

Gadea, I. P485

Gagliardini, R. P055, P152, P155, P170, P255, P344, P374, P412, P449, P454

Gaigne, A.‐M. P073

Gainzarain, J. C. O38.2

Gajek‐Flanczewska, W. P199

Galai, T. P141

Galbraith, N. P197, P355

Galera Peñaranda, C. P103, P104, P105

Galindo Puerto, M. J. O35, O38.2, P087, P092, P120

Gallant, J. P049

Gallo, M. P117

Gandhi‐Patel, B. P167

Gañete, M. P428, P505

Garcia Abellan, J. P103, P104, P105

Garcia Ocaña, P. P119

Garcia Vallecillos, C. P119, P190, P446

Garcia Villalba, E. P104

García, F. P250, P293, P320

García‐Deltoro, M. P087

García Fraile Fraile, L. J. P033, P322

García‐Ruiz de Morales, A. P351

Gardin, S. P196, P443

Garg, M. P064

Garijo, R. P103

Garner, A. P088

Garrett, G. C. P268

Garuba, O. P340

Garvey, L. P365, P392

Gaseitsiwe, S. P303, P334, P515

Gasperin, A. P138, P218, P453

Gatell, J. M. P408, P468

Gay, C. P050

Gaytán Martínez, J. E. P404, P425, P434, P435, P463, P510

Gbedo, H. P373

Gedik, H. P519

Geerders, G. P182, P271

Generoso, S. P399

Gennari, W. P153

Gentile, J. P428, P505

Geny, C. P448

Gerber, F. P471

Geretti, A. M. P282, P374

Gergyek, L. P034, P035, P187

Gervasoni, C. P174

Ghosh, I. P419

Ghosn, J. O21, P053, P175, P234

Giacinta, A. P192, P449

Giacomelli, A. P085, P134, P152, P168, P174, P255, P394, P442, P445, P465

Giacomini, M. P458

Giandhari, J. O42, P305

Gibson, M. P297

Gil Alonso, L. P324

Gil Anguita, C. P103, P104, P105

Gil‐Girona, V. P360

Gili, S. P010

Gill, M. J. P246

Gilleece, Y. P212

Gilles, C. P005

Gillespie, G. P265

Gilson, R. P419, P421

Gimeno, A. P322

Giner Oncina, L. P103, P104, P105

Giomi, C. P224, P460

Giovannelli, A. P282

Girardi, E. P192, P380, P465

Girdziuniene, R. P497

Girometti, N. P514

Girshengorn, S. P341

Gisbert Pérez, L. O35, O38.2

Giupponi, G. P373

Giurco, M. P093, P142

Glidden, D. V. P015

Gliga, S. P381

Gmizić, I. P438

Godemert, M. P382

Goegana Sánchez, M. Á. P324

Goetghebuer, T. P462

Gogos, C. P343

Gohil, J. P054

Gohil, S. P109

Gomes, P. P181

Gomez Verdu, J. M. P104

Gompels, M. P408

Gonzalez‐Garcia, J. O38.3, P106

González, I. P223

González‐Cordón, A. P070, P357, P358, P408

González‐Jiménez, A. P490

Gonçalves, F. P181

Gonçalves, I. P122

Gonçalves, M. J. P114, P129, P452

Gonçalves Veloso, V. P506

Gopalakrishnan, K. P260

Gori, A. P009, P010, P055, P083, P134, P174, P394, P465, P486

Gorman, C. P002, P241

Gormezano, A. P026

Gotti, I. P280

Govender, N. P. P520

Goyache Moreno, A. P324

Gozzi, L. P396

Gómez‐Ayerbe, C. P489, P490

Gómez‐Muñoz, N. P087

Gökengin, A. D. P094, P319

Grace, D. P026

Graça, H. P122, P211, P220, P474

Grand, M. P428, P505

Grandhi, A. P017

Grasa, C. P089

Grasfeder, L. P240

Gravett, R. M. P023

Graziani‐Taugeron, C. P056

Greathouse, M. P188

Greco, M. P223

Greenberg, L. O38.1

Greene, L. P365

Gremel, G. P127

Grier‐Gavin, R. P365

Grinsztejn, B. P015, P384

Grove, R. P051, P052, P053, P058, P059, P154

Grunwald, S. P151

Gruters, R. P089

Guan, Y. P189

Guaragna, G. P428, P505

Guaraldi, G. P091, P107, P373, P380, P396, P399, P402, P407, P408, P412, P414, P415, P433, P465

Guardiani, M. P123, P138

Guignard, A. P013

Guilhou, T. O39.1

Guillaume, X. P217

Guimarães, M. P114

Guimond, T. P034, P035, P187

Gul Koca, Z. P133

Gulminetti, R. P213, P214, P491

Gunal, O. P319

Gundogdu, A. P249

Guo, Y. P062

Gupta, S. P042

Gupta‐Wright, A. P121

Gursel, E. P314, P342

Gusenheimer‐Bartmeyer, B. P381

Gutierrez, L.‐A. P448

Gutierrez‐Arroyo, A. P485

Gutierrez Sanjuan, J. P130, P178

Gutiérrez Liarte, Á. P033

Gutner, C. A. P046, P076, P077

Güerri, R. P087, P210

Gül, Ö. P183

Gündüz, A. P183, P226

Günthard, H. F. P247, P400, P480

Gwamna, J. P340

### H

Ha, B. P053

Haberer, J. P206

Haberl, A. P180

Hachfeld, A. P209

Haerry, D. P325, P400

Haigh, D. P088

Haimowski, G. P235, P236, P283, P384

Halfpenny, N. P064

Halim, A. P079

Halim, N. P036, P208, P237, P279, P368, P369

Halliday, F. P267

Hamel, A. P186

Hamid, H. O36

Hampel, B. O22

Hansen, H. J. O37.3

Hansen, S. P487

Hansson, D. P306

Harding, R. P468

Hardy, W. D. P275, P276, P277

Hareedy, R. P039

Harrison, B. P410

Harrison, R. P094, P167

Haslund Roed, A. K. P415

Hassan, I. A. H. P185

Hassler, S. P050

Hassman, M. P268

Hasson, H. P389, P464

Hatfield, K. P019

Hatip, A. P133

Haufroid, V. P266

Hay, J. P240

Hayes, R. P279

Hayward, A. P030

Hazra, A. P014

He, H. P343

He, Y. P179

He, Y. P189

Hedgcock, M. P095

Heekin, A. P377

Heffron, R. P017

Hellström, E. P238, P239

Henderson, M. P054, P450

Hendou, S. P455

Hendricks, M. R. P012

Henegar, C. P462

Hennelly, L. P421

Henrard, S. P005

Hentzien, M. P173

Herliyana, L. P261

Hermine Gbedo, L. O39.2, P020

Hernandez Cabrera, M. P059

Hernandez Lorente, M. D. P103, P105

Hernández Gutiérrez, C. P322, P431

Herrera, K. P206

Heslegrave, A. P450

Hetman, L. P242, P339

Hickey, C. P172

Hicks, C. P468

Hidalgo‐Tenorio, C. O35, O38.2, P092, P119, P190, P301, P446

Hilemichel, K. P457

Hill, A. P054, P302

Hill, J. N. P046

Hindman, J. P004

Hine, P. P172, P410, P457

Hirota, K. P273

Hisari, A. P273

Hlebowicz, M. O44

Hmissi, A. P064

Ho, T. H. Y. P398

Hocqueloux, L. P095, P263

Hodson, M. O39.3, P091

Hoffmann, C. P151, P487

Hong, K. P179

Hoogerwerf, J. P271

Horton, J. P043

Hovaguimian, F. P480

Hove, K. P058, P059, P076, P077

Hoving, C. P310

Hower, M. P440

Hsiao, A. P314, P342

Hsieh, A. P099

Hsieh, H.‐T. P512

Hsu, R. P013, P078, P166

Hsu, S.‐H. P317

Hu, M. P094

Hu, Y.‐H. P004

Huang, Y. P265

Huckaba, T. P044

Huergo, O. P322

Hug, O. P365

Hughes, A. P421

Huhn, G. P194

Huisman, R. P089

Hull, M. P026

Hung, C.‐C. P417

Hung, T.‐C. P072

Hunter, A. P109

Hurley, C. P483

Hussain, L. P288

Hwang, P. M. T. P015

Hwang, T.‐F. P079, P118

### I

Ian Kibet Ouko Babu, R. O39.2, P020

Iannetta, M. P517

Iannone, V. P083, P084, P280

Iannuzzi, F. P479

Iannuzzi, S. P111

Iaquinandi, S. P218

Iardino, R. P458

Ibarguren Pinilla, M. P324

Ideguchi, S. P383

Idelovici‐Marchal, C. P022

Ifedigbo, C. P363

Igartua, S. P301, P446

Ijimdiya Stephen, R. P432

Ikemiyagi, N. P383

Iliopoulos, P. P316

Illescas‐López, M. P250, P320

Imahashi, M. P383

Imamura, A. P201

Imamura, J. P383

Inacio Santos, C. P181

Ince, N. P319

Inchboard, L. P022

Inci Sezen, A. P495

Inciarte, A. P357, P358, P485

Ingrid Tasca, K. P327

Iribarren Loyarte, J. A. P324

Isernia, V. P234

Ishak, J. P149, P186

Ishizaka, A. P447

Isip, K. O39.2, P020

Ismael, A. P363

Ito, T. P272

Iwahashi, K. P275, P276, P277

Izuzquiza, I. P378

İnal, A. S. P071, P336

İnan, D. P319, P395

İnkaya, A. Ç. P095, P319, P395

### J

Jackson, A. P094, P095

Jackson, C. P462

Jacob, I. P074, P076, P077, P118, P120, P194, P468

Jacobs, N. P088

Jacobs, T. P270

Jager, N. P270

Jaha, B. O22

Jakubowski, P. O44

Jallow, A. E. P132

Janse, M. P252

Janssen‐van den Hurk, G. P270

Jarrett, J. P056, P057, P343

Jarrín Vera, I. P033, P159

Jaume, M. P258, P307

Jaume, M. P193

Javier Martinez, R. P190

Javorić, I. P475

Jayanth, A. P308

Jeffrey, J. P043

Jennings, L. O43

Jensen, B.‐E. O. P381

Jeong, H. P140

Jewell, A. P041, P051, P193

Jilich, D. O39.3, O44

Jimbo, G. P363

Jin, J. P189

Johnson, C. P421

Johnson, K. P419

Johnson, M. P392, P408

Jonas, K. O39.2, P020, P252, P353

Jones, B. P074, P076, P077, P118, P120

Jones, G. P347

Jongen, V. P354

Jonsson‐Oldenbüttel, C. P059, P061

Joos, M. P480

Jordão, S. P114, P129

Joseph Davey, D. O43

Joshi, M. P054

Jovelin, T. P175

Jover‐Díaz, F. P360, P367

Juárez Hernández, E. P403

Judd, A. P462

Juliano Romero Alibert, M. P384

Jung, S. P201

Jung, Y. P140

### K

Kadar, L. P254

Kafkalia, A. P241

Kahlert, C. R. P462

Kaiprath, L. P052, P053

Kakalou, E. P501

Kaleebi, J. P244

Kalin Unuvar, G. P319

Kall, M. P312

Kalogianni, E. P216

Kamio, S. P272

Kamphuis, A. P270

Kanno, Y. P447

Kantesaria, B. P268

Kaplan, R. P049

Kapoor, Y. P017, P268

Karabulut, N. P183

Karaca, G. P482

Karacaer, Z. P319

Karagiannis, A. P064

Karakök, T. P319

Karaoğlan, İ. P071, P319, P395

Karathanasis, C. P421

Karmochkine, M. O39.1, P393

Kart Yasar, K. P495, P519

Kassim, S. P401

Katana, E. B. P112, P113

Katchman, E. P341

Katlama, C. P408

Kausas, A. P497

Kavatha, D. P409

Kawai, M. P273

Kawuma, A. N. P318

Kaya Beyendi, E. P183

Kaya Kaptan, M. P183

Kaya, S. P319

Kayaaslan, B. P319

Kazooba, P. P244

Kedem, E. P094

Keegan, M. P045

Keegan, S. P027

Kefkir, A. P133

Kelly, B. P312

Kelly, C. P312

Kelly, C. P366

Kelly, M. P387

Kemper, A. P419

Kersey, K. P238

Khan, K. P285

Khaykin, P. O44, P180

Khoo, S. O43

Kilinc Toker, A. P133

Killer, A. P381

Kim, C. P203, P217

Kim, E. J. P102

Kim, G. P148

Kim, J. P050

Kim, M.‐K. P148

Kim, S.‐W. P095, P102

Kim, T. H. P102

Kim, Y.‐S. P094, P102, P140

Kim, Y. P148

Kinai, E. P056

King, C. P293

King, S. P370

Kirby, R. P345

Kirk, G. P507

Kirk, O. P207, P372

Kisare, M. P051, P052, P053, P193

Kitutu, F. E. P112

Kiweewa, F. M. P238, P239

Kılınç Toker, A. P319

Klein, M. P186

Kleinman, D. P274

Klicpera, A. O42, P305

Klint, J. P347

Klopfer, S. P060, P401

Knechten, H. P195

Knudsen, A. D. O37.3, P371, P467, P513

Kobayashi, T. P201

Kofoed, K. F. O37.3

Koga, M. P447

Kok, M. P182

Kokeyo, A. P204, P205

Koklu, D. P519

Kollan, C. P381

Kolobova, I. P046

Komano, J. P273

Konishi, K. P273

Konopnicki, D. P005

Koofhethile, C. P303, P515

Kopeykiniene, J. P497

Koppe, U. P381

Kortajarena Urkola, X. P324

Kostaki, E. G. P316

Kot, A. P024, P110

Kouadio, T. O39.2, P020

Kouriannidi, E. P501

Koussoulakos‐Vassilounis, K. P409

Kouyos, R. O22, O42, P111, P173, P209, P305, P480

Kowalska, J. P343

Kömür, Sü. P071, P336

Krankowska, D. P199

Krasidis, C. O39.2, P020

Kremser, I. P067

Krifors, A. P145

Kronfli, N. P186

Ku, H.‐Y. P072, P191

Kubwa, M. P230, P340

Kuhlman, J. P311

Kumar, P. O33, P057

Kunisaki, K. M. P467

Kuo, C.‐Y. P007, P021

Kuo, K.‐L. O31, P040

Kuran, G. P183

Kurle, S. P385

Kurtaran, B. P071, P336, P395

Kusejko, K. O22, O38.1, P247

Kuşcu, F. P071, P336

Kutsch, O. P044

Kyeyune, J. P318

### L

La Salete Martins, M. P481

Labhardt, N. D. O42, P305, P471

Lachowsky, N. P026

Lackey, P. P013, P016

Lacombe, K. O21, P193

Lagarde, M. P056, P057

Lagi, F. P083, P085, P124, P125, P126, P134, P394, P442

Lagiou, P. P316

Laguno, M. P357, P358

Lahl, M. P246

Lai, H.‐H. P317

Lai, Y.‐A. O31, P040

Lakatošová, Z. A. O44

Lal, A. P026

Lalezari, J. P. O31, P040

Lalloo, U. P238, P239

Lam, H.‐T. P253

Lamaizón, C. P221, P223, P429

LaMarca, A. P063

Lamb, K. P478

Lambertenghi, L. P488

Lambotte, O. P455

Lampe, F. P312

Landman, R. P175

Landovitz, R. J. P015

Lanini, S. P412

Lanzafame, M. P115

Lapadula, G. P055, P082, P152, P225, P445, P479, P504

Lara, M. P320

Larrieu, S. P022

Laškaj, R. P475

Lathrop, B. P297

Lattuada, E. P115

Laufer, N. P428, P505

Launay, O. O39.1

Laurans, S. P455

Lautner, M. P484

Lauw, F. P391

Lawrence, D. S. P520

Lawton, M. P027

Laymouna, M. P149, P186

Lazzarin, S. P009, P010, P032, P225

Le Chenadec, J. P455

Le Moal, G. P101, P461

Leal Martinez‐Bujanda, J. P087, P159

Lebech, A.‐M. P145

Lebouché, B. P050, P149, P186

Lee, J.‐A. P108

Lee, S.‐Y. P072

Lee, S.‐S. P398

Lee, S. H. P102

Lee, V. P345

Legac, S. P175

Legius, L. P424

Legkostup, L. P339

Legramante, J. M. P282

Lehmann, C. P050

Lehtoranta, L. P145

Leigh Brown, A. P111

Lekuona Sanz, M. P324

Lemaignen, A. P448

Lembas, A. P024, P110, P199

Lemogang, G. P334

Lemos, B. P210

Lener, A. P454

Lentini, N. P255

Leone, M. P282

Leone, P. P045, P046

Leone, S. P025, P032

Leoni, D. P176, P196, P443

Leopold, L. P039

Lerotholi, M. P471

Leroy, P. P086

Lesourd, A. P101

Lessard, D. P149, P186

Lessells, R. O42, P305

Lešnjak, I. P438

Letang, E. P120, P154

Leteemane, Q. P334

Leth, S. P145

Leung, S. P514

Leverette, B. P154

Levintow, S. N. P090, P127

Levy, I. P095, P476

Levytskyi, K. P341

Lewandowski, M. P194

Lewandowski‐Fowler‐Porter, C. P285

Lezama Mora, J. P403

Li, J. P012

Li, L. P072, P189

Li, M. P038, P063

Li, S.‐Y. P007, P191

Li, X. P189

Li, Y. P418

Li, Y. P370

Liang, Y. P265

Liberty, A. P057, P238, P239

Lichtner, M. P068, P123, P138, P218, P255, P376, P453

Liegeon, G. O21

Lim, T. P004, P090

Lim, Y. L. P387

Lin, M.‐H. P191

Lin, Y. P108

Lin, Y.‐W. P191

Lipman, M. P312

Lipnickiene, V. P497

Liu, H. P143

Liu, J. P189

Liu, L. P206, P405

Liu, S.‐L. P317

Liu, S.‐Y. P050

Liu, X. O33

Liu, Y. P265

Livrozet, J.‐M. P086

Ljujić, B. P438

Llamoso, C. P049, P050, P056, P057

Lleixa‐Martin, J. P511

Llenas‐García, J. O38.2, O38.3, P106

Llibre, J. M. P052

Lloyd‐Houldey, O. P368, P369

Lo Caputo, S. P152, P155, P225, P412

Lobo, G. P353

Locatelli, M. E. P479

Lock, E. P285

Lockman, S. P515

Loft, J. A. P467, P513

Loghmari Bouchneb, W. P101

Loi, G. P009, P420, P444, P464

Lolatto, R. O36, P003, P066, P163, P171, P389, P406, P420, P442, P444, P464, P493

Lombardi, F. P084, P134, P477

Longman, M. P308

Loosli, T. O42, P111, P305

Lopardo, G. P193

Lopes, L. P122, P211, P220, P474

Lopez Hernandez, M. A. P165, P200

Lopez Hidalgo, J. P301, P446

Lopez Lirola, A. P119

Lopez, T. P119

Losa García, J. E. O38.2

Losos, J. P045, P046

Losso, M. H. P049

Loufty, M. P150

Loukaidou, A. M. P409

Lovato, V. P066

Love, J. P017

López, A. A. P094

López Cabanillas, M. P033

López Cortés, L. F. P049

López García, S. P510

López Iñiguez, A. P337, P502, P503, P506

López‐Jódar, M. P489, P490

López Méndez, I. P403

Lu, L. P418

Lu, Y.‐C. P203, P217

Lucas Dato, A. O38.2

Lucchini, A. P011, P025, P032

Luedde, T. P381

Lui, G. C. Y. P398

Luk, F. W. L. P398

Lukau, B. O42, P305, P471

Lukubuya, J. D. P112, P113

Luna, L. P378

Lund Laursen, A. P145

Lung, S. P067

Luo, P. P418

Luo, Y.‐Z. P417

Lutfiyati, A. P290

Luz, E. P384

Lwembawo, J. P244

Lykos, E. E. P409

Łukasik, W. O44

### M

M'Bayo, R. O39.2, P020

Macías Moreno, L. P224

Mackie, K. P157

Mackie, N. P365

Macorigh Mantilla, L. M. P130, P178

Madeddu, G. P083, P085, P152, P168, P255, P380, P394, P406, P412, P413, P458

Madonia, S. P451

Madruga, J. P283

Maestripieri, C. P376

Magbadelo, D. P230

Magenta, L. P422, P423, P426, P427

Maggi, F. P170, P192, P486

Maggiolo, F. P055, P153, P225

Magiorkinis, G. P316

Magnenat, E. P400

Magoola, J. P318

Mahlab‐Guri, K. P254

Mahlatsi, P. P471

Maiocchi, L. P214

Maischack, F. P440

Majdoub, H. O39.2, P020, P353

Majerholc, C. P086

Makhema, J. P515

Makinson, A. P448

Makwakwa, R. P334

Malagnino, V. P282, P406

Malena, M. P115

Malki, A. P141, P386

Mallolas, J. P203, P217, P357, P358

Mallon, P. W. G. P371, P472

Maltez, F. P028

Mamarelis, C. P501

Manata, M. J. P028

Mancarella, G. P068, P123, P138, P218, P453

Mancini, G. P399, P402

Mancon, A. P486

Manda, V. P461

Mandaza, V. P036, P208, P237

Mangafas, N. P343

Mankge, N. P515

Mann, L. P004

Mansinho, K. P028

Mantilla, M. P223

Manzano, S. P322, P456

Mar, K. P203

Marake, N. B. P471

Maravitsas, V. P501

Marcellin, F. P101

Marchetti, G. C. P011, P032, P055, P095, P135, P154, P171, P255, P374, P376, P380, P406, P458, P517

Marcus, E. P230

Maresca, M. P170, P449, P454

Margot, N. P048

Maria, M. P443

Mariaggi, A.‐A. O39.1

Marinho, J. P122, P211, P220, P228, P474

Marino, A. P219, P492

Marinosci, A. P173, P209

Mariot, P. P073

Markarian, C. P166

Marković, M. P438

Markowitz, M. O31, P040

Marocco, R. P068, P123, P138, P168, P218, P453

Marom, R. P341

Marongiu, A. P094, P095, P159

Marques Silva, L. P481

Marta, R. P351

Martel‐Laferrière, V. P149

Martell, E. P206

Martin, C. P005

Martin‐Herrero, J. P104

Martin‐Iguacel, R. P145

Martin‐Segarra, O. P322

Martina, E. P010

Martina, M. P125

Martinez, B. P430

Martinez, L. P103, P104, P105

Martinez, M. J. P485

Martinez Martinez, M. P104

Martinez Rodriguez, R. P104

Martinez Sanz, J. P351

Martins, A. J. P116, P228

Martins, M. D. P051

Martín‐Carbonero, L. O35, O38.2, O38.3, P106

Martín‐Cortés, S. P489, P490

Martín‐Torres, J. P473

Martínez, A. P210

Martínez, E. P357, P358, P408, P415, P485, P502, P503

Martínez Álvarez, R. M. O38.2, P092

Martínez Buitrago, E. P223

Martínez Madrid, O. P103, P104, P105, P119

Martínez‐Rebollar, M. P357, P358

Maruapula, D. P303, P334, P515

Marukutira, T. P303

Maruotto, M. P124, P126

Maruri, F. P506

Mascarell, P. P103, P104, P105

Masci, D. P373

Mashele, N. O43

Masía, M. P408

Massobrio, G. P093, P142

Mastroianni, C. M. P123, P138

Mastrorosa, I. P155, P170, P449, P454

Mata Marín, J. A. P404, P425, P434, P435, P463, P510

Mateos, B. P351

Mathew, J. P297

Matila, D. P054

Matone, M. P083, P174, P213, P442

Matos, J. P100, P146, P481

Matovu Kiweewa, F. P017

Matthews, R. P. P038

Matulionyte, R. P497

Matus, N. P341

Mauas, R. P333

Maury, I. P028

Mavarani, L. P440

Maya Salazar, C. P403

Mayer, K. P004, P023

Mazzanti, L. P218

Mazzitelli, M. P115, P458, P466

Mazzoleni, L. P202

Mazzoni, S. P344

Mazzotta, V. P009, P010, P011, P025, P032, P055, P152, P170, P192, P225, P346, P374, P445, P449, P454, P486

Mbaba, E. P363

Mbewe, R. O39.3, P091

Mbudwini, S. P275, P276, P277

McBritton, M. P275, P276, P277

McCabe, M. E. P023

McCallister, S. P158

McCann, K. P054

McCarthy, M. P293

McCoy, D. P401

McDonald, C. P076, P077

McDwyer, P. P090

McEwan, A. P088

McGann, K. P238

McGettrick, P. P366

McGinty, T. P366

McGuire, D. P293

McKeon, T. P013

McKinnon, T. P312

McLaren, J. P197

McLellan, N. P387

McMahon, J. P157

McQuillan, O. P088

Medeiros, F. P122, P211, P220, P228, P474

Meiman, J. P265

Meissner, E. P048, P062

Mejia, J. P337

Melanophy, G. P377

Meli, P. P373

Mellor, J. P345

Mellors, S. P275, P276, P277

Meloni, D. P282

Memisoglu, F. P319

Mena de Cea, A. P050

Mena, L. P040

Mena, M. P229

Mendão, L. O39.3, P091

Mendes de Leon, K. P354

Mendes Fernandes, S. P181

Mendoza Ceacero, S. P459

Mendoza, A. P485

Meneses Cisneros, B. P404

Mengato, D. P196

Mengoni, F. P138

Menichini, B. P124, P125, P126

Menon, S. P309

Menozzi, M. P032, P055, P084, P085, P153, P396, P399, P402, P407, P433

Menozzi, M. P399

Menzaghi, B. P413

Menzies‐Banton, P. P036, P208, P237

Mercedes Cabral Amdi, N. P235, P236

Mercier, R.‐C. P050

Meriç Koç, M. P183, P226

Merino, E. P103

Mermutluoğlu, Ç. P071, P395

Mesplede, T. P089

Messeri, D. P124, P125, P126, P406

Messiaen, P. P136

Messina, E. O36, P420, P493

Mete, A. Ö. P071, P395

Mete, B. P226

Metzner, A. P019, P240

Meybeck, A. P461

Meyer, L. P448, P455

Meyer, N. P400

Méndez, J. P028, P100, P146, P154, P481

Mfondoum, P. H. P331

Mican, R. O38.3, P106, P322

Michard, F. P234

Micheli, G. P011, P025, P192, P225, P449

Micheli, V. P153

Mielczak, K. P316

Mijalovsky, D. P224, P460

Mikuła, T. P199

Milić, J. P107, P373, P396, P399, P402, P407, P414, P415, P433

Milinkovic, A. P154

Milla‐Olaya, B. P322

Mills, A. P013, P016, P050, P167

Min, S. P043

Minami, R. P383

Miranda, A. C. P028, P147, P181

Miranda, E. P285

Miró, J. M. P357, P358

Mishra, S. P466

Missel, M. P207

Mitchell, J. P291

Mmotsa, T. M. P520

Modica, S. P124, P125, P126

Mohamed, A. P392

Mohammed, N. P019

Mohammed, T. P334

Moi, G. P466

Moioli, M. C. P080

Mokebe, M. O42, P305

Mokgethi, P. T. P303, P334

Molai, M. P260

Molebatsi, K. P303

Moleboheng, M. P471

Molina, J.‐M. O21, P015, P401

Mondaray‐Marin, R. P105

Mondi, A. P255

Mondoka, C. P400

Monroy, V. P034, P035, P187

Montalbano, A. P445

Montaner, J. S. G. P356

Montañes, J. C. P485

Montejano, R. O38.3, P106

Montero‐Alonso, M. P431

Montero Hernández, M. d. C. O38.2

Montes, M. L. O38.3, P106

Montezuma‐Rusca, J. M. P062

Monyake, M. M. P305

Monzon, J. R. G. P059

Moodley, R. P154, P267

Moore, D. P026

Moorhouse, M. P158

Mora, L. P489

Mora‐Peris, B. P365

Morace, D. P493

Moraes, C. P283

Moraka‐Mankge, N. P303, P334

Morales, F. P428, P505

Morales‐Ramirez, J. P040, P050

Morano Amado, L. E. O35, O38.2, P092

Mordi, U. P127

Moreno, S. P119, P159, P351

Moreno Macias, L. P460

Moreno Zamora, A. P087, P351, P378

Morgan, L. P039, P041

Moriarty, M. P377

Mortier, V. P309

Moscatt, V. P492

Moschese, D. P009, P010, P011, P025, P032, P174, P280, P486

Moschopoulos, C. D. P409

Moseholm, E. P145

Mota, J. P223

Motaboli, L. O42, P305, P471

Mottamal, M. P044

Mouanga, C. P455

Mounzer, K. P013, P167

Moussallem, A. P217

Moutswi, S. T. P334

Movahedi, R. P054

Moyle, G. P408

Moyo, S. P303, P334, P515

Moznic, A. P373

Møller, D. L. O37.3, P513

Mpoloka, S. W. P303

Mponponsuo, K. P042, P048, P062

Mu, G. P240

Muccini, C. P080, P081, P082, P374, P380, P504

Mufudza, A. P371, P472

Mugo, N. P017

Muhindo, R. P113

Mujugira, A. P017, P112, P113

Mukhtar, S. P419

Mukonda, E. O43

Mularska, E. O44

Mulders, D. P089

Muldoon, E. P366

Mulenga, G. P303

Munoz, C. P087

Muñoz, S. P485

Muñoz‐Báez, P. P250, P320

Muñoz Perez, A. P103, P105

Muogbo, P. P340

Mura, T. P448

Muralitharan, O. P315

Muret, P. P073

Murphy, R. B. P356

Murray, C. P410

Murshid, M. V. R. P053

Mussini, C. P055, P083, P084, P085, P107, P115, P134, P153, P225, P255, P374, P376, P394, P396, P399, P402, P407, P414, P415, P433

Muwonge, T. R. P112, P113

Mvdudu, R. O43

Mwangi, D. P253

Myburgh, N. P520

Myer, L. O43

Müller, M. P381

### N

Nahass, R. P056, P057

Nahoum, N. P224, P460

Naidoo, S. J. O42

Naime Barbosa, A. P131, P222, P327

Najarro Cermeño, J. P144, P159

Nakagawa, F. P312

Nakamura, H. P057, P383

Nakauchi, T. P273

Nakceriene, B. P497

Nakijoba, R. P318

Nakir, İ. Y. P226

Nakmura, H. P056

Nalbantoglu, Ö. U. P249

Namale, G. P244

Namiba, A. O39.3, P091, P208, P237

Nannini, L. P258, P307

Naous, N. P364

Naqvi, A. P382

Nardi, S. P163

Narita, M. P383

Nasreddine, R. P136

Natter, N. C. P422

Natukunda, E. P238, P239

Navadeh, S. P144

Navarro, M. P462

Navarro Sáez, M. d. C. O35, P092, P130, P178, P335, P511

Navarro‐Soler, R. P473

Navarro Vilasaró, M. P130, P178, P335, P511

Naver, L. P462

Nazziwa, J. P145

Ndungu, M. P275, P276, P277

Neesgaard, B. P306

Negredo, E. P070, P210

Neill, C. P345

Neiman, A. P430

Nekrasova, L. P280

Nemet, S. P254

Nemeth, J. P247, P362, P480

Nencioni, C. P124, P125, P126

Neto, L. P114, P129, P156, P452

Nevado Pavón, C. P324

Neves, I. P114

Neves, N. P162

Neves, T. P116, P228

Nevoret, C. P086

Nguyen, C. P194

Nguyen, T. M. P508

Ngwoke, I. P278

Nicholls, L. P365

Nickel, P. P274

Nicolé, S. P115

Nielsen, S. D. O37.3, P207, P371, P467, P513

Nirmalasari, N. P290

Nishida, Y. P273

Niu, S. P137

Niyigena, E. P266

Nkengurutse, E. P008

Nodzynski, K. P149, P186

Noe, S. P061, P151

Noel, N. P455

Nogueira, R. P283

Noguera‐Julian, A. P462

Noori, T. P306

Norcross, C. P121

Nordestgaard, B. P467, P513

Notter, J. O22, P209

Nouwen, J. P089

Novella, M. P322

Nowak, P. P099, P145

Nozza, S. O36, P003, P009, P011, P025, P032, P055, P066, P091, P152, P168, P346, P389, P420, P444, P464, P465, P486, P493

Nsibirwa, S. P507

Nui, J. P239

Numa, R. P476

Nunnari, G. P219, P492

Nuñez, J. M. P416

Nuñez Orantos, M. J. O35

Nur Kirana, O. P261

Nussbaum, J. C. P265, P268

Nwagagbo, F. P230, P340

Nwaosu, Z. P036, P208, P237

Nwoke, U. P060

Nwokolo, N. P079, P275, P276, P277, P468

Nyaboke, S. P287

### O

O'Connell, I. P293

O'Connor, M. P366

O'Driscoll, J. P293

O'Farrell, B. P293

O'Neil, C. P274

O'Reilly, M. P056, P057

O'Rourke, J. P462

Oakley, R. P332

Obara, T. P272

Obermeier, M. P253, P484

Ocama, P. P507

Odemis, I. P319

Odiwuor Ondiek, S. P017

Odongpiny, E. A. L. P112, P113

Ogbuagu, O. P056

Oh Zhi Yang, B. P099

Ojara, F. W. P318

Okazaki, A. P447

Oke, O. P230, P340

Oko, J. P230, P340

Okoboi, S. P113

Okoli, C. P058, P059, P079, P120, P468

Okoro, U. P038

Okunoye, O. P340

Okwor, C. P230, P340

Okwuokei, N. P363

Olczak, A. O44

Oliva, A. P170

Oliver, S. P478

Ollivier, M. P448

Olmer, L. P476

Onotu, D. P230

Oluokun, Y. P230, P340

Omar, M. P119, P301, P446

Omri, A. P217

Oncul, A. P226

Ondari, R. P287

Onis, G. M. P500

Onofrio, M. P451

Onu, E. P230, P340

Onwuchekwa, C. P230

Onyango, D. O39.2, P020, P353

Onyedinachi, O. P363

Oosterhof, P. P270, P271, P348, P349

Op de Coul, E. P304

Oraegbu, G. P363

Oragwu, A. P166

Oreni, L. P085, P134, P174, P442

Orkin, C. O21, O39.3, P036, P050, P056, P062, P063, P091, P208, P237, P279, P368, P369, P392, P502, P503

Orlandi, C. P135

Orofino, G. P163, P168, P413

Orrell, C. O43

Ortblad, K. F. P112, P113

Orzechowska, J. P024, P110

Oshaben, K. P293

Osiyemi, O. P049

Osińska, J. P024, P110

Osterman, M. P166, P167

Ostrowski, S. R. O37.3, P371

Otieno, F. O. P015

Otti, J. O39.3, P019, P036, P208, P237

Ottou, S. P055, P170, P449

Overton, T. P043

Owino, M. P203, P217

Owori, J. P112, P113

Owoyemi, B. P278

Oyeka, A. P230, P340

Ozdemir, Y. E. P495, P519

Ozer, D. P319

Ozkan Ertay, B. N. P519

Ozkan, B. B. P249

Ozobodo, H. P230

Oztoprak Cuvalci, N. P319

### P

Pacheco, P. P028, P227

Pacífico, N. P500

Padilla Noguera, P. E. P425, P434

Padilla, S. P103, P104, P105, P431

Paganelli, C. P282

Pagani, A. A. M. P202

Pagani, G. P229

Pagliano, P. P451

Pagnucco, L. P163, P213, P214, P491

Pai Mangalore, R. P157

Paioni, P. P209

Paixão, L. P028

Palacios, R. P058, P489, P490

Palich, R. O39.1, P022, P393

Pallay, S. P263

Palmieri Weber, R. P016, P166

Pampaloni, A. P124, P125, P126

Pan, X. P343

Panagopoulos, P. P375

Panin, N. P266

Panopoulou, M. P375

Pansambal, P. P385

Pant, H. P158

Paoletti, M. P346

Paolini, M. A. P258, P307

Paolozzi, V. P093, P142

Papachristou, E. P316

Papadopoulos, A. P409

Papalini, C. P083, P394

Paparini, S. P036, P208, P237, P279, P368, P369

Papatheodoridis, G. P343

Papazoglou, D. P375

Pappalardo, L. P492

Paraskevis, D. P316

Parczewski, M. O44, P049, P057, P316

Park, D. W. P102

Park, K.‐H. P102

Park, S. P166

Parra Fariñas, R. P092

Pascual‐Bernaldez, M. P076, P077

Pasin, C. O39.3, P036, P091, P208, P209, P237

Pasquazzi, C. P218

Passini, F. P003, P389, P486

Patanè, R. P464

Patel, P. P051, P053, P154

Patel, R. P079, P275, P276, P277

Patel, T. P060

Paterson, A. P502

Patrat‐Delon, S. P461

Patrocínio Jesus, R. P227

Patrucco, S. P373

Patterson, B. P355

Paulicelli, J. P055, P170

Pavie, J. O39.1, P393

Pearce, V. P285

Pecchi, A. P399, P402, P433

Pecero García, E. I. P404, P425, P434, P435, P463, P510

Pecheny, M. M. P333

Pedersen, S. K. P207

Pedrosa Aragon, M. P130, P178, P511

Pehlivan, C. P151

Pelagalli, M. P282

Pellecchia, M. T. P451

Pellicano, G. F. P413

Pema, M. N. P334

Pena, E. P114, P129

Peng, X. P044

Peng, X. P262

Penon, S. P373

Pepe Razzolini, I. P310

Perazzo, H. P506

Pereira, C. P235, P236

Pereira‐Greene, L. P308

Perez, L. P320

Perez‐del‐Pulgar, S. P485

Perez Elias, M. J. P351

Perez‐Florido, J. P320

Perez Navarro, A. P302

Perez Trejo, M. E. P150

Perfezou, P. P101

Peris‐Garcia, J. P360, P367

Pernas Pardavila, H. O38.2, P092

Perrone, P. P373

Perrone, V. P344

Perugini, E. P282

Peters, H. P001, P002, P212, P241

Peters, J. P108

Petersen, N. P151

Petrakis, V. P375

Petroli, L. P458

Pett, S. P054, P392, P419, P421

Peytavin, G. P234

Pérez Hernández, I. A. P489

Pérez Martínez, D. O38.2

Pérez, M. G. P224, P460

Pérez‐Valero, I. P117, P459

Pfauth, A. P061

Pham, M. M. P038, P265

Pham, T. N. P508

Phansalkar, P. P240

Phanuphak, N. P015

Philibert, P. O21

Phillips, A. P312

Pi, C. P191

Piacentini, D. P115

Piccica, M. P124, P125, P126

Piconi, S. P413

Piecha, A. P275, P276, P277

Piercy, A. O39.2, O39.3, P020

Pieri, M. P282

Pierone Jr, G. P013, P016, P167, P430

Pietroluongo, D. P488

Pimenta, M. H. P384

Pimentel González, J. F. P435

Pinaire, M. P194

Pinheiro da Silva, D. P235, P236

Pinnetti, C. P170, P449, P454

Pinto Alves, M. P481

Pinto, V. P258, P307

Pinto‐Martinez, A. P473

Pinzón Meza, R. P223

Pires Roteia, D. P263

Pisano, L. P124, P125, P126

Pisculli, M. P060, P401

Piselli, P. P445

Pisoni, A. P382

Pistarà, E. P492

Pitchford, A. P154

Piñeiro, C. P162, P231

Plaçais, L. P455

Plank, R. M. P015, P017

Plazzi, M. M. P010, P055, P170

Ploumidis, M. P216

Pluta, M. P462

Poblete, G. P428, P500, P505

Poland, B. P038

Policeck, N. P300

Policek, N. O39.3, P091, P373

Poliseno, M. C. P458

Poliseno, M. P376

Polli, J. P240

Polokaova, A. P343

Poloni, A. P174

Pompa Mera, E. N. P404, P425, P434, P435, P463, P510

Pontali, E. P413

Pool, E. P030

Popovska Jovičić, B. P438

Portela Nunes, E. P506

Post, F. P056, P057, P392

Potthoff, A. P440

Poulakou, G. P501

Pourcher, V. P101

Pozniak, A. P070, P408

Pozza, G. P174, P442

Pozzi, A. P213

Pozzi, M. P124, P125, P126

Pradier, C. P382

Prajapati, G. P063, P064

Prakash, M. P158

Prata Menezes, N. P159, P167, P203, P217

Prata, M. P220

Pratolongo, E. P093

Prazuck, T. P263

Prendergast, E. P520

Prentice, M. P253, P484

Prevoteau du Clary, F. P086

Priest, J. P076, P077, P193, P347

Priscott, E. P370

Pristerà, P. P355

Proietti, M. P138

Prolo, A. P490

Protopapas, K. P409

Psichogiou, M. P343

Pucci, D. P453

Puchulu, M. M. P416

Puga, A. P462

Puig, J. P210

Pulido, F. P473

Pullukçu, H. P319, P395

Puoti, M. P080, P081, P082, P504

Pura, L. P261

Purwaha, D. P313

Puszkiewicz, P. P308

Puzzolante, C. P115

### Q

Qiao, J. P189

Quinn, K. O21

Quintero, J. P322

Quiros‐Roldan, E. P081, P082, P155, P376, P380, P504

### R

Rabatoko, L. P334

Raben, D. P306

Raccagni, A. R. O36, P003, P009, P025, P032, P066, P389, P444, P486, P493

Rachline, A. O39.1, P393

Raeber, F. P471

Rafailidis, P. P375

Raffi, F. P408, P461

Ragone, L. P070

Rajab, K. P112

Rajabzadeh, V. P036, P208, P237

Rajković, I. P438

Rakhmanina, N. P238, P239

Ralegoreng, C. P334

Ramadhan, D. P008

Ramalho Madruga, J. V. P235, P236

Ramgopal, M. O31, P040, P057, P063, P154

Ramirez‐Hurtado, A. P320

Ramirez Taboada, J. P301

Ramos, F. P337

Ramos, L. P283

Ramos, V. P164

Ramírez Eguía, S. P. P463

Ramírez Ramírez, M. D. P434

Ranadive, P. P384

Randazzo, N. P093, P142

Ranin, J. P438

Ranin, J. P438

Ranzani, A. P479

Rao, R. P042

Rapaport, S. P341

Rapti, V. P501

Ratanasuwan, W. P056, P057

Rauch, A. P173, P400

Raudonis, S. P497

Rava, M. P159, P431

Rawlins, S. P038

Rayment, M. P514

Raziano, V. P023

Read, P. P370

Rebelo, B. P147

Rebrina, M. P475

Reekie, J. P306

Reeve, A. P466

Reeves, I. O21

Reh, M. P206

Reichert, E. P194

Reis Luís, I. P227

Reitsma, T. O37.1

Remedios Alemán, M. O38.2

Repossi, R. P280

Resuli, S. P491

Reus Bañuls, S. P159

Reus, S. P103, P104, P105

Reyes, J. P432

Reyes‐Urueña, J. P306

Rezzonico, F. L. P082

Rezzonico, L. F. P080

Rhee, J. Y. P102

Riabokon, S. P242

Riako Anam, F. P468

Rial‐Crestelo, D. O38.2, P473

Riaño‐Pérez, A. P360, P367

Ribeiro, M. P181

Ribes Mengual, I. P103, P104, P105

Ricart, J. P223, P224, P460

Ricci, E. D. P413

Richman, B. P275, P276, P277

Ricucci, V. P093, P142, P153

Ridolfo, A. L. P174

Rienzo, P. P003

Riera, J. P485

Riera, M. P159

Rigo, A. P115

Rigo, F. P115

Rigoni, M. P229

Rijnders, B. P089

Ring, K. P036, P121, P208, P237, P502, P503

Ripamonti, D. P055, P081, P161, P202, P344, P504

Ritter, S. P195

Riva, A. P174, P213

Rivera, H. P337

Rivero, A. P485

Rivero, A. P117, P119, P459

Rivero, M. P190, P446

Rivero‐Juarez, A. P459

Rizzo, G. P483

Robbins, S. P019

Roberts, C. P002

Robineau, O. P022, P461

Robinson, D. P377

Robinson, E. P343

Robinson, M. P265

Roccio, M. P214

Rocha, F. P283

Rocker Ramos, L. P384

Rockstroh, J. P050, P343, P401, P408, P502, P503

Rodanò, A. P255

Rodger, A. P030, P312

Rodriguez Brieschke, M. T. P338

Rodriguez Evaristo, M. P404

Rodriguez, A. P258, P307

Rodriguez, C. A. P238, P239

Rodriguez, C. P291

Rodriguez, J. P446

Rodriguez‐Fernandez, C. P446

Rodríguez, A. O35, O38.2, P092

Roel, M. P221, P416, P429

Roemer, K. P151

Rogatto, F. P145, P468

Rogers, R. P167

Rogg, L. P043, P267

Rojas Castro, D. P275, P276, P277

Rokka, C. P316

Rokx, C. O37.1, O39.3, P089, P252, P310, P313, P391, P424

Roldan, G. P320

Rolla, S. O36, P493

Rolle, C.‐P. P057, P058, P059

Rolón, M. J. P233, P258, P307

Roma, E. P396

Rombini, M. F. P333

Romero, A. P119

Romero‐Hernández, B. P351

Romih Pintar, V. P037

Romijnders, K. P391

Ronit, A. P467, P513

Rosenberg‐Bezalel, S. P254

Rosińska, M. P343

Rossetti, B. P124, P125, P126, P153

Rossi, M. P115

Rossotti, R. P081, P082, P168, P280, P300, P488, P504

Rottenburg, A. P392

Roukens, A. O37.2

Rouquette, C. O39.3, P091

Routy, J.‐P. P149, P186

Rovito, R. P135, P517

Rozera, G. P192

Röhl, M. O42, P305

Ruane, P. J. P056, P057, P062

Rubio García, R. P473

Rudak, A. P150

Ruf, M. P343

Ruffolo, C. P443

Ruiz‐Sancho, A. P119

Ruiz‐Torres, L. P117

Rukmi, D. K. P290

Rusconi, S. P083, P085, P134, P152, P229, P406, P408

Russell, E. S. P017

Russell‐Puleri, S. P022

Russenberger, D. P247

Russo, P. P280

Rutland, E. P285

Ryan, P. P322, P456

Ryom Nielsen, L. O38.1, P306, P372

### S

Sabin, C. P121, P450

Sabone, P. P334

Sabranski, M. P487

Sadeghi, H. P376

Safarova, N. P249

Saglam Eslik, H. P133

Sağtaş, E. P319

Sahin Horasan, E. P071

Sailer, A. P381

Sakai, N. P392

Sakka, V. P501

Sala, E. P458

Sala, V. P009, P135, P376, P517

Saladini, F. P161

Salfa, F. P488

Sali, A. P. P056, P057

Salinas Velázquez, G. E. P404, P425, P434, P435, P510

Sally, D. P054, P421

Salmoni, L. P399, P415

Salter, A. P326

Saluzzo, S. O39.3

Salvador Rodríguez, A. P257

Salvadori, A. G. P488

Salvo, P. F. P083, P477

Sambo, M. P126

Sammet, S. P440

Samperiz, V. P322

Sampériz Rubio, V. P033

Samsonova, O. P242

Samukawa, S. P383

Sanchez Guirao, A. P104

Sanchez Labadia Hypolito, L. P384

Sanchez Samaniego, G. O42, P305

Sanchez, C. P320

Sanchez‐Lopez, B. P322

Sanders, T. N. P207

Sani, S. P124, P125, P126

SanJoaquin, I. P119

Santacreu‐Guerrero, M. P473

Santillán Arroyo, S. P435

Santini, M. P037

Santoro, A. P025, P380

Santoro, C. P376

Santoro, M. M. P153, P163, P171

Santos, J. P489, P490

Santos, L. P162, P231

Sanz, N. P322

Sapuła, M. P199

Saracino, A. P155, P255, P376

Sarakar, S. P016

Saretsky, T. L. P057

Sarmati, L. P282, P374, P445

Sarralde, F. P500

Sarteschi, G. P124, P125, P126

Sasset, L. P115, P176, P196, P443

Sato, E. P272

Saucedo, C. P203, P217

Saumoy, M. P431

Sayın, Ö. O. P336

Sánchez López, M. B. O35

Scansetti, V. P346

Scapellato, P. P338

Schafer, T. P014

Schäfer, G. P381, P487

Schembri, G. O21

Scherzer, J. P019, P061, P145

Schewe, C. K. P487

Schiavon Nogueira, R. P235, P236, P384

Schmid, P. O22, P173, P325

Schmidt, B. P440

Schmidt‐Radde, O. P180

Schoepf, I. C. P400

Scholten, S. P061, P091

Schouten, I. W. E. M. P182

Schreiber, S. P151

Schreuder, C. O43

Schroeder, M. P145, P347

Schuettfort, G. P180

Schuler, A. P293

Schurink, K. P089

Schübel, N. P061

Schürmann, D. P381

Schütz, S. P209

Schwarzmüller, M. P247

Sciattella, P. P346

Scifo, M. P219

Sconza, R. P002, P212

Scuderi, G. P219

Searle, S. P039, P041

Segala, D. P155

Segala, F. V. P115, P176, P196, P443

Segawa, I. P112, P113

Sekiya, R. P201

Sekulić Marković, S. P438

Selebalo, M. P305

Sell, L. P332

Semorile, K. P500

Sempere, A. P357, P358, P408

Sendagorta, E. P485

Sendaula, E. P244

SenGupta, D. O33, P049, P050, P056, P057

Senoglu, S. P495, P519

Sension, M. O31, P013, P016, P050, P078, P166, P167

Sequera, S. P190

Sereda, P. P356

Serejo, M. P108

Serraino, D. P445

Serraino, R. P168, P477

Serrano, C. P416

Serrão, R. P162, P231

Serwin, K. P316

Sevgi, D. Y. P226

Sewell, J. P312

Sezen, A. I. P519

Ségéral, O. O34

Shafran, S. P274

Shah, A. P070

Shakya, I. P023

Shapiro, A. E. P015

Shapiro, R. P515

Shaugnessy, M. P056

Shen, Y. P251, P405, P522

Sheng, W.‐H. P072

Shepherd, B. P041

Sherman, C. P166

Shevchenko, D. P242, P339

Shi, J. P179

Shibemba, M. P079, P275, P276, P277

Shih, C.‐Y. P284

Shihadeh, F. P049, P050

Shin, D. H. P102

Shirano, M. P050, P383

Shirasaka, T. P063, P273

Short, C.‐E. P302

Short, W. P120

Sides, K. P039

Sidhom, D. P365

Sierra Madero, J. G. P506

Sievers, J. P039

Siguier, M. P022

Sikwana, N. P017

Silva, D. P283

Silva, G. P122, P474

Silva, V. P283

Simmons, B. P054

Simsek Bozok, T. P071

Sinclair, G. P042, P063, P401

Sindín‐Martín, L. P322

Singh, N. P041

Sinness, M. P326

Sisto, A. P258, P307

Siwak, E. O44, P199

Sklar, P. P042, P048, P062, P144

Slama, L. O39.1, P059, P154, P393

Slobbe, L. P089

Smiles, J. P088

Smit, C. O37.2

Smith, A. P206

Smith, C. P312, P373

Smith, J. P150

Smuk, M. O39.3, P036, P091, P208, P237, P279, P368, P369

Sobczyk, M. O33

Sobrado, E. P. P258, P307

Soeiro, C. P114, P129, P156

Soetanto, H. P466

Solari, R. P224, P460

Solico, C. P373

Soloperto, S. P010, P161

Song, J. Y. P102

Song, W. P405

Songtaweesin, W. N. P462

Soochit, W. P089

Soothill, G. P111

Sophus, A. P291

Sorbian‐Gajewska, K. O44

Soria, A. P300, P479

Soriguer, C. P320

Sorni, P. P119

Soto, J. P353

Soulette, C. P048

Sousa, E. P231

Sönmezer, M. Ç. P319, P395

Sönnerborg, A. P099

Spagnuolo, V. O36, P066, P171, P389, P406, P420, P442, P444, P464, P493

Spampinato, S. P219, P492

Sparrowhawk, A. P312

Speciale, V. P068, P138, P218, P453

Spera, A. M. P451

Spínola Duarte de Oliveira, V. P235, P236

Spolverato, G. P443

Spoulou, V. P462

Spyrou, A. P216

Squillace, N. P155, P412, P413, P479

Sridhar, G. P070, P078

Stagnitta, M. P373

Stamm, L. M. P015, P017, P060

Stanicki, P. P131, P222, P327

PrEP‐Position Steering Group Member P036, P208, P237

Stefanelli, F. P093, P142

Steiner, R. J. P010, P192, P486

Stellbrink, H.‐J. P408

Stemkens, R. P270

Stempel, A. P440

Stempkowska‐Rejek, J. P024, P110

Stengaard, A. R. P306

Stephan, C. P151, P180, P408

Stirrup, O. P421

Stocker, H. P050, P343

Stoeckle, M. P173

Stolfi, P. P486

Stona, L. P462

Stöckle, M. P325

Strachan, S. O39.3, P036, P208, P237

Strachan, V. P334

Straga, F. P335

Stravodimou, E. P501

Striglia, P. O36

Stupnišek, M. P475

Stutterheim, S. P310

Suarez‐Zdunek, M. A. O37.3, P371

Sudhakaran, A. P238, P239

Sugiyama, M. P508

Sukumaran, L. P450

Sulaiman, A. P230

Sullivan, P. P314, P342

Sumer, S. P319

Sun, H.‐Y. P417

Sun, L. P184

Sun, N.‐L. P191, P243, P286

Surial, B. O22, P173, P325, P400, P480

Sutinen, J. P145

Swaine, T. P109

Swann, O. P450

Swygard, H. P019

Szetela, B. O44, P049

Szymczak, A. O44

Şimşek, F. P395

### T

Tabak, F. P226, P319

Tacconelli, E. P488

Tacconi, D. P124, P125, P126

Taguchi, N. P108, P144

Taiwo, B. P041

Taiwo, E. P054, P421

Takahashi, N. P272

Takak, H. P226

Tam, C. P356

Tamaki, H. P383

Tamanini, A. P488

Tan, D. P026

Tan, L. P039

Tanaka, M. P201

Tang, Z. P179

Tanner, C. P121

Tanuma, J. P508

Tapiwa, T. P471

Taramasso, L. P055, P093, P142, P155, P225, P413

Tariq, S. P030

Tarr, P. E. P400

Tarumbiswa, T. P305

Tasca, K. I. P131, P222

Tascon, G. C. P059

Tassi, M. P022

Taşova, Y. P071, P319, P336, P395

Tau, L. P341

Tavelli, A. P152, P155, P168, P374, P376, P380, P412, P445, P486

Taylor, B. P370

Taylor, S. O21

Taylor, S. P392

Te Brake, L. P270

Tejo, S. P116

Tempestilli, M. P010

Teocchi, R. P202

Teran, J. P103, P104, P105

Teruya, K. P095

Tesoro, D. P025

Teti, E. P255

Thakkar, N. P041

Theillay, A. P263

Thielen, A. P151

Thierry, A. P173

Thomas, K. P265

Thompson, R. P308

Thorne, C. P001, P002, P212, P241

Thornhill, J. P074

Thorpe, D. P094, P095

Thouvenot, E. P448

Ticchioni, M. P382

Ticozzelli, M. P229

Tiecco, G. P081, P082, P504

Tilston, P. P088

Timiryasova, A. O38.1

Tincati, C. P135, P376, P517

Ting Tan, L. P432

Tinguely, E. P325

Tiraboschi, J. O35

Tittle, V. O21, P302

To, H. W. K. P398

Todaro, L. P492

Toeque, M.‐G. P166

Togan, T. P128

Toledo, G. P462

Tomas Jimenez, C. P103

Tomassetti, F. P282

Tomás, J. G. P416

Tony‐Monye, I. P230, P340

Tookes, H. P127

Topcu, E. P519

Tordue, B. P340

Torkjazi, G. P389, P406, P420, P464

Torralba González de Suso, M. O35, O38.2

Torralba, M. P087, P092

Torres Macho, J. P456

Torres, B. P357, P358

Torres, T. S. P384

Tortellini, E. P123, P138

Torti, C. P055, P081, P412, P477, P504

Toscano, D. P227

Tosh, J. P019, P347

Tournay, A.‐C. P461

Towner, W. J. P057

Toy, J. K. P356

Toygar Deniz, M. P395, P482

Tran, G. V. P508

Tran, T. O39.3, P091

Tregoning, J. P450

Trehan, R. P076, P077

Trevisan, S. P083, P124, P125, P126, P394

Trigg, J. P026, P356

Trkola, A. P247

Troisi, P. P451

Trottier, B. P050, P094

Troya García, J. O35, O38.2, P092

Trøseid, M. P371

Trunfio, M. P449, P466

Truong, D. P096, P097, P098

Tsai, C. C. P317

Tsai, H.‐C. P264, P321

Tsai, M.‐S. P284

Tsai, M. J. P203

Tschumi, N. O42, P305, P471

Tseng, Y.‐T. P321

Tshishimbi, A. M. P229

Tuaillon, E. P382

Tumbarello, M. P124, P125, P126

Turco, M. P124, P125, P126

Turner, D. P341

Turriziani, O. P123

Turyahabwe, C. P318

Tutone, M. P025

Tutumlu, T. P247

Tweed, M. P285

Twinomujuni, I. P281

### U

Udenenwu, H. P363

Uehira, T. P273

Ueji, T. P273

Ueland, T. P371

Ukoaka, B. P363

Ulu Kilic, A. P249

Ummard‐Berger, K. P061

Upton, S. P206

Urda, L. P305

Uriel, A. P121

Uwimana, N. P008

### V

Vadlamudi, H. P240

Vagoraite, K. P497

Vai, D. P458

Valencia, J. P456

Valente, C. P116, P122, P211, P220, P228, P474

Valero Cifuentes, S. P103, P104, P105

Valero Rovira, S. P130, P178

Vallejo, A. P378

Valli, M. B. P486

Vallribera Olmos, E. P130, P178

Van de Klundert, M. P099

Van de Laar, T. P271

Van de Vijver, D. P089

Van der Mannen, J. P006, P182

Van der Meche, N. P182

Van der Valk, M. O37.1, O37.2, O39.3, P304, P354, P372

Van Druten, W. P310, P313

Van Frankenhuijsen, M. P136

Van Gorp, E. P089

Van Kampen, J. P089

Van Nood, E. P089

Van Osch, J. P089

Van Praet, J. P136

Van Schouwenburg, L. P089

Van Sighem, A. P304

Van Solingen‐Ristea, R. P039

Van Welzen, B. O37.1, O37.2, P095, P391, P424

Van Wyk, J. P019, P051, P070, P076, P077, P078, P154, P193

Vandermeulen, K. P039

Vania, E. P176, P196, P443

Vannappagari, V. P013, P016, P070, P078, P462

Varasi, I. P161

Vargo, R. P038, P268

Varlı, A. P319

Varol, Z. S. P319

Vassallo, M. P382, P448

Vassilakis, A. P316

Vasylyev, A. P185

Vavrek, M. P265

Vaz Alves, J. P181

Vaz Cardoso, J. P100, P146

Vazquez, M. J. P105

Vazquez‐Blanquiño, A. P320

Vázquez, E. P224, P460

Vecchiet, J. P412

Vega Calderón, A. P223

Vega, J. P258, P307

Vega, V. P275, P276, P277

Velluto, D. P451

Veloso, M. P181

Velyvyte, D. P497

Vendrame, E. O33

Vera, J. P121, P372

Vera Méndez, F. P103, P104, P105

Vera‐Méndez, J. P087

Verbon, A. O37.1, P391, P424

Verdier, G. P052, P053, P158

Vergori, A. P152, P168, P374

Verluyten, J. P353

Vesnesky, M. P060

Vetters, L. L. P036, P368, P369

Viale, P. P084

Viard, J.‐P. O39.1, P393, P461

Vicente, S. P149, P186

Vicenti, I. P153, P161

Vidal‐Barraquer, E. P019

Vieira, V. A. P238, P239

Vierling, M. P022

Viget, N. P461

Viglietti, R. P465

Vijeratnam, D. P478

Villacian, J. P043, P267

Villafañe Fioti, M. F. P224, P460

Villalobos, M. P490

Villanueva‐Cañas, J. L. P485

Villari, N. P219, P492

Vincenti, A. P124, P126

Vinti, P. P280

Vinuesa García, D. O35, O38.2

Vinuesa, D. P092

Vita, S. P123, P138, P218, P255, P453

Vitiello, P. P081, P082, P504

Vitor de Souza Oliveira, J. P235, P236

Vittar, N. S. P224, P460

Vivancos, M. J. P092, P322, P351

Vliegenthart‐Jongbloed, K. P310, P313

Vock, F. O22

Voermans, J. P089

Vogelmann, R. P095

Volny Anne, A. O39.3, P091

Volokha, A. P462

von Kleist, M. P111

von Shlosser, F. P373

Vonzun, L. P209

Vrouenraets, S. M. E. P182

Vulchanova, K. P362

### W

Wagner Cardoso, S. P384, P506

Wagner, C. P206

Waitt, C. O43, P318

Waldecker, M. P209

Walder, L. P355

Wali, H. P185

Walko, S. P194

Walsh, A. P366

Walsh, L. P293

Walshe, J. P293

Wan, Z. P047, P439

Wandeler, G. O34, P400

Wang, A. P042

Wang, C. C. P317

Wang, G. P044

Wang, H. O39.2, P020, P353

Wang, H. P049, P057

Wang, J.‐H. P072

Wang, K. P051, P058, P059, P076, P077, P154, P193

Wang, L. P179

Wang, L. P026

Wang, Q. P418

Wang, R. P051, P193

Wang, S.‐C. P512

Wang, T.‐C. P191

Wang, W. P004, P090

Wang, X. P137

Wang, Y. P143

Wang, Y. O22

Wang, Z. P143

Ward, M. P019

Warwick‐Sanders, M. P046

Wasswa, F. P244

Watanabe, D. P051, P062, P144, P273

Waters, L. P355, P408

Weber, A. P240

Weber, R. P167

Wei, H. P418

Weibull Wärnberg, A. P099

Weinman, J. P197

Wells, J. P421

Weng, Y.‐W. P321

West, E. P247

Westermayer, B. P061

Whetham, J. P285

White, J. P246

Whiteside, Y. P023, P057

Wick, G. O42, P305

Wiercińska‐Drapało, A. P024, P110, P199

Wiesmann, C. P096, P097, P098

Wiesmann, F. P195

Wiesner, L. O43

Wilches, V. P046

Williams, A. P309

Williams, B. M. P019

Williamson, M. P108, P144

Wilson, S. P363

Winston, A. P054, P450

Wit, F. O37.1, O37.2, P354, P372

Witzel, C. P030

Wohlfeiler, M. P013, P016, P078

Wolf, E. P151

Wong, A. P387

Wong, B. C. K. P398

Wong, C. N. S. P398

Wong, P. O21

Woo, J. P366

Wood, M. P027, P285

Woodward, K. P034, P035, P187

Wordi, D. P466

Workowski, K. O33

Wright, A. P430

Wu, K.‐S. P321

Wu, P.‐Y. P417

Wu, X. P137

Wyen, C. P061, P151

### X

Xiao, J. P038

Xiong, Y. P262

Xu, G. P179

Xu, L. P179

Xu, S. P038

Xu, W. P405

Xu, Y. P038, P060, P401

Xun, J. P251

### Y

Yager, J. O21

Yagura, H. P273

Yajima, K. P201

Yamamoto, K. P383

Yamaniha, K. P383

Yamashiro, S. P383

Yamaura, R. P144

Yan, X. P522

Yang, C.‐J. P072, P095, P284

Yang, J.‐H. P072, P191

Yang, S. P418

Yang, X. P137

Yang, X. P251

Yang, X. P137

Yapha, M. O39.2, P020

Yaphe, S. P149, P186

Yardımcı, Ö. F. P336

Yen, T.‐F. P317

Yeniceri, O. P071

Yetkin, M. A. P319

Yi, S. P096

Yiğit, Y. P183

Yildirim, Ö. P484

Yildiz, Y. P319

Yin, Z. P090

Yıldız, İ. P395

Yıldız Kaya, S. P226

Yıldız Sevgi, D. P183

Yılmaz Tuğan, B. P482

Yombi, J. C. P136, P266

Yoshida, K. P201

Yotsuyanagi, H. P447

You, W. P056

Young, B. P468

Young, K. P064

Yu, J. P184

Yuan, Z. P179

Yuen, R. K. K. P398

Yurdakul, Z. Z. P519

Yusuf, Y. P340

Yüksel, N. P482

### Z

Zace, D. P282

Zacher, C. P440

Zachry, W. P127, P314, P342

Zaegel, O. P448

Zaegel‐Faucher, O. P461

Zagato, D. P280

Załęski, A. P024, P110

Zamora‐Clemente, E. P357, P358

Zanchi, C. P488

Zaninetta, E. P517

Zapolska, N. P339

Zazzi, M. P161, P171

Zeeb, M. P173, P209, P247, P325, P362, P480

Zekan, Š. P037, P475

Zerdali, E. P226

Zetterberg, H. P450

Zhang, C. P063

Zhang, F. P184

Zhang, H. P184

Zhang, L. P111

Zhang, N. P042

Zhang, R. P405

Zhang, W. P356

Zhang, X. P522

Zhao, H. P184, P189

Zhao, Y. P189

Zheng, Y. O33

Zhou, X. P047

Zhu, B. P047, P262, P439

Zhu, H. P251

Zhu, X. P047

Zhu, Y. P143

Zhumi, R. P428, P505

Zibli, D. P476

Zimmermann, H. O39.2, P020, P353

Zimovetz, E. P185

Zingaropoli, M. A. P123, P138

Zingman, B. S. P019

Zitko, P. P223

Zou, C. P418

Zuccaro, V. P214

Zucman, D. P086

Zuppiroli, A. P084, P085, P115, P445

